# Supplementary material for: Biomarker Expression of Peri-Implantitis Lesions before and after Treatment: A Systematic Review
Source: Int J Environ Res Public Health. 2022 Oct 28;19(21):14085. doi: 10.3390/ijerph192114085 (PMC9659150; doi:10.3390/ijerph192114085)
Supplement: Supplementary file 1 [file ijerph-19-14085-s001.zip › SM1_Table S1 _PrimaryOutcome.pdf]

International Journal of
Environmental Research and Public Health

MDPI

Systematic review

Biomarker Expression of Peri-Implantitis Lesions Before and After Treatment: A Systematic Review.

Supplemental Table S1. Primary outcome measures of included studies. SSD (Statistically significantly differences p<0.05), NSSD (No statistically significant differences p>0.05). Antibiotic, AF: access Flap, ALKP: Alkaline Phosphatase, AZM: Azithromycin, CCL- 5: Chemokine ligand- 5,CHX: Chlorhexidine, CRP: C- Reactive Protein, ELISA: enzyme-linked immunoassay, EMD: Enamel Matrix Derivative, F: Female, GM-CSF: Granulocyte Macrophage Colony-Stimulating Factor, IFN-g: Interferon- gamma, IL-1b: Interleukin-1beta, LILT: Low Intensity Laser Treatment, LDD: Local Drug Delivery, M: Male, MBL: Marginal Bone Loss, MC: Minocycline Hydrochloride, MCP-1 : Monocyte Chemotactic Protein-1, MIP: Macrophage Inflammatory Protein , MMP: Matrix Metallo Proteinase , MPO: Myeloperoxidase, MW: Mouthwash, n: number, NR: Not Reported, NSPT: Non-surgical periodontal treatment, NSSD: Not Statistically Significant Different, OC: Osteocalcin, OFD: Open Flap Debridement, OPG: Osteoprotegerin, OPN: Osteopontin, PCR: Polymerase Chain reaction, PDT: Photo Dynamic Therapy, PDGF: Platelet Derived Growth factor, pg: Picogram, PICF: Peri Implant Crevicular Fluid, PIM: peri-Implant Mucositis, PIP :Peri-implantitis, PIH: Peri-implant Health, RANK: Receptor Activator of Nuclear factor Kappa-B, RANKL: Receptor Activator of Nuclear factor Kappa-B Ligand, RANTES: Regulated on Activation, Normal T Cell Expressed and Secreted, RBL: Radiographic Bone Loss, rCAL: relative Clinical Attachment Loss, RCT: Randomised Clinical Trial, REC: Recession, SUP: Suppuration, SSD: Statistically Significant Difference, TNF: Tumour Necrosis Factor, TGF: Tissue Growth Factor, Ti: titanium, VEGF: Vascular.

Investigator, year	Study group	Biomarker concentration	Baseline	Final assessment	Comments*
Bassetti et al., 2014	LDD	IL-1b (pg/site)	11	4	Final assessment 12 months post treatment.<br>SSD (P < 0.05)
		IL-8 (pg/site)	20	11	NSSD
		IL-10 (pg/site)	24	7	SSD (P < 0.05)
		MMP-1 (pg/site)	8	10	NSSD
		MMP-8 (ng/site)	0.75	0.55	NSSD
	a-PDT	IL-1b (pg/site)	7	3	SSD (P < 0.05)
		IL-8 (pg/site)	17	12	NSSD
		IL-10 (pg/site)	20	8	SSD (P < 0.05)
		MMP-1 (pg/site)	11	5	NSSD

		MMP-8 (ng/site)	0.6	0.45	NSSD
Bhavsar et al., 2019	PIH	IL1-b (pg/ µL)	1.7 ± 2.9	-	-
		MMP-1 (pg/µL)	1.3 ± 1.1	-	-
		MMP-8 (pg/µL)	10.7 ± 9.7	-	-
	AF + Anti-infective therapy	IL1-b (pg/µL)	17.9 ± 41.8	5.3 ± 8.2	Concentration reduced after treatment but still significantly higher than control group.
		MMP-1 (pg/µL)	1.2 ± 1.2	1.7 ± 1.2	Similar concentration between 2 groups always.
		MMP-8 (pg/µL)	12.1 ± 10.0	6.7 ± 3.9	Significant reduction post treatment in test group but still higher than control group.
De Mendonga et al., 2009	PIP	TNF-a (pg/site)	2.8 – 3.0	0.4 – 0.9	Final assessment was carried out 12 months after surgery<br>SSD
Duarte et al., 2009	PIH	IL-12(pg/site)	12.5	-	NSSD
		IL-4(pg/site)	0.5	-	NSSD
		IL-10(pg/site)	80	-	NSSD
		TNF-a(pg/site)	0	-	-

		OPG (pg/site)	90	-	-
		RANKL (pg/site)	40	-	-
		OPG/RANKL	2.4	-	-
	PIM	IL-12(pg/site)	13	14	NSSD
		IL-4(pg/site)	0.2	0	NSSD
		IL-10(pg/site)	100	80	NSSD
		TNF-a(pg/site)	1	0	SSD
		OPG (pg/site)	83	83	-
		RANKL (pg/site)	45	43	-
		OPG/RANKL (pg/site)	2.2	2.2	-
	PIP	IL-12(pg/site)	15	11	NSSD
		IL-4(pg/site)	0.5	0.55	NSSD
		IL-10(pg/site)	80	85	NSSD
		TNF-a(pg/site)	3	0	SSD before and after treatment, significantly higher in PIP compared to PIH.
		OPG (pg/site)	70	72	SSD at baseline vs. PIH.
		RANKL (pg/site)	52	37	-
		OPG/RANKL (pg/site)	2.1	2.3	SSD between PIH and baseline in PIP.
Esberg et al., 2019	EMD group	Proteome analysis	-	-	

	Non EMD group				EMD treatment significantly associated with a decreased prevalence of 40 PICF proteins.
Gershenfeld et al., 2018	AZM+ NSPT	IL-1b (pg/mL)	-	-	No raw data reported<br>Both treatment modalities resulted in reduced concentration of investigated biomarker.<br>NSSD between 2 groups.
	Placebo +NSPT	IL-1b (pg/mL)			
Granfeldt et al., 2010	OFD	MMP	-	-	No raw data reported<br>SSD in MMP-8 levels reduction after 12 months compared to before surgery. NSSD in MMP8 reduction between 2 groups.
	OFD + Ti	MMP8			
Hallstrom et al., 2016	Placebo	IL-1b	17.1	8.2	Final assessment at 12 weeks after treatment.<br>There was a tendency of reduced levels of the pro-inflammatory cytokines and chemokines during the intervention period in both investigated groups compared to baseline, but there were NSSD between the two groups.
		IL-1RAd	7.1	2.3	
		IL-4	1.2	0.8	
		IL-6	2.8	3.3	
		IL-8	278	176	
		IL-17A	3.9	1.9	
		CCL5/ RANTES	8.6	3.2	
		TNF-a	2.7	1.8	
		IFN-g	97	36	
		GM-CSF	0.2	0.4	
	Probiotic supplement	IL-1b	14.2	13.6	

		IL-1RAd	4.4	3.5	
		IL-4	2.7	0.5	
		IL-6	7.2	2.5	
		IL-8	474	251	
		IL-17A	11.3	1.3	
		CCL5/ RANTES	8.6	2.8	
		TNF-a	4.7	1.2	
		IFN-g	278	33	
		GM-CSF	1.6	0.1	
Kalos et al., 2015	PIH	IL1-b	10.301 (SD=1.150)	-	Final assessment was carried out 6 months after therapy.<br>NSSD between 2 groups at any time point.
	NSPT+AZM	IL1-b	114.348 (SD=160.291)	21.396 (SD=19.74)	
	NSPT	IL1-b	85.919 (SD=77.072)	36.059 (SD=28.614)	
Komatsu et al., 2018	Laser	IL-1a (ng/ml)	5.05	4.46	Final assessment was 3 months after treatment.
		IL-1b (ng/ml)	0.10	0.05	-
		IL-6 (ng/ml)	0.93	0.66	-
		IL-8 (ng/ml)	0.38	0.36	-

		TNF-a (ng/ml)	6.16*	4.32	*: Significantly higher in laser group compared to Minocycline group at baseline.
		CRP (ng/ml)	0.35	0.34	-
		MMP-1 (ng/ml)	0.09	0.07	-
		MMp-3 (ng/ml)	0.20	0.12	-
		MMP-9 (ng/ml)	9.84	9.84**	**: Significantly Lower in laser group compared to Minocycline group at 3 months.
		MMP-13 (ng/ml)	0.41	0.22	-
	Minocycline	IL-1a (ng/ml)	3.57	5.22	-
		IL-1b (ng/ml)	0.04	0.95	-
		IL-6 (ng/ml)	0.00	0.00	-
		IL-8 (ng/ml)	0.16	0.29	-
		TNF-a (ng/ml)	2.95*	4.91	*: Significantly higher in laser group compared to Minocycline group at baseline.
		CRP (ng/ml)	0.32	0.28	-
		MMP-1 (ng/ml)	0.03	0.03	-
		MMp-3 (ng/ml)	0.17	0.08	-
		MMP-9 (ng/ml)	20.96	19.21**	**: Significantly Lower in laser group compared to Minocycline group at 3 months.
		MMP-13 (ng/ml)	0.20	0.12	-
Malik et al., 2015	Healthy peri-implant tissue	ALKP	20	-	-

		MPO	4	-	-
	PIP + NSPT	ALKP	58	20	Final assessment was 3 months after baseline.<br><br>PIP group had SS higher MPO and ALKP at baseline and after 3 months compared to healthy sites (P < 0.001) .
		MPO	18	7	
Peres Pimentel et al., 2019	Triclosan/fluoride toothpaste<br><br>Mean (± *SD*)	IFN-γ (pg/µl)	3.0 ± 3.6	2.3 ± 2.2	Final assessment was made at 3 weeks' time.<br><br>No intra- or inter-group differences were observed for IFN-γ, IL10, IL-1β, IL8, IL-17, IL-6, TNF-α, MMP-2, MMP-9, TGF-β, OC, OPN, ICTP, OPG and RANKL (*p* > 0.05).<br><br>RANKL/OPG ratio was significantly higher in fluoride toothpaste-treated sites when compared to triclosan/fluoride-treated sites at the end of period without mechanical tooth brushing, on the 21st day (*p* = 0.041).
		IL−10 (pg/µl)	1.9 ± 2.1	1.9 ± 2.4	
		IL−17(pg/µl)	9.5 ± 21.7	1.9 ± 1.4	
		IL−1β (pg/µl)	38.4 ± 48.9	70.2 ± 104.5	
		IL−6 (pg/µl)	1.0 ± 2.0	1.9 ± 3.6	

		IL−8 (pg/µl)	109.5 ± 275.1	48.7±145.5	
		TNF-α (pg/µl)	2.9 ± 9.4	2.4 ± 5.9	
		MMP−2 (pg/µl)	731.0 ± 806.1	1,030.8 ± 945.3	
		MMP−9 (pg/µl)	20,631.5 ± 20,103.2	16,961.8 ± 17,064.1	
		OPG (pg/µl)	53.2 ± 62.5	37.6 ± 31.5	
		RANKL (pg/µl)	2.9 ± 1.7	1.9 ± 1.4	
		RANKL/OPG (E1) (pg/µl)	1.4 ± 1.6	0.8 ± 0.5	
		OC (pg/µl)	708.7 ± 898.7	680.7 ± 767.7	
		OPN (pg/µl)	509.2 ± 582.9	474.3 ± 484.5	
		TGF-β(pg/µl)	5.5 ± 5.6	5.4 ± 3.1	

		ICTP (pg/µl)	204.3 ± 194.7	197.4 ± 145.0	
	Fluoride toothpaste Mean (± *SD*)	IFN-γ(pg/µl)	2.5 ± 2.9	2.1 ± 2.1	
		IL−10(pg/µl)	1.7 ± 2.2	1.1 ± 1.1	
		IL−17(pg/µl)	9.2 ± 23.9	2.7 ± 2.6	
		IL−1β(pg/µl)	55.3 ± 53.7	154.7 ± 364.8	
		IL−6(pg/µl)	0.8 ± 2.4	0.1 ± 0.2	
		IL−8(pg/µl)	77.0 ± 200.5	34.6± 77.5	
		TNF-α(pg/µl)	0.6 ± 1.2	0.6 ± 0.2	
		MMP−2(pg/µl)	1,310.6± 580.2	819.1 ± 524.7	
		MMP−9(pg/µl)	28,097.8 ± 22,041.1	59,899.2 ± 66,690.4	
		OPG (pg/µl)	55.3 ± 46.9	34.4 ± 37.9	

		RANKL (pg/µl)	2.8 ± 1.7	3.5 ± 3.5	
		RANKL/OPG (E1) (pg/µl)	0.9 ± 1.1	1.8 ± 1.5a	
		OC (pg/µl)	918.4 ± 772.3	1706.5 ± 1,474.2	
		OPN (pg/µl)	445.6 ± 324.8	1,089.8 ± 914.8	
		TGF-β(pg/µl)	7.3 ± 8.0	8.2± 8.7	
		ICTP (pg/µl)	449.9 ± 679.8	257.4 ± 258.6	
Renvert et al., 2017	Stable outcome	IL-1ß (pg/ml)	365.9	88.4	In methodology 10 biomarkers were assessed: IL-1b, IL-1ra (receptor antagonist) IL-6, IL-8, IL-17A, IP-10 MIP-1a , PDGFBB , TNF-a , and VEGF .however, only results of 5 of them reported. It is only reported that Statistical analysis failed to identify differences in cytokine levels for the remaining six cytokines assessed.<br>Final assessment was carried out 6 months after treatment.

					Overall lower value compared to unstable patients (mean diff: 408.4, 95% CI: 153.9, 662.8, P = 0.002) .
		IL-6 (pg/ml)	12.3	6.6	Overall lower value compared to unstable patients (mean diff: 11.5, 95% CI: 3.4, 19.5, P = 0.007) .
		VEGF (pg/ml)	507.9	242.3	Overall lower value compared to unstable patients (mean diff: 326, 95% CI: 134.0, 518.2, P = 0.002)
		TNF-α (pg/ml)	4	4.8	-
		PDGFBB (pg/ml)	11.7	6.9	-
	Unstable outcome	IL-1ß (pg/ml)	376	496.7	-
		IL-6 (pg/ml)	43.2	18.1	-
		VEGF (pg/ml)	996.5	568.4	-
		TNF-α (pg/ml)	17.1	8.2	TNF-a levels at baseline were higher at those implants with unstable treatment outcome at 6 months (P < 0.01).

		PDGFBB (pg/ml)	30.4	12.9	-
Ribeiro et al., 2018	Triclosan	INF-y (pg/µL)	7.5 ± 14.4	± 3.1	Final assessment was taken 3 weeks after treatment.<br>Different capital letters represent significant intergroup differences by Wilcoxon (*P* < 0.05).<br>Different non-capital letters represent significant intragroup differences by Friedman (*P* < 0.05).<br>No intra- or intergroup differences were observed
		IL-10 (pg/µL)	4.0 ± 5.4	3.8 ± 2.3	-
		IL-17 (pg/µL)	2.5 ± 2.3	2.0 ± 1.3	No intra- or intergroup differences were observed.
		IL-1β (pg/µL)	21.3 ± 19.8	20.9 ± 17.8	-
		IL-4	13.3 ± 21.7	19.0 ± 26.3	No intra- or intergroup differences were observed.

		(pg/μL)			
		IL-6 (pg/μL)	2.1 ± 3.5	3.2 ± 2.9	No intra- or intergroup differences were observed.
		IL-23 (pg/μL)	0.1 ± 0.3	0.0 ± 0.2	No intra- or intergroup differences were observed.
		TNF-α<br>MMP-2 (pg/μL)	2.2 ± 3.9	1.3 ± 1.2	No intra- or intergroup differences were observed.
		MMP-2 (pg/μL)	519.6 ± 718.0	771.7 ± 674.5	No intra- or intergroup differences were observed.
		MMP-9 (pg/μL)	49515.5 ± 99652.7	32119.2 ± 31704.5	No intra- or intergroup differences were observed.
		OPG<br>(pg/μL)	38.4 ± 28.5	178.5 ± 145.8	Was significantly augmented from the 14th ($P = 0.02$) until the 21st day ($P = 0.034$) when compared with baseline and 7 days only in triclosan-treated sites.

		OC (pg/μL)	333.9 ± 373.3	164.1 ± 127.4	No intra- or intergroup differences were observed.
		OPN (pg/μL)	299.7 ± 391.5	538.0 ± 345.8	No intra- or intergroup differences were observed.
		TGF-β (pg/μL)	18.2 ± 24.9	7.5 ± 6.8	No intra- or intergroup differences were observed.
		ICTP (pg/μL)	215.0 ± 360.3	156.1 ± 126.2	No intra- or intergroup differences were observed.
	Placebo	INF-y (pg/μL)	5.7 ± 16.1	2.4 ± 5.1	-
		IL-10 (pg/μL)	3.1 ± 4.2	0.9 ± 1.3	IL-10 levels decreased only in placebo-treated implant sites, presenting significantly lower concentrations at 21 days when compared with baseline (*P* = 0.0051) and compared with Triclosan group at 21 days (*P* = 0.0036).

		IL-17 (pg/μL)	1.6 ± 1.9	2.5 ± 4.2	-
		IL-1β (pg/μL)	14.9 ± 19.3	194.7 ± 226.1	Increased during the progression of mucositis only in the placebo group, with significantly higher amounts at 21 days when compared with baseline (*P* = 0.041).<br>No intergroup differences have been detected (*P* > 0.05).
		IL-4<br>(pg/μL)	12.5 ± 18.8	10.8 ± 12.7	No intra- or intergroup differences were observed.
		IL-6 (pg/μL)	1.8 ± 2.6	5.6 ± 12.4	
		IL-23 (pg/μL)	0.1 ± 0.1	0.1 ± 0.2	
		TNF-α<br>MMP-2 (pg/μL)	4.9 ± 15.8	0.9 ± 1.5	

		MMP-2 (pg/µL)	519.5 ± 423.1	558.7 ± 327.5	
		MMP-9 (pg/µL)	25994.3 ± 28476.5	33481.8 ± 37594.3	
		OPG (pg/µL)	66.6 ± 86.9	119.9 ± 179.3	
		OC (pg/µL)	444.1 ± 645.9	230.7 ± 192.5	
		OPN (pg/µL)	462.6 ± 580.6	314.1 ± 314.8	
		TGF-β (pg/µL)	10.9 ± 12.2	9.8 ± 19.8	
		ICTP (pg/µL)	353.5 ± 449.3	195.1 ± 137.4	

Thierbach et al., 2016	Healthy/Gingivitis group	smokers	MMP8 (ng/mL)	40.2 (-11.7-92.1)	13.3 (-0.7-27.3)	Final assessment was carried out 6 months after treatment.
		Non-smokers		40.4 (-2-82.8)	5.5 (-2.6-13.6)	
	Periodontitis Group	Smokers	MMP8 (ng/mL)	108.7 (22.6-194.8)	27.0 (-12.4-66.4)	Broader MMP-8 levels' distribution in periodontitis group vs healthy/gingivitis sites.<br><br>P-value: 0.043
		Non smokers		141.8 (32.5-251.0)	12.4 (0.62-24.2)	P-Value: 0.047
Wohlfahrt et al., 2014	OFD		MMp-8 (pg/ml)	-	-	No raw data for biomarkers in different groups were reported.<br><br>No difference in bone marker levels between the test and control group was found either at baseline or at 12 months (*p* > 0.05).<br><br>No difference between the test and control groups in change from baseline to 12 months for any of the bone markers was found (*p* > 0.05) .
			IL- 6 (pg/ml)			
			OPG (pg/ml)	-	-	
			OC (pg/ml)			
			Leptin (pg/ml)	-	-	
			OPN (pg/ml)			

		PTH (pg/ml)	-	-	Comparing the levels between baseline and 12 months, irrespective of treatment group, TP, MMP-8 , IL-6, OPG , leptin and adiponectin were significantly reduced.
		TNF-a (pg/ml)			
		Adiponectin (pg/ml)	-	-	Comparing the levels between baseline and 12 months irrespective of treatment, the levels of OC, OPN, PTH, TNF-α and insulin were not changed.
		Insulin (pg/ml)			
	OFD+ porous titanium granules	MMp-8 (pg/ml)	-	-	Comparing the levels between baseline and 12 months, irrespective of treatment group, when Bonferroni corrections were applied, statistically significant differences were only present for leptin ($p < 0.002$) and adiponectin ($p < 0.001$).
		IL- 6 (pg/ml)			
		OPG (pg/ml)	-	-	
		OC (pg/ml)			
		Leptin (pg/ml)	-	-	
		OPN (pg/ml)			
		PTH (pg/ml)	-	-	
		TNF-a (pg/ml)			
		Adiponectin (pg/ml)	-	-	
		Insulin (pg/ml)			
Hentenaar et al., 2021	PIH	IL-1β (pg/ml) (median; (inter quartile range))	390.5 (87.0;555.5)	-	SSD between PIH and PIP at baseline (higher in PIP)
		IL- 6 (pg/ml) (median; (inter quartile range))	20.3 (10.3;48.9)	-	NSSD
		TNF-α (pg/ml) (median; (inter quartile range))	11.3 (7.8;16.6)	-	NSSD

		MCP-1 (pg/ml) (median; (Inter quartile range))	48.13(26.9;72.9)	-	NSSD
		MIP-1α (pg/ml) (median; (Inter quartile range))	15.63 (8.8;31.8)	-	NSSD
		MMP-8 (pg/ml) (median; (Inter quartile range))	20590.2 (13512.4;26929.4)	-	SSD between PIH and PIP at baseline (higher in PIP)
		OPG (pg/ml) (median; (Inter quartile range))	34.3 (19.3;53.0)	-	NSSD
		G-CSF (pg/ml) (median; (Inter quartile range))	0.0 (0.0;16.7)	-	NSSD
	PIP	IL-1β (pg/ml) (median; (interquartile range))	783.5 (414.0;2607.3)	738.5 (358.3;1516.8)	NSSD
		IL- 6(pg/ml) (median; (inter quartile range))	30.6 (10.6;67.4)	38.5 (11.3;76.4)	NSSD
		TNF-α (pg/ml) (median; (Inter quartile range))	13.0 (10.5;20.4)	11.5 (9.0;16.8)	NSSD
		MCP-1 (pg/ml) (median; (Inter quartile range))	58.2 (40.3;92.8)	57.6 (40.0;120.3)	NSSD
		MIP-1α (pg/ml) (median; (Inter quartile range))	10.8 (7.2;17.9)	14.9 (9.4;30.0)	SSD
		MMP-8 (pg/ml) (median; (Inter quartile range))	34829.5 (24145.0;41791.5)	36775.0 (23351.5;65973.3)	NSSD
		OPG (pg/ml) (median; (Inter quartile range))	33.9 (20.3;66.2)	32.4 (25.0;59.0)	NSSD
		G-CSF (pg/ml) (median; (Inter quartile range))	0.0 (0.0;24.0)	32.3 (26.8;41.6)	SSD
