# Supplementary material for: Biomarker Expression of Peri-Implantitis Lesions before and after Treatment: A Systematic Review
Source: Int J Environ Res Public Health. 2022 Oct 28;19(21):14085. doi: 10.3390/ijerph192114085 (PMC9659150; doi:10.3390/ijerph192114085)
Supplement: Supplementary file 1 [file ijerph-19-14085-s001.zip › SM2_Table S2_SecondaryOutcomes.pdf]

International Journal of
Environmental Research and Public Health

MDPI

Systematic review

Biomarker Expression 0f Peri-Implantitis Lesions Before and After Treatment: A Systematic Review.

Supplemental Table S2. Details of secondary outcomes of the included studies in the systematic review. *A.a: Aggregatibacter actinomycetemcomitans*, AB: Antibiotic, ALKP: Alkaline Phosphatase, AZM: Azithromycin, BI: Bleeding index, BOP: Bleeding on Probing, CAL: Clinical Attachment Loss, CCL- 5: Chemokine ligand- 5, CHX: Chlorhexidine, CRP: C- Reactive Protein, ELISA: enzyme-linked immunoassay, EMD: Enamel Matrix Derivative, F: Female, *F.n Fusobacterium nucleatum*, GI: Gingival index, GM-CSF: Granulocyte Macrophage Colony-Stimulating Factor, IFN-g: Interferon- gamma, IL-1b: Interleukin-1beta, LILT: Low Intensity Laser Treatment, LDD: Local Drug Delivery, M: Male, MBL: Marginal Bone Loss, MC: Minocycline Hydrochloride, MCP-1 : Monocyte Chemotactic Protein-1, MIP: Macrophage Inflammatory Protein , MMP: Matrix Metallo Proteinase , MPO: Myeloperoxidase, MW: Mouthwash, n: number, NR: Not Reported, NSPT: Non-surgical periodontal treatment, NSSD (No statistically significant differences p>0.05), OC: Osteocalcin, OFD: Open Flap Debridement, OPG: Osteoprotegerin, OPN: Osteopontin, PCR: Polymerase Chain reaction, PDT: Photo Dynamic Therapy, PDGF: Platelet Derived Growth factor, *P.g: Porphyromonas gingivalis*, PI: Plaque Index, PICF: Peri Implant Crevicular Fluid, PIM: peri-Implant Mucositis, PIP :Peri- implantitis, PIH: Peri-implant Health, RANK: Receptor Activator of Nuclear factor Kappa-B, RANKL: Receptor Activator of Nuclear factor Kappa-B Ligand, RBL: Radiographic Bone Loss, rCAL: relative Clinical Attachment Loss, RCT: Randomised Clinical Trial, REC: Recession, SSD (Statistically significantly p<0.05), SUP: Suppuration, *T.d: Treponema denticola, T.f.: Tanerella forsythia*, Ti: titanium, TNF: Tumour Necrosis Factor, PPD: Probing Pocket Depth, TGF: Tissue Growth Factor, VEGF: Vascular Endothelial Growth Factor.

Investigator, year	Study group	Time point	PPD (mm) (Mean ± SD)	CAL (mm) (Mean ± SD)	Recession (mm) (Mean ± SD)	RBL (mm) (Mean ± SD)	BOP (%) (Mean ± SD)	SUP (%) (Mean ± SD)	Plaque index (%) (Mean ± SD)	Microbiome	PICF volume	Others*
Bassetti et al., 2014	LDD (Control)	Baseline	4.39 ± 0.77	2.72 ± 0.72	1.68 ± 1.04	-	4.41 ± 1.47	-	0.21 ± 0.27	Except for *C. rectus* at baseline (P < 0.01), the counts in submucosal biofilm were not SSD between test and control groups at any time point.<br>*P.g* and *T.f* significantly decreased from baseline to 6 months in a-PDT group and to 12 months in LDD group.<br>At baseline, the most frequently identified species	-	- Mean BOP+ ± SD<br>- *: SS change from baseline to 3 months.<br>- †: SS change from baseline to 6 months.<br>- ‡: SS change from baseline to 9 months.<br>- §: SS change from baseline to 12 months.<br>- **: P < 0.01 compared with baseline (Wilcoxon test).
		12 months	3.83 ± 0.85 *†‡§	2.41 ± 0.70	1.41 ± 1.18 *†	-	1.55 ± 1.26 *†§	-	0.00 ± 0.00 *†‡§		-	
	a-PDT(Test)	Baseline	4.19 ± 0.55	2.66 ± 0.73	1.53 ± 0.91	-	4.03 ± 1.66		0.13 ± 0.21		-	

		12 months	4.08 ± 0.81 (N = 19) *†‡	2.58 ± 0.94 (N = 19)	1.5 ± 0.86 (N = 19) *†‡	-	1.74 ± 1.37 *†‡§	-	0.01 ± 0.04 (N = 19) *†‡§	in the submucosal biofilm were *C. gingivalis, F. nucleatum, P. micra and T. forsythia.*	-	
Bhavsar et al., 2019	PIH (Control)	Baseline	2.56 ± 0.60	-	-	-	20.8 (5/24)	0	-	-	-	
		Post-surgery	-	-	-	-	-	-				
	AF+Anti-infective therapy	Baseline	4.85 ± 1.38	-	-	-	91.7 * (22/24)	16.7 (4/24)	-	-	-	-
		Post-surgery	4.11 ± 1.27 *	-	-	-	61.9 (13/21) †	0†				*: Statistically significant difference between groups are represented as (*P*-value ≤ 0.05). † SSD before and after Tx in test group
De Mendonga et al., 2009	AF+ abrasive air powder + resin curette	Baseline	6.7 ± 1.2	-	-	-	100	30.0± 26.9	-	-	-	-
		12 months post-surgery	4.3 ± 1.0*	2.0 ± 0.64	-	-	26.6 ± 16.1*	0 *	-	-	-	*: SSD from baseline to final assessment. NSSD for - CAL red (mm), PPD reduction at 3 and 12 months.

Duarte et al., 2009	PIH	Baseline	2.0 ± 0.4	-	-	-	4.0 ± 8.7		11.8 ± 10.3	-	-	Data reported for clinical parameters mean of full mouth data for PIH group.
		3 months after TX	-				-	-	-			Data reported for PIM and PIP are reported in site level
	PIM	Baseline	5.4 ± 1.4	-	-	-	93.3 ± 14.1		85.0 ± 24.18	-	-	
		3 months post TX	3.9 ± 0.8 *				25.0 ± 41.0		28.3 ± 41.6 *†			*: SSD before and after Tx.
	PIP	Baseline	7.5 ± 2.2	-	-	-	100		90 ± 30.8	-	-	†: SSD before and after treatment
		3 months post TX	4.4 ± 1.1 *				52.5± 41.3 *		50 ± 39.4* †			
Esberg et al., 2019	No raw data for secondary outcome reported in the study.<br>Cluster 2 of the proteome was associated with implant loss and BOP.<br>Cluster 3 was associated with implant survival and EMD treatment.											
Gershenfeld et al., 2018	AZM+ NSPT	Baseline	Baseline data were counted as zero.<br>0	-	Baseline data were counted as zero.<br>0	4.98 ± 1.24	Baseline data were counted as zero.<br>0	Baseline data were counted as zero.<br>0	Baseline data were counted as zero.<br>0	Orange complex had highest frequency at all times. Red complex bacteria had lowest frequency. NSSD in bacterial at baseline between 2 groups.		No raw data reported, estimation based on the presented graphs.
		6 months	Mean change:<br>0	-	-1	4.77 ± 1.21	-55	-20	-0.15			
	Placebo +NSPT	Baseline	Baseline data were counted as zero 0	-	Baseline data were counted as zero.	6.21 ± 1.57	Baseline data were counted as zero.	Baseline data were counted as zero.	Baseline data were counted as zero.		-	-

					0		0	0	0			
		6 months	Mean change: -1	-	0.4	6.22 ± 1.39	-20	-18	-0.4		-	
Granfeldt et al., 2010	No data for secondary outcome reported in the study.											
Hallstrom et al., 2016	Probiotic supplement	Baseline	4.3	-	-	-	54	13	26	NSSD over time between groups.<br><br>The most prevalent strains were *F.n., P. micra, P. i and P. nigrescens*	-	- *: SSD compared to baseline
		26 weeks	3.7	-	-	-	14*	2	12		-	
	Placebo supplement	Baseline	4	-	-	-	58	5	32		-	
		26 weeks	3.5	-	-	-	17*	2	15		-	
Kalos et al., 2015	AZM	Baseline	-	-	-	-	-	-	-	NSSD in treatment groups among the effect of treatment in aerobic and anaerobic bacterial count.	-	
		6 months										
	Placebo	Baseline	-	-	-	-	-	-	-			
		6 months										
Komatsu et al., 2018	Laser group	Baseline	6.6 ± 1.4	6.9 ± 1.5	-	-	94.4	-	-	NSSD in both G+ and G- in laser group at any time point.	-	- *: SSD from baseline to 1 months(P=0.002).<br><br>- †: SSD from baseline to 3 months (P=0.007).
		3 months	6 ± 1.4 *†	6.4 ± 1.9	-	-	66.7	-	-			
		Baseline	6.1 ± 1.2	6.3 ± 1.7	-	-	84.2	-	-			

	Minocycline Group	3 months	5.7 ± 2.0	6.2 ± 2.0	-	-	63.2	-	-	SSD decrease of all bacterial groups at 3 months after treatment.		
Malik et al., 2015	PIH	Baseline	2	-	-	-	mBI<br>0.1	-	mPI<br>0.8	-	-	- No raw data reported, estimation carried out based on reported bar charts.
		3 months	-	-	-	-	-	-	-	-	-	-
	PIP+NSPT	Baseline	5.1	-	-	-	mBI<br>2.3	-	mPI<br>2.5	-	-	-
		3 months	4.8	-	-	-	mBI<br>0.7	-	mPI<br>0.7	-	-	-
Peres Pimentel et al., 2019	Triclosan/fluoride toothpaste	Baseline	3.1 ± 1.0	rCAL<br>9.0 ± 1.5	-	-	0.0 ± 0.0	-	6.9 ± 10.4c	-	-	-[a]: Significant intra-group differences by the ANOVA/Tukey when compared to baseline ($p < 0.05$).<br>- [b]: Significant inter-group differences by the ANOVA/ Tukey ($p < 0.05$).<br>- [c]: Significant intra-group differences by the ANOVA/Tukey when compared to baseline and 3 days ($p < 0.05$).<br>- [d]: Significant intra-group differences by the ANOVA/Tukey when compared to baseline, 3 and 7 days ($p < 0.05$).
		3 weeks	2.7 ± 0.7	rCAL<br>8.6 ± 1.3	-	-	0.0 ± 0.0	-	74.0 ± 19.1d	-	-	
	Fluoride toothpaste	Baseline	3.6 ± 0.5	rCAL<br>9.2 ± 1.1	-	-	0.0 ± 0.0	-	9.8 ± 13.2	-	-	
		3 weeks	3.7 ± 0.9	rCAL<br>9.2 ± 1.1	-	-	6.9 ± 16.8	-	75.5 ± 17.7	-	-	
Renvert et al., 2017	Overall	Baseline	6.4	-	-	Bone level:	100	41.5	-	-	-	-

						5.6						
		6 months	5.5	-	-	Bone level: 5.8	63	12.2	-	-	-	-
Ribeiro et al., 2018	Triclosan-fluoride toothpaste	Baseline	3.1 ± 0.8	8.9 ± 1.1	-	-	0.0 ± 0.0	-	9.1 ± 14.3	-	-	Different capital letters represent significant intergroup differences by ANOVA/Tukey (*P* < 0.05). Different non-capital letters represent significant intragroup differences by ANOVA/Tukey (*P* < 0.05).
		3 weeks	3.6 ± 0.9	9.2 ± 1.1	-	-	8.4 ± 10.1	-	56.9 ± 19.0	-	-	
	Fluoride toothpaste	Baseline	3.1 ± 1.2	8.3 ± 1.2	-	-	0.0 ± 0.0	-	12.8 ± 12.7	-	-	
		3 weeks	3.4 ± 0.9	8.9 ± 1.0	-	-	13.6 ± 17.2	-	61.1 ± 23.7	-	-	
Thierbach et al., 2016	Healthy/Gingivitis	Baseline	Total PD (Mean (SD)) 2.9 (0.4)	-	-	-	15.8 (12.3)	-	-	-	-	-
		6 months	-	-	-	-	-	-	-	-	-	-
	Periodontitis	Baseline	3.5 (0.8)	-	-	-	28.3 (14.8)	-	-	-	-	
		6 months	-	-	-	-	-	-	-	-	-	-

Wohlfahrt et al., 2014	PIP TX with porous Ti	No clinical data per group is reported in study.<br>Overall no differences in clinical parameter between test and control group were found.										
	PIP Tx without porous Ti											
Hentenaar et al., 2021	PIH		1.9 (±0.6)	-	-	0.56 (±0.5)	3.4	0	NR	NR	0.14	SSD between PIH and PIP at baseline in terms of PPD and MBL, no SSD at baseline and after 3 months in PIP group
	PIP	Baseline	5.0 (±1.1)	-	-	4.17 (±1.75)	58.4	19.2	17.3		0.42	
		3 months	4.7 (±1.3)	-	-	4.24 (±1.84)	47.5	20.8	6.6		0.39	
