# Supplementary material for: Biomarker Expression of Peri-Implantitis Lesions before and after Treatment: A Systematic Review
Source: Int J Environ Res Public Health. 2022 Oct 28;19(21):14085. doi: 10.3390/ijerph192114085 (PMC9659150; doi:10.3390/ijerph192114085)
Supplement: Supplementary file 1 [file ijerph-19-14085-s001.zip › SM3_Table S3_RiskOfBias.pdf]

International Journal of
Environmental Research and Public Health

MDPI

Systematic review

Biomarker Expression of Peri-Implantitis Lesions Before and After Treatment: A Systematic Review.

Supplemental Table S3. Risk of bias and applicability judgements in QUADAS-2. * Low risk =! ; High risk = + ; Unclear risk = ?

Study	Risk of bias*				Applicability concerns*		
	**Participant Selection**	**Index test**	**Reference standard**	**Flow and timing**	**Participant Selection**	**Index test**	**Reference standard**
Bassetti et al., 2014	!	+	+	!	!	!	+
Bhavsar et al., 2019	+	+	!	!	!	!	!
De Mendonga et al., 2009	+	?	+	!	?	?	+
Duarte et al., 2009	+	+	+	!	?	?	+
Esberg et al., 2019	?	?	!	!	!	!	!
Gershenfeld et al., 2018	?	+	!	!	!	!	!
Granfeldt et al., 2010	+	+	+	!	?	?	+
Hallstrom et al., 2016	!	+	+	!	!	!	+
Kalos et al., 2015	?	+	+	!	!	!	+
Komatsu et al., 2018	!	+	!	!	!	!	!
Malik et al., 2015	+	+	+	!	?	?	+
Peres Pimentel et al., 2019	?	+	!	!	!	!	!
Renvert et al., 2017	+	+	?	!	!	!	?
Ribeiro et al., 2018	?	+	+	!	!	!	+
Thierbach et al., 2016	+	+	+	!	?	?	?
Wohlfahrt et al., 2014	!	+	+	!	!	!	?
Hentenaar et al., 2021	?	+	+	!	!	!	?
