# Supplementary material for: Biomarker Expression of Peri-Implantitis Lesions before and after Treatment: A Systematic Review
Source: Int J Environ Res Public Health. 2022 Oct 28;19(21):14085. doi: 10.3390/ijerph192114085 (PMC9659150; doi:10.3390/ijerph192114085)
Supplement: Supplementary file 1 [file ijerph-19-14085-s001.zip › SM4_Table S4_ExcludedStudies .pdf]

International Journal of
Environmental Research and Public Health

MDPI

Systematic review

Biomarker Expression of Peri-Implantitis Lesions Before and After Treatment: A Systematic Review.

Supplemental Table S4. Excluded studies (title, author and publication year) following eligibility criteria assessment.

Study not in English		
Title	Author	Publication Year
Differenzierte Untersuchung der lokalen und systemischen Entzündungsreaktionen nach Implantation von Niedertemperatur-Plasmapolymer-modifizierten Titanplättchen im Tiermodell Ratte	Walschus, Uwe	2013
Avaliação comparativa da microinfiltração bacteriológica na interface pilar/implante em implantes hexágono externo com 10 e 20 Ncm de torque	Neves, Flávio Domingues; Carneiro, Thiago de Almeida Prado Naves; Prudente, Marcel Santana; Neto, João Paulo Silva; Penatti, Mário Paulo Amante; Prado, Ricardo Alves	2010
Orale Rehabilitation eines atrophischen Oberkiefers mit intraoralen Knochentransplantaten	Berthold, Michael; Doliveux, Romain; Khoury, Fouad	
Carga inmediata en implantes dentales	Concejo Cútoli, C.; Montesdeoca García, N.	2005
Procjena stabilnosti implantata nakon podizanja dna sinusa i primjene različitih nadomjesnih materijala	Jelušić, Damir; Puhar, Ivan; Plančak, Darije	2014
CHARAKTERISIERUNG POTENTIELLER	HINSICHTLICH, KNOCHENERSATZ; EIGENSCHAFTEN, ZYTOTOXISCHER	
Ocena w\lasności fizycznych i biologicznych samoorganizującego się biomateria\lu otrzymanego z chitozanu/fosforanu wapnia/alginianu do zastosowań stomatologicznych	Kucharska, MARTYNA; Bubak, GRZEGORZ; Kowalczyk, MAGDALENA; Bojar, WITOLD; Brynk, TOMASZ; Koperski, \LUKASZ; Ciach, TOMASZ	2011
Actae of the 54 Congrès du GIRSO (Montpéllier, 2010)	Céspedes, Maria Cristina Manzanares	2010
Biverkningar av dentala implantat i titan och zirkoniumdioxid: en systematisk översikt av vetenskapliga studier	Berglund, Anders; Konradsson, Katarina	2014
Der Einfluss des Implantat Designs von Mini Dental Implants auf die Belastung der umgebenden Knochenstruktur: eine Finite-Elemente-Analyse	Toth, Arpad Alexander	2018
Marginální přesnost tří typů keramickỳch abutmentũ	Baldassarri, Marta; Hjerppe, Jenni; Romeo, Davide; Fickl, Stefan; Thompson, Van P.; Stappert, Christian FJ	
AöIZ øÇø CANDIDA ENFEKSøYONLARI VE TEDAVøSø	ÖNDER, Canan	
세라믹과 지르코니아의 골유착에 관한 고찰	송영균	2012
Dentala kompositmaterials inverkan p\a a närliggande celler.	Nikolovska, Emilia; Rivera, Maria	2011
DA Deporter	Campelo, L. Domínguez	2002
Síndrome de colapso de mordida posterior	Baldión, Paula Alejandra; Castro, Diego Enrique Betancourt	2012
牙种植体的生物力学研究.	甘雪琦; 肖宇; 马瑞阳; 黄椿棚; 吴尧; 杨帮成; 杨齐; 包崇云; 于海洋	2019
Rehabilitación del maxilar superior con atrofia severa horizontal utilizando implantes dentales palatinizados.	Candel Martí, Eugenia	2016
Evaluación de la micro-deformación de la zona maxilar anterior con regeneración. Análisis de elementos finitos	Henao, Johan Ivan Aguilar; Ossa, Junes Abdul Villarraga; Correa, Federico Latorre	
Durchmesserreduzierte Implantate Eine Übersicht	Schiegnitz, Eik; Al-Nawas, Bilal	2013
Reabilitação de maxila atrófica sem uso de abordagem reconstrutiva. Relato de caso.	Tabosa e Silva, Tércio Pessoa; Miranda, Caio César Leite	2014
BIOFILM W ORTOPEDII ORAZ W CHIRURGII SZCZĘKOWOTWARZOWEJ–ZNACZENIE ZJAWISKA I MODYFIKACJE BIOMATERIA\LÓW OGRANICZAJĄCE CZĘSTOŚĆ ZAKAŻEŃ.	REETZ, ANDRZEJ; JUNKA, ADAM; BARTOSZEWICZ, MARZENNA	2016
Anàlisi numèrica de tensions i deformacions en un implant dental	Serra Cantarell, Anna; Sarrá Paloma, Maria	2017
Análisis de la pérdida marginal ósea temprana en implantes colocados en hueso injertado frente a implantes colocados en hueso pristino	Fernández Jiménez, Andrés	2016
チタンの超親水性処理がラミニン 332 の吸着特性に及ぼす影響	柴垣博一; 野本秀材; 野村智義; 老川秀紀; 吉成正雄	2018
Lifetime Achievement Award—2005 Prix de mérite à vie—2005	Simmons, Edward Henry	1982
Comportamento do biofilme bacteriano em superfícies de implantes com diferentes tratamentos de superfície	Castro, Maura Filipa Fernandes de	2019
Rugosidad superficial del zirconio para implantes dentarios y la adhesión de biofilm	Butler, Teresa Adela; Lazo, Sergio Daniel; Basal, Roxana Lía; Escudero Giachella, Ezequiel; Friso, Nélida Ester;	2014

	Viscovick, Cristina; Alfaro, Gabriel Enrique; Amaro, Emilio Gustavo; Merlo, Diego; Belloni, Federico	
Etiologia zakażeń oko\lowszczepowych w implantologii stomatologicznej cz. 2-rola w\laściwości fizykochemicznych powierzchni implantu”	i Ceramiki, Wydzia\l Inżynierii Materia\lowej	
A influência do tabagismo na aposição óssea peri-implantar ao redor de micro-implantes removidos de maxilares humanos.	FERREIRA, José Divino Bezerra	2012
Evaluación clínica del porcentaje de éxito de implantes realizados en la Facultad de Odontología de Universidad Andrés Bello según el criterio de pisa del año 2009	Leyton Sepúlveda, Rubén Alejandro	2015
Quais as indicações para membranas absorvíveis e não absorvíveis?	Benfatti, César Augusto	
Caracterización morfológica y Evaluación clínica de sustitutos óseos de origen porcino de la casa 3Biomat para su aplicación en lesiones óseas bimaxilares	Nausa, Julian Gallón; Haiek, Diego Ernesto Castro	2017
최후방 하악 구치 임프란트 지지 단일 수복물에서 인접치와의 거리가 합병증에 미치는 영향	신혜승; 김명래; 이재훈	2009
Pedro Martín Vera Juan Manuel Aragoneses Lamas	Lamas, Juan Manuel Aragoneses	2012
ПЕРИИМПЛАНТИТ–ОСНОВНОЕ ОСЛОЖНЕНИЕ ДЕНТАЛЬНОЙ ИМПЛАНТАЦИИ (ОБЗОР ЛИТЕРАТУРЫ)	Блинова, А. В.; Рюмшин, Р. А.; Румянцев, В. А.	
Importancia de la pérdida marginal ósea como criterio de éxito en implantología a corto plazo	León Cano, Ana Isabel	2016
POLİETER ETER KETON (PEEK) ve DENTAL KULLANIMI	ÇULHAOĞLU, Ahmet Kürşat; ÖZKIR, Serhat Emre; TÜRKKAL, Fatoş	
Implante imediato com provisionalização imediata através de cicatrizador multifuncional de PEEK	Passoni, Bernardo Born; Venâncio, Fernanda; Formiga, Márcio de Carvalho; Schuldt Filho, Guenther; Magini, Ricardo de Souza; Benfatti, César Augusto Magalhães	2017
Análise fotoelástica da distribuição de tensões peri-implantares em overdenture mandibular retida por implante unitário com diferentes tipos de conexão	Nascimento, João Francisco Machado do	2013
Utjecaj socijalno-ekonomskog statusa, pušenja i zdravstvenog statusa pacijenata na neuspjeh implantološke terapije	Chatzopoulos, Georgios S.; Wolff, Larry F.	2018
Dental İmplant Yüzey Özellikleri ve Biyolojik Ortamla Etkileşimler: Bölüm II: İmplant Yüzeyleri ve Özellikleri	KILINÇ, Yeliz; ERGÜVEN, Sara SAMUR; ERKMEN, Erkan	
Biofunctionalization of Titanium Granules with Simvastatin for Improving Osteogenic Activity and Antibacterial Properties (Ex Vivo Study).	Karaji, Zahra Gorgin; Houshmand, Behzad; Abbasi, Shahsanam; Shafiei, Sara; Faghihi, Shahab	2017
Sofortfunktion in der zahnärztlichen Praxis	Siebers, D.; Gehrke, P.; Schliephake, H.	2011
Seleção do diâmetro do implante em alvéolos pós-exodontia: uma nova abordagem.	da ROSA, José Carlos Martins; Rosa, Ariádene Cristina Pértile de Oliveira; Francischone, Carlos Eduardo; Sotto-Maior, Bruno Salles	2014
Influência do fenótipo gengival na saúde e na estética peri-implantar	Mata, Vanessa Couto da	2017
Mateus Bertolini Fernandes dos Santos	Zen, Bruno Massucato; Bacchi, Atais	2016

Preclinical Study		
Bone response to titanium alloy implants placed in diabetic rats.	McCracken, Michael; Lemons, Jack E.; Rahemtulla, Firoz; Prince, Charles W.; Feldman, Dale	2000
RADIOGRAPHICAL AND IMMUNOHISTOCHEMICAL EVALUATION OF SILVER NANOPARTICLES IN TREATMENT OF INTRA-OSSEUS DEFECTS. AN ANIMAL STUDY	Basha, Soha M.; Shawky, Heba A.; Hanafi, Rania	
The Effect of Different Implant Surfaces and Photodynamic Therapy on Periodontopathic Bacteria Using TaqMan PCR Assay following Peri-Implantitis Treatment in Dog Model	Madi M.; Alagl A.S.	2018

Biomechanic study		
Biomechanics of the Wrist	Patterson, Rita; Viegas, Steven F.	1995

Bone graft material		
Densitometric analysis of prf vs. xenograft for sinus augmentation procedures–2 years follow-up	Sušić, Mato; Blašković, Marko; Brozović, Juraj; Gikić, Matija; Zore, Irina Filipović; Katanec, Davor; Granić, Marko; Pandurić, Dragana Gabrić	2013

Dental material study		
2.8 Assessment of Immuno-Allergological Properties of Ceramic and Metallic Compounds In Vitro	Thomas, P.; Barnstorf, S.; Summer, B.	2001

Imaging technology		
Virtual Dental Patient: A 3D Oral Cavity Model and its Use in	Nikolaidis, Nikos; Marras, Ioannis; Mikrogeorgis, Georgios; Lyroudia, Kleoniki; Pitas, Ioannis	2009
Osseo-integration assessment by means of digital panoramic radiography and cone beam CT due to platelet rich plasma employment and graft placement in jaw bone defects	Γε$ømega$ργακόπουλος, I$ømega$$\acute\alpha$ννης	2015

Dental material		
Effect of scaffold design on bone morphology in vitro	Uebersax, Lorenz; Hagenmüller, Henri; Hofmann, Sandra; Gruenblatt, Emanuel; Müller, Ralph; Vunjaknovakovic, Gordana; Kaplan, David L.; Merkle, H. P.; Meinel, Lorenz	2006
Characterization of the surface properties of commercially available dental implants using scanning electron microscopy, focused ion beam, and high-resolution transmission electron microscopy	Jarmar, Tobias; Palmquist, Anders; Br\a anemark, Rickard; Hermansson, Leif; Engqvist, H\a ak\a an; Thomsen, Peter	2008

Not relevant to peri-implant disease		
Water flow on erbium: yttrium–aluminum–garnet laser irradiation: effects on dental tissues	Colucci, Vivian; Do Amaral, Flávia Lucisano Botelho; Pécora, Jesus Djalma; Palma-Dibb, Regina Guenka; Corona, Silmara Aparecida Milori	2009
Diode laser vs. conventional technique for second stage surgery–a pilot study	Pandurić, Dragana Gabrić; Sušić, Mato; Brozović, Juraj; Žagar, Maja; Katanec, Davor; Jurič, Ivona Bago; Zore, Irina Filipović	2013
Rehabilitation of patients with extreme mandibular alveolar ridge atrophy by means of slim and short mini dental implants and mandibular overdenture	Kovačić, Ines; Kiršić, Sanja Peršić; Čelebić, Asja	2017
Osseo-integration assessment by means of digital panoramic radiography and cone beam CT due to platelet rich plasma employment and graft placement in jaw bone defects	Γε$ømega$ργακόπουλος, I$ømega$$\acute\alpha$ννης	2015
Vertical Bone Augmentation Using Deproteinized Bovine Bone Mineral, Absorbable Collagen Sponge, and Recombinant Human Bone Morphogenetic Protein-2: An In Vivo Study in Rabbits.	Kim, Yeon Jung; Scaf de Molon, Rafael; Horiguti, Fausto Rioiti; Piragine Contador, Guilherme; Antonio Coelho, Marco; Ibiapina Mascarenhas, Vinicius; Paula de Souza Faloni, Ana; Cirelli, Joni Augusto; Sendyk, Wilson Roberto	2018
MRI and MRS investigation of patients with artificial hip joints at 3 T	Rzanny, R.; Sander, K.; Hiepe, P.; Gussew, A.; Roth, A.; Kinne, R. W.; Reichenbach, J. R.	2012
Biomaterials	Stevens, Molly; Sardinha, Jose; Lim, Erh-Hsuin	2011
Arytenoid adduction as an adjunct to type I thyroplasty for unilateral vocal cord paralysis	Kraus, Dennis H.; Orlikoff, Robert F.; Rizk, Samieh S.; Rosenberg, David B.	1999
Effects of new probiotic mouthwash in patients with diabetes mellitus and cardiovascular diseases	Bollero, P.; Di Renzo, L.; Franco, R.; Rampello, T.; Pujia, A.; Merra, G.; De Lorenzo, A.; Docimo, R.	2017
ECHTES TEAMWORK	Zwanzig, Kai; Hannker, Ztm Christian	
Influence of Frontal and Sagittal Position of Total DiscArthroplasty on Clinical Outcomes at 3 Years Follow-Up	Aunoble, S.; Le Huec, J. C.	2007
Current problems associated with toxicity evaluation of medical device materials and future research needs	Northup, Sharon J.	1989
Osteoprotegerin (OPG) gene therapy in animal models of osteoarticular disease	Bolon, B.; Carter, C.; Daris, M.; Morony, S.; Campagnuolo, G.; Feige, U.; Sheng, J.	2001
Tension band stabilization of fractures and luxations of the thoracolumbar vertebrae in dogs and cats: 38 cases (1993–2002)	Voss, Katja; Montavon, Pierre M.	2004
Experimental evaluation of peritoneum and pericardium as dural substitutes	Gök, Abdülvahap; Zorludemir, Suzan; Polat, Sait; Tap, Özgül; Kaya, Mehmet	1995
The rheumatoid wrist	Stanley, J. K.	1991
Fossils as Candidate Material for Orthopedic Applications	Pesenti, Hector; Leoni, Matteo; Motta, Antonella; Scardi, Paolo	2011
Termical and voluminous bone effect comparison between Er-YAG laser and cortical drill-an experimental study	Pandurić, Dragana Gabrić; Anić, Ivica; Katanec, Davor; Žabkar, Janez; Ban, Ticijana; Kuna, Tihomir; Sušić, Mato	2009
Biomaterials and Immune Response: Complications, Mechanisms and Immunomodulation	Vrana, Nihal Engin	2018
Long term results of limb salvage with the Fabroni custom made endoprosthesis	Fabroni, Roberto H.; Castagno, Aldo; Aguilera, Antonio L.; Steverlynck, Alejandro M.; Zeballos, Joaquin	1999
Methodology protocol for assess systemic and local effects of LLLT on osseointegration of dental implants in the rabbits jaws	Mayer, Luciano; Oliveira, Marília Gerhardt de; Massotti, Fabrício P.; Gomes, Fernando V.; Guyoti, Viviane; González, Félix HD; Weber, João BB	2013
Genotype Analysis Identifies the Cause of the 'Royal Disease'	Organization, WHO: World Health; Kaminski, R.; Bella, R.; Yin, C.; Otte, J.; Ferrante, P.; Gendelman, H. E.; Li, H.; Booze, R.; Gordon, J.	2017
On various protocols for direct loading of implant-supported fixed prostheses	Östman, Pär-Olov	2007
Treatment of Failed Hip Resurfacing	Amstutz, Harlan C.; Ball, Scott T.	2008
Handbook of biomaterial properties	Black, Jonathan; Hastings, Garth	2013
Applications of titania nanotubes in bone biology	Nair, Manitha; Elizabeth, Elmy	2015

PET Imaging of Osteomyelitis. Feasibility of 18F-FDG, 68Ga-Chloride and 68Ga-DOTAVAP-P1 Tracers in Staphylococcal Bone Infections	Lankinen, Petteri	2013
Hydrophile Implantatoberflächen	Bosch, Gabriel; Stübinger, Stefan; Rücker, Martin; Stadlinger, Bernd	
Effect of surface characteristics on cellular adherence and activity	Dorkhan, Marjan	2014
Management peri	KÖSTERS, C.; WÄHNERT, D.; SCHLIEMANN, B.; STANGE, R.; RASCHKE, MJ	
Sinus augmentation using rhBMP-2-loaded synthetic bone substitute with simultaneous implant placement in rabbits	주명재	2017
Polydopamine-assisted functionalization of heparin and vancomycin onto microarc-oxidized 3D printed porous Ti6Al4V for improved hemocompatibility, osteogenic and anti-infection potencies	Zhang, Teng; Zhou, Wenhao; Jia, Zhaojun; Wei, Qingguang; Fan, Daoyang; Yan, Jianglong; Yin, Chuan; Cheng, Yan; Cai, Hong; Liu, Xiaoguang	2018
20: The cost of anatomic versus semi-constrained (reverse) shoulder arthroplasty	Collins, David N.	2007
One-step fabrication of AgNPs embedded hybrid dual nanofibrous oral wound dressings	Lee, Sang Jin; Heo, Dong Nyoung; Lee, Donghyun; Heo, Min; Rim, Hyunjoon; Zhang, Lijie Grace; Park, Su A.; Do, Sun Hee; Moon, Ji-Hoi; Kwon, Il Keun	2016
The infected total knee arthroplasty	Habermann, Edward T.	1991
Driving tissue morphogenetic cascades using tunable nanolayered surface coatings	Shah, Nisarg Jaydeep	2014
Haemocompatibility of Medical Devices	van Oeveren, W.	2008
Calcium phosphate bioceramics	Arcos, Daniel	2014
Nutritional pharmacology and malignant disease: a therapeutic modality in patients with cancer	Heys, S. D.; Gough, D. B.; Khan, L.; Ermin, O.	1996
Endometrial thickness is not independent of luteal phase day in a rural Polish population	Clancy, Kathryn BH; Ellison, Peter T.; Jasienska, Grazyna; Bribiescas, Richard G.	2009
Biomaterials for musculoskeletal regeneration	Basu, Bikramjit; Ghosh, Sourabh	2017
Biological effects of metal degradation in hip arthroplasties	Granchi, Donatella; Savarino, Lucia Maria; Ciapetti, Gabriela; Baldini, Nicola	2018
Establishment and clinical application of a novel design protocol for custom-made titanium devices in alveolar bone augmentation for dental implants	Otawa, N.; Sumida, T.; Nakano, H.; Yamada, T.; Mori, Y.	2014
Numerical investigation of bone remodelling around immediately loaded dental implants using sika deer (Cervus nippon) antlers as implant bed	He, Yun; Hasan, Istabrak; Keilig, Ludger; Fischer, Dominik; Ziegler, Luisa; Abboud, Marcus; Wahl, Gerhard; Bourauel, Christoph	2018
Dental Implant Macro-Design Features Can Impact the Dynamics of Osseointegration	Judith De Rycker, M. D.; Bart Van Meerbeek, D. D. S.; Ignace Naert, D. D. S.	
Comparative 3D FEM analysis of three different dental implant shapes	Stanis\law, K. U. T.	
Pre-implant Surgical Interventions with Focus on the Maxilla	Protocol, Early Surgical	2008
Implants in the Posterior Maxilla: Open Sinus Lift Versus Conventional Implant Placement. A Systematic Review.	Romero-Millán, Javier; Aizcorbe-Vicente, Javier; Peñarrocha-Diago, Maria; Galindo-Moreno, Pablo; Canullo, Luigi; Peñarrocha-Oltra, David	2019
Periodontal-Restorative Interactions	Dumitrescu, Alexandrina L.; Okada, Mitsugi; Inagaki, Koji	2010
Overtreatment: an efective alternative in implant therapy on a single tooth with root resorption in patient with chronic periodontitis	CAMPOS, André Luiz Oliveira; SHIBLI, Jamil Awad; IUROVSCHI, Ronaldo; WATINAGA, Sidney Eiji	2015
Soft Tissue Attachment to Titanium Implants Coated with Growth Factors	Marino, Victor	
Patterns of stress and strain in complete-arch prostheses supported by four or six implants: A literature review of finite element analyses	Valian, Nasrin Keshavarz; Ardakani, Mohammad Reza Talebi; Ahari, Alireza Aziz; Taghi, Mohammad	2018
The Implant Design and Biological Response	DESIGN, INFLUENCES OF IMPLANT	2008
Short implants to retain/support overdentures in extremely resorbed mandibles with different transverse widths: a finite element analysis	Erbasar, Güzin Neda Hasanoglu; Soganci, Gokce; Aykent, Filiz; Ertem, Sinan Yasin	2019
Towards the Optimal Crown-to-Implant Ratio in Dental Implants	Sego, T. J.; Hsu, Yung-Ting; Chu, Tien-Min Gabriel; Tovar, Andres	2017
Nordic contribution to dental and orofacial sciences	Könönen, Eija; Lynge Pedersen, Anne Marie	2018
Clinical and Radiological Evaluation of Screw-retained and Cement-retained Single-implant Restorations-A Comparative Study	Francis, Litty; Pillai, S. Babukuttan; Lylajam, S.	
Four Stable and Functioning Dental Implants Retrieved for Fracture After 14 and 17 Years from the Same Patient: A Histologic and Histomorphometric Report.	Botticelli, Daniele; Perrotti, Vittoria; Piattelli, Adriano; Iezzi, Giovanna	2019
Clinical and radiographic assessment of implant-supported rehabilitation of partial and complete edentulism: a 2 to 8 years clinical follow-up	Manicone, P. F.; Passarelli, P. C.; Bigagnoli, S.; Pastorino, R.; Manni, A.; Pasquantonio, G.; D'Addona, A.	2018
Case Report Mandibular reconstruction with single barrel vascular free fibula flap and implants-borne fixed prosthesis: usage of a modified healing abutment	Si, Jiawen; Li, Hongliang; Wang, Minjiao; Ren, Rong; Fei, Jiongmei; Shen, Steve GF; Shi, Jun	2017
Research Article Comparative Evaluation of Osseointegrated Dental Implants Based on Platform-Switching Concept: Influence of Diameter, Length, Thread Shape, and In-Bone Positioning Depth on Stress-Based Performance	Vairo, Giuseppe; Sannino, Gianpaolo	

rhBMP-2 for Alveolar Bone Reconstruction in Implant Dentistry	Hanisch, Pr Ulf Wikesjo1-Dr Oliver; Danesh-Meyer, Pr Michael J.	2000
Implant Abutment Selection Criteria	Mumcu, E.; Erdinc, G.	2018
Biological behavior of titanium, zirconia or PEEK dental implant-abutments	Sordi, M. B.; Sarwer-Foner, S. N. D.; Schünemann, F. H.; Apaza-Bedoya, K.; Juanito, G. M. P.; Henriques, B.; Magini, R. S.; Benfatti, C. A. M.	2019
High-Performance Polymers and Their Potential Application as Medical and Oral Implant Materials: A Review	Ti, Titanium	2015
Novel Techniques in Dentoalveolar and Implant Surgery	Motamedi, Mohammad Hosein Kalantar; Hassani, Ali	2016
Risk Factors and Early Survival Rate of Biomet 3i Dental Implants. A Retrospective Study	Aghdam, Mina Kahyaie	
Strain distribution of immediately loaded implants with fixed prosthesis using the "All-on-Four" protocol in the edentulous mandible: nonlinear three-dimensional finite element analysis	Horita, S.; Sugiura, T.; Yamamoto, K.; Kawakami, M.; Murakami, K.; Kirita, T.	2014
Implant Surgery Interventions	IMPLANT, OPTIMAL DENTAL	2008
The experimental study of HA-Coated titanium dental implant in dogs	Li, Liu	1996
Clinical Significance of Immediate implant placement	Sakamoto, Y.	2014
Peri-implant alveolar bone resorption in an innovative peri-implantitis murine model: Effect of implant surface and onset of infection	Varon-Shahar, Einat; Shusterman, Ariel; Piattelli, Adriano; Iezzi, Giovanna; Weiss, Ervin I.; Houri-Haddad, Yael	2019
Porous polymethylmethacrylate cement: development and evaluation of a potential implant material	de Wijn, Joost Robert	1982
An Unusual Bone Loss Around Implants.	Reza Rokn, Amir; Sajedinejad, Neda; Yousefyfakhr, Hosnieh; Badri, Samare	2013
The influence of implant-abutment connection to peri-implant bone loss: A systematic review and meta-analysis	Paolo Ghensi, D. D. S.; Dent, Oral Surgery MClin	
Effect of Platform switching on peri-implant tissues: A review	Baig, Nazish; Kadam, Pranit; Yeshwante, Babita; Mhaske, Maya; Jadhav, Vivek	2015
Current Protocols for the Treatment of Peri-implantitis	Garaicoa-Pazmino, Carlos; Sinjab, Khaled; Wang, Hom-Lay	2019
Influence of bone density on peri-implant bone strain distribution in an immediately loaded implant	Sugiura, T.; Yamamoto, K.; Horita, S.; Kawakami, M.; Murakami, K.; Kirita, T.	2014
Circularly polarized light standards for investigations of collagen fiber orientation in bone	Bromage, Timothy G.; Goldman, Haviva M.; McFarlin, Shannon C.; Warshaw, Johanna; Boyde, Alan; Riggs, Christopher M.	2003
Management of loading forces on mandibular distal-extension prostheses. Part I: Evaluation of concepts for design	Monteith, Brian D.	1984
Virtual implant planning in the edentulous maxilla: criteria for decision making of prosthesis design	Avrampou, Marianna; Mericske-Stern, Regina; Blatz, Markus B.; Katsoulis, Joannis	2013
In vivo evaluation of biodegradability and biocompatibility of Fe30Mn alloy	Traverson, Marine; Heiden, Michael; Stanciu, Lia A.; Nauman, Eric A.; Jones-Hall, Yava; Breur, Gert J.	2018
Current Status of Dental Implants: A Periodontal Perspective	PERIODONTICS, OF	2000
Biomechanical in vitro evaluation of two full-arch rehabilitations supported by four or five implants.	Francetti, Luca; Cavalli, Nicolo; Villa, Tomaso; La Barbera, Luigi; Taschieri, Silvio; Corbella, Stefano; Del Fabbro, Massimo	2015
Textbook of prosthodontics	Nallaswamy, Deepak	2017
Guided Bone Regeneration for Dental Implants	Weshler, Mishel; Antoniac, Iulian Vasile	2014
Simplified Protocol for Relining Provisional Prosthesis on Natural Abutments: A Technical Note.	Galli, Fabio; Deflorian, Matteo; Testori, Tiziano	2018
On the influence of biochemical coating on implant bone incorporation	Bougas, Kostas	2012
Vertical bone augmentation with simultaneous dental implantation using crestal biomaterial rings: a rabbit animal study	Draenert, Florian G.; Kämmerer, Peer W.; Palarie, Victor; Wagner, Wilfried	2012
Carga inmediata en implantes dentales	Concejo Cútoli, C.; Montesdeoca García, N.	2005
Síndrome de colapso de mordida posterior	Baldión, Paula Alejandra; Castro, Diego Enrique Betancourt	2012
Rehabilitación del maxilar superior con atrofia severa horizontal utilizando implantes dentales palatinizados.	Candel Martí, Eugenia	2016
Evaluación de la micro-deformación de la zona maxilar anterior con regeneración. Análisis de elementos finitos	Henao, Johan Ivan Aguilar; Ossa, Junes Abdul Villarraga; Correa, Federico Latorre	
ARTHRITIS AND OTHER JOINT DISEASES: TRANSLATIONAL AND CLINICAL	Nanoparticle, Iron Oxide; During, MRI Approach	2015
Arytenoid adduction as an adjunct to type I thyroplasty for unilateral vocal cord paralysis	Kraus, Dennis H.; Orlikoff, Robert F.; Rizk, Samieh S.; Rosenberg, David B.	1999
Synthesis, characterization, and bioactivity of SrTiO 3-incorporated titanium coating	Sahoo, Souvik; Sinha, Arijit; Balla, Vamsi Krishna; Das, Mitun	2018
牙种植体周围微生物研光.	程磊; 于海洋; 吴尧; 包崇云; 杨帮成; 满毅; 孙瑶; 岩晓丽; 周学东	2019
Joshua Jacobs	Levine, Jonathan Black Brett	2007

Análise da Resposta Tecidual Perimplantar em Regiões com Baixa Densidade Óssea Analysis of Peri-implant Tissue Response in Areas with a Low Bone Density	Mayer, Luciano	
Bone Tissue Responses to Zirconia Implants Modified by Biomimetic Coating Incorporated with BMP-2.	Teng, Fei; Zheng, Yuanna; Wu, Gang; Beekmans, Bart; Wismeijer, Daniel; Lin, Xingnan; Liu, Yuelian	2019
Current Concepts for the Basis of Dental Biological Implants	Equilibrium, Foreign Body	2015
Dermal zygomatic peri-implant reaction—gene expression?	Sampaio-Fernandes, M. A.; Tovim, L.; Rau, L.; Silva, C.; Sampaio-Fernandes, J. C.; Vaz, P.	2017
Treatment of Implant Surfaces: Insights, Outcomes and Limitations	Corrêa, Cássia Bellotto; Ribeiro, Ana Lúcia Roselino; Moretti, Lívia Alves Corrêa; Consolaro, Alberto; Junior, Elcio Marcantonio	2015
Peri-implant Mucosal Tissues and Inflammation: Clinical Implications.	Fiorellini, Joseph P.; Luan, Kevin WanXin; Chang, Yu-Cheng; Kim, David Minjoon; Sarmiento, Hector L.	2019
Hard tissue-biomaterial interactions	Korkusuz, Petek; Korkusuz, Feza	2004
Applications of medical implant materials	Drummond, Colin K.; Khan, Fahd R.	2012
Cellular proliferation and macrophage populations associated with implanted expanded polytetrafluoroethylene and polyethyleneterephthalate	Hagerty, R. Daniel; Salzmann, Dennis L.; Kleinert, Leigh B.; Williams, Stuart K.	2000
Synergetic inactivation of Staphylococcus epidermidis and Streptococcus mutansin a TiO2/H2O2/UV system	Unosson, Erik; Tsekoura, Eleni K.; Engqvist, H\a akan; Welch, Ken	2013
Surgical treatment of periodontal intrabony defects with calcium sulfate implant and barrier versus collagen barrier or open flap debridement alone: a 12-month randomized controlled clinical trial	Paolantonio, Michele; Perinetti, Giuseppe; Dolci, Marco; Perfetti, Giorgio; Tetè, Stefano; Sammartino, Gilberto; Femminella, Beatrice; Graziani, Filippo	2008
Systematic review of pre-clinical models assessing implant integration in locally compromised sites and/or systemically compromised animals	Thoma, Daniel S.; Martin, Ignacio Sanz; Mühlemann, Sven; Jung, Ronald E.	2012
Top-Cited Articles in Implant Dentistry.	Fardi, Anastasia; Kodonas, Konstantinos; Lillis, Theodoros; Veis, Alexander	2017
Prosthetic gingival reconstruction in the fixed partial restoration. Part 2: diagnosis and treatment planning.	Salama, Maurice; Coachman, Christian; Garber, David; Calamita, Marcelo; Salama, Henry; Cabral, Guilherme	2009
Bone formation on biomimetic calcium phosphate-coated and zoledronate-immobilized titanium implants in osteoporotic rat tibiae.	Pyo, Sung Woon; Kim, Young Mi; Kim, Chul Seung; Lee, In Seop; Park, Je Uk	2014
Clinical applications of polytetrafluoroethylene (PTFE) tape in restorative dentistry	Sattar, M. M.; Patel, M.; Alani, A.	2017
Suture materials affect peri-implant bone healing and implant osseointegration	Villa, Oscar; Lyngstadaas, Staale P.; Monjo, Marta; Satué, Maria; Rønold, Hans J.; Petzold, Christiane; Wohlfahrt, Johan C.	2015
Biofunctional coatings for dental implants	Chen, Xi; Li, Yuping; Aparicio, Conrado	2013
Maxillary sinus grafting with a nano-structured biomaterial: preliminary clinical and histological results	Stübinger, S.; Ghanaati, S.; Orth, C.; Hilbig, U.; Saldamli, B.; Biesterfeld, S.; Kirkpatrick, C. J.; Sader, R. A.	2009
Fundamentals of implant dentistry	Byrne, Gerard	2014
Peri-implant bone remodeling around an extraction socket: predictions of bone maintenance by finite element method.	Chou, Hsuan-Yu; Romanos, Georgios; Müftü, Ali; Müftü, Sinan	2012
Augmentation of localized defects of the anterior maxillary ridge with autogenous bone before insertion of implants	Raghoebar, Gerry M.; Batenburg, Rutger HK; Vissink, Arjan; Reintsema, Harry	1996
A 36-month randomized controlled split-mouth trial comparing immediately loaded titanium oxide-anodized and machined implants supporting fixed partial dentures in the posterior mandible.	Fung, Karen; Marzola, Riccardo; Scotti, Roberto; Tadinada, Aditya; Schincaglia, Gian Pietro	2011
Effects of magnesium-substituted nanohydroxyapatite coating on implant osseointegration	Zhao, Shi-fang; Jiang, Qiao-hong; Peel, Sean; Wang, Xiao-xiang; He, Fu-ming	2013
Does immediate loading affect clinical and patient-centered outcomes of mandibular 2-unsplinted-implant overdenture? A 2-year within-case analysis	Emami, Elham; Cerutti-Kopplin, Daiane; Menassa, Mélanie; Audy, Nicholas; Kodama, Naoki; Durand, Robert; Rompré, Pierre; de Grandmont, Pierre	2016
Immediate loading of four or six implants in completely edentulous patients	Antoun, Hadi; Belmon, Patrick; Cherfane, Pierre; Sitbon, Jean Max	2012
Collageneous matrix coatings on titanium implants modified with decorin and chondroitin sulfate: characterization and influence on osteoblastic cells	Bierbaum, Susanne; Douglas, Timothy; Hanke, Thomas; Scharnweber, Dieter; Tippelt, Sonja; Monsees, Thomas K.; Funk, Richard HW; Worch, Hartmut	2006
A Novel Osseous Densification Approach in Implant Osteotomy Preparation to Increase Biomechanical Primary Stability, Bone Mineral Density, and Bone-to-Implant Contact.	Huwais, Salah; Meyer, Eric G.	2017
Biomechanical investigation of thread designs and interface conditions of zirconia and titanium dental implants with bone: three-dimensional numeric analysis.	Fuh, Lih-Jyh; Hsu, Jui-Ting; Huang, Heng-Li; Chen, Michael YC; Shen, Yen-Wen	2013
Osseointegration surgery: Host determinants and outcome criteria	Sennerby, Lars; Rasmusson, Lars	2002
Effects of occlusal inclination and loading on mandibular bone remodeling: a finite element study.	Rungsiyakull, Chaiy; Rungsiyakull, Pimduen; Li, Qing; Li, Wei; Swain, Michael	2011
Influence of static overload on the bony interface around implants in dogs.	Miyamoto, Yasunari; Koretake, Katsunori; Hirata, Makoto; Kubo, Takayasu; Akagawa, Yasumasa	2008
Surface characterization analysis of failed dental implants using scanning electron microscopy	Daood, Umer; Bandey, Ninette; Qasim, Saad Bin; Omar, Hanan; Khan, Saad A.	2011
Influence of bone and dental implant parameters on stress distribution in the mandible: a finite element study	Guan, Hong; Van Staden, Rudi; Loo, Yew-Chaye; Johnson, Newell; Ivanovski, Saso; Meredith, Neil	2009
Finite element stress analysis of dental prostheses supported by straight and angled implants.	Cruz, Mauro; Wassall, Thomaz; Toledo, Elson Magalhaes; da Silva Barra, Luis Paulo; Cruz, Silvia	2009

Bone strains around immediately loaded implants supporting mandibular overdentures in human cadavers.	Akca, Kivanc; Akkocaoğlu, Murat; Cömert, Ayhan; Tekdemir, Ibrahim; Cehreli, Murat Cavit	2007
Periodontal repair in dogs: a bioabsorbable calcium carbonate coral implant enhances space provision for alveolar bone regeneration in conjunction with guided tissue regeneration	Wikesjö, Ulf ME; Lim, Won Hee; Razi, Saghi S.; Sigurdsson, Thorarinn J.; Lee, Michael B.; Tatakis, Dimitris N.; Hardwick, W. Ross	2003
Marginal bone loss around tilted implants in comparison to straight implants: a meta-analysis.	Monje, Alberto; Chan, Hsun-Liang; Suarez, Fernando; Galindo-Moreno, Pablo; Wang, Hom-Lay	2012
Biomaterial osseointegration enhancement with biophysical stimulation	Dimitriou, R.; Babis, G. C.	2007
Implant survival rate and marginal bone loss of 6-mm short implants: a 2-year clinical report.	Bratu, Emanuel; Chan, Hsun-Liang; Mihali, Sorin; Karancsi, Olimpiu; Bratu, Dana Cristina; Fu, Jia-Hui; Wang, Hom-Lay	2014
Surgical-prosthodontic reconstruction of advanced maxillary bone compromise with autogenous onlay block bone grafts and osseointegrated endosseous implants: a 12-year study of 32 consecutive patients.	Keller, Eugene E.; Tolman, Dan E.; Eckert, Steven	1999
Implant materials, design, and surface topographies: their influence on osseointegration of dental implants	Triplett, R. Gilbert; Frohberg, Uwe; Sykaras, Nikitas; Woody, Ronald D.	2003
Effects of implant thread geometry on percentage of osseointegration and resistance to reverse torque in the tibia of rabbits	Steigenga, Jennifer; Al-Shammari, Khalaf; Misch, Carl; Nociti, Francisco H.; Wang, Hom-Lay	2004
Bone morphogenetic protein 2 incorporated into biomimetic coatings retains its biological activity	Liu, Yuelian; Hunziker, Ernst B.; Layrolle, Pierre; De Bruijn, Joost D.; De Groot, Klaas	2004
Ridge preservation after tooth extraction	Wang, Ren E.; Lang, Niklaus P.	2012
Preliminary 3-dimensional surface texture measurement and early loading results with a microtextured implant surface.	Mazor, Ziv; Cohen, Donald K.	2003
In vitro osteoclast resorption of bone substitute biomaterials used for implant site augmentation: a pilot study.	Taylor, James C.; Cuff, Sheldon E.; Leger, James PL; Morra, Amani; Anderson, Gail I.	2002
Tooth-implant connection: a bibliographic review	Hita-Carrillo, Celso; Hernández-Aliaga, Manuel; Calvo-Guirado, Jose-Luis	2010
Influence of Laser-Lok surface on immediate functional loading of implants in single-tooth replacement: a 2-year prospective clinical study	Farronato, Davide; Mangano, Francesco; Briguglio, Francesco; Iorio-Siciliano, Vincenzo; Riccitiello, Francesco; Guarnieri, Renzo	2014
Osteointegration of titanium and hydroxyapatite rough surfaces in healthy and compromised cortical and trabecular bone: in vivo comparative study on young, aged, and estrogen-deficient sheep	Borsari, Veronica; Fini, Milena; Giavaresi, Gianluca; Rimondini, Lia; Consolo, Ugo; Chiusoli, Loris; Salito, Armando; Volpert, Andreas; Chiesa, Roberto; Giardino, Roberto	2007
The combined use of rhBMP-2/ACS, autogenous bone graft, a bovine bone mineral biomaterial, platelet-rich plasma, and guided bone regeneration at nonsubmerged implant placement for supracrestal bone augmentation. A case report.	Sclar, Anthony G.; Best, Steven P.	2013
Bone-to-implant apposition with machined and MTX microtextured implant surfaces in human sinus grafts.	Trisi, Paolo; Marcato, Carlo; Todisco, Marzio	2003
Adherence of Streptococcus mutans to implant materials	Fujioka-Hirai, Yumi; Akagawa, Yasumasa; Minagi, Shogo; Tsuru, Hiromichi; Miyake, Yoichiro; Suginaka, Hidekazu	1987
The relationship of Periotest values, biomaterial, and torque to failure in adult baboons.	Carr, Alan B.; Papazoglou, Efstratios; Larsen, Peter E.	1995
Coating with artificial matrices from collagen and sulfated hyaluronan influences the osseointegration of dental implants	Schulz, Matthias C.; Korn, Paula; Stadlinger, Bernd; Range, Ursula; Möller, Stephanie; Becher, Jana; Schnabelrauch, Matthias; Mai, Ronald; Scharnweber, Dieter; Eckelt, Uwe	2014
Significance of nano-and microtopography for cell-surface interactions in orthopaedic implants	Jäger, M.; Zilkens, C.; Zanger, K.; Krauspe, R.	2007
Differences in osseointegration rate due to implant surface geometry can be explained by local tissue strains	Simmons, Craig A.; Meguid, Shaker A.; Pilliar, Robert M.	2001
Dental implant biomaterials	Lemons, Jack E.	1990
Dental zirconia implants up to three years in function: a retrospective clinical study and evaluation of prosthetic restorations and failures.	Gahlert, Michael; Burtscher, Doris; Pfundstein, George; Grunert, Ingrid; Kniha, Heinz; Roehling, Stefan	2013
Effect of tilted and short distal implants on axial forces and bending moments in implants supporting fixed dental prostheses: an in vitro study.	Ogawa, Toru; Dhaliwal, Sandra; Naert, Ignace; Mine, Atsushi; Kronstrom, Mats; Sasaki, Keiichi; Duyck, Joke	2010
Characterization of bone around titanium implants and bioactive glass particles: an experimental study in rats.	Gorustovich, Alejandro; Rosenbusch, Mariana; Guglielmotti, Maria B.	2002
Peri-implant pathology—relation to implant failure and tumor formation	Athanasou, N. A.	2007
Functional assessment of dental implant osseointegration.	Chang, Po-Chun; Giannobile, William V.	2012
Platform switching: biomechanical evaluation using three-dimensional finite element analysis.	Tabata, Lucas Fernando; Rocha, Eduardo Passos; Barao, Valentim Adelino Ricardo; Assunçao, Wirley Gonçalves	2011
Color atlas of oral diseases	Laskaris, George	2003
Ultraviolet photofunctionalization of titanium implants.	Ogawa, Takahiro	2014
Alveolar bone regeneration for immediate implant placement using an injectable bone substitute: an experimental study in dogs	Boix, Damien; Gauthier, Olivier; Guicheux, Jérôme; Pilet, Paul; Weiss, Pierre; Grimandi, Gaël; Daculsi, Guy	2004
Comparison of implant body designs and threaded designs of dental implants: a 3-dimensional finite element analysis.	Huang, Heng-Li; Chang, Chin-Han; Hsu, Jui-Ting; Faligatter, Alison M.; Ko, Ching-Chang	2007
Interface shear strength of titanium implants with a sandblasted and acid-etched surface: A biomechanical study in the maxilla of miniature pigs	Buser, Daniel; Nydegger, Thomas; Oxland, Thomas; Cochran, David L.; Schenk, Robert K.; Hirt, Hans Peter; Snétivy, Daniel; Nolte, Lutz-Peter	1999

Influence of extracellular matrix coatings on implant stability and osseointegration: an animal study	Stadlinger, Bernd; Pilling, Eckart; Huhle, Matthias; Mai, Ronald; Bierbaum, Susanne; Bernhardt, Ricardo; Scharnweber, Dieter; Kuhlisch, Eberhard; Hempel, Ute; Eckelt, Uwe	2007
Trial with Platelet-Rich Fibrin and Bio-Oss used as grafting materials in the treatment of the severe maxillar bone atrophy: clinical and radiological evaluations	Inchingolo, F.; Tatullo, M.; Marrelli, M.; Inchingolo, A. M.; Scacco, S.; Inchingolo, A. D.; Dipalma, G.; Vermesan, D.; Abbinante, A.; Cagiano, R.	2010
Periodontal and peri-implant bone regeneration: clinical and histologic observations.	Artzi, Zvi; Zohar, Ron; Tal, Haim	1997
In vitro bone strain analysis of implant following occlusal overload	Kan, Janice PM; Judge, Roy B.; Palamara, Joseph EA	2014
Effects of Repeated Screw Tightening on Implant Abutment Interfaces in Terms of Bacterial and Yeast Leakage in Vitro: One-Time Abutment Versus the Multiscrewing Technique.	Calcaterra, Roberta; Di Girolamo, Michele; Mirisola, Concetta; Baggi, Luigi	2016
In vitro attachment of human gingival fibroblasts to endosseous implant materials	Guy, S. C.; McQuade, M. J.; Scheidt, M. J.; McPherson, JC d; Rossmann, J. A.; Van Dyke, T. E.	1993
Direct implant loading in the edentulous maxilla using a bone density–adapted surgical protocol and primary implant stability criteria for inclusion	Östman, Pär-Olov; Hellman, Mats; Sennerby, Lars	2005
Surface modifications of dental implants	Stanford, C. M.	2008
Effects of implant surface coatings and composition on bone integration: a systematic review	Junker, Rüdiger; Dimakis, Athanasios; Thoneick, Maurice; Jansen, John A.	2009
Early and immediately restored and loaded dental implants for single-tooth and partial-arch applications.	Ganeles, Jeffrey; Wismeijer, Daniel	2004
Titanium endosseous implant-soft tissue interface: a literature review	Donley, Timothy G.; Gillette, William B.	1991
Implant positioning errors in freehand and computer-aided placement methods: a single-blind clinical comparative study.	Arısan, Volkan; Karabuda, Cüneyt Z.; Mumcu, Emre; Özdemir, Tayfun	2013
Effect of recombinant human bone morphogenetic protein-2 in an absorbable collagen sponge with space-providing biomaterials on the augmentation of chronic alveolar ridge defects	Barboza, Eliane Porto; Caúla, André Luis; de Oliveira Caúla, Fernanda; de Souza, Rogério Oliveira; Neto, Luiz Geolás; Sorensen, Rachel G.; Li, X. Jian; Wikesjö, Ulf ME	2004
Five-year prospective study of immediate/early loading of fixed prostheses in completely edentulous jaws with a bone quality-based implant system	Misch, Carl E.; Degidi, Marco	2003
Immediate loading of Trabecular Metal-enhanced titanium dental implants: interim results from an international proof-of-principle study	Schlee, Marcus; van der Schoor, W. Peter; van der Schoor, Alexandra RM	2015
Alveolar distraction osteogenesis for the correction of vertically deficient edentulous ridges: a multicenter prospective study on humans.	Chiapasco, Matteo; Consolo, Ugo; Bianchi, Alberto; Ronchi, Paolo	2004
Stimulation of directed bone growth at oxidized titanium implants by macroscopic grooves: an in vivo study	Hall, Jan; Miranda-Burgos, Patricia; Sennerby, Lars	2005
Two-year prospective follow-up of implant/tooth-supported versus freestanding implant-supported fixed partial dentures.	Akça, Kıvanç; Çehreli, Murat C.	2008
Effects of bone morphogenetic protein-2 and hyaluronic acid on the osseointegration of hydroxyapatite-coated implants: An experimental study in sheep	Aebli, Nikolaus; Stich, Hermann; Schawalder, Peter; Theis, Jean-Claude; Krebs, Jörg	2005
Biomechanical optimization of implant diameter and length for immediate loading: a nonlinear finite element analysis.	Kong, Liang; Gu, Zexu; Li, Tao; Wu, Junjie; Hu, Kaijin; Liu, Yanpu; Zhou, Hongzhi; Liu, Baolin	2009
Histologic Evaluation of Sinus Grafting Materials After Peri-implantitis–Induced Failure: A Case Series.	Scarano, Antonio; Cholakis, Anastasia Kelekis; Piattelli, Adriano	2017
Biomechanics and load resistance of short dental implants: a review of the literature	Hasan, Istabrak; Bourauel, Christoph; Mundt, Torsten; Heinemann, Friedhelm	2013
Oral microbiomes: more and more importance in oral cavity and whole body	Gao, Lu; Xu, Tiansong; Huang, Gang; Jiang, Song; Gu, Yan; Chen, Feng	2018
Osseointegration of sintered porous-surfaced and plasma spray–coated implants: An animal model study of early postimplantation healing response and mechanical stability	Simmons, Craig A.; Valiquette, Nancy; Pilliar, Robert M.	1999
Modification of Ti6Al4V surfaces using collagen I, III, and fibronectin. I. Biochemical and morphological characteristics of the adsorbed matrix	Bierbaum, Susanne; Beutner, René; Hanke, Thomas; Scharnweber, Dieter; Hempel, Ute; Worch, Hartmut	2003
Computational analyses of small endosseous implants in osteoporotic bone	Wirth, A. J.; Müller, Ralph; van Lenthe, G. Harry	2010
Evaluation of 3D printed PCL/PLGA/β-TCP versus collagen membranes for guided bone regeneration in a beagle implant model	Won, J. Y.; Park, C. Y.; Bae, J. H.; Ahn, G.; Kim, C.; Lim, D. H.; Cho, D. W.; Yun, W. S.; Shim, J. H.; Huh, J. B.	2016
Posterior partially edentulous jaws, planning a rehabilitation with dental implants	Monteiro, Douglas R.; Silva, Emily VF; Pellizzer, Eduardo P.; Magro Filho, Osvaldo; Goiato, Marcelo C.	2015
Osteoinductive implants: the mise-en-scene for drug-bearing biomimetic coatings	Liu, Yuelian; De Groot, K.; Hunziker, Ernst Bruno	2004
Basic peptide protamine exerts antimicrobial activity against periodontopathic bacteria	Miura, Tadashi; Iohara, Keishi; Kato, Tetsuo; Ishihara, Kazuyuki; Yoshinari, Masao	2010
A computed tomographic scan–derived customized surgical template and fixed prosthesis for flapless surgery and immediate loading of implants in fully edentulous maxillae: A prospective multicenter study	van Steenberghe, Daniel; Glauser, Roland; Blombäck, Ulf; Andersson, Matts; Schutyser, Filip; Pettersson, Andreas; Wendelhag, Inger	2005
Dental implants in edentulous adults with cognitive disabilities: report of a pilot project	Durham, Timothy M.; King, Thomas; Salinas, Thomas; Franco, Theodore; Ross, Jason	2006
Microleakage at the different implant abutment interface: A systematic review	Mishra, Sunil Kumar; Chowdhary, Ramesh; Kumari, Shail	2017
Influence of preparation and wall thickness on the resistance to fracture of zirconia implant abutments	Att, Wael; Yajima, Nao-Daniel; Wolkewitz, Martin; Witkowski, Siegbert; Strub, Joerg Rudolf	2012

PhyloToAST: Bioinformatics tools for species-level analysis and visualization of complex microbial datasets	Dabdoub, Shareef M.; Fellows, Megan L.; Paropkari, Akshay D.; Mason, Matthew R.; Huja, Sarandeep S.; Tsigarida, Alexandra A.; Kumar, Purnima S.	2016
Early implant loading after vertical ridge augmentation (VRA) using e-PTFE titaniumreinforced membrane and nano-structured hydroxyapatite: 2-year prospective study.	Canullo, Luigi; Sisti, Angelo	2010
Bone healing and mineralization, implant corrosion, and trace metals after nickel–titanium shape memory metal intramedullary fixation	Ryhänen, J.; Kallioinen, M.; Serlo, W.; Perämäki, P.; Junila, J.; Sandvik, P.; Niemelä, E.; Tuukkanen, J.	1999
Current research in the pathogenesis of aseptic implant loosening associated with particulate wear debris	Jiang, Yunpeng; Jia, Tanghong; Wooley, Paul H.; Yang, Shang-You	2013
Implant loading protocols for the partially edentulous posterior mandible.	Cordaro, Luca; Torsello, Ferruccio; Roccuzzo, Mario	2009
Biofilm Inactivation and prevention on common implant material surfaces by nonthermal DBD plasma treatment	Ibis, Fatma; Oflaz, Hakan; Ercan, Utku Kürsat	2016
Nonlinear finite element analysis versus ex vivo strain gauge measurements on immediately loaded implants.	Eser, Atilim; Akca, Kivanc; Eckert, Steven; Cehreli, Murat Cavit	2009
In vivo effects of RGD-coated titanium implants inserted in two bone-gap models	Elmengaard, Brian; Bechtold, Joan E.; Søballe, Kjeld	2005
Removal torque values of titanium implants in the maxilla of miniature pigs.	Buser, Daniel; Nydegger, Thomas; Hirt, Hans Peter; Cochran, David L.; Nolte, Lutz-Peter	1998
Dental implants placed by undergraduate students: clinical outcomes and patients'/students' perceptions	Vandeweghe, Stefan; Koole, Sebastiaan; Younes, Faris; De Coster, Peter; De Bruyn, Hugo	2014
Acid attack and cathepsin K in bone resorption around total hip replacement prosthesis	Konttinen, Yrjö T.; Takagi, Michiaki; Mandelin, Jami; Lassus, Jan; Salo, Jari; Ainola, Mari; Li, Tian-Fang; Virtanen, Ismo; Liljeström, Mikko; Sakai, Hideaki	2001
Influence of a Laser-Lok Surface on Immediate Functional Loading of Implants in Single-Tooth Replacement: Three-Year Results of a Prospective Randomized Clinical Study on Soft Tissue Response and Esthetics.	Guarnieri, Renzo; Grande, Maurizio; Ippoliti, Stefano; Iorio-Siciliano, Vincenzo; Riccitiello, Francesco; Farronato, Davide	2015
Increased bone formation around coated implants	Stadlinger, Bernd; Bierbaum, Susanne; Grimmer, Silke; Schulz, Matthias C.; Kuhlisch, Eberhard; Scharnweber, Dieter; Eckelt, Uwe; Mai, Ronald	2009
Osseoperception: active tactile sensibility of osseointegrated dental implants	Enkling, Norbert; Utz, Karl Heinz; Bayer, Stefan; Stern, Regina Mericske	2010
Long-term bone response to titanium implants coated with thin radiofrequent magnetron-sputtered hydroxyapatite in rabbits.	Mohammadi, Shams; Esposito, Marco; Hall, Jan; Emanuelsson, Lena; Krozer, Anatol; Thomsen, Peter	2004
Extramaxillary surgical technique: clinical outcome of 352 patients rehabilitated with 747 zygomatic implants with a follow-up between 6 months and 7 years	Maló, Paulo; de Araújo Nobre, Miguel; Lopes, Armando; Ferro, Ana; Moss, Steven	2015
"All-on-4" immediate-function concept for completely edentulous maxillae: a clinical report on the medium (3 years) and long-term (5 years) outcomes	Maló, Paulo; de Araújo Nobre, Miguel; Lopes, Armando; Francischone, Carlos; Rigolizzo, Mauricio	2012
Injectable magnesium-enriched hydroxyapatite putty in peri-implant defects: a histomorphometric analysis in pigs.	Crespi, Roberto; Capparè, Paolo; Addis, Alessandro; Gherlone, Enrico	2012
A histomorphometric study of bone reactions to titanium implants in irradiated bone and the effect of hyperbaric oxygen treatment	Johnsson, Ase A.; Sawaii, Toshihiro; Jacobsson, Magnus; Granström, Gösta; Turesson, Ingela	1999
Alveolar Ridge and Maxillary Sinus Augmentation Using rh BMP-2: A Systematic Review	de Freitas, Rubens Moreno; Spin-Neto, Rubens; Junior, Elcio Marcantonio; Pereira, Luís Antônio Violin Dias; Wikesjö, Ulf ME; Susin, Cristiano	2015
The influence of clip material and cross sections of the bar framework associated with vertical misfit on stress distribution in implant-retained overdentures.	dos Santos, Mateus Bertolini Fernandes; Bacchi, Atais; Correr-Sobrinho, Lourenço; Consani, Rafael Leonardo Xediek	2014
Immediately loaded blade implant retrieved from a man after a 20-year loading period: a histologic and histomorphometric case report	Di Stefano, Danilo; Iezzi, Giovanna; Scarano, Antonio; Perrotti, Vittoria; Piattelli, Adriano	2006
In vitro study of the influence of the type of connection on the fracture load of zirconia abutments with internal and external implant-abutment connections	Sailer, Irena; Sailer, Thomas; Stawarczyk, Bogna; Jung, Ronald Ernst; Hämmerle, C. H.	2009
Histologic aspects of the bone and soft tissues surrounding three titanium non-submerged plasma-sprayed implants retrieved at autopsy: a case report	Piattelli, A.; Scarano, A.; Piattelli, M.; Bertolai, R.; Panzoni, E.	1997
Influence of nicotine administration on different implant surfaces: a histometric study in rabbits	Stefani, Cristine M.; Nogueira Filho, Getúlio R.; Sallum, Enilson A.; De Toledo, Sérgio; Sallum, Antonio W.; Nociti, Francisco H.	2002
General and oral aspects of osteoporosis: a review	von Wowern, Nina	2001
Implants inserted into homografts bearing fixed restorations.	Viscioni, Alessandro; Franco, Maurizio; Rigo, Leone; Guidi, Riccardo; Brunelli, Giorgio; Carinci, Francesco	2009
A retrospective case series of implants used to restore partially edentulous patients with implant-supported removable partial dentures: 31-month mean follow-up results.	Grossmann, Yoav; Levin, Liran; Sadan, Avishai	2008
Are ceramic implants a viable alternative to titanium implants? A systematic literature review	Andreiotelli, Marina; Wenz, Hans J.; Kohal, Ralf-Joachim	2009
Immediate rehabilitation of completely edentulous arches with a four-implant prosthesis concept in difficult conditions: an open cohort study with a mean follow-up of 2 years.	Malo, Paulo; Nobre, Miguel de Araujo; Lopes, Armando	2012
Clinical and radiographic evaluation, following delivery of fixed reconstructions, at GBR treated titanium fixtures	Mayfield, Lisa; Skoglund, Annika; Nobréus, Nils; Attström, Rolf	1998
Dental implant thread pitch and its influence on the osseointegration process: an in vivo comparison study.	Orsini, Ester; Giavaresi, Gianluca; Trirè, Alessandra; Ottani, Vittoria; Salgarello, Stefano	2012

An XPS and SEM evaluation of six chemical and physical techniques for cleaning of contaminated titanium implants	Mouhyi, Jaafar; Sennerby, Lars; Pireaux, Jean-jacques; Dourov, Nicolas; Nammour, Samir; Van Reck, Jack	1998
Functionalization of dental implant surfaces using adhesion molecules	Schliephake, H.; Scharnweber, D.; Dard, M.; Sewing, A.; Aref, A.; Roessler, S.	2005
Immediate loading with complete implant-supported restorations in an edentulous heavy smoker: histologic and histomorphometric analyses.	Romanos, George E.; Johansson, Carina B.	2005
Hydrothermal treatment for TiN as abrasion resistant dental implant coating and its fibroblast response	Shi, Xingling; Xu, Lingli; Munar, Melvin L.; Ishikawa, Kunio	2015
Use of endosseous one-piece yttrium-stabilized zirconia dental implants in premolar region: a two-year clinical preliminary report	Borgonovo, A.; Censi, R.; Dolci, M.; Vavassori, V.; Bianchi, A.; Maiorana, C.	2011
The bases for using a particular occlusal design in tooth and implant-borne reconstructions and complete dentures	Klineberg, Iven; Kingston, Dianna; Murray, Greg	2007
Mechanical and technical risks in implant therapy	Salvi, Giovanni E.; Bragger, Urs	2009
Hyaluronan supports recombinant human bone morphogenetic protein-2 induced bone reconstruction of advanced alveolar ridge defects in dogs. A pilot study	Hunt, Dennis R.; Jovanovic, Sascha A.; Wikesjö, Ulf ME; Wozney, John M.; Bernard, George W.	2001
Outcomes and their measurement in clinical trials of endosseous oral implants	van Steenberghe, Daniel	1997
The team approach to managing dental implant complications: Strategies for treating periimplantitis	Rosen, Paul S.	2013
Clinical Evaluation of Immediate Loading of Electroeroded Screw-Retained Titanium Fixed Prostheses Supported by Tilted Implant: A Multicenter Retrospective Study	Acocella, Alessandro; Ercoli, Carlo; Geminiani, Alessandro; Feng, Changyong; Billi, Mauro; Acocella, Gabriele; Giannini, Domenico; Sacco, Roberto	2012
Diamondlike carbon coating as a galvanic corrosion barrier between dental implant abutments and nickel-chromium superstructures.	Ozkomur, Ahmet; Erbil, Mehmet; Akova, Tolga	2013
Laser-Deposited Hydroxyapatite Films on Dental Implants–Biological Evaluation in vivo	Dostálová, T.; Jelínek, M.; Himmlová, L.; Grivas, Ch	1998
The effects of metal implants on inflammatory and healing processes	Tsaryk, Roman; Peters, Kirsten; Unger, Ronald E.; Scharnweber, Dieter; Kirkpatrick, C. James	2007
Short-term retrospective case series of implant-assisted removable partial dentures with locator abutments.	Ortiz-Puigpelat, Octavi; Gargallo-Albiol, Jordi; Hernández-Alfaro, Federico; Cabratosa-Termes, Josep	2014
Osteopontin at mineralized tissue interfaces in bone, teeth, and osseointegrated implants: ultrastructural distribution and implications for mineralized tissue formation, turnover, and repair	McKee, M. D.; Nanci, A.	1996
Morphometric Changes Induced by Cold Argon Plasma Treatment on Osteoblasts Grown on Different Dental Implant Surfaces.	Canullo, Luigi; Genova, Tullio; Mandracci, Pietro; Mussano, Federico; Abundo, Roberto; Fiorellini, Joseph P.	2017
Repair of symptomatic cartilage lesions of the knee: the place of autologous chondrocyte implantation.	Vanlauwe, Johan; Almqvist, Frederik; Bellemans, Johan; Huskin, Jean-Pierre; Verdonk, Rene; Victor, Jan	2007
Influence of Alveolar Bone Loss and Different Alloys on the Biomechanical Behavior of Internal-and External-Connection Implants: A Three-Dimensional Finite Element Analysis.	Tsouknidas, Alexander; Lympoudi, Evdokia; Michalakis, Konstantinos; Giannopoulos, Dimitrios; Michailidis, Nikolaos; Pissiotis, Argirios; Fytanidis, Dimitrios; Kugiumtzis, Dimitrios	2015
Fixation of 5-unit implant-supported fixed partial dentures and resulting bone loading: a finite element assessment based on in vivo strain measurements.	Karl, Matthias; Winter, Werner; Taylor, Thomas D.; Heckmann, Siegfried M.	2006
Unconventional implants for distal cantilever fixed full-arch prostheses: a long-term evaluation of four cases.	Minoretti, Roger; Triaca, Albino; Saulacic, Nikola	2012
Membranes and Bone Substitutes in a One-Stage Procedure for Horizontal Bone Augmentation: A Histologic Double-Blind Parallel Randomized Controlled Trial.	Merli, Mauro; Moscatelli, Marco; Mariotti, Giorgia; Pagliaro, Umberto; Breschi, Lorenzo; Mazzoni, Annalisa; Nieri, Michele	2015
In vivo analysis of tissue response to plasma-treated collagen-I-coated titanium alloys	Hauser, J.; Ring, A.; Schaffran, A.; Henrich, L.; Esenwein, S. A.; Steinau, H. U.; Stricker, I.; Langer, S.	2009
Principles of attachment selection	Shafie, Hamid	2007
Osseointegration of zirconia implants with different surface characteristics: an evaluation in rabbits.	Hoffmann, Oliver; Angelov, Nikola; Zafiropoulos, Gregory-George; Andreana, Sebastiano	2012
The Survival of Morse Cone-Connection Implants with Platform Switch.	Cassetta, Michele; Di Mambro, Alfonso; Giansanti, Matteo; Brandetti, Giulia	2016
Biomechanics and peri-implantitis: The effect of a subcrestal wing-thread to decrease alveolar crestal bone strain. theory, finite element analysis, and clinical application.	Laster, Zvi; Weissberg, Ilan; Kablan, Fares	2012
Fence technique for localized three-dimensional bone augmentation: a technical description and case reports.	Merli, Mauro; Mariotti, Giorgia; Moscatelli, Marco; Motroni, Alessandro; Mazzoni, Annalisa; Mazzoni, Simona; Nieri, Michele	2015
Bone and bone substitutes	Nasr, Hisham F.; Aichelmann-Reidy, Mary Elizabeth; Yukna, Raymond A.	1999
Current Implant Designs to Maintain Crestal Bone and Gingiva	Horowitz, R.	2008
Application of confocal laser scanning microscopy in dentistry	Rashid, Haroon	2014
In Vivo Effect of Titanium Implants with Porous Zinc-Containing Coatings Prepared by Plasma Electrolytic Oxidation Method on Osseointegration in Rabbits.	He, Jing; Feng, Wei; Zhao, Bao-Hong; Zhang, Wei; Lin, Zeng	2018
Biomechanical evaluation of amorphous calcium phosphate coated TNTZ implants prepared using a radiofrequency magnetron sputtering system	Shiraishi, Naru; Tu, Rong; Uzuka, Risa; Anada, Takahisa; Narushima, Takayuki; Goto, Takashi; Niinomi, Mitsuo; Sasaki, Keiichi; Suzuki, Osamu	2012
Multicenter Clinical Randomized Controlled Trial Evaluation of an Implant System Designed for Enhanced Primary Stability.	Stanford, Clark M.; Barwacz, Chris; Raes, Stephanie; De Bruyn, Hugo; Cecchinato, Denis; Bittner, Nurit; Brandt, Jan	2016
Peri-implantitis Induced by Stainless Steel Ligature in Beagle Dogs	Xingnan Lin, D. D. S.	2017

Impact of Dynamic and Static Load on Bone Around Implants: An Experimental Study in a Rat Model.	Yagihara, Atsushi; Kawasaki, Ryo; Mita, Atsushi; Takakuda, Kazuo	2016
The rabbit as experimental model for research in implant dentistry and related tissue regeneration	Stübinger, Stefan; Dard, Michel	2013
Placement of plate-form implants using osteotomes	Roberts, Ralph	2002
Finite element analysis relative to the crestal position of a 3.0-mm-diameter implant.	León, Javier; Carrascosa, Alejandro; Rodríguez, Xavier; Ruiz-Magaz, Vanessa; Pascual, Andrés; Nart, José	2014
Influence of superstructure geometry on the mechanical behavior of zirconia implant abutments: A finite element analysis	Geringer, Alexander; Diebels, Stefan; Nothdurft, Frank P.	2014
Effect of microthread design of dental implants on stress and strain patterns: a three-dimensional finite element analysis	Amid, Reza; Raoofi, Saeed; Kadkhodazadeh, Mahdi; Movahhedi, Mohammad Reza; Khademi, Maryam	2013
Primary and Secondary Stability of Implants in Postextraction and Healed Sites: A Randomized Controlled Clinical Trial.	Malchiodi, Luciano; Balzani, Lucio; Cucchi, Alessandro; Ghensi, Paolo; Nocini, Pier Francesco	2016
Ultrasonic vs. drill osteotomy. A clinical and histologic study in the sheep mandible	Paolo Trisi, D.; Marco Colagiovanni, D.	2011
The application of HA-coated dental implants in patients with severe bone defects	De Lange, G. L.; Kuiper, L.; Blijdorp, P. A.	1994
Capability of differently charged plasma polymer coatings for control of tissue interactions with titanium surfaces	Schröder, K.; Finke, B.; Ohl, A.; Lüthen, F.; Bergemann, C.; Nebe, B.; Rychly, J.; Walschus, U.; Schlosser, M.; Liefeith, K.	2010
Comorbidity of periodontal disease: two sides of the same coin? An introduction for the clinician	Holmstrup, Palle; Damgaard, Christian; Olsen, Ingar; Klinge, Björn; Flyvbjerg, Allan; Nielsen, Claus Henrik; Hansen, Peter Riis	2017
Performance of zirconia for dental healthcare	Silva, Nelson RFA; Sailer, Irena; Zhang, Yu; Coelho, Paulo G.; Guess, Petra C.; Zembic, Anja; Kohal, Ralf J.	2010
Influence of anisotropic bone properties on the biomechanical behavior of the acetabular cup implant: a multiscale finite element study	Nguyen, Vu-Hieu; Rosi, Giuseppe; Naili, Salah; Michel, Adrien; Raffa, Maria-Letizia; Bosc, Romain; Meningaud, Jean-Paul; Chappard, Christine; Takano, Naoki; Haiat, Guillaume	2017
No influence of simultaneous bone-substitute application on the success of immediately loaded dental implants: a retrospective cohort study	Kopp, Sigmar; Behrend, Detlef; Kundt, Guenther; Ottl, Peter; Frerich, Bernhard; Warkentin, Mareike	2013
Peri-implantitis: A re-view of the disease and report of a case treated with allograft to achieve bone regeneration	Rashid, H.; Sheikh, Z.; Vohra, F.; Hanif, A.; Glogauer, M.	2015
Microbial adhesion on novel yttria-stabilized tetragonal zirconia (Y-TZP) implant surfaces with nitrogen-doped hydrogenated amorphous carbon (aC: H: N) coatings	Schienle, Stefanie; Al-Ahmad, Ali; Kohal, Ralf Joachim; Bernsmann, Falk; Adolfsson, Erik; Montanaro, Laura; Palmero, Paola; Fürderer, Tobias; Chevalier, Jérôme; Hellwig, Elmar	2016
Ligature-Induced Peri-implant Bone Loss Around Loaded Zirconia and Titanium Implants.	Roehling, Stefan; Gahlert, Michael; Janner, Simone; Meng, Bo; Woelfler, Henriette; Cochran, David L.	2019
Horizontal bone augmentation in full-arch maxillary implant-supported restorations: a preliminary clinical report	Taschieri, Silvio; Corbella, Stefano; Francetti, Luca; Del Fabbro, Massimo	2014
Retrospective cohort clinical investigation of a dental implant with a narrow diameter and short length for the partial rehabilitation of extremely atrophic jaws	Maló, Paulo S.; de Araújo Nobre, Miguel A.; Lopes, Armando V.; Ferro, Ana S.	2017
Qualitative assessment of natural apatite in vitro and in vivo	Guizzardi, Stefano; Montanari, Cristiana; Migliaccio, Stefano; Strocchi, Rita; Solmi, Rossella; Martini, Desiree; Ruggeri, Alessandro	2000
Corrosion and biocompatibility of orthopedic implants	Hallab, Nadim James; Urban, Robert M.; Jacobs, Joshua J.	2004
Long-term marginal periimplant bone loss in edentulous patients.	Carlsson, Gunnar E.; Lindquist, Lars W.; Jemt, Torsten	2000
Low BMD affects initial stability and delays stem osseointegration in cementless total hip arthroplasty in women: a 2-year RSA study of 39 patients	Aro, Hannu T.; Alm, Jessica J.; Moritz, Niko; Mäkinen, Tatu J.; Lankinen, Petteri	2012
Combined collagen membrane and hydroxyapatite/collagen chondroitin-sulfate spacer placement in the treatment of 2-wall intrabony defects in chronic adult and rapidly progressive periodontitis patients	Benque, Edmond; Zahedi, Shahram; Brocard, Daniel; Oscaby, Françoise; Justumus, Pierre; Brunel, Gérard	1997
Overview of clinical trials on endosseous implants	Fritz, Michael E.	1997
Zirconia in dentistry: part 2. Evidence-based clinical breakthrough	Vagkopoulou, Thaleia	2009
Plasma of Argon Cleaning Treatment on Implant Abutments in Periodontally Healthy Patients: Six Years Postloading Results of a Randomized Controlled Trial.	Canullo, Luigi; Tallarico, Marco; Penarrocha, Miguel; Corrente, Giuseppe; Fiorellini, Joseph; Penarrocha, David	2017
The dynamics in implantation for patients with clefts	Nagasao, Tomohisa; Miyamoto, Junpei; Jin, Hongmei; Tamaki, Tamotsu; Isshiki, Yasushige; Kaneko, Tsuyoshi; Nakajima, Tatsuo	2006
Advanced reconstructive technologies for periodontal tissue repair	Ramseier, Christoph A.; Rasperini, Giulio; Batia, Salvatore; Giannobile, William V.	2012
Stem cells in dentistry–Part II: Clinical applications	Egusa, Hiroshi; Sonoyama, Wataru; Nishimura, Masahiro; Atsuta, Ikiru; Akiyama, Kentaro	2012
The 300 Most-Cited Articles in Implant Dentistry.	Antonio Alarcon, Marco; Esparza, Diana; Montoya, Carmen; Monje, Alberto; Faggion Jr, Clovis Mariano	2017
The effect of hyaluronan on osteoblast proliferation and differentiation in rat calvarial-derived cell cultures	Huang, L.; Cheng, Y. Y.; Koo, P. L.; Lee, K. M.; Qin, L.; Cheng, J. C. Y.; Kumta, S. M.	2003
Methodology and criteria in evaluation of dental endosseous implants	Langeland, Kaare; Sp\a angberg, Larz	1975
Zygomatic implant placement in conjunction with sinus bone grafting: the “extended sinus elevation technique.” a case-cohort study	Hinze, Marc; Vrielinck, Luc; Thalmair, Tobias; Wachtel, Hannes; Bolz, Wolfgang	2013
Section plane selection influences the results of histomorphometric studies: the example of dental implants	Kopp, Sigmar; Warkentin, Mareike; Öri, Ferenc; Ottl, Peter; Kundt, Günther; Frerich, Bernhard	2012

A Study of Osseointegration—3-D Culture of KUSA/A1 Cells with a Collagen Scaffold on Titanium Implants with Different Surface Modifications—	MIKAMI, Toshiko; KUMABE, Shunji; IWAI, Yasutomo	2010
Research of the relief and element composition of the surface coatings based on hydroxyapatite implants from titanium alloys	Pogrebjak, A. D.; Krivets, A. S.; Dyadyura, K. A.; Maksakova, O. V.; Shaimardanov, Zh K.; Peplinska, B.	2016
Tibial component fixation with a peri-apatite coating: evaluation by radiostereometric analysis in a canine total knee arthroplasty model	Allen, Matthew J.; Leone, Kendall A.; Dunbar, Michael J.; Race, Amos; Rosenbaum, Paula F.; Sacks, Jonathan M.	2012
Evaluation of Different Combinations of Biphasic Calcium Phosphate and Growth Factors for Bone Formation in Calvarial Defects in a Rabbit Model.	Chung, Sung-Min; Jung, In Kwon; Yoon, Byung-Ho; Choi, Bok Ryul; Kim, David M.; Jang, Jung Sun	2016
Periimplant Bone Healing under Experimental Hepatic Osteodystrophy Induced by a Choline-Deficient Diet: A Histomorphometric Study in Rats	Gorustovich, Alejandro; de los Angeles Esposito, Maria; Guglielmotti, María Beatriz; Juan Giglio, Maximo	2003
Deproteinized bovine bone xenograft	Stavropoulos, Andreas	2008
Understanding biomaterial-tissue interface quality: combined in vitro evaluation	Gasik, Michael	2017
Biomechanics of jaw bone considering structural properties of trabecular bone	Matsunaga, Satoru; Takano, Naoki; Tamatsu, Yuichi; Abe, Shinichi; Ide, Yoshinobu	2011
Bone Response to Four Dental Implants with Different Surface Topographies: A Histologic and Histometric Study in Minipigs.	Kalemaj, Zamira; Scarano, Antonio; Valbonetti, Luca; Rapone, Biagio; Grassi, Felice Roberto	2016
Sinus augmentation using rhBMP-2-loaded synthetic bone substitute with simultaneous implant placement in rabbits	Joo, Myung-Jae; Cha, Jae-Kook; Lim, Hyun-Chang; Choi, Seong-Ho; Jung, Ui-Won	2017
Implantable zirconia bioceramics for bone repair and replacement: A chronological review	Afzal, Adeel	2014
Paranasal bone: the prime factor affecting the decision to use transsinus vs zygomatic implants for biomechanical support for immediate function in maxillary dental implant reconstruction.	Jensen, Ole T.; Adams, Mark W.; Smith, Edmund	2012
The influence of extraction on the stability of implanted titanium microscrews: a biomechanical and histomorphometric study.	Zheng, Leilei; Tang, Tian; Deng, Feng; Zhao, Zhihe	2009
Combined immediate loading of zygomatic and mandibular implants: a preliminary 2-year report of 19 patients.	Butura, Caesar C.; Galindo, Daniel F.	2014
Comparing the influence of crestal cortical bone and sinus floor cortical bone in posterior maxilla bi-cortical dental implantation: a three-dimensional finite element analysis	Yan, Xu; Zhang, Xinwen; Chi, Weichao; Ai, Hongjun; Wu, Lin	2015
Effectiveness of resonance frequency in predicting orthopedic implant strength and stability in an in vitro osseointegration model	Isaacson, Brad M.; Vance, Richard E.; Chou, Teri G. Rosenbaum; Bloebaum, Roy D.; Bachus, Kent N.; Webster, Joseph B.	2009
Correlation between the bone density recorded by a computerized implant motor and by a histomorphometric analysis: a preliminary in vitro study on bovine ribs	Iezzi, Giovanna; Scarano, Antonio; Di Stefano, Danilo; Arosio, Paolo; Doi, Kazuya; Ricci, Laura; Piattelli, Adriano; Perrotti, Vittoria	2015
Overdentures on Implants for Better Quality of Life Among the Fully Edentulous Patients–Case Reports	Nikolovska, Julijana; Petrovski, Dragan; Petricevic, Nikola; Kapusevska, Biljana; Korunoska-Stevkovska, Vesna	2015
Vojin Savić, Stevo Najman, Dragan Mihailović, Perica Vasiljević, Zoran Stojanović, et al.	Ignjatović, Nenad; Ajduković, Zorica	2013
Best practice: surgeon driven application in pelvic operations	Krueger, Thilo B.; Wegner, Celine; Somerlik-Fuchs, Karin H.; Hoffmann, Klaus-Peter; Mattmueller, Rudi	2016
Cell culture methods for testing biocompatibility	Pizzoferrato, Arturo; Ciapetti, Gabriela; Stea, Susanna; Cenni, Elisabetta; Arciola, Carla Renata; Granchi, Donatella	1994
Does periodontal tissue regeneration really work?	Bosshardt, Dieter D.; Sculean, Anton	2009
Integrin 2 1 plays a critical role in osteoblast response to micron-scale surface structure and surface energy of titanium substrates	Ti, Titanium	2008
Systemic and local effects of radiotherapy: an experimental study on implants placed in rats	da Cruz Vegian, Mariana Raquel; Costa, Bruno César Almeida; de Fátima Santana-Melo, Gabriela; Godoi, Fernanda Herrera Costa; Kaminagakura, Estela; Tango, Rubens Nisie; do Prado, Renata Falchete; de Oliveira, Luciane Dias; Federico, Claudio Antonio; Avelino, Sarah de Oliveira Marco	2019
Bone physiology of tooth movement, ankylosis, and osseointegration	Roberts, W. Eugene	2000
Treatment of Circumferential Defects with Osseoconductive Xenografts of Different Porosities: A Histological, Histometric, Resonance Frequency Analysis, and Micro-CT Study in Dogs	Antunes, Antonio Azoubel; Grossi-Oliveira, Gustavo Augusto; Martins-Neto, Evandro Carneiro; De Almeida, Adriana Luisa Gonçalves; Salata, Luiz Antonio	2015
Chitosan as a barrier membrane material in periodontal tissue regeneration	Xu, Chun; Lei, Chang; Meng, Liuyan; Wang, Changning; Song, Yaling	2012
Dental Medicine Nanosystems: Nanoparticles and their use in Dentistry and Oral Health Care	Lboutounne, Hassan	2017
Thermally Sprayed Porous PEEK Coating for Biomedical Implants	Tailor, Satish; Vashishtha, Nitesh; Modi, Ankur; Modi, S. C.	2019
Cytocompatible and Anti-bacterial Adhesion Nanotextured Titanium Oxide Layer on Titanium Surfaces for Dental and Orthopaedic Implants	Ferraris, Sara; Cochis, Andrea; Cazzola, Martina; Tortello, Mauro; Scalia, Alessandro; Spriano, Silvia; Rimondini, Lia	2019
The 5 Hallmarks of Biomaterials Success: An Emphasis on Orthopaedics	Williams, Dustin L.; Isaacson, Brad M.	2014
Biomaterial-Associated Infection: Locating the Finish Line in the Race for	Busscher, Henk J.	
Interventions to Maintain Locator-Retained Mandibular Overdentures on Both External Hex and Internal Connection Implants: A Retrospective Study.	Fernandez-Estevan, Lucia; Montero, Javier; Selva Otaolaurruchi, Eduardo J.; Ruiz, Sola; Fernanda, Maria	2018
Plasma rich in growth factors for the treatment of ocular surface diseases	Anitua, Eduardo; Muruzabal, Francisco; de la Fuente, María; Merayo, Jesús; Durán, Juan; Orive, Gorka	2016

Predictive values of resonance frequency analysis as a diagnostic tool in palatal implant loss	Wieczorek, Katja; Harzer, Winfried; Wehrbein, Heinrich; Moergel, Maximilian; Kunkel, Martin; Jung, Britta A.	2019
The effect of titanium dental implant surfaces with varying microtopography on the temporal expression of the osteogenic phenotype in vitro	Knabe, C.; Naumann, I.; Houshmand, A.; Lopez-Heredia, M. A.; Stiller, M.; Niem, T.; Wostmann, B.; Hiibner, A.; Miiller-Mai, C.	2015
Dental implant surface chemistry and energy alter macrophage activation in vitro	Boyan, Barbara D.; Olivares-Navarrete, Rene	2016
The Rationale and Technique in Relation to Immediate Implant Stability	Wu, Chun-Ying; Yang, Chin-Hua; Huang, Peng-Hao; Chen, Kuo-Ching	2014
Myomucosal cheek flaps: applications in intraoral reconstruction using three different techniques	Ferrari, S.	2011
Comparison of titanium and FGM dental implants with different coating types	Aldousari, Saad M.; Fouda, Noha; Hedia, Hassan S.; AlThobiani, Faisal WH	2018
Implant design and intraosseous stability of immediately placed implants: a human cadaver study	Akca, Kivanc; Cehreli, Murat Cavit	2004
4 Nanotechnology in dental implants	Jurczyk, Karolina; Braegger, Urs; Jurczyk, Mieczyslaw	2018
Biomaterial-Associated Infection: Locating the Finish Line in the Race for the Surface	TISSUE, BACTERIA VERSUS HOST	
UNCORRECTED PROOFS	Toffler, Michael; Rosen, Paul S.	2016
The oral biome in the aetiology and management of dental disease: Current concepts and ethical considerations	Ma, Yonghui; Oliver, Richard; Chen, Hua	2019
Periprocedural myocardial infarction enhances the predictive value of inflammatory biomarkers for patients with obstructive coronary artery disease after implantation of drug-eluting stent	Jesika, Anastasia; Zuo-Ying, Hu; Kan, Jing; Shao-Liang, Chen	2015
Prevalence of short implants use in the jaws: a retrospective pilot study.	GUMES, Irving; GUEDES, Gabriel; SÓRIO, Ana Luisa; CYSNEIROS, Henrique; FALCÃO, Antônio; MIRANDA, Dario Augusto Oliveira	2015
Early osseointegration of implants with cortex-like TiO 2 coatings formed by micro-arc oxidation: A histomorphometric study in rabbits	Zhou, Hong-zhi; Liu, Lin; Chen, Xiao-dong; Wang, Wei-qiang; Ma, Guo-wu; Su, Yu-cheng; Qi, Min; Shi, Bin	2017
Tissue Engineering and Dental Implantology: Biomaterials, New Technologies, and Stem Cells	Galindo-Moreno, Pablo	
Osseointegration of Orthopaedic Implants	Goharian, Amirhossein	2019
Stress Distribution in an Implant-Supported Mandibular Complete Denture Using Different Cantilever Lengths and Occlusal Coating Materials	De Medeiros, Rodrigo Antonio; Goiato, Marcelo Coelho; Pesqueira, Aldiéris Alves; Vechiato, Filho; José, Aljomar; Bonatto, Liliane da Rocha; dos Santos, Daniela Micheline	2017
Effect of alendronate on bone remodeling around implant in the	Park, R.; Kim, J. H.; Choi, H.; Park, Y. B.; Jung, H. S.; Moon, H. S.	2003
4 Sequelae Caused by Wearing Complete Dentures	THEIR, DENTURES IN	2013
A Prospective Clinical Study on Titanium Implants in the Zygomatic Arch for Prosthetic Rehabilitation of the Atrophic Edentulous Maxilla with a Follow-Up of 6 Months to 5 Years	Fortes, Vanessa	
A study of the bone healing kinetics of plateau versus screw root design titanium dental implants	Coelho, Paulo; Polyzois, Ioannis; Stassen, Leo F. A.; Claffey, Noel	2008
The effects of implant surface roughness and surgical technique on implant fixation in an in vitro model	Jansen, John A.	2006
Extraction Site Management for Ridge Preservation and Implant Site Development	Bartee, Barry; Cullum, Daniel R.	2015
2.1. 1 Clinical demands and purpose of surface modification	Hanawa, T.	2018
Micromovement measurements of endosseous dental implants with 3D Digital Image Correlation (DIC) method	Rodrigues, A. T.; Neto, B. A.; Nicolau, C. P.	2011
implants use in the jaws: a retrospective pilot study	GUMES, Irving; GUEDES, Gabriel; SÓRIO, Ana Luisa; CYSNEIROS, Henrique; FALCÃO, Antônio; MIRANDA, Dario Augusto Oliveira	2015
Computational structural analysis of dental implants using radial point interpolation meshless methods	Coelho, C. C. C.; Belinha, J.; Jorge, RM Natal	2019
Functionalization of Titanium surface with Chitosan via silanation: 3D CLSM imaging of cell biocompatibility behaviour	Attik, G. N.; D'Almeida, M.; Toury, B.; Grosgogeat, B.	2013
Immunomodulation for Biomaterial Applications	Knopf-Marques, Helena; Paiva, Lilian; Prévot, Flavie; Barthès, Julien	
Alveolar ridge alterations in the maxillary anterior region after tooth extraction through orthodontic forced eruption for implant site development: a clinical CBCT study.	Papadopoulou, Alexandra K.; Papageorgiou, Spyridon N.; Hatzopoulos, Stavros A.; Tsirlis, Anastasios; Athanasiou, Athanasios E.	2019
Beta/tricalcium phosphate for Ridge Augmentation with Simultaneous Implant Placement in the Esthetic Zone-Clinical Case and Surgical Considerations	Božidar Brkovic, D. D. S.	
Swedish Dental Journal Supplement 188, 2007	Roos-Jans\a aker, Ann-Marie	2007
Long term results of therapy of sleep apnea syndrome by electromyostimulation (EMS)	Ludwig, A.; Hegewald, R.	2011
A retrospective study on interobserver variation of Pap smear reporting at Kenyatta National Hospital (KNH) cytology laboratory, Nairobi, Kenya	Mwaniki, Jackrogers Njuki; Waweru, Wairimu; Ndungu, Joseph Rugumi; Muchiri, Lucy Wangari	2014

Plant-derived rhamnogalacturonan-i's modulate proinflammatory cytokine gene expression in neutrophils stimulated by E. coli LPS and P. gingivalis bacteria	Folkert, J.; Mieszkowska, A.; Burke, B.; Addison, O.; Gurzawska, K.	2018
Interdisciplinary Planning	Phillips, Keith M.	2017
Marginal bone and soft tissue behavior following platform switching abutment connection/disconnection–a dog model study	Blanco, Juan	2014
Treatment Complications in the Esthetic Zone	El Askary, Abd El Salam	2008
Osteointegration of the bone tissue with titanium implants	Dedukh, Ninel; Malyshkina, Svitlana	2010
Clinical performance of one-piece zirconia dental implants: A systematic review	ArRejaie, Aws S.; Al-Hamdan, Rana S.; Basunbul, Ghadeer I.; Abduljabbar, Tariq; Al-Aali, Khulud A.; Labban, Nawaf	2019
Using MicroCT to Assess Periodontal Regeneration Outcomes–Comparison of Image-Based and Histologic Results: A Case Report.	Rebaudi, Alberto; Trisi, Paolo; Pagni, Giorgio; Wang, Hom-Lay	2017
Bone Particles and the Undersized Surgical Technique	Jansen, J. A.	2010
Zirconia compared to titanium dental implants in preclinical studies—A systematic review and meta-analysis	Roehling, Stefan; Schlegel, Karl A.; Woelfler, Henriette; Gahlert, Michael	2019
Oral and Maxillofacial Surgery Advances in Implant Dentistry	AUGMENTATION, BONE GRAFT	2000
Bone morphology changes around two types of bone-level implants installed in fresh extraction sockets–a histomorphometric study in Beagle dogs	Jansen, John A.	2014
Bone characteristics and implant stability	Sabeva, Elitsa; Peev, Stefan; Miteva, Mariya; Georgieva, Milena	2017
Stability of intraosseous dental implants with guided bone regeneration (in vivo experiment in dogs).	Indjova, Jermen; Fakih, Kh; Sivrev, D.; Yovchev, D.; Chaprazov, Ts	2014
Biofilm formation on polyetheretherketone and titanium surfaces	Barkarmo, Sargon; Longhorn, Daniel; Leer, Kiran; Johansson, Carina B.; Stenport, Victoria; Franco-Tabares, Sebastian; Kuehne, Sarah A.; Sammons, Rachel	2019
Effect of Biologic Aging of Implants on Osseointegration in the Dog.	Hwang, Byung-Moon; Park, Yong Bum; Lee, Jae Hoon	2019
'Go graftless'	KHAN, DR BURZIN	
Tooth Extraction and	Preservation, Site	2013
10 Biomaterials and Immune	Prakasam, Sivaraman; Gajendrareddy, Praveen; Louie, Christopher; Lee, Clarence; Bertassoni, Luiz E.	
Effects of occlusal trauma on the periodontium, alveolar bone, temporomandibular joint and central nervous system	Ispas, Ana; Crăciun, Antarinia; Kui, Andreea; Lascu, Liana; Constantiniuc, Mariana	2018
Tooth Extraction and Site Preservation	Caplanis, Nicholas; Lozada, Jaime L.; Mesquida, Juan	2013
CONTEMPORARY CONCEPTS AND TREATMENT	SEOTNTEGRATION, OS	
Biomechanical Behaviors of Implant-Supported Zirconia Restorations Cemented to Novel Screw-Retained Abutment Systems: A Three-Dimensional Finite Element Analysis Study	KALELİ, Necati; URAL, Çağrı	
Multimodal analysis of the tissue response to a bone-anchored hearing implant: a two-year case report of a patient with recurrent inflammation and pain	Calon, Tim GA; Johansson, Martin L.; Omar, Omar; Shah, Furqan; Budding, Dries; Trobos, Margarita; Thomson, Peter; Stokroos, Robert-Jan; Palmquist, Anders	2019
Three-dimensional finite-element analysis of osseointegrated dental implants	Baggi, Luigi; Cappelloni, Ilaria; Maceri, Franco; Vairo, Giuseppe	2007
Histologic and Clinical Evaluation of a Bioactive Calcium-Phosphosilicate Bone Graft	Gonshor, Aron; Lanka Mahesh, B. D. S.	
Occlusal Principles and Considerations for Implants: An Overview	Paliwal, Siddhartha; Saxena, Deepesh; Mittal, Rohit; Chaudhary, Shivangi	2014
Evaluating the role of extracellular matrix molecules and enzymes in periodontitis.	Rezvi, Fathima Bareera; Vishnu Priya, V.; Gayathri, R.	2018
Minimally Invasive Complete Arch Treatment: The Versatility of Angled Implants	Jensen, Ole T.; Cullum, Daniel R.	2015
MATRITM BONE	Code, Re-order	2017
Division of Advanced Ceramics, Nagoya Institute of Technology, Nagoya, Japan	Kasuga, Toshihiro	2019
IMPLANT SURVIVAL: BIOLOGICAL AND MECHANICAL INFLUENCES ON IMPLANTS'LIFE SPAN	Rini, Maria Sofia; Borea, Giorgio; Nuzzolese, Emilio; Meleo, Deborah; Betti, Dario	
Tissue engineering applications and nanobiomaterials in periodontology and implant dentistry	Keceli, Huseyin Gencay; Akman, Abdullah Cevdet; Bayram, Cem; Nohutcu, Rahime Meral	2016
E unctional stability of the dentition is a prerequi-site to normal mastication, and periodic mea	Efstratios Papazoglou, D. D. S.	
TiO 2-Modified Zirconia Surface Improves Epithelial Cell Attachment.	Riivari, Sini; Shahramian, Khalil; Kangasniemi, Ilkka; Willberg, Jaana; Närhi, Timo O.	2019
COMPARISON OF BIOMECHANICAL EFFECTS OF DIFFERENT DEGREES OF PLATFORM SWITCHED IMPLANTS ON THE BONE BY VARYING SUPERSTRUCTURE PROSTHETIC MATERIALS AND LOADING CONDITIONS: A THREE DIMENSIONAL FINITE ELEMENT STUDY"	Pushkar, Desai; Kumar, Vyas Anup	
PROSTHODONTICS	Lachmann, S.; Kimmerle-Müller, E.; Axmann, D.; Gomez-Roman, G.	2008

On the Significance and Predicted Functional Effects of the Crown-to-Implant Ratio: a Finite Element Study of Long-Term Implant Stability Using High-Resolution, Nonlinear Numerical Analysis	Sego, T. J.; Hsu, Yung-Ting; Chu, Tien-Min Gabriel; Tovar, Andres	2016
ENDO TRIBUNE	Musikant, Barry Lee	2010
Part 3: Implant Surgery	Rasmusson, Lars; Sennerby, Lars; Dahlin, Christer; Rosenquist, Bo; Sándor, George KB; Lam, David K.; Ylikontiola, Leena P.; Kainulainen, Vesa T.; Oikarinen, Kyösti S.; Clokie, Cameron ML	2010
The effect of zirconia and titanium implant abutments on light reflection of the supporting soft tissues	de Roode, Rowland; de Wit, Gerard C.; Cune, Marco S.	2010
Clinical and histologic evaluation of fresh frozen human bone grafts for horizontal reconstruction of maxillary alveolar ridges	Roberto Di Lenarda, D. D. S.	2011
Knowledge Gaps in Oral and Maxillofacial Surgery: a Systematic Mapping	Österberg, Marie; Holmlund, Anders; Sunzel, Bo; Tranaeus, Sofia; Twetman, Svante; Lund, Bodil	2017
Biomaterials for Dental Applications	Tuzuner, Tamer	
In Vitro Study on Biological Potential of Tissue-Engineered Cage by Using Surface-Modified Technique: A Preliminary Evaluation	Guo, Hong-Gang; Guo, Hong-Sheng; Yao, Fang-Lian; Yang, Shao-Guang; Chen, Zhi; Wang, Tao	2016
Poly (propylene fumarate) and poly (DL-lactic-co-glycolic acid) as scaffold materials for solid and foam-coated composite tissue-engineered constructs for cranial reconstruction	Dean, David; Topham, Neal S.; Meneghetti, S. Cristina; Wolfe, Michael S.; Jepsen, Karl; He, Shulin; Chen, Jeffrey E.-K.; Fisher, John P.; Cooke, Malcolm; Rimnac, Clare	2003
Synthesis, characterization and in vitro activity of a surface-attached antimicrobial cationic peptide	Chen, Renxun; Cole, Nerida; Willcox, Mark DP; Park, Josephine; Rasul, Riaz; Carter, Elizabeth; Kumar, Naresh	2009
Effect of abutment screw surface treatment on reliability of implant-supported crowns.	Anchieta, Rodolfo Bruniera; Machado, Lucas Silveira; Bonfante, Estevam Augusto; Hirata, Ronaldo; Freitas Jr, Amilcar Chagas; Coelho, Paulo G.	2014
Finite Element Analysis of and Multiscale Skeletal Tissue Mechanics Concerning a Single Dental Implant Site	Sego, Timothy James	2016
Surgery Phase Planning	EMPOWERMENT, TOOLS OF	2008
Application of Finite Element Analysis for Nanobiomedical Study	Wiwanitkit, Viroj	2012
The bone growing chamber: a new model to investigate spontaneous and guided bone regeneration of artificial defects in the human jawbone.	Trisi, Paolo; Rao, Walter	1998
Flow perfusion culture of marrow stromal osteoblasts in titanium fiber mesh	van den Dolder, Juliette; Bancroft, Gregory N.; Sikavitsas, Vassilios I.; Spauwen, Paul HM; Jansen, John A.; Mikos, Antonios G.	2003
Epidemiology and pathophysiology of coronary artery disease	Yahagi, Kazuyuki; Kolodgie, Frank D.; Virmani, Renu	2017
Peri-interventional endothelin-A receptor blockade improves long-term outcome in patients with ST-elevation acute myocardial infarction	Adlbrecht, Christopher; Wurm, Raphael; Humenberger, Michael; Andreas, Martin; Redwan, Bassam; Distelmaier, Klaus; Klappacher, Günter; Lang, Irene M.	2014
Safety of transcranial magnetic stimulation in patients with implanted deep brain stimulators	Kumar, Rajeev; Chen, Robert; Ashby, Peter	1999
Tissue Engineering in Ophthalmology: Implications for Eyelid Reconstruction	Sun, Michelle T.; O'connor, Andrea J.; Wood, John; Casson, Robert; Selva, Dinesh	2017
Oestrogen replacement therapy and cardiovascular disease in post-menopausal women. A review	Sitruk-Ware, R.; de Palacios, P. Ibarra	1989
Three-Dimensional Radiographic Imaging for Implant Positioning	Nattestad, Anders	2016
The Affect of the Oral Microbiome in Health and Disease on the Uptake of HPV in Oral Keratinocyte Cells	Carmosino, Andrew J.	2015
Inhibition of Orthopaedic Implant Infections by Immunomodulatory Effects of Host Defense Peptides	Greenfield, Edward	2014
Physico/chemical characterization, in vitro, and in vivo evaluation of hydroxyapatite/PLGA composite and tricalcium phosphate particulate grafting materials	Coimbra, Maria E.; Salles, Marcos B.; Yoshimoto, Marcelo; Allegrini Jr, Sergio; Fancio, Elizabeth; Higa, Olga; Suzuki, Marcelo; Coelho, Paulo G.	2009
Drug treatments for prosthetic joint infections in the era of multidrug resistance	Perez-Jorge, Concepcion; Gomez-Barrena, Enrique; Horcajada, Juan-Pablo; Puig-Verdie, Lluis; Esteban, Jaime	2016
Biomolecular Coating For Implants	García, Andrés J.; Reyes, Catherine D.; Petrie, Timothy; Schwartz, Zvi; Boyan, Barbara D.; Raynor, Jenny E.; Collard, David M.; Wojtowicz, Abigail M.; Guldberg, Robert E.	2012
CAMLOG Compendium: Surgery	Kirsch, Axel	2007
Complications in lateral window sinus elevation surgery	Wallace, Stephen S.; Testori, Tiziano	2010
Esthetic Implant Dentistry: Diagnosis and Treatment Planning	JIVRAJ, SAJ; RESHAD, MAMALY	2017
Surface-active biomaterials	Hench, L. Larry; Wilson, June	1984
32 Implantable Hearing Devices	Kuhn, Jeffery J.; Perez, Angel J.	
Biomaterials for facial bone augmentation: comparative studies	Wilson, June; Merwin, G. E.	1988
Biomechanical testing of microblasted, acid-etched/microblasted, anodized, and discrete crystalline deposition surfaces: an experimental study in beagle dogs.	Bonfante, Estevam A.; Granato, Rodrigo; Marin, Charles; Jimbo, Ryo; Giro, Gabriela; Suzuki, Marcelo; Coelho, Paulo G.	2013
Influence of maxillary sinus width on new bone formation after transcrestal sinus floor elevation: A proof-of-concept prospective cohort study	Lombardi, Teresa; Stacchi, Claudio; Berton, Federico; Traini, Tonino; Torelli, Lucio; Di Lenarda, Roberto	2017

Implant Strategies in Orthopedics	Koksal, Ismet	2015
Antibiotic Release from Calcium Phosphate Materials in Oral and Maxillofacial Surgery. Molecular, Cellular and Pharmaceutical Aspects	Manchon, Angel; C Prados-Frutos, Juan; Rueda-Rodriguez, Carmen; Salinas-Goodier, Carmen; H Alkhraisat, Mohammad; Rojo, Rosa; Rodriguez-Gonzalez, Arantza; Berlanga, Ana; Lopez-Cabarcos, Enrique	2017
REVIEW Open Access	Zhang, Lonnie Schneider Jianhua; Ning, Zhangchi; Lu, Cheng; Zhao, Siyu; Wang, Jianfen; Liu, Baoqin; Xu, Xuegong; Liu, Yuanyan	2018
Biotransformation of silver released from nanoparticle coated titanium implants revealed in regenerating bone	Geng, Hua; Poologasundarampillai, Gowsihan; Todd, Naomi; Devlin-Mullin, Aine; Moore, Katie L.; Golrokhi, Zahra; Gilchrist, James B.; Jones, Eric; Potter, Richard J.; Sutcliffe, Chris	2017
Micro-to Macroroughness of Additively Manufactured Titanium Implants in Terms of Coagulation and Contact Activation.	Ek, Rebecca Klingvall; Hong, Jaan; Thor, Andreas; Bäckström, Mikael; Rännar, Lars-Erik	2017
Combined vitrectomy and intravitreal dexamethasone (Ozurdex) sustained-release implant	Zheng, Andrew; Chin, Eric K.; Almeida, David RP; Tsang, Stephen H.; Mahajan, Vinit B.	2016
Induction of myofibroblastic differentiation in vitro by covalently immobilized transforming growth factor-β1	Metzger, Wolfgang; Grenner, Nadine; Motsch, Sandra E.; Strehlow, Rothin; Pohlemann, Tim; Oberringer, Martin	2007
Complications after surgery for metastatic humeral lesions	Janssen, Stein J.; van Dijke, Maarten; Lozano-Calderón, Santiago A.; Ready, John E.; Raskin, Kevin A.; Ferrone, Marco L.; Hornicek, Francis J.; Schwab, Joseph H.	2016
State of the union: a review of lumbar fusion indications and techniques for degenerative spine disease: JNSPG 75th Anniversary Invited Review Article	Reid, Patrick C.; Morr, Simon; Kaiser, Michael G.	2019
A review of mathematical modelling in total hip replacement	Srimongkol, Sineenart	2012
Fracture Resistance of Straight and Angulated Zirconia Implant Abutments Supporting Anterior Three-Unit Lithium Disilicate Fixed Dental Prostheses.	Saker, Samah; El-Shahat, Sameh; Ghazy, Mohamed	2016
The influence of implant body and thread design of mini dental implants on the loading of surrounding bone: a finite element analysis	Toth, Arpad; Hasan, Istabrak; Bourauel, Christoph; Mundt, Torsten; Biffar, Reiner; Heinemann, Friedhelm	2017
Influence of finish line in the distribution of stress trough an all ceramic implant-supported crown. A 3D finite element analysis	Sonnino, G.; Gloria, F.; Ottria, Liliana; Barlattani, Alberto	2009
Regenerative Dentistry Using Stem Cells and Nanotechnology	Mitsiadis, Thimios A.; Orsini, Giovanna	2017
Biologic effects of surface roughness and fluorhydroxyapatite coating on osteointegration in external fixation systems: an in vivo experimental study	Savarino, L.; Fini, M.; Ciapetti, G.; Cenni, E.; Granchi, D.; Baldini, N.; Greco, M.; Rizzi, G.; Giardino, R.; Giunti, A.	2003
SurFACTS in Biomaterials	McGonigle, Joe; Joslin, Jessica M.; Damodaran, Vinod B.; Reynolds, Melissa M.; Salvati, Lawrence; Fairman, Colin; Property, SurFACTS Intellectual; Aparicio, Conrado; Triolo, Phil; Fleming, Wendy	2012
Adaptive immune response inhibits ectopic mature bone formation induced by BMSCs/BCP/plasma composite in immune-competent mice	Bouvet-Gerbettaz, Sébastien; Boukhechba, Florian; Balaguer, Thierry; Schmid-Antomarchi, Heidy; Michiels, Jean-François; Scimeca, Jean-Claude; Rochet, Nathalie	2014
Dental Research	Fang, W. E. I.; David, T. W.; Xing, LONG; LASSILA, Lippo VJ; NAGAS, Emre; VALLITTU, Pekka K.; GAROUSHI, Sufyan; Ruo Xi, D. U.; Yi Ming, L. I.; Jian Feng, M. A.	2012
Removal Torque Values of Titanium Implants in the Maxilla of Miniature Pigs	Nolte, Lutz-Peter	2000
Historical Perspectives on Biomedical Coatings in Medical Devices	Hendriks, M.; Cahalan, P. T.	2016
New dimensions in tooth implant and transplantation	Anssari Moin, D.	2018
The potential of tissue engineering and regeneration for craniofacial bone	Yamano, Seiichi; Haku, K.; Ishioka, M.; Lin, T. Y.; Hanatani, S.; Dai, J.; Moursi, A. M.	2012
Temporomandibular joint total joint replacement—TMJ TJR	Mercuri, Louis G.	2016
Finite element modelling and simulations in dentistry: a bibliography 1990–2003	Mackerle, Jaroslav	2004
A Comparative Study on the Effect of Integrin Subunits Beta One and Beta Three on Osteoblast Implant Interactions	Duqum, Ibrahim	2017
Impact of metabolic bone disease on craniofacial bones and teeth	Bashutski, Jill; Taichman, L. Susan; McCauley, Laurie K.	2012
targeted preventive and not resistant-strain-producing	Beachey, E. H.; Bullen, J. J.; Rogers, H. J.; Griffiths, E.	1996
Adhesion to biomaterials	Mittelman, MARC W.	1996
Biomechanical techniques for pre-clinical testing of prostheses and implants	Prendergast, Patrick J.	2001
The story of Bioglass®	Hench, Larry L.	2006
Ceramic-polylactide composite material used in a model of healing of osseous defects in rabbits	Myciński, Pawe\l; Zarzecka, Joanna; Skórska-Stania, Agnieszka; Jelonek, Agnieszka; Okoń, Krzysztof; Wróbel, Maria	2017
Influence of periodontal biotype on buccal bone remodeling after tooth extraction using the flapless approach with a xenograft: A histomorphometric and fluorescence study in small dogs	Maia, Luciana Prado; Reino, Danilo Maeda; Novaes Jr, Arthur Belém; Muglia, Valdir Antonio; Taba Jr, Mário; de Morais Grisi, Márcio Fernando; de Souza, Sérgio Luís Scombatti; Palioto, Daniela Bazan	2015

Alendronate release from calcium phosphate cement for bone regeneration in osteoporotic conditions	van Houdt, Claire IA; Gabbai-Armelin, Paulo R.; Lopez-Perez, Paula M.; Ulrich, Dietmar JO; Jansen, John A.; Renno, Ana Claudia M.; van den Beucken, Jeroen JJP	2018
The numerical analysis of 4-On-Pillars technique using meshless methods	Vargas, K. F.; Caldas, G. A. R.; Belinha, J.; Jorge, RM Natal; Hernandez, P. A. G.; Ozkomur, A.; Smidt, R.; Naconecy, M. M.; Schneider, L. E.	2019
Impact of Surface Chemistry Modifications on Speed and Strength of Osseointegration.	Kang, Hyeon-Goo; Jeong, Yong-Soo; Huh, Yoon-Hyuk; Park, Chan-Jin; Cho, Lee-Ra	2018
Computed tomographic evaluation of alterations of the buccolingual width of the alveolar ridge after immediate implant placement associated with the use of a synthetic bone substitute.	Assaf, Jamal Hassan; Zanatta, Fabricio Batistin; de Brito Jr, Rui Barbosa; Gomes França, Fabiana Mantovani	2013
Biofilm associated microorganisms on removable oral orthodontic appliances in children in the mixed dentition	Pathak, A. K.; Sharma, D. S.	2013
ALVEOLAR RIDGE PRESERVATION AND REGENERATION WITH BIOMATERIALS	de Souza, Sérgio Luís Scombatti; Martins, Sérgio Henrique Lago; Bezerra, Fábio José Barbosa; Shibli, Jamil Awad; Mantovani, Rafael Verleigia; da Costa, Leandro Fabiano Alves; Júnior, José Augusto Barbanti Gabarra	2015
Polymeric drug delivery systems for localized cancer chemotherapy	De Souza, Raquel; Zahedi, Payam; Allen, Christine J.; Piquette-Miller, Micheline	2010
Medical biofilms—nanotechnology approaches	Neethirajan, Suresh; Clond, Morgan A.; Vogt, Adam	2014
Use of biologic agents to promote bone formation in implant dentistry: A critical assessment of systematic reviews	Alarcón, Marco Antonio; Diaz, Karla Tatiana; Aranda, Luisiana; Cafferata, Emilio Alfredo; Faggion Jr, Clovis Mariano; Monje, Alberto	2017
Craig's restorative dental materials-e-book	Sakaguchi, Ronald L.; Powers, John M.	2012
Surgical strategies for high-energy fractures in patients with osteoporosis.	Lepri, Andrea Cozzi; Capone, Antonio; Del Prete, Armando; Soderi, Stefano; Muncibì, Francesco; Innocenti, Massimo	2018
Quad Zygoma	Davó, Rubén; DOMFS, FRCDC	2019
Will There be a Role for Disc Prostheses in Small Animals?	Adamo, Filippo; Forterre, Franck	2015
Corrosion of Metallic Implants	Hallab, Nadim James; Jacobs, Joshua J.; Gilbert, Jeremy L.	2005
Intoxicated by Implants?	Helsen, Jozef A.; Missirlis, Yannis	2010
Biocompatibility, Metal Ions, and Corrosion Products	Tkaczyk, Cathy; Tabrizian, Maryam	2012
TOWARDS THE OPTIMUM SPINAL FUSION DEVICE	MEDVRETEN, V. ISKANJU OPTIMALNEGA UMETNEGA; VSADKA, NEGA	2018
2: Metal Surfaces in Medicine: Current Knowledge of Properties, Modeling and Biological Response	Artini, Cristina	2017
FORM, Frankfurt Oral Regenerative Medicine, Clinic for Maxillofacial and Plastic Surgery, Johann Wolfgang Goethe University, Frankfurt, Germany	Ghanaati, Shahram; Al-Maawi, Sarah	2018
Finite Element Analysis of Uncemented Total Hip Replacement: The effect of bone-implant interface	Ismail, Nur Faiqa; Shuib, Solehuddin; Yahaya, Muhd Azman; Romli, Ahmad Zafir; Shokri, Amran Ahmed	2016
A Critical Review of Contemporary Clinical Treatment	Nevins, Myron	2007
1Laboratory of Ecology and Ecotechnology, School of Biosciences, Mahatma Gandhi University, Kottayam, Kerala, India, 2International and Inter University Centre for Nanoscience and Nanotechnology, Mahatma Gandhi University, Kottayam, Kerala, India	Soman, Soumya; Ajitha, A. R.	2018
Nanobiotechnology perspectives on prevention and treatment of ortho-paedic implant associated infection	Borse, Vivek; Pawar, Vaishali; Shetty, Gautam; Mullaji, Arun; Srivastava, Rohit	2016
Biomimetic hydroxyapatite coated titanium screws demonstrate rapid implant stabilization and safe removal in-vivo	Sörensen, Jan Henrik; Dürselen, Lutz; Welch, Ken; Sörensen, Torben Christian; Procter, Philip; Engqvist, H\a akan; Strømme, Maria; Ignatius, Anita; Steckel, Hartwig	2015
The effect of seprafilm and interceed on capsule formation around silicone discs in a rat model	Friedman, H. I.; Stonerock, C.; Lefaivre, J. F.; Yost, M.	2004
Dental implant treatment planning: improvement in clinical and radiological diagnosis	Kubilius, Marius	
Methodological Consideration of Various Intraosseus and Heterotopic Bone Grafts Implantation in Animal Models	Xu, W.; Spilker, G.; Weinand, C.	2015
Histologic evaluation of new bone in post-extraction sockets induced by melatonin and apigenin: an experimental study in American fox hound dogs	Calvo Guirado, José Luis; López, López; Jara, Patricia; Fernández Domínguez, Manuel; Prados Frutos, Juan Carlos; Gehrke, Sergio Alexandre	2016
Current knowledge and future perspectives of bone replacement grafts	Kassir, Abdel Rahman; Chakar, Carole	2018
Design of artificial human joints & organs	Pal, Subrata	2014
MiniTHR for Young Adult Hip Disease 24	Santori, N.; Potestio, D.; Santori, F. S.	2013
Oral fungal-bacterial biofilm models in vitro	VITROME, IHU; Côte, d'Azur	2017
A review of the surface modifications of titanium alloys for biomedical applications	UPORABO, BIOMEDICINSKO	2017
Assessment of the symmetry of bone strains in the proximal femoral medial cortex under load in bilateral pairs of cadaver femurs	Sedlacek, R. C.; O'Connor, D. O.; Lozynsky, A. J.; Harris, W. H.	1997

In vitro phenotypic differentiation towards commensal and pathogenic oral biofilms	Janus, Marleen M.; Keijser, Bart JF; Bikker, Floris J.; Exterkate, Rob AM; Crielaard, Wim; Krom, Bastiaan P.	2015
Wear behavior of oxidized Ti-48Al-2Cr-2Nb (at.%), Ti-6Al-4V and Cp-Ti under dry and lubricated conditions	Ramos-Saenz, Carlos R.; Sundaram, Paul A.	
cruciate ligament reconstruction	Field, J. R.; Adachi, N.	
Fractionation and characterization of particles simulating wear of total joint replacement (TJR) following ASTM standards	Musib, Mrinal K.; Saha, Subrata	2011
Prosthetic Joint Infection due to Burkholderia cenocepacia: An Opportunistic Pathogen with an Expanding Spectrum of Disease.	Sebastian, Sujeesh; Malhotra, Rajesh; Das, Rojaleen; Kapil, Arti; Dhawan, Benu	2017
Bioactive peptide-modified biomaterials for bone regeneration	Lee, Jue-Yeon; Choi, Young-Suk; Lee, Seung-Jin; Chung, Chong-Pyoung; Park, Yoon-Jeong	2011
Bioceramics—An introductory overview	Shanmugam, K.; Sahadevan, R.	2018
Implantable Auditory Devices, an Issue of Otolaryngologic Clinics of North America E-Book	Kohan, Darius; Chandrasekhar, Sujana S.	2019
Effect of surface modification of zirconia on cell adhesion, metabolic activity and proliferation of human osteoblasts	Al Qahtani, Waleed MS; Schille, Christine; Spintzyk, Sebastian; Al Qahtani, Mohammed SA; Engel, Eva; Geis-Gerstorfer, Juergen; Rupp, Frank; Scheideler, Lutz	2017
Wolfgang Lehmann, Martin Rupprecht, Jacob Nuechtern, Daniel Melzner, Kai Sellenschloh, Jan Kolb, Florian Fensky	Hoffmann, Michael	2012
Adhesion and Proliferation of Human Adipose-Derived Stem Cells on Titania Nanotube Surfaces	Cowden, Kari; Dias-Netipanyj, Marcela Ferreira; Popat, Ketul C.	2019
Mechanisms how implant-associated blood clots could affect wound healing responses	Burkhardt, Melanie A.	2015
Incidence and risk factors for infection when teicoplanin is included for prophylaxis in patients with hip fracture	Capdevila, Aina; Navarro, Margarita; Bori, Guillem; Tornero, Eduard; Camacho, Pilar; Bosch, Jordi; García, Sebastián; Mensa, Josep; Soriano, Alex	2016
The influence of leukocyte and platelet concentrate enrich in stem cells on bone regeneration processes: a clinical and flow cytometry study	Bielecki, Tomasz; Wójcik, Krzysztof; Bold, Tadeusz; Osadnik, Bart\lomiej; Szczepański, Tomasz	2015
Bone Augmentation Techniques for Horizontal and Deficiency Vertical in Alveolar Oral Implantology Ridge	Tolstunov, Len; Hamrick, John F. Eric	2019
Uncemented hip arthroplasty in primary and revision surgery: patterns of bone remodelling and options to influence periprosthetic bone loss	Salemyr, Mats	2013
Annuloplasty for valve repair with a new biodegradable ring: an experimental study	Kalangos, Afksendiyos; Sierra, Jorge; Vala, Dominique; Cikirikcioglu, Mustafa; Walpoth, Beat; Orrit, Xavier; Pomar, José; Mestres, Carlos; Albanese, Sonia; Jhurry, Dhanjay	2006
Biocompatibility of anionic collagen matrices and its influence on the orientation of cellular growth.	Buchaim, Rogério L.; Goissis, Gilberto; Andreo, Jesus Carlos; Roque, Domingos D.; Roque, José S.; Buchaim, Daniela V.; Rodrigues, Antonio C.	2007
Treatment of a bone defect consequent to the removal of a periapical cyst with equine bone and equine membranes: clinical and histological outcome	Di Stefano, D. A.; Andreasi Bassi, M.; Cinci, L.; Pieri, L.; Ammirabile, G.	2012
Response to metallic implants	Wapner, Keith L.	1990
Advanced Coating Nanomaterials for Drug Release Applications	Scilletta, Natalia A.; Municoy, Sofía; Bellino, Martín G.; Soler-Illia, Galo JAA; Desimone, Martín F.; Catalano, Paolo N.	2019
Tightening of healing abutments: influence of torque on bacterial proliferation risk, an in vitro investigation	Bousquet, Philippe; Bennasar, Isabelle Calas; Tramini, Paul; Jacquemot, Maxime; Cuisinier, Frédéric	2014
Implant Associated Infection: Victorious Germs or Avoidable Complication?	Gehrke, Thorsten	
New telescopic crown protocol for partially edentulous patients: report of 32 cases.	Longoni, Salvatore; Apruzzese, Domenico; Careddu, Giovanni; Sartori, Matteo; Davide, Roberto	2005
Tanshinone IIA protects against polyethylene particle-induced osteolysis response in a mouse calvarial model	Yao, Jun; Ma, Shiting; Feng, Wenyu; Wei, Yan; Lu, Huiping; Zhong, Gang; Wu, Zhengyuan; Wang, Hongtao; Su, Wei; Li, Jia	2018
Cooperative catechol-functionalized polypept (o) ide brushes and ag nanoparticles for combination of protein resistance and antimicrobial activity on metal oxide surfaces	Yoo, Jin; Birke, Alexander; Kim, Joonwon; Jang, Yeongseon; Song, Seuk Young; Ryu, Seungmi; Kim, Byung-Soo; Kim, Byung-Gee; Barz, Matthias; Char, Kookheon	2018
Reduction of Inflammatory Reaction of Poly (D, L-Lactic-C o-Glycolic Acid) Using Demineralized Bone Particles	Yoon, Sun Jung; Kim, Soon Hee; Ha, Hyun Jung; Ko, Youn Kyung; So, Jung Won; Kim, Moon Suk; Yang, Young Il; Khang, Gilson; Rhee, John M.; Lee, Hai Bang	2008
In vivo and in vitro assessment of the biocompatibility and degradation of high-purity Mg anastomotic staples	Qu, Su; Xia, Jiazeng; Yan, Jun; Wu, Hongliu; Wang, Hao; Yi, Yi; Zhang, Xiaonong; Zhang, Shaoxiang; Zhao, ChangLi; Chen, Yigang	2017
Bioengineering, blood flow and stem cells	Kirkpatrick, C. J.	2004
The Wound-Healing Process	Price, Albert	2007
Cochlear and auditory brainstem implantation	Wackym, P. Ashley; Runge-Samuelson, Christina L.	2003
Digital image analysis for morphometric evaluation of tissue response after implanting alloplastic vascular prostheses	Zippel, Roland; Hoene, Andreas; Walschus, Uwe; Jarchow, Raymond; Ueberrueck, Torsten; Patrzyk, Maciej; Schlosser, Michael; Wilhelm, Lutz	2006
Kumari†, Ethirajulu Radha*, Narayanan Vanisri*, Soundarajan Aisverya*, Sukumarn Anil‡* DKM College for Women, Vellore, India,† Tagore Engineering College, Chennai, India	Sudha, Parappurath N.; Sangeetha, Kirubanandam; Jisha, Arumugam V.	2018
10 Anterior Lumbar Interbody Fusion	Sasso, Rick C.; Reichard, A. Kirk; Shah, Shenil	

Mechanisms of disease: the human N-glycome	Lauc, Gordan; Pezer, Marija; Rudan, Igor; Campbell, Harry	2016
Surface Functionalization of Biomaterials	Kyzio\l, Karol; Kaczmarek, \Lukasz; Kyzio\l, Agnieszka	2017
Porous metals and metallic foams in orthopedic applications	Lefebvre, L. P.	2013
New biomaterials for orthopedic implants	Ong, Kevin L.; Yun, Brian Min; White, Joshua B.	2015
Jeetesh Gawande1, Sanjay Upadhyay1	Upadhyay, Sanjay	2018
Aseptic Loosening of Total Hip Replacements-Acetabulum	Gollwitzer, Hans; von Eisenhart-Rothe, Rüdiger; Gradinger, Reiner	2014
Maxillofac. Surg. 2012; xxx: xxx–xxx.\copyright 2012 International Association of Oral and Maxillofacial Surgeons. Published by Elsevier Ltd. All rights reserved.	Ishak, M. I.; Kadir, MR Abdul; Sulaiman, E.; Kasim, NH Abu	
The promotion of antibacterial effects of ti6al4v alloy modified with TiO 2 nanotubes using a superoxidized solution	Beltrán-Partida, Ernesto; Valdez-Salas, Benjamin; Escamilla, Alan; Moreno-Ulloa, Aldo; Burtseva, Larysa; Valdez-Salas, Ernesto; Alvarez, Mario Curiel; Nedev, Nicola	2015
Biocompatibility of materials	Xiang, Zhou; Spector, Myron	2006
Osteotome-mediated sinus floor elevation: a systematic review and meta-analysis.	Călin, Claudiu; Petre, Alexandru; Drafta, Sergiu	2014
The Contribution of Periodontics to Orthodontic Therapy	Orthodontics, Periodontally Accelerated Osteogenic	
9.1 Antibacterial Coating of Implants in Orthopaedics and Trauma–313	Romanò, Carlo Luca; Morelli, Ilaria; Drago, Lorenzo; Gallazzi, Enrico; Scarponi, Sara	2017
Impact of Overlapping on 1-Year Clinical Outcomes in Patients Undergoing Everolimus-Eluting Bioresorbable Scaffolds Implantation in Routine Clinical Practice	i Sunyer, August Pi	
The influence of the alveolar ridge shape on the stress distribution in a free-end saddle removable partial denture supported by implant	Junior, Manoel M.; Anchieta, Rodolfo B.; Rocha, Eduardo P.; Pereira, Joao A.; Archangelo, Carlos M.; Freitas-Junior, Amilcar C.; Almeida, Erika O.	2011
GF109203X attenuates RANKL-induced osteoclastogenesis and suppresses osteolysis in a mouse model	Xie, Dawang; Yao, Jun; Feng, Wenyu; Wei, Yan; Lu, Huiping; Li, Jia; Wei, Qingjun	2017
Biomechanics of artificial disc	Goel, V.; Dooris, Andrew P.; Dennis, M.; Rengachary, S.	2003
CeraNews	Parvizi, Javad; Thomas, Peter; Summer, Burkhard; Krenn, Veit	
Lateral alveolar ridge augmentation using tenting screws, acellular dermal matrix, and freeze-dried bone allograft alone or with particulate autogenous bone.	Caldwell, Gregory R.; Mills, Michael P.; Finlayson, Richard; Mealey, Brian L.	2015
Response of cortical bone to local controlled release of sodium fluoride: the effect of implant insertion site	Anderson, Paul A.; Copenhaver, James C.; Tencer, Allan F.; Clark, John M.	1991
Induction of leukocyte activation by meshes surgically implanted in the peritoneal cavity	Devereux, Dennis F.; O'Connell, Sean M.; Liesch, Janice B.; Weinstein, Melvin; Robertson, Fredika M.	1991
Titania nanotube arrays as interfaces for blood-contacting implantable devices: a study evaluating the nanotopography-associated activation and expression of blood plasma components	Smith, Barbara S.; Popat, Ketul C.	2012
Biological Coatings for Implant Surface Modification	Xie, Youtao; Li, Kai; Zheng, Xuebin	
Progress in the Prevention and Treatment of Bacterial Biofilm [J]	DING, Jin-ya; HUANG, Qian-chuan; CAO, Jun-hao	2011
Efficacy and safety of anti-TNF therapy for inflammatory bowel disease (IBD) in liver transplant recipients for primary sclerosing cholangitis (PSC): a multicenter experience.	Ryska, O.; Serclova, Z.; Mestak, O.; Matouskova, E.; Vesely, P.; Mrazova, I.	
Titanium dioxide nanoparticles and nanotubular surfaces: potential applications in nanomedicine	Ribeiro, Ana Rosa; Gemini-Piperni, Sara; Alves, Sofia Afonso; Granjeiro, José Mauro; Rocha, Luís Augusto	2017
Management of Complications of Alveolar Distraction Osteogenesis Procedure	Drew, Stephanie J.	2016
Video-Assisted ALIF with Cage	Aunoble, S.; Hoste, D.; Bley, B.; Basso, Y.; Villet, L.; Liquois, F.	2006
Possible Role of Microcrystallinity on Surface Properties of Titanium Surfaces for Biomedical Application	Mussano, Federico; Genova, Tullio; Guastella, Salvatore; Faga, Maria Giulia; Carossa, Stefano	2016
Evaluation of cartilage repair tissue after biomaterial implantation in rat patella by using T2 mapping	Watrin-Pinzano, A.; Ruaud, J.-P.; Cheli, Y.; Gonord, P.; Grossin, L.; Bettembourg-Brault, I.; Gillet, P.; Payan, E.; Guillot, G.; Netter, P.	2004
Electrophoretic-deposited novel ternary silk fibroin/graphene oxide/hydroxyapatite nanocomposite coatings on titanium substrate for orthopedic applications	Li, Ming; Xiong, Pan; Mo, Maosong; Cheng, Yan; Zheng, Yufeng	2016
3.1 General introduction and chapter overview	Mas-Moruno, C.	2017
Effects of a calcium phosphate-coated and anodized titanium surface on early bone response	Koh, Jung-Woo; Kim, Young-Sung; Yang, Jae-Ho; Yeo, In-Sung	2013
Entamoeba gingivalis key aggressive predator in periodontal disease	Bonner, M.	2015
Elevated prostate specific antigen (PSA) levels a risk factor for pathological hip fracture in metastatic prostatic carcinoma	Thornes, B.; O'Malley, P.; O'Brien, M.; McCabe, J.; O'Sullivan, M.; Curtin, W.	2002
Cervical motion segment replacement	Bryan, Vincent E.	2004
Can Osseointegration Be Achieved Without Primary Stability?	Khabbaz, Yasser	2019

Surface modification of metallic biomaterials for better tribological properties: A review	Uwais, Zahid A.; Hussein, Mohamed A.; Samad, M. Abdul; Al-Aqeeli, Naser	2017
正畸微种植体周围炎对骨结合界面影响的研究	胡赟; 郑雷蕾; 唐甜; 赵志河; 宋锦璘; 邓锋	2011
Evaluation of the association of sinus mucosal thickening with dental and periodontal status using cone beam computed tomographic imaging	Shahidi, Shoaleh; Zamiri, Barbod; Panahi, Roghayeh	2016
Surface Characterization Techniques Used for Dentistry	Rashid, Haroon	2017
Prevention of tissue calcification on bioprosthetic heart valve by using epoxy compounds: a study of calcification tests in vitro and in vivo	Tingfei, Xi; Jiazhen, Ma; Wenhua, Tian; Xuehui, Lei; Shuhui, Long; Baoshu, Xi	1992
Engineering biocompatible implant surfaces. Part II: cellular recognition of biomaterial surfaces: lessons from cell–matrix interactions	Von Der Mark, Klaus; Park, Jung	2013
Tailored Biomaterials for Therapeutic Strategies Applied in Periodontal Tissue Engineering	Seciu, Ana-Maria; Craciunescu, Oana; Stanciuc, Ana-Maria; Zarnescu, Otilia	2019
Detectability of normal anatomy in digital panoramic radiographs	Gross, Heidi; Nilsson, Mats; Hellén-Halme, Kristina	2014
Effects of Ultraviolet Photofunctionalization on Bone Augmentation and Integration Capabilities of Titanium Mesh and Implants.	Hirota, Makoto; Ikeda, Takayuki; Tabuchi, Masako; Ozawa, Tomomichi; Tohnai, Iwai; Ogawa, Takahiro	2017
In vivo degradation of magnesium alloy LA63 scaffolds for temporary stabilization of biological myocardial grafts in a swine model	Schilling, Tobias; Brandes, Gudrun; Tudorache, Igor; Cebotari, Serghei; Hilfiker, Andres; Meyer, Tanja; Biskup, Christian; Bauer, Michael; Waldmann, Karl-Heinz; Bach, Friedrich-Wilhelm	2013
Alexandra Roman, Cosmin Cioban, Stefan-Ioan Stratul, Frank Schwarz, Aurel Muste, Stefan-Adrian Petrutiu	Zaganescu, Raluca	
106 Section lll: Disease-Specific Considerations	Shoulder, RCDA	2005
Dependence of neovascularization mechanisms on the molecular microenvironment	Popa, Eliane R.; Van Der Strate, Barry WA; Brouwer, Linda A.; Tadema, Henko; Schipper, Martin; Fernandes, Brian; Hendriks, Marc; Van Luyn, Marja JA; Harmsen, Martin C.	2007
18 The Titanium-Bone Interface In Vivo	Larsson, Cecilia; Esposito, Marco; Liao, Haihong; Thomsen, Peter	
Percutaneous coronary intervention of single-or multi-vessel disease	Buchanan, Gill Louise	2017
Maxillary Sinus in relation to Modern Oral and Maxillofacial Surgery	Corbella, Stefano	
Numerical investigation of complete mandibular dentures stabilized by conventional or mini implants in patient individual models	Lohmann, Anna; Keilig, Ludger; Heinemann, Friedhelm; Bourauel, Christoph; Hasan, Istabrak	2019
Effect of Local Puerarin Administration on Xenograft Lokal Puerarin Uygulamasının Hayvansal Kemik Grefti Üzerine Etkisi	Türer, Akif; Türer, Çiğdem Coşkun; Önger, Mehmet Emin	
Biological performance of a new β-TCP/PLLA composite material for applications in spine surgery: In vitro and in vivo studies	Aunoble, Stephane; Clément, Denis; Frayssinet, Patrick; Harmand, Marie Francois; Le Huec, Jean Charles	2006
Surface modification and drug delivery for biointegration	Jeong, Kyung Jae; Kohane, Daniel S.	2011
16S rDNA analysis of periodontal plaque in chronic obstructive pulmonary disease and periodontitis patients	Wu, Xingwen; Chen, Jiazhen; Xu, Meng; Zhu, Danting; Wang, Xuyang; Chen, Yulin; Wu, Jing; Cui, Chenghao; Zhang, Wenhong; Yu, Liying	2017
Healing of canine articular cartilage defects treated with microfracture, a type-II collagen matrix, or cultured autologous chondrocytes	Breinan, Howard A.; Martin, Scott D.; Hsu, Hu-Ping; Spector, Myron	2000
Zeeshan Sheikh1, 2, 3, Nader Hamdan4, Mohamed-Nur Abdallah1	Glogauer, Michael; Grynpas, Marc	2019
1D Titania Nanoarchitecture as Bioactive and Photoactive Coatings for Modern Implants: A Review	Radtke, Aleksandra	2017
Tissue reaction to prosthetic materials	Ravi, Nathan; Aliyar, Hyder Ali	2006
Factores pronósticos en implantología oral	Manzano Martínez, Guillermo	
Investigational drugs for fracture healing: preclinical & clinical data	E. Klontzas, Michail; I. Kenanidis, Eustathios; J. MacFarlane, Robert; Michail, Theodoros; E. Potoupnis, Michael; Heliotis, Manolis; Mantalaris, Athanasios; Tsiridis, Eleftherios	2016
Retrospective study of sandblasted, large-grit and acid-etched implant	Jo, Ji-Ho; Kim, Su-Gwan; Moon, Seong-Yong; Oh, Ji-Su; Park, Jin-Ju; Jung, Jong-Won; Yoon, Dae-Woong; Yang, Seong-Su; Jeong, Mi	2011
Исследование структуры взаимодействий в системе «имплантат-кость»	Зекий, А. О.; Широкий, А. А.	2016
* Mittelhessen University of Applied Sciences, Giessen, Germany	Moseke, C.; Gbureck, U.	2019
(ii) Principles and practice of antimicrobial therapy of the infected joint replacement	Jones, N.; Berendt, A. R.	2000
El diseño de un sistema de implante dental y su posible influencia para lograr y mantener resultados estéticos a largo plazo	Lazzara, Richard J.	2012
Ultrastructure and Analytical Features of the Sinus Floor Augmentation with Osteograph® and PepGen P-15®	Dahlet, Jean-Christophe; Boukari, Abdessamad; Collavini, Marc; Hemmerlé, Joseph	2013
Implantes cortos. Reporte de 30 implantes. Parte I: oseointegración	Arismendi, Jorge Alberto; Cabrales, Manuel; Duque, Jaiver Andrés; Castañeda, Wilmer	2011
Herodontics–is there a place for maintaining the apparently hopeless tooth?	Meyers, I. A.	2019
Stem cell biology and tissue engineering in dental sciences	Vishwakarma, Ajaykumar; Sharpe, Paul; Shi, Songtao; Ramalingam, Murugan	2014
Tooth and Jaw, Biomechanics of	Müftü, Sinan; Müftü, Ali	2006

Injectable biomaterials for regenerating complex craniofacial tissues	Kretlow, James D.; Young, Simon; Klouda, Leda; Wong, Mark; Mikos, Antonios G.	2009
Flap versus flapless procedure for ridge preservation in alveolar extraction sockets: a histological evaluation in a randomized clinical trial	Iezzi, Giovanna	2014
Histomorphometric Evaluation of a Calcium-Phosphosilicate Putty Bone	Kotsakis, Georgios A.	
Effects of 3D-printed polycaprolactone/β-tricalcium phosphate membranes on guided bone regeneration	Shim, Jin-Hyung; Won, Joo-Yun; Park, Jung-Hyung; Bae, Ji-Hyeon; Ahn, Geunseon; Kim, Chang-Hwan; Lim, Dong-Hyuk; Cho, Dong-Woo; Yun, Won-Soo; Bae, Eun-Bin	2017
Biomaterials for Oral and Dental Tissue Engineering	Tayebi, Lobat; Moharamzadeh, Keyvan	2017
Differences in salivary α-amylase levels among women ith different taste sensitivities.	Sequeira, Márcia; Rodrigues, Lénia; R Costa, Ana; Antunes, Célia; Pinheiro, Cristina; Lamy, Elsa	2012
「生体多機能化インプラント」 を目指して	吉成正雄	2015
Cage subsidence in lateral interbody fusion with transpsoas approach: intraoperative endplate injury or late-onset settling	Satake, Kotaro; Kanemura, Tokumi; Nakashima, Hiroaki; Yamaguchi, Hidetoshi; Segi, Naoki; Ouchida, Jun	2017
Different dental lasers vs conventional technique for second stage surgery	Gabrić, Dragana; Katanec, Davor; Smojver, Igor; Plančak, Darije; Sušić, Mato	2015
Digital Library for Dental Biomaterials	Letic-Gavrilovic, Anka	2009
J. Annie Kamala Florence 2 and PN Sudha 3 1Department of Chemistry, DKM College for Women, Vellore, Tamil Nadu, India, 2Department of Chemistry, Voorhees College, Vellore, Tamil Nadu, India, 3PG & Research Department of Chemistry, DKM College for Women, Vellore, Tamil Nadu, India	Thandapani, Gomathi; Radha, E.; Jayashri, J.	2018
Nano-apatites with designed chemistry and crystallinity for bone regeneration and nanomedical applications	Iafisco, M.; Catalucci, D.	2016
Materials and Bioactive Factors in Dental Restoration and Periodontal Therapy	Bosnjak, Andrija Petar; D'Alpino, Paulo Henrique Perlatti	
New molecules in the tumor necrosis factor ligand and receptor superfamilies with importance for physiological and pathological bone resorption	Lerner, Ulf H.	2004
Application of micro-CT and MRI in clinical and preclinical studies of osteoporosis and related disorders	Jiang, Ye-Bin; Jacobson, Jon; Genant, Harry K.; Zhao, Jenny	2007
Autologous Chondrocyte Implantation	Hambly, Karen; Mithoefer, Kai; Silvers, Holly J.; Mandelbaum, Bert R.	2013
Use of bone morphogenetic protein-2 and dentin matrix protein-1 to enhance the osteointegration of the Onplant system	Hassan, Ali H.; Evans, Carla A.; Zaki, A. Moneim; George, Anne	2003
Anterior cervical discectomy for one-and two-level cervical disc disease: the controversy surrounding the question of whether to fuse, plate, or both	Alvarez, Jaime A.; Hardy, R. W.	1999
1.2 Electrochemical Behavior of Metals in the Biological Milieu	Gilbert, J. L.	2017
Diagnostic guidelines for the histological particle algorithm in the periprosthetic neo-synovial tissue	Perino, G.; Sunitsch, S.; Huber, M.; Ramirez, D.; Gallo, J.; Vaculova, J.; Natu, S.; Kretzer, J. P.; Müller, S.; Thomas, P.	2018
Inducing bone growth using extracellular matrix proteins	Lyngstadaas, Staale Petter; Ellingsen, Jan Eirik; Spahr, Axel; Slaby, Ivan	2003
Reconstruction of extensive long bone defects in sheep using resorbable bioceramics based on silicon stabilized tricalcium phosphate	Mastrogiacomo, Maddalena; Corsi, Alessandro; Francioso, Edda; Comite, Mariasevera Di; Monetti, Francesco; Scaglione, Silvia; Favia, Angela; Crovace, Antonio; Bianco, Paolo; Cancedda, Ranieri	2006
The microbiome and disease: reviewing the links between the oral microbiome, aging, and Alzheimer's disease	Shoemark, Deborah K.; Allen, Shelley J.	2015
Fatores que influenciam no sucesso ou falha de implantes dentários.	Matos, Geraldo Roberto Martins	2010
Biointegrating Materials	Amédée, J.; Bordenave, L.; Durrieu, M.-C.; Fricain, J.-C.; Pothuaud, L.	2009
Do serotonin reuptake inhibitor antidepressants worsen Parkinson's disease? A retrospective case series	Richard, Irene Hegeman; Maughn, Angeline; Kurlan, Roger	1999
Nanofibre based smart pharmaceutical scaffolds for wound repair and regenerations	Dwivedi, Charu; Pandey, Himanshu; C Pandey, Avinash; W Ramteke, Pramod	2016
Therapeutic options for postmenopausal female sexual dysfunction	Al-Azzawi, F.; Bitzer, J.; Brandenburg, U.; Castelo-Branco, C.; Graziottin, A.; Kenemans, P.; Lachowsky, M.; Mimoun, S.; Nappi, R. E.; Palacios, S.	2010
Biological fixation in hip replacement	Hickok, Noreen J.; Purtill, James J.; Marcolongo, Michele; Tuan, Rocky S.	2002
Calcium orthophosphates (CaPO4) and dentistry	Dorozhkin, S. V.	2016
Introduction to corrosion of bioimplants	Novikova, G. E.	2011
Study on repairing canine mandibular defect with Mg–Sr alloy	Wang, Cheng-Yue; Kang, Chun-Yu; Zhang, Shan-Ning; Yang, Jing-Xin; Cheng, Guang	2018
Plaqlle forlnatio11 on Sllrface Inodified de11tal ilnplants	Haustein, Ingn111; MÜller, Wolf-Dieter; Lange, Klaus-Peta; Briedigkeit, Helga; Gäbel, Ulf Bathold	2001
Manejo clínico y tratamiento de la infección periimplantaria. Presentación de 6 casos	Delgado Molina, Esther; Sánchez Garcés, M. A.; Berini Aytés, Leonardo; Gay Escoda, Cosme	1998
INTRODUCTION TO PROCESSING OF BIOIVIATERIALS	Katti, Dhirendra S.; Pandya, Shaunak; Bora, Meghali; Mahida, Rakesh	2010

A 3D FINITE ELEMENT ANALYSIS.	SANNINO, G.; GLORIA, F.; OTTRIA, L.; BARLATTANI, A.	
Zinc-based alloys for degradable vascular stent applications	Mostaed, Ehsan; Sikora-Jasinska, Malgorzata; Drelich, Jaroslaw W.; Vedani, Maurizio	2018
Osteolysis in total hip arthroplasty: biological and clinical aspects	PATHOPHYSIOLOGY, I.	2002
Compatibility of resorbable and nonresorbable guided tissue regeneration membranes in cultures of primary human periodontal ligament fibroblasts and human osteoblast-like cells	Alpar, Berna; Leyhausen, Gabriele; Günay, Huesamettin; Geurtsen, W.	2000
Periostin promotes migration, proliferation, and differentiation of human periodontal ligament mesenchymal stem cells	Wu, Ziqiang; Dai, Wenyong; Wang, Pei; Zhang, Xiaozhen; Tang, Yi; Liu, Lin; Wang, Qiaona; Li, Ming; Tang, Chunbo	2018
Materials for bone regeneration: Current types, bioactive mechanism and updated investigations	He, Yuqi; He, Wei; Fan, Yubo; Li, Xiaoming	2019
Cemented versus cementless hip arthroplasty A review of prosthetic biocompatibility	Santavirta, Seppo; Gristina, Anthony; Konttinen, YrjÖ T.	1992
Current Knowledge: The Regenerative Potential of Periosteum-Derived Cells and Their Application in Oral and Craniofacial Therapy	Stern, J. Kobi; Zhou, Zheng; Rawlings, Aaron; Kao, Solon; Cutler, Christopher; Rios, Hector	2016
Transplantation and Implantation, November 17-20, 2015, Mashhad, Iran	Ghasemi, D.; Momeni, M.	
Abstracts from International Congress of Transplantation and Implantation, November 17-20, 2015, Mashhad, Iran	Aliakbarian, M.	2016
Pathogenesis of prosthesis-related infection	Gallo, Jiří; Kolar, M.; Novotny, R.; Rihakova, P.; Tichá, Vlasta	2003
Recombinant human plasminogen activator inhibitor-1 promotes cementogenic differentiation of human periodontal ligament stem cells	Jin, Hexiu; Choung, Han-Wool; Lim, Ki-Taek; Jin, Bin; Jin, Chengbiao; Chung, Jong-Hoon; Choung, Pill-Hoon	2015
Gomathi Thandapani1, E. Radha1, J. Jayashri1	Florence, J. Annie Kamala; Sudha, P. N.	2018
Self-Assembling Peptide Amphiphile Nanofibers for Angiogenesisand Cardiovascular Disease	Webber, Matthew J.	2011
Total disc replacement arthroplasty using the AcroFlex lumbar disc: a non-human primate model	Cunningham, Bryan W.; Lowery, Gary L.; Serhan, Hassan A.; Dmitriev, Anton E.; Orbegoso, Carlos M.; McAfee, Paul C.; Fraser, Robert D.; Ross, Raymond E.; Kulkarni, Samir S.	2004
Interim use of the Jarvik-7 artificial heart: lessons learned at Presbyterian-University Hospital of Pittsburgh	Griffith, Bartley P.	1989
Urea and creatinine levels in saliva of patients with and without periodontitis	Gaál Kovalčíková, Alexandra; Pančíková, Alexandra; Konečná, Barbora; Klamárová, Tatiana; Novák, Bohuslav; Koval'ová, Eva; Podracká, L'udmila; Celec, Peter; Tóthová, L'ubomíra	
SR Dutta, D. Passi, P. Singh &	Bhuibhar, A.	2015
Bone regeneration in a rabbit critical-sized skull defect using autologous adipose-derived cells	Di Bella, Claudia; Farlie, Peter; Penington, Anthony J.	2008
Annual review of selected scientific literature: A report of the Committee on Scientific Investigation of the American Academy of Restorative Dentistry	Donovan, Terence E.; Marzola, Riccardo; Murphy, Kevin R.; Cagna, David R.; Eichmiller, Frederick; McKee, James R.; Metz, James E.; Albouy, Jean-Pierre; Troeltzsch, Mathias	2018
Multifunctional biomaterials and their bioinspired systems for bioactive molecules delivery	Swartjes, JJTM	2017
Materials, Chemicals and Methods for Dental Applications	Fink, Johannes Karl	2018
Biodegradable polymers for controlled delivery of chemotherapy with and without radiation therapy in the monkey brain	Brem, Henry; Tamargo, Rafael J.; Olivi, Alessandro; Pinn, Michael; Weingart, Jon D.; Wharam, Moody; Epstein, Jonathan I.	1994
TREATMENT OF THE MUCOALVEOLAR COMPLEX IMMEDIA^{TEL}Y AFTER TOOTH EXTRACTION	de Souza Pinto, Vicente; Couy, Karina Chaves; de Souza Pinto, Midori Nozaki; Paulon, Pedro	2015
Nutritional correlates of human oral microbiome	Kato, Ikuko; Vasquez, Adrian; Moyerbrailean, Gregory; Land, Susan; Djuric, Zora; Sun, Jun; Lin, Ho-Sheng; Ram, Jeffrey L.	2017
An ovine model of cranial cruciate ligament reconstruction	Field, J. R.; Adachi, N.; Ochi, M.	2001
M. Saquib Hasnain \S		
Hypersensitivity to Cardiovascular	Svedman, Cecilia; Bruze, Magnus	2018
Distribution of pressures and forces on the wrist after simulated intercarpal fusion and Kienböck's disease	Short, W. H.; Werner, F. W.; Fortino, M. D.; Palmer, A. K.	1992
Assessment of Adhesion and Proliferation of Bone Marrow Mesenchymal Stem Cells in Polymer Matrices with rhGH.	da Silveira Gerzson, Alexandre; Machado, Denise Cantarelli; Rodrigo Marinovic, Daniel; Pagnoncelli, Rogério Miranda	2017
Fungi at the Scene of the Crime: Innocent Bystanders or Accomplices in Oral Infections?	Delaney, Christopher; Kean, Ryan; Short, Bryn; Tumelty, Maria; McLean, William; Nile, Christopher J.; Ramage, Gordon	2018
Mohamadreza Baghaban Eslaminejad Royan Institute for Stem Cell Biology and Technology, ACECR, Tehran, Iran	Hosseini, Samaneh; Jahangir, Shahrbanoo	2017
Adenosine-associated delivery systems	Kazemzadeh-Narbat, Mehdi; Annabi, Nasim; Tamayol, Ali; Oklu, Rahmi; Ghanem, Amyl; Khademhosseini, Ali	2015
BIO-ARTIFICIAL MYOCARDIUM FOR THE TREATMENT OF ISCHEMIC HEART DISEASE: FIRST CLINICAL EXPERIENCE	Chachques, Juan C.	2011
Department of BiomeDical engineering	Family, J. Crayton Pruitt	
Advanced regenerative techniques based on dental pulp stem cells for the treatment of periodontal disease	Seciu, Ana-Maria; Craciunescu, Oana; Zarnescu, Otilia	2018
FACULTAD DE ODONTOLOGÍA	DE CRECIMIENTO, EFECTO DE LA HORMONA	2016

Porosity effect of 3D-printed polycaprolactone membranes on calvarial defect model for guided bone regeneration	Shim, Jin-Hyung; Jeong, Jae-hyang; Won, Joo-Yun; Bae, Ji-Hyeon; Ahn, Geunseon; Jeon, Hojun; Yun, Won-Soo; Bae, Eun-Bin; Choi, Jae-Won; Lee, So-Hyoun	2017
State of the art and recent patents on Mg-based biodegradable bone implants	Neacsu, Patricia; N Ion, Raluca; Mitran, Valentina; I Staras, Adela; Cimpean, Anisoara	2014
Simulation of bone ingrowth	Andreykiv, Andriy	2006
Blood vessel maturation in health and disease and its implications for vascularization of engineered tissues	Sun, Xuetao; Evren, Sevan; Nunes, Sara S.	2015
Nanobiomaterials in dentistry: applications of nanobiomaterials	Grumezescu, Alexandru	2016
Periprosthetic infection—bacteria and the interface between prosthesis and bone	Frommelt, L.	2000
Reoperation rates for symptomatic nonunions in posterior cervical (subaxial) fusions with and without bone morphogenetic protein in a cohort of 1158 patients	Guppy, Kern H.; Harris, Jessica; Chen, Jason; Paxton, Elizabeth W.; Alvarez, Julie; Bernbeck, Johannes	2016
Assessing the potential of mesenchymal stem cells in craniofacial bone repair and regeneration	Waddington, Rachel J.; Jones, S. Quentin; Moseley, Ryan	1988
A comprehensive simulation-based methodology for the design and optimization of orthopaedic internal fixation implants	Arnone, Joshua	2011
Materials for 3D printing in medicine: Metals, polymers, ceramics, hydrogels	Poologasundarampillai, Gowsihan; Nommeots-Nomm, Amy	2017
Histomorphometric evaluation of a calcium-phosphosilicate putty bone substitute in extraction sockets.	Kotsakis, Georgios A.; Joachim, C.; Frédéric, P.; Saroff, Stephen A.; Mahesh, Lanka; Prasad, Hari; Rohrer, Michael D.	2014
Bone Grafting and the Materials for Using in Orthopedics	Harsini, Somayeh Monazzah; Oryan, Ahmad	2018
The oral microbiota: living with a permanent guest	Avila, Maria; Ojcius, David M.; Yilmaz, Özlem	2009
1Thapar University, Patiala, India; 2Bhabha Atomic Research Centre, Mumbai, India	Agnihotri, Shekhar; Dhiman, Navneet K.; Tripathi, Anuj	2017
Current patents on osteoinductive molecules for bone tissue engineering	WH Lo, Kevin; D Ulery, Bret; Deng, Meng; M Ashe, Keshia; T Laurencin, Cato	2011
Pharmacological interventions for osteoporosis in people with chronic kidney disease stages 3-5D	Hara, , T, Hijikata, Y, Matsubara, Y; Watanabe, N	2019
Metformin for preventing the progression of chronic kidney disease	El-Damanawi, , R, Viecelli, AK, Pascoe, EM, Craig, JC, Johnson, DW, Mallett, AJ, Hawley, CM; Hiemstra, TF	2019
Acupuncture for slowing the progression of myopia in children and adolescents	Wei, , ML, Liu, JP, Li, N; Liu, M	2011
Acupuncture for glaucoma	Law, , SK; Li, T	2013
Acupuncture for shoulder pain	Green, , S, Buchbinder, R; Hetrick, SE	2005
Acupuncture for vascular dementia	Peng, , W, Wang, Y, Zhang, Y; Liang, CM	2007
Adjuvant chemotherapy for invasive bladder cancer (individual patient data)	Vale, CL	2006
Action plans with brief patient education for exacerbations in chronic obstructive pulmonary disease	Howcroft, , M, Walters, EH, Wood-Baker, R; Walters, JAE	2016
Abstinence-only programs for HIV infection prevention in high-income countries	Underhill, , K, Operario, D; Montgomery, P	2007
Adenoidectomy for recurrent or chronic nasal symptoms in children	van den Aardweg, , MTA, Schilder, AGM, Herkert, E, Boonacker, CWB; Rovers, MM	2010
Addition of long-acting beta2-agonists to inhaled steroids versus higher dose inhaled steroids in adults and children with persistent asthma	Ducharme, , FM, Ni Chroinin, M, Greenstone, I; Lasserson, TJ	2010
Addition of anti-leukotriene agents to inhaled corticosteroids for adults and adolescents with persistent asthma	Chauhan, , BF, Jeyaraman, MM, Singh Mann, A, Lys, J, Abou-Setta, AM, Zarychanski, R; Ducharme, FM	2017
Acetylcysteine and carbocysteine for acute upper and lower respiratory tract infections in paediatric patients without chronic broncho-pulmonary disease	Chalumeau, , M; Duijvestijn, YCM	2013
Adjunctive steroid therapy for managing pulmonary tuberculosis	Critchley, , JA, Orton, LC; Pearson, F	2014
Abdominal decompression for suspected fetal compromise/pre-eclampsia	Hofmeyr, GJ	2012
Adjunctive therapies for AIDS dementia complex	Uthman, , OA; Abdulmalik, JO	2008
Adjuvant (post-surgery) chemotherapy for early stage epithelial ovarian cancer	Lawrie, , TA, Winter-Roach, BA, Heus, P; Kitchener, HC	2015
Acetylcholinesterase inhibitor treatment for myasthenia gravis	Mehndiratta, , MM, Pandey, S; Kuntzer, T	2014
Adenoidectomy for otitis media in children	van den Aardweg, , MTA, Schilder, AGM, Herkert, E, Boonacker, CWB; Rovers, MM	2010
Adjuvant corticosteroids for reducing death in neonatal bacterial meningitis	Ogunlesi, , TA, Odigwe, CC; Oladapo, OT	2015
Acupuncture for stroke rehabilitation	Yang, , A, Wu, HM, Tang, JL, Xu, L, Yang, M; Liu, GJ	2016
18F-FDG PET for the early diagnosis of Alzheimer's disease dementia and other dementias in people with mild cognitive impairment (MCI)	Smailagic, , N, Vacante, M, Hyde, C, Martin, S, Ukoumunne, O; Sachpekidis, C	2015
Acetaminophen for osteoarthritis	Towheed, , T, Maxwell, L, Judd, M, Catton, M, Hochberg, MC; Wells, GA	2006

Acupuncture for insomnia	Cheuk, , DKL, Yeung, WF, Chung, KF; Wong, V	2012
Abatacept for rheumatoid arthritis	Maxwell, , L; Singh, JA	2009
Acupuncture for symptomatic gastroparesis	Kim, , KH, Lee, MS, Choi, TY; Kim, TH	2018
Active cycle of breathing technique for cystic fibrosis	Mckoy, , NA, Wilson, LM, Saldanha, IJ, Odelola, OA; Robinson, KA	2016
Human' insulin versus animal insulin in people with diabetes mellitus	Richter, , B; Neises, G	2005
A 'test and treat' strategy for elevated wound protease activity for healing in venous leg ulcers	Norman, , G, Westby, MJ, Stubbs, N, Dumville, JC; Cullum, N	2016
Third wave' cognitive and behavioural therapies versus other psychological therapies for depression	Hunot, , V, Moore, THM, Caldwell, DM, Furukawa, TA, Davies, P, Jones, H, Honyashiki, M, Chen, P, Lewis, G; Churchill, R	2013
Third wave' cognitive and behavioural therapies versus treatment as usual for depression	Churchill, , R, Moore, THM, Furukawa, TA, Caldwell, DM, Davies, P, Jones, H, Shinohara, K, Imai, H, Lewis, G; Hunot, V	2013
5-FU for genital warts in non-immunocompromised individuals	Batista, , CS, Atallah, ÁN, Saconato, H; da Silva, EMK	2010
Additional plerixafor to granulocyte colony-stimulating factors for haematopoietic stem cell mobilisation for autologous transplantation in people with malignant lymphoma or multiple myeloma	Hartmann, , T, Hübel, K, Monsef, I, Engert, A; Skoetz, N	2015
Active chest compression-decompression for cardiopulmonary resuscitation	Lafuente-Lafuente, , C; Melero-Bascones, M	2013
Acupuncture for restless legs syndrome	Cui, , Y, Wang, Y; Liu, Z	2008
Acanthopanax for acute ischaemic stroke	Li, , W, Liu, M, Feng, S, Wu, B, Zhang, S, Yang, W; Liu, GJ	2009
Acupuncture for treating fibromyalgia	Deare, , JC, Zheng, Z, Xue, CCL, Liu, JP, Shang, J, Scott, SW; Littlejohn, G	2013
Adjuvant antiviral therapy for recurrent respiratory papillomatosis	Chadha, , NK; James, A	2012
Abdominal drainage versus no drainage post-gastrectomy for gastric cancer	Wang, , Z, Chen, J, Su, K; Dong, Z	2015
Active mind-body movement therapies as an adjunct to or in comparison with pulmonary rehabilitation for people with chronic obstructive pulmonary disease	Gendron, , LM, Nyberg, A, Saey, D, Maltais, F; Lacasse, Y	2018
11C-PIB-PET for the early diagnosis of Alzheimer's disease dementia and other dementias in people with mild cognitive impairment (MCI)	Zhang, , S, Smailagic, N, Hyde, C, Noel-Storr, AH, Takwoingi, Y, McShane, R; Feng, J	2014
Acupuncture for lateral elbow pain	Green, , S, Buchbinder, R, Barnsley, L, Hall, S, White, M, Smidt, N; Assendelft, WJJ	2002
Addition of long-acting beta2-agonists to inhaled corticosteroids versus same dose inhaled corticosteroids for chronic asthma in adults and children	Ducharme, , FM, Ni Chroinin, M, Greenstone, I; Lasserson, TJ	2010
Acupuncture for acute management and rehabilitation of traumatic brain injury	Wong, , V, Cheuk, DKL, Lee, S; Chu, V	2013
5-alpha-reductase inhibitors for prostate cancer prevention	Wilt, , TJ, MacDonald, R, Hagerty, K, Schellhammer, P; Kramer, BS	2008
Addition of anti-leukotriene agents to inhaled corticosteroids for chronic asthma	Ducharme, FM	2004
Addition of long-acting beta2-agonists to inhaled corticosteroids for chronic asthma in children	Chauhan, , BF, Chartrand, C, Ni Chroinin, M, Milan, SJ; Ducharme, FM	2015
Acupuncture for Bell's palsy	Chen, , N, Zhou, M, He, L, Zhou, D; Li, N	2010
Acupuncture for Attention Deficit Hyperactivity Disorder (ADHD) in children and adolescents	Li, , S, Yu, B, Zhou, D, He, C, Kang, L, Wang, X, Jiang, S; Chen, X	2011
Adjunctive steroid therapy versus antibiotics alone for acute endophthalmitis after intraocular procedure	Kim, , CH, Chen, MF; Coleman, AL	2017
Acupuncture and electroacupuncture for the treatment of rheumatoid arthritis	Casimiro, , L, Barnsley, L, Brosseau, L, Milne, S, Welch, V, Tugwell, P; Wells, GA	2005
Acetylsalicylic acid (aspirin) for schizophrenia	Schmidt, , L, Phelps, E, Friedel, J; Shokraneh, F	2019
Adenosine and verapamil for no-reflow during primary percutaneous coronary intervention in people with acute myocardial infarction	Su, , Q, Nyi, TS; Li, L	2015
Adenosine-diphosphate (ADP) receptor antagonists for the prevention of cardiovascular disease in type 2 diabetes mellitus	Valentine, , N, Van de Laar, FA; van Driel, ML	2012
Active body surface warming systems for preventing complications caused by inadvertent perioperative hypothermia in adults	Madrid, , E, Urrútia, G, Roqué i Figuls, M, Pardo-Hernandez, H, Campos, JM, Paniagua, P, Maestre, L; Alonso-Coello, P	2016
Abdominal drainage to prevent intra-peritoneal abscess after open appendectomy for complicated appendicitis	Li, , Z, Zhao, L, Cheng, Y, Cheng, N; Deng, Y	2018
Acyclovir for treating varicella in otherwise healthy children and adolescents	Klassen, , TP; Hartling, L	2005
A therapeutic-only versus prophylactic platelet transfusion strategy for preventing bleeding in patients with haematological disorders after myelosuppressive chemotherapy or stem cell transplantation	Crighton, , GL, Estcourt, LJ, Wood, EM, Trivella, M, Doree, C; Stanworth, S	2015
Acupuncture for mumps in children	He, , J, Jia, P, Zheng, M, Zhang, M; Jiang, H	2015
Active placebos versus antidepressants for depression	Moncrieff, , J, Wessely, S; Hardy, R	2004
Addition of anti-leukotriene agents to inhaled corticosteroids in children with persistent asthma	Chauhan, , BF, Ben Salah, R; Ducharme, FM	2013

Active versus expectant management for women in the third stage of labour	Begley, , CM, Gyte, GML, Devane, D, McGuire, W, Weeks, A; Biesty, LM	2019
Ab interno trabecular bypass surgery with iStent for open-angle glaucoma	Le, , JT, Bicket, AK, Wang, L; Li, T	2019
Adjuvant gonadotropin-releasing hormone analogues for the prevention of chemotherapy-induced premature ovarian failure in premenopausal women	Chen, , H, Xiao, L, Li, J, Cui, L; Huang, W	2019
Acetyl-L-carnitine for the treatment of diabetic peripheral neuropathy	Rolim, , LCSP, da Silva, EMK, Flumignan, RLG, Abreu, MM; Dib, SA	2019
Acupuncture and acupressure for premenstrual syndrome	Armour, , M, Ee, CC, Hao, J, Wilson, TM, Yao, SS; Smith, CA	2018
Acupuncture for functional dyspepsia	Lan, , L, Zeng, F, Liu, GJ, Ying, L, Wu, X, Liu, M; Liang, FR	2014
Addition of intravenous aminophylline to inhaled beta2-agonists in adults with acute asthma	Nair, , P, Milan, SJ; Rowe, BH	2012
Acupuncture for treatment of irritable bowel syndrome	Manheimer, , E, Cheng, K, Wieland, LS, Min, LS, Shen, X, Berman, BM; Lao, L	2012
Adalimumab for treating rheumatoid arthritis	Navarro-Sarabia, , F, Ariza-Ariza, R, Hernandez-Cruz, B; Villanueva, I	2005
Acupuncture for acute stroke	Xu, , M, Li, D; Zhang, S	2018
Activity monitors for increasing physical activity in adult stroke survivors	Lynch, , EA, Jones, TM, Simpson, DB, Fini, NA, Kuys, SS, Borschmann, K, Kramer, S, Johnson, L, Callisaya, ML, Mahendran, N, Janssen, H; English, C	2018
Active case finding in contacts of people with tuberculosis	Fox, , GJ, Dobler, CC; Marks, GB	2011
Addition of intravenous beta2-agonists to inhaled beta2-agonists for acute asthma	Travers, , AH, Milan, SJ, Jones, AP, Camargo Jr, CA; Rowe, BH	2012
Adhesion prevention agents for gynaecological surgery: an overview of Cochrane reviews	Hindocha, , A, Beere, L, Dias, S, Watson, A; Ahmad, G	2015
Abacavir-based triple nucleoside regimens for maintenance therapy in patients with HIV	Cruciani, , M, Mengoli, C, Serpelloni, G, Parisi, SG, Malena, M; Bosco, O	2013
Addition to inhaled corticosteroids of long-acting beta2-agonists versus anti-leukotrienes for chronic asthma	Chauhan, , BF; Ducharme, FM	2014
Acupuncture for peripheral joint osteoarthritis	Manheimer, , E, Cheng, K, Linde, K, Lao, L, Yoo, J, Wieland, S, van der Windt, DAWM, Berman, BM; Bouter, LM	2010
Acamprosate for alcohol dependence	Rösner, , S, Hackl-Herrwerth, A, Leucht, S, Lehert, P, Vecchi, S; Soyka, M	2010
123I-MIBG scintigraphy and 18F-FDG-PET imaging for diagnosing neuroblastoma	Bleeker, , G, Tytgat, GAM, Adam, JA, Caron, HN, Kremer, LCM, Hooft, L; van Dalen, EC	2015
Acupuncture for chronic hepatitis B	Kong, , DZ, Liang, N, Yang, GL, Zhang, Z, Liu, Y, Yang, Y, Liu, YX, Wang, QG, Zhang, F, Zhang, HY, Nikolova, D, Jakobsen, JC, Gluud, C; Liu, JP	2019
Acupuncture for polycystic ovarian syndrome	Lim, , CED, Ng, RWC, Cheng, NCL, Zhang, GS; Chen, H	2019
Adjuvant radiotherapy following radical prostatectomy for prostate cancer	Daly, , T, Hickey, BE, Lehman, M, Francis, DP; See, AM	2011
Alemtuzumab for patients with chronic lymphocytic leukaemia	Skoetz, , N, Bauer, K, Elter, T, Monsef, I, Roloff, V, Hallek, M; Engert, A	2012
Anabolic steroids for the treatment of weight loss in HIV-infected individuals	Johns, , KKJ, Beddall, MJ; Corrin, RC	2005
Adjuvant radiotherapy for stage I endometrial cancer	Kong, , A, Johnson, N, Kitchener, HC; Lawrie, TA	2012
Alpha-2 agonists for long-term sedation during mechanical ventilation in critically ill patients	Chen, , K, Lu, Z, Xin, YC, Cai, Y, Chen, Y; Pan, SM	2015
Airway clearance techniques for chronic obstructive pulmonary disease	Osadnik, , CR, McDonald, CF, Jones, AP; Holland, AE	2012
Alternatives to inpatient mental health care for children and young people	Shepperd, , S, Doll, H, Gowers, S, James, A, Fazel, M, Fitzpatrick, R; Pollock, J	2009
Aldosterone antagonists for preventing the progression of chronic kidney disease	Bolignano, , D, Palmer, SC, Navaneethan, SD; Strippoli, GFM	2014
Angiotensin-converting enzyme inhibitors and angiotensin receptor blockers for adults with early (stage 1 to 3) non-diabetic chronic kidney disease	Sharma, , P, Blackburn, RC, Parke, CL, McCullough, K, Marks, A; Black, C	2011
Alpha lipoic acid for dementia	Klugman, , A, Sauer, J, Tabet, N; Howard, R	2004
Ambulatory and short-burst oxygen for interstitial lung disease	Sharp, , C, Adamali, H; Millar, AB	2016
Amniotomy for shortening spontaneous labour	Smyth, , RMD, Markham, C; Dowswell, T	2013
Androgens for the anaemia of chronic kidney disease in adults	Yang, , Q, Abudou, M, Xie, XS; Wu, T	2014
An overview of reviews evaluating the effectiveness of financial incentives in changing healthcare professional behaviours and patient outcomes	Flodgren, , G, Eccles, MP, Shepperd, S, Scott, A, Parmelli, E; Beyer, FR	2011
Amodiaquine for treating malaria	Olliaro, , PL; Mussano, P	2003
Alcoholics Anonymous and other 12-step programmes for alcohol dependence	Ferri, , M, Amato, L; Davoli, M	2006

Amniocentesis and chorionic villus sampling for prenatal diagnosis	Alfirevic, , Z, Navaratnam, K; Mujezinovic, F	2017
Aminosalicylates for induction of remission or response in Crohn's disease	Lim, , WC, Wang, Y, MacDonald, JK; Hanauer, S	2016
Advanced training in trauma life support for ambulance crews	Jayaraman, , S, Sethi, D; Wong, R	2014
Ambroxol for women at risk of preterm birth for preventing neonatal respiratory distress syndrome	Gonzalez Garay, , AG, Reveiz, L, Velasco Hidalgo, L; Solis Galicia, C	2014
Angioplasty versus stenting for iliac artery lesions	Bekken, , J, Jongsma, H, Ayez, N, Hoogewerf, CJ, Van Weel, V; Fioole, B	2015
Adjuvant Therapy for completely resected Stage II Colon Cancer	Figueredo, , A, Coombes, ME; Mukherjee, S	2008
Anaesthetic and sedative agents used for electrical cardioversion	Lewis, , SR, Nicholson, A, Reed, SS, Kenth, JJ, Alderson, P; Smith, AF	2015
Alpha-2 adrenergic agonists for the prevention of cardiac complications among adults undergoing surgery	Duncan, , D, Sankar, A, Beattie, WS; Wijeysundera, DN	2018
Adjuvant therapy with antidepressants for the management of inflammatory bowel disease	Mikocka-Walus, , A, Prady, SL, Pollok, J, Esterman, AJ, Gordon, AL, Knowles, S; Andrews, JM	2019
Aerosolized prostacyclins for acute respiratory distress syndrome (ARDS)	Afshari, , A, Bastholm Bille, A; Allingstrup, M	2017
Alternative lipid emulsions versus pure soy oil based lipid emulsions for parenterally fed preterm infants	Kapoor, , V, Glover, R; Malviya, MN	2015
Advanced trauma life support training for hospital staff	Jayaraman, , S, Sethi, D, Chinnock, P; Wong, R	2014
Allopurinol for chronic gout	Seth, , R, Kydd, ASR, Buchbinder, R, Bombardier, C; Edwards, CJ	2014
Anaesthesia for treating distal radial fracture in adults	Handoll, , HHG, Madhok, R; Dodds, C	2002
Allopurinol for chronic prostatitis	McNaughton Collins, , M; Wilt, TJ	2002
Air versus saline in the loss of resistance technique for identification of the epidural space	Antibas, , PL, do Nascimento Junior, P, Braz, LG, Vitor Pereira Doles, J, Módolo, NSP; El Dib, R	2014
Adrenaline auto-injectors for the treatment of anaphylaxis with and without cardiovascular collapse in the community	Sheikh, , A, Simons, FER, Barbour, V; Worth, A	2012
Alendronate for the primary and secondary prevention of osteoporotic fractures in postmenopausal women	Wells, , GA, Cranney, A, Peterson, J, Boucher, M, Shea, B, Welch, V, Coyle, D; Tugwell, P	2008
Allogeneic hematopoietic cell transplantation for adult acute lymphoblastic leukemia (ALL) in first complete remission	Pidala, , J, Djulbegovic, B, Anasetti, C, Kharfan-Dabaja, M; Kumar, A	2011
Altered dietary salt intake for people with chronic kidney disease	McMahon, , EJ, Campbell, KL, Bauer, JD; Mudge, DW	2015
Aerobic exercise interventions for adults living with HIV/AIDS	O'Brien, , K, Nixon, S, Tynan, AM; Glazier, R	2010
Aloe vera for prevention and treatment of infusion phlebitis	Zheng, , GH, Yang, L, Chen, HY, Chu, JF; Mei, L	2014
Adjuvant platinum-based chemotherapy for early stage cervical cancer	Falcetta, , FS, Medeiros, LRF, Edelweiss, MI, Pohlmann, PR, Stein, AT; Rosa, DD	2016
Angiotensin-converting enzyme (ACE) inhibitors for proteinuria and microalbuminuria in people with sickle cell disease	Sasongko, , TH, Nagalla, S; Ballas, SK	2015
Advance provision of emergency contraception for pregnancy prevention	Polis, , CB, Grimes, DA, Schaffer, K, Blanchard, K, Glasier, A; Harper, C	2007
Amantadine and rimantadine for influenza A in children and the elderly	Alves Galvão, , MG, Rocha Crispino Santos, MA; Alves da Cunha, AJL	2014
Anaesthetic interventions for prevention of awareness during surgery	Messina, , AG, Wang, M, Ward, MJ, Wilker, CC, Smith, BB, Vezina, DP; Pace, NL	2016
Adjuvant progestagens for endometrial cancer	Martin-Hirsch, , PPL, Bryant, A, Keep, SL, Kitchener, HC; Lilford, R	2011
AD-8 for detection of dementia across a variety of healthcare settings	Hendry, , K, Green, C, McShane, R, Noel-Storr, AH, Stott, DJ, Anwer, S, Sutton, AJ, Burton, JK; Quinn, TJ	2019
Advance misoprostol distribution for preventing and treating postpartum haemorrhage	Oladapo, , OT, Fawole, B, Blum, J; Abalos, E	2012
Allergen injection immunotherapy for seasonal allergic rhinitis	Calderon, , MA, Alves, B, Jacobson, M, Hurwitz, B, Sheikh, A; Durham, S	2007
Aerobic exercise to improve cognitive function in older people without known cognitive impairment	Young, , J, Angevaren, M, Rusted, J; Tabet, N	2015
Ambulatory oxygen for people with chronic obstructive pulmonary disease who are not hypoxaemic at rest	Ameer, , F, Carson, KV, Usmani, ZA; Smith, BJ	2014
Adverse effects of biologics: a network meta-analysis and Cochrane overview	Singh, , JA, Wells, GA, Christensen, R, Tanjong Ghogomu, E, Maxwell, LJ, MacDonald, JK, Filippini, G, Skoetz, N, Francis, DK, Lopes, LC, Guyatt, GH, Schmitt, J, La Mantia, L, Weberschock, T, Roos, JF, Siebert, H, Hershan, S, Cameron, C, Lunn, MPT, Tugwell, P; Buchbinder, R	2011
Altered dietary salt for preventing pre-eclampsia, and its complications	Duley, , L, Henderson-Smart, DJ; Meher, S	2005
alemtuzumab versus interferon beta 1a for relapsing-remitting multiple sclerosis	Zhang, , J, Shi, S, Zhang, Y, Luo, J, Xiao, Y, Meng, L; Yang, X	2017
Almitrine-Raubasine combination for dementia	Yang, , W, Liu, M, Teng, J, Hao, Z, Wu, B, Wu, T; Liu, GJ	2011
Alternative injectable materials for vocal fold medialisation in unilateral vocal fold paralysis	Lakhani, , R, Fishman, JM, Bleach, N, Costello, D; Birchall, M	2012

Albendazole alone or in combination with microfilaricidal drugs for lymphatic filariasis	Macfarlane, , CL, Budhathoki, SS, Johnson, S, Richardson, M; Garner, P	2019
Angiotensin converting enzyme inhibitors and angiotensin II receptor antagonists for preventing the progression of diabetic kidney disease	Strippoli, , GFM, Bonifati, C, Craig, ME, Navaneethan, SD; Craig, JC	2006
Amitriptyline versus placebo for major depressive disorder	Leucht, , C, Huhn, M; Leucht, S	2012
Allogeneic stem cell transplantation versus conventional therapy for advanced primary cutaneous T-cell lymphoma	Schlaak, , M, Pickenhain, J, Theurich, S, Skoetz, N, von Bergwelt-Baildon, M; Kurschat, P	2013
Adverse side effects of dexamethasone in surgical patients	Polderman, , JAW, Farhang-Razi, V, Van Dieren, S, Kranke, P, DeVries, JH, Hollmann, MW, Preckel, B; Hermanides, J	2018
Alternative agents to prophylactic platelet transfusion for preventing bleeding in people with thrombocytopenia due to chronic bone marrow failure: a meta-analysis and systematic review	Desborough, , M, Hadjinicolaou, AV, Chaimani, A, Trivella, M, Vyas, P, Doree, C, Hopewell, S, Stanworth, SJ; Estcourt, LJ	2016
Angioplasty versus stenting for subclavian artery stenosis	Iared, , W, Mourão, JE, Puchnick, A, Soma, F; Shigueoka, DC	2014
Allergen-specific oral immunotherapy for peanut allergy	Nurmatov, , U, Venderbosch, I, Devereux, G, Simons, FER; Sheikh, A	2012
Alprazolam for depression	van Marwijk, , H, Allick, G, Wegman, F, Bax, A; Riphagen, II	2012
Anabolic-androgenic steroids for alcoholic liver disease	Rambaldi, , A; Gluud, C	2006
Advising patients to increase fluid intake for treating acute respiratory infections	Guppy, , MPB, Mickan, SM, Del Mar, CB, Thorning, S; Rack, A	2011
Aminoadamantanes for chronic hepatitis C	Lamers, , MH, Broekman, M, Drenth, JPH; Gluud, C	2014
Alpha-1 proteinase inhibitor (a1PI) for preventing chronic lung disease in preterm infants	Shah, , PS; Ohlsson, A	2001
Alpha-blockers as medical expulsive therapy for ureteral stones	Campschroer, , T, Zhu, X, Vernooij, RWM; Lock, MTWT	2018
Alpha-foetoprotein and/or liver ultrasonography for screening of hepatocellular carcinoma in patients with chronic hepatitis B	Aghoram, , R, Cai, P; Dickinson, JA	2012
Airway physical examination tests for detection of difficult airway management in apparently normal adult patients	Roth, , D, Pace, NL, Lee, A, Hovhannisyan, K, Warenits, AM, Arrich, J; Herkner, H	2018
Amphotericin B lipid soluble formulations versus amphotericin B in cancer patients with neutropenia	Johansen, , HK; Gøtzsche, PC	2014
Alexander technique for chronic asthma	Dennis, , JA; Cates, CJ	2012
Adverse events in people taking macrolide antibiotics versus placebo for any indication	Hansen, , MP, Scott, AM, McCullough, A, Thorning, S, Aronson, JK, Beller, EM, Glasziou, PP, Hoffmann, TC, Clark, J; Del Mar, CB	2019
Alternative magnesium sulphate regimens for women with pre-eclampsia and eclampsia	Duley, , L, Matar, HE, Almerie, MQ; Hall, DR	2010
Alginate dressings for treating pressure ulcers	Dumville, , JC, Keogh, SJ, Liu, Z, Stubbs, N, Walker, RM; Fortnam, M	2015
Aflibercept for neovascular age-related macular degeneration	Sarwar, , S, Clearfield, E, Soliman, MK, Sadiq, MA, Baldwin, AJ, Hanout, M, Agarwal, A, Sepah, YJ, Do, DV; Nguyen, QD	2016
Anaesthetic regimens for day-procedure laparoscopic cholecystectomy	Vaughan, , J, Nagendran, M, Cooper, J, Davidson, BR; Gurusamy, KS	2014
Agomelatine versus other antidepressive agents for major depression	Guaiana, , G, Gupta, S, Chiodo, D, Davies, SJC, Haederle, K; Koesters, M	2013
Anakinra for rheumatoid arthritis	Mertens, , M; Singh, JA	2009
Adrenaline (epinephrine) for the treatment of anaphylaxis with and without shock	Sheikh, , A, Shehata, YA, Brown, SGA; Simons, FER	2008
Aerosolized diuretics for preterm infants with (or developing) chronic lung disease	Brion, , LP, Primhak, RA; Yong, W	2006
Alginate dressings for venous leg ulcers	O'Meara, , S, Martyn-St James, M; Adderley, UJ	2015
Alkylating agents for Waldenstrom's macroglobulinaemia	Yang, , K, Tan, J; Wu, T	2009
Ambulatory versus conventional methods for monitoring blood pressure during pregnancy	Bergel, , E, Carroli, G; Althabe, F	2002
Altered dietary salt intake for preventing and treating diabetic kidney disease	Suckling, , RJ, He, FJ; MacGregor, GA	2010
Angiotensin receptor blockers for heart failure	Heran, , BS, Musini, VM, Bassett, K, Taylor, RS; Wright, JM	2012
Angioplasty versus bare metal stenting for superficial femoral artery lesions	Chowdhury, , MM, McLain, AD; Twine, CP	2014
Analgesia in patients with acute abdominal pain	Manterola, , C, Vial, M, Moraga, J; Astudillo, P	2011
Androgens (dehydroepiandrosterone or testosterone) for women undergoing assisted reproduction	Nagels, , HE, Rishworth, JR, Siristatidis, CS; Kroon, B	2015
Alemtuzumab for multiple sclerosis	Riera, , R, Porfírio, GJM; Torloni, MR	2016
Aerobic physical exercise for adult patients with haematological malignancies	Knips, , L, Bergenthal, N, Streckmann, F, Monsef, I, Elter, T; Skoetz, N	2019
Aspirin for vascular dementia	Rands, , G; Orrell, M	2000
Artesunate versus quinine for treating severe malaria	Sinclair, , D, Donegan, S, Isba, R; Lalloo, DG	2012

Ascorbic acid for the treatment of Charcot-Marie-Tooth disease	Gess, , B, Baets, J, De Jonghe, P, Reilly, MM, Pareyson, D; Young, P	2015
Aspirin and/or heparin for women with unexplained recurrent miscarriage with or without inherited thrombophilia	de Jong, , PG, Kaandorp, S, Di Nisio, M, Goddijn, M; Middeldorp, S	2014
Ataluren and similar compounds (specific therapies for premature termination codon class I mutations) for cystic fibrosis	Aslam, , AA, Higgins, C, Sinha, IP; Southern, KW	2017
Antiretroviral regimens for patients with HIV who fail first-line antiretroviral therapy	Humphreys, , EH, Chang, LW; Harris, J	2010
Anti-fibrinolytic use for minimising perioperative allogeneic blood transfusion	Henry, , DA, Carless, PA, Moxey, AJ, O'Connell, D, Stokes, BJ, Fergusson, DA; Ker, K	2011
Anti-GD2 antibody-containing immunotherapy postconsolidation therapy for people with high-risk neuroblastoma treated with autologous haematopoietic stem cell transplantation	Peinemann, , F, van Dalen, EC, Enk, H; Tytgat, GAM	2019
Artemisinin derivatives for treating severe malaria	McIntosh, , H; Olliaro, P	1998
Aripiprazole versus placebo for schizophrenia	Belgamwar, , RB; El-Sayeh, HGG	2011
Anti-leukotriene agents compared to inhaled corticosteroids in the management of recurrent and/or chronic asthma in adults and children	Chauhan, , BF; Ducharme, FM	2012
Antithrombotic treatment after stroke due to intracerebral haemorrhage	Perry, , LA, Berge, E, Bowditch, J, Forfang, E, Rønning, OM, Hankey, GJ, Villanueva, E; Al-Shahi Salman, R	2017
Antiviral treatment for preventing postherpetic neuralgia	Chen, , N, Li, Q, Yang, J, Zhou, M, Zhou, D; He, L	2014
Aspirin, steroidal and non-steroidal anti-inflammatory drugs for the treatment of Alzheimer's disease	Jaturapatporn, , D, Isaac, MGEKN, McCleery, J; Tabet, N	2012
Antivirals for idiopathic sudden sensorineural hearing loss	Awad, , Z, Huins, C; Pothier, DD	2012
Anti-vascular endothelial growth factor for neovascular glaucoma	Simha, , A, Braganza, A, Abraham, L, Samuel, P; Lindsley, K	2013
Anti-IL-12/23p40 antibodies for induction of remission in Crohn's disease	MacDonald, , JK, Nguyen, TM, Khanna, R; Timmer, A	2016
Aripiprazole for schizophrenia	El-Sayeh, , HG; Morganti, C	2006
Anti-hypertensive drugs as disease-modifying agents for Parkinson's disease: evidence from observational studies and clinical trials	Rees, , K, Stowe, R, Patel, S, Ives, N, Breen, K, Ben-Shlomo, Y; Clarke, CE	2011
Aromatherapy for dementia	Forrester, , LT, Maayan, N, Orrell, M, Spector, AE, Buchan, LD; Soares-Weiser, K	2014
Anti-spasticity agents for multiple sclerosis	Shakespeare, , D, Boggild, M; Young, CA	2003
Anti-inflammatory medications for obstructive sleep apnea in children	Kuhle, , S; Urschitz, MS	2011
Aripiprazole versus typical antipsychotic drugs for schizophrenia	Bhattacharjee, , J; El-Sayeh, HG	2008
Antithrombin for the prevention of intraventricular hemorrhage in very preterm infants	Bruschettini, , M, Romantsik, O, Zappettini, S, Banzi, R, Ramenghi, LA; Calevo, MG	2016
Audio-visual presentation of information for informed consent for participation in clinical trials	Synnot, , A, Ryan, R, Prictor, M, Fetherstonhaugh, D; Parker, B	2014
Aqueous shunts for glaucoma	Tseng, , VL, Coleman, AL, Chang, MY; Caprioli, J	2017
Anti-vascular endothelial growth factor for macular oedema secondary to branch retinal vein occlusion	Mitry, , D, Bunce, C; Charteris, D	2013
Antiretroviral post-exposure prophylaxis (PEP) for occupational HIV exposure	Young, , T, Arens, FJ, Kennedy, GE, Laurie, JW; Rutherford, GW	2007
Antiviral agents for infectious mononucleosis (glandular fever)	De Paor, , M, O'Brien, K, Fahey, T; Smith, SM	2016
Antiretroviral therapy for prevention of HIV transmission in HIV-discordant couples	Anglemyer, , A, Rutherford, GW, Horvath, T, Baggaley, RC, Egger, M; Siegfried, N	2013
Appendectomy versus antibiotic treatment for acute appendicitis	Wilms, , IMHA, de Hoog, DENM, de Visser, DC; Janzing, HMJ	2011
Antithyroid drug regimen for treating Graves' hyperthyroidism	Abraham, , P, Avenell, A, McGeoch, SC, Clark, LF; Bevan, JS	2010
Antiviral prophylaxis for the prevention of chronic hepatitis C virus in patients undergoing liver transplantation	Gurusamy, , KS, Tsochatzis, E, Toon, CD, Davidson, BR; Burroughs, AK	2013
Atorvastatin for lowering lipids	Adams, , SP, Tsang, M; Wright, JM	2015
Aripiprazole alone or in combination for acute mania	Brown, , R, Taylor, MJ; Geddes, J	2013
Antithrombin for respiratory distress syndrome in preterm infants	Bassler, , D, Millar, D; Schmidt, B	2006
Antiviral interventions for liver transplant patients with recurrent graft infection due to hepatitis C virus	Gurusamy, , KS, Tsochatzis, E, Toon, CD, Xirouchakis, E, Burroughs, AK; Davidson, BR	2013
Atypical antipsychotics for aggression and psychosis in Alzheimer's disease	Ballard, , CG, Waite, J; Birks, J	2006
Anti-histamines for prolonged non-specific cough in children	Chang, , AB, Peake, J; McElrea, MS	2008
Aqueous shunts with mitomycin C versus aqueous shunts alone for glaucoma	Foo, , VHX, Htoon, HM, Welsbie, DS; Perera, SA	2019
Antistaphylococcal immunoglobulins to prevent staphylococcal infection in very low birth weight infants	Shah, , PS; Kaufman, DA	2009
Anti-vascular endothelial growth factor for proliferative diabetic retinopathy	Martinez-Zapata, , MJ, Martí-Carvajal, AJ, Solà, I, Pijoán, JI, Buil-Calvo, JA, Cordero, JA; Evans, JR	2014

Anti-inflammatory drugs and analgesics for managing symptoms in people with cystic fibrosis-related arthritis	Thornton, , J; Rangaraj, S	2016
Anti-angiogenic therapies for metastatic colorectal cancer	Wagner, , ADADW, Arnold, D, Grothey, AAG, Haerting, J; Unverzagt, S	2009
Anti-tuberculous therapy for maintenance of remission in Crohn's disease	Patton, , PH, Parker, CE, MacDonald, JK; Chande, N	2016
Atrial natriuretic peptide for preventing and treating acute kidney injury	Nigwekar, , SU, Navaneethan, SD, Parikh, CR; Hix, JK	2009
Anti-inflammatory treatment for carditis in acute rheumatic fever	Cilliers, , A, Adler, AJ; Saloojee, H	2015
Anti-IL5 therapies for asthma	Farne, , HA, Wilson, A, Powell, C, Bax, L; Milan, SJ	2017
Anti-D administration after childbirth for preventing Rhesus alloimmunisation	Crowther, , CA; Middleton, P	1997
Arthroplasties (with and without bone cement) for proximal femoral fractures in adults	Parker, , MJ, Gurusamy, KS; Azegami, S	2010
Appetite stimulants for people with cystic fibrosis	Chinuck, , R, Dewar, J, Baldwin, DR; Hendron, E	2014
Antiviral agents for treatment of herpes simplex virus infection in neonates	Jones, , CA, Walker, KS; Badawi, N	2009
Arginine supplementation for prevention of necrotising enterocolitis in preterm infants	Shah, , PS, Shah, VS; Kelly, LE	2017
Anti-D administration after spontaneous miscarriage for preventing Rhesus alloimmunisation	Karanth, , L, Jaafar, SH, Kanagasabai, S, Nair, NS; Barua, A	2013
Antiviral treatment for Bell's palsy (idiopathic facial paralysis)	Gagyor, , I, Madhok, VB, Daly, F; Sullivan, F	2019
Artemether for severe malaria	Esu, , EB, Effa, EE, Opie, ON; Meremikwu, MM	2019
Aquablation of the prostate for the treatment of lower urinary tract symptoms in men with benign prostatic hyperplasia	Hwang, , EC, Jung, JH, Borofsky, M, Kim, MH; Dahm, P	2019
Atherectomy for peripheral arterial disease	Ambler, , GK, Radwan, R, Hayes, PD; Twine, CP	2014
Anti-TNF-α treatment for pelvic pain associated with endometriosis	Lu, , D, Song, H; Shi, G	2013
Antiretroviral therapy (ART) for treating HIV infection in ART-eligible pregnant women	Sturt, , AS, Dokubo, EK; Sint, TT	2010
Assistive technology for memory support in dementia	Van der Roest, , HG, Wenborn, J, Pastink, C, Dröes, RM; Orrell, M	2017
Atypical antipsychotics for disruptive behaviour disorders in children and youths	Loy, , JH, Merry, SN, Hetrick, SE; Stasiak, K	2017
Antiretrovirals for reducing the risk of mother-to-child transmission of HIV infection	Siegfried, , N, van der Merwe, L, Brocklehurst, P; Sint, TT	2011
Antiretroviral pre-exposure prophylaxis (PrEP) for preventing HIV in high-risk individuals	Okwundu, , CI, Uthman, OA; Okoromah, CAN	2012
Aquatic exercise for the treatment of knee and hip osteoarthritis	Bartels, , EM, Juhl, CB, Christensen, R, Hagen, KB, Danneskiold-Samsøe, B, Dagfinrud, H; Lund, H	2016
Anti-vascular endothelial growth factor for choroidal neovascularisation in people with pathological myopia	Zhu, , Y, Zhang, T, Xu, G; Peng, L	2016
Aspirin as adjunctive treatment for giant cell arteritis	Mollan, , SP, Sharrack, N, Burdon, MA; Denniston, AK	2014
Arthroscopic debridement for knee osteoarthritis	Laupattarakasem, , W, Laopaiboon, M, Laupattarakasem, P; Sumananont, C	2008
Anti-vascular endothelial growth factor for neovascular age-related macular degeneration	Solomon, , SD, Lindsley, K, Vedula, SS, Krzystolik, MG; Hawkins, BS	2019
Atypical antipsychotics for psychosis in adolescents	Kumar, , A, Datta, SS, Wright, SD, Furtado, VA; Russell, PS	2013
Antithrombotic drugs for carotid artery dissection	Lyrer, , P; Engelter, S	2010
Anti-IgE therapy for allergic bronchopulmonary aspergillosis in people with cystic fibrosis	Jat, , KR, Walia, DK; Khairwa, A	2018
Aromatase inhibitors (letrozole) for subfertile women with polycystic ovary syndrome	Franik, , S, Eltrop, SM, Kremer, JAM, Kiesel, L; Farquhar, C	2018
Aromatase inhibitors for uterine fibroids	Song, , H, Lu, D, Navaratnam, K; Shi, G	2013
Anti-angiogenic therapy for high-grade glioma	Ameratunga, , M, Pavlakis, N, Wheeler, H, Grant, R, Simes, J; Khasraw, M	2018
Atypical antipsychotics for people with both schizophrenia and depression	Furtado, , VA, Srihari, V; Kumar, A	2008
Assisted reproductive technology: an overview of Cochrane Reviews	Farquhar, , C; Marjoribanks, J	2018
Artemisinin-based combination therapy for treating uncomplicated Plasmodium vivax malaria	Gogtay, , N, Kannan, S, Thatte, UM, Olliaro, PL; Sinclair, D	2013
Artificial corneas versus donor corneas for repeat corneal transplants	Akpek, , EK, Alkharashi, M, Hwang, FS, Ng, SM; Lindsley, K	2014
Assistive devices, hip precautions, environmental modifications and training to prevent dislocation and improve function after hip arthroplasty	Smith, , TO, Jepson, P, Beswick, A, Sands, G, Drummond, A, Davis, ET; Sackley, CM	2016
Anti-tumour necrosis factor biological therapies for the treatment of uveitic macular oedema (UMO) for non-infectious uveitis	Barry, , RJ, Tallouzi, MO, Bucknall, N, Mathers, JM, Murray, PI, Calvert, MJ, Moore, DJ; Denniston, AK	2018

Audit and feedback: effects on professional practice and healthcare outcomes	Ivers, , N, Jamtvedt, G, Flottorp, S, Young, JM, Odgaard-Jensen, J, French, SD, O'Brien, MA, Johansen, M, Grimshaw, J; Oxman, AD	2012
Antistreptococcal interventions for guttate and chronic plaque psoriasis	Dupire, , G, Droitcourt, C, Hughes, C; Le Cleach, L	2019
Anti-vascular endothelial growth factor for macular oedema secondary to central retinal vein occlusion	Braithwaite, , T, Nanji, AA, Lindsley, K; Greenberg, PB	2014
Artemisinin-based combination therapy for treating uncomplicated malaria	Sinclair, , D, Zani, B, Donegan, S, Olliaro, P; Garner, P	2009
Aripiprazole versus other atypical antipsychotics for schizophrenia	Khanna, , P, Suo, T, Komossa, K, Ma, H, Rummel-Kluge, C, El-Sayeh, HG, Leucht, S; Xia, J	2014
Art therapy for people with dementia	Deshmukh, , SR, Holmes, J; Cardno, A	2018
Anti-vascular endothelial growth factor for prevention of postoperative vitreous cavity haemorrhage after vitrectomy for proliferative diabetic retinopathy	Smith, , JM; Steel, DHW	2015
Anti-vascular endothelial growth factor for control of wound healing in glaucoma surgery	Cheng, , JW, Cheng, SW, Wei, RL; Lu, GC	2016
Anti-vascular endothelial growth factor for diabetic macular oedema: a network meta-analysis	Virgili, , G, Parravano, M, Evans, JR, Gordon, I; Lucenteforte, E	2018
Behavioral interventions to promote condom use among women living with HIV	Carvalho, , FT, Gonçalves, TR, Faria, ER, Shoveller, JA, Piccinini, CA, Ramos, MC; Medeiros, LRF	2011
Beclometasone for chronic obstructive pulmonary disease	De Coster, , DA, Jones, M; Thakrar, N	2013
Biologics for rheumatoid arthritis: an overview of Cochrane reviews	Singh, , JA, Christensen, R, Wells, GA, Suarez-Almazor, ME, Buchbinder, R, Lopez-Olivo, MA, Tanjong Ghogomu, E; Tugwell, P	2009
Bile acids for viral hepatitis	Chen, , W, Liu, JP; Gluud, C	2007
Back schools for acute and subacute non-specific low-back pain	Poquet, , N, Lin, CWC, Heymans, MW, van Tulder, MW, Esmail, R, Koes, BW; Maher, CG	2016
Biologics or tofacitinib for rheumatoid arthritis in incomplete responders to methotrexate or other traditional disease-modifying anti-rheumatic drugs: a systematic review and network meta-analysis	Singh, , JA, Hossain, A, Tanjong Ghogomu, E, Kotb, A, Christensen, R, Mudano, AS, Maxwell, LJ, Shah, NP, Tugwell, P; Wells, GA	2016
Behavioural and cognitive-behavioural interventions for outwardly-directed aggressive behaviour in people with intellectual disabilities	Ali, , A, Hall, I, Blickwedel, J; Hassiotis, A	2015
Bezafibrate for primary biliary cirrhosis	Rudic, , JS, Poropat, G, Krstic, MN, Bjelakovic, G; Gluud, C	2012
Biofeedback for pain management during labour	Barragán Loayza, , IM, Solà, I; Juandó Prats, C	2011
Bendamustine for patients with indolent B cell lymphoid malignancies including chronic lymphocytic leukaemia	Vidal, , L, Gafter-Gvili, A, Gurion, R, Raanani, P, Dreyling, M; Shpilberg, O	2012
Beta-blockers for preventing aortic dissection in Marfan syndrome	Koo, , HK, Lawrence, KAK; Musini, VM	2017
Benzodiazepines for neuroleptic-induced acute akathisia	Resende Lima, , A, Soares-Weiser, K, Bacaltchuk, J; Barnes, TRE	1999
Beta2-agonists for acute cough or a clinical diagnosis of acute bronchitis	Becker, , LA, Hom, J, Villasis-Keever, M; van der Wouden, JC	2015
Betamimetics for inhibiting preterm labour	Neilson, , JP, West, HM; Dowswell, T	2014
Azathioprine or 6-mercaptopurine for maintenance of remission in Crohn's disease	Chande, , N, Patton, PH, Tsoulis, DJ, Thomas, BS; MacDonald, JK	2015
Baclofen for alcohol withdrawal	Liu, , J; Wang, LN	2017
Betahistine for symptoms of vertigo	Murdin, , L, Hussain, K; Schilder, AGM	2016
Auranofin versus placebo in rheumatoid arthritis	Suarez-Almazor, , ME, Spooner, C, Belseck, E; Shea, B	2000
Bicyclol for chronic hepatitis B	Wu, , T, Xie, L, Liu, GJ, Hao, B; Harrison, RA	2006
Beta-blockers and inhibitors of the renin-angiotensin aldosterone system for chronic heart failure with preserved ejection fraction	Martin, , N, Manoharan, K, Thomas, J, Davies, C; Lumbers, RT	2018
Back schools for non-specific low-back pain.	Heymans, , MW, van Tulder, MW, Esmail, R, Bombardier, C; Koes, BW	2004
Ayurvedic treatments for diabetes mellitus	Sridharan, , K, Mohan, R, Ramaratnam, S; Panneerselvam, D	2011
Back Schools for chronic non-specific low back pain	Parreira, , P, Heymans, MW, van Tulder, MW, Esmail, R, Koes, BW, Poquet, N, Lin, CWC; Maher, CG	2017
Autologous serum eye drops for dry eye	Pan, , Q, Angelina, A, Marrone, M, Stark, WJ; Akpek, EK	2017
Barriers and facilitators to the implementation of lay health worker programmes to improve access to maternal and child health: a qualitative evidence synthesis	Glenton, , C, Colvin, CJ, Carlsen, B, Swartz, A, Lewin, S, Noyes, J; Rashidian, A	2013
Behavioural and cognitive-behavioural group-based parenting programmes for early-onset conduct problems in children aged 3 to 12 years	Furlong, , M, McGilloway, S, Bywater, T, Hutchings, J, Smith, SM; Donnelly, M	2012
Behavioral interventions for improving dual-method contraceptive use	Lopez, , LM, Stockton, LL, Chen, M, Steiner, MJ; Gallo, MF	2014
Azithromycin versus penicillin G benzathine for early syphilis	Bai, , ZG, Wang, B, Yang, K, Tian, JH, Ma, B, Liu, Y, Jiang, L, Gai, QY, He, X; Li, Y	2012

Band ligation versus no intervention for primary prevention of upper gastrointestinal bleeding in adults with cirrhosis and oesophageal varices	Vadera, , S, Yong, CWK, Gluud, LL; Morgan, MY	2019
Autologous hematopoietic stem cell transplantation following high-dose chemotherapy for nonrhabdomyosarcoma soft tissue sarcomas	Peinemann, , F, Enk, H; Smith, LA	2017
Beclomethasone for asthma in children: effects on linear growth	Sharek, , PJ, Bergman, D; Ducharme, FM	1999
Beta-blockers for preventing stroke recurrence	De Lima, , LG, Saconato, H, Atallah, ÁN; da Silva, EMK	2014
Beclomethasone at different doses for chronic asthma	Adams, , NP, Bestall, JC; Jones, P	1999
Bed rest during pregnancy for preventing miscarriage	Aleman, , A, Althabe, F, Belizán, JM; Bergel, E	2005
Biofeedback and/or sphincter exercises for the treatment of faecal incontinence in adults	Norton, , C; Cody, JD	2012
Bicarbonate- versus lactate-buffered solutions for acute continuous haemodiafiltration or haemofiltration	Tian, , JH, Ma, B, Yang, K, Liu, Y, Tan, J; Liu, TX	2015
Behavioural and cognitive interventions with or without other treatments for the management of faecal incontinence in children	Brazzelli, , M, Griffiths, PV, Cody, JD; Tappin, D	2011
Beta-blockers for prevention and treatment of retinopathy of prematurity in preterm infants	Kaempfen, , S, Neumann, RP, Jost, K; Schulzke, SM	2018
Beta-blocker supplementation of standard drug treatment for schizophrenia	Shek, , E, Bardhan, S, Cheine, MV, Ahonen, J; Wahlbeck, K	2001
Bed rest for pressure ulcer healing in wheelchair users	Moore, , ZEH, van Etten, MT; Dumville, JC	2016
Beta-blockers for congestive heart failure in children	Alabed, , S, Sabouni, A, Al Dakhoul, S, Bdaiwi, Y; Frobel-Mercier, AK	2016
Benzodiazepines for restless legs syndrome	Carlos, , K, Prado, GF, Teixeira, CDM, Conti, C, de Oliveira, MM, Prado, LBF; Carvalho, LBC	2017
Autogenic drainage for airway clearance in cystic fibrosis	McCormack, , P, Burnham, P; Southern, KW	2017
Banding ligation versus beta-blockers for primary prevention in oesophageal varices in adults	Gluud, , LL; Krag, A	2012
Behavioral interventions for improving condom use for dual protection	Lopez, , LM, Otterness, C, Chen, M, Steiner, M; Gallo, MF	2013
Bioidentical hormones for women with vasomotor symptoms	Gaudard, , AMIS, Silva de Souza, S, Puga, MES, Marjoribanks, J, da Silva, EMK; Torloni, MR	2016
Beta-blockers for hypertension	Wiysonge, , CS, Bradley, HA, Volmink, J, Mayosi, BM; Opie, LH	2017
Belatacept for kidney transplant recipients	Masson, , P, Henderson, L, Chapman, JR, Craig, JC; Webster, AC	2014
Azithromycin for acute lower respiratory tract infections	Laopaiboon, , M, Panpanich, R; Swa Mya, K	2015
Azathioprine for primary biliary cirrhosis	Gong, , Y, Christensen, E; Gluud, C	2007
Barbiturates for acute traumatic brain injury	Roberts, , I; Sydenham, E	2012
Betamimetics for suspected impaired fetal growth	Say, , L, Gülmezoglu, AM; Hofmeyr, GJ	2001
Balneotherapy for osteoarthritis	Verhagen, , AP, Bierma-Zeinstra, SMA, Boers, M, Cardoso, JR, Lambeck, J, de Bie, R; de Vet, HCW	2007
Betahistine for Ménière's disease or syndrome	James, , A; Burton, MJ	2001
Benzo-pyrones for reducing and controlling lymphoedema of the limbs	Badger, , CMA, Preston, NJ, Seers, K; Mortimer, PS	2004
Azathioprine as an oral corticosteroid sparing agent for asthma	Dean, , TP, Dewey, A, Bara, A, Lasserson, TJ; Walters, EH	2003
Benzodiazepines for schizophrenia	Dold, , M, Li, C, Tardy, M, Khorsand, V, Gillies, D; Leucht, S	2012
Bed rest in hospital for suspected impaired fetal growth	Say, , L, Gülmezoglu, AM; Hofmeyr, GJ	1996
Balneotherapy for chronic venous insufficiency	de Moraes Silva, , MA, Nakano, LCU, Cisneros, LL; Miranda Jr, F	2019
Biologic or tofacitinib monotherapy for rheumatoid arthritis in people with traditional disease-modifying anti-rheumatic drug (DMARD) failure: a Cochrane Systematic Review and network meta-analysis (NMA)	Singh, , JA, Hossain, A, Tanjong Ghogomu, E, Mudano, AS, Tugwell, P; Wells, GA	2016
Biomarkers as point-of-care tests to guide prescription of antibiotics in patients with acute respiratory infections in primary care	Aabenhus, , R, Jensen, JUS, Jørgensen, KJ, Hróbjartsson, A; Bjerrum, L	2014
Beclomethasone versus budesonide for chronic asthma	Adams, , NP, Bestall, JC; Jones, P	2002
Bile acids for liver-transplanted patients	Poropat, , G, Giljaca, V, Stimac, D; Gluud, C	2010
Beta2-agonists for exercise-induced asthma	Bonini, , M, Di Mambro, C, Calderon, MA, Compalati, E, Schünemann, H, Durham, S; Canonica, GW	2013
Biomedical risk assessment as an aid for smoking cessation	Clair, , C, Mueller, Y, Livingstone-Banks, J, Burnand, B, Camain, JY, Cornuz, J, Rège-Walther, M, Selby, K; Bize, R	2019
Bariatric surgery for non-alcoholic steatohepatitis in obese patients	Chavez-Tapia, , NC, Tellez-Avila, FI, Barrientos-Gutierrez, T, Mendez-Sanchez, N, Lizardi-Cervera, J; Uribe, M	2010
Bed rest with or without hospitalisation for hypertension during pregnancy	Meher, , S, Abalos, E; Carroli, G	2005
Bed rest with and without hospitalisation in multiple pregnancy for improving perinatal outcomes	da Silva Lopes, , K, Takemoto, Y, Ota, E, Tanigaki, S; Mori, R	2017

Automated weaning and spontaneous breathing trial systems versus non-automated weaning strategies for discontinuation time in invasively ventilated postoperative adults	Burns, , KEA, Lellouche, F, Lessard, MR; Friedrich, JO	2014
Automated weaning and SBT systems versus non-automated weaning strategies for weaning time in invasively ventilated critically ill adults	Burns, , KEA, Lellouche, F, Nisenbaum, R, Lessard, MR; Friedrich, JO	2014
Biologics, colchicine, corticosteroids, immunosuppressants and interferon-alpha for Neuro-Behçet's Syndrome	Nava, , F, Ghilotti, F, Maggi, L, Hatemi, G, Del Bianco, A, Merlo, C, Filippini, G; Tramacere, I	2014
Azathioprine for treating rheumatoid arthritis	Suarez-Almazor, , ME, Spooner, C; Belseck, E	2000
Bile acids for non-alcoholic fatty liver disease and/or steatohepatitis	Orlando, , R, Azzalini, L, Orando, S; Lirussi, F	2007
Betahistine for tinnitus	Wegner, , I, Hall, DA, Smit, AL, McFerran, D; Stegeman, I	2018
Behavioral interventions to reduce the transmission of HIV infection among sex workers and their clients in low- and middle-income countries	Wariki, , WMV, Ota, E, Mori, R, Koyanagi, A, Hori, N; Shibuya, K	2012
Baclofen for alcohol use disorder	Minozzi, , S, Saulle, R; Rösner, S	2018
Barriers and facilitators to the implementation of doctor-nurse substitution strategies in primary care: a qualitative evidence synthesis	Karimi-Shahanjarini, , A, Shakibazadeh, E, Rashidian, A, Hajimiri, K, Glenton, C, Noyes, J, Lewin, S, Laurant, M; Colvin, CJ	2019
Autologous platelet-rich plasma for treating chronic wounds	Martinez-Zapata, , MJ, Martí-Carvajal, AJ, Solà, I, Expósito, JA, Bolíbar, I, Rodríguez, L, Garcia, J; Zaror, C	2016
Automated mandatory bolus versus basal infusion for maintenance of epidural analgesia in labour	Sng, , BL, Zeng, Y, de Souza, NNA, Leong, WL, Oh, TT, Siddiqui, FJ, Assam, PN, Han, NLR, Chan, ESY; Sia, AT	2018
Beta2-adrenoceptor agonists for dysmenorrhoea	Fedorowicz, , Z, Nasser, M, Jagannath, VA, Beaman, JH, Ejaz, K; van Zuuren, EJ	2012
Azithromycin for treating uncomplicated malaria	van Eijk, , AM; Terlouw, DJ	2011
Biochemical tests of placental function for assessment in pregnancy	Neilson, JP	2012
Bicarbonate versus lactate solutions for acute peritoneal dialysis	Bai, , ZG, Yang, K, Tian, JH, Ma, B, Liu, Y, Jiang, L, Tan, J, Liu, TX; Chi, I	2014
Automated telephone communication systems for preventive healthcare and management of long-term conditions	Posadzki, , P, Mastellos, N, Ryan, R, Gunn, LH, Felix, LM, Pappas, Y, Gagnon, MP, Julious, SA, Xiang, L, Oldenburg, B; Car, J	2016
Biocompatible hemodialysis membranes for acute renal failure	Alonso, , A, Lau, J; Jaber, BL	2008
Bile acids for primary sclerosing cholangitis	Poropat, , G, Giljaca, V, Stimac, D; Gluud, C	2011
Auricular acupuncture for cocaine dependence	Gates, , S, Smith, LA; Foxcroft, D	2006
Azathioprine and 6-mercaptopurine for maintenance of surgically-induced remission in Crohn's disease	Gjuladin-Hellon, , T, Iheozor-Ejiofor, Z, Gordon, M; Akobeng, AK	2019
Azathioprine or 6-mercaptopurine for induction of remission in Crohn's disease	Chande, , N, Townsend, CM, Parker, CE; MacDonald, JK	2016
Benzodiazepines versus placebo for panic disorder in adults	Breilmann, , J, Girlanda, F, Guaiana, G, Barbui, C, Cipriani, A, Castellazzi, M, Bighelli, I, Davies, SJC, Furukawa, TA; Koesters, M	2019
Fluoride supplementation (with tablets, drops, lozenges or chewing gum) in pregnant women for preventing dental caries in the primary teeth of their children	Takahashi, , R, Ota, E, Hoshi, K, Naito, T, Toyoshima, Y, Yuasa, H, Mori, R; Nango, E	2017
First trimester serum tests for Down's syndrome screening	Alldred, , SK, Takwoingi, Y, Guo, B, Pennant, M, Deeks, JJ, Neilson, JP; Alfirevic, Z	2015
Face-to-face interventions for promoting physical activity	Richards, , J, Hillsdon, M, Thorogood, M; Foster, C	2013
Face-to-face versus remote and web 2.0 interventions for promoting physical activity	Richards, , J, Thorogood, M, Hillsdon, M; Foster, C	2013
Fluoride for treating postmenopausal osteoporosis	Haguenauer, , D, Shea, B, Tugwell, P, Wells, GA; Welch, V	2000
Exercises for mechanical neck disorders	Gross, , A, Kay, TM, Paquin, JP, Blanchette, S, Lalonde, P, Christie, T, Dupont, G, Graham, N, Burnie, SJ, Gelley, G, Goldsmith, CH, Forget, M, Hoving, JL, Brønfort, G; Santaguida, PL	2015
Fluticasone at different doses for chronic asthma in adults and children	Adams, , NP, Bestall, JC, Jones, P, Lasserson, TJ, Griffiths, B; Cates, CJ	2008
First rank symptoms for schizophrenia	Soares-Weiser, , K, Maayan, N, Bergman, H, Davenport, C, Kirkham, AJ, Grabowski, S; Adams, CE	2015
Fluid and pharmacological agents for adhesion prevention after gynaecological surgery	Ahmad, , G, Mackie, FL, Iles, DA, O'Flynn, H, Dias, S, Metwally, M; Watson, A	2014
Exercise-based cardiac rehabilitation for adults after heart valve surgery	Sibilitz, , KL, Berg, SK, Tang, LH, Risom, SS, Gluud, C, Lindschou, J, Kober, L, Hassager, C, Taylor, RS; Zwisler, AD	2016
Enteral lactoferrin for the treatment of sepsis and necrotizing enterocolitis in neonates	Pammi, , M; Abrams, SA	2019
Excisional surgery versus ablative surgery for ovarian endometriomata	Hart, , RJ, Hickey, M, Maouris, P; Buckett, W	2008
Enteral iron supplementation in preterm and low birth weight infants	Mills, , RJ; Davies, MW	2012
Erythropoiesis-stimulating agents for anaemia in chronic heart failure patients	Ngo, , K, Kotecha, D, Walters, JAE, Manzano, L, Palazzuoli, A, van Veldhuisen, DJ; Flather, M	2010

Exercise interventions for upper-limb dysfunction due to breast cancer treatment	McNeely, , ML, Campbell, K, Ospina, M, Rowe, BH, Dabbs, K, Klassen, TP, Mackey, J; Courneya, K	2010
Extracorporeal photopheresis versus standard treatment for acute graft-versus-host disease after haematopoietic stem cell transplantation in paediatric patients	Weitz, , M, Strahm, B, Meerpohl, JJ, Schmidt, M; Bassler, D	2015
Eradication therapy for Burkholderia cepacia complex in people with cystic fibrosis	Regan, , KH; Bhatt, J	2019
Enzyme replacement therapy with idursulfase for mucopolysaccharidosis type II (Hunter syndrome)	da Silva, , EMK, Strufaldi, MWL, Andriolo, RB; Silva, LA	2016
Exercise for the management of cancer-related fatigue in adults	Cramp, , F; Byron-Daniel, J	2012
Fluoropyrimidine-HAI (hepatic arterial infusion) versus systemic chemotherapy (SCT) for unresectable liver metastases from colorectal cancer	Mocellin, , S, Pasquali, S; Nitti, D	2009
Finasteride for benign prostatic hyperplasia	Tacklind, , J, Fink, HA, MacDonald, R, Rutks, I; Wilt, TJ	2010
Exercise for diabetic pregnant women	Ceysens, , G, Rouiller, D; Boulvain, M	2006
Feedback or biofeedback to augment pelvic floor muscle training for urinary incontinence in women	Herderschee, , R, Hay-Smith, EJC, Herbison, GP, Roovers, JP; Heineman, MJ	2011
Estrogen for schizophrenia	Chua, , WLLC, Izquierdo de Santiago, A, Kulkarni, J; Mortimer, A	2005
Family-based programmes for preventing smoking by children and adolescents	Thomas, , RE, Baker, PRA, Thomas, BC; Lorenzetti, DL	2015
Enteral nutrition formulations for acute pancreatitis	Poropat, , G, Giljaca, V, Hauser, G; Štimac, D	2015
Exercise-based cardiac rehabilitation for people with implantable ventricular assist devices	Yamamoto, , S, Hotta, K, Ota, E, Matsunaga, A; Mori, R	2018
Exercise for intermittent claudication	Lane, , R, Harwood, A, Watson, L; Leng, GC	2017
Flupenthixol versus low-potency first-generation antipsychotic drugs for schizophrenia	Tardy, , M, Dold, M, Engel, RR; Leucht, S	2014
Excimer laser refractive surgery versus phakic intraocular lenses for the correction of moderate to high myopia	Barsam, , A; Allan, BDS	2014
Exercise training for adults with chronic kidney disease	Heiwe, , S; Jacobson, SH	2011
Fluoride mouthrinses for preventing dental caries in children and adolescents	Marinho, , VCC, Chong, LY, Worthington, HV; Walsh, T	2016
Flumazenil versus placebo or no intervention for people with cirrhosis and hepatic encephalopathy	Goh, , ET, Andersen, ML, Morgan, MY; Gluud, LL	2017
Exercise therapy for fatigue in multiple sclerosis	Heine, , M, van de Port, I, Rietberg, MB, van Wegen, EEH; Kwakkel, G	2015
Exercise interventions on health-related quality of life for cancer survivors	Mishra, , SI, Scherer, RW, Geigle, PM, Berlanstein, DR, Topaloglu, O, Gotay, CC; Snyder, C	2012
Erythromycin for the prevention and treatment of feeding intolerance in preterm infants	Ng, , E; Shah, VS	2008
Flow-regulated versus differential pressure-regulated shunt valves for adult patients with normal pressure hydrocephalus	Ziebell, , M, Wetterslev, J, Tisell, M, Gluud, C; Juhler, M	2013
Exenterative surgery for recurrent gynaecological malignancies	Ang, , C, Bryant, A, Barton, DPJ, Pomel, C; Naik, R	2014
Etidronate for the primary and secondary prevention of osteoporotic fractures in postmenopausal women	Wells, , GA, Cranney, A, Peterson, J, Boucher, M, Shea, B, Welch, V, Coyle, D; Tugwell, P	2008
Enzyme replacement and substrate reduction therapy for Gaucher disease	Shemesh, , E, Deroma, L, Bembi, B, Deegan, P, Hollak, C, Weinreb, NJ; Cox, TM	2015
Exhaled nitric oxide levels to guide treatment for adults with asthma	Petsky, , HL, Kew, KM, Turner, C; Chang, AB	2016
Exercise for osteoarthritis of the hip	Fransen, , M, McConnell, S, Hernandez-Molina, G; Reichenbach, S	2014
Fluvastatin for lowering lipids	Adams, , SP, Sekhon, SS, Tsang, M; Wright, JM	2018
Eversion versus conventional carotid endarterectomy for preventing stroke	Cao, , P, De Rango, P, Zannetti, S, Giordano, G, Ricci, S; Celani, MG	2000
Environmental interventions to reduce the consumption of sugar-sweetened beverages and their effects on health	von Philipsborn, , P, Stratil, JM, Burns, J, Busert, LK, Pfadenhauer, LM, Polus, S, Holzapfel, C, Hauner, H; Rehfuess, E	2019
Fluvoxamine versus other anti-depressive agents for depression	Omori, , IM, Watanabe, N, Nakagawa, A, Cipriani, A, Barbui, C, McGuire, H, Churchill, R; Furukawa, TA	2010
Feverfew for preventing migraine	Wider, , B, Pittler, MH; Ernst, E	2015
Exercise versus no exercise for the occurrence, severity and duration of acute respiratory infections	Grande, , AJ, Keogh, J, Hoffmann, TC, Beller, EM; Del Mar, CB	2015
Enteral versus parenteral nutrition for acute pancreatitis	Al-Omran, , M, AlBalawi, ZH, Tashkandi, MF; Al-Ansary, LA	2010
Epidural local anaesthetics versus opioid-based analgesic regimens for postoperative gastrointestinal paralysis, vomiting and pain after abdominal surgery	Guay, , J, Nishimori, M; Kopp, S	2016
Flupenthixol versus placebo for schizophrenia	Shen, , X, Xia, J; Adams, CE	2012
Epinephrine injection versus epinephrine injection and a second endoscopic method in high-risk bleeding ulcers	Vergara, , M, Bennett, C, Calvet, X; Gisbert, JP	2014

Exercise training undertaken by people within 12 months of lung resection for non-small cell lung cancer	Cavalheri, , V, Burtin, C, Formico, VR, Nonoyama, ML, Jenkins, S, Spruit, MA; Hill, K	2019
Exercise for people with high cardiovascular risk	Seron, , P, Lanas, F, Pardo Hernandez, H; Bonfill Cosp, X	2014
First aid glucose administration routes for symptomatic hypoglycaemia	De Buck, , E, Borra, V, Carlson, JN, Zideman, DA, Singletary, EM; Djärv, T	2019
Ethical case interventions for adult patients	Schildmann, , J, Nadolny, S, Haltaufderheide, J, Gysels, M, Vollmann, J; Bausewein, C	2019
Exercise for pregnant women with pre-existing diabetes for improving maternal and fetal outcomes	Brown, , J, Ceysens, G; Boulvain, M	2017
Exercise for preventing falls in older people living in the community	Sherrington, , C, Fairhall, NJ, Wallbank, GK, Tiedemann, A, Michaleff, ZA, Howard, K, Clemson, L, Hopewell, S; Lamb, SE	2019
Epinephrine for transient tachypnea of the newborn	Moresco, , L, Calevo, MG, Baldi, F, Cohen, A; Bruschettini, M	2016
Fast track surgery versus conventional recovery strategies for colorectal surgery	Spanjersberg, , WR, Reurings, J, Keus, F; van Laarhoven, CJHM	2011
Epidural therapy for the treatment of severe pre-eclampsia in non labouring women	Ray, , A; Ray, S	2017
Fluid therapy for acute bacterial meningitis	Maconochie, , IK; Bhaumik, S	2016
Exercise for preventing and treating osteoporosis in postmenopausal women	Howe, , TE, Shea, B, Dawson, LJ, Downie, F, Murray, A, Ross, C, Harbour, RT, Caldwell, LM; Creed, G	2011
Enzyme replacement therapy for Anderson-Fabry disease	El Dib, , R, Gomaa, H, Carvalho, RP, Camargo, SE, Bazan, R, Barretti, P; Barreto, FC	2016
Erythropoiesis-stimulating agents for anemia in rheumatoid arthritis	Martí-Carvajal, , AJ, Agreda-Pérez, LH; Solà, I	2013
First-line beta-blockers versus other antihypertensive medications for chronic type B aortic dissection	Chan, , KK, Lai, P; Wright, JM	2014
Etanercept for the treatment of rheumatoid arthritis	Lethaby, , A, Lopez-Olivo, MA, Maxwell, LJ, Burls, A, Tugwell, P; Wells, GA	2013
Exercise therapy for treatment of non-specific low back pain	Hayden, , J, van Tulder, MW, Malmivaara, A; Koes, BW	2005
Family therapy for asthma in children	Yorke, , J; Shuldham, C	2005
Environmental sanitary interventions for preventing active trachoma	Rabiu, , M, Alhassan, MB, Ejere, HOD; Evans, JR	2012
Exercise therapy in juvenile idiopathic arthritis	Takken, , T, Van Brussel, M, Engelbert, RH, van der Net, JJ, Kuis, W; Helders, PPJM	2008
Face-down positioning or posturing after macular hole surgery	Solebo, , AL, Lange, CAK, Bunce, C; Bainbridge, JW	2011
Fibrinogen depleting agents for acute ischaemic stroke	Hao, , Z, Liu, M, Counsell, C, Wardlaw, JM, Lin, S; Zhao, X	2012
Fluorides for the prevention of early tooth decay (demineralised white lesions) during fixed brace treatment	Benson, , PE, Parkin, N, Dyer, F, Millett, DT, Furness, S; Germain, P	2013
First and second trimester serum tests with and without first trimester ultrasound tests for Down's syndrome screening	Alldred, , SK, Takwoingi, Y, Guo, B, Pennant, M, Deeks, JJ, Neilson, JP; Alfirevic, Z	2017
Folate supplementation in people with sickle cell disease	Dixit, , R, Nettem, S, Madan, SS, Soe, HHK, Abas, ABL, Vance, LD; Stover, PJ	2018
Family therapy for autism spectrum disorders	Spain, , D, Sin, J, Paliokosta, E, Furuta, M, Prunty, JE, Chalder, T, Murphy, DG; Happé, FG	2017
Exercise for rheumatoid arthritis of the hand	Williams, , MA, Srikesavan, C, Heine, PJ, Bruce, J, Brosseau, L, Hoxey-Thomas, N; Lamb, SE	2018
Extubation from low-rate intermittent positive airway pressure versus extubation after a trial of endotracheal continuous positive airway pressure in intubated preterm infants	Davis, , PG; Henderson-Smart, DJ	2001
Fluoride varnishes for preventing dental caries in children and adolescents	Marinho, , VCC, Worthington, HV, Walsh, T; Clarkson, JE	2013
Evening versus morning dosing regimen drug therapy for hypertension	Zhao, , P, Xu, P, Wan, C; Wang, Z	2011
E-learning for health professionals	Vaona, , A, Banzi, R, Kwag, KH, Rigon, G, Cereda, D, Pecoraro, V, Tramacere, I; Moja, L	2018
Fluoroquinolones for treating typhoid and paratyphoid fever (enteric fever)	Effa, , EE, Lassi, ZS, Critchley, JA, Garner, P, Sinclair, D, Olliaro, PL; Bhutta, ZA	2011
Exercise-based cardiac rehabilitation for coronary heart disease	Anderson, , L, Thompson, DR, Oldridge, N, Zwisler, AD, Rees, K, Martin, N; Taylor, RS	2016
Etrolizumab for induction of remission in ulcerative colitis	Rosenfeld, , G, Parker, CE, MacDonald, JK; Bressler, B	2015
Fibrinolytic agents for peripheral arterial occlusion	Robertson, , I, Kessel, DO; Berridge, DC	2013
Fluids and diuretics for acute ureteric colic	Worster, , AS; Bhanich Supapol, W	2012
Fetal and umbilical Doppler ultrasound in high-risk pregnancies	Alfirevic, , Z, Stampalija, T; Dowswell, T	2017
Fentanyl for neuropathic pain in adults	Derry, , S, Stannard, C, Cole, P, Wiffen, PJ, Knaggs, R, Aldington, D; Moore, RA	2016
Factor Xa inhibitors versus vitamin K antagonists for preventing cerebral or systemic embolism in patients with atrial fibrillation	Bruins Slot, , KMH; Berge, E	2018
Fluoxetine versus other types of pharmacotherapy for depression	Magni, , LR, Purgato, M, Gastaldon, C, Papola, D, Furukawa, TA, Cipriani, A; Barbui, C	2013
Exercise programs for people with dementia	Forbes, , D, Forbes, SC, Blake, CM, Thiessen, EJ; Forbes, S	2015

Fluoroquinolones for treating tuberculosis (presumed drug-sensitive)	Ziganshina, , LE, Titarenko, AF; Davies, GR	2013
Fluid supplementation for neonatal unconjugated hyperbilirubinaemia	Lai, , NM, Ahmad Kamar, A, Choo, YM, Kong, JY; Ngim, CF	2017
Exercise prior to influenza vaccination for limiting influenza incidence and its related complications in adults	Grande, , AJ, Reid, H, Thomas, EE, Nunan, D; Foster, C	2016
Eplerenone for hypertension	Tam, , TSC, Wu, MHY, Masson, SC, Tsang, MP, Stabler, SN, Kinkade, A, Tung, A; Tejani, AM	2017
Exercise interventions for smoking cessation	Ussher, , MH, Taylor, AH; Faulkner, GEJ	2014
Exercise for improving balance in older people	Howe, , TE, Rochester, L, Neil, F, Skelton, DA; Ballinger, C	2011
Fixed dose subcutaneous low molecular weight heparins versus adjusted dose unfractionated heparin for the initial treatment of venous thromboembolism	Robertson, , L; Jones, LE	2017
Exercise-based cardiac rehabilitation for adults with stable angina	Long, , L, Anderson, L, Dewhirst, AM, He, J, Bridges, C, Gandhi, M; Taylor, RS	2018
Epidural pain relief versus systemic opioid-based pain relief for abdominal aortic surgery	Guay, , J; Kopp, S	2016
Erythropoietin as an adjuvant treatment with (chemo) radiation therapy for head and neck cancer	Lambin, , P, Ramaekers, BLT, van Mastrigt, GAPG, Van den Ende, P, de Jong, J, De Ruysscher, DKM; Pijls-Johannesma, M	2009
Fibrates for primary prevention of cardiovascular disease events	Jakob, , T, Nordmann, AJ, Schandelmaier, S, Ferreira-González, I; Briel, M	2016
Extracorporeal shock wave lithotripsy (ESWL) versus ureteroscopic management for ureteric calculi	Aboumarzouk, , OM, Kata, SG, Keeley, FX, McClinton, S; Nabi, G	2012
Fluticasone versus 'extrafine' HFA-beclomethasone dipropionate for chronic asthma in adults and children	Lasserson, , TJ, Cates, CJ, Lasserson, EH; White, J	2006
Family and carer smoking control programmes for reducing children's exposure to environmental tobacco smoke	Behbod, , B, Sharma, M, Baxi, R, Roseby, R; Webster, P	2018
Environmental and behavioural interventions for reducing physical activity limitation in community-dwelling visually impaired older people	Skelton, , DA, Howe, TE, Ballinger, C, Neil, F, Palmer, S; Gray, L	2013
First-line treatment of advanced epidermal growth factor receptor (EGFR) mutation positive non-squamous non-small cell lung cancer	Greenhalgh, , J, Dwan, K, Boland, A, Bates, V, Vecchio, F, Dundar, Y, Jain, P; Green, JA	2016
Evaluation of follow-up strategies for patients with epithelial ovarian cancer following completion of primary treatment	Clarke, , T, Galaal, K, Bryant, A; Naik, R	2014
Exercise for hand osteoarthritis	Østerås, , N, Kjeken, I, Smedslund, G, Moe, RH, Slatkowsky-Christensen, B, Uhlig, T; Hagen, KB	2017
External counterpulsation for acute ischaemic stroke	Lin, , S, Liu, M, Wu, B, Hao, Z, Yang, J; Tao, W	2012
Exercise-based cardiac rehabilitation in heart transplant recipients	Anderson, , L, Nguyen, TT, Dall, CH, Burgess, L, Bridges, C; Taylor, RS	2017
Exercises for adolescent idiopathic scoliosis	Romano, , M, Minozzi, S, Bettany-Saltikov, J, Zaina, F, Chockalingam, N, Kotwicki, T, Maier-Hennes, A; Negrini, S	2012
Eszopiclone for insomnia	Rösner, , S, Englbrecht, C, Wehrle, R, Hajak, G; Soyka, M	2018
Flexible sigmoidoscopy versus faecal occult blood testing for colorectal cancer screening in asymptomatic individuals	Holme, , Ø, Bretthauer, M, Fretheim, A, Odgaard-Jensen, J; Hoff, G	2013
Exercise for improving outcomes after osteoporotic vertebral fracture	Gibbs, , JC, MacIntyre, NJ, Ponzano, M, Templeton, JA, Thabane, L, Papaioannou, A; Giangregorio, LM	2019
Extended versus standard lymph node dissection for urothelial carcinoma of the bladder in patients undergoing radical cystectomy	Hwang, , EC, Sathianathen, NJ, Imamura, M, Kuntz, GM, Risk, MC; Dahm, P	2019
Fetal biometry for guiding the medical management of women with gestational diabetes mellitus for improving maternal and perinatal health	Rao, , U, de Vries, B, Ross, GP; Gordon, A	2019
Flavonoids for treating venous leg ulcers	Scallon, , C, Bell-Syer, SEM; Aziz, Z	2013
Enzyme replacement therapy with laronidase (Aldurazyme®) for treating mucopolysaccharidosis type I	Jameson, , E, Jones, S; Remmington, T	2019
Exercise interventions for shoulder dysfunction in patients treated for head and neck cancer	Carvalho, , APV, Vital, FMR; Soares, BGO	2012
Extended-field radiotherapy for locally advanced cervical cancer	Thamronganantasakul, , K, Supakalin, N, Kietpeerakool, C, Pattanittum, P; Lumbiganon, P	2018
First-line tandem high-dose chemotherapy and autologous stem cell transplantation versus single high-dose chemotherapy and autologous stem cell transplantation in multiple myeloma, a systematic review of controlled studies	Naumann-Winter, , F, Greb, A, Borchmann, P, Bohlius, J, Engert, A; Schnell, R	2012
Exercise-based cardiac rehabilitation for adult patients with an implantable cardioverter defibrillator	Nielsen, , KM, Zwisler, AD, Taylor, RS, Svendsen, JH, Lindschou, J, Anderson, L, Jakobsen, JC; Berg, SK	2019
Enteral lactoferrin supplementation for prevention of sepsis and necrotizing enterocolitis in preterm infants	Pammi, , M; Suresh, G	2017
Exercise interventions on health-related quality of life for people with cancer during active treatment	Mishra, , SI, Scherer, RW, Snyder, C, Geigle, PM, Berlanstein, DR; Topaloglu, O	2012
Exercise training for advanced lung cancer	Peddle-McIntyre, , CJ, Singh, F, Thomas, R, Newton, RU, Galvão, DA; Cavalheri, V	2019
Excitatory amino acid antagonists for acute stroke	Muir, , KW; Lees, KR	2003
Ezetimibe for the prevention of cardiovascular disease and all-cause mortality events	Zhan, , S, Tang, M, Liu, F, Xia, P, Shu, M; Wu, X	2018
Fluticasone versus beclomethasone or budesonide for chronic asthma in adults and children	Adams, , NP, Lasserson, TJ, Cates, CJ; Jones, P	2007

Fluticasone versus placebo for chronic asthma in adults and children	Adams, , NP, Bestall, JC, Lasserson, TJ, Jones, P; Cates, CJ	2008
Febuxostat for treating chronic gout	Tayar, , JH, Lopez-Olivo, MA; Suarez-Almazor, ME	2012
Family-centred care for hospitalised children aged 0-12 years	Shields, , L, Zhou, H, Pratt, J, Taylor, M, Hunter, J; Pascoe, E	2012
Fluoridated milk for preventing dental caries	Yeung, , CA, Chong, LY; Glenny, AM	2015
Femoral nerve blocks for acute postoperative pain after knee replacement surgery	Chan, , EY, Fransen, M, Parker, DA, Assam, PN; Chua, N	2014
Exercise for haemophilia	Strike, , K, Mulder, K; Michael, R	2016
Exercise-based rehabilitation programmes for pulmonary hypertension	Morris, , NR, Kermeen, FD; Holland, AE	2017
Family intervention (brief) for schizophrenia	Okpokoro, , U, Adams, CE; Sampson, S	2014
Fibrin glue versus sutures for conjunctival autografting in primary pterygium surgery	Romano, , V, Cruciani, M, Conti, L; Fontana, L	2016
Exercise for cancer cachexia in adults	Grande, , AJ, Silva, V, Riera, R, Medeiros, A, Vitoriano, SGP, Peccin, MS; Maddocks, M	2014
Ergonomic positioning or equipment for treating carpal tunnel syndrome	O'Connor, , D, Page, MJ, Marshall, SC; Massy-Westropp, N	2012
Epidural analgesia for adults undergoing cardiac surgery with or without cardiopulmonary bypass	Guay, , J; Kopp, S	2019
Exercise-based cardiac rehabilitation for adults with heart failure	Long, , L, Mordi, IR, Bridges, C, Sagar, VA, Davies, EJ, Coats, AJS, Dalal, H, Rees, K, Singh, SJ; Taylor, RS	2019
Enzyme replacement therapy with galsulfase for mucopolysaccharidosis type VI	Brunelli, , MJ, Atallah, ÁN; da Silva, EMK	2016
Ergonomic interventions for preventing work-related musculoskeletal disorders of the upper limb and neck among office workers	Hoe, , VCW, Urquhart, DM, Kelsall, HL, Zamri, EN; Sim, MR	2018
Exercise for pregnant women for preventing gestational diabetes mellitus	Han, , S, Middleton, P; Crowther, CA	2012
Ephedrine for myasthenia gravis, neonatal myasthenia and the congenital myasthenic syndromes	Vrinten, , C, van der Zwaag, AM, Weinreich, SS, Scholten, RJPM; Verschuuren, JJGM	2014
Exercise and mobilisation interventions for carpal tunnel syndrome	Page, , MJ, O'Connor, D, Pitt, V; Massy-Westropp, N	2012
Environmental and behavioural modifications for improving food and fluid intake in people with dementia	Herke, , M, Fink, A, Langer, G, Wustmann, T, Watzke, S, Hanff, AM; Burckhardt, M	2018
Ergonomic interventions for preventing musculoskeletal disorders in dental care practitioners	Mulimani, , P, Hoe, VCW, Hayes, MJ, Idiculla, JJ, Abas, ABL; Karanth, L	2018
E-Health interventions for anxiety and depression in children and adolescents with long-term physical conditions	Thabrew, , H, Stasiak, K, Hetrick, SE, Wong, S, Huss, JH; Merry, SN	2018
Extracranial-intracranial arterial bypass surgery for occlusive carotid artery disease	Fluri, , F, Engelter, S; Lyrer, P	2010
Folic acid for fragile X syndrome	Rueda, , JR, Ballesteros, J, Guillen, V, Tejada, MI; Solà, I	2011
Enteral tube feeding for amyotrophic lateral sclerosis/motor neuron disease	Katzberg, , HD; Benatar, M	2011
Exercise for people with peripheral neuropathy	White, , CM, Pritchard, J; Turner-Stokes, L	2004
Foam dressings for venous leg ulcers	O'Meara, , S; Martyn-St James, M	2013
Exercise for pregnant women with gestational diabetes for improving maternal and fetal outcomes	Brown, , J, Ceysens, G; Boulvain, M	2017
Fibrin glue instillation under skin flaps to prevent seroma-related morbidity following breast and axillary surgery	Sajid, , MS, Hutson, KH, Rapisarda, IF; Bonomi, R	2013
Epidural analgesia versus patient-controlled intravenous analgesia for pain following intra-abdominal surgery in adults	Salicath, , JH, Yeoh, ECY; Bennett, MH	2018
Fermented milk for hypertension	Usinger, , L, Reimer, C; Ibsen, H	2012
Flow-cycled versus time-cycled synchronized ventilation for neonates	Schulzke, , SM, Pillow, J, Ewald, B; Patole, SK	2010
Factor Xa inhibitors for acute coronary syndromes	Brito, , V, Ciapponi, A; Kwong, J	2011
Fish oil for kidney transplant recipients	Lim, , AKH, Manley, KJ, Roberts, MA; Fraenkel, MB	2016
Fixed-dose combinations of drugs versus single-drug formulations for treating pulmonary tuberculosis	Gallardo, , CR, Rigau Comas, D, Valderrama Rodríguez, A, Roqué i Figuls, M, Parker, LA, Caylà, J; Bonfill Cosp, X	2016
Flexible working conditions and their effects on employee health and wellbeing	Joyce, , K, Pabayo, R, Critchley, JA; Bambra, C	2010
Evaluation of the efficacy and safety of adjuvant treatment to levodopa therapy in Parkinson´s disease patients with motor complications	Stowe, , R, Ives, N, Clarke, CE, Deane, K, van Hilten, , Wheatley, K, Gray, R, Handley, K; Furmston, A	2010
Exercise therapy for multiple sclerosis	Rietberg, , MB, Brooks, D, Uitdehaag, BMJ; Kwakkel, G	2005
Fixed-dose combination therapy for the prevention of atherosclerotic cardiovascular diseases	Bahiru, , E, de Cates, AN, Farr, MRB, Jarvis, MC, Palla, M, Rees, K, Ebrahim, S; Huffman, MD	2017
Erythropoietin or Darbepoetin for patients with cancer - meta-analysis based on individual patient data	Bohlius, , J, Schmidlin, K, Brillant, C, Schwarzer, G, Trelle, S, Seidenfeld, J, Zwahlen, M, Clarke, MJ, Weingart, O, Kluge, S, Piper, M, Napoli, M, Rades, D, Steensma, D, Djulbegovic, B, Fey, MF, Ray-Coquard, I, Moebus, V, Thomas, G, Untch, M, Schumacher, M, Egger, M; Engert, A	2009

Fecal transplantation for treatment of inflammatory bowel disease	Imdad, , A, Nicholson, MR, Tanner-Smith, EE, Zackular, JP, Gomez-Duarte, OG, Beaulieu, DB; Acra, S	2018
Exercise-based cardiac rehabilitation for adults with atrial fibrillation	Risom, , SS, Zwisler, AD, Johansen, PP, Sibilitz, KL, Lindschou, J, Gluud, C, Taylor, RS, Svendsen, JH; Berg, SK	2017
Extended peginterferon plus ribavirin treatment for 72 weeks versus standard peginterferon plus ribavirin treatment for 48 weeks in chronic hepatitis C genotype 1 infected slow-responder adult patients	Katz, , LH, Goldvaser, H, Gafter-Gvili, A; Tur-Kaspa, R	2012
Ethamsylate for the prevention of morbidity and mortality in preterm or very low birth weight infants	Hunt, , R; Hey, E	2010
First-line allogeneic hematopoietic stem cell transplantation of HLA-matched sibling donors compared with first-line ciclosporin and/or antithymocyte or antilymphocyte globulin for acquired severe aplastic anemia	Peinemann, , F, Bartel, C; Grouven, U	2013
Enteral versus parenteral nutrition and enteral versus a combination of enteral and parenteral nutrition for adults in the intensive care unit	Lewis, , SR, Schofield-Robinson, OJ, Alderson, P; Smith, AF	2018
Face-to-face interventions for informing or educating parents about early childhood vaccination	Kaufman, , J, Ryan, R, Walsh, L, Horey, D, Leask, J, Robinson, P; Hill, S	2018
Fingolimod for relapsing-remitting multiple sclerosis	La Mantia, , L, Tramacere, I, Firwana, B, Pacchetti, I, Palumbo, R; Filippini, G	2016
Exposure to the smell and taste of milk to accelerate feeding in preterm infants	Muelbert, , M, Lin, L, Bloomfield, FH; Harding, JE	2019
Folic acid supplementation during pregnancy for maternal health and pregnancy outcomes	Lassi, , ZS, Salam, RA, Haider, BA; Bhutta, ZA	2013
Families and Schools Together (FAST) for improving outcomes for children and their families	Valentine, , JC, Leach, SM, Fowler, AP, Stojda, DK; Macdonald, G	2019
Enteral tube feeding for cystic fibrosis	Shimmin, , D, Lowdon, J; Remmington, T	2019
Family-based prevention programmes for alcohol use in young people	Gilligan, , C, Wolfenden, L, Foxcroft, DR, Williams, AJ, Kingsland, M, Hodder, RK, Stockings, E, McFadyen, TR, Tindall, J, Sherker, S, Rae, J; Wiggers, J	2019
Exercise for reducing falls in people living with and beyond cancer	Williams, , AD, Bird, ML, Hardcastle, SGK, Kirschbaum, M, Ogden, KJ; Walters, JAE	2018
Fluoride toothpastes of different concentrations for preventing dental caries	Walsh, , T, Worthington, HV, Glenny, AM, Marinho, VCC; Jeroncic, A	2019
Exfoliative cytology for diagnosing basal cell carcinoma and other skin cancers in adults	Ferrante di Ruffano, , L, Dinnes, J, Chuchu, N, Bayliss, SE, Takwoingi, Y, Davenport, C, Matin, RN, O'Sullivan, C, Roskell, D, Deeks, JJ; Williams, HC	2018
Enteral nutrition for maintenance of remission in Crohn's disease	Akobeng, , AK, Zhang, D, Gordon, M; MacDonald, JK	2018
First-line drugs for hypertension	Wright, , JM, Musini, VM; Gill, R	2018
First-line drugs inhibiting the renin angiotensin system versus other first-line antihypertensive drug classes for hypertension	Chen, , YJ, Li, LJ, Tang, WL, Song, JY, Qiu, R, Li, Q, Xue, H; Wright, JM	2018
Hematopoietic stem cell transplantation for people with sickle cell disease	Oringanje, , C, Nemecek, E; Oniyangi, O	2016
Helminth therapy (worms) for induction of remission in inflammatory bowel disease	Garg, , SK, Croft, AM; Bager, P	2014
Glycerol for acute stroke	Righetti, , E, Celani, MG, Cantisani, TA, Sterzi, R, Boysen, G; Ricci, S	2004
Garlic for peripheral arterial occlusive disease	Jepson, , RG, Kleijnen, J; Leng, GC	2013
Granulopoiesis-stimulating factors to prevent adverse effects in the treatment of malignant lymphoma	Bohlius, , J, Herbst, C, Reiser, M, Schwarzer, G; Engert, A	2008
Heparin for the prevention of intraventricular haemorrhage in preterm infants	Bruschettini, , M, Romantsik, O, Zappettini, S, Banzi, R, Ramenghi, LA; Calevo, MG	2016
Hepatitis B vaccination during pregnancy for preventing infant infection	Sangkomkamhang, , US, Lumbiganon, P; Laopaiboon, M	2014
Heparin for the treatment of thrombosis in neonates	Romantsik, , O, Bruschettini, M, Zappettini, S, Ramenghi, LA; Calevo, MG	2016
Ginkgo biloba extract for age-related macular degeneration	Evans, JR	2013
Ganoderma lucidum mushroom for the treatment of cardiovascular risk factors	Klupp, , NL, Chang, D, Hawke, F, Kiat, H, Cao, H, Grant, SJ; Bensoussan, A	2015
Frequency of endotracheal suctioning for the prevention of respiratory morbidity in ventilated newborns	Bruschettini, , M, Zappettini, S, Moja, L; Calevo, MG	2016
Hawthorn extract for treating chronic heart failure	Guo, , R, Pittler, MH; Ernst, E	2008
Group behaviour therapy programmes for smoking cessation	Stead, , LF, Carroll, AJ; Lancaster, T	2017
Gonadotrophin-releasing hormone analogues for endometriosis: bone mineral density	Farmer, , JE, Prentice, A, Breeze, A, Ahmad, G, Duffy, JMN, Watson, A; Pick, A	2003
Garlic for preventing pre-eclampsia and its complications	Meher, , S; Duley, L	2006
Haloperidol for agitation in dementia	Lonergan, , E, Luxenberg, J, Colford, JM; Birks, J	2002
H1-antihistamines for the treatment of anaphylaxis with and without shock	Sheikh, , A, ten Broek, VM, Brown, SGA; Simons, FER	2007
Herbal medicines for treatment of irritable bowel syndrome	Liu, , JP, Yang, M, Liu, Y, Wei, ML; Grimsgaard, S	2006

Hepatic late adverse effects after antineoplastic treatment for childhood cancer	Mulder, , RL, Bresters, D, Van den Hof, M, Koot, BGP, Castellino, SM, Loke, YKK, Post, PN, Postma, A, Szőnyi, LP, Levitt, GA, Bardi, E, Skinner, R; van Dalen, EC	2019
Graduated driver licensing for reducing motor vehicle crashes among young drivers	Russell, , KF, Vandermeer, B; Hartling, L	2011
Glucocorticosteroids for people with alcoholic hepatitis	Pavlov, , CS, Varganova, DL, Casazza, G, Tsochatzis, E, Nikolova, D; Gluud, C	2019
Gangliosides for acute ischaemic stroke	Candelise, , L; Ciccone, A	2001
Glucocorticoids for the treatment of anaphylaxis	Choo, , KJL, Simons, FER; Sheikh, A	2012
Growth hormone therapy for people with thalassaemia	Ngim, , CF, Lai, NM, Hong, JYH, Tan, SL, Ramadas, A, Muthukumarasamy, P; Thong, MK	2017
Ginkgo biloba for intermittent claudication	Nicolaï, , SPA, Kruidenier, LM, Bendermacher, BLW, Prins, MH, Stokmans, RA, Broos, PPHL; Teijink, JAW	2013
Gangliosides for acute spinal cord injury	Chinnock, , P; Roberts, I	2005
Gefitinib for advanced non-small cell lung cancer	Sim, , EHA, Yang, IA, Wood-Baker, R, Bowman, RV; Fong, KM	2018
Glucocorticosteroids for primary biliary cirrhosis	Prince, , M, Christensen, E; Gluud, C	2005
Helminth therapy (worms) for allergic rhinitis	Croft, , AM, Bager, P; Garg, SK	2012
Helicopter emergency medical services for adults with major trauma	Galvagno Jr, , SM, Sikorski, R, Hirshon, JM, Floccare, D, Stephens, C, Beecher, D; Thomas, S	2015
Histologic scoring indices for evaluation of disease activity in Crohn’s disease	Novak, , G, Parker, CE, Pai, RK, MacDonald, JK, Feagan, BG, Sandborn, WJ, D'Haens, G, Jairath, V; Khanna, R	2017
High dose rate versus low dose rate intracavity brachytherapy for locally advanced uterine cervix cancer	Liu, , R, Wang, X, Tian, JH, Yang, K, Wang, J, Jiang, L; Hao, XY	2014
High-dose versus low-dose oxytocin infusion regimens for induction of labour at term	Budden, , A, Chen, LJY; Henry, A	2014
High-dose therapy with autologous stem cell transplantation versus chemotherapy or immuno-chemotherapy for follicular lymphoma in adults	Schaaf, , M, Reiser, M, Borchmann, P, Engert, A; Skoetz, N	2012
Healing by primary versus secondary intention after surgical treatment for pilonidal sinus	AL-Khamis, , A, McCallum, I, King, PM; Bruce, J	2010
Home safety education and provision of safety equipment for injury prevention	Kendrick, , D, Young, B, Mason-Jones, AJ, Ilyas, N, Achana, FA, Cooper, NJ, Hubbard, SJ, Sutton, AJ, Smith, S, Wynn, P, Mulvaney, CA, Watson, MC; Coupland, C	2012
Head-to-head oral prophylactic antibiotic therapy for chronic obstructive pulmonary disease	Threapleton, , CJD, Janjua, S, Fortescue, R; Baker, EH	2019
Glucose-lowering agents for treating pre-existing and new-onset diabetes in kidney transplant recipients	Lo, , C, Jun, M, Badve, SV, Pilmore, H, White, SL, Hawley, C, Cass, A, Perkovic, V; Zoungas, S	2017
Home-based HIV voluntary counselling and testing (VCT) for improving uptake of HIV testing	Bateganya, , M, Abdulwadud, OA; Kiene, SM	2010
Glutamatergic drugs for schizophrenia	Tiihonen, , J; Wahlbeck, K	2006
Hearing aids for mild to moderate hearing loss in adults	Ferguson, , MA, Kitterick, PT, Chong, LY, Edmondson-Jones, M, Barker, F; Hoare, DJ	2017
Hormonal versus non-hormonal contraceptives in women with diabetes mellitus type 1 and 2	Visser, , J, Snel, M; Van Vliet, HAAM	2013
Heparin and related substances for preventing diabetic kidney disease	Li, , J, Wu, HM, Zhang, L, Zhu, B; Dong, BR	2010
Group-based parent training programmes for improving parental psychosocial health	Barlow, , J, Smailagic, N, Huband, N, Roloff, V; Bennett, C	2014
Garlic for the common cold	Lissiman, , E, Bhasale, AL; Cohen, M	2014
Heparin for assisted reproduction	Akhtar, , MA, Sur, SD, Raine-Fenning, N, Jayaprakasan, K, Thornton, JG; Quenby, S	2013
HMG CoA reductase inhibitors (statins) for kidney transplant recipients	Palmer, , SC, Navaneethan, SD, Craig, JC, Perkovic, V, Johnson, DW, Nigwekar, SU, Hegbrant, J; Strippoli, GFM	2014
High-flux versus low-flux membranes for end-stage kidney disease	Palmer, , SC, Rabindranath, KS, Craig, JC, Roderick, PJ, Locatelli, F; Strippoli, GFM	2012
Gloves, gowns and masks for reducing the transmission of meticillin-resistant Staphylococcus aureus (MRSA) in the hospital setting	López-Alcalde, , J, Mateos-Mazón, M, Guevara, M, Conterno, LO, Solà, I, Cabir Nunes, S; Bonfill Cosp, X	2015
Gamma aminobutyric acid (GABA) modulators for amyotrophic lateral sclerosis/motor neuron disease	Diana, , A, Pillai, R, Bongioanni, P, O'Keeffe, AG, Miller, RG; Moore, DH	2017
Follow-up strategies for women treated for early breast cancer	Moschetti, , I, Cinquini, M, Lambertini, M, Levaggi, A; Liberati, A	2016
Glucocorticoids for acute viral bronchiolitis in infants and young children	Fernandes, , RM, Bialy, LM, Vandermeer, B, Tjosvold, L, Plint, AC, Patel, H, Johnson, DW, Klassen, TP; Hartling, L	2013
Hand washing promotion for preventing diarrhoea	Ejemot-Nwadiaro, , RI, Ehiri, JE, Arikpo, D, Meremikwu, MM; Critchley, JA	2015
Galantamine for vascular cognitive impairment	Birks, , J; Craig, D	2006
Gowning by attendants and visitors in newborn nurseries for prevention of neonatal morbidity and mortality	Webster, , J; Pritchard, MA	2003
Glutamine supplementation for young infants with severe gastrointestinal disease	Brown, , JVE, Moe-Byrne, T; McGuire, W	2014

Force platform feedback for standing balance training after stroke	Barclay-Goddard, , RE, Stevenson, TJ, Poluha, W, Moffatt, M; Taback, SP	2004
Home uterine monitoring for detecting preterm labour	Urquhart, , C, Currell, R, Harlow, F; Callow, L	2017
Gene therapy for haemophilia	Sharma, , A, Easow Mathew, M, Sriganesh, V; Reiss, UM	2016
Glucocorticoid supplementation during ovarian stimulation for IVF or ICSI	Kalampokas, , T, Pandian, Z, Keay, SD; Bhattacharya, S	2017
Golimumab for rheumatoid arthritis	Singh, , JA, Noorbaloochi, S; Singh, G	2010
Home fortification of foods with multiple micronutrient powders for health and nutrition in children under two years of age	De-Regil, , LM, Suchdev, PS, Vist, GE, Walleser, S; Peña-Rosas, JP	2011
High-dose chemotherapy followed by autologous stem cell transplantation for patients with relapsed/refractory Hodgkin lymphoma	Rancea, , M, Monsef, I, von Tresckow, B, Engert, A; Skoetz, N	2013
Heliox inhalation therapy for bronchiolitis in infants	Liet, , JM, Ducruet, T, Gupta, V; Cambonie, G	2015
Ginseng for cognition	Geng, , J, Dong, J, Ni, H, Lee, MS, Wu, T, Jiang, K, Wang, G, Zhou, AL; Malouf, R	2010
High-dose chemotherapy for children and young adults with stage IV rhabdomyosarcoma	Admiraal, , R, van der Paardt, M, Kobes, J, Kremer, LCM, Bisogno, G; Merks, JHM	2010
High frequency jet ventilation versus high frequency oscillatory ventilation for pulmonary dysfunction in preterm infants	Ethawi, , YH, Abou Mehrem, A, Minski, J, Ruth, CA; Davis, PG	2016
Home versus in-patient treatment for deep vein thrombosis	Othieno, , R, Okpo, E; Forster, R	2018
Haemophilus influenzae oral vaccination for preventing acute exacerbations of chronic bronchitis and chronic obstructive pulmonary disease	Teo, , E, Lockhart, K, Purchuri, SN, Pushparajah, J, Cripps, AW; van Driel, ML	2017
Gastrostomy feeding versus oral feeding alone for children with cerebral palsy	Gantasala, , S, Sullivan, PB; Thomas, AG	2013
Heparin-bonded catheters for prolonging the patency of central venous catheters in children	Shah, , PS; Shah, N	2014
Guidelines in professions allied to medicine	Thomas, , LH, Cullum, NA, McColl, E, Rousseau, N, Soutter, J; Steen, N	1999
Fundal pressure during the second stage of labour	Hofmeyr, , GJ, Vogel, JP, Cuthbert, A; Singata, M	2017
Functional analysis-based interventions for challenging behaviour in dementia	Moniz Cook, , ED, Swift, K, James, I, Malouf, R, De Vugt, M; Verhey, F	2012
Folic acid with or without vitamin B12 for the prevention and treatment of healthy elderly and demented people	Malouf, , R; Grimley Evans, J	2008
Fortification of staple foods with zinc for improving zinc status and other health outcomes in the general population	Shah, , D, Sachdev, HS, Gera, T, De-Regil, LM; Peña-Rosas, JP	2016
Genomics-based non-invasive prenatal testing for detection of fetal chromosomal aneuploidy in pregnant women	Badeau, , M, Lindsay, C, Blais, J, Nshimyumukiza, L, Takwoingi, Y, Langlois, S, Légaré, F, Giguère, Y, Turgeon, AF, Witteman, W; Rousseau, F	2017
Herbal medicines for fatty liver diseases	Liu, , ZL, Xie, LZ, Zhu, J, Li, GQ, Grant, SJ; Liu, JP	2013
Granulocyte and granulocyte-macrophage colony stimulating factors for newly diagnosed patients with myelodysplastic syndromes	Hutzschenreuter, , F, Monsef, I, Kreuzer, KA, Engert, A; Skoetz, N	2016
Granulocyte-colony stimulating factors as adjunctive therapy for diabetic foot infections	Cruciani, , M, Lipsky, BA, Mengoli, C; de Lalla, F	2013
Glatiramer acetate for multiple sclerosis	La Mantia, , L, Munari, LM; Lovati, R	2010
Garlic for the prevention of cardiovascular morbidity and mortality in hypertensive patients	Stabler, , SN, Tejani, AM, Huynh, F; Fowkes, C	2012
Granulocyte transfusions for neonates with confirmed or suspected sepsis and neutropenia	Pammi, , M; Brocklehurst, P	2011
Hand assisted laparoscopic surgery versus conventional laparoscopy for colorectal surgery	Moloo, , H, Haggar, F, Coyle, D, Hutton, B, Duhaime, S, Mamazza, J, Poulin, EC, Boushey, RP; Grimshaw, J	2010
Higher versus lower protein intake in formula-fed low birth weight infants	Fenton, , TR, Premji, SS, Al-Wassia, H; Sauve, RS	2014
Heliox for non-intubated acute asthma patients	Rodrigo, , GJ, Pollack, CV, Rodrigo, C; Rowe, BH	2006
Granulocyte-Colony Stimulating Factor (G-CSF) as an adjunct to antibiotics in the treatment of pneumonia in adults	Cheng, , AC, Stephens, DP; Currie, BJ	2007
Home-based child development interventions for preschool children from socially disadvantaged families	Miller, , S, Maguire, LK; Macdonald, G	2011
Homeopathy for chronic asthma	McCarney, , RW, Linde, K; Lasserson, TJ	2004
HMG CoA reductase inhibitors (statins) for people with chronic kidney disease not requiring dialysis	Palmer, , SC, Navaneethan, SD, Craig, JC, Johnson, DW, Perkovic, V, Hegbrant, J; Strippoli, GFM	2014
Growth factors for angiogenesis in peripheral arterial disease	Gorenoi, , V, Brehm, MU, Koch, A; Hagen, A	2017
Ginkgo biloba for tinnitus	Hilton, , MP, Zimmermann, EF; Hunt, WT	2013
Haemoglobin and haematocrit targets for the anaemia of chronic kidney disease	Strippoli, , GFM, Navaneethan, SD, Craig, JC; Palmer, SC	2006
Hepatitis A immunisation in persons not previously exposed to hepatitis A	Irving, , GJ, Holden, J, Yang, R; Pope, D	2012
Group versus conventional antenatal care for women	Catling, , CJ, Medley, N, Foureur, M, Ryan, C, Leap, N, Teate, A; Homer, CSE	2015

High versus low dose of initial thyroid hormone replacement for congenital hypothyroidism	Ng, , SM, Anand, D; Weindling, AM	2009
H1-antihistamines for chronic spontaneous urticaria	Sharma, , M, Bennett, C, Cohen, SN; Carter, B	2014
Haloperidol dose for the acute phase of schizophrenia	Donnelly, , L, Rathbone, J; Adams, CE	2013
Frequency of dressing changes for central venous access devices on catheter-related infections	Gavin, , NC, Webster, J, Chan, RJ; Rickard, CM	2016
Gamma-hydroxybutyrate (GHB) for treatment of alcohol withdrawal and prevention of relapses	Leone, , MA, Vigna-Taglianti, F, Avanzi, G, Brambilla, R; Faggiano, F	2010
High-carbohydrate, high-protein, low-fat versus low-carbohydrate, high-protein, high-fat enteral feeds for burns	Masters, , B, Aarabi, S, Sidhwa, F; Wood, F	2012
Histamine type 2 receptor antagonists as adjuvant treatment for resected colorectal cancer	Deva, , S; Jameson, M	2012
Genital ulcer disease treatment for reducing sexual acquisition of HIV	Mutua, , FM, M'Imunya, JM; Wiysonge, CS	2012
HMG CoA reductase inhibitors (statins) for preventing acute kidney injury after surgical procedures requiring cardiac bypass	Lewicki, , M, Ng, I; Schneider, AG	2015
Haloperidol versus low-potency first-generation antipsychotic drugs for schizophrenia	Tardy, , M, Huhn, M, Kissling, W, Engel, RR; Leucht, S	2014
Hormonal contraception for women exposed to HIV infection	Hofmeyr, , GJ, Singata, M; Sneden, J	2014
Hands-on therapy interventions for upper limb motor dysfunction following stroke	Winter, , J, Hunter, S, Sim, J; Crome, P	2011
Growth hormone for children with chronic kidney disease	Hodson, , EM, Willis, NS; Craig, JC	2012
Haloperidol for psychosis-induced aggression or agitation (rapid tranquillisation)	Ostinelli, , EG, Brooke-Powney, MJ, Li, X; Adams, CE	2017
Gastro-oesophageal reflux treatment for prolonged non-specific cough in children and adults	Chang, , AB, Lasserson, TJ, Gaffney, J, Connor, FL; Garske, LA	2011
GenoType® MTBDRsl assay for resistance to second-line anti-tuberculosis drugs	Theron, , G, Peter, J, Richardson, M, Warren, R, Dheda, K; Steingart, KR	2016
Galactomannan detection in broncho-alveolar lavage fluid for invasive aspergillosis in immunocompromised patients	de Heer, , K, Gerritsen, MG, Visser, CE; Leeflang, MMG	2019
Fundoplication versus postoperative medication for gastro-oesophageal reflux in children with neurological impairment undergoing gastrostomy	Vernon-Roberts, , A; Sullivan, PB	2013
Habit retraining for the management of urinary incontinence in adults	Ostaszkiewicz, , J, Chestney, T; Roe, B	2004
Formula versus maternal breast milk for feeding preterm or low birth weight infants	Brown, , JVE, Walsh, V; McGuire, W	2019
Green tea (Camellia sinensis) for the prevention of cancer	Boehm, , K, Borrelli, F, Ernst, E, Habacher, G, Hung, SK, Milazzo, S; Horneber, M	2009
Follow-up strategies for patients treated for non-metastatic colorectal cancer	Jeffery, , M, Hickey, BE; Hider, PN	2019
Glucocorticoid with cyclophosphamide for paraquat-induced lung fibrosis	Li, , LR, Sydenham, E, Chaudhary, B, Beecher, D; You, C	2014
Helicobacter pylori eradication for the prevention of gastric neoplasia	Ford, , AC, Forman, D, Hunt, R, Yuan, Y; Moayyedi, P	2015
Home-based educational interventions for children with asthma	Welsh, , EJ, Hasan, M; Li, P	2011
Home versus center based physical activity programs in older adults	Ashworth, , NL, Chad, KE, Harrison, EL, Reeder, BA; Marshall, SC	2005
General physical health advice for people with serious mental illness	Tosh, , G, Clifton, AV, Xia, J; White, MM	2014
Framing of health information messages	Akl, , EA, Oxman, AD, Herrin, J, Vist, GE, Terrenato, I, Sperati, F, Costiniuk, C, Blank, D; Schünemann, H	2011
High frequency oscillatory ventilation versus conventional ventilation for infants with severe pulmonary dysfunction born at or near term	De Paoli, , AG, Clark, RH, Bhuta, T; Henderson-Smart, DJ	2009
Guanylate cyclase stimulators for pulmonary hypertension	Wardle, , AJ, Seager, MJ, Wardle, R, Tulloh, RMR; Gibbs, JSR	2016
Gold as an oral corticosteroid sparing agent in stable asthma	Evans, , DJ, Cullinan, P, Geddes, DM, Walters, EH, Milan, SJ; Jones, P	2000
Gene therapy for peripheral arterial disease	Forster, , R, Liew, A, Bhattacharya, V, Shaw, J; Stansby, G	2018
Glutamine for induction of remission in Crohn's disease	Akobeng, , AK, Elawad, M; Gordon, M	2016
Home-based therapy programmes for upper limb functional recovery following stroke	Coupar, , F, Pollock, A, Legg, LA, Sackley, C; van Vliet, P	2012
Grommets (ventilation tubes) for recurrent acute otitis media in children	Venekamp, , RP, Mick, P, Schilder, AGM; Nunez, DA	2018
Guided imagery for treating hypertension in pregnancy	Haruna, , M, Matsuzaki, M, Ota, E, Shiraishi, M, Hanada, N; Mori, R	2019
Home or foster home care versus institutional long-term care for functionally dependent older people	Young, , C, Hall, AM, Gonçalves-Bradley, DC, Quinn, TJ, Hooft, L, van Munster, BC; Stott, DJ	2017
Hormonal therapy in advanced or recurrent endometrial cancer	Kokka, , F, Brockbank, E, Oram, D, Gallagher, C; Bryant, A	2010
Heparin for prolonging peripheral intravenous catheter use in neonates	Shah, , PS, Ng, E; Sinha, AK	2005
Home- versus hospital-based phototherapy for the treatment of non-haemolytic jaundice in infants at more than 37 weeks' gestation	Malwade, , US; Jardine, LA	2014

Foot orthoses for patellofemoral pain in adults	Hossain, , M, Alexander, P, Burls, A; Jobanputra, P	2011
Holding chambers versus nebulisers for inhaled steroids in chronic asthma	Cates, , CJ, Bestall, JC; Adams, NP	2006
High-intensity versus low-intensity physical activity or exercise in people with hip or knee osteoarthritis	Regnaux, , JP, Lefevre-Colau, MM, Trinquart, L, Nguyen, C, Boutron, I, Brosseau, L; Ravaud, P	2015
Green and black tea for the primary prevention of cardiovascular disease	Hartley, , L, Flowers, N, Holmes, J, Clarke, A, Stranges, S, Hooper, L; Rees, K	2013
Heliox for croup in children	Moraa, , I, Sturman, N, McGuire, TM; van Driel, ML	2018
G-CSF and GM-CSF for treating or preventing neonatal infections	Carr, , R, Modi, N; Doré, CJ	2003
Haematological interventions for treating disseminated intravascular coagulation during pregnancy and postpartum	Martí-Carvajal, , AJ, Comunián-Carrasco, G; Peña-Martí, GE	2011
Fortification of staple foods with vitamin A for vitamin A deficiency	Hombali, , AS, Solon, JA, Venkatesh, BT, Nair, NS; Peña-Rosas, JP	2019
Glucocorticosteroids for infants with biliary atresia following Kasai portoenterostomy	Tyraskis, , A, Parsons, C; Davenport, M	2018
Glucocorticosteroids for primary sclerosing cholangitis	Giljaca, , V, Poropat, G, Stimac, D; Gluud, C	2010
Glycoprotein IIb-IIIa inhibitors for acute ischaemic stroke	Ciccone, , A, Motto, C, Abraha, I, Cozzolino, F; Santilli, I	2014
Glucocorticosteroid-free versus glucocorticosteroid-containing immunosuppression for liver transplanted patients	Fairfield, , C, Penninga, L, Powell, J, Harrison, EM; Wigmore, SJ	2018
Guidewire-assisted cannulation of the common bile duct for the prevention of post-endoscopic retrograde cholangiopancreatography (ERCP) pancreatitis	Tse, , F, Yuan, Y, Moayyedi, P; Leontiadis, GI	2012
High-dose chemotherapy with autologous stem cell transplantation in the first line treatment of aggressive Non-Hodgkin Lymphoma (NHL) in adults	Greb, , A, Bohlius, J, Schiefer, D, Schwarzer, G, Schulz, H; Engert, A	2008
Home-based versus centre-based cardiac rehabilitation	Anderson, , L, Sharp, GA, Norton, RJ, Dalal, H, Dean, SG, Jolly, K, Cowie, A, Zawada, A; Taylor, RS	2017
Histamine H2-receptor antagonists for urticaria	Fedorowicz, , Z, van Zuuren, EJ; Hu, N	2012
Helicobacter pylori eradication therapy vs. antisecretory non-eradication therapy (with or without long-term maintenance antisecretory therapy) for the prevention of recurrent bleeding from peptic ulcer	Gisbert, , JP, Khorrami, S, Carballo, F, Calvet, X, Gené, E; Dominguez-Muñoz, E	2004
Haemodilution for acute ischaemic stroke	Chang, , TS; Jensen, MB	2014
Glutamine supplementation for critically ill adults	Tao, , KM, Li, XQ, Yang, LQ, Yu, WF, Lu, ZJ, Sun, YM; Wu, FX	2014
Fortification of maize flour with iron for controlling anaemia and iron deficiency in populations	Garcia-Casal, , MN, Peña-Rosas, JP, De-Regil, LM, Gwirtz, JA; Pasricha, SR	2018
Granulocyte colony stimulating factor therapy for acute myocardial infarction	Moazzami, , K, Roohi, A; Moazzami, B	2013
Histologic scoring indices for evaluation of disease activity in ulcerative colitis	Mosli, , MH, Parker, CE, Nelson, SA, Baker, KA, MacDonald, JK, Zou, GY, Feagan, BG, Khanna, R, Levesque, BG; Jairath, V	2017
Gloves, extra gloves or special types of gloves for preventing percutaneous exposure injuries in healthcare personnel	Mischke, , C, Verbeek, JH, Saarto, A, Lavoie, MC, Pahwa, M; Ijaz, S	2014
Gene therapy for sickle cell disease	Olowoyeye, , A; Okwundu, CI	2018
Glutamine supplementation to prevent morbidity and mortality in preterm infants	Moe-Byrne, , T, Brown, JVE; McGuire, W	2016
Heparin versus 0.9% sodium chloride intermittent flushing for the prevention of occlusion in long term central venous catheters in infants and children	Bradford, , NK, Edwards, RM; Chan, RJ	2015
High-flow nasal cannula therapy for respiratory support in children	Mayfield, , S, Jauncey-Cooke, J, Hough, JL, Schibler, A, Gibbons, K; Bogossian, F	2014
Home-based care for reducing morbidity and mortality in people infected with HIV/AIDS	Young, , T; Busgeeth, K	2010
Green tea for weight loss and weight maintenance in overweight or obese adults	Jurgens, , TM, Whelan, AM, Killian, L, Doucette, S, Kirk, S; Foy, E	2012
High-dose chemotherapy and autologous bone marrow or stem cell transplantation versus conventional chemotherapy for women with early poor prognosis breast cancer	Farquhar, , C, Marjoribanks, J, Lethaby, A; Azhar, M	2016
Formoterol versus short-acting beta-agonists as relief medication for adults and children with asthma	Welsh, , EJ; Cates, CJ	2010
Functional endoscopic balloon dilation of sinus ostia for chronic rhinosinusitis	Ahmed, , J, Pal, S, Hopkins, C; Jayaraj, S	2011
Health system and community level interventions for improving antenatal care coverage and health outcomes	Mbuagbaw, , L, Medley, N, Darzi, AJ, Richardson, M, Habiba Garga, K; Ongolo-Zogo, P	2015
Graduated compression stockings for prevention of deep vein thrombosis	Sachdeva, , A, Dalton, M; Lees, T	2018
Honey for acute cough in children	Oduwole, , O, Udoh, EE, Oyo-Ita, A; Meremikwu, MM	2018
Fortification of wheat and maize flour with folic acid for population health outcomes	Centeno Tablante, , E, Pachón, H, Guetterman, HM; Finkelstein, JL	2019
High-frequency ultrasound for diagnosing skin cancer in adults	Dinnes, , J, Bamber, J, Chuchu, N, Bayliss, SE, Takwoingi, Y, Davenport, C, Godfrey, K, O'Sullivan, C, Matin, RN, Deeks, JJ; Williams, HC	2018

Genetic testing for prevention of severe drug-induced skin rash	Alfirevic, , A, Pirmohamed, M, Marinovic, B, Harcourt-Smith, L, Jorgensen, AL; Cooper, TE	2019
Hypnotherapy for smoking cessation	Barnes, , J, McRobbie, H, Dong, CY, Walker, N; Hartmann-Boyce, J	2019
Insulin for glycaemic control in acute ischaemic stroke	Bellolio, , MF, Gilmore, RM; Ganti, L	2014
Inhaled corticosteroid effects on bone metabolism in asthma and mild chronic obstructive pulmonary disease	Jones, , A, Fay, JK, Burr, ML, Stone, M, Hood, K; Roberts, G	2002
Indoor residual spraying for preventing malaria	Pluess, , B, Tanser, FC, Lengeler, C; Sharp, BL	2010
Insulin and oral agents for managing cystic fibrosis-related diabetes	Onady, , GM; Stolfi, A	2016
Increased energy intake for preterm infants with (or developing) bronchopulmonary dysplasia/chronic lung disease	Lai, , NM, Rajadurai, SV; Tan, K	2006
Hydroxyurea (hydroxycarbamide) for sickle cell disease	Nevitt, , SJ, Jones, AP; Howard, J	2017
In vitro fertilisation versus tubal reanastomosis (sterilisation reversal) for subfertility after tubal sterilisation	Yossry, , M, Aboulghar, M, D'Angelo, A; Gillett, W	2006
Hormone replacement for osteoporosis in women with primary biliary cirrhosis	Rudic, , JS, Poropat, G, Krstic, MN, Bjelakovic, G; Gluud, C	2011
Image-guided versus blind glucocorticoid injection for shoulder pain	Bloom, , JE, Rischin, A, Johnston, RV; Buchbinder, R	2012
Inotropic agents and vasodilator strategies for the treatment of cardiogenic shock or low cardiac output syndrome	Schumann, , J, Henrich, EC, Strobl, H, Prondzinsky, R, Weiche, S, Thiele, H, Werdan, K, Frantz, S; Unverzagt, S	2018
Immunonutrition for acute respiratory distress syndrome (ARDS) in adults	Dushianthan, , A, Cusack, R, Burgess, VA, Grocott, MPW; Calder, PC	2019
Hysterectomy with radiotherapy or chemotherapy or both for women with locally advanced cervical cancer	Kokka, , F, Bryant, A, Brockbank, E, Powell, M; Oram, D	2015
Hypertonic salt solution for peri-operative fluid management	Shrum, , B, Church, B, McArthur, E, Burns, KEA, Znajda, T; McAlister, V	2016
Incentive spirometry for prevention of postoperative pulmonary complications in upper abdominal surgery	do Nascimento Junior, , P, Módolo, NSP, Andrade, S, Guimarães, MMF, Braz, LG; El Dib, R	2014
Hypothermia for neuroprotection in adults after cardiopulmonary resuscitation	Arrich, , J, Holzer, M, Havel, C, Müllner, M; Herkner, H	2016
Influenza vaccines for preventing cardiovascular disease	Clar, , C, Oseni, Z, Flowers, N, Keshtkar-Jahromi, M; Rees, K	2015
Hydroxyurea (hydroxycarbamide) for transfusion-dependent β-thalassaemia	Ansari, , SH, Lassi, ZS, Khowaja, SM, Adil, SO; Shamsi, TS	2019
Hyperbaric oxygen therapy for delayed onset muscle soreness and closed soft tissue injury	Bennett, , MH, Best, TM, Babul-Wellar, S; Taunton, JE	2005
Immunotherapy (excluding checkpoint inhibitors) for stage I to III non-small cell lung cancer treated with surgery or radiotherapy with curative intent	Zhu, , J, Li, R, Tiselius, E, Roudi, R, Teghararian, O, Suo, C; Song, H	2017
Hyperbaric oxygen therapy for promoting fracture healing and treating fracture non-union	Bennett, , MH, Stanford, RE; Turner, R	2012
Immunosuppressant and immunomodulatory treatment for dermatomyositis and polymyositis	Gordon, , PA, Winer, JB, Hoogendijk, JE; Choy, EHS	2012
Immunostimulants for preventing respiratory tract infection in children	Del-Rio-Navarro, , BE, Espinosa-Rosales, FJ, Flenady, V; Sienra-Monge, JJL	2006
Hormone therapy in postmenopausal women and risk of endometrial hyperplasia	Furness, , S, Roberts, H, Marjoribanks, J; Lethaby, A	2012
Human papillomavirus testing versus repeat cytology for triage of minor cytological cervical lesions	Arbyn, , M, Roelens, J, Simoens, C, Buntinx, F, Paraskevaidis, E, Martin-Hirsch, PPL; Prendiville, WJ	2013
Injection sclerotherapy for varicose veins	Tisi, , PV, Beverley, C; Rees, A	2006
Indoor air modification interventions for prolonged non-specific cough in children	Donnelly, , D, Everard, M; Chang, AB	2006
Immunotherapy for idiopathic lumbosacral plexopathy	van Eijk, , J, Chan, YC; Russell, JW	2013
Inhaled nitric oxide for the postoperative management of pulmonary hypertension in infants and children with congenital heart disease	Bizzarro, , M, Gross, I; Barbosa, FT	2014
Inhaled nitric oxide for respiratory failure in preterm infants	Barrington, , KJ, Finer, N; Pennaforte, T	2017
Immunotherapy for recurrent miscarriage	Wong, , LF, Porter, TF; Scott, JR	2014
Injection therapy for subacute and chronic low-back pain	Staal, , JB, de Bie, R, de Vet, HCW, Hildebrandt, J; Nelemans, P	2008
Inhaled versus systemic corticosteroids for the treatment of bronchopulmonary dysplasia in ventilated very low birth weight preterm infants	Shah, , SS, Ohlsson, A, Halliday, HL; Shah, VS	2017
Hyperbaric oxygen therapy for people with autism spectrum disorder (ASD)	Xiong, , T, Chen, H, Luo, R; Mu, D	2016
Human chorionic gonadotrophin (hCG) for preventing miscarriage	Morley, , LC, Simpson, N; Tang, T	2013
Inhaled versus systemic corticosteroids for preventing bronchopulmonary dysplasia in ventilated very low birth weight preterm neonates	Shah, , SS, Ohlsson, A, Halliday, HL; Shah, VS	2017
Hypothermia to reduce neurological damage following coronary artery bypass surgery	Rees, , K, Beranek-Stanley, M, Burke, M; Ebrahim, S	2001

Hyperfractionated or accelerated radiotherapy for head and neck cancer	Baujat, , B, Bourhis, J, Blanchard, P, Overgaard, J, Ang, KK, Saunders, M, Le Maître, A, Bernier, J, Horiot, JC, Maillard, E, Pajak, TF, Poulsen, MG, Bourredjem, A, O'Sullivan, B, Dobrowsky, W, Andrzej, H, Skladowski, K, Hay, JH, Pinto, LHJ, Fu, KK, Fallai, C, Sylvester, R; Pignon, JP	2010
Hypothermia for neuroprotection in children after cardiopulmonary arrest	Scholefield, , B, Duncan, H, Davies, P, Gao Smith, F, Khan, K, Perkins, GD; Morris, K	2013
Infusion techniques for peripheral arterial thrombolysis	Kessel, , DO, Berridge, DC; Robertson, I	2004
Hydralazine for essential hypertension	Kandler, , MR, Mah, GT, Tejani, AM, Stabler, SN; Salzwedel, DM	2011
Indwelling bladder catheterisation as part of intraoperative and postoperative care for caesarean section	Abdel-Aleem, , H, Aboelnasr, MF, Jayousi, TM; Habib, FA	2014
Implementation strategies for health systems in low-income countries: an overview of systematic reviews	Pantoja, , T, Opiyo, N, Lewin, S, Paulsen, E, Ciapponi, A, Wiysonge, CS, Herrera, CA, Rada, G, Peñaloza, B, Dudley, L, Gagnon, MP, Garcia Marti, S; Oxman, AD	2017
Inhaled corticosteroids in children with persistent asthma: effects on growth	Zhang, , L, Prietsch, SOM; Ducharme, FM	2014
Immediate start of hormonal contraceptives for contraception	Lopez, , LM, Newmann, SJ, Grimes, DA, Nanda, K; Schulz, KF	2012
Injectable gold for rheumatoid arthritis	Clark, , P, Tugwell, P, Bennett, KJ, Bombardier, C, Shea, B, Wells, GA; Suarez-Almazor, ME	1997
Information provision for people with multiple sclerosis	Köpke, , S, Solari, A, Rahn, A, Khan, F, Heesen, C; Giordano, A	2018
Inhaled steroids for episodic viral wheeze of childhood	McKean, , MC; Ducharme, F	2000
Hyperbaric oxygen for carbon monoxide poisoning	Buckley, , NA, Juurlink, DN, Isbister, G, Bennett, MH; Lavonas, EJ	2011
Influenza vaccination in children being treated with chemotherapy for cancer	Goossen, , GM, Kremer, LCM; van de Wetering, MD	2013
Information for pregnant women about caesarean birth	Horey, , D, Weaver, J; Russell, H	2004
Inhaled cromones for prolonged non-specific cough in children	Chang, , AB, Marchant, JM, McKean, MC; Morris, PS	2004
Hypothermia for traumatic brain injury	Lewis, , SR, Evans, DJW, Butler, AR, Schofield-Robinson, OJ; Alderson, P	2017
Immediate versus delayed treatment for recently symptomatic carotid artery stenosis	Vasconcelos, , V, Cassola, N, da Silva, EMK; Baptista-Silva, JCC	2016
Hormones for suspected impaired fetal growth	Say, , L, Gülmezoglu, AM; Hofmeyr, GJ	2003
Increased versus stable doses of inhaled corticosteroids for exacerbations of chronic asthma in adults and children	Kew, , KM, Quinn, M, Quon, BS; Ducharme, FM	2016
House dust mite avoidance measures for perennial allergic rhinitis	Sheikh, , A, Hurwitz, B, Nurmatov, U; van Schayck, CP	2010
Inhaled nitric oxide for acute chest syndrome in people with sickle cell disease	Al Hajeri, , A, Serjeant, GR; Fedorowicz, Z	2008
Hysterectomy versus hysterectomy plus oophorectomy for premenopausal women	Orozco, , LJ, Tristan, M, Vreugdenhil, MMT; Salazar, A	2014
Inhaled versus oral steroids for adults with chronic asthma	Mash, , BRJ, Bheekie, A; Jones, P	2001
Hormone replacement therapy for women with type 1 diabetes mellitus	Mackay, , L, Kilbride, L, Adamson, KA; Chisholm, J	2013
Imaging for the exclusion of pulmonary embolism in pregnancy	van Mens, , TE, Scheres, LJJ, de Jong, PG, Leeflang, MMG, Nijkeuter, M; Middeldorp, S	2017
Insecticide space spraying for preventing malaria transmission	Pryce, , J, Choi, L, Richardson, M; Malone, D	2018
Inhaled anticholinergics and short-acting beta2-agonists versus short-acting beta2-agonists alone for children with acute asthma in hospital	Vézina, , K, Chauhan, BF; Ducharme, FM	2014
Hyperbaric oxygen therapy for multiple sclerosis	Bennett, , MH; Heard, R	2004
Household interventions for preventing domestic lead exposure in children	Nussbaumer-Streit, , B, Yeoh, B, Griebler, U, Pfadenhauer, LM, Busert, LK, Lhachimi, SK, Lohner, S; Gartlehner, G	2016
Indomethacin for Alzheimer's disease	Tabet, , N; Feldman, H	2002
Image-guided percutaneous procedure plus metronidazole versus metronidazole alone for uncomplicated amoebic liver abscess	Chavez-Tapia, , NC, Hernandez-Calleros, J, Tellez-Avila, FI, Torre, A; Uribe, M	2009
Image guided surgery for the resection of brain tumours	Barone, , DG, Lawrie, TA; Hart, MG	2014
Immediate versus deferred delivery of the preterm baby with suspected fetal compromise for improving outcomes	Stock, , SJ, Bricker, L, Norman, JE; West, HM	2016
Hydration for treatment of preterm labour	Stan, , CM, Boulvain, M, Pfister, R; Hirsbrunner-Almagbaly, P	2013
Horticultural therapy for schizophrenia	Liu, , Y, Bo, L, Sampson, S, Roberts, S, Zhang, G; Wu, W	2014
Hospital nurse-staffing models and patient- and staff-related outcomes	Butler, , M, Schultz, TJ, Halligan, P, Sheridan, A, Kinsman, L, Rotter, T, Beaumier, J, Kelly, RG; Drennan, J	2019
Inhaled short acting beta2-agonist use in chronic asthma: regular versus as needed treatment	Walters, , EH, Walters, JAE, Gibson, PG; Jones, P	2003
Human recombinant activated factor VII for upper gastrointestinal bleeding in patients with liver diseases	Martí-Carvajal, , AJ, Karakitsiou, DE; Salanti, G	2012

Immunosuppressants for the prophylaxis of corneal graft rejection after penetrating keratoplasty	Abudou, , M, Wu, T, Evans, JR; Chen, X	2015
Ibuprofen for acute treatment of episodic tension-type headache in adults	Derry, , S, Wiffen, PJ, Moore, RA; Bendtsen, L	2015
Immunosuppressive treatment for non-systemic vasculitic neuropathy	Vrancken, , AFJE, Hughes, RAC, Said, G, Wokke, JHJ; Notermans, NC	2007
Immunomodulators and immunosuppressants for multiple sclerosis: a network meta-analysis	Filippini, , G, Del Giovane, C, Vacchi, L, D'Amico, R, Di Pietrantonj, C, Beecher, D; Salanti, G	2013
Hyperbaric oxygen therapy for vascular dementia	Xiao, , Y, Wang, J, Jiang, S; Luo, H	2012
Immediate postabortal insertion of intrauterine devices	Okusanya, , BO, Oduwole, O; Effa, EE	2014
House dust mite control measures for asthma	Gøtzsche, , PC; Johansen, HK	2008
Increasing antipsychotic dose versus switching antipsychotic for non response in schizophrenia	Samara, , MT, Klupp, E, Helfer, B, Rothe, PH, Schneider-Thoma, J; Leucht, S	2018
Immune tolerance induction for treating inhibitors in people with congenital haemophilia A or B	Athale, , AH, Marcucci, M; Iorio, A	2014
Hydralazine in infants with persistent hypoxemic respiratory failure	Kawaguchi, , A, Isayama, T, Mori, R, Minami, H, Yang, Y; Tamura, M	2013
Hydroxyzine for generalised anxiety disorder	Guaiana, , G, Barbui, C; Cipriani, A	2010
Inhaled corticosteroids with combination inhaled long-acting beta2-agonists and long-acting muscarinic antagonists for chronic obstructive pulmonary disease	Tan, , DJ, White, CJ, Walters, JAE; Walters, EH	2016
Inhaled non-steroid anti-inflammatories for children and adults with bronchiectasis	Pizzutto, , SJ, Upham, JW, Yerkovich, ST; Chang, AB	2016
Immunosuppressant and immunomodulatory treatments for multifocal motor neuropathy	Umapathi, , T, Hughes, RAC, Nobile-Orazio, E; Léger, JM	2015
Hydromorphone for neuropathic pain in adults	Stannard, , C, Gaskell, H, Derry, S, Aldington, D, Cole, P, Cooper, TE, Knaggs, R, Wiffen, PJ; Moore, RA	2016
Individual patient education for low back pain	Engers, , AJ, Jellema, P, Wensing, M, van der Windt, DAWM, Grol, R; van Tulder, MW	2008
Immunosuppressive treatment for focal segmental glomerulosclerosis in adults	Braun, , N, Schmutzler, F, Lange, C, Perna, A, Remuzzi, G; Willis, NS	2008
Inhaled corticosteroids for subacute cough in children	Anderson-James, , S, Marchant, JM, Acworth, JP, Turner, C; Chang, AB	2013
Horse chestnut seed extract for chronic venous insufficiency	Pittler, , MH; Ernst, E	2012
Human growth hormone and glutamine for patients with short bowel syndrome	Wales, , PW, Nasr, A, de Silva, N; Yamada, J	2010
Impact of institutional smoking bans on reducing harms and secondhand smoke exposure	Frazer, , K, McHugh, J, Callinan, JE; Kelleher, C	2016
Immunotherapy (oral and sublingual) for food allergy to fruits	Yepes-Nuñez, , JJ, Zhang, Y, Roqué i Figuls, M, Bartra Tomas, J, Reyes, JM, Pineda de la Losa, F; Enrique, E	2015
Inhaled corticosteroids for subacute and chronic cough in adults	Johnstone, , KJ, Chang, AB, Fong, KM, Bowman, RV; Yang, IA	2013
Inhaled corticosteroids for cystic fibrosis	Balfour-Lynn, , IM, Welch, K; Smith, S	2019
Impact of Haemophilus influenzae type B (Hib) and viral influenza vaccinations in pregnancy for improving maternal, neonatal and infant health outcomes	Salam, , RA, Das, JK, Dojo Soeandy, C, Lassi, ZS; Bhutta, ZA	2015
Immunosuppressive treatment for proliferative lupus nephritis	Tunnicliffe, , DJ, Palmer, SC, Henderson, L, Masson, P, Craig, JC, Tong, A, Singh-Grewal, D, Flanc, RS, Roberts, MA, Webster, AC; Strippoli, GFM	2018
Hydroxyethyl starch (HES) versus other fluid therapies: effects on kidney function	Mutter, , TC, Ruth, CA; Dart, AB	2013
Inpatient versus other settings for detoxification for opioid dependence	Day, , E, Ison, J; Strang, J	2005
Individual psychodynamic psychotherapy and psychoanalysis for schizophrenia and severe mental illness	Malmberg, , L, Fenton, M; Rathbone, J	2001
Inspiratory muscle training for the recovery of function after stroke	Xiao, , Y, Luo, M, Wang, J; Luo, H	2012
Immunoglobulin prophylaxis in hematological malignancies and hematopoietic stem cell transplantation	Raanani, , P, Gafter-Gvili, A, Paul, M, Ben-Bassat, I, Leibovici, L; Shpilberg, O	2008
Hydrogel dressings for healing diabetic foot ulcers	Dumville, , JC, O'Meara, S, Deshpande, S; Speak, K	2013
Inhaled mannitol for cystic fibrosis	Nevitt, , SJ, Thornton, J, Murray, CS; Dwyer, T	2018
Imiquimod for anogenital warts in non-immunocompromised adults	Grillo-Ardila, , CF, Angel-Müller, E, Salazar-Díaz, LC, Gaitán, HG, Ruiz-Parra, AI; Lethaby, A	2014
Influenza vaccination for healthcare workers who care for people aged 60 or older living in long-term care institutions	Thomas, , RE, Jefferson, T; Lasserson, TJ	2016
Hyperbaric oxygen therapy for acute ischaemic stroke	Bennett, , MH, Weibel, S, Wasiak, J, Schnabel, A, French, C; Kranke, P	2014
Infant pacifiers for reduction in risk of sudden infant death syndrome	Psaila, , K, Foster, JP, Pulbrook, N; Jeffery, HE	2017
Indoor residual spraying for preventing malaria in communities using insecticide-treated nets	Choi, , L, Pryce, J; Garner, P	2019
Hypofractionation for clinically localized prostate cancer	Hickey, , BE, James, ML, Daly, T, Soh, FY; Jeffery, M	2019

Immunotherapy for metastatic renal cell carcinoma	Unverzagt, , S, Moldenhauer, I, Nothacker, M, Roßmeißl, D, Hadjinicolaou, AV, Peinemann, F, Greco, F; Seliger, B	2017
Informant Questionnaire on Cognitive Decline in the Elderly (IQCODE) for the early diagnosis of dementia across a variety of healthcare settings	Harrison, , JK, Stott, DJ, McShane, R, Noel-Storr, AH, Swann-Price, RS; Quinn, TJ	2016
Huperzine A for vascular dementia	Hao, , Z, Liu, M, Liu, Z; Lu, D	2009
Infant formulas containing hydrolysed protein for prevention of allergic disease	Osborn, , DA, Sinn, JKH; Jones, LJ	2018
Ibuprofen and/or paracetamol (acetaminophen) for pain relief after surgical removal of lower wisdom teeth	Bailey, , E, Worthington, HV, van Wijk, A, Yates, JM, Coulthard, P; Afzal, Z	2013
Hyperbaric oxygen therapy for the adjunctive treatment of traumatic brain injury	Bennett, , MH, Trytko, B; Jonker, B	2012
Infant position in neonates receiving mechanical ventilation	Rivas-Fernandez, , M, Roqué i Figuls, M, Diez-Izquierdo, A, Escribano, J; Balaguer, A	2016
Inhaled corticosteroids in children with persistent asthma: dose-response effects on growth	Pruteanu, , AI, Chauhan, BF, Zhang, L, Prietsch, SOM; Ducharme, FM	2014
Inhaled steroids for acute asthma following emergency department discharge	Edmonds, , ML, Milan, SJ, Brenner, BE, Camargo Jr, CA; Rowe, BH	2012
Hyperbaric oxygen therapy for treating acute surgical and traumatic wounds	Eskes, , A, Vermeulen, H, Lucas, C; Ubbink, DT	2013
Human albumin for intradialytic hypotension in haemodialysis patients	Fortin, , PM, Bassett, K; Musini, VM	2010
Indigenous healthcare worker involvement for Indigenous adults and children with asthma	Chang, , AB, Taylor, B, Masters, IB, Laifoo, Y; Brown, ADH	2010
Incentives for preventing smoking in children and adolescents	Hefler, , M, Liberato, SC; Thomas, DP	2017
Immediate referral to colposcopy versus cytological surveillance for minor cervical cytological abnormalities in the absence of HPV test	Kyrgiou, , M, Kalliala, IEJ, Mitra, A, Fotopoulou, C, Ghaem-Maghami, S, Martin-Hirsch, PPL, Cruickshank, M, Arbyn, M; Paraskevaidis, E	2017
Ibuprofen for the treatment of patent ductus arteriosus in preterm or low birth weight (or both) infants	Ohlsson, , A, Walia, R; Shah, SS	2018
Implantable defibrillators versus medical therapy for cardiac channelopathies	McNamara, , DA, Goldberger, JJ, Berendsen, MA; Huffman, MD	2015
Immunosuppressive agents for myasthenia gravis	Hart, , IK, Sathasivam, S; Sharshar, T	2007
Immersion in water during labour and birth	Cluett, , ER, Burns, E; Cuthbert, A	2018
Information provision for stroke patients and their caregivers	Forster, , A, Brown, L, Smith, J, House, A, Knapp, P, Wright, JJ; Young, J	2012
Hyperbaric oxygenation for tumour sensitisation to radiotherapy	Bennett, , MH, Feldmeier, J, Smee, R; Milross, C	2018
Immediate versus deferred zidovudine (AZT) in asymptomatic or mildly symptomatic HIV infected adults	Darbyshire, , J, Foulkes, M, Peto, R, Duncan, W, Babiker, A, Collins, R, Hughes, M, Peto, TEA; Walker, SA	2000
Inhaled antibiotics for pulmonary exacerbations in cystic fibrosis	Smith, , S, Rowbotham, NJ; Charbek, E	2018
Hyperbaric oxygen as an adjuvant treatment for malignant otitis externa	Phillips, , JS; Jones, SEM	2013
Informant Questionnaire on Cognitive Decline in the Elderly (IQCODE) for the diagnosis of dementia within a secondary care setting	Harrison, , JK, Fearon, P, Noel-Storr, AH, McShane, R, Stott, DJ; Quinn, TJ	2015
Informant Questionnaire on Cognitive Decline in the Elderly (IQCODE) for the diagnosis of dementia within a general practice (primary care) setting	Harrison, , JK, Fearon, P, Noel-Storr, AH, McShane, R, Stott, DJ; Quinn, TJ	2014
Humanized PA14 (a monoclonal CCR5 antibody) for treatment of people with HIV infection	Li, , L, Tian, JH, Yang, K, Zhang, P; Jia, WQ	2014
Industry sponsorship and research outcome	Lundh, , A, Lexchin, J, Mintzes, B, Schroll, JB; Bero, L	2017
Immunosuppressive and cytotoxic therapy for pulmonary sarcoidosis	Paramothayan, , NS, Lasserson, TJ; Walters, EH	2006
Inositol in preterm infants at risk for or having respiratory distress syndrome	Howlett, , A, Ohlsson, A; Plakkal, N	2019
Inhaled anti-pseudomonal antibiotics for long-term therapy in cystic fibrosis	Smith, , S, Rowbotham, NJ; Regan, KH	2018
Individual participant data meta-analyses compared with meta-analyses based on aggregate data	Tudur Smith, , C, Marcucci, M, Nolan, SJ, Iorio, A, Sudell, M, Riley, R, Rovers, MM; Williamson, PR	2016
Immediate antiepileptic drug treatment, versus placebo, deferred, or no treatment for first unprovoked seizure	Leone, , MA, Giussani, G, Nevitt, SJ, Marson, AG; Beghi, E	2016
Identification of children in the first four years of life for early treatment for otitis media with effusion	Simpson, , SA, Thomas, CL, van der Linden, M, MacMillan, H, van der Wouden, JC; Butler, CC	2007
Huperzine A for mild cognitive impairment	Yue, , J, Dong, BR, Lin, X, Yang, M, Wu, HM; Wu, T	2012
Influenza vaccine for chronic obstructive pulmonary disease (COPD)	Kopsaftis, , Z, Wood-Baker, R; Poole, P	2018
Increasing antipsychotic dose for non response in schizophrenia	Samara, , MT, Klupp, E, Helfer, B, Rothe, PH, Schneider-Thoma, J; Leucht, S	2018
Inhaled hyperosmolar agents for bronchiectasis	Hart, , A, Sugumar, K, Milan, SJ, Fowler, SJ; Crossingham, I	2014
Hormone therapy for sexual function in perimenopausal and postmenopausal women	Nastri, , CO, Lara, LA, Ferriani, RA, Rosa-e-Silva, ACJS, Figueiredo, JBP; Martins, WP	2013
Inferior turbinate surgery for nasal obstruction in allergic rhinitis after failed medical treatment	Jose, , J; Coatesworth, AP	2010

Hormone replacement therapy for women previously treated for endometrial cancer	Edey, , KA, Rundle, S; Hickey, M	2018
Immunosuppressive drug therapy for preventing rejection following lung transplantation in cystic fibrosis	Saldanha, , IJ, Akinyede, O; Robinson, KA	2018
Increased police patrols for preventing alcohol-impaired driving	Goss, , CW, Van Bramer, LD, Gliner, JA, Porter, TR, Roberts, IG; DiGuiseppi, C	2008
Informant Questionnaire on Cognitive Decline in the Elderly (IQCODE) for the diagnosis of dementia within community dwelling populations	Quinn, , TJ, Fearon, P, Noel-Storr, AH, Young, C, McShane, R; Stott, DJ	2014
Immunonutrition as an adjuvant therapy for burns	Tan, , HB, Danilla, S, Murray, A, Serra, R, El Dib, R, Henderson, TOW; Wasiak, J	2014
Immunoglobulins, vaccines or interferon for preventing cytomegalovirus disease in solid organ transplant recipients	Hodson, , EM, Jones, CA, Strippoli, GFM, Webster, AC; Craig, JC	2007
Impact of medication reconciliation for improving transitions of care	Redmond, , P, Grimes, TC, McDonnell, R, Boland, F, Hughes, C; Fahey, T	2018
Insecticide-treated nets for preventing malaria	Pryce, , J, Richardson, M; Lengeler, C	2018
Inhaled corticosteroids versus long-acting beta2-agonists for chronic obstructive pulmonary disease	Spencer, , S, Karner, C, Cates, CJ; Evans, DJ	2011
Impact of public release of performance data on the behaviour of healthcare consumers and providers	Metcalfe, , D, Rios Diaz, AJ, Olufajo, OA, Massa, MS, Ketelaar, NABM, Flottorp, SA; Perry, DC	2018
Immunosuppressive T-cell antibody induction for heart transplant recipients	Penninga, , L, Møller, CH, Gustafsson, F, Gluud, C; Steinbrüchel, DA	2013
Incentives for increasing prenatal care use by women in order to improve maternal and neonatal outcomes	Till, , SR, Everetts, D; Haas, DM	2015
Inhaled nitric oxide for acute respiratory distress syndrome (ARDS) in children and adults	Gebistorf, , F, Karam, O, Wetterslev, J; Afshari, A	2016
Hospitalisation in short-stay units for adults with internal medicine diseases and conditions	Strøm, , C, Stefansson, JS, Fabritius, ML, Rasmussen, LS, Schmidt, TA; Jakobsen, JC	2018
Inositol for subfertile women with polycystic ovary syndrome	Showell, , MG, Mackenzie-Proctor, R, Jordan, V, Hodgson, R; Farquhar, C	2018
Individual-, family-, and school-level interventions targeting multiple risk behaviours in young people	MacArthur, , G, Caldwell, DM, Redmore, J, Watkins, SH, Kipping, R, White, J, Chittleborough, C, Langford, R, Er, V, Lingam, R, Pasch, K, Gunnell, D, Hickman, M; Campbell, R	2018
Hysteroscopy for treating subfertility associated with suspected major uterine cavity abnormalities	Bosteels, , J, van Wessel, S, Weyers, S, Broekmans, FJ, D'Hooghe, TM, Bongers, MY; Mol, BWJ	2018
Hydroxyurea for reducing blood transfusion in non-transfusion dependent beta thalassaemias	Foong, , WC, Ho, JJ, Loh, CK; Viprakasit, V	2016
Housing improvements for health and associated socio-economic outcomes	Thomson, , H, Thomas, S, Sellstrom, E; Petticrew, M	2013
Increased consumption of fruit and vegetables for the primary prevention of cardiovascular diseases	Hartley, , L, Igbinedion, E, Holmes, J, Flowers, N, Thorogood, M, Clarke, A, Stranges, S, Hooper, L; Rees, K	2013
Inhaled corticosteroids for non-specific chronic cough in children	Tomerak, , AAT, McGlashan, J, Lakhanpaul, M, Vyas, HHV; McKean, MC	2005
House dust mite reduction and avoidance measures for treating eczema	Nankervis, , H, Pynn, EV, Boyle, RJ, Rushton, L, Williams, HC, Hewson, DM; Platts-Mills, T	2015
Immunonutrition for patients undergoing surgery for head and neck cancer	Howes, , N, Atkinson, C, Thomas, S; Lewis, SJ	2018
Insulin and glucose-lowering agents for treating people with diabetes and chronic kidney disease	Lo, , C, Toyama, T, Wang, Y, Lin, J, Hirakawa, Y, Jun, M, Cass, A, Hawley, CM, Pilmore, H, Badve, SV, Perkovic, V; Zoungas, S	2018
Inhaled corticosteroids for bronchiectasis	Kapur, , N, Petsky, HL, Bell, S, Kolbe, J; Chang, AB	2018
Incentives for smoking cessation	Notley, , C, Gentry, S, Livingstone-Banks, J, Bauld, L, Perera, R; Hartmann-Boyce, J	2019
Hysterectomy with opportunistic salpingectomy versus hysterectomy alone	van Lieshout, , LAM, Steenbeek, MP, De Hullu, JA, Vos, MC, Houterman, S, Wilkinson, J; Piek, JMJ	2019
Ibuprofen for Alzheimer's disease	Tabet, , N; Feldman, H	2003
Information or education interventions for adult intensive care unit (ICU) patients and their carers	Lewis, , SR, Pritchard, MW, Schofield-Robinson, OJ, Evans, DJW, Alderson, P; Smith, AF	2018
Intermittent drug techniques for schizophrenia	Sampson, , S, Mansour, M, Maayan, N, Soares-Weiser, K; Adams, CE	2013
Interventions for preventing unintended pregnancies among adolescents	Oringanje, , C, Meremikwu, MM, Eko, H, Esu, E, Meremikwu, A; Ehiri, JE	2016
Interventions for acute otitis externa	Kaushik, , V, Malik, T; Saeed, SR	2010
Interventions for increasing the proportion of health professionals practising in rural and other underserved areas	Grobler, , L, Marais, BJ; Mabunda, S	2015
Intermediate acting versus long acting insulin for type 1 diabetes mellitus	Vardi, , M, Jacobson, E, Nini, A; Bitterman, H	2008
Interventions for improving sit-to-stand ability following stroke	Pollock, , A, Gray, C, Culham, E, Durward, BR; Langhorne, P	2014
Interferon Beta for Primary Progressive Multiple Sclerosis	Rojas, , JI, Romano, M, Ciapponi, A, Patrucco, L; Cristiano, E	2010
Interventions for encouraging sexual behaviours intended to prevent cervical cancer	Shepherd, , JP, Frampton, GK; Harris, P	2011
Interventions for educating children who are at risk of asthma-related emergency department attendance	Boyd, , M, Lasserson, TJ, McKean, MC, Gibson, PG, Ducharme, FM; Haby, M	2009
Interventions for latent autoimmune diabetes (LADA) in adults	Brophy, , S, Davies, H, Mannan, S, Brunt, H; Williams, R	2011

Integrated versus non-integrated orbital implants for treating anophthalmic sockets	Schellini, , S, El Dib, R, Silva, LRE, Farat, JG, Zhang, Y; Jorge, EC	2016
Interventions for preventing delirium in older people in institutional long-term care	Woodhouse, , R, Burton, JK, Rana, N, Pang, YL, Lister, JE; Siddiqi, N	2019
Interventions for prevention of giant retinal tear in the fellow eye	Ang, , GS, Townend, J; Lois, N	2012
Insulin-sensitising drugs (metformin, rosiglitazone, pioglitazone, D-chiro-inositol) for women with polycystic ovary syndrome, oligo amenorrhoea and subfertility	Morley, , LC, Tang, T, Yasmin, E, Norman, RJ; Balen, AH	2017
Interventions for prevention of neonatal hyperglycemia in very low birth weight infants	Sinclair, , JC, Bottino, M; Cowett, RM	2011
Interventions for preventing and managing advanced liver disease in cystic fibrosis	Palaniappan, , SK, Than, NN, Thein, AW, Moe, S; van Mourik, I	2017
Interventions for cutaneous molluscum contagiosum	van der Wouden, , JC, van der Sande, R, Kruithof, EJ, Sollie, A, van Suijlekom-Smit, LWA; Koning, S	2017
Interventions for mycosis fungoides	Weberschock, , T, Strametz, R, Lorenz, M, Röllig, C, Bunch, C, Bauer, A; Schmitt, J	2012
Insulin-sensitising drugs versus the combined oral contraceptive pill for hirsutism, acne and risk of diabetes, cardiovascular disease, and endometrial cancer in polycystic ovary syndrome	Costello, , MF, Shrestha, B, Eden, J, Johnson, N; Moran, LJ	2007
Intermittent versus daily therapy for treating tuberculosis in children	Bose, , A, Kalita, S, Rose, W; Tharyan, P	2014
Interventions for chronic blepharitis	Lindsley, , K, Matsumura, S, Hatef, E; Akpek, EK	2012
Intensive glucose control versus conventional glucose control for type 1 diabetes mellitus	Fullerton, , B, Jeitler, K, Seitz, M, Horvath, K, Berghold, A; Siebenhofer, A	2014
Interventions for dysphagia in oesophageal cancer	Dai, , Y, Li, C, Xie, Y, Liu, X, Zhang, J, Zhou, J, Pan, X; Yang, S	2014
Interventions for fatigue in Parkinson's disease	Elbers, , RG, Verhoef, J, van Wegen, EEH, Berendse, HW; Kwakkel, G	2015
Interventions for managing relapse of the lower front teeth after orthodontic treatment	Yu, , Y, Sun, J, Lai, W, Wu, T, Koshy, S; Shi, Z	2013
Integrating prevention of mother-to-child HIV transmission (PMTCT) programmes with other health services for preventing HIV infection and improving HIV outcomes in developing countries	Tudor Car, , L, van-Velthoven, MHMMT, Brusamento, S, Elmoniry, H, Car, J, Majeed, A; Atun, R	2011
Interventions for melanoma in situ, including lentigo maligna	Tzellos, , T, Kyrgidis, A, Mocellin, S, Chan, AW, Pilati, P; Apalla, Z	2014
Interventions for impetigo	Koning, , S, van der Sande, R, Verhagen, AP, van Suijlekom-Smit, LWA, Morris, AD, Butler, CC, Berger, M; van der Wouden, JC	2012
Interventions for disorders of eye movement in patients with stroke	Pollock, , A, Hazelton, C, Henderson, CA, Angilley, J, Dhillon, B, Langhorne, P, Livingstone, K, Munro, FA, Orr, H, Rowe, FJ; Shahani, U	2011
Interventions for preventing critical illness polyneuropathy and critical illness myopathy	Hermans, , G, De Jonghe, B, Bruyninckx, F; Van den Berghe, G	2014
Interventions for preventing voice disorders in adults	Ruotsalainen, , JH, Sellman, J, Lehto, L, Isotalo, LK; Verbeek, JH	2007
Interventions for helping people adhere to compression treatments for venous leg ulceration	Weller, , CD, Buchbinder, R; Johnston, RV	2016
Internal versus external tocodynamometry during induced or augmented labour	Bakker, , JJH, Janssen, PF, van Halem, K, van der Goes, BY, Papatsonis, DNM, van der Post, JAM; Mol, BWJ	2013
Intermittent inhaled corticosteroid therapy versus placebo for persistent asthma in children and adults	Chong, , J, Haran, C, Chauhan, BF; Asher, I	2015
Interventions for intermittent exotropia	Hatt, , SR; Gnanaraj, L	2013
Intermittent oral iron supplementation during pregnancy	Peña-Rosas, , JP, De-Regil, LM, Gomez Malave, H, Flores-Urrutia, MC; Dowswell, T	2015
Interventions for increasing ankle range of motion in patients with neuromuscular disease	Rose, , KJ, Burns, J, Wheeler, DM; North, KN	2010
Interventions for pityriasis rosea	Chuh, , AAT, Dofitas, BL, Comisel, G, Reveiz, L, Sharma, V, Garner, SE; Chu, FKM	2007
Interferon alpha for chronic hepatitis D	Abbas, , Z, Khan, MA, Salih, M; Jafri, W	2011
Interventions for promoting reintegration and reducing harmful behaviour and lifestyles in street-connected children and young people	Coren, , E, Hossain, R, Pardo Pardo, J; Bakker, B	2016
Interventions for dysarthria due to stroke and other adult-acquired, non-progressive brain injury	Mitchell, , C, Bowen, A, Tyson, S, Butterfint, Z; Conroy, P	2017
Interventions for pemphigus vulgaris and pemphigus foliaceus	Martin, , LK, Agero, AL, Werth, V, Villanueva, E, Segall, J; Murrell, DF	2009
Interventions for preventing abuse in the elderly	Baker, , PRA, Francis, DP, Hairi, NN, Othman, S; Choo, WY	2016
Interventions for improving adherence to treatment recommendations in people with type 2 diabetes mellitus	Vermeire, , EIJJ, Wens, J, Van Royen, P, Biot, Y, Hearnshaw, H; Lindenmeyer, A	2005
Interventions for improving coverage of childhood immunisation in low- and middle-income countries	Oyo-Ita, , A, Wiysonge, CS, Oringanje, C, Nwachukwu, CE, Oduwole, O; Meremikwu, MM	2016
Interventions for iatrogenic inferior alveolar and lingual nerve injury	Coulthard, , P, Kushnerev, E, Yates, JM, Walsh, T, Patel, N, Bailey, E; Renton, TF	2014
Interventions for atrophic rhinitis	Mishra, , A, Kawatra, R; Gola, M	2012

Interactive computer-based interventions for sexual health promotion	Bailey, , JV, Murray, E, Rait, G, Mercer, CH, Morris, RW, Peacock, R, Cassell, J; Nazareth, I	2010
Interventions for dysphagia in long-term, progressive muscle disease	Jones, , K, Pitceathly, RDS, Rose, MR, McGowan, S, Hill, M, Badrising, UA; Hughes, T	2016
Interventions for clinical and subclinical hypothyroidism pre-pregnancy and during pregnancy	Reid, , SM, Middleton, P, Cossich, MC, Crowther, CA; Bain, E	2013
Interactive computer-based interventions for weight loss or weight maintenance in overweight or obese people	Wieland, , LS, Falzon, L, Sciamanna, CN, Trudeau, KJ, Brodney Folse, S, Schwartz, JE; Davidson, KW	2012
Interventions for lowering plasma homocysteine levels in dialysis patients	Nigwekar, , SU, Kang, A, Zoungas, S, Cass, A, Gallagher, MP, Kulshrestha, S, Navaneethan, SD, Perkovic, V, Strippoli, GFM; Jardine, MJ	2016
Intermittent versus continuous androgen suppression for prostatic cancer	De Conti, , P, Atallah, ÁN, Arruda, HO, Soares, BGO, El Dib, RP; Wilt, TJ	2007
Interventions for prevention and treatment of vulvovaginal candidiasis in women with HIV infection	Ray, , A, Ray, S, George, AT; Swaminathan, N	2011
Interventions for congenital talipes equinovarus (clubfoot)	Gray, , K, Pacey, V, Gibbons, P, Little, D; Burns, J	2014
Interventions for age-related visual problems in patients with stroke	Pollock, , A, Hazelton, C, Henderson, CA, Angilley, J, Dhillon, B, Langhorne, P, Livingstone, K, Munro, FA, Orr, H, Rowe, FJ; Shahani, U	2012
Interventions for preventing silent cerebral infarcts in people with sickle cell disease	Estcourt, , LJ, Fortin, PM, Hopewell, S, Trivella, M, Doree, C; Abboud, MR	2017
Interventions for infantile esotropia	Elliott, , S; Shafiq, A	2013
Interventions for preoperative smoking cessation	Thomsen, , T, Villebro, N; Møller, AM	2014
Interventions for bullous pemphigoid	Kirtschig, , G, Middleton, P, Bennett, C, Murrell, DF, Wojnarowska, F; Khumalo, NP	2010
Interventions for preventing bone disease in kidney transplant recipients	Palmer, , SC, McGregor, DO; Strippoli, GFM	2007
Interventions for erosive lichen planus affecting mucosal sites	Cheng, , S, Kirtschig, G, Cooper, S, Thornhill, M, Leonardi-Bee, J; Murphy, R	2012
Interprofessional education: effects on professional practice and healthcare outcomes	Reeves, , S, Perrier, L, Goldman, J, Freeth, D; Zwarenstein, M	2013
Interventions for basal cell carcinoma of the skin	Bath-Hextall, , FJ, Perkins, W, Bong, J; Williams, HC	2007
Interventions for central serous chorioretinopathy: a network meta-analysis	Salehi, , M, Wenick, AS, Law, HA, Evans, JR; Gehlbach, P	2015
Interventions for drug-using offenders with co-occurring mental illness	Perry, , AE, Neilson, M, Martyn-St James, M, Glanville, JM, Woodhouse, R, Godfrey, C; Hewitt, C	2015
Interventions for promoting participation in shared decision-making for children with cancer	Coyne, , I, O'Mathúna, DP, Gibson, F, Shields, L, Leclercq, E; Sheaf, G	2016
Interventions for non-metastatic squamous cell carcinoma of the skin	Lansbury, , L, Leonardi-Bee, J, Perkins, W, Goodacre, T, Tweed, JA; Bath-Hextall, FJ	2010
Interventions for preventing depression after stroke	Hackett, , ML, Anderson, CS, House, A; Halteh, C	2008
Interventions for nail psoriasis	de Vries, , ACQ, Bogaards, NA, Hooft, L, Velema, M, Pasch, M, Lebwohl, M; Spuls, PI	2013
Interventions for men and women with their first episode of genital herpes	Heslop, , R, Roberts, H, Flower, D; Jordan, V	2016
Interventions for cutaneous Bowen's disease	Bath-Hextall, , FJ, Matin, RN, Wilkinson, D; Leonardi-Bee, J	2013
Interventions for preventing relapse and recurrence of a depressive disorder in children and adolescents	Cox, , GR, Fisher, CA, De Silva, S, Phelan, M, Akinwale, OP, Simmons, MB; Hetrick, SE	2012
Interventions for preventing or reducing domestic violence against pregnant women	Jahanfar, , S, Howard, LM; Medley, N	2014
Interventions for acute auricular haematoma	Jones, , SEM; Mahendran, S	2004
Interventions for cellulitis and erysipelas	Kilburn, , SA, Featherstone, P, Higgins, B; Brindle, R	2010
Interventions for preventing and treating cardiac complications in Duchenne and Becker muscular dystrophy and X-linked dilated cardiomyopathy	Bourke, , JP, Bueser, T; Quinlivan, R	2018
Interventions for female pattern hair loss	van Zuuren, , EJ, Fedorowicz, Z; Schoones, J	2016
Interventions for ingrowing toenails	Eekhof, , JAH, Van Wijk, B, Knuistingh Neven, A; van der Wouden, JC	2012
Interventions for involutional lower lid entropion	Boboridis, , KG; Bunce, C	2011
Interventions for improving outcomes in patients with multimorbidity in primary care and community settings	Smith, , SM, Wallace, E, O'Dowd, T; Fortin, M	2016
Interventions for improving patients' trust in doctors and groups of doctors	Rolfe, , A, Cash-Gibson, L, Car, J, Sheikh, A; McKinstry, B	2014
Interventions for melasma	Rajaratnam, , R, Halpern, J, Salim, A; Emmett, C	2010
Interventions for minimal change disease in adults with nephrotic syndrome	Palmer, , SC, Nand, K; Strippoli, GFM	2008
Interventions for acne scars	Abdel Hay, , R, Shalaby, K, Zaher, H, Hafez, V, Chi, CC, Dimitri, S, Nabhan, AF; Layton, AM	2016

Interventions for preventing infection in nephrotic syndrome	Wu, , HM, Tang, JL, Cao, L, Sha, ZH; Li, Y	2012
Interventions for promoting physical activity in people with cystic fibrosis	Cox, , NS, Alison, JA; Holland, AE	2013
Interventions for prophylaxis of hepatic veno-occlusive disease in people undergoing haematopoietic stem cell transplantation	Cheuk, , DKL, Chiang, AKS, Ha, SY; Chan, GCF	2015
Interventions for promoting participation in shared decision-making for children and adolescents with cystic fibrosis	Malone, , H, Biggar, S, Javadpour, S, Edworthy, Z, Sheaf, G; Coyne, I	2019
Interventions for chronic palmoplantar pustulosis	Chalmers, , R, Hollis, S, Leonardi-Bee, J, Griffiths, CEM; Marsland Bsc MRCP, A	2006
Interventions for primary prevention of suicide in university and other post-secondary educational settings	Harrod, , CS, Goss, CW, Stallones, L; DiGuiseppi, C	2014
Interventions for covert bacteriuria in children	Fitzgerald, , A, Mori, R; Lakhanpaul, M	2012
Intermittent iron supplementation for improving nutrition and development in children under 12 years of age	De-Regil, , LM, Jefferds, MED, Sylvetsky, AC; Dowswell, T	2011
Interventions for actinic keratoses	Gupta, , AK, Paquet, M, Villanueva, E; Brintnell, W	2012
Interventions for alopecia areata	Delamere, , FM, Sladden, MJ, Dobbins, HM; Leonardi-Bee, J	2008
Interventions for improving the appropriate use of imaging in people with musculoskeletal conditions	French, , SD, Green, S, Buchbinder, R; Barnes, H	2010
Interleukin-2 as an adjunct to antiretroviral therapy for HIV-positive adults	Onwumeh, , J, Okwundu, CI; Kredo, T	2017
Intensive case management for severe mental illness	Dieterich, , M, Irving, CB, Bergman, H, Khokhar, MA, Park, B; Marshall, M	2017
Interventions for obtaining and maintaining employment in adults with severe mental illness, a network meta-analysis	Suijkerbuijk, , YB, Schaafsma, FG, van Mechelen, JC, Ojajärvi, A, Corbière, M; Anema, JR	2017
Interferon in relapsing-remitting multiple sclerosis	Rice, , GPA, Incorvaia, B, Munari, LM, Ebers, G, Polman, C, D'Amico, R, Parmelli, E; Filippini, G	2001
Interventions for leg cramps in pregnancy	Zhou, , K, West, HM, Zhang, J, Xu, L; Li, W	2015
Interventions for preventing high altitude illness: Part 2. Less commonly-used drugs	Gonzalez Garay, , AG, Molano Franco, D, Nieto Estrada, VH, Martí-Carvajal, AJ; Arevalo-Rodriguez, I	2018
Interventions for Mooren's ulcer	Alhassan, , MB, Rabiu, M; Agbabiaka, IO	2014
Interventions for deliberately altering blood pressure in acute stroke	Bath, , PMW; Krishnan, K	2014
Interventions based on the Theory of Mind cognitive model for autism spectrum disorder (ASD)	Fletcher-Watson, , S, McConnell, F, Manola, E; McConachie, H	2014
Interleukin 2 receptor antagonists for kidney transplant recipients	Webster, , AC, Ruster, LP, McGee, RG, Matheson, SL, Higgins, GY, Willis, NS, Chapman, JR; Craig, JC	2010
Intermittent preventive treatment for malaria in children living in areas with seasonal transmission	Meremikwu, , MM, Donegan, S, Sinclair, D, Esu, E; Oringanje, C	2012
Interventions for prevention of post-operative recurrence of Crohn's disease	Doherty, , G, Bennett, G, Patil, S, Cheifetz, A; Moss, AC	2009
Interventions for enhancing consumers' online health literacy	Car, , J, Lang, B, Colledge, A, Ung, C; Majeed, A	2011
Insulin monotherapy compared with the addition of oral glucose-lowering agents to insulin for people with type 2 diabetes already on insulin therapy and inadequate glycaemic control	Vos, , RC, van Avendonk, MJP, Jansen, H, Goudswaard, AN, van den Donk, M, Gorter, K, Kerssen, A; Rutten, GEHM	2016
Interventions for preventing infectious complications in haemodialysis patients with central venous catheters	McCann, , M; Moore, ZEH	2010
Interventions for preventing lower limb soft-tissue running injuries	Yeung, , SS, Yeung, EW; Gillespie, LD	2011
Interventions for providers to promote a patient-centred approach in clinical consultations	Dwamena, , F, Holmes-Rovner, M, Gaulden, CM, Jorgenson, S, Sadigh, G, Sikorskii, A, Lewin, S, Smith, RC, Coffey, J, Olomu, A; Beasley, M	2012
Interventions for anal canal intraepithelial neoplasia	Macaya, , A, Muñoz-Santos, C, Balaguer, A; Barberà, MJ	2012
Interventions for preventing delirium in hospitalised non-ICU patients	Siddiqi, , N, Harrison, JK, Clegg, A, Teale, EA, Young, J, Taylor, J; Simpkins, SA	2016
Interventions for erythropoietin-resistant anaemia in dialysis patients	Badve, , SV, Beller, EM, Cass, A, Francis, DP, Hawley, C, Macdougall, IC, Perkovic, V; Johnson, DW	2013
Interventions for primary (intrinsic) tracheomalacia in children	Goyal, , V, Masters, IB; Chang, AB	2012
Interventions for orbital lymphangioma	Patel, , SR, Rosenberg, JB; Barmettler, A	2019
Interventions for lowering plasma homocysteine levels in kidney transplant recipients	Kang, , A, Nigwekar, SU, Perkovic, V, Kulshrestha, S, Zoungas, S, Navaneethan, SD, Cass, A, Gallagher, MP, Ninomiya, T, Strippoli, GFM; Jardine, MJ	2015
Interventions for preventing the progression of autosomal dominant polycystic kidney disease	Bolignano, , D, Palmer, SC, Ruospo, M, Zoccali, C, Craig, JC; Strippoli, GFM	2015
Interventions for cleaning dentures in adults	de Souza, , RF, de Freitas Oliveira Paranhos, H, Lovato da Silva, CH, Abu-Naba'a, L, Fedorowicz, Z; Gurgan, CA	2009
Interferon-alpha for maintenance of follicular lymphoma	Baldo, , P, Rupolo, M, Compagnoni, A, Lazzarini, R, Bearz, A, Cannizzaro, R, Spazzapan, S, Truccolo, I; Moja, L	2010
Interventions for preventing recurrent urinary tract infection during pregnancy	Schneeberger, , C, Geerlings, SE, Middleton, P; Crowther, CA	2015

Interventions for improving palliative care for older people living in nursing care homes	Hall, , S, Kolliakou, A, Petkova, H, Froggatt, K; Higginson, IJ	2011
Internal dressings for healing perianal abscess cavities	Smith, , SR, Newton, K, Smith, JA, Dumville, JC, Iheozor-Ejiofor, Z, Pearce, LE, Barrow, PJ, Hancock, L; Hill, J	2016
Intermittent versus daily inhaled corticosteroids for persistent asthma in children and adults	Chauhan, , BF, Chartrand, C; Ducharme, FM	2013
Interventions during pregnancy to prevent preterm birth: an overview of Cochrane systematic reviews	Medley, , N, Vogel, JP, Care, A; Alfirevic, Z	2018
Interventions for promoting booster seat use in four to eight year olds travelling in motor vehicles	Ehiri, , JE, Ejere, HOD, Magnussen, L, Emusu, D, King, W; Osberg, SJ	2006
Interferon for interferon nonresponding and relapsing patients with chronic hepatitis C	Koretz, , RL, Pleguezuelo, M, Arvaniti, V, Barrera Baena, P, Ciria, R, Gurusamy, KS, Davidson, BR; Burroughs, AK	2013
Interventions for chronic kidney disease in people with sickle cell disease	Roy, , NBA, Fortin, PM, Bull, KR, Doree, C, Trivella, M, Hopewell, S; Estcourt, LJ	2017
Interventions for erythema nodosum leprosum	Van Veen, , NHJ, Lockwood, DNJ, van Brakel, WH, Ramirez Jr, J; Richardus, JH	2009
Interconception care for women with a history of gestational diabetes for improving maternal and infant outcomes	Tieu, , J, Shepherd, E, Middleton, P; Crowther, CA	2017
Interventions for drooling in children with cerebral palsy	Walshe, , M, Smith, M; Pennington, L	2012
Interventions for prevention of herpes simplex labialis (cold sores on the lips)	Chi, , CC, Wang, SH, Delamere, FM, Wojnarowska, F, Peters, MC; Kanjirath, PP	2015
Interventions for hyperthyroidism pre-pregnancy and during pregnancy	Earl, , R, Crowther, CA; Middleton, P	2013
Interventions for preventing oral mucositis for patients with cancer receiving treatment	Worthington, , HV, Clarkson, JE, Bryan, G, Furness, S, Glenny, AM, Littlewood, A, McCabe, MG, Meyer, S; Khalid, T	2011
Interventions for HIV-associated nephropathy	Yahaya, , I, Uthman, OA; Uthman, MMB	2013
Interventions for improving community ambulation in individuals with stroke	Barclay, , RE, Stevenson, TJ, Poluha, W, Ripat, J, Nett, C; Srikesavan, CS	2015
Interventions for improving communication with children and adolescents about their cancer	Ranmal, , R, Prictor, M; Scott, JT	2008
Interventions for motor apraxia following stroke	West, , C, Bowen, A, Hesketh, A; Vail, A	2008
Internet-based cognitive and behavioural therapies for post-traumatic stress disorder (PTSD) in adults	Lewis, , C, Roberts, NP, Bethell, A, Robertson, L; Bisson, JI	2018
Interventions aimed at communities to inform and/or educate about early childhood vaccination	Saeterdal, , I, Lewin, S, Austvoll-Dahlgren, A, Glenton, C; Munabi-Babigumira, S	2014
Interventions for morphea	Albuquerque, , JVD, Andriolo, BNG, Vasconcellos, MRA, Civile, VT, Lyddiatt, A; Trevisani, VFM	2019
Interventions for preventing intensive care unit delirium in adults	Herling, , SF, Greve, IE, Vasilevskis, EE, Egerod, I, Bekker Mortensen, C, Møller, AM, Svenningsen, H; Thomsen, T	2018
Interventions for non-oliguric hyperkalaemia in preterm neonates	Vemgal, , P; Ohlsson, A	2012
Interventions for preventing venous thromboembolism in adults undergoing knee arthroscopy	Ramos, , J, Perrotta, C, Badariotti, G; Berenstein, G	2008
Interventions for mucous membrane pemphigoid and epidermolysis bullosa acquisita	Kirtschig, , G, Murrell, DF, Wojnarowska, F; Khumalo, NP	2003
Interventions for protecting renal function in the perioperative period	Zacharias, , M, Mugawar, M, Herbison, GP, Walker, RJ, Hovhannisyan, K, Sivalingam, P; Conlon, NP	2013
Interventions for haemolytic uraemic syndrome and thrombotic thrombocytopenic purpura	Michael, , M, Elliott, EJ, Ridley, GF, Hodson, EM; Craig, JC	2009
Interventions for patients and caregivers to improve knowledge of sickle cell disease and recognition of its related complications	Asnani, , MR, Quimby, KR, Bennett, NR; Francis, DK	2016
Interventions for preventing blood loss during the treatment of cervical intraepithelial neoplasia	Martin-Hirsch, , PPL; Bryant, A	2013
Interventions for promoting the initiation of breastfeeding	Balogun, , OO, O'Sullivan, EJ, McFadden, A, Ota, E, Gavine, A, Garner, CD, Renfrew, MJ; MacGillivray, S	2016
Interventions for preventing venous thromboembolism following abdominal aortic surgery	Bani-Hani, , M, Titi, MA, Jaradat, I; Al-Khaffaf, H	2008
Interventions for preventing late postnatal mother-to-child transmission of HIV	Horvath, , T, Madi, BC, Iuppa, IM, Kennedy, GE, Rutherford, GW; Read, JS	2009
Interventions for preventing distal intestinal obstruction syndrome (DIOS) in cystic fibrosis	Green, , J, Gilchrist, FJ; Carroll, W	2018
Interventions for increasing the use of shared decision making by healthcare professionals	Légaré, , F, Adekpedjou, R, Stacey, D, Turcotte, S, Kryworuchko, J, Graham, ID, Lyddiatt, A, Politi, MC, Thomson, R, Elwyn, G; Donner-Banzhoff, N	2018
Integrated management of childhood illness (IMCI) strategy for children under five	Gera, , T, Shah, D, Garner, P, Richardson, M; Sachdev, HS	2016
Interferon beta for secondary progressive multiple sclerosis	La Mantia, , L, Vacchi, L, Di Pietrantonj, C, Ebers, G, Rovaris, M, Fredrikson, S; Filippini, G	2012
Interventions for improving modifiable risk factor control in the secondary prevention of stroke	Bridgwood, , B, Lager, KE, Mistri, AK, Khunti, K, Wilson, AD; Modi, P	2018
Interleukin-2 as maintenance therapy for children and adults with acute myeloid leukaemia in first complete remission	Mao, , C, Fu, XH, Yuan, JQ, Yang, ZY, Huang, YF, YE, QL, Wu, XY, Hu, XF, Zhai, ZM; Tang, JL	2015
Interventions for chronic non-hypovolaemic hypotonic hyponatraemia	Nagler, , EV, Haller, MC, Van Biesen, W, Vanholder, R, Craig, JC; Webster, AC	2018

Interferon after surgery for women with advanced (Stage II-IV) epithelial ovarian cancer	Lawal, , AO, Musekiwa, A; Grobler, L	2013
Interventions for preventing high altitude illness: Part 3. Miscellaneous and non-pharmacological interventions	Molano Franco, , D, Nieto Estrada, VH, Gonzalez Garay, AG, Martí-Carvajal, AJ; Arevalo-Rodriguez, I	2019
Interventions for preventing falls in people after stroke	Verheyden, , GSAF, Weerdesteyn, V, Pickering, RM, Kunkel, D, Lennon, S, Geurts, ACH; Ashburn, A	2013
Interventionist versus expectant care for severe pre-eclampsia between 24 and 34 weeks' gestation	Churchill, , D, Duley, L, Thornton, JG, Moussa, M, Ali, HSM; Walker, KF	2018
Interventions for increasing fruit and vegetable consumption in children aged five years and under	Hodder, , RK, O'Brien, KM, Stacey, FG, Wyse, RJ, Clinton-McHarg, T, Tzelepis, F, James, EL, Bartlem, KM, Nathan, NK, Sutherland, R, Robson, E, Yoong, SL; Wolfenden, L	2018
Interventions for improving sleep quality in people with chronic kidney disease	Natale, , P, Ruospo, M, Saglimbene, VM, Palmer, SC; Strippoli, GFM	2019
Interventions for preventing obesity in children	Brown, , T, Moore, THM, Hooper, L, Gao, Y, Zayegh, A, Ijaz, S, Elwenspoek, M, Foxen, SC, Magee, L, O'Malley, C, Waters, E; Summerbell, CD	2019
Integration of HIV/AIDS services with maternal, neonatal and child health, nutrition, and family planning services	Lindegren, , ML, Kennedy, CE, Bain-Brickley, D, Azman, H, Creanga, AA, Butler, LM, Spaulding, AB, Horvath, T; Kennedy, GE	2012
Interventions for preventing falls in older people in care facilities and hospitals	Cameron, , ID, Dyer, SM, Panagoda, CE, Murray, GR, Hill, KD, Cumming, RG; Kerse, N	2018
Interventions for necrotizing soft tissue infections in adults	Hua, , C, Bosc, R, Sbidian, E, De Prost, N, Hughes, C, Jabre, P, Chosidow, O; Le Cleach, L	2018
Interventions for preventing upper gastrointestinal bleeding in people admitted to intensive care units	Toews, , I, George, AT, Peter, JV, Kirubakaran, R, Fontes, LES, Ezekiel, JPB; Meerpohl, JJ	2018
Interventions for improving adherence to iron chelation therapy in people with sickle cell disease or thalassaemia	Fortin, , PM, Fisher, SA, Madgwick, KV, Trivella, M, Hopewell, S, Doree, C; Estcourt, LJ	2018
Intermittent iron supplementation for reducing anaemia and its associated impairments in adolescent and adult menstruating women	Fernández-Gaxiola, , AC; De-Regil, LM	2019
Momordica charantia for type 2 diabetes mellitus	Ooi, , CP, Yassin, Z; Hamid, TA	2012
Maintenance therapy with oxytocin antagonists for inhibiting preterm birth after threatened preterm labour	Papatsonis, , DNM, Flenady, V; Liley, HG	2013
Multidisciplinary rehabilitation for adults with multiple sclerosis	Khan, , F, Turner-Stokes, L, Ng, L, Kilpatrick, T; Amatya, B	2007
Music interventions for preoperative anxiety	Bradt, , J, Dileo, C; Shim, M	2013
Methotrexate for primary biliary cirrhosis	Giljaca, , V, Poropat, G, Stimac, D; Gluud, C	2010
MVA85A vaccine to enhance BCG for preventing tuberculosis	Kashangura, , R, Jullien, S, Garner, P; Johnson, S	2019
Music therapy for people with autism spectrum disorder	Geretsegger, , M, Elefant, C, Mössler, KA; Gold, C	2014
Management of reported decreased fetal movements for improving pregnancy outcomes	Hofmeyr, , GJ; Novikova, N	2012
Mirtazapine adjunct for people with schizophrenia	Perry, , LA, Ramson, D; Stricklin, S	2018
Multinutrient fortification of human breast milk for preterm infants following hospital discharge	Young, , L, Embleton, ND, McCormick, FM; McGuire, W	2013
Mass media interventions for preventing smoking in young people	Carson-Chahhoud, , KV, Ameer, F, Sayehmiri, K, Hnin, K, van Agteren, JEM, Sayehmiri, F, Brinn, MP, Esterman, AJ, Chang, AB; Smith, BJ	2017
Methadone maintenance at different dosages for opioid dependence	Faggiano, , F, Vigna-Taglianti, F, Versino, E; Lemma, P	2003
Mesenchymal stromal cells as treatment or prophylaxis for acute or chronic graft-versus-host disease in haematopoietic stem cell transplant (HSCT) recipients with a haematological condition	Fisher, , SA, Cutler, A, Doree, C, Brunskill, SJ, Stanworth, SJ, Navarrete, C; Girdlestone, J	2019
Methylxanthines for prolonged non-specific cough in children	Chang, , AB, Halstead, RAP; Petsky, HL	2005
Mobile phone text messaging to improve medication adherence in secondary prevention of cardiovascular disease	Adler, , AJ, Martin, N, Mariani, J, Tajer, CD, Owolabi, OO, Free, C, Serrano, NC, Casas, JP; Perel, P	2017
Mosquito repellents for malaria prevention	Maia, , MF, Kliner, M, Richardson, M, Lengeler, C; Moore, SJ	2018
Monoamine oxidase B inhibitors for early Parkinson's disease	Turnbull, , K, Caslake, R, Macleod, A, Ives, N, Stowe, R; Counsell, C	2005
Media-delivered cognitive behavioural therapy and behavioural therapy (self-help) for anxiety disorders in adults	Mayo-Wilson, , E; Montgomery, P	2013
Methods of milk expression for lactating women	Becker, , GE, Smith, HA; Cooney, F	2016
Methotrexate for maintenance of remission in Crohn's disease	Patel, , V, Wang, Y, MacDonald, JK, McDonald, JWD; Chande, N	2014
Medical day hospital care for older people versus alternative forms of care	Brown, , L, Forster, A, Young, J, Crocker, T, Benham, A; Langhorne, P	2015
Medically assisted nutrition for adult palliative care patients	Good, , P, Richard, R, Syrmis, W, Jenkins-Marsh, S; Stephens, J	2014
Management of toxoplasmic encephalitis in HIV-infected adults (with an emphasis on resource-poor settings)	Dedicoat, , M; Livesley, N	2006
Milrinone for persistent pulmonary hypertension of the newborn	Bassler, , D, Kreutzer, K, McNamara, P; Kirpalani, H	2010
Multidisciplinary biopsychosocial rehabilitation for subacute low back pain	Marin, , TJ, Van Eerd, D, Irvin, E, Couban, R, Koes, BW, Malmivaara, A, van Tulder, MW; Kamper, SJ	2017

Mechanical ventilation for newborn infants with respiratory failure due to pulmonary disease	Henderson-Smart, , DJ, Wilkinson, AR; Raynes-Greenow, CH	2002
Medical interventions for high-grade vulval intraepithelial neoplasia	Pepas, , L, Kaushik, S, Nordin, A, Bryant, A; Lawrie, TA	2015
Maintenance immunosuppression for adults undergoing liver transplantation: a network meta-analysis	Rodríguez-Perálvarez, , M, Guerrero-Misas, M, Thorburn, D, Davidson, BR, Tsochatzis, E; Gurusamy, KS	2017
Mesalamine (5-ASA) for the prevention of recurrent diverticulitis	Carter, , F, Alsayb, M, Marshall, JK; Yuan, Y	2017
Medical treatment for botulism	Chalk, , CH, Benstead, TJ, Pound, JD; Keezer, MR	2019
Magnesium sulphate versus phenytoin for eclampsia	Duley, , L, Henderson-Smart, DJ; Chou, D	2010
Mini-Cog for the diagnosis of Alzheimer's disease dementia and other dementias within a primary care setting	Seitz, , DP, Chan, CCH, Newton, HT, Gill, SS, Herrmann, N, Smailagic, N, Nikolaou, V; Fage, BA	2018
Metformin treatment before and during IVF or ICSI in women with polycystic ovary syndrome	Tso, , LO, Costello, MF, Albuquerque, LET, Andriolo, RB; Macedo, CR	2014
Mesh versus non-mesh for inguinal and femoral hernia repair	Lockhart, , K, Dunn, D, Teo, S, Ng, JY, Dhillon, M, Teo, E; van Driel, ML	2018
Methylxanthine treatment for apnoea in preterm infants	Henderson-Smart, , DJ; De Paoli, AG	2010
Maternal oxygen administration for suspected impaired fetal growth	Say, , L, Gülmezoglu, AM; Hofmeyr, GJ	2003
Mass media interventions for smoking cessation in adults	Bala, , MM, Strzeszynski, L; Topor-Madry, R	2017
Medical interventions for the prevention of platinum-induced hearing loss in children with cancer	van As, , JW, van den Berg, H; van Dalen, EC	2019
Methotrexate for maintenance of remission in ulcerative colitis	Wang, , Y, MacDonald, JK, Vandermeer, B, Griffiths, AM; El-Matary, W	2015
Maternal dietary antigen avoidance during pregnancy or lactation, or both, for preventing or treating atopic disease in the child	Kramer, , MS; Kakuma, R	2012
Methotrexate for psoriatic arthritis	Wilsdon, , TD, Whittle, SL, Thynne, TRJ; Mangoni, AA	2019
Multidisciplinary rehabilitation for fibromyalgia and musculoskeletal pain in working age adults	Karjalainen, , KA, Malmivaara, A, van Tulder, MW, Roine, R, Jauhiainen, M, Hurri, H; Koes, BW	1999
Multiple risk factor interventions for primary prevention of coronary heart disease	Ebrahim, , S, Taylor, F, Ward, K, Beswick, A, Burke, M; Davey Smith, G	2011
Melatonin for women in pregnancy for neuroprotection of the fetus	Wilkinson, , D, Shepherd, E; Wallace, EM	2016
Malaria chemoprophylaxis in sickle cell disease	Oniyangi, , O; Omari, AAA	2006
Maternal nutrient supplementation for suspected impaired fetal growth	Say, , L, Gülmezoglu, AM; Hofmeyr, GJ	2003
Maternal and foetal outcomes following natural vaginal versus caesarean section (c-section) delivery in women with bleeding disorders and carriers	Karanth, , L, Kanagasabai, S; Abas, ABL	2017
Morphine for chronic neuropathic pain in adults	Cooper, , TE, Chen, J, Wiffen, PJ, Derry, S, Carr, DB, Aldington, D, Cole, P; Moore, RA	2017
Mass media interventions for reducing mental health-related stigma	Clement, , S, Lassman, F, Barley, E, Evans-Lacko, S, Williams, P, Yamaguchi, S, Slade, M, Rüsch, N; Thornicroft, G	2013
Mupirocin ointment for preventing Staphylococcus aureus infections in nasal carriers	van Rijen, , M, Bonten, M, Wenzel, R; Kluytmans, J	2008
Medical versus surgical interventions for open angle glaucoma	Burr, , J, Azuara-Blanco, A, Avenell, A; Tuulonen, A	2012
Mycophenolate mofetil versus methotrexate for prevention of graft-versus-host disease in people receiving allogeneic hematopoietic stem cell transplantation	Kharfan-Dabaja, , M, Mhaskar, R, Reljic, T, Pidala, J, Perkins, JB, Djulbegovic, B; Kumar, A	2014
Methods of preventing bacterial sepsis and wound complications after liver transplantation	Gurusamy, , KS, Nagendran, M; Davidson, BR	2014
Mosquito larval source management for controlling malaria	Tusting, , LS, Thwing, J, Sinclair, D, Fillinger, U, Gimnig, J, Bonner, KE, Bottomley, C; Lindsay, SW	2013
Mirror therapy for improving motor function after stroke	Thieme, , H, Morkisch, N, Mehrholz, J, Pohl, M, Behrens, J, Borgetto, B; Dohle, C	2018
Moderately early (7-14 days) postnatal corticosteroids for preventing chronic lung disease in preterm infants	Halliday, , HL, Ehrenkranz, RA; Doyle, LW	2003
Management of sexual dysfunction due to antipsychotic drug therapy	Schmidt, , HM, Hagen, M, Kriston, L, Soares-Weiser, K, Maayan, N; Berner, MM	2012
Muscle relaxants for pain management in rheumatoid arthritis	Richards, , BL, Whittle, SL; Buchbinder, R	2012
Motivational interviewing for improving recovery after stroke	Cheng, , D, Qu, Z, Huang, J, Xiao, Y, Luo, H; Wang, J	2015
Mechanical bowel preparation for elective colorectal surgery	Güenaga, , KF, Matos, D; Wille-Jørgensen, P	2011
Mannitol for acute stroke	Bereczki, , D, Liu, M, Fernandes do Prado, G; Fekete, I	2007
Massage therapy for people with HIV/AIDS	Hillier, , SL, Louw, Q, Morris, L, Uwimana, J; Statham, S	2010
Melatonin and agomelatine for preventing seasonal affective disorder	Nussbaumer-Streit, , B, Greenblatt, A, Kaminski-Hartenthaler, A, Van Noord, MG, Forneris, CA, Morgan, LC, Gaynes, BN, Wipplinger, J, Lux, LJ, Winkler, D; Gartlehner, G	2019
Methylphenidate for children and adolescents with autism spectrum disorder	Sturman, , N, Deckx, L; van Driel, ML	2017

Melatonin for the treatment of dementia	Jansen, , SL, Forbes, D, Duncan, V, Morgan, DG; Malouf, R	2006
Mucolytic agents versus placebo for chronic bronchitis or chronic obstructive pulmonary disease	Poole, , P, Sathananthan, K; Fortescue, R	2019
Microwave thermotherapy for benign prostatic hyperplasia	Hoffman, , RM, Monga, M, Elliott, SP, MacDonald, R, Langsjoen, J, Tacklind, J; Wilt, TJ	2012
Methotrexate for ankylosing spondylitis	Chen, , J, Veras, MMS, Liu, C; Lin, J	2013
Methods of vascular occlusion for elective liver resections	Gurusamy, , KS, Sheth, H, Kumar, Y, Sharma, D; Davidson, BR	2009
Mitoxantrone for multiple sclerosis	Martinelli Boneschi, , F, Vacchi, L, Rovaris, M, Capra, R; Comi, G	2013
Moderate-term, low-dose corticosteroids for rheumatoid arthritis	Criswell, , L, Saag, K, Sems, KM, Welch, V, Shea, B, Wells, GA; Suarez-Almazor, ME	1998
Mobile phone-based interventions for improving adherence to medication prescribed for the primary prevention of cardiovascular disease in adults	Palmer, , MJ, Barnard, S, Perel, P; Free, C	2018
Mechanical traction for neck pain with or without radiculopathy	Graham, , N, Gross, A, Goldsmith, CH, Klaber Moffett, J, Haines, T, Burnie, SJ; Peloso, PMJ	2008
Motivational interviewing for the prevention of alcohol misuse in young adults	Foxcroft, , DR, Coombes, L, Wood, S, Allen, D, Almeida Santimano, NML; Moreira, MT	2016
Management strategies for pancreatic pseudocysts	Gurusamy, , KS, Pallari, E, Hawkins, N, Pereira, SP; Davidson, BR	2016
Misoprostol for induction of labour to terminate pregnancy in the second or third trimester for women with a fetal anomaly or after intrauterine fetal death	Dodd, , JM; Crowther, CA	2010
Management of faecal incontinence and constipation in adults with central neurological diseases	Coggrave, , M, Norton, C; Cody, JD	2014
Mohs micrographic surgery versus surgical excision for periocular basal cell carcinoma	Narayanan, , K, Hadid, OH; Barnes, EA	2014
Micronutrient supplementation for children with HIV infection	Irlam, , JH, Siegfried, N, Visser, ME; Rollins, NC	2013
Metal protein attenuating compounds for the treatment of Alzheimer's dementia	Sampson, , EL, Jenagaratnam, L; McShane, R	2014
Medical and dietary interventions for preventing recurrent urinary stones in children	Kern, , A, Grimsby, G, Mayo, H; Baker, LA	2017
Methotrexate for multiple sclerosis	Gray, , O, McDonnell, GV; Forbes, RB	2004
Mifepristone for uterine fibroids	Tristan, , M, Orozco, LJ, Steed, A, Ramírez-Morera, A; Stone, P	2012
Monoaminergic agonists for acute traumatic brain injury	Forsyth, , RJ, Jayamoni, B, Paine, TC; Mascarenhas, S	2006
Multidisciplinary care for adults with amyotrophic lateral sclerosis or motor neuron disease	Ng, , L; Khan, F	2009
Magnetic resonance imaging versus computed tomography for detection of acute vascular lesions in patients presenting with stroke symptoms	Brazzelli, , M, Sandercock, PAG, Chappell, FM, Celani, MG, Righetti, E, Arestis, N, Wardlaw, JM; Deeks, JJ	2009
Microwave therapy for cervical ectropion	Liu, , Y, Yang, K, Wu, T, Roberts, H, Li, J, Tian, J, Ma, B, Tan, J; Zhang, P	2007
Male circumcision for prevention of heterosexual acquisition of HIV in men	Siegfried, , N, Muller, M, Deeks, JJ; Volmink, J	2009
Melatonin as add-on treatment for epilepsy	Brigo, , F, Igwe, SC; Del Felice, A	2016
Mono and multifaceted inhalant and/or food allergen reduction interventions for preventing asthma in children at high risk of developing asthma	Maas, , T, Kaper, J, Sheikh, A, Knottnerus, JA, Wesseling, G, Dompeling, E, Muris, JWM; van Schayck, CP	2009
Methotrexate for high-grade osteosarcoma in children and young adults	van Dalen, , EC, van As, JW; de Camargo, B	2011
Metformin for endometrial hyperplasia	Clement, , NS, Oliver, TRW, Shiwani, H, Sanner, JRF, Mulvaney, CA; Atiomo, W	2017
Management of drainage for malignant ascites in gynaecological cancer	Keen, , A, Fitzgerald, D, Bryant, A; Dickinson, HO	2010
Methods to decrease blood loss and transfusion requirements for liver transplantation	Gurusamy, , KS, Pissanou, T, Pikhart, H, Vaughan, J, Burroughs, AK; Davidson, BR	2011
Metered dose inhalers versus nebulizers for aerosol bronchodilator delivery for adult patients receiving mechanical ventilation in critical care units	Holland, , A, Smith, F, Penny, K, McCrossan, G, Veitch, L; Nicholson, C	2013
Music for insomnia in adults	Jespersen, , KV, Koenig, J, Jennum, P; Vuust, P	2015
Methadone for neuropathic pain in adults	McNicol, , ED, Ferguson, MC; Schumann, R	2017
Mefloquine for preventing malaria during travel to endemic areas	Tickell-Painter, , M, Maayan, N, Saunders, R, Pace, C; Sinclair, D	2017
Massage for promoting mental and physical health in typically developing infants under the age of six months	Bennett, , C, Underdown, A; Barlow, J	2013
Monoclonal antibody for reducing the risk of respiratory syncytial virus infection in children	Andabaka, , T, Nickerson, JW, Rojas-Reyes, MX, Rueda, JD, Bacic Vrca, V; Barsic, B	2013
Midazolam for sedation before procedures	Conway, , A, Rolley, J; Sutherland, JR	2016
Medical treatments for incomplete miscarriage	Kim, , C, Barnard, S, Neilson, JP, Hickey, M, Vazquez, JC; Dou, L	2017

Micronutrient supplementation in pregnant women with HIV infection	Siegfried, , N, Irlam, JH, Visser, ME; Rollins, NN	2012
Medical and surgical treatment for ocular myasthenia	Benatar, , M; Kaminski, H	2012
Motivational interviewing for substance abuse	Smedslund, , G, Berg, RC, Hammerstrøm, KT, Steiro, A, Leiknes, KA, Dahl, HM; Karlsen, K	2011
Methods for securing endotracheal tubes in newborn infants	Lai, , M, Inglis, GDT, Hose, K, Jardine, LA; Davies, MW	2014
Medications for increasing milk supply in mothers expressing breastmilk for their preterm hospitalised infants	Donovan, , TJ; Buchanan, K	2012
Magnetic resonance perfusion for differentiating low-grade from high-grade gliomas at first presentation	Abrigo, , JM, Fountain, DM, Provenzale, JM, Law, EK, Kwong, JSW, Hart, MG; Tam, WWS	2018
Mobile phone text messaging for promoting adherence to antiretroviral therapy in patients with HIV infection	Horvath, , T, Azman, H, Kennedy, GE; Rutherford, GW	2012
Medically assisted hydration for adult palliative care patients	Good, , P, Richard, R, Syrmis, W, Jenkins-Marsh, S; Stephens, J	2014
Medical and surgical interventions for the treatment of urinary stones in children	Barreto, , L, Jung, JH, Abdelrahim, A, Ahmed, M, Dawkins, GPC; Kazmierski, M	2018
Mindfulness-based stress reduction for family carers of people with dementia	Liu, , Z, Sun, YY; Zhong, BL	2018
Medical methods for mid-trimester termination of pregnancy	Wildschut, , H, Both, MI, Medema, S, Thomee, E, Wildhagen, MF; Kapp, N	2011
Mammography in combination with breast ultrasonography versus mammography for breast cancer screening in women at average risk	Gartlehner, , G, Thaler, K, Chapman, A, Kaminski-Hartenthaler, A, Berzaczy, D, Van Noord, MG; Helbich, TH	2013
Methods of term labour induction for women with a previous caesarean section	West, , HM, Jozwiak, M; Dodd, JM	2017
Male circumcision for prevention of homosexual acquisition of HIV in men	Wiysonge, , CS, Kongnyuy, EJ, Shey, M, Muula, AS, Navti, OB, Akl, EA; Lo, YR	2011
Microwave coagulation for liver metastases	Bala, , MM, Riemsma, RP, Wolff, R; Kleijnen, J	2013
Mycobacterium vaccae immunotherapy for treating tuberculosis	de Bruyn, , G; Garner, P	2003
Minimally invasive surgery versus radiotherapy/chemoradiotherapy for small-volume primary oropharyngeal carcinoma	Howard, , J, Masterson, L, Dwivedi, RC, Riffat, F, Benson, R, Jefferies, S, Jani, P, Tysome, JR; Nutting, C	2016
Mindfulness-based stress reduction for women diagnosed with breast cancer	Schell, , LK, Monsef, I, Wöckel, A; Skoetz, N	2019
Medical treatment for early fetal death (less than 24 weeks)	Lemmers, , M, Verschoor, MAC, Kim, BV, Hickey, M, Vazquez, JC, Mol, BWJ; Neilson, JP	2019
Monosodium glutamate avoidance for chronic asthma in adults and children	Zhou, , Y, Yang, M; Dong, BR	2012
Modifying the consistency of food and fluids for swallowing difficulties in dementia	Flynn, , E, Smith, CH, Walsh, CD; Walshe, M	2018
Motivational interviewing for improving outcomes in youth living with HIV	Mbuagbaw, , L, Ye, C; Thabane, L	2012
Mobile phone messaging for communicating results of medical investigations	Gurol-Urganci, , I, de Jongh, T, Vodopivec-Jamsek, V, Car, J; Atun, R	2012
Maternal prenatal and/or postnatal n-3 long chain polyunsaturated fatty acids (LCPUFA) supplementation for preventing allergies in early childhood	Gunaratne, , AW, Makrides, M; Collins, CT	2015
Multifactorial and multiple component interventions for preventing falls in older people living in the community	Hopewell, , S, Adedire, O, Copsey, BJ, Boniface, GJ, Sherrington, C, Clemson, L, Close, JCT; Lamb, SE	2018
Mobile phone-based interventions for improving contraception use	Smith, , C, Gold, J, Ngo, TD, Sumpter, C; Free, C	2015
Multidisciplinary care for Guillain-Barré syndrome	Khan, , F, Ng, L, Amatya, B, Brand, C; Turner-Stokes, L	2010
Monoamine oxidase inhibitors (MAOIs) for fibromyalgia syndrome	Tort, , S, Urrútia, G, Nishishinya, MB; Walitt, B	2012
Methadone for chronic non-cancer pain in adults	Haroutounian, , S, McNicol, ED; Lipman, AG	2012
Massage for low-back pain	Furlan, , AD, Giraldo, M, Baskwill, A, Irvin, E; Imamura, M	2015
Mefloquine for preventing malaria in non-immune adult travellers	Croft, , AM; Garner, P	2000
Modification of the home environment for the reduction of injuries	Turner, , S, Arthur, G, Lyons, RA, Weightman, AL, Mann, MK, Jones, SJ, John, A; Lannon, S	2011
Methotrexate as a steroid sparing agent for asthma in adults	Davies, , HRHR, Olson, LLG; Gibson, PG	1998
Massage for promoting growth and development of preterm and/or low birth-weight infants	Vickers, , A, Ohlsson, A, Lacy, J; Horsley, A	2004
Methotrexate for treating juvenile idiopathic arthritis	Takken, , T, van der Net, JJ; Helders, PPJM	2001
Melatonin for pre- and postoperative anxiety in adults	Hansen, , MV, Halladin, NL, Rosenberg, J, Gögenur, I; Møller, AM	2015
Mediterranean-style diet for the primary and secondary prevention of cardiovascular disease	Rees, , K, Takeda, A, Martin, N, Ellis, L, Wijesekara, D, Vepa, A, Das, A, Hartley, L; Stranges, S	2019
Media campaigns for the prevention of illicit drug use in young people	Ferri, , M, Allara, E, Bo, A, Gasparrini, A; Faggiano, F	2013
Mannitol and other osmotic diuretics as adjuncts for treating cerebral malaria	Okoromah, , CAN, Afolabi, BB; Wall, ECB	2011

Magnetic resonance imaging, magnetic resonance arthrography and ultrasonography for assessing rotator cuff tears in people with shoulder pain for whom surgery is being considered	Lenza, , M, Buchbinder, R, Takwoingi, Y, Johnston, RV, Hanchard, NCA; Faloppa, F	2013
Methylphenidate for attention deficit hyperactivity disorder (ADHD) in children and adolescents – assessment of adverse events in non-randomised studies	Storebø, , OJ, Pedersen, N, Ramstad, E, Kielsholm, ML, Nielsen, SS, Krogh, HB, Moreira-Maia, CR, Magnusson, FL, Holmskov, M, Gerner, T, Skoog, M, Rosendal, S, Groth, C, Gillies, D, Buch Rasmussen, K, Gauci, D, Zwi, M, Kirubakaran, R, Håkonsen, SJ, Aagaard, L, Simonsen, E; Gluud, C	2018
Music interventions for acquired brain injury	Magee, , WL, Clark, I, Tamplin, J; Bradt, J	2017
Modes of exercise training for intermittent claudication	Lauret, , GJ, Fakhry, F, Fokkenrood, HJP, Hunink, MGM, Teijink, JAW; Spronk, S	2014
Mirtazapine for fibromyalgia in adults	Welsch, , P, Bernardy, K, Derry, S, Moore, RA; Häuser, W	2018
Massage, reflexology and other manual methods for pain management in labour	Smith, , CA, Levett, KM, Collins, CT, Dahlen, HG, Ee, CC; Suganuma, M	2018
Multidimensional rehabilitation programmes for adult cancer survivors	Scott, , DA, Mills, M, Black, A, Cantwell, M, Campbell, A, Cardwell, CR, Porter, S; Donnelly, M	2013
Metformin added to insulin therapy for type 1 diabetes mellitus in adolescents	Abdelghaffar, , S; Attia, AM	2009
Mobile clinics for women's and children's health	Abdel-Aleem, , H, El-Gibaly, OMH, EL-Gazzar, AFES; Al-Attar, GST	2016
Mini-Mental State Examination (MMSE) for the detection of dementia in clinically unevaluated people aged 65 and over in community and primary care populations	Creavin, , ST, Wisniewski, S, Noel-Storr, AH, Trevelyan, CM, Hampton, T, Rayment, D, Thom, VM, Nash, KJE, Elhamoui, H, Milligan, R, Patel, AS, Tsivos, DV, Wing, T, Phillips, E, Kellman, SM, Shackleton, HL, Singleton, GF, Neale, BE, Watton, ME; Cullum, S	2016
Mentoring adolescents to prevent drug and alcohol use	Thomas, , RE, Lorenzetti, D; Spragins, W	2011
Methotrexate for treating rheumatoid arthritis	Lopez-Olivo, , MA, Siddhanamatha, HR, Shea, B, Tugwell, P, Wells, GA; Suarez-Almazor, ME	2014
Multidisciplinary rehabilitation following botulinum toxin and other focal intramuscular treatment for post-stroke spasticity	Demetrios, , M, Khan, F, Turner-Stokes, L, Brand, C; McSweeney, S	2013
Metformin for women who are overweight or obese during pregnancy for improving maternal and infant outcomes	Dodd, , JM, Grivell, RM, Deussen, AR; Hague, WM	2018
Mechanical insufflation-exsufflation for people with neuromuscular disorders	Morrow, , B, Zampoli, M, van Aswegen, H; Argent, A	2013
Maxillary distraction osteogenesis versus orthognathic surgery for cleft lip and palate patients	Kloukos, , D, Fudalej, P, Sequeira-Byron, P; Katsaros, C	2018
Melatonin for the promotion of sleep in adults in the intensive care unit	Lewis, , SR, Pritchard, MW, Schofield-Robinson, OJ, Alderson, P; Smith, AF	2018
Midwife-led continuity models versus other models of care for childbearing women	Sandall, , J, Soltani, H, Gates, S, Shennan, A; Devane, D	2016
Maintenance agonist treatments for opiate-dependent pregnant women	Minozzi, , S, Amato, L, Bellisario, C, Ferri, M; Davoli, M	2013
Mass media interventions: effects on health services utilisation	Grilli, , R, Ramsay, C; Minozzi, S	2002
Motor control exercise for acute non-specific low back pain	Macedo, , LG, Saragiotto, BT, Yamato, TP, Costa, LOP, Menezes Costa, LC, Ostelo, RWJG; Maher, CG	2016
Music therapy for people with schizophrenia and schizophrenia-like disorders	Geretsegger, , M, Mössler, KA, Bieleninik, Ł, Chen, XJ, Heldal, TO; Gold, C	2017
Maintenance treatment with antipsychotic drugs for schizophrenia	Leucht, , S, Tardy, M, Komossa, K, Heres, S, Kissling, W; Davis, JM	2012
Mobile phone messaging for preventive health care	Vodopivec-Jamsek, , V, de Jongh, T, Gurol-Urganci, I, Atun, R; Car, J	2012
Maternal probiotic supplementation for prevention of morbidity and mortality in preterm infants	Grev, , J, Berg, M; Soll, R	2018
Mobile phone messaging for facilitating self-management of long-term illnesses	de Jongh, , T, Gurol-Urganci, I, Vodopivec-Jamsek, V, Car, J; Atun, R	2012
Methylphenidate for children and adolescents with attention deficit hyperactivity disorder (ADHD)	Storebø, , OJ, Ramstad, E, Krogh, HB, Nilausen, TD, Skoog, M, Holmskov, M, Rosendal, S, Groth, C, Magnusson, FL, Moreira-Maia, CR, Gillies, D, Buch Rasmussen, K, Gauci, D, Zwi, M, Kirubakaran, R, Forsbøl, B, Simonsen, E; Gluud, C	2015
Motor control exercise for chronic non-specific low-back pain	Saragiotto, , BT, Maher, CG, Yamato, TP, Costa, LOP, Menezes Costa, LC, Ostelo, RWJG; Macedo, LG	2016
Micronutrient supplementation in adults with HIV infection	Visser, , ME, Durao, S, Sinclair, D, Irlam, JH; Siegfried, N	2017
Milk thistle for alcoholic and/or hepatitis B or C virus liver diseases	Rambaldi, , A, Jacobs, BP; Gluud, C	2007
Managed alcohol as a harm reduction intervention for alcohol addiction in populations at high risk for substance abuse	Muckle, , W, Muckle, J, Welch, V; Tugwell, P	2012
Mobile phone messaging reminders for attendance at healthcare appointments	Gurol-Urganci, , I, de Jongh, T, Vodopivec-Jamsek, V, Atun, R; Car, J	2013
Mycophenolate mofetil for relapsing-remitting multiple sclerosis	Xiao, , Y, Huang, J, Luo, H; Wang, J	2014
Medical interventions for traumatic hyphema	Gharaibeh, , A, Savage, HI, Scherer, RW, Goldberg, MF; Lindsley, K	2019
Male involvement for increasing the effectiveness of prevention of mother-to-child HIV transmission (PMTCT) programmes	Brusamento, , S, Ghanotakis, E, Tudor Car, L, van-Velthoven, MHMMT, Majeed, A; Car, J	2012

Medical treatments for idiopathic thrombocytopenic purpura during pregnancy	Martí-Carvajal, , AJ, Peña-Martí, GE; Comunián-Carrasco, G	2009
Memantine for dementia	McShane, , R, Westby, MJ, Roberts, E, Minakaran, N, Schneider, L, Farrimond, LE, Maayan, N, Ware, J; Debarros, J	2019
Memory rehabilitation for people with multiple sclerosis	das Nair, , R, Martin, KJ; Lincoln, NB	2016
Music-based therapeutic interventions for people with dementia	van der Steen, , JT, Smaling, HJA, van der Wouden, JC, Bruinsma, MS, Scholten, RJPM; Vink, AC	2018
Multiple-micronutrient supplementation for women during pregnancy	Keats, , EC, Haider, BA, Tam, E; Bhutta, ZA	2019
Miscellaneous treatments for antipsychotic-induced tardive dyskinesia	Soares-Weiser, , K, Rathbone, J, Ogawa, Y, Shinohara, K; Bergman, H	2018
Marine-derived n-3 fatty acids therapy for stroke	Alvarez Campano, , CG, Macleod, MJ, Aucott, L; Thies, F	2019
Planned early birth versus expectant management for women with preterm prelabour rupture of membranes prior to 37 weeks' gestation for improving pregnancy outcome	Bond, , DM, Middleton, P, Levett, KM, van der Ham, DP, Crowther, CA, Buchanan, SL; Morris, J	2017
Pioglitazone for type 2 diabetes mellitus	Richter, , B, Bandeira-Echtler, E, Bergerhoff, K, Clar, C; Ebrahim, SH	2006
Person-directed, non-pharmacological interventions for sleepiness at work and sleep disturbances caused by shift work	Slanger, , TE, Gross, JV, Pinger, A, Morfeld, P, Bellinger, M, Duhme, AL, Reichardt Ortega, RA, Costa, G, Driscoll, TR, Foster, RG, Fritschi, L, Sallinen, M, Liira, J; Erren, TC	2016
Pharmacological treatment for aphasia following stroke	Greener, , J, Enderby, P; Whurr, R	2001
Plasma transfusion strategies for critically ill patients	Karam, , O, Tucci, M, Combescure, C, Lacroix, J; Rimensberger, PC	2013
Physiotherapy for Parkinson's disease: a comparison of techniques	Tomlinson, , CL, Herd, CP, Clarke, CE, Meek, C, Patel, S, Stowe, R, Deane, KHO, Shah, L, Sackley, CM, Wheatley, K; Ives, N	2014
Pharmacological interventions for pain in children and adolescents with life-limiting conditions	Beecham, , E, Candy, B, Howard, R, McCulloch, R, Laddie, J, Rees, H, Vickerstaff, V, Bluebond-Langner, M; Jones, L	2015
Pharmacotherapy for smoking cessation: effects by subgroup defined by genetically informed biomarkers	Schuit, , E, Panagiotou, OA, Munafò, MR, Bennett, DA, Bergen, AW; David, SP	2017
Pessaries (mechanical devices) for pelvic organ prolapse in women	Bugge, , C, Adams, EJ, Gopinath, D; Reid, F	2013
Physiotherapy interventions for ankylosing spondylitis	Dagfinrud, , H, Hagen, KB; Kvien, TK	2008
Pharmacotherapy for sleep bruxism	Macedo, , CR, Macedo, EC, Torloni, MR, Silva, AB; Prado, GF	2014
Piracetam for acute ischaemic stroke	Ricci, , S, Celani, MG, Cantisani, TA; Righetti, E	2012
Phototherapy for treating foot ulcers in people with diabetes	Wang, , HT, Yuan, JQ, Zhang, B, Dong, ML, Mao, C; Hu, D	2017
Phosphodiesterase III inhibitors for heart failure	Amsallem, , E, Kasparian, C, Haddour, G, Boissel, JP; Nony, P	2005
Pilates for low back pain	Yamato, , TP, Maher, CG, Saragiotto, BT, Hancock, MJ, Ostelo, RWJG, Cabral, CMN, Menezes Costa, LC; Costa, LOP	2015
Pharmacotherapies for sleep disturbances in dementia	McCleery, , J, Cohen, DA; Sharpley, AL	2016
Planned early delivery versus expectant management for hypertensive disorders from 34 weeks gestation to term	Cluver, , C, Novikova, N, Koopmans, CM; West, HM	2017
Physiotherapy versus placebo or no intervention in Parkinson's disease	Tomlinson, , CL, Patel, S, Meek, C, Herd, CP, Clarke, CE, Stowe, R, Shah, L, Sackley, CM, Deane, KHO, Wheatley, K; Ives, N	2013
Pharmacological interventions for clozapine-induced hypersalivation	Syed, , R, Au, K, Cahill, C, Duggan, L, He, Y, Udu, V; Xia, J	2008
Plasma interleukin-6 concentration for the diagnosis of sepsis in critically ill adults	Molano Franco, , D, Arevalo-Rodriguez, I, Roqué i Figuls, M, Montero Oleas, NG, Nuvials, X; Zamora, J	2019
Physiotherapy interventions for shoulder pain	Green, , S, Buchbinder, R; Hetrick, SE	2003
Plugs for containing faecal incontinence	Deutekom, , M; Dobben, AC	2015
Post-operative therapy for metacarpophalangeal arthroplasty	Massy-Westropp, , N, Johnston, RV; Hill, CL	2008
Polyunsaturated fatty acid supplementation for schizophrenia	Irving, , CB, Mumby-Croft, R; Joy, LA	2006
Positioning for acute respiratory distress in hospitalised infants and children	Gillies, , D, Wells, D; Bhandari, AP	2012
Physical training for asthma	Carson, , KV, Chandratilleke, MG, Picot, J, Brinn, MP, Esterman, AJ; Smith, BJ	2013
Physical health care monitoring for people with serious mental illness	Tosh, , G, Clifton, AV, Xia, J; White, MM	2014
Pharmacologic interventions for treating phantom limb pain	Alviar, , MJM, Hale, T; Lim-Dungca, M	2016
Pharmacological interventions for ischaemia reperfusion injury in liver resection surgery performed under vascular control	Abu-Amara, , M, Gurusamy, KS, Glantzounis, G, Fuller, B; Davidson, BR	2009
Pharmacological interventions for non-alcohol related fatty liver disease (NAFLD)	Lombardi, , R, Onali, S, Thorburn, D, Davidson, BR, Gurusamy, KS; Tsochatzis, E	2017
Phototherapy for treating pressure ulcers	Chen, , C, Hou, WH, Chan, ESY, Yeh, ML; Lo, HLD	2014
Positive end-expiratory pressure (PEEP) during anaesthesia for prevention of mortality and postoperative pulmonary complications	Barbosa, , FT, Castro, AA; de Sousa-Rodrigues, CF	2014
Pit and fissure sealants for preventing dental decay in permanent teeth	Ahovuo-Saloranta, , A, Forss, H, Walsh, T, Nordblad, A, Mäkelä, M; Worthington, HV	2017

Pharmacotherapy for chronic cognitive impairment in traumatic brain injury	Dougall, , D, Poole, N; Agrawal, N	2015
Physical conditioning as part of a return to work strategy to reduce sickness absence for workers with back pain	Schaafsma, , FG, Whelan, K, van der Beek, AJ, van der Es-Lambeek, LC, Ojajärvi, A; Verbeek, JH	2013
Placebo interventions for all clinical conditions	Hróbjartsson, , A; Gøtzsche, PC	2010
Post-pyloric versus gastric tube feeding for preventing pneumonia and improving nutritional outcomes in critically ill adults	Alkhawaja, , S, Martin, C, Butler, RJ; Gwadry-Sridhar, F	2015
Physical training for bronchiectasis	Bradley, , JM, Moran, F; Greenstone, M	2002
Perioperative statin therapy for improving outcomes during and after noncardiac vascular surgery	Sanders, , RD, Nicholson, A, Lewis, SR, Smith, AF; Alderson, P	2013
Pharmacological interventions for treating heart failure in patients with Chagas cardiomyopathy	Martí-Carvajal, , AJ; Kwong, JSW	2016
Planned hospital birth versus planned home birth	Olsen, , O; Clausen, JA	2012
Postoperative adjuvant chemotherapy in rectal cancer operated for cure.	Petersen, , SH, Harling, H, Kirkeby, LT, Wille-Jørgensen, P; Mocellin, S	2012
Pneumococcal vaccination during pregnancy for preventing infant infection	Chaithongwongwatthana, , S, Yamasmit, W, Limpongsanurak, S, Lumbiganon, P; Tolosa, JE	2015
Phlebotonics for venous insufficiency	Martinez-Zapata, , MJ, Vernooij, RWM, Uriona Tuma, SM, Stein, AT, Moreno, RM, Vargas, E, Capellà, D; Bonfill Cosp, X	2016
Physical interventions to interrupt or reduce the spread of respiratory viruses	Jefferson, , T, Del Mar, CB, Dooley, L, Ferroni, E, Al-Ansary, LA, Bawazeer, GA, van Driel, ML, Nair, S, Jones, MA, Thorning, S; Conly, JM	2011
Plasma transfusions prior to lumbar punctures and epidural catheters for people with abnormal coagulation	Estcourt, , LJ, Desborough, MJ, Doree, C, Hopewell, S; Stanworth, SJ	2017
Platinum versus non-platinum chemotherapy regimens for small cell lung cancer	Amarasena, , IU, Chatterjee, S, Walters, JAE, Wood-Baker, R; Fong, KM	2015
Pharmacotherapy for hypertension in adults aged 18 to 59 years	Musini, , VM, Gueyffier, F, Puil, L, Salzwedel, DM; Wright, JM	2017
Pharmacological interventions for alcoholic liver disease (alcohol-related liver disease)	Buzzetti, , E, Kalafateli, M, Thorburn, D, Davidson, BR, Thiele, M, Gluud, LL, Del Giovane, C, Askgaard, G, Krag, A, Tsochatzis, E; Gurusamy, KS	2017
Pharmacological treatment of vascular risk factors for reducing mortality and cardiovascular events in patients with abdominal aortic aneurysm	Robertson, , L, Atallah, E; Stansby, G	2017
Phosphodiesterase 4 inhibitors for chronic obstructive pulmonary disease	Chong, , J, Leung, B; Poole, P	2017
Post-exposure passive immunisation for preventing rubella and congenital rubella syndrome	Young, , MK, Cripps, AW, Nimmo, GR; van Driel, ML	2015
Pharmacological interventions for acute hepatitis B infection	Mantzoukis, , K, Rodríguez-Perálvarez, M, Buzzetti, E, Thorburn, D, Davidson, BR, Tsochatzis, E; Gurusamy, KS	2017
Pimozide for schizophrenia or related psychoses	Mothi, , M; Sampson, S	2013
Portion, package or tableware size for changing selection and consumption of food, alcohol and tobacco	Hollands, , GJ, Shemilt, I, Marteau, TM, Jebb, SA, Lewis, HB, Wei, Y, Higgins, JPT; Ogilvie, D	2015
Pharmacological interventions for antisocial personality disorder	Khalifa, , N, Duggan, C, Stoffers, J, Huband, N, Völlm, BA, Ferriter, M; Lieb, K	2010
Platelet glycoprotein IIb/IIIa blockers during percutaneous coronary intervention and as the initial medical treatment of non-ST segment elevation acute coronary syndromes	Bosch, , X, Marrugat, J; Sanchis, J	2013
Physical rehabilitation approaches for the recovery of function and mobility following stroke	Pollock, , A, Baer, G, Campbell, P, Choo, PL, Forster, A, Morris, J, Pomeroy, VM; Langhorne, P	2014
Piggy-back graft for liver transplantation	Gurusamy, , KS, Pamecha, V; Davidson, BR	2011
Polysaccharide vaccines for preventing serogroup A meningococcal meningitis	Patel, , M; Lee, CK	2005
Personally tailored activities for improving psychosocial outcomes for people with dementia in long-term care	Möhler, , R, Renom, A, Renom, H; Meyer, G	2018
Photodynamic therapy for recurrent respiratory papillomatosis	Lieder, , A, Khan, MK; Lippert, BM	2014
Postnatal thyroid hormones for preterm infants with transient hypothyroxinaemia	Osborn, , DA; Hunt, R	2007
Pharmacological interventions for sleepiness and sleep disturbances caused by shift work	Liira, , J, Verbeek, JH, Costa, G, Driscoll, TR, Sallinen, M, Isotalo, LK; Ruotsalainen, JH	2014
Pharmacological interventions for those who have sexually offended or are at risk of offending	Khan, , O, Ferriter, M, Huband, N, Powney, MJ, Dennis, JA; Duggan, C	2015
Piracetam for dementia or cognitive impairment	Flicker, , L; Grimley Evans, J	2004
Polyclonal anti-thymocyte globulins for the prophylaxis of graft-versus-host disease after allogeneic stem cell or bone marrow transplantation in adults	Theurich, , S, Fischmann, H, Shimabukuro-Vornhagen, A, Chemnitz, JM, Holtick, U, Scheid, C, Skoetz, N; von Bergwelt-Baildon, M	2012
Pharmacological interventions for generalised itching (not caused by systemic disease or skin lesions) in pregnancy	Rungsiprakarn, , P, Laopaiboon, M, Sangkomkamhang, US; Lumbiganon, P	2016
Polyunsaturated fatty acids (PUFA) for attention deficit hyperactivity disorder (ADHD) in children and adolescents	Gillies, , D, Sinn, JKH, Lad, SS, Leach, MJ; Ross, MJ	2012
Pit and fissure sealants versus fluoride varnishes for preventing dental decay in the permanent teeth of children and adolescents	Ahovuo-Saloranta, , A, Forss, H, Hiiri, A, Nordblad, A; Mäkelä, M	2016
Post-operative radiotherapy for ductal carcinoma in situ of the breast	Goodwin, , A, Parker, S, Ghersi, D; Wilcken, N	2013

Positive end-expiratory pressure for preterm infants requiring conventional mechanical ventilation for respiratory distress syndrome or bronchopulmonary dysplasia	Bamat, , N, Fierro, J, Wang, Y, Millar, D; Kirpalani, H	2019
Planned birth at or near term for improving health outcomes for pregnant women with gestational diabetes and their infants	Biesty, , LM, Egan, AM, Dunne, F, Dempsey, E, Meskell, P, Smith, V, Ni Bhuinneain, GM; Devane, D	2018
Pharmacological therapies for maintenance treatments of opium dependence	Rahimi-Movaghar, , A, Amin-Esmaeili, M, Hefazi, M; Yousefi-Nooraie, R	2013
Polyclonal and monoclonal antibodies for treating acute rejection episodes in kidney transplant recipients	Webster, , AC, Wu, S, Tallapragada, K, Park, MY, Chapman, JR; Carr, SJ	2017
Pharmacological interventions for preventing complications in idiopathic hypercalciuria	Escribano, , J, Balaguer, A, Pagone, F, Feliu, A; Roqué i Figuls, M	2009
Plasma exchange for chronic inflammatory demyelinating polyradiculoneuropathy	Mehndiratta, , MM, Hughes, RAC; Pritchard, J	2015
Phyllanthus species for chronic hepatitis B virus infection	Xia, , Y, Luo, H, Liu, JP; Gluud, C	2011
Pharmacological and nutritional treatment for McArdle disease (Glycogen Storage Disease type V)	Quinlivan, , R, Martinuzzi, A; Schoser, B	2014
Peritoneal drainage versus laparotomy as initial surgical treatment for perforated necrotizing enterocolitis or spontaneous intestinal perforation in preterm low birth weight infants	Rao, , SC, Basani, L, Simmer, K, Samnakay, N; Deshpande, G	2011
Personalised digital interventions for reducing hazardous and harmful alcohol consumption in community-dwelling populations	Kaner, , EFS, Beyer, FR, Garnett, C, Crane, D, Brown, J, Muirhead, C, Redmore, J, O'Donnell, A, Newham, JJ, de Vocht, F, Hickman, M, Brown, H, Maniatopoulos, G; Michie, S	2017
Pharmacological interventions for somatoform disorders in adults	Kleinstäuber, , M, Witthöft, M, Steffanowski, A, van Marwijk, H, Hiller, W; Lambert, MJ	2014
Pool fencing for preventing drowning of children	Thompson, , DC; Rivara, F	1998
Physical fitness training for stroke patients	Saunders, , DH, Sanderson, M, Hayes, S, Kilrane, M, Greig, CA, Brazzelli, M; Mead, GE	2016
Pharmacological interventions for hypertension in children	Chaturvedi, , S, Lipszyc, DH, Licht, C, Craig, JC; Parekh, R	2014
Powered versus manual toothbrushing for oral health	Yaacob, , M, Worthington, HV, Deacon, SA, Deery, C, Walmsley, AD, Robinson, PG; Glenny, AM	2014
Pharmacotherapy for Behcet's syndrome	Saenz, , A, Ausejo, M, Shea, B, Wells, GA, Welch, V; Tugwell, P	1998
Pharmacological treatments for Friedreich ataxia	Kearney, , M, Orrell, RW, Fahey, M, Brassington, R; Pandolfo, M	2016
Personalised asthma action plans for adults with asthma	Gatheral, , TL, Rushton, A, Evans, DJW, Mulvaney, CA, Halcovitch, NR, Whiteley, G, Eccles, FJR; Spencer, S	2017
Pharmacological treatment for depression during opioid agonist treatment for opioid dependence	Pani, , PP, Vacca, R, Trogu, E, Amato, L; Davoli, M	2010
Positron emission tomography-adapted therapy for first-line treatment in individuals with Hodgkin lymphoma	Sickinger, , MT, von Tresckow, B, Kobe, C, Engert, A, Borchmann, P; Skoetz, N	2015
Posterior versus lateral surgical approach for total hip arthroplasty in adults with osteoarthritis	Jolles, , BM; Bogoch, ER	2006
Plasma exchange for generalised myasthenia gravis	Gajdos, , P, Chevret, S; Toyka, KV	2002
Postoperative radiotherapy for non-small cell lung cancer	Burdett, , S, Rydzewska, L, Tierney, J, Fisher, D, Parmar, MKB, Arriagada, R, Pignon, JP; Le Pechoux, C	2016
Peri-operative glycaemic control regimens for preventing surgical site infections in adults	Kao, , LS, Meeks, D, Moyer, VA; Lally, KP	2009
Planned early delivery versus expectant management of the term suspected compromised baby for improving outcomes	Bond, , DM, Gordon, A, Hyett, J, de Vries, B, Carberry, AE; Morris, J	2015
Physical activity programs for promoting bone mineralization and growth in preterm infants	Schulzke, , SM, Kaempfen, S, Trachsel, D; Patole, SK	2014
Pharmacological interventions for acute pancreatitis	Moggia, , E, Koti, R, Belgaumkar, AP, Fazio, F, Pereira, SP, Davidson, BR; Gurusamy, KS	2017
Pharmacologic treatment of depression in multiple sclerosis	Koch, , MW, Glazenborg, A, Uyttenboogaart, M, Mostert, J; De Keyser, J	2011
Peri-implantation glucocorticoid administration for assisted reproductive technology cycles	Boomsma, , CM, Keay, SD; Macklon, NS	2012
Pneumococcal vaccines for children and adults with bronchiectasis	Chang, , CC, Singleton, RJ, Morris, PS; Chang, AB	2009
Plasma volume expansion for treatment of pre-eclampsia	Duley, , L, Williams, J; Henderson-Smart, DJ	1999
Pneumococcal vaccine for asthma	Sheikh, , A, Alves, B; Dhami, S	2002
Pharmacological interventions for self-injurious behaviour in adults with intellectual disabilities	Rana, , F, Gormez, A; Varghese, S	2013
Planned caesarean section versus planned vaginal birth for severe pre-eclampsia	Amorim, , MMR, Souza, ASR; Katz, L	2017
Peroxisome proliferator-activated receptor gamma agonists for preventing recurrent stroke and other vascular events in people with stroke or transient ischaemic attack	Liu, , J; Wang, LN	2017
Perphenazine for schizophrenia	Hartung, , B, Sampson, S; Leucht, S	2015
Physical activity for women with breast cancer after adjuvant therapy	Lahart, , IM, Metsios, GS, Nevill, AM; Carmichael, AR	2018
Placebo response and remission rates in randomised trials of induction and maintenance therapy for ulcerative colitis	Jairath, , V, Zou, GY, Parker, CE, MacDonald, JK, AlAmeel, T, Al Beshir, M, Almadi, MA, Al-Taweel, T, Atkinson, NSS,	2017

	Biswas, S, Chapman, T, Dulai, PS, Glaire, MA, Hoekman, DR, Koutsoumpas, A, Minas, E, Mosli, MH, Samaan, M, Khanna, R, Travis, S, D'Haens, G, Sandborn, WJ; Feagan, BG	
Positive expiratory pressure physiotherapy for airway clearance in people with cystic fibrosis	McIlwaine, , M, Button, B; Dwan, K	2015
Pharmacological agents for the prevention of vestibular migraine	Maldonado Fernández, , M, Birdi, JS, Irving, GJ, Murdin, L, Kivekäs, I; Strupp, M	2015
Physical training for McArdle disease	Quinlivan, , R, Vissing, J, Hilton-Jones, D; Buckley, J	2011
Plasma and cerebrospinal fluid amyloid beta for the diagnosis of Alzheimer's disease dementia and other dementias in people with mild cognitive impairment (MCI)	Ritchie, , C, Smailagic, N, Noel-Storr, AH, Takwoingi, Y, Flicker, L, Mason, SE; McShane, R	2014
Physician anaesthetists versus non-physician providers of anaesthesia for surgical patients	Lewis, , SR, Nicholson, A, Smith, AF; Alderson, P	2014
Pharmacological interventions for pain in patients with temporomandibular disorders	Mujakperuo, , HR, Watson, M, Morrison, R; Macfarlane, TV	2010
Pneumococcal vaccines for sickle cell disease	Davies, , EG, Hirst, C, Lottenberg, R; Dower, N	2004
Population-based biomedical sexually transmitted infection control interventions for reducing HIV infection	Ng, , BE, Butler, LM, Horvath, T; Rutherford, GW	2011
Perioperative nutrition for the treatment of bladder cancer by radical cystectomy	Burden, , S, Billson, HA, Lal, S, Owen, KA; Muneer, A	2019
Physician advice for smoking cessation	Stead, , LF, Buitrago, D, Preciado, N, Sanchez, G, Hartmann-Boyce, J; Lancaster, T	2013
Plasma volume expansion for suspected impaired fetal growth	Say, , L, Gülmezoglu, AM; Hofmeyr, GJ	1996
Pharmaceutical policies: effects of restrictions on reimbursement	Green, , CJ, Maclure, M, Fortin, PM, Ramsay, CR, Aaserud, M; Bardal, S	2010
Polyunsaturated fatty acids (PUFAs) for children with specific learning disorders	Tan, , ML, Ho, JJ; Teh, KH	2016
Polyclonal and monoclonal antibodies for induction therapy in kidney transplant recipients	Hill, , P, Cross, NB, Barnett, ANR, Palmer, SC; Webster, AC	2017
Pharmacological interventions for the treatment of delirium in critically ill adults	Burry, , L, Hutton, B, Williamson, DR, Mehta, S, Adhikari, NKJ, Cheng, W, Ely, EW, Egerod, I, Fergusson, DA; Rose, L	2019
Positron emission tomography (PET) and magnetic resonance imaging (MRI) for assessing tumour resectability in advanced epithelial ovarian/fallopian tube/primary peritoneal cancer	Roze, , JF, Hoogendam, JP, van de Wetering, FT, Spijker, R, Verleye, L, Vlayen, J, Veldhuis, WB, Scholten, RJPM; Zweemer, RP	2018
Population-level interventions in government jurisdictions for dietary sodium reduction	McLaren, , L, Sumar, N, Barberio, AM, Trieu, K, Lorenzetti, DL, Tarasuk, V, Webster, J; Campbell, NRC	2016
Pharmacotherapies that specifically target ammonia for the prevention and treatment of hepatic encephalopathy in adults with cirrhosis	Zacharias, , HD, Zacharias, AP, Gluud, LL; Morgan, MY	2019
Pharmacological interventions for clozapine-induced sinus tachycardia	Lally, , J, Docherty, MJ; MacCabe, JH	2016
Planned home versus hospital care for preterm prelabour rupture of the membranes (PPROM) prior to 37 weeks' gestation	Abou El Senoun, , G, Dowswell, T; Mousa, HA	2014
Post-surgical chemotherapy versus surgery alone for resectable gastric cancer	Diaz-Nieto, , R, Orti-Rodríguez, R; Winslet, M	2013
Pharmacological interventions versus no pharmacological intervention for ischaemia reperfusion injury in liver resection surgery performed under vascular control	Abu-Amara, , M, Gurusamy, KS, Hori, S, Glantzounis, G, Fuller, B; Davidson, BR	2009
Personalised risk communication for informed decision making about taking screening tests	Edwards, , AGK, Naik, G, Ahmed, H, Elwyn, GJ, Pickles, T, Hood, K; Playle, R	2013
Personal protective equipment for preventing highly infectious diseases due to exposure to contaminated body fluids in healthcare staff	Verbeek, , JH, Rajamaki, B, Ijaz, S, Tikka, C, Ruotsalainen, JH, Edmond, MB, Sauni, R; Kilinc Balci, FS	2019
Permissive hypoxaemia versus normoxaemia for mechanically ventilated critically ill patients	Gilbert-Kawai, , ET, Mitchell, K, Martin, D, Carlisle, J; Grocott, MPW	2014
Pharmacological interventions for preventing dry mouth and salivary gland dysfunction following radiotherapy	Riley, , P, Glenny, AM, Hua, F; Worthington, HV	2017
Phlebotonics for haemorrhoids	Perera, , N, Liolitsa, D, Iype, S, Croxford, A, Yassin, M, Lang, P, Ukaegbu, O; van Issum, C	2012
Potentiators (specific therapies for class III and IV mutations) for cystic fibrosis	Skilton, , M, Krishan, A, Patel, S, Sinha, IP; Southern, KW	2019
Pharmacological interventions for borderline personality disorder	Stoffers, , J, Völlm, BA, Rücker, G, Timmer, A, Huband, N; Lieb, K	2010
Pharmacological treatment other than corticosteroids, intravenous immunoglobulin and plasma exchange for Guillain-Barré syndrome	Pritchard, , J, Hughes, RAC, Hadden, RDM; Brassington, R	2016
Pharmacological interventions for acute hepatitis C infection	Kalafateli, , M, Buzzetti, E, Thorburn, D, Davidson, BR, Tsochatzis, E; Gurusamy, KS	2018
Physical rehabilitation for critical illness myopathy and neuropathy	Mehrholz, , J, Pohl, M, Kugler, J, Burridge, J, Mückel, S; Elsner, B	2015
Physical methods for preventing deep vein thrombosis in stroke	Naccarato, , M, Chiodo Grandi, F, Dennis, M; Sandercock, PAG	2010
Physical exercise training for type 3 spinal muscular atrophy	Bartels, , B, Montes, J, van der Pol, WL; de Groot, JF	2019
Pharmacological interventions for apathy in Alzheimer's disease	Ruthirakuhan, , MT, Herrmann, N, Abraham, EH, Chan, S; Lanctôt, KL	2018
Pharyngeal instillation of surfactant before the first breath for prevention of morbidity and mortality in preterm infants at risk of respiratory distress syndrome	Abdel-Latif, , ME; Osborn, DA	2011

Photorefractive keratectomy (PRK) versus laser assisted in situ keratomileusis (LASIK) for hyperopia correction	Settas, , G, Settas, C, Minos, E; Yeung, IYL	2012
Pneumococcal conjugate vaccines for preventing acute otitis media in children	Fortanier, , AC, Venekamp, RP, Boonacker, CWB, Hak, E, Schilder, AGM, Sanders, EAM; Damoiseaux, RAMJ	2019
Pharmaceutical policies: effects of reference pricing, other pricing, and purchasing policies	Acosta, , A, Ciapponi, A, Aaserud, M, Vietto, V, Austvoll-Dahlgren, A, Kösters, JP, Vacca, C, Machado, M, Diaz Ayala, DH; Oxman, AD	2014
Plasma expanders for people with cirrhosis and large ascites treated with abdominal paracentesis	Simonetti, , RG, Perricone, G, Nikolova, D, Bjelakovic, G; Gluud, C	2019
Pharmacological treatment for psychotic depression	Wijkstra, , J, Lijmer, J, Burger, H, Cipriani, A, Geddes, J; Nolen, WA	2015
Pre and peri-operative erythropoeitin for reducing allogeneic blood transfusions in colorectal cancer surgery.	Devon, , KM; McLeod, RS	2009
PET-CT for assessing mediastinal lymph node involvement in patients with suspected resectable non-small cell lung cancer	Schmidt-Hansen, , M, Baldwin, DR, Hasler, E, Zamora, J, Abraira, V; Roqué i Figuls, M	2014
Pharmacological treatments for preventing epilepsy following traumatic head injury	Thompson, , K, Pohlmann-Eden, B, Campbell, LA; Abel, H	2015
Physical exercise for people with cirrhosis	Aamann, , L, Dam, G, Rinnov, AR, Vilstrup, H; Gluud, LL	2018
Platelet-rich-plasmapheresis for minimising peri-operative allogeneic blood transfusion	Carless, , PA, Rubens, FD, Anthony, DM, O'Connell, D; Henry, DA	2011
Prazosin for Raynaud's phenomenon in progressive systemic sclerosis	Harding, , SE, Tingey, PC, Pope, J, Fenlon, D, Furst, D, Shea, B, Silman, A, Thompson, A; Wells, GA	1998
Phosphodiesterase inhibitors for lower urinary tract symptoms consistent with benign prostatic hyperplasia	Pattanaik, , S, Mavuduru, RS, Panda, A, Mathew, JL, Agarwal, MM, Hwang, EC, Lyon, JA, Singh, SK; Mandal, AK	2018
Pharmacological interventions for the treatment of anxiety disorders in chronic obstructive pulmonary disease	Usmani, , ZA, Carson, KV, Cheng, JN, Esterman, AJ; Smith, BJ	2011
Pharmacological interventions for promoting smoking cessation during pregnancy	Coleman, , T, Chamberlain, C, Davey, MA, Cooper, SE; Leonardi-Bee, J	2015
Pet allergen control measures for allergic asthma in children and adults	Kilburn, , SA, Lasserson, TJ; McKean, MC	2001
Postoperative epidural analgesia versus systemic analgesia for thoraco-lumbar spine surgery in children	Guay, , J, Suresh, S, Kopp, S; Johnson, RL	2019
Postnatal phenobarbital for the prevention of intraventricular haemorrhage in preterm infants	Smit, , E, Odd, D; Whitelaw, A	2013
Post-exposure passive immunisation for preventing measles	Young, , MK, Nimmo, GR, Cripps, AW; Jones, MA	2014
Pharmacological treatment for antipsychotic-related constipation	Every-Palmer, , S, Newton-Howes, G; Clarke, MJ	2017
Peripheral retinal ablation for threshold retinopathy of prematurity in preterm infants	Andersen, , C; Phelps, D	1999
Platinum-induced hearing loss after treatment for childhood cancer	van As, , JW, van den Berg, H; van Dalen, EC	2016
Pharmacological interventions for the treatment of depression in chronic obstructive pulmonary disease	Pollok, , J, van Agteren, JEM; Carson-Chahhoud, KV	2018
Policies for replacing long-term indwelling urinary catheters in adults	Cooper, , FPM, Alexander, CE, Sinha, S; Omar, MI	2016
Pharmacotherapy for mild hypertension	Diao, , D, Wright, JM, Cundiff, DK; Gueyffier, F	2012
Pharmacotherapy for hyperuricemia in hypertensive patients	Gois, , PHF; Souza, ERDM	2017
Polyunsaturated fatty acid supplementation in infancy for the prevention of allergy	Schindler, , T, Sinn, JKH; Osborn, DA	2016
Piperonyl butoxide (PBO) combined with pyrethroids in insecticide-treated nets to prevent malaria in Africa	Gleave, , K, Lissenden, N, Richardson, M, Choi, L; Ranson, H	2018
Pharmaceutical policies: effects of financial incentives for prescribers	Rashidian, , A, Omidvari, AH, Vali, Y, Sturm, H; Oxman, AD	2015
Phosphodiesterase 5 inhibitors for pulmonary hypertension	Barnes, , H, Brown, Z, Burns, A; Williams, T	2019
Pharmacological interventions for recurrent abdominal pain in childhood	Martin, , AE, Newlove-Delgado, TV, Abbott, RA, Bethel, A, Thompson-Coon, J, Whear, R; Logan, S	2017
Phenobarbital prior to preterm birth for preventing neonatal periventricular haemorrhage	Crowther, , CA; Crosby, DD	2010
Positive end-expiratory pressure for resuscitation of newborn infants at birth	O'Donnell, , CPF, Davis, PG; Morley, CJ	2003
Perioperative nutrition interventions for women with ovarian cancer	Billson, , HA, Holland, C, Curwell, J, Davey, VL, Kinsey, L, Lawton, LJ, Whitworth, AJ; Burden, S	2013
Positive pressure therapy for Ménière's disease or syndrome	van Sonsbeek, , S, Pullens, B; van Benthem, PP	2015
Positional therapy for obstructive sleep apnoea	Srijithesh, , PR, Aghoram, R, Goel, A; Dhanya, J	2019
Polymerase chain reaction blood tests for the diagnosis of invasive aspergillosis in immunocompromised people	Cruciani, , M, Mengoli, C, Barnes, R, Donnelly, JP, Loeffler, J, Jones, BL, Klingspor, L, Maertens, J, Morton, CO; White, LP	2019
Pharmacological interventions for prevention and treatment of upper gastrointestinal bleeding in newborn infants	Green, , DS, Abdel-Latif, ME, Jones, LJ, Lui, K; Osborn, DA	2019
Pharmacotherapies for cannabis dependence	Nielsen, , S, Gowing, L, Sabioni, P; Le Foll, B	2019
Polyunsaturated fatty acids for the primary and secondary prevention of cardiovascular disease	Abdelhamid, , AS, Martin, N, Bridges, C, Brainard, JS, Wang, X, Brown, TJ, Hanson, S, Jimoh, OF, Ajabnoor, SM, Deane, KHO, Song, F; Hooper, L	2018

Phosphate binders for preventing and treating chronic kidney disease-mineral and bone disorder (CKD-MBD)	Ruospo, , M, Palmer, SC, Natale, P, Craig, JC, Vecchio, M, Elder, GJ; Strippoli, GFM	2018
Prebiotics for the prevention of hyperbilirubinaemia in neonates	Armanian, , AM, Jahanfar, S, Feizi, A, Salehimehr, N, Molaeinezhad, M; Sadeghi, E	2019
Pharmaceutical interventions for emotionalism after stroke	Allida, , S, Patel, K, House, A; Hackett, ML	2019
Psychosocial and psychological interventions for preventing postpartum depression	Dennis, , CL; Dowswell, T	2013
Resection versus no intervention or other surgical interventions for colorectal cancer liver metastases	Fedorowicz, , Z, Lodge, M, Al-asfoor, A; Carter, B	2008
Rest during pregnancy for preventing pre-eclampsia and its complications in women with normal blood pressure	Meher, , S; Duley, L	2006
Rectal 5-aminosalicylic acid for maintenance of remission in ulcerative colitis	Marshall, , JK, Thabane, M, Steinhart, AH, Newman, JR, Anand, A; Irvine, EJ	2012
Radioiodine treatment for pediatric Graves' disease	Ma, , C, Kuang, A, Xie, J; Liu, GJ	2008
Rosiglitazone for type 2 diabetes mellitus	Richter, , B, Bandeira-Echtler, E, Bergerhoff, K, Clar, C; Ebrahim, SH	2007
Remote ischaemic preconditioning for coronary artery bypass grafting (with or without valve surgery)	Benstoem, , C, Stoppe, C, Liakopoulos, OJ, Ney, J, Hasenclever, D, Meybohm, P; Goetzenich, A	2017
Renal denervation for resistant hypertension	Coppolino, , G, Pisano, A, Rivoli, L; Bolignano, D	2017
Resistance exercise training for fibromyalgia	Busch, , AJ, Webber, SC, Richards, RS, Bidonde, J, Schachter, CL, Schafer, LA, Danyliw, A, Sawant, A, Dal Bello-Haas, V, Rader, T; Overend, TJ	2013
Rimonabant for overweight or obesity	Curioni, , C; André, C	2006
Rituximab, ofatumumab and other monoclonal anti-CD20 antibodies for chronic lymphocytic leukaemia	Bauer, , K, Rancea, M, Roloff, V, Elter, T, Hallek, M, Engert, A; Skoetz, N	2012
Rectal 5-aminosalicylic acid for induction of remission in ulcerative colitis	Marshall, , JK, Thabane, M, Steinhart, AH, Newman, JR, Anand, A; Irvine, EJ	2010
Remote and web 2.0 interventions for promoting physical activity	Foster, , C, Richards, J, Thorogood, M; Hillsdon, M	2013
Routine vitamin A supplementation for the prevention of blindness due to measles infection in children	Bello, , S, Meremikwu, MM, Ejemot-Nwadiaro, RI; Oduwole, O	2016
Radiofrequency (thermal) ablation versus no intervention or other interventions for hepatocellular carcinoma	Weis, , S, Franke, A, Mössner, J, Jakobsen, JC; Schoppmeyer, K	2013
Pyridoxine (vitamin B6) supplementation during pregnancy or labour for maternal and neonatal outcomes	Salam, , RA, Zuberi, NF; Bhutta, ZA	2015
Risk scoring for the primary prevention of cardiovascular disease	Karmali, , KN, Persell, SD, Perel, P, Lloyd-Jones, DM, Berendsen, MA; Huffman, MD	2017
Remote ischaemic conditioning for preventing and treating ischaemic stroke	Zhao, , W, Zhang, J, Sadowsky, MG, Meng, R, Ding, Y; Ji, X	2018
Routine neonatal circumcision for the prevention of urinary tract infections in infancy	Jagannath, , VA, Fedorowicz, Z, Sud, V, Verma, AK; Hajebrahimi, S	2012
Recombinant growth hormone for children and adolescents with Turner syndrome	Baxter, , L, Bryant, J, Cave, CB; Milne, R	2007
Puerarin for ischaemic stroke	Liu, , B, Tan, Y, Wang, D; Liu, M	2016
Radioiodine therapy for differentiated thyroid carcinoma with thyroglobulin positive and radioactive iodine negative metastases	Ma, , C, Kuang, A; Xie, J	2009
Role of chemotherapy additional to high-dose methotrexate for primary central nervous system lymphoma (PCNSL)	Bergner, , N, Monsef, I, Illerhaus, G, Engert, A; Skoetz, N	2012
Radical multimodality therapy for malignant pleural mesothelioma	Abdel-Rahman, , O, Elsayed, Z, Mohamed, H; Eltobgy, M	2018
Rifamycins (rifampicin, rifabutin and rifapentine) compared to isoniazid for preventing tuberculosis in HIV-negative people at risk of active TB	Sharma, , SK, Sharma, A, Kadhiravan, T; Tharyan, P	2013
Routine oro/nasopharyngeal suction versus no suction at birth	Foster, , JP, Dawson, JA, Davis, PG; Dahlen, HG	2017
Radiotherapy for diffuse brainstem glioma in children and young adults	Hu, , X, Fang, Y, Hui, X, Jv, Y; You, C	2016
Recombinant human insulin-like growth factor I (rhIGF-I) for the treatment of amyotrophic lateral sclerosis/motor neuron disease	Beauverd, , M, Mitchell, JD, Wokke, JHJ; Borasio, GD	2012
Rituximab for thyroid-associated ophthalmopathy	Minakaran, , N; Ezra, DG	2013
Radiotherapy versus open surgery versus endolaryngeal surgery (with or without laser) for early laryngeal squamous cell cancer	Warner, , L, Chudasama, J, Kelly, CG, Loughran, S, McKenzie, K, Wight, R; Dey, P	2014
Rapid diagnostic tests versus clinical diagnosis for managing people with fever in malaria endemic settings	Odaga, , J, Sinclair, D, Lokong, JA, Donegan, S, Hopkins, H; Garner, P	2014
Safety of regular formoterol or salmeterol in adults with asthma: an overview of Cochrane reviews	Cates, , CJ, Wieland, LS, Oleszczuk, M; Kew, KM	2014
Red-light cameras for the prevention of road traffic crashes	Aeron-Thomas, , A; Hess, S	2005
Regular treatment with formoterol and an inhaled corticosteroid versus regular treatment with salmeterol and an inhaled corticosteroid for chronic asthma: serious adverse events	Cates, , CJ; Lasserson, TJ	2010
Regular treatment with salmeterol for chronic asthma: serious adverse events	Cates, , CJ; Cates, MJ	2008
Routine blood cultures in the management of pyelonephritis in pregnancy for improving outcomes	Gomi, , H, Goto, Y, Laopaiboon, M, Usui, R; Mori, R	2015

Rapid diagnostic tests for typhoid and paratyphoid (enteric) fever	Wijedoru, , L, Mallett, S; Parry, CM	2017
Rubber band ligation versus excisional haemorrhoidectomy for haemorrhoids	Shanmugam, , V, Hakeem, A, Campbell, KL, Rabindranath, KS, Steele, RJC, Thaha, MA; Loudon, MA	2005
Repeated use of pre- and postcoital hormonal contraception for prevention of pregnancy	Halpern, , V, Raymond, EG; Lopez, LM	2014
Recombinant growth hormone for idiopathic short stature in children and adolescents	Bryant, , J, Baxter, L, Cave, CB; Milne, R	2007
Recombinant factor VIIa concentrate versus plasma-derived concentrates for treating acute bleeding episodes in people with haemophilia and inhibitors	Matino, , D, Makris, M, Dwan, K, D'Amico, R; Iorio, A	2015
Regular treatment with formoterol versus regular treatment with salmeterol for chronic asthma: serious adverse events	Cates, , CJ; Lasserson, TJ	2012
Relaxation therapies for the management of primary hypertension in adults	Dickinson, , HO, Beyer, FR, Ford, GA, Nicolson, D, Campbell, F, Cook, JV; Mason, J	2008
Reminder packaging for improving adherence to self-administered long-term medications	Mahtani, , KR, Heneghan, CJ, Glasziou, PP; Perera, R	2011
Safety of non-steroidal anti-inflammatory drugs, including aspirin and paracetamol (acetaminophen) in people receiving methotrexate for inflammatory arthritis (rheumatoid arthritis, ankylosing spondylitis, psoriatic arthritis, other spondyloarthritis)	Colebatch, , AN, Marks, JL; Edwards, CJ	2011
Routine anticonvulsants for treating cerebral malaria	Meremikwu, , MM; Marson, AG	2002
Risk assessment tools for the prevention of pressure ulcers	Moore, , ZEH; Patton, D	2019
Recombinant factor VIIa for the prevention and treatment of bleeding in patients without haemophilia	Simpson, , E, Lin, Y, Stanworth, S, Birchall, J, Doree, C; Hyde, C	2012
Retention procedures for stabilising tooth position after treatment with orthodontic braces	Littlewood, , SJ, Millett, DT, Doubleday, B, Bearn, DR; Worthington, HV	2016
Routine drainage for orthotopic liver transplantation	Gurusamy, , KS, Naik, P; Davidson, BR	2011
Respite care for people with dementia and their carers	Maayan, , N, Soares-Weiser, K; Lee, H	2014
Regular treatment with long acting beta agonists versus daily regular treatment with short acting beta agonists in adults and children with stable asthma	Walters, , EH, Walters, JAE; Gibson, PG	2002
Rigid dressings versus soft dressings for transtibial amputations	Kwah, , LK, Webb, MT, Goh, L; Harvey, LA	2019
Rivastigmine for vascular cognitive impairment	Birks, , J, McGuinness, B; Craig, D	2013
Route of antibiotic prophylaxis for prevention of cerebrospinal fluid-shunt infection	Arts, , SHHMJ, Boogaarts, HD; van Lindert, EJ	2019
Psychosocial interventions for benzodiazepine harmful use, abuse or dependence	Darker, , CD, Sweeney, BP, Barry, JM, Farrell, MF; Donnelly-Swift, E	2015
Regional analgesia for improvement of long-term functional outcome after elective large joint replacement	Atchabahian, , A, Schwartz, G, Hall, CB, Lajam, CM; Andreae, MH	2015
Risedronate for the primary and secondary prevention of osteoporotic fractures in postmenopausal women	Wells, , GA, Cranney, A, Peterson, J, Boucher, M, Shea, B, Welch, V, Coyle, D; Tugwell, P	2008
Relaxin for preventing preterm birth	Bain, , E, Heatley, E, Hsu, K; Crowther, CA	2013
Regional Cerebral Blood Flow Single Photon Emission Computed Tomography for detection of Frontotemporal dementia in people with suspected dementia	Archer, , HA, Smailagic, N, John, C, Holmes, RB, Takwoingi, Y, Coulthard, EJ; Cullum, S	2015
Regular treatment with formoterol for chronic asthma: serious adverse events	Cates, , CJ; Cates, MJ	2012
Pyronaridine-artesunate for treating uncomplicated Plasmodium falciparum malaria	Pryce, , J; Hine, P	2019
Retinoic acid postconsolidation therapy for high-risk neuroblastoma patients treated with autologous haematopoietic stem cell transplantation	Peinemann, , F, van Dalen, EC, Enk, H; Berthold, F	2017
Remediating buildings damaged by dampness and mould for preventing or reducing respiratory tract symptoms, infections and asthma	Sauni, , R, Verbeek, JH, Uitti, J, Jauhiainen, M, Kreiss, K; Sigsgaard, T	2015
Restricting oral fluid and food intake during labour	Singata, , M, Tranmer, J; Gyte, GML	2013
Removal of nail polish and finger rings to prevent surgical infection	Arrowsmith, , VA; Taylor, R	2014
QTc interval screening for cardiac risk in methadone treatment of opioid dependence	Pani, , PP, Trogu, E, Maremmani, I; Pacini, M	2013
Radiotherapy for neovascular age-related macular degeneration	Evans, , JR, Sivagnanavel, V; Chong, V	2010
Salbutamol for transient tachypnea of the newborn	Moresco, , L, Bruschettini, M, Cohen, A, Gaiero, A; Calevo, MG	2016
Psychosocial and psychological interventions for treating antenatal depression	Dennis, , CL, Ross, LE; Grigoriadis, S	2007
Psychosocial interventions for supporting women to stop smoking in pregnancy	Chamberlain, , C, O'Mara-Eves, A, Porter, J, Coleman, T, Perlen, SM, Thomas, J; McKenzie, JE	2017
Repeated lumbar or ventricular punctures in newborns with intraventricular haemorrhage	Whitelaw, , A; Lee-Kelland, R	2017
Risk of ovarian cancer in women treated with ovarian stimulating drugs for infertility	Rizzuto, , I, Behrens, RF; Smith, LA	2019

Repeat doses of prenatal corticosteroids for women at risk of preterm birth for improving neonatal health outcomes	Crowther, , CA, McKinlay, CJD, Middleton, P; Harding, JE	2015
Rehabilitation following carpal tunnel release	Peters, , S, Page, MJ, Coppieters, MW, Ross, M; Johnston, V	2016
Rapid initiation of antiretroviral therapy for people living with HIV	Mateo-Urdiales, , A, Johnson, S, Smith, R, Nachega, JB; Eshun-Wilson, I	2019
Reminder systems to improve patient adherence to tuberculosis clinic appointments for diagnosis and treatment	Liu, , Q, Abba, K, Alejandria, MM, Sinclair, D, Balanag, VM; Lansang, MAD	2014
Repetitive task training for improving functional ability after stroke	French, , B, Thomas, LH, Coupe, J, McMahon, NE, Connell, L, Harrison, J, Sutton, CJ, Tishkovskaya, S; Watkins, CL	2016
Psychosocial interventions for patients with head and neck cancer	Semple, , C, Parahoo, K, Norman, A, McCaughan, E, Humphris, G; Mills, M	2013
Risk-scoring systems for predicting preterm birth with the aim of reducing associated adverse outcomes	Davey, , MA, Watson, L, Rayner, JA; Rowlands, S	2015
Psychosocial interventions for psychostimulant misuse	Minozzi, , S, Saulle, R, De Crescenzo, F; Amato, L	2016
Psychostimulants for hypersomnia (excessive daytime sleepiness) in myotonic dystrophy	Annane, , D, Moore, DH; Miller, RG	2006
Reduction in saturated fat intake for cardiovascular disease	Hooper, , L, Martin, N, Abdelhamid, A; Davey Smith, G	2015
Recombinant human activated protein C for severe sepsis in neonates	Kylat, , RI; Ohlsson, A	2012
Regular long-term red blood cell transfusions for managing chronic chest complications in sickle cell disease	Estcourt, , LJ, Fortin, PM, Hopewell, S, Trivella, M, Hambleton, IR; Cho, G	2016
Radiofrequency ablation in the treatment of liver metastases from colorectal cancer	Cirocchi, , R, Trastulli, S, Boselli, C, Montedori, A, Cavaliere, D, Parisi, A, Noya, G; Abraha, I	2012
Quinolones for uncomplicated acute cystitis in women	Rafalsky, , VV, Andreeva, IV; Rjabkova, EL	2006
Routine intraoperative ureteric stenting for kidney transplant recipients	Wilson, , CH, Rix, DA; Manas, DM	2013
Psychosocial interventions to improve quality of life and emotional wellbeing for recently diagnosed cancer patients	Galway, , K, Black, A, Cantwell, M, Cardwell, CR, Mills, M; Donnelly, M	2012
Reiki for depression and anxiety	Joyce, , J; Herbison, GP	2015
Pulmonary rehabilitation for interstitial lung disease	Dowman, , L, Hill, CJ; Holland, AE	2014
Reduced or modified dietary fat for preventing cardiovascular disease	Hooper, , L, Summerbell, CD, Thompson, R, Sills, D, Roberts, FG, Moore, HJ; Davey Smith, G	2012
Repetitive transcranial magnetic stimulation for improving function after stroke	Hao, , Z, Wang, D, Zeng, Y; Liu, M	2013
Radical prostatectomy versus watchful waiting for prostate cancer	Hegarty, , J, Beirne, PV, Walsh, E, Comber, H, Fitzgerald, T; Wallace Kazer, M	2010
Psychosocial interventions for depression in dialysis patients	Rabindranath, , KS, Daly, C, Butler, J, Roderick, PJ, Wallace, SA; MacLeod, AM	2005
Recombinant growth hormone therapy for X-linked hypophosphatemia in children	Yang, , HM, Mao, M, Yang, F; Wan, C	2005
Risk of endometrial cancer in women treated with ovary-stimulating drugs for subfertility	Skalkidou, , A, Sergentanis, TN, Gialamas, SP, Georgakis, MK, Psaltopoulou, T, Trivella, M, Siristatidis, CS, Evangelou, E; Petridou, E	2017
Red cell transfusion for the management of upper gastrointestinal haemorrhage	Jairath, , V, Hearnshaw, S, Brunskill, SJ, Doree, C, Hopewell, S, Hyde, C, Travis, S; Murphy, MF	2010
Routine versus selective antifungal administration for control of fungal infections in patients with cancer	Gøtzsche, , PC; Johansen, HK	2014
Pulmonary artery perfusion versus no perfusion during cardiopulmonary bypass for open heart surgery in adults	Buggeskov, , KB, Grønlykke, L, Risom, EC, Wei, ML; Wetterslev, J	2018
Pycnogenol® (extract of French maritime pine bark) for the treatment of chronic disorders	Schoonees, , A, Visser, J, Musekiwa, A; Volmink, J	2012
Radiotherapy for malignant pleural mesothelioma	Chapman, , E; García Diéguez, M	2006
Rehabilitation after lumbar disc surgery	Oosterhuis, , T, Costa, LOP, Maher, CG, de Vet, HCW, van Tulder, MW; Ostelo, RWJG	2014
Repetitive transcranial magnetic stimulation for the treatment of amyotrophic lateral sclerosis or motor neuron disease	Fang, , J, Zhou, M, Yang, M, Zhu, C; He, L	2013
Red cell transfusion management for patients undergoing cardiac surgery for congenital heart disease	Wilkinson, , KL, Brunskill, SJ, Doree, C, Trivella, M, Gill, R; Murphy, MF	2014
Rituximab for eradicating inhibitors in people with acquired haemophilia A	Zeng, , Y, Zhou, R, Duan, X; Long, D	2016
Psychosocial interventions for premature ejaculation	Melnik, , T, Althof, S, Atallah, ÁN, Puga, MEDS, Glina, S; Riera, R	2011
Repositioning for treating pressure ulcers	Moore, , ZEH; Cowman, S	2015
Pulmonary rehabilitation following exacerbations of chronic obstructive pulmonary disease	Puhan, , MA, Gimeno-Santos, E, Cates, CJ; Troosters, T	2016
Restricted versus liberal water intake for preventing morbidity and mortality in preterm infants	Bell, , EF; Acarregui, MJ	2014
Radiotherapy and chemoradiation after surgery for early cervical cancer	Rogers, , L, Siu, SSN, Luesley, D, Bryant, A; Dickinson, HO	2012
Recombinant human erythropoietin versus placebo or no treatment for the anaemia of chronic kidney disease in people not requiring dialysis	Cody, , JD; Hodson, EM	2016

Repetitive transcranial magnetic stimulation for tinnitus	Meng, , Z, Liu, S, Zheng, Y; Phillips, JS	2011
Rapid tests for the diagnosis of visceral leishmaniasis in patients with suspected disease	Boelaert, , M, Verdonck, K, Menten, J, Sunyoto, T, van Griensven, J, Chappuis, F; Rijal, S	2014
Pulsatile gonadotrophin releasing hormone for ovulation induction in subfertility associated with polycystic ovary syndrome	Bayram, , N, van Wely, M; Van der Veen, F	2003
Quit and Win contests for smoking cessation	Cahill, , K; Perera, R	2008
Rituximab for relapsing-remitting multiple sclerosis	He, , D, Guo, R, Zhang, F, Zhang, C, Dong, S; Zhou, H	2013
Restricting or banning alcohol advertising to reduce alcohol consumption in adults and adolescents	Siegfried, , N, Pienaar, DC, Ataguba, JE, Volmink, J, Kredo, T, Jere, M; Parry, CDH	2014
Retinoids for preventing the progression of cervical intra-epithelial neoplasia	Helm, , CW, Lorenz, DJ, Meyer, NJ, Rising, WWR; Wulff, JL	2013
Punctal occlusion for dry eye syndrome	Ervin, , AM, Law, A; Pucker, AD	2017
Purine Antagonists for Chronic Lymphocytic Leukaemia	Steurer, , M, Pall, G, Richards, S, Schwarzer, G, Bohlius, J; Greil, R	2006
Relaxation for perimenopausal and postmenopausal symptoms	Saensak, , S, Vutyavanich, T, Somboonporn, W; Srisurapanont, M	2014
Respiratory function monitoring to reduce mortality and morbidity in newborn infants receiving resuscitation	Schmölzer, , GM, Morley, CJ; Davis, PG	2010
Regional (spinal, epidural, caudal) versus general anaesthesia in preterm infants undergoing inguinal herniorrhaphy in early infancy	Jones, , LJ, Craven, PD, Lakkundi, A, Foster, JP; Badawi, N	2015
Ribavirin plus interferon versus interferon for chronic hepatitis C	Brok, , J, Gluud, LL; Gluud, C	2010
Routine or selective carotid artery shunting for carotid endarterectomy (and different methods of monitoring in selective shunting)	Chongruksut, , W, Vaniyapong, T; Rerkasem, K	2014
Restriction of salt, caffeine and alcohol intake for the treatment of Ménière's disease or syndrome	Hussain, , K, Murdin, L; Schilder, AGM	2018
Radix Sophorae flavescentis versus other drugs or herbs for chronic hepatitis B	Liang, , N, Kong, DZ, Lu, CL, Ma, SS, Li, YQ, Nikolova, D, Jakobsen, JC, Gluud, C; Liu, JP	2019
Robotic versus open radical cystectomy for bladder cancer in adults	Rai, , BP, Bondad, J, Vasdev, N, Adshead, J, Lane, T, Ahmed, K, Khan, MS, Dasgupta, P, Guru, K, Chlosta, PL; Aboumarzouk, OM	2019
Routine monitoring of gastric residual for prevention of necrotising enterocolitis in preterm infants	Abiramalatha, , T, Thanigainathan, S; Ninan, B	2019
Psychosocial interventions for recurrent abdominal pain in childhood	Abbott, , RA, Martin, AE, Newlove-Delgado, TV, Bethel, A, Thompson-Coon, J, Whear, R; Logan, S	2017
Safety of topical corticosteroids in pregnancy	Chi, , CC, Wang, SH, Wojnarowska, F, Kirtschig, G, Davies, E; Bennett, C	2015
Risperidone dose for schizophrenia	Li, , C, Xia, J; Wang, J	2009
Rosuvastatin for lowering lipids	Adams, , SP, Sekhon, SS; Wright, JM	2014
Riluzole for amyotrophic lateral sclerosis (ALS)/motor neuron disease (MND)	Miller, , RG, Mitchell, JD; Moore, DH	2012
Respiratory muscle training for cystic fibrosis	Hilton, , N; Solis-Moya, A	2018
Quality of life after rectal resection for cancer, with or without permanent colostomy.	Pachler, , J; Wille-Jørgensen, P	2012
Routine invasive strategies versus selective invasive strategies for unstable angina and non-ST elevation myocardial infarction in the stent era	Fanning, , JP, Nyong, J, Scott, IA, Aroney, CN; Walters, DL	2016
Psychosocial interventions for reducing antipsychotic medication in care home residents	Richter, , T, Meyer, G, Möhler, R; Köpke, S	2012
Ribavirin for treating Crimean Congo haemorrhagic fever	Johnson, , S, Henschke, N, Maayan, N, Mills, I, Buckley, BS, Kakourou, A; Marshall, R	2018
Respiratory muscle training for cervical spinal cord injury	Berlowitz, , DJ; Tamplin, J	2013
Regional versus general anaesthesia for caesarean section	Afolabi, , BB; Lesi, FEA	2012
Recall intervals for oral health in primary care patients	Riley, , P, Worthington, HV, Clarkson, JE; Beirne, PV	2013
Red blood cell transfusion for people undergoing hip fracture surgery	Brunskill, , SJ, Millette, SL, Shokoohi, A, Pulford, EC, Doree, C, Murphy, MF; Stanworth, S	2015
Red flags to screen for vertebral fracture in patients presenting with low-back pain	Williams, , CM, Henschke, N, Maher, CG, van Tulder, MW, Koes, BW, Macaskill, P; Irwig, L	2013
Recompression and adjunctive therapy for decompression illness	Bennett, , MH, Lehm, JP, Mitchell, SJ; Wasiak, J	2012
Pulp treatment for extensive decay in primary teeth	Smaïl-Faugeron, , V, Glenny, AM, Courson, F, Durieux, P, Muller-Bolla, M; Fron Chabouis, H	2018
Recombinant interferon beta or glatiramer acetate for delaying conversion of the first demyelinating event to multiple sclerosis	Clerico, , M, Faggiano, F, Palace, J, Rice, GPA, Tintorè Subirana, M; Durelli, L	2008
Psychosocial interventions for reducing injection and sexual risk behaviour for preventing HIV in drug users	Meader, , N, Li, R, Des Jarlais, DC; Pilling, S	2010
Rheum officinale (a traditional Chinese medicine) for chronic kidney disease	Wang, , H, Song, H, Yue, J, Li, J, Hou, YB; Deng, JL	2012
Rapid COJEC versus standard induction therapies for high-risk neuroblastoma	Peinemann, , F, Kahangire, DA, van Dalen, EC; Berthold, F	2015

Risk of fatal and nonfatal lactic acidosis with metformin use in type 2 diabetes mellitus	Salpeter, , SR, Greyber, E, Pasternak, GA; Salpeter, EE	2010
Re-feeding versus discarding gastric residuals to improve growth in preterm infants	Abiramalatha, , T, Thanigainathan, S; Balakrishnan, U	2019
Robot-assisted surgery in gynaecology	Lawrie, , TA, Liu, H, Lu, D, Dowswell, T, Song, H, Wang, L; Shi, G	2019
Rehabilitation for people with multiple sclerosis: an overview of Cochrane Reviews	Amatya, , B, Khan, F; Galea, M	2019
Risk-reducing medications for primary breast cancer: a network meta-analysis	Mocellin, , S, Goodwin, A; Pasquali, S	2019
Risk-reducing mastectomy for the prevention of primary breast cancer	Carbine, , NE, Lostumbo, L, Wallace, J; Ko, H	2018
Reading aids for adults with low vision	Virgili, , G, Acosta, R, Bentley, SA, Giacomelli, G, Allcock, C; Evans, JR	2018
Risperidone versus olanzapine for schizophrenia	Jayaram, , MB, Hosalli, P; Stroup, TS	2006
Risperidone for attention-deficit hyperactivity disorder in people with intellectual disabilities	Thomson, , A, Maltezos, S, Paliokosta, E; Xenitidis, K	2009
Rapamycin and rapalogs for tuberous sclerosis complex	Sasongko, , TH, Ismail, NFD; Zabidi-Hussin, ZAMH	2016
Reduction of the number of fetuses for women with a multiple pregnancy	Dodd, , JM, Dowswell, T; Crowther, CA	2015
Rofecoxib for rheumatoid arthritis	Garner, , SE, Fidan, D, Frankish, RR, Judd, M, Towheed, T, Tugwell, P; Wells, GA	2005
Psychosocial interventions for cannabis use disorder	Gates, , PJ, Sabioni, P, Copeland, J, Le Foll, B; Gowing, L	2016
Recombinant human interleukin 10 for induction of remission in Crohn's disease	Buruiana, , FE, Solà, I; Alonso-Coello, P	2010
Rivastigmine for Alzheimer's disease	Birks, , JS, Chong, LY; Grimley Evans, J	2015
Rehabilitation for improving automobile driving after stroke	George, , S, Crotty, M, Gelinas, I; Devos, H	2014
Remote ischaemic preconditioning versus no remote ischaemic preconditioning for vascular and endovascular surgical procedures	Desai, , M, Gurusamy, KS, Ghanbari, H, Hamilton, G; Seifalian, AM	2011
Reminder systems for women with previous gestational diabetes mellitus to increase uptake of testing for type 2 diabetes or impaired glucose tolerance	Middleton, , P; Crowther, CA	2014
Rofecoxib for osteoarthritis	Garner, , SE, Fidan, D, Frankish, RR; Maxwell, L	2005
Routine preoperative medical testing for cataract surgery	Keay, , L, Lindsley, K, Tielsch, J, Katz, J; Schein, O	2019
Rituximab for treating inhibitors in people with inherited severe hemophilia	Jiang, , L, Liu, Y, Zhang, L, Santoro, C; Rodriguez, A	2017
Psychosocial interventions for men with prostate cancer	Parahoo, , K, McDonough, S, McCaughan, E, Noyes, J, Semple, C, Halstead, EJ, Neuberger, MM; Dahm, P	2013
Resection versus other treatments for locally advanced pancreatic cancer	Gurusamy, , KS, Kumar, S, Davidson, BR; Fusai, G	2014
Rituximab for rheumatoid arthritis	Lopez-Olivo, , MA, Amezaga Urruela, M, McGahan, L, Pollono, EN; Suarez-Almazor, ME	2015
Recombinant growth hormone therapy for cystic fibrosis in children and young adults	Thaker, , V, Carter, B; Putman, M	2018
Public stewardship of private for-profit healthcare providers in low- and middle-income countries	Wiysonge, , CS, Abdullahi, LH, Ndze, VN; Hussey, GD	2016
Psychosocial interventions for fatigue during cancer treatment with palliative intent	Poort, , H, Peters, M, Bleijenberg, G, Gielissen, MFM, Goedendorp, MM, Jacobsen, P, Verhagen, S; Knoop, H	2017
Psychotropic analgesic nitrous oxide for alcoholic withdrawal states	Gillman, , MA, Lichtigfeld, F; Young, T	2007
Red flags to screen for malignancy in patients with low-back pain	Henschke, , N, Maher, CG, Ostelo, RWJG, de Vet, HCW, Macaskill, P; Irwig, L	2013
Psychosocial interventions to reduce alcohol consumption in concurrent problem alcohol and illicit drug users	Klimas, , J, Fairgrieve, C, Tobin, H, Field, CA, O'Gorman, CSM, Glynn, LG, Keenan, E, Saunders, J, Bury, G, Dunne, C; Cullen, W	2018
Respiratory muscle training for multiple sclerosis	Rietberg, , MB, Veerbeek, JM, Gosselink, R, Kwakkel, G; van Wegen, EEH	2017
Relapse prevention interventions for smoking cessation	Livingstone-Banks, , J, Norris, E, Hartmann-Boyce, J, West, R, Jarvis, M; Hajek, P	2019
Salicylate for the treatment of Kawasaki disease in children	Baumer, , JH, Love, S, Gupta, A, Haines, L, Maconochie, IK; Dua, JS	2006
Reflectance confocal microscopy for diagnosing cutaneous melanoma in adults	Dinnes, , J, Deeks, JJ, Saleh, D, Chuchu, N, Bayliss, SE, Patel, L, Davenport, C, Takwoingi, Y, Godfrey, K, Matin, RN, Patalay, R; Williams, HC	2018
Reflectance confocal microscopy for diagnosing keratinocyte skin cancers in adults	Dinnes, , J, Deeks, JJ, Chuchu, N, Saleh, D, Bayliss, SE, Takwoingi, Y, Davenport, C, Patel, L, Matin, RN, O'Sullivan, C, Patalay, R; Williams, HC	2018
Radix Sophorae flavescentis versus no intervention or placebo for chronic hepatitis B	Liang, , N, Kong, DZ, Ma, SS, Lu, CL, Yang, M, Feng, LD, Shen, C, Diao, RH, Cui, LJ, Lu, XY, Nikolova, D, Jakobsen, JC, Gluud, C; Liu, JP	2019
Supervised dosing with a long-acting opioid medication in the management of opioid dependence	Saulle, , R, Vecchi, S; Gowing, L	2017
Stem cell transplantation for ischemic stroke	Boncoraglio, , GB, Ranieri, M, Bersano, A, Parati, EA; Del Giovane, C	2019

Statins for primary prevention of venous thromboembolism	Li, , L, Zhang, P, Tian, JH; Yang, K	2014
Tacrolimus versus cyclosporin as primary immunosuppression for lung transplant recipients	Penninga, , L, Penninga, EI, Møller, CH, Iversen, M, Steinbrüchel, DA; Gluud, C	2013
Steroidal contraceptives: effect on bone fractures in women	Lopez, , LM, Grimes, DA, Schulz, KF, Curtis, KM; Chen, M	2014
Statins for women with polycystic ovary syndrome not actively trying to conceive	Raval, , AD, Hunter, T, Stuckey, B; Hart, RJ	2011
Surgical techniques for uterine incision and uterine closure at the time of caesarean section	Dodd, , JM, Anderson, ER, Gates, S; Grivell, RM	2014
Surgical decompression for cerebral oedema in acute ischaemic stroke	Cruz-Flores, , S, Berge, E; Whittle, IR	2012
Steroids for traumatic optic neuropathy	Yu-Wai-Man, , P; Griffiths, PG	2013
Surgery for faecal incontinence in adults	Brown, , SR, Wadhawan, H; Nelson, RL	2013
Support during pregnancy for women at increased risk of low birthweight babies	East, , CE, Biro, MA, Fredericks, S; Lau, R	2019
Supplementation with multiple micronutrients for breastfeeding women for improving outcomes for the mother and baby	Abe, , SK, Balogun, OO, Ota, E, Takahashi, K; Mori, R	2016
Surgical hand antisepsis to reduce surgical site infection	Tanner, , J, Dumville, JC, Norman, G; Fortnam, M	2016
Stent placement versus surgery for coarctation of the thoracic aorta	Pádua, , LMS, Garcia, LC, Rubira, CJ; de Oliveira Carvalho, PE	2012
Surgery for obstructive sleep apnoea in adults	Sundaram, , S, Lim, J; Lasserson, TJ	2005
Support for healthy breastfeeding mothers with healthy term babies	McFadden, , A, Gavine, A, Renfrew, MJ, Wade, A, Buchanan, P, Taylor, JL, Veitch, E, Rennie, AM, Crowther, SA, Neiman, S; MacGillivray, S	2017
Structured telephone support or non-invasive telemonitoring for patients with heart failure	Inglis, , SC, Clark, RA, Dierckx, R, Prieto-Merino, D; Cleland, JGF	2015
Surgery for lateral elbow pain	Buchbinder, , R, Johnston, RV, Barnsley, L, Assendelft, WJJ, Bell, SN; Smidt, N	2011
Tai chi for primary prevention of cardiovascular disease	Hartley, , L, Flowers, N, Lee, MS, Ernst, E; Rees, K	2014
Strategies for the discontinuation of humidified high flow nasal cannula (HHFNC) in preterm infants	Farley, , RC, Hough, JL; Jardine, LA	2015
Surgery for limited-stage small-cell lung cancer	Barnes, , H, See, K, Barnett, S; Manser, R	2017
Sweet potato for type 2 diabetes mellitus	Ooi, , CP; Loke, SC	2013
Tai chi for treating rheumatoid arthritis	Han, , A, Judd, M, Welch, V, Wu, T, Tugwell, P; Wells, GA	2004
Surgical management for upper urinary tract transitional cell carcinoma	Rai, , BP, Shelley, M, Coles, B, Biyani, CS, El-Mokadem, I; Nabi, G	2011
Surgery versus radiosurgery for patients with a solitary brain metastasis from non-small cell lung cancer	Fuentes, , R, Bonfill Cosp, X; Expósito Hernandez, J	2006
Supraglottic airway devices versus tracheal intubation for airway management during general anaesthesia in obese patients	Nicholson, , A, Cook, TM, Smith, AF, Lewis, SR; Reed, SS	2013
Tacrolimus (FK506) for induction of remission in refractory ulcerative colitis	Baumgart, , DC, MacDonald, JK; Feagan, B	2008
Structured treatment interruptions (STI) in chronic unsuppressed HIV infection in adults	Pant Pai, , N, Lawrence, J, Reingold, AL; Tulsky, JP	2006
Statins for the prevention of dementia	McGuinness, , B, Craig, D, Bullock, R; Passmore, P	2016
Stepping down the dose of inhaled corticosteroids for adults with asthma	Crossingham, , I, Evans, DJW, Halcovitch, NR; Marsden, PA	2017
Strategies of testing for syphilis during pregnancy	Shahrook, , S, Mori, R, Ochirbat, T; Gomi, H	2014
Steroid avoidance or withdrawal for pancreas and pancreas with kidney transplant recipients	Montero, , N, Webster, AC, Royuela, A, Zamora, J, Crespo Barrio, M; Pascual, J	2014
Strategies for detecting colon cancer in patients with inflammatory bowel disease	Bye, , WA, Nguyen, TM, Parker, CE, Jairath, V; East, JE	2017
Symptomatic treatment of the cough in whooping cough	Wang, , K, Bettiol, S, Thompson, MJ, Roberts, NW, Perera, R, Heneghan, CJ; Harnden, A	2014
Systemic corticosteroids for acute gout	Janssens, , HJ, Lucassen, PLBJ, Van de Laar, FA, Janssen, M; Van de Lisdonk, EH	2008
Systemic pharmacological treatments for chronic plaque psoriasis: a network meta-analysis	Sbidian, , E, Chaimani, A, Garcia-Doval, I, Do, G, Hua, C, Mazaud, C, Droitcourt, C, Hughes, C, Ingram, JR, Naldi, L, Chosidow, O; Le Cleach, L	2017
Subconjunctival draining minimally-invasive glaucoma devices for medically uncontrolled glaucoma	King, , AJ, Shah, A, Nikita, E, Hu, K, Mulvaney, CA, Stead, R; Azuara-Blanco, A	2018
Strategies to improve the implementation of healthy eating, physical activity and obesity prevention policies, practices or programmes within childcare services	Wolfenden, , L, Jones, J, Williams, CM, Finch, M, Wyse, RJ, Kingsland, M, Tzelepis, F, Wiggers, J, Williams, AJ, Seward, K, Small, T, Welch, V, Booth, D; Yoong, SL	2016
Strategies for enhancing the implementation of school-based policies or practices targeting risk factors for chronic disease	Wolfenden, , L, Nathan, NK, Sutherland, R, Yoong, SL, Hodder, RK, Wyse, RJ, Delaney, T, Grady, A, Fielding, A, Tzelepis, F, Clinton-McHarg, T, Parmenter, B, Butler, P, Wiggers, J, Bauman, A, Milat, A, Booth, D; Williams, CM	2017
Systemic prokinetic pharmacologic treatment for postoperative adynamic ileus following abdominal surgery in adults	Traut, , U, Brügger, L, Kunz, R, Pauli-Magnus, C, Haug, K, Bucher, H; Koller, MT	2008

Surgical cytoreduction for recurrent epithelial ovarian cancer	Al Rawahi, , T, Lopes, AD, Bristow, RE, Bryant, A, Elattar, A, Chattopadhyay, S; Galaal, K	2013
Surgical interventions for pharyngeal pouch	Sen, , P, Lowe, DA; Farnan, T	2005
Steroid hormones for contraception in women with sickle cell disease	Manchikanti Gomez, , A, Grimes, DA, Lopez, LM; Schulz, KF	2007
Systemic corticosteroids for acute otitis media in children	Ranakusuma, , RW, Pitoyo, Y, Safitri, ED, Thorning, S, Beller, EM, Sastroasmoro, S; Del Mar, CB	2018
Steroidal contraceptives: effect on carbohydrate metabolism in women without diabetes mellitus	Lopez, , LM, Grimes, DA; Schulz, KF	2014
Strategies for the withdrawal of nasal continuous positive airway pressure (NCPAP) in preterm infants	Jardine, , LA, Inglis, GDT; Davies, MW	2011
Surgical excision margins for primary cutaneous melanoma	Sladden, , MJ, Balch, C, Barzilai, DA, Berg, D, Freiman, A, Handiside, T, Hollis, S, Lens, MB; Thompson, JF	2009
Supplementary vitamin E, selenium, cysteine and riboflavin for preventing kwashiorkor in preschool children in developing countries	Odigwe, , CC, Smedslund, G, Ejemot-Nwadiaro, RI, Anyanechi, CC; Krawinkel, MB	2010
Stretching to prevent or reduce muscle soreness after exercise	Herbert, , RD, de Noronha, M; Kamper, SJ	2011
Surgery for tympanic membrane retraction pockets	Nankivell, , PC; Pothier, DD	2010
Surgical versus conservative treatment for acute injuries of the lateral ligament complex of the ankle in adults	Kerkhoffs, , GMMJ, Handoll, HHG, de Bie, R, Rowe, BH; Struijs, PAA	2007
Sumatriptan (oral route of administration) for acute migraine attacks in adults	Derry, , CJ, Derry, S; Moore, RA	2012
Strategies to increase the ownership and use of insecticide-treated bednets to prevent malaria	Augustincic Polec, , L, Petkovic, J, Welch, V, Ueffing, E, Tanjong Ghogomu, E, Pardo Pardo, J, Grabowsky, M, Attaran, A, Wells, GA; Tugwell, P	2015
Supportive care for patients with gastrointestinal cancer	Ahmed, , N, Ahmedzai, S, Vora, V, Harrison, S; Paz, S	2004
Steroid-eluting sinus stents for improving symptoms in chronic rhinosinusitis patients undergoing functional endoscopic sinus surgery	Huang, , Z, Hwang, P, Sun, Y; Zhou, B	2015
Sublingual immunotherapy for allergic rhinitis	Radulovic, , S, Calderon, MA, Wilson, D; Durham, S	2010
Systemic treatments for metastatic cutaneous melanoma	Pasquali, , S, Hadjinicolaou, AV, Chiarion Sileni, V, Rossi, CR; Mocellin, S	2018
Submacular surgery for choroidal neovascularisation secondary to age-related macular degeneration	Giansanti, , F, Eandi, CM; Virgili, G	2009
Steroid therapy for meconium aspiration syndrome in newborn infants	Ward, , MC; Sinn, JKH	2003
Statins for multiple sclerosis	Wang, , J, Xiao, Y, Luo, M; Luo, H	2011
Systematic screening and assessment of psychosocial well-being and care needs of people with cancer	Schouten, , B, Avau, B, Bekkering, GTRUDYE, Vankrunkelsven, P, Mebis, J, Hellings, J; Van Hecke, A	2019
Surgery for cervical intraepithelial neoplasia	Martin-Hirsch, , PPL, Paraskevaidis, E, Bryant, A; Dickinson, HO	2013
Surgical versus non-surgical interventions for vocal cord nodules	Pedersen, , M; McGlashan, J	2012
Sulpiride augmentation for schizophrenia	Wang, , J, Omori, IM, Fenton, M; Soares, BGO	2010
Surveillance of gastric intestinal metaplasia for the prevention of gastric cancer	O'Connor, , A, McNamara, D; O'Moráin, CA	2013
Surgical approach to hysterectomy for benign gynaecological disease	Aarts, , JWM, Nieboer, TE, Johnson, N, Tavender, E, Garry, R, Mol, BWJ; Kluivers, KB	2015
Surgical versus non-surgical treatment for lumbar spinal stenosis	Zaina, , F, Tomkins-Lane, C, Carragee, E; Negrini, S	2016
Surgical interventions for symptomatic mild to moderate knee osteoarthritis	Palmer, , JS, Monk, AP, Hopewell, S, Bayliss, LE, Jackson, W, Beard, DJ; Price, AJ	2019
Surgery for scoliosis in Duchenne muscular dystrophy	Cheuk, , DKL, Wong, V, Wraige, E, Baxter, P; Cole, A	2015
Systemic treatments for the prevention of venous thrombo-embolic events in paediatric cancer patients with tunnelled central venous catheters	Schoot, , RA, Kremer, LCM, van de Wetering, MD; van Ommen, CH	2013
Surgical portosystemic shunts versus transjugular intrahepatic portosystemic shunt for variceal haemorrhage in people with cirrhosis	Brand, , M, Prodehl, L; Ede, CJ	2018
Steam inhalation or humidified oxygen for acute bronchiolitis in children up to three years of age	Umoren, , R, Odey, F; Meremikwu, MM	2011
Surgical treatment for tubal disease in women due to undergo in vitro fertilisation	Johnson, , N, van Voorst, S, Sowter, MC, Strandell, A; Mol, BWJ	2010
Surgery for primary supratentorial intracerebral haemorrhage	Prasad, , K, Mendelow, AD; Gregson, B	2008
Statins for children with familial hypercholesterolemia	Vuorio, , A, Kuoppala, J, Kovanen, PT, Humphries, SE, Tonstad, S, Wiegman, A, Drogari, E; Ramaswami, U	2017
Systemic corticosteroids for acute exacerbations of chronic obstructive pulmonary disease	Walters, , JAE, Tan, DJ, White, CJ, Gibson, PG, Wood-Baker, R; Walters, EH	2014
Surfactant therapy for bronchiolitis in critically ill infants	Jat, , KR; Chawla, D	2015
Surgical orbital decompression for thyroid eye disease	Boboridis, , KG; Bunce, C	2011
Subcutaneous closure versus no subcutaneous closure after non-caesarean surgical procedures	Gurusamy, , KS, Toon, CD; Davidson, BR	2014

Surgical treatment options for carpal tunnel syndrome	Scholten, , RJPM, Mink van der Molen, A, Uitdehaag, BMJ, Bouter, LM; de Vet, HCW	2007
Surgery or embolization for varicoceles in subfertile men	Kroese, , ACJ, de Lange, NM, Collins, J; Evers, JLH	2012
Surgical interventions for the rheumatoid shoulder	Christie, , A, Dagfinrud, H, Engen Matre, K, Flaatten, HI, Ringen Osnes, H; Hagen, KB	2010
Statins for age-related macular degeneration	Gehlbach, , P, Li, T; Hatef, E	2016
Surgery for congenital choanal atresia	Cedin, , AC, Atallah, ÁN, Andriolo, RB, Cruz, OL; Pignatari, SN	2012
Sulodexide for treating venous leg ulcers	Wu, , B, Lu, J, Yang, M; Xu, T	2016
Task-oriented interventions for children with developmental co-ordination disorder	Miyahara, , M, Hillier, SL, Pridham, L; Nakagawa, S	2017
Surgical resection versus liver transplant for patients with hepatocellular carcinoma	Taefi, , A, Abrishami, A, Nasseri-Moghaddam, S, Eghtesad, B; Sherman, M	2013
Systemic antibiotics versus topical treatments for chronically discharging ears with underlying eardrum perforations	Macfadyen, , CA, Acuin, JM; Gamble, CL	2006
Tailored interventions based on sputum eosinophils versus clinical symptoms for asthma in children and adults	Petsky, , HL, Li, A; Chang, AB	2017
Sucrose for analgesia in newborn infants undergoing painful procedures	Stevens, , B, Yamada, J, Ohlsson, A, Haliburton, S; Shorkey, A	2016
Survival differences with immediate versus delayed chemotherapy for asymptomatic incurable metastatic colorectal cancer	Claassen, , YHM, van der Valk, MJM, Breugom, AJ, Frouws, MA, Bastiaannet, E, Liefers, GJ, van de Velde, CJH; Kapiteijn, E	2018
Stavudine, lamivudine and nevirapine combination therapy for treatment of HIV infection and AIDS in adults.	Siegfried, , N, van Deventer, PJU, Mahomed, FA; Rutherford, GW	2006
Statins for the treatment of dementia	McGuinness, , B, Craig, D, Bullock, R, Malouf, R; Passmore, P	2014
Steroid avoidance or withdrawal for kidney transplant recipients	Haller, , MC, Royuela, A, Nagler, EV, Pascual, J; Webster, AC	2016
Surgery versus medical therapy for heavy menstrual bleeding	Marjoribanks, , J, Lethaby, A; Farquhar, C	2016
Systematic versus opportunistic risk assessment for the primary prevention of cardiovascular disease	Dyakova, , M, Shantikumar, S, Colquitt, JL, Drew, CM, Sime, M, MacIver, J, Wright, N, Clarke, A; Rees, K	2016
Tai Chi for chronic obstructive pulmonary disease (COPD)	Ngai, , SPC, Jones, AYM; Tam, WWS	2016
Surgery for traumatic optic neuropathy	Yu-Wai-Man, , P; Griffiths, PG	2013
Structural and community-level interventions for increasing condom use to prevent the transmission of HIV and other sexually transmitted infections	Moreno, , R, Nababan, HY, Ota, E, Wariki, WMV, Ezoe, S, Gilmour, S; Shibuya, K	2014
Systemic antibiotics for treating malignant wounds	Ramasubbu, , DA, Smith, V, Hayden, F; Cronin, P	2017
Surgical versus medical treatment with cyclooxygenase inhibitors for symptomatic patent ductus arteriosus in preterm infants	Malviya, , MN, Ohlsson, A; Shah, SS	2013
Surgical intervention for anorectal fistula	Jacob, , TJ, Perakath, B; Keighley, MR	2010
Surgery for the treatment of obesity in children and adolescents	Ells, , LJ, Mead, E, Atkinson, G, Corpeleijn, E, Roberts, K, Viner, R, Baur, L, Metzendorf, MI; Richter, B	2015
Surgical interventions for treating pectus excavatum	de Oliveira Carvalho, , PE, da Silva, MVM, Rodrigues, OR; Cataneo, AJM	2014
Statins for acute ischemic stroke	Squizzato, , A, Romualdi, E, Dentali, F; Ageno, W	2011
Subacromial decompression surgery for rotator cuff disease	Karjalainen, , TV, Jain, NB, Page, CM, Lähdeoja, TA, Johnston, RV, Salamh, P, Kavaja, L, Ardern, CL, Agarwal, A, Vandvik, PO; Buchbinder, R	2019
Surgery for the resolution of symptoms in malignant bowel obstruction in advanced gynaecological and gastrointestinal cancer	Cousins, , SE, Tempest, E; Feuer, DJ	2016
Systemic administration of local anesthetic agents to relieve neuropathic pain	Challapalli, , V, Tremont-Lukats, IW, McNicol, ED, Lau, J; Carr, DB	2005
Systemic and topical antibiotics for chronic rhinosinusitis	Head, , K, Chong, LY, Piromchai, P, Hopkins, C, Philpott, C, Schilder, AGM; Burton, MJ	2016
Surgical versus non-surgical interventions in people with adolescent idiopathic scoliosis	Bettany-Saltikov, , J, Weiss, HR, Chockalingam, N, Taranu, R, Srinivas, S, Hogg, J, Whittaker, V, Kalyan, RV; Arnell, T	2015
Surgical versus conservative interventions for displaced intra-articular calcaneal fractures	Bruce, , J; Sutherland, A	2013
Surgery for trigger finger	Fiorini, , HJ, Tamaoki, MJ, Lenza, M, Gomes dos Santos, JB, Faloppa, F; Belloti, JC	2018
Stavudine or zidovudine in three-drug combination therapy for initial treatment of HIV infection in antiretroviral-naïve individuals	Spaulding, , A, Rutherford, GW; Siegfried, N	2010
Street lighting for preventing road traffic injuries	Beyer, , FR; Ker, K	2009
Systemic antimicrobial prophylaxis for percutaneous endoscopic gastrostomy	Lipp, , A; Lusardi, G	2013
Surgery versus sclerotherapy for the treatment of varicose veins	Rigby, , KA, Palfreyman, SSJ, Beverley, C; Michaels, JA	2004
Surgery for small asymptomatic abdominal aortic aneurysms	Filardo, , G, Powell, JT, Martinez, MAM; Ballard, DJ	2015
Surgical interventions for chronic rhinosinusitis with nasal polyps	Sharma, , R, Lakhani, R, Rimmer, J; Hopkins, C	2014

Superoxide dismutase for preventing chronic lung disease in mechanically ventilated preterm infants	Suresh, , G, Davis, JM; Soll, R	2001
Stopping long-acting beta2-agonists (LABA) for children with asthma well controlled on LABA and inhaled corticosteroids	Kew, , KM, Beggs, S; Ahmad, S	2015
Surgery versus radiotherapy for muscle invasive bladder cancer	Shelley, , M, Barber, J, Wilt, TJ; Mason, M	2001
Tamponade in surgery for retinal detachment associated with proliferative vitreoretinopathy	Schwartz, , SG, Flynn Jr, HW, Lee, WH; Wang, X	2014
Targeted mass media interventions promoting healthy behaviours to reduce risk of non-communicable diseases in adult, ethnic minorities	Mosdøl, , A, Lidal, IB, Straumann, GH; Vist, GE	2017
Surgical interventions for bilateral congenital cataract	Long, , V, Chen, S; Hatt, SR	2006
Strabismus surgery before versus after completion of amblyopia therapy in children	Korah, , S, Philip, S, Jasper, S, Antonio-Santos, A; Braganza, A	2014
Surgical sealant for preventing air leaks after pulmonary resections in patients with lung cancer	Belda-Sanchís, , J, Serra-Mitjans, M, Iglesias Sentis, M; Rami, R	2010
System change interventions for smoking cessation	Thomas, , D, Abramson, MJ, Bonevski, B; George, J	2017
Structured treatment interruptions (STI) in chronic suppressed HIV infection in adults	Pant Pai, , N, Tulsky, JP, Lawrence, J, Colford, JM; Reingold, AL	2005
Systemic antibiotics for symptomatic apical periodontitis and acute apical abscess in adults	Cope, , AL, Francis, N, Wood, F; Chestnutt, IG	2018
Sumatriptan (subcutaneous route of administration) for acute migraine attacks in adults	Derry, , CJ, Derry, S; Moore, RA	2012
Swallowing therapy for dysphagia in acute and subacute stroke	Bath, , PM, Lee, HS; Everton, LF	2018
Sumatriptan (rectal route of administration) for acute migraine attacks in adults	Derry, , CJ, Derry, S; Moore, RA	2012
Sugammadex, a selective reversal medication for preventing postoperative residual neuromuscular blockade	Abrishami, , A, Ho, J, Wong, J, Yin, L; Chung, F	2009
Subcutaneous unfractionated heparin for the initial treatment of venous thromboembolism	Robertson, , L; Strachan, J	2017
Sumatriptan (intranasal route of administration) for acute migraine attacks in adults	Derry, , CJ, Derry, S; Moore, RA	2012
Sweet-tasting solutions for needle-related procedural pain in infants one month to one year of age	Kassab, , M, Foster, JP, Foureur, M; Fowler, C	2012
Systemic corticosteroids for the management of cancer-related breathlessness (dyspnoea) in adults	Haywood, , A, Duc, J, Good, P, Khan, S, Rickett, K, Vayne-Bossert, P; Hardy, JR	2019
Stopping long-acting beta2-agonists (LABA) for adults with asthma well controlled by LABA and inhaled corticosteroids	Ahmad, , S, Kew, KM; Normansell, R	2015
Surgical interventions for the prevention or treatment of lymphoedema after breast cancer treatment	Markkula, , SP, Leung, N, Allen, VB; Furniss, D	2019
Swimming training for asthma in children and adolescents aged 18 years and under	Beggs, , S, Foong, YC, Le, HCT, Noor, D, Wood-Baker, R; Walters, JAE	2013
Surgery versus non-surgical management for unilateral ureteric-pelvic junction obstruction in newborns and infants less than two years of age	Weitz, , M, Portz, S, Laube, GF, Meerpohl, JJ; Bassler, D	2016
Subfascial endoscopic perforator surgery (SEPS) for treating venous leg ulcers	Lin, , ZC, Loveland, PM, Johnston, RV, Bruce, M; Weller, CD	2019
S-Adenosylmethionine for osteoarthritis of the knee or hip	Rutjes, , AWS, Nüesch, E, Reichenbach, S; Jüni, P	2009
Sulfasalazine for ankylosing spondylitis	Chen, , J, Lin, S; Liu, C	2014
Subpial transection surgery for epilepsy	Krishnaiah, , B, Ramaratnam, S; Ranganathan, LN	2018
Tailored interventions based on exhaled nitric oxide versus clinical symptoms for asthma in children and adults	Petsky, , HL, Cates, CJ, Li, A, Kynaston, JA, Turner, C; Chang, AB	2009
Sun protection for preventing basal cell and squamous cell skin cancers	Sánchez, , G, Nova, J, Rodriguez-Hernandez, AE, Medina, RD, Solorzano-Restrepo, C, Gonzalez, J, Olmos, M, Godfrey, K; Arevalo-Rodriguez, I	2016
Surgery versus radical endotherapies for early cancer and high-grade dysplasia in Barrett's oesophagus	Bennett, , C, Green, S, DeCaestecker, J, Almond, M, Barr, H, Bhandari, P, Ragunath, K, Singh, R; Jankowski, J	2012
Statins for non-alcoholic fatty liver disease and non-alcoholic steatohepatitis	Eslami, , L, Merat, S, Malekzadeh, R, Nasseri-Moghaddam, S; Aramin, H	2013
Stent placement versus surgical palliation for adults with malignant gastric outlet obstruction	Upchurch, , E, Ragusa, M; Cirocchi, R	2018
Sublingual immunotherapy for asthma	Normansell, , R, Kew, KM; Bridgman, AL	2015
Supportive devices for preventing and treating subluxation of the shoulder after stroke	Ada, , L, Foongchomcheay, A; Canning, CG	2005
Surfactant for bacterial pneumonia in late preterm and term infants	Tan, , K, Lai, NM; Sharma, A	2012
Systemic interventions for recurrent aphthous stomatitis (mouth ulcers)	Brocklehurst, , P, Tickle, M, Glenny, AM, Lewis, MA, Pemberton, MN, Taylor, J, Walsh, T, Riley, P; Yates, JM	2012
Task shifting from doctors to non-doctors for initiation and maintenance of antiretroviral therapy	Kredo, , T, Adeniyi, FB, Bateganya, M; Pienaar, ED	2014
Surgical techniques for the removal of mandibular wisdom teeth	Coulthard, , P, Bailey, E, Esposito, M, Furness, S, Renton, TF; Worthington, HV	2014
Surgery for local and locally advanced non-small cell lung cancer	Manser, , R, Wright, G, Hart, D, Byrnes, G, Campbell, D, Wainer, Z; Tort, S	2005

Systemic antifungal therapy for tinea capitis in children	Chen, , X, Jiang, X, Yang, M, González, U, Lin, X, Hua, X, Xue, S, Zhang, M; Bennett, C	2016
Surgical removal versus retention for the management of asymptomatic disease-free impacted wisdom teeth	Ghaeminia, , H, Perry, J, Nienhuijs, MEL, Toedtling, V, Tummers, M, Hoppenreijs, TJM, Van der Sanden, WJM; Mettes, TG	2016
Supportive interventions for enhancing dietary intake in malnourished or nutritionally at-risk adults	Baldwin, , C, Kimber, KL, Gibbs, M; Weekes, CE	2016
Subcutaneous rapid-acting insulin analogues for diabetic ketoacidosis	Andrade-Castellanos, , CA, Colunga-Lozano, LE, Delgado-Figueroa, N; Gonzalez-Padilla, DA	2016
Strategies for improving adherence to antiepileptic drug treatment in people with epilepsy	Al-aqeel, , S, Gershuni, O, Al-sabhan, J; Hiligsmann, M	2017
Stent graft types for endovascular repair of thoracic aortic aneurysms	Rolph, , R, Duffy, JMN; Waltham, M	2015
Strategies for integrating primary health services in low- and middle-income countries at the point of delivery	Dudley, , L; Garner, P	2011
Sulthiame monotherapy for epilepsy	Milburn-McNulty, , P, Powell, G, Sills, GJ; Marson, AG	2014
Surgical versus conservative interventions for treating ankle fractures in adults	Donken, , CCMA, Al-Khateeb, H, Verhofstad, MHJ; van Laarhoven, CJHM	2012
Surgical resection versus non-surgical treatment for hepatic node positive patients with colorectal liver metastases	Gurusamy, , KS, Ramamoorthy, R, Imber, C; Davidson, BR	2010
Target of rapamycin inhibitors (TOR-I; sirolimus and everolimus) for primary immunosuppression in kidney transplant recipients	Webster, , AC, Lee, VWS, Chapman, JR; Craig, JC	2006
Tacrolimus versus cyclosporin as primary immunosuppression for kidney transplant recipients	Webster, , AC, Taylor, RRS, Chapman, JR; Craig, JC	2005
Statins for aortic valve stenosis	Thiago, , L, Tsuji, SR, Nyong, J, Puga, MES, Gois, AFT, Macedo, CR, Valente, O; Atallah, ÁN	2016
Surfactant for pulmonary haemorrhage in neonates	Aziz, , A; Ohlsson, A	2012
Steroids for symptom control in infectious mononucleosis	Rezk, , E, Nofal, YH, Hamzeh, A, Aboujaib, MF, AlKheder, MA; Al Hammad, MF	2015
Surgical interventions for early squamous cell carcinoma of the vulva	van der Velden, J	2000
Systemic corticosteroid regimens for prevention of bronchopulmonary dysplasia in preterm infants	Onland, , W, De Jaegere, APMC, Offringa, M; van Kaam, A	2017
Steroid hormones for contraception in men	Grimes, , DA, Lopez, LM, Gallo, MF, Halpern, V, Nanda, K; Schulz, KF	2012
Stretch for the treatment and prevention of contractures	Harvey, , LA, Katalinic, OM, Herbert, RD, Moseley, AM, Lannin, NA; Schurr, K	2017
Stiripentol add-on therapy for focal refractory epilepsy	Brigo, , F, Igwe, SC; Bragazzi, NL	2018
Steroid sex hormones for lower limb atherosclerosis	Price, , J; Leng, GC	2012
Surgery for varicose veins: use of tourniquet	Rigby, , KA, Palfreyman, SJ, Beverley, C; Michaels, JA	2013
Steroidal contraceptives and bone fractures in women: evidence from observational studies	Lopez, , LM, Chen, M, Mullins Long, S, Curtis, KM; Helmerhorst, FM	2015
Surgical versus endoscopic treatment of bile duct stones	Dasari, , BVM, Tan, CJ, Gurusamy, KS, Martin, DJ, Kirk, G, McKie, L, Diamond, T; Taylor, MA	2013
Surgery for epilepsy	West, , S, Nevitt, SJ, Cotton, J, Gandhi, S, Weston, J, Sudan, A, Ramirez, R; Newton, R	2019
Statins versus placebo for people with chronic obstructive pulmonary disease	Walsh, , A, Perrem, L, Khashan, AS, Henry, MT; Ni Chroinin, M	2019
Veno-venous bypass versus none for liver transplantation	Gurusamy, , KS, Koti, R, Pamecha, V; Davidson, BR	2011
Vitamin E for Alzheimer's dementia and mild cognitive impairment	Farina, , N, Llewellyn, D, Isaac, MGEKN; Tabet, N	2017
Vasopressin and its analogues for the treatment of refractory hypotension in neonates	Shivanna, , B, Rios, D, Rossano, J, Fernandes, CJ; Pammi, M	2013
Types of indwelling urethral catheters for short-term catheterisation in hospitalised adults	Lam, , TBL, Omar, MI, Fisher, E, Gillies, K; MacLennan, S	2014
Vaccines for preventing typhoid fever	Milligan, , R, Paul, M, Richardson, M; Neuberger, A	2018
Ursodeoxycholic acid for primary biliary cirrhosis	Rudic, , JS, Poropat, G, Krstic, MN, Bjelakovic, G; Gluud, C	2012
Types of indwelling urinary catheters for long-term bladder drainage in adults	Jahn, , P, Beutner, K; Langer, G	2012
Treadmill training and body weight support for walking after stroke	Mehrholz, , J, Thomas, S; Elsner, B	2017
Treatment of infantile spasms	Hancock, , EC, Osborne, JP; Edwards, SW	2013
Vaccines for preventing influenza in people with cystic fibrosis	Dharmaraj, , P; Smyth, RL	2014
Trihexyphenidyl for dystonia in cerebral palsy	Harvey, , AR, Baker, LB, Reddihough, DS, Scheinberg, A; Williams, K	2018
Triclosan/copolymer containing toothpastes for oral health	Riley, , P; Lamont, T	2013
Vitamin B and its derivatives for diabetic kidney disease	Raval, , AD, Thakker, D, Rangoonwala, AN, Gor, D; Walia, R	2015
Urinary diversion and bladder reconstruction/replacement using intestinal segments for intractable incontinence or following cystectomy	Cody, , JD, Nabi, G, Dublin, N, McClinton, S, Neal, DE, Pickard, R; Yong, SM	2012

Urine tests for Down's syndrome screening	Alldred, , SK, Guo, B, Takwoingi, Y, Pennant, M, Wisniewski, S, Deeks, JJ, Neilson, JP; Alfirevic, Z	2015
Vaccines for preventing influenza in healthy adults	Demicheli, , V, Jefferson, T, Ferroni, E, Rivetti, A; Di Pietrantonj, C	2018
Treatment including anthracyclines versus treatment not including anthracyclines for childhood cancer	van Dalen, , EC, Raphaël, MF, Caron, HN; Kremer, LCM	2014
Treatment of hypertension in peripheral arterial disease	Lane, , DA; Lip, GYH	2013
Treatment for Fisher syndrome, Bickerstaff's brainstem encephalitis and related disorders	Overell, , JR, Hseih, ST, Odaka, M, Yuki, N; Willison, HJ	2007
Vitamin A supplements for reducing mother-to-child HIV transmission	Wiysonge, , CS, Ndze, VN, Kongnyuy, EJ; Shey, MS	2017
Vaginal disinfection for preventing mother-to-child transmission of HIV infection	Wiysonge, , CS, Shey, M, Shang, J, Sterne, JAC; Brocklehurst, P	2005
Treatment of obstructive sleep apnoea for chronic cough in children	Teoh, , L, Hurwitz, M, Acworth, JP, van Asperen, P; Chang, AB	2011
Treatments for chronic inflammatory demyelinating polyradiculoneuropathy (CIDP): an overview of systematic reviews	Oaklander, , AL, Lunn, MPT, Hughes, RAC, van Schaik, IN, Frost, C; Chalk, CH	2017
Treatment of latent tuberculosis infection in HIV infected persons	Akolo, , C, Adetifa, I, Shepperd, S; Volmink, J	2010
Treatments for breast abscesses in breastfeeding women	Irusen, , H, Rohwer, AC, Steyn, DW; Young, T	2015
Treatment for women with postpartum iron deficiency anaemia	Dodd, , JM, Dare, MR; Middleton, P	2004
Vitamin A supplementation to prevent mortality and short- and long-term morbidity in very low birth weight infants	Darlow, , BA, Graham, PJ; Rojas-Reyes, MX	2016
Vitamin C supplementation in pregnancy	Rumbold, , A, Ota, E, Nagata, C, Shahrook, S; Crowther, CA	2015
Type of incision for below knee amputation	Tisi, , PV; Than, MM	2014
Vapocoolants (cold spray) for pain treatment during intravenous cannulation	Griffith, , RJ, Jordan, V, Herd, D, Reed, PW; Dalziel, SR	2016
Vaccines for the common cold	Simancas-Racines, , D, Franco, JVA, Guerra, CV, Felix, ML, Hidalgo, R; Martinez-Zapata, MJ	2017
Trastuzumab-containing regimens for metastatic breast cancer	Balduzzi, , S, Mantarro, S, Guarneri, V, Tagliabue, L, Pistotti, V, Moja, L; D'Amico, R	2014
Treatment for hepatitis C virus-associated mixed cryoglobulinaemia	Montero, , N, Favà, A, Rodriguez, E, Barrios, C, Cruzado, JM, Pascual, J; Soler, MJ	2018
Uric acid lowering therapies for preventing or delaying the progression of chronic kidney disease	Sampson, , AL, Singer, RF; Walters, GD	2017
Vision screening for amblyopia in childhood	Powell, , C; Hatt, SR	2009
Vaccines for preventing smallpox	Metzger, , W; Mordmueller, BG	2007
Treatment for mitochondrial disorders	Pfeffer, , G, Majamaa, K, Turnbull, DM, Thorburn, D; Chinnery, PF	2012
Vector and reservoir control for preventing leishmaniasis	González, , U, Pinart, M, Sinclair, D, Firooz, A, Enk, C, Vélez, ID, Esterhuizen, TM, Tristan, M; Alvar, J	2015
Uricosuric medications for chronic gout	Kydd, , ASR, Seth, R, Buchbinder, R, Edwards, CJ; Bombardier, C	2014
Treatment for idiopathic and hereditary neuralgic amyotrophy (brachial neuritis)	van Alfen, , N, van Engelen, BGM; Hughes, RAC	2009
Treatment for preventing bleeding in people with haemophilia or other congenital bleeding disorders undergoing surgery	Coppola, , A, Windyga, J, Tufano, A, Yeung, C; Di Minno, MND	2015
Ultra-radical (extensive) surgery versus standard surgery for the primary cytoreduction of advanced epithelial ovarian cancer	Ang, , C, Chan, KKL, Bryant, A, Naik, R; Dickinson, HO	2011
Treatment for postpolio syndrome	Koopman, , FS, Beelen, A, Gilhus, NE, de Visser, M; Nollet, F	2015
Vitamin D supplementation for sickle cell disease	Soe, , HHK, Abas, ABL, Than, NN, Ni, H, Singh, J, Said, ARBM; Osunkwo, I	2017
Vitamin B for treating peripheral neuropathy	Ang, , CD, Alviar, MJM, Dans, AL, Bautista-Velez, GGP, Villaruz-Sulit, MVC, Tan, JJ, Co, HU, Bautista, MRM; Roxas, AA	2008
Transfusion thresholds and other strategies for guiding allogeneic red blood cell transfusion	Carson, , JL, Stanworth, SJ, Roubinian, N, Fergusson, DA, Triulzi, D, Doree, C; Hebert, PC	2016
Trial of instrumental delivery in theatre versus immediate caesarean section for anticipated difficult assisted births	Majoko, , F; Gardener, G	2012
Treadmill interventions in children under six years of age at risk of neuromotor delay	Valentín-Gudiol, , M, Mattern-Baxter, K, Girabent-Farrés, M, Bagur-Calafat, C, Hadders-Algra, M; Angulo-Barroso, RM	2017
Vitamin D compounds for people with chronic kidney disease requiring dialysis	Palmer, , SC, McGregor, DO, Craig, JC, Elder, G, Macaskill, P; Strippoli, GFM	2009
Treatment for paraneoplastic neuropathies	Giometto, , B, Vitaliani, R, Lindeck-Pozza, E, Grisold, W; Vedeler, C	2012
Treatment of dental complications in sickle cell disease	Mulimani, , P, Ballas, SK, Abas, ABL; Karanth, L	2016
Vena caval filters for the prevention of pulmonary embolism	Young, , T, Tang, H; Hughes, R	2010
Very early discharge versus early discharge versus non-early discharge in children with cancer and febrile neutropenia	Loeffen, , EAH, te Poele, EM, Tissing, WJE, Boezen, HM; de Bont, ESJM	2016

Treatment of valvular heart disease during pregnancy for improving maternal and neonatal outcome	Henriquez, , DDCA, Roos-Hesselink, JW, Schalij, MJ, Klautz, RJM, Helmerhorst, FM; de Groot, CJM	2011
Treatment for POEMS (polyneuropathy, organomegaly, endocrinopathy, M-protein, and skin changes) syndrome	Kuwabara, , S, Dispenzieri, A, Arimura, K, Misawa, S; Nakaseko, C	2012
Ultrasound for fetal assessment in early pregnancy	Whitworth, , M, Bricker, L; Mullan, C	2015
Vitamin B12 for cognition	Malouf, , R; Areosa Sastre, A	2003
Vaccines for preventing influenza in healthy children	Jefferson, , T, Rivetti, A, Di Pietrantonj, C; Demicheli, V	2018
Vitamin A supplementation for postpartum women	Oliveira, , JM, Allert, R; East, CE	2016
Vaccines for preventing enterotoxigenic Escherichia coli (ETEC) diarrhoea	Ahmed, , T, Bhuiyan, TR, Zaman, K, Sinclair, D; Qadri, F	2013
Vaccines for preventing influenza in the elderly	Demicheli, , V, Jefferson, T, Di Pietrantonj, C, Ferroni, E, Thorning, S, Thomas, RE; Rivetti, A	2018
Valproic acid, valproate and divalproex in the maintenance treatment of bipolar disorder	Cipriani, , A, Reid, K, Young, AH, Macritchie, K; Geddes, J	2013
Transpyloric versus gastric tube feeding for preterm infants	Watson, , J; McGuire, W	2013
Vitamin C for asthma and exercise-induced bronchoconstriction	Milan, , SJ, Hart, A; Wilkinson, M	2013
Vitamin D for the treatment of chronic painful conditions in adults	Straube, , S, Derry, S, Straube, C; Moore, RA	2015
Vasodilators and vasoactive substances for idiopathic sudden sensorineural hearing loss	Agarwal, , L; Pothier, DD	2009
Transparent Cap Colonoscopy versus Standard Colonoscopy to Improve Caecal Intubation	Morgan, , J, Thomas, K, Lee-Robichaud, H, Nelson, RL; Braungart, S	2012
Vitamin C for preventing and treating tetanus	Hemilä, , H; Koivula, T	2013
Tyrosine supplementation for phenylketonuria	Webster, , D; Wildgoose, J	2013
Virtual reality for stroke rehabilitation	Laver, , KE, Lange, B, George, S, Deutsch, JE, Saposnik, G; Crotty, M	2017
Treatments for iron-deficiency anaemia in pregnancy	Reveiz, , L, Gyte, GML, Cuervo, LG; Casasbuenas, A	2011
Vaccines for preventing anthrax	Donegan, , S, Bellamy, R; Gamble, CL	2009
Unconditional cash transfers for reducing poverty and vulnerabilities: effect on use of health services and health outcomes in low- and middle-income countries	Pega, , F, Liu, SY, Walter, S, Pabayo, R, Saith, R; Lhachimi, SK	2017
Types of intraocular lenses for cataract surgery in eyes with uveitis	Leung, , TG, Lindsley, K; Kuo, IC	2014
Treatment and prevention of pouchitis after ileal pouch-anal anastomosis for chronic ulcerative colitis	Nguyen, , N, Zhang, B, Holubar, SD, Pardi, DS; Singh, S	2019
Treatment for cramps in amyotrophic lateral sclerosis/motor neuron disease	Baldinger, , R, Katzberg, HD; Weber, M	2012
Umeclidinium bromide versus placebo for people with chronic obstructive pulmonary disease (COPD)	Ni, , H, Htet, A; Moe, S	2017
Treatment for meralgia paraesthetica	Khalil, , N, Nicotra, A; Rakowicz, W	2012
Treatment for anemia in people with AIDS	Martí-Carvajal, , AJ, Solà, I, Peña-Martí, GE; Comunián-Carrasco, G	2011
Treatment for speech disorder in Friedreich ataxia and other hereditary ataxia syndromes	Vogel, , AP, Folker, J; Poole, ML	2014
Vascular occlusion for elective liver resections	Gurusamy, , KS, Kumar, Y, Ramamoorthy, R, Sharma, D; Davidson, BR	2009
Treatment for cryoglobulinemic and non-cryoglobulinemic peripheral neuropathy associated with hepatitis C virus infection	Benstead, , TJ, Chalk, CH; Parks, NE	2014
Treatment for inclusion body myositis	Rose, , MR, Jones, K, Leong, K, Walter, MC, Miller, J, Dalakas, MC, Brassington, R; Griggs, R	2015
Ursodeoxycholic acid for cystic fibrosis-related liver disease	Cheng, , K, Ashby, D; Smyth, RL	2017
Twenty-four hour care for schizophrenia	Macpherson, , R, Edwards, TR, Chilvers, R, David, C; Elliott, HJ	2009
Utero-placental Doppler ultrasound for improving pregnancy outcome	Stampalija, , T, Gyte, GML; Alfirevic, Z	2010
Vitamin D supplementation for cystic fibrosis	Ferguson, , JH; Chang, AB	2014
Intravenous immunoglobulin for the treatment of childhood encephalitis	Iro, , MA, Martin, NG, Absoud, M; Pollard, AJ	2017
Transient elastography for diagnosis of stages of hepatic fibrosis and cirrhosis in people with alcoholic liver disease	Pavlov, , CS, Casazza, G, Nikolova, D, Tsochatzis, E, Burroughs, AK, Ivashkin, VT; Gluud, C	2015
Interventions for the treatment of brain radionecrosis after radiotherapy or radiosurgery	Chung, , C, Bryant, A; Brown, PD	2018
Vaccines for preventing influenza in people with asthma	Cates, , CJ; Rowe, BH	2013
Intravenous fluids for reducing the duration of labour in low risk nulliparous women	Dawood, , F, Dowswell, T; Quenby, S	2013
Vitamin A for preventing acute lower respiratory tract infections in children up to seven years of age	Chen, , H, Zhuo, Q, Yuan, W, Wang, J; Wu, T	2008

Unfractionated heparin versus low molecular weight heparins for avoiding heparin-induced thrombocytopenia in postoperative patients	Junqueira, , DR, Zorzela, LM; Perini, E	2017
Interventions for treating depression after stroke	Hackett, , ML, Anderson, CS, House, A; Xia, J	2008
Trypanocidal drugs for chronic asymptomatic Trypanosoma cruzi infection	Villar, , JC, Perez, JG, Cortes, OL, Riarte, A, Pepper, M, Marin-Neto, JA; Guyatt, GH	2014
Intrauterine insemination versus intracervical insemination in donor sperm treatment	Kop, , PAL, Mochtar, MH, O'Brien, PA, Van der Veen, F; van Wely, M	2018
Vaginal dilator therapy for women receiving pelvic radiotherapy	Miles, , T; Johnson, N	2014
Interventions to improve adherence to exercise for chronic musculoskeletal pain in adults	Jordan, , JL, Holden, MA, Mason, EEJ; Foster, NE	2010
Vagus nerve stimulation for partial seizures	Panebianco, , M, Rigby, A, Weston, J; Marson, AG	2015
Interventions to improve antibiotic prescribing practices in ambulatory care	Arnold, , SR; Straus, SE	2005
Vaccination for preventing postherpetic neuralgia	Chen, , N, Li, Q, Zhang, Y, Zhou, M, Zhou, D; He, L	2011
Interventions for replacing missing teeth: dental implants in zygomatic bone for the rehabilitation of the severely deficient edentulous maxilla	Esposito, , M; Worthington, HV	2013
Treatment for Barrett's oesophagus	Rees, , JRE, Lao-Sirieix, P, Wong, A; Fitzgerald, RC	2010
Interventions for smoking cessation in Indigenous populations	Carson, , KV, Brinn, MP, Peters, M, Veale, A, Esterman, AJ; Smith, BJ	2012
Vestibular rehabilitation for unilateral peripheral vestibular dysfunction	McDonnell, , MN; Hillier, SL	2015
Interventions for supporting informal caregivers of patients in the terminal phase of a disease	Candy, , B, Jones, L, Drake, R, Leurent, B; King, M	2011
Vitamin A for non-measles pneumonia in children	Wu, , T, Ni, J; Wei, J	2005
Interventions for treating sexual dysfunction in patients with chronic kidney disease	Vecchio, , M, Navaneethan, SD, Johnson, DW, Lucisano, G, Graziano, G, Saglimbene, V, Ruospo, M, Querques, M, Jannini, EA; Strippoli, GFM	2010
Vitamin D supplementation for preventing infections in children under five years of age	Yakoob, , MY, Salam, RA, Khan, FR; Bhutta, ZA	2016
Interventions for treating chronic ankle instability	de Vries, , JS, Krips, R, Sierevelt, IN, Blankevoort, L; van Dijk, CN	2011
Vaccines for preventing hepatitis B in health-care workers	Chen, , W; Gluud, C	2005
Interventions for treating traumatised ankylosed permanent front teeth	de Souza, , RF, Travess, H, Newton, T; Marchesan, MA	2015
Vitamin D supplementation for improving bone mineral density in children	Winzenberg, , TM, Powell, S, Shaw, KA; Jones, G	2010
Interventions to improve water quality and supply, sanitation and hygiene practices, and their effects on the nutritional status of children	Dangour, , AD, Watson, L, Cumming, O, Boisson, S, Che, Y, Velleman, Y, Cavill, S, Allen, E; Uauy, R	2013
Ultrasonography for diagnosis of alcoholic cirrhosis in people with alcoholic liver disease	Pavlov, , CS, Casazza, G, Semenistaia, M, Nikolova, D, Tsochatzis, E, Liusina, E, Ivashkin, VT; Gluud, C	2016
Interventions for treating placental abruption	Neilson, JP	2003
Treatment for primary postpartum haemorrhage	Mousa, , HA, Blum, J, Abou El Senoun, G, Shakur, H; Alfirevic, Z	2014
Treatment of Kaposi sarcoma in children with HIV-1 infection	Anglemyer, , A, Agrawal, AK; Rutherford, GW	2014
Interventions to improve access to cataract surgical services and their impact on equity in low- and middle-income countries	Ramke, , J, Petkovic, J, Welch, V, Blignault, I, Gilbert, C, Blanchet, K, Christensen, R, Zwi, AB; Tugwell, P	2017
Treatment for women with postpartum iron deficiency anaemia	Markova, , V, Norgaard, A, Jørgensen, KJ; Langhoff-Roos, J	2015
Interventions for treating AIDS-associated Hodgkin´s lymphoma in treatment-naive adults	Martí-Carvajal, , AJ, Cardona, AF; Rodríguez, ML	2007
Urinary catheter policies for long-term bladder drainage	Niël-Weise, , BS, van den Broek, PJ, da Silva, EMK; Silva, LA	2012
Interventions for treating simple bone cysts in the long bones of children	Zhao, , JG, Wang, J, Huang, WJ, Zhang, P, Ding, N; Shang, J	2017
Urethral (indwelling or intermittent) or suprapubic routes for short-term catheterisation in hospitalised adults	Kidd, , EA, Stewart, F, Kassis, NC, Hom, E; Omar, MI	2015
Tricyclic antidepressants for attention deficit hyperactivity disorder (ADHD) in children and adolescents	Otasowie, , J, Castells, X, Ehimare, UP; Smith, CH	2014
Intraoperative frozen section analysis for the diagnosis of early stage ovarian cancer in suspicious pelvic masses	Ratnavelu, , NDG, Brown, AP, Mallett, S, Scholten, RJPM, Patel, A, Founta, C, Galaal, K, Cross, P; Naik, R	2016
Transient neurologic symptoms (TNS) following spinal anaesthesia with lidocaine versus other local anaesthetics	Zaric, , D; Pace, NL	2009
Interventions to increase tuberculosis case detection at primary healthcare or community-level services	Mhimbira, , FA, Cuevas, LE, Dacombe, R, Mkopi, A; Sinclair, D	2017
Treatment for familial amyotrophic lateral sclerosis/motor neuron disease	Benatar, , M, Kurent, J; Moore, DH	2009
Interventions for treating osteoarthritis of the big toe joint	Zammit, , GV, Menz, HB, Munteanu, SE, Landorf, KB; Gilheany, MF	2010
Trypanocidal drugs for late stage, symptomatic Chagas disease (Trypanosoma cruzi infection)	Vallejo, , M; Reyes, PPA	2005
Interventions for the treatment of twin-twin transfusion syndrome	Roberts, , D, Neilson, JP, Kilby, MD; Gates, S	2014

Vaccines for prophylaxis of viral infections in patients with hematological malignancies	Cheuk, , DKL, Chiang, AKS, Lee, TL, Chan, GCF; Ha, SY	2011
Interventions to increase the reporting of occupational diseases by physicians	Curti, , S, Sauni, R, Spreeuwers, D, De Schryver, A, Valenty, M, Rivière, S; Mattioli, S	2015
Vaginal chlorhexidine during labour to prevent early-onset neonatal group B streptococcal infection	Ohlsson, , A, Shah, VS; Stade, BC	2014
Interventions for the treatment of borderline ovarian tumours	Faluyi, , O, Mackean, M, Gourley, C, Bryant, A; Dickinson, HO	2010
Treatment for femoral pseudoaneurysms	Tisi, , PV; Callam, MJ	2013
Intrastromal corneal ring segments for treating keratoconus	Zadnik, , K, Money, S; Lindsley, K	2019
Treadmill training for patients with Parkinson's disease	Mehrholz, , J, Kugler, J, Storch, A, Pohl, M, Hirsch, K; Elsner, B	2015
Interventions for rosacea	van Zuuren, , EJ, Fedorowicz, Z, Carter, B, van der Linden, MMD; Charland, L	2015
Vision screening of older drivers for preventing road traffic injuries and fatalities	Desapriya, , E, Harjee, R, Brubacher, J, Chan, H, Hewapathirane, DS, Subzwari, S; Pike, I	2014
Interventions to improve adherence to inhaled steroids for asthma	Normansell, , R, Kew, KM; Stovold, E	2017
Vancomycin for prophylaxis against sepsis in preterm neonates	Craft, , AP, Finer, N; Barrington, KJ	2000
Interventions for treating gas gangrene	Yang, , Z, Hu, J, Qu, Y, Sun, F, Leng, X, Li, H; Zhan, S	2015
Treatment for Lambert-Eaton myasthenic syndrome	Keogh, , M, Sedehizadeh, S; Maddison, P	2011
Interventions to reduce Staphylococcus aureus in the management of atopic eczema	Birnie, , AJ, Bath-Hextall, FJ, Ravenscroft, JC; Williams, HC	2008
Vitamin A supplementation for the prevention of morbidity and mortality in infants one to six months of age	Imdad, , A, Ahmed, Z; Bhutta, ZA	2016
Interventions to reduce emigration of health care professionals from low- and middle-income countries	Peñaloza, , B, Pantoja, T, Bastías, G, Herrera, CA; Rada, G	2011
Tumor necrosis factor-alpha antibody for maintenance of remission in Crohn's disease	Behm, , BW; Bickston, SJ	2008
Transtheoretical model stages of change for dietary and physical exercise modification in weight loss management for overweight and obese adults	Mastellos, , N, Gunn, LH, Felix, LM, Car, J; Majeed, A	2014
Interventions for psychotic symptoms concomitant with epilepsy	Farooq, , S; Sherin, A	2015
Intravenous immunoglobulin for Guillain-Barré syndrome	Hughes, , RAC, Swan, AV; van Doorn, PA	2014
Ultrasound-guided transvaginal ovarian needle drilling for clomiphene-resistant polycystic ovarian syndrome in subfertile women	Zhang, , J, Tang, L, Kong, L, Wu, T, Xu, L, Pan, X; Liu, GJ	2019
Interventions to enhance return-to-work for cancer patients	de Boer, , AGEM, Taskila, TK, Tamminga, SJ, Feuerstein, M, Frings-Dresen, MHW; Verbeek, JH	2015
Use of biochemical tests of placental function for improving pregnancy outcome	Heazell, , AEP, Whitworth, M, Duley, L; Thornton, JG	2015
Interventions for treating persistent and intractable hiccups in adults	Moretto, , EN, Wee, B, Wiffen, PJ; Murchison, AG	2013
Treatment for dysphagia (swallowing difficulties) in hereditary ataxia	Vogel, , AP, Keage, MJ, Johansson, K; Schalling, E	2015
Intramuscular versus intravenous anti-D for preventing Rhesus alloimmunization during pregnancy	Okwundu, , CI; Afolabi, BB	2013
Interventions for reducing medication errors in children in hospital	Maaskant, , JM, Vermeulen, H, Apampa, B, Fernando, B, Ghaleb, MA, Neubert, A, Thayyil, S; Soe, A	2015
Interventions for the reduction of prescribed opioid use in chronic non-cancer pain	Eccleston, , C, Fisher, E, Thomas, KH, Hearn, L, Derry, S, Stannard, C, Knaggs, R; Moore, RA	2017
Interventions for treating mallet finger injuries	Handoll, , HHG; Vaghela, MV	2004
Intracystic bleomycin for cystic craniopharyngiomas in children	Zhang, , S, Fang, Y, Cai, BW, Xu, JG; You, C	2016
Intracavity lavage and wound irrigation for prevention of surgical site infection	Norman, , G, Atkinson, RA, Smith, TA, Rowlands, C, Rithalia, AD, Crosbie, EJ; Dumville, JC	2017
Interventions for treating tuberculous pericarditis	Wiysonge, , CS, Ntsekhe, M, Thabane, L, Volmink, J, Majombozi, D, Gumedze, F, Pandie, S; Mayosi, BM	2017
Interventions to improve inhaler technique for people with asthma	Normansell, , R, Kew, KM; Mathioudakis, AG	2017
Intravenous antibiotics for pulmonary exacerbations in people with cystic fibrosis	Hurley, , MN, Prayle, AP; Flume, P	2015
Interventions for squamous cell carcinoma of the conjunctiva in HIV-infected individuals	Gichuhi, , S; Irlam, JH	2013
Interventions for the treatment of decreased bone mineral density associated with HIV infection	Lin, , D; Rieder, MJ	2007
Interventions to improve professional adherence to guidelines for prevention of device-related infections	Flodgren, , G, Conterno, LO, Mayhew, A, Omar, O, Pereira, CR; Shepperd, S	2013
Interventions for recruiting smokers into cessation programmes	Marcano Belisario, , JS, Bruggeling, MN, Gunn, LH, Brusamento, S; Car, J	2012
Intravenous magnesium sulfate for treating children with acute asthma in the emergency department	Griffiths, , B; Kew, KM	2016
Interventions to enhance adherence to dietary advice for preventing and managing chronic diseases in adults	Desroches, , S, Lapointe, A, Ratté, S, Gravel, K, Légaré, F; Turcotte, S	2013

Interventions for the treatment of oral and oropharyngeal cancers: targeted therapy and immunotherapy	Chan, , KKW, Glenny, AM, Weldon, JC, Furness, S, Worthington, HV; Wakeford, H	2015
Interventions for tobacco use prevention in Indigenous youth	Carson, , KV, Brinn, MP, Labiszewski, NA, Peters, M, Chang, AB, Veale, A, Esterman, AJ; Smith, BJ	2012
Intravenous immunoglobulin as adjuvant therapy for Wegener's granulomatosis	Fortin, , PM, Tejani, AM, Bassett, K; Musini, VM	2013
Interventions to slow progression of myopia in children	Walline, , JJ, Lindsley, K, Vedula, SS, Cotter, SA, Mutti, DO; Twelker, JD	2011
Intravenous immunoglobulin for the treatment of Kawasaki disease in children	Oates-Whitehead, , RM, Baumer, JH, Haines, L, Love, S, Maconochie, IK, Gupta, A, Roman, K, Dua, JS; Flynn, I	2003
Interventions for vaginismus	Melnik, , T, Hawton, K; McGuire, H	2012
Interventions to improve adherence to antiretroviral therapy in children with HIV infection	Bain-Brickley, , D, Butler, LM, Kennedy, GE; Rutherford, GW	2011
Interventions to improve antibiotic prescribing practices for hospital inpatients	Davey, , P, Marwick, CA, Scott, CL, Charani, E, McNeil, K, Brown, E, Gould, IM, Ramsay, CR; Michie, S	2017
Interventions for treating painful sickle cell crisis during pregnancy	Martí-Carvajal, , AJ, Peña-Martí, GE, Comunián-Carrasco, G; Martí-Peña, AJ	2009
Interventions for the prevention of nutritional rickets in term born children	Lerch, , C; Meissner, T	2007
Interventions for tobacco use cessation in people in treatment for or recovery from substance use disorders	Apollonio, , D, Philipps, R; Bero, L	2016
Interventions for the treatment of fractures of the mandibular condyle	Sharif, , MO, Fedorowicz, Z, Drews, P, Nasser, M, Dorri, M, Newton, T; Oliver, R	2010
Interventions for the prevention and management of oropharyngeal candidiasis associated with HIV infection in adults and children	Pienaar, , ED, Young, T; Holmes, H	2010
Interventions to modify sexual risk behaviours for preventing HIV in homeless youth	Naranbhai, , V, Abdool Karim, Q; Meyer-Weitz, A	2011
Interventions to improve safe and effective medicines use by consumers: an overview of systematic reviews	Ryan, , R, Santesso, N, Lowe, D, Hill, S, Grimshaw, JM, Prictor, M, Kaufman, C, Cowie, G; Taylor, M	2014
Interventions to facilitate shared decision making to address antibiotic use for acute respiratory infections in primary care	Coxeter, , P, Del Mar, CB, McGregor, L, Beller, EM; Hoffmann, TC	2015
Interventions for the restorative care of amelogenesis imperfecta in children and adolescents	Dashash, , M, Yeung, CA, Jamous, I; Blinkhorn, A	2013
Intranasal corticosteroids for asthma control in people with coexisting asthma and rhinitis	Taramarcaz, , P; Gibson, PG	2003
Interventions for the management of dry mouth: topical therapies	Furness, , S, Worthington, HV, Bryan, G, Birchenough, S; McMillan, R	2011
Intravenous immunoglobulin for chronic inflammatory demyelinating polyradiculoneuropathy	Eftimov, , F, Winer, JB, Vermeulen, M, de Haan, R; van Schaik, IN	2013
Interventions for treating psoriatic arthritis	Jones, , G, Crotty, M; Brooks, P	2000
Interventions for treating postpartum constipation	Turawa, , EB, Musekiwa, A; Rohwer, AC	2014
Interventions to improve outpatient referrals from primary care to secondary care	Akbari, , A, Mayhew, A, Al-Alawi, MA, Grimshaw, J, Winkens, R, Glidewell, E, Pritchard, C, Thomas, R; Fraser, C	2008
Intravenous magnesium sulfate for treating adults with acute asthma in the emergency department	Kew, , KM, Kirtchuk, L; Michell, CI	2014
Intravenous or intramuscular parecoxib for acute postoperative pain in adults	Lloyd, , R, Derry, S, Moore, RA; McQuay, HJ	2009
Interventions for raising breast cancer awareness in women	O'Mahony, , M, Comber, H, Fitzgerald, T, Corrigan, MA, Fitzgerald, E, Grunfeld, EA, Flynn, MG; Hegarty, J	2017
Interventions for treating collagenous colitis	Kafil, , TS, Nguyen, TM, Patton, PH, MacDonald, JK, Chande, N; McDonald, JWD	2017
Interventions for sensory impairment in the upper limb after stroke	Doyle, , S, Bennett, S, Fasoli, SE; McKenna, KT	2010
Interventions for the management of oral ulcers in Behçet's disease	Taylor, , J, Glenny, AM, Walsh, T, Brocklehurst, P, Riley, P, Gorodkin, R; Pemberton, MN	2014
Interventions to reduce ambient particulate matter air pollution and their effect on health	Burns, , J, Boogaard, H, Polus, S, Pfadenhauer, LM, Rohwer, AC, van Erp, AM, Turley, R; Rehfuess, E	2019
Interventions to promote informed consent for patients undergoing surgical and other invasive healthcare procedures	Kinnersley, , P, Phillips, K, Savage, K, Kelly, MJ, Farrell, E, Morgan, B, Whistance, R, Lewis, V, Mann, MK, Stephens, BL, Blazeby, J, Elwyn, G; Edwards, AGK	2013
Intravenous magnesium for acute myocardial infarction	Li, , J, Zhang, Q, Zhang, M; Egger, M	2007
Interventions for the management of fatigue in adults with a primary brain tumour	Day, , J, Yust-Katz, S, Cachia, D, Wefel, J, Katz, LH, Tremont Lukats, IW, Bulbeck, H, Armstrong, T; Rooney, AG	2016
Intravenous immunoglobulin for myasthenia gravis	Gajdos, , P, Chevret, S; Toyka, KV	2012
Interventions for treating cholestasis in pregnancy	Gurung, , V, Stokes, M, Middleton, P, Milan, SJ, Hague, W; Thornton, JG	2013
Interventions to reduce waiting times for elective procedures	Ballini, , L, Negro, A, Maltoni, S, Vignatelli, L, Flodgren, G, Simera, I, Holmes, J; Grilli, R	2015
Intranasal steroids versus placebo or no intervention for chronic rhinosinusitis	Chong, , LY, Head, K, Hopkins, C, Philpott, C, Schilder, AGM; Burton, MJ	2016
Intratympanic gentamicin for Ménière's disease or syndrome	Pullens, , B; van Benthem, PP	2011
Intranasal ipratropium bromide for the common cold	AlBalawi, , ZH, Othman, SS; AlFaleh, K	2013

Interventions for tobacco use cessation in people living with HIV and AIDS	Pool, , ERM, Dogar, O, Lindsay, RP, Weatherburn, P; Siddiqi, K	2016
Interventions for treating acute elbow dislocations in adults	Taylor, , F, Sims, M, Theis, JC; Herbison, GP	2012
Interventions for replacing missing teeth: augmentation procedures of the maxillary sinus	Esposito, , M, Felice, P; Worthington, HV	2014
Interventions for renal vasculitis in adults	Walters, , G, Willis, NS; Craig, JC	2015
Interventions for trichomoniasis in pregnancy	Gülmezoglu, , AM; Azhar, M	2011
Interventions other than anticoagulants and systemic antibiotics for prevention of central venous catheter-related infections in children with cancer	Arora, , RS, Roberts, R, Eden, TOB; Pizer, B	2010
Intraoperative Mitomycin C for glaucoma surgery	Wilkins, , M, Indar, A; Wormald, R	2005
Interventions for strabismic amblyopia	Taylor, , K; Elliott, S	2014
Interventions for treating anxiety after stroke	Knapp, , P, Campbell Burton, CA, Holmes, J, Murray, J, Gillespie, D, Lightbody, CE, Watkins, CL, Chun, HYY; Lewis, SR	2017
Intramedullary nailing for tibial shaft fractures in adults	Duan, , X, Al-Qwbani, M, Zeng, Y, Zhang, W; Xiang, Z	2012
Interventions for the treatment of Frey's syndrome	Li, , C, Wu, F, Zhang, Q, Gao, Q, Shi, Z; Li, L	2015
Interventions to facilitate return to work in adults with adjustment disorders	Arends, , I, Bruinvels, DJ, Rebergen, DS, Nieuwenhuijsen, K, Madan, I, Neumeyer-Gromen, A, Bültmann, U; Verbeek, JH	2012
Intravenous naftidrofuryl for critical limb ischaemia	Smith, , FB, Bradbury, A; Fowkes, G	2012
Interventions to reduce acute and late adverse gastrointestinal effects of pelvic radiotherapy for primary pelvic cancers	Lawrie, , TA, Green, JT, Beresford, M, Wedlake, L, Burden, S, Davidson, SE, Lal, S, Henson, CC; Andreyev, HJN	2018
Interventions for the prevention of recurrent erysipelas and cellulitis	Dalal, , A, Eskin-Schwartz, M, Mimouni, D, Ray, S, Days, W, Hodak, E, Leibovici, L; Paul, M	2017
Interventions to improve adherence to lipid-lowering medication	van Driel, , ML, Morledge, MD, Ulep, R, Shaffer, JP, Davies, P; Deichmann, R	2016
Interventions for the prevention and treatment of pes cavus	Burns, , J, Landorf, KB, Ryan, MM, Crosbie, J; Ouvrier, RA	2007
Interventions for treating genital chlamydia trachomatis infection in pregnancy	Brocklehurst, , P; Rooney, G	1998
Interventions for recurrent idiopathic epistaxis (nosebleeds) in children	Qureishi, , A; Burton, MJ	2012
Interventions for treating fractures of the distal femur in adults	Griffin, , XL, Parsons, N, Zbaeda, MM; McArthur, J	2015
Interventions for smoking cessation in hospitalised patients	Rigotti, , NA, Clair, C, Munafò, MR; Stead, LF	2012
Interventions to prevent injuries in construction workers	van der Molen, , HF, Basnet, P, Hoonakker, PLT, Lehtola, MM, Lappalainen, J, Frings-Dresen, MHW, Haslam, R; Verbeek, JH	2018
Interventions for the physical aspects of sexual dysfunction in women following pelvic radiotherapy	Denton, , AS; Maher, J	2003
Interventions for weight reduction in obesity to improve survival in women with endometrial cancer	Kitson, , S, Ryan, N, MacKintosh, ML, Edmondson, R, Duffy, JMN; Crosbie, EJ	2018
Interventions to prevent hypothermia at birth in preterm and/or low birth weight infants	McCall, , EM, Alderdice, F, Halliday, HL, Vohra, S; Johnston, L	2018
Interventions to improve hand hygiene compliance in patient care	Gould, , DJ, Moralejo, D, Drey, N, Chudleigh, JH; Taljaard, M	2017
Interventions for treating genital Chlamydia trachomatis infection in pregnancy	Cluver, , C, Novikova, N, Eriksson, DOA, Bengtsson, K; Lingman, GK	2017
Intravenous immunoglobulins for multiple sclerosis	Gray, , O, McDonnell, GV; Forbes, RB	2003
Intravenous immunoglobulin for presumed viral myocarditis in children and adults	Robinson, , J, Hartling, L, Vandermeer, B; Klassen, TP	2015
Interventions for treating post-extraction bleeding	Kumbargere Nagraj, , S, Prashanti, E, Aggarwal, H, Lingappa, A, Muthu, MS, Kiran Kumar Krishanappa, S; Hassan, H	2018
Interventions for replacing missing teeth: antibiotics at dental implant placement to prevent complications	Esposito, , M, Grusovin, MG; Worthington, HV	2013
Intramuscular penicillin for the prevention of early onset group B streptococcal infection in newborn infants	Woodgate, , PG, Flenady, V; Steer, PA	2004
Interventions for treating painful nipples among breastfeeding women	Dennis, , CL, Jackson, K; Watson, J	2014
Intralesional treatment versus wide resection for central low-grade chondrosarcoma of the long bones	Dierselhuis, , EF, Goulding, KA, Stevens, M; Jutte, PC	2019
Intratracheal Clara cell secretory protein (CCSP) administration in preterm infants with or at risk of respiratory distress syndrome	Abdel-Latif, , ME; Osborn, DA	2011
Interventions for treating peripartum cardiomyopathy to improve outcomes for women and babies	Carlin, , AJ, Alfirevic, Z; Gyte, GML	2010
Intramuscular versus oral corticosteroids to reduce relapses following discharge from the emergency department for acute asthma	Kirkland, , SW, Cross, E, Campbell, S, Villa-Roel, C; Rowe, BH	2018
Interventions for treating burning mouth syndrome	McMillan, , R, Forssell, H, Buchanan, JAG, Glenny, AM, Weldon, JC; Zakrzewska, JM	2016
Interventions for replacing missing teeth: hyperbaric oxygen therapy for irradiated patients who require dental implants	Esposito, , M; Worthington, HV	2013

Intraoperative use of low volume ventilation to decrease postoperative mortality, mechanical ventilation, lengths of stay and lung injury in adults without acute lung injury	Guay, , J, Ochroch, EA; Kopp, S	2018
Interventions for treating leg ulcers in people with sickle cell disease	Martí-Carvajal, , AJ, Knight-Madden, JM; Martinez-Zapata, MJ	2014
Interventions to reduce risky sexual behaviour for preventing HIV infection in workers in occupational settings	Ojo, , O, Verbeek, JH, Rasanen, K, Heikkinen, J, Isotalo, LK, Mngoma, N; Ruotsalainen, E	2011
Intravenous or enteral loop diuretics for preterm infants with (or developing) chronic lung disease	Stewart, , A; Brion, LP	2011
Intravenous fluids for abdominal aortic surgery	Toomtong, , P; Suksompong, S	2010
Interventions to improve disposal of human excreta for preventing diarrhoea	Clasen, , TF, Bostoen, K, Schmidt, WP, Boisson, S, Fung, ICH, Jenkins, MW, Scott, B, Sugden, S; Cairncross, S	2010
Interventions for unilateral and bilateral refractive amblyopia	Taylor, , K, Powell, C, Hatt, SR; Stewart, C	2012
Interventions to improve question formulation in professional practice and self-directed learning	Horsley, , T, O'Neill, J, McGowan, J, Perrier, L, Kane, G; Campbell, C	2010
Interventions for treating intrahepatic cholestasis in people with sickle cell disease	Martí-Carvajal, , AJ; Martí-Amarista, CE	2017
Interventions for the treatment of metastatic extradural spinal cord compression in adults	George, , R, Sundararaj, JJ, Govindaraj, R, Chacko, AG; Tharyan, P	2015
Interventions for the management of dry mouth: non-pharmacological interventions	Furness, , S, Bryan, G, McMillan, R, Birchenough, S; Worthington, HV	2013
Interventions for treating oro-antral communications and fistulae due to dental procedures	Kiran Kumar Krishanappa, , S, Eachempati, P, Kumbargere Nagraj, S, Shetty, NY, Moe, S, Aggarwal, H; Mathew, RJ	2018
Intravascular brachytherapy for peripheral vascular disease	Andras, , A, Hansrani, M, Stewart, M; Stansby, G	2014
Interventions for smoking cessation in people diagnosed with lung cancer	Zeng, , L, Yu, X, Yu, T, Xiao, J; Huang, Y	2019
Intrauterine administration of human chorionic gonadotropin (hCG) for subfertile women undergoing assisted reproduction	Craciunas, , L, Tsampras, N, Raine-Fenning, N; Coomarasamy, A	2018
Interventions for treating fingertip entrapment injuries in children	Capstick, , R; Giele, H	2014
Interventions to reduce weight gain in schizophrenia	Faulkner, , G, Cohn, T; Remington, G	2007
Interventions for women with endometrioma prior to assisted reproductive technology	Benschop, , L, Farquhar, C, van der Poel, N; Heineman, MJ	2010
Interventions targeted at women to encourage the uptake of cervical screening	Everett, , T, Bryant, A, Griffin, MF, Martin-Hirsch, PPL, Forbes, CA; Jepson, RG	2011
Interventions for restoring patency of occluded central venous catheter lumens	van Miert, , C, Hill, R; Jones, L	2012
Intraoperative neuromonitoring versus visual nerve identification for prevention of recurrent laryngeal nerve injury in adults undergoing thyroid surgery	Cirocchi, , R, Arezzo, A, D'Andrea, V, Abraha, I, Popivanov, GI, Avenia, N, Gerardi, C, Henry, BM, Randolph, J; Barczyñski, M	2019
Interventions to help support caregivers of people with a brain or spinal cord tumour	Boele, , FW, Rooney, AG, Bulbeck, H; Sherwood, P	2019
Interventions for tobacco cessation in the dental setting	Carr, , AB; Ebbert, J	2012
Intranasal steroids for acute sinusitis	Zalmanovici Trestioreanu, , A; Yaphe, J	2013
Interventions for the eradication of meticillin-resistant Staphylococcus aureus (MRSA) in people with cystic fibrosis	Lo, , DKH, Muhlebach, MS; Smyth, AR	2018
Interventions for reducing inflammation in familial Mediterranean fever	Wu, , B, Xu, T, Li, Y; Yin, X	2018
Interventions in the alcohol server setting for preventing injuries	Ker, , K; Chinnock, P	2008
Interventions to reduce harm from continued tobacco use	Lindson-Hawley, , N, Hartmann-Boyce, J, Fanshawe, TR, Begh, R, Farley, A; Lancaster, T	2016
Interventions for treating acute bleeding episodes in people with acquired hemophilia A	Zeng, , Y, Zhou, R, Duan, X, Long, D; Yang, S	2014
Interventions for tophi in gout	Sriranganathan, , MK, Vinik, O, Bombardier, C; Edwards, CJ	2014
Interventions for reducing the risk of mother-to-child transmission of HIV infection	Brocklehurst, P	2002
Interventions to increase clinical incident reporting in health care	Parmelli, , E, Flodgren, G, Fraser, SG, Williams, N, Rubin, G; Eccles, MP	2012
Intravenous immunoglobulin for preventing infection in preterm and/or low birth weight infants	Ohlsson, , A; Lacy, JB	2013
Intramuscular versus intravenous prophylactic oxytocin for the third stage of labour	Oladapo, , OT, Okusanya, BO; Abalos, E	2018
Interventions to reduce corruption in the health sector	Gaitonde, , R, Oxman, AD, Okebukola, PO; Rada, G	2016
Interventions for treatment of neonatal hyperglycemia in very low birth weight infants	Bottino, , M, Cowett, RM; Sinclair, JC	2011
Interventions to improve the management of diabetes mellitus in primary care, outpatient and community settings	Renders, , CM, Valk, GD, Griffin, SJ, Wagner, E, van Eijk, JT; Assendelft, WJJ	2000
Intravenous immunoglobulins for epilepsy	Geng, , J, Dong, J, Li, Y, Ni, H, Jiang, K, Shi, LL; Wang, G	2017
Interventions to improve the appropriate use of polypharmacy for older people	Rankin, , A, Cadogan, CA, Patterson, SM, Kerse, N, Cardwell, CR, Bradley, MC, Ryan, C; Hughes, C	2018

Interventions using social networking sites to promote contraception in women of reproductive age	Jawad, , A, Jawad, I; Alwan, NA	2019
Intravenous beta2-agonists versus intravenous aminophylline for acute asthma	Travers, , AH, Jones, AP, Camargo Jr, CA, Milan, SJ; Rowe, BH	2012
Interventions to increase adherence to medications for tobacco dependence	Hollands, , GJ, Naughton, F, Farley, A, Lindson, N; Aveyard, P	2019
Interventions for treating neuropathic pain in people with sickle cell disease	Asnani, , MR, Francis, DK, Brandow, AM, Hammond Gabbadon, CEO; Ali, A	2019
Interventions to support return to work for people with coronary heart disease	Hegewald, , J, Wegewitz, UE, Euler, U, van Dijk, JL, Adams, J, Fishta, A, Heinrich, P; Seidler, A	2019
Interventions for treating acute high altitude illness	Simancas-Racines, , D, Arevalo-Rodriguez, I, Osorio, D, Franco, JVA, Xu, Y; Hidalgo, R	2018
Omega-3 fatty acids for the primary and secondary prevention of cardiovascular disease	Abdelhamid, , AS, Brown, TJ, Brainard, JS, Biswas, P, Thorpe, GC, Moore, HJ, Deane, KHO, AlAbdulghafoor, FK, Summerbell, CD, Worthington, HV, Song, F; Hooper, L	2018
Optimum duration of regimens for Helicobacter pylori eradication	Yuan, , Y, Ford, AC, Khan, KJ, Gisbert, JP, Forman, D, Leontiadis, GI, Tse, F, Calvet, X, Fallone, C, Fischbach, L, Oderda, G, Bazzoli, F; Moayyedi, P	2013
Non-steroidal anti-inflammatory drugs versus corticosteroids for controlling inflammation after uncomplicated cataract surgery	Juthani, , VV, Clearfield, E; Chuck, RS	2017
Naftopidil for the treatment of lower urinary tract symptoms compatible with benign prostatic hyperplasia	Hwang, , EC, Gandhi, S, Jung, JH, Imamura, M, Kim, MH, Pang, R; Dahm, P	2018
Non-invasive ventilation during exercise training for people with chronic obstructive pulmonary disease	Menadue, , C, Piper, AJ, van 't Hul, AJ; Wong, KK	2014
Omega-3 fatty acids for cystic fibrosis	Oliver, , C; Watson, H	2016
Nicotine for Alzheimer's disease	López-Arrieta, , J; Sanz, FJFJS	2001
Nutrient-enriched formula milk versus human breast milk for preterm infants following hospital discharge	Henderson, , G, Fahey, T; McGuire, W	2007
Non surgical therapy for anal fissure	Nelson, , RL, Thomas, K, Morgan, J; Jones, A	2012
Neoadjuvant chemotherapy for locally advanced cervix cancer	Tierney, , J; Rydzewska, L	2004
Omalizumab for asthma in adults and children	Normansell, , R, Walker, S, Milan, SJ, Walters, EH; Nair, P	2014
One-to-one dietary interventions undertaken in a dental setting to change dietary behaviour	Harris, , R, Gamboa, A, Dailey, Y; Ashcroft, A	2012
Non-steroidal anti-inflammatory agents to induce regression and prevent the progression of cervical intraepithelial neoplasia	Grabosch, , SM, Shariff, OM; Helm, CW	2018
Nutritional supplementation for hip fracture aftercare in older people	Avenell, , A, Smith, TO, Curtain, JP, Mak, JCS; Myint, PK	2016
Opioids for acute pancreatitis pain	Basurto Ona, , X, Rigau Comas, D; Urrútia, G	2013
One dose per day compared to multiple doses per day of gentamicin for treatment of suspected or proven sepsis in neonates	Rao, , SC, Srinivasjois, R; Moon, K	2016
Non-pharmacological interventions for fatigue in rheumatoid arthritis	Cramp, , F, Hewlett, S, Almeida, C, Kirwan, JR, Choy, EHS, Chalder, T, Pollock, J; Christensen, R	2013
Optimisation of chemotherapy and radiotherapy for untreated Hodgkin lymphoma patients with respect to second malignant neoplasms, overall and progression-free survival: individual participant data analysis	Franklin, , J, Eichenauer, DA, Becker, I, Monsef, I; Engert, A	2017
Mycophenolic acid versus azathioprine as primary immunosuppression for kidney transplant recipients	Wagner, , M, Earley, AK, Webster, AC, Schmid, CH, Balk, EM; Uhlig, K	2015
Non-pharmacological interventions for chronic pain in people with spinal cord injury	Boldt, , I, Eriks-Hoogland, I, Brinkhof, MWG, de Bie, R, Joggi, D; von Elm, E	2014
Nicotine for schizophrenia	Punnoose, , S; Belgamwar, MR	2006
Occupational therapy for rheumatoid arthritis	Steultjens, , EEMJ, Dekker, JJ, Bouter, LM, Schaardenburg, DD, Kuyk, MAMAH; Van den Ende, ECHM	2004
Nutritional support for acute kidney injury	Li, , Y, Tang, X, Zhang, J; Wu, T	2012
On-site mental health workers delivering psychological therapy and psychosocial interventions to patients in primary care: effects on the professional practice of primary care providers	Harkness, , EF; Bower, PJ	2009
Nutritional support in children and young people with cancer undergoing chemotherapy	Ward, , EJ, Henry, LM, Friend, AJ, Wilkins, S; Phillips, RS	2015
Opioid antagonists for alcohol dependence	Rösner, , S, Hackl-Herrwerth, A, Leucht, S, Vecchi, S, Srisurapanont, M; Soyka, M	2010
Non-steroid agents for idiopathic pulmonary fibrosis	Spagnolo, , P, Del Giovane, C, Luppi, F, Cerri, S, Balduzzi, S, Walters, EH, D'Amico, R; Richeldi, L	2010
Non-pharmaceutical management of respiratory morbidity in children with severe global developmental delay	Winfield, , NR, Barker, NJ, Turner, ER; Quin, GL	2014
Nutritional advice for improving outcomes in multiple pregnancies	Bricker, , L, Reed, K, Wood, L; Neilson, JP	2015
Neonatal vitamin A supplementation for the prevention of mortality and morbidity in term neonates in low and middle income countries	Haider, , BA, Sharma, R; Bhutta, ZA	2017
Non pharmacological interventions for spasticity in multiple sclerosis	Amatya, , B, Khan, F, La Mantia, L, Demetrios, M; Wade, DT	2013

Notification and support for people exposed to the risk of Creutzfeldt-Jakob disease (CJD) (or other prion diseases) through medical treatment (iatrogenically)	Ryan, , R, Hill, S, Lowe, D, Allen, K, Taylor, M; Mead, C	2011
Nimodipine for primary degenerative, mixed and vascular dementia	Birks, , J; López-Arrieta, J	2002
Narrow-band ultraviolet B phototherapy versus broad-band ultraviolet B or psoralen-ultraviolet A photochemotherapy for psoriasis	Chen, , X, Yang, M, Cheng, Y, Liu, GJ; Zhang, M	2013
Non-steroidal anti-inflammatory drugs for sciatica	Rasmussen-Barr, , E, Held, U, Grooten, WJA, Roelofs, PDDM, Koes, BW, van Tulder, MW; Wertli, MM	2016
Non-surgical interventions for treating heavy menstrual bleeding (menorrhagia) in women with bleeding disorders	Ray, , S; Ray, A	2016
Non-opioid drugs for pain management in labour	Othman, , M, Jones, L; Neilson, JP	2012
Optimal time for initiating antiretroviral therapy (ART) in HIV-infected, treatment-naive children aged 2 to 5 years old	Siegfried, , N, Davies, MA, Penazzato, M, Muhe, LM; Egger, M	2013
Non steroidal anti-inflammatory drugs (NSAID) and aspirin for preventing colorectal adenomas and carcinomas	Asano, , TK; McLeod, RS	2004
Optimisation of antiretroviral therapy in HIV-infected children under 3 years of age	Penazzato, , M, Prendergast, AJ, Muhe, LM, Tindyebwa, D; Abrams, E	2014
Nicotine receptor partial agonists for smoking cessation	Cahill, , K, Lindson-Hawley, N, Thomas, KH, Fanshawe, TR; Lancaster, T	2016
Optimal primary surgical treatment for advanced epithelial ovarian cancer	Elattar, , A, Bryant, A, Winter-Roach, BA, Hatem, M; Naik, R	2011
Nicotine vaccines for smoking cessation	Hartmann-Boyce, , J, Cahill, K, Hatsukami, D; Cornuz, J	2012
Opioids for agitation in dementia	Brown, , R, Howard, R, Candy, B; Sampson, EL	2015
Opioid therapy for treating rheumatoid arthritis pain	Whittle, , SL, Richards, BL, Husni, E; Buchbinder, R	2011
Neonatal screening for sickle cell disease	Lees, , C, Davies, SC; Dezateux, C	2000
Nutritional interventions for preventing and treating pressure ulcers	Langer, , G; Fink, A	2014
Opioid antagonists under heavy sedation or anaesthesia for opioid withdrawal	Gowing, , L, Ali, R; White, JM	2010
Neuromuscular electrostimulation for adults with chronic obstructive pulmonary disease	Hill, , K, Cavalheri, V, Mathur, S, Roig, M, Janaudis-Ferreira, T, Robles, P, Dolmage, TE; Goldstein, R	2018
Non-penetrating filtration surgery versus trabeculectomy for open-angle glaucoma	Eldaly, , MA, Bunce, C, ElSheikha, OZ; Wormald, R	2014
Non-contraceptive oestrogen-containing preparations for controlling symptoms of premenstrual syndrome	Naheed, , B, Kuiper, JH, Uthman, OA, O'Mahony, F; O'Brien, PMS	2017
Oestrogen and progestogen hormone replacement therapy for peri-menopausal and post-menopausal women: weight and body fat distribution	Kongnyuy, , EJ, Norman, RJ, Flight, IHK; Rees, MC	1999
Nebulised hypertonic saline for cystic fibrosis	Wark, , P; McDonald, VM	2018
Negative pressure wound therapy for treating leg ulcers	Dumville, , JC, Land, L, Evans, D; Peinemann, F	2015
Neuromodulators for pain management in rheumatoid arthritis	Richards, , BL, Whittle, SL; Buchbinder, R	2012
Nutritional interventions for preventing stunting in children (birth to 59 months) living in urban slums in low- and middle-income countries (LMIC)	Goudet, , SM, Bogin, BA, Madise, NJ; Griffiths, PL	2019
Natalizumab for induction of remission in Crohn's disease	Nelson, , SML, Nguyen, TM, McDonald, JWD; MacDonald, JK	2018
Niacin for primary and secondary prevention of cardiovascular events	Schandelmaier, , S, Briel, M, Saccilotto, R, Olu, KK, Arpagaus, A, Hemkens, LG; Nordmann, AJ	2017
Non-antipsychotic catecholaminergic drugs for antipsychotic-induced tardive dyskinesia	El-Sayeh, , HG, Rathbone, J, Soares-Weiser, K; Bergman, H	2018
Nebulised morphine for severe interstitial lung disease	Polosa, , R, Simidchiev, A; Walters, EH	2002
Nonsteroidal anti-inflammatory drugs for pain in women with endometriosis	Brown, , J, Crawford, TJ, Allen, C, Hopewell, S; Prentice, A	2017
Natural cycle in vitro fertilisation (IVF) for subfertile couples	Allersma, , T, Farquhar, C; Cantineau, AEP	2013
Nutritional support for liver disease	Koretz, , RL, Avenell, A; Lipman, TO	2012
Nebuliser systems for drug delivery in cystic fibrosis	Daniels, , T, Mills, N; Whitaker, P	2013
Non-surgical interventions for late rectal problems (proctopathy) of radiotherapy in people who have received radiotherapy to the pelvis	van de Wetering, , FT, Verleye, L, Andreyev, HJN, Maher, J, Vlayen, J, Pieters, BR, van Tienhoven, G; Scholten, RJPM	2016
Nutritional interventions for liver-transplanted patients	Langer, , G, Großmann, K, Fleischer, S, Berg, A, Grothues, D, Wienke, A, Behrens, J; Fink, A	2012
Omega 3 fatty acids (fish oil) for maintenance of remission in ulcerative colitis	Turner, , D, Steinhart, AH; Griffiths, AM	2007
Nasal decontamination for the prevention of surgical site infection in Staphylococcus aureus carriers	Liu, , Z, Norman, G, Iheozor-Ejiofor, Z, Wong, JKF, Crosbie, EJ; Wilson, P	2017
Omega 3 fatty acid for the prevention of cognitive decline and dementia	Sydenham, , E, Dangour, AD; Lim, WS	2012
Nonlatex versus latex male condoms for contraception	Gallo, , MF, Grimes, DA, Lopez, LM; Schulz, KF	2006

Optimal timing for intravascular administration set replacement	Ullman, , AJ, Cooke, ML, Gillies, D, Marsh, N, Daud, A, McGrail, MR, O'Riordan, E; Rickard, CM	2013
Nonsteroidal anti-inflammatory drugs (NSAIDS) versus opioids for acute renal colic	Holdgate, , A; Pollock, T	2004
Neuraminidase inhibitors for preventing and treating influenza in children (published trials only)	Wang, , K, Shun-Shin, M, Gill, P, Perera, R; Harnden, A	2012
Once versus twice daily low molecular weight heparin for the initial treatment of venous thromboembolism	Bhutia, , S; Wong, PF	2013
Opioids for the palliation of refractory breathlessness in adults with advanced disease and terminal illness	Barnes, , H, McDonald, J, Smallwood, N; Manser, R	2016
Nutritional supplements for people being treated for active tuberculosis	Grobler, , L, Nagpal, S, Sudarsanam, TD; Sinclair, D	2016
Nitric oxide donors for treating preterm labour	Duckitt, , K, Thornton, S, O'Donovan, OP; Dowswell, T	2014
Neuromuscular electrical stimulation for muscle weakness in adults with advanced disease	Jones, , S, Man, WDC, Gao, W, Higginson, IJ, Wilcock, A; Maddocks, M	2016
Non-pharmacological interventions for somatoform disorders and medically unexplained physical symptoms (MUPS) in adults	van Dessel, , N, den Boeft, M, van der Wouden, JC, Kleinstäuber, M, Leone, SS, Terluin, B, Numans, ME, van der Horst, HE; van Marwijk, H	2014
Newborn screening for cystic fibrosis	Southern, , KW, Mérelle, MME, Dankert-Roelse, JE; Nagelkerke, A	2009
Nitazoxanide for chronic hepatitis C	Nikolova, , K, Gluud, C, Grevstad, B; Jakobsen, JC	2014
Non-resection versus resection for an asymptomatic primary tumour in patients with unresectable Stage IV colorectal cancer	Cirocchi, , R, Trastulli, S, Abraha, I, Vettoretto, N, Boselli, C, Montedori, A, Parisi, A, Noya, G; Platell, C	2012
Non-nutritive sucking for increasing physiologic stability and nutrition in preterm infants	Foster, , JP, Psaila, K; Patterson, T	2016
Omega 3 fatty acids (fish oil) for maintenance of remission in Crohn's disease	Lev-Tzion, , R, Griffiths, AM, Ledder, O; Turner, D	2014
Omega-3 polyunsaturated fatty acids (PUFA) for type 2 diabetes mellitus	Hartweg, , J, Perera, R, Montori, VM, Dinneen, SF, Neil, AHAWN; Farmer, AJ	2008
Nebulised deoxyribonuclease for viral bronchiolitis in children younger than 24 months	Enriquez, , A, Chu, IW, Mellis, C; Lin, WY	2012
Oestrogens for treatment or prevention of pelvic organ prolapse in postmenopausal women	Ismail, , SI, Bain, C; Hagen, S	2010
Nebulised hypertonic saline solution for acute bronchiolitis in infants	Zhang, , L, Mendoza-Sassi, RA, Wainwright, C; Klassen, TP	2017
Non-immunosuppressive treatment for IgA nephropathy	Reid, , S, Cawthon, PM, Craig, JC, Samuels, JA, Molony, DA; Strippoli, GFM	2011
Non-steroidal anti-inflammatory drugs for low back pain	Roelofs, , PDDM, Deyo, RA, Koes, BW, Scholten, RJPM; van Tulder, MW	2008
Needle size for vaccination procedures in children and adolescents	Beirne, , PV, Hennessy, S, Cadogan, SL, Shiely, F, Fitzgerald, T; MacLeod, F	2018
Needle syringe programmes and opioid substitution therapy for preventing hepatitis C transmission in people who inject drugs	Platt, , L, Minozzi, S, Reed, J, Vickerman, P, Hagan, H, French, C, Jordan, A, Degenhardt, L, Hope, V, Hutchinson, S, Maher, L, Palmateer, N, Taylor, A, Bruneau, J; Hickman, M	2017
Nitric oxide for preventing pre-eclampsia and its complications	Meher, , S; Duley, L	2007
Octreotide for the treatment of chylothorax in neonates	Das, , A; Shah, PS	2010
Olanzapine for schizophrenia	Duggan, , L, Fenton, M, Rathbone, J, Dardennes, R, El-Dosoky, A; Indran, S	2005
Non-steroidal anti-inflammatory drugs for acute gout	van Durme, , CMPG, Wechalekar, MD, Buchbinder, R, Schlesinger, N, van der Heijde, D; Landewé, RBM	2014
N-acetylcarnosine (NAC) drops for age-related cataract	Dubois, , VDJP; Bastawrous, A	2017
Off-pump versus on-pump coronary artery bypass grafting for ischaemic heart disease	Møller, , CH, Penninga, L, Wetterslev, J, Steinbrüchel, DA; Gluud, C	2012
Omega-3 fatty acids for the treatment of dementia	Burckhardt, , M, Herke, M, Wustmann, T, Watzke, S, Langer, G; Fink, A	2016
Non-steroidal antiandrogen monotherapy compared with luteinising hormone–releasing hormone agonists or surgical castration monotherapy for advanced prostate cancer	Kunath, , F, Grobe, HR, Rücker, G, Motschall, E, Antes, G, Dahm, P, Wullich, B; Meerpohl, JJ	2014
Newer generation antidepressants for depressive disorders in children and adolescents	Hetrick, , SE, McKenzie, JE, Cox, GR, Simmons, MB; Merry, SN	2012
Nedocromil sodium for chronic asthma in children	Sridhar, , AV; McKean, MC	2006
Low protein diets for non-diabetic adults with chronic kidney disease	Hahn, , D, Hodson, EM; Fouque, D	2018
Open general medical wards versus specialist psychiatric units for acute psychoses	Hickling, , FW, Abel, W, Garner, P; Rathbone, J	2007
Lateral pararectal versus transrectal stoma placement for prevention of parastomal herniation	Hardt, , J, Meerpohl, JJ, Metzendorf, MI, Kienle, P, Post, S; Herrle, F	2019
Operative procedures for fissure in ano	Nelson, , RL, Chattopadhyay, A, Brooks, W, Platt, I, Paavana, T; Earl, S	2011
Lactase treated feeds to promote growth and feeding tolerance in preterm infants	Tan-Dy, , CRY; Ohlsson, A	2013
Non-pharmacological interventions for caregivers of stroke survivors	Legg, , LA, Quinn, TJ, Mahmood, F, Weir, CJ, Tierney, J, Stott, DJ, Smith, LN; Langhorne, P	2011

Magnesium for treating sickle cell disease	Than, , NN, Soe, HHK, Palaniappan, SK, Abas, ABL; De Franceschi, L	2019
N-acetylcysteine for sepsis and systemic inflammatory response in adults	Szakmany, , T, Hauser, B; Radermacher, P	2012
Ischaemic preconditioning for the reduction of renal ischaemia reperfusion injury	Menting, , TP, Wever, KE, Ozdemir-van Brunschot, DMD, Van der Vliet, DJA, Rovers, MM; Warle, MC	2017
Non-pharmacological interventions for preventing secondary vascular events after stroke or transient ischemic attack	MacKay-Lyons, , M, Thornton, M, Ruggles, T; Che, M	2013
Low molecular weight heparin for prevention of microvascular occlusion in digital replantation	Chen, , YC, Chi, CC, Chan, FC; Wen, YW	2013
Naftidrofuryl for intermittent claudication	de Backer, , TLM, Vander Stichele, R, Lehert, P; Van Bortel, L	2012
Laser photocoagulation for proliferative diabetic retinopathy	Evans, , JR, Michelessi, M; Virgili, G	2014
Noninvasive positive pressure ventilation for acute respiratory failure following upper abdominal surgery	Faria, , DAS, da Silva, EMK, Atallah, ÁN; Vital, FMR	2015
Laser-assisted subepithelial keratectomy (LASEK) versus photorefractive keratectomy (PRK) for correction of myopia	Li, , SM, Zhan, S, Li, SY, Peng, XX, Hu, J, Law, HA; Wang, NL	2016
Non-steroidal anti-inflammatory agents for treating cystoid macular oedema following cataract surgery	Sivaprasad, , S, Bunce, C; Crosby-Nwaobi, R	2012
Lay health workers in primary and community health care for maternal and child health and the management of infectious diseases	Lewin, , S, Munabi-Babigumira, S, Glenton, C, Daniels, K, Bosch-Capblanch, X, van Wyk, BE, Odgaard-Jensen, J, Johansen, M, Aja, GN, Zwarenstein, M; Scheel, IB	2010
Optimal loading dose of warfarin for the initiation of oral anticoagulation	Mahtani, , KR, Heneghan, CJ, Nunan, D, Bankhead, C, Keeling, D, Ward, AM, Harrison, SE, Roberts, NW, Hobbs, FDR; Perera, R	2012
Long-term pituitary down-regulation before in vitro fertilization (IVF) for women with endometriosis	Sallam, , HN, Garcia-Velasco, JA, Dias, S, Arici, A; Abou-Setta, AM	2006
Neuroreflexotherapy for non-specific low-back pain	Urrútia, , G, Burton, AK, Morral Fernández, A, Bonfill Cosp, X; Zanoli, G	2004
Janus kinase-1 and Janus kinase-2 inhibitors for treating myelofibrosis	Martí-Carvajal, , AJ, Anand, V; Solà, I	2015
Non-pharmacological interventions for chronic pain in multiple sclerosis	Amatya, , B, Young, J; Khan, F	2018
Laparoscopic versus open liver resection for benign and malignant hepatic lesions in adults	Rao, , AM; Ahmed, I	2013
Once-daily versus multiple-daily dosing with intravenous aminoglycosides for cystic fibrosis	Bhatt, , J, Jahnke, N; Smyth, AR	2019
Intra-articular steroids and splints/rest for children with juvenile idiopathic arthritis and adults with rheumatoid arthritis	Wallen, , MM; Gillies, D	2006
Non-invasive brain stimulation techniques for chronic pain	O'Connell, , NE, Marston, L, Spencer, S, DeSouza, LH; Wand, BM	2018
Naftidrofuryl for dementia	Lu, , D, Song, H, Hao, Z, Wu, T; McCleery, J	2011
Nutritional interventions for reducing gastrointestinal toxicity in adults undergoing radical pelvic radiotherapy	Henson, , CC, Burden, S, Davidson, SE; Lal, S	2013
Nivolumab for adults with Hodgkin's lymphoma (a rapid review using the software RobotReviewer)	Goldkuhle, , M, Dimaki, M, Gartlehner, G, Monsef, I, Dahm, P, Glossmann, JP, Engert, A, von Tresckow, B; Skoetz, N	2018
Neuraxial blockade for the prevention of postoperative mortality and major morbidity: an overview of Cochrane systematic reviews	Guay, , J, Choi, P, Suresh, S, Albert, N, Kopp, S; Pace, NL	2014
Non-surgical interventions for convergence insufficiency	Scheiman, , M, Gwiazda, J; Li, T	2011
Non-invasive positive pressure ventilation for treatment of respiratory failure due to severe acute exacerbations of asthma	Lim, , WJ, Mohammed Akram, R, Carson, KV, Mysore, S, Labiszewski, NA, Wedzicha, JA, Rowe, BH; Smith, BJ	2012
Non-steroidal anti-inflammatory drugs for heavy menstrual bleeding	Lethaby, , A, Duckitt, K; Farquhar, C	2013
Nonoperative treatment for lumbar spinal stenosis with neurogenic claudication	Ammendolia, , C, Stuber, KJ, Rok, E, Rampersaud, R, Kennedy, CA, Pennick, V, Steenstra, IA, de Bruin, LK; Furlan, AD	2013
Non-steroidal anti-inflammatory drugs as disease-modifying agents for Parkinson's disease: evidence from observational studies	Rees, , K, Stowe, R, Patel, S, Ives, N, Breen, K, Clarke, CE; Ben-Shlomo, Y	2011
Non-pharmacological interventions for preventing job loss in workers with inflammatory arthritis	Hoving, , JL, Lacaille, D, Urquhart, DM, Hannu, TJ, Sluiter, JK; Frings-Dresen, MHW	2014
One-to-one oral hygiene advice provided in a dental setting for oral health	Soldani, , FA, Lamont, T, Jones, K, Young, L, Walsh, T, Lala, R; Clarkson, JE	2018
Nitrous Oxide for Colonoscopy	Aboumarzouk, , OM, Agarwal, T, Syed Nong Chek, SAH, Milewski, PJ; Nelson, RL	2011
Non-steroidal anti-inflammatory drugs (NSAIDs) for axial spondyloarthritis (ankylosing spondylitis and non-radiographic axial spondyloarthritis)	Kroon, , FPB, van der Burg, LRA, Ramiro, S, Landewé, RBM, Buchbinder, R, Falzon, L; van der Heijde, D	2015
Nutrient-enriched formula versus standard formula for preterm infants following hospital discharge	Young, , L, Embleton, ND; McGuire, W	2016
Nitric oxide donors (nitrates), L-arginine, or nitric oxide synthase inhibitors for acute stroke	Bath, , PMW, Krishnan, K; Appleton, JP	2017
Optical reading aids for children and young people with low vision	Barker, , L, Thomas, R, Rubin, G; Dahlmann-Noor, A	2015
Nitrates for the prevention of cardiac morbidity and mortality in patients undergoing non-cardiac surgery	Zhao, , N, Xu, J, Singh, B, Yu, X, Wu, T; Huang, Y	2016
Non-operative management versus operative management in high-grade blunt hepatic injury	Cirocchi, , R, Trastulli, S, Pressi, E, Farinella, E, Avenia, S, Morales Uribe, CH, Botero, AM; Barrera, LM	2015

N-acetylcysteine as an adjuvant therapy for Helicobacter pylori eradication	Fontes, , LES, Martimbianco, ALC, Zanin, C; Riera, R	2019
Occlusal splints for treating sleep bruxism (tooth grinding)	Macedo, , CR, Silva, AB, Machado, MAC, Saconato, H; Prado, GF	2007
Optimal intensity and type of leg exercise training for people with chronic obstructive pulmonary disease	Zainuldin, , R, Mackey, MG; Alison, JA	2011
Non-pharmacological interventions for preventing venous insufficiency in a standing worker population	Robertson, , L, Yeoh, SE; Kolbach, DN	2013
Optimal monitoring strategies for guiding when to switch first-line antiretroviral therapy regimens for treatment failure in adults and adolescents living with HIV in low-resource settings	Chang, , LW, Harris, J; Humphreys, EH	2010
Opioid antagonists with minimal sedation for opioid withdrawal	Gowing, , L, Ali, R; White, JM	2017
Neurosurgical interventions for the treatment of classical trigeminal neuralgia	Zakrzewska, , JM; Akram, H	2011
Nutritional supplementation for stable chronic obstructive pulmonary disease	Ferreira, , IM, Brooks, D, White, J; Goldstein, R	2012
Omentoplasty for oesophagogastrostomy after oesophagectomy	Yuan, , Y, Zeng, X, Hu, Y, Xie, T; Zhao, Y	2014
Non-medical prescribing versus medical prescribing for acute and chronic disease management in primary and secondary care	Weeks, , G, George, J, Maclure, K; Stewart, D	2016
Olanzapine versus other atypical antipsychotics for schizophrenia	Komossa, , K, Rummel-Kluge, C, Hunger, H, Schmid, F, Schwarz, S, Duggan, L, Kissling, W; Leucht, S	2010
Nebulised surfactant in preterm infants with or at risk of respiratory distress syndrome	Abdel-Latif, , ME; Osborn, DA	2012
Olanzapine for the prevention and treatment of cancer-related nausea and vomiting in adults	Sutherland, , A, Naessens, K, Plugge, E, Ware, L, Head, K, Burton, MJ; Wee, B	2018
New treatments compared to established treatments in randomized trials	Djulbegovic, , B, Kumar, A, Glasziou, PP, Perera, R, Reljic, T, Dent, L, Raftery, J, Johansen, M, Di Tanna, GL, Miladinovic, B, Soares, HP, Vist, GE; Chalmers, I	2012
Non-invasive positive pressure ventilation (CPAP or bilevel NPPV) for cardiogenic pulmonary oedema	Berbenetz, , N, Wang, Y, Brown, J, Godfrey, C, Ahmad, M, Vital, FMR, Lambiase, P, Banerjee, A, Bakhai, A; Chong, M	2019
Omega-3 fatty acid addition during pregnancy	Middleton, , P, Gomersall, JC, Gould, JF, Shepherd, E, Olsen, SF; Makrides, M	2018
Non-pharmacological interventions for treating chronic prostatitis/chronic pelvic pain syndrome	Franco, , JVA, Turk, T, Jung, JH, Xiao, YT, Iakhno, S, Garrote, V; Vietto, V	2018
Nutritional interventions for reducing morbidity and mortality in people with HIV	Grobler, , L, Siegfried, N, Visser, ME, Mahlungulu, SSN; Volmink, J	2013
Omega-3 fatty acids for intermittent claudication	Campbell, , A, Price, J; Hiatt, WR	2013
Newborn screening for homocystinuria	Walter, , JH, Jahnke, N; Remmington, T	2015
Oestrogen therapy for urinary incontinence in post-menopausal women	Cody, , JD, Jacobs, ML, Richardson, K, Moehrer, B; Hextall, A	2012
Nurse versus physician-led care for the management of asthma	Kuethe, , MC, Vaessen-Verberne, AAPH, Elbers, RG; Van Aalderen, WMC	2013
Neo-adjuvant chemotherapy for invasive bladder cancer	Collaboration, Advanced Bladder Cancer Meta-analysis	2004
Omega 3 fatty acids for preventing or slowing the progression of age-related macular degeneration	Lawrenson, , JG; Evans, JR	2015
Nutritional interventions for survivors of childhood cancer	Cohen, , JE, Wakefield, CE; Cohn, RJ	2016
Non-invasive ventilation for cystic fibrosis	Moran, , F, Bradley, JM; Piper, AJ	2017
Occlusion for stimulus deprivation amblyopia	Antonio-Santos, , A, Vedula, SS, Hatt, SR; Powell, C	2014
Occupational safety and health enforcement tools for preventing occupational diseases and injuries	Mischke, , C, Verbeek, JH, Job, J, Morata, TC, Alvesalo-Kuusi, A, Neuvonen, K, Clarke, S; Pedlow, RI	2013
Nitrous oxide-based techniques versus nitrous oxide-free techniques for general anaesthesia	Sun, , R, Jia, WQ, Zhang, P, Yang, K, Tian, JH, Ma, B, Liu, Y, Jia, RH, Luo, XF; Kuriyama, A	2015
Non-drug therapies for lower limb muscle cramps	Hawke, , F, Chuter, V, Walter, KEL; Burns, J	2012
Nitrates for acute heart failure syndromes	Wakai, , A, McCabe, A, Kidney, R, Brooks, SC, Seupaul, RA, Diercks, DB, Salter, N, Fermann, GJ; Pospisil, C	2013
Olanzapine IM or velotab for acutely disturbed/agitated people with suspected serious mental illnesses	Belgamwar, , RB; Fenton, M	2005
Nebulized and oral thiol derivatives for pulmonary disease in cystic fibrosis	Tam, , J, Nash, EF, Ratjen, F, Tullis, E; Stephenson, A	2013
Non-surgical interventions for eosinophilic esophagitis	Elliott, , EJ, Thomas, D; Markowitz, JE	2010
Omega-3 fatty acids for depression in adults	Appleton, , KM, Sallis, HM, Perry, R, Ness, AR; Churchill, R	2015
Neostigmine for reversal of neuromuscular block in paediatric patients	Yang, , L, Yang, D, Li, Q, Zuo, Y; Lu, D	2014
Occupational therapy for care home residents with stroke	Fletcher-Smith, , JC, Walker, MF, Cobley, CS, Steultjens, EMJ; Sackley, CM	2013
Neuropsychological and psychological interventions for people with newly diagnosed epilepsy	Jackson, , CF, Makin, SM; Baker, GA	2015
Nut consumption for the primary prevention of cardiovascular disease	Martin, , N, Germanò, R, Hartley, L, Adler, AJ; Rees, K	2015

Non-pharmacological interventions for cognitive impairment due to systemic cancer treatment	Treanor, , CJ, McMenamin, UC, O'Neill, RF, Cardwell, CR, Clarke, MJ, Cantwell, M; Donnelly, M	2016
Omega-6 fats for the primary and secondary prevention of cardiovascular disease	Hooper, , L, Al-Khudairy, L, Abdelhamid, AS, Rees, K, Brainard, JS, Brown, TJ, Ajabnoor, SM, O'Brien, AT, Winstanley, LE, Donaldson, DH, Song, F; Deane, KHO	2018
Negative pressure wound therapy for surgical wounds healing by primary closure	Webster, , J, Liu, Z, Norman, G, Dumville, JC, Chiverton, L, Scuffham, P, Stankiewicz, M; Chaboyer, WP	2019
Nurse-led versus doctor-led care for bronchiectasis	Lawton, , K, Royals, K, Carson-Chahhoud, KV, Campbell, F; Smith, BJ	2018
Nerve-sparing radical hysterectomy compared to standard radical hysterectomy for women with early stage cervical cancer (stage Ia2 to IIa)	Kietpeerakool, , C, Aue-aungkul, A, Galaal, K, Ngamjarus, C; Lumbiganon, P	2019
Once daily long-acting beta2-agonists and long-acting muscarinic antagonists in a combined inhaler versus placebo for chronic obstructive pulmonary disease	Maqsood, , U, Ho, TN, Palmer, K, Eccles, FJR, Munavvar, M, Wang, R, Crossingham, I; Evans, DJW	2019
Optical coherence tomography for diagnosing skin cancer in adults	Ferrante di Ruffano, , L, Dinnes, J, Deeks, JJ, Chuchu, N, Bayliss, SE, Davenport, C, Takwoingi, Y, Godfrey, K, O'Sullivan, C, Matin, RN, Tehrani, H; Williams, HC	2018
Non-invasive positive pressure ventilation for prevention of complications after pulmonary resection in lung cancer patients	Torres, , MFS, Porfírio, GJM, Carvalho, APV; Riera, R	2019
Non-pharmacological interventions for perceptual disorders following stroke and other adult-acquired, non-progressive brain injury	Bowen, , A, Knapp, P, Gillespie, D, Nicolson, DJ; Vail, A	2011
Nutrient-enriched formula versus standard formula for preterm infants	Walsh, , V, Brown, JVE, Askie, LM, Embleton, ND; McGuire, W	2019
Palliative endobronchial brachytherapy for non-small cell lung cancer	Reveiz, , L, Rueda, JR; Cardona, AF	2012
Patellar tendon versus hamstring tendon autograft for anterior cruciate ligament rupture in adults	Mohtadi, , NGH, Chan, DS, Dainty, KN; Whelan, DB	2011
Oxytocin for preventing postpartum haemorrhage (PPH) in non-facility birth settings	Pantoja, , T, Abalos, E, Chapman, E, Vera, C; Serrano, VP	2016
Palivizumab for prophylaxis against respiratory syncytial virus infection in children with cystic fibrosis	Robinson, , KA, Odelola, OA; Saldanha, IJ	2016
Oral anticoagulants versus antiplatelet therapy for preventing stroke in patients with non-valvular atrial fibrillation and no history of stroke or transient ischemic attacks	Aguilar, , MI, Hart, R; Pearce, LA	2007
Oral substitution treatment of injecting opioid users for prevention of HIV infection	Gowing, , L, Farrell, MF, Bornemann, R, Sullivan, LE; Ali, R	2011
Parent-initiated oral corticosteroid therapy for intermittent wheezing illnesses in children	Vuillermin, , P, South, M; Robertson, C	2006
Pegloticase for chronic gout	Anderson, , A; Singh, JA	2010
Patient isolation measures for infants with candida colonization or infection for preventing or reducing transmission of candida in neonatal units	Pammi, , M, Eddama, O; Weisman, LE	2011
Oral versus intra-vaginal imidazole and triazole anti-fungal treatment of uncomplicated vulvovaginal candidiasis (thrush)	Nurbhai, , M, Grimshaw, J, Watson, M, Bond, CM, Mollison, JA; Ludbrook, A	2007
Perioperative antibiotics to prevent infection after first-trimester abortion	Low, , N, Mueller, M, Van Vliet, HAAM; Kapp, N	2012
Pain relief for the removal of femoral sheath after percutaneous coronary intervention	Wensley, , C, Kent, B, McAleer, MB, Savage, SM; Stewart, JT	2008
Oxygen therapy for cystic fibrosis	Elphick, , HE; Mallory, G	2013
Oxcarbazepine for acute affective episodes in bipolar disorder	Vasudev, , A, Macritchie, K, Vasudev, K, Watson, S, Geddes, J; Young, AH	2011
Paracetamol versus nonsteroidal anti-inflammatory drugs for rheumatoid arthritis	Wienecke, , T; Gøtzsche, PC	2004
Oral immunoglobulin for preventing necrotizing enterocolitis in preterm and low birth weight neonates	Foster, , JP, Seth, R; Cole, MJ	2016
Perioperative dexmedetomidine for acute pain after abdominal surgery in adults	Jessen Lundorf, , L, Korvenius Nedergaard, H; Møller, AM	2016
Oral paracetamol (acetaminophen) for cancer pain	Wiffen, , PJ, Derry, S, Moore, RA, McNicol, ED, Bell, RF, Carr, DB, McIntyre, M; Wee, B	2017
Options for self-management education for adults with asthma	Powell, , H; Gibson, PG	2002
Oral 5-aminosalicylic acid for maintenance of surgically-induced remission in Crohn's disease	Gjuladin-Hellon, , T, Gordon, M, Iheozor-Ejiofor, Z; Akobeng, AK	2019
Pentoxifylline for treating venous leg ulcers	Jull, , AB, Arroll, B, Parag, V; Waters, J	2012
Oral antivirals for preventing recurrent herpes simplex keratitis in people with corneal grafts	Bhatt, , UK, Abdul Karim, MN, Prydal, JI, Maharajan, SV; Fares, U	2016
Perianal injectable bulking agents as treatment for faecal incontinence in adults	Maeda, , Y, Laurberg, S; Norton, C	2013
Oral antihistamine-decongestant-analgesic combinations for the common cold	De Sutter, , AIM, van Driel, ML, Kumar, AA, Lesslar, O; Skrt, A	2012
Perioperative fluid volume optimization following proximal femoral fracture	Lewis, , SR, Butler, AR, Brammar, A, Nicholson, A; Smith, AF	2016
Palliative biliary stents for obstructing pancreatic carcinoma	Moss, , AC, Morris, E; MacMathuna, P	2006

Oxytocin receptor antagonists for inhibiting preterm labour	Flenady, , V, Reinebrant, HE, Liley, HG, Tambimuttu, EG; Papatsonis, DNM	2014
Percutaneous transluminal coronary angioplasty with stents versus coronary artery bypass grafting for people with stable angina or acute coronary syndromes	Bakhai, , A, Hill, RA, Dundar, Y, Dickson, RC; Walley, T	2005
Oral appliances and functional orthopaedic appliances for obstructive sleep apnoea in children	Carvalho, , FR, Lentini-Oliveira, DA, Prado, LBF, Prado, GF; Carvalho, LBC	2016
Oral immunoglobulin for the prevention of rotavirus infection in low birth weight infants	Pammi, , M; Haque, KN	2011
Oral or topical nasal steroids for hearing loss associated with otitis media with effusion in children	Simpson, , SA, Lewis, R, van der Voort, J; Butler, CC	2011
Oral appliances for obstructive sleep apnoea	Lim, , J, Lasserson, TJ, Fleetham, J; Wright, JJ	2006
Organisational interventions for improving wellbeing and reducing work-related stress in teachers	Naghieh, , A, Montgomery, P, Bonell, CP, Thompson, M; Aber, JL	2015
Oral immunoglobulin for the treatment of rotavirus diarrhea in low birth weight infants	Pammi, , M; Haque, KN	2011
Perioperative antibiotics for prevention of acute endophthalmitis after cataract surgery	Gower, , EW, Lindsley, K, Tulenko, SE, Nanji, AA, Leyngold, I; McDonnell, PJ	2017
Orthotic devices for the treatment of tennis elbow	Struijs, , PAA, Smidt, N, Arola, H, van Dijk, CN, Buchbinder, R; Assendelft, WJJ	2002
Perioperative blood transfusions and recurrence of colorectal cancer	Amato, , A; Pescatori, M	2006
Oral naltrexone maintenance treatment for opioid dependence	Minozzi, , S, Amato, L, Vecchi, S, Davoli, M, Kirchmayer, U; Verster, A	2011
Oral budesonide for induction of remission in ulcerative colitis	Sherlock, , ME, MacDonald, JK, Griffiths, AM, Steinhart, AH; Seow, CH	2015
Oxatomide for stable asthma in adults and children	Hayashi, , K, Yanagi, M, Wood-Baker, R, Takamatsu, IIT; Anami, KKA	2003
Oral deferiprone for iron chelation in people with thalassaemia	Fisher, , SA, Brunskill, SJ, Doree, C, Chowdhury, O, Gooding, S; Roberts, DJ	2013
Oral contraceptives for functional ovarian cysts	Grimes, , DA, Jones, LB, Lopez, LM; Schulz, KF	2014
Oral potassium iodide for the treatment of sporotrichosis	Xue, , S, Gu, R, Wu, T, Zhang, M; Wang, X	2009
Perioperative angiotensin-converting enzyme inhibitors or angiotensin II type 1 receptor blockers for preventing mortality and morbidity in adults	Zou, , Z, Yuan, HB, Yang, B, Xu, F, Chen, XY, Liu, GJ; Shi, XY	2016
Percutaneous cholecystostomy for high-risk surgical patients with acute calculous cholecystitis	Gurusamy, , KS, Rossi, M; Davidson, BR	2013
Oscillating devices for airway clearance in people with cystic fibrosis	Morrison, , L; Milroy, S	2017
Patch angioplasty versus primary closure for carotid endarterectomy	Rerkasem, , K; Rothwell, PM	2009
Oxycodone for neuropathic pain in adults	Gaskell, , H, Derry, S, Stannard, C; Moore, RA	2016
Percutaneous vascular interventions for acute ischaemic stroke	O'Rourke, , K, Berge, E, Walsh, CD; Kelly, PJ	2010
Oral versus intravenous fluoropyrimidines for colorectal cancer	Chionh, , F, Lau, D, Yeung, Y, Price, T; Tebbutt, N	2017
Oral theophylline for chronic obstructive pulmonary disease	Ram, , FSF, Jones, P, Jardim, J, Castro, AA, Atallah, ÁN, Lacasse, Y, Goldstein, R; Cendon, S	2002
Oral anti-diabetic pharmacological therapies for the treatment of women with gestational diabetes	Brown, , J, Martis, R, Hughes, B, Rowan, J; Crowther, CA	2017
Orbital radiotherapy for adult thyroid eye disease	Rajendram, , R, Bunce, C, Lee, RWJ; Morley, AMS	2012
Oral and sublingual immunotherapy for egg allergy	Romantsik, , O, Tosca, MA, Zappettini, S; Calevo, MG	2018
Parent-mediated early intervention for young children with autism spectrum disorders (ASD)	Oono, , IP, Honey, EJ; McConachie, H	2013
Parenteral anticoagulation in ambulatory patients with cancer	Akl, , EA, Kahale, LA, Hakoum, MB, Matar, CF, Sperati, F, Barba, M, Yosuico, VED, Terrenato, I, Synnot, A; Schünemann, H	2017
Patches of different types for carotid patch angioplasty	Rerkasem, , K; Rothwell, PM	2010
Oral anti-diabetic agents for women with established diabetes/impaired glucose tolerance or previous gestational diabetes planning pregnancy, or pregnant women with pre-existing diabetes	Tieu, , J, Coat, S, Hague, W, Middleton, P; Shepherd, E	2017
Patient reminder and recall interventions to improve immunization rates	Jacobson Vann, , JC, Jacobson, RM, Coyne-Beasley, T, Asafu-Adjei, JK; Szilagyi, PG	2018
Pathogen-reduced platelets for the prevention of bleeding	Estcourt, , LJ, Malouf, R, Hopewell, S, Trivella, M, Doree, C, Stanworth, SJ; Murphy, MF	2017
Oral steroids for adhesive capsulitis	Buchbinder, , R, Green, S, Youd, JM; Johnston, RV	2006
Oral H1 antihistamines as monotherapy for eczema	Apfelbacher, , CJ, van Zuuren, EJ, Fedorowicz, Z, Jupiter, A, Matterne, U; Weisshaar, E	2013
Oral vitamin B12 versus intramuscular vitamin B12 for vitamin B12 deficiency	Wang, , H, Li, L, Qin, LL, Song, Y, Vidal-Alaball, J; Liu, TH	2018
Oral beta-blockers for mild to moderate hypertension during pregnancy	Magee, , L; Duley, L	2003
Oral antiviral therapy for prevention of genital herpes outbreaks in immunocompetent and nonpregnant patients	Le Cleach, , L, Trinquart, L, Do, G, Maruani, A, Lebrun-Vignes, B, Ravaud, P; Chosidow, O	2014

PCSK9 monoclonal antibodies for the primary and secondary prevention of cardiovascular disease	Schmidt, , AF, Pearce, LS, Wilkins, JT, Overington, JP, Hingorani, AD; Casas, JP	2017
Oral adsorbents for preventing or delaying the progression of chronic kidney disease	Wu, , HM, Sun, HJ, Wang, F, Yang, M, Dong, BR; Liu, GJ	2014
Penicillin for secondary prevention of rheumatic fever	Manyemba, , J; Mayosi, BM	2002
Pancreatic enzymes for chronic pancreatitis	Shafiq, , N, Rana, S, Bhasin, D, Pandhi, P, Srivastava, P, Sehmby, SS, Kumar, R; Malhotra, S	2009
Penile rehabilitation for postprostatectomy erectile dysfunction	Philippou, , YA, Jung, JH, Steggall, MJ, O'Driscoll, ST, Bakker, CJ, Bodie, JA; Dahm, P	2018
Pancreatic enzyme replacement therapy for people with cystic fibrosis	Somaraju, , UR; Solis-Moya, A	2016
Orthodontic treatment for prominent upper front teeth (Class II malocclusion) in children and adolescents	Batista, , KBSL, Thiruvenkatachari, B, Harrison, JE; O'Brien, KD	2018
Oral contraceptive pill for primary dysmenorrhoea	Wong, , CL, Farquhar, C, Roberts, H; Proctor, M	2009
Oral treatments for fungal infections of the skin of the foot	Bell-Syer, , SEM, Khan, SM; Torgerson, DJ	2012
Oral nonsteroidal anti-inflammatory drugs for fibromyalgia in adults	Derry, , S, Wiffen, PJ, Häuser, W, Mücke, M, Tölle, TR, Bell, RF; Moore, RA	2017
Over-the-counter (OTC) medications to reduce cough as an adjunct to antibiotics for acute pneumonia in children and adults	Chang, , CC, Cheng, AC; Chang, AB	2014
Oral betamimetics for maintenance therapy after threatened preterm labour	Dodd, , JM, Crowther, CA; Middleton, P	2012
Oxygen for breathlessness in patients with chronic obstructive pulmonary disease who do not qualify for home oxygen therapy	Ekström, , M, Ahmadi, Z, Bornefalk-Hermansson, A, Abernethy, A; Currow, D	2016
Palliative chemotherapy and targeted therapies for esophageal and gastroesophageal junction cancer	Janmaat, , VT, Steyerberg, EW, van der Gaast, A, Mathijssen, RHJ, Bruno, MJ, Peppelenbosch, MP, Kuipers, EJ; Spaander, MCW	2017
Overground physical therapy gait training for chronic stroke patients with mobility deficits	States, , RA, Pappas, E; Salem, Y	2009
Pain management for inflammatory arthritis (rheumatoid arthritis, psoriatic arthritis, ankylosing spondylitis and other spondyloarthritis) and gastrointestinal or liver comorbidity	Radner, , H, Ramiro, S, Buchbinder, R, Landewé, RBM, van der Heijde, D; Aletaha, D	2012
Oxygen therapy for lower respiratory tract infections in children between 3 months and 15 years of age	Rojas-Reyes, , MX, Granados Rugeles, C; Charry-Anzola, LP	2014
Percutaneous lines for delivering intravenous antibiotics in people with cystic fibrosis	Prayle, , AP, Hurley, MN; Smyth, AR	2010
Oral anticoagulation in people with cancer who have no therapeutic or prophylactic indication for anticoagulation	Kahale, , LA, Hakoum, MB, Tsolakian, IG, Matar, CF, Barba, M, Yosuico, VED, Terrenato, I, Sperati, F, Schünemann, H; Akl, EA	2017
Oral health educational interventions for nursing home staff and residents	Albrecht, , M, Kupfer, R, Reissmann, DR, Mühlhauser, I; Köpke, S	2016
Oral protein calorie supplementation for children with chronic disease	Francis, , DK, Smith, J, Saljuqi, T; Watling, RM	2015
Perioperative enhanced recovery programmes for gynaecological cancer patients	Lu, , D, Wang, X; Shi, G	2015
Perioperative chemo(radio)therapy versus primary surgery for resectable adenocarcinoma of the stomach, gastroesophageal junction, and lower esophagus	Ronellenfitsch, , U, Schwarzbach, M, Hofheinz, R, Kienle, P, Kieser, M, Slanger, TE; Jensen, K	2013
Pelargonium sidoides extract for treating acute respiratory tract infections	Timmer, , A, Günther, J, Motschall, E, Rücker, G, Antes, G; Kern, WV	2013
Oral iron supplements for children in malaria-endemic areas	Neuberger, , A, Okebe, J, Yahav, D; Paul, M	2016
Pentoxifylline for diabetic kidney disease	Shan, , D, Wu, HM, Yuan, QY, Li, J, Zhou, RL; Liu, GJ	2012
Pedicle screw fixation for traumatic fractures of the thoracic and lumbar spine	Cheng, , LM, Wang, JJ, Zeng, ZL, Zhu, R, Yu, Y, Li, C; Wu, ZR	2013
Perioperative increase in global blood flow to explicit defined goals and outcomes following surgery	Grocott, , MPW, Dushianthan, A, Hamilton, MA, Mythen, MG, Harrison, D; Rowan, K	2012
Oral non-steroidal anti-inflammatory drugs versus other oral analgesic agents for acute soft tissue injury	Jones, , P, Dalziel, SR, Lamdin, R, Miles-Chan, JL; Frampton, C	2015
Orthodontic treatment for distalising upper first molars in children and adolescents	Jambi, , S, Thiruvenkatachari, B, O'Brien, KD; Walsh, T	2013
Oral contraceptives containing drospirenone for premenstrual syndrome	Lopez, , LM, Kaptein, AA; Helmerhorst, FM	2012
Oral xanthines as maintenance treatment for asthma in children	Seddon, , P, Bara, A, Lasserson, TJ; Ducharme, FM	2006
Percutaneous ethanol injection for liver metastases	Riemsma, , RP, Bala, MM, Wolff, R; Kleijnen, J	2013
Ovarian stimulation protocols (anti-oestrogens, gonadotrophins with and without GnRH agonists/antagonists) for intrauterine insemination (IUI) in women with subfertility	Cantineau, , AEP; Cohlen, BJ	2007
Palliative surgery versus medical management for bowel obstruction in ovarian cancer	Kucukmetin, , A, Naik, R, Galaal, K, Bryant, A; Dickinson, HO	2010
Patient-controlled analgesia with remifentanil versus alternative parenteral methods for pain management in labour	Weibel, , S, Jelting, Y, Afshari, A, Pace, NL, Eberhart, LHJ, Jokinen, J, Artmann, T; Kranke, P	2017
Pain management for rheumatoid arthritis and cardiovascular or renal comorbidity	Marks, , JL, Colebatch, AN, Buchbinder, R; Edwards, CJ	2011
Periodontal therapy as adjunctive treatment for gastric Helicobacter pylori infection	Ren, , Q, Yan, X, Zhou, Y; Li, WX	2016

Pentoxifylline, propentofylline and pentifylline for acute ischaemic stroke	Bath, , PMW; Bath-Hextall, FJ	2004
Oral stimulation for promoting oral feeding in preterm infants	Greene, , Z, O'Donnell, CPF; Walshe, M	2016
Pentoxifylline for treatment of sepsis and necrotizing enterocolitis in neonates	Pammi, , M; Haque, KN	2015
Partial exchange transfusion to prevent neurodevelopmental disability in infants with polycythemia	Özek, , E, Soll, R; Schimmel, MS	2010
Oximes for acute organophosphate pesticide poisoning	Buckley, , NA, Eddleston, M, Li, Y, Bevan, M; Robertson, J	2011
Parenting interventions for the prevention of unintentional injuries in childhood	Kendrick, , D, Mulvaney, CA, Ye, L, Stevens, T, Mytton, JA; Stewart-Brown, S	2013
Oral steroids for long-term use in cystic fibrosis	Cheng, , K, Ashby, D; Smyth, RL	2015
Participation in environmental enhancement and conservation activities for health and well-being in adults: a review of quantitative and qualitative evidence	Husk, , K, Lovell, R, Cooper, C, Stahl-Timmins, W; Garside, R	2016
Oral zinc for treating diarrhoea in children	Lazzerini, , M; Wanzira, H	2016
Oxcarbazepine for neuropathic pain	Zhou, , M, Chen, N, He, L, Yang, M, Zhu, C; Wu, F	2017
Ovulation suppression for endometriosis for women with subfertility	Hughes, , E, Brown, J, Collins, JJ, Farquhar, C, Fedorkow, DM; Vanderkerchove, P	2007
Patient education for preventing diabetic foot ulceration	Dorresteijn, , JAN, Kriegsman, DMW, Assendelft, WJJ; Valk, GD	2014
Percutaneous transluminal balloon angioplasty and stenting for carotid artery stenosis	Bonati, , LH, Lyrer, P, Ederle, J, Featherstone, R; Brown, MM	2012
Percutaneous transluminal angioplasty and stenting for vertebral artery stenosis	Coward, , L, Featherstone, R; Brown, MM	2005
Oral calorie supplements for cystic fibrosis	Smyth, , RL; Rayner, O	2017
Pegylated liposomal doxorubicin for first-line treatment of epithelial ovarian cancer	Lawrie, , TA, Rabbie, R, Thoma, C; Morrison, J	2013
Osteotomy for treating knee osteoarthritis	Brouwer, , RW, Huizinga, MR, Duivenvoorden, T, van Raaij, TM, Verhagen, AP, Bierma-Zeinstra, SMA; Verhaar, JAN	2014
Oral versus injectable ovulation induction agents for unexplained subfertility	Athaullah, , N, Proctor, M; Johnson, N	2002
Oral antifungal medication for toenail onychomycosis	Kreijkamp-Kaspers, , S, Hawke, K, Guo, L, Kerin, G, Bell-Syer, SEM, Magin, P, Bell-Syer, SV; van Driel, ML	2017
Peginterferon plus ribavirin versus interferon plus ribavirin for chronic hepatitis C	Hauser, , G, Awad, T, Brok, J, Thorlund, K, Štimac, D, Mabrouk, M, Gluud, C; Gluud, LL	2014
Orientation and mobility training for adults with low vision	Virgili, , G; Rubin, G	2010
Partial nephrectomy versus radical nephrectomy for clinical localised renal masses	Kunath, , F, Schmidt, S, Krabbe, LM, Miernik, A, Dahm, P, Cleves, A, Walther, M; Kroeger, N	2017
Penfluridol for schizophrenia	Soares, , BGO; Silva de Lima, M	2006
Pericyazine for schizophrenia	Matar, , HE, Almerie, MQ, Makhoul, S, Xia, J; Humphreys, P	2014
Percutaneous transluminal angioplasty for treatment of chronic cerebrospinal venous insufficiency (CCSVI) in people with multiple sclerosis	Jagannath, , VA, Pucci, E, Asokan, GV; Robak, EW	2019
Parenteral versus oral iron therapy for adults and children with chronic kidney disease	O'Lone, , EL, Hodson, EM, Nistor, I, Bolignano, D, Webster, AC; Craig, JC	2019
Oral Astragalus (Huang qi) for preventing frequent episodes of acute respiratory tract infection in children	Su, , G, Chen, X, Liu, Z, Yang, L, Zhang, L, Stålsby Lundborg, C, Wen, Z, Guo, X, Qin, X, Liang, J; Liu, X	2016
Payment methods for outpatient care facilities	Yuan, , B, He, L, Meng, Q; Jia, L	2017
Patient support and education for promoting adherence to highly active antiretroviral therapy for HIV/AIDS	Rueda, , S, Park-Wyllie, LY, Bayoumi, A, Tynan, AM, Antoniou, T, Rourke, S; Glazier, R	2006
Oxcarbazepine in the maintenance treatment of bipolar disorder	Vasudev, , A, Macritchie, K, Watson, S, Geddes, J; Young, AH	2008
Perioperative glycaemic control for diabetic patients undergoing surgery	Buchleitner, , AM, Martínez-Alonso, M, Hernández, M, Solà, I; Mauricio, D	2012
Oxygen therapy for acute myocardial infarction	Cabello, , JB, Burls, A, Emparanza, JI, Bayliss, SE; Quinn, T	2016
Oral anticoagulants for preventing stroke in patients with non-valvular atrial fibrillation and no previous history of stroke or transient ischemic attacks	Aguilar, , MI; Hart, R	2005
Palliative care interventions in advanced dementia	Murphy, , E, Froggatt, K, Connolly, S, O'Shea, E, Sampson, EL, Casey, D; Devane, D	2016
Patellar taping for patellofemoral pain syndrome in adults	Callaghan, , MJ; Selfe, J	2012
Pentoxifylline for alcoholic hepatitis	Whitfield, , K, Rambaldi, A, Wetterslev, J; Gluud, C	2009
Pain-relieving agents for infantile colic	Biagioli, , E, Tarasco, V, Lingua, C, Moja, L; Savino, F	2016
Oropharyngeal colostrum in preventing mortality and morbidity in preterm infants	Nasuf, , AWA, Ojha, S; Dorling, J	2018
Orthodontic and orthopaedic treatment for anterior open bite in children	Lentini-Oliveira, , DA, Carvalho, FR, Rodrigues, CG, Ye, Q, Hu, R, Minami-Sugaya, H, Carvalho, LBC, Prado, LBF; Prado, GF	2014

Oxcarbazepine versus phenytoin monotherapy for epilepsy: an individual participant data review	Nevitt, , SJ, Tudur Smith, C; Marson, AG	2018
Oral and intrauterine progestogens for atypical endometrial hyperplasia	Luo, , L, Luo, B, Zheng, Y, Zhang, H, Li, J; Sidell, N	2018
Paracetamol (acetaminophen) or non-steroidal anti-inflammatory drugs, alone or combined, for pain relief in acute otitis media in children	Sjoukes, , A, Venekamp, RP, van de Pol, AC, Hay, AD, Little, P, Schilder, AGM; Damoiseaux, RAMJ	2016
Perazine for schizophrenia	Leucht, , S, Helfer, B; Hartung, B	2014
Padma 28 for intermittent claudication	Stewart, , M, Morling, JR; Maxwell, H	2016
Paracetamol versus placebo or physical methods for treating fever in children	Meremikwu, , MM; Oyo-Ita, A	2002
Oral corticosteroids for stable chronic obstructive pulmonary disease	Walters, , JAE, Walters, EH; Wood-Baker, R	2005
Paracetamol (acetaminophen) for prevention or treatment of pain in newborns	Ohlsson, , A; Shah, PS	2016
Oral care measures for preventing nursing home-acquired pneumonia	Liu, , C, Cao, Y, Lin, J, Ng, L, Needleman, I, Walsh, T; Li, C	2018
Outpatient treatment for people with cancer who develop a low-risk febrile neutropaenic event	Rivas-Ruiz, , R, Villasis-Keever, M, Miranda-Novales, G, Castelán-Martínez, OD; Rivas-Contreras, S	2019
Oxygen therapy during exercise training in chronic obstructive pulmonary disease	Nonoyama, , M, Brooks, D, Lacasse, Y, Guyatt, GH; Goldstein, R	2007
Perioperative alcohol cessation intervention for postoperative complications	Egholm, , JWM, Pedersen, B, Møller, AM, Adami, J, Juhl, CB; Tønnesen, H	2018
Oral traditional Chinese medication for adhesive small bowel obstruction	Suo, , T, Gu, X, Andersson, R, Ma, H, Zhang, W, Deng, W, Zhang, B, Cai, D; Qin, X	2012
Patient education for adults with rheumatoid arthritis	Riemsma, , RP, Kirwan, JR, Taal, E; Rasker, HJJ	2003
Pentoxifylline for the prevention of bronchopulmonary dysplasia in preterm infants	Schulzke, , SM, Kaempfen, S; Patole, SK	2014
Patient education for neck pain	Gross, , A, Forget, M, St George, K, Fraser, MMH, Graham, N, Perry, L, Burnie, SJ, Goldsmith, CH, Haines, T; Brunarski, D	2012
Parenteral fluid regimens for improving functional outcome in people with acute stroke	Visvanathan, , A, Dennis, M; Whiteley, W	2015
Oral immunotherapy for milk allergy	Yeung, , JP, Kloda, LA, McDevitt, J, Ben-Shoshan, M; Alizadehfar, R	2012
Partial liquid ventilation for preventing death and morbidity in adults with acute lung injury and acute respiratory distress syndrome	Galvin, , IM, Steel, A, Pinto, R, Ferguson, ND; Davies, MW	2013
Orthodontic treatment for posterior crossbites	Agostino, , P, Ugolini, A, Signori, A, Silvestrini-Biavati, A, Harrison, JE; Riley, P	2014
Pentoxifylline for intermittent claudication	Salhiyyah, , K, Forster, R, Senanayake, E, Abdel-Hadi, M, Booth, A; Michaels, JA	2015
Peginterferon alpha-2a versus peginterferon alpha-2b for chronic hepatitis C	Hauser, , G, Awad, T, Thorlund, K, Štimac, D, Mabrouk, M; Gluud, C	2014
Pacing for drug-refractory or drug-intolerant hypertrophic cardiomyopathy	Qintar, , M, Morad, A, Alhawasli, H, Shorbaji, K, Firwana, B, Essali, A; Kadro, W	2012
Percutaneous needle aspiration, injection, and re-aspiration with or without benzimidazole coverage for uncomplicated hepatic hydatid cysts	Nasseri-Moghaddam, , S, Abrishami, A, Taefi, A; Malekzadeh, R	2011
Oral H1 antihistamines as 'add-on' therapy to topical treatment for eczema	Matterne, , U, Böhmer, MM, Weisshaar, E, Jupiter, A, Carter, B; Apfelbacher, CJ	2019
Oral anti-pseudomonal antibiotics for cystic fibrosis	Remmington, , T, Jahnke, N; Harkensee, C	2016
Perioperative medications for preventing temporarily increased intraocular pressure after laser trabeculoplasty	Zhang, , L, Weizer, JS; Musch, DC	2017
Peer support for people with schizophrenia or other serious mental illness	Chien, , WT, Clifton, AV, Zhao, S; Lui, S	2019
Paracetamol versus placebo for knee and hip osteoarthritis	Leopoldino, , AO, Machado, GC, Ferreira, PH, Pinheiro, MB, Day, R, McLachlan, AJ, Hunter, DJ; Ferreira, ML	2019
Percutaneous central venous catheters versus peripheral cannulae for delivery of parenteral nutrition in neonates	Ainsworth, , S; McGuire, W	2015
Percutaneous vascular interventions versus intravenous thrombolytic treatment for acute ischaemic stroke	Lindekleiv, , H, Berge, E, Bruins Slot, KMH; Wardlaw, JM	2018
Oral isotretinoin for acne	Costa, , CS, Bagatin, E, Martimbianco, ALC, da Silva, EMK, Lúcio, MM, Magin, P; Riera, R	2018
Oral vasodilators for primary Raynaud's phenomenon	Stewart, , M; Morling, JR	2012
Parent-mediated interventions for promoting communication and language development in young children with Down syndrome	O'Toole, , C, Lee, ASY, Gibbon, FE, van Bysterveldt, AK; Hart, NJ	2018
Pay for performance for hospitals	Mathes, , T, Pieper, D, Morche, J, Polus, S, Jaschinski, T; Eikermann, M	2019
Selective serotonin reuptake inhibitors for premenstrual syndrome	Marjoribanks, , J, Brown, J, O'Brien, PMS; Wyatt, K	2013
Selenium supplementation for asthma	Allam, , MF; Lucena, RA	2004
Splenectomy for people with thalassaemia major or intermedia	Easow Mathew, , M, Sharma, A; Aravindakshan, R	2016
Screening women for intimate partner violence in healthcare settings	O'Doherty, , L, Hegarty, K, Ramsay, J, Davidson, LL, Feder, G; Taft, A	2015
School-based programmes for preventing smoking	Thomas, , RE, McLellan, J; Perera, R	2013

Single layer versus double layer suture anastomosis of the gastrointestinal tract	Sajid, , MS, Siddiqui, MRS; Baig, MK	2012
Short-term late-generation antibiotics versus longer term penicillin for acute streptococcal pharyngitis in children	Altamimi, , S, Khalil, A, Khalaiwi, KA, Milner, RA, Pusic, MV; Al Othman, MA	2012
Specialist outreach clinics in primary care and rural hospital settings.	Gruen, , RL, Weeramanthri, TS, Knight, SS; Bailie, RS	2003
Standard (head-down tilt) versus modified (without head-down tilt) postural drainage in infants and young children with cystic fibrosis	Freitas, , DA, Chaves, GSS, Santino, TA, Ribeiro, CTD, Dias, FAL, Guerra, RO; Mendonça, KMPP	2018
Standard versus biofilm antimicrobial susceptibility testing to guide antibiotic therapy in cystic fibrosis	Waters, , V; Ratjen, F	2017
Scalpel versus no-scalpel incision for vasectomy	Cook, , LA, Pun, A, Gallo, MF, Lopez, LM; Van Vliet, HAAM	2014
Semen preparation techniques for intrauterine insemination	Boomsma, , CM, Heineman, MJ, Cohlen, BJ; Farquhar, C	2007
Specialist home-based nursing services for children with acute and chronic illnesses	Parab, , CS, Cooper, C, Woolfenden, S; Piper, SM	2013
Sodium channel blockers for cystic fibrosis	Burrows, , EF, Southern, KW; Noone, PG	2014
Slow advancement of enteral feed volumes to prevent necrotising enterocolitis in very low birth weight infants	Oddie, , SJ, Young, L; McGuire, W	2017
Self-management education and regular practitioner review for adults with asthma	Gibson, , PG, Powell, H, Wilson, A, Abramson, MJ, Haywood, P, Bauman, A, Hensley, MJ, Walters, EH; Roberts, JJL	2002
Sleep positioning systems for children with cerebral palsy	Blake, , SF, Logan, S, Humphreys, G, Matthews, J, Rogers, M, Thompson-Coon, J, Wyatt, K; Morris, C	2015
Screening for lung cancer	Manser, , R, Lethaby, A, Irving, LB, Stone, C, Byrnes, G, Abramson, MJ; Campbell, D	2013
Short-term treatment with proton pump inhibitors, H2-receptor antagonists and prokinetics for gastro-oesophageal reflux disease-like symptoms and endoscopy negative reflux disease	Sigterman, , KE, van Pinxteren, B, Bonis, PA, Lau, J; Numans, ME	2013
Stage-based interventions for smoking cessation	Cahill, , K, Lancaster, T; Green, N	2010
Second-line chemotherapy for non-small cell lung cancer	Bonfill Cosp, , X, Serra, C, Sacristan, M, Nogué, M, Losa, F; Montesinos, J	2002
Single dose oral aspirin for acute postoperative pain in adults	Derry, , S; Moore, RA	2012
Screening for colorectal cancer using the faecal occult blood test, Hemoccult	Hewitson, , P, Glasziou, PP, Irwig, L, Towler, B; Watson, E	2007
Specific allergen immunotherapy for the treatment of atopic eczema	Tam, , H, Calderon, MA, Manikam, L, Nankervis, H, García Núñez, I, Williams, HC, Durham, S; Boyle, RJ	2016
Sound therapy (masking) in the management of tinnitus in adults	Hobson, , J, Chisholm, E; El Refaie, A	2012
Single dose oral diflunisal for acute postoperative pain in adults	Wasey, , JO, Derry, S, Moore, RA; McQuay, HJ	2010
Short-course antibiotics for acute otitis media	Kozyrskyj, , AL, Klassen, TP, Moffatt, M; Harvey, K	2010
Shared care across the interface between primary and specialty care in management of long term conditions	Smith, , SM, Cousins, G, Clyne, B, Allwright, S; O'Dowd, T	2017
Single dose oral sulindac for acute postoperative pain in adults	Moore, , RA, Derry, S; McQuay, HJ	2009
Sertraline versus other antidepressive agents for depression	Cipriani, , A, La Ferla, T, Furukawa, TA, Signoretti, A, Nakagawa, A, Churchill, R, McGuire, H; Barbui, C	2010
Single dose oral dexibuprofen [S(+)-ibuprofen] for acute postoperative pain in adults	Derry, , S, Best, J; Moore, RA	2013
Special care units for dementia individuals with behavioural problems	Lai, , CKY, Yeung, JHM, Mok, V; Chi, I	2009
Single agent versus combination chemotherapy for metastatic breast cancer	Carrick, , S, Parker, S, Thornton, CE, Ghersi, D, Simes, J; Wilcken, N	2009
Single dose dipyrone for acute renal colic pain	Edwards, , J, Meseguer, F, Faura, C, Moore, RA, McQuay, HJ; Derry, S	2002
Second-generation antipsychotics for obsessive compulsive disorder	Komossa, , K, Depping, AM, Meyer, M, Kissling, W; Leucht, S	2010
Single dose oral tenoxicam for acute postoperative pain in adults	Moore, , OA, McIntyre, M, Moore, RA, Derry, S; McQuay, HJ	2009
Simple behavioural interventions for nocturnal enuresis in children	Caldwell, , PHY, Nankivell, G; Sureshkumar, P	2013
Sodium-glucose cotransporter (SGLT) 2 inhibitors for prevention or delay of type 2 diabetes mellitus and its associated complications in people at risk for the development of type 2 diabetes mellitus	Hemmingsen, , B, Krogh, J, Metzendorf, MI; Richter, B	2016
Silver-coated endotracheal tubes for prevention of ventilator-associated pneumonia in critically ill patients	Tokmaji, , G, Vermeulen, H, Müller, MCA, Kwakman, PHS, Schultz, MJ; Zaat, SAJ	2015
Sodium bicarbonate supplements for treating acute kidney injury	Hewitt, , J, Uniacke, M, Hansi, NK, Venkat-Raman, G; McCarthy, K	2012
Single dose oral fenbufen for acute postoperative pain in adults	Moore, , RA, Derry, S; McQuay, HJ	2009
Selenium supplementation to prevent short-term morbidity in preterm neonates	Darlow, , BA; Austin, N	2003
Single dose oral tiaprofenic acid for acute postoperative pain in adults	Moore, , RA, Derry, S, Moore, M; McQuay, HJ	2009
Saline nasal irrigation for acute upper respiratory tract infections	King, , D, Mitchell, B, Williams, CP; Spurling, GKP	2015

Second-generation antipsychotics for major depressive disorder and dysthymia	Komossa, , K, Depping, AM, Gaudchau, A, Kissling, W; Leucht, S	2010
Second-generation antipsychotics for anxiety disorders	Depping, , AM, Komossa, K, Kissling, W; Leucht, S	2010
Sentinel lymph node biopsy followed by lymph node dissection for localised primary cutaneous melanoma	Kyrgidis, , A, Tzellos, T, Mocellin, S, Apalla, Z, Lallas, A, Pilati, P; Stratigos, A	2015
Short-course versus prolonged-course antibiotic therapy for hospital-acquired pneumonia in critically ill adults	Pugh, , R, Grant, C, Cooke, RPD; Dempsey, G	2015
School-based interventions for improving contraceptive use in adolescents	Lopez, , LM, Bernholc, A, Chen, M; Tolley, EE	2016
Sound reduction management in the neonatal intensive care unit for preterm or very low birth weight infants	Almadhoob, , A; Ohlsson, A	2015
Single dose oral nabumetone for acute postoperative pain in adults	Moore, , RA, Derry, S, Moore, M; McQuay, HJ	2009
Selective serotonin reuptake inhibitors (SSRIs) for stroke recovery	Mead, , GE, Hsieh, CF, Lee, R, Kutlubaev, MA, Claxton, A, Hankey, GJ; Hackett, ML	2012
Self-certification versus physician certification of sick leave for reducing sickness absence and associated costs	Kausto, , J, Verbeek, JH, Ruotsalainen, JH, Halonen, JI, Virta, LJ; Kankaanpää, E	2019
Self-management education for cystic fibrosis	Savage, , E, Beirne, PV, Ni Chroinin, M, Duff, A, Fitzgerald, T; Farrell, D	2014
Skin antisepsis for reducing central venous catheter-related infections	Lai, , NM, Lai, NA, O'Riordan, E, Chaiyakunapruk, N, Taylor, JE; Tan, K	2016
Short versus long duration infusions of paclitaxel for any advanced adenocarcinoma	Williams, , C; Bryant, A	2011
Shouldice technique versus other open techniques for inguinal hernia repair	Amato, , B, Moja, L, Panico, S, Persico, G, Rispoli, C, Rocco, N; Moschetti, I	2012
Selenium supplementation for the primary prevention of cardiovascular disease	Rees, , K, Hartley, L, Day, C, Flowers, N, Clarke, A; Stranges, S	2013
Short acting beta2-agonists for recurrent wheeze in children under two years of age	Chavasse, , RJPG, Seddon, P, Bara, A; McKean, MC	2002
Single dose oral codeine, as a single agent, for acute postoperative pain in adults	Derry, , S, Moore, RA; McQuay, HJ	2010
Simulated presence therapy for dementia	Abraha, , I, Rimland, JM, Lozano-Montoya, I, Dell'Aquila, G, Vélez-Díaz-Pallarés, M, Trotta, FM, Cruz-Jentoft, AJ; Cherubini, A	2017
Self-management education programmes for osteoarthritis	Kroon, , FPB, van der Burg, LRA, Buchbinder, R, Osborne, RH, Johnston, RV; Pitt, V	2014
Simultaneous bilateral training for improving arm function after stroke	Coupar, , F, Pollock, A, van Wijck, F, Morris, J; Langhorne, P	2010
Self management for patients with chronic obstructive pulmonary disease	Zwerink, , M, Brusse-Keizer, M, van der Valk, PDLPM, Zielhuis, GA, Monninkhof, EM, van der Palen, J, Frith, PA; Effing, T	2014
Sodium channel blockers for neuroprotection in multiple sclerosis	Yang, , C, Hao, Z, Zhang, L, Zeng, L; Wen, J	2015
Skin patch and vaginal ring versus combined oral contraceptives for contraception	Lopez, , LM, Grimes, DA, Gallo, MF, Stockton, LL; Schulz, KF	2013
Sequential versus standard triple first-line therapy for Helicobacter pylori eradication	Nyssen, , OP, McNicholl, AG, Megraud, F, Savarino, V, Oderda, G, Fallone, CA, Fischbach, L, Bazzoli, F; Gisbert, JP	2016
Sequential combination of glucocorticosteroids and alfa interferon versus alfa interferon alone for HBeAg-positive chronic hepatitis B	Mellerup, , MT, Krogsgaard, K, Mathurin, P, Gluud, C; Poynard, T	2005
Sildenafil citrate for erectile dysfunction in patients with multiple sclerosis	Xiao, , Y, Wang, J; Luo, H	2012
Scalpel versus electrosurgery for major abdominal incisions	Charoenkwan, , K, Iheozor-Ejiofor, Z, Rerkasem, K; Matovinovic, E	2017
Single-incision sling operations for urinary incontinence in women	Nambiar, , A, Cody, JD, Jeffery, ST; Aluko, P	2017
Screening for oesophageal cancer	Yang, , S, Wu, S, Huang, Y, Shao, Y, Chen, XY, Xian, L, Zheng, J, Wen, Y, Chen, X, Li, H; Yang, C	2012
Stapled versus conventional surgery for hemorrhoids	Lumb, , KJ, Colquhoun, PH, Malthaner, R; Jayaraman, S	2006
Selenium supplementation for critically ill adults	Allingstrup, , M; Afshari, A	2015
Sentinel node assessment for diagnosis of groin lymph node involvement in vulval cancer	Lawrie, , TA, Patel, A, Martin-Hirsch, PPL, Bryant, A, Ratnavelu, NDG, Naik, R; Ralte, A	2014
Shared decision making interventions for people with mental health conditions	Duncan, , E, Best, C; Hagen, S	2010
Simple urethral dilatation, endoscopic urethrotomy, and urethroplasty for urethral stricture disease in adult men	Wong, , SSW, Aboumarzouk, OM, Narahari, R, O'Riordan, A; Pickard, R	2012
Secondary suturing compared to non-suturing for broken down perineal wounds following childbirth	Dudley, , LM, Kettle, C; Ismail, KMK	2013
Single versus combination intravenous anti-pseudomonal antibiotic therapy for people with cystic fibrosis	Elphick, , HE; Scott, A	2016
Single dose oral fenoprofen for acute postoperative pain in adults	Traa, , MX, Derry, S; Moore, RA	2011
Saline irrigation for chronic rhinosinusitis	Chong, , LY, Head, K, Hopkins, C, Philpott, C, Glew, S, Scadding, G, Burton, MJ; Schilder, AGM	2016
Speech therapy for children with dysarthria acquired before three years of age	Pennington, , L, Parker, NK, Kelly, H; Miller, N	2016
Short-course oral steroids alone for chronic rhinosinusitis	Head, , K, Chong, LY, Hopkins, C, Philpott, C, Burton, MJ; Schilder, AGM	2016

School-based physical activity programs for promoting physical activity and fitness in children and adolescents aged 6 to 18	Dobbins, , M, Husson, H, DeCorby, K; LaRocca, RL	2013
Selective serotonin reuptake inhibitors for fibromyalgia syndrome	Walitt, , B, Urrútia, G, Nishishinya, MB, Cantrell, SE; Häuser, W	2015
Single dose oral mefenamic acid for acute postoperative pain in adults	Moll, , R, Derry, S, Moore, RA; McQuay, HJ	2011
Sedation versus general anaesthesia for provision of dental treatment to patients younger than 18 years	Ashley, , PF, Williams, CECS, Moles, DR; Parry, J	2015
Short-course oral steroids as an adjunct therapy for chronic rhinosinusitis	Head, , K, Chong, LY, Hopkins, C, Philpott, C, Schilder, AGM; Burton, MJ	2016
Single-dose intravesical chemotherapy after nephroureterectomy for upper tract urothelial carcinoma	Hwang, , EC, Sathianathen, NJ, Jung, JH, Kim, MH, Dahm, P; Risk, MC	2019
Six months therapy for tuberculous meningitis	Jullien, , S, Ryan, H, Modi, M; Bhatia, R	2016
School feeding for improving the physical and psychosocial health of disadvantaged students	Kristjansson, , B, Petticrew, M, MacDonald, B, Krasevec, J, Janzen, L, Greenhalgh, T, Wells, GA, MacGowan, J, Farmer, AP, Shea, B, Mayhew, A, Tugwell, P; Welch, V	2007
Single dose oral acemetacin for acute postoperative pain in adults	Moore, , RA, Derry, S; McQuay, HJ	2009
Sliding scale insulin for non-critically ill hospitalised adults with diabetes mellitus	Colunga-Lozano, , LE, Gonzalez Torres, FJ, Delgado-Figueroa, N, Gonzalez-Padilla, DA, Hernandez, AV, Roman, Y; Cuello-García, CA	2018
Short-acting insulin analogues versus regular human insulin for adults with type 1 diabetes mellitus	Fullerton, , B, Siebenhofer, A, Jeitler, K, Horvath, K, Semlitsch, T, Berghold, A, Plank, J, Pieber, TR; Gerlach, FM	2016
Short-acting beta2-agonists for stable chronic obstructive pulmonary disease	Sestini, , P, Renzoni, E, Robinson, S, Poole, P; Ram, FSF	2002
Single dose oral lumiracoxib for postoperative pain in adults	Roy, , YM, Derry, S; Moore, RA	2010
Stapler versus scalpel resection followed by hand-sewn closure of the pancreatic remnant for distal pancreatectomy	Probst, , P, Hüttner, FJ, Klaiber, U, Knebel, P, Ulrich, A, Büchler, MW; Diener, MK	2015
Selective noradrenaline reuptake inhibitors for schizophrenia	Matthews, , PRL, Horder, J; Pearce, M	2018
Speech and language therapy versus placebo or no intervention for speech problems in Parkinson's disease	Herd, , CP, Tomlinson, CL, Deane, KHO, Brady, MC, Smith, CH, Sackley, CM; Clarke, CE	2012
Silver based wound dressings and topical agents for treating diabetic foot ulcers	Bergin, , S; Wraight, P	2006
Short-term low-dose corticosteroids vs placebo and nonsteroidal antiinflammatory drugs in rheumatoid arthritis	Gøtzsche, , PC; Johansen, HK	2005
Self-management for bronchiectasis	Kelly, , C, Grundy, S, Lynes, D, Evans, DJW, Gudur, S, Milan, SJ; Spencer, S	2018
Screening for genital chlamydia infection	Low, , N, Redmond, S, Uusküla, A, van Bergen, J, Ward, H, Andersen, B; Götz, H	2016
Scapular fixation in muscular dystrophy	Orrell, , RW, Copeland, S; Rose, MR	2010
Skin preparation with alcohol versus alcohol followed by any antiseptic for preventing bacteraemia or contamination of blood for transfusion	Webster, , J, Bell-Syer, SEM; Foxlee, R	2015
Stapled versus handsewn methods for colorectal anastomosis surgery	Neutzling, , CB, Lustosa, SAS, Proenca, IM, da Silva, EMK; Matos, D	2012
Single dose oral aceclofenac for postoperative pain in adults	Moore, , RA, Derry, S; McQuay, HJ	2009
Statins for acute coronary syndrome	Vale, , N, Nordmann, AJ, Schwartz, GG, de Lemos, J, Colivicchi, F, den Hartog, F, Ostadal, P, Macin, SM, Liem, AH, Mills, EJ, Bhatnagar, N, Bucher, HC; Briel, M	2014
Self-management interventions including action plans for exacerbations versus usual care in patients with chronic obstructive pulmonary disease	Lenferink, , A, Brusse-Keizer, M, van der Valk, PDLPM, Frith, PA, Zwerink, M, Monninkhof, EM, van der Palen, J; Effing, TW	2017
Selective serotonin reuptake inhibitors (SSRIs) and serotonin-norepinephrine reuptake inhibitors (SNRIs) for the prevention of tension-type headache in adults	Banzi, , R, Cusi, C, Randazzo, C, Sterzi, R, Tedesco, D; Moja, L	2015
Skin grafting for venous leg ulcers	Jones, , JE, Nelson, EA; Al-Hity, A	2013
Screening for gestational diabetes mellitus based on different risk profiles and settings for improving maternal and infant health	Tieu, , J, McPhee, AJ, Crowther, CA, Middleton, P; Shepherd, E	2017
Screening for prostate cancer	Ilic, , D, Neuberger, MM, Djulbegovic, M; Dahm, P	2013
Screening for peripheral arterial disease	Andras, , A; Ferket, B	2014
Silodosin for the treatment of lower urinary tract symptoms in men with benign prostatic hyperplasia	Jung, , JH, Kim, J, MacDonald, R, Reddy, B, Kim, MH; Dahm, P	2017
Screening for breast cancer with mammography	Gøtzsche, , PC; Jørgensen, KJ	2013
School-based education programmes for the prevention of child sexual abuse	Walsh, , K, Zwi, K, Woolfenden, S; Shlonsky, A	2015
Sequencing of chemotherapy and radiotherapy for early breast cancer	Hickey, , BE, Francis, DP; Lehman, M	2013
Splints and Orthosis for treating rheumatoid arthritis	Egan, , M, Brosseau, L, Farmer, M, Ouimet, MA, Rees, S, Tugwell, P; Wells, GA	2001
Splinting for carpal tunnel syndrome	Page, , MJ, Massy-Westropp, N, O'Connor, D; Pitt, V	2012
Speed cameras for the prevention of road traffic injuries and deaths	Wilson, , C, Willis, C, Hendrikz, JK, Le Brocque, R; Bellamy, N	2010

Service organisation for the secondary prevention of ischaemic heart disease in primary care	Buckley, , BS, Byrne, MC; Smith, SM	2010
Statin withdrawal in people with dementia	McGuinness, , B, Cardwell, CR; Passmore, P	2016
Screening programmes for the early detection and prevention of oral cancer	Brocklehurst, , P, Kujan, O, O'Malley, LA, Ogden, G, Shepherd, S; Glenny, AM	2013
Spermicide used alone for contraception	Grimes, , DA, Lopez, LM, Raymond, EG, Halpern, V, Nanda, K; Schulz, KF	2013
Short courses of antibiotics for children and adults with bronchiectasis	Wurzel, , D, Marchant, JM, Yerkovich, ST, Upham, JW, Masters, IB; Chang, AB	2011
Screening with urinary dipsticks for reducing morbidity and mortality	Krogsbøll, , LT, Jørgensen, KJ; Gøtzsche, PC	2015
Single dose oral naproxen and naproxen sodium for acute postoperative pain in adults	Derry, , CJ, Derry, S, Moore, RA; McQuay, HJ	2009
Screening for abdominal aortic aneurysm	Cosford, , PA, Leng, GC; Thomas, J	2007
Single dose oral indometacin for the treatment of acute postoperative pain	Moore, , RA, Derry, S, Mason, L, McQuay, HJ; Edwards, J	2004
Single dose oral meloxicam for acute postoperative pain in adults	Moore, , RA, Derry, S; McQuay, HJ	2009
Single dose oral ibuprofen plus codeine for acute postoperative pain in adults	Derry, , S, Karlin, SM; Moore, RA	2015
Slow-release oral morphine as maintenance therapy for opioid dependence	Ferri, , M, Minozzi, S, Bo, A; Amato, L	2013
Short acting insulin analogues versus regular human insulin in patients with diabetes mellitus	Siebenhofer, , A, Plank, J, Berghold, A, Jeitler, K, Horvath, K, Narath, M, Gfrerer, R; Pieber, TR	2006
Short-term ambulatory oxygen for chronic obstructive pulmonary disease	Bradley, , JM; O'Neill, BM	2005
Specially formulated foods for treating children with moderate acute malnutrition in low- and middle-income countries	Lazzerini, , M, Rubert, L; Pani, P	2013
Stapled versus handsewn methods for ileocolic anastomoses	Choy, , PYG, Bissett, IP, Docherty, JG, Parry, BR, Merrie, A; Fitzgerald, A	2011
Sedation of children undergoing dental treatment	Ashley, , PF, Chaudhary, M; Lourenço-Matharu, L	2018
Single dose oral nefopam for acute postoperative pain in adults	Kakkar, , M, Derry, S, Moore, RA; McQuay, HJ	2009
Single dose oral dihydrocodeine for acute postoperative pain	Moore, , RA, Edwards, J, Derry, S; McQuay, HJ	2000
Sequencing of anthracyclines and taxanes in neoadjuvant and adjuvant therapy for early breast cancer	Zaheed, , M, Wilcken, N, Willson, ML, O'Connell, DL; Goodwin, A	2019
Sealing procedures for preterm prelabour rupture of membranes	Crowley, , AE, Grivell, RM; Dodd, JM	2016
Sargramostim (GM-CSF) for induction of remission in Crohn's disease	Roth, , L, MacDonald, JK, McDonald, JWD; Chande, N	2011
Staff-led interventions for improving oral hygiene in patients following stroke	Brady, , MC, Furlanetto, D, Hunter, R, Lewis, SC; Milne, V	2006
Skin preparation for preventing infection following caesarean section	Hadiati, , DR, Hakimi, M, Nurdiati, DS, da Silva Lopes, K; Ota, E	2018
Saline irrigation for the management of skin extravasation injury in neonates	Gopalakrishnan, , PN, Goel, N; Banerjee, S	2017
Selective estrogen receptor modulators (SERMs) for uterine leiomyomas	Deng, , L, Wu, T, Chen, XY, Xie, L; Yang, J	2012
Single dose oral oxycodone and oxycodone plus paracetamol (acetaminophen) for acute postoperative pain in adults	Gaskell, , H, Derry, S, Moore, RA; McQuay, HJ	2009
Single dose oral piroxicam for acute postoperative pain	Moore, , RA, Edwards, J, Loke, YKK, Derry, S; McQuay, HJ	2000
Single dose oral lornoxicam for acute postoperative pain in adults	Hall, , PE, Derry, S, Moore, RA; McQuay, HJ	2009
Single dose oral ibuprofen plus caffeine for acute postoperative pain in adults	Derry, , S, Wiffen, PJ; Moore, RA	2015
Semi-recumbent position versus supine position for the prevention of ventilator-associated pneumonia in adults requiring mechanical ventilation	Wang, , L, Li, X, Yang, Z, Tang, X, Yuan, Q, Deng, L; Sun, X	2016
Single incision versus conventional multi-incision appendicectomy for suspected appendicitis	Rehman, , H, Rao, AM; Ahmed, I	2011
Singing for children and adults with bronchiectasis	Irons, , JY, Kenny, DT; Chang, AB	2010
Single dose oral rofecoxib for acute postoperative pain in adults	Bulley, , S, Derry, S, Moore, RA; McQuay, HJ	2009
Singing as an adjunct therapy for children and adults with cystic fibrosis	Irons, , JY, Petocz, P, Kenny, DT; Chang, AB	2019
Slum upgrading strategies involving physical environment and infrastructure interventions and their effects on health and socio-economic outcomes	Turley, , R, Saith, R, Bhan, N, Rehfuess, E; Carter, B	2013
Sclerotherapy for lower limb telangiectasias	Schwartz, , L; Maxwell, H	2011
Short-course versus long-course therapy of the same antibiotic for community-acquired pneumonia in adolescent and adult outpatients	López-Alcalde, , J, Rodriguez-Barrientos, R, Redondo-Sánchez, J, Muñoz-Gutiérrez, J, Molero García, JM, Rodríguez-Fernández, C, Heras-Mosteiro, J, Marin-Cañada, J, Casanova-Colominas, J, Azcoaga-Lorenzo, A, Hernandez Santiago, V; Gómez-García, M	2018

Spectacle correction versus no spectacles for prevention of strabismus in hyperopic children	Jones-Jordan, , L, Wang, X, Scherer, RW; Mutti, DO	2014
Smartphone and tablet self management apps for asthma	Marcano Belisario, , JS, Huckvale, K, Greenfield, G, Car, J; Gunn, LH	2013
Screening and subsequent management for thyroid dysfunction pre-pregnancy and during pregnancy for improving maternal and infant health	Spencer, , L, Bubner, T, Bain, E; Middleton, P	2015
Smoking cessation interventions for pulmonary tuberculosis treatment outcomes	Jeyashree, , K, Kathirvel, S, Shewade, HD, Kaur, H; Goel, S	2016
Smectite for acute infectious diarrhoea in children	Pérez-Gaxiola, , G, Cuello-García, CA, Florez, ID; Pérez-Pico, VM	2018
Sapropterin dihydrochloride for phenylketonuria	Somaraju, , UR; Merrin, M	2015
Second trimester serum tests for Down's Syndrome screening	Alldred, , SK, Deeks, JJ, Guo, B, Neilson, JP; Alfirevic, Z	2012
Selective serotonin reuptake inhibitors (SSRIs) and serotonin-norepinephrine reuptake inhibitors (SNRIs) for the prevention of migraine in adults	Banzi, , R, Cusi, C, Randazzo, C, Sterzi, R, Tedesco, D; Moja, L	2015
Single dose oral flurbiprofen for acute postoperative pain in adults	Sultan, , A, McQuay, HJ, Moore, RA; Derry, S	2009
Single dose oral etodolac for acute postoperative pain in adults	Tirunagari, , SK, Derry, S, Moore, RA; McQuay, HJ	2009
Screening for nasopharyngeal cancer	Yang, , S, Wu, S, Zhou, J; Chen, XY	2015
Selenium supplementation for Hashimoto's thyroiditis	van Zuuren, , EJ, Albusta, AY, Fedorowicz, Z, Carter, B; Pijl, H	2013
Short-acting insulin analogues versus regular human insulin for adult, non-pregnant persons with type 2 diabetes mellitus	Fullerton, , B, Siebenhofer, A, Jeitler, K, Horvath, K, Semlitsch, T, Berghold, A; Gerlach, FM	2018
School-based education programmes for the prevention of unintentional injuries in children and young people	Orton, , E, Whitehead, J, Mhizha-Murira, J, Clarkson, M, Watson, MC, Mulvaney, CA, Staniforth, JUL, Bhuchar, M; Kendrick, D	2016
Six-month therapy for abdominal tuberculosis	Jullien, , S, Jain, S, Ryan, H; Ahuja, V	2016
Specialist teams for neonatal transport to neonatal intensive care units for prevention of morbidity and mortality	Chang, , ASM, Berry, A, Jones, LJ; Sivasangari, S	2015
Social skills training for attention deficit hyperactivity disorder (ADHD) in children aged 5 to 18 years	Storebø, , OJ, Elmose Andersen, M, Skoog, M, Joost Hansen, S, Simonsen, E, Pedersen, N, Tendal, B, Callesen, HE, Faltinsen, E; Gluud, C	2019
Smoking cessation intervention for reducing disease activity in chronic autoimmune inflammatory joint diseases	Roelsgaard, , IK, Esbensen, BA, Østergaard, M, Rollefstad, S, Semb, AG, Christensen, R; Thomsen, T	2019
Screening for reducing morbidity and mortality in malignant melanoma	Johansson, , M, Brodersen, J, Gøtzsche, PC; Jørgensen, KJ	2019
School-based self-management interventions for asthma in children and adolescents: a mixed methods systematic review	Harris, , K, Kneale, D, Lasserson, TJ, McDonald, VM, Grigg, J; Thomas, J	2019
Smartphone applications for triaging adults with skin lesions that are suspicious for melanoma	Chuchu, , N, Takwoingi, Y, Dinnes, J, Matin, RN, Bassett, O, Moreau, JF, Bayliss, SE, Davenport, C, Godfrey, K, O'Connell, S, Jain, A, Walter, FM, Deeks, JJ; Williams, HC	2018
Second-generation antidepressants for preventing seasonal affective disorder in adults	Gartlehner, , G, Nussbaumer-Streit, B, Gaynes, BN, Forneris, CA, Morgan, LC, Greenblatt, A, Wipplinger, J, Lux, LJ, Van Noord, MG; Winkler, D	2019
School dental screening programmes for oral health	Arora, , A, Khattri, S, Ismail, NM, Kumbargere Nagraj, S; Eachempati, P	2019
Transcutaneous electrostimulation for osteoarthritis of the knee	Rutjes, , AWS, Nüesch, E, Sterchi, R, Kalichman, L, Hendriks, E, Osiri, M, Brosseau, L, Reichenbach, S; Jüni, P	2009
Transcutaneous carbon dioxide monitoring for the prevention of neonatal morbidity and mortality	Bruschettini, , M, Romantsik, O, Zappettini, S, Ramenghi, LA; Calevo, MG	2016
Therapeutic drug monitoring of antiretrovirals for people with HIV	Kredo, , T, Van der Walt, JS, Siegfried, N; Cohen, K	2009
Topical treatments for HIV-related oral ulcers	Kuteyi, , T; Okwundu, CI	2012
Total versus subtotal hysterectomy for benign gynaecological conditions	Lethaby, , A, Mukhopadhyay, A; Naik, R	2012
Thromboelastography (TEG) or thromboelastometry (ROTEM) to monitor haemostatic treatment versus usual care in adults or children with bleeding	Wikkelsø, , A, Wetterslev, J, Møller, AM; Afshari, A	2016
Transcranial direct current stimulation (tDCS) for improving aphasia in adults with aphasia after stroke	Elsner, , B, Kugler, J, Pohl, M; Mehrholz, J	2019
Telehealthcare for chronic obstructive pulmonary disease	McLean, , S, Nurmatov, U, Liu, JLY, Pagliari, C, Car, J; Sheikh, A	2011
Telehealthcare for asthma	McLean, , S, Chandler, D, Nurmatov, U, Liu, JLY, Pagliari, C, Car, J; Sheikh, A	2010
Tocilizumab for rheumatoid arthritis	Singh, , JA, Beg, S; Lopez-Olivo, MA	2010
Thrombolysis for cerebral vein and dural sinus thrombosis	Ciccone, , A, Canhão, P, Falcão, F, Ferro, JM; Sterzi, R	2004
The effect of inotropes on morbidity and mortality in preterm infants with low systemic or organ blood flow	Osborn, , DA, Paradisis, M; Evans, NJ	2007
Tight control of mild-moderate pre-existing or non-proteinuric gestational hypertension	Nabhan, , AF; Elsedawy, MM	2011
Topiramate for the prophylaxis of episodic migraine in adults	Linde, , M, Mulleners, WM, Chronicle, EP; McCrory, DC	2013

Transcutaneous electrical nerve stimulation (TENS) for the treatment of rheumatoid arthritis in the hand	Brosseau, , L, Yonge, KA, Welch, V, Marchand, S, Judd, M, Wells, GA; Tugwell, P	2003
Totally implantable vascular access devices for cystic fibrosis	A-Rahman, , AKM; Spencer, D	2012
Transdermal fentanyl for cancer pain	Hadley, , G, Derry, S, Moore, RA; Wiffen, PJ	2013
The effect of pharmacist-provided non-dispensing services on patient outcomes, health service utilisation and costs in low- and middle-income countries	Pande, , S, Hiller, JE, Nkansah, N; Bero, L	2013
Topiramate for neuropathic pain and fibromyalgia in adults	Wiffen, , PJ, Derry, S, Lunn, MPT; Moore, RA	2013
Topotecan for ovarian cancer	Lihua, , P, Chen, XY; Wu, T	2008
Transfusion of red blood cells stored for shorter versus longer duration for all conditions	Shah, , A, Brunskill, SJ, Desborough, MJR, Doree, C, Trivella, M; Stanworth, SJ	2018
The role of high-frequency oscillations in epilepsy surgery planning	Gloss, , D, Nevitt, SJ; Staba, R	2017
Tobacco cessation interventions for young people	Fanshawe, , TR, Halliwell, W, Lindson, N, Aveyard, P, Livingstone-Banks, J; Hartmann-Boyce, J	2017
Topically applied anaesthetics for treating perineal pain after childbirth	Hedayati, , H, Parsons, J; Crowther, CA	2005
The Epley (canalith repositioning) manoeuvre for benign paroxysmal positional vertigo	Hilton, , MP; Pinder, DK	2014
Tolerability of selective cyclooxygenase 2 inhibitors used for the treatment of rheumatological manifestations of inflammatory bowel disease	Miao, , XP, Li, JS, Ouyang, Q, Hu, RW, Zhang, Y; Li, HY	2014
Thrombolysis (different doses, routes of administration and agents) for acute ischaemic stroke	Wardlaw, , JM, Koumellis, P; Liu, M	2013
Therapeutic ultrasound for osteoarthritis of the knee or hip	Rutjes, , AWS, Nüesch, E, Sterchi, R; Jüni, P	2010
Third generation cephalosporins versus conventional antibiotics for treating acute bacterial meningitis	Prasad, , K, Kumar, A, Singhal, T; Gupta, PK	2007
Topiramate for acute affective episodes in bipolar disorder in adults	Pigott, , K, Galizia, I, Vasudev, K, Watson, S, Geddes, J; Young, AH	2016
Tenofovir or zidovudine in three-drug combination therapy with one nucleoside reverse transcriptase inhibitor and one non-nucleoside reverse transcriptase inhibitor for initial treatment of HIV infection in antiretroviral-naïve individuals	Spaulding, , A, Rutherford, GW; Siegfried, N	2010
Tonsillectomy or adenotonsillectomy versus non-surgical treatment for chronic/recurrent acute tonsillitis	Burton, , MJ, Glasziou, PP, Chong, LY; Venekamp, RP	2014
Taxane-containing regimens for metastatic breast cancer	Ghersi, , D, Willson, ML, Chan, MMK, Simes, J, Donoghue, E; Wilcken, N	2015
Topical pimecrolimus for eczema	Ashcroft, , DM, Chen, LC, Garside, R, Stein, K; Williams, HC	2007
Telephone consultation and triage: effects on health care use and patient satisfaction	Bunn, , F, Byrne, G; Kendall, S	2004
Telephone delivered interventions for reducing morbidity and mortality in people with HIV infection	Gentry, , S, van-Velthoven, MHMMT, Tudor Car, L; Car, J	2013
The role of additional radiotherapy for primary central nervous system lymphoma	Zacher, , J, Kasenda, B, Engert, A; Skoetz, N	2014
Tramadol with or without paracetamol (acetaminophen) for cancer pain	Wiffen, , PJ, Derry, S; Moore, RA	2017
Total or near-total thyroidectomy versus subtotal thyroidectomy for multinodular non-toxic goitre in adults	Cirocchi, , R, Trastulli, S, Randolph, J, Guarino, S, Di Rocco, G, Arezzo, A, D'Andrea, V, Santoro, A, Barczyñski, M; Avenia, N	2015
Techniques for liver parenchymal transection in liver resection	Gurusamy, , KS, Pamecha, V, Sharma, D; Davidson, BR	2009
Thalidomide for managing cancer cachexia	Reid, , J, Mills, M, Cantwell, MM, Cardwell, CR, Murray, LJ; Donnelly, M	2012
Transcranial magnetic stimulation for the treatment of epilepsy	Chen, , R, Spencer, DC, Weston, J; Nolan, SJ	2016
Tracheal intubation with a flexible intubation scope versus other intubation techniques for obese patients requiring general anaesthesia	Nicholson, , A, Smith, AF, Lewis, SR; Cook, TM	2014
The effects of idarubicin versus other anthracyclines for induction therapy of patients with newly diagnosed leukaemia	Li, , X, Xu, S, Tan, Y; Chen, J	2015
Topical silver for preventing wound infection	Storm-Versloot, , MN, Vos, CG, Ubbink, DT; Vermeulen, H	2010
The impact of user fees on access to health services in low- and middle-income countries	Lagarde, , M; Palmer, N	2011
Thymectomy for non-thymomatous myasthenia gravis	Cea, , G, Benatar, M, Verdugo, RJ; Salinas, RA	2013
Thyrotropin-releasing hormone added to corticosteroids for women at risk of preterm birth for preventing neonatal respiratory disease	Crowther, , CA, Alfirevic, Z, Han, S; Haslam, RR	2013
Time-limited home-care reablement services for maintaining and improving the functional independence of older adults	Cochrane, , A, Furlong, M, McGilloway, S, Molloy, DW, Stevenson, M; Donnelly, M	2016
Therapeutic monitoring of antiepileptic drugs for epilepsy	Tomson, , T, Dahl, ML; Kimland, E	2007
Theory-based interventions for contraception	Lopez, , LM, Grey, TW, Chen, M, Tolley, EE; Stockton, LL	2016
Transcutaneous electrostimulation for suspected placental insufficiency (diagnosed by Doppler studies)	Say, , L, Gülmezoglu, AM; Hofmeyr, GJ	1996

Tests for detecting strabismus in children aged 1 to 6 years in the community	Hull, , S, Tailor, V, Balduzzi, S, Rahi, J, Schmucker, C, Virgili, G; Dahlmann-Noor, A	2017
Tiotropium versus long-acting beta-agonists for stable chronic obstructive pulmonary disease	Chong, , J, Karner, C; Poole, P	2012
Techniques of monitoring blood glucose during pregnancy for women with pre-existing diabetes	Jones, , LV, Ray, A, Moy, FM; Buckley, BS	2019
The impact of surgical therapies for inflammatory bowel disease on female fertility	Lee, , S, Crowe, M, Seow, CH, Kotze, PG, Kaplan, GG, Metcalfe, A, Ricciuto, A, Benchimol, EI; Kuenzig, ME	2019
Tramadol for osteoarthritis	Toupin April, , K, Bisaillon, J, Welch, V, Maxwell, LJ, Jüni, P, Rutjes, AWS, Husni, ME, Vincent, J, El Hindi, T, Wells, GA; Tugwell, P	2019
Topical agents and dressings for fungating wounds	Adderley, , UJ; Holt, IGS	2014
Teicoplanin versus vancomycin for proven or suspected infection	Cavalcanti, , AB, Goncalves, AR, Almeida, CS, Bugano, DDG; Silva, E	2010
Training health professionals in smoking cessation	Carson, , KV, Verbiest, MEA, Crone, MR, Brinn, MP, Esterman, AJ, Assendelft, WJJ; Smith, BJ	2012
Topical corticosteroids for treating phimosis in boys	Moreno, , G, Corbalán, J, Peñaloza, B; Pantoja, T	2014
The effects of anaesthetic agents on cortical mapping during neurosurgical procedures involving eloquent areas of the brain	Adhikary, , SD, Thiruvenkatarajan, V, Babu, KS; Tharyan, P	2011
Transarterial (chemo)embolisation for unresectable hepatocellular carcinoma	Oliveri, , RS, Wetterslev, J; Gluud, C	2011
Topical agents or dressings for pain in venous leg ulcers	Briggs, , M, Nelson, EA; Martyn-St James, M	2012
Tidal versus other forms of peritoneal dialysis for acute kidney injury	Jiang, , L, Zeng, R, Yang, K, Mi, DH, Tian, JH, Ma, B; Liu, Y	2012
Telephone communication of HIV testing results for improving knowledge of HIV infection status	Tudor Car, , L, Gentry, S, van-Velthoven, MHMMT; Car, J	2013
Therapeutic ultrasound for venous leg ulcers	Cullum, , N; Liu, Z	2017
Thioridazine for dementia	Kirchner, , V, Kelly, CA; Harvey, RJ	2001
Therapeutic ultrasound for chronic low-back pain	Ebadi, , S, Henschke, N, Nakhostin Ansari, N, Fallah, E; van Tulder, MW	2014
The effect of adding inhaled corticosteroids to tiotropium and long-acting beta2-agonists for chronic obstructive pulmonary disease	Karner, , C; Cates, CJ	2011
The effects of antimicrobial therapy on bacterial vaginosis in non-pregnant women	Oduyebo, , OO, Anorlu, RI; Ogunsola, FT	2009
Telephone delivered interventions for preventing HIV infection in HIV-negative persons	van-Velthoven, , MHMMT, Tudor Car, L, Gentry, S; Car, J	2013
Timing and volume of fluid administration for patients with bleeding	Kwan, , I, Bunn, F, Chinnock, P; Roberts, I	2014
The WHO Health Promoting School framework for improving the health and well-being of students and their academic achievement	Langford, , R, Bonell, CP, Jones, HE, Pouliou, T, Murphy, SM, Waters, E, Komro, KA, Gibbs, LF, Magnus, D; Campbell, R	2014
Topical anti-inflammatory agents for seborrhoeic dermatitis of the face or scalp	Kastarinen, , H, Oksanen, T, Okokon, EO, Kiviniemi, VV, Airola, K, Jyrkkä, J, Oravilahti, T, Rannanheimo, PK; Verbeek, JH	2014
Tools developed and disseminated by guideline producers to promote the uptake of their guidelines	Flodgren, , G, Hall, AM, Goulding, L, Eccles, MP, Grimshaw, JM, Leng, GC; Shepperd, S	2016
Therapeutic interventions for disease progression in Huntington's disease	Mestre, , T, Ferreira, J, Coelho, MM, Rosa, M; Sampaio, C	2009
Techniques for pelvic surgery in subfertility	Ahmad, , G, Watson, A, Vanderkerchove, P; Lilford, R	2006
Topical antihistamines and mast cell stabilisers for treating seasonal and perennial allergic conjunctivitis	Castillo, , M, Scott, NW, Mustafa, MZ, Mustafa, MS; Azuara-Blanco, A	2015
Frailty and the biochemical effects of recombinant human growth hormone in women after surgery for hip fracture	Herling, , SF, Dreijer, B, Wrist Lam, G, Thomsen, T; Møller, AM	2017
Therapeutic interventions for symptomatic treatment in Huntington's disease	Mestre, , T, Ferreira, J, Coelho, MM, Rosa, M; Sampaio, C	2009
Topical cyclosporine for atopic keratoconjunctivitis	González-López, , JJ, López-Alcalde, J, Morcillo Laiz, R, Fernández Buenaga, R; Rebolleda Fernández, G	2012
Therapies for depression in Parkinson's disease	Ghazi-Noori, , S, Chung, TH, Deane, K, Rickards, HE; Clarke, CE	2003
Techniques of flushing and reperfusion for liver transplantation	Gurusamy, , KS, Naik, P, Abu-Amara, M, Fuller, B; Davidson, BR	2012
Tramadol for neuropathic pain in adults	Duehmke, , RM, Derry, S, Wiffen, PJ, Bell, RF, Aldington, D; Moore, RA	2017
Topical antifungals for seborrhoeic dermatitis	Okokon, , EO, Verbeek, JH, Ruotsalainen, JH, Ojo, OA; Bakhoya, VN	2015
Topical herbal therapies for treating osteoarthritis	Cameron, , M; Chrubasik, S	2013
Transcatheter arterial chemoembolisation followed by three-dimensional conformal radiotherapy versus transcatheter arterial chemoembolisation alone for primary hepatocellular carcinoma in adults	Lu, , L, Zeng, J, Wen, Z, Tang, C; Xu, N	2019
Topical glyceryl trinitrate for rotator cuff disease	Cumpston, , M, Johnston, RV, Wengier, L; Buchbinder, R	2009
Topical interventions for genital lichen sclerosus	Chi, , CC, Kirtschig, G, Baldo, M, Brackenbury, F, Lewis, F; Wojnarowska, F	2011
Thrombopoietin receptor agonists for prevention and treatment of chemotherapy-induced thrombocytopenia in patients with solid tumours	Zhang, , X, Chuai, Y, Nie, W, Wang, A; Dai, G	2017

Techniques of intrauterine fetal transfusion for women with red-cell isoimmunisation for improving health outcomes	Dodd, , JM, Windrim, RC; van Kamp, IL	2012
Topical microbicides for prevention of sexually transmitted infections	Obiero, , J, Mwethera, PG; Wiysonge, CS	2012
Topical antibiotics for preventing surgical site infection in wounds healing by primary intention	Heal, , CF, Banks, JL, Lepper, PD, Kontopantelis, E; van Driel, ML	2016
Transarterial (chemo)embolisation versus no intervention or placebo intervention for liver metastases	Riemsma, , RP, Bala, MM, Wolff, R; Kleijnen, J	2013
Tianma Gouteng Yin Formula for treating primary hypertension	Zhang, , HW, Tong, J, Zhou, G, Jia, H; Jiang, JY	2012
Tongxinluo (Tong xin luo or Tong-xin-luo) capsule for unstable angina pectoris	Wu, , T, Harrison, RA, Chen, XY, Ni, J, Zhou, L, Qiao, J, Wang, Q, Wei, J, Duan, X; Zheng, J	2006
Timed voiding for the management of urinary incontinence in adults	Ostaszkiewicz, , J, Johnston, L; Roe, B	2004
Thrombopoietin mimetics for patients with myelodysplastic syndromes	Dodillet, , H, Kreuzer, KA, Monsef, I; Skoetz, N	2017
Telerehabilitation for persons with multiple sclerosis	Khan, , F, Amatya, B, Kesselring, J; Galea, M	2015
Thioridazine for schizophrenia	Fenton, , M, Rathbone, J; Reilly, J	2007
Telephone counselling for smoking cessation	Matkin, , W, Ordóñez-Mena, JM; Hartmann-Boyce, J	2019
Telephone interventions, delivered by healthcare professionals, for providing education and psychosocial support for informal caregivers of adults with diagnosed illnesses	Corry, , M, Neenan, K, Brabyn, S, Sheaf, G; Smith, V	2019
Topiramate versus carbamazepine monotherapy for epilepsy: an individual participant data review	Nevitt, , SJ, Sudell, M, Tudur Smith, C; Marson, AG	2019
Training and supportive programs for palliative care volunteers in community settings	Horey, , D, Street, AF, O'Connor, M, Peters, L; Lee, SF	2015
The effect of social franchising on access to and quality of health services in low- and middle-income countries	Koehlmoos, , TP, Gazi, R, Hossain, SS; Zaman, K	2009
Timing of renal replacement therapy initiation for acute kidney injury	Fayad, , AII, Buamscha, DG; Ciapponi, A	2018
Tracheal gas insufflation for the prevention of morbidity and mortality in mechanically ventilated newborn infants	Davies, , MW; Woodgate, PG	2002
Topical non-steroidal anti-inflammatory agents for diabetic cystoid macular oedema	Sahoo, , S, Barua, A, Myint, KT, Haq, A, Abas, ABL; Nair, NS	2015
Training interventions for improving telephone consultation skills in clinicians	Vaona, , A, Pappas, Y, Grewal, RS, Ajaz, M, Majeed, A; Car, J	2017
Topical silver for treating infected wounds	Vermeulen, , H, van Hattem, JM, Storm-Versloot, MN, Ubbink, DT; Westerbos, SJ	2007
Tirilazad for aneurysmal subarachnoid haemorrhage	Zhang, , S, Wang, L, Liu, M; Wu, B	2010
Tinnitus Retraining Therapy (TRT) for tinnitus	Phillips, , JS; McFerran, D	2010
Thrombolysis for acute ischaemic stroke	Wardlaw, , JM, Murray, V, Berge, E; del Zoppo, GJ	2014
Traditional Chinese Medicine herbs for stopping bleeding from haemorrhoids	Gan, , T, Liu, YD, Wang, Y; Yang, J	2010
Total serum bile acids or serum bile acid profile, or both, for the diagnosis of intrahepatic cholestasis of pregnancy	Manzotti, , C, Casazza, G, Stimac, T, Nikolova, D; Gluud, C	2019
Tong-xin-luo capsule for patients with coronary heart disease after percutaneous coronary intervention	Mao, , C, Fu, XH, Yuan, JQ, Yang, ZY, Chung, VCH, Qin, Y, Huang, Y, Tam, WWS, Kwong, JSW, Xie, W; Tang, JL	2015
Thienopyridine derivatives versus aspirin for preventing stroke and other serious vascular events in high vascular risk patients	Sudlow, , CLM, Mason, G, Maurice, JB, Wedderburn, CJ; Hankey, GJ	2009
Therapy-based rehabilitation services for stroke patients at home	Trialists, Outpatient Service	2003
Therapist-supported Internet cognitive behavioural therapy for anxiety disorders in adults	Olthuis, , JV, Watt, MC, Bailey, K, Hayden, JA; Stewart, SH	2016
Thyroxine replacement for subfertile women with euthyroid autoimmune thyroid disease or subclinical hypothyroidism	Akhtar, , MA, Agrawal, R, Brown, J, Sajjad, Y; Craciunas, L	2019
The medical use of cannabis for reducing morbidity and mortality in patients with HIV/AIDS	Lutge, , EE, Gray, A; Siegfried, N	2013
Topical anaesthetic or vasoconstrictor preparations for flexible fibre-optic nasal pharyngoscopy and laryngoscopy	Sunkaraneni, , VS; Jones, SEM	2011
Tonsillectomy or adenotonsillectomy versus non-surgical management for obstructive sleep-disordered breathing in children	Venekamp, , RP, Hearne, BJ, Chandrasekharan, D, Blackshaw, H, Lim, J; Schilder, AGM	2015
Therapeutic interventions for Burkitt lymphoma in children	Okebe, , JU, Skoetz, N, Meremikwu, MM; Richards, S	2011
Tranexamic acid for patients with nasal haemorrhage (epistaxis)	Joseph, , J, Martinez-Devesa, P, Bellorini, J; Burton, MJ	2018
Terlipressin versus placebo or no intervention for people with cirrhosis and hepatorenal syndrome	Allegretti, , AS, Israelsen, M, Krag, A, Jovani, M, Goldin, AH, Schulman, AR, Winter, RW; Gluud, LL	2017
Tonsillectomy for periodic fever, aphthous stomatitis, pharyngitis and cervical adenitis syndrome (PFAPA)	Burton, , MJ, Pollard, AJ, Ramsden, JD, Chong, LY; Venekamp, RP	2014
Topical antifungal treatments for tinea cruris and tinea corporis	El-Gohary, , M, van Zuuren, EJ, Fedorowicz, Z, Burgess, H, Doney, L, Stuart, B, Moore, M; Little, P	2014
Thrombolytic therapy for pulmonary embolism	Hao, , Q, Dong, BR, Yue, J, Wu, T; Liu, GJ	2018
Therapeutic exercise for people with amyotrophic lateral sclerosis or motor neuron disease	Dal Bello-Haas, , V; Florence, JM	2013

Timing of surgery for aneurysmal subarachnoid haemorrhage	Whitfield, , PC; Kirkpatrick, P	2001
Topical lidocaine for neuropathic pain in adults	Derry, , S, Wiffen, PJ, Moore, RA; Quinlan, J	2014
Tiagabine in the maintenance treatment of bipolar disorder	Vasudev, , A, Macritchie, K, Rao, SNK, Geddes, J; Young, AH	2011
Topical anaesthesia alone versus topical anaesthesia with intracameral lidocaine for phacoemulsification	Ezra, , DG; Allan, BDS	2007
Training to recognise the early signs of recurrence in schizophrenia	Morriss, , R, Vinjamuri, I, Faizal, MA, Bolton, CA; McCarthy, JP	2013
The role of maintenance therapy in acute promyelocytic leukemia in the first complete remission	Muchtar, , E, Vidal, L, Ram, R, Gafter-Gvili, A, Shpilberg, O; Raanani, P	2013
Temozolomide for high grade glioma	Hart, , MG, Garside, R, Rogers, G, Stein, K; Grant, R	2013
Thrombolysis for acute deep vein thrombosis	Watson, , L, Broderick, C; Armon, MP	2016
Timing of dornase alfa inhalation for cystic fibrosis	Dentice, , R; Elkins, M	2018
Traditional birth attendant training for improving health behaviours and pregnancy outcomes	Sibley, , LM, Sipe, TA; Barry, D	2012
Thermotherapy for treating rheumatoid arthritis	Welch, , V, Brosseau, L, Casimiro, L, Judd, M, Shea, B, Tugwell, P; Wells, GA	2002
Topical antibiotics without steroids for chronically discharging ears with underlying eardrum perforations	Macfadyen, , CA, Acuin, JM; Gamble, CL	2005
Traditional Chinese herbal medicine for vascular dementia	Chan, , ESY, Bautista, DT, Zhu, Y, You, Y, Long, JT, Li, W; Chen, C	2018
Thermotherapy for treatment of osteoarthritis	Brosseau, , L, Yonge, KA, Welch, V, Marchand, S, Judd, M, Wells, GA; Tugwell, P	2003
Thoracic stent graft versus surgery for thoracic aneurysm	Abraha, , I, Romagnoli, C, Montedori, A; Cirocchi, R	2016
Topiramate for essential tremor	Bruno, , E, Nicoletti, A, Quattrocchi, G, Allegra, R, Filippini, G, Colosimo, C; Zappia, M	2017
Topical cystic fibrosis transmembrane conductance regulator gene replacement for cystic fibrosis-related lung disease	Perry, , LA, Penny-Dimri, JC, Aslam, AA, Lee, TWR; Southern, KW	2016
TNF-alpha inhibitors for ankylosing spondylitis	Maxwell, , LJ, Zochling, J, Boonen, A, Singh, JA, Veras, MMS, Tanjong Ghogomu, E, Benkhalti Jandu, M, Tugwell, P; Wells, GA	2015
The effect of financial incentives on the quality of health care provided by primary care physicians	Scott, , A, Sivey, P, Ait Ouakrim, D, Willenberg, L, Naccarella, L, Furler, J; Young, D	2011
Topiramate add-on for drug-resistant partial epilepsy	Pulman, , J, Jette, N, Dykeman, J, Hemming, K, Hutton, JL; Marson, AG	2014
Tiotropium versus ipratropium bromide for chronic obstructive pulmonary disease	Cheyne, , L, Irvin-Sellers, MJ; White, J	2015
Topical treatments for scalp psoriasis	Schlager, , JG, Rosumeck, S, Werner, RN, Jacobs, A, Schmitt, J, Schlager, C; Nast, A	2016
Tobacco packaging design for reducing tobacco use	McNeill, , A, Gravely, S, Hitchman, SC, Bauld, L, Hammond, D; Hartmann-Boyce, J	2017
Time course for blood pressure lowering of dihydropyridine calcium channel blockers	Ghamami, , N, Chiang, SHY, Dormuth, C; Wright, JM	2014
Totally percutaneous versus surgical cut-down femoral artery access for elective bifurcated abdominal endovascular aneurysm repair	Gimzewska, , M, Jackson, AIR, Yeoh, SE; Clarke, M	2017
Techniques for caesarean section	Hofmeyr, , GJ, Mathai, M, Shah, AN; Novikova, N	2008
Timing of breast surgery in premenopausal breast cancer patients	Samuel, , M, Khin, LW, Brennan, VK; Yong, WS	2011
Transabdominal amnioinfusion for improving fetal outcomes after oligohydramnios secondary to preterm prelabour rupture of membranes before 26 weeks	Van Teeffelen, , S, Pajkrt, E, Willekes, C, Van Kuijk, SMJ; Mol, BWJ	2013
Tiagabine for acute affective episodes in bipolar disorder	Vasudev, , A, Macritchie, K, Rao, SK, Geddes, J; Young, AH	2012
The impact of conditional cash transfers on health outcomes and use of health services in low and middle income countries	Lagarde, , M, Haines, A; Palmer, N	2009
Thyroid hormones for preventing neurodevelopmental impairment in preterm infants	Osborn, DA	2001
Thrombophilia testing for prevention of recurrent venous thromboembolism	Cohn, , DM, Vansenne, F, de Borgie, CA; Middeldorp, S	2012
Topical capsaicin (low concentration) for chronic neuropathic pain in adults	Derry, , S; Moore, RA	2012
The effect of different methods of remuneration on the behaviour of primary care dentists	Brocklehurst, , P, Price, J, Glenny, AM, Tickle, M, Birch, S, Mertz, E; Grytten, J	2013
Thiazide diuretics and the risk of hip fracture	Aung, , K; Htay, T	2011
The impact of biological interventions for ulcerative colitis on health-related quality of life	LeBlanc, , K, Mosli, MH, Parker, CE; MacDonald, JK	2015
Thalidomide and thalidomide analogues for induction of remission in Crohn's disease	Srinivasan, , R; Akobeng, AK	2009
Thalidomide and thalidomide analogues for maintenance of remission in Crohn's disease	Akobeng, , AK; Stokkers, PC	2009
Topical corticosteroids as adjunctive therapy for bacterial keratitis	Herretes, , S, Wang, X; Reyes, JMG	2014

Therapeutic ultrasound for acute ankle sprains	van den Bekerom, , MPJ, van der Windt, DAWM, ter Riet, G, van der Heijden, GJ; Bouter, LM	2011
Technique modifications for reducing the risks from amniocentesis or chorionic villus sampling	Mujezinovic, , F; Alfirevic, Z	2012
Telerehabilitation services for stroke	Laver, , KE, Schoene, D, Crotty, M, George, S, Lannin, NA; Sherrington, C	2013
The role of alpha blockers prior to removal of urethral catheter for acute urinary retention in men	Fisher, , E, Subramonian, K; Omar, MI	2014
Tranexamic acid for reducing mortality in emergency and urgent surgery	Perel, , P, Ker, K, Morales Uribe, CH; Roberts, I	2013
Thrombolytic agents for arterial and venous thromboses in neonates	John, , CM; Harkensee, C	2005
Teriflunomide for multiple sclerosis	He, , D, Zhang, C, Zhao, X, Zhang, Y, Dai, Q, Li, Y; Chu, L	2016
Tiagabine add-on for drug-resistant partial epilepsy	Pulman, , J, Hutton, JL; Marson, AG	2014
Traditional corticosteroids for induction of remission in Crohn's disease	Benchimol, , EI, Seow, CH, Steinhart, AH; Griffiths, AM	2008
Traditional Chinese herbal products for stable angina	Zhuo, , Q, Yuan, Z, Chen, H; Wu, T	2010
TNF-α blockers for the treatment of Kawasaki disease in children	Yamaji, , N, da Silva Lopes, K, Shoda, T, Ishitsuka, K, Kobayashi, T, Ota, E; Mori, R	2019
Topical and systemic antifungal therapy for chronic rhinosinusitis	Head, , K, Sharp, S, Chong, LY, Hopkins, C; Philpott, C	2018
Teledermatology for diagnosing skin cancer in adults	Chuchu, , N, Dinnes, J, Takwoingi, Y, Matin, RN, Bayliss, SE, Davenport, C, Moreau, JF, Bassett, O, Godfrey, K, O'Sullivan, C, Walter, FM, Motley, R, Deeks, JJ; Williams, HC	2018
Written information for patients (or parents of child patients) to reduce the use of antibiotics for acute upper respiratory tract infections in primary care	O'Sullivan, , JW, Harvey, RT, Glasziou, PP; McCullough, A	2016
Volume-targeted versus pressure-limited ventilation in neonates	Klingenberg, , C, Wheeler, KI, McCallion, N, Morley, CJ; Davis, PG	2017
Washout policies in long-term indwelling urinary catheterisation in adults	Shepherd, , AJ, Mackay, WG; Hagen, S	2017
Whole grain foods for the prevention of type 2 diabetes mellitus	Priebe, , M, van Binsbergen, J, de Vos, R; Vonk, RJ	2008
Vitamins C and E for asthma and exercise-induced bronchoconstriction	Wilkinson, , M, Hart, A, Milan, SJ; Sugumar, K	2014
Zotepine for schizophrenia	DeSilva, , P, Fenton, M; Rathbone, J	2006
Yoga as part of a package of care versus standard care for schizophrenia	Broderick, , J; Vancampfort, D	2017
Water-based exercises for improving activities of daily living after stroke	Mehrholz, , J, Kugler, J; Pohl, M	2011
Zotepine versus other atypical antipsychotics for schizophrenia	Subramanian, , S, Rummel-Kluge, C, Hunger, H, Schmid, F, Schwarz, S, Kissling, W, Leucht, S; Komossa, K	2010
Zinc supplements for preventing otitis media	Gulani, , A; Sachdev, HS	2014
Vitamin supplementation for preventing miscarriage	Balogun, , OO, da Silva Lopes, K, Ota, E, Takemoto, Y, Rumbold, A, Takegata, M; Mori, R	2016
Yellow fever vaccine for patients with HIV infection	Barte, , H, Horvath, TH; Rutherford, GW	2014
Vitamin K antagonists versus antiplatelet therapy after transient ischaemic attack or minor ischaemic stroke of presumed arterial origin	De Schryver, , ELLM, Algra, A, Kappelle, LJ, van Gijn, J; Koudstaal, PJ	2012
Voxel-based morphometry for separation of schizophrenia from other types of psychosis in first episode psychosis	Palaniyappan, , L, Maayan, N, Bergman, H, Davenport, C, Adams, CE; Soares-Weiser, K	2015
Walk-in clinics versus physician offices and emergency rooms for urgent care and chronic disease management	Chen, , CE, Chen, CT, Hu, J; Mehrotra, A	2017
Vitamin K for upper gastrointestinal bleeding in people with acute or chronic liver diseases	Martí-Carvajal, , AJ; Solà, I	2015
Vitamin K antagonists versus low-molecular-weight heparin for the long term treatment of symptomatic venous thromboembolism	Andras, , A, Sala Tenna, A; Stewart, M	2017
Water-based exercise for adults with asthma	Grande, , AJ, Silva, V, Andriolo, BNG, Riera, R, Parra, SA; Peccin, MS	2014
Zuclopenthixol acetate for acute schizophrenia and similar serious mental illnesses	Jayakody, , K, Gibson, RC, Kumar, A; Gunadasa, S	2012
Whole brain radiotherapy for the treatment of newly diagnosed multiple brain metastases	Tsao, , MN, Xu, W, Wong, RKS, Lloyd, N, Laperriere, N, Sahgal, A, Rakovitch, E; Chow, E	2018
Weight loss interventions for chronic asthma	Adeniyi, , FB; Young, T	2012
Written emotional disclosure for asthma	Paudyal, , P, Hine, P, Theadom, A, Apfelbacher, CJ, Jones, CJ, Yorke, J, Hankins, M; Smith, HE	2014
Workload and surgeon´s specialty for outcome after colorectal cancer surgery	Archampong, , D, Borowski, D, Wille-Jørgensen, P; Iversen, LH	2012
Yoga for asthma	Yang, , ZY, Zhong, HB, Mao, C, Yuan, JQ, Huang, YF, Wu, XY, Gao, YM; Tang, JL	2016
Whole-body vibration training for patients with neurodegenerative disease	Sitjà Rabert, , M, Rigau Comas, D, Fort Vanmeerhaeghe, A, Santoyo Medina, C, Roqué i Figuls, M, Romero-Rodríguez, D; Bonfill Cosp, X	2012
Vitamin E for intermittent claudication	Kleijnen, , J; Mackerras, D	1998

Wound drainage after axillary dissection for carcinoma of the breast	Thomson, , DR, Sadideen, H; Furniss, D	2013
Workplace interventions to prevent work disability in workers on sick leave	van Vilsteren, , M, van Oostrom, SH, de Vet, HCW, Franche, RL, Boot, CRL; Anema, JR	2015
Zuclopenthixol versus placebo for schizophrenia	Lacey, , M; Jayaram, MB	2015
Vitamins for epilepsy	Ranganathan, , LN; Ramaratnam, S	2005
Zinc supplementation for mental and motor development in children	Gogia, , S; Sachdev, HS	2012
Washed versus unwashed red blood cells for transfusion for the prevention of morbidity and mortality in preterm infants	Keir, , AK, Wilkinson, D, Andersen, C; Stark, MJ	2016
Workplace interventions for treatment of occupational asthma	de Groene, , GJ, Pal, TM, Beach, J, Tarlo, SM, Spreeuwers, D, Frings-Dresen, MHW, Mattioli, S; Verbeek, JH	2011
Yoga for the primary prevention of cardiovascular disease	Hartley, , L, Dyakova, M, Holmes, J, Clarke, A, Lee, MS, Ernst, E; Rees, K	2014
Vitamin K for the primary prevention of cardiovascular disease	Hartley, , L, Clar, C, Ghannam, O, Flowers, N, Stranges, S; Rees, K	2015
Workplace interventions for smoking cessation	Cahill, , K; Lancaster, T	2014
Water for preventing urinary stones	Bao, , Y; Wei, Q	2012
Zinc supplements for treating thalassaemia and sickle cell disease	Swe, , KMM, Abas, ABL, Bhardwaj, A, Barua, A; Nair, NS	2013
Vitamin E supplementation in people with cystic fibrosis	Okebukola, , PO, Kansra, S; Barrett, J	2017
Wound-care teams for preventing and treating pressure ulcers	Moore, , ZEH, Webster, J; Samuriwo, R	2015
Zonisamide add-on therapy for focal epilepsy	Brigo, , F, Lattanzi, S, Igwe, SC, Behzadifar, M; Bragazzi, NL	2018
Zinc supplementation for the prevention of pneumonia in children aged 2 months to 59 months	Lassi, , ZS, Moin, A; Bhutta, ZA	2016
Vitamin E supplementation in pregnancy	Rumbold, , A, Ota, E, Hori, H, Miyazaki, C; Crowther, CA	2015
Yoga treatment for chronic non-specific low back pain	Wieland, , LS, Skoetz, N, Pilkington, K, Vempati, R, D'Adamo, CR; Berman, BM	2017
Workplace pedometer interventions for increasing physical activity	Freak-Poli, , RLA, Cumpston, M, Peeters, A; Clemes, SA	2013
Vitrectomy with internal limiting membrane (ILM) peeling versus vitrectomy with no peeling for idiopathic full-thickness macular hole (FTMH)	Spiteri Cornish, , K, Lois, N, Scott, N, Burr, J, Cook, J, Boachie, C, Tadayoni, R, la Cour, M, Christensen, U; Kwok, A	2013
Water fluoridation for the prevention of dental caries	Iheozor-Ejiofor, , Z, Worthington, HV, Walsh, T, O'Malley, L, Clarkson, JE, Macey, R, Alam, R, Tugwell, P, Welch, V; Glenny, AM	2015
Wound drainage following groin dissection for malignant disease in adults	Thomson, , DR, Sadideen, H; Furniss, D	2014
Whole grain cereals for the primary or secondary prevention of cardiovascular disease	Kelly, , SAM, Hartley, L, Loveman, E, Colquitt, JL, Jones, HM, Al-Khudairy, L, Clar, C, Germanò, R, Lunn, HR, Frost, G; Rees, K	2017
Water-based exercise training for chronic obstructive pulmonary disease	McNamara, , RJ, McKeough, ZJ, McKenzie, DK; Alison, JA	2013
Voluntary counseling and testing (VCT) for changing HIV-related risk behavior in developing countries	Fonner, , VA, Denison, J, Kennedy, CE, O'Reilly, K; Sweat, M	2012
Xylitol for preventing acute otitis media in children up to 12 years of age	Azarpazhooh, , A, Lawrence, HP; Shah, PS	2016
Work-break schedules for preventing musculoskeletal symptoms and disorders in healthy workers	Luger, , T, Maher, CG, Rieger, MA; Steinhilber, B	2019
Zinc supplementation for preventing mortality, morbidity, and growth failure in children aged 6 months to 12 years of age	Mayo-Wilson, , E, Junior, JA, Imdad, A, Dean, S, Chan, XHS, Chan, ES, Jaswal, A; Bhutta, ZA	2014
Zinc supplementation as an adjunct to antibiotics in the treatment of pneumonia in children 2 to 59 months of age	Haider, , BA, Lassi, ZS, Ahmed, A; Bhutta, ZA	2011
Vitamin E supplementation for prevention of morbidity and mortality in preterm infants	Brion, , LP, Bell, EF; Raghuveer, TS	2003
Yoga for treating urinary incontinence in women	Wieland, , LS, Shrestha, N, Lassi, ZS, Panda, S, Chiaramonte, D; Skoetz, N	2019
Vocational rehabilitation for people with severe mental illness	Crowther, , R, Marshall, M, Bond, GR; Huxley, P	2001
Xylitol-containing products for preventing dental caries in children and adults	Riley, , P, Moore, D, Ahmed, F, Sharif, MO; Worthington, HV	2015
Which anticholinergic drug for overactive bladder symptoms in adults	Madhuvrata, , P, Cody, JD, Ellis, G, Herbison, GP; Hay-Smith, EJC	2012
Weight reduction for non-alcoholic fatty liver disease	Peng, , L, Wang, J; Li, F	2011
Zidovudine (AZT) versus AZT plus didanosine (ddI) versus AZT plus zalcitabine (ddC) in HIV infected adults	Darbyshire, , J, Foulkes, M, Peto, R, Duncan, W, Babiker, A, Collins, R, Hughes, M, Peto, TEA; Walker, SA	2000
Zinc supplementation for the prevention of type 2 diabetes mellitus in adults with insulin resistance	El Dib, , R, Gameiro, OLF, Ogata, MSP, Módolo, NSP, Braz, LG, Jorge, EC, do Nascimento Junior, P; Beletate, V	2015
Vortioxetine for depression in adults	Koesters, , M, Ostuzzi, G, Guaiana, G, Breilmann, J; Barbui, C	2017
Water infusion versus air insufflation for colonoscopy	Hafner, , S, Zolk, K, Radaelli, F, Otte, J, Rabenstein, T; Zolk, O	2015

Zinc supplementation for tinnitus	Person, , OC, Puga, MES, da Silva, EMK; Torloni, MR	2016
Yoga for improving health-related quality of life, mental health and cancer-related symptoms in women diagnosed with breast cancer	Cramer, , H, Lauche, R, Klose, P, Lange, S, Langhorst, J; Dobos, GJ	2017
Xiongshao for restenosis after percutaneous coronary intervention in patients with coronary heart disease	Zheng, , GH, Liu, JP, Chu, JF, Mei, L; Chen, HY	2013
Vitrectomy for idiopathic macular hole	Parravano, , M, Giansanti, F, Eandi, CM, Yap, YC, Rizzo, S; Virgili, G	2015
Vitamin E for antipsychotic-induced tardive dyskinesia	Soares-Weiser, , K, Maayan, N; Bergman, H	2018
Withdrawal of immunosuppressant or biologic therapy for patients with quiescent Crohn's disease	Boyapati, , RK, Torres, J, Palmela, C, Parker, CE, Silverberg, OM, Upadhyaya, SD, Nguyen, TM; Colombel, JF	2018
Vitamin K supplementation for cystic fibrosis	Jagannath, , VA, Thaker, V, Chang, AB; Price, AI	2017
Whole-body cryotherapy (extreme cold air exposure) for preventing and treating muscle soreness after exercise in adults	Costello, , JT, Baker, PRA, Minett, GM, Bieuzen, F, Stewart, IB; Bleakley, C	2015
Zonisamide for neuropathic pain in adults	Moore, , RA, Wiffen, PJ, Derry, S; Lunn, MPT	2015
Vitamin K prior to preterm birth for preventing neonatal periventricular haemorrhage	Crowther, , CA; Crosby, DD	2010
Yoga as part of a package of care versus non-standard care for schizophrenia	Broderick, , J; Vancampfort, D	2019
Xpert MTB/RIF and Xpert MTB/RIF Ultra for pulmonary tuberculosis and rifampicin resistance in adults	Horne, , DJ, Kohli, M, Zifodya, JS, Schiller, I, Dendukuri, N, Tollefson, D, Schumacher, SG, Ochodo, EA, Pai, M; Steingart, KR	2019
Randomised, phase II, placebo-controlled, trial of fulvestrant plus vandetanib in postmenopausal women with bone only or bone predominant, hormone-receptor-positive metastatic breast cancer (MBC): the OCOG ZAMBONEY study	Clemons, , MJ, Cochrane, B, Pond, GR, Califaretti, N, Chia, SK, Dent, RA, Song, X, Robidoux, A, Parpia, S, Warr, D, Rayson, D, Pritchard, KI; Levine, MN	2014
Evaluation of Antimicrobial Photodynamic Therapy in Multiple Applications as a Coadjuvant in the Surgical Therapy of Access to Scaling	NCT03498404	2018
Total Therapy XVII for Newly Diagnosed Patients With Acute Lymphoblastic Leukemia and Lymphoma	NCT03117751	2017
Frailty and the biochemical effects of recombinant human growth hormone in women after surgery for hip fracture	Yeo, , AL, Levy, D, Martin, FC, Sönksen, P, Sturgess, I, Wheeler, MM; Young, A	2003
Vitamin D supplementation for the prevention of type 2 diabetes in overweight adults: study protocol for a randomized controlled trial	de Courten, , B, Mousa, A, Naderpoor, N, Teede, H, de Courten, MP; Scragg, R	2015
Thermotherapy application increases the mRNA expression of angiogenic factors in human skeletal muscle	Roseguini, , BT, Kuhlenhoelter, AM, Neff, D, Nie, Y, Wong, B; Gavin, TP	2016
Oral paricalcitol for the treatment of secondary hyperparathyroidism in patients on hemodialysis or peritoneal dialysis	Ross, , EA, Tian, J, Abboud, H, Hippensteel, R, Melnick, JZ, Pradhan, RS, Williams, LA, Hamm, LL; Sprague, SM	2008
A phase I/II clinical trial to evaluate a combination of recombinant human platelet-derived growth factor-BB and recombinant human insulin-like growth factor-I in patients with periodontal disease	Howell, , TH, Fiorellini, JP, Paquette, DW, Offenbacher, S, Giannobile, WV; Lynch, SE	1997
Effect of adjunctive systemic azithromycin with periodontal surgery in the treatment of chronic periodontitis in smokers: a pilot study	Dastoor, , SF, Travan, S, Neiva, RF, Rayburn, LA, Giannobile, WV; Wang, HL	2007
Phase 1/2 study of immunotherapy with dendritic cells pulsed with autologous tumor lysate in patients with refractory bone and soft tissue sarcoma	Miwa, , S, Nishida, H, Tanzawa, Y, Takeuchi, A, Hayashi, K, Yamamoto, N, Mizukoshi, E, Nakamoto, Y, Kaneko, S; Tsuchiya, H	2017
Effect of rhPDGF-BB on bone turnover during periodontal repair	Sarment, , DP, Cooke, JW, Miller, SE, Jin, Q, McGuire, MK, Kao, RT, McClain, PK, McAllister, BS, Lynch, SE; Giannobile, WV	2006
Efficacy and safety of very-low-calorie ketogenic diet: a double blind randomized crossover study	Colica, , C, Merra, G, Gasbarrini, A, De Lorenzo, A, Cioccoloni, G, Gualtieri, P, Perrone, MA, Bernardini, S, Bernardo, V, Di Renzo, L; Marchetti, M	2017
Dose intensity and high dose therapy: two different concepts	Livingston, RB	1994
Impaired plasmacytoid dendritic cell maturation and differential chemotaxis in chronic hepatitis C virus: associations with antiviral treatment outcomes	Mengshol, , JA, Golden-Mason, L, Castelblanco, N, Im, KA, Dillon, SM, Wilson, CC; Rosen, HR	2009
Phase II trial evaluating the palliative benefit of second-line zoledronic acid in breast cancer patients with either a skeletal-related event or progressive bone metastases despite first-line bisphosphonate therapy	Clemons, , MJ, Dranitsaris, G, Ooi, WS, Yogendran, G, Sukovic, T, Wong, BYL, Verma, S, Pritchard, KI, Trudeau, M; Cole, DEC	2006
Melatonin Osteoporosis Prevention Study	NCT01152580	2010
Growth hormone replacement therapy improves body composition and increases bone metabolism in elderly patients with pituitary disease	Fernholm, , R, Bramnert, M, Hägg, E, Hilding, A, Baylink, DJ, Mohan, S; Thorén, M	2000
The engineered human anti-tumor necrosis factor-alpha antibody CDP571 inhibits inflammatory pathways but not T cell activation in patients with rheumatoid arthritis	Choy, , EH, Rankin, EC, Kassimos, D, Vetterlein, O, Garyfallos, A, Ravirajan, CT, Sopwith, M, Eastell, R, Kingsley, GH, Isenberg, DA; Panayi, GS	1999
Clinical evaluation of autologous platelet rich fibrin in horizontal alveolar bony defects	Rosamma Joseph, , V, Sam, G; Vijay Amol, N	2014
Is breast magnetic resonance imaging (MRI) useful for diagnosis of additional sites of disease in patients recently diagnosed with ductal carcinoma in situ (DCIS)?	Ortiz-Perez, , T, Benveniste, AP, Ebuoma, LO, Sepulveda, KA, Severs, FJ, Kapoor, M; Sedgwick, EL	2017
Immunomodulatory Properties of Ketamine in Sepsis	NCT01089361	2010

Fibroblast growth factor 23 and parathyroid hormone after treatment with active vitamin D and sevelamer carbonate in patients with chronic kidney disease stage 3b, a randomized crossover trial	Bleskestad, , IH, Bergrem, H, Hartmann, A, Godang, K; Gøransson, LG	2012
Phase II open label, multi-center clinical trial of modulation of intermediate endpoint biomarkers by 1alpha-hydroxyvitamin D2 in patients with clinically localized prostate cancer and high grade pin	Gee, , J, Bailey, H, Kim, K, Kolesar, J, Havighurst, T, Tutsch, KD, See, W, Cohen, MB, Street, N, Levan, L, Jarrard, D; Wilding, G	2013
Paricalcitol for secondary hyperparathyroidism in renal transplantation	Trillini, , M, Cortinovis, M, Ruggenenti, P, Reyes Loaeza, J, Courville, K, Ferrer-Siles, C, Prandini, S, Gaspari, F, Cannata, A, Villa, A, Perna, A, Gotti, E, Caruso, MR, Martinetti, D, Remuzzi, G; Perico, N	2015
Predictors of prolonged benefit from palbociclib plus fulvestrant in women with endocrine-resistant hormone receptor-positive/human epidermal growth factor receptor 2-negative metastatic breast cancer in PALOMA-3	Cristofanilli, , M, DeMichele, A, Giorgetti, C, Turner, NC, Slamon, DJ, Im, S-A, Masuda, N, Verma, S, Loi, S, Colleoni, M, Theall, KP, Huang, X, Liu, Y; Bartlett, CH	2018
Pilot of a Prebiotic and Probiotic Trial in Young Infants With Severe Acute Malnutrition	NCT03666572	2018
Subantimicrobial-dose doxycycline modulates gingival crevicular fluid biomarkers of periodontitis in postmenopausal osteopenic women	Golub, , LM, Lee, HM, Stoner, JA, Sorsa, T, Reinhardt, RA, Wolff, MS, Ryan, ME, Nummikoski, PV; Payne, JB	2008
Overall survival (OS) and updated disease-free survival (DFS) results of the NSABP C-08 trial assessing bevacizumab (B) in stage II and III colon cancer	Allegra, , CJ, Yothers, GA, O'Connell, MJ, Sharif, S, Petrelli, NJ, Colangelo, LH; Wolmark, N	2011
Synthetic human parathyroid hormone 1-34 vs calcitriol and calcium in the treatment of hypoparathyroidism	Winer, , KK, Yanovski, JA; Cutler, GB	1996
Phase I trial of the matrix metalloproteinase inhibitor marimastat combined with carboplatin and paclitaxel in patients with advanced non-small cell lung cancer	Goffin, , JR, Anderson, IC, Supko, JG, Eder Jr, JP, Shapiro, GI, Lynch, TJ, Shipp, M, Johnson, BE; Skarin, AT	2005
Effects of Percutaneous Kyphoplasty on Bone Metabolism and Oxidative Stress in Elderly Patients with Osteoporotic Spinal Fractures	Liu, , Q, Cao, J; Kong, J	2019
A Randomized, Controlled Trial of Autologous Platelet Gel Treatment in Diabetic Foot Ulcers	NCT00338702	2006
Danish Cardiovascular Screening Trial II	NCT03946410	2019
Assessment of OPG/RANK/RANKL gene expression levels in peripheral blood mononuclear cells (PBMC) after treatment with strontium ranelate and ibandronate in patients with postmenopausal osteoporosis	Stuss, , M, Rieske, P, Cegłowska, A, Stêpień-Kłos, W, Liberski, PP, Brzeziańska, E; Sewerynek, E	2013
Increased expression of genes after periodontal treatment with photodynamic therapy	Franco, , EJ, Pogue, RE, Sakamoto, LHT, Cavalcante, LLM, Carvalho, DRD; de Andrade, RV	2014
Role of sevoflurane in organ protection during cardiac surgery in children: a randomized controlled trial	Bettex, , DA, Wanner, PM, Bosshart, M, Balmer, C, Knirsch, W, Dave, H, Dillier, C, Bürki, C, Hug, M, Seifert, B, Spahn, DR; Beck-Schimmer, B	2015
Biochemical markers comparison of dynamic hip screw and Gamma nail implants in the treatment of stable intertrochanteric fracture: a prospective study of 60 patients	Song, , W, Chen, Y, Shen, H, Yuan, T, Zhang, C; Zeng, B	2011
Use of doxycycline to decrease the growth rate of abdominal aortic aneurysms: a randomized, double-blind, placebo-controlled pilot study	Mosorin, , M, Juvonen, J, Biancari, F, Satta, J, Surcel, HM, Leinonen, M, Saikku, P; Juvonen, T	2001
Lack of a clear disease modifying activity of celecoxib in treatment of end-stage knee osteoarthritis: a rondomized observer blinded clinical trial	Van Helvoort, , EM, Coeleveld, K, Huisman, AM, Polak, AA, Bijlsma, JW, Van Laar, JM, Lafeber, FP; Mastbergen, SC	2017
Oral Probiotics to Reduce Vaginal Group B Streptococcal Colonization in Late Pregnancy	NCT03008421	2017
Clodronate treatment influences MMP-2 associated outcome in node positive breast cancer	Leppä, , S, Saarto, T, Vehmanen, L, Blomqvist, C; Elomaa, I	2005
Clinical evaluation of insulin like growth factor-i and vascular endothelial growth factor with alloplastic bone graft material in the management of human two wall intra-osseous defects	Devi, , R; Dixit, J	2016
Tocotrienol supplementation suppressed bone resorption and oxidative stress in postmenopausal osteopenic women: a 12-week randomized double-blinded placebo-controlled trial	Shen, , CL, Yang, S, Tomison, MD, Romero, AW, Felton, CK; Mo, H	2018
Evaluating an Exercise Program to Reduce Cardiovascular Risk Factors in Children Infected With HIV	NCT00908284	2009
Assessment of and Treatment Applied to Food Addiction in a Rural Healthy Behaviors Clinic	NCT03431831	2018
Effect of Orthodontic Treatment on the Stability of Pre-Orthodontic Recession Coverage	NCT03886051	2019
Angiogenic biomarkers and healing of living cellular constructs	Morelli, , T, Neiva, R, Nevins, ML, McGuire, MK, Scheyer, ET, Oh, TJ, Braun, TM, Nör, JE, Bates, D; Giannobile, WV	2011
Early control of PTH and FGF23 in normophosphatemic CKD patients: a new target in CKD-MBD therapy?	Oliveira, , RB, Cancela, AL, Graciolli, FG, Dos Reis, LM, Draibe, SA, Cuppari, L, Carvalho, AB, Jorgetti, V, Canziani, ME; Moysés, RM	2010
Safety, pharmacokinetics, and changes in bone metabolism associated with zoledronic acid treatment in Japanese patients with primary osteoporosis	Shiraki, , M, Tanaka, S, Suzuki, H, Ueda, S; Nakamura, T	2016
Serum osteoprotegerin and its ligand in Paget's disease of bone: relationship to disease activity and effect of treatment with bisphosphonates	Alvarez, , L, Peris, P, Guanabens, N, Vidal, S, Ros, I, Pons, F, Filella, X, Monegal, A, Munoz-Gomez, J; Ballesta, AM	2003
Bovine-derived bone protein extract in the treatment of mandibular Class II furcations	Camargo, , PM, Wolinsky, LE, Burgess, AV, Wagner, WR, Paluk, SF; Kenney, EB	2002

Alendronate reduces serum TNFalpha and IL-1beta, increases neutrophil counts, and improves bone mineral density and bone metabolism indices in patients with chronic idiopathic neutropenia (CIN)-associated osteopenia/osteoporosis	Papadaki, , HA, Tsatsanis, C, Christoforidou, A, Malliaraki, N, Psyllaki, M, Pontikoglou, C, Miliaki, M, Margioris, AN; Eliopoulos, GD	2004
The DART Study: results from the Dose-Escalation and Expansion Cohorts Evaluating the Combination of Dalantercept plus Axitinib in Advanced Renal Cell Carcinoma	Voss, , MH, Bhatt, RS, Plimack, ER, Rini, BI, Alter, RS, Beck, JT, Wilson, D, Zhang, X, Mutyaba, M, Glasser, C, Attie, KM, Sherman, ML, Pandya, SS; Atkins, MB	2017
Effect of systemic matrix metalloproteinase inhibition on periodontal wound repair: a proof of concept trial	Gapski, , R, Barr, JL, Sarment, DP, Layher, MG, Socransky, SS; Giannobile, WV	2004
Raloxifene treatment is associated with increased serum estradiol and decreased bone remodeling in healthy middle-aged men with low sex hormone levels	Uebelhart, , B, Herrmann, F, Pavo, I, Draper, MW; Rizzoli, R	2004
Etiological periodontal treatment with and without low level laser therapy on il-1beta level in gingival crevicular fluid: an in vivo multicentric pilot study	Mastrangelo, , F, Dedola, A, Cattoni, F, Ferrini, F, Bova, F, Tatullo, M, Gherlone, E; Muzio, LLO	2018
Treatment evidences beyond 5 years	Olarte, OR	2017
Photodynamic therapy decrease immune-inflammatory mediators levels during periodontal maintenance	da Cruz Andrade, , PV, Euzebio Alves, VT, de Carvalho, VF, De Franco Rodrigues, M, Pannuti, CM, Holzhausen, M, De Micheli, G; Conde, MC	2017
Community-based Clinical Trial With Microbiota-directed Complementary Foods (MDCFs) Made of Locally Available Food Ingredients for the Management of Children With Primary Moderate Acute Malnutrition	NCT04015999	2019
Phase II, parallel-design study of preoperative combined modality therapy and the matrix metalloprotease (mmp) inhibitor prinomastat in patients with esophageal adenocarcinoma	Heath, , EI, Burtness, BA, Kleinberg, L, Salem, RR, Yang, SC, Heitmiller, RF, Canto, MI, Knisely, JP, Topazian, M, Montgomery, E, Tsottles, N, Pithavala, Y, Rohmiller, B, Collier, M; Forastiere, AA	2006
Community-based Clinical Trial With Microbiota Directed Complementary Foods (MDCFs) Made of Locally Available Food Ingredients for the Management of Children With Post-severe Acute Malnutrition Moderate Acute Malnutrition (Post-SAM MAM)	NCT04015986	2019
Coordinate production of PGE2 and IL-1 beta in the gingival crevicular fluid of adults with periodontitis: its relationship to alveolar bone loss and disruption by twice daily treatment with ketorolac tromethamine oral rinse	Cavanaugh, , PF, Meredith, MP, Buchanan, W, Doyle, MJ, Reddy, MS; Jeffcoat, MK	1998
Granulocyte-colony-stimulating factor induces increased serum levels of soluble interleukin 2 receptors preceding engraftment in autologous bone marrow transplantation	Dreger, , P, Grelle, K, Eckstein, V, Suttorp, M, Muller-Ruchholtz, W, Loffler, H; Schmitz, N	1993
Evidenced-Based Review of Clinical Studies on Periodontics		2009
Signatures of mesenchymal cell lineages and microenvironment factors are dysregulated in high risk myeloma	Yaccoby, , S, Qu, P, Mehdi, S, Hoering, A, Epstein, J, Johnson, SK, Van Rhee, F, Zangari, M, Schinke, C, Thanendrarajan, S, Barlogie, B, Davies, FE; Morgan, GJ	2016
Effects of intravitreal pegaptanib or bevacizumab and laser in treatment of threshold retinopathy of prematurity in zone I and posterior zone II–four years results	Autrata, , R, Senková, K, Holousová, M, Krejcírová, I, Dolezel, Z; Borek, I	2012
Targeting inflammatory pathways: a phase 2 trial of the JAK-inhibitor ruxolitinib in combination with exemestane for aromatase inhibitorresistant, estrogen receptor-positive breast cancer	DeMichele, , AM, Clark, AS, Holmes, R, Volpe, M, Medrano, C, Troxel, A, Fox, K, Domchek, S, Matro, J, Bradbury, A, Shih, N, Feldman, M, Hexner, E; Bromberg, J	2017
Melatonin improves bone mineral density at the femoral neck in postmenopausal women with osteopenia: a randomized controlled trial	Amstrup, , AK, Sikjaer, T, Heickendorff, L, Mosekilde, L; Rejnmark, L	2015
Intravenous ibandronate acutely reduces bone hyperresorption in chronic critical illness	Via, , MA, Potenza, MV, Hollander, J, Liu, X, Peng, Y, Li, J, Sun, L, Zaidi, M; Mechanick, JI	2012
Adalimumab reduces hand bone loss in rheumatoid arthritis independent of clinical response: subanalysis of the PREMIER study	Hoff, , M, Kvien, TK, Kälvesten, J, Elden, A, Kavanaugh, A; Haugeberg, G	2011
Effects of Supplementation of Vitamin D in Patients With Crohn`s Disease	NCT02704624	2016
Early parathyroidectomy increases bone mineral density in patients with mild primary hyperparathyroidism: a prospective and randomized study	Almqvist, , EG, Becker, C, Bondeson, AG, Bondeson, L; Svensson, J	2004
Effects of N-acetylcysteine on the cardiac remodeling biomarkers and major adverse events following acute myocardial infarction: a randomized clinical trial	Talasaz, , AH, Khalili, H, Fahimi, F, Jenab, Y, Broumand, MA, Salarifar, M; Darabi, F	2014
A prospective, randomised, placebo-controlled, double-masked, three-armed, multicentre phase II/III trial for the Study of a Topical Treatment of Ischaemic Central Retinal Vein Occlusion to Prevent Neovascular Glaucoma - the STRONG study: study protocol for a randomised controlled trial	Lorenz, , K, Scheller, Y, Bell, K, Grus, F, Ponto, KA, Bock, F, Cursiefen, C, Flach, J, Gehring, M, Peto, T, Silva, R, Tal, Y; Pfeiffer, N	2017
First prospective data on impact of minimal residual disease on long-term clinical outcomes after venetoclax plus rituximab versus bendamustine plus rituximab: phase III murano study	Kater, , AP, Hillmen, P, Eichhorst, B, Langerak, AW, Kipps, TJ, Owen, C, Boyer, M, Humphrey, K, Punnoose, EA, Wang, J, Chyla, B, Verdugo, ME, Wu, J, Jiang, Y, Mobasher, M; Seymour, JF	2018
Reduced bone loss by alphacalcidol in glucocorticoid-induced osteoporosis	Lakatos, , P, Kiss, L, Horvath, C, Takacs, I, Foldes, J, Bossanyi, A, Buzas, E; Major, T	1996
Evaluation of cyclosporine A with β-TCP in the treatment of human infra bony defects – A randomized controlled pilot study	Amalakara, , J, Reddy, K, Avula, H, Mishra, A, Kalakonda, B; Pandey, R	2017
Randomized Trial Comparing Single vs. Maintenance Fecal Microbiota Transplant for Refractory Crohn's Disease in Children	NCT03747718	2018

Long-term effects of magnesium carbonate on coronary artery calcification and bone mineral density in hemodialysis patients: a pilot study	Spiegel, , DM; Farmer, B	2009
A prospective, randomized, controlled trial comparing radiographic and clinical outcomes between stand-alone lateral interbody lumbar fusion with either silicate calcium phosphate or rh-BMP2	Pimenta, , L, Marchi, L, Oliveira, L, Coutinho, E; Amaral, R	2013
Mud-bath treatment in spondylitis associated with inflammatory bowel disease–a pilot randomised clinical trial	Cozzi, , F, Podswiadek, M, Cardinale, G, Oliviero, F, Dani, L, Sfriso, P; Punzi, L	2007
The effect of neonatal sepsis on bone turnover in very-low birth weight premature infants	Eliakim, , A, Shiff, Y, Nemet, D; Dolfin, T	2003
Effect of High-Flux Dialysis on Circulating FGF-23 Levels in End-Stage Renal Disease Patients: results from a Randomized Trial	Schneider, , A, Schneider, MP, Krieter, DH, Genser, B, Scharnagl, H, Stojakovic, T, Wanner, C; Drechsler, C	2015
Elevation of serum tumor necrosis factor alpha in patients with periprosthetic osteolysis: a case-control study	Chaganti, , RK, Purdue, E, Sculco, TP; Mandl, LA	2014
Breakfast replacement with a low-glycaemic response liquid formula in patients with type 2 diabetes: a randomised clinical trial	Stenvers, , DJ, Schouten, LJ, Jurgens, J, Endert, E, Kalsbeek, A, Fliers, E; Bisschop, PH	2014
Effects of GM-CSF on the stem cells mobilization and plasma C-reactive protein levels in patients with acute myocardial infarction	Deng, , Z, Yang, C, Deng, H, Yang, A, Geng, T, Chen, X, Ma, A; Liu, Z	2006
Vitamin D and Carboxy PTH Fragments in Coronary Calcification	NCT00502268	2007
Matrix metalloproteinase-3 serum levels are correlated with disease activity and predict clinical response in rheumatoid arthritis	Ribbens, , C, Andre, B, Jaspar, J-M, Kaye, O, Kaiser, M-J, De Groote, D; Malaise, MG	2000
Efficacy of modified LiuJunZi decoction on functional dyspepsia of spleen-deficiency and qi-stagnation syndrome: a randomized controlled trial	Zhang, , S, Zhao, L, Wang, H, Wang, C, Huang, S, Shen, H, Wei, W, Tao, L; Zhou, T	2013
Matrix metalloproteinase-3 serum levels are correlated with disease activity and predict clinical response in rheumatoid arthritis		2000
Phase I study of PF-03446962, a fully human monoclonal antibody against activin receptor-like kinase-1, in patients with hepatocellular carcinoma	Simonelli, , M, Zucali, P, Santoro, A, Thomas, MB, de Braud, FG, Borghaei, H, Berlin, J, Denlinger, CS, Noberasco, C, Rimassa, L, Kim, T-Y, English, PA, Abbattista, A, Gallo Stampino, C, Carpentieri, M; Williams, JA	2016
Effect of unfractionated and low-molecular-weight heparin on OPG, sRANKL, and von Willebrand factor concentrations during hemodialysis	Klejna, , K, Naumnik, B, Koc-Żórawska, E; Myśliwiec, M	2014
The Effectiveness of Different Methods for Healing a Palatal Donor Site	NCT03567148	2018
Calcitriol and doxercalciferol are equivalent in controlling bone turnover, suppressing parathyroid hormone, and increasing fibroblast growth factor-23 in secondary hyperparathyroidism	Wesseling-Perry, , K, Pereira, RC, Sahney, S, Gales, B, Wang, HJ, Elashoff, R, Jüppner, H; Salusky, IB	2011
Cilostazol prevents foot ulcers in diabetic patients with peripheral vascular disease	de Franciscis, , S, Gallelli, L, Battaglia, L, Molinari, V, Montemurro, R, Stillitano, DM, Buffone, G; Serra, R	2015
The Efficacy of TGF for Treating Osteoarthritis of the Knee	NCT03562429	2018
Dose selection trial of metronomic oral vinorelbine monotherapy in patients with metastatic cancer: a hellenic cooperative oncology group clinical translational study	Briasoulis, , E, Aravantinos, G, Kouvatseas, G, Pappas, P, Biziota, E, Sainis, I, Makatsoris, T, Varthalitis, I, Xanthakis, I, Vassias, A, Klouvas, G, Boukovinas, I, Fountzilas, G, Syrigos, KN, Kalofonos, H; Samantas, E	2013
Correction of vitamin D status by calcidiol: pharmacokinetic profile, safety, and biochemical effects on bone and mineral metabolism of daily and weekly dosage regimens	Minisola, , S, Cianferotti, L, Biondi, P, Cipriani, C, Fossi, C, Franceschelli, F, Giusti, F, Leoncini, G, Pepe, J, Bischoff-Ferrari, HA; Brandi, ML	2017
Increased Bile Acids and FGF19 After Sleeve Gastrectomy and Roux-en-Y Gastric Bypass Correlate with Improvement in Type 2 Diabetes in a Randomized Trial	Nemati, , R, Lu, J, Dokpuang, D, Booth, M, Plank, LD; Murphy, R	2018
Diagnostic Biomarkers Related to Periodontal Disease Activity in Diabetic	NCT02220751	2014
Randomized clinical trial comparing efficacy and safety of brand versus generic alendronate (Bonmax) for osteoporosis treatment	Unnanuntana, , A, Jarusriwanna, A; Songcharoen, P	2017
Pharmacokinetics and tolerability of multiple-dose rosuvastatin: an open-label, randomized-sequence, three-way crossover trial in healthy Chinese volunteers	Zhang, , R, Li, Y, Jiang, X; Wang, L	2009
Subconjunctival Bevacizumab to Prevent Bleb Failure After Glaucoma Filtration Surgery	NCT00468429	2007
Clinical treatment of alcoholic liver disease	Kim, DJ	2012
The SongDance Study: stress, Ongoing Self- Monitoring and Diabetes, Nerve Stimulation and Cognitive Empowerment	NCT03576430	2018
Effect of atorvastatin on chronic periodontitis: a randomized pilot study	Fajardo, , ME, Rocha, ML, Sánchez-Marin, FJ; Espinosa-Chávez, EJ	2010
Small for gestational age (SGA): endocrine and metabolic consequences and effects of growth hormone treatment	Hokken-Koelega, , AC, De Waal, WJ, Sas, TC, Van Pareren, Y; Arends, NJ	2004
The effect of calcium and phosphorus supplementation on metabolic bone disorders in premature infants	Torabi, , Z, Moemeni, N, Ahmadiafshar, A; Mazloomzadeh, S	2014
The ACT-OUT Trial: aCTivity OUTcomes Based on High Carbohydrate or High Fat Diet in Metabolic Syndrome	NCT01357382	2011
Effects of Sevelamer Carbonate in Patients With CKD and Proteinuria: the ANSWER Randomized Trial	Ruggiero, , B, Trillini, M, Tartaglione, L, Rotondi, S, Perticucci, E, Tripepi, R, Aparicio, C, Lecchi, V, Perna, A, Peraro, F, Villa, D, Ferrari, S, Cannata, A, Mazzaferro, S, Mallamaci, F, Zoccali, C, Bellasi, A, Cozzolino, M, Remuzzi, G, Ruggenenti, P; Kohan, DE	2019

TBCRC-010: phase I/II study of dasatinib in combination with zoledronic acid for the treatment of breast cancer bone metastasis	Mitri, , Z, Nanda, R, Blackwell, K, Costelloe, CM, Hood, I, Wei, C, Brewster, AM, Ibrahim, NK, Koenig, KB, Hortobagyi, GN, Van Poznak, C, Rimawi, MF; Moulder-Thompson, S	2016
Clinical study on effect of Astragalus Injection and its immuno-regulation action in treating chronic aplastic anemia	Wang, , MS, Li, J, Di, HX, Li, ZL, Yang, SL, Hou, W, Yan, JY; Zhao, XM	2007
Growth Factors Release of PRF and PRGF	NCT02447510	2015
On the neuroendocrinopathy of critical illness: perspectives for feeding and novel treatments	Van Den Berghe, G	2016
New targets in AML	Yilmaz, M	2014
The role of 25-hydroxyvitamin D deficiency in promoting insulin resistance and inflammation in patients with chronic kidney disease: a randomised controlled trial	Petchey, , WG, Hickman, IJ, Duncan, E, Prins, JB, Hawley, CM, Johnson, DW, Barraclough, K; Isbel, NM	2009
The efficacy and safety of tocilizumab combined with disease-modifying anti-rheumatoid drugs in the treatment of active rheumatoid arthritis: a multi-center, randomized, double-blinded, placebo-controlled trial	Shi, , Q, Zhao, Y, Bao, CD, Li, XF, Huang, F, Zhu, P, Li, ZG, Gu, JR, Zhang, ZY, Zhao, DB, Zhao, SL, Jiang, QD, Tian, J; Zhang, FC	2013
Clinical effects of hormone replacement therapy with estradiol valerate and cyproterone acetate in perimenopausal women	Tinelli, , FG, Tinelli, A; Sena, T	2002
Loop diuretics increase bone turnover and decrease BMD in osteopenic postmenopausal women: results from a randomized controlled study with bumetanide	Rejnmark, , L, Vestergaard, P, Heickendorff, L, Andreasen, F; Mosekilde, L	2006
Maternal serum interleukin-6, C-reactive protein, and matrix metalloproteinase-9 concentrations as risk factors for preterm birth <32 weeks and adverse neonatal outcomes	Sorokin, , Y, Romero, R, Mele, L, Wapner, RJ, Iams, JD, Dudley, DJ, Spong, CY, Peaceman, AM, Leveno, KJ, Harper, M, Caritis, SN, Miodovnik, M, Mercer, BM, Thorp, JM, O'Sullivan, MJ, Ramin, SM, Carpenter, MW, Rouse, DJ; Sibai, B	2010
Effect of metformin treatment on circulating osteoprotegerin in patients with nonalcoholic fatty liver disease	Sofer, , E; Shargorodsky, M	2016
Insulin Therapy Reduce Post-Operative Inflammatory Response After Curative Colorectal Cancer Resection: randomization Controlled Trial	NCT02746432	2016
Stem cells cultured on beta tricalcium phosphate (beta-TCP) in combination with recombinant human platelet-derived growth Factor-BB (rh-PDGF-BB) for the treatment of human infrabony defects	Dhote, , R, Charde, P, Bhongade, M; Rao, J	2015
Spinal Cord Stimulation in Patients With Complex Regional Pain Syndrome: a Possible Target for Immunomodulation?	Kriek, , N, Schreurs, MWJ, Groeneweg, JG, Dik, WA, Tjiang, GCH, Gültuna, I, Stronks, DL; Huygen, FJPM	2018
Systemic MMP inhibition for periodontal wound repair: results of a multi-centre randomized-controlled clinical trial	Gapski, , R, Hasturk, H, Van Dyke, TE, Oringer, RJ, Wang, S, Braun, TM; Giannobile, WV	2009
Effect of dietary calcium vs calcium citrate on conventional biochemical markers in perimenopausal women	Aguilera-Barreiro Mde, , L, Guerrero-Mercado Adel, S, Méndez-Jiménez, TE; Milián-Suazo, F	2005
Effect of nonsurgical periodontal therapy on interleukin-34 levels in periodontal health and disease	Guruprasad, , CN; Pradeep, AR	2018
Ketogenic Diet: a Novel Metabolic Strategy to Treat Lymphedema Patients?	NCT03991897	2019
Risk factors for the development of vertebral fractures after percutaneous vertebroplasty	Martinez-Ferrer, , A, Blasco, J, Carrasco, JL, Macho, JM, Román, LS, López, A, Monegal, A, Guañabens, N; Peris, P	2013
Investigating the Effect of a Single Infusion of Reconstituted High-Density Lipoprotein in Patients with Symptomatic Carotid Plaques	Nasr, , H, Torsney, E, Poston, RN, Hayes, L, Gaze, DC, Basser, R, Thompson, MM, Loftus, IM; Cockerill, GW	2015
Bone Health in Gynecologic Cancers-does FOSAVANCE Help?	NCT00593580	2008
Antimicrobial photodynamic therapy in the non-surgical treatment of aggressive periodontitis: cytokine profile in gingival crevicular fluid, preliminary results	de Oliveira, , RR, Schwartz-Filho, HO, Novaes, AB, Garlet, GP, de Souza, RF, Taba, M, Scombatti de Souza, SL; Ribeiro, FJ	2009
Melatonin as an Adjunctive Therapy for Chronic Periodontitis	NCT03368430	2017
Randomized phase 2 trial of anti-tumor necrosis factor therapy for cachexia in patients with early rheumatoid arthritis	Marcora, , SM, Chester, KR, Mittal, G, Lemmey, AB; Maddison, PJ	2006
Long-term safety of intravenous ibandronic acid for up to 4 years in metastatic breast cancer: an open-label trial	Pecherstorfer, , M, Rivkin, S, Body, JJ, Diel, I; Bergström, B	2006
Sequential intravenous high dose oral antibiotics in the treatment of osteoarticular infections in children: a randomized controlled trial	Tsui, , K, Crawford, H, Mow, FC, Webb, R, Voss, LM, Stott, NS, Stewart, J; Lennon, DR	2016
Decortication With Calcium Phosphosilicate Putty in the Treatment of Non-contained Intrabony Periodontal Defects	NCT03435757	2018
Effects of telmisartan on inflammatory cytokines and coronary plaque component as assessed on integrated backscatter intravascular ultrasound in hypertensive patients	Yamaguchi, , K, Wakatsuki, T, Soeki, T, Niki, T, Taketani, Y, Oeduka, H, Kusunose, K, Ise, T, Iwase, T, Yamada, H; Sata, M	2014
Evaluation of Platelet Rich Fibrin Matrix as Regenerative Material in Intraosseous Defects	NCT03616925	2018
Effect of percutaneous vertebroplasty and percutaneous kyphoplasty on adjacent intervertebral disc degeneration and mechanism	Tian, , L, Meng, C-Y; Xu, X-M	2014
Etiological periodontal treatment with and without low-level laser therapy on IL-1β level in gingival crevicular fluid: an in vivo multicentric pilot study	Mastrangelo, , F, Dedola, A, Cattoni, F, Ferrini, F, Bova, F, Tatullo, M, Gherlone, E; Lo Muzio, L	2018
PTH (1-84) replacement therapy in hypoparathyroidism: effects on bone metabolism and structure	Sikjaer, , T, Rejnmark, L, Bruel, A, Thomsen, J; Mosekilde, L	2011
Peri-Implantitis Surgical Treatment an RCT Study	NCT02575274	2015
Immunomodulatory effect of the topical ophthalmic Janus kinase inhibitor tofacitinib (CP-690,550) in patients with dry eye disease	Huang, , JF, Yafawi, R, Zhang, M, McDowell, M, Rittenhouse, KD, Sace, F, Liew, SH, Cooper, SR; Pickering, EH	2012

Efficacy of risedronate with cholecalciferol on 25-hydroxyvitamin D level and bone turnover in Korean patients with osteoporosis	Chung, , HY, Chin, SO, Kang, MI, Koh, JM, Moon, SH, Yoon, BK, Yoon, HK, Chung, YS; Park, HM	2011
Peripheral blood circulating multiple myeloma cells (CMMCs) correlate with disease burden and can be used to characterize high-risk cytogenetics in newly diagnosed and smoldering myeloma	Foulk, , B, Schaffer, M, Gross, S, Rao, C, Smirnov, D, Chaturvedi, S, Reddy, M, Repollet, M, Rojas, C, Auclair, D, DeRome, M, Weiss, B; Sasser, AK	2016
Thrombospondin-1 is a plasmatic marker of peripheral arterial disease that modulates endothelial progenitor cell angiogenic properties	Smadja, , DM, d'Audigier, C, Bièche, I, Evrard, S, Mauge, L, Dias, JV, Labreuche, J, Laurendeau, I, Marsac, B, Dizier, B, Wagner-Ballon, O, Boisson-Vidal, C, Morandi, V, Duong-Van-Huyen, JP, Bruneval, P, Dignat-George, F, Emmerich, J; Gaussem, P	2011
Gingival crevicular fluid matrix metalloproteinase-8 levels following adjunctive use of meloxicam and initial phase of periodontal therapy	Buduneli, , N, Vardar, S, Atilla, G, Sorsa, T, Luoto, H; Baylas, H	2002
Improving Community Ambulation After Hip Fracture	NCT01783704	2013
MELABLOCK: a Clinical Trial on the Efficacy and Safety of Propranolol 80 mg in Melanoma Patients	NCT02962947	2016
Local bone treatment in osteoporosis	Kurth, A	2018
Subantimicrobial dose doxycycline efficacy as a matrix metalloproteinase inhibitor in chronic periodontitis patients is enhanced when combined with a non-steroidal anti-inflammatory drug	Lee, , HM, Ciancio, SG, Tüter, G, Ryan, ME, Komaroff, E; Golub, LM	2004
High-intensity Training in an Enriched Environment in Late Phase After Stroke	NCT02889939	2016
Efficacy of low doses of pamidronate in osteopenic patients administered in the early post-renal transplant	Torregrosa, , JV, Fuster, D, Monegal, A, Gentil, MA, Bravo, J, Guirado, L, Muxí, A; Cubero, J	2011
A Trial of Tadalafil in Interstitial Lung Disease of Scleroderma	NCT01553981	2012
Dietary Supplements and Periodontal Wound Healing	NCT02315222	2014
The effects of denosumab and alendronate on glucocorticoid-induced osteoporosis in patients with glomerular disease: a randomized, controlled trial	Iseri, , K, Iyoda, M, Watanabe, M, Matsumoto, K, Sanada, D, Inoue, T, Tachibana, S; Shibata, T	2018
Infusions of interleukin-1 alpha after autologous transplantation for Hodgkin's disease and non-Hodgkin's lymphoma induce effector cells with antilymphoma cytolytic activity	Katsanis, , E, Weisdorf, DJ, Xu, Z, Dancisak, BB, Halet, ML; Blazar, BR	1994
Safety and effectiveness profile of raloxifene in long-term, prospective, postmarketing surveillance	Iikuni, , N, Hamaya, E, Nihojima, S, Yokoyama, S, Goto, W, Taketsuna, M, Miyauchi, A; Sowa, H	2012
MIV-711, a highly selective cathepsin K inhibitor, reduces biomarkers of bone resorption and cartilage degradation in healthy subjects	Lindstrom, , E, Grabowska, U, Jerling, M; Edenius, C	2014
Effect of danhong injection on the mobilisation of endothelial progenitor cells to vascular repair after percutaneous coronary intervention: a randomised controlled trial	Hu, , Z, Wang, H, Fan, G, Zhang, H, Wang, X, Mao, J, Zhao, Y, An, Y, Huang, Y, Li, C, Chang, L, Chu, X, Li, L, Li, Y, Zhang, Y, Qin, G, Gao, X; Zhang, B	2016
CTX biochemical marker of bone metabolism. Is it a reliable predictor of bisphosphonate-associated osteonecrosis of the jaws after surgery? Part II: a prospective clinical study	Lee, , CY; Suzuki, JB	2010
Clinical response of azithromycin as an adjunct to non-surgical periodontal therapy in smokers	Mascarenhas, , P, Gapski, R, Al-Shammari, K, Hill, R, Soehren, S, Fenno, JC, Giannobile, WV; Wang, HL	2005
Interferon-α/β receptor as a prognostic marker in osteosarcoma	Kubo, , T, Shimose, S, Matsuo, T, Fujimori, J, Arihiro, K; Ochi, M	2011
Treatment of newly diagnosed multiple myeloma: goals and options	Tacchetti, , P, Zamagni, E, Pantani, L; Cavo, M	2017
Surgical techniques, open versus minimally invasive gastrectomy after chemotherapy (STOMACH trial): study protocol for a randomized controlled trial	Straatman, , J, van der Wielen, N, Cuesta, MA, Gisbertz, SS, Hartemink, KJ, Alonso Poza, A, Weitz, J, Mateo Vallejo, F, Ahktar, K, Diez Del Val, I, Roig Garcia, J; van der Peet, DL	2015
Osteoclast inhibitory effects of vitamin K2 alone or in combination with etidronate or risedronate in patients with rheumatoid arthritis: 2-year results	Morishita, , M, Nagashima, M, Wauke, K, Takahashi, H; Takenouchi, K	2008
Growth hormone favorably affects bone turnover and bone mineral density in patients with short bowel syndrome undergoing intestinal rehabilitation	Tangpricha, , V, Luo, M, Fernández-Estívariz, C, Gu, LH, Bazargan, N, Klapproth, JM, Sitaraman, SV, Galloway, JR, Leader, LM; Ziegler, TR	2006
Effect of a short-term treatment with alendronate on bone density and bone markers in patients with central diabetes insipidus	Pivonello, , R, Faggiano, A, Di Somma, C, Klain, M, Filippella, M, Salvatore, M, Lombardi, G; Colao, A	1999
Interleukin-18 has antipermeablity and antiangiogenic activities in the eye: reciprocal suppression with VEGF	Shen, , J, Choy, DF, Yoshida, T, Iwase, T, Hafiz, G, Xie, B, Hackett, SF, Arron, JR; Campochiaro, PA	2014
Endothelial progenitor cells in subclinical hypothyroidism: the effect of thyroid hormone replacement therapy	Shakoor, , SK, Aldibbiat, A, Ingoe, LE, Campbell, SC, Sibal, L, Shaw, J, Home, PD, Razvi, S; Weaver, JU	2010
A clinical, radiological and IL-6 evaluation of subgingivally delivered simvastatin in the treatment of chronic periodontitis	Rath, , A, Mahenra, J, Thomas, L, Sandhu, M, Namasi, A; Ramakrishna, T	2012
Effects of antioxidant supplements intervention on the level of plasma inflammatory molecules and disease severity of rheumatoid arthritis patients	Bae, , SC, Jung, WJ, Lee, EJ, Yu, R; Sung, MK	2009
Two-level Coflex interlaminar stabilization compared to two-level lumbar spinal fusion for the treatment of spinal stenosis with low-grade spondylolisthesis	Auerbach, , JD; Field, JS	2013
Early application of nerve growth factor affects serum inflammatory cytokine levels in neonatal hypoxic ischemic encephalopathy	Huang, , H, Zheng, K, Xia, R, Chen, J, Yang, X, Ye, C; Lin, Q	2007
Effect of Intensive Periodontal Therapy on Vascular Function	NCT00327561	2006
Open-label randomized parallel controlled study comparing bone mineral density between alendronate plus alfacalcidol combination and	Saito, , M; Matsuoka, J	2015

single administration of alfacalcidol in postmenopausal women receiving aromatase inhibitor as adjuvant therapy		
The efficacy of host response modulation therapy (omega-3 plus low-dose aspirin) as an adjunctive treatment of chronic periodontitis (clinical and biochemical study)	Elkhouli, AM	2011
PTEN as a prognostic and predictive marker in postoperative radiotherapy for squamous cell cancer of the head and neck	Snietura, , M, Jaworska, M, Mlynarczyk-Liszka, J, Goraj-Zajac, A, Piglowski, W, Lange, D, Wozniak, G, Nowara, E; Suwinski, R	2012
Evaluation of treadmill exercise in a lower body negative pressure chamber as a countermeasure for weightlessness-induced bone loss: a bed rest study with identical twins	Smith, , SM, Davis-Street, JE, Fesperman, JV, Calkins, DS, Bawa, M, Macias, BR, Meyer, RS; Hargens, AR	2003
Effect of high-protein meals during hemodialysis combined with lanthanum carbonate in hypoalbuminemic dialysis patients: findings from the FrEDI randomized controlled trial	Rhee, , CM, You, AS, Koontz Parsons, T, Tortorici, AR, Bross, R, St-Jules, DE, Jing, J, Lee, ML, Benner, D, Kovesdy, CP, Mehrotra, R, Kopple, JD; Kalantar-Zadeh, K	2017
Biochemical markers of bone turnover associated with calcium supplementation in children with juvenile rheumatoid arthritis: results of a double-blind, placebo-controlled intervention trial	Carrasco, , R, Lovell, DJ, Giannini, EH, Henderson, CJ, Huang, B, Kramer, S, Ranz, J, Heubi, J; Glass, D	2008
Chemerin and IL-6 Levels in Diabetes and Periodontitis	NCT02596581	2015
Treatment of poor-risk myelodysplastic syndromes and acute myeloid leukemia with a combination of 5-azacytidine and valproic acid	Kuendgen, , A, Bug, G, Ottmann, OG, Haase, D, Schanz, J, Hildebrandt, B, Nachtkamp, K, Neukirchen, J, Dienst, A, Haas, R, Germing, U; Gattermann, N	2011
Effect of Periodontitis on Bone Mineral Density in Postmenopausal Women	NCT02628197	2015
The Intraocular Cytokine Profile and Therapeutic Response in Persistent Neovascular Age-Related Macular Degeneration	Rezar-Dreindl, , S, Sacu, S, Eibenberger, K, Pollreisz, A, Bühl, W, Georgopoulos, M, Krall, C, Weigert, G; Schmidt-Erfurth, U	2016
The effect of leptin replacement on parathyroid hormone, RANKL-osteoprotegerin axis, and Wnt inhibitors in young women with hypothalamic amenorrhea	Foo, , JP, Polyzos, SA, Anastasilakis, AD, Chou, S; Mantzoros, CS	2014
Comparison of the Effect of Teflon vs Non-Teflon Hand Scalers in the Maintenance of Peri-Implant Tissue	NCT03316937	2017
Novel biomarkers to detect infection in revision hip and knee arthroplasties infection	Glehr, , M, Friesenbichler, J, Hofmann, G, Bernhardt, GA, Zacherl, M, Avian, A, Windhager, R; Leithner, A	2013
Non-surgical Periodontal Therapy and Myo-inositol in Polycystic Ovary Syndrome Women Having Chronic Periodontitis	NCT02633462	2015
Changes in bone turnover and in bone mass in women with breast cancer switched from tamoxifen to exemestane	Gonnelli, , S, Cadirni, A, Caffarelli, C, Petrioli, R, Montagnani, A, Franci, MB, Lucani, B, Francini, G; Nuti, R	2007
Comparison of Nd: YAG laser versus scaling and root planing in periodontal therapy	Liu, , CM, Hou, LT, Wong, MY; Lan, WH	1999
Perforated Collagen Membrane With Nanohydroxyapatite for Intrabony Defects	NCT03399279	2018
Efficacy and Safety of ABT-494, a Selective JAK-1 Inhibitor, in a Phase IIb Study in Patients With Rheumatoid Arthritis and an Inadequate Response to Methotrexate	Genovese, , MC, Smolen, JS, Weinblatt, ME, Burmester, GR, Meerwein, S, Camp, HS, Wang, L, Othman, AA, Khan, N, Pangan, AL; Jungerwirth, S	2016
Growth hormone treatment during hemodialysis in a randomized trial improves nutrition, quality of life, and cardiovascular risk	Feldt-Rasmussen, , B, Lange, M, Sulowicz, W, Gafter, U, Lai, KN, Wiedemann, J, Christiansen, JS; El Nahas, M	2007
Clinical outcomes and fusion rates following anterior lumbar interbody fusion with bone graft substitute i-FACTOR, an anorganic bone matrix/P-15 composite	Mobbs, , RJ, Maharaj, M; Rao, PJ	2014
Effects of fermentable high fiber diet supplementation on gut derived and conventional nitrogenous product in patients on maintenance hemodialysis: a randomized controlled trial	Khosroshahi, , HT, Abedi, B, Ghojazadeh, M, Samadi, A; Jouyban, A	2019
The Effect of Different Dental Implant Surface Characteristics on Immunological and Microbiological Parameters	NCT03693196	2018
Heterotopic ossification following single-level anterior cervical discectomy and fusion: results from the prospective, multicenter, historically controlled trial comparing allograft to an optimized dose of rhBMP-2	Arnold, , PM, Anderson, KK, Selim, A, Dryer, RF; Kenneth Burkus, J	2016
Long-term treatment of 12 children with chronic hypoparathyroidism: a randomized trial comparing synthetic human parathyroid hormone 1-34 versus calcitriol and calcium	Winer, , KK, Sinaii, N, Reynolds, J, Peterson, D, Dowdy, K; Cutler, GB	2010
Preoperative prediction of cancer-specific mortality after nephroureterectomy for patients with upper urinary tract urothelial carcinoma: preoperative multivariate model incorporating C-reactive protein	Saito, , K, Ishioka, J, Fujii, Y, Matsuoka, Y, Numao, N, Koga, F, Ohtsuka, Y, Arisawa, C, Kamata, S, Nagahama, K, Morimoto, S, Tsujii, T, Kitahara, S, Noro, A, Goto, S, Kageyama, Y, Yonese, J; Kihara, K	2014
Testosterone in Bariatric Patients	NCT03721497	2018
A prospective, open-label, single center, cross-over, controlled, randomized, phase III study to test superiority of the oral solution of synthetic thyroxine compared to tablet preparation for treatment of hypothyrod patients under chronic gastric pump inhibitors treatment	EUCTR2013-000759-41-IT	2013
Effects of raloxifene on bone metabolism in hemodialysis patients with type 2 diabetes	Saito, , O, Saito, T, Asakura, S, Akimoto, T, Inoue, M, Ando, Y, Muto, S; Kusano, E	2012
Quality of Life in Motion: a combined physical exercise and psychosocial training program to improve physical fitness in children with cancer	NTR1531	2008
Improvement of glycated hemoglobin in Japanese subjects with type 2 diabetes by resolution of periodontal inflammation using adjunct topical antibiotics: results from the Hiroshima Study	Munenaga, , Y, Yamashina, T, Tanaka, J; Nishimura, F	2013

Local antibiotic therapy in peri- implant inflammation	IRCT2013100114847N1	2014
The short-term effects of low-level lasers as adjunct therapy in the treatment of periodontal inflammation	Qadri, , T, Miranda, L, Tunér, J; Gustafsson, A	2005
Switch from tenofovir/emtricitabine to raltegravir in female HIV-positive patients taking atazanavir/ritonavir evaluating bone mineral density improvement after one year	EUCTR2013-002349-12-IT	2013
Clinical significance of increased serum levels of FGF23 in fibrous dysplasia	Florez, , H, Mandelikova, S, Filella, X, Monegal, A, Guanabens, N; Peris, P	2017
A clinical study (phase 2) in hereditary Rickets (XLH) in children to assess the safety and working of the antibody anti-FGF23	EUCTR2014-000406-35-FR	2015
Feasibility and Efficacy of Interval Walking in Patients With Colorectal Cancer	NCT02403024	2015
clinical trial to evaluate the efficacy in the infusion with mononuclear autologous bone marrow stem cells in patients with ischemic stroke	EUCTR2013-002135-15-ES	2014
IL-1 in gingival crevicular fluid following closed root planing and papillary flap debridement	Reinhardt, , RA, Masada, MP, Johnson, GK, DuBois, LM, Seymour, GJ; Allison, AC	1993
A trial to determine the how safe and effective the test drug is for obese males with hypogonadotropic hypogonadism. The trial is blinded and the subjects may be given the test drug or a placebo	EUCTR2015-005760-42-GB	2016
Evaluation of treatment for isolated bilateral miller's class i or II gingival recession with platelet rich fibrin membrane- A comparative study	Shivakumar, , MA, Gopal, SV, Govindaraju, P, Ramayya, S, Bennadi, D; Mruthyuenjaya, RK	2016
Evaluation of peri-implant mucositis at microgrooved and machined abutments: a two-center study	DRKS00009394	2015
Osteoporosis and bone metabolism in systemic sclerosis	Horvath, , A, Gulyas, K, Bhattoa, HP, Szucs, G, Szekanecz, Z; Szamosi, S	2017
A Phase 2, Multi-center, Randomized, Double-blind, Placebo-controlled, Multiple-dose Study to Determine the Safety and Efficacy of Daily Orally Administered LX3305 in Subjects with Active Rheumatoid Arthritis (RA) on Stable Methotrexate (MTX) Therapy - LX3305.201	EUCTR2009-012705-19-BG	2009
Denosumab increases sublesional bone mass in osteoporotic patients with recent spinal cord injury	Vidal, , J, Carrasco, JL, Muxi, A, Portell, E, Monegal, A, Guanabens, N; Peris, P	2015
Leptin is an effective treatment for hypothalamic amenorrhea	Chou, , SH, Chamberland, JP, Liu, X, Matarese, G, Gao, C, Stefanakis, R, Brinkoetter, MT, Gong, H, Arampatzi, K; Mantzoros, CS	2011
Influence of modified transdermal hormone replacement therapy on the concentrations of hormones, growth factors, and bone mineral density in women with osteopenia	Stanosz, , S, Zochowska, E, Safranow, K, Sieja, K; Stanosz, M	2009
BENEFIT study	NTR1274	2008
Investigating the role of targeted therapy Sorafenib - the Fms-like tyrosine kinase 3 (FLT3) inhibitor, in combination with intensive chemotherapy, for previously untreated adult patients with Acute Myeloid Leukaemia (AML) with FLT3 mutations. A Phase II randomised placebo-controlled multi-centre study	ACTRN12611001112954	2011
A trial comparing continuous subcutaneous hydrocortisone therapy with conventional oral glucocorticoid therapy in congenital adrenal hyperplasia	EUCTR2011-005822-23-NO	2013
Cabozantinib (XL184) in patients with metastatic breast cancer: results from a phase 2 randomized discontinuation trial	Tolaney, , SM, Nechushtan, H, Berger, R, Kurzrock, R, Ron, IG, Schoffski, P, Awada, A, Yasenchak, CA, Burris, HA, Ramies, DA, Shen, X; Winer, EP	2011
Efficacy, safety, and confirmation of the recommended phase 2 starting dose of the combination of ruxolitinib (RUX) and panobinostat (PAN) in patients (pts) with myelofibrosis (MF)	Harrison, , CN, Kiladjian, J-J, Heidel, FH, Vannucchi, AM, Passamonti, F, Hayat, A, Conneally, E, Martino, B, Kindler, T, Lipka, DB, Acharyya, S, Gopalakrishna, P, Ide, S, Liu, T, Mu, S; Ribrag, V	2015
A randomized Phase II, open label multicenter cross-over study, to evaluate biomarkers, in 2nd line treatment of metastatic Castration Resistant Prostate Cancer (mCRPC) with abiraterone and cabazitaxel	EUCTR2015-000270-36-SE	2015
Randomized, double-blind, placebo controlled Phase II study to evaluate the efficacy and safety of Sorafenib treatment in patients with advanced (recurrent, persistent and/or metastasizing) medullary thyroid carcinoma (SUMMIT)	DRKS00004455	2012
Comparative effect of nimesulide and ibuprofen on the urinary levels of collagen type II C-telopeptide degradation products and on the serum levels of hyaluronan and matrix metalloproteinases-3 and -13 in patients with flare-up of osteoarthritis	Manicourt, , DH, Bevilacqua, M, Righini, V, Famaey, JP; Devogelaer, JP	2005
Comparative analysis in implant-supported metal ceramic and hibrid ceramic crown	RBR-9w9m83	2018
What is the clinical relevance of radiographic nonunion after single-level lumbar interbody arthrodesis in degenerative disc disease?: a meta-analysis of the YODA project database	Noshchenko, , A, Lindley, EM, Burger, EL, Cain, CMJ; Patel, VV	2016
A study to examine the safety, tolerability and effects on abnormal bone formation of REGN2477 in patients with Fibrodysplasia Ossificans Progressiva	EUCTR2016-005035-33-IT	2018
Effect of Nonsurgical Periodontal Therapy Verses Oral Hygiene Instructions on Patients With Chronic Periodontitis	NCT02208739	2014
Platelet concentrates derived from own blood for the treatment of jaw bone defect caused by gum disease	CTRI/2018/12/016566	2018

Changes in bone turnover after parathyroidectomy in dialysis patients: role of calcitriol administration	Mazzaferro, , S, Chicca, S, Pasquali, M, Zaraca, F, Ballanti, P, Taggi, F, Coen, G, Cinotti, GA; Carboni, M	2000
Platelet rich fibrin in periodontal surgery	ISRCTN13520922	2018
Dendritic cell-based immunotherapy targeting the tumor protein WT1 to treat adult patients with acute myeloid leukemia	EUCTR2012-001494-91-BE	2012
Laser-assisted delivery of vitamin C, vitamin E, and ferulic acid formula serum decreases fractional laser postoperative recovery by increased beta fibroblast growth factor expression	Waibel, , JS, Mi, QS, Ozog, D, Qu, L, Zhou, L, Rudnick, A, Al-Niaimi, F, Woodward, J, Campos, V; Mordon, S	2016
A PROSPECTIVE, PILOT, CROSS-OVER STUDY TO ASSESS THE EFFICACY OF PARICALCITOL IN REDUCING PARATHYROID HORMONE LEVELS AND AMELIORATING MARKERS OF BONE REMODELLING IN RENAL TRANSPLANT RECIPIENTS WITH SECONDARY HYPERPARATHYROIDISM (APPLE STUDY) - ND	EUCTR2008-006380-36-IT	2008
Meditation interventions for treatment of PTSD in veterans	Lim, , K, Erbes, C, Thuras, P, Rodman, J, Sponheim, S; Polusny, M	2014
A study of the Combination of Ibrutinib plus Venetoclax versus Chlorambucil plus Obinutuzumab for the First-line Treatment of Patients with Chronic Lymphocytic Leukemia (CLL)/Small Lymphocytic Lymphoma (SLL)	EUCTR2017-004699-77-PL	2018
The effect of recombinant human bone morphogenetic protein-2 in single-level posterior lumbar interbody arthrodesis	Chutkan, NB	2013
Ketogenic diets as an adjuvant therapy in glioblastoma (the KEATING trial)	ISRCTN71665562	2017
Doxycycline speeds up healing of chronic venous ulcers	Serra, , R, Gallelli, L, Buffone, G, Molinari, V, Stillitano, DM, Palmieri, C; de Franciscis, S	2015
Therapeutic efficacy of magnesium oxide for peripheral artery disease among patients undergoing hemodialysis - Open label randomized controlled trial	JPRN-UMIN000024275	2016
A preliminary trial of the effect of recombinant human growth hormone on short-term linear growth and glucose homeostasis in children with Crohn's disease	Wong, , SC, Kumar, P, Galloway, PJ, Blair, JC, Didi, M, Dalzell, AM, Hassan, K, McGrogan, P; Ahmed, SF	2011
A Phase 2, Open Label, Randomized, Dose Ranging, Safety, Efficacy, Pharmacokinetic and Pharmacodynamic Study of AG-348 in Adult Patients with Pyruvate Kinase Deficiency	EUCTR2015-000484-13-GB	2015
Effect of recent spinal cord injury on the OPG/RANKL system and its relationship with bone loss and antiosteoporotic response to denosumab therapy	Gifre, , L, Vidal, J, Ruiz-Gaspa, S, Portell, E, Monegal, A, Muxi, A, Guanabens, N; Peris, P	2017
Statin- and bisphosphonate treatment in patients with the Philadelphia-negative chronic myeloproliferative neoplasms - essential thrombocytosis, polycythemia vera and hypercellular myelofibrosis	EUCTR2016-001406-42-DK	2016
Salivary biomarkers of periodontal disease in response to treatment	Sexton, , WM, Lin, Y, Kryscio, RJ, Dawson, DR, Ebersole, JL; Miller, CS	2011
Randomized double-blind placebo controlled Phase II study to evaluate the efficacy and safety of Sorafenib treatment in patients with advanced (recurrent, persistent and/or metastasizing) medullary thyroid carcinoma (SUMMIT)	EUCTR2011-006250-90-DE	2012
TBCRC 008: early change in 18F-FDG uptake on PET predicts response to preoperative systemic therapy in human epidermal growth factor receptor 2-negative primary operable breast cancer	Connolly, , RM, Leal, JP, Goetz, MP, Zhang, Z, Zhou, XC, Jacobs, LK, Mhlanga, J, O, JH, Carpenter, J, Storniolo, AM, Watkins, S, Fetting, JH, Miller, RS, Sideras, K, Jeter, SC, Walsh, B, Powers, P, Zorzi, J, Boughey, JC, Davidson, NE, Carey, LA, Wolff, AC, Khouri, N, Gabrielson, E, Wahl, RL; Stearns, V	2015
Effects of exercise on Sexual function and CArdiovascular health in men with prostate cancer (ESCA) Study	ACTRN12619000143123	2019
Immunological predictors of overall survival in treatment naive metastatic pancreatic cancer patients	Farren, , MR, Mace, T, Geyer, S, Mikhail, S, Wu, C, Ciombor, K, Tahiri, S, Ahn, D, Noonan, A, Villalona-Calero, M, Bekaii-Saab, T; Lesinski, G	2015
A PHASE I/II, OPEN LABEL STUDY OF SPC2996 IN COMBINATION WITH RITUXIMAB FOR THE TREATMENT OF RELAPSED FOLLICULAR OR LYMPHOPLASMACYTIC NON-HODGKIN'S LYMPHOMA	EUCTR2008-001353-17-HU	2008
Bone metabolism changes during anti-TNF-alpha therapy in patients with active rheumatoid arthritis	Seriolo, , B, Paolino, S, Sulli, A, Ferretti, V; Cutolo, M	2006
A randomised, open label, prospective study to assess two different therapeutic strategies following first treatment failure in HIV-1 infected subjects. 'The First Failure Study' : 'FAST' - The First Failure Study	EUCTR2009-011816-39-GB	2010
Postoperative radiotherapy after radical prostatectomy: a randomised controlled trial (EORTC trial 22911)	Bolla, , M, van Poppel, H, Collette, L, van Cangh, P, Vekemans, K, Da Pozzo, L, de Reijke, TM, Verbaeys, A, Bosset, JF, van Velthoven, R, Maréchal, JM, Scalliet, P, Haustermans, K; Piérart, M	2005
Application of laser assisted anti-infection therapy for the gum disease around dental implants	IRCT201609281248N3	2016
Interleukin-3 in vivo: kinetic of response of target cells	Aglietta, , M, Sanavio, F, Stacchini, A, Morelli, S, Fubini, L, Severino, A, Pasquino, P, Volta, C, Bretti, S; Tafuto, S	1993
Constitutional Delay of Growth and Puberty: towards evidence-based treatment	EUCTR2012-002477-59-FI	2012

Dynamic contrast-enhanced magnetic resonance imaging as a surrogate biomarker for bevacizumab in colorectal cancer liver metastasis: a single-arm, exploratory trial	Kim, , Y-E, Joo, B, Park, M-S, Shin, SJ, Ahn, JB; Kim, M-J	2016
Treatment with Long Acting hGH Product in Adult subjects with Growth Hormone Deficiency	EUCTR2013-000830-37-AT	2013
The influence of denosumab on the immune system in women after the menopause with HER2 negative breastcancer	EUCTR2016-005210-22-NL	2017
Effects of laser moxibustion on patients with knee osteoarthritis	ISRCTN15030019	2017
Changes in inflammation and bone turnover markers after treatment for periodontal disease in patients with diabetes	Izuora, , KE, Ezeanolue, E, Neubauer, M, Gewelber, C, Allenback, G, Shan, G; Umpierrez, GE	2015
Clinical trial to investigate efficacy and safety of FE 203799 (GLP-2 analogue) or placebo administered as subcutaneous injections once weekly in two treatment periods to patients with short bowel syndrome with intestinal failure	EUCTR2017-002486-21-DK	2017
Singapore Health and Biomedical Congress, SHBC 2013		2013
Study of XL184 in subjects with advanced solid tumors	EUCTR2009-012964-14-BE	2009
Evaluation of the usefulness of suction drainage in the treatment of nonspecific bone infections monitored with C-reactive protein	Milankov, , M, Jovanović, A, Milicić, A, Somer, T, Vukadinović, S; Savić, D	1999
Down's syndrome in children males – biochemical characterisations in different media using non-randomised trial and systematic study	DRKS00014074	2018
How Bone is Made in Children Receiving Dialysis	NCT01799317	2013
suPERficial slow-flow vascular malFORMations treated with sirolimUS	EUCTR2015-001096-43-FR	2015
A phase 2, randomized, placebo-controlled study evaluating matrix metalloproteinase-9 inhibitor, andecaliximab, in patients with moderately to severely active Crohn's disease	Schreiber, , S, Siegel, CA, Friedenberg, KA, Younes, ZH, Seidler, U, Bhandari, BR, Wang, K, Wendt, E, McKevitt, M, Zhao, S, Sundy, JS, Lee, SD; Loftus, EV	2018
Effect of testosterone in male patients on regular hemodialysis	EUCTR2011-005439-20-SE	2011
Translational Biomarkers and Ex Vivo Models of Joint Tissues as a Tool for Drug Development in Rheumatoid Arthritis	Kjelgaard-Petersen, , CF, Platt, A, Braddock, M, Jenkins, MA, Musa, K, Graham, E, Gantzel, T, Slynn, G, Weinblatt, ME, Karsdal, MA, Thudium, CS; Bay-Jensen, A-C	2018
The healing of pressure ulcers treated with electrical stimulation delivered by the cathode versus the alternating use of the cathode and anode	ACTRN12614000992606	2014
Improved treatment of mandibular odontogenic cysts with platelet-rich gel	Cieslik-Bielecka, , A, Bielecki, T, Gazdzik, TS, Cieslik, T; Szczepanski, T	2008
BA058 Transdermal Phase 2 Study in Women with Osteoporosis	EUCTR2012-001921-29-DK	2012
Quantification of surgical trauma: comparison of posterolateral surgical approach and anterior minimally invasive (AMIS) for total hip arthroplasty based on markers of inflammation (interleukins). Preliminary report		2014
Evaluation of pneumococcal conjugate vaccine (Prevenar) in patients with myeloma and chronic lymphocytic leukaemia	ISRCTN21541376	2006
Bioidentical 'Natural' Hormone Evaluation in Early Menopause	NCT00302731	2006
A Randomized Trial of Bortezomib in Late Antibody-Mediated Kidney Transplant Rejection	Eskandary, , F, Regele, H, Baumann, L, Bond, G, Kozakowski, N, Wahrmann, M, Hidalgo, LG, Haslacher, H, Kaltenecker, CC, Aretin, MB, Oberbauer, R, Posch, M, Staudenherz, A, Handisurya, A, Reeve, J, Halloran, PF; Böhmig, GA	2018
Adjunctive treatment of chronic periodontitis with daily dietary supplementation with omega-3 Fatty acids and low-dose aspirin	El-Sharkawy, , H, Aboelsaad, N, Eliwa, M, Darweesh, M, Alshahat, M, Kantarci, A, Hasturk, H; Van Dyke, TE	2010
Molecular profiling of postmenopausal women with breast cancer on neoadjuvant exemestane or tamoxifen	EUCTR2005-001698-89-GB	2006
Nivolumab for pediatric and adult relapsing/refractory ALK+ anaplastic large cell lymphoma in patients with progressive disease or in patients in complete remission after relapse	EUCTR2018-001447-31-FR	2018
Changes in fibroblast growth factor 23 during treatment of secondary hyperparathyroidism with alfacalcidol or paricalcitol	Hansen, , D, Rasmussen, K, Pedersen, SM, Rasmussen, LM; Brandi, L	2012
Relationship of herpes virus with interleukin in gum diseases	CTRI/2018/07/015141	2018
Clinical outcomes and fusion success at 2 years of single-level instrumented posterolateral fusions with recombinant human bone morphogenetic protein-2/compression resistant matrix versus iliac crest bone graft	Dimar, , JR, Glassman, SD, Burkus, KJ; Carreon, LY	2006
Comparative Study of Maxillary Bone Preservation after Dental Extraction with Different Biomaterials	RBR-29nbjw	2018
Effect of rhPDGF-BB delivery on mediators of periodontal wound repair	Cooke, , JW, Sarment, DP, Whitesman, LA, Miller, SE, Jin, Q, Lynch, SE; Giannobile, WV	2006
The influence of cathodal and anodal electrical stimulation on skin blood flow and the healing of pressure ulcers	ACTRN12615001281583	2015
Extended-release calcifediol (ERC) improves bone marker levels in CKD patients with diabetes		2018
A clinical trial of allogeneic mesenchymal precursor cells (MPCs) in the treatment of heart attacks	EUCTR2010-020497-41-ES	2014

Effect of dietary supplementation with collagen hydrolysates on bone metabolism of postmenopausal women with low mineral density	Cúneo, , F, Costa-Paiva, L, Pinto-Neto, AM, Morais, SS; Amaya-Farfan, J	2010
A pilot 24-week open-label, randomized, controlled clinical trial to assess the safety, tolerability and efficacy of dual therapy with Raltegravir/Lamivudine combination when replacing standard combination therapy in HIV-infected patients with prolonged virological suppression. RALAM Study	EUCTR2014-003142-27-ES	2015
International collaborative treatment protocol for children and adolescents with acute lymphoblastic leukemia	EUCTR2007-004270-43-DE	2009
The effects of denosumab and alendronate on glucocorticoid-induced osteoporosis in patients with glomerular disease: a randomized, controlled trial		2018
Effect of cathodal and anodal electrical stimulation on pressure ulcer healing	ACTRN12616001709437	2016
A study of the Combination of Ibrutinib plus Venetoclax versus Chlorambucil plus Obinutuzumab for the First-line Treatment of Patients with Chronic Lymphocytic Leukemia (CLL)/Small Lymphocytic Lymphoma (SLL)	EUCTR2017-004699-77-NL	2018
Cinacalcet hydrochloride reduces the serum calcium concentration in inoperable parathyroid carcinoma	Silverberg, , SJ, Rubin, MR, Faiman, C, Peacock, M, Shoback, DM, Smallridge, RC, Schwanauer, LE, Olson, KA, Klassen, P; Bilezikian, JP	2007
A Study to Assess the efficacy and the safety of Vamorolone in Boys with Duchenne Muscular Dystrophy (DMD)	EUCTR2017-002704-27-SE	2018
A study investigating the use of additional chemotherapy for the improvement of efficacy, and the selection of breast cancer patients for such treatment in two paitent cohorts: before surgery in curable patients and at time of metastatic disease	EUCTR2013-004418-17-NO	2014
Effects of prasterone (dehydroepiandrosterone) on markers of cardiovascular risk and bone turnover in premenopausal women with systemic lupus erythematosus: a pilot study	Marder, , W, Somers, EC, Kaplan, MJ, Anderson, MR, Lewis, EE; McCune, WJ	2010
The effect of plasma rich in growth factors in treatment of gingival recession	IRCT201201168747N1	2012
Peri-interventional endothelin–a receptor blockade improves long-term outcome in patients with ST-elevation acute myocardial infarction	Adlbrecht, , C, Wurm, R, Humenberger, M, Andreas, M, Redwan, B, Distelmaier, K, Klappacher, G; Lang, IM	2014
Weight loss and bone health in postmenopausal breast cancer survivors	Toriola, , AT, Liu, J, Ganz, PA, Colditz, GA, Yang, L, Izadi, S, Schwartz, AL; Wolin, KY	2015
Changes in bone mineral density in women following 1-year gastric bypass surgery	Casagrande, , DS, Repetto, G, Mottin, CC, Shah, J, Pietrobon, R, Worni, M; Schaan, BD	2012
Melatonin-micronutrients Osteopenia Treatment Study (MOTS): a translational study assessing melatonin, strontium (citrate), vitamin D3 and vitamin K2 (MK7) on bone density, bone marker turnover and health related quality of life in postmenopausal osteopenic women following a one-year double-blind RCT and on osteoblast-osteoclast co-cultures		2017
The Immune and Clinical Impacts of Vitamin D in Patients With Chronic Musculo-skeletal Pain	NCT01417923	2011
Effects of sub-antimicrobial dose doxycycline therapy on crevicular fluid MMP-8, and gingival tissue MMP-9, TIMP-1 and IL-6 levels in chronic periodontitis	Choi, , DH, Moon, IS, Choi, BK, Paik, JW, Kim, YS, Choi, SH; Kim, CK	2004
Randomized controlled trial of pericardial blood processing with a cell-saving device on neurologic markers in elderly patients undergoing coronary artery bypass graft surgery	Carrier, , M, Denault, A, Lavoie, J; Perrault, LP	2006
The effect of sitagliptin on markers of bone metabolism in patients with type 2 diabetes mellitus	Gilbert, , MP, Bunn, JY, Maple, R, Christian, R; Pratley, RE	2010
Prognostic Utility of Biomarkers in Predicting of One-Year Outcomes in Patients with Aortic Stenosis Treated with Transcatheter or Surgical Aortic Valve Implantation	Parenica, , J, Nemec, P, Tomandl, J, Ondrasek, J, Pavkova-Goldbergova, M, Tretina, M, Jarkovsky, J, Littnerova, S, Poloczek, M, Pokorny, P, Spinar, J, Cermakova, Z, Miklik, R, Malik, P, Pes, O, Lipkova, J, Tomandlova, M; Kala, P	2012
Combined Intra-articular Shoulder Injection and Stellate Ganglion Block in Chronic Post-mastectomy Shoulder Pain	NCT03586154	2018
Effect of CoQ10 on rheumatoid arthritis	IRCT201311014105N16	2013
Effect of cleaning teeth in the levels of c-reactive protein in the blood of patients with severe disease in teeth and bone of the maxilla and mandible	RBR-24t799	2012
Recombinant human IL-12 but not filgrastim decrease allcause mortality in a primate model of acute radiation syndrome: results from blinded vehicle-controlled glp study	Gluzman-Poltorak, , Z, Vainstein, V; Basile, L	2014
Efficacy and safety of AZD9056 200 mg once daily versus placebo in adult patients with active Crohn's disease – A randomized, double-blind, four week, parallel-group, multicentre, phase II study - POPCRON	EUCTR2005-002319-26-AT	2006
The Neurocognitive effects of Lacprodan(Registered Trademark) PL-20 in elderly participants with age-associated memory impairment	ACTRN12613000347763	2013
Influence of low voltage monophasic pulsed current and low voltage biphasic pulsed current on pressure ulcer healing based on clinical treatment effects and basic research	ACTRN12618000345280	2018
Enhanced External Counterpulsation (EECP) in Patients with Ischaemic Heart Disease and Chronic Left Ventricular Systolic Dysfunction Evaluation	ISRCTN67553357	2005

The effect of subantimicrobial-dose-doxycycline periodontal therapy on serum biomarkers of systemic inflammation: a randomized, double-masked, placebo-controlled clinical trial	Payne, , JB, Golub, LM, Stoner, JA, Lee, HM, Reinhardt, RA, Sorsa, T; Slepian, MJ	2011
Insulin-like growth factors (IGFs) and IGF binding proteins in active Crohn's disease treated with omega-3 or omega-6 fatty acids and corticosteroids	Eivindson, , M, Grønbaek, H, Nielsen, JN, Frystyk, J, Flyvbjerg, A, Jørgensen, L, Vind, I, Munkholm, P, Jensen, S, Brandslund, I; Hey, H	2005
AIN457 shows a good safety profile and clinical benefit in patients with active rheumatoid arthritis (RA) despite methotrexate therapy: 16-weeks results from a randomized proof-of-concept trial		2009
Clinical trials of a matrix metalloproteinase inhibitor in human periodontal disease. SDD Clinical Research Team	Ashley, RA	1999
Protocol of a randomised controlled, open-label trial of ex vivo normothermic perfusion versus static cold storage in donation after circulatory death renal transplantation	Hosgood, , SA, Saeb-Parsy, K, Wilson, C, Callaghan, C, Collett, D; Nicholson, ML	2017
Effect of intracanal medicaments on matrix metalloproteinase-9 and vasoactive intestinal peptide secretion in periapical lesions of re-treated canals: a randomized controlled clinical study	Özdemir, , MB, Karataş, E, Albayrak, M; Bayır, Y	2019
Effect of periodontal surgery on osteoprotegerin levels in gingival crevicular fluid, saliva, and gingival tissues of chronic periodontitis patients	Hassan, , SH, El-Refai, MI, Ghallab, NA, Kasem, RF; Shaker, OG	2015
Mechanisms of LPRF Action in the Promotion of Wound Healing and Tissue Regeneration	NCT03992638	2019
A novel dose reduction therapy using biological disease-modifying anti-rheumatic drugs to target matrix metalloproteinase 3 normalization together with a simplified disease activity index <3.3 yields effects non-inferior to standard care in rheumatoid arthritis with regards maintaining remission	Urata, , Y, Abe, S, Devers, B, Nakamura, Y, Takemoto, H; Furukawa, K-I	2016
Disodium pamidronate identifies differential osteoclastic bone resorption in metastatic prostate cancer	Clarke, , NW, McClure, J; George, NJ	1992
Therapeutic Approaches to Malnutrition Enteropathy	NCT03716115	2018
Efficacy of Roflumilast in Treatment of Severe Asthma	EUCTR2011-002101-29-DE	2011
Clinical Efficacy of Permanent Internal Mammary Artery Occlusion in Stable Coronary Artery Disease	NCT03710070	2018
Dental implants installed and restored provisionally immediately after tooth extraction in the aesthetic region of the smile	RBR-8t6c99	2018
Evaluating the management of hyperphosphatemia with PA21 in dialysis patients-rationale and design of an open-label, randomised, active-controlled phase 3 study	Tumlin, , J, Chong, E, Gaillard, S; Floege, J	2012
The effect of doxycycline and licorice therapy on gingival cervicular fluid matrix metalloproteinase-8 levels in chronic periodontitis	IRCT2012121611771N1	2013
Omega-3 Plus Low-dose Aspirin Daily Supplementation in Non-surgical Therapy to Treat Aggressive Periodontitis	NCT03093246	2017
The Efficacy of Plasma Rich in Growth Factors for the Treatment of Alveolar Osteitis: a Randomized Controlled Trial	King, , EM, Cerajewska, TL, Locke, M, Claydon, NCA, Davies, M; West, NX	2018
Linezolid Trough Concentrations Correlate with Mitochondrial Toxicity-Related Adverse Events in the Treatment of Chronic Extensively Drug-Resistant Tuberculosis	Song, , T, Lee, M, Jeon, HS, Park, Y, Dodd, LE, Dartois, V, Follman, D, Wang, J, Cai, Y, Goldfeder, LC, Olivier, KN, Xie, Y, Via, LE, Cho, SN, Barry, CE; Chen, RY	2015
Effect of isolated vitamin D supplementation on bone turnover markers in younger postmenopausal women: a randomized, double-blind, placebo-controlled trial	Nahas-Neto, , J, Cangussu, LM, Orsatti, CL, Bueloni-Dias, FN, Poloni, PF, Schmitt, EB; Nahas, EAP	2018
Allogeneic mesenchymal precursor cells (MPCS): a novel approach to treating biologic refractory rheumatoid arthritis	Kafaja, , S, Segal, KR, Skerrett, D, Itescu, S; Furst, DE	2017
An Open Label, Randomized Phase 2 Clinical Trial of Nivolumab investigating Effiacy and safety of Nivolumab given once prior to, concurrent to the radiotherapy (RT) and as maintenance therapy over 12 months in patients with advanced resectable HNSCC after surgery (NadiHN)	EUCTR2016-004787-20-DE	2017
FLT3 ligand concentrations are elevated in anca-associated vasculitides (AAV) and are influenced by immunosuppressive therapy	Venhoff, , N, Thiel, J, Voll, RE, Venhoff, AC; Salzer, U	2017
Linear growth and anthropometric and nutritional measurements in children with mild to moderate renal insufficiency: a report of the Growth Failure in Children with Renal Diseases Study	Abitbol, , CL, Warady, BA, Massie, MD, Baluarte, HJ, Fleischman, LE, Geary, DF, Kaiser, BA, McEnery, PT; Chan, JC	1990
Detection of circulating tumor cells using manually performed immunocytochemistry (MICC) does not correlate with outcome in patients with early breast cancer - Results of the German SUCCESS-A-trial	Jueckstock, , J, Rack, B, Friedl, TWP, Scholz, C, Steidl, J, Trapp, E, Tesch, H, Forstbauer, H, Lorenz, R, Rezai, M, Haberle, L, Alunni-Fabbroni, M, Schneeweiss, A, Beckmann, MW, Lichtenegger, W, Fasching, PA, Pantel, K; Janni, W	2016
Serum collagen crosslinks as markers of bone turn-over during GH replacement therapy in growth hormone deficient adults	Rodríguez-Arnao, , J, James, I, Jabbar, A, Trainer, PJ, Perrett, D, Besser, GM; Ross, RJ	1998
The effect of 1-year transdermal estrogen replacement therapy on bone mineral density and biochemical markers of bone turnover in osteopenic postmenopausal systemic lupus erythematosus patients: a randomized, double-blind, placebo-controlled trial	Bhattoa, , HP, Bettembuk, P, Balogh, A, Szegedi, G; Kiss, E	2004
A study to test the effect of a new treatment designed to improve the outcome of periodontal surgery	ISRCTN31193447	2014
Widespread increase in myeloid calcifying cells contributes to ectopic vascular calcification in type 2 diabetes	Fadini, , GP, Albiero, M, Menegazzo, L, Boscaro, E, Vigili de Kreutzenberg, S, Agostini, C, Cabrelle, A, Binotto, G, Rattazzi, M,	2011

	Bertacco, E, Bertorelle, R, Biasini, L, Mion, M, Plebani, M, Ceolotto, G, Angelini, A, Castellani, C, Menegolo, M, Grego, F, Dimmeler, S, Seeger, F, Zeiher, A, Tiengo, A; Avogaro, A	
Comparison of the effect of doxycycline with Omega-3 fatty acid and low-dose Aspirin on gingival crevicular fluid levels of matrix metalloproteinase-8	IRCT2013052311771N6	2013
Randomized placebo-controlled trial of risedronate in patients with crohn's disease and osteopenia	Pierik, , M, Hommes, DW, Dijkstra, G, Van Hogezand, RA, Lips, P, Russel, M, Van Bodegraven, AA, Van Der Woude, CJ, Van De Langerijt, L, Stokkers, P, Peeters, GMEE, Oldenburg, B; Netelenbos, CJC	2010
Investigating relationships between IL-17, Th17 pathway activation and therapeutic response to TNF inhibition in rheumatoid arthritis patients	ISRCTN18262002	2015
Phase II and coagulation cascade biomarker study of bevacizumab with or without docetaxel in patients with previously treated metastatic pancreatic adenocarcinoma	Astsaturov, , IA, Meropol, NJ, Alpaugh, RK, Burtness, BA, Cheng, JD, McLaughlin, S, Rogatko, A, Xu, Z, Watson, JC, Weiner, LM; Cohen, SJ	2011
Effects of a Cyclic NSAID Regimen on Levels of Prostaglandin-E2 and Interleukin-1beta in Gingival Crevicular Fluid of Subjects with Periodontitis: a Randomised Clinical Trial	TCTR20160202001	2016
Phase I, pharmacokinetic, and biological studies of TSU-68, a novel multiple receptor tyrosine kinase inhibitor, administered after meals with solid tumors	Murakami, , H, Ueda, Y, Shimoyama, T, Yamamoto, N, Yamada, Y, Arioka, H; Tamura, T	2011
A Phase 3 study to see the benefit and safety of Luspatercept in comparison with Epoetin Alfa to treat anaemia due to very low, low or intermediate risk Myelodysplastic Syndromes (MDS) in people who have not taken erythropoiesis-stimulating agents (ESA's) before and who require red blood cell transfusions	EUCTR2017-003190-34-LT	2018
TNT009 prevents erythrocyte c3 fragment opsonization and rescues reticulocytes from destruction in patients with cold agglutinin disease	Panicker, , S, Drucker, C, Hussain, S, Parry, GC, Gilbert, JC, Jilma, B; Jaeger, U	2016
Effectiveness of chronic treatment with alendronate in the osteoporosis of Cushing's disease	Di Somma, , C, Colao, A, Pivonello, R, Klain, M, Faggiano, A, Tripodi, FS, Merola, B, Salvatore, M; Lombardi, G	1998
BGB324, an orally available selective Axl inhibitor exerts anti-leukemic activity in the first-in-patient trial BGBC003 and induces unique changes in biomarker profiles	Sonja, , L, Gjertsen, BT, Heuser, M, Chromik, J, Batalla, IB, Akyuz, N, Micklem, D, Brown, A, Lorens, J, Kebenko, M, Janning, M, Binder, M, Fiedler, W; Cortes, JE	2016
Clonal evolution revealed by exome sequencing in a case of primary myelofibrosis associated with subsequent development of aggressive systemic mastocytosis/mast cell leukemia	Oh, , ST, Miller, CA, Gindin, Y, Brost, TM, Chan, J, Fulbright, MC, Fisher, DAC, Duncavage, EJ, O'Laughlin, M, Griffith, M, Griffith, OL; Wartman, LD	2016
Dental Pain 4	EUCTR2012-002996-32-GB	2012
B-cell maturation antigen (BCMA)-specific chimeric antigen receptor T cells (CART-BCMA) for Multiple Myeloma (MM): initial safety and efficacy from a phase i study	Cohen, , AD, Garfall, AL, Stadtmauer, EA, Lacey, SF, Lancaster, E, Vogl, DT, Dengel, K, Ambrose, DE, Chen, F, Plesa, G, Kulikovskaya, I, Gonzalez, VE, Gupta, M, Young, RM, Carey, T, Ferthio, R, Weiss, BM, Richardson, C, Isaacs, RE, Melenhorst, JJ, Levine, BL, June, CH; Milone, MC	2016
Human dental pulp stem cells (hDPSCs) as treatment for periodontal disease	ISRCTN12831118	2016
Determination of IDH1 mutational burden and clearance via next-generation sequencing in patients with IDH1 mutation-positive hematologic malignancies receiving AG-120, a first-in-class inhibitor of mutant IDH1	DiNardo, , CD, De Botton, S, Stein, EM, Roboz, GJ, Swords, RT, Pollyea, DA, Fathi, AT, Collins, R, Altman, JK, Flinn, IW, Mannis, GN, Mims, AS, Foran, JM, Pigneux, A, Prince, GT, Uy, GL, Tallman, MS, Kantarjian, HM, Liu, H, Attar, EC, Sacolick, J, Yen, K, Hurov, JB, Choe, S, Wu, B; Stone, RM	2016
Effect of photodynamic therapy on inflammatory biomarkers and clinical parameters in moderate to severe chronic periodontitis	IRCT2012121611770N1	2013
Interim results from a phase 1/2 clinical study of lentiglobin gene therapy for severe sickle cell disease	Kanter, , J, Walters, MC, Hsieh, MM, Krishnamurti, L, Kwiatkowski, J, Kamble, RT, Von Kalle, C, Kuypers, FA, Cavazzana, M, Leboulch, P, Joseney-Antoine, M, Asmal, M, Thompson, AA; Tisdale, JF	2016
Preliminary safety and clinical activity in a phase 1 study of Blu- 285, a potent, highly-selective inhibitor of KIT D816V in advanced systemic mastocytosis (SM)	Drummond, , MW, DeAngelo, DJ, Deininger, MW, Radia, D, Quiery, AT, Hexner, EO, Shi, H, Alvarez-Diez, T, Evans, EK, Healy, ME, Wolf, BB; Verstovsek, S	2016
TCA cycle inhibition by CPI-613 increases sensitivity to chemotherapy in older and poor risk acute myeloid leukemia (AML)	Pardee, , TS, Miller, LD, Pladna, K, Isom, S, Ellis, LR, Berenzon, D, Howard, D, Manuel, M, Dralle, S, Lyerly, S; Powell, BL	2016
A clinical study of OPN -305, a toll-like receptor 2 (TLR-2) antibody, in patients with lower risk myelodysplastic syndromes (MDS) that have received prior hypomethylating agent (HMA) therapy	Garcia-Manero, , G, Montalban-Bravo, G, Yang, H, Wei, Y, Alvarado, Y, DiNardo, CD, Daver, NG, Konopleva, M, Hearn, KP, Miller, R, Arbe-Barnes, S, Mc Guirk, P, Kearney, T, Keogh, B, Kantarjian, HM; Reilly, M	2016
Non-surgical periodontal therapy improves serum levels of C-reactive protein and edematous states in female patients with idiopathic edema	Joseph, , R, Narayan, V, Krishnan, R; Melemadathil, S	2011
High-dose chemotherapy and peripheral blood stem cell infusion in patients with non-Hodgkin's lymphoma: results of outpatient treatment in community cancer centers	Weaver, , CH, Schwartzberg, L, Zhen, B, Mangum, M, Leff, R, Tauer, K, Rosenberg, A, Pendergrass, K, Kaywin, P, Hainsworth, J, Greco, FA, West, WH; Buckner, CD	1997
The Effect of Ischemic Preconditioning on Postoperative Pain After Total Knee Arthroplasty, a Randomized, Controlled Trial	NCT01333969	2011
Dose-effect relations of loop- and thiazide-diuretics on calcium homeostasis: a randomized, double-blinded Latin-square multiple cross-over study in postmenopausal osteopenic women	Rejnmark, , L, Vestergaard, P, Pedersen, AR, Heickendorff, L, Andreasen, F; Mosekilde, L	2003
Effects of changing strategies of fracture fixation on immunologic changes and systemic complications after multiple trauma: damage control orthopedic surgery	Pape, H-C	2008

Matrix metalloproteinases and the activity of their tissue inhibitors in patients with ST-elevation myocardial infarction treated with primary angioplasty	Kuliczkowski, , W, Urbaniak, J, Hallén, J, Woźniak, M, Poloński, L, Mysiak, A, Atar, D, Zembala, M; Serebruany, V	2013
Short-term clinical and immunologic effects of scaling and root planing with Er: YAG laser in chronic periodontitis	Lopes, , BM, Marcantonio, RA, Thompson, GM, Neves, LH; Theodoro, LH	2008
Calcifediol: perspectives for clinical applications in 2017-control of mineral metabolism: the addid study	Minisola, , S, Cianferotti, L, Biondi, P, Pepe, J, Cipriani, C, Fossi, C, Giusti, F, Franceschelli, F, Leoncini, G; Brandi, ML	2017
Effect of β-cryptoxanthin (β-Cx), Plant Sterols and Galactooligosaccharides on Systemic and Gastrointestinal Markers	NCT03469518	2018
FUTURE-2: results from an open-label, long-term safety and tolerability extension study using the pediatric FormUlation of bosenTan in pUlmonary arterial hypeRtEnsion	Berger, , RM, Haworth, SG, Bonnet, D, Dulac, Y, Fraisse, A, Galiè, N, Ivy, DD, Jaïs, X, Miera, O, Rosenzweig, EB, Efficace, M, Kusic-Pajic, A; Beghetti, M	2016
Adjuvant spleen ultrafiltrate immunotherapy in unresectable advanced non small cell lung cancer	Tomeczko, , J, Napora, P, Dlubek, D, Pacuszko, T, Bochenska, J, Teucher, T, Schmitz, H; Lange, A	1997
Effects of erythropoietin on mobilisation of haemopoietic progenitor cells	Pettengell, , R, Woll, PJ, Chang, J, Coutinho, L, Testa, NG; Crowther, D	1994
A phase 3 randomized controlled trial of the efficacy and safety of atrasentan in men with metastatic hormone-refractory prostate cancer	Carducci, , MA, Saad, F, Abrahamsson, PA, Dearnaley, DP, Schulman, CC, North, SA, Sleep, DJ, Isaacson, JD; Nelson, JB	2007
Combination of granulocyte colony-stimulating factor and erythropoietin improves outcomes of patients with decompensated cirrhosis	Kedarisetty, , CK, Anand, L, Bhardwaj, A, Bhadoria, AS, Kumar, G, Vyas, AK, David, P, Trehanpati, N, Rastogi, A, Bihari, C, Maiwall, R, Garg, HK, Vashishtha, C, Kumar, M, Bhatia, V; Sarin, SK	2015
Nivolumab combined with ibrutinib for CLL and richter transformation: a phase II trial	Jain, , N, Basu, S, Thompson, PA, Ohanian, M, Ferrajoli, A, Pemmaraju, N, Cortes, JE, Estrov, Z, Burger, JA, Neelapu, SS, Lopez, W, Thakral, B, Bueso-Ramos, CE, Blando, J, O'Brien, SM, Kantarjian, HM, Allison, J, Keating, M, Sharma, P; Wierda, WG	2016
Omega 3 fatty acids as a host modulator in chronic periodontitis patients: a randomised, double-blind, palcebo-controlled, clinical trial		2014
Efficacy of honey in healing diabetic ulcers - a pilot study	Ullal, , S; Adhikari, P	2014
Effect of intensive atorvastatin therapy on prostaglandin E2 levels and metalloproteinase-9 activity in the plasma of patients with non-ST-elevation acute coronary syndrome	Gómez-Hernández, , A, Sánchez-Galán, E, Ortego, M, Martín-Ventura, JL, Blanco-Colio, LM, Tarín-Vicente, N, Jiménez-Nacher, JJ, López-Bescos, L, Egido, J; Tuñón, J	2008
Impact of mild or moderate renal insufficiency on the intravascular ultrasonic analysis of chronic vascular response to paclitaxel-eluting and bare-metal stents (from the TAXUS IV, V, and VI trials)	Aoki, , J, Mintz, GS, Weissman, NJ, Mandinov, L, Grube, E, Dawkins, KD, Ellis, SG, Greenberg, J, Yu, A, Mann, JT, Cannon, L, Cambier, PA; Stone, GW	2008
Metabolic syndrome and breast cancer: effects of a 16-week combined exercise intervention	Dieli-Conwright, , CM, Tripathy, D, Schroeder, ET, Mortimer, JE; Bernstein, L	2013
The assessment of prognostic factors in surgical treatment of low-grade gliomas: a prospective study	Majchrzak, , K, Kaspera, W, Bobek-Billewicz, B, Hebda, A, Stasik-Pres, G, Majchrzak, H; Ladzinski, P	2012
ATG induction therapy: long-term effects on Th1 but not on Th2 responses	Weimer, , R, Staak, A, Süsal, C, Streller, S, Yildiz, S, Pelzl, S, Renner, F, Dietrich, H, Daniel, V, Rainer, L, Kamali-Ernst, S, Ernst, W, Padberg, W; Opelz, G	2005
Thalidomide versus active supportive care for maintenance in patients with malignant mesothelioma after first-line chemotherapy (NVALT 5): an open-label, multicentre, randomised phase 3 study	Buikhuisen, , WA, Burgers, JA, Vincent, AD, Korse, CM, van Klaveren, RJ, Schramel, FM, Pavlakis, N, Nowak, AK, Custers, FL, Schouwink, JH, Gans, SJ, Groen, HJ, Strankinga, WF; Baas, P	2013
Free DKK-1 serum levels are unchanged in spondyloarthritis patients treated by anti-TNF-data from the sparse study	Miceli-Richard, , C, Combe, B, Berenbaum, F, Schaeverbeke, T, Koppiker, N, Logeart, I, Dubanchet, A; Dougados, M	2016
The dose-related effects of dexmedetomidine on renal functions and serum neutrophil gelatinase-associated lipocalin values after coronary artery bypass grafting: a randomized, triple-blind, placebo-controlled study	Balkanay, , OO, Goksedef, D, Omeroglu, SN; Ipek, G	2015
The Influence of Food Matrix Delivery System on the Bioavailability of Vitamin D3	NCT03783273	2018
Phase I trial of a melanoma vaccine with gp100(280-288) peptide and tetanus helper peptide in adjuvant: immunologic and clinical outcomes	Slingluff, , CL, Yamshchikov, G, Neese, P, Galavotti, H, Eastham, S, Engelhard, VH, Kittlesen, D, Deacon, D, Hibbitts, S, Grosh, WW, Petroni, G, Cohen, R, Wiernasz, C, Patterson, JW, Conway, BP; Ross, WG	2001
Immunomodulatory effects of high-dose and low-dose interferon alpha2b in patients with high-risk resected melanoma: the E2690 laboratory corollary of intergroup adjuvant trial E1690	Kirkwood, , JM, Richards, T, Zarour, HM, Sosman, J, Ernstoff, M, Whiteside, TL, Ibrahim, J, Blum, R, Wieand, S; Mascari, R	2002
Resection of hepatocellular carcinoma in elderly patients and the role of energy balance	Cannistra, , M, Grande, R, Ruggiero, M, Novello, M, Zullo, A, Bonaiuto, E, Vaccarisi, S, Cavallari, G, Serra, R; Nardo, B	2016
Exposure-Response and Tumor Growth Inhibition Analyses of the Monovalent Anti-c-MET Antibody Onartuzumab (MetMAb) in the Second- and Third-Line Non-Small Cell Lung Cancer	Han, , K, Chanu, P, Jonsson, F, Winter, H, Bruno, R, Jin, J; Stroh, M	2017
Initial Experience with Covered Endovascular Reconstruction of the Aortic Bifurcation in Conjunction with Chimney Grafts	Dijkstra, , ML, Goverde, PCJM, Holden, A, Zeebregts, CJ; Reijnen, MMPJ	2017
Minimised closed circuit coronary artery bypass grafting in the elderly is associated with lower levels of rgan-specific biomarkers: a prospective randomised study	Van Boven, , W-JP, Gerritsen, WB, Driessen, AH, Van Dongen, EP, Klautz, RJ; Aarts, LP	2013
Combination of hemofiltration and peritoneal dialysis in the treatment of severe acute pancreatitis	Feng, , GH, Cai, Y, Jia, PH, Yang, QJ, Jia, Z, Zhang, J; Zhang, XP	2004
Influence of a probiotic mixture on antibiotic induced microbiota disturbances	Forssten, , S, Evans, M, Wilson, D; Ouwehand, AC	2014
A combination treatment of prednisone, aspirin, folate, and progesterone in women with idiopathic recurrent miscarriage: a matched-pair study	Tempfer, , CB, Kurz, C, Bentz, EK, Unfried, G, Walch, K, Czizek, U; Huber, JC	2006

CD4+FOXP3+ regulatory T cell depletion by low-dose cyclophosphamide prevents recurrence in patients with large condylomata acuminata after laser therapy	Cao, , Y, Zhao, J, Yang, Z, Cai, Z, Zhang, B, Zhou, Y, Shen, GX, Chen, X, Li, S; Huang, B	2010
Common beans and cowpeas as complementary foods to reduce environmental enteric dysfunction and stunting in Malawian children: study protocol for two randomized controlled trials	Trehan, , I, Benzoni, NS, Wang, AZ, Bollinger, LB, Ngoma, TN, Chimimba, UK, Stephenson, KB, Agapova, SE, Maleta, KM; Manary, MJ	2015
Efficacy and safety of ustekinumab in patients with active psoriatic arthritis: 1 year results of the phase 3, multicentre, double-blind, placebo-controlled PSUMMIT 1 trial	McInnes, , IB, Kavanaugh, A, Gottlieb, AB, Puig, L, Rahman, P, Ritchlin, C, Brodmerkel, C, Li, S, Wang, Y, Mendelsohn, AM; Doyle, MK	2013
Skincare and synbiotics for the prevention of atopic dermatitis or food allergy in newborn infants: a 2 × 2 factorial randomized non-treatment controlled trial	Dissanayake, , E, Tani, Y, Sahara, M, Mitsuishi, C, Nagai, K, Sato, Y, Suzuki, Y, Nakano, T, Yamaide, F; Shimojo, N	2018
Adjuvant immunotherapy with autologous cytokine-induced killer cells for hepatocellular carcinoma	Lee, , JH, Lee, JH, Lim, YS, Yeon, JE, Song, TJ, Yu, SJ, Gwak, GY, Kim, KM, Kim, YJ, Lee, JW; Yoon, JH	2015
Cerebral protective effect of nicorandil premedication on patients undergoing liver transplantation	Xia, , YF, Wang, ZP, Zhou, YC, Yan, T; Li, ST	2012
pCMV-vegf165 Intramuscular Gene Transfer is an Effective Method of Treatment for Patients With Chronic Lower Limb Ischemia	Deev, , RV, Bozo, IY, Mzhavanadze, ND, Voronov, DA, Gavrilenko, AV, Chervyakov, YV, Staroverov, IN, Kalinin, RE, Shvalb, PG; Isaev, AA	2015
1-Year Results of a Multicenter Randomized Controlled Trial Comparing Heparin-Bonded Endoluminal to Femoropopliteal Bypass	Reijnen, , MMPJ, van Walraven, LA, Fritschy, WM, Lensvelt, MMA, Zeebregts, CJ, Lemson, MS, Wikkeling, ORM, Smeets, L; Holewijn, S	2017
Therapy with autologous adipose-derived regenerative cells for the care of chronic ulcer of lower limbs in patients with peripheral arterial disease	Marino, , G, Moraci, M, Armenia, E, Orabona, C, Sergio, R, De Sena, G, Capuozzo, V, Barbarisi, M, Rosso, F, Giordano, G, Iovino, F; Barbarisi, A	2013
Trichuris suis ova in relapsing-remitting multiple sclerosis and clinically isolated syndrome (TRIOMS): study protocol for a randomized controlled trial	Rosche, , B, Wernecke, KD, Ohlraun, S, Dörr, JM; Paul, F	2013
Abatacept in children with juvenile idiopathic arthritis: a randomised, double-blind, placebo-controlled withdrawal trial	Ruperto, , N, Lovell, DJ, Quartier, P, Paz, E, Rubio-Pérez, N, Silva, CA, Abud-Mendoza, C, Burgos-Vargas, R, Gerloni, V, Melo-Gomes, JA, Saad-Magalhães, C, Sztajnbok, F, Goldenstein-Schainberg, C, Scheinberg, M, Penades, IC, Fischbach, M, Orozco, J, Hashkes, PJ, Hom, C, Jung, L, Lepore, L, Oliveira, S, Wallace, CA, Sigal, LH, Block, AJ, Covucci, A, Martini, A; Giannini, EH	2008
Results of the swiss epo neuroprotection trial in very preterm infants	Fauchere, , J-C, Leuchter, RH, Natalucci, G, Dame, C, Koller, BM, Ruegger, CM, Hagmann, C, Huppi, PS; Bucher, HU	2015
Pulsed oral sirolimus in advanced autosomal-dominant polycystic kidney disease (Vienna RAP Study): study protocol for a randomized controlled trial	Riegersperger, , M, Herkner, H; Sunder-Plassmann, G	2015
Angiogenesis - A new goal in peripheral artery occlusive disease therapy	Makinen, K	2003
Neutrophil transfusions in the treatment of neutropenic patients submitted to allogeneic HSCT: possible role on graft failure	Giammarco, , S, Chiusolo, P, Laurenti, L, Sor, F, Piccirillo, N, Teofili, L; Sica, S	2017
Improvement in disability after alemtuzumab treatment of multiple sclerosis is associated with neuroprotective autoimmunity	Jones, , JL, Anderson, JM, Phuah, CL, Fox, EJ, Selmaj, K, Margolin, D, Lake, SL, Palmer, J, Thompson, SJ, Wilkins, A, Webber, DJ, Compston, DA; Coles, AJ	2010
Bovine colostrum as a modulator of immune system in allergic children	Snejdarova, , I, Hrbkova, M, Votruba, M; Snejdarova, V	2001
A phase II, randomized, multicenter study evaluating the combination of lapatinib and vinorelbine in women with ErbB2 overexpressing metastatic breast cancer	Janni, , W, Sarosiek, T, Karaszewska, B, Pikiel, J, Staroslawska, E, Potemski, P, Salat, C, Brain, E, Caglevic, C, Briggs, K, Desilvio, M, Marini, L; Papadimitriou, C	2014
Gestational diabetes and pregnancy morbidity and outcome	Bratila, , E, Comandasu, DE, Iacob, G, Teodorescu, C, Cirstoiu, M, Bohiltea, R, Berceanu, C; Mehedintu, C	2016
Latent classes of nonresponders, rapid responders, and gradual responders in depressed outpatients receiving antidepressant medication and psychotherapy	Thibodeau, , MA, Quilty, LC, De Fruyt, F, De Bolle, M, Rouillon, F; Bagby, RM	2015
Moxifloxacin as an adjunctive antibiotic in the treatment of severe chronic periodontitis	Guentsch, , A, Jentsch, H, Pfister, W, Hoffmann, T; Eick, S	2008
Macular atrophy progression and 7-year vision outcomes in subjects from the ANCHOR, MARINA, and HORIZON studies: the SEVEN-UP study	Bhisitkul, , RB, Mendes, TS, Rofagha, S, Enanoria, W, Boyer, DS, Sadda, SR; Zhang, K	2015
Reducing polycystic liver volume in ADPKD: effects of somatostatin analogue octreotide	Caroli, , A, Antiga, L, Cafaro, M, Fasolini, G, Remuzzi, A, Remuzzi, G; Ruggenenti, P	2010
Chemically modified tetracyclines an emerging host modulator in chronic periodontitis patients: a randomized, double-blind, placebo-controlled, clinical trial	Alyousef, , AA, Divakar, DD; Muzaheed, null	2017
Zinc supplementation in the management of shigellosis in malnourished children in Bangladesh	Roy, , SK, Raqib, R, Khatun, W, Azim, T, Chowdhury, R, Fuchs, GJ; Sack, DA	2008
A randomized clinical trial of bifocal glasses for myopic children with esophoria: results after 54 months	Fulk, , GW, Cyert, LA; Parker, DE	2002
Comparison Between Two Different Antibiotic Regimens for the Placement of Dental Implants	NCT01851681	2013
The skeletal consequences of growth hormone therapy in dialyzed children: a randomized trial	Bacchetta, , J, Wesseling-Perry, K, Kuizon, B, Pereira, RC, Gales, B, Wang, HJ, Elashoff, R; Salusky, IB	2013
Regional angiogenesis with vascular endothelial growth factor in peripheral arterial disease: a phase II randomized, double-blind,	Rajagopalan, , S, Mohler, ER, Lederman, RJ, Mendelsohn, FO, Saucedo, JF, Goldman, CK, Blebea, J, Macko, J, Kessler, PD, Rasmussen, HS; Annex, BH	2003

controlled study of adenoviral delivery of vascular endothelial growth factor 121 in patients with disabling intermittent claudication		
The GoodNEWS (Genes, Nutrition, Exercise, Wellness, and Spiritual Growth) Trial: a community-based participatory research (CBPR) trial with African-American church congregations for reducing cardiovascular disease risk factors–recruitment, measurement, and randomization	DeHaven, , MJ, Ramos-Roman, MA, Gimpel, N, Carson, J, DeLemos, J, Pickens, S, Simmons, C, Powell-Wiley, T, Banks-Richard, K, Shuval, K, Duvahl, J, Duval, J, Tong, L, Hsieh, N; Lee, JJ	2011
Outcomes after multiple courses of granulocyte colony-stimulating factor and growth hormone in decompensated cirrhosis: a randomized trial	Verma, , N, Kaur, A, Sharma, R, Bhalla, A, Sharma, N, De, A; Singh, V	2018
A randomized, double-blind, phase II study of ramucirumab plus docetaxel vs placebo plus docetaxel in Japanese patients with stage IV non-small cell lung cancer after disease progression on platinum-based therapy	Yoh, , K, Hosomi, Y, Kasahara, K, Yamada, K, Takahashi, T, Yamamoto, N, Nishio, M, Ohe, Y, Koue, T, Nakamura, T, Enatsu, S, Lee, P, Ferry, D, Tamura, T; Nakagawa, K	2016
Standard medical therapies do not alter colonic transit time in children with treatment-resistant slow-transit constipation	Clarke, , MCC, Chase, JW, Gibb, S, Catto-Smith, AG, Hutson, JM; Southwell, BR	2009
A phase II study of aflibercept in patients with advanced epithelial ovarian cancer and symptomatic malignant ascites	Colombo, , N, Mangili, G, Mammoliti, S, Kalling, M, Tholander, B, Sternas, L, Buzenet, G; Chamberlain, D	2012
Extracorporeal Shock Wave Lithotripsy and Endotherapy for Pain in Chronic Pancreatitis	NCT03966781	2019
EULAR response criteria for polymyalgia rheumatica: results of an initiative of the European Collaborating Polymyalgia Rheumatica Group (subcommittee of ESCISIT)	Leeb, , BF, Bird, HA, Nesher, G, Andel, I, Hueber, W, Logar, D, Montecucco, CM, Rovensky, J, Sautner, J; Sonnenblick, M	2003
Four-step high-dose sequential chemotherapy with hematopoietic progenitor-cell support as induction treatment for patients with solid tumors	Culine, , S, Fabbro, M, Assens, C, Ychou, M, Romieu, G, Kramar, A, Cupissol, D, Pinguet, F; Pujol, H	1997
IL-1β as a New Early Predictive Biomarker for Non-Small Cell Lung Cancers Outcome	Missiroli, , S, Tamburini, N, Perrone, M, Maniscalco, P, Gafa, R, Lanza, G, Pinton, P, Cavallesco, G; Giorgi, C	2018
The Lake Victoria Island Intervention Study on Worms and Allergy-related diseases (LaVIISWA): study protocol for a randomised controlled trial	Nampijja, , M, Webb, EL, Kaweesa, J, Kizindo, R, Namutebi, M, Nakazibwe, E, Oduru, G, Kabuubi, P, Kabagenyi, J, Kizito, D, Muhangi, L, Akello, M, Verweij, JJ, Nerima, B, Tukahebwa, E; Elliott, AM	2015
Efficacy and safety of individualized growth hormone treatment in adult Japanese patients with growth hormone deficiency	Chihara, , K, Kato, Y, Shimatsu, A, Tanaka, T; Kohno, H	2008
Comparison of low-normal and high-normal IGF-1 target levels during growth hormone replacement therapy: a randomized clinical trial in adult growth hormone deficiency	van Bunderen, , CC, Lips, P, Kramer, MH; Drent, ML	2016
High-fibre diet and Lactobacillus paracasei B21060 in symptomatic uncomplicated diverticular disease	Lahner, , E, Esposito, G, Zullo, A, Hassan, C, Cannaviello, C, Paolo, MC, Pallotta, L, Garbagna, N, Grossi, E; Annibale, B	2012
A disintegrin and metalloprotease-17 and galectin-9 are important regulators of local 4-1BB activity and disease outcome in rheumatoid arthritis	Nielsen, , MA, Andersen, T, Etzerodt, A, Kragstrup, TW, Rasmussen, TK, Stengaard-Pedersen, K, Hetland, ML, Hørslev-Petersen, K, Junker, P, Østergaard, M, Hvid, M, Moestrup, SK; Deleuran, B	2016
High-dose ifosfamide/carboplatin/etoposide: maximum tolerable doses, toxicities, and hematopoietic recovery after autologous stem cell reinfusion	Fields, , KK, Elfenbein, GJ, Perkins, JB, Janssen, WE, Ballester, OF, Hiemenz, JW, Zorsky, PE, Kronish, LE; Foody, MC	1994
Add-Aspirin trial: a phase III, double blind, placebo-controlled, randomized trial assessing the effects of aspirin on disease recurrence and survival after primary therapy in common nonmetastatic solid tumors	Langley, , RE, Wilson, RH, Ring, AE, Kynaston, HG, Cameron, DA, Coyle, C, Gilbert, DC, Patrono, C, Rowley, S, Murphy, C, Adlam, D, Hubner, R, Iveson, T, Steele, RJ, Thomas, AL, Underwood, TJ, Jankowski, J, Gupta, S, Pramesh, CS; Parmar, M	2014
Primary hormo-chemotherapy in metastatic prostate cancer	Janknegt, , RA; Boon, TA	1996
Efficacy and safety of pegylated liposomal doxorubicin in combination with bortezomib for multiple myeloma: effects of adverse prognostic factors on outcome	Blade, , J, Sonneveld, P, San Miguel, JF, Sutherland, HJ, Hajek, R, Nagler, A, Spencer, A, Robak, T, Lantz, KC, Zhuang, SH, Harousseau, J-L; Orlowski, RZ	2011
Immunomodulator Intensification of Etioropic Therapy in Patients with Advanced Pulmonary Tuberculosis	Kolomiets, , VM, Abramov, AV, Rachina, NV; Rubleva, NV	2015
A randomized, placebo-controlled trial of IGF-1 for delayed graft function: a human model to study postischemic ARF	Hladunewich, , MA, Corrigan, G, Derby, GC, Ramaswamy, D, Kambham, N, Scandling, JD; Myers, BD	2003
The decision to extract: part II. Analysis of clinicians' stated reasons for extraction	Baumrind, , S, Korn, EL, Boyd, RL; Maxwell, R	1996
Duration for apical barrier formation in necrotic immature permanent incisors treated with calcium hydroxide apexification using ultrasonic or hand filing	Lee, , LW, Hsiao, SH, Chang, CC; Chen, LK	2010
Novel and targeted therapies for OA	Lane, N	2012
Ciprofloxacin during upper respiratory tract infections to reduce Pseudomonas aeruginosa infection in paediatric cystic fibrosis: a pilot study	Connett, , GJ, Pike, KC, Legg, JP, Cathie, K, Dewar, A, Foote, K, Harris, A; Faust, SN	2015
Clarithromycin Plus Intravenous Immunoglobulin Therapy Can Reduce the Relapse Rate of Kawasaki Disease: a Phase 2, Open-Label, Randomized Control Study	Nanishi, , E, Nishio, H, Takada, H, Yamamura, K, Fukazawa, M, Furuno, K, Mizuno, Y, Saigo, K, Kadoya, R, Ohbuchi, N, Onoe, Y, Yamashita, H, Nakayama, H, Hara, T, Ohno, T, Takahashi, Y, Hatae, K, Harada, T, Shimose, T, Kishimoto, J, Ohga, S; Hara, T	2017
The Design and Rationale of a Clinical Trial Evaluating Limb Postconditioning in Young Patients with Intracranial Arterial Stenosis	Wei, , M, Huo, K, Liu, R, Yang, J, Cheng, Y, Chang, S, Ren, D; Luo, G	2016
Use of Oxandrolone to Promote Growth in Neonates following Surgery for Complex Congenital Heart Disease: an Open-Label Pilot Trial	Burch, , PT, Spigarelli, MG, Lambert, LM, Loftus, PD, Sherwin, CM, Linakis, MW, Sheng, X, LuAnn Minich, L; Williams, RV	2016

Anti-TNFα Use During Elective Foot and Ankle Surgery in Patients With Rheumatoid Arthritis	NCT02242474	2014
Primary transpupillary thermotherapy for small choroidal melanoma	Chojniak, , MM, Chojniak, R, Nishimoto, IN, Allemann, N; Erwenne, CM	2011
General stress response to conventional and laparoscopic cholecystectomy	Glaser, , F, Sannwald, GA, Buhr, HJ, Kuntz, C, Mayer, H, Klee, F; Herfarth, C	1995
Long-term Efficacy of Orthokeratology Contact Lens Wear in Controlling the Progression of Childhood Myopia	Santodomingo-Rubido, , J, Villa-Collar, C, Gilmartin, B, Gutiérrez-Ortega, R; Sugimoto, K	2017
Effects of YF476 and Rabeprazole on Gastric Function	NCT01699113	2012
Patients with metastatic renal cell carcinoma who benefit from axitinib dose titration: analysis from a randomised, double-blind phase II study	Tomita, , Y, Uemura, H, Oya, M, Shinohara, N, Habuchi, T, Fujii, Y, Kamei, Y, Umeyama, Y, Bair, AH; Rini, BI	2019
Rationale and design of a prospective, randomised study of retrograde application of bone marrow aspirate concentrate (BMAC) through coronary sinus in patients with congestive heart failure of ischemic etiology (the RETRO study)	Pleva, , L, Kukla, P, Vitkova, K; Prochazka, V	2019
Assessment of prognostic and predictive properties of blood biomarkers in pivotal clinical studies of Pirfenidone'	Neighbors, , M, Cabanski, CR, DePianto, DJ, Ramalingam, TR, Tew, GW, Jia, G, Abbas, AR, Peng, K, Ray, J, Palme, S, Dziadek, S, Ritter, M, Kirchgaessler, K, Ley, B, Wolters, PJ, Collard, HR; Arron, JR	2016
Alteration in angiogenic and anti-angiogenic forms of vascular endothelial growth factor-A in skeletal muscle of patients with intermittent claudication following exercise training	Jones, , WS, Duscha, BD, Robbins, JL, Duggan, NN, Regensteiner, JG, Kraus, WE, Hiatt, WR, Dokun, AO; Annex, BH	2012
Management of adolescents with very poorly controlled type 1 diabetes by nurses: a parallel group randomized controlled trial	Kassai, , B, Rabilloud, M, Bernoux, D, Michal, C, Riche, B, Ginhoux, T, Laudy, V, Terral, D, Didier-Wright, C, Maire, V, Dumont, C, Cottancin, G, Plasse, M, Jeannoel, GP, Khoury, J, Bony, C, Lièvre, M, Drai, J; Nicolino, M	2015
Quality-of-Life After Everolimus-Eluting Stents or Bypass Surgery for Left-Main Disease: results From the EXCEL Trial	Baron, , SJ, Chinnakondepalli, K, Magnuson, EA, Kandzari, DE, Puskas, JD, Ben-Yehuda, O, van Es, GA, Taggart, DP, Morice, MC, Lembo, NJ, Brown, WM, Banning, A, Simonton, CA, Kappetein, AP, Sabik, JF, Serruys, PW, Stone, GW; Cohen, DJ	2017
High-dose fractionated total-body irradiation, etoposide and cyclophosphamide for treatment of malignant lymphoma: comparison of autologous bone marrow and peripheral blood stem cells	Brunvand, , MW, Bensinger, WI, Soll, E, Weaver, CH, Rowley, SD, Appelbaum, FR, Lilleby, K, Clift, RA, Gooley, TA, Press, OW, Fefer, A, Storb, R, Sanders, JE, Martin, PL, Chauncey, T, Maziarz, RT, Zuckerman, N, Montgomery, P, Dorn, R, Weiden, PL, Demirer, T, Holmberg, LA, Schiffman, K, McSweeney, PA; Buckner, CD	1996
Risk factors and perinatal outcome in women with mid trimester urinary tract infection in pregnancy	Ruman, , U, Rahman, MM; Khan, R	2018
Impact of neo-adjuvant Sorafenib treatment on liver transplantation in HCC patients - a prospective, randomized, double-blind, phase III trial	Hoffmann, , K, Ganten, T, Gotthardtp, D, Radeleff, B, Settmacher, U, Kollmar, O, Nadalin, S, Karapanagiotou-Schenkel, I, von Kalle, C, Jäger, D, Büchler, MW; Schemmer, P	2015
A phase II, randomized, blinded study of the farnesyltransferase inhibitor tipifarnib combined with letrozole in the treatment of advanced breast cancer after antiestrogen therapy	Johnston, , SR, Semiglazov, VF, Manikhas, GM, Spaeth, D, Romieu, G, Dodwell, DJ, Wardley, AM, Neven, P, Bessems, A, Park, YC, De Porre, PM, Perez Ruixo, JJ; Howes, AJ	2008
Serum phosphate modifies the vascular response to vitamin D receptor activation in chronic kidney disease (CKD) patients	Zoccali, , C, Torino, C, Curatola, G, Panuccio, V, Tripepi, R, Pizzini, P, Versace, M, Bolignano, D, Cutrupi, S, Ghiadoni, L, Thadhani, R, Tripepi, G; Mallamaci, F	2016
Comparing the effect of sucrose gel and metronidazole gel in treatment of clinical symptoms of bacterial vaginosis: a randomized controlled trial	Khazaeian, , S, Navidian, A, Navabi-Rigi, SD, Araban, M, Mojab, F; Khazaeian, S	2018
Effects of early intensive blood pressure-lowering treatment on the growth of hematoma and perihematomal edema in acute intracerebral hemorrhage: the Intensive Blood Pressure Reduction in Acute Cerebral Haemorrhage Trial (INTERACT)	Anderson, , CS, Huang, Y, Arima, H, Heeley, E, Skulina, C, Parsons, MW, Peng, B, Li, Q, Su, S, Tao, QL, Li, YC, Jiang, JD, Tai, LW, Zhang, JL, Xu, E, Cheng, Y, Morgenstern, LB, Chalmers, J; Wang, JG	2010
Effects of antiproteinuric intervention on elevated connective tissue growth factor (CTGF/CCN-2) plasma and urine levels in nondiabetic nephropathy	Slagman, , MC, Nguyen, TQ, Waanders, F, Vogt, L, Hemmelder, MH, Laverman, GD, Goldschmeding, R; Navis, G	2011
Treatment of Active Crohn's Disease With an Ordinary Food-based Diet That Replicates Exclusive Enteral Nutrition	Svolos, , V, Hansen, R, Nichols, B, Quince, C, Ijaz, UZ, Papadopoulou, RT, Edwards, CA, Watson, D, Alghamdi, A, Brejnrod, A, Ansalone, C, Duncan, H, Gervais, L, Tayler, R, Salmond, J, Bolognini, D, Klopfleisch, R, Gaya, DR, Milling, S, Russell, RK; Gerasimidis, K	2019
The influence of patient and wound variables on healing of venous leg ulcers in a randomized controlled trial of growth-arrested allogeneic keratinocytes and fibroblasts	Lantis, , JC, Marston, WA, Farber, A, Kirsner, RS, Zhang, Y, Lee, TD, Cargill, DI; Slade, HB	2013
Evaluation of melatonin effectiveness in the adjuvant treatment of ulcerative colitis	Chojnacki, , C, Wisniewska-Jarosinska, M, Walecka-Kapica, E, Klupinska, G, Jaworek, J; Chojnacki, J	2011
Proteinuria in a placebo-controlled study of basic fibroblast growth factor for intermittent claudication	Cooper, , LT, Hiatt, WR, Creager, MA, Regensteiner, JG, Casscells, W, Isner, JM, Cooke, JP; Hirsch, AT	2001
Isolated central nervous system metastases in patients with HER2-overexpressing advanced breast cancer treated with first-line trastuzumab-based therapy	Burstein, , HJ, Lieberman, G, Slamon, DJ, Winer, EP; Klein, P	2005
Role of insulin-like growth factor-I in the treatment of painful small fiber predominant neuropathy	Windebank, , AJ, Sorenson, EJ, Civil, R; O'Brien, PC	2004
Prevention of malnutrition among young children in rural Bangladesh by a food-health-care educational intervention: a randomized, controlled trial	Roy, , SK, Jolly, SP, Shafique, S, Fuchs, GJ, Mahmud, Z, Chakraborty, B; Roy, S	2007

Long-term inhaled corticosteroids in preschool children at high risk for asthma	Guilbert, , TW, Morgan, WJ, Zeiger, RS, Mauger, DT, Boehmer, SJ, Szefler, SJ, Bacharier, LB, Lemanske, RF, Strunk, RC, Allen, DB, Bloomberg, GR, Heldt, G, Krawiec, M, Larsen, G, Liu, AH, Chinchilli, VM, Sorkness, CA, Taussig, LM; Martinez, FD	2006
The role of systemic steroids and phototherapy in the treatment of stable vitiligo: a randomized controlled trial	El Mofty, , M, Essmat, S, Youssef, R, Sobeih, S, Mahgoub, D, Ossama, S, Saad, A, El Tawdy, A, Mashaly, HM, Saney, I, Helal, R; Shaker, O	2016
Prediction of outcome in the treatment of onychomycosis	Sommer, , S, Sheehan-Dare, RA, Goodfield, MJ; Evans, EG	2003
Intra-operative fracture risk - quantifying forces and strains during femoral stem implantation for compaction and extraction broached femurs	Stockwell, , KD, Burnell, CD, Gascoyne, TC; Morrison, JB	2016
Short-term angiopeptin therapy and the incidence of graft vessel disease after heart transplantation	Meiser, , BM, Mueller, M, Foegh, M, Von Scheidt, W; Reichart, B	2002
A Randomized Trial of Providing BCG Vaccination Immediately	NCT01989026	2013
Edrecolomab in Treating Patients With Stage II Colon Cancer	NCT00002968	1999
Predictive factors of antiproliferative activity of octreotide LAR as first-line therapy for advanced neuroendocrine tumours	Laskaratos, , F-M, Walker, M, Naik, K, Maragkoudakis, E, Oikonomopoulos, N, Grant, L, Meyer, T, Caplin, M; Toumpanakis, C	2016
Effect of a Proposed Trastuzumab Biosimilar Compared With Trastuzumab on Overall Response Rate in Patients With ERBB2 (HER2)-Positive Metastatic Breast Cancer: a Randomized Clinical Trial	Rugo, , HS, Barve, A, Waller, CF, Hernandez-Bronchud, M, Herson, J, Yuan, J, Sharma, R, Baczkowski, M, Kothekar, M, Loganathan, S, Manikhas, A, Bondarenko, I, Mukhametshina, G, Nemsadze, G, Parra, JD, Abesamis-Tiambeng, ML, Baramidze, K, Akewanlop, C, Vynnychenko, I, Sriuranpong, V, Mamillapalli, G, Ray, S, Yanez Ruiz, EP; Pennella, E	2017
TRIBUTE: a phase III trial of erlotinib hydrochloride (OSI-774) combined with carboplatin and paclitaxel chemotherapy in advanced non-small-cell lung cancer	Herbst, , RS, Prager, D, Hermann, R, Fehrenbacher, L, Johnson, BE, Sandler, A, Kris, MG, Tran, HT, Klein, P, Li, X, Ramies, D, Johnson, DH; Miller, VA	2005
Evaluation of stress-related hormones after surgery by laparoscopy or laparotomy	Muzii, , L, Marana, R, Marana, E, Paielli, FV, Meo, F, Maussier, ML, Sciarra, M; Mancuso, S	1996
Immediate incubation of blood cultures outside routine laboratory hours of operation accelerates antibiotic switching	Kerremans, , JJ, van der Bij, AK, Goessens, W, Verbrugh, HA; Vos, MC	2009
Randomized controlled trial of febuxostat versus allopurinol or placebo in individuals with higher urinary uric acid excretion and calcium stones	Goldfarb, , DS, MacDonald, PA, Gunawardhana, L, Chefo, S; McLean, L	2013
Treatment of transsphincteric fistula-in-ano with growth factors from autologous platelets: results of a phase II clinical trial	de la Portilla, , F, Segura-Sampedro, JJ, Reyes-Diaz, ML, Maestre, MV, Cabrera, AM, Jimenez-Rodriguez, RM, Vazquez-Monchul, JM, Diaz-Pavon, JM; Padillo-Ruiz, FJ	2017
MERIBEL study: single-agent eribulin as first-line therapy for taxane-resistant HER2 metastatic breast cancer (MBC) patients (pts)	Ortega, , V, Lao, J, Garau, I, Afonso, N, Calvo, L, Fernandez, Y, Martinez-Garcia, M, Blanco, E, Zamora, P, Garcia, M, Illarramendi, JJ, Rodriguez, C, Aguirre, E, Perez, J, Cortes Castan, J; Llombart-Cussac, A	2016
Acupuncture to Prevent Chemotherapy Dose Reduction Due to Chemotherapy-induced Peripheral Neuropathy in Breast and Colorectal Cancer Patients (GCC1232)	NCT01881932	2013
Serial enumeration of circulating tumor cells predicts treatment response and prognosis in metastatic breast cancer: a prospective study in 393 patients	Wallwiener, , M, Riethdorf, S, Hartkopf, AD, Modugno, C, Nees, J, Madhavan, D, Sprick, MR, Schott, S, Domschke, C, Baccelli, I, Schonfisch, B, Burwinkel, B, Marme, F, Heil, J, Sohn, C, Pantel, K, Trumpp, A; Schneeweiss, A	2014
Safety and efficacy of intensive intraoperative glycaemic control in cardiopulmonary bypass surgery: a randomised trial	Rujirojindakul, , P, Liabsuetrakul, T, McNeil, E, Chanchayanon, T, Wasinwong, W, Oofuvong, M, Rergkliang, C; Chittithavorn, V	2014
Improvement of short- and long-term outcomes for very low birth weight infants: edmonton NIDCAP trial	Peters, , KL, Rosychuk, RJ, Hendson, L, Coté, JJ, McPherson, C; Tyebkhan, JM	2009
Trial of Radiofrequency Thermo-ablation Treatments of Great Saphenous Varicose Veins (3-RF Study)	NCT02441881	2015
A randomized pilot trial testing the safety and immunologic effects of a MAGE-A3 protein plus AS15 immunostimulant administered into muscle or into dermal/subcutaneous sites	Slingluff, , CL, Petroni, GR, Olson, WC, Smolkin, ME, Chianese-Bullock, KA, Mauldin, IS, Smith, KT, Deacon, DH, Varhegyi, NE, Donnelly, SB, Reed, CM, Scott, K, Galeassi, NV; Grosh, WW	2016
Secukinumab for ankylosing spondylitis and psoriatic arthritis	Lubrano, , E; Perrotta, FM	2016
Risk adapted therapy of AML in MRC/NCRI trials	Russell, , NH; Hills, RK	2017
The posttrial effect of oral periodic presumptive treatment for vaginal infections on the incidence of bacterial vaginosis and Lactobacillus colonization	Balkus, , JE, Jaoko, W, Mandaliya, K, Richardson, BA, Masese, L, Gitau, R, Kiarie, J, Marrazzo, J, Farquhar, C; McClelland, RS	2012
Temporary epicardial cardiac resynchronisation versus conventional right ventricular pacing after cardiac surgery: study protocol for a randomised control trial	Russell, , SJ, Tan, C, O'Keefe, P, Ashraf, S, Zaidi, A, Fraser, AG; Yousef, ZR	2012
Results of 5-year follow-up study in patients with peripheral artery disease treated with PL-VEGF165 for intermittent claudication	Deev, , R, Plaksa, I, Bozo, I, Mzhavanadze, N, Suchkov, I, Chervyakov, Y, Staroverov, I, Kalinin, R; Isaev, A	2018
Prospective safety surveillance of GH-deficient adults: comparison of GH-treated vs untreated patients	Hartman, , ML, Xu, R, Crowe, BJ, Robison, LL, Erfurth, EM, Kleinberg, DL, Zimmermann, AG, Woodmansee, WW, Cutler Jr, GB, Chipman, JJ; Melmed, S	2013
Health Status After Transcatheter or Surgical Aortic Valve Replacement in Patients With Severe Aortic Stenosis at Increased Surgical Risk: results From the CoreValve US Pivotal Trial	Arnold, , SV, Reynolds, MR, Wang, K, Magnuson, EA, Baron, SJ, Chinnakondepalli, KM, Reardon, MJ, Tadros, PN, Zorn, GL, Maini, B, Mumtaz, MA, Brown, JM, Kipperman, RM, Adams, DH, Popma, JJ; Cohen, DJ	2015

Effects of a mouthwash with chlorine dioxide on oral malodor and salivary bacteria: a randomized placebo-controlled 7-day trial	Shinada, , K, Ueno, M, Konishi, C, Takehara, S, Yokoyama, S, Zaitsu, T, Ohnuki, M, Wright, FA; Kawaguchi, Y	2010
An intensive cardiovascular rehabilitation programme improves maximal oxygen uptake and quality of life in Danish patients with ischaemic heart disease	Skov, , J, Sidelmann, JJ, Pedersen, SFM, Fournaise, AV; Bladbjerg, EM	2012
Morphological, Functional, and Biological Vascular Healing Response 6 Months After Drug-Eluting Stent Implantation: a Randomized Comparison of Three Drug-Eluting Stents	Nakata, , T, Fujii, K, Fukunaga, M, Shibuya, M, Kawai, K, Kawasaki, D, Naito, Y, Ohyanagi, M; Masuyama, T	2016
Short-term effects of NPH insulin, insulin detemir, and insulin glargine on the GH-IGF1-IGFBP axis in patients with type 1 diabetes	Ma, , Z, Christiansen, JS, Laursen, T, Lauritzen, T; Frystyk, J	2014
Efficacy and Tolerability of Subcutaneous Methylnaltrexone in Patients with Advanced Illness and Opioid-Induced Constipation: a Responder Analysis of 2 Randomized, Placebo-Controlled Trials	Nalamachu, , SR, Pergolizzi, J, Taylor, R, Slatkin, NE, Barrett, AC, Yu, J, Bortey, E, Paterson, C; Forbes, WP	2015
A pilot study of the short term effects of testosterone in infants with 47,XXY klinefelter syndrome	Davis, , SM, Reynolds, R, Martin, S, Howell, S, Nokoff, NJ, Zeitler, PS; Tartaglia, N	2017
Efficacy of a triclosan formula in controlling early subgingival biofilm formation: a randomized trial	Andrade, , E, Weidlich, P, Angst, PD, Gomes, SC; Oppermann, RV	2015
A randomized study comparing filgrastim versus lenograstim versus molgramostim plus chemotherapy for peripheral blood progenitor cell mobilization	Kopf, , B, De Giorgi, U, Vertogen, B, Monti, G, Molinari, A, Turci, D, Dazzi, C, Leoni, M, Tienghi, A, Cariello, A, Argnani, M, Frassineti, L, Scarpi, E, Rosti, G; Marangolo, M	2006
Intravenous lipid dose and incidence of bacteremia and fungemia in patients undergoing bone marrow transplantation	Lenssen, , P, Bruemmer, BA, Bowden, RA, Gooley, T, Aker, SN; Mattson, D	1998
Mechanism of Indocyanine Green-based Photodynamic Therapy for Treating Periodontitis	NCT03557827	2018
Overall survival of patients with relapsed multiple myeloma treated with panobinostat or placebo plus bortezomib and dexamethasone (the PANORAMA 1 trial): a randomised, placebo-controlled, phase 3 trial	San-Miguel, , JF, Hungria, VT, Yoon, SS, Beksac, M, Dimopoulos, MA, Elghandour, A, Jedrzejczak, WW, Günther, A, Nakorn, TN, Siritanaratkul, N, Schlossman, RL, Hou, J, Moreau, P, Lonial, S, Lee, JH, Einsele, H, Sopala, M, Bengoudifa, BR, Binlich, F; Richardson, PG	2016
Tight Glycemic Control Reduces Heart Inflammation and Remodeling During Acute Myocardial Infarction in Hyperglycemic Patients	Marfella, , R, Di Filippo, C, Portoghese, M, Ferraraccio, F, Rizzo, MR, Siniscalchi, M, Musacchio, E, D'Amico, M, Rossi, F; Paolisso, G	2009
Effect of recombinant human growth hormone with total parenteral nutrition on albumin synthesis in patients with peritoneal sepsis	Li, , W, Li, J, Xu, B, Yin, L, Wang, L, Gu, J, Ren, J; Quan, Z	1998
Fiblast (trafermin) in acute stroke: results of the European-Australian phase II/III safety and efficacy trial	Bogousslavsky, , J, Victor, SJ, Salinas, EO, Pallay, A, Donnan, GA, Fieschi, C, Kaste, M, Orgogozo, JM, Chamorro, A; Desmet, A	2002
Acute effects of very-low-protein diet on FGF23 levels: a randomized study	Di Iorio, , B, Di Micco, L, Torraca, S, Sirico, ML, Russo, L, Pota, A, Mirenghi, F; Russo, D	2012
Eribulin across multiple lines of chemotherapy: a retrospective study on quality of life and efficacy in metastatic breast cancer patients	Quaquarini, , E, Sottotetti, F, D'Ambrosio, D, Malovini, A, Morganti, S, Marinello, A, Pavesi, L; Frascaroli, M	2017
A prospective randomised study on endotoxaemia, mediator release and morbidity in conventional, compared with laparoscopic cholecystectomy	Bolke, , E, Jehle, PM, Nothnagel, B, Seidelmann, M, Storck, M; Orth, K	2000
Effects of bovine serum concentrate, with or without supplemental micronutrients, on the growth, morbidity, and micronutrient status of young children in a low-income, peri-urban Guatemalan community	Bégin, , F, Santizo, MC, Peerson, JM, Torún, B; Brown, KH	2008
The clinical evaluation of Platelet-Rich Plasma effects on Free Gingival Graft's donor site wound healing	IRCT138812093451N1	2011
Aspirin plus calcium supplementation to prevent superimposed preeclampsia: a randomized trial	Souza, , EV, Torloni, MR, Atallah, AN, Santos, GM, Kulay, L; Sass, N	2014
Influence of exercise training on proangiogenic TIE-2 monocytes and circulating angiogenic cells in patients with peripheral arterial disease	Dopheide, , JF, Geissler, P, Rubrech, J, Trumpp, A, Zeller, GC, Daiber, A, Münzel, T, Radsak, MP; Espinola-Klein, C	2016
Safety and efficacy of intravenous ultra-high dose methylcobalamin treatment for peripheral neuropathy: a phase I/II open label clinical trial	Shibuya, , K, Misawa, S, Nasu, S, Sekiguchi, Y, Beppu, M, Iwai, Y, Mitsuma, S, Isose, S, Arimura, K, Kaji, R; Kuwabara, S	2014
Feeding late and moderately preterm infants	ISRCTN15469594	2018
Magnesium sulphate in continuous administration compare to discontinuous administration in the expectant management of severe preeclampsia: randomized controlled trial	EUCTR2011-002095-17-ES	2011
Differentially Enhancing Effects of Long-term Treatment with Serrazyme, Boswellia and Pine on Seminal Bacterial Detection in Patients with Chronic Bacterial or Inflammatory Prostatitis, Probably Related to Several Degrees of Bacterial Adherence	Vicari, , E, Malaguarnera, G, Vicari, BO, Salmeri, M, Salemi, M; Castiglione, R	2018
An international multi-centre open-label 2-arm phase III trial of adjuvant bevacizumab in triple negative breast cancer. - BEATRICE	EUCTR2007-001128-11-AT	2007
A clinical trial assessing the effect of a new antibiotic (Solithromycin) on inflammation in the small air passages of patients with chronic obstructive pulmonary disease	EUCTR2014-003077-42-GB	2015
Cell therapy for cardiac repair through mobilization of hematopoietic stem cells and endothelial progenitors in patients with chronic ischemic cardiomyopathy. - NA	EUCTR2004-004545-18-BE	2005
Clinical evaluation of the efficacy of a novel wound dressing on treatment of wagner ulcer grade II	IRCT2016071328903N1	2016
Prevention of dialysate-induced peritoneal damage by pyridoxal phosphate	JPRN-UMIN000001287	2008
Dose finding study of OMT-28 in subjects with persistent atrial fibrillation	EUCTR2018-001626-26-HU	2019

ATTACHE: a Trial in the Timing of Surgery and Adjuvant Chemotherapy for Hepatic Metastases from Colorectal Cancer	ACTRN12610000647033	2010
Effects of non-surgical periodontal treatment on clinical, immunological responses and metabolic control in type 2 diabetic patients: a randomized study	ChiCTR-TRC-10001062	2010
The clinical efficacy of non-surgical periodontal debridement alone, adjunctive systemic azithromycin, or amoxicillin-metronidazole therapy in patients with chronic moderate-to-advanced periodontitis	ACTRN12618000957291	2018
Effect of probiotic supplementation on presence of supplemented bacteria in breast milk, some of milk's immune and oxidative factors, mothers' nutritional status and infants' growth	IRCT201110181197N12	2011
Endophthalmitis Post Intravitreal Injections	NCT04035369	2019
The effect of introduction of umbilical Doppler recordings to obstetric practice	Johnstone, , FD, Prescott, R, Hoskins, P, Greer, IA, McGlew, T; Compton, M	1993
Liraglutide to Short Bowel Syndrome, a cross-over study	EUCTR2015-000825-35-DK	2015
Low level laser effect on improving the blood flow in diabetic foot ulcers	IRCT2014051717723N1	2015
A Phase 2 placebo-controlled study to evaluate the mechanistic effect, safety, and tolerability of alvelestat (MPH966) in participants with alpha-1 (PiZZ or null genotype/phenotype) antitrypsin deficiency	EUCTR2018-001309-95-ES	2018
Pre-operative volume replacement versus usual care in diabetic patients having coronary artery bypass graft (CABG) surgery: a randomised controlled trial	ISRCTN02159606	2008
Atezolizumab with or without cobimetinib versus regorafenib in previously treated metastatic colorectal cancer (IMblaze370): a multicentre, open-label, phase 3, randomised, controlled trial	Eng, , C, Kim, TW, Bendell, J, Argiles, G, Tebbutt, NC, Di Bartolomeo, M, Falcone, A, Fakih, M, Kozloff, M, Segal, NH, Sobrero, A, Yan, Y, Chang, I, Uyei, A, Roberts, L, Ciardiello, F, Ahn, JB, Asselah, J, Badarinath, S, Baijal, S, Begbie, S, Berry, S, Canon, JL, Carbone, RG, Cervantes, A, Cha, YJ, Chang, K, Chaudhry, A, Chmielowska, E, Cho, SH, Chu, D, Couture, F, Cultrera, J, Cunningham, D, Van Cutsem, E, Cuyle, PJ, Davies, J, Dowden, S, Dvorkin, M, Ganju, V, Garcia, RV, Kerr, R, Kim, TY, King, K, Kortmansky, J, Kozloff, M, Lam, KO, Lee, J, Lee, AS, Lesperance, B, Luppi, G, Ma, B, Maiello, E, Mandanas, R, Marshall, J, Marx, G, Mullamitha, S, Nechaeva, M, Park, JO, Pavlakis, N, Ponce, CG, Potemski, P, Raouf, S, Reeves, J, Segal, N, Siena, S, Smolin, A, Streb, JO, Strickland, A, Szutowicz-Zielinska, E, Tabernero, JM, Tan, B, Valera, JS, Van den Eynde, M, Vergauwe, P, Vickers, M, Womack, M, Wroblewska, M; Young, R	2019
Evaluation of the effect of two gingival treatments in the development of gingival disease in children with heart disease	RBR-9gfmm5	2013
Efficacy and safety of Shen-Yuan-Dan capsules for peri-procedural myocardial injury following percutaneous coronary intervention: study protocol for a randomized, double-blind, placebo-controlled trial	Li, , X, Lai, X-L, Fei, Y-T, Shang, J-J, Zhou, Q, Sun, X-Y, Xing, W-L, Jia, S-H; Liu, H-X	2019
Predictors for use of psychosocial services in patients with metastatic colorectal cancer receiving first line systemic treatment	Schuurhuizen, , CSEW, Braamse, AMJ, Konings, IRHM, Verheul, HMW; Dekker, J	2019
Multi-center, randomized, double-blind, placebo-controlled, cross-over Phase II study to evaluate the safety and efficacy of inhaled Bimosiamose for the treatment of patients with moderate to severe Chronic Obstructive Pulmonary Disease (COPD)	EUCTR2009-017257-35-DE	2009
Oral anabolic steroid increases muscle products in hemodialysis patients	ISRCTN41591818	2012
The Interval between Treatments of Bevacizumab and Dexamethasone Implants for Diabetic Macular Edema Increased over Time in the BEVORDEX Trial	Mehta, , H, Fraser-Bell, S, Nguyen, V, Lim, LL; Gillies, MC	2018
A Randomized (Phase II), Double-blind, Multicenter Phase I/II trial of Pemetrexed, Carboplatin plus or minus Sorafenib in the First-line Treatment of Patients with Stage IIIb or IV Non-Small Cell Lung Cancer - PECASO	EUCTR2006-005970-26-DE	2007
Double-Blind Placebo-Controlled Study of Rifaximin and Lactulose Hydrogen Breath Test in Gulf War Veterans with Irritable Bowel Syndrome	Tuteja, , AK, Talley, NJ, Stoddard, GJ; Verne, GN	2019
The adjunctive effects of Omega-3 dietary supplementation in the treatment of periodontitis	ISRCTN23257928	2015
The role of growth factor administration and T-cell recovery after peripheral blood progenitor cell transplantation in the treatment of solid tumors: results from a randomized comparison of G-CSF and GM-CSF	Pierelli, , L, Perillo, A, Ferrandina, G, Salerno, G, Rutella, S, Fattorossi, A, Battaglia, A, Rughetti, A, Nuti, M, Cortesi, E, Leone, G, Mancuso, S; Scambia, G	2001
Effect of ?-3 fatty acids on gastric cancer patients	IRCT201011095144N1	2011
Effect of dried hydro alcoholic extract of pomegranate pericarp on oxidattive and inflammation state, Metaloproteinase 1, metabolic markers and clinical sign in women with knee osteoarthritis	IRCT201405183664N11	2014
A Phase 3 Study to Compare the Efficacy and Safety of CT-P6 and Herceptin as Neoadjuvant and Adjuvant Treatment in Patients with HER2-Positive Early Breast Cancer	EUCTR2013-004525-84-PL	2014

Clinical Safety and Preliminary Efficacy of Plasmid pUDK-HGF Expressing Human Hepatocyte Growth Factor (HGF) in Patients with Critical Limb Ischemia	Cui, , S, Guo, L, Li, X, Gu, Y, Fu, J, Dong, L, Song, H, Chen, X, Lu, Y, Hu, C, Xiao, F, Zhu, D, Wu, Z; Zhang, Q	2015
Study to Evaluate Imetelstat (JNJ-63935937) in Subjects with IPSS Low or Intermediate-1 Risk Myelodysplastic Syndrome (MDS)	EUCTR2015-002874-19-DE	2015
Effect of carvedilol medication and vitamins E and C in patients with Chagas' disease	RBR-95jnqp	2012
A Phase II Study of Efficacy of Rabbit Anti-thymocyte Globulin (rATG) in patients with Low and Itermediate-1 Risk Mylodysplastic Syndrome - Genzyme ThymoHEMO1206: phase II study of Thymoglobulin in MDS patients	EUCTR2007-002532-28-NL	2008
CORE Trial	ISRCTN45961438	2016
Targeting on the values of Wolbachia endosymbionts as novel feasible anti-filarial chemotherapeutic approach to prevent/reduce/clear disease in lymphatic filariasis and interrupt transmission in endemic communities of Tanzania	ISRCTN12273637	2009
A study of test product setrusumab in adults with brittle bone syndrome	EUCTR2016-005096-27-DK	2017
Leukocyte filter enhances neutrophil activation during combined aortic valve and coronary artery bypass surgery	Koskenkari, , JK, Rimpiläinen, J, Ohman, H, Surcel, HM, Vainionpää, V, Biancari, F, Ala-Kokko, T; Juvonen, T	2006
Effect of essential fatty acids on natural cytotoxicity in patients with colorectal cancer	Purasiri, , P, Ashby, J, Heys, SD; Eremin, O	1995
Epigenetic alterations at diagnosis predict susceptibility, prognosis and treatment escalation in inflammatory bowel disease and IBD character	Kalla, , R, Adams, A, Vatn, S, Bonfiglio, F, Nimmo, E, Kennedy, N, Ventham, N, Vatn, MH, Ricanek, P, Bergemalm, D, Halfvarson, J, Soderholm, J, Pierik, M, Torkvist, L, Gomollon, F, Gut, I, Jahnsen, J; Satsangi, J	2017
Sarcopenia Improves the Muscle Mass and Muscle Strength of Patients With Liver Cirrhosis-Child C	NCT03633279	2018
Ezetimibe and simvastatin combination inhibits and reverses the pro-inflammatory and pro-atherogenic effects of cream in obese patients	Ghanim, , H, Green, K, Abuaysheh, S, Patel, R, Batra, M, Chaudhuri, A, Makdissi, A, Kuhadiya, ND; Dandona, P	2017
Short-term effects of an anti-inflammatory treatment on clinical parameters and serum levels of C-reactive protein and proinflammatory cytokines in subjects with periodontitis	Renvert, , S, Lindahl, C, Roos-Jansåker, AM; Lessem, J	2009
Surgical and not analgesic technique affects postoperative inflammation following colorectal cancer surgery: a prospective, randomized study	Siekmann, , W, Eintrei, C, Magnuson, A, Sjölander, A, Matthiessen, P, Myrelid, P; Gupta, A	2017
Effects of minimal dose aprotinin on blood loss and fibrinolytic system-complement activation in coronary artery bypass grafting surgery	Cicekcioglu, , F, Cagli, K, Emir, M, Topbas, M, Catav, Z, Sener, E; Tasdemir, O	2006
Quetiapine and flupentixol differentially improve anterior cingulate cortex function in schizophrenia patients: an event-related potential study	Schneider, , S, Bahmer, TJ, Metzger, FG, Reif, A, Polak, T, Pfuhlmann, B, Walter, G, Eberle, MC, Ernst, LH, Fallgatter, AJ; Ehlis, AC	2013
Limited efficacy of thalidomide in the treatment of febrile attacks of the hyper-IgD and periodic fever syndrome: a randomized, double-blind, placebo-controlled trial	Drenth, , JP, Vonk, AG, Simon, A, Powell, R; van der Meer, JW	2001
Non-surgical periodontal therapy with and without subgingival minocycline administration in patients with poorly controlled type II diabetes: a randomized controlled clinical trial	Lin, , SJ, Tu, YK, Tsai, SC, Lai, SM; Lu, HK	2012
High-dose insulin therapy attenuates systemic inflammatory response in coronary artery bypass grafting patients	Albacker, , T, Carvalho, G, Schricker, T; Lachapelle, K	2008
Spironolactone for People Age 70 Years and Older With Osteoarthritic Knee Pain: a Proof-of-Concept Trial	Mcmurdo, , ME, Sumukadas, D, Donnan, PT, Cvoro, V, Rauchhaus, P, Argo, I, Waldie, H, Littleford, R, Struthers, AD; Witham, MD	2016
Predictors of post-operative response to treatment: a double blind placebo controlled study in chronic rhinosinusitis patients	Ebbens, , FA, Toppila-Salmi, S, de Groot, EJ, Renkonen, J, Renkonen, R, van Drunen, CM, Dijkgraaf, MG; Fokkens, WJ	2011
Multicenter analysis of neoadjuvant docetaxel, carboplatin, and trastuzumab in HER2-positive breast cancer	Echavarria, , I, Granja, M, Bueno, C, Lopez-Tarruella, S, Peinado, P, Sotelo, M, Jerez, Y, Moreno, F, Torres, G, Lobo, M, Marquez-Rodas, I, Del Monte-Millan, M, Martin, M; Garcia-Saenz, JA	2017
Periodontal treatment decreases levels of antibodies to Porphyromonas gingivalis and citrulline in patients with rheumatoid arthritis and periodontitis	Okada, , M, Kobayashi, T, Ito, S, Yokoyama, T, Abe, A, Murasawa, A; Yoshie, H	2013
A Novel Drug for Treatment of Necrotizing Soft-Tissue Infections: a Randomized Clinical Trial	Bulger, , EM, Maier, RV, Sperry, J, Joshi, M, Henry, S, Moore, FA, Moldawer, LL, Demetriades, D, Talving, P, Schreiber, M, Ham, B, Cohen, M, Opal, S, Segalovich, I, Maislin, G, Kaempfer, R; Shirvan, A	2014
Combined photodynamic and low-level laser therapies as an adjunct to nonsurgical treatment of chronic periodontitis	Lui, , J, Corbet, EF; Jin, L	2011
Characteristics of patients who respond poorly to reduction of biological disease-modifying anti-rheumatic drugs in rheumatoid arthritis; Rt-4 study post hoc analysis	Urata, , Y, Abe, S, Devers, B, Nakamura, Y, Takemoto, H; Furukawa, K-I	2017
Prevention of postoperative recurrence with azathioprine or infliximab in patients with Crohn's disease: an open-label pilot study	Armuzzi, , A, Felice, C, Papa, A, Marzo, M, Pugliese, D, Andrisani, G, Federico, F, De Vitis, I, Rapaccini, GL; Guidi, L	2013
Joint Inflammatory Bowel Disease-Obstetric clinics: outcomes in 95 pregnancies at a tertiary centre over a 3-year period	Chew, , TS, Carmichael, MS, Hoare, TW, Waugh, J; Gunn, MC	2017
Preoperative steroid treatment does not improve markers of inflammation after cardiac surgery in neonates: results from a randomized trial	Graham, , EM, Atz, AM, McHugh, KE, Butts, RJ, Baker, NL, Stroud, RE, Reeves, ST, Bradley, SM, McGowan, FX; Spinale, FG	2014
Evaluation of a protease inhibitor in the prevention of ischemia and reperfusion injury in hepatectomy under intermittent Pringle maneuver	Kim, , YI, Chung, HJ, Song, KE, Hwang, YJ, Lee, JW, Lee, YJ; Chun, BY	2006

Efficacy and tolerability of an undenatured type II collagen supplement in modulating knee osteoarthritis symptoms: a multicenter randomized, double-blind, placebo-controlled study	Lugo, , JP, Saiyed, ZM; Lane, NE	2016
Doppler tissue imaging is an independent predictor of outcome in patients with ST-segment elevation myocardial infarction treated with primary percutaneous coronary intervention	Biering-Sørensen, , T, Jensen, JS, Pedersen, S, Galatius, S, Hoffmann, S, Jensen, MT; Mogelvang, R	2014
The effect of initial treatment of periodontitis on systemic markers of inflammation and cardiovascular risk: a randomized controlled trial	Taylor, , B, Tofler, G, Morel-Kopp, MC, Carey, H, Carter, T, Elliott, M, Dailey, C, Villata, L, Ward, C, Woodward, M; Schenck, K	2010
GnRH-a and Pregnancy Rate in In Vitro Fertilization (IVF) Cycles	NCT01269125	2011
Transvaginal/transumbilical hybrid–NOTES–versus 3-trocar needlescopic cholecystectomy: short-term results of a randomized clinical trial	Bulian, , DR, Knuth, J, Cerasani, N, Sauerwald, A, Lefering, R; Heiss, MM	2015
Controversy of hormone treatment and cardiovascular function: need for strengthened collaborations between preclinical and clinical scientists	Miller, , VM, Shuster, LT; Hayes, SN	2003
Prospective randomized trial evaluating mandatory second look surgery with HIPEC and CRS vs. standard of care in patients at high risk of developing colorectal peritoneal metastases	Ripley, , RT, Davis, JL, Kemp, CD, Steinberg, SM, Toomey, MA; Avital, I	2010
Post Implantation Syndrome and Administration on NSAIDs in Patients Undergoing EVAR for AAA	NCT03727412	2018
Comparison of Functional Recovery Between Laparoscopic and Open Pancreaticoduodenectomy	NCT03870698	2019
Association between biochemical cartilage markers and clinical symptoms in patients with hip osteoarthritis: cohort study with two-year follow-up	Dorleijn, , DM, Luijsterburg, PA, Bay-Jensen, AC, Siebuhr, AS, Karsdal, M, Rozendaal, RM, Bos, PK; Bierma-Zeinstra, SM	2014
Protective effect of propofol and its relation to postoperation recovery in children undergoing cardiac surgery with cardiopulmonary bypass	Xia, , WF, Liu, Y, Zhou, QS, Tang, QZ; Zou, HD	2011
The importance of the tumor marker CYFRA 21-1 in patients with lung cancer after surgery or chemotherapy	Zissimopoulos, , A, Stellos, K, Permenopoulou, V, Petrakis, G, Theodorakopoulos, P, Baziotis, N; Thalassinos, N	2007
Homeopathy for Perennial Asthma in Adolescents: pilot Feasibility Study Testing a Randomised Withdrawal Design	Mitchiguian Hotta, , L, Cardinalli Adler, U, de Toledo Cesar, A, Martinez, EZ; Demarzo, MMP	2018
Effect of Non-surgical Periodontal Treatment on HbA1c in Type 2 Diabetic Patients	NCT02652026	2015
The role of genetic-based prognostic factors in predicting minimal residual disease negativity in chronic lymphocytic leukemia patients treated with fludarabine, cyclophosphamide and ofatumumab	Raponi, , S, Del Giudice, I, Ilari, C, Luciana, C, Della Starza, I, De Propris, MS, Nanni, M, Cavalli, M, De Novi, LA, Mariglia, P, Mancini, F, Nardacci, MG, Piciocchi, A, Albano, F, Specchia, G, Cuneo, A, Fabris, S, Neri, A, Vignetti, M, Mauro, FR, Guarini, A; Foa, R	2017
Novel PAradigm to improve Inflammatory burden in end stage Renal disease (rePAIR): study protocol for a randomized controlled trial	Trivedi, , R, Fares, G, Nunez, VB, Campbell, R, Clement, M, Burleson, J, Himmelfarb, J; Ioannidou, E	2018
A prospective randomized study of systemic inflammation and immune response after laparoscopic nissen fundoplication performed with standard and low-pressure pneumoperitoneum	Schietroma, , M, Carlei, F, Cecilia, EM, Piccione, F, Sista, F, De Vita, F; Amicucci, G	2013
AMPLIFYing NEOepitope-specific VACcine Responses in Progressive Diffuse Glioma	NCT03893903	2019
Effective flow performances and dialysis doses delivered with permanent catheters: a 24-month comparative study of permanent catheters versus arterio-venous vascular accesses	Canaud, , B, Leray-Moragues, H, Kerkeni, N, Bosc, JY; Martin, K	2002
N-Acetylcysteine inhalation improves pulmonary function in patients received liver transplantation	Li, , X, Wei, X, Chen, C, Zhang, Z, Liu, D, Hei, Z; Yao, W	2018
Protein array profiling of circulating angiogenesis-related factors during bevacizumab containing treatment in metastatic colorectal cancer	Hagman, , H, Bendahl, P-O, Lidfeldt, J, Belting, M; Johnsson, A	2018
Re: 'Long-term Results of a Randomized Controlled Trial Analyzing the Role of Systematic Pre-operative Coronary Angiography before Elective Carotid Endarterectomy in Patients with Asymptomatic Coronary Artery Disease'	Galyfos, , G, Sigala, F, Aggeli, K; Filis, K	2015
SHINBARO, a new herbal medicine with multifunctional mechanism for joint disease: first therapeutic application for the treatment of osteoarthritis	Lee, , S-Y, Kwon, H-K; Lee, S-M	2011
Systemic cytokine response after laparoscopic-assisted resection of rectosigmoid carcinoma: a prospective randomized trial	Leung, , KL, Lai, PB, Ho, RL, Meng, WC, Yiu, RY, Lee, JF; Lau, WY	2000
The anti-rheumatic effect of multiple synovectomy in patients with refractory rheumatoid arthritis	Nakamura, , H, Nagashima, M, Ishigami, S, Wauke, K; Yoshino, S	2000
The effects of cardiopulmonary bypass temperature on inflammatory response following cardiopulmonary bypass	Birdi, , I, Caputo, M, Underwood, M, Bryan, AJ; Angelini, GD	1999
7-day Atorvastatin and Emotional Processing	NCT03966859	2019
Treatment of adult chronic indeterminate Chagas disease with benznidazole and three E1224 dosing regimens: a proof-of-concept, randomised, placebo-controlled trial	Torrico, , F, Gascon, J, Ortiz, L, Alonso-Vega, C, Pinazo, MJ, Schijman, A, Almeida, IC, Alves, F, Strub-Wourgaft, N; Ribeiro, I	2018
Infusion of the allogeneic cell line NK-92 in patients with advanced renal cell cancer or melanoma: a phase I trial	Arai, , S, Meagher, R, Swearingen, M, Myint, H, Rich, E, Martinson, J; Klingemann, H	2008
Efficacy of pertuzumab/trastuzumab/paclitaxel over standard trastuzumab/paclitaxel therapy for HER2+ breast cancer: results from the neoadjuvant I-SPY 2 TRIAL	Buxton, , M, DeMichele, AM, Chia, S, Van't Veer, L, Chien, J, Wallace, A, Kaplan, H, Lang, J, Yee, D, Isaacs, C, Moulder, S, Albain, K, Boughey, J, Kemmer, K, Haley, B, Minton, S, Forero, A, Nanda, R, Elias, A, Korde, L, Viscuzi, R, Rugo, H, Schwab, R,	2016

	Symmans, F, Paoloni, M, Hylton, N, Hogarth, M, Lyandres, J, Perlmutter, J, Sanil, A, Yau, C, Esserman, L; Berry, D	
Effect of preoperative oral pravastatin reload in systemic inflammatory response and myocardial damage after coronary artery bypass grafting. A pilot double-blind placebo-controlled study	Castaño, , M, González-Santos, JM, López, J, García, B, Centeno, JE, Aparicio, B, Bueno, MJ, Díez, R, Sagredo, V, Rodríguez, JM; García-Criado, FJ	2015
Real-world data on clinical characteristics, prognosis and outcome of primary plasma cell leukemia: a study of the greek myeloma study group in the era of novel agents	Katodritou, , E, Terpos, E, Delimpasi, S, Kotsopoulou, M, Michalis, E, Kyrtsonis, M-C, Symeonidis, A, Giannakoulas, N, Vadikolia, C, Michael, M, Kalpadakis, C, Gougopoulou, T, Prokopiou, C, Kaiafa, GD, Christoulas, D, Gavriatopoulou, M, Giannopoulou, E, Lampropoulou, V, Verrou, E, Kastritis, E, Konstantinidou, P, Anagnostopoulos, A; Dimopoulos, MA	2016
Phase II trial of pertuzumab, trastuzumab, and nab-paclitaxel in patients (pts) with HER2 overexpressing (HER2+) locally advanced or inflammatory breast cancer (LABC) or untreated stage IV metastatic breast cancer (MBC)	Somlo, , G, Frankel, P, Yeon, C, Yuan, Y, Yim, J, Kruper, L, Taylor, L, Mortimer, J, Waisman, J, Jones, V, Vito, C, Paz, B, Huria, A, Li, D, Gaal, C, Tong, T; Tumyan, L	2017
Dexamethasone: benefit and prejudice for patients undergoing on-pump coronary artery bypass grafting: a study on myocardial, pulmonary, renal, intestinal, and hepatic injury	Morariu, , AM, Loef, BG, Aarts, LP, Rietman, GW, Rakhorst, G, van Oeveren, W; Epema, AH	2005
Sleep-time blood pressure as a therapeutic target for cardiovascular risk reduction in type 2 diabetes	Hermida, , RC, Ayala, DE, Mojon, A; Fernandez, JR	2012
Immunomodulatory effects of acupuncture in the treatment of allergic asthma	Joos, , S, Schott, C, Zou, H, Daniel, V, Martin, E; Brinkhaus, B	1999
Efficacy of brief behavioral counselling by allied health professionals to promote physical activity in people with peripheral arterial disease (BIPP): study protocol for a multi-center randomized controlled trial	Burton, , NW, Ademi, Z, Best, S, Fiatarone Singh, MA, Jenkins, JS, Lawson, KD, Leicht, AS, Mavros, Y, Noble, Y, Norman, P, Norman, R, Parmenter, BJ, Pinchbeck, J, Reid, CM, Rowbotham, SE, Yip, L; Golledge, J	2016
The beta agonist lung injury trial prevention. A randomized controlled trial	Perkins, , GD, Gates, S, Park, D, Gao, F, Knox, C, Holloway, B, McAuley, DF, Ryan, J, Marzouk, J, Cooke, MW, Lamb, SE; Thickett, DR	2014
Next-generation sequencing-based detection of circulating tumour DNA After allogeneic stem cell transplantation for lymphoma	Herrera, , AF, Kim, HT, Kong, KA, Faham, M, Sun, H, Sohani, AR, Alyea, EP, Carlton, VE, Chen, YB, Cutler, CS, Ho, VT, Koreth, J, Kotwaliwale, C, Nikiforow, S, Ritz, J, Rodig, SJ, Soiffer, RJ, Antin, JH; Armand, P	2016
Prospective randomized trial of ACUSEAL versus Vascu-Guard patching in carotid endarterectomy	Stone, , PA, AbuRahma, AF, Mousa, AY, Phang, D, Hass, SM, Modak, A; Dearing, D	2014
Perineural invasion and lymph node involvement as indicators of surgical outcome and pattern of recurrence in the setting of preoperative gemcitabine-based chemoradiation therapy for resectable pancreatic cancer	Takahashi, , H, Ohigashi, H, Ishikawa, O, Gotoh, K, Yamada, T, Nagata, S, Tomita, Y, Eguchi, H, Doki, Y; Yano, M	2012
Effects of low-dose tamoxifen on breast cancer biomarkers Ki-67, estrogen and progesterone receptors	de Sousa, , JA, Facina, G, da Silva, BB; Gebrim, LH	2006
Tumor necrosis factor-alpha blockade in recurrent and disabling chronic sciatica associated with post-operative peridural lumbar fibrosis: results of a two-year double-blind, randomized, controlled study	Nguyen, , C, Sanchez, K, Palazzo, C, Zee, N, Feydy, A, Quinquis, L, Grabar, S, Revel, M, Lefèvre-Colau, M-M, Poiraudeau, S; Rannou, F	2015
Oral steroids and doxycycline: two different approaches to treat nasal polyps	Van Zele, , T, Gevaert, P, Holtappels, G, Beule, A, Wormald, PJ, Mayr, S, Hens, G, Hellings, P, Ebbens, FA, Fokkens, W, Van Cauwenberge, P; Bachert, C	2010
Treatment of locally advanced pancreatic cancer by percutaneous and intraoperative irreversible electroporation: general hospital cancer center experience	Lambert, , L, Horejs, J, Krska, Z, Hoskovec, D, Petruzelka, L, Krechler, T, Kriz, P; Briza, J	2016
Effects of high dose corticosteroids in open rhinoplasty	Gürlek, , A, Fariz, A, Aydoğan, H, Ersöz-Öztürk, A; Evans, GR	2009
Anesthetic type and risk of myocardial infarction after carotid endarterectomy in the Carotid Revascularization Endarterectomy versus Stenting Trial (CREST)	Hye, , RJ, Voeks, JH, Malas, MB, Tom, M, Longson, S, Blackshear, JL; Brott, TG	2016
Effect of immunotherapy and spleen preservation on immunological function in patients with gastric cancer	Okinaga, , K, Iinuma, H, Kitamura, Y, Yokohata, T, Inaba, T; Fukushima, R	2006
Longitudinal and cross-sectional variability in markers of joint metabolism in patients with knee pain and articular cartilage abnormalities	Lohmander, , LS, Dahlberg, L, Eyre, D, Lark, M, Thonar, EJ; Ryd, L	1998
Aged garlic extract improves endothelial function in men with coronary artery disease	Williams, , MJ, Sutherland, WH, McCormick, MP, Yeoman, DJ; de Jong, SA	2005
Treatment of oral cavity and oropharynx squamous cell carcinoma with perilymphatic interleukin-2: clinical and pathologic correlations	De Stefani, , A, Valente, G, Forni, G, Lerda, W, Ragona, R; Cortesina, G	1996
Randomized trial of low-dose interleukin-2 vs. cyclosporine A and interferon-g after high dose chemotherapy with peripheral blood progenitor support in women with high risk primary breast cancer(HRPBC)	Cohen DJ Vahdat LT Zipin, D Lo KS Frederick D Donovan D Sharpe E Savage D Tiersten A Nichols G Troxel A Hesdorffer CS	2004
Influence of cardiopulmonary bypass on the state of cognitive functions in patients with ischemic heart disease	Buziashvili, , YI, Ambat'ello, SG, Aleksakhina, YA; Pashchenkov, MV	2006
The efficacy of the proteolytic medication longidaza in combined treatment of adhesions in patients with genital endometriosis	Yarmolinskaya, , MI, Selkov, SA, Manuilova, TYu, Bezhenar, VF, Rulev, VV, Seljutin, AV; Thazaplizheva, SSh	2015
Combined capcitabine and stereotactic ablative radiotherapy using EUS-guided fiducial placement for locally advanced pancreatic cancer: early experience in a tertiary centre	Phan, , V-A, Nguyen, N, Le, H; Singhal, N	2016
Combined capecitabine and stereotactic ablative radiotherapy using EUS guided fiducial placement for locally advanced pancreatic cancer: early experience in a tertiary centre	Phan, , VA, Le, H, Singhal, N; Nguyen, N	2016

Effect of periodontal treatment on the clinical parameters of patients with rheumatoid arthritis: study protocol of the randomized, controlled ESPERA trial	Monsarrat, , P, Vergnes, JN, Cantagrel, A, Algans, N, Cousty, S, Kémoun, P, Bertrand, C, Arrivé, E, Bou, C, Sédarat, C, Schaeverbeke, T, Nabet, C; Sixou, M	2013
The periprocedural myocardial damage prevention during elective percutaneous coronary intervention as a result of pharmacological preconditioning with an oral form of nicorandil in patients with stable coronary artery disease. Pilot study	Gostishchev, , RV, Soboleva, GN, Samko, AN, Rogoza, AN; Minasyan, AA	2018
Aerobic exercise training reduces arterial stiffness in metabolic syndrome	Donley, , DA, Fournier, SB, Reger, BL, DeVallance, E, Bonner, DE, Olfert, IM, Frisbee, JC; Chantler, PD	2014
Immunologic and clinical outcomes of vaccination with a multiepitope melanoma peptide vaccine plus low-dose interleukin-2 administered either concurrently or on a delayed schedule	Slingluff, , CL, Petroni, GR, Yamshchikov, GV, Hibbitts, S, Grosh, WW, Chianese-Bullock, KA, Bissonette, EA, Barnd, DL, Deacon, DH, Patterson, JW, Parekh, J, Neese, PY, Woodson, EM, Wiernasz, CJ; Merrill, P	2004
The effects of Crenotherapy and exercise in peripheral arterial occlusive disease. A comparison with simple exercise training	Quarto, , G, Amato, B, Serra, R, Benassai, G, Monti, MG, Salzano, A, D'Assante, R; Furino, E	2017
Perioperative Simvastatin Therapy in Major Colorectal Surgery: a Prospective, Double-Blind Randomized Controlled Trial	Singh, , PP, Lemanu, DP, Soop, M, Bissett, IP, Harrison, J; Hill, AG	2016
Infliximab prevents Crohn's disease recurrence after ileal resection	Regueiro, , M, Schraut, W, Baidoo, L, Kip, KE, Sepulveda, AR, Pesci, M, Harrison, J; Plevy, SE	2009
Recombinant human soluble thrombomodulin for acute exacerbation of idiopathic pulmonary fibrosis: a historically controlled study	Sakamoto, , S, Shimizu, H, Isshiki, T, Sugino, K, Kurosaki, A; Homma, S	2018
Does inchinkoto, a herbal medicine, have hepatoprotective effects in major hepatectomy? A prospective randomized study	Mizutani, , T, Yokoyama, Y, Kokuryo, T, Ebata, T, Igami, T, Sugawara, G; Nagino, M	2015
The effect of intravenous vitamin C infusion on periprocedural myocardial injury for patients undergoing elective percutaneous coronary intervention	Wang, , ZJ, Hu, WK, Liu, YY, Shi, DM, Cheng, WJ, Guo, YH, Yang, Q, Zhao, YX; Zhou, YJ	2014
Low preoperative hepcidin concentration as a risk factor for mortality after cardiac surgery: a pilot study	Haase-Fielitz, , A, Plaß, M, Kuppe, H, Hetzer, R, Ostland, V, Westphal, S, Hoffmann, J, Prowle, J, Mertens, PR, Westerman, M, Bellomo, R; Haase, M	2013
Diffuse large B-cell lymphoma: the relevance of gene expression profiling	Pileri, , SA, Melle, F, Motta, G, Fabbri, M, Tabanelli, V, Calleri, A, Bruna, R, Derenzini, E, Corsini, C, Agostinelli, C, Cesano, A, Cortelazzo, S, Gianni, AM, Tarella, C, Rambaldi, A, Chiappella, A; Vitolo, U	2017
Long-term follow-up of a prospective trial of intensified chemo-immunotherapy with autologous or allogeneic stem cell transplantation in patients affected by peripheral T-cell lymphoma	Corradini, , P, Vitolo, U, Rambaldi, A, Miceli, R, Patriarca, F, Gallamini, A, Benedetti, F, Todeschini, G, Rossi, G, Salvi, F, Bruno, B, Tarella, C, Pileri, S; Dodero, A	2017
Humoral rejection in kidney transplantation: new concepts in diagnosis and treatment	Mauiyyedi, , S; Colvin, RB	2002
Cardiotomy suction, but not open venous reservoirs, activates coagulofibrinolysis in coronary artery surgery	Nakahira, , A, Sasaki, Y, Hirai, H, Matsuo, M, Morisaki, A, Suehiro, S; Shibata, T	2011
Alpha lipoic acid attenuates inflammatory response during extracorporeal circulation	Uyar, , IS, Onal, S, Akpinar, MB, Gonen, I, Sahin, V, Uguz, AC; Burma, O	2013
Role of Antioxidants Supplementation in Chronic Pancreatitis	NCT00319358	2006
Demonstration and operative influence of low prime volume closed pump	Takai, , H, Eishi, K, Yamachika, S, Hazama, S, Ariyoshi, T; Nishi, K	2005
Effect of Daikenchuto, a Traditional Japanese Herbal Medicine, after Total Gastrectomy for Gastric Cancer: a Multicenter, Randomized, Double-Blind, Placebo-Controlled, Phase II Trial	Yoshikawa, , K, Shimada, M, Wakabayashi, G, Ishida, K, Kaiho, T, Kitagawa, Y, Sakamoto, J, Shiraishi, N, Koeda, K, Mochiki, E, Saikawa, Y, Yamaguchi, K, Watanabe, M, Morita, S, Kitano, S, Saji, S, Kanematsu, T; Kitajima, M	2015
Vertical Sleeve Gastrectomy and Lifestyle Modification for the Treatment of Non-Alcoholic Steatohepatitis	NCT03587831	2018
The Post-Anaesthesia N-acetylcysteine Cognitive Evaluation (PANACEA) trial: study protocol for a randomised controlled trial	Skvarc, , DR, Dean, OM, Byrne, LK, Gray, LJ, Ives, K, Lane, SE, Lewis, M, Osborne, C, Page, R, Stupart, D, Turner, A, Berk, M; Marriott, AJ	2016
Effects of tofacitinib on lymphocytes in rheumatoid arthritis: relation to efficacy and infectious adverse events	Sonomoto, , K, Yamaoka, K, Kubo, S, Hirata, S, Fukuyo, S, Maeshima, K, Suzuki, K, Saito, K; Tanaka, Y	2014
Pimecrolimus cream in the long-term management of atopic dermatitis in adults: a six-month study	Meurer, , M, Fölster-Holst, R, Wozel, G, Weidinger, G, Jünger, M; Bräutigam, M	2002
Pharmacological prevention of the deleterious effects of cardiopulmonary bypass	Hassantash, , SA, Omrani, GR, Givtaj, N; Afrakhteh, M	2007
Inhibition of inflammation mediates the protective effect of atorvastatin reload in patients with coronary artery disease undergoing noncardiac emergency surgery	Qu, , Y, Wei, L; Zhang, H	2014
Noninvasive markers of liver fibrosis: on-treatment changes of serum markers predict the outcome of antifibrotic therapy	Tanwar, , S, Trembling, PM, Hogan, BJ, Srivastava, A, Parkes, J, Harris, S, Grant, P, Nastouli, E, Ocker, M, Wehr, K, Herold, C, Neureiter, D, Schuppan, D; Rosenberg, WM	2017
Intravenous immunoglobulin for ANCA-associated systemic vasculitis with persistent disease activity	Jayne, , DR, Chapel, H, Adu, D, Misbah, S, O'Donoghue, D, Scott, D; Lockwood, CM	2000
Randomized controlled trial of preoperative oral carbohydrate treatment in major abdominal surgery	Mathur, , S, Plank, LD, McCall, JL, Shapkov, P, McIlroy, K, Gillanders, LK, Merrie, AE, Torrie, JJ, Pugh, F, Koea, JB, Bissett, IP; Parry, BR	2010
Late remote ischemic preconditioning in children undergoing cardiopulmonary bypass: a randomized controlled trial	Pavione, , MA, Carmona, F, de Castro, M; Carlotti, AP	2012
Rectal administration of Lactobacillus casei DG modifies flora composition and Toll-like receptor expression in colonic mucosa of patients with mild ulcerative colitis	D'Incà, , R, Barollo, M, Scarpa, M, Grillo, AR, Brun, P, Vettorato, MG, Castagliuolo, I; Sturniolo, GC	2011

Systemic inflammatory response related to cardiopulmonary bypass and its modification by methyl prednisolone: high dose versus low dose	Varan, , B, Tokel, K, Mercan, S, Dönmez, A; Aslamaci, S	2002
The role of parenteral glutamine supplement for surgical patient perioperatively: result of a single center, prospective and controlled study	Yeh, , CN, Lee, HL, Liu, YY, Chiang, KC, Hwang, TL, Jan, YY; Chen, MF	2008
Decreasing asleep ambulatory blood pressure reduces cardiovascular risk in chronic kidney disease	Hermida, , RC, Ayala, DE, Mojon, A; Fernandez, JR	2012
Lack of renal protection of ultrafiltration during cardiac surgery: a randomized clinical trial	Foroughi, , M, Argani, H, Hassntash, SA, Hekmat, M, Majidi, M, Beheshti, M, Mehdizadeh, B; Yekani, B	2014
Phase-II randomized study of preoperative IL-2 administration in radically operable gastric cancer patients	Romano, , F, Piacentini, MG, Franciosi, C, Caprotti, R, De Fina, S, Cesana, G, Uggeri, F, Conti, M; Uggeri, F	2004
Health Benefits of HIT for Breast Cancer Patients	NCT03176888	2017
A study to assess if a new antiviral drug called FF-3 is safe and can protect healthy volunteers against infection with a type of flu virus	EUCTR2015-001103-31-GB	2015
An early phase clinical study to investigate the combination of chemotherapy (Gemcitabine) and the study drug Atu027 in patients with advanced or metastatic pancreas cancer	EUCTR2012-004429-26-DE	2012
Femoral-Express-I - FemExp-I	EUCTR2009-012676-27-SE	2009
Peripheral artery disease, biomarkers, and darapladib	Berger, , JS, Ballantyne, CM, Davidson, MH, Johnson, JL, Tarka, EA, Lawrence, D, Trivedi, T, Zalewski, A; Mohler, ER	2011
Roflumilast and cognition in memory-impaired elderly	ISRCTN96013814	2017
Study of efficacy of ibuprofen vaginal solution	EUCTR2018-001143-32-GR	2018
Clinical efficacy and mechanistic evaluation of Eplerenone for central serous chorio-retinopathy – the VICI randomised trial	ISRCTN92746680	2016
Periodontal treatment and reduction of vascular inflammation in patients with peripheral arterial disease	DRKS00004554	2012
A Multi-Centre, Placebo-Controlled Phase II Study of Canakinumab for the Treatment of Adult-onset Still's disease (AOSD) including an open-label long term extension	EUCTR2011-001027-20-DE	2011
A Randomised Phase II Study Evaluating Cediranib vs. Cediranib and Saracatanib in patients with relapsed metastatic clear cell renal cancer - COSAK	EUCTR2009-018014-20-GB	2010
A multicentre randomised double-blind placebo-controlled study comparing two regimens of combination induction therapy in early disease-modifying anti-rheumatic drug naïve rheumatoid arthritis	ISRCTN48638981	2007
A single center, randomized, double-blind, placebo-controlled 2-way crossover study to investigate the mechanism of action of etoricoxib in subjects with osteoarthritis knee pain	EUCTR2011-004179-35-DK	2011
CORAL: cancer of the OvaRy Abiraterone triaL	ISRCTN63407050	2013
Scaling effect on 2 salivary factors in patients with gingival inflammation	IRCT2016053025649N5	2016
A PHASE 1B DOSE ESCALATION/PHASE 2 RANDOMIZED, NON-COMPARATIVE, MULTIPLE CENTER, OPEN LABEL STUDY OF CP-751,871 IN COMBINATION WITH PACLITAXEL AND CARBOPLATIN AND OF PACLITAXEL AND CARBOPLATIN ALONE AS FIRST LINE TREATMENT FOR ADVANCED NON SMAL CELL LUNG CANCER	EUCTR2005-002249-38-ES	2005
Effects of Tibolone on cardiovascular disease in menopausal women	IRCT138901293743N1	2010
Multicenter, explorative phase II study of perioperative 5-FU, leucovorin, docetaxel, and oxaliplatin (FLOT) in combination with Trastuzumab in patients with HER2-positive, locally advanced resectable adenocarcinoma of the gastroesophageal junction or stomach (HerFLOT) - HerFLOT	EUCTR2011-001507-13-DE	2011
Globifer Forte in Heart Failure (GLOBIFER HF)	EUCTR2013-004704-19-GB	2015
A Clinical Trial to test the effect of Carboplatin and Paclitaxel, with or without Debio 1143 in patients with newly diagnosed Advanced Epithelial Ovarian Cancer	EUCTR2015-005137-42-BE	2016
Targeting the rheumatoid arthritis synovial fibroblast via cyclin dependent kinase inhibition - an early phase trial	ISRCTN36667085	2014
Examination of the effectiveness of early diagnosis and treatment performed by magnetic resonance imaging (MRI) of hepatocellular carcinoma with liver specific contrast agent	JPRN-UMIN000009830	2013
A Study with a product called Multikine used in treatment of Mouth Cancer	EUCTR2010-019952-35-HU	2010
Rifaximin tablets in the prevention of recurrent acute diverticulitis and diverticular complications	EUCTR2017-002708-28-DE	2017
A validity inspection study of the treat-to-target strategy with golimumab for the treatment of rheumatoid arthritis patient	JPRN-UMIN000009425	2012
Repurposing anti-TNF for treating Dupuytren’s disease	EUCTR2015-001780-40-GB	2015
A Prospective, Single-centre, Randomised Study Evaluating the Clinical, Imaging and Immunological Depth of Remission Achieved by Very Early versus Delayed Etanercept in patients with Rheumatoid Arthritis	EUCTR2010-023910-30-GB	2011

(VEDERA) - Very Early versus Delayed Etanercept in patients with RA (VEDERA)		
The efficacy and mechanism of Imrecoxib in axial spondyloarthritis	ChiCTR-TRC-14004718	2014
In this trial patients with newly diagnosed glioblastoma with an unmethylated MGMT-promoter are treated with targeted substances according to the results of a molecular and genomic characterization of their tumor tissue. One out of 8 drugs is given to the patient in combination with radiotherapy	EUCTR2015-002752-27-DE	2017
Effect of antisecretory factor in ulcerative colitis on histological and laborative outcome: a short period clinical trial	Eriksson, , A, Shafazand, M, Jennische, E; Lange, S	2003
A randomized phase II trial comparing two different sequence combinations of autologous vaccine and human recombinant interferon gamma and human recombinant interferon alpha2B therapy in patients with metastatic renal cell carcinoma: clinical outcome and analysis of immunological parameters	Schwaab, , T, Heaney, JA, Schned, AR, Harris, RD, Cole, BF, Noelle, RJ, Phillips, DM, Stempkowski, L; Ernstoff, MS	2000
The Oxford Marfan Trial	EUCTR2010-023612-14-GB	2012
Phase 2 study in patient with HER2-negative inflammatory breast cancer	EUCTR2016-001868-11-FR	2017
A Study of Atezolizumab and Paclitaxel Versus Placebo and Paclitaxel in Participants With Previously Untreated Locally Advanced or Metastatic Triple Negative Breast Cancer (TNBC) (IMpassion131)	CTRI/2017/10/010010	2017
Study of Abraxane® and gemcitabine followed by modified FOLFOX in patients with previously untreated, metastatic pancreatic adenocarcinoma	EUCTR2014-005350-19-ES	2015
Clinical study on using the MEMS micro-needle to Promote penetration and Layers adjusting extemal application technique for treating end-stage knee osteoarthritis,based on the theory of pain as pivot and truncating and reversing	ChiCTR-IOR-17013361	2017
A randomized, double-blind, placebo controlled, multicenter, phase II study of adding AMG 479, a fully human monoclonal antibody against insulin-like growth factor type 1 receptor (IGF-1R) to first line chemotherapy in patients with optimally debulked (< 1cm) epithelial ovarian cancer	EUCTR2008-001551-22-ES	2008
A Phase III study where the treatment groups are assigned by chance and neither the patient or the study doctor know which treatment the patient will receive that will take place in many sites and will compare the efficacy of Retosiban with placebo in stopping spontaneous preterm labor and prolonging labor	EUCTR2014-003326-41-GB	2015
A Comparison of Prasugrel and Clopidogrel in Acute Coronary Syndrome (ACS) Subjects with Unstable Angina/Non-ST-Elevation Myocardial Infarction (UA/NSTEMI) Who are Medically Managed - The TRILOGY ACS Study - Trilogy ACS	EUCTR2007-005210-39-PT	2008
A study comparing standard therapy with or without TG4010 in patients with advanced non small cell lung cancer	EUCTR2011-001468-23-DE	2011
Effect of soybean flour enriched bread consumption on inflammation, lipid profile, glycemic indices and Anthropometric indices	IRCT2013061613684N1	2013
Reumap: an Open Label phase II study of Bovine Intestinal Alkaline Phosphatase (bIAP), An Inflammation modulating moiety, in RA patients - REUMAP	EUCTR2008-007346-63-GB	2010
A 12-Week, Double-Blind, Randomized, Parallel Group, Placebo-Controlled Study of Two Doses of VX-702 in Subjects with Moderate to Severe Rheumatoid Arthritis - VeRA	EUCTR2005-000549-13-CZ	2005
Prevention of recurrent narrowing of the esophagus after correction of esophageal atresia by injecting steroids	EUCTR2018-002863-24-NL	2018
Evaluation of two preventive schemes of dental extraction with amoxicillin in patients with rheumatoid arthritis treated with tumor necrosis factor-alpha inhibitors (anti-TNF-?) and methotrexate	RBR-3kzp25	2015
Effect of Multiple Doses of Oral Tranexamic Acid on Haemostasis and Inflammatory Reaction in Total Hip Arthroplasty: a Randomized Controlled Trial	Wang, , D, Yang, Y, He, C, Luo, ZY, Pei, FX, Li, Q, Zhou, ZK; Zeng, WN	2019
Trial of pimasertib with SAR245409 or placebo in ovarian cancer	EUCTR2013-000902-40-IT	2013
A study to investigate the safety and effectiveness of a new anti cancer treatment - AZD5363 and assess its ability to affect levels of key proteins in cancer cells prior to the surgical removal of breast cancer	EUCTR2012-005019-14-GB	2013
Open label, prospective observational, multi-center trial in postmenopausal, hormone receptor positive breast cancer patients treated with eithertamoxifen or an aromatase inhibitor. Examination of the predictive value of biological markers AEBS/mEH, active tamoxifen metabolites and TGF beta system for response and non response of tamoxifen treatment	DRKS00000605	2010
pre-operative nivolumab, ipilimumab and COC2-inhibition in olon cancer (NICHE)	EUCTR2016-002940-17-NL	2016
Renal, efficacy and safety outcomes following late conversion of kidney transplant patients from calcineurin inhibitor therapy to everolimus: the randomized APOLLO study	Budde, , K, Rath, T, Sommerer, C, Haller, H, Reinke, P, Witzke, O, Suwelack, B, Baeumer, D, May, C, Porstner, M; Arns, W	2015
AG-013736 (axitinib) for the treatment of metastatic renal cell cancer (mRCC) - Specific for Bulgaria only	EUCTR2010-018585-23-BG	2010

To estimate and compare a biomarker in saliva in gum disease patients with and without diabetes before and after treatment	CTRI/2018/03/012649	2018
Toreforant, an orally active histamine H4-receptor antagonist, in patients with active rheumatoid arthritis despite methotrexate: mechanism of action results from a phase 2, multicenter, randomized, double-blind, placebo-controlled synovial biopsy study	Boyle, , DL, DePrimo, SE, Calderon, C, Chen, D, Dunford, PJ, Barchuk, W, Firestein, GS; Thurmond, RL	2019
Randomized clinical trial assessing the effect of Doppler-optimized fluid management on outcome after elective colorectal resection	Noblett, , SE, Snowden, CP, Shenton, BK; Horgan, AF	2006
The effect of melatonin supplementation in the treatment of type 2 diabetic patients	IRCT2017030831993N4	2017
Clinical study aimed to assess in patients with triple-negative operable breast cancer the activity of the drug zoledronate administered before surgery, according to the the tumor aggressiveness (determined by the level of expression of the p53 protein)	EUCTR2014-004194-16-IT	2015
Study for children with medulloblastoma of standard risk, between 3-5 years old and less than 22 years old	EUCTR2011-004868-30-FR	2015
A study to find out whether maraviroc given once daily is as effective as Truvada given once daily in treating HIV-infected patients never previously treated with maraviroc	EUCTR2010-021785-30-FI	2011
Feasibility study of radiofrequency endoscopic ablation, with ultrasound guidance, as a non-surgical, adrenal sparing treatment for aldosterone-producing adenomas	ISRCTN86062633	2018
Standard-Dose Combination Chemotherapy or High-Dose Combination Chemotherapy and Stem Cell Transplant in Treating Patients With Relapsed or Refractory Germ Cell Tumors	ACTRN12618001236280	2018
Efficacy and safety of a Double Icodextrin Dose in ederly patients, starting treatment by Continuous Ambulatory Peritoneal Dialysis (CAPD)	EUCTR2011-005274-30-FR	2012
Study to Evaluate the Safety and Efficacy of the MON4STRAT Approach for Optimizing Meropenem Therapy in Intubated and Mechanically-Ventilated, Adult Patients with Gram-Negative Lower Respiratory Tract Infection Including Nosocomial Pneumonia and Severe Tracheobronchitis	EUCTR2018-000450-21-ES	2018
Clinical trial for the treatment of pulmonary alveolar proteinosis by inhalation of recombinant human granulocyte-macrophage colony stimulating factor (GMCSF)	ISRCTN18931678	2008
TElmisartan in the management of abDominal aortic aneurYsm (TEDY)	EUCTR2012-001859-39-NL	2012
Abiraterone treatment for patients with relapsed ovarian cancer	EUCTR2013-000293-29-GB	2013
Effect of Propolis Extract on Chronic Periodontitis Patients	IRCT2016122030475N3	2017
Study of relation between gum infection and pregnancy complications	CTRI/2015/02/005581	2015
Systemic Chemotherapy With or Without Intraperitoneal Chemohyperthermia in Treating Patients Undergoing Surgery for Peritoneal Carcinomatosis From Colorectal Cancer	EUCTR2006-006175-20-ES	2012
Atorvastatin and endothelial function in systemic lupus erythematosus (SLE) patients	ISRCTN19073445	2009
Effect of root planning in microbiological, White blood cells,immunoglobulins and pro and anti inflamatory cytokines dosage in blood and saliva from periodontitis and type 2 diabet persons	RBR-6268zz	2016
Rifaximin tablets in the prevention of recurrent acute diverticulitis and diverticular complications	EUCTR2017-002708-28-HU	2018
Afatinib as Cancer therapy for Exocrine Pancreatic Tumours	EUCTR2011-004063-77-DE	2012
Research on treatment strategies for adequate anitplatelet therapy for the prevention of major cardiac events after vascular surgery	EUCTR2016-000686-23-NL	2016
Cardioprotective Effects of Carvedilol in Inhibiting Doxorubicin-induced Cardiotoxicity	Nabati, , M, Janbabai, G, Baghyari, S, Esmaili, K; Yazdani, J	2017
Acupuncture and moxibustion as complementary therapy in Crohn's disease	Joos, , S, Brinkhaus, B, Maluche, C, Maupai, N, Kohnen, R, Hahn, E; Schuppan, D	2005
Inhibition of platelet function with clopidogrel is associated with a reduction of inflammation in patients with peripheral artery disease	Meyer, , A, Weithaeuser, A, Steffens, D, Bobbert, P, Hassanein, A, Ayral, Y, Schultheiss, HP; Rauch, U	2016
Asthma control cost-utility randomized trial evaluation (ACCURATE): the goals of asthma treatment	Honkoop, , PJ, Loymans, RJ, Termeer, EH, Snoeck-Stroband, JB, Bakker, MJ, Assendelft, WJ, Sterk, PJ, Ter Riet, G, Schermer, TR; Sont, JK	2011
Effect of long-term treatment with low-dose mifepristone on the endometrium	Baird, , DT, Brown, A, Critchley, HOD, Williams, AR, Lin, S; Cheng, L	2003
Mallory-denk bodies are associated with outcomes and histologic features in patients with chronic hepatitis C	Rakoski, , MO, Brown, MB, Fontana, RJ, Bonkovsky, HL, Brunt, EM, Goodman, ZD, Lok, AS; Omary, MB	2011
A randomized, double-blind study comparing tremelimumab to placebo in second and third line treatment of subjects with unresectable pleural or peritoneal mesothelioma	Kindler, , HL, Di Pietro, A, Robbins, PB, Hong, S, Shalabi, A, Ibrahim, R, Calabro, L; Maio, M	2013
Bronchial hyperresponsiveness in asthmatic adults–a long-term correlation study	Carbone, , R, Luppi, F, Monselise, A; Bottino, G	2005
Association of the bevacizumab pharmacokinetics with efficacy and toxicity in advanced non-squamous non-small cell lung cancer: kumamoto Thoracic Oncology Study Group (KTOSG) 1003 study	Saeki, , S, Sasaki, J, Sakata, S, Sato, R, Saruwatari, K, Sakamoto, Y, Inaba, M, Kishi, H, Fujii, S, Tsumura, S, Ouchi, M, Saito, H, Hamada, A; Kohrogi, H	2017

Effect of tart cherry juice on arterial stiffness, inflammation and other risk markers for cardiovascular disease in healthy adults	Lynn, , A, Mathew, S, Moore, CT, Russell, J, Robinson, E, Soumpasi, V; Barker, ME	2013
The effects of remote ischaemic preconditioning on coronary artery function in patients with stable coronary artery disease	Corcoran, , D, Young, R, Cialdella, P, McCartney, P, Bajrangee, A, Hennigan, B, Collison, D, Carrick, D, Shaukat, A, Good, R, Watkins, S, McEntegart, M, Watt, J, Welsh, P, Sattar, N, McConnachie, A, Oldroyd, KG; Berry, C	2018
Sulforaphane-rich broccoli sprout extract improves hepatic abnormalities in male subjects	Kikuchi, , M, Ushida, Y, Shiozawa, H, Umeda, R, Tsuruya, K, Aoki, Y, Suganuma, H; Nishizaki, Y	2015
Effect of Mud-Bath Therapy on Serum Biomarkers in Patients with Knee Osteoarthritis: results from a Randomized Controlled Trial	Pascarelli, , NA, Cheleschi, S, Bacaro, G, Guidelli, GM, Galeazzi, M; Fioravanti, A	2016
Rituximab Is Ineffective for Treatment of Fatigue in Primary Biliary Cholangitis: a Phase 2 Randomized Controlled Trial	Khanna, , A, Jopson, L, Howel, D, Bryant, A, Blamire, A, Newton, JL; Jones, DE	2018
The use of bone markers in a 6-week study to assess the efficacy of oral clodronate in patients with metastatic bone disease	Brown, , JE, McCloskey, EV, Dewar, JA, Body, JJ, Cameron, DA, Harnett, AN, Ruutu, M, Purohit, OP, Tähtelä, R; Coleman, RE	2007
Randomized, double-blind, placebo-controlled study of tremelimumab for second-and third-line treatment of unresectable pleural or peritoneal mesothelioma	Maio, , M, Scherpereel, A, Di Pietro, A, Vasey, P, Tsao, AS, Calabro, L, Fu, D, Robbins, PB, Ibrahim, RA; Kindler, HL	2014
CD4+CD28-T-cell expansions in ANCA-associated vasculitis and association with arterial stiffness: baseline data from a randomised controlled trial	Chanouzas, , D, Dyall, L, Dale, J, Moss, P, Morgan, M; Harper, L	2015
Effect of Omega-3 fatty acid supplementation on markers of platelet and endothelial function in patients with peripheral arterial disease	Mackay, , I, Ford, I, Thies, F, Fielding, S, Bachoo, P; Brittenden, J	2012
Preliminary safety and outcome report of the metronomic therapy from the Latin American osteosarcoma treatment protocol 2006	Petrilli, , AS, Macedo, CR, Toledo, SRC, Pavoni-Ferreira, PC, Grings, M, Scopinaro, M, Ferman, S, Boldrini, E, A Almeida MT, , De Oliveira, CT, Rey, GL, Castillo, LA, Borsato, ML, Barreto, JH, L Morais VL, , Brunetto, AL; Lustosa, D	2011
Multicentric Randomized Study of H. Pylori Eradication and Pepsinogen Testing for Prevention of Gastric Cancer Mortality	NCT02047994	2014
Beneficial effects of ramipril on myocardial diastolic function in patients with type 2 diabetes mellitus, normal LV systolic function and without coronary artery disease: a prospective study using tissue Doppler	Siegmund, , T, Schumm-Draeger, PM, Antoni, D; Bibra, HV	2007
Evaluation of step up therapy in patients with early ulcerative colitis: a prospective cohort study	Bossuyt, , P, Baert, F, Coenegrachts, J-L, De Vos, M, Dewit, O, Ferrante, M, Fontaine, F, Mana, F, Vandervoort, J; Moreels, T	2017
Community-based scheduled screening and treatment of malaria in pregnancy for improved maternal and infant health in The Gambia, Burkina Faso and Benin: study protocol for a randomized controlled trial	Scott, , S, Mens, PF, Tinto, H, Nahum, A, Ruizendaal, E, Pagnoni, F, Grietens, KP, Kendall, L, Bojang, K, Schallig, H; D'Alessandro, U	2014
The effect of a periodontal intervention on cardiovascular risk markers in Indigenous Australians with periodontal disease: the PerioCardio study	Skilton, , MR, Maple-Brown, LJ, Kapellas, K, Celermajer, DS, Bartold, M, Brown, A, O'Dea, K, Slade, GD; Jamieson, LM	2011
A clinical study of adefovir dipivoxil treatment for chronic hepatitis patients with cirrhosis in their decompensation period	Yang, , Q, Gong, ZJ; Hu, DF	2009
N-Acetylcysteine and Milk Thistle for Treatment of Diabetic Nephropathy	NCT01265563	2010
Impact of oral ibandronate 150 mg once monthly on bone structure and density in post-menopausal osteoporosis or osteopenia derived from in vivo µCT	Bock, , O, Börst, H, Beller, G, Armbrecht, G, Degner, C, Martus, P, Roth, HJ; Felsenberg, D	2012
Phase II trial of mapatumumab, a fully human agonist monoclonal antibody to tumor necrosis factor-related apoptosis-inducing ligand receptor 1 (TRAIL-R1), in combination with paclitaxel and carboplatin in patients with advanced non-small-cell lung cancer	von Pawel, , J, Harvey, JH, Spigel, DR, Dediu, M, Reck, M, Cebotaru, CL, Humphreys, RC, Gribbin, MJ, Fox, NL; Camidge, DR	2014
Levels of NT-proBNP, markers of low-grade inflammation, and endothelial dysfunction during spironolactone treatment in patients with diabetic kidney disease	Nielsen, , SE, Schjoedt, KJ, Rossing, K, Persson, F, Schalkwijk, CG, Stehouwer, CD, Parving, HH; Rossing, P	2013
Effect of pioglitazone versus metformin on cardiovascular risk markers in type 2 diabetes	Genovese, , S, De Berardis, G, Nicolucci, A, Mannucci, E, Evangelista, V, Totani, L, Pellegrini, F; Ceriello, A	2013
Mediation by patient-reported outcomes on the association between film-coated versus dispersible formulations of deferasirox and serum ferritin reduction: a post hoc analysis of the eclipse trial	Taher, , AT, Origa, R, Perrotta, S, Kouraklis, A, Belhoul, K, Huang, V, Han, J, Bruederle, A, Bobbili, P, Duh, MS; Porter, JB	2017
The efficacy of antithrombin administration in the acute phase of burn injury	Lavrentieva, , A, Kontakiotis, T, Bitzani, M, Parlapani, A, Thomareis, O, Scourtis, H, Tsotsolis, N, Lazaridis, L; Giala, MA	2008
Anti-inflammatory treatment of depression: study protocol for a randomised controlled trial of vortioxetine augmented with celecoxib or placebo	Fourrier, , C, Sampson, E, Mills, NT; Baune, BT	2018
Antibiotic treatment In patients with chronic low back pain and Modic changes (the AIM study): study protocol for a randomised controlled trial	Storheim, , K, Espeland, A, Grovle, L, Skouen, JS, Assmus, J, Anke, A, Froholdt, A, Pedersen, LM, Haugen, AJ, Fors, T, Schistad, E, Lutro, O, Marchand, GH, Kadar, T, Vetti, N, Randen, S, Nygaard, P, Brox, JI, Grotle, M; Zwart, J-A	2017
Influence of Treatment With Olanzapine or Ziprasidone on Transcapillary Glucose Transport in Human Skeletal Muscle	NCT00297960	2006
Body composition is improved during 12 months' treatment with metformin alone or combined with oral contraceptives compared with treatment with oral contraceptives in polycystic ovary syndrome	Glintborg, , D, Altinok, ML, Mumm, H, Hermann, AP, Ravn, P; Andersen, M	2014
Phase 1/2 study of cyclin-dependent kinase (CDK)4/6 inhibitor palbociclib (PD-0332991) with bortezomib and dexamethasone in relapsed/refractory multiple myeloma	Niesvizky, , R, Badros, AZ, Costa, LJ, Ely, SA, Singhal, SB, Stadtmauer, EA, Haideri, NA, Yacoub, A, Hess, G, Lentzsch, S, Spicka, I, Chanan-Khan, AA, Raab, MS, Tarantolo, S, Vij, R, Zonder, JA, Jayabalan, D, Di Liberto, M, Huang, X, Jiang, Y, Kim, ST, Randolph, S; Chen-Kiang, S	2015
Acupuncture and moxibustion in the treatment of active Crohn's disease: a randomized controlled study	Joos, , S, Brinkhaus, B, Maluche, C, Maupai, N, Kohnen, R, Kraehmer, N, Hahn, EG; Schuppan, D	2004

Analysis of surrogate gene expression markers in peripheral blood of melanoma patients to predict treatment outcome of adjuvant pegylated interferon alpha 2b (EORTC 18991 side study)	Busse, , A, Rapion, J, Fusi, A, Suciu, S, Nonnenmacher, A, Santinami, M, Kruit, WH, Testori, A, Punt, CJ, Dalgleish, AG, Spatz, A, Eggermont, AM; Keilholz, U	2013
Total lymphoid irradiation in multiple sclerosis	Wiles, , CM, Omar, L, Swan, AV, Sawle, G, Frankel, J, Grunewald, R, Joannides, T, Jones, P, Laing, H; Richardson, PH	1994
Blood Chromogranin A is Not Effective as a Biomarker for Diagnosis or Management of Bronchopulmonary NET/NET	Matar, , S, Malczewska, A, Oberg, K, Bodei, L, Aslanian, H, Lewczuk-Myslicka, A, Filosso, PL, Suarez, AL, Kolasinska-Cwikla, A, Roffinella, M, Kos-Kudla, B, Cwikla, JB, Drozdov, IA, Kidd, M; Modlin, IM	2019
Why randomized controlled clinical trials do not depict accurately long-term outcomes in rheumatoid arthritis: some explanations and suggestions for future studies	Pincus, , T; Stein, CM	1997
Statin-associated muscle symptoms in coronary patients: design of a randomized study	Munkhaugen, , J, Vethe, NT, Fagerland, MW, Dammen, T, Perk, J, Gjertsen, E, Otterstad, JE, Gullestad, L, Bergan, S; Husebye, E	2019
A double-blind, placeho-controlled randomized clinical trial of alpha-tocopherol (vitamin E) in the treatment of amyotrophic lateral sclerosis	Desnuelle, , C, Dib, M, Garrel, C; Favier, A	2001
Blood metabolism study on protection of residual renal function of hemodialysis patients by traditional Chinese medicine Kidney Flaccidity Compound	Hu, , QD, Wu, WH, Zeng, Y, Wen, J, Li, XJ, Pan, W, Zhang, MP, Hu, B, Lei, CY; Fan, J	2018
An Intensive Lifestyle Intervention Program in CKD (Move to Health 2)	NCT02842957	2016
Acupuncture and moxibustion in the treatment of ulcerative colitis: a randomized controlled study	Joos, , S, Wildau, N, Kohnen, R, Szecsenyi, J, Schuppan, D, Willich, SN, Hahn, EG; Brinkhaus, B	2006
Caplacizumab Treatment for Acquired Thrombotic Thrombocytopenic Purpura	Scully, , M, Cataland, SR, Peyvandi, F, Coppo, P, Knöbl, P, Kremer Hovinga, JA, Metjian, A, de la Rubia, J, Pavenski, K, Callewaert, F, Biswas, D, De Winter, H; Zeldin, RK	2019
A randomized, double blind, placebo-controlled trial of alendronate treatment for fibrous dysplasia of bone	Boyce, , AM, Kelly, MH, Brillante, BA, Kushner, H, Wientroub, S, Riminucci, M, Bianco, P, Robey, PG; Collins, MT	2014
Treatment with ω-3 fatty acids reduces serum C-reactive protein concentration	Muhammad, , KI, Morledge, T, Sachar, R, Zeldin, A, Wolski, K; Bhatt, DL	2011
Mitochondrial DNA and RNA increase in peripheral blood mononuclear cells from HIV-1-infected patients randomized to receive stavudine-containing or stavudine-sparing combination therapy	Casula, , M, Weverling, GJ, Wit, FW, Timmermans, EC, Stek, M, Lange, JM; Reiss, P	2005
Metformin reduces serum mullerian-inhibiting substance levels in women with polycystic ovary syndrome after protracted treatment	Fleming, , R, Harborne, L, MacLaughlin, DT, Ling, D, Norman, J, Sattar, N; Seifer, DB	2005
Effects of bayberry juice on inflammatory and apoptotic markers in young adults with features of non-alcoholic fatty liver disease	Guo, , H, Zhong, R, Liu, Y, Jiang, X, Tang, X, Li, Z, Xia, M; Ling, W	2014
Evaluation of Mangosteen juice blend on biomarkers of inflammation in obese subjects: a pilot, dose finding study	Udani, , JK, Singh, BB, Barrett, ML; Singh, VJ	2009
Pioglitazone improves fat distribution, the adipokine profile and hepatic insulin sensitivity in non-diabetic end-stage renal disease subjects on maintenance dialysis: a randomized cross-over pilot study	Zanchi, , A, Tappy, L, Le, K-A, Bortolotti, M, Theumann, N, Halabi, G, Gauthier, T, Mathieu, C, Tremblay, S, Bertrand, PC, Burnier, M; Teta, D	2014
Trichuris suis ova therapy in relapsing multiple sclerosis is safe but without signals of beneficial effect	Voldsgaard, , A, Bager, P, Garde, E, Åkeson, P, Leffers, AM, Madsen, CG, Kapel, C, Roepstorff, A, Thamsborg, SM, Melbye, M, Siebner, H, Søndergaard, HB, Sellebjerg, F; Sørensen, PS	2015
Quantitative mrd is prognostic for progression-free & overall survival in elderly patients receiving chlorambucil alone or with obinutuzumab/rituximab: a prospective analysis of the GCLLSG cll11 study	Ritgen, , M, Langerak, A, Goede, V, Bahlo, J, Kluth, S, Fischer, K, Steurer, M, Trneny, M, Mulligan, S, Mey, U, Trunzer, K, Humphrey, K, Fingerle-Rowson, G, Stilgenbauer, S, Bottcher, S, Bruggemann, M, Hallek, M, Kneba, M; Van Dongen, J	2016
Antioxidant supplementation with or without B-group vitamins after acute ischemic stroke: a randomized controlled trial	Ullegaddi, , R, Powers, HJ; Gariballa, SE	2006
Long-term individual shear rate therapy counterpulsation enhances plasma nitrite release in patients with PAD	Brix, , M, Buschmann, EE, Zietzer, A, Jaurigue, JA, Li, L, Jungk, C, Buschmann, I, Janke, D; Hillmeister, P	2017
Early Posttransplant Tryptophan Metabolism Predicts Long-term Outcome of Human Kidney Transplantation	Vavrincova-Yaghi, , D, Seelen, MA, Kema, IP, Deelman, LE, van der Heuvel, MC, Breukelman, H, Van den Eynde, BJ, Henning, RH, van Goor, H; Sandovici, M	2015
The effect of a Mediterranean diet with adequate dairy foods on cardio-metabolic and cognitive health outcomes	ACTRN12616000309482	2016
A double-blind, placebo-controlled randomized clinical trial of alpha-tocopherol (vitamin E) in the treatment of amyotrophic lateral sclerosis. ALS riluzole-tocopherol Study Group	Desnuelle, , C, Dib, M, Garrel, C; Favier, A	2001
Prophylactic irradiation of para-aortic lymph nodes for patients with locally advanced cervical cancers with and without high CA9 expression (KROG 07-01): a randomized, open-label, multicenter, phase 2 trial	Kim, , JH, Kim, JY, Yoon, MS, Kim, YS, Lee, JH, Kim, HJ, Kim, H, Kim, YJ, Yoo, CW, Nam, BH, Kim, TH, Kim, SK, Kim, SH, Kang, S, Seo, SS, Lim, MC; Park, SY	2016
Effects of intermittent exposure to hyperbaric oxygen for the treatment of an acute soft tissue injury	Babul, , S, Rhodes, EC, Taunton, JE; Lepawsky, M	2003
Prediction of clinical responders to treatment with dims0150 a toll-like receptor 9 agonist in therapy refractory ulcerative colitis patients	Von Stein, , O Musch E Lofberg R Kuznetsov N; Von Stein, P	2013
Evaluating Genes in Sputum to Measure Drug Response in COPD	NCT00233051	2005
Omega-3 fatty acids for indicated prevention: treatment results and pathomechanisms	Amminger, , GP, Harris, MS, McGorry, PD; Henry, LP	2013
Long-term benefit of sebelipase alfa over 76 weeks in children and adults with lysosomal acid lipase deficiency (LALD) ARISE	Burton, , BK, Marulkar, S, Friedman, M, Tripuraneni, R; Furuya, KN	2017
Intracoronary nitrite suppresses the inflammatory response following primary percutaneous coronary intervention	Jones, , DA, Khambata, RS, Andiapen, M, Rathod, KS, Mathur, A; Ahluwalia, A	2017
Telomere length is an independent predictor of outcome after therapy in CLL: results from the UKCLL4 trial	Parker, , A, Forster, J, Reichter, S, De Castro, DG, Catovsky, D; Oscier, D	2011

Viral Inception of Asthma: prospective Study From Infancy to School-age	NCT00731575	2008
Room-air resuscitation causes less damage to heart and kidney than 100% oxygen	Vento, , M, Sastre, J, Asensi, MA; Viña, J	2005
Effect of weight loss on the cardiovascular risk profile of obese patients with psoriasis	Jensen, , P, Zachariae, C, Christensen, R, Geiker, NR, Schaadt, BK, Stender, S, Astrup, A, Hansen, PR; Skov, L	2014
Early Versus Late DC-cardioversion of Persistent Atrial Fibrillation. Effect on Atrial Remodeling,Inflammatory and Neurohumoral Markers and Recurrence of Atrial Fibrillation	NCT01593150	2012
The benefits of exercise training in interstitial lung disease: protocol for a multicentre randomised controlled trial	Dowman, , L, McDonald, CF, Hill, C, Lee, A, Barker, K, Boote, C, Glaspole, I, Goh, N, Southcott, A, Burge, A, Ndongo, R, Martin, A; Holland, AE	2013
Autologous CD34+cell transplantation promotes angiogenesis in older adult patients with atherosclerotic ischemia: study protocol for a prospective, single-center, open-label, randomized controlled clinical trial	Zhou, , C-H, Xu, L-L, Hao, X-X, Sun, X-J, Guo, M-J; Liu, B	2017
Anamorelin for patients with cancer cachexia: an integrated analysis of two phase 2, randomised, placebo-controlled, double-blind trials	Garcia, , JM, Boccia, RV, Graham, CD, Yan, Y, Duus, EM, Allen, S; Friend, J	2015
Neurofeedback Treatment of Affective Instability in Premenstrual Dysphoric Disorder(PMDD)	NCT01945372	2013
Kidney and Periodontal Disease Study	NCT01802216	2013
Monotherapy with Levetiracetam Versus Older AEDs: a Randomized Comparative Trial of Effects on Bone Health	Hakami, , T, O'Brien, TJ, Petty, SJ, Sakellarides, M, Christie, J, Kantor, S, Todaro, M, Gorelik, A, Seibel, MJ, Yerra, R; Wark, JD	2016
Nociceptive blink reflex habituation biofeedback in migraine	de Tommaso, , M; Delussi, M	2017
Short-term Curcuminoid Supplementation for Chronic Pulmonary Complications due to Sulfur Mustard Intoxication: positive Results of a Randomized Double-blind Placebo-controlled Trial	Panahi, , Y, Ghanei, M, Bashiri, S, Hajihashemi, A; Sahebkar, A	2015
Long-term benefit of sebelipase alfa over 100 weeks in children and adults with lysosomal acid lipase deficiency (arise study)	Burton, , BK, Feillet, F, Furuya, K, Friedman, M, Marulkar, S; Balwani, M	2017
Periodontal Treatment and Metabolic Control in Type 2 Diabetic Patients	NCT01291875	2011
Varenicline for treatment of alcohol dependence: a randomized, placebo-controlled trial	de Bejczy, , A, Löf, E, Walther, L, Guterstam, J, Hammarberg, A, Asanovska, G, Franck, J, Isaksson, A; Söderpalm, B	2015
Long-term effects of metformin on endothelial function in type 2 diabetes: a randomized controlled trial	de Jager, , J, Kooy, A, Schalkwijk, C, van der Kolk, J, Lehert, P, Bets, D, Wulffelé, MG, Donker, AJ; Stehouwer, CD	2014
An increase in the absolute count of CD56dimCD16+CD69+ NK cells in the peripheral blood is associated with a poorer IVF treatment and pregnancy outcome	Thum, , MY, Bhaskaran, S, Abdalla, HI, Ford, B, Sumar, N, Shehata, H; Bansal, AS	2004
Immune Correlates of GM-CSF and Melanoma Peptide Vaccination in a Randomized Trial for the Adjuvant Therapy of Resected High-Risk Melanoma (E4697)	Butterfield, , LH, Zhao, F, Lee, S, Tarhini, AA, Margolin, KA, White, RL, Atkins, MB, Cohen, GI, Whiteside, TL, Kirkwood, JM; Lawson, DH	2017
N-Acetylcysteine and Milk Thistle for Treatment of Diabetic Nephropathy	NCT00915200	2009
Dietary nitrate supplementation enhances exercise performance in peripheral arterial disease	Kenjale, , AA, Ham, KL, Stabler, T, Robbins, JL, Johnson, JL, Vanbruggen, M, Privette, G, Yim, E, Kraus, WE; Allen, JD	2011
A multiple dose study of AMG 811 (anti-IFN-gamma) in subjects with systemic lupus erythematosus and active nephritis	Martin, , DA, Amoura, Z, Romero-Diaz, J, Chong, YB, Sanchez-Guerrero, J, Chan, T, Arnold, GE, Damore, MA, Sohn, W, Chirmule, N, Chiu, K, Wang, C, Boedigheimer, M, Sullivan, BA, Welcher, A, Kotzin, B; Chung, JB	2015
Neutrophil-Related Gene Expression and Low-Density Granulocytes Associated With Disease Activity and Response to Treatment in Antineutrophil Cytoplasmic Antibody-Associated Vasculitis	Grayson, , PC, Carmona-Rivera, C, Xu, L, Lim, N, Gao, Z, Asare, AL, Specks, U, Stone, JH, Seo, P, Spiera, RF, Langford, CA, Hoffman, GS, Kallenberg, CG, St Clair, EW, Tchao, NK, Ytterberg, SR, Phippard, DJ, Merkel, PA, Kaplan, MJ; Monach, PA	2015
A Trial of Ischemic Preconditioning in Raynaud's Phenomenon (RP)	NCT02506062	2015
Metabolic modulation with perhexiline in chronic heart failure: a randomized, controlled trial of short-term use of a novel treatment	Lee, , L, Campbell, R, Scheuermann-Freestone, M, Taylor, R, Gunaruwan, P, Williams, L, Ashrafian, H, Horowitz, J, Fraser, AG, Clarke, K; Frenneaux, M	2005
Effects of alpha-linolenic acid versus those of EPA/DHA on cardiovascular risk markers in healthy elderly subjects	Goyens, , PL; Mensink, RP	2006
Analysis of Cytokine Levels in Tears and Clinical Correlations After Intense Pulsed Light Treating Meibomian Gland Dysfunction	Liu, , R, Rong, B, Tu, P, Tang, Y, Song, W, Toyos, R, Toyos, M; Yan, X	2017
The effect of diet beverage intake on measures of diabetes control: a pilot study	Odegaard, , A; Hirahatake, K	2017
Interferon trials in small cell lung cancer at one institution: a comparison of results obtained before and after initiation of systematic treatment trials using IFN-α in combination with other modalities	Ruotsalainen, , TM; Mattson, K	2002
Continuous effectiveness of canakinumab treatment in Schnitzler's syndrome: a 4-year open-label multi-center study	Krause, , K, Bonnekoh, H, Ellrich, A, Tsianakas, A, Wagner, N, Fischer, J; Maurer, M	2019
Research study of Ibrutinib in Combination with Venetoclax in patients with Mantle Cell Lymphoma	EUCTR2017-000129-12-NL	2018
Clinical study over a period of at least 6 months to an individualized strength and endurance training in COPD patients in Marburger	DRKS00006842	2014
Early physical activity intervention prevents decrease of bone strength in very low birth weight infants	Litmanovitz, , I, Dolfin, T, Friedland, O, Arnon, S, Regev, R, Shainkin-Kestenbaum, R, Lis, M; Eliakim, A	2003

An open label, multicenter, non randomized phase II study to evaluate anti-tumor efficacy and safety of GM-CSF (sargramostim, Leukine®) associated with Rituximab (MabThera®) in patients with follicular non Hodgkin's lymphoma with no prior treatment - FL2008 RGM	EUCTR2007-005580-95-FR	2008
Insulin Independence Trial	EUCTR2015-000105-39-ES	2015
A MULTICENTER OPEN LABEL PHASE II STUDY OF THE EFFICACY AND SAFETY OF AMG 479, A FULLY HUMAN MONOCLONAL ANTIBODY AGAINST INSULIN-LIKE GROWTH FACTOR TYPE 1 RECEPTOR (IGF-1R) AS SECOND LINE THERAPY IN PATIENTS WITH RECURRENT PLATINUMSENSITIVE OVARIAN CANCER	EUCTR2008-001552-44-ES	2008
Vascular adhesion molecule-1 and markers of platelet function before and after a treatment with iloprost or a supervised physical exercise program in patients with peripheral arterial disease	Arosio, , E, Minuz, P, Prior, M, Zuliani, V, Gaino, S, De Marchi, S, Fontana, L, Andrioli, G, Lechi, C; Lechi, A	2001
A Clinical Trial for the comparison of Ticagrelor and Clopidogrel in Patients with CoronarY Artery Disease	EUCTR2013-004376-35-GR	2013
A Phase IIIb study to openly compare a group treated with only pegylated interferon alfa-2a (PEG-IFN) to an untreated group in children with chronic hepatitis B (CHB) who are HBeAg positive (a marker of their stage of CHB infection)	EUCTR2011-002732-70-IT	2012
TRON: a trial to examine the effectiveness of the study drug (Everolimus) in the treatment of cognitive problems experienced by patients with tuberous sclerosis	EUCTR2011-004854-25-GB	2011
A Randomized Phase II Study to Determine Potential Predictive Markers of Response to MDX-010 BMS-734016 in Patients with Unresectable Stage III or IV Malignant Melanoma - ND	EUCTR2005-002126-64-IT	2006
The effect of long-term weight-loss intervention strategies on the dynamics of pancreatic-fat and morphology: an MRI RCT study	Tene, , L, Shelef, I, Schwarzfuchs, D, Gepner, Y, Yaskolka Meir, A, Tsaban, G, Zelicha, H, Bilitzky, A, Komy, O, Cohen, N, Bril, N, Rein, M, Serfaty, D, Kenigsbuch, S, Chassidim, Y, Sarusy, B, Ceglarek, U, Stumvoll, M, Blüher, M, Thiery, J, Stampfer, MJ, Rudich, A; Shai, I	2018
Efficacy of mindfulness-based intervention (Ã¢ mindfulness-based joyful sleep') in young and middle-aged individuals with insomnia using a biomarker of inflammatory responses: a prospective protocol of a randomised controlled trial in China	Pan, , C, Wang, X, Deng, Y, Li, P, Liao, Y, Ma, X, Yang, G-P, Dai, L; Tang, Q	2019
A randomised, double-blind, placebo controlled study to assess the safety and the efficacy of neridronate 100 mg - 4 i.v. infusions in a course of 10 days treatment - in patients with algodystrophic syndrome. - ND	EUCTR2007-003372-18-IT	2008
Markers of bone in diabetics and the effect of bone treatment	EUCTR2013-001222-26-DK	2013
The Effects of Inorganic Nitrite on skeletal muscle: physiology, Pharmacology and Therapeutic Potential in patients suffering from Intermittent Claudication	EUCTR2012-000201-72-GB	2012
A randomized, double-blind, placebo controlled study to assess the safety and the efficacy of Neridronate ampoules 25 mg, after repeated intramuscular administrations, in patients with Complex Regional Pain Syndrome type I (CRPS-I)	EUCTR2014-001156-28-IT	2014
A Randomized, Multicenter, Open-Label, 3 Arm Phase 3 Study of Obinutuzumab in Combination with Chlorambucil, ACP 196 in Combination with Obinutuzumab, and ACP-196 Monotherapy in Subjects with Previously Untreated Chronic Lymphocytic Leukemia	EUCTR2014-005582-73-HU	2015
A Randomized, Multicenter, Open-Label, Non-Inferiority, Phase 3 Study of ACP-196 Versus Ibrutinib in Previously Treated Subjects with High Risk Chronic Lymphocytic Leukemia	EUCTR2014-005530-64-BE	2015
Clinical trial for assessing if gentic information obtained with NEUROFARMAGEN test is effective for selecting the treatment in patients with mental disorders	EUCTR2013-002228-18-ES	2013
A PHASE 2 RANDOMIZED, DOUBLE-BLIND PLACEBO-CONTROLLED TRIAL OF MHAA4549A, A MONOCLONAL ANTIBODY, IN COMBINATION WITH OSELTAMIVIR VERSUS OSELTAMIVIR FOR TREATMENT OF SEVERE INFLUENZA A INFECTION	PER-077-14	2015
Dopamine transporter brain imaging to assess the effects of pramipexole vs levodopa on Parkinson disease progression		2002
Phase 3 Study of Venetoclax and Dexamethasone Compared with Pomalidomide and Dexamethasone in Subjects with t(11;14)-Positive Relapsed or Refractory Multiple Myeloma	EUCTR2017-003838-88-SE	2018
Effectiveness and safety evaluation of biomembrane hemicellulose versus curative collagenase in the treatment of chronic venous ulcers	RBR-2q5yjx	2016
EFFECT OF ADJUVANT TREATMENT WITH N - ACETYLCYSTEINE DURING 48 WEEKS ON THE LOSS OF GREY SUBSTANCE AND OXIDATIVE METABOLISM IN PATIENTS WITH EARLY ONSET PSYCHOTIC EPISODES: BLIND, PLACEBO-CONTROLLED, RANDOMIZED CLINICAL TRIAL	EUCTR2012-005435-87-ES	2012
Study to evaluate 3 type of treatment (masitinib + FOLFIRI, or masitinib alone, or FOLFIRI alone) in the treatment of patients with metastatic colorectal cancer that have received 2 or 3 previous therapies	EUCTR2013-000493-30-SK	2015

STUDY EVALUATING ALX-0061 ADMINISTERED SUBCUTANEOUSLY IN PATIENTS WITH SYSTEMIC LUPUS ERYTHEMATOSUS	PER-048-15	2016
A Randomized, Open-label, Multi-center, Phase 3, 2-arm Study Evaluating the Efficacy and Safety of Peg interferon Alfa-2b Low-dose Maintenance Monotherapy Versus Standard Supportive Care in Patients With Cirrhotic Hepatitis C Co-infected With Human Immunodeficiency Virus – The ENDURE Study. - ENDURE	EUCTR2005-003876-39-BE	2006
A Clinical trial to compare Acalabrutinib (ACP-196) versus investigator's choice standard treatment with Bendamustine/Rituximab or Idelalisib/Rituximab alone in patients with Relapsed or Refractory Chronic Lymphocytic Leukemia	EUCTR2015-004454-17-FR	2016
The effect of therapeutic models of Traditional Persian Medicine and Electro-Acupuncture on Polycystic Ovarian Syndrome	IRCT2015040921671N1	2015
A trial to test the effect of nintedanib together with chemotherapy in patients with acute myeloid leukemia	EUCTR2011-001086-41-DE	2011
TRIAL TO EVALUATE CHEMOTHERAPY WITH CAPECITABINE OR CAPECITABINE PLUS MITOMYCIN C IN ADVANCED BILIARY TRACT CANCER	EUCTR2011-002002-70-IT	2012
Franciscus Reumatoide Artritis en Cardiovasculaire interventie studie	NTR3873	2013
Exploratory Clinical Trial to Investigate Safety and Efficacy of COMBOPROFEN for treatment of muscular pain associated with DOMS	EUCTR2016-003139-39-ES	2017
NutriBrain: a controlled study to investigate the effect of a food supplement on brain development in very early born infants	ISRCTN96620855	2017
Safety and immunogenicity of a candidate tuberculosis (TB) vaccine in adults with TB disease	EUCTR2012-001820-36-EE	2012
Prospective, double-blind, randomised study of the efficacy of ketamine for oropharyngeal mucositis pain	ACTRN12619000108112	2019
Effect of SGLT2 inhibition on the function of small vessels of the heart in type 2 diabetes	EUCTR2017-000240-17-DK	2017
Effects of pulsed currents applied by cathode and anode in the treatment of category I pressure ulcers	ACTRN12617001534370	2017
A trial investigating different treatment plans of the medication nab-Paclitaxel, a chemotherapeutic agent in patients with breast cancer that has spread to other parts of the body	EUCTR2012-003058-10-IE	2013
A Phase I/II study to evaluate Trappsol Cyclo (hydroxypropyl-ß-cyclodextrin) in patients with Niemann-Pick disease type C (NPC-1) to assess what the drug does to the body, and what the body does to the drug, and the side effects and benefits experienced by patients	EUCTR2015-005761-23-GB	2016
Regulating intestinal microecologic health agent combination chemotherapy in the treatment of advanced colorectal cancer research	ChiCTR-INR-17011221	2017
Estimating the magnitude of clinical benefit of systemic therapy in patients with DCIS	Lazzeroni, , M; De Censi, A	2019
Treating the gut flora to improve heart failure	EUCTR2015-000192-27-NO	2015
A short term open, randomized cross over trial trial exploring the effect of carbonic anhydrase inhibition by acetazolamide on sleep apnea associated hypertension	EUCTR2013-004866-33-SE	2014
A Study to Assess Treatment Efficacy and Safety of Rifaximin DR Twice Daily in Subjects with Active Crohn's Disease for 52 weeks	EUCTR2014-001645-24-PL	2015
Aprepitant – effect and safety in treatment of atopic dermatitis	EUCTR2013-002029-40-SE	2013
Effects of dapagliflozin treatment on urinary proteomic patterns in patients with type 2 diabetes	EUCTR2015-000335-32-DK	2015
Treatment of osteoporosis in patients with rheumatoid arthritis	EUCTR2015-003638-28-DK	2015
A MULTICENTRE CLINICAL STUDY TO EVALUATE THE SAFETY AND EFFICACY OF LUM001, AN AGENT THAT INHIBITS BILE ACID REUPTAKE FROM THE INTESTINE, IN THE TREATMENT OF CHOLESTATIC LIVER DISEASE IN PAEDIATRIC PATIENTS WITH ALAGILLE SYNDROME	EUCTR2013-003832-54-GB	2013
Test if adding Vitamin D, a safe standard vitamin supplement to steroid tablets, will improve the clinical response to steroids in patients who are considered to be steroid resistant	EUCTR2008-002244-42-GB	2009
Research study of Ibrutinib in Combination with Venetoclax in patients with Mantle Cell Lymphoma	EUCTR2017-000129-12-HU	2017
A study in which patients with type 2 diabetes and who have been told they have a certain type of protein, albumin, in their urine, are given orally GKT137831 or placebo, no active, to determine whether GKT137831 has any effect in these patients and it is safe	EUCTR2013-002507-34-CZ	2013
Phase II Randomized, Parallel-Group Trial on PTK-ZK with or without DTIC in Patients with non-resectable Metastatic Malignant Melanoma	EUCTR2005-002192-32-DE	2006
Safety of Maraviroc in HIV-1 Infected Subjects Coinfected with Hepatitis B and/or Hepatitis C virus	EUCTR2010-021994-35-CZ	2011
A Randomized, Double-Blind, Placebo-Controlled Phase II Study of the Efficacy and Safety of Monotherapy Ontuxizumab (MORAb-004) Plus	Grothey, , A, Strosberg, JR, Renfro, LA, Hurwitz, HI, Marshall, JL, Safran, H, Guarino, MJ, Kim, GP, Hecht, JR, Weil, SC, Heyburn, J, Wang, W, Schweizer, C, O'Shannessy, DJ; Diaz, LA	2018

Best Supportive Care in Patients with Chemorefractory Metastatic Colorectal Cancer		
Integrated Care for Atrial Fibrillation Management: a Randomised Controlled Trial	ACTRN12616001109493	2016
This is a clinical study to investigate how well Biostate works in treatment of male patients below the age of 12 years who have a clotting factor deficiency that is aggravated by the development of antibodies. The antibodies are directed against the clotting factor that is given for replacement therapy and usually make therapy unsuccessful. The treatment used in this study is called immune tolerance therapy	EUCTR2010-020113-85-AT	2011
N-Acetylcysteine in patients with Sickle Cell Disease: reducing the incidence of daily life pain in patients with sickle cell disease	EUCTR2012-004892-37-NL	2013
Treatment of bile acid malabsorption with liraglutid	EUCTR2018-003575-34-DK	2018
Study to evaluate effect of MIN-102 compared to Placebo after 96 weeks of randomized, double-blind Treatment followed by open-label extension study to evaluate effect of long term treatment with MIN-102 on the progression of adrenomyeloneuropathy (AMN) in male patients	EUCTR2017-000748-16-ES	2017
Study to evaluate the use of allogeneic mesenchymal stromal cells for the treatment of skin disease in children with recessive dystrophic epidermolysis bullosa	ISRCTN46615946	2012
The analysis of the factors related to efficacy of asunaprevir and daclatasvir hydrochloride for hepatitis C-related liver disease	JPRN-UMIN000016633	2015
A 2 year study to investigate belimumab in membranous nephropathy	EUCTR2011-000242-38-ES	2012
Effect of Everolimus Initiation and Calcineurin Inhibitor Elimination on Cardiac Allograft Vasculopathy in De Novo Heart Transplant Recipients	Arora, , S, Andreassen, AK, Karason, K, Gustafsson, F, Eiskjær, H, Bøtker, HE, Rådegran, G, Gude, E, Ioanes, D, Solbu, D, Dellgren, G, Ueland, T, Aukrust, P; Gullestad, L	2018
PROXIMUS (PRotective role of OXcabazepine In MUltiple Sclerosis)	EUCTR2013-002419-87-GB	2014
Phase II trial for assessing different benznidazol regimens in the treatment of Chagas disease in adult patients on chronic phase . BERINECE project	EUCTR2016-003789-21-ES	2016
Obstructive sleep apnoea and retinal vasculature reactivity	ISRCTN78082983	2014
A Study to Assess Treatment Efficacy and Safety of Rifaximin DR Twice Daily in Subjects with Active Crohn's Disease for 52 weeks	EUCTR2014-001644-38-HU	2014
Study of efficacy, safety and tolerability of ACZ885 (canakinumab) in pediatric and young adult patients with sickle cell anemia	EUCTR2016-002101-19-GB	2016
Determination of efficacy and safety of azithromycin maintenance therapy for 6 months in subjects with primary ciliary dyskinesia	EUCTR2013-004664-58-DE	2014
A Phase 3, Randomized, Double-Blind, Placebo-Controlled Study to Evaluate the Efficacy and Safety of AG-348 in Not Regularly Transfused Adult Subjects With Pyruvate Kinase Deficiency	EUCTR2017-003823-31-DK	2018
Effects of benfotiamine treatment over 12 months on variables of peripheral diabetic neuropathy	EUCTR2013-001058-85-DE	2013
A Phase III Multicenter, Randomized, Double-Blind, placebo-controlled Study to Assess short-term changes in synovitis and structural damage outcomes in subjects with active Rheumatoid Arthritis and inadequate response to Methotrexate, Treated with Abatacept versus Placebo on a Background Therapy with Methotrexate Revised Protocol 03, incorporating Protocol Amendment 01 (Version 1.0, Date 16-May-2007), Protocol Amendment 03 (Version 1.0, Date 19-Mar-2008), Protocol Amendment 04 (Version 1.0, Date 05-Dec-2008), and Administrative Letter dated 13-Jun-2007	EUCTR2006-003768-67-SE	2007
Effects of diet and omeprazole in patients with eosinophilic oesophagitis – a multi-centre, randomised controlled trial and investigation of novel disease mechanisms	ACTRN12613001210763	2013
The purpose of this study is to evaluate a drug (LUM001) that may help treat the liver and control itching in Alagille Syndrome. In this study, all children who are eligible to enrol will take study drug for 18 weeks, followed by a 4 week period where they will either take LUM001 or placebo. After this 4 week period, all patients will go back on active study drug treatment for the remaining 26 weeks	EUCTR2013-005373-43-BE	2015
An open label, multicenter, non randomized phase II study to evaluate anti-tumor efficacy and safety of GM-CSF (sargramostim, Leukine®) associated with RCHOP chemotherapy and Rituximab (MabThera®) maintenance in patients with first-line advanced follicular non Hodgkin's lymphoma - FL2008 RCHOPGM	EUCTR2007-007056-33-FR	2008
Efficacy and safety study evaluating a new sublingual treatment in HDM allergic rhinitis	EUCTR2014-004223-46-BE	2015
A double-blind, placebo-controlled, randomized, multi-center phase II trial to assess the efficacy of Sorafenib added to standard primary therapy in patients with newly diagnosed AML = 60 years of age	EUCTR2008-004968-40-DE	2008
MINERAL (Magnetic-resonance Image of Nutraceutical Efficacy on Relapsing-ms Autoimmune Lesions) study: a novel nutraceutical formula NEUROASPIS PLP10® for the treatment of relapsing-remitting multiple sclerosis	ISRCTN06166891	2013

A prospective, multicenter, phase-II trial of ibrutinib plus venetoclax in patients with creatinine clearance >= 30 ml/min who have relapsed or refractory chronic lymphocytic leukemia (RR-CLL) with or without TP53 aberrations	NTR6249	2017
BUTEO: a clinical trial of BTT1023 in patients with primary sclerosing cholangitis (PSC)	ISRCTN11233255	2015
A study comparing several dose levels of LY3015014 in patients with high cholesterol studying the safety and ability of LY3015014 to reduce cholesterol	EUCTR2013-000622-55-PL	2013
Study to test whether apremilast is better than placebo (inactive substance in the same form as the drug) for the treatment of active Behçet's disease (oral ulcers). This study also tests how well the body tolerates apremilast. The study is conducted in several centers in different countries	EUCTR2014-002108-25-GR	2016
Investigation of correlation between antitumor effect of combined lapatinib/capecitabine therapy and p95HER2, PTEN and PIK3CA in patients with HER2 positive breast cancer	JPRN-UMIN000007153	2012
A Phase 3 Study of 2 Dose Regimens of Telaprevir in Combination with Peginterferon Alfa-2a (Pegasys) and Ribavirin (Copegus) in Treatment-Naive Subjects with Genotype 1 Chronic Hepatitis C	EUCTR2007-004720-20-IT	2008
A phase I/II trial for evaluating both safety and preliminary efficacy of one cycle of Promethera Hepastem in Urea Cycle Disorders and Crigler-Najjar Syndrome patients	EUCTR2011-004074-28-IT	2013
Therapeutic efficacy of Wilms tumor gene (WT1) mRNA-electroporated autologous dendritic cell vaccination in patients with myeloid malignancies and multiple myeloma: a phase II trial	EUCTR2009-015720-28-BE	2009
A randomized, double-blind, placebo-controlled, multicenter, dose-range, proof-of-concept, 24-week treatment study of IVA337 in adult subjects with nonalcoholic steatohepatitis (NASH)	EUCTR2016-001979-70-PL	2017
SOFIA Study	RPCEC00000118	2011
PROBE TRIAL proof-of-concept study on the use of rilpivirine as substitutive agent for the HAART nucleosidic backbone in virologic suppressed patients	EUCTR2013-002573-22-IT	2013
Interleukin-10 Levels and Clinical Outcome : comparison of Retroperitoneal versus Transperitoneal Approaches in Infra-Renal Abdominal Aorta Reconstruction	Erkalp, , K, Inan, B, Abut, Y, Teker, G, Basaranoglu, G, Kalko, Y; Gumus, F	2014
Developing microbiome restoration biomarkers for clostridium difficile infections: evaluation and plan of a prototype microbiome rehabilitation index™	Blount, , K, Jones, C, Carter, S, Deych, E; Shannon, B	2017
Preoperative oral immune-enhancing nutritional supplementation corrects TH1/TH2 imbalance in patients undergoing elective surgery for colorectal cancer	Matsuda, , A, Furukawa, K, Takasaki, H, Suzuki, H, Kan, H, Tsuruta, H, Shinji, S; Tajiri, T	2006
Osteoarthritis year in review: rehabilitation and outcomes	Maly, M	2014
COPART Risk Score Predicts Long-term Mortality in Peripheral Arterial Occlusive Disease	Hackl, , G, Belaj, K, Gary, T, Rief, P, Deutschmann, H, Seinost, G, Brodmann, M; Hafner, F	2015
Protocol for the Gut Bugs Trial: a randomised double-blind placebo-controlled trial of gut microbiome transfer for the treatment of obesity in adolescents	Leong, , KSW, Jayasinghe, TN, Derraik, JGB, Albert, BB, Chiavaroli, V, Svirskis, DM, Beck, KL, Conlon, CA, Jiang, Y, Schierding, W, Vatanen, T, Holland, DJ, O'Sullivan, JM; Cutfield, WS	2019
Randomized comparison of conventional carbon dioxide insufflation and abdominal wall lifting for laparoscopic cholecystectomy	Uen, , YH, Liang, AI; Lee, HH	2002
Systemic inflammatory response syndrome after pediatric congenital heart surgery: incidence, risk factors, and clinical outcome	Boehne, , M, Sasse, M, Karch, A, Dziuba, F, Horke, A, Kaussen, T, Mikolajczyk, R, Beerbaum, P; Jack, T	2017
The GRAFT Study: gut RecolonizAtion by Fecal Transplantation	NCT03621657	2018
Can fish oil supplementation improve endothelial function in asymptomatic offspring of patients with peripheral arterial disease?	Ian Spark, , J, Delaney, CL, Allan, RB, Ho, MHL; Miller, MD	2012
Effects of epidural anesthesia on stress-induced immune supression during major corrective spine surgery	Ezhevskaia, , AA, Prusakova, ZhB, Maksimova, LP, Sholkina, MN, Balmusova, EA; Ovechkin, AM	2014
Novel bioactive from Lactobacillus brevis DSM17250 to stimulate the growth of Staphylococcus epidermidis: a pilot study	Holz, , C, Benning, J, Schaudt, M, Heilmann, A, Schultchen, J, Goelling, D; Lang, C	2017
Nitrotyrosine (NT), a Nitrosative Stress Biomarker, Plasma Concentrations in Gallstone Disease and Cancer Patients	Saimanen, , I, Rahkola, D, Kuosmanen, V, Kärkkäinen, J, Selander, T, Holopainen, A, Aspinen, S; Eskelinen, M	2019
The Effects of Synbiotic Supplementation on Body Mass Index, Metabolic and Inflammatory Biomarkers, and Appetite in Patients with Metabolic Syndrome: a Triple-Blind Randomized Controlled Trial	Rabiei, , S, Hedayati, M, Rashidkhani, B, Saadat, N; Shakerhossini, R	2019
The elucidation of the action mechanism on the effectiveness of propagermanium for Crohn's disease	JPRN-UMIN000013534	2014
A multicenter, prospective, randomised, cross-over, exploratory, open pilot study to evaluate the influence on METAbolomics and PROteomics in end stage renal disease patients with post-dilution On-LINE-HDF versus conventional haemodialysis	DRKS00010917	2016
Efficacy of sleep apnoea (OSA) therapy for the reduction of atrial fibrilliation (AF) burden and morbidity in adults with OSA and AF	ACTRN12616000903482	2016
A Probiotic and Magnesium orotate combination for the treatment of depression	ACTRN12617000419369	2017

Faecal microbiota transplantation with tenofovir is superior to tenofovir alone in improving clinical outcomes in acute-on-chronic liver failure due to hepatitis B: an open label randomized controlled trial (NCT02689245)	Ahmad, , J, Kumar, M, Sarin, SK, Sharma, S, Choudhury, A, Jindal, A, Trehanpati, N, Kulkarni, A, Choudhury, S; Verma, A	2019
Structural and histological differences between connective tissue grafts harvested from the lateral palatal mucosa or from the tuberosity area	Sanz-Martín, , I, Rojo, E, Maldonado, E, Stroppa, G, Nart, J; Sanz, M	2019
Faecal microbiota transplantation with tenofovir is superior to tenofovir alone in improving clinical outcomes in acute-onchronic liver failure due to hepatitis B: an open label randomized controlled trial (NCT02689245)	Ahmad, , J, Kumar, M, Sarin, SK, Sharma, S, Choudhury, A, Jindal, A, Trehanpati, N, Kulkarni, A, Choudhury, S; Verma, A	2019
COlchicine for the preVention of postopErative atrial fibRillation in patients undergoing Coronary Artery By-pass Grafting	ISRCTN72835417	2013
Dental implant placement techniques	CTRI/2018/02/012200	2018
Probiotics and Orotic Acid for treatment resistant depression	ACTRN12616000401459	2016
Gonadotropin-releasing hormone agonist treatment reduced serum interleukin-6 concentrations in patients with ovarian endometriomas	Iwabe, , T, Harada, T, Sakamoto, Y, Iba, Y, Horie, S, Mitsunari, M; Terakawa, N	2003
Alteration in salivary proteins following non surgical periodontal therapy in generalized chronic periodontitis subjects	Sharma, , A, Nagtilak, S, Khattak, BP, Singh, G; Bano, T	2015
Multimodal treatment of perianal fistulas in Crohn's disease: seton versus anti-TNF versus advancement plasty (PISA): study protocol for a randomized controlled trial	de Groof, , EJ, Buskens, CJ, Ponsioen, CY, Dijkgraaf, MG, D'Haens, GR, Srivastava, N, van Acker, GJ, Jansen, JM, Gerhards, MF, Dijkstra, G, Lange, JF, Witteman, BJ, Kruyt, PM, Pronk, A, van Tuyl, SA, Bodelier, A, Crolla, RM, West, RL, Vrijland, WW, Consten, EC, Brink, MA, Tuynman, JB, de Boer, NK, Breukink, SO, Pierik, MJ, Oldenburg, B, van der Meulen, AE, Bonsing, BA, Spinelli, A, Danese, S, Sacchi, M, Warusavitarne, J, Hart, A, Yassin, NA, Kennelly, RP, Cullen, GJ, Winter, DC, Hawthorne, AB, Torkington, J; Bemelman, WA	2015
Phase II clinical trial of cediranib in patients with metastatic castration-resistant prostate cancer	Dahut, , WL, Madan, RA, Karakunnel, JJ, Adelberg, D, Gulley, JL, Turkbey, IB, Chau, CH, Spencer, SD, Mulquin, M, Wright, J, Parnes, HL, Steinberg, SM, Choyke, PL; Figg, WD	2013
Influence of prophylactic, endovascularly based normothermia on inflammation in patients with severe cerebrovascular disease: a prospective, randomized trial	Broessner, , G, Lackner, P, Fischer, M, Beer, R, Helbok, R, Pfausler, B, Schneider, D; Schmutzhard, E	2010
Fenofibrate in the management of AbdoMinal aortic anEurysm (FAME): study protocol for a randomised controlled trial	Rowbotham, , SE, Cavaye, D, Jaeggi, R, Jenkins, JS, Moran, CS, Moxon, JV, Pinchbeck, JL, Quigley, F, Reid, CM; Golledge, J	2017
A T-cell-based enzyme-linked immunospot assay for tuberculosis screening in Chinese patients with rheumatic diseases receiving infliximab therapy	Xie, , X, Chen, J-W, Li, F, Tian, J, Gao, J-S; Zhang, D	2011
Efficacy of thiopurines and adalimumab in preventing Crohn's disease recurrence in high-risk patients - a POCER study analysis	De Cruz, , P, Kamm, MA, Hamilton, AL, Ritchie, KJ, Krejany, EO, Gorelik, A, Liew, D, Prideaux, L, Lawrance, IC, Andrews, JM, Bampton, PA, Jakobovits, S, Florin, TH, Gibson, PR, Debinski, H, Gearry, RB, Macrae, FA, Leong, RW, Kronborg, I, Radford-Smith, G, Selby, W, Johnston, MJ, Woods, R, Elliott, PR, Bell, SJ, Brown, SJ, Connell, WR; Desmond, PV	2015
The relevance of hyperglycemia in pregnancy on perinatal outcomes and future burden of non-communicable diseases in europe and the figo guideline	Hod, M	2017
A randomized, open-label, comparative, 6-month trial of oral ultra-low doses of antibodies to tumor necrosis factor-alpha and diclofenac in rheumatoid arthritis	Dugina, , JL, Petrov, VI, Babayeva, AR, Martyushev-Poklad, AV, Tcherevkova, EV, Epstein, OI; Sergeeva, SA	2005
Adverse outcome and resistance to adjuvant antiestrogen therapy in node-positive postmenopausal breast cancer patients-The role of p53	Rahko, , E, Blanco, G, Bloigu, R, Soini, Y, Talvensaari-Mattila, A; Jukkola, A	2006
Lumbosacral radicular pain due to disc herniation	Vissers, K	2012
Coronary Artery Disease Evaluation in Rheumatoid Arthritis (CADERA): study protocol for a randomized controlled trial	Erhayiem, , B, Pavitt, S, Baxter, P, Andrews, J, Greenwood, JP, Buch, MH; Plein, S	2014
Extended adjuvant intermittent letrozole versus continuous letrozole in postmenopausal women with breast cancer (SOLE): a multicentre, open-label, randomised, phase 3 trial	Colleoni, , M, Luo, W, Karlsson, P, Chirgwin, J, Aebi, S, Jerusalem, G, Neven, P, Hitre, E, Graas, M-P, Simoncini, E, Kamby, C, Thompson, A, Loibl, S, Gavila, J, Kuroi, K, Marth, C, Muller, B, O'Reilly, S, Di Lauro, V, Gombos, A, Ruhstaller, T, Burstein, H, Ribi, K, Bernhard, J, Viale, G, Maibach, R, Rabaglio-Poretti, M, Gelber, RD, Coates, AS, Di Leo, A, Regan, MM; Goldhirsch, A	2018
Efficacy and safety of infliximab in active SLE: a pilot study	Uppal, , SS, Hayat, SJ; Raghupathy, R	2009
Treatment of chronic myeloid leukemia with autologous transplantation using peripheral blood stem cells or bone marrow cultured in IL-2 followed by IL-2, GM-CSF, and IFN-alpha administration	Hajek, , R, Zackova, D, Buchler, T, Penka, M, Krahulcova, E, Koristek, Z, Vinklarkova, J, Adler, J, Janovska, E, Indrak, K, Faber, E, Doubek, M, Klabusay, M, Oltova, A, Kuglik, P, Bourkova, L, Dusek, L, Mareschova, I, Mayer, J; Vorlicek, J	2003
Role of biomarkers in early postoperative period of lung transplantation	Suberviola, , B, Riera, J, Rellan, L, Sanchez, M, Robles, JC, Lopez, E, Vicente, R, Minambres, E; Santibanez, M	2016
Phase II study of adjuvant chemotherapy of S-1 plus oxaliplatin for patients with stage III gastric cancer after D2 gastrectomy	Shitara, , K, Chin, K, Yoshikawa, T, Katai, H, Terashima, M, Ito, S, Hirao, M, Yoshida, K, Oki, E, Sasako, M, Emi, Y; Tsujinaka, T	2017
Afatinib versus cisplatin plus gemcitabine for first-line treatment of Asian patients with advanced non-small-cell lung cancer harbouring EGFR mutations (LUX-Lung 6): an open-label, randomised phase 3 trial	Wu, , YL, Zhou, C, Hu, CP, Feng, J, Lu, S, Huang, Y, Li, W, Hou, M, Shi, JH, Lee, KY, Xu, CR, Massey, D, Kim, M, Shi, Y; Geater, SL	2014
Epigenetics and Periodontal Treatment	NCT02835898	2016

Intraoperative cerebral oxygenation, oxidative injury, and delirium following cardiac surgery	Lopez, , MG, Pandharipande, P, Morse, J, Shotwell, MS, Milne, GL, Pretorius, M, Shaw, AD, Roberts, LJ; Billings, FT	2017
IL-1beta, TNF-alpha, total antioxidative status and microbiological findings in chronic periodontitis treated with fluorescence-controlled Er: YAG laser radiation	Domínguez, , A, Gómez, C, García-Kass, AI; García-Nuñez, JA	2010
A Diet Low in FODMAPs Reduces Symptoms in Patients With Irritable Bowel Syndrome and A Probiotic Restores Bifidobacterium Species: a Randomized Controlled Trial	Staudacher, , HM, Lomer, MCE, Farquharson, FM, Louis, P, Fava, F, Franciosi, E, Scholz, M, Tuohy, KM, Lindsay, JO, Irving, PM; Whelan, K	2017
Tumor necrosis factor-alpha antibody for induction of remission in Crohn's disease	Akobeng, , AK; Zachos, M	2004
Can regional analgesia reduce the risk of recurrence after breast cancer? Methodology of a multicenter randomized trial	Sessler, , DI, Ben-Eliyahu, S, Mascha, EJ, Parat, MO; Buggy, DJ	2008
Effect of Parathyroidectomy on Cardiovascular Health	NCT02989428	2016
Steroids in biliary atresia: single surgeon, single centre, prospective study	Davenport, , M, Parsons, C, Tizzard, S; Hadzic, N	2013
Comparative Efficacy of Self-directed & Therapist-assisted Telehealth Parent Training Intervention for Children With ASD	NCT02721381	2016
Periodontal therapy reduces plasma levels of interleukin-6, C-reactive protein, and fibrinogen in patients with severe periodontitis and refractory arterial hypertension	Vidal, , F, Figueredo, CM, Cordovil, I; Fischer, RG	2009
E-23596 - Use of NCPAP Cycling to Wean Preterm Infants	NCT02114112	2014
Simultaneous hybrid coronary revascularization reduces postoperative morbidity compared with results from conventional off-pump coronary artery bypass	Kon, , ZN, Brown, EN, Tran, R, Joshi, A, Reicher, B, Grant, MC, Kallam, S, Burris, N, Connerney, I, Zimrin, D; Poston, RS	2008
A comparison of clinical variables that predict adverse outcome in term infants with severe respiratory failure randomised to a policy of extracorporeal membrane oxygenation or to conventional neonatal intensive care	Bennett, , CC, Johnson, A; Field, DJ	2002
Immunomodulatory effects of acupuncture in the treatment of allergic asthma: a randomized controlled study	Joos, , S, Schott, C, Zou, H, Daniel, V; Martin, E	2000
The clinical effects of dendritic cell vaccines combined with cytokine-induced killer cells intraperitoneal injected on patients with malignant ascites	Ai, , Y-Q, Cai, K, Hu, J-H, Jiang, L-W, Gao, Y-R, Zhao, H; Jia, S-C	2014
Neoadjuvant plus adjuvant or only adjuvant nab-paclitaxel plus gemcitabine for resectable pancreatic cancer - The NEONAX trial (AIO-PAK-0313), a prospective, randomized, controlled, phase II study of the AIO pancreatic cancer group	Ettrich, , TJ, Berger, AW, Perkhofer, L, Daum, S, Konig, A, Dickhut, A, Wittel, U, Wille, K, Geissler, M, Algul, H, Gallmeier, E, Atzpodien, J, Kornmann, M, Muche, R, Prasnikar, N, Tannapfel, A, Reinacher-Schick, A, Uhl, W; Seufferlein, T	2018
Doxycycline-loaded β-tricalcium phosphate release following EDTA root surface etching improved the clinical outcomes in chronic periodontitis: an in vivo study	Gamal, , AY, Kumper, RM; Al Gendy Ael, R	2013
Exogenous nitric oxide inhibits Rho-associated kinase activity in patients with angina pectoris: a randomized controlled trial	Maruhashi, , T, Noma, K, Fujimura, N, Kajikawa, M, Matsumoto, T, Hidaka, T, Nakashima, A, Kihara, Y, Liao, JK; Higashi, Y	2015
A phase 3 randomized, double-blind, placebo-controlled trial of ganitumab or placebo in combination with gemcitabine as first-line therapy for metastatic adenocarcinoma of the pancreas: the GAMMA trial	Fuchs, , CS, Azevedo, S, Okusaka, T, Van Laethem, JL, Lipton, LR, Riess, H, Szczylik, C, Moore, MJ, Peeters, M, Bodoky, G, Ikeda, M, Melichar, B, Nemecek, R, Ohkawa, S, Świeboda-Sadlej, A, Tjulandin, SA, Van Cutsem, E, Loberg, R, Haddad, V, Gansert, JL, Bach, BA; Carrato, A	2015
Randomized Clinical Trial of Labetalol Versus Hydralazine for Severe Hypertension in Obstetric Patients	NCT02050529	2014
HPR self-management exercise program associated to spa therapy increased the physical activity level of people with symptomatic knee osteoarthritis: a quasi-randomized controlled trial	Gay, , C, Auclair, C, Boisseau, N, Gerbaud, L; Coudeyre, E	2017
Short daily versus conventional hemodialysis for hypertensive patients: a randomized cross-over study	Zimmerman, , DL, Ruzicka, M, Hebert, P, Fergusson, D, Touyz, RM; Burns, KD	2014
The effects of elastic tubing-based resistance training compared with conventional resistance training in patients with moderate chronic obstructive pulmonary disease: a randomized clinical trial	Ramos, , EM, de Toledo-Arruda, AC, Fosco, LC, Bonfim, R, Bertolini, GN, Guarnier, FA, Cecchini, R, Pastre, CM, Langer, D, Gosselink, R; Ramos, D	2014
Effect of various mouthwashes on the levels of interleukin-2 and interferon-gamma in chronic gingivitis	Sharma, , S, Saimbi, CS, Koirala, B; Shukla, R	2008
Procalcitonin to guide duration of antibiotic therapy in intensive care patients: a randomized prospective controlled trial	Hochreiter, , M, Köhler, T, Schweiger, AM, Keck, FS, Bein, B, von Spiegel, T; Schroeder, S	2009
Incremental Value of Preoperative Copeptin for Predicting Myocardial Injury	Mauermann, , E, Bolliger, D, Seeberger, E, Puelacher, C, Corbiere, S, Filipovic, M, Seeberger, M, Mueller, C; Lurati Buse, G	2016
Association of HLA-DR genotypes and IL-1ra gene polymorphism with treatment failure of budesonide and disease patterns in Crohn's disease	Gelbmann, , CM, Rogler, G, Gierend, M, Gross, V, Schölmerich, J; Andus, T	2001
Ramucirumab versus placebo as second-line treatment in patients with advanced hepatocellular carcinoma following first-line therapy with sorafenib (REACH): a randomised, double-blind, multicentre, phase 3 trial	Zhu, , AX, Park, JO, Ryoo, BY, Yen, CJ, Poon, R, Pastorelli, D, Blanc, JF, Chung, HC, Baron, AD, Pfiffer, TE, Okusaka, T, Kubackova, K, Trojan, J, Sastre, J, Chau, I, Chang, SC, Abada, PB, Yang, L, Schwartz, JD; Kudo, M	2015
Comparative evaluation of adjunctive oral irrigation in diabetics	Al-Mubarak, , S, Ciancio, S, Aljada, A, Mohanty, P, Ross, C; Dandona, P	2002
Risk of post-operative complications among Crohn's disease patients treated pre-operatively with vedolizumab. A matched case-control study	Kim, , JY, Zaghiyan, K; Fleshner, P	2018

Prospective Dutch colorectal cancer cohort: an infrastructure for long-term observational, prognostic, predictive and (randomized) intervention research	Burbach, , JPM, Kurk, SA, Coebergh van den Braak, RRJ, Dik, VK, May, AM, Meijer, GA, Punt, CJA, Vink, GR, Los, M, Hoogerbrugge, N, Huijgens, PC, Ijzermans, JNM, Kuipers, EJ, de Noo, ME, Pennings, JP, van der Velden, AMT, Verhoef, C, Siersema, PD, van Oijen, MGH, Verkooijen, HM; Koopman, M	2016
Peripheral blood stem cell mobilization with cyclophosphamide in combination with G-CSF, GM-CSF, or sequential GM-CSF/G-CSF in non-Hodgkin's lymphoma patients: a randomized prospective study	Gazitt, , Y, Callander, N, Freytes, CO, Shaughnessy, P, Liu, Q, Tsai, TW; Devore, P	2000
The functional dyspepsia treatment trial (FDTT): 5-HTT LPR and GNbeta3 825C>T are not pharmacogenetic markers for FD antidepressant treatment response	Saito, , YA, Almazar, AE, Locke, GR, Bouras, EP, Howden, CW, Lacy, BE, DiBaise, JK, Prather, CM, Abraham, B, El-Serag, H, Moayyedi, P, Szarka, LA, Herrick, LM, Tilkes, KE, Schleck, CD, Zinsmeister, AR; Talley, NJ	2014
Study design for a randomised controlled trial to explore the modality and mechanism of Tai Chi in the pulmonary rehabilitation of chronic obstructive pulmonary disease	Fu, , JJ, Min, J, Yu, PM, McDonald, VM; Mao, B	2016
The effects of ozone therapy on periodontal therapy: a randomized placebo-controlled clinical trial	Tasdemir, , Z, Oskaybas, MN, Alkan, AB; Cakmak, O	2019
The Marigot Osteoarthritis Nutritional Intervention (MOANi) Trial	NCT03106584	2017
Effects of phospholipid-coated extracorporeal circuits on clinical outcome parameters and systemic inflammatory response in coronary artery bypass graft patients	Schulz, , CM, Pritisanac, A, Schütz, A, Kilger, E, Platzer, H, Reichart, B; Wildhirt, SM	2002
Evaluation of Efficacy and Safety of Neoadjuvant Treatment With Pamrevlumab in Combination With Chemotherapy (Gemcitabine and Nab-paclitaxel) in Locally Advanced Pancreatic Cancer	NCT03941093	2019
Radiation treatment intensification	Belderbos, J	2016
Comparison of an intravenous pulse of methylprednisolone versus oral corticosteroid in severe acute rheumatic carditis: a randomized clinical trial	Câmara, , EJ, Braga, JC, Alves-Silva, LS, Câmara, GF; da Silva Lopes, AA	2002
Efficacy trial of bioresonance in children with atopic dermatitis	Schöni, , MH, Nikolaizik, WH; Schöni-Affolter, F	1997
Changes in the Risk Factors of Coronary Heart Disease Observed After Scaling and Root Planing	NCT02417376	2015
Prognostic value of serial blood S100B determinations in stage IIB-III melanoma patients: a corollary study to EORTC trial 18952	Bouwhuis, , MG, Suciu, S, Kruit, W, Salès, F, Stoitchkov, K, Patel, P, Cocquyt, V, Thomas, J, Liénard, D, Eggermont, AM; Ghanem, G	2011
Spanish Diabetes Self-Management Program	NCT00496145	2007
Treatment of older patients with HER2-positive metastatic breast cancer with pertuzumab, trastuzumab, and docetaxel: subgroup analyses from a randomized, double-blind, placebo-controlled phase III trial (CLEOPATRA)	Miles, , D, Baselga, J, Amadori, D, Sunpaweravong, P, Semiglazov, V, Knott, A, Clark, E, Ross, G; Swain, SM	2013
Efficacy of Combined Photoablative-Photodynamic Diode Laser Therapy Adjunctive to Scaling and Root Planing in Periodontitis: randomized Split-Mouth Trial with 4-Year Follow-Up	Giannelli, , M, Formigli, L, Lorenzini, L; Bani, D	2015
Effects of an immuno-enhanced diet containing antioxidants in esophageal cancer surgery following neoadjuvant therapy	Aiko, , S, Kumano, I, Yamanaka, N, Tsujimoto, H, Takahata, R; Maehara, T	2012
The use of dose-intensified chemotherapy in the treatment of metastatic nonseminomatous testicular germ cell tumors. German Testicular Cancer Study Group	Bokemeyer, , C, Harstrick, A, Beyer, J, Metzner, B, Rüther, U, Hartmann, JT, Holstein, K, Derigs, HG, de Wit, R, Casper, J, Schöffski, P, Kührer, I, Illiger, HJ, Kempf, B, Reichle, A, Föller, A, Hossfeld, DK, Fischer, JT, Berdel, WE, Gerhartz, HH, Kirchner, H, Pflüger, KH, Ostermann, H, Kanz, L; Schmoll, HJ	1998
The efficacy of lactobacillus reuteri 4659 in reducing abdominal symptoms and inflammatory biomarkers in acute uncomplicated diverticulatis: a double-blind, randomized, placebo-controlled trial	Petruzziello, , C, Franceschi, F, Covino, M, Migneco, A; Ojetti, V	2018
Lower tidal volume strategy (3 ml/kg) combined with extracorporeal CO2 removal versus 'conventional' protective ventilation (6 ml/kg) in severe ARDS: the prospective randomized Xtravent-study	Bein, , T, Weber-Carstens, S, Goldmann, A, Muller, T, Staudinger, T, Brederlau, J, Muellenbach, R, Dembinski, R, Graf, BM, Wewalka, M, Philipp, A, Wernecke, K-D, Lubnow, M; Slutsky, AS	2013
Carpal tunnel syndrome and musculoskeletal symptoms in postmenopausal women with early breast cancer treated with exemestane or tamoxifen after 2-3 years of tamoxifen: a retrospective analysis of the Intergroup Exemestane Study	Mieog, , JSD, Morden, JP, Bliss, JM, Coombes, RC; Van de Velde, CJH	2012
Cytokine profiles in pemphigus vulgaris patients treated with intravenous immunoglobulins as compared to conventional immunosuppressive therapy	Keskin, , DB, Stern, JN, Fridkis-Hareli, M; Razzaque Ahmed, A	2008
The inflammatory response to recycled pericardial suction blood and the influence of cell-saving	Svenmarker, , S; Engström, KG	2003
Curative effect of Xuebijing injection on severe pulmonary contusion	Chen, , Y, Tong, H, Zhang, W, Zhang, X, Pan, Z, Qiu, J, Pan, R; Su, L	2013
Clinical observation on the effect of minimally invasive flapless technique and implant prognosis in oral implants	Jiao, , G; Gao, M	2018
Short- versus long-term antimicrobial treatment for acute hematogenous osteomyelitis of childhood: prospective, randomized trial on 131 culture-positive cases	Peltola, , H, Pääkkönen, M, Kallio, P; Kallio, MJ	2010
Grape resveratrol increases serum adiponectin and downregulates inflammatory genes in peripheral blood mononuclear cells: a triple-blind,	Tomé-Carneiro, , J, Gonzálvez, M, Larrosa, M, Yáñez-Gascón, MJ, García-Almagro, FJ, Ruiz-Ros, JA, Tomás-Barberán, FA, García-Conesa, MT; Espín, JC	2013

placebo-controlled, one-year clinical trial in patients with stable coronary artery disease		
Carpal tunnel syndrome and musculoskeletal symptoms in postmenopausal women with early breast cancer treated with exemestane or tamoxifen after 2-3 years of tamoxifen: a retrospective analysis of the Intergroup Exemestane Study	Mieog, , JS, Morden, JP, Bliss, JM, Coombes, RC; van de Velde, CJ	2012
A phase II study of neoadjuvant combination chemotherapy with docetaxel, cisplatin, and S-1 for locally advanced resectable gastric cancer: nucleotide excision repair (NER) as potential chemoresistance marker	Hirakawa, , M, Ohnuma, H, Takayama, T, Sagawa, T, Nobuoka, T, Harada, K, Miyamoto, H, Sato, Y, Takahashi, Y, Katsuki, S, Hirayama, M, Takahashi, M, Ono, M, Maeda, M, Takada, K, Hayashi, T, Sato, T, Miyanishi, K, Takimoto, R, Kobune, M, Hirata, K; Kato, J	2013
Local or Regional or General Anesthesia for Hernia Repair: a Randomized Controlled Trial	NCT01845376	2013
Ferritin levels, inflammatory biomarkers, and mortality in peripheral arterial disease: a substudy of the Iron (Fe) and Atherosclerosis Study (FeAST) Trial	Depalma, , RG, Hayes, VW, Chow, BK, Shamayeva, G, May, PE; Zacharski, LR	2010
Pravastatin immunomodulates IL-6 and C-reactive protein, but not IL-1 and TNF-alpha, in cardio-pulmonary bypass	Caorsi, , C, Pineda, F; Munoz, C	2008
Clinical trial of a novel non-thermal LED array for reversal of photoaging: clinical, histologic, and surface profilometric results	Weiss, , RA, McDaniel, DH, Geronemus, RG; Weiss, MA	2005
28th Annual Meeting of the European Association of Cardiothoracic Anaesthesiologists, EACTA 2013		2014
Shenfu injection for improving cellular immunity and clinical outcome in patients with sepsis or septic shock	Zhang, , N, Liu, J, Qiu, Z, Ye, Y, Zhang, J; Lou, T	2017
Hypocaloric versus normocaloric nutrition in critically ill patients	Petros, , S, Horbach, M, Weidhase, L, Seidel, F, Schwabe, K, Vogel, I; Dafova, E	2010
Effect of a Sugar-Free Chewing Gum Containing Magnolia Bark Extract on Caries Lesions in Healthy Adult Volunteers	NCT02310308	2014
Timing of pre-operative Beta-blocker treatment in vascular surgery patients: influence on post-operative outcome	Flu, , WJ, van Kuijk, JP, Chonchol, M, Winkel, TA, Verhagen, HJ, Bax, JJ; Poldermans, D	2010
Efficacy and safety of abiraterone acetate in an elderly patient subgroup (aged 75 and older) with metastatic castration-resistant prostate cancer after docetaxel-based chemotherapy	Mulders, , PF, Molina, A, Marberger, M, Saad, F, Higano, CS, Chi, KN, Li, J, Kheoh, T, Haqq, CM; Fizazi, K	2014
The anti-inflammatory effect of locally delivered nano-doxycycline gel in therapy of chronic periodontitis	Madi, , M, Pavlic, V, Samy, W; Alagl, A	2018
Effectiveness of Narrative Medicine on Pain Intensity and Quality of Life	NCT00106717	2005
Abatacept for the treatment of adults with psoriatic arthritis: patient selection and perspectives		2018
Study of Pamidronate for the Prevention of Heterotopic Ossification	NCT00262392	2005
Cardiovascular patients: regional anesthesia revisited	Moka, , E, Argyra, E, Siafaka, I; Vadalouca, A	2014
Nutritional effect of nandrolone decanoate in predialysis patients with chronic kidney disease	Eiam-Ong, , S, Buranaosot, S, Eiam-Ong, S, Wathanavaha, A; Pansin, P	2007
Urinary cystatin C and acute kidney injury after cardiac surgery	Koyner, , JL, Garg, AX, Shlipak, MG, Patel, UD, Sint, K, Hong, K, Devarajan, P, Edelstein, CL, Zappitelli, M, Thiessen-Philbrook, H; Parikh, CR	2013
Sensitivity and specificity of a point-of-care matrix metalloproteinase 9 immunoassay for diagnosing inflammation related to dry eye	Sambursky, , R, Davitt, WF, Latkany, R, Tauber, S, Starr, C, Friedberg, M, Dirks, MS; McDonald, M	2013
The intraoperative effect of pentoxifylline on the inflammatory process and leukocytes in cardiac surgery patients undergoing cardiopulmonary bypass	Cağli, , K, Ulaş, MM, Ozişik, K, Kale, A, Bakuy, V, Emir, M, Balci, M, Topbaş, M, Sener, E; Taşdemir, O	2005
Periodontal therapy reduces the severity of active rheumatoid arthritis in patients treated with or without tumor necrosis factor inhibitors	Ortiz, , P, Bissada, NF, Palomo, L, Han, YW, Al-Zahrani, MS, Panneerselvam, A; Askari, A	2009
Incidental Genomics	NCT03597165	2018
Final analysis of a randomised trial comparing pembrolizumab versus investigator-choice chemotherapy for ipilimumab-refractory advanced melanoma	Hamid, , O, Puzanov, I, Dummer, R, Schachter, J, Daud, A, Schadendorf, D, Blank, C, Cranmer, LD, Robert, C, Pavlick, AC, Gonzalez, R, Hodi, FS, Ascierto, PA, Salama, AKS, Margolin, KA, Gangadhar, TC, Wei, Z, Ebbinghaus, S, Ibrahim, N; Ribas, A	2017
Comparison of the acute-phase response after laparoscopic versus open aortobifemoral bypass surgery: a substudy of a randomized controlled trial	Krog, , AH, Sahba, M, Pettersen, EM, Sandven, I, Thorsby, PM, Jørgensen, JJ, Sundhagen, JO; Kazmi, SS	2016
Presentations of major peripheral arterial disease and risk of major outcomes in patients with type 2 diabetes: results from the ADVANCE-ON study	Mohammedi, , K, Woodward, M, Hirakawa, Y, Zoungas, S, Colagiuri, S, Hamet, P, Harrap, S, Poulter, N, Matthews, DR, Marre, M; Chalmers, J	2016
Biomarker analysis from the OpACIN trial (Neo-/adjuvant ipilimumab + nivolumab (IPI+NIVO) in palpable stage 3 melanoma)	Rozeman, , L, Franchi, L, Kuilman, T, Krijgsman, O, Van Akkooi, A, Kvistborg, P, Van Thienen, H, Stegenga, B, Cullen, D, Lamon, B, Haanen, J, Cesano, A, Warren, S, Broeks, A, Schumacher, T; Blank, C	2017
Plasma phospholipid omega-3 fatty acids and incidence of postoperative atrial fibrillation in the OPERA trial	Wu, , JH, Marchioli, R, Silletta, MG, Macchia, A, Song, X, Siscovick, DS, Harris, WS, Masson, S, Latini, R, Albert, C, Brown, NJ, Lamarra, M, Favaloro, RR; Mozaffarian, D	2013
Perioperative Δ9-THC for Postsurgical Pain	NCT01790555	2013
Effectiveness of Emdogain in the periodontal treatment	Kurhańska-Flisykowska, , A, Łojewski, W; Wyganowska-Swiatkowska, M	2012

Mepolizumab for the treatment of eosinophilic granulomatosis with polyangiitis		2017
Phase II randomised discontinuation trial of cabozantinib in patients with advanced solid tumours	Schöffski, , P, Gordon, M, Smith, DC, Kurzrock, R, Daud, A, Vogelzang, NJ, Lee, Y, Scheffold, C; Shapiro, GI	2017
Persistent clinical response to the anti-TNF-alpha antibody infliximab in patients with ankylosing spondylitis over 3 years	Braun, , J, Baraliakos, X, Brandt, J, Listing, J, Zink, A, Alten, R, Burmester, G, Gromnica-Ihle, E, Kellner, H, Schneider, M, Sörensen, H, Zeidler, H; Sieper, J	2005
AB-Intra- and Post-Operative Measures of Auditory Function	NCT03685461	2018
Non-surgical periodontal therapy affects metabolic control in diabetics: a randomized controlled clinical trial	Moeintaghavi, , A, Arab, HR, Bozorgnia, Y, Kianoush, K; Alizadeh, M	2012
Microbiological effect of the use of an ultrasonic device and iodine irrigation in patients with severe chronic periodontal disease: a randomized controlled clinical study	Leonhardt, , A, Bergström, C, Krok, L; Cardaropoli, G	2007
Nicotine treatment improves Toll-like receptor 2 and Toll-like receptor 9 responsiveness in active pulmonary sarcoidosis	Julian, , MW, Shao, G, Schlesinger, LS, Huang, Q, Cosmar, DG, Bhatt, NY, Culver, DA, Baughman, RP, Wood, KL; Crouser, ED	2013
Effect of puerarin preconditioning on cytokine levels in patients undergoing cardiopulmonary bypass in perioperative period	Jiang, , XX, Li, Y; Wu, YB	2009
Pressure-Controlled vs Volume-Controlled Ventilation During One Lung Ventilation	NCT00975468	2009
Prehabilitation in Esophageal Surgery (PRESS)	NCT03798951	2019
Randomised clinical trial: the ileal bile acid transporter inhibitor A3309 vs. placebo in patients with chronic idiopathic constipation–a double-blind study	Simrén, , M, Bajor, A, Gillberg, PG, Rudling, M; Abrahamsson, H	2011
Liposomal cisplatin combined with paclitaxel versus cisplatin and paclitaxel in non-small-cell lung cancer: a randomized phase III multicenter trial	Stathopoulos, , GP, Antoniou, D, Dimitroulis, J, Michalopoulou, P, Bastas, A, Marosis, K, Stathopoulos, J, Provata, A, Yiamboudakis, P, Veldekis, D, Lolis, N, Georgatou, N, Toubis, M, Pappas, Ch; Tsoukalas, G	2010
Perioperative therapy in HER2+ patients	A'Hern, , RP; Bundred, NJ	2009
STAR VaS–Short Term Atorvastatin Regime for Vasculopathic Subjects: a randomized placebo-controlled trial evaluating perioperative atorvastatin therapy in noncardiac surgery	Neilipovitz, , DT, Bryson, GL; Taljaard, M	2012
Peri-operative acute phase response and cytokine releasein women with breast cancer: modulation bypolyadenylic-polyuridylic acid	Khan, , AL, Larsen, F, Heys, SD; Eremin, O	1999
The Fitness, Game Bike Adherence, Motivation and Exercise Study	NCT01373762	2011
Standard Versus Intensity-Modulated Pelvic Radiation Therapy in Treating Patients With Endometrial or Cervical Cancer	NCT01672892	2012
Pre- or Postoperative Accelerated Radiotherapy	NCT03783364	2018
Circulating cardiac biomarkers and postoperative atrial fibrillation in the OPERA trial	Masson, , S, Wu, JH, Simon, C, Barlera, S, Marchioli, R, Mariani, J, Macchia, A, Lombardi, F, Vago, T, Aleksova, A, Dreas, L, Favaloro, RR, Hershson, AR, Puskas, JD, Dozza, L, Silletta, MG, Tognoni, G, Mozaffarian, D; Latini, R	2015
Effects of an aquatic physical exercise program on glycemic control and perinatal outcomes of gestational diabetes: study protocol for a randomized controlled trial	da Silva, , JR, Borges, PS, Agra, KF, Pontes, IA; Alves, JG	2013
Biomarkers of anti-angiogenic therapy in metastatic colorectal cancer (mCRC): original data and review of the literature	Pohl, , M, Werner, N, Munding, J, Tannapfel, A, Graeven, U, Nickenig, G, Schmiegel, W; Reinacher-Schick, A	2011
Sequential combined treatment with allopurinol and benznidazole in the chronic phase of Trypanosoma cruzi infection: a pilot study	Perez-Mazliah, , DE, Alvarez, MG, Cooley, G, Lococo, BE, Bertocchi, G, Petti, M, Albareda, MC, Armenti, AH, Tarleton, RL, Laucella, SA; Viotti, R	2013
Treatment of HIV/AIDS patients with Glutamine Dipeptide	RBR-843tnq	2015
A study to assess the safety and tolerability of combining G1T28 with etoposide and carboplatin therapy and to evaluate the effect of G1T28 on blood cell production affected by chemotherapy	EUCTR2016-001583-11-HU	2016
An Open-Label, Study to Evaluate safety and efficacy of Edoxaban tosylate in children with heart disease at risk of a blood clot	EUCTR2017-000475-90-ES	2017
Melatonin reduces oxidative stress in surgical neonates	Gitto, , E, Romeo, C, Reiter, RJ, Impellizzeri, P, Pesce, S, Basile, M, Antonuccio, P, Trimarchi, G, Gentile, C, Barberi, I; Zuccarello, B	2004
Efficacy and safety of pimecrolimus cream in the long-term management of atopic dermatitis in children	Wahn, , U, Bos, JD, Goodfield, M, Caputo, R, Papp, K, Manjra, A, Dobozy, A, Paul, C, Molloy, S, Hultsch, T, Graeber, M, Cherill, R; de Prost, Y	2002
Cediranib or placebo in combination with cisplatin and gemcitabine chemotherapy for patients with advanced biliary tract cancer (ABC-03): a randomised phase 2 trial	Valle, , JW, Wasan, H, Lopes, A, Backen, AC, Palmer, DH, Morris, K, Duggan, M, Cunningham, D, Anthoney, DA, Corrie, P, Madhusudan, S, Maraveyas, A, Ross, PJ, Waters, JS, Steward, WP, Rees, C, Beare, S, Dive, C; Bridgewater, JA	2015
SB-480848/026: an International, Multicenter, Randomized, Placebo-controlled, Parallel-group, 1 Year Treatment, Integrated Biomarkers and Imaging Study in Subjects with Angiographically Documented Coronary Heart Disease (CHD) to Examine the Effects of the Novel Lipoprotein-associated Phospholipase A2 (Lp-PLA2) inhibitor SB-480848 on Intermediate Cardiovascular Endpoints, Patient Safety and Tolerability	EUCTR2005-001556-20-DE	2005
The effect of non-surgical periodontal therapy on periodontal status and systemic metabolic status in end-stage renal disease patients	ChiCTR-TRC-14005045	2014

Randomized study to assess the added value of Laromustine in combination with standard remission-induction chemotherapy in patients aged 18-65 years with previously untreated acute myeloid leukemia (AML) or myelodysplasia (MDS) (RAEB with IPSS >= 1.5)	NTR1446	2008
treatment of gum disease/ pyorrhoea	CTRI/2017/03/008231	2017
Exploration of TNF-alpha Blockade with golimumab in the Induction of Clinical Remission in Patients with Early Peripheral Spondyloarthritis (SpA) according to ASAS-criteria	EUCTR2011-003678-97-BE	2011
Treating bone defects in patients with gum diseases using bone graft	CTRI/2018/04/013441	2018
Effect of pomegranate supplement on Rheumatoid Arthritis patients	IRCT201202183236N2	2012
Eliminating periodontal infection in patients with type 2 diabetes	ISRCTN57210949	2010
An Open-Label, Study to Evaluate safety and efficacy of Edoxaban tosylate in children with heart disease at risk of a blood clot	EUCTR2017-000475-90-HU	2017
The effect of peri-operative anti-inflammatory treatment on postoperative muscle weakness and muscle fatigue in elderly elective surgery patients	ISRCTN51508868	2011
Response to Optimal Selection of neo-adjuvant Chemotherapy in Operable breast cancer	EUCTR2013-004307-39-GB	2015
The effect of vitamin D supplementation in Non Alcoholic Fatty Liver Disease treatment	IRCT2012071810333N1	2012
A multicenter randomized phase III trial of neo-adjuvant chemotherapy followed by surgery and chemotherapy or by surgery and chemoradiotherapy in resectable gastric cancer (CRITICS-study: chemoRadiotherapy after Induction chemoTherapy In Cancer of the Stomach) - CRITICS	EUCTR2006-004130-32-DK	2010
A clinical trial to study the effect of periodontal treatment on rheumatoid arthritis disease	CTRI/2017/01/007713	2017
A RANDOMIZED MULTICENTER, DOUBLE-BLIND, PLACEBO-CONTROLLED COMPARISON OF CHEMOTHERAPY PLUS TRASTUZUMAB PLUS PLACEBO VERSUS CHEMOTHERAPY PLUS TRASTUZUMAB PLUS PERTUZUMAB AS ADJUVANT THERAPY IN PATIENTS WITH OPERABLE HER2-POSITIVE PRIMARY BREAST CANCER	PER-092-11	2011
Biomarkers in diagnosis & treatment of patients with Crohn?s disease treated with immunosuppressants	EUCTR2011-003966-34-ES	2011
Randomized phase 3 clinical trial for patients with metastasized childhood renal tumour	EUCTR2018-000533-13-DE	2018
A randomised, double-blind, placebo-controlled, parallel-group study to investigate the effect of Symbicort® and Pulmicort® on HAT and HDAC expression and activity in induced sputum cells obtained from COPD patients	EUCTR2005-003297-13-GB	2005
Humoral immune response during coronary artery bypass grafting: a comparison of limited approach, "off-pump" technique, and conventional cardiopulmonary bypass	Diegeler, , A, Doll, N, Rauch, T, Haberer, D, Walther, T, Falk, V, Gummert, J, Autschbach, R; Mohr, FW	2000
A clinical trial to study the effect of a drug, curcumin in patients with periodontitis	CTRI/2019/01/016927	2019
Prevention of the return of nephrotic syndrom in children by adding levamisole to the prednison treatment	EUCTR2017-001025-41-NL	2017
CIMAvax-EGF(R) in metastatic asymptomatic or mildly symptomatic Castration Resistant Prostate Cancer patients. Phase II-III Clinical Trial	RPCEC00000291	2018
Periodontal therapy in diabetic subjects	ISRCTN11742127	2008
Myocardial and lung injury after cardiopulmonary bypass: role of interleukin (IL)-10	Giomarelli, , P, Scolletta, S, Borrelli, E; Biagioli, B	2003
Suppression of the hormone aldosterone to diminish scar tissue in heart in patients with atrial fibrillation	EUCTR2013-000797-30-DK	2013
The Effect of Grape Seed and Grapefruit Seed Extracts on Bone Regeneration: randomized clinical trial	RBR-56q7h9	2017
Uncemented total hip implant and subcutaneous injection of Denosumab for patients with degenerative joint disease of the hip. A randomised double blind placebo controlled study on the effects on bone evaluated with bone densitometry, uptake of Fluoride isotop measure with Positron Emission Tomography and Computed Tomography, and blood samples analyzed for bone turnover	EUCTR2011-001481-18-SE	2011
A PHASE, DOUBLE-BLIND, PLACEBO-CONTROLLED,MULTI-CENTER, RANDOMIZED WITHDRAWAL DESIGN TRIAL USING ADAPTIVE RANDOMIZATION COMPARING Z102 WITH PLACEBO IN PATIENTS WITH MODERATE TO SEVERE RHEUMATOID ARTHRITIS	PER-009-12	2012
A study to assess the safety and efficacy of a new medicine SBT-020 in patients with Early Stage Huntington's Disease	EUCTR2016-003730-25-NL	2017
Non-surgical periodontal therapy in rheumatoid arthritis patients	ISRCTN60187959	2016
Chinese herbal medicine bi min fang for allergic rhinitis: protocol for a double-blind, double-dummy, randomized controlled trial	Luo, , Q, Zhou, S, Li, X, Chen, Q, Lin, W, Lu, L, Li, H, Chen, C, Chen, W; Li, Y	2019

Use of circulating tumour DNA (ctDNA) results to inform the decision for adjuvant chemotherapy in patients with locally advanced rectal cancer who have been treated with pre-operative chemo-radiation and surgery	ACTRN12617001560381	2017
Alternatives to prophylactic antibiotics for the treatment of recurrent urinary tract infection in women	EUCTR2015-003487-36-GB	2015
Efficacy of enteral nutrition in adult Ileal Crohn's disease- the RICE study	ACTRN12616000251426	2016
Evaluation of efficacy and safety of using Nintedanib (Vargatef®) as part of the neoadjuvant and adjuvant treatment surrounding interval debulking surgery in patients with advanced ovarian cancer	EUCTR2011-006288-23-FR	2012
Surgical treatment for secundum atrial septal defects in patients >40 years old. A randomized clinical trial	Attie, , F, Rosas, M, Granados, N, Zabal, C, Buendía, A; Calderón, J	2001
Randomized study with a run-in feasibility phase to assess the added value of Clofarabine in combination with standard remission-induction chemotherapy in patients aged 18-65 years with previously untreated acute myeloid leukemia (AML) or myelodysplasia (MDS) (RAEB with IPSS =>1.5)	NTR2187	2010
Effect of ginger supplementation in treatment of type 2 diabetic patients with chronic periodontitis	IRCT20170304032874N2	2018
The Effect of Alogliptin on Cardiovascular Disease in Patients with Acute Coronary Syndromes	JPRN-UMIN000010093	2013
Open randomised phase II study evaluating the anti-tumour activity, safety and pharmacology of two different dose regimens of IPH 2101, a human monoclonal anti-KIR antibody, in patients with multiple myeloma in stable partial response after a first line therapy	EUCTR2009-012136-33-FR	2009
Upfront debulking surgery versus neoadjuvant chemotherapy in ovarian cancer	ISRCTN67331344	2010
A Randomized Phase III Study Comparing Conventional Dose Treatment Using a Combination of Lenalidomide, Bortezomib and Dexamethasone (RVD) to High-Dose Treatment with Peripheral Stem Cell Transplant in the Initial Management of Myeloma in Patients up to 65 Years of Age (IFM/DFCI 2009) - IFM/DFCI 2009	EUCTR2009-016871-32-FR	2010
Randomised, double-blind, multicentric phase III trial evaluating the safety and benefit of adding everolimus to adjuvant hormone therapy in women with poor prognosis, ER+ and HER2- primary breast cancer who remain free of disease	EUCTR2012-003187-44-BE	2013
Trial of Perioperative Endocrine Therapy - Individualising Care (POETIC)	EUCTR2007-003877-21-GB	2008
The effect of Ginger supplementation in the treatment of type 2 Diabetic patients with Periodontitis	IRCT2017030432874N1	2017
Survivors of intensive care with type 2 diabetes and the effect of shared care follow-up clinics: the SWEET-AS feasibility study	ACTRN12616000206426	2016
Circulating Tumour DNA Analysis Informing Adjuvant Chemotherapy in Early Stage Pancreatic Cancer: a Multicentre Randomised Study (DYNAMIC- Pancreas)	ACTRN12618000335291	2018
Surgery alone In low rectal cancer	ISRCTN02406823	2013
Single Center Pilot Clinical Trial on cell therapy in acute ictus	EUCTR2011-003551-18-ES	2011
A study to determine the interrelationship between diabetes and gum disease	CTRI/2014/08/004849	2014
The SSTARS (STeroids and Stents Against Re-Stenosis) Trial: different stent alloys and the use of peri-procedural oral corticosteroids to prevent in-segment restenosis after percutaneous coronary intervention	Adam, , Z, Turley, A, Mason, JM, Kasim, AS, Newby, D, Mills, N, Padfield, G, Thompson, L, Morley, R, Hall, JA, Wright, RA, Muir, DF, Sutton, AG, Swanson, N, Carter, J, Bilous, R, Jones, S; de Belder, MA	2016
Preexisting levels of CD4 T cells expressing PD-1 are related to overall survival in prostate cancer patients treated with ipilimumab	Kwek, , SS, Lewis, J, Zhang, L, Weinberg, V, Greaney, SK, Harzstark, AL, Lin, AM, Ryan, CJ, Small, EJ; Fong, L	2015
Photobiomodulation therapy (PBMT) on acute pain and inflammation in patients who underwent total hip arthroplasty—a randomized, triple-blind, placebo-controlled clinical trial	Langella, , LG, Casalechi, HL, Tomazoni, SS, Johnson, DS, Albertini, R, Pallotta, RC, Marcos, RL, de Carvalho, PTC; Leal-Junior, ECP	2018
Effects of gastric bypass followed by a randomized study of physical training on markers of coagulation activation, fibrin clot properties, and fibrinolysis	Stolberg, , CR, Mundbjerg, LH, Funch-Jensen, P, Gram, B, Juhl, CB; Bladbjerg, EM	2018
The anti-inflammatory effect of bradykinin preconditioning in coronary artery bypass grafting (bradykinin and preconditioning)	Wang, , X, Wei, M, Kuukasjärvi, P, Laurikka, J, Rinne, T, Moilanen, E; Tarkka, M	2009
3-day versus 5-day course of intravenous antibiotics for suspected early onset neonatal sepsis: a randomized controlled trial	Pasha, , YZ, Ahmadpour-Kacho, M, Behmadi, R; Jahangir, T	2014
Plasma Glutathione Peroxidase (GPX1) Levels and Oxidative Stress in Gallstone Patients Operated with Two Different Cholecystectomy Techniques: a Randomized Study with Special Reference to Cancer Patients	Kärkkäinen, , J, Aspinen, S, Harju, J, Juvonen, P, Pulkki, K; Eskelinen, M	2017
Inflammatory response to surgical trauma in patients with minilaparotomy cholecystectomy versus laparoscopic cholecystectomy: a randomised multicentre study	Aspinen, , S, Kinnunen, M, Harju, J, Juvonen, P, Selander, T, Holopainen, A, Kokki, H, Pulkki, K; Eskelinen, M	2016
Teriparatide and osseous regeneration in the oral cavity	Bashutski, , JD, Eber, RM, Kinney, JS, Benavides, E, Maitra, S, Braun, TM, Giannobile, WV; McCauley, LK	2010

Relationship between periodontitis and rheumatoid arthritis and the effect of non-surgical periodontal treatment	Pinho Mde, , N, Oliveira, RD, Novaes, AB; Voltarelli, JC	2009
Does preoperative oral carbohydrate treatment reduce the postoperative surgical stress response in lumbar disc surgery?	Dilmen, , OK, Yentur, E, Tunali, Y, Balci, H; Bahar, M	2017
Early nasogastric feeding in predicted severe acute pancreatitis: a clinical, randomized study	Eckerwall, , GE, Axelsson, JB; Andersson, RG	2006
A prospective randomized study of the inflammatory responses to multiport and singleport laparoscopic hysterectomies	Tormena, , RA, Ribeiro, SC, Soares, JM, Maciel, GAR; Baracat, EC	2017
Efficacy of long-term remote ischaemic conditioning on vascular and neuronal function in type 2 diabetes patients with peripheral arterial disease	Hansen, , C, Jorgense, ME, Fleischer, J, Botker, H; Rossing, P	2018
Efficacy of Early Enteral Immunonutrition on Immune Function and Clinical Outcome for Postoperative Patients With Gastrointestinal Cancer	Luo, , Z, Wang, J, Zhang, Z, Li, H, Huang, L, Qiao, Y, Wang, D, Huang, J, Guo, L, Liu, J, Liu, Y; Zhang, Y	2017
Compliance with enhanced recovery after surgery criteria and preoperative and postoperative counselling reduces length of hospital stay in colorectal surgery: results of a randomized controlled trial	Forsmo, , HM, Pfeffer, F, Rasdal, A, Østgaard, G, Mohn, AC, Körner, H; Erichsen, C	2016
Sivelestat attenuates lung injury in surgery for congenital heart disease with pulmonary hypertension	Nomura, , N, Asano, M, Saito, T, Nakayama, T; Mishima, A	2013
Perfusion Pressure Cerebral Infarct (PPCI) trial - the importance of mean arterial pressure during cardiopulmonary bypass to prevent cerebral complications after cardiac surgery: study protocol for a randomised controlled trial	Vedel, , AG, Holmgaard, F, Rasmussen, LS, Paulson, OB, Thomsen, C, Danielsen, ER, Langkilde, A, Goetze, JP, Lange, T, Ravn, HB; Nilsson, JC	2016
The effect of periodontal therapy on C-reactive protein, endothelial function, lipids and proinflammatory biomarkers in patients with stable coronary artery disease: study protocol for a randomized controlled trial	Saffi, , MA, Furtado, MV, Montenegro, MM, Ribeiro, IW, Kampits, C, Rabelo-Silva, ER, Polanczyk, CA, Rösing, CK; Haas, AN	2013
Second-versus first-generation "limus"-eluting stents in diabetic patients with coronary artery disease: a randomized comparison in setting of ISAR-TEST-4 trial	Kufner, , S, Byrne, RA, Mehilli, J, Massberg, S, Birkmeier, KA, Schulz, S, Pache, J, Schomig, A; Kastrati, A	2013
Periodontal tissue regeneration using fibroblast growth factor-2: randomized controlled phase II clinical trial	Kitamura, , M, Nakashima, K, Kowashi, Y, Fujii, T, Shimauchi, H, Sasano, T, Furuuchi, T, Fukuda, M, Noguchi, T, Shibutani, T, Iwayama, Y, Takashiba, S, Kurihara, H, Ninomiya, M, Kido, J, Nagata, T, Hamachi, T, Maeda, K, Hara, Y, Izumi, Y, Hirofuji, T, Imai, E, Omae, M, Watanuki, M; Murakami, S	2008
Reducing the post-pump syndrome by using heparin-coated circuits, steroids, or aprotinin	Harig, , F, Feyrer, R, Mahmoud, FO, Blum, U; von der Emde, J	1999
Beneficial effects of clopidogrel combined with aspirin in reducing cerebral emboli in patients undergoing carotid endarterectomy	Payne, , DA, Jones, CI, Hayes, PD, Thompson, MM, London, NJ, Bell, PR, Goodall, AH; Naylor, AR	2004
Ultrasonographic Changes at 12 Weeks of Anti-TNF Drugs Predict 1-year Sonographic Response and Clinical Outcome in Crohn's Disease: a Multicenter Study	Ripolles, , T, Paredes, JM, Martinez-Perez, MJ, Rimola, J, Jauregui-Amezaga, A, Bouzas, R, Martin, G; Moreno-Osset, E	2016
Aggressive clinical approach to obesity improves metabolic and clinical outcomes and can prevent bariatric surgery: a single center experience	Cadegiani, , FA, Diniz, GC; Alves, G	2017
Non-surgical periodontal therapy reduces coronary heart disease risk markers: a randomized controlled trial	Bokhari, , SA, Khan, AA, Butt, AK, Azhar, M, Hanif, M, Izhar, M; Tatakis, DN	2012
Bone resorption following weight loss surgery is associated with treatment procedure and changes in secreted Wnt antagonists	Hofsø, , D, Bollerslev, J, Sandbu, R, Jørgensen, A, Godang, K, Hjelmesæth, J; Ueland, T	2016
GnRH analogues, transvaginal ultrasound-guided drainage and intracystic injection of recombinant interleukin-2 in the treatment of endometriosis	Acién, , P, Quereda, FJ, Gómez-Torres, MJ, Bermejo, R; Gutierrez, M	2003
Routine drainage of the subhepatic area after laparoscopic cholecystectomy. Prospective, controlled study with random patient selection	Mrozowicz, , A, Rucinski, P; Polkowski, WP	2006
Adaptive servo-ventilation suppresses elevation of C-reactive protein and sympathetic activity in acute uncomplicated type B aortic dissection	Hiraoka, , A, Suzuki, K, Chikazawa, G, Nogami, S, Sakaguchi, T; Yoshitaka, H	2017
Cardiovascular events and biomarkers in a randomized trial comparing LHRH agonist and antagonist among patients with advanced prostate cancer	Margel, , D, Pe'er, A, Ber, Y, Shaparberg, M, Sela, S, Ozalvo, R, Tabachnik, T, Duivenvoorden, W, Pinthus, J; Baniel, J	2018
Congenital hyperinsulinism in children with paternal 11p uniparental isodisomy and Beckwith- Wiedemann syndrome	Kalish, , JM, Boodhansingh, KE, Bhatti, TR, Ganguly, A, Conlin, LK, Becker, SA, Givler, S, Mighion, L, Palladino, AA, Adzick, NS, De Leon, DD, Stanley, CA; Deardorff, MA	2016
Contrasted outcomes to gefitinib on tumoral IGF1R expression in head and neck cancer patients receiving postoperative chemoradiation (GORTEC trial 2004-02)	Thariat, , J, Bensadoun, RJ, Etienne-Grimaldi, MC, Grall, D, Penault-Llorca, F, Dassonville, O, Bertucci, F, Cayre, A, De Raucourt, D, Geoffrois, L, Finetti, P, Giraud, P, Racadot, S, Morinière, S, Sudaka, A, Van Obberghen-Schilling, E; Milano, G	2012
Management of endometriosis-associated infertility	Surrey, , ES; Schoolcraft, WB	2003
Effect of lamivudine against HBV reinfection after liver transplantation	Lu, , S-C, Yan, L-N, Li, B, Wen, T-F, Zhao, J-C, Cheng, N-S, Liu, C, Liu, J, Wang, X-B, Li, X-D, Qin, S, Zhao, L-S, Lei, B-J; Zhang, X-H	2003
The role of leukocyte depleting filters in heart transplantation: early outcomes in prospective, randomized clinical trial	Dvorak, , L, Pirk, J, Cerny, S; Kovar, J	2006
RETRACTED: how does high-concentration supplemental perioperative oxygen influence surgical outcomes after thyroid surgery? A prospective, randomized, double-blind, controlled, monocentric trial	Schietroma, , M, Piccione, F, Cecilia, EM, Carlei, F, De Santis, G, Sista, F; Amicucci, G	2015
Targeting nonhealing ulcers of lower extremity in human through autologous bone marrow-derived mesenchymal stem cells	Dash, , NR, Dash, SN, Routray, P, Mohapatra, S; Mohapatra, PC	2009

Ischemic brain lesions after carotid artery stenting increase future cerebrovascular risk	Gensicke, , H, Van Der Worp, HB, Nederkoorn, PJ, Macdonald, S, Gaines, PA, Van Der Lugt, A, Mali, WPTM, Lyrer, PA, Peters, N, Featherstone, RL, De Borst, GJ, Engelter, ST, Brown, MM; Bonati, LH	2015
Pre-emptive treatment of fungal infection based on plasma β-D-glucan levels after gastric surgery for gastric cancer in elderly patients	Namikawa, , T, Kitagawa, H, Yamatsuji, T, Naomoto, Y, Kobayashi, M; Hanazaki, K	2013
Preoperative chemotherapy is safe in early breast cancer, even after 10 years of follow-up; clinical and translational results from the EORTC trial 10902	van Nes, , JG, Putter, H, Julien, JP, Tubiana-Hulin, M, van de Vijver, M, Bogaerts, J, de Vos, M; van de Velde, CJ	2009
Muscadine Grape Seed Supplementation and Vascular Function	NCT01011517	2009
Non-surgical periodontal therapy reduces coronary heart disease risk markers: a randomized controlled trial	Bokhari, , SA, Khan, AA, Butt, AK, Azhar, M, Hanif, M, Izhar, M; Tatakis, DN	2013
Role of serum interleukin-6 in comparing surgical stress after laparoscopic-assisted vaginal hysterectomy and non-descent vaginal hysterectomy for large uteri	Roy, , KK, Subbaiah, M, Singla, S, Kumar, S, Sharma, JB; Mitra, DK	2012
The effect of ulinastatin on hyperglycemia in patients undergoing hepatectomy	Zhao, , G, Zhu, Y, Yu, D; Ma, J	2015
Propolis Improves Glycemic Control in Subjects With Type 2 Diabetes and Chronic Periodontitis	NCT02794506	2016
3-D implantable marker provides benefits for radiation targeting and cosmesis	Kaufman, , C, Cross, M, Dekhne, N, Devisetty, K, Edmonson, D, Gass, J, Graham, C, Gold, L, Goyal, S, Hall, W, Hong, R, Jones, S, Kuske, R, Pandya, S, Phillips, R, Schonholz, S, Smith, L; Tafra, L	2017
Rosuvastatin for Reduction of Myocardial Damage during Coronary Angioplasty - the Remedy Trial	Briguori, , C, Madonna, R, Zimarino, M, Calabrò, P, Quintavalle, C, Salomone, M, Condorelli, G; De Caterina, R	2016
Clinical response to infliximab after secondary failure with adalimumab or certolizumab pegol in crohn's disease	Trivella, , J, Yarur, A, Moroney, J, Deshpande, A, Abreu, M; Sussman, D	2013
Chronic lower extremity ischemia: a human model of ischemic tolerance	Badhwar, , A, Forbes, TL, Lovell, MB, Dungey, AA, McCarter, SD, Scott, JR, DeRose, G, Harris, KA; Potter, RF	2004
Tripterygium wilfordii Hook. f. versus azathioprine for prevention of postoperative recurrence in patients with Crohn's disease: a randomized clinical trial	Zhu, , W, Li, Y, Gong, J, Zuo, L, Zhang, W, Cao, L, Gu, L, Guo, Z, Li, N; Li, J	2015
Prednisone Versus Doxycycline in the Treatment of Graves' Orbitopathy	NCT01809444	2012
Effects of preoperative short term use of atorvastatin on endothelial progenitor cells after coronary surgery: a randomized, controlled trial	Baran, , Ç, Durdu, S, Dalva, K, Zaim, Ç, Dogan, A, Ocakoglu, G, Gürman, G, Arslan, Ö; Akar, AR	2012
LongoVital in the treatment of Sjögren's syndrome	Pedersen, , A, Gerner, N, Palmvang, I; Høier-Madsen, M	1999
Bipolar versus monopolar transurethral resection of the prostate: a prospective randomized trial focusing on bleeding complications	Stucki, , P, Marini, L, Mattei, A, Xafis, K, Boldini, M; Danuser, H	2015
Evaluation of clinical effectiveness of MIST ultrasound therapy for the healing of chronic wounds	Ennis, , WJ, Valdes, W, Gainer, M; Meneses, P	2006
Greater improvement in LRRK2 G2019S patients undergoing Subthalamic Nucleus Deep Brain Stimulation compared to non-mutation carriers	Sayad, , M, Zouambia, M, Chaouch, M, Ferrat, F, Nebbal, M, Bendini, M, Lesage, S, Brice, A, Brahim Errahmani, M; Asselah, B	2016
Perioperative use of a beta blocker in coronary artery bypass grafting	Ogawa, , S, Okawa, Y, Goto, Y, Aoki, M; Baba, H	2013
Effect of low molecular weight heparin (Certoparin) versus unfractionated heparin on cancer survival following breast and pelvic cancer surgery: a prospective randomized double-blind trial	von Tempelhoff, , GF, Harenberg, J, Niemann, F, Hommel, G, Kirkpatrick, CJ; Heilmann, L	2000
Effects of anti-CD4 antibody treatment on lymphocyte subsets and stimulated tumor necrosis factor alpha production: a study of 29 multiple sclerosis patients entered into a clinical trial of cM-T412	Llewellyn-Smith, , N, Lai, M, Miller, DH, Rudge, P, Thompson, AJ; Cuzner, ML	1997
Does dexmedetomidine have a cardiac protective effect during non-cardiac surgery? A randomised controlled trial	Xu, , L, Hu, Z, Shen, J; McQuillan, PM	2014
Clinical effects of the heparin coated surface in cardiopulmonary bypass	Svenmarker, , S, Sandström, E, Karlsson, T, Jansson, E, Häggmark, S, Lindholm, R, Appelblad, M; Aberg, T	1997
Effects of remote ischemic preconditioning in high-risk patients undergoing cardiac surgery (Remote IMPACT): a randomized controlled trial	Walsh, , M, Whitlock, R, Garg, AX, Légaré, JF, Duncan, AE, Zimmerman, R, Miller, S, Fremes, S, Kieser, T, Karthikeyan, G, Chan, M, Ho, A, Nasr, V, Vincent, J, Ali, I, Lavi, R, Sessler, DI, Kramer, R, Gardner, J, Syed, S, VanHelder, T, Guyatt, G, Rao-Melacini, P, Thabane, L; Devereaux, PJ	2016
Probiotics administration following sleeve gastrectomy surgery: a randomized double-blind trial	Sherf-Dagan, , S, Zelber-Sagi, S, Zilberman-Schapira, G, Webb, M, Buch, A, Keidar, A, Raziel, A, Sakran, N, Goitein, D, Goldenberg, N, Mahdi, JA, Pevsner-Fischer, M, Zmora, N, Dori-Bachash, M, Segal, E, Elinav, E; Shibolet, O	2018
SWOG S1505: a randomized phase II study of perioperative mFOLFIRINOX versus gemcitabine/nab-paclitaxel as therapy for resectable pancreatic adenocarcinoma	Sohal, , D, McDonough, SL, Ahmad, SA, Gandhi, N, Beg, MS, Wang-Gillam, A, Guthrie, KA, Lowy, AM, Philip, PA; Hochster, HS	2018
Hilar en-bloc resection for perihilar cholangiocarcinoma (PHCC); a single-center experience	Bednarsch, , J, Czigany, Z, Schlebusch, I, Schoning, W, Ulmer, T, Amygdalos, I, Santana, DM, Meister, F, Bocker, J, Neumann, U; Lurje, G	2018
Impact of vitamin C on endothelial function and exercise capacity in patients with a Fontan circulation	Goldstein, , BH, Sandelin, AM, Golbus, JR, Warnke, N, Gooding, L, King, KK, Donohue, JE, Yu, S, Gurney, JG, Goldberg, CS, Rocchini, AP; Charpie, JR	2012
Early ankle mobilization promotes healing in a rabbit model of achilles tendon rupture	Jielile, , J, Asilehan, B, Wupuer, A, Qianman, B, Jialihasi, A, Tangkejie, W, Maimaitiaili, A, Shawutali, N, Badelhan, A,	2016

	Niyazebieke, H, Aizezi, A, AisaiDing, A, Bakyt, Y, Aibek, R; Wuerliebieke, J	
Preop CPET Testing and Exercise Training in Colorectal Patients	NCT01924897	2013
Effect of preoperative abstinence on poor postoperative outcome in alcohol misusers: randomised controlled trial	Tonnesen, , H, Rosenberg, J, Nielsen, HJ, Rasmussen, V, Hauge, C, Pedersen, IK; Kehlet, H	1999
Treatment of scleroderma	Sapadin, , AN; Fleischmajer, R	2002
Does Rectus Sheath Block Analgesia Alter Levels of the Oxidative Stress Biomarker Glutathione Peroxidase: a Randomised Trial of Patients with Cancer and Benign Disease	Purdy, , M, Kärkkäinen, J, Kokki, M, Anttila, M, Aspinen, S, Juvonen, P, Kokki, H, Pulkki, K, Rantanen, T; Eskelinen, M	2017
Prospective Randomized Trial Comparing Three-port and Single-port TEP Repair in Adults	NCT01591395	2012
The efficacy of human atrial natriuretic peptide in patients with renal dysfunction undergoing cardiac surgery	Izumi, , K, Eishi, K, Yamachika, S, Hashizume, K, Tada, S, Yamane, K, Takai, H, Tanigawa, K, Miura, T; Nakaji, S	2008
Anti-Inflammatory Effect of Surfactant Lipid in the Vaginal Mucosa: a Pilot Study	MacNeill, , C, de Guzman, GS, Lupo, K, Umstead, T, Phelps, DS; Floros, J	2019
Hypotheses, rationale, design, and methods for prognostic evaluation of cardiac biomarker elevation after percutaneous and surgical revascularization in the absence of manifest myocardial infarction. A comparative analysis of biomarkers and cardiac magnetic resonance. The MASS-V Trial	Hueb, , W, Gersh, BJ, Rezende, PC, Garzillo, CL, Lima, EG, Vieira, RD, Garcia, RMR, Favarato, D, Segre, CAW, Pereira, AC, Soares, PR, Ribeiro, E, Lemos, P, Perin, MA, Strunz, CC, Dallan, LAO, Jatene, FB, Stolf, NAG, Hueb, AC, Dias, R, Gaiotto, FA, da Costa, LMA, Oikawa, FTC, de Melo, RMV, Serrano Junior, CV, de Avila, LFR, Villa, AV, Filho, JRP, Nomura, C, Ramires, JAF; Filho, RK	2012
Protective effect of eicosapentaenoic acid on insulin resistance in hyperlipidemic patients and on the postoperative course of cardiac surgery patients: the possible involvement of adiponectin	Yamamoto, , T, Kajikawa, Y, Otani, S, Yamada, Y, Takemoto, S, Hirota, M, Ikeda, M, Iwagaki, H, Saito, S; Fujiwara, T	2014
Use of ivabradine and atorvastatin in emergent orthopedic lower limb surgery and computed tomography coronary plaque imaging and novel biomarkers of cardiovascular stress and lipid metabolism for the study and prevention of perioperative myocardial infarction: study protocol for a randomized controlled trial	Rudd, , N, Subiakto, I, Asrar Ul Haq, M, Mutha, V; Van Gaal, WJ	2014
The effect of oxcarbazepine in peripheral neuropathic pain depends on pain phenotype: a randomised, double-blind, placebo-controlled phenotype-stratified study	Demant, , DT, Lund, K, Vollert, J, Maier, C, Segerdahl, M, Finnerup, NB, Jensen, TS; Sindrup, SH	2014
A 2-year follow-up results of knee arthroscopic synovectomy plus disease modifying antirheumatic drugs for the treatment of rheumatoid arthritis	Chen, , GQ, Zhang, HW, Li, ZF, Yu, YT; Guo, DM	2011
Laryngeal cancer and angiogenesis	Beatrice, , F, Valente, G, Cammarota, R, Bisciari, T, Ragona, R, Giusti, U, Bussolino, F; Sartoris, A	1996
Amoxicillin plus clavulanic acid versus appendicectomy for treatment of acute uncomplicated appendicitis: an open-label, non-inferiority, randomised controlled trial	Vons, , C, Barry, C, Maitre, S, Pautrat, K, Leconte, M, Costaglioli, B, Karoui, M, Alves, A, Dousset, B, Valleur, P, Falissard, B; Franco, D	2011
Randomised, double blind, placebo controlled trial of intravenous antioxidant (n-acetylcysteine, selenium, vitamin C) therapy in severe acute pancreatitis	Siriwardena, , AK, Mason, JM, Balachandra, S, Bagul, A, Galloway, S, Formela, L, Hardman, JG; Jamdar, S	2007
Bolus high dose interleukin-2 for the treatment of malignant melanoma	Pappo, , I, Lotem, M, Klein, M; Orda, R	2001
Effects of Atorvastatin (80 mg) Therapy on Quantity of Epicardial Adipose Tissue in Patients Undergoing Pulmonary Vein Isolation for Atrial Fibrillation	Soucek, , F, Covassin, N, Singh, P, Ruzek, L, Kara, T, Suleiman, M, Lerman, A, Koestler, C, Friedman, PA, Lopez-Jimenez, F; Somers, VK	2015
The role of coagulation and inflammation after angioplasty in patients with peripheral arterial disease	Wahlgren, , CM, Sten-Linder, M, Egberg, N, Kalin, B, Blohmé, L; Swedenborg, J	2006
Efficacy And Safety Of Smoking Cessation With Varenicline Tartrate In Diabetic Smokers: (DIASMOKE)	NCT01387425	2011
Early MRI in Acute GallstonE Disease	NCT03709030	2018
A comparison among infusion of lidocaine and dexmedetomidine alone and in combination in subjects undergoing coronary artery bypass graft: a randomized trial	Kim, , HJ, Kim, WH, Kim, G, Kim, E, Park, MH, Shin, BS, Sim, WS, Kim, CS, Lee, YT; Cho, HS	2014
Diagnosis of cardiac surgery-associated acute kidney injury: differential roles of creatinine, chitinase 3-like protein 1 and neutrophil gelatinase-associated lipocalin: a prospective cohort study	De Loor, , J, Herck, I, Francois, K, Van Wesemael, A, Nuytinck, L, Meyer, E; Hoste, EAJ	2017
Transarterial embolisation	Okuno, Y	2017
The Claudication: exercise Vs. Endoluminal Revascularization (CLEVER) study: rationale and methods	Murphy, , TP, Hirsch, AT, Ricotta, JJ, Cutlip, DE, Mohler, E, Regensteiner, JG, Comerota, AJ; Cohen, DJ	2008
Lack of nephrotoxicity by 6% hydroxyethyl starch 130/0.4 during hip arthroplasty: a randomized controlled trial	Kancir, , AS, Pleckaitiene, L, Hansen, TB, Ekeløf, NP; Pedersen, EB	2014
Withdrawal of clopidogrel is not associated with rebound platelet activity in stable cardiovascular and vascular patients	Ford, , I, Scott, NW, Herd, V, Mitchell, L, Williams, DJ; Brittenden, J	2013
CYP2D6 metabolism and patient outcome in the Austrian Breast and Colorectal Cancer Study Group trial (ABCSG) 8	Goetz, , MP, Suman, VJ, Hoskin, TL, Gnant, M, Filipits, M, Safgren, SL, Kuffel, M, Jakesz, R, Rudas, M, Greil, R, Dietze, O, Lang, A, Offner, F, Reynolds, CA, Weinshilboum, RM, Ames, MM; Ingle, JN	2013
Comparison of high reloading ROsuvastatin and Atorvastatin pretreatment in patients undergoing elective PCI to reduce the incidence of MyocArdial periprocedural necrosis. The ROMA II trial	Sardella, , G, Lucisano, L, Mancone, M, Conti, G, Calcagno, S, Stio, RE, Pennacchi, M, Biondi-Zoccai, G, Canali, E; Fedele, F	2013

Preoperative biliary drainage by plastic or self-expandable metal stents in patients with periampullary tumors: results of a randomized clinical study	Olsson, , G, Frozanpor, F, Lundell, L, Enochsson, L, Ansorge, C, Del Chiaro, M, Reuterwall-Hansson, M, Shetye, A; Arnelo, U	2017
Duloxetine for Treatment of Painful Temporomandibular Joint Disorder	NCT00981149	2009
A randomized phase II study of Xilonix, a targeted therapy against interleukin 1α, for the prevention of superficial femoral artery restenosis after percutaneous revascularization	El Sayed, , H, Kerensky, R, Stecher, M, Mohanty, P; Davies, M	2016
Outcome of colectomy for combined colonic inertia and pelvic floor dysfunction	Fan, , C-W, Wang, J-Y, Chang-Chien, C-R, Chen, J-S, Hsu, K-C, Tang, R-P, Chiang, J-M, Yeh, C-Y; Hsieh, P-S	2001
Surgical treatment of haemorrhoids according to Longo and Milligan Morgan: an evaluation of postoperative tissue response	Krska, , Z, Kvasnièka, J, Faltýn, J, Schmidt, D, Sváb, J, Kormanová, K; Hubík, J	2003
Serum acute phase protein concentrations after hysterectomy with and without low-molecular-weight heparin thrombosis prophylaxis	Maciejczyk-Pencuła, , M, Polak, G; Kotarski, J	2005
The Kidney and Periodontal Disease (KAPD) study: a pilot randomized controlled trial testing the effect of non-surgical periodontal therapy on chronic kidney disease	Grubbs, , V, Garcia, F, Jue, BL, Vittinghoff, E, Ryder, M, Lovett, D, Carrillo, J, Offenbacher, S, Ganz, P, Bibbins-Domingo, K; Powe, NR	2017
Clinical and microbiological efficacy of moxifloxacin versus amoxicillin/clavulanic acid in severe odontogenic abscesses: a pilot study	Al-Nawas, , B, Walter, C, Morbach, T, Seitner, N, Siegel, E, Maeurer, M; Krummenauer, F	2009
Laparoendoscopic single-site versus conventional laparoscopic total extraperitoneal hernia repair: a prospective randomized clinical trial	Tsai, , YC, Ho, CH, Tai, HC, Chung, SD; Chueh, SC	2013
Crohn's disease activity index does not correlate with endoscopic recurrence one year after ileocolonic resection	Regueiro, , M, Kip, KE, Schraut, W, Baidoo, L, Sepulveda, AR, Pesci, M, El-Hachem, S, Harrison, J; Binion, D	2011
Compare the efficacy of inhaled budesonide and systemic methylprednisolone on systemic inflammation of AECOPD	Sun, , X, He, Z, Zhang, J, Deng, J, Bai, J, Li, M; Zhong, X	2015
Effects of atorvastatin on endothelial function and the expression of proinflammatory cytokines and adhesion molecules in young subjects with successfully repaired coarctation of aorta	Brili, , S, Tousoulis, D, Antonopoulos, AS, Antoniades, C, Hatzis, G, Bakogiannis, C, Papageorgiou, N; Stefanadis, C	2012
Preconditioning with sevoflurane decreases PECAM-1 expression and improves one-year cardiovascular outcome in coronary artery bypass graft surgery	Garcia, , C, Julier, K, Bestmann, L, Zollinger, A, von Segesser, LK, Pasch, T, Spahn, DR; Zaugg, M	2005
SWOG S1505: a randomized phase II study of perioperative mFOLFIRINOX vs. gemcitabine/nab-paclitaxel as therapy for resectable pancreatic adenocarcinom	Sohal, , D, McDonough, SL, Ahmad, SA, Gandhi, N, Beg, MS, Wang-Gillam, A, Guthrie, KA, Lowy, AM, Philip, PA; Hochster, HS	2017
Anti-infective periodontal therapy promoting improvement in systemic markers of HIV infection	Noro Filho, , GA, Salgado, DM, Casarin, RC, Casati, MZ, Costa, C; Giovani, EM	2013
Cell saver for on-pump coronary operations reduces systemic inflammatory markers: a randomized trial	Damgaard, , S, Nielsen, CH, Andersen, LW, Bendtzen, K, Tvede, M; Steinbrüchel, DA	2010
A Double-Blinded Randomized Trial to Compare the Effectiveness of Minimally Invasive Procedures Using Patient-Reported Outcomes	Bingener, , J, Skaran, P, McConico, A, Novotny, P, Wettstein, P, Sletten, DM, Park, M, Low, P; Sloan, J	2015
Randomized controlled trial of comparison between the SuperPATH and posterolateral approaches in total hip arthroplasty	Ouyang, , C, Wang, H, Meng, W, Luo, Z, Wang, D, Pei, F; Zhou, Z	2018
A poetic story: lessons learnt from the world's largest breast cancer window of opportunity study	Bliss, , J, Gillman, A, Kilburn, L, Morden, J, Sidhu, K, Wilcox, M, Evans, A, Holcombe, C, Horgan, K, Skene, A, Vidya, R, Robertson, J, Dowsett, M; Smith, I	2015
Arthroscopy and Manipulation vs a Home Therapy Program in the Treatment of Adhesive Capsulitis	NCT00160784	2005
Tacrolimus Versus Cyclosporine for Immunosuppression After Lung Transplantation	NCT01429844	2011
18F-fluoromisonidazole PET and activity of neoadjuvant nintedanib in early HER2-negative breast cancer: a window-of-opportunity randomized trial	Quintela-Fandino, , M, Lluch, A, Manso, L, Calvo, I, Cortes, J, Garcia-Saenz, JA, Gil-Gil, M, Martinez-Janez, N, Gonzalez-Martin, A, Adrover, E, De Andres, R, Vinas, G, Llombart-Cussac, A, Alba, E, Guerra, J, Bermejo, B, Zamora, E, Moreno-Anton, F, Simon, SP, Carrato, A, Lopez-Alonso, A, Escudero, MJ, Campo, R, Carrasco, E, Palacios, J, Mulero, F; Colomer, R	2017
Stress echo 2020: the international stress echo study in ischemic and non-ischemic heart disease	Picano, , E, Ciampi, Q, Citro, R, D'Andrea, A, Scali, MC, Cortigiani, L, Olivotto, I, Mori, F, Galderisi, M, Costantino, MF, Pratali, L, Di Salvo, G, Bossone, E, Ferrara, F, Gargani, L, Rigo, F, Gaibazzi, N, Limongelli, G, Pacileo, G, Andreassi, MG, Pinamonti, B, Massa, L, Torres, MAR, Miglioranza, MH, Daros, CB, De Castro, E Silva Pretto JL, Beleslin, B, Djordjevic-Dikic, A, Varga, A, Palinkas, A, Agoston, G, Gregori, D, Trambaiolo, P, Severino, S, Arystan, A, Paterni, M, Carpeggiani, C; Colonna, P	2017
Periodontal infection as a possible severity factor for rheumatoid arthritis	Ribeiro, , J, Leão, A; Novaes, AB	2005
Management of peripheral arterial interventions with mono or dual antiplatelet therapy–the MIRROR study: a randomised and double-blinded clinical trial	Tepe, , G, Bantleon, R, Brechtel, K, Schmehl, J, Zeller, T, Claussen, CD; Strobl, FF	2012
Maintenance effect of polyglycosides of Tripterygium wilfordii on remission in postoperative Crohn disease	Tao, , QS, Ren, JA, Ji, ZL, Li, JS, Wang, XB; Jiang, XH	2009
A heparin-bonded vascular graft generates no systemic effect on markers of hemostasis activation or detectable heparin-induced thrombocytopenia-associated antibodies in humans	Heyligers, , JM, Lisman, T, Verhagen, HJ, Weeterings, C, de Groot, PG; Moll, FL	2008
Effect of Gemcitabine and nab-Paclitaxel with or Without Hydroxychloroquine on Patients with Advanced Pancreatic Cancer: a Phase 2 Randomized Clinical Trial	Karasic, , TB, O'Hara, MH, Loaiza-Bonilla, A, Reiss, KA, Teitelbaum, UR, Borazanci, E, De Jesus-Acosta, A, Redlinger, C, Burrell, JA, Laheru, DA, Von Hoff, DD, Amaravadi, RK, Drebin, JA; O'Dwyer, PJ	2019

Effects of anesthesia based on large versus small doses of fentanyl on natural killer cell cytotoxicity in the perioperative period	Beilin, , B, Shavit, Y, Hart, J, Mordashov, B, Cohn, S, Notti, I; Bessler, H	1996
Anesthetics to Prevent Lung Injury in Cardiac Surgery (APLICS): a protocol for a randomized controlled trial	O'Gara, , B, Subramaniam, B, Shaefi, S, Mueller, A, Banner-Goodspeed, V; Talmor, D	2019
Rationale and design of the Clinical and Histologic Analysis of Mesenchymal Stromal Cells in AmPutations (CHAMP) trial investigating the therapeutic mechanism of mesenchymal stromal cells in the treatment of critical limb ischemia	Wang, , SK, Green, LA, Drucker, NA, Motaganahalli, RL, Fajardo, A; Murphy, MP	2018
A PHASE IIA OPEN LABEL, ADAPTATIVE, RANDOMIZED CLINICAL TRIAL OF DALOTUZUMAB (MK-0646) TREATMENT IN COMBINATION WITH IRINOTECAN VERSUS CETUXIMAB AND IRINOTECAN FOR PARIENTS WITH METASTASIC RECTAL CANCERS (MRC) EXPRESSING HIGH IGF-1/LOW IGF-2 LEVELS	PER-075-12	2012
A randomised, double-blind, placebo-controlled, parallel group study to investigate the anti-inflammatory and metabolic effects of rosiglitazone XR, 8mg once daily, in subjects with rheumatoid arthritis. - HM2004/00255/03	EUCTR2004-000482-35-LT	2005
Randomised trial of basiliximab versus placebo for control of acute cellular rejection in renal allograft recipients. CHIB 201 International Study Group	Nashan, , B, Moore, R, Amlot, P, Schmidt, AG, Abeywickrama, K; Soulillou, JP	1997
Neoadjuvant Therapy in Advanced OVarian Cancer with Avastin	EUCTR2012-003883-31-ES	2013
Periostin and periodontal disease as modifiers of atherosclerotic coronary artery disease	ISRCTN17884414	2015
Shed-blood-separation and cell-saver: an integral Part of MiECC? Shed-blood-separation and its influence on the perioperative inflammatory response during coronary revascularization with minimal invasive extracorporeal circulation systems - a randomized controlled trial	Bauer, , A, Hausmann, H, Schaarschmidt, J, Scharpenberg, M, Troitzsch, D, Johansen, P, Nygaard, H, Eberle, T; Hasenkam, JM	2018
A clincial study comparing the effectiveness of two drugs, named Rituximab and Dimethyl Fumarate (Tecfidera®), for the neurological disease Multiple Sclerosis	EUCTR2015-004116-38-SE	2015
Effect of periodontal surgery on reduction of serum concentration of CRP	IRCT201101291606N2	2011
PROFILE - personalised medicine in Crohn's disease	ISRCTN11808228	2017
Does conversion to Nulojix reduce the risk of cardiovascular disease in kidney transplant recipients?	EUCTR2013-001178-20-NL	2015
A Study of the Efficacy and PK/PD Relationship of Monotherapy MORAb-004 in Subjects with Metastatic Melanoma	EUCTR2011-001282-40-GB	2011
Intra-articular and intravenous infliximab in the treatment of resistant seronegative oligoarthritis of the knee - Seronegative Oligoarthritis of the Knee Study	EUCTR2009-015810-23-GB	2010
GEM 3: combination methotrexate and gefitinib for the treatment of ectopic pregnancy	EUCTR2015-005013-76-GB	2016
Randomised controlled trial of soluble plantain fibre for maintenance of health during periods of remission in Crohn's disease	ISRCTN62819212	2007
A clinical study to evaluate the tolerability of repeated doses of CEP-33457 administered in patients with a chronic autoimmune disease (Lupus)	EUCTR2010-019293-32-PT	2010
A clinical trial studying Paclitaxel with or without MM-121 for patients with advanced ovarian cancer	EUCTR2011-003576-36-CZ	2011
A study of atezolizumab compared to placebo for subjects with late relapse ovarian cancer receiving a treatment with platinum-basedchemotherapy and bevacizumab	EUCTR2015-005471-24-FR	2016
Effects of ISIS 329993, a C Reactive Protein Antisense Oligonucleotide, in patients with sporadic Atrial Fibrillation	EUCTR2012-000833-38-GB	2012
Safety Evaluation of the Maintenance treatment of Sorafenib in Hepatocellular carcinoma patients with Tumor thrombus after palliative surgery	JPRN-UMIN000005910	2011
An open pilot study to assess the effects of pemirolast on C-reactive protein levels in subjects with coronary artery disease	EUCTR2009-016823-74-SE	2009
A randomised control trial of SEEG electrode placement methods	ISRCTN17209025	2016
A phase II trial with bevacizumab and irinotecan for patients with primary brain tumors and progression after standard therapy - BI-Brain-01	EUCTR2006-006011-74-DK	2006
An Investigation of the Safety of Continued Treatment with Sorafenib in Patients with Unresectable Hepatocellular Carcinoma after the First Disease Progression	JPRN-UMIN000005818	2011
Randomized phase III study evaluating hyperthermic intraperitoneal chemotherapy in the treatment of ovarian cancer relapse	EUCTR2010-023035-42-ES	2012
PHASE 3, MULTICENTER, RANDOMIZED, DOUBLE BLIND, PARALLEL GROUP, PLACEBO CONTROLLED STUDY TO EVALUATE THE EFFICACY AND SAFETY OF CERTOLIZUMAB PEGOL IN SUBJECTS WITH ADULT ONSET ACTIVE AND PROGRESSIVE PSORIATIC ARTHRITIS (PSA)	EUCTR2009-011720-59-NL	2010
Dose section Phase II study to compare the inhibition of blood loss in patients undergoing primary heart surgery and treatment with the	EUCTR2011-004666-15-DE	2011

investigational drug MDCO-2010, the active comparator tanexamic acid or placebo		
Indicators of Gum Diseases	CTRI/2018/11/016360	2018
Clinical trial to evaluate the efficacy and safety of K(D)PT in patients with mild to moderate ulcerative colitis	EUCTR2011-002462-20-SK	2011
Response to neoadjuvant chemotherapy in ICON8: a GCIG phase III randomised trial evaluating weekly dose-dense chemotherapy integration in first-line epithelial ovarian/ fallopian tube/ primary peritoneal carcinoma (EOC) treatment	Clamp, , AR, McNeish, IA, Dean, A, Gallardo-Rincon, D, Kim, J-W, O'Donnell, DM, Hook, J, Blagden, S, Brenton, JD, Naik, R, Perren, TJ, Sundar, S, Cook, AD, James, EC, Gabra, H, Lord, R, Hall, M, Dark, G, Kaplan, RS; Ledermann, JA	2018
Subgroups analysis and circulating biomarkers evaluation of RESORT trial: a randomized phase II study in metastatic renal cell carcinoma (mRCC) patients (pts) to evaluate the efficacy of sorafenib after metastasectomy	Procopio, , G, Cognetti, F, Miceli, R, Milella, M, Mosca, A, Chiuri, VE, Bearz, A, Morelli, F, Ortega, C, Atzori, F, Passalacqua, R, Ratta, R, De Braud, FGM, Cappelletti, V; Verzoni, E	2018
COMBINATION ANTI-PARATUBERCULOSIS THERAPY WITH RIFABUTIN, CLARITHROMYCIN AND CLOFAZIMINE FOR CROHNâ¿¿S DISEASE: a THREE YEAR PROSPECTIVE, RANDOMISED, PLACEBO-CONTROLLED, MULTICENTRE STUDY	ACTRN12605000429651	2005
The effect of melatonin supplementation in the treatment of type 2 diabetic patients with chronic periodontitis disorder	IRCT2017011631993N1	2017
Comparison of the effects of oral hygiene instruction and periodontal treatment on patients with Type 2 diabetes mellitus	JPRN-UMIN000013278	2014
Clinical trial to shortening the treatment for pulmonary tuberculosis with rifapentin containing regimen	EUCTR2015-003446-45-ES	2015
RANDOMIZED, OPEN LABEL, PHASE 3 CLINICAL STUDY TO EVALUATE THE EFFECT OF THE ADDITION OF CP-751,871 TO GEMCITABINE AND CISPLATIN IN PATIENTS WITH ADVANCED NON-SMALL CELL LUNG CANCER	EUCTR2008-004008-30-BE	2009
A study to investigate the safety and effectiveness of treatment with the drug Elafibranor in Patients with Non-Alcoholic Steatohepatitis (NASH) and fibrosis (a form of liver disease)	EUCTR2015-005385-38-BE	2016
Sodium valproate for epigenetic reprogramming in the management of high risk oral epithelial dysplasia	ISRCTN12448611	2018
Conventional open versus laparoscopic surgery for peritoneal dialysis (PD) peritonitis	ISRCTN55802603	2009
Relook Laparoscopy for Peritoneal Surface Disease (ReLaPSeD) Trial A prospective randomised controlled trial comparing relook laparoscopy vs. standard follow-up for early detection and treatment of patients at high risk for peritoneal metastases after resection of colorectal cancer	ACTRN12618000372280	2018
Interactions between diet and gut microbes in preterm infants	ISRCTN16799022	2017
A clinical study comparing Acelarin with gemcitabine in patient with metastatic pancreatic carcinoma	ISRCTN16765355	2015
A 24-WEEK, DOUBLE-BLIND, RANDOMIZED, PARALLEL GROUP, PLACEBO-CONTROLLED, PHASE 2 STUDY OF DIFFERENT DOSES OF VX509 IN ADULT SUBJECTS WITH ACTIVE RHEUMATOID ARTHRITIS ON STABLE METHOTREXATE THERAPY WITH 104-WEEK OPEN LABEL EXTENSION	PER-076-12	2012
A phase 2 Study of the efficay and safty of erlotinib therapy for 3/4 stage EGFR gene mutation positive EGFR-TKI untreated non-small cell lung cancer	JPRN-UMIN000011986	2013
Health of gums effecting health of heart in heart disease	CTRI/2018/02/011970	2018
PGL5001 Proof of Concept study in inflammatory endometriosis	EUCTR2012-001219-22-PL	2012
Ward based goal-directed fluid therapy (GDFT) in acute pancreatitis	ISRCTN36077283	2018
Phase II clinical trial, conducted in different sites, with random assignation of treatment, where neither the patient or the medical doctor know the assigned treatment, drug or placebo, to evaluate the efficacy and safety of Rifaximin delayed release 400 mg tablet in the prevention of post-operative endoscopic Crohn’s disease recurrence	EUCTR2017-002258-36-DE	2017
High dose vitamin D treatment in Crohn's disease affects the gut immune cells	EUCTR2013-000971-34-DK	2013
A study to evaluate the efficacy and safety of a new anticoagulation drug REG1 compared with bivalirudin in patients undergoing angioplasty	EUCTR2013-001384-23-AT	2013
Treatment of focal nerve pain with lidocaine patch	EUCTR2012-003077-26-DK	2012
ROLO The Renoprotective value of Leukodepletion in Heart Valve surgery	ISRCTN42121335	2014
The antioxidant effects of Q10 co enzyme on oxidative stress and lipid per oxidation among gastric patients infected by h.pylori	IRCT2014070118310N1	2014
A double-blind, randomized, vehicle-controlled Proof of Concept (PoC) study to evaluate the efficacy, safety, local tolerability, pharmacokinetics and pharmacodynamics of multiple topical administrations of LDE225 (a specific Smoothened inhibitor) on sporadic superficial skin basal cell carcinomas	EUCTR2009-013665-26-AT	2009

A 4 week double-blind, placebo-controlled, randomized, parallel group phase IIa study to assess the efficacy and safety of AZD1981 in patients with moderate to severe Chronic Obstructive Pulmonary Disease (COPD)	EUCTR2008-000340-13-SE	2008
Imatinib therapy for agressive colon cancers	EUCTR2014-003965-11-NL	2015
A window of opportunity study investigating biological effects of metformin in localised prostate cancer	EUCTR2014-005193-11-GB	2015
A Randomized, Double-Blind, Multicenter, Phase 2 Trial Comparing Veliparib Plus Carboplatin and Paclitaxel Versus Placebo Plus Carboplatin and Paclitaxel in Previously Untreated Metastatic or Advanced Non-Small-Cell Lung Cancer (NSCLC)	EUCTR2011-003427-36-HU	2012
EFFECTS OF NEBIVOLOL ON THE WALKING ABILITY IN PATIENTS WITH ESSENTIAL HYPERTENSION AND PERIPHERAL ARTERIAL DISEASE INTERMITTENT CLAUDICATION	EUCTR2004-004863-32-DE	2005
MULTICENTRIC, RANDOMISED, DOUBLE BLIND, PLACEBO CONTROLLED CLINICAL TRIAL TO EVALUATE THE EFFICACY AND THE SAFETY OF THE PROPHYLAXIS WITH BEMIPARIN 3,500 IU/day FOR 28 DAYS COMPARED TO 8 DAYS, IN VENOUS THROMBOEMBOLIC DISEASE IN PATIENTS UNDERGOING ONCOLOGICAL ABDOMINAL OR PELVIC SURGERY [CANBESURE STUDY: cancer, Bemiparin and Surgery Evaluation] - CANBESURE	EUCTR2005-004373-23-PT	2007
EXPERIMENTAL STUDY IN HUMANS TO EVALUATE THE EFFICACY OF A PRE-SURGERY CHEMOTHERAPY SCHEME CUSTOMIZED BY LEVELS OF TUMORAL BIOMARKERS IN CERTAIN TYPES OF EARLY BREAST CANCER	EUCTR2011-005843-28-ES	2013
The effect study of treament of triple negative breast cancer by integrated traditional and western medicine	ChiCTR-IPR-16008590	2016
A clinical trial to evaluate the influence of electrical stimulation on sensory and motoric deficits in patients with lumbar disc herniation	ISRCTN12741173	2017
A randomized controlled trail on the efficacy and safety of autologous cytokine-induced killer (CIK) cells infusion treatment in advanced primary lung cancer	ChiCTR-TRC-11001558	2011
Effect of periodontal non-surgical treatment for pre-diabetes/type 2 diabetes patients	ISRCTN25474337	2015
PROVENT: a randomised, placebo controlled feasibility study to examine the effectiveness of aspirin and/or Vitamin D to prevent disease progression in men undergoing monitoring for prostate cancer	EUCTR2014-001784-13-GB	2015
Continued effectiveness of a biosimilar adalimumab after stoppage of initial treatment in patients with ankylosing spondylitis	Bandyopadhyay, , S, Ray, A, Sarkar, RN, Dash, S; Mondal, S	2017
Effects of off-pump versus on-pump coronary artery bypass grafting: apoptosis, inflammation, and oxidative stress	Bicer, , M, Senturk, T, Yanar, M, Tutuncu, A, Oral, AY, Ulukaya, E, Serdar, Z; Signak, IS	2014
Improved memory function 12 weeks after bariatric surgery	Gunstad, , J, Strain, G, Devlin, MJ, Wing, R, Cohen, RA, Paul, RH, Crosby, RD; Mitchell, JE	2011
The efficacy of cardiac shock wave therapy in the treatment of refractory angina: a pilot prospective, randomized, double-blind trial	Leibowitz, , D, Weiss, AT, Rott, D, Durst, R; Lotan, C	2013
Prognostic significance of T-cell phenotype in aggressive non-Hodgkin's lymphomas	Gisselbrecht, , C, Gaulard, P, Lepage, E, Coiffier, B, Briere, J, Haioun, C, Cazals-Hatem, D, Bosly, A, Xerri, L, Tilly, H, Berger, F, Bouhabdallah, R; Diebold, J	1998
Randomized, controlled trial of TNF-alpha antagonist in CTL-mediated severe cutaneous adverse reactions	Wang, , C-W, Yang, L-Y, Chen, C-B, Ho, H-C, Hung, S-I, Yang, C-H, Chang, C-J, Su, S-C, Hui, RC-Y, Chin, S-W, Huang, L-F, Lin, YY-W, Chang, W-Y, Fan, W-L, Yang, C-Y, Ho, J-C, Chang, Y-C, Lu, C-W, Chung, W-H; Lai, C-H	2018
On-line hemodiafiltration does not induce inflammatory response in end-stage renal disease patients: results from a multicenter cross-over study	Vaslaki, , LR, Berta, K, Major, L, Weber, V, Weber, C, Wojke, R, Passlick-Deetjen, J; Falkenhagen, D	2005
Long-term moderate calorie restriction inhibits inflammation without impairing cell-mediated immunity: a randomized controlled trial in non-obese humans	Meydani, , SN, Das, SK, Pieper, CF, Lewis, MR, Klein, S, Dixit, VD, Gupta, AK, Villareal, DT, Bhapkar, M, Huang, M, Fuss, PJ, Roberts, SB, Holloszy, JO; Fontana, L	2016
Preconditioning by sevoflurane decreases biochemical markers for myocardial and renal dysfunction in coronary artery bypass graft surgery: a double-blinded, placebo-controlled, multicenter study	Julier, , K, Da Silva, R, Garcia, C, Bestmann, L, Frascarolo, P, Zollinger, A, Chassot, P-G, Schmid, ER, Turina, MI, Von Segesser, LK, Pasch, T, Spahn, DR; Zaugg, M	2003
Cytokine profiles in 1-yr-old very low-birth-weight infants after enteral glutamine supplementation in the neonatal period	van Zwol, , A, van den Berg, A, Nieuwenhuis, EE, Twisk, JW, Fetter, WP; van Elburg, RM	2009
Immunological consequences of thalidomide treatment in Sjögren's syndrome	Moutsopoulos, , NM, Angelov, N, Sankar, V, Leakan, RA, Pillemer, S; Wahl, SM	2006
Rational control on postoperative blood glucose levels in infants with congenital heart disease	Liu, , M, Zheng, P, Zheng, XY; Meng, XK	2012
Efficacy and safety of sirukumab in patients with active rheumatoid arthritis refractory to anti-TNF therapy (SIRROUND-T): a randomised, double-blind, placebo-controlled, parallel-group, multinational, phase 3 study	Aletaha, , D, Bingham, CO, Tanaka, Y, Agarwal, P, Kurrasch, R, Tak, PP; Popik, S	2017
Effects of the Chinese herb formula Yufeining on stable chronic obstructive pulmonary disease: a randomized, double-blind, placebo-controlled trial	Hong, , M, Hong, C, Chen, H, Ke, G, Huang, J, Huang, X, Liu, Y, Li, F; Li, C	2018

The effect of probiotics on serum levels of cytokine and endotoxin in peritoneal dialysis patients: a randomised, double-blind, placebo-controlled trial	Wang, , IK, Wu, YY, Yang, YF, Ting, IW, Lin, CC, Yen, TH, Chen, JH, Wang, CH, Huang, CC; Lin, HC	2015
Translational research in the Gynecologic Oncology Group: evaluation of ovarian cancer markers, profiles, and novel therapies	Darcy, , KM; Birrer, MJ	2010
TNF-alpha Directed Therapy in Asthma	NCT00278083	2006
Leukocyte filtration ameliorates the inflammatory response in patients with mild to moderate lung dysfunction	Onorati, , F, Santini, F, Mariscalco, G, Bertolini, P, Sala, A, Faggian, G; Mazzucco, A	2011
Estimate of CRP and TNF-alpha level before and after periodontal therapy in cardiovascular disease patients	Koppolu, , P, Durvasula, S, Palaparthy, R, Rao, M, Sagar, V, Reddy, SK; Lingam, S	2013
Dynamic changes in cytokine levels in serum and synovial fluid following filtration leukocytapheresis therapy in patients with rheumatoid arthritis	Hidaka, , T, Suzuki, K, Kawakami, M, Okada, M, Kataharada, K, Shinohara, T, Takamizawa-Matsumoto, M; Ohsuzu, F	2001
Complete Endoscopic Thyroidectomy via Oral Vestibular Approach Versus Areola Approach for Treatment of Thyroid Diseases	Yang, , J, Wang, C, Li, J, Yang, W, Cao, G, Wong, HM, Zhai, H; Liu, W	2015
Effect of low-dose oral prednisolone on symptoms and systemic inflammation in older adults with moderate to severe knee osteoarthritis: a randomized placebo-controlled trial	Abou-Raya, , A, Abou-Raya, S, Khadrawi, T; Helmii, M	2014
Combination of hemofiltration and peritoneal dialysis in the treatment of severe acute pancreatitis	Yang, , C, Guanghua, F, Wei, Z, Zhong, J, Penghui, J, Xin, F; Xiping, Z	2010
Effects of combined administration of low dose atorvastatin and vitamin E on inflammatory markers and endothelial function in patients with heart failure	Tousoulis, , D, Antoniades, C, Vassiliadou, C, Toutouza, M, Pitsavos, C, Tentolouris, C, Trikas, A; Stefanadis, C	2005
Can neutrophil to lymphocyte ratio (NLR) or eosinophil to lymphocyte ratio (ELR) be used to predict the outcome of omalizumab treatment in eosinophilic and non-eosinophilic severe allergic asthma?	Ozgen, , H, Bulut, I, Tepetam, FM; Orcen, C	2018
Acupuncture Combined with Hydrotherapy in Diabetes Patients with Mild Lower-Extremity Arterial Disease: a Prospective, Randomized, Nonblinded Clinical Study	Qi, , Z, Pang, Y, Lin, L, Zhang, B, Shao, J, Liu, X; Zhang, X	2018
Efficacy of short-term adjunctive subantimicrobial dose doxycycline in diabetic patients–randomized study	Gilowski, , L, Kondzielnik, P, Wiench, R, Płocica, I, Strojek, K; Krzemiński, TF	2012
Does the Oral Contraceptive Pill Increase Plasma Intercellular Adhesion Molecule-1, Monocyte Chemoattractant Protein-1, and Tumor Necrosis Factor-alpha Levels in Women with Polycystic Ovary Syndrome: a Pilot Study	Yousuf, , SD, Rashid, F, Mattoo, T, Shekhar, C, Mudassar, S, Zargar, MA; Ganie, MA	2016
Short-term efficacy and safety of sitagliptin treatment in long-term stable renal recipients with new-onset diabetes after transplantation	Strøm Halden, , TA, Åsberg, A, Vik, K, Hartmann, A; Jenssen, T	2014
Longterm safety and efficacy of abatacept through 5 years of treatment in patients with rheumatoid arthritis and an inadequate response to tumor necrosis factor inhibitor therapy	Genovese, , MC, Schiff, M, Luggen, M, Le Bars, M, Aranda, R, Elegbe, A; Dougados, M	2012
Gene polymorphisms and serological markers of patients with active Crohn's disease in a clinical trial of antisense to ICAM-1	Yacyshyn, , BR, Schievella, A, Sewell, KL; Tami, JA	2005
Rationale and design of SuPPoRT: a multi-centre randomised controlled trial to compare three treatments: cervical cerclage, cervical pessary and vaginal progesterone, for the prevention of preterm birth in women who develop a short cervix	Hezelgrave, , NL, Watson, HA, Ridout, A, Diab, F, Seed, PT, Chin-Smith, E, Tribe, RM; Shennan, AH	2016
Effects of adalimumab therapy in adult subjects with moderate-to-severe psoriasis on Th17 pathway	Balato, , A, Schiattarella, M, Di Caprio, R, Lembo, S, Mattii, M, Balato, N; Ayala, F	2014
Therapeutic effectiveness of intravenous immunoglobulin at 1 g/kg and 2 g/kg on Kawasaki disease: a comparative and follow-up study	Qin, , LJ, Wang, HW, Hu, XF, Liu, QJ, Shi, H, Wei, YX, Chen, QJ; Cheng, PX	2006
Adherence to the Mediterranean diet attenuates inflammation and coagulation process in healthy adults: the ATTICA Study	Chrysohoou, , C, Panagiotakos, DB, Pitsavos, C, Das, UN; Stefanadis, C	2004
Safety and Efficacy of Open-label Subcutaneous Ixekizumab Treatment for 48 Weeks in a Phase II Study in Biologic-naive and TNF-IR Patients with Rheumatoid Arthritis	Genovese, , MC, Braun, DK, Erickson, JS, Berclaz, PY, Banerjee, S, Heffernan, MP; Carlier, H	2016
Effects of mud-bath therapy in psoriatic arthritis patients treated with TNF inhibitors. Clinical evaluation and assessment of synovial inflammation by contrast-enhanced ultrasound (CEUS)	Cozzi, , F, Raffeiner, B, Beltrame, V, Ciprian, L, Coran, A, Botsios, C, Perissinotto, E, Grisan, E, Ramonda, R, Oliviero, F, Stramare, R; Punzi, L	2015
Epigenetic alterations at diagnosis predict susceptibility, prognosis and treatment escalation in inflammatory bowel disease-IBD character	Kalla, , R, Adams, AT, Vatn, S, Bonfiglio, F, Nimmo, ER, Kennedy, NA, Ventham, N, Vatn, MH, Ricanek, P, Bergemalm, D, Halfvarson, J, Soderholm, JD, Pierik, M, Torkvist, L, Gomollon, F, Gut, I, Jahnsen, J; Satsangi, J	2017
Arterialization of peripheral venous blood in sickle cell disease	Nahavandi, , M, Millis, RM, Tavakkoli, F, Wyche, MQ, Perlin, E, Winter, WP; Castro, O	2002
Impact of mechanical and biological aortic valve replacement on coronary perfusion: a prospective, randomized study	Bakhtiary, , F, Abolmaali, N, Dzemali, O, Wittlinger, T, Doss, M, Moritz, A; Kleine, P	2006
Matrix Metalloproteinases in Patients With Critical Limb Ischemia Undergoing Surgical Revascularization	NCT02388867	2015
Individualized cryoballoon energy pulmonary vein isolation guided by real-time pulmonary vein recordings, the randomized ICE-T trial	Chun, , KR, Stich, M, Fürnkranz, A, Bordignon, S, Perrotta, L, Dugo, D, Bologna, F; Schmidt, B	2017
ACE inhibition with perindopril and biomarkers of atherosclerosis and thrombosis: results from the PERTINENT study	Ceconi, , C, Fox, KM, Remme, WJ, Simoons, ML, Deckers, JW, Bertrand, M, Parrinello, G, Kluft, C, Blann, A, Cokkinos, D; Ferrari, R	2009
Change of peripheral levels of pituitary hormones and cytokines after injection of interferon (IFN)-beta in patients with chronic hepatitis C	Ohno, , Y, Fujimoto, M, Nishimura, A; Aoki, N	1998
Early detection of melanoma progression by quantitative real-time RT-PCR analysis for multiple melanoma markers	Arenberger, , P, Arenbergerova, M, Vohradnikova, O; Kremen, J	2008

Reduced intensity allogeneic stem cell transplantation for younger patients with myelofibrosis	Mannina, , D, Wolschke, C, Zabelina, T, Zeck, G, Von Pein, U-M, Ayuk, FA; Kroeger, N	2018
EULAR recommendations for the use of imaging in spondyloarthritis in clinical practice	Mandl, , P, Navarro-Compán, V, Bakker, P, Terslev, L, Aegerter, P, Van Der Heijde, D, D'Agostino, M-A, Baraliakos, X, Pedersen, SJ, Jurik, AG, Naredo, E, Schueller-Weidekamm, C, Weber, U, Wick, M, Filippucci, E, Conaghan, PG, Rudwaleit, M, Schett, GA, Sieper, J, Tarp, S, Marzo-Ortega, H; Ostergaard, M	2014
Improvement in immune parameters and human immunodeficiency virus-1 viral response in individuals treated with 16alpha-bromoepiandrosterone (HE2000)	Reading, , C, Dowding, C, Schramm, B, Garsd, A, Onizuka-Handa, N, Stickney, D; Frincke, J	2006
Effects of dietary supplementation with a standardized aqueous extract of Terminalia chebula fruit (AyuFlex®) on joint mobility, comfort, and functional capacity in healthy overweight subjects: a randomized placebo-controlled clinical trial	Lopez, , HL, Habowski, SM, Sandrock, JE, Raub, B, Kedia, A, Bruno, EJ; Ziegenfuss, TN	2017
Effects of Angiotensin-converting enzyme inhibition and statin treatment on inflammatory markers and endothelial functions in patients with longterm rheumatoid arthritis	Tikiz, , C, Utuk, O, Pirildar, T, Bayturan, O, Bayindir, P, Taneli, F, Tikiz, H; Tuzun, C	2005
Preoperative evaluation of chronic alcoholics assessed for surgery of the upper digestive tract	Martin, , MJ, Heymann, C, Neumann, T, Schmidt, L, Soost, F, Mazurek, B, Böhm, B, Marks, C, Helling, K, Lenzenhuber, E, Müller, C, Kox, WJ; Spies, CD	2002
Effect of Locally-Applied Simvastatin on Clinical Attachment Level and Alveolar Bone in Periodontal Maintenance Patients	NCT03452891	2018
A novel in vivo bioassay of topical drug potency and stability: an effective correlate to clinical SCORAD in atopic dermatitis	Alex, , P, Williams, S, Sutton, L, Sutton, C, Payne, A; Centola, M	2016
Diminished in vitro production of interleukin-1 and tumor necrosis factor-alpha during acute visceral leishmaniasis and recovery after therapy	Ho, , JL, Badaró, R, Schwartz, A, Dinarello, CA, Gelfand, JA, Sobel, J, Barral, A, Netto, MB, Carvalho, EM; Reed, SG	1992
A phase II randomized clinical trial of high-dose versus standard-dose vitamin d3 in an ethnically diverse sample of older adults	Olichney, , J, DeCarli, C, Miller, JW, Johnson, D, Tomaszewski-Farias, S, Hammock, B, Dugger, B, Jin, L-W, McPhail-Ciufo, M, Soohoo, R, Mungas, D; Harvey, D	2018
Efficacy of Different Perioperative Statin Regimens on Protection Against Post-Coronary Artery Bypass Grafting Major Adverse Cardiac and Cerebral Events	Elmarsafawi, , AG, Abbassi, MM, Elkaffas, S, Elsawy, HM; Sabry, NA	2016
The effect of anticoagulant therapy on coagulation and inflammation markers in sepsis patients and its significance	Han, , Y, Gao, C, Qin, B, Xu, H, Song, X, Li, B, Peng, B, Fan, T; Cheng, Z	2015
Anti-inflammatory Effects of GTS-21 After LPS	NCT00783068	2008
Pentraxin 3 is uninfluenced by high doses of concentrated omega-3 fatty acids administered for 12 months following an acute myocardial infarction	Poenitz, , V, Grundt, H, Bottazzi, B, Cuccovillo, I, Mantovani, A; Nilsen, DW	2012
The influence of selective pulmonary perfusion on the inflammatory response and clinical outcome of patients with chronic obstructive pulmonary disease undergoing cardiopulmonary bypass	Kiessling, , AH, Guo, FW, Gökdemir, Y, Thudt, M, Reyher, C, Scherer, M, Beiras-Fernandez, A; Moritz, A	2014
Effects of LLLT on the periarthritis of the shoulder: a clinical study on different treatments with low level laser therapy, corticosteroid injections or a wait-and-see policy	Tam, G	2012
Effects of pulsatile and nonpulsatile perfusion on vital organ recovery in pediatric heart surgery: a pilot clinical study	Alkan, , T, Akçevin, A, Undar, A, Türkoğlu, H, Paker, T; Aytaç, A	2006
Minimally invasive cardiac surgery–the efficacy of right parasterna approach	Sawa, , Y; Matsuda, H	1998
Clinical efficacy, safety and anti-inflammatory activity of two sevelamer tablet forms in patients on low-flux hemodialysis	Tzanno-Martins, , C, Biavo, BM, Ferreira-Filho, O, Ribeiro-Junior, E, João-Luiz, MV, Degaspari, S, Scavone, C; Kawamoto, E	2014
Meta-analysis: factors predicting post-operative recurrence with placebo therapy in patients with Crohn's disease	Pascua, , M, Su, C, Lewis, JD, Brensinger, C; Lichtenstein, GR	2008
Identification of breast cancers with an indolent disease course: 70 gene indolent threshold validation in a Swedish randomized trial of tamoxifen vs. not, with 20 year outcomes	Esserman, , LJ, Yau, C, Thompson, CK, Van't Veer, LJ, Borowsky, AD, Hoadley, KA, Tobin, NP, Nordenskjold, B, Fornander, T, Stal, O, Benz, CC; Lindstrom, LS	2017
Denosumab for treating periprosthetic osteolysis; Study protocol for a randomized, double-blind, placebo-controlled trial	Skoldenberg, , O, Rysinska, A, Eisler, T, Salemyr, M, Boden, H; Muren, O	2016
Low-dose theophylline does not exert its anti-inflammatory effects in mild asthma through upregulation of interleukin-10 in alveolar macrophages	Oliver, , B, Tomita, K, Keller, A, Caramori, G, Adcock, I, Chung, KF, Barnes, PJ; Lim, S	2001
Effects of Nd: YAG and CO2 laser treatment and ultrasonic scaling on periodontal pockets of chronic periodontitis patients	Miyazaki, , A, Yamaguchi, T, Nishikata, J, Okuda, K, Suda, S, Orima, K, Kobayashi, T, Yamazaki, K, Yoshikawa, E; Yoshie, H	2003
Bosentan Improves Clinical Outcome of Adults With Congenital Heart Disease or Mitral Valve Lesions Who Undergo CArdiac Surgery	NCT01184404	2010
Evaluation of perfusion modes on vital organ recovery and thyroid hormone homeostasis in pediatric patients undergoing cardiopulmonary bypass	Akçevin, , A, Alkan-Bozkaya, T, Qiu, F; Undar, A	2010
Interleukin-6 enhancement after direct autologous retransfusion of shed thoracic blood does not influence haemodynamic stability following coronary artery bypass grafting	Schroeder, , S, von Spiegel, T, Stuber, F, Hoeft, A, Preusse, CJ, Welz, A, Kampe, S; Lier, H	2007
Dietary supplementation with low-dose omega-3 fatty acids reduces salivary tumor necrosis factor-α levels in patients with chronic periodontitis: a randomized controlled clinical study	Keskiner, , I, Saygun, I, Bal, V, Serdar, M; Kantarci, A	2017
Curcumin reduces muscle damage and soreness following muscle-damaging exercise	Jager, , R, Caldwell, AR, Sanders, E, Mitchell, JB, Rogers, J, Purpura, M; Oliver, JM	2017

Preoperative oral pentoxifylline in case of coronary artery bypass grafting with left ventricular dysfunction (Ejection fraction equal to/less than 30%)	Mansourian, , S, Bina, P, Fehri, A, Karimi, AA, Boroumand, MA; Abbasi, K	2015
Ingested type I interferon: state of the art as treatment for autoimmunity	Brod, SA	2002
Macrophage activity assessed by soluble CD163 in early rheumatoid arthritis: association with disease activity but different response patterns to synthetic and biologic DMARDs	Greisen, , SR, Møller, HJ, Stengaard-Pedersen, K, Hetland, ML, Hørslev-Petersen, K, Junker, P, Østergaard, M, Hvid, M; Deleuran, B	2015
Resveratrol improves treatment outcome and laboratory parameters in patients with Takayasu arteritis: a randomized double-blind and placebo-controlled trial	Shi, , G, Hua, M, Xu, Q; Ren, T	2017
Successful short term treatment of severe undifferentiated spondyloarthropathy with the anti-tumor necrosis factor-alpha monoclonal antibody infliximab	Brandt, , J, Haibel, H, Reddig, J, Sieper, J; Braun, J	2002
Effects of high-dose mucosolvin on lung functions in infant patients with cardiopulmonary bypass	Zhao, , K, Wang, W, Zhang, J, Zhao, R, Chen, T, Su, J, Ma, C; Cui, Q	2011
Efficacy and safety of olokizumab in patients with rheumatoid arthritis with an inadequate response to TNF inhibitor therapy: outcomes of a randomised Phase IIb study	Genovese, , MC, Fleischmann, R, Furst, D, Janssen, N, Carter, J, Dasgupta, B, Bryson, J, Duncan, B, Zhu, W, Pitzalis, C, Durez, P; Kretsos, K	2014
Prehabilitation in Frail Colon Cancer	NCT03097224	2017
Cardiac rehabilitation versus standard care after aortic aneurysm repair (Aneurysm CaRe): study protocol for a randomised controlled trial	Bahia, , SS, Holt, PJ, Ray, KK, Ussher, M, Poloniecki, JD, Sharma, R, Bown, MJ, Hinchliffe, RJ, Thompson, MM; Karthikesalingam, A	2015
Abnormal high-expression of CD154 on T lymphocytes of ankylosing spondylitis patients is down-regulated by etanercept treatment	Lin, , Q, Lin, Z, Gu, J, Huang, F, Li, T, Wei, Q, Liao, Z, Cao, S, Jiang, Y; Huang, J	2010
Beating heart revascularization with or without cardiopulmonary bypass: evaluation of inflammatory response in a prospective randomized study	Wan, , IY, Arifi, AA, Wan, S, Yip, JH, Sihoe, AD, Thung, KH, Wong, EM; Yim, AP	2004
Effect of meloxicam on gingival crevicular fluid IL-1beta and IL1 receptor antagonist levels in subjects with chronic periodontitis, and its effects on clinical parameters	Toker, , H, Marakoglu, I; Poyraz, O	2006
Clinical and histological evaluation of a single high energy microwave treatment for primary axillary hyperhidrosis in Asians: a prospective, randomized, controlled, split-area comparative trial	Kaminaka, , C, Mikita, N, Inaba, Y, Kunimoto, K, Okuhira, H, Jinnin, M, Kao, B, Tanino, R, Tanioka, K, Shimokawa, T; Yamamoto, Y	2019
Treatment with levamisole and colchicine can result in a significant reduction of IL-6, IL-8 or TNF-alpha level in patients with mucocutaneous type of Behcet's disease	Sun, , A, Wang, YP, Chia, JS, Liu, BY; Chiang, CP	2009
Systemic levels of endothelin correlate with systemic inflammation and not with myocardial injury or left ventricular ejection fraction in patients undergoing percutaneous coronary intervention and on-pump coronary artery bypass grafting	Cuculi, , F, Lim, CC, van Gaal, W, Testa, L, Westaby, S, Arnold, JR, Neubauer, S; Banning, AP	2011
Colchicine for prevention of atrial fibrillation recurrence after pulmonary vein isolation: mid-term efficacy and effect on quality of life	Deftereos, , S, Giannopoulos, G, Efremidis, M, Kossyvakis, C, Katsivas, A, Panagopoulou, V, Papadimitriou, C, Karageorgiou, S, Doudoumis, K, Raisakis, K, Kaoukis, A, Alexopoulos, D, Manolis, AS, Stefanadis, C; Cleman, MW	2014
Randomised controlled trial of inhaled corticosteroids (fluticasone propionate) in cystic fibrosis	Balfour-Lynn, , IM, Klein, NJ; Dinwiddie, R	1997
Efficacy of scaling and root planning with and without adjunct Nd: YAG laser therapy on clinical periodontal parameters and gingival crevicular fluid interleukin 1-beta and tumor necrosis factor-alpha levels among patients with periodontal disease: a prospective randomized split-mouth clinical study	Abduljabbar, , T, Vohra, F, Kellesarian, SV; Javed, F	2017
Increased use of inhaled nitric oxide in single ventricle patients with low nasal nitric oxide undergoing congenital heart surgery	Adams, , PS, Zahid, M, Khalifa, O; Lo, CW	2016
CD4+CD28-T-cell expansions in anca-associated vasculitis are associated with arterial stiffness	Chanouzas, , D, Dyall, L, Dale, J, Moss, P, Morgan, MD; Harper, L	2015
Flaxseed Oil Supplementation Improve Gene Expression Levels of PPAR-γ, LP(a), IL-1 and TNF-α in Type 2 Diabetic Patients with Coronary Heart Disease	Hashemzadeh, , AA, Nasoohi, N, Raygan, F, Aghadavod, E, Akbari, E, Taghizadeh, M, Memarzadeh, MR; Asemi, Z	2017
Nitric oxide decreases acute kidney injury and stage 3 chronic kidney disease after cardiac surgery	Lei, , C, Berra, L, Rezoagli, E, Yu, B, Dong, H, Yu, S, Hou, L, Chen, M, Chen, W, Wang, H, Zheng, Q, Shen, J, Jin, Z, Chen, T, Zhao, R, Christie, E, Sabbisetti, VS, Nordio, F, Bonventre, JV, Xiong, L; Zapol, WM	2018
5-Aminosalicylic Acid Modulates the Immune Response in Chronic Beryllium Disease Subjects	Day, , BJ, Huang, J, Barkes, BQ, Gillespie, M, Li, L; Maier, LA	2018
Evaluation of cutaneous microcirculation of three cosmaceuticals containing different percentage of active (biotropicsFD101): an open, single centric, placebo control, intra-individual study in healthy volunteers	Ming, , YK, Haris, HH, Vandana Kotak, C; Choudhary, YK	2013
Pentoxifylline lowers plasminogen activator inhibitor 1 levels in obese individuals: a pilot study	Muldowney, , JA, Chen, Q, Blakemore, DL; Vaughan, DE	2012
Relationship between non-specific prescription pill adherence and ischemic stroke outcomes	Ovbiagele, , B, Campbell, S, Faiz, A; Chambless, LE	2010
ASDAS, a highly discriminatory ASAS-endorsed disease activity score in patients with ankylosing spondylitis	van der Heijde, , D, Lie, E, Kvien, TK, Sieper, J, Van den Bosch, F, Listing, J, Braun, J; Landewé, R	2009
Modified glucose-insulin-potassium regimen provides cardiac and renal protection with improved tissue perfusion and metabolism in patients undergoing cardiopulmonary bypass surgery	Li, , J, Zhao, K, Zhang, Y, Cui, Q, Zhao, R, Yang, J, Yu, S, Yi, D; Gao, F	2015

The Effects of Probiotic Supplementation on Gene Expression Related to Inflammation, Insulin, and Lipids in Patients With Multiple Sclerosis: a Randomized, Double-Blind, Placebo-Controlled Trial	Tamtaji, , OR, Kouchaki, E, Salami, M, Aghadavod, E, Akbari, E, Tajabadi-Ebrahimi, M; Asemi, Z	2017
Cardioprotective effect of transcutaneous electric acupoint stimulation in the pediatric cardiac patients: a randomized controlled clinical trial	Ni, , X, Xie, Y, Wang, Q, Zhong, H, Chen, M, Wang, F; Xiong, L	2012
Early onset hyperuricemia is a prognostic marker for kidney allograft dysfunction	Jung, , IM, Han, M, Yang, SH, Park, S-K, Han, DJ, Kim, YS, Kim, YH; Lee, JP	2016
Effect of erythropoietin on the incidence of acute kidney injury following complex valvular heart surgery: a double blind, randomized clinical trial of efficacy and safety	Kim, , JH, Shim, JK, Song, JW, Song, Y, Kim, HB; Kwak, YL	2013
Adjunctive benefits of subantimicrobial dose doxycycline in the management of severe, generalized, chronic periodontitis	Novak, , MJ, Johns, LP, Miller, RC; Bradshaw, MH	2002
Side effects from intense pulsed light: importance of skin pigmentation, fluence level and ultraviolet radiation-A randomized controlled trial	Thaysen-Petersen, , D, Erlendsson, AM, Nash, JF, Beerwerth, F, Philipsen, PA, Wulf, HC, Paasch, U; Haedersdal, M	2017
Peritoneal dialysis does not adversely affect kidney function recovery after congenital heart surgery	Riley, , AA, Jefferies, JL, Nelson, DP, Bennett, MR, Blinder, JJ, Ma, Q, Devarajan, P; Goldstein, SL	2014
Efficacy and safety of the selective co-stimulation modulator abatacept following 2 years of treatment in patients with rheumatoid arthritis and an inadequate response to anti-tumour necrosis factor therapy	Genovese, , MC, Schiff, M, Luggen, M, Becker, JC, Aranda, R, Teng, J, Li, T, Schmidely, N, Le Bars, M; Dougados, M	2008
The effects of omega-3 and vitamin E co-supplementation on parameters of mental health and gene expression related to insulin and inflammation in subjects with polycystic ovary syndrome	Jamilian, , M, Shojaei, A, Samimi, M, Afshar Ebrahimi, F, Aghadavod, E, Karamali, M, Taghizadeh, M, Jamilian, H, Alaeinasab, S, Jafarnejad, S; Asemi, Z	2018
First-in-man results of a novel vascular graft coated with resorbable polymer for aortic reconstructions–a multicenter, non-randomized safety study	Halloul, , Z, Rimpler, H, Waliszewski, M, Beier, N, Meyer, F, Rappe, A, Fuellert, A; Tautenhahn, J	2014
High-dose Vitamin D Supplements in Older Adults	NCT03613116	2018
Cardiovascular effects of tumour necrosis factor alpha antagonism in patients with acute myocardial infarction: a first in human study	Padfield, , GJ, Din, JN, Koushiappi, E, Mills, NL, Robinson, SD, Le May Cruden, N, Lucking, AJ, Chia, S, Harding, SA; Newby, DE	2013
Inspired supplemental oxygen reduces markers of oxidative stress during elective colon surgery	García de la Asunción, , J, Belda, FJ, Greif, R, Barber, G, Viña, J; Sastre, J	2007
Blood cardioplegia with N-acetylcysteine may reduce coronary endothelial activation and myocardial oxidative stress	Rodrigues, , AJ, Evora, PR, Bassetto, S, Alves, L, Scorzoni Filho, A, Origuela, EA; Vicente, WV	2009
Preoperative predictors of postsurgical adhesion formation and the Prevention of Adhesions with Plasminogen Activator (PAPA-study): results of a clinical pilot study	Hellebrekers, , BW, Trimbos-Kemper, TC, Boesten, L, Jansen, FW, Kolkman, W, Trimbos, JB, Press, RR, van Poelgeest, MI, Emeis, SJ; Kooistra, T	2009
Effectiveness of pulmonary rehabilitation and correlations in between functional parameters, extent of thoracic surgery and severity of post-operative complications: randomized clinical trial	Vagvolgyi, , A, Rozgonyi, Z, Kerti, M, Agathou, G, Vadasz, P; Varga, J	2018
Preoperative oral feeding reduces stress response after laparoscopic cholecystectomy	Zelić, , M, Štimac, D, Mendrila, D, Tokmadžić, VS, Fišić, E, Uravić, M; Šustić, A	2013
Impact of vitamin D supplementation on inflammatory markers in African Americans: results of a four-arm, randomized, placebo-controlled trial	Chandler, , PD, Scott, JB, Drake, BF, Ng, K, Manson, JE, Rifai, N, Chan, AT, Bennett, GG, Hollis, BW, Giovannucci, EL, Emmons, KM; Fuchs, CS	2014
Cardioprotective effect of pretreatment with beta-glucan in coronary artery bypass grafting	Aarsaether, , E, Rydningen, M, Einar Engstad, R; Busund, R	2006
Dietary supplementation with rice bran fermented with Lentinus edodes increases interferon- activity without causing adverse effects: a randomized, double-blind, placebo-controlled, parallel-group study	Choi, , J-Y, Paik, D-J, Kwon, DY; Park, Y	2014
No evidence of an effect of alterations in dietary fatty acids on fasting adiponectin over 3 weeks	Lithander, , FE, Keogh, GF, Wang, Y, Cooper, GJ, Mulvey, TB, Chan, YK, McArdle, BH; Poppitt, SD	2008
Pathologic Response, When Increased by Longer Interval, Is a Marker but Not the Cause of Good Prognosis in Rectal Cancer: 17-year Follow-up of the Lyon R90-01 Randomized Trial		2016
Preoperative peripheral plasma fibrinogen level is an independent prognostic marker in penile cancer	Ma, , C, Zhou, Y, Zhou, S, Zhao, K, Lu, B; Sun, E	2017
Beneficial effects of 1-year optimal medical treatment with and without additional PTA on inflammatory markers of atherosclerosis in patients with PAD. Results from the Oslo Balloon Angioplasty versus Conservative Treatment (OBACT) study	Nylaende, , M, Kroese, AJ, Morken, B, Stranden, E, Sandbaek, G, Lindahl, AK, Arnesen, H; Seljeflot, I	2007
Effect of renin angiotensin system blockade on pentraxin 3 levels in type-2 diabetic patients with proteinuria	Yilmaz, , MI, Axelsson, J, Sonmez, A, Carrero, JJ, Saglam, M, Eyileten, T, Caglar, K, Kirkpantur, A, Celik, T, Oguz, Y, Vural, A, Yenicesu, M, Lindholm, B; Stenvinkel, P	2009
Thalidomide for the Treatment of Malnutrition Inflammation Syndrome in Peritoneal Dialysis Patients	NCT00390247	2006
Omega-3 fatty acids-supplemented parenteral nutrition decreases hyperinflammatory response and attenuates systemic disease sequelae in severe acute pancreatitis: a randomized and controlled study	Wang, , X, Li, W, Li, N; Li, J	2008
Fresh whole blood versus reconstituted blood for pump priming in heart surgery in infants	Mou, , SS, Giroir, BP, Molitor-Kirsch, EA, Leonard, SR, Nikaidoh, H, Nizzi, F, Town, DA, Roy, LC, Scott, W; Stromberg, D	2004
A phase II multicenter trial to evaluate efficacy and safety of HF10 oncolytic virus immunotherapy and ipilimumab in patients with unresectable or metastatic melanoma	Andtbacka, , RHI, Ross, M, Agarwala, S, Grossmann, K, Taylor, M, Vetto, J, Neves, R, Daud, A, Khong, H, Meek, SM, Ungerleider, R, Welden, S, Tanaka, M; Williams, M	2016
A study of safety and efficacy of lenabasum in Cystic Fibrosis	EUCTR2017-003723-29-GR	2018

Clinical trial of Methotrexate associated to Hydroxyurea versus placebo and Hydroxyurea in patients with sickle cell disease	RBR-57qbjv	2018
Telomerase activator TA-65MD® in patients with ACS (TACTIC)	ISRCTN16613292	2018
Physical exercise for hemodialysis patients	RBR-29s6x3	2016
Effect of the immunomodulating agent, pentoxifylline, in the treatment of sepsis in prematurely delivered infants: a placebo-controlled, double-blind trial	Lauterbach, , R, Pawlik, D, Kowalczyk, D, Ksycínski, W, Helwich, E; Zembala, M	1999
Anti-TNF-induced remission in very early peripheral spondyloarthritis: the CRESPA study	Carron, , P, Varkas, G, Cypers, H, Van Praet, L, Elewaut, D; Van den Bosch, F	2017
Sepsis-induced cardiac dysfunction and beta-adrenergic blockade therapy for sepsis	Suzuki, , T, Suzuki, Y, Okuda, J, Kurazumi, T, Suhara, T, Ueda, T, Nagata, H; Morisaki, H	2017
Protocol for the Imagine HEALTH Study: guided imagery lifestyle intervention to improve obesity-related behaviors and salivary cortisol patterns in predominantly Latino adolescents	Weigensberg, , MJ, Spruijt-Metz, D, Wen, CKF, Davis, JN, Avila, Q, Juarez, M, Brown-Wade, N; Lane, CJ	2018
Clinical factors affecting the survival of patients diagnosed with non-small cell lung cancer and metastatic malignant pleural effusion, treated with hyperthermic intrathoracic chemotherapy or chemical talc pleurodesis: a monocentric, prospective, randomized trial	Sioga, , A, Pandria, N, Barbetakis, N, Lazopoulos, A, Katsikas, I, Asteriou, C, Paliouras, D, Kamperis, E, Ikonomou, D, Papamitsou, T, Filippou, D, Destouni, C, Ikonomou, L, Zarogoulidis, K, Papagiannopoulos, K; Kleontas, A	2019
Phase II Trial of Weekly Intravenous Paclitaxel plus Three Weekly Intraperitoneal Carboplatin Administration for Suboptimally Debulked Ovarian and Primary Peritoneal Carcinoma Incorporating Pharmacogenomic Analysis	JPRN-UMIN000001713	2009
CD19-CART01 in pediatric patients affected by relapsed/refractory CD19+ Acute Lymphoblastic Leukemia and Non Hodgkin Lymphoma	EUCTR2017-002088-16-IT	2018
Clinical efficacy of nitroglycerin in patients with septic shock with ARDS	Liu, , L-P, Li, B, Zhu, L, Dou, Z-M; Li, Y-M	2015
Efficacy and safety of thalidomide in pediatric patients with Crohn's disease	EUCTR2016-000786-24-IT	2016
Effects of Quercetin on plasma total antioxidant capacity, lipid peroxidation, blood pressure, inflammatory markers and disease activity in patients with Rheumatoid Arthritis	IRCT138807252394N2	2010
An Open Label, 1-Year Trial, including a Double-Blind Placebo-Controlled Withdrawal Period, of Setmelanotide (RM-493), a Melanocortin 4 Receptor (MC4R) Agonist, in Early Onset POMC Deficiency Obesity due to Bi-Allelic Loss-of-Function POMC or PCSK1 Genetic Mutation	EUCTR2016-002320-83-DE	2016
Randomized controlled trial of Stopping Perioperative Angiotensin-II Converting Enzyme inhibitors and/or Angiotensin Receptor Blockers in major noncardiac surgery	EUCTR2016-004141-90-GB	2018
Study of Durvalumab or Placebo Given along with Platinum-based Chemo-radiation Therapy in Patients with Locally Advanced where cancer cannot be removed completely through surgery for Non-small Cell Lung Cancer (Stage III)	CTRI/2018/07/014783	2018
Effects of Different Volatile Anesthetics on Cytokine and Chemokine Production After Ischemia-Reperfusion Injury in Patients Undergoing Living-Donor Kidney Transplant	Savran Karadeniz, , M, Senturk Ciftci, H, Tefik, T, Oktar, T, Nane, I, Turkmen, A, Oguz, F; Tugrul, KM	2019
Efficacy and safety of pulsed infusions of levosimendan in outpatients with advanced heart failure	EUCTR2008-007407-86-DE	2011
Anti-inflammatory effects of exercise training in the skeletal muscle of patients with chronic heart failure	Gielen, , S, Adams, V, Möbius-Winkler, S, Linke, A, Erbs, S, Yu, J, Kempf, W, Schubert, A, Schuler, G; Hambrecht, R	2003
Effect of homocysteine-lowering treatment with folic acid plus vitamin B6 on progression of subclinical atherosclerosis: a randomised, placebo-controlled trial	Vermeulen, , EG, Stehouwer, CD, Twisk, JW, van den Berg, M, de Jong, SC, Mackaay, AJ, van Campen, CM, Visser, FC, Jakobs, CA, Bulterjis, EJ; Rauwerda, JA	2000
Every-other-week methotrexate in patients with rheumatoid arthritis. A double-blind, placebo-controlled prospective study	Kremer, , JM, Davies, JM, Rynes, RI, Fink, S, Lawrence, DA, Petrillo, GF; Mullaly, PM	1995
STUDY OF THE EFFECTIVENESS OF THE COMBINED TREATMENT WITH DONEPEZILO, INTENSIVE REHABILITATION AND ELECTRIC STIMULATION OF THE BRAIN IN PATIENTS WITH AFASIA AFTER STROKE	EUCTR2017-002858-36-ES	2018
A Rheumatoid Arthritis Study to Test the Safety, Tolerability, and Efficacy of MK-8457 in Patients with an Inadequate Response or Intolerance to Anti-TNF-alpha Therapy	ACTRN12612000774820	2012
A clinical trial of MDNA55 in adults with a brain tumour	EUCTR2016-003841-27-PL	2017
Efficacy of Melatonin on Serum Pro-inflammatory Cytokines and Oxidative Stress Markers in Relapsing Remitting Multiple Sclerosis	Sánchez-López, , AL, Ortiz, GG, Pacheco-Moises, FP, Mireles-Ramírez, MA, Bitzer-Quintero, OK, Delgado-Lara, DLC, Ramírez-Jirano, LJ; Velázquez-Brizuela, IE	2018
A study of safety and efficacy of lenabasum in Cystic Fibrosis	EUCTR2017-003723-29-PT	2018
Clinical trial to demonstrate safety and efficacy of an intravenous (i.v.) administration of Feramyl compared to i.v. Ferinject and to iron tablets in patients with iron deficiency diganosis before a planned operation who develop anaemia after surgery	EUCTR2017-003439-12-DE	2017
Community-Acquired Pneumonia : evaluation of COrticosteroiDs (CAPE COD)	EUCTR2015-001239-19-FR	2015
Liposomal bupivacaine in knee replacement surgery	ISRCTN54191675	2017

Clinical study to assess the efficacy and safety of GFT505 80 mg and GFT505 120 mg daily for 52 weeks in Patients with Non-Alcoholic Steatohepatitis (accumulation of fat in the liver associated with inflammation and liver cell injury at microscopic examination of liver biopsy)	EUCTR2012-000295-42-NL	2012
The effect of green tea tablets in the treatment of polycystic ovary syndrome	IRCT2015021816123N4	2015
A clinical study of recurrent ovarian ovarian cancer in the treatment of platinum resistance in apatinib tablet	ChiCTR-OPN-17011652	2017
Phase 2 Study to see if QPI-1002 is effective and safe of for the Prevention of Acute Kidney Injury in Subjects at High Risk for AKI following Cardiac Surgery	EUCTR2015-003113-15-DE	2016
(OSKIRA-3): a PHASE III, MULTI-CENTRE, RANDOMIZED, DOUBLE-BLIND, PLACEBO-CONTROLLED, PARALLEL GROUP STUDY OF TWO DOSING REGIMENS OF FOSTAMATINIB DISODIUM IN RHEUMATOID ARTHRITIS PATIENTS WOTH INADEQUATE RESPONSE TO A TNF-ALPHA ANTAGONIST	PER-003-12	2012
Intermittent furosemide therapy as a prophylaxis of acute renal failure in CABG patients	IRCT201502122804N8	2015
Antioxidant therapy in implementing lithotripsy in patients with renal lithiasis	ISRCTN98702137	2010
Phyllantus amarus for the protection of liver health	ISRCTN90321640	2012
A PHASE 3, MULTICENTER, RANDOMIZED, DOUBLED-BLIND, PLACEBO-CONTROLLED STUDY TO EVALUATE THE EFFICACY AND SAFETY OF LY2127399 IN PATIENTS WITH MODERATE TO SEVERE RHEUMATOID ARTHRITIS (RA) WHO HAD AN INADEQUATE RESPONSE TO ONE OR MORE TNF-α INHIBITORS	PER-088-11	2012
A Study of IMM 101 in combination with various standard cancer treatments in patients with advanced cancer or cancer which cannot be removed by surgery	EUCTR2016-001459-28-GB	2016
Off-pump coronary bypass operations significantly reduce S100 release: an indicator for less cerebral damage?	Wandschneider, , W, Thalmann, M, Trampitsch, E, Ziervogel, G; Kobinia, G	2000
Randomized evaluation of the safety and efficacy of enoxaparin versus unfractionated heparin in high-risk patients with non-ST-segment elevation acute coronary syndromes receiving the glycoprotein IIb/IIIa inhibitor eptifibatide	Goodman, , SG, Fitchett, D, Armstrong, PW, Tan, M; Langer, A	2003
A PHASE 2A, RANDOMIZED, DOUBLE-BLIND, PLACEBO-CONTROLLED STUDY TO EVALUATE THE EFFICACY, SAFETY AND PHARMACOKINETICS OF ORALLY ADMINISTERED ALS-008176 IN ADULTS HOSPITALIZED WITH A RESPIRATORY SYNCYTIAL VIRUS-RELATED ILLNESS	PER-014-16	2016
A Study to Evaluate the Efficacy and Safety of Ustekinumab in the Treatment of Anti-TNF(alpha) Refractory Participants With Active Radiographic Axial Spondyloarthritis	EUCTR2015-000288-16-DE	2015
Pembrolizumab as First Line Treatment in Subjects with Recurrent/Metastatic HNSCC	PER-016-15	2015
A clinical study on the application of physostigminsalicylate (Anticholium®) as auxiliary measure in perioperative blood poisoning/its life-threatening form	EUCTR2012-001650-26-DE	2012
An Open Label, 1-Year Trial, including a Double-Blind Placebo-Controlled Withdrawal Period, of Setmelanotide (RM-493), a Melanocortin 4 Receptor (MC4R) Agonist, in Early Onset POMC Deficiency Obesity due to Bi-Allelic Loss-of-Function POMC or PCSK1 Genetic Mutation	EUCTR2016-002320-83-ES	2018
A Phase II Study Of Sunitinib And Low Dose Metronomic Cyclophosphamide In Advanced Renal Cell Cancer - SUN-CYCL (Sunitinib + Cyclophosphamide in RCC)	EUCTR2008-008676-13-GB	2009
A multicenter, randomized, double-blind, parallel-arm, two-stage study of the efficacy and safety of AVE0005 (VEGF Trap) administered intravenously every 2 weeks in patients with platinum-resistant and topotecan- and/or liposomal doxorubicin-resistant advanced ovarian cancer	EUCTR2005-005024-14-PT	2006
To estimate and compare blood sugar levels and a biomarker in gingival fluid in gum disease patients with diabetes before and after teeth cleaning and laser treatment	CTRI/2018/05/013807	2018
Immune responses in the treatment of drug-sensitive pulmonary tuberculosis with phenylbutyrate and vitamin D3 as host directed therapy	Rekha, , RS, Mily, A, Sultana, T, Haq, A, Ahmed, S, Mostafa Kamal, SM, van Schadewijk, A, Hiemstra, PS, Gudmundsson, GH, Agerberth, B; Raqib, R	2018
Preliminary study: comparative effects of lung volume therapy between slow and fast deep-breathing techniques on pulmonary function, respiratory muscle strength, oxidative stress, cytokines, 6-minute walking distance, and quality of life in persons with COPD	Leelarungrayub, , J, Puntumetakul, R, Sriboonreung, T, Pothasak, Y; Klaphajone, J	2018
Phase IIa trial of MK0646 Tx in combo w/ Irinotecan vs Cetuximab and Irinotecan for mRC patients	EUCTR2012-000317-36-BE	2012
A multicenter, randomized, double-blind, placebo-controlled, parallel-group clinical trial to assess efficacy and safety of the herbal medicinal	EUCTR2015-001952-31-DE	2015

product Sinupret extract coated tablets in patients with chronic rhinosinusitis		
A feasibility trial to evaluate the use of an an imaging biomarker called magnetic resonance tumour regression grade (mrTRG) to identify and manage good and poor responders to chemoradiotherapy for use in patients with rectal (bowel) cancer	EUCTR2015-003009-40-GB	2015
Effect of training and ginger on obesity	IRCT201008304663N1	2012
A Phase 3, Randomized, Placebo-Controlled, Blinded, Multicenter Study of the Induction and Maintenance of Clinical Response and Remission by Vedolizumab (MLN0002) in Patients with Moderate to Severe Crohn's Disease	EUCTR2008-002783-33-EE	2008
Effect of pre-operative pain treatment on postoperative outcome after total hip or knee replacement surgery	EUCTR2013-004313-41-NL	2014
ORENCIA VERSUS ROACTEMRA BY SUBCUTANEOUS ADMINISTRATION FOR THE TREATMENT OF RHEUMATOID ARTHRITIS IN TNF ALPHA INHIBITOR INADEQUATE RESPONDER PATIENTS	EUCTR2017-000947-41-FR	2017
Venlafaxine vs. paroxetine in the acute phase of treatment for major depressive disorder among Han Chinese population in Taiwan	Wu, , Y-S, Chen, Y-C; Lu, R-B	2007
Prenatal vitamin D supplementation reduces risk of asthma/recurrent wheeze in early childhood: a combined analysis of two randomized controlled trials	Wolsk, , HM, Chawes, BL, Litonjua, AA, Hollis, BW, Waage, J, Stokholm, J, Bonnelykke, K, Bisgaard, H; Weiss, ST	2017
Effect of remote ischemia or nicorandil on myocardial injury following percutaneous coronary intervention in patients with stable coronary artery disease: a randomized controlled trial	Miyoshi, , T, Ejiri, K, Kohno, K, Nakahama, M, Doi, M, Munemasa, M, Murakami, M, Takaishi, A, Kawai, Y, Sato, T, Sato, K, Oka, T, Takahashi, N, Sakuragi, S, Mima, A, Enko, K, Hosogi, S, Nanba, S, Hirami, R, Nakamura, K; Ito, H	2017
Effect of Bosentan on Claudication Distance and Endothelium-Dependent Vasodilation in Hispanic Patients With Peripheral Arterial Disease	De Haro, , J, Bleda, S, Varela, C, Esparza, L; Acin, F	2016
High Loading Dose of Atorvastatin for the Prevention of Serum Creatinine and Cystatin C-Based Contrast-Induced Nephropathy Following Percutaneous Coronary Intervention	Fu, , N, Liang, M; Yang, S	2018
Effect of Micronized Purified Flavonoid Fraction Therapy on Endothelin-1 and TNF-α Levels in Relation to Antioxidant Enzyme Balance in the Peripheral Blood of Women with Varicose Veins	Pietrzycka, , A, Kózka, M, Urbanek, T, Stpniewski, M; Kucharzewski, M	2015
The effect of exercise training on cardiac remodelling in children and young adults with corrected tetralogy of Fallot or Fontan circulation: a randomized controlled trial	Duppen, , N, Kapusta, L, de Rijke, YB, Snoeren, M, Kuipers, IM, Koopman, LP, Blank, AC, Blom, NA, Dulfer, K, Utens, EM, Hopman, MT; Helbing, WA	2015
Effects of periodontal basic treatment on periodontal condition,clinical response and serum inflammatory parameters in rheumatoid arthritis (RA) patients with moderate to severe periodontitis	Xiao, , F, Zhang, P, Li, X, Mou, Y, Chen, H; Cai, Y	2017
Randomized trial of fish oil infusion to prevent atrial fibrillation after cardiac surgery: data from an implantable continuous cardiac monitor		2014
Effects of ingestion of Krill oil on quality of life related to mild knee pain: a randomized double-blind placebo-controlled trial	Watanabe, , H, Matahira, Y, Suzuki, N; Takara, T	2017
Nuclear imaging techniques in the assessment of myocardial perfusion and function after CABG: does it correlate with CK-MB elevation?	Kwinecki, , P, Jemielity, M, Czepczyński, R, Baszko, A, Ruchała, M, Sowiński, J; Dyszkiewicz, W	2003
Fixed-dose combination fenofibrate/pravastatin 160/40 mg versus simvastatin 20 mg monotherapy in adults with type 2 diabetes and mixed hyperlipidemia uncontrolled with simvastatin 20 mg: a double-blind, randomized comparative study	Farnier, , M, Steinmetz, A, Retterstøl, K; Császár, A	2011
Pharmacokinetic characteristics of ibandronate and tolerability of DP-R206 (150 mg Ibandronate/24,000 IU Vitamin D3) compared to the ibandronate (150 mg) monotherapy in healthy adults	Choi, , HY, Kim, MJ, Kim, YH, Noh, Y-H, Lee, J-W, Lee, T-W, Kim, M-G; Bae, K-S	2014
Oral paricalcitol versus oral calcitriol in continuous ambulatory peritoneal dialysis patients with secondary hyperparathyroidism	Jamaluddin, , EJ, Gafor, AH, Yean, LC, Cader, R, Mohd, R, Kong, NC; Shah, SA	2014
Rosiglitazone reduces insulin requirement and C-reactive protein levels in type 2 diabetic patients receiving peritoneal dialysis	Wong, , TY, Szeto, CC, Chow, KM, Leung, CB, Lam, CW; Li, PK	2005
Weight gain and increased concentrations of receptor proteins for tumor necrosis factor after patients with symptomatic HIV infection received fortified nutrition support	Süttmann, , U, Ockenga, J, Schneider, H, Selberg, O, Schlesinger, A, Gallati, H, Wolfram, G, Deicher, H; Müller, MJ	1996
Long-term anti-tumour necrosis factor therapy reverses the progression of carotid intima-media thickness in female patients with active rheumatoid arthritis	Ferrante, , A, Giardina, AR, Ciccia, F, Parrinello, G, Licata, G, Avellone, G, Giardina, E, Impastato, R; Triolo, G	2009
Interleukin-1 blockade in heart failure with preserved ejection fraction: rationale and design of the Diastolic Heart Failure Anakinra Response Trial 2 (D-HART2)	Van Tassell, , BW, Buckley, LF, Carbone, S, Trankle, CR, Canada, JM, Dixon, DL, Abouzaki, N, Oddi-Erdle, C, Biondi-Zoccai, G, Arena, R; Abbate, A	2017
Predicting treatable traits for long-acting bronchodilators in patients with stable COPD	Kang, , J, Kim, KT, Lee, JH, Kim, EK, Kim, TH, Yoo, KH, Lee, JS, Kim, WJ, Kim, JH; Oh, YM	2017
Sustained efficacy, safety and patient-reported outcomes of certolizumab pegol in axial spondyloarthritis: 4-year outcomes from RAPID-axSpA	van der Heijde, , D, Dougados, M, Landewé, R, Sieper, J, Maksymowych, WP, Rudwaleit, M, Van den Bosch, F, Braun, J, Mease, PJ, Kivitz, AJ, Walsh, J, Davies, O, Bauer, L, Hoepken, B, Peterson, L; Deodhar, A	2017
Lessons from ATTIRE feasibility study: differential inflammatory response profiles following treatment with albumin correlate with 6 months survival	Becares, , N, Harmala, S, China, L, Skene, SS, Bennett, K, Shabir, Z; O'brien, A	2018

Efficacy and tolerability of diphenyl-dimethyl-dicarboxylate plus garlic oil in patients with chronic hepatitis	Lee, , MH, Kim, YM; Kim, SG	2012
The randomized clinical trial of coenzyme Q10 for the prevention of periprocedural myocardial injury following elective percutaneous coronary intervention	Aslanabadi, , N, Safaie, N, Asgharzadeh, Y, Houshmand, F, Ghaffari, S, Garjani, A, Dousti, S, Hamishehkar, H; Entezari-Maleki, T	2016
Impacts of intensive insulin therapy in patients undergoing heart valve replacement	Zheng, , R, Gu, C, Wang, Y, Yang, Z, Dou, K, Wang, J, Yu, S, Wang, H, Xie, J, Wang, Y, Yi, D; Pei, J	2010
Effect on Periodontal Markers by Bleaching Intracoronal Technique	NCT02718183	2016
Lebrikizumab treatment in adults with asthma	Corren, , J, Lemanske, RF, Hanania, NA, Korenblat, PE, Parsey, MV, Arron, JR, Harris, JM, Scheerens, H, Wu, LC, Su, Z, Mosesova, S, Eisner, MD, Bohen, SP; Matthews, JG	2011
Effect of rosuvastatin therapy on troponin I release following percutaneous coronary intervention in nonemergency patients (from the TIP 3 study)	Veselka, , J, Hájek, P, Tomašov, P, Tesař, D, Brůhová, H, Matějovič, M, Branny, M, Studenčan, M; Zemánek, D	2014
Treating periodontal disease in patients with ST-elevation myocardial infarction: a randomized clinical trial	Lobo, , MG, Dipp, T, Petry, I, Schmidt, K, Moura, M, Gazeta, C, Azeredo, M, Wang, Y, Lopes, R, Pellanda, L, Gottschall, C; Galougahi, KK	2016
Eldecalcitol improves chair-rising time in postmenopausal osteoporotic women treated with bisphosphonates	Iwamoto, , J; Sato, Y	2014
Treatment with high-dose n-3 PUFAs has no effect on platelet function, coagulation, metabolic status or inflammation in patients with atherosclerosis and type 2 diabetes	Poreba, , M, Mostowik, M, Siniarski, A, Golebiowska-Wiatrak, R, Malinowski, KP, Haberka, M, Konduracka, E, Nessler, J, Undas, A; Gajos, G	2017
Myocardial damage prevented by volatile anesthetics: a multicenter randomized controlled study	Guarracino, , F, Landoni, G, Tritapepe, L, Pompei, F, Leoni, A, Aletti, G, Scandroglio, AM, Maselli, D, De Luca, M, Marchetti, C, Crescenzi, G; Zangrillo, A	2006
Adalimumab improves treatment satisfaction with medication (TS-M) in patients with moderate to severe Hidradenitis Suppurativa (HS) in a 12-week randomised controlled trial (PIONEER I)	Jemec, , GB, Sundaram, M, Pinsky, B, Gu, Y, Williams, D; Bao, Y	2014
Safety and effectiveness of rituximab in patients with rheumatoid arthritis following an inadequate response to 1 prior tumor necrosis factor inhibitor: the RESET trial	Haraoui, , B, Bokarewa, M, Kallmeyer, I; Bykerk, VP	2011
Pharmacokinetic/pharmacodynamic relationships of FTY720 in kidney transplant recipients	Park, , SI, Felipe, CR, Machado, PG, Garcia, R, Skerjanec, A, Schmouder, R, Tedesco-Silva, H; Medina-Pestana, JO	2005
Effect of Interactive Neurostimulation Therapy on Inflammatory Response in Patients With Chronic and Recurrent Mechanical Neck Pain	Teodorczyk-Injeyan, , JA, Triano, JJ, McGregor, M, Woodhouse, L; Injeyan, HS	2015
The safety of autologous and metabolically fit bone marrow mesenchymal stromal cells in medically refractory Crohn's disease - a phase 1 trial with three doses		2016
A phase III study of infliximab and corticosteroids for the initial treatment of acute graft-versus-host disease	Couriel, , DR, Saliba, R, de Lima, M, Giralt, S, Andersson, B, Khouri, I, Hosing, C, Ippoliti, C, Shpall, EJ, Champlin, R; Alousi, A	2009
Immunomodulation in type 1 diabetes by NBI-6024, an altered peptide ligand of the insulin B epitope	Alleva, , DG, Maki, RA, Putnam, AL, Robinson, JM, Kipnes, MS, Dandona, P, Marks, JB, Simmons, DL, Greenbaum, CJ, Jimenez, RG, Conlon, PJ; Gottlieb, PA	2006
Prucalopride, a systemic enterokinetic, for the treatment of constipation	Emmanuel, , AV, Roy, AJ, Nicholls, TJ; Kamm, MA	2002
Calf raise exercise increases walking performance in patients with intermittent claudication	Van Schaardenburgh, , M, Wohlwend, M, Rognmo, Ø; Mattsson, E	2017
Transplantation of autologous bone marrow mononuclear cells on patients with idiopathic dilated cardiomyopathy: early results on effect and security	Huang, , RC, Yao, K, Li, YL, Zhang, YQ, Xu, SK, Shi, HY, Pan, CZ, Yang, S, Zhang, SH, Ge, L, Niu, YH, Zhang, F, Qian, JY, Zou, YZ; Ge, JB	2006
Effects of SPR periodontal basic therapy on cervical crevicular fluid C-reactive protein in patients with chronic periodontitis	Zhong, , ZQ; Fu, H	2018
Prospective randomized trial of heated humidified versus cold dry carbon dioxide insufflation during laparoscopic gastric bypass	Champion, , JK; Williams, M	2006
Healing response to non-surgical periodontal therapy in patients with diabetes mellitus: clinical, microbiological, and immunologic results	Christgau, , M, Palitzsch, KD, Schmalz, G, Kreiner, U; Frenzel, S	1998
Bezafibrate improves postprandial hypertriglyceridemia and associated endothelial dysfunction in patients with metabolic syndrome: a randomized crossover study	Ohno, , Y, Miyoshi, T, Noda, Y, Oe, H, Toh, N, Nakamura, K, Kohno, K, Morita, H; Ito, H	2014
Outcome of rheumatoid arthritis following adjunct statin therapy	Das, , S, Mohanty, M; Padhan, P	2015
Randomized clinical trial of folate supplementation in patients with peripheral arterial disease	Khandanpour, , N, Armon, MP, Jennings, B, Finglas, PM, Willis, G, Clark, A; Meyer, FJ	2009
Randomized, controlled trial of recombinant human interleukin-11 in patients with active Crohn's disease	Sands, , BE, Winston, BD, Salzberg, B, Safdi, M, Barish, C, Wruble, L, Wilkins, R, Shapiro, M; Schwertschlag, US	2002
Headache in the Emergency Department (ED) - A Multi-Center Research Network to Optimize the ED Treatment of Migraines	NCT00122278	2005
Bivalirudin inhibits periprocedural platelet function and tissue factor expression of human smooth muscle cells	Pepke, , W, Eisenreich, A, Jaster, M, Ayral, Y, Bobbert, P, Mayer, A, Schultheiss, HP; Rauch, U	2013
Long-term efficacy and safety of etanercept after readministration in patients with active ankylosing spondylitis	Brandt, , J, Listing, J, Haibel, H, Sörensen, H, Schwebig, A, Rudwaleit, M, Sieper, J; Braun, J	2005
Low dose dopamine infusion reduces renal tubular injury following cardiopulmonary bypass surgery	Sumeray, , M, Robertson, C, Lapsley, M, Bomanji, J, Norman, AG; Woolfson, RG	2001
Individualized Angiotensin-Converting Enzyme (ACE)-Inhibitor Therapy in Stable Coronary Artery Disease Based on Clinical and	Oemrawsingh, , RM, Akkerhuis, KM, Van Vark, LC, Redekop, WK, Rudez, G, Remme, WJ, Bertrand, ME, Fox, KM, Ferrari, R, Danser, AH, de Maat, M, Simoons, ML, Brugts, JJ; Boersma, E	2016

Pharmacogenetic Determinants: the PERindopril GENEtic (PERGENE) Risk Model		
Methotrexate in the Treatment of Axial Spondyloarthritis	NCT00298012	2006
Adalimumab: new preparation. Rheumatoid arthritis: no therapeutic advance		2004
Niclosamide as an adjuvant to etanercept in treatment patients with active rheumatoid arthritis: an 8-week randomized controlled pilot study	Al-Gareeb, , AIA, Gorial, FI; Mahmood, AS	2018
Efficacy of a novel PEGylated humanized anti-TNF fragment (CDP870) in patients with rheumatoid arthritis: a phase II double-blinded, randomized, dose-escalating trial	Choy, , EH, Hazleman, B, Smith, M, Moss, K, Lisi, L, Scott, DG, Patel, J, Sopwith, M; Isenberg, DA	2002
Safety and Efficacy of Bempedoic Acid to Reduce LDL Cholesterol	Ray, , KK, Bays, HE, Catapano, AL, Lalwani, ND, Bloedon, LT, Sterling, LR, Robinson, PL; Ballantyne, CM	2019
Warm induction cardioplegia and reperfusion dose influence the occurrence of the post CABG TnI level	Ji, , B, Liu, M, Lu, F, Liu, J, Wang, G, Feng, Z; Hu, Q	2006
The effect of exercise intensity on the response to exercise rehabilitation in patients with intermittent claudication	Gardner, , AW, Montgomery, PS, Flinn, WR; Katzel, LI	2005
Efficacy and safety of rilonacept (interleukin-1 Trap) in patients with cryopyrin-associated periodic syndromes: results from two sequential placebo-controlled studies	Hoffman, , HM, Throne, ML, Amar, NJ, Sebai, M, Kivitz, AJ, Kavanaugh, A, Weinstein, SP, Belomestnov, P, Yancopoulos, GD, Stahl, N; Mellis, SJ	2008
Interferon-alpha treatment induces delayed CD4 proliferative responses to the hepatitis C virus nonstructural protein 3 regardless of the outcome of therapy	Zhang, , ZX, Milich, DR, Peterson, DL, Birkett, A, Schvarcz, R, Weiland, O; Sällberg, M	1997
Lack of cardioprotective efficacy of allopurinol in coronary artery surgery	Taggart, , DP, Young, V, Hooper, J, Kemp, M, Walesby, R, Magee, P; Wright, JE	1994
Protocol for a randomised, placebo-controlled pilot study for assessing feasibility and efficacy of faecal microbiota transplantation in a paediatric ulcerative colitis population: pediFETCh trial	Pai, , N; Popov, J	2017
The role of IL-1 in postprandial fatigue	Lehrskov, , LL, Dorph, E, Widmer, AM, Hepprich, M, Siegenthaler, J, Timper, K; Donath, MY	2018
The FOsmetpantotenate Replacement Therapy (FORT) Randomized, Double-Blind, Placebo-Controlled Pivotal Trial: study Design and Development Methodology of a Novel Primary Efficacy Outcome in Patients With Pantothenate Kinase-Associated Neurodegeneration	Klopstock, , T, Escolar, ML, Marshall, RD, Perez-Duenas, B, Tuller, S, Videnovic, A; Greblikas, F	2019
Hydroxyethyl starch resuscitation downregulate pro-inflammatory cytokines in the early phase of severe acute pancreatitis: a retrospective study	Chen, , Q-J, Yang, Z-Y, Wang, C-Y, Dong, L-M, Zhang, Y-S, Xie, C, Chen, C-Z, Zhu, S-K, Yang, H-J, Wu, H-S; Yang, C	2016
Surfactant Protein-D: a sensitive predictor for efficiency of preoperative pulmonary rehabilitation	Huang, , J, Lai, Y, Gao, K, Wang, Y, Du, H, Su, J, Che, G; Zhou, X	2017
Rational molecular assessment and innovative drug selection (RAIDs): exome data from cervical cancer	Scholl, , S, Kamal, M, Berns, E, Balint, B, Kereszt, A, De Koning, L, Jeannot, E, Luscaprondof, W, Sibut, V, Hupe, P, Kenter, G, Samuels, S, Jordanova, K, Blanchet, S, Lafanachere, L; Billaud, M	2016
Effects of rosiglitazone vs metformin on circulating osteoclast and osteogenic precursor cells in postmenopausal women with type 2 diabetes mellitus	Rubin, , MR, Manavalan, JS, Agarwal, S, McMahon, DJ, Nino, A, Fitzpatrick, LA; Bilezikian, JP	2014
Weight loss and improvements in fatigue in systemic lupus erythematosus: a controlled trial of a low glycaemic index diet versus a calorie restricted diet in patients treated with corticosteroids	Davies, , RJ, Lomer, MC, Yeo, SI, Avloniti, K, Sangle, SR; D'Cruz, DP	2012
Progesterone Treatment of Blunt Traumatic Brain Injury	NCT00048646	2002
Comparative effects of hemodilutional anemia and transfusion during cardiopulmonary bypass on acute kidney injury: a prospective randomized study	Arıtürk, , C, Ozgen, ZS, Kilercik, M, Ulugöl, H, Ökten, EM, Aksu, U, Karabulut, H; Toraman, F	2015
Synbiotic therapy (Bifidobacterium longum/Synergy 1) initiates resolution of inflammation in patients with active ulcerative colitis: a randomised controlled pilot trial	Furrie, , E, Macfarlane, S, Kennedy, A, Cummings, JH, Walsh, SV, O'neil, DA; Macfarlane, GT	2005
Imiquimod applied topically: a novel immune response modifier	Tyring, S	2001
Effect of non-surgical periodontal therapy on superoxide dismutase levels in gingival tissues of chronic periodontitis patients: a clinical and spectophotometric analysis	Sukhtankar, , L, Kulloli, A, Kathariya, R; Shetty, S	2013
Prehabilitation in Colorectal Cancer	NCT03096951	2017
Targets of therapy of patients with congestive heart heart failure caused by ischemic heart disease and diabetes	Megreladze, , II, Gongadze, NV, Kezeli, TD, Kakabadze, KS, Mirziashvili, MG, Chapichadze, ZV; Sukoyan, GV	2013
Influence of Roux-en-Y Gastric Bypass on the Hepatocellular Function and Bile Flow of Obese Patients Assessed by Scintigraphy with DISIDA	Salgado Júnior, , W, Donadelli, CA, Dos Santos, JS; Nonino, CB	2016
Influence of food on the oral bioavailability of rupatadine tablets in healthy volunteers: a single-dose, randomized, open-label, two-way crossover study	Solans, , A, Carbó, ML, Peña, J, Nadal, T, Izquierdo, I; Merlos, M	2007
Rheumatoid arthritis secondary non-responders to TNF can attain an efficacious and safe response by switching to certolizumab pegol: a phase IV, randomised, multicentre, double-blind, 12-week study, followed by a 12-week open-label phase	Schiff, , MH, von Kempis, J, Goldblum, R, Tesser, JR; Mueller, RB	2014
Pretreatment intracerebral and peripheral blood immune responses in vietnamese adults with tuberculous meningitis: diagnostic value and relationship to disease severity and outcome	Simmons, , CP, Thwaites, GE, Quyen, NTH, Torok, E, Hoang, DM, Chau, TTH, Mai, PP, Lan, NTN, Dung, NH, Quy, HT, Bang, ND, Hien, TT; Farrar, J	2006

Remote Ischaemic PrEconditioning of Human Myocardium (RIPE): study protocol for a double-blinded randomised controlled trial	Deja, , MA, Wiaderkiewicz, R, Czekaj, P, Czech, E, Malinowski, M, Machej, L, Węglarzy, A, Kowalówka, A, Piekarska, M, Szurlej, B; Latusek, T	2018
Expanded allogeneic adipose-derived stem cells (eASCs) for the treatment of complex perianal fistula in Crohn's disease: results from a multicenter phase I/IIa clinical trial	De La Portilla, , F, Alba, F, Garcia-Olmo, D, Herrerias, JM, Gonzalez, FX; Galindo, A	2013
Docosahexaenoic Acid and Periodontitis in Adults: a Randomized Controlled Trial	Naqvi, , AZ, Hasturk, H, Mu, L, Phillips, RS, Davis, RB, Halem, S, Campos, H, Goodson, JM, Van Dyke, TE; Mukamal, KJ	2014
Efficacy and tolerability of combination therapy with valsartan plus hydrochlorothiazide compared with amlodipine monotherapy in hypertensive patients with other cardiovascular risk factors: the VAST study	Ruilope, , LM, Malacco, E, Khder, Y, Kandra, A, Bönner, G; Heintz, D	2005
Colonic acetate infusions promote fat oxidation and improve metabolic parameters in overweight males	Canfora, , EE, Van Der Beek, CM, Lenaerts, K, Troost, FJ, Holst, JJ, Masclee, AAM, Dejong, CHC; Blaak, EE	2014
Interleukin-1 blockade with anakinra to prevent adverse cardiac remodeling after acute myocardial infarction (Virginia Commonwealth University Anakinra Remodeling Trial Pilot study)	Abbate, , A, Kontos, MC, Grizzard, JD, Biondi-Zoccai, GG, Van Tassell, BW, Robati, R, Roach, LM, Arena, RA, Roberts, CS, Varma, A, Gelwix, CC, Salloum, FN, Hastillo, A, Dinarello, CA; Vetrovec, GW	2010
Asthma Clinical Research Network (ACRN)	NCT00000577	1999
THE LASER-AMI STUDY - Excimer Laser Versus Manual Thrombus Aspiration in Acute Myocardial Infarction	NCT01826006	2013
Long-term safety of anti-TNF agents on the liver of patients with spondyloarthritis and potential occult hepatitis B viral infection: an observational multicentre study	Giannitti, , C, Lopalco, G, Vitale, A, Rigante, D, Anelli, MG, Fabbroni, M, Manganelli, S, Galeazzi, M, Frediani, B, Barone, M, Lapadula, G, Iannone, F; Cantarini, L	2017
Comparative effects of 10-mg versus 80-mg Atorvastatin on high-sensitivity C-reactive protein in patients with stable coronary artery disease: results of the CAP (Comparative Atorvastatin Pleiotropic effects) study	Bonnet, , J, McPherson, R, Tedgui, A, Simoneau, D, Nozza, A, Martineau, P; Davignon, J	2008
Acetyl-coenzyme A carboxylase inhibition reduces de novo lipogenesis in overweight male subjects: a randomized, double-blind, crossover study	Stiede, , K, Miao, W, Blanchette, HS, Beysen, C, Harriman, G, Harwood, HJ, Kelley, H, Kapeller, R, Schmalbach, T; Westlin, WF	2017
The 'Switch' study protocol: a randomised-controlled trial of switching to an alternative tumour-necrosis factor (TNF)-inhibitor drug or abatacept or rituximab in patients with rheumatoid arthritis who have failed an initial TNF-inhibitor drug	Navarro Coy, , NC, Brown, S, Bosworth, A, Davies, CT, Emery, P, Everett, CC, Fernandez, C, Gray, JC, Hartley, S, Hulme, C, Keenan, AM, McCabe, C, Redmond, A, Reynolds, C, Scott, D, Sharples, LD, Pavitt, S; Buch, MH	2014
Myocardial injury following coronary artery surgery versus angioplasty (MICASA): a randomised trial using biochemical markers and cardiac magnetic resonance imaging	van Gaal, , WJ, Arnold, JR, Testa, L, Karamitsos, T, Lim, CC, Ponnuthurai, FA, Petersen, S, Francis, JM, Selvanayagam, J, Sayeed, R, West, N, Westaby, S, Neubauer, S; Banning, AP	2011
Effectiveness of Whole-body Hyperthermia by Mild Water-filtered Infrared A Radiation in Psoriatic Arthritis: a Controlled, Randomised, Prospective Trial	Lange, , U, Schwab, F, Muller-Ladner, U; Dischereit, G	2014
The clinical response and systemic effects of non-surgical periodontal therapy in end-stage renal disease patients: a 6-month randomized controlled clinical trial	Fang, , F, Wu, B, Qu, Q, Gao, J, Yan, W, Huang, X, Ma, D, Yue, J, Chen, T, Liu, F; Liu, Y	2015
Low-dose atrial natriuretic peptide for chronic kidney disease in coronary surgery	Yoshitake, , I, Sezai, A, Hata, M, Niino, T, Unosawa, S, Wakui, S; Shiono, M	2011
Calprotectin levels in gingival crevicular fluid predict disease activity in patients treated for generalized aggressive periodontitis	Kaner, , D, Bernimoulin, JP, Dietrich, T, Kleber, BM; Friedmann, A	2011
Hyper osmolality does not modulate natriuretic peptide concentration in patients after coronary artery surgery	Honkonen, , EL, Järvelä, K, Huhtala, H, Holm, P; Lindgren, L	2009
Membrane versus centrifuge-based therapeutic plasma exchange: a randomized prospective crossover study	Hafer, , C, Golla, P, Gericke, M, Eden, G, Beutel, G, Schmidt, JJ, Schmidt, BM, De Reys, S; Kielstein, JT	2016
IL-1 receptor antagonist reduces endotoxin-induced airway inflammation in healthy volunteers	Hernandez, , ML, Mills, K, Almond, M, Todoric, K, Aleman, MM, Zhang, H, Zhou, H; Peden, DB	2015
Late post-conditioning with sevoflurane after cardiac surgery - Are surrogate markers associated with clinical outcome?	Bonvini, , JM, Beck-Schimmer, B, Kuhn, SJ, Graber, SM, Neff, TA; Schlapfer, M	2015
Effects of inhaled tiotropium plus transdermal tulobuterol versus tiotropium alone on impulse oscillation system (IOS)-assessed measures of peripheral airway resistance and reactance, lung function and quality of life in patients with COPD: a randomized crossover study	Abe, , T, Setoguchi, Y, Kono, Y, Togashi, Y, Sugiyama, S, Tanakadate, M, Soeda, S, Nakai, M, Sugiyama, N, Fujiwara, A, Yamaguchi, K, Yamaguchi, A; Kurita, N	2011
Incidence and risk factors for serious infection in patients with rheumatoid arthritis treated with tumor necrosis factor inhibitors: a report from the registry of Japanese rheumatoid arthritis patients for longterm safety	Komano, , Y, Tanaka, M, Nanki, T, Koike, R, Sakai, R, Kameda, H, Nakajima, A, Saito, K, Takeno, M, Atsumi, T, Tohma, S, Ito, S, Tamura, N, Fujii, T, Sawada, T, Ida, H, Hashiramoto, A, Koike, T, Ishigatsubo, Y, Eguchi, K, Tanaka, Y, Takeuchi, T, Miyasaka, N; Harigai, M	2011
Efficacy of probiotic therapy on atopic dermatitis in children: a randomized, double-blind, placebo-controlled trial	Yang, , H-J, Min, TK, Lee, HW; Pyun, BY	2014
Evaluation of sleep quality in patients with ankylosing spondylitis and efficacy of anti-TNF-alpha therapy on sleep problems: a polisomnographic study		2018
Comparison of laparoscopically assisted vaginal hysterectomy with abdominal hysterectomy. Technique and results	Kunz, , G, Plath, T; Leyendecker, G	1996
Corticosteroids and Anti TNF in Methotrexate Inadequate Responder Rheumatoid Arthritis Patient	NCT01724268	2012
Infliximab Therapy in Patients With Refractory Polymyalgia Rheumatica	NCT01423591	2011

Aberrant expression and dysfunction of TLR2 and its soluble form in chronic HBV infection and its regulation by antiviral therapy	Huang, , Z, Ge, J, Pang, J, Liu, H, Chen, J, Liao, B, Huang, X, Zuo, D, Sun, J, Lu, M, Zhang, X; Hou, J	2015
Prognosis and risk factors in patients with asymptomatic aortic stenosis and their modulation by atorvastatin (20 mg)	Dichtl, , W, Alber, HF, Feuchtner, GM, Hintringer, F, Reinthaler, M, Bartel, T, Süssenbacher, A, Grander, W, Ulmer, H, Pachinger, O; Müller, S	2008
The effects of pentoxifylline administration on NFKB P50 transcription factor expression	Shamsara, , J, Behravan, J, Falsoleiman, H, Mohammadpour, AH; Ramezani, M	2012
Chinese herbal medicine (Ma Zi Ren Wan) for functional constipation: study protocol for a prospective, double-blinded, double-dummy, randomized controlled trial	Zhong, , LLD, Cheng, CW, Chan, Y, Chan, KH, Lam, TW, Chen, XR, Wong, CT, Wu, JCY; Bian, ZX	2013
Cost-Effectiveness of TNF-Blocker Injection Spacing for Patients with Established Rheumatoid Arthritis in Remission: an Economic Evaluation from the Spacing of TNF-Blocker Injections in Rheumatoid Arthritis Trial	Vanier, , A, Mariette, X, Tubach, F; Fautrel, B	2017
Human anti-tumor necrosis factor monoclonal antibody (adalimumab) in Crohn's disease: the CLASSIC-I trial	Hanauer, , SB, Sandborn, WJ, Rutgeerts, P, Fedorak, RN, Lukas, M, MacIntosh, D, Panaccione, R, Wolf, D; Pollack, P	2006
Predicting clinical outcomes in cancer patients based on a novel computational biology method of mapping the cancer mutanome	Drusbosky, , L, Martuscello, R, Abbasi, T, Vali, S; Cogle, CR	2016
Intensive statin therapy stabilizes C-reactive protein, but not chemokine in stable coronary artery disease treated with an everolimus-eluting stent	Sukegawa, , H, Maekawa, Y, Yuasa, S, Anzai, A, Kodaira, M, Takei, M, Sano, F, Ueda, I, Kawakami, T, Hayashida, K, Kohno, T, Kohsaka, S, Abe, T; Fukuda, K	2016
Acute normovolaemic haemodilution with crystalloids in coronary artery bypass graft surgery: a preliminary survey of haemostatic markers	Jalali, , A, Naseri, MH, Chalian, M; Dolatabadi, HL	2008
A randomised phase II study of interleukin-1 receptor antagonist in acute stroke patients	Emsley, , HC, Smith, CJ, Georgiou, RF, Vail, A, Hopkins, SJ, Rothwell, NJ; Tyrrell, PJ	2005
Systemic Probiotics in the Periodontal Treatment	NCT03733379	2018
Mid-term echocardiographic follow up of left ventricular function with permanent right ventricular pacing in pediatric patients with and without structural heart disease	Shalganov, , TN, Paprika, D, Vatasescu, R, Kardos, A, Mihalcz, A, Kornyei, L, Szatmari, A; Szili-Torok, T	2007
Micronized purified flavonoid fraction in pretreating CABG patients	Sirlak, , M, Akar, AR, Eryilmaz, S, Cetinkanat, EK, Ozcinar, E, Kaya, B, Elhan, AH; Ozyurda, U	2010
Successful lower extremity angioplasty improves brachial artery flow-mediated dilation in patients with peripheral arterial disease	Husmann, , M, Dörffler-Melly, J, Kalka, C, Diehm, N, Baumgartner, I; Silvestro, A	2008
lnterferon-alpha treatment induces delayed CD4 proliferative responses to the hepatitis C virus nonstructural protein 3 regardless of the outcome of therapy	Zhang, , Z-X, Milich, DR, Peterson, DL, Bitkett, A, Schvarcz, R; Weiland, O	1997
Efficacy of warm gutta-percha root canal filling in the treatment of dental pulpal and periapical diseases	Wang, , J, Du, Y, Deng, J, Wang, X; He, J	2019
The effect of Gluten Free Diet on Ulcerative Colitis	IRCT2015062113678N4	2016
The impact of two Models of Aerobic Training on Cognitive View, Body Composition and Metabolism of Young People with Obesity	RBR-7pdc8y	2018
A Phase IIb, Placebo-Controlled, Study to Optimize Diamyd® Therapy Administered into Lymph Nodes Combined with Vitamin D oral drops to Investigate the Impact on the Progression of Type 1 diabetes	EUCTR2017-001861-25-NL	2018
A RANDOMIZED, DOUBLE-BLIND, PARALLEL GROUP STUDY OF THE SAFETY AND THE EFFECT ON CLINICAL OUTCOME OF TOCILIZUMAB SC VERSUS PLACEBO SC IN COMBINATION WITH TRADITIONAL DISEASE MODIFYING ANTI-RHEUMATIC DRUGS (DMARDS) IN PATIENTS WITH MODERATE TO SEVERE ACTIVE RHEUMATOID ARTHRITIS	PER-028-11	2011
Measures of inflammation in low back pain and anti-TNFa (anti-inflammation treatment) for the treatment of deemed low back pain - an explorative study	EUCTR2014-005722-35-DK	2015
study, utilizing Zinc as enhancer of immune recovery and immune reconstitution in auto-transplant for multiple myeloma	EUCTR2014-004499-47-IT	2015
Albumin administration in the prevention of hepatorenal syndrome and death in patients with cirrhosis, bacterial infections other than spontaneous bacterial peritonitis and high risk of hospital mortality	EUCTR2013-002416-27-AT	2014
Effects of physical activity and anti-TNF-alpha disease control on lipid profile of rheumatoid arthritis patients	Cacciapaglia, , F, Iervolino, S, Anelli, MG, Mazzotta, D, Zincarelli, C, Pappone, N, Iannone, F; Lapadula, G	2015
A randomized, double-blind, placebo controlled, multicenter Phase II study to assess the efficacy and safety of Sorafenib added to standard treatment with Topotecan in patients with platinum-resistant recurrent ovarian cancer - TRIAS 2009	EUCTR2009-011922-33-DE	2009
Curcumin and hypertriglyceridemia	IRCT2015071314521N4	2017
The cost-effectiveness of abatacept, rituximab or anti-TNF alpha for patients with rheumatoid arthritis. - Dutch Rheumatoid Arthritis Monitoring (DREAM) Targetted Immune Modulator Evaluation (TIME)	EUCTR2008-005450-20-BE	2009
The study to confirm the effect of a food containing lactic acid bacteria on oral health : a randomized, double-blind, placebo controlled trial	JPRN-UMIN000028407	2018
A clinical study to evaluate the efficacy and safety of Morinidazole and Sodium Chloride Injection in the treatment of Acute biliary pancreatitis with infection	ChiCTR1900020552	2019
Treatment of chronic periodontitis decreases serum prohepcidin levels in patients with chronic kidney disease	Vilela, , EM, Bastos, JA, Fernandes, N, Ferreira, AP, Chaoubah, A; Bastos, MG	2011

Maternal, placental and cord blood cytokines and the risk of adverse birth outcomes among pregnant women infected with Schistosoma japonicum in the Philippines	Abioye, , AI, McDonald, EA, Park, S, Joshi, A, Kurtis, JD, Wu, H, Pond-Tor, S, Sharma, S, Ernerudh, J, Baltazar, P, Acosta, LP, Olveda, RM, Tallo, V; Friedman, JF	2019
Effect of sesamin supplementation in rheumatoid arthritis women	IRCT2013111715424N1	2014
A PHASE II RANDOMIZED, PLACEBO-CONTROLLED CLINICAL TRIAL TO STUDY THE SAFETY AND IMMUNOGENICITY OF V212 IN ADULT PATIENTS WITH AUTOIMMUNE DISEASE	PER-031-12	2012
Study conducted on humans in Europe to test the importance of a small molecule normally produced by the body, called Interleukin 2 (IL-2) to treat a specific type of diabetes (called type 1 diabetes)	EUCTR2014-002522-12-NL	2014
Effects of Cichorium Intybus L. Leaf Extract on improvement of the clinical status of patients with periodontal	IRCT2015112825274N1	2016
A MULTICENTER, RANDOMIZED, DOUBLE-BLIND, PLACEBO-CONTROLLED, PARALLEL-GROUP STUDY TO EVALUATE THE EFFICACY AND SAFETY OF JTE-051 ADMINISTERED FOR 12 WEEKS TO SUBJECTS WITH ACTIVE RHEUMATOID ARTHRITIS (MOVE-RA)	PER-046-15	2015
Extension study to evaluate the long term efficacy, safety and tolerability of secukinumab in patients with rheumatoid arthritis	EUCTR2013-000944-25-HU	2013
Immune reconstitution in severely immunosuppressed antiretroviral-naive HIV-1?infected patients (<100 CD4+ T cells/?L) taking antiretroviral regimens based on dolutegravir or ritonavir-boosted darunavir (the AdvanZ-4 Trial)	EUCTR2014-002281-70-ES	2014
Is peritoneal inflammation during peritoneal dialysis inhibited by drugs that block effects of substance P?	EUCTR2012-005063-28-SE	2012
Clinical phase IV study assessing the efficacy and safety of the medicinal product Groprinosin in treatment of recurrent airway inflammation in children with impaired cellular immunity and diagnosed or undiagnosed allergic disease	EUCTR2013-003303-18-PL	2014
Treating Idiopathic Inflammatory Myopathies Related Reduced Bone Mineral Density With Denosumab or Zoledronic Acid	NCT04034199	2019
Randomized Study of Nivolumab Combined With Ipilimumab Versus Pemetrexed and Cisplatin or Carboplatin as First Line Therapy in Unresectable Pleural Mesothelioma Patients	EUCTR2016-001859-43-GR	2016
The effect of fish oil on red blood cell function and walking distance in patients with arterial occlusion in the legs	EUCTR2013-004342-42-NL	2014
Biocompatibility of a new haemodialysis concentrate containing gluconic and citric acid (Honeydew) compared to acetic acid (SelectBagOne®) and citric acid (Honeycit)	ISRCTN60694941	2010
Prevention of bone loss following spinal cord injury	ISRCTN46977778	2004
A PHASE III RANDOMIZED CLINICAL TRIAL OF PEMBROLIZUMAB (MK-3475) VERSUS PACLITAXEL, DOCETAXEL OR VINFLUNINE IN SUBJECTS WITH RECURRENT OR PROGRESSIVE METASTATIC UROTHELIAL CANCER	PER-074-14	2015
Controlled, international study conducted in several centres on the safety and therapeutic effect of a Hydroxylethyl-starch (HES) solution versus an electrolyte solution in patients undergoing elective abdominal surgery	EUCTR2016-002162-30-NL	2017
A Randomized, Open-Label, Phase 3 Study to Assess the Efficacy and Safety of KRN23 Versus Oral Phosphate and Active Vitamin D Treatment in Pediatric Patients with X-linked Hypophosphatemia (XLH)	EUCTR2016-000600-29-IE	2016
A phase II study of Erlotinib and Bevacizumab combination therapy for previously treated III B / IV Stage non-squamous non-small cell lung cancer: IMSLC-001	JPRN-UMIN000011791	2013
Pragmatic, controlled, international study conducted in several centres on the safety and therapeutic effect of a Hydroxylethyl starch (HES) solution versus an electrolyte solution in trauma patients	EUCTR2016-002176-27-DE	2016
Impact of resveratrol supplementation in treatment of periodontal patients with type 2 diabetes mellitus	IRCT2015012420765N1	2015
This study is aimed at assessing the safety of SmofKabivenÂ® (to be registered in India) in comparison to Kabiven (already marketed in India) in patients with gastrointestinal disorders requiring parenteral nutrition	CTRI/2014/06/004672	2014
A Randomised, Double-blind, Placebo-controlled, Parallel Group, Multicentre, Phase II Study to Assess The Efficacy of AZD9056 (single oral 400 mg dose) when Administered for 4 Weeks in Patients with Moderate to Severe COPD	EUCTR2005-004110-32-SE	2005
A Phase 2 study to investigate the safety and efficacy of ABBV-105 given alone or in combination with ABT-494 (Upadacitinib) in patients with active rheumatoid arthritis who have failed prior treatment with biologic therapy	EUCTR2018-000666-10-HU	2018
A Clinical Trial to Look at the Effects of the Drug Bempedoic Acid in Patients with Frequent Major Cardiovascular Events, or at High Risk of Cardiovascular Disease who Cannot be Treated with Statins. Patients to be randomly allocated to either placebo or investigational drug; assignment will be unknown to patient and doctor	EUCTR2016-003486-26-GB	2017
DIFFERENTIATING THE MECHANISM OF ACTION OF ANTI TNF-alpha AGENTS - DATA Study	EUCTR2006-000363-28-GB	2006

A study examining the effect of etanercept on inflammation in the brain	ISRCTN12472821	2015
Pain mechanisms and fatigue in rheumatoid arthritis (RA) and healthy volunteers. Can antirheumatic and biological therapy affect pain processing and fatigue in RA? - RA PAIN PROCESSING	EUCTR2008-005212-40-SE	2008
APRICOT - Anakinra for pustular psoriasis	ISRCTN13127147	2016
Pilot Trial to preserve residual insulin secretion in children and adolescents with recent onset Type 1 diabetes by using GAD-antigen (Diamyd) therapy in combination with Vitamin D and Ibuprofen	EUCTR2012-003251-11-SE	2012
MULTICENTER STUDY WITH A 16-WEEK DOUBLE-BLIND, PLACEBO-CONTROLLED (DURING THE INITIAL 2 WEEKS) RANDOMIZED PERIOD, FOLLOWED BY A 24-WEEK OPEN LABEL EXTENSION TO ASSESS MAGNETIC RESONANCE IMAGE-VERIFIED EARLY RESPONSE TO CERTOLIZUMAB PEGOL IN SUBJECTS WITH ACTIVE RHEUMATOID ARTHRITIS	EUCTR2009-013758-33-SE	2010
A study to investigate the effectiveness of mechanical traction in treating people with lower back pain and sciatica caused by a herniated lumbar disc	ISRCTN15050704	2018
A three-part multicenter study, with a randomized, doubleblind, placebo controlled, withdrawal design in Part II to assess efficacy, safety, and tolerability of ACZ885 (antiinterleukin-1ß monoclonal antibody) in patients with Muckle-Wells Syndrome	EUCTR2006-005455-15-DE	2007
Efficacy and Safety of SAR339658 in Patients with Moderate to Severe Ulcerative Colitis	EUCTR2012-002013-19-DE	2013
AnakInRa Treatment of Recurrent Idiopathic Pericarditis	EUCTR2013-001849-13-IT	2016
Clinical trial to study the effects and safety of stem cells, derived from fat tissue from human donors, administered intravenously to treat patients with severe bacterial pneumonia acquired outside the hospital, nursing home or long-term care facilities	EUCTR2015-002994-39-NL	2015
The effects of a DPP4 inhibitor on endothelial function in diabetic patients with coronary artery disease	JPRN-UMIN000008806	2012
A Phase IIb, Placebo-Controlled, Study to Optimize Diamyd® Therapy Administered into Lymph Nodes Combined with Vitamin D oral drops to Investigate the Impact on the Progression of Type 1 diabetes	EUCTR2017-001861-25-SE	2017
Cytokines and Inflammatory Marker during Therapy in Major Depression with Celecoxib - CITICOX	EUCTR2009-011990-34-DE	2010
Comparing intervention levels for the support of blood pressure in extremely premature newborn babies	ISRCTN83507686	2012
Phase I with CIGB-814 in rheumatoid arthritis patients	RPCEC00000238	2017
A 12-week, double-blind, randomized, parallel-group, placebo-controlled study of 4 doses of VX-509 in subjects with active rheumatoid arthritis	EUCTR2009-017438-32-BE	2010
The Randomized, Double-Blind, Placebo-Controlled Study of Efficacy and Safety of Propolis and N-Acetylcysteine Compared to Placebo in Adults in Acute Condition with Sputum Production	ACTRN12617000344392	2017
Impact of probiotic supplementation in treatment of periodontal patients with type 2 diabetes mellitus	IRCT2016110430694N1	2017
A study examining the effect of etanercept (Enbrel)on inflammation in the brain	EUCTR2015-002145-63-GB	2016
Comparison of salivary IL-1 beta in moderate to severe chronic generalized periodontitis among healthy subjects and patients with type 2 diabetes	IRCT2014110916246N2	2014
A prospective, monocentric clinical trial with adalimumab for topical treatment of chronic wounds	EUCTR2009-015749-22-DE	2010
Cardiomyocyte apoptosis and ischemic preconditioning in open heart operations	Wu, , ZK, Laurikka, J, Saraste, A, Kytö, V, Pehkonen, EJ, Savunen, T; Tarkka, MR	2003
The effect of inhaled corticosteroids on bronchoalveolar lavage cells and IL-8 levels in stable COPD patients	Ozol, , D, Aysan, T, Solak, ZA, Mogulkoc, N, Veral, A; Sebik, F	2005
Fluoxetine for Maintenance of Remission and to Improve Quality of Life in Patients with Crohn's Disease: a Pilot Randomized Placebo-Controlled Trial	Mikocka-Walus, , A, Hughes, PA, Bampton, P, Gordon, A, Campaniello, MA, Mavrangelos, C, Stewart, BJ, Esterman, A; Andrews, JM	2017
A randomised controlled trial of two supervised exercise regimens and their impact on inflammatory burden in patients with intermittent claudication	Delaney, , CL; Spark, JI	2016
The Role of Biomarkers and Genetics in Peripheral Arterial Disease	McDermott, , MM; Lloyd-Jones, DM	2009
Antimicrobial photodynamic therapy combined with periodontal treatment for metabolic control in patients with type 2 diabetes mellitus: study protocol for a randomized controlled trial	Evangelista, , E, Franca, CM, Veni, P, de Oliveira Silva, T, Goncalves, RM, de Carvalho, FV, Deana, MA, Fernandes, KP, Mesquita-Ferrari, RA, Camacho, CP, Bussadori, KS, Alvarenga, HL; Prates, AR	2015
Effects of sitagliptin therapy on markers of low-grade inflammation and cell adhesion molecules in patients with type 2 diabetes	Tremblay, , AJ, Lamarche, B, Deacon, CF, Weisnagel, SJ; Couture, P	2014
Modifying immune response and outcome in ALS (MIROCALS): a novel trial design to test the efficacy and safety of low dose interleukin-2	Leigh, , PN; Bensimon, G	2016
Different effects of perindopril and enalapril on monocyte cytokine release in coronary artery disease patients with normal blood pressure	Krysiak, , R; Okopień, B	2012

Host-directed therapies for multidrug resistant tuberculosis	Zumla, , A; Maeurer, M	2016
Comparison between subcutaneous and intravenous interleukin-2 treatment in HIV disease	Witzke, , O, Winterhagen, T, Reinhardt, W, Heemann, U, Grosse-Wilde, H, Kreuzfelder, E, Roggendorf, M; Philipp, T	1998
Effect of long-term, low-dose clarithromycin on T helper 2 cytokines, eosinophilic cationic protein and the 'regulated on activation, normal T cell expressed and secreted' chemokine in the nasal secretions of patients with nasal polyposis	Peric, , A, Vojvodic, D; Matkovic-Jozin, S	2012
Effect of alpha-lipoic acid and vitamin E supplementation on oxidative stress, inflammation, and malnutrition in hemodialysis patients	Ahmadi, , A, Mazooji, N, Roozbeh, J, Mazloom, Z; Hasanzade, J	2013
Clinical outcome and IL-17, IL-23, IL-27 and FOXP3 expression in peripheral blood mononuclear cells of pollen-allergic children during sublingual immunotherapy	Nieminen, , K, Valovirta, E; Savolainen, J	2010
Clinical improvement and immunohistochemical findings in severe atopic dermatitis treated with interferon gamma	Jang, , IG, Yang, JK, Lee, HJ, Yi, JY, Kim, HO, Kim, CW; Kim, TY	2000
Effect of hydroxymethylglutaryl-CoA reductase inhibitors on low-density lipoprotein cholesterol, interleukin-6, and high-sensitivity C-reactive protein in end-stage renal disease	Soliemani, , A, Nikoueinejad, H, Tabatabaizade, M, Mianehsaz, E; Tamadon, M	2011
The combination of the selective cyclooxygenase-2 inhibitor celecoxib with weekly paclitaxel is a safe and active second-line therapy for non-small cell lung cancer: a phase II study with biological correlates	Gasparini, , G, Meo, S, Comella, G, Stani, SC, Mariani, L, Gamucci, T, Avallone, A, Lo Vullo, S, Mansueto, G, Bonginelli, P, Gattuso, D; Gion, M	2005
Impact of sodium nitrate on measures of vascular function and biomarkers of inflammation in patients with peripheral artery disease: a preliminary assessment	Norton, , SL, Ueda, K, Ganger IV, CT, Treichler, DP; Casey, DP	2016
Thermotherapy reduces blood pressure and circulating endothelin-1 concentration and enhances leg blood flow in patients with symptomatic peripheral artery disease	Neff, , D, Kuhlenhoelter, AM, Lin, C, Wong, BJ, Motaganahalli, RL; Roseguini, BT	2016
Adipocytokines, C-reactive protein, and cardiovascular disease: a population-based prospective study	Seven, , E, Husemoen, LL, Sehested, TS, Ibsen, H, Wachtell, K, Linneberg, A; Jeppesen, JL	2015
Vaccination of women with metastatic breast cancer, using a costimulatory gene (CD80)-modified, HLA-A2-matched, allogeneic, breast cancer cell line: clinical and immunological results	Dols, , A, Smith, JW, Meijer, SL, Fox, BA, Hu, HM, Walker, E, Rosenheim, S, Moudgil, T, Doran, T, Wood, W, Seligman, M, Alvord, WG, Schoof, D; Urba, WJ	2003
Recombinant human interleukin-2, recombinant human interferon alfa-2a, or both in metastatic renal-cell carcinoma. Groupe Français d'Immunothérapie	Negrier, , S, Escudier, B, Lasset, C, Douillard, JY, Savary, J, Chevreau, C, Ravaud, A, Mercatello, A, Peny, J, Mousseau, M, Philip, T; Tursz, T	1998
Effect of extra virgin olive oil on biomarkers of inflammation in HIV-infected patients: a randomized, crossover, controlled clinical trial	Dokmanovic, , SK, Kolovrat, K, Laskaj, R, Jukic, V, Vrkic, N; Begovac, J	2015
The addition of vildagliptin to metformin prevents the elevation of interleukin 1ß in patients with type 2 diabetes and coronary artery disease: a prospective, randomized, open-label study	Younis, , A, Eskenazi, D, Goldkorn, R, Leor, J, Naftali-Shani, N, Fisman, EZ, Tenenbaum, A, Goldenberg, I; Klempfner, R	2017
Lung cancer chemoprevention with celecoxib in former smokers	Mao, , JT, Roth, MD, Fishbein, MC, Aberle, DR, Zhang, ZF, Rao, JY, Tashkin, DP, Goodglick, L, Holmes, EC, Cameron, RB, Dubinett, SM, Elashoff, R, Szabo, E; Elashoff, D	2011
Short-term effects of Mediterranean-type diet intervention on soluble cellular adhesion molecules in subjects with abdominal obesity	Rallidis, , LS, Kolomvotsou, A, Lekakis, J, Farajian, P, Vamvakou, G, Dagres, N, Zolindaki, M, Efstathiou, S, Anastasiou-Nana, M; Zampelas, A	2017
Impact of resistance training in chronic obstructive pulmonary disease patients during periods of acute exacerbation	Borges, , RC; Carvalho, CR	2014
A randomized controlled pilot trial of valacyclovir for attenuating inflammation and immune activation in HIV/herpes simplex virus 2-coinfected adults on suppressive antiretroviral therapy	Yi, , TJ, Walmsley, S, Szadkowski, L, Raboud, J, Rajwans, N, Shannon, B, Kumar, S, Kain, KC, Kaul, R; Tan, DH	2013
Five years of Kawasaki disease in the Netherlands a national surveillance study	Tacke, , CE, Breunis, WB, Pereira, RR, Breur, JM, Kuipers, IM; Kuijpers, TW	2014
The LITE study: rationale and protocol for a randomized controlled trial of light therapy for cancer-related fatigue in cancer survivors	Johnson, , JA, Garland, SN, Carlson, LE, Savard, J, Simpson, JS, Ancoli-Israel, S; Campbell, TS	2016
Convalescent plasma treatment reduced mortality in patients with severe pandemic influenza A (H1N1) 2009 virus infection	Hung, , IFN, To, KKW, Lee, C-K, Lee, K-L, Chan, K, Yan, W-W, Liu, R, Watt, C-L, Chan, W-M, Lai, K-Y, Koo, C-K, Buckley, T, Chow, F-L, Wong, K-K, Chan, H-S, Ching, C-K, Tang, BSF, Lau, CCY, Li, IWS, Liu, S-H, Chan, K-H, Lin, C-K; Yuen, K-Y	2011
Epitope-Specific Immunotherapy Targeting CD4-Positive T Cells in Celiac Disease: safety, Pharmacokinetics, and Effects on Intestinal Histology and Plasma Cytokines with Escalating Dose Regimens of Nexvax2 in a Randomized, Double-Blind, Placebo-Controlled Phase 1 Study	Daveson, , AJM, Ee, HC, Andrews, JM, King, T, Goldstein, KE, Dzuris, JL, MacDougall, JA, Williams, LJ, Treohan, A, Cooreman, MP; Anderson, RP	2017
The effect of an antenatal lifestyle intervention in overweight and obese women on circulating cardiometabolic and inflammatory biomarkers: secondary analyses from the LIMIT randomised trial	Moran, , LJ, Fraser, LM, Sundernathan, T, Deussen, AR, Louise, J, Yelland, LN, Grivell, RM, Macpherson, A, Gillman, MW, Robinson, JS, Owens, JA; Dodd, JM	2017
Changes in Trypanosoma cruzi-specific immune responses after treatment: surrogate markers of treatment efficacy	Laucella, , SA, Mazliah, DP, Bertocchi, G, Alvarez, MG, Cooley, G, Viotti, R, Albareda, MC, Lococo, B, Postan, M, Armenti, A; Tarleton, RL	2009
Simple advice on lifestyle habits and long-term changes in biomarkers of inflammation and vascular adhesion in healthy middle-aged men	Sjogren, , P, Cederholm, T, Heimburger, M, Stenvinkel, P, Vedin, I, Palmblad, J; Hellenius, M-L	2010
Effects of glucan treatment on the Th1/Th2 balance in patients with allergic rhinitis: a double-blind placebo-controlled study	Kirmaz, , C, Bayrak, P, Yilmaz, O; Yuksel, H	2005
Effects of surgical periodontal therapy on serum levels of TNF-alpha in patients with chronic periodontitis	Yun, , F, Firkova, EI, Xun, H; Jun-Qi, L	2007

Phase II trial of everolimus in patients with previously treated recurrent or metastatic head and neck squamous cell carcinoma	Geiger, , JL, Bauman, JE, Gibson, MK, Gooding, WE, Varadarajan, P, Kotsakis, A, Martin, D, Gutkind, JS, Hedberg, ML, Grandis, JR; Argiris, A	2016
Three months of moderate-intensity exercise reduced plasma 3-nitrotyrosine in rheumatoid arthritis patients	Wadley, , AJ, Veldhuijzen van Zanten, JJ, Stavropoulos-Kalinoglou, A, Metsios, GS, Smith, JP, Kitas, GD; Aldred, S	2014
Double-blind randomized controlled clinical trial of the interleukin-6 receptor antagonist, tocilizumab, in European patients with rheumatoid arthritis who had an incomplete response to methotrexate	Maini, , RN, Taylor, PC, Szechinski, J, Pavelka, K, Bröll, J, Balint, G, Emery, P, Raemen, F, Petersen, J, Smolen, J, Thomson, D; Kishimoto, T	2006
Effects of aspirin on serum C-reactive protein and interleukin-6 levels in patients with type 2 diabetes without cardiovascular disease: a randomized placebo-controlled crossover trial	Hovens, , MM, Snoep, JD, Groeneveld, Y, Frölich, M, Tamsma, JT; Huisman, MV	2008
A randomized trial of sodium-restriction on kidney function, fluid volume and adipokines in CKD patients	Campbell, , KL, Johnson, DW, Bauer, JD, Hawley, CM, Isbel, NM, Stowasser, M, Whitehead, JP, Dimeski, G; McMahon, E	2014
Plasma fatty acid changes following consumption of dietary oils containing n-3, n-6, and n-9 fatty acids at different proportions: preliminary findings of the Canola Oil Multicenter Intervention Trial (COMIT)	Senanayake, , VK, Pu, S, Jenkins, DA, Lamarche, B, Kris-Etherton, PM, West, SG, Fleming, JA, Liu, X, McCrea, CE; Jones, PJ	2014
Antipruritic Effect of Acupuncture in Patients with Atopic Dermatitis: feasibility Study Protocol for a Randomised, Sham-Controlled Trial	Kim, , Y-K, Yeom, M, Kang, S, Park, H-J, Kim, K; Lee, H	2017
Anti-IL-5 recombinant humanized monoclonal antibody (mepolizumab) for the treatment of atopic dermatitis	Oldhoff, , JM, Darsow, U, Werfel, T, Katzer, K, Wulf, A, Laifaoui, J, Hijnen, DJ, Plötz, S, Knol, EF, Kapp, A, Bruijnzeel-Koomen, CA, Ring, J; de Bruin-Weller, MS	2005
Effect of nonsurgical periodontal therapy on serum and gingival crevicular fluid cytokine levels during pregnancy and postpartum	Fiorini, , T, Susin, C, da Rocha, JM, Weidlich, P, Vianna, P, Moreira, CH, Bogo Chies, JA, Rösing, CK; Oppermann, RV	2013
Low-Calorie Vegetarian Versus Mediterranean Diets for Reducing Body Weight and Improving Cardiovascular Risk Profile: CARDIVEG Study (Cardiovascular Prevention With Vegetarian Diet)	Sofi, , F, Dinu, M, Pagliai, G, Cesari, F, Gori, AM, Sereni, A, Becatti, M, Fiorillo, C, Marcucci, R; Casini, A	2018
Sirukumab in rheumatoid arthritis refractory to sulfasalazine or methotrexate: a randomized phase 3 safety and efficacy study in Japanese patients	Takeuchi, , T, Yamanaka, H, Harigai, M, Tamamura, R, Kato, Y, Ukyo, Y, Nakano, T, Hsu, B; Tanaka, Y	2018
A Phase IIb dose-ranging efficacy and safety study of tralokinumab in adult patients with moderate-to-severe atopic dermatitis (AD)	Wollenberg, , A, Howell, MD, Guttman-Yassky, E, Silverberg, JI, Birrell, C, Kell, C, Ranade, K, Dawson, M; Van Der Merwe, R	2018
Efficacy and safety of lebrikizumab in patients with uncontrolled asthma (LAVOLTA I and LAVOLTA II): replicate, phase 3, randomised, double-blind, placebo-controlled trials	Hanania, , NA, Korenblat, P, Chapman, KR, Bateman, ED, Kopecky, P, Paggiaro, P, Yokoyama, A, Olsson, J, Gray, S, Holweg, CT, Eisner, M, Asare, C, Fischer, SK, Peng, K, Putnam, WS; Matthews, JG	2016
Protective HLA class I alleles are associated with reduced immune activation and fibrinolysis in individuals with primary HIV infection	Hamlyn, , E, Hickling, S, Frater, J, Phillips, R, Babiker, A, McClure, M; Fidler, S	2010
Efficacy of subgingivally applied minocycline in the treatment of chronic periodontitis	Lu, , HK; Chei, CJ	2005
A genomic predictive signature for rigosertib in lower risk MDS derived by integrating clinical response, mechanism of action data and simulation	Fruchtman, , SM, Ali, AM, Petrone, ME, Zbyszewski, PS, Hoffman, B, Vali, S, Singh, NK, Usmani, S, Grover, H, Abbasi, T; Raza, A	2016
Qigong/Tai Chi Easy for fatigue in breast cancer survivors: rationale and design of a randomized clinical trial	Larkey, , L, Huberty, J, Pedersen, M; Weihs, K	2016
Adding salmeterol to an inhaled corticosteroid reduces allergen-induced serum IL-5 and peripheral blood eosinophils	Koopmans, , JG, Lutter, R, Jansen, HM; van der Zee, JS	2005
Influenza virus vaccination of patients with chronic lung disease	Gorse, , GJ, Otto, EE, Daughaday, CC, Newman, FK, Eickhoff, CS, Powers, DC; Lusk, RH	1997
Improvement Thresholds for Morning Stiffness Duration in Patients Receiving Delayed- Versus Immediate-Release Prednisone for Rheumatoid Arthritis	Buttgereit, , F, Kent, JD, Holt, RJ, Grahn, AY, Rice, P, Alten, R; Yazici, Y	2015
Immune Reconstitution in Severely Immunosuppressed Antiretroviral-Naive HIV-1-Infected Patients Starting Efavirenz, Lopinavir-Ritonavir, or Atazanavir-Ritonavir Plus Tenofovir/Emtricitabine: final 48-Week Results (The Advanz-3 Trial)	Miro, , JM, Manzardo, C, Ferrer, E, Loncà, M, Guardo, AC, Podzamczer, D, Domingo, P, Curran, A, Clotet, B, Cruceta, A, Lozano, F, Pérez, I, Plana, M; Gatell, JM	2015
Walnut Consumption Alters the Gastrointestinal Microbiota, Microbially Derived Secondary Bile Acids, and Health Markers in Healthy Adults: a Randomized Controlled Trial	Holscher, , HD, Guetterman, HM, Swanson, KS, An, R, Matthan, NR, Lichtenstein, AH, Novotny, JA; Baer, DJ	2018
Innovative Steroid Treatment to Reduce Asthma Development in Children After First-time Rhinovirus Induced Wheezing	NCT03889743	2019
Simvastatin Suppresses Airway IL-17 and Upregulates IL-10 in Patients With Stable COPD	Maneechotesuwan, , K, Wongkajornsilp, A, Adcock, IM; Barnes, PJ	2015
Effects of increased wholegrain consumption on immune and inflammatory markers in healthy low habitual wholegrain consumers	Ampatzoglou, , A, Williams, CL, Atwal, KK, Maidens, CM, Ross, AB, Thielecke, F, Jonnalagadda, SS, Kennedy, OB; Yaqoob, P	2016
Omega-3 fatty acids inhibit the up-regulation of endothelial chemokines in maintenance hemodialysis patients	Hung, , AM, Booker, C, Ellis, CD, Siew, ED, Graves, AJ, Shintani, A, Abumrad, NN, Himmelfarb, J; Ikizler, TA	2015
Genome-wide association analysis identifies CXCR4 gene as predictor of therapeutic response to teriparatide in severe osteoporosis	Alonso, , N, Albagha, OME, Azfer, A, Riches, P, Ostanek, B, Kocjan, T, Marc, J, Langdahl, BL; Ralston, SH	2018
Brief Report: pharmacodynamics, Safety, and Clinical Efficacy of AMG 811, a Human Anti-Interferon-gamma Antibody, in Patients With Discoid Lupus Erythematosus	Werth, , VP, Fiorentino, D, Sullivan, BA, Boedigheimer, MJ, Chiu, K, Wang, C, Arnold, GE, Damore, MA, Bigler, J, Welcher, AA, Russell, CB, Martin, DA; Chung, JB	2017
Effect of treatment of chronic periodontitis on levels of serum markers of acute-phase inflammatory and vascular responses	Ide, , M, McPartlin, D, Coward, PY, Crook, M, Lumb, P; Wilson, RF	2003
Benefits of micronized purified flavonoid fraction as adjuvant therapy on inflammatory response after sclerotherapy	Bogachev, , VY, Boldin, BV; Lobanov, VN	2018

Adiponectin, interleukin-6, monocyte chemoattractant protein-1, and regional fat mass during 12-month randomized treatment with metformin and/or oral contraceptives in polycystic ovary syndrome	Glintborg, , D, Mumm, H, Altinok, ML, Richelsen, B, Bruun, JM; Andersen, M	2014
N-acetylcysteine administration prevents nonthyroidal illness syndrome in patients with acute myocardial infarction: a randomized clinical trial	Vidart, , J, Wajner, SM, Leite, RS, Manica, A, Schaan, BD, Larsen, PR; Maia, AL	2014
Serial urinary IL-2, IL-6, IL-8, TNFalpha, UBC, CYFRA 21-1 and NMP22 during follow-up of patients with bladder cancer receiving intravesical BCG	Sanchez-Carbayo, , M, Urrutia, M, Romani, R, Herrero, M, Gonzalez de Buitrago, JM; Navajo, JA	2001
Symptom modifying effect of S-adenosil l-methionin in patients with diabetes-associated knee osteoarthritis: open randomized cross-over study	Trifonova, , E, Shirinsky, I, Sazonova, O, Shirinsky, V; Kalinovskaya, N	2015
Lipid profiles and inflammatory markers after periodontal treatment in children with congenital heart disease and at risk for atherosclerosis	Bresolin, , AC, Pronsatti, MM, Pasqualotto, LN, Nassar, PO, Jorge, AS, da Silva, EA; Nassar, CA	2013
Influence of acarbose on postprandial dysmetabolism: results of an open-label randomized study	Cherniak, , AY, Petrov, IM; Medvedeva, IV	2013
Lack of additional effects of long-term, low-dose clarithromycin combined treatment compared with topical steroids alone for chronic rhinosinusitis in China: a randomized, controlled trial	Deng, , J, Chen, F, Lai, Y, Luo, Q, Xu, R, Ou, C, Fu, Q; Shi, J	2018
The activity of nonspecific inflammation in hypertensive patients	Oshchepkova, , EV, Dmitriev, VA, Titiov, VN, Rogoza, AN; Masenko, VP	2007
Effect of anakinra on recurrent pericarditis among patients with colchicine resistance and corticosteroid dependence: the AIRTRIP randomized clinical trial	Brucato, , A, Imazio, M, Gattorno, M, Lazaros, G, Maestroni, S, Carraro, M, Finetti, M, Cumetti, D, Carobbio, A, Ruperto, N, Marcolongo, R, Lorini, M, Rimini, A, Valenti, A, Erre, GL, Sormani, MP, Belli, R, Gaita, F; Martini, A	2016
Pharmacodynamics and predictive biomarkers in patients treated with atacicept: data from the APRIL-SLE trial	Isenberg, , DA, Wofsy, D, Li, Y, Licu, D, Wax, SD, Gordon, C; Rossi, CP	2013
Intravenous anti-IL-13 mAb QAX576 for the treatment of eosinophilic esophagitis	Rothenberg, , ME, Wen, T, Greenberg, A, Alpan, O, Enav, B, Hirano, I, Nadeau, K, Kaiser, S, Peters, T, Perez, A, Jones, I, Arm, JP, Strieter, RM, Sabo, R; Gunawardena, KA	2015
Lebrikizumab idiopathic pulmonary fibrosis trial: a phase II randomized, double-blind, placebo controlled study to assess efficacy and safety (RIFF)	Scalori, , A, Belloni, P, Ackrill, A, Kapugampola, L, Doyle, R; Kaminski, J	2014
Analysis of177Lu-DOTA-octreotate therapy-induced DNA damage in peripheral blood lymphocytes of patients with neuroendocrine tumors	Denoyer, , D, Lobachevsky, P, Jackson, P, Thompson, M, Martin, OA; Hicks, RJ	2015
Cognitive-behavioral therapy for sleep disturbance in patients undergoing peritoneal dialysis: a pilot randomized controlled trial	Chen, , HY, Chiang, CK, Wang, HH, Hung, KY, Lee, YJ, Peng, YS, Wu, KD; Tsai, TJ	2008
EASI P-EASI: utilizing a combination of serum biomarkers offers an objective measurement tool for disease severity in patients with atopic dermatitis	Thijs, , JL, Drylewicz, J, Fiechter, R, Strickland, I, Sleeman, MA, Herath, A, May, RD, Bruijnzeel-Koomen, CAFM, Knol, EF, Giovannone, B, De Bruin-Weller, S, Nierkens, S; Hijnen, DJ	2018
Effect of short-term folic acid supplementation on insulin sensitivity and inflammatory markers in overweight subjects	Solini, , A, Santini, E; Ferrannini, E	2006
The effects of lebrikizumab in patients with mild asthma following whole lung allergen challenge	Scheerens, , H, Arron, JR, Zheng, Y, Putnam, WS, Erickson, RW, Choy, DF, Harris, JM, Lee, J, Jarjour, NN; Matthews, JG	2014
Cilostazol reduces the progression of carotid intima-media thickness without increasing the risk of bleeding in patients with acute coronary syndrome during a 2-year follow-up	Ahn, , CM, Hong, SJ, Park, JH, Kim, JS; Lim, DS	2011
Inhaled salmeterol and albuterol in asthmatic patients receiving high-dose inhaled corticosteroids	Lai, , CK, Chan, CH, Ho, SS, Hui, AC; Lai, KN	1995
Gemcitabine, oxaliplatin, levofolinate, 5-fluorouracil, granulocyte-macrophage colony-stimulating factor, and interleukin-2 (GOLFIG) versus FOLFOX chemotherapy in metastatic colorectal cancer patients: the GOLFIG-2 multicentric open-label randomized phase III trial	Correale, , P, Botta, C, Rotundo, MS, Guglielmo, A, Conca, R, Licchetta, A, Pastina, P, Bestoso, E, Ciliberto, D, Cusi, MG, Fioravanti, A, Guidelli, GM, Bianco, MT, Misso, G, Martino, E, Caraglia, M, Tassone, P, Mini, E, Mantovani, G, Ridolfi, R, Pirtoli, L; Tagliaferri, P	2014
Role of vitamin D in flare ups of rheumatoid arthritis	Dehghan, , A, Rahimpour, S, Soleymani-Salehabadi, H; Owlia, MB	2014
Effects of Liraglutide on Reperfusion Injury in Patients With ST-Segment-Elevation Myocardial Infarction	Chen, , WR, Chen, YD, Tian, F, Yang, N, Cheng, LQ, Hu, SY, Wang, J, Yang, JJ, Wang, SF; Gu, XF	2016
Influence of Kneipp-hydrotherapy on immunoregulation	Kreutzfeldt, , A, Albrecht, B; Muller, K	2003
Modulation of neutrophil and inflammation markers in chronic obstructive pulmonary disease by short-term azithromycin treatment	Parnham, , MJ, Culić, O, Eraković, V, Munić, V, Popović-Grle, S, Barisić, K, Bosnar, M, Brajsa, K, Cepelak, I, Cuzić, S, Glojnarić, I, Manojlović, Z, Novak-Mircetić, R, Oresković, K, Pavicić-Beljak, V, Radosević, S; Sucić, M	2005
Logistical challenges and design considerations for studies using acute anterior cruciate ligament injury as a potential model for early posttraumatic osteoarthritis	Lattermann, , C, Jacobs, CA, Bunnell, MP, Jochimsen, KN, Abt, JP, Reinke, EK, Gammon, LG, Huebner, JL, Kraus, VB; Spindler, KP	2017
Safety and efficacy of subcutaneous tocilizumab in early systemic sclerosis: results from the open-label period of the fasscinate trial	Khanna, , D, Denton, CP, Jahreis, A, Van Laar, JM, Burke, L, Spotswood, H, Lin, CJ, Pope, JE, Allanore, Y, Muller-Ladner, U, Siegel, J; Furst, DE	2016
Effect of fermented milk prepared with two probiotic strains on Japanese cedar pollinosis in a double-blind placebo-controlled clinical study	Kawase, , M, He, F, Kubota, A, Hiramatsu, M, Saito, H, Ishii, T, Yasueda, H; Akiyama, K	2009
Myeloma cast nephropathy	Bridoux, , F, Hutchison, CA; Leung, N	2011
Efficacy and safety of a probiotic preparation in the secondary prophylaxis of hepatic encephalopathy in cirrhotic patients: final results of a double blind, randomized, placebo controlled study	Dhiman, , RK, Rana, BS, Agrawal, S, Garg, A, Chopra, M, Thumburu, KK, Khattri, A, Malhotra, S, Duseja, AK; Chawla, YK	2014

Potential therapeutic impact of omega-3 long chain-polyunsaturated fatty acids on inflammation markers in Duchenne muscular dystrophy: a double-blind, controlled randomized trial	Rodriguez-Cruz, , M, Cruz-Guzman, ODR, Almeida-Becerril, T, Solis-Serna, AD, Atilano-Miguel, S, Sanchez-Gonzalez, JR, Barbosa-Cortes, L, Ruiz-Cruz, ED, Huicochea, JC, Cardenas-Conejo, A, Escobar-Cedillo, RE, Yam-Ontiveros, CA; Ricardez-Marcial, EF	2018
Procalcitonin guided antibiotic therapy of acute exacerbations of asthma: a randomized controlled trial	Tang, , J, Long, W, Yan, L, Zhang, Y, Xie, J, Lu, G; Yang, C	2013
Aspirin desensitization for patients with aspirin-exacerbated respiratory disease: a randomized double-blind placebo-controlled trial	Esmaeilzadeh, , H, Nabavi, M, Aryan, Z, Arshi, S, Bemanian, MH, Fallahpour, M; Mortazavi, N	2015
Human intervention study to assess the effects of supplementation with olive leaf extract on peripheral blood mononuclear cell gene expression	Boss, , A, Kao, CH-J, Murray, PM, Marlow, G, Barnett, MPG; Ferguson, LR	2016
The effect of lornoxicam on TLR2 and TLR4 messenger RNA expression and tumor necrosis factor-α, interleukin-6, and interleukin-8 secretion in patients with systemic complications of acute pancreatitis	Gorsky, , VA, Agapov, MA, Khoreva, MV; Leonenko, IV	2015
Gingyo-san enhances immunity and potentiates infectious bursal disease vaccination	Chen, , C-M, Hung, C-M, Yeh, C-C, Chong, K-Y, Chen, H-L, Chen, J-Y, Kao, S-T, Yen, C-C, Yeh, M-H; Lin, M-S	2011
A comparison of continuous intravenous insulin and subcutaneous insulin among patients with type 2 diabetes and congestive heart failure exacerbation	Dungan, , KM, Osei, K, Gaillard, T, Moore, J; Binkley, P	2015
Effects of a Spirulina-based dietary supplement on cytokine production from allergic rhinitis patients	Mao, , TK, Van de Water, J; Gershwin, ME	2005
Protective effect of atorvastatin on acute systemic inflammation-induced endothelial dysfunction in hypercholesterolaemic subjects	Vlachopoulos, , C, Aznaouridis, K, Dagre, A, Vasiliadou, C, Masoura, C, Stefanadi, E, Skoumas, J, Pitsavos, C; Stefanadis, C	2007
Monocyte-suppressing effects of simvastatin in patients with isolated hypertriglyceridemia	Krysiak, , R; Okopien, B	2013
Combination of maintenance hemodialysis with hemoperfusion: a safe and effective model of artificial kidney	Chen, , SJ, Jiang, GR, Shan, JP, Lu, W, Huang, HD, Ji, G, Wu, P, Wu, GF, Wang, W, Zhu, C; Bian, F	2011
Vitamin D supplementation improves cytokine profiles in patients with congestive heart failure: a double-blind, randomized, placebo-controlled trial	Schleithoff, , SS, Zittermann, A, Tenderich, G, Berthold, HK, Stehle, P; Koerfer, R	2006
Dose-dependent interleukin-3 stimulation of thrombopoiesis and neutropoiesis in patients with small-cell lung carcinoma before and following chemotherapy: a placebo-controlled randomized phase Ib study	D'Hondt, , V, Weynants, P, Humblet, Y, Guillaume, T, Canon, JL, Beauduin, M, Duprez, P, Longueville, J, Müll, R; Chatelain, C	1993
Supervised oral protein supplementation during dialysis in patients with elevated C-reactive protein levels: a two phase, longitudinal, single center, open labeled study Clinical Research	Beddhu, , S, Filipowicz, R, Chen, X, Neilson, JL, Wei, G, Huang, Y; Greene, T	2015
Modulation of peripheral immune responses by paclitaxel-ifosfamide-cisplatin chemotherapy in advanced non-small-cell lung cancer	Koufos, , N, Michailidou, D, Xynos, ID, Tomos, P, Athanasiadou, K, Kosmas, C; Tsavaris, N	2013
Effect of atorvastatin on circulating proinflammatory T-lymphocyte subsets and soluble CD40 ligand in patients with stable coronary artery disease–a randomized, placebo-controlled study	Alber, , HF, Frick, M, Suessenbacher, A, Doerler, J, Schirmer, M, Stocker, EM, Dichtl, W, Pachinger, O; Weidinger, F	2006
Peripheral Biomarkers of Parkinson's Disease Progression and Pioglitazone Effects	Simon, , DK, Simuni, T, Elm, J, Clark-Matott, J, Graebner, AK, Baker, L, Dunlop, SR, Emborg, M, Kamp, C, Morgan, JC, Ross, GW, Sharma, S; Ravina, B	2015
Immune regulatory effects of high dose vitamin D3 supplementation in a randomized controlled trial in relapsing remitting multiple sclerosis patients receiving IFNbeta; the SOLARIUM study	Muris, , A-H, Smolders, J, Rolf, L, Thewissen, M, Hupperts, R; Damoiseaux, J	2016
Prospective study of neurological responses to treatment with macrophage-targeted glucocerebrosidase in patients with type 3 Gaucher's disease	Schiffmann, , R, Heyes, MP, Aerts, JM, Dambrosia, JM, Patterson, MC, DeGraba, T, Parker, CC, Zirzow, GC, Oliver, K, Tedeschi, G, Brady, RO; Barton, NW	1997
Effects of short-term therapy with glibenclamide and repaglinide on incretin hormones and oxidative damage associated with postprandial hyperglycaemia in people with type 2 diabetes mellitus	Stephens, , JW, Bodvarsdottir, TB, Wareham, K, Prior, SL, Bracken, RM, Lowe, GD, Rumley, A, Dunseath, G, Luzio, S, Deacon, CF, Holst, JJ; Bain, SC	2011
A randomized placebo controlled trial of aspirin effects on immune activation in chronically human immunodeficiency virus-infected adults on virologically suppressive antiretroviral therapy	O'Brien, , MP, Hunt, PW, Kitch, DW, Klingman, K, Stein, JH, Funderburg, NT, Berger, JS, Tebas, P, Clagett, B, Moisi, D, Utay, NS, Aweeka, F; Aberg, JA	2017
Effects of periodontal therapy during pregnancy on periodontal status, biologic parameters, and pregnancy outcomes: a pilot study	Offenbacher, , S, Lin, D, Strauss, R, McKaig, R, Irving, J, Barros, SP, Moss, K, Barrow, DA, Hefti, A; Beck, JD	2006
Induction of interleukin-10 and suppressor of cytokine signalling-3 gene expression following peptide immunotherapy	Tarzi, , M, Klunker, S, Texier, C, Verhoef, A, Stapel, SO, Akdis, CA, Maillere, B, Kay, AB; Larché, M	2006
Effects of whole grain rye, with and without resistant starch type 2 supplementation, on glucose tolerance, gut hormones, inflammation and appetite regulation in an 11-14.5 hour perspective; a randomized controlled study in healthy subjects	Sandberg, , JC, Björck, IME; Nilsson, AC	2017
Rosuvastatin might have an effect on C-reactive protein but not on rheumatoid disease activity: tayside randomized controlled study	Kumar, , P, Kennedy, G, Khan, F, Pullar, T; Belch, JJ	2012
No effect of homocysteine-lowering therapy on vascular inflammation and haemostasis in peripheral arterial occlusive disease	Schernthaner, , GH, Plank, C, Minar, E, Bieglmayer, C, Koppensteiner, R; Schernthaner, G	2006
Randomized, double blind controlled trial of subcutaneous recombinant human interleukin-11 versus prednisolone in active Crohn's disease	Herrlinger, , KR, Witthoeft, T, Raedler, A, Bokemeyer, B, Krummenerl, T, Schulzke, JD, Boerner, N, Kueppers, B, Emmrich, J, Mescheder, A, Schwertschlag, U, Shapiro, M; Stange, EF	2006
Long-term effects of angiotensin II blockade with irbesartan on inflammatory markers in hemodialysis patients: a randomized double blind placebo controlled trial (SAFIR study)	Peters, , CD, Kjaergaard, KD, Nielsen, CH, Christensen, KL, Bibby, BM, Jensen, JD; Jespersen, B	2017
Recurrent pericarditis in children and adolescents: a multicentre cohort study		2016

Sustained remission of symptoms and improved health-related quality of life in patients with cryopyrin-associated periodic syndrome treated with canakinumab: results of a double-blind placebo-controlled randomized withdrawal study	Koné-Paut, , I, Lachmann, HJ, Kuemmerle-Deschner, JB, Hachulla, E, Leslie, KS, Mouy, R, Ferreira, A, Lheritier, K, Patel, N, Preiss, R; Hawkins, PN	2011
Neither moxifloxacin nor cefuroxime produces significant attenuation of inflammatory mediator release in patients exposed to cardiopulmonary bypass: a randomized controlled trial	Wiesner, , G, Braun, SL, Gruber, M, Gertler, R, Lange, R, Tassani, P; Martin, K	2012
Reduced Levels of D-dimer and Changes in Gut Microbiota Composition After Probiotic Intervention in HIV-Infected Individuals on Stable ART	Stiksrud, , B, Nowak, P, Nwosu, FC, Kvale, D, Thalme, A, Sonnerborg, A, Ueland, PM, Holm, K, Birkeland, SE, Dahm, AE, Sandset, PM, Rudi, K, Hov, JR, Dyrhol-Riise, AM; Trøseid, M	2015
Intradialytic exercise training reduces oxidative stress and epicardial fat: a pilot study	Wilund, , KR, Tomayko, EJ, Wu, PT, Ryong Chung, H, Vallurupalli, S, Lakshminarayanan, B; Fernhall, B	2010
Therapy of type 1 diabetes with CD4+CD25highCD127-regulatory T cells prolongs survival of pancreatic islets - Results of one year follow-up	Marek-Trzonkowska, , N, Mysliwiec, M, Dobyszuk, A, Grabowska, M, Derkowska, I, Juscinska, J, Owczuk, R, Szadkowska, A, Witkowski, P, Mlynarski, W, Jarosz-Chobot, P, Bossowski, A, Siebert, J; Trzonkowski, P	2014
Rosuvastatin and fenofibrate in patients with diabetes and low high density lipoprotein cholesterol: comparison of changes of lipid levels and some markers of inflammation	Polenova, , NV, Vaulin, NA, Masenko, VP, Iavelov, IS; Gratsianskiĭ, NA	2009
Dupilumab progressively improves systemic and cutaneous abnormalities in patients with atopic dermatitis	Guttman-Yassky, , E, Bissonnette, R, Ungar, B, Suarez-Farinas, M, Ardeleanu, M, Esaki, H, Suprun, M, Estrada, Y, Xu, H, Peng, X, Silverberg, JI, Menter, A, Krueger, JG, Zhang, R, Chaudhry, U, Swanson, B, Graham, NMH, Pirozzi, G, Yancopoulos, GD; D Hamilton JD	2019
Sustainable Efficacy of Switching From Intravenous to Subcutaneous Tocilizumab Monotherapy in Patients With Rheumatoid Arthritis	Ogata, , A, Atsumi, T, Fukuda, T, Hirabayashi, Y, Inaba, M, Ishiguro, N, Kai, M, Kawabata, D, Kida, D, Kohsaka, H, Matsumura, R, Minota, S, Mukai, M, Sumida, T, Takasugi, K, Tamaki, S, Takeuchi, T, Ueda, A, Yamamoto, K, Yamanaka, H, Yoshifuji, H; Nomura, A	2015
A placebo-controlled, multicenter, double-blind, randomised, pharmacokinetic and pharmacodynamic trial of emricasan (IDN-6556) in subjects with acute-on chronic liver failure (ACLF)	Jalan, , R, Wright, G, McPherson, S, Frenette, C, Cave, M, Morris, M, Huyghe, M; Burgess, G	2015
Phase ii study of digitoxin for CF	Zeitlin, , P, Diener-West, M, Callahan, KA, Pollard, B, Lechtzin, N; Boyle, MP	2015
The Influence of Chronic Inflammation on Peripheral Motor Nerve Conduction Following Spinal Cord Injury: a Randomized Clinical Trial	Allison, , DJ, Gabriel, DA, Klentrou, P, Josse, AR; Ditor, DS	2017
A phase 2b dose-ranging efficacy and safety study of tralokinumab in adult patients with moderate to severe atopic dermatitis (AD)	Wollenberg, , A, Howell, MD, Guttman-Yassky, E, Silverberg, JI, Birrell, C, Kell, C, Dawson, M; Van Der Merwe, R	2017
Comparison of outcome of allogeneic bone marrow transplantation with and without granulocyte colony-stimulating factor (lenograstim) donor-marrow priming in patients with chronic myelogenous leukemia	Ji, , SQ, Chen, HR, Wang, HX, Yan, HM, Pan, SP; Xun, CQ	2002
Neurologic manifestations of the cryopyrin-associated periodic syndrome	Kitley, , JL, Lachmann, HJ, Pinto, A; Ginsberg, L	2010
Safety and Efficacy of Anakinra in Severe Hidradenitis Suppurativa: a Randomized Clinical Trial	Tzanetakou, , V, Kanni, T, Giatrakou, S, Katoulis, A, Papadavid, E, Netea, MG, Dinarello, CA, van der Meer, JWM, Rigopoulos, D; Giamarellos-Bourboulis, EJ	2016
Neutrophil-related gene expression and low-density granulocytes associated with disease activity and response to treatment in ANCA-associated vasculitis	Grayson, , P, Carmona-Rivera, C, Xu, L, Lim, N, Gao, Z, Asare, A, Specks, U, Stone, J, Seo, P, Spiera, R, Langford, C, Kallenberg, C, St Clair, E, Tchao, N, Ytterberg, S, Merkel, P, Kaplan, M; Monach, P	2015
A randomized, clinical trial to evaluate two therapies, mycophenolate and rilonacept, in subjects with biopsy-proven alcoholic hepatitis	Morgan, T	2014
Long-term clarithromycin in cystic fibrosis: effects on inflammatory markers in BAL and clinical status	Doğru, , D, Dalgiç, F, Kiper, N, Ozçelik, U, Yalçin, E, Aslan, AT, Gürcan, N, Saricaoğlu, F, Gür, D, Karayazgan, Y; Firat, P	2009
Resistance training in clinical, inflammatory process control in patients with moderate, severe and difficult control asthma	RBR-4tbd4p	2012
Evaluation of Perindopril and Telmisartan for the Treatment of Nonalcoholic Fatty Liver Disease: a Randomized controlled trial	ChiCTR-TRC-14005028	2014
Effect of punica granatum extract gel on gingival crevicular fluid levels of interleukin-1β, interleukin-8 and CCL28 levels: randomised controlled clinical trial	Prakash, , J, Bhatnagar, V, Nath, S, Pulikkotil, S; Prajapati, VK	2017
Lactobacillus and bifidobacterium in irritable bowel syndrome: symptom responses and relationship to cytokine profiles	O'Mahony, , L, McCarthy, J, Kelly, P, Hurley, G, Luo, F, Chen, K, O'Sullivan, GC, Kiely, B, Collins, JK, Shanahan, F; Quigley, EM	2005
Effects of Melatonin on clinical manifestations, inflammation and sleep quality in patients with Rheumatoid Arthritis	RBR-8pjqh2	2018
Parenteral nutrition providing a restricted amount of linoleic acid in severely burned patients: a randomised double-blind study of an olive oil-based lipid emulsion v. medium/long-chain triacylglycerols	García-de-Lorenzo, , A, Denia, R, Atlan, P, Martinez-Ratero, S, Le Brun, A, Evard, D; Bereziat, G	2005
A randomized, double-masked, placebo-controlled trial of recombinant granulocyte colony-stimulating factor administration to preterm infants with the clinical diagnosis of early-onset sepsis	Miura, , E, Procianoy, RS, Bittar, C, Miura, CS, Miura, MS, Mello, C; Christensen, RD	2001
Evaluating the effect of Radio frequency and Cavitation on Biochemical Markers and Body Contouring in Overweight People	IRCT2014042817475N1	2014
Inflammatory markers in orthodontic treatment with clear aligners: split mouth clinical trial	ISRCTN32624758	2017
Nox 1 and 4 inhibition in type 1 diabetic kidney disease	ACTRN12617001187336	2017

Tralokinumab for severe, uncontrolled asthma (STRATOS 1 and STRATOS 2): two randomised, double-blind, placebo-controlled, phase 3 clinical trials	Panettieri, , RA, Sjobring, U, Peterffy, A, Wessman, P, Bowen, K, Piper, E, Colice, G; Brightling, CE	2018
A long-term extension study to investigate the safety and effectiveness of Tildrakizumab in patients with Psoriatic Arthritis and Ankylosing Spondylitis or Non-Radiographic Axial Spondyloarthritis who have previously completed other studies with Tildrakizumab	EUCTR2018-001060-35-HU	2018
L-cure in the treatment of chronic hepatitis B	ChiCTR1800016645	2018
Effect of vitamin D on inflammation and muscle damage in athletes	IRCT2017042613678N27	2017
Effects of protein supplementation in nutritional status of hemodialysis patients	RBR-8r4jpg	2018
Activity and safety of oral administration of SSR150106XB for the reduction of inflammation in patients with active rheumatoid arthritis (RA): a 4-week, multi-center, randomized, double-blind, placebo-controlled parallel group study of 90 µg administrered once daily and 90µg once every other day - ACCORD-RA	EUCTR2007-000760-24-CZ	2007
Extract of Perilla frutescens enriched for rosmarinic acid, a polyphenolic phytochemical, inhibits seasonal allergic rhinoconjunctivitis in humans	Takano, , H, Osakabe, N, Sanbongi, C, Yanagisawa, R, Inoue, K, Yasuda, A, Natsume, M, Baba, S, Ichiishi, E; Yoshikawa, T	2004
Nicotinamide Riboside Supplementation for Treating Arterial Stiffness and Elevated Systolic Blood Pressure in Patients With Moderate to Severe CKD	NCT04040959	2019
Calorie Restriction in Multiple Sclerosis	NCT04042415	2019
Clinical and immunologic assessment of patients with psoriasis in a randomized, double-blind, placebo-controlled trial using recombinant human interleukin 10	Kimball, , AB, Kawamura, T, Tejura, K, Boss, C, Hancox, AR, Vogel, JC, Steinberg, SM, Turner, ML; Blauvelt, A	2002
The benefit of minocycline on negative symptoms of schizophrenia in patients with recent-onset psychosis (BeneMin): a randomised, double-blind, placebo-controlled trial	Deakin, , B, Suckling, J, Barnes, TRE, Byrne, K, Chaudhry, IB, Dazzan, P, Drake, RJ, Giordano, A, Husain, N, Jones, PB, Joyce, E, Knox, E, Krynicki, C, Lawrie, SM, Lewis, S, Lisiecka-Ford, DM, Nikkheslat, N, Pariante, CM, Smallman, R, Watson, A, Williams, SCR, Upthegrove, R; Dunn, G	2018
CD25 Blockade Delays Regulatory T Cell Reconstitution and Does Not Prevent Graft-versus-Host Disease After Allogeneic Hematopoietic Cell Transplantation	Locke, , FL, Pidala, J, Storer, B, Martin, PJ, Pulsipher, MA, Chauncey, TR, Jacobsen, N, Kröger, N, Walker, I, Light, S, Shaw, BE, Beato, F, Laport, GG, Nademanee, A, Keating, A, Socie, G; Anasetti, C	2017
Study on the positive effects of treatment with linagliptin on the arteries of patients with diabetes	EUCTR2012-005220-15-NL	2013
Safety, Tolerability and Efficacy of an antibody, which blocks the pro-inflammatory mediator Interleukin-6, in patients with late Antibody-mediated rejection of a transplanted kidney - A Pilot Trial	EUCTR2017-001604-30-AT	2017
comparison between methotrexate versus methotrexate plus leflunomide treatment in arthritis associated with psoriasis	CTRI/2017/06/008888	2017
Anti-Inflammatory Therapy With Canakinumab for the Prevention and Management of Diabetes	Everett, , BM, Donath, MY, Pradhan, AD, Thuren, T, Pais, P, Nicolau, JC, Glynn, RJ, Libby, P; Ridker, PM	2018
Phase I/II study of peptide vaccination associated with tumoral immunomodulation with proinflammatory cytokines and imiquimod in patients with advanced metastatic melanoma	EUCTR2010-020435-40-BE	2010
Effect of a high-egg diet on cardiometabolic risk factors in people with type 2 diabetes: the Diabetes and Egg (DIABEGG) Study-randomized weight-loss and follow-up phase	Fuller, , NR, Sainsbury, A, Caterson, ID, Denyer, G, Fong, M, Gerofi, J, Leung, C, Lau, NS, Williams, KH, Januszewski, AS, Jenkins, AJ; Markovic, TP	2018
IMMUNOTHERAPY OF MAINTENANCE IN THE PATIENTS WITH METASTATIC MELANOMA, CLINICAL BENEFIT AFTER CHEMOTHERAPY - ND	EUCTR2008-006366-29-IT	2009
The effect of atorvastatin plus aspirin compared with a non-therapeutic substance on the function of the blood vessels and inflammation in HIV patients under treatment presenting a low risk of cardiovascular disease	RBR-39bjm4	2015
Children with human immunodeficiency virus (HIV) in Africa, pharmacokinetics adn acceptability/adherence of simple antiretroviral regimen (CHAPAS-3)	PACTR201006000222401	2010
Renal and Humoral Effects of Sevelamer Carbonate in Patients with proteinuric nephropathies	EUCTR2012-005416-26-IT	2013
The PRIMO II Study: paricalcitol Injection benefits in Renal failure Induced cardiac Morbidity in Subjects with Chronic Kidney Disease Stage 5. Estudio Primo II: beneficios de Paricalcitol inyectable sobre la morbilidad cardíaca provocada por la insuficiencia renal en los sujetos con enfermedad renal crónica en estadio 5. - PRIMO II	EUCTR2007-005092-33-ES	2008
The PRIMO Study: paricalcitol Capsules benefits in Renal failure Induced cardiac Morbidity in Subjects with Chronic Kidney Disease Stage 3/4 - PRIMO I	EUCTR2007-001689-34-CZ	2008
An open, randomized study treating refractory adult-onset Still´s disease (AOSD) with interleukin-1 receptor antagonist anakinra (KineretR), compared to an established, single anti-rheumatic drug treatment. - AOSD Study 05	EUCTR2005-003173-24-FI	2005
Roxithromycin treatment prevents progression of peripheral arterial occlusive disease in Chlamydia pneumoniae seropositive men: a randomized, double-blind, placebo-controlled trial	Wiesli, , P, Czerwenka, W, Meniconi, A, Maly, FE, Hoffmann, U, Vetter, W; Schulthess, G	2002

Children with human immunodeficiency virus (HIV) in Africa - pharmacokinetics and acceptability/adherence of simple antiretroviral regimens (CHAPAS-3 trial)	ISRCTN69078957	2010
An evaluation of potential therapies to inhibit cerebral emboli in dementia	ISRCTN43384456	2007
The genotype CC of IL-28BSNP rs12979860 is significantly associated with a sustained virological response in chronic HCV-infected Pakistani patients	Khubaib, , B, Saleem, S, Idrees, M, Afzal, S; Wasim, M	2015
Maintenance of skin clearance with ixekizumab treatment of psoriasis: three-year results from the UNCOVER-3 study	Leonardi, , C, Maari, C, Philipp, S, Goldblum, O, Zhang, L, Burkhardt, N, Ball, S, Mallbris, L, Gonzalez, P, Fernández-Peñas, P; Puig, L	2018
Effects of 17beta-oestradiol plus different doses of drospirenone on adipose tissue, adiponectin and atherogenic metabolites in postmenopausal women	Tankó, , LB; Christiansen, C	2005
Evaluation of nutrition in adults on capd by bioelectrical impedence analysis (BIA) and anthropometry and the impact of intervention on their nutrition	Krishnamoorthy, V	2015
Cotreatment with the alpha-glucosidase inhibitor miglitol and DPP-4 inhibitor sitagliptin improves glycemic control and reduces the expressions of CVD risk factors in type 2 diabetic Japanese patients	Imai, , C, Saito, M, Mochizuki, K, Fuchigami, M, Goda, T; Osonoi, T	2014
Macrolide Use and Neutrophil Function/Cytokine Levels in Hospitalized Patients with Community-Acquired Pneumonia: a Pilot Study	Arnold, , FW, Bordon, J, Fernandez-Botran, R, Rane, MJ, Uriarte, SM, Kelley, R, Wiemken, TL, Peyrani, P; Ramirez, JA	2016
Effect of Laparoscopic Peritoneal Lavage and Drainage and Continuous Venovenous Diahemofiltration on Severe Acute Pancreatitis	Wang, , G, Liu, H, Xu, L, Wen, P, Wen, J, Zhou, SF; Xiao, X	2017
A therapeutic trial of decitabine and vorinostat in combination with chemotherapy for relapsed/refractory acute lymphoblastic leukemia	Burke, , MJ, Lamba, JK, Pounds, S, Cao, X, Ghodke-Puranik, Y, Lindgren, BR, Weigel, BJ, Verneris, MR; Miller, JS	2014
Control of airway inflammation maintained at a lower steroid dose with 100/50 mug of fluticasone propionate/salmeterol	Jarjour, , NN, Wilson, SJ, Koenig, SM, Laviolette, M, Moore, WC, Davis, WB, Doherty, DE, Hamid, Q, Israel, E, Kavuru, MS, Ramsdell, JW, Tashkin, DP, Reilly, DS, Yancey, SW, Edwards, LD, Stauffer, JL, Dorinsky, PM; Djukanovic, R	2006
Sputum eosinophilia and the short term response to inhaled mometasone in chronic obstructive pulmonary disease	Brightling, , CE, McKenna, S, Hargadon, B, Birring, S, Green, R, Siva, R, Berry, M, Parker, D, Monteiro, W, Pavord, ID; Bradding, P	2005
Activity of secukinumab, an anti-IL-17A antibody, on brain lesions in RRMS: results from a randomized, proof-of-concept study	Havrdová, , E, Belova, A, Goloborodko, A, Tisserant, A, Wright, A, Wallstroem, E, Garren, H, Maguire, RP; Johns, DR	2016
Impact of immune interventions on proviral HIV-1 DNA decay in patients receiving highly active antiretroviral therapy	Lafeuillade, , A, Poggi, C, Chadapaud, S, Hittinger, G, Khiri, H; Halfon, P	2001
Effect of acupuncture compared with placebo-acupuncture at P6 as additional antiemetic prophylaxis in high-dose chemotherapy and autologous peripheral blood stem cell transplantation: a randomized controlled single-blind trial	Streitberger, , K, Friedrich-Rust, M, Bardenheuer, H, Unnebrink, K, Windeler, J, Goldschmidt, H; Egerer, G	2003
The effects of Ginkgo biloba extract added to haloperidol on peripheral T cell subsets in drug-free schizophrenia: a double-blind, placebo-controlled trial	Zhang, , XY, Zhou, DF, Cao, LY; Wu, GY	2006
Efficacy and safety of tralokinumab in patients with severe uncontrolled asthma: a randomised, double-blind, placebo-controlled, phase 2b trial	Brightling, , CE, Chanez, P, Leigh, R, O'Byrne, PM, Korn, S, She, D, May, RD, Streicher, K, Ranade, K; Piper, E	2015
Randomized study on the efficacy of cognitive-behavioral therapy for insomnia secondary to breast cancer, part II: immunologic effects	Savard, , J, Simard, S, Ivers, H; Morin, CM	2005
Interleukin-2 therapy in relapsed acute myelogenous leukemia	Meloni, , G, Vignetti, M, Pogliani, E, Invernizzi, R, Allione, B, Mirto, S, Sica, S, Leoni, F, Selleri, C; Mandelli, F	1997
Bioequivalence of canakinumab liquid pre-filled syringe and reconstituted lyophilized formulations following 150 mg subcutaneous administration: a randomized study in healthy subjects	Chioato, , A, Noseda, E, Colin, L, Matott, R, Skerjanec, A; Dietz, AJ	2013
Relationships between cellular immune responses and treatment outcomes with interferon and ribavirin in HIV/hepatitis C virus co-infection	Graham, , CS, Wells, A, Liu, T, Sherman, KE, Peters, M, Chung, RT, Bhan, AK, Andersen, J; Koziel, MJ	2006
The rationale and design of the perindopril genetic association study (PERGENE): a pharmacogenetic analysis of angiotensin-converting enzyme inhibitor therapy in patients with stable coronary artery disease	Brugts, , JJ, De Maat, MPM, Boersma, E, Witteman, JCM, Van Duijn, C, Uitterlinden, AG, Bertrand, M, Remme, W, Fox, K, Ferrari, R, Danser, AHJ; Simoons, ML	2009
Phase 1 clinical trial of adoptive immunotherapy for acute myelogenous leukemia and myelodysplastic syndrome, using gene-modified autologous lymphocytes expressing WT1-Specific T-Cell receptor	Tanimoto, , K, Fujiwara, H, Tawara, I, Masuya, M, Kageyama, S, Nishida, T, Murata, M, Terakura, S, Akatsuka, Y, Ikeda, H, Miyahara, Y, Nukaya, I, Takesako, K, Emi, N, Katayama, N, Shiku, H; Yasukawa, M	2016
A phase I trial of Janus kinase (JAK) inhibition with INCB039110 in acute graft-versus-host disease (aGVHD)	Schroeder, , MA, Khoury, HJ, Jagasia, M, Ali, H, Schiller, GJ, Arbushites, M, Delaite, P, Yan, Y, Rhein, K, Perales, M-A, Chen, Y-B; DiPersio, JF	2016
Ixekizumab provides improvements through 52 weeks in physical function, quality of life, and work productivity in biologic disease-modifying antirheumatic drug-naive patients with active psoriatic arthritis	Gottlieb, , A, Husni, E, Lee, C, Lin, C-Y, Burge, R, Shuler, C; Gladman, D	2017
Acute rejection characteristics from a prospective, randomized, double-blind, placebo-controlled multicenter trial of early corticosteroid withdrawal	Gaber, , AO, Moore, LW, Alloway, RR, Woodle, ES, Pirsch, J, Shihab, F, Henning, A, Fitzsimmons, W, Holman, J, Reisfield, R; First, MR	2013
Randomized pilot trial of a synbiotic dietary supplement in chronic HIV-1 infection	Schunter, , M, Chu, H, Hayes, TL, McConnell, D, Crawford, SS, Luciw, PA, Bengmark, S, Asmuth, DM, Brown, J, Bevins, CL, Shacklett, BL; Critchfield, JW	2012

Long-term safety and efficacy of rilonacept in patients with systemic juvenile idiopathic arthritis	Lovell, , DJ, Giannini, EH, Reiff, AO, Kimura, Y, Li, S, Hashkes, PJ, Wallace, CA, Onel, KB, Foell, D, Wu, R, Biedermann, S, Hamilton, JD; Radin, AR	2013
Longterm safety and effectiveness of the anti-interleukin 6 receptor monoclonal antibody tocilizumab in patients with systemic juvenile idiopathic arthritis in Japan	Yokota, , S, Imagawa, T, Mori, M, Miyamae, T, Takei, S, Iwata, N, Umebayashi, H, Murata, T, Miyoshi, M, Tomiita, M, Nishimoto, N; Kishimoto, T	2014
Efficacy and safety of an anti-IL-13 mAb in patients with severe asthma: a randomized trial	De Boever, , EH, Ashman, C, Cahn, AP, Locantore, NW, Overend, P, Pouliquen, IJ, Serone, AP, Wright, TJ, Jenkins, MM, Panesar, IS, Thiagarajah, SS; Wenzel, SE	2014
Safety of tildrakizumab for moderate-to-severe plaque psoriasis: pooled analysis of three randomized controlled trials	Blauvelt, , A, Reich, K, Papp, KA, Kimball, AB, Gooderham, M, Tyring, SK, Sinclair, R, Thaci, D, Li, Q, Cichanowitz, N, Green, S; La Rosa, C	2018
Combination pravastatin and valsartan treatment has additive beneficial effects to simultaneously improve both metabolic and cardiovascular phenotypes beyond that of monotherapy with either drug in patients with primary hypercholesterolemia	Koh, , KK, Lim, S, Choi, H, Lee, Y, Han, SH, Lee, K, Oh, PC, Sakuma, I, Shin, EK; Quon, MJ	2013
Differences in responses upon corticosteroid therapy between smoking and non-smoking patients with COPD	Van Overveld, , FJ, Demkow, U, Górecka, D, De Backer, WA; Zieliński, J	2006
Subcutaneous methotrexate in patients with moderate-to-severe psoriasis: a critical appraisal	Tsakok, , T, Jabbar-Lopez, ZK; Smith, CH	2018
HIV-1 vaccine-induced immunity in the test-of-concept Step Study: a case-cohort analysis	McElrath, , MJ, De Rosa, SC, Moodie, Z, Dubey, S, Kierstead, L, Janes, H, Defawe, OD, Carter, DK, Hural, J, Akondy, R, Buchbinder, SP, Robertson, MN, Mehrotra, DV, Self, SG, Corey, L, Shiver, JW; Casimiro, DR	2008
The HYDILE trial: efficacy and tolerance of a quadruple combination of reverse transcriptase inhibitors versus the same regimen plus hydroxyurea or hydroxyurea and interleukin-2 in HIV-infected patients failing protease inhibitor-based combinations	Lafeuillade, , A, Hittinger, G, Chadapaud, S, Maillefet, S, Rieu, A; Poggi, C	2002
A prospective randomized controlled study of long-term combination therapy using ursodeoxycholic acid and bezafibrate in patients with primary biliary cirrhosis and dyslipidemia	Hosonuma, , K, Sato, K, Yamazaki, Y, Yanagisawa, M, Hashizume, H, Horiguchi, N, Kakizaki, S, Kusano, M; Yamada, M	2015
Effects of intermittent IL-2 alone or with peri-cycle antiretroviral therapy in early HIV infection: the STALWART study	Tavel, , JA, Babiker, A, Fox, L, Gey, D, Lopardo, G, Markowitz, N, Paton, N, Wentworth, D; Wyman, N	2010
Treatment extension of pegylated interferon alpha and ribavirin does not improve SVR in patients with genotypes 2/3 without rapid virological response (OPTEX Trial): a prospective, randomized, two-arm, multicentre phase IV clinical trial	Heidrich, , B, Cordes, H-J, Klinker, H, Moller, B, Naumann, U, Rossle, M, Kraus, MR, Boker, KH, Roggel, C, Schuchmann, M, Stoehr, A, Trein, A, Hardtke, S, Gonnermann, A, Koch, A, Wedemeyer, H, Manns, MP; Cornberg, M	2015
Gene Therapy in Neovascular Age-related Macular Degeneration: three-Year Follow-up of a Phase 1 Randomized Dose Escalation Trial	Constable, , IJ, Lai, CM, Magno, AL, French, MA, Barone, SB, Schwartz, SD, Blumenkranz, MS, Degli-Esposti, MA; Rakoczy, EP	2017
Minimal residual disease by MFC in acute lymphoblastic leukemia in children	Buldini, , B, Varotto, E; Basso, G	2018
Regulation of YKL-40 expression by corticosteroids: effect on pro-inflammatory macrophages in vitro and its modulation in COPD in vivo	Kunz, , LI, van't Wout, EF, van Schadewijk, A, Postma, DS, Kerstjens, HA, Sterk, PJ; Hiemstra, PS	2015
Clinical outcome and immune recovery after adoptive infusion of BPX-501 cells (donor T cells transduced with iC9 suicide gene) in children with hemoglobinopathies and diamond-blackfan anemia given alpha/beta T cell depleted HLA-haploidentical stem cell transplantation (HSCT)	Bertaina, , A, Merli, P, Pagliara, D, Pira, GL, De Angelis, B, Brescia, LP, Strocchio, L, Del Bufalo, F, Montanari, M, Lucarelli, B, Algeri, M, French, M, Moseley, A; Locatelli, F	2016
Quality-of-life improvements in cutaneous T-cell lymphoma patients treated with denileukin diftitox (ONTAK)	Duvic, , M, Kuzel, TM, Olsen, EA, Martin, AG, Foss, FM, Kim, YH, Heald, PW, Bacha, P, Nichols, J; Liepa, A	2002
Daclizumab high-yield process in relapsing-remitting multiple sclerosis (SELECT): a randomised, double-blind, placebo-controlled trial	Gold, , R, Giovannoni, G, Selmaj, K, Havrdova, E, Montalban, X, Radue, EW, Stefoski, D, Robinson, R, Riester, K, Rana, J, Elkins, J; O'Neill, G	2013
Efficacy and safety of lebrikizumab in adult patients with mild-to-moderate asthma not receiving inhaled corticosteroids	Korenblat, , P, Kerwin, E, Leshchenko, I, Yen, K, Holweg, CTJ, Anzures-Cabrera, J, Martin, C, Putnam, WS, Governale, L, Olsson, J; Matthews, JG	2018
eSET or eDET Associated to PGT in IVF	NCT03758833	2018
Secukinumab for moderate-to-severe palmoplantar pustular psoriasis: results of the 2PRECISE study	Mrowietz, , U, Bachelez, H, Burden, AD, Rissler, M, Sieder, C, Orsenigo, R; Chaouche-Teyara, K	2019
Effective treatment of refractory CMV reactivation after allogeneic stem cell transplantation with in vitro-generated CMV pp65-specific CD8+ T-cell lines	Meij, , P, Jedema, I, Zandvliet, ML, Van Der Heiden, PLJ, Van De Meent, M, Van Egmond, HME, Van Liempt, E, Hoogstraten, C, Kruithof, S, Veld, S, Marijt, EWA, Von Dem Borne, PA, Lankester, AC, Halkes, CJM; Falkenburg, JHF	2012
Imiquimod 5-percent cream does not alter the natural history of recurrent herpes genitalis: a phase II, randomized, double-blind, placebo-controlled study	Schacker, , TW, Conant, M, Thoming, C, Stanczak, T, Wang, Z; Smith, M	2002
Blood Eosinophils and Outcomes in Severe Hospitalized Exacerbations of COPD		2016
Management and outcome of BK viremia in renal transplant recipients: a prospective single-center study	Sood, , P, Senanayake, S, Sujeet, K, Medipalli, R, Zhu, YR, Johnson, CP; Hariharan, S	2012
Treatment of metastatic melanoma with combined chemotherapy containing cisplatin, vinblastine and dacarbazine (CVD) and biotherapy using interleukin-2 and interferon-alpha	Legha, , SS, Ring, S, Bedikian, A, Plager, C, Eton, O, Buzaid, AC; Papadopoulos, N	1996
Randomised phase II study of docetaxel plus vandetanib versus docetaxel followed by vandetanib in patients with persistent or recurrent epithelial ovarian, fallopian tube or primary peritoneal carcinoma: SWOG S0904	Coleman, , RL, Moon, J, Sood, AK, Hu, W, Delmore, JE, Bonebrake, AJ, Anderson, GL, Chambers, SK; Markman, M	2014

The effect of ixekizumab on scalp and nail psoriasis and health outcome measures over four years of open-label treatment in a phase 2 study in chronic plaque psoriasis	Kimball, , A, Zachariae, C, Burge, R, Cameron, G, McKean-Matthews, M; Lebwohl, M	2017
Ustekinumab in treatment of Crohn's disease: design, development, and potential place in therapy	Deepak, , P; Loftus, EV	2016
Acute and short-term chronic testosterone fluctuation effects on glucose homeostasis, insulin sensitivity, and adiponectin: a randomized, double-blind, placebo-controlled, crossover study	Host, , C, Gormsen, LC, Hougaard, DM, Christiansen, JS, Pedersen, SB; Gravholt, CH	2014
Effect of Bifidobacterium bifidum fermented milk on Helicobacter pylori and serum pepsinogen levels in humans	Miki, , K, Urita, Y, Ishikawa, F, Iino, T, Shibahara-Sone, H, Akahoshi, R, Mizusawa, S, Nose, A, Nozaki, D, Hirano, K, Nonaka, C; Yokokura, T	2007
Sequential peginterferon ALPHA-2a and telbivudine or vice versa for HBEAG-negative chronic hepatitis B: preliminary end-of-treatment results of the TEL-B-PEG randomized trial	Piccolo, , P, Lenci, I, Di Paolo, D, Demelia, L, Sorbello, O, Nosotti, L; Angelico, M	2011
Daclizumab hyp monotherapy improved health-related quality of life parameters in relapsing-remitting multiple sclerosis: findings of the SELECT trial	Selmaj, , K, Havrdova, E, Gold, R, Greenberg, S, Umans, K; Elkins, J	2012
Combination of SABR and L19-IL2 in Patients With Stage IV Lung Cancer (ImmunoSABR)	NCT02735850	2016
Regulatory effects of stage-treatment with established Chinese herbal formulas on inflammatory mediators in pediatric asthma	Li, , S, Wang, Y, Shi, Y, Yu, J, Sun, W, Hu, H; Zhang, Y	2013
Randomised trial and open-label extension study of an anti-interleukin-6 antibody in Crohn's disease (ANDANTE I and II)	Danese, , S, Vermeire, S, Hellstern, P, Panaccione, R, Rogler, G, Fraser, G, Kohn, A, Desreumaux, P, Leong, RW, Comer, GM, Cataldi, F, Banerjee, A, Maguire, MK, Li, C, Rath, N, Beebe, J; Schreiber, S	2019
In vitro glucocorticoid sensitivity is associated with clinical glucocorticoid therapy outcome in rheumatoid arthritis	Quax, , RA, Koper, JW, de Jong, PH, van Heerebeek, R, Weel, AE, Huisman, AM, van Zeben, D, de Jong, FH, Lamberts, SW, Hazes, JM; Feelders, RA	2012
Dornase alfa should be the first drug used in CF	Anbar, RD	2013
Interleukin 6 inhibition and coronary artery disease in a high-risk population: a prospective community-based clinical study	Bacchiega, , BC, Bacchiega, AB, Usnayo, MJG, Bedirian, R, Singh, G; Pinheiro, GRC	2017
A randomized study of low-dose interleukin-2 subcutaneous immunotherapy versus interleukin-2 plus interferon-alpha as first line therapy for metastatic renal cell carcinoma	Lissoni, , P, Barni, S, Ardizzoia, A, Andres, M, Scardino, E, Cardellini, P, Della Bitta, R; Tancini, G	1993
Phase 3 trials of ixekizumab in moderate-to-severe plaque psoriasis		2016
Immunotherapy for advanced renal cell cancer	Coppin, , C, Porzsolt, F, Awa, A, Kumpf, J, Coldman, A; Wilt, T	2005
Immunomodulatory effects of extracorporeal photochemotherapy in systemic sclerosis	Papp, , G, Horvath, IF, Barath, S, Gyimesi, E, Vegh, J, Szodoray, P; Zeher, M	2012
Probiotics during weaning reduce the incidence of eczema	West, , CE, Hammarström, ML; Hernell, O	2009
Impact of rosiglitazone on body composition, hepatic fat, fatty acids, adipokines and glucose in persons with impaired fasting glucose or impaired glucose tolerance: a sub-study of the DREAM trial	Punthakee, , Z, Alméras, N, Després, JP, Dagenais, GR, Anand, SS, Hunt, DL, Sharma, AM, Jung, H, Yusuf, S; Gerstein, HC	2014
Efficacy and safety of secukinumab in patients with generalized pustular psoriasis: a 52-week analysis from phase III open-label multicenter Japanese study	Imafuku, , S, Honma, M, Okubo, Y, Komine, M, Ohtsuki, M, Morita, A, Seko, N, Kawashima, N, Ito, S, Shima, T; Nakagawa, H	2016
Optimisation of Nutrition and Medication for Acutely Admitted Older Medical Patients	NCT03741283	2018
Increased circulating follicular helper T cells with decreased programmed death-1 in chronic renal allograft rejection	Shi, , J, Luo, F, Shi, Q, Xu, X, He, X; Xia, Y	2015
Interleukin-2-activated autologous bone marrow and peripheral blood stem cells in the treatment of acute leukemia and lymphoma	Margolin, , KA, Van Besien, K, Wright, C, Niland, J, Champlin, R, Fung, HC, Kashyap, A, Molina, A, Nademanee, AP, O'Donnell, MR, Parker, P, Smith, E, Spielberger, R, Somlo, G, Snyder, D, Stein, A, Woo, D, Thomas, M, Sniecinski, I; Forman, SJ	1999
Efficacy of a Triptan in the Treatment of Hostility and Aggression Among Convicts With a Psychiatric Treatment Order	NCT00282165	2006
A phase I, randomized study of combined IL-2 and therapeutic immunisation with antiretroviral therapy	Hardy, , GAD, Imami, N, Nelson, MR, Sullivan, AK, Moss, R, Aasa-Chapman, MMI, Gazzard, B; Gotch, FM	2007
Nebulized budesonide in the treatment of exacerbations of chronic obstructive pulmonary disease: efficacy, safety, and effects on the serum levels of soluble differentiation molecules	Makarova, , EV, Varvarina, GN, Menkov, NV, Czapaeva, MY, Lazareva, ES, Kazatskaya, ZA, Novikov, VV; Karaulov, AV	2016
Therapeutic effect of paroxetine on patients with early poststroke depression and the serum interleukins	Yang, , J, Zhao, X-H, Zhu, Y-P, Bai, Q-K, Chen, J, Xia, W-Y, Chen, L-J; Lin, T-S	2011
Randomized study of recombinant interleukin-2 after autologous bone marrow transplantation for acute leukemia in first complete remission	Blaise, , D, Attal, M, Reiffers, J, Michallet, M, Bellanger, C, Pico, JL, Stoppa, AM, Payen, C, Marit, G, Bouabdallah, R, Sotto, JJ, Rossi, JF, Brandely, M, Hercend, T; Maraninchi, D	2000
Etanercept, mycophenolate, denileukin, or pentostatin plus corticosteroids for acute graft-versus-host disease: a randomized phase 2 trial from the Blood and Marrow Transplant Clinical Trials Network	Alousi, , AM, Weisdorf, DJ, Logan, BR, Bolaños-Meade, J, Carter, S, Difronzo, N, Pasquini, M, Goldstein, SC, Ho, VT, Hayes-Lattin, B, Wingard, JR, Horowitz, MM; Levine, JE	2009
Increase of HIV-1 pro-viral DNA per million peripheral blood mononuclear cells in patients with advanced HIV disease (CD4<200 cells/mm3) receiving interleukin 2 combined with HAART versus HAART alone (ANRS-082 trial)	Delaugerre, , C, Gourlain, K, Tubiana, R, Carcelain, G, Marcelin, AG, Chouquet, C, Mouroux, M, Duvivier, C, Autran, B, Costagliola, D, Katlama, C; Calvez, V	2003
Results of a phase-I/II randomized, masked, placebo-controlled trial of recombinant human interleukin-11 (rhIL-11) in the treatment of subjects with active rheumatoid arthritis	Moreland, , L, Gugliotti, R, King, K, Chase, W, Weisman, M, Greco, T, Fife, R, Korn, J, Simms, R, Tesser, J, Hillson, J, Caldwell, J, Schnitzer, T, Lyons, D; Schwertschlag, U	2001

Early pregnancy vitamin D status and risk of preeclampsia	Mirzakhani, , H, Litonjua, AA, McElrath, TF, O'Connor, G, Lee-Parritz, A, Iverson, R, Macones, G, Strunk, RC, Bacharier, LB, Zeiger, R, Hollis, BW, Handy, DE, Sharma, A, Laranjo, N, Carey, V, Qiu, W, Santolini, M, Liu, S, Chhabra, D, Enquobahrie, DA, Williams, MA, Loscalzo, J; Weiss, ST	2016
Rucaparib maintenance treatment for recurrent ovarian carcinoma after response to platinum therapy (ARIEL3): a randomised, double-blind, placebo-controlled, phase 3 trial	Coleman, , RL, Oza, AM, Lorusso, D, Aghajanian, C, Oaknin, A, Dean, A, Colombo, N, Weberpals, JI, Clamp, A, Scambia, G, Leary, A, Holloway, RW, Gancedo, MA, Fong, PC, Goh, JC, O'Malley, DM, Armstrong, DK, Garcia-Donas, J, Swisher, EM, Floquet, A, Konecny, GE, McNeish, IA, Scott, CL, Cameron, T, Maloney, L, Isaacson, J, Goble, S, Grace, C, Harding, TC, Raponi, M, Sun, J, Lin, KK, Giordano, H; Ledermann, JA	2017
Effect of a single autologous cord blood infusion on beta-cell and immune function in children with new onset type 1 diabetes: a non-randomized, controlled trial	Giannopoulou, , EZ, Puff, R, Beyerlein, A, von Luettichau, I, Boerschmann, H, Schatz, D, Atkinson, M, Haller, MJ, Egger, D, Burdach, S; Ziegler, AG	2014
Prophylactic use of low-dose interleukin-2 and the clinical outcomes of hematopoietic stem cell transplantation: a randomized study	Zhao, , X-Y, Zhao, X-S, Wang, Y-T, Chen, Y-H, Xu, L-P, Zhang, X-H, Han, W, Chen, H, Wang, Y, Yan, C-H, Wang, F-R, Wang, J-Z, Liu, K-Y, Chang, Y-J; Huang, X-J	2016
A phase two randomised controlled double blind trial of high dose intravenous methylprednisolone and oral prednisolone versus intravenous normal saline and oral prednisolone in individuals with leprosy type 1 reactions and/or nerve function impairment	Walker, , SL, Nicholls, PG, Dhakal, S, Hawksworth, RA, Macdonald, M, Mahat, K, Ruchal, S, Hamal, S, Hagge, DA, Neupane, KD; Lockwood, DN	2011
A pilot study of the effect of spironolactone therapy on exercise capacity and endothelial dysfunction in pulmonary arterial hypertension: study protocol for a randomized controlled trial	Elinoff, , JM, Rame, JE, Forfia, PR, Hall, MK, Sun, J, Gharib, AM, Abd-Elmoniem, K, Graninger, G, Harper, B, Danner, RL; Solomon, MA	2013
A phase 3 randomized trial comparing inolimomab vs usual care in steroid-resistant acute GVHD	Socié, , G, Vigouroux, S, Yakoub-Agha, I, Bay, JO, Fürst, S, Bilger, K, Suarez, F, Michallet, M, Bron, D, Gard, P, Medeghri, Z, Lehert, P, Lai, C, Corn, T; Vernant, JP	2017
Pre-transplant r-bendamustine induces high rates of minimial residual disease in MCL patients: updated results of S1106: US intergroup study of a randomized phase II trial of R-HCVAD Vs. R-bendamustine followed by autologous stem cell transplants for patients with mantle cell lymphoma	Chen, , R, Li, H, Bernstein, SH, Rimsza, LM, Forman, SJ, Constine, L, Shea, TC, Cashen, AF, Blum, KA, Fenske, TS, Barr, PM, Leblanc, M, Fisher, RI, Cheson, BD, Smith, SM, Faham, M, Wilkins, J, Leonard, JP, Kahl, BS; Friedberg, JW	2015
Efficacy and safety of a monoclonal antibody recognizing interleukin-8 in COPD: a pilot study	Mahler, , DA, Huang, S, Tabrizi, M; Bell, GM	2004
Inhaled interleukin-2 therapy in pulmonary metastatic renal cell carcinoma: six years of experience	Huland, , E, Heinzer, H, Mir, TS; Huland, H	1997
Sarilumab plus methotrexate in patients with active rheumatoid arthritis and inadequate response to methotrexate: results of a randomized, placebo-controlled phase III trial in Japan	Tanaka, , Y, Wada, K, Takahashi, Y, Hagino, O, Van Hoogstraten, H, Graham, NMH; Kameda, H	2019
Curative effect of donor lymphocyte and interleukin-2 on acute myelogenous leukemia after mixed hematopoietic stem cell transplantation	Wang, , C-B, Bai, H, Ou, Y-X, Pan, YZ, Zhang, Q; Xi, R	2007
High dose versus low dose fludarabine in the treatment of patients with severe refractory rheumatoid arthritis	Davis, , JC, Fessler, BJ, Tassiulas, IO, McInnes, IB, Yarboro, CH, Pillemer, S, Wilder, R, Fleisher, TA, Klippel, JH; Boumpas, DT	1998
Randomized trial of low-dose interleukin-2 vs cyclosporine A and interferon-gamma after high-dose chemotherapy with peripheral blood progenitor support in women with high-risk primary breast cancer	Vahdat, , LT, Cohen, DJ, Zipin, D, Lo, KS, Donovan, D, Savage, D, Tiersten, A, Nichols, G, Troxel, A; Hesdorffer, CS	2007
Randomized phase II trial of BCDT [carmustine (BCNU), cisplatin, dacarbazine (DTIC) and tamoxifen] with or without interferon alpha (IFN-alpha) and interleukin (IL-2) in patients with metastatic melanoma	Johnston, , SR, Constenla, DO, Moore, J, Atkinson, H, A'Hern, RP, Dadian, G, Riches, PG; Gore, ME	1998
Treatment beyond progression with nivolumab in patients with recurrent or metastatic (R/M) squamous cell carcinoma of the head and neck (SCCHN) in the phase 3 checkmate 141 study: a biomarker analysis and updated clinical outcomes	Haddad, , R, Blumenschein, G, Fayette, J, Guigay, J, Colevas, AD, Licitra, L, Kasper, S, Vokes, EE, Worden, F, Saba, NF, Tahara, M, Concha-Benavente, F, Monga, M, Lynch, M, Li, L, Shaw, JW, Gillison, ML, Harrington, KJ; Ferris, RL	2017
Anti-Interleukin-31 Receptor A Antibody for Atopic Dermatitis	Ruzicka, , T, Hanifin, JM, Furue, M, Pulka, G, Mlynarczyk, I, Wollenberg, A, Galus, R, Etoh, T, Mihara, R, Yoshida, H, Stewart, J; Kabashima, K	2017
PAINTER: evaluation of eribulin tolerability and correlation between a set of polymorphisms and neuropathy in patients with metastatic breast cancer	La Verde, , N, Moretti, A, Damia, G, Paterno, E, Santini, D, Garrone, O, Fabi, A, Ciccarese, M, Cretella, E, Torri, V, Generali, D, Grasso, D, Puglisi, F, Collova, E, Roila, F, Bertolini, A, Barni, S, Vici, P, Luigi, C, Scandurra, G, Bramati, A, Dazzani, MC; Farina, G	2017
Reduced helminth burden increases allergen skin sensitization but not clinical allergy: a randomized, double-blind, placebo-controlled trial in Vietnam	Flohr, , C, Tuyen, LN, Quinnell, RJ, Lewis, S, Minh, TT, Campbell, J, Simmons, C, Telford, G, Brown, A, Hien, TT, Farrar, J, Williams, H, Pritchard, DI; Britton, J	2010
Efficacy and safety of sirukumab in patients with active rheumatoid arthritis refractory to anti-TNF therapy (SIRROUND-T): a randomised, double-blind, placebo-controlled, parallel-group, multinational, phase 3 study	Aletaha, , D, Bingham, CO, Tanaka, Y, Agarwal, P, Kurrasch, R, Tak, PP; Popik, S	2017
The mitochondrial pharmacogenomics of haplogroup T: mTND2*LHON4917G and antiretroviral therapy-associated peripheral neuropathy	Canter, , JA, Haas, DW, Kallianpur, AR, Ritchie, MD, Robbins, GK, Shafer, RW, Clifford, DB, Murdock, DG; Hulgan, T	2008
A pilot exercise intervention to improve lower extremity functioning in peripheral arterial disease unaccompanied by intermittent claudication	McDermott, , MM, Tiukinhoy, S, Greenland, P, Liu, K, Pearce, WH, Guralnik, JM, Unterreiner, S, Gluckman, TJ, Criqui, MH; Ferrucci, L	2004
HAT-01, a novel herbal preparation, is superior to corticosteroids and pimecrolimus for the treatment of moderate to severe atopic dermatitis	Alex, , P, Payne, A, Desai, A, Centola, M, Thomas, S; Yesudas, T	2013

Deciphering antitumour response and resistance with intratumour heterogeneity (DARWIN II)	Hiley, , CT, Ahmad, T, Jamal-Hanjani, M, Abbosh, C, Ngai, Y, Hackshaw, A, Patterson, P, Baijal, S, Nicolson, M, Lester, JF, Krebs, M, Ottensmeier, CHH, Fennell, DA, Schmid, P, Ezhil, M, Danson, S, Steele, NL, Middleton, GW, Dive, C; Swanton, C	2017
Robust mucosal-homing antibody-secreting B cell responses induced by intramuscular administration of adjuvanted bivalent human norovirus-like particle vaccine	Sundararajan, , A, Sangster, MY, Frey, S, Atmar, RL, Chen, WH, Ferreira, J, Bargatze, R, Mendelman, PM, Treanor, JJ; Topham, DJ	2015
Anti-interleukin-17A monoclonal antibody secukinumab in treatment of ankylosing spondylitis: a randomised, double-blind, placebo-controlled trial	Baeten, , D, Baraliakos, X, Braun, J, Sieper, J, Emery, P, van der Heijde, D, McInnes, I, van Laar, JM, Landewé, R, Wordsworth, P, Wollenhaupt, J, Kellner, H, Paramarta, J, Wei, J, Brachat, A, Bek, S, Laurent, D, Li, Y, Wang, YA, Bertolino, AP, Gsteiger, S, Wright, AM; Hueber, W	2013
Lebrikizumab in moderate-to-severe asthma: pooled data from two randomised placebo-controlled studies	Hanania, , NA, Noonan, M, Corren, J, Korenblat, P, Zheng, Y, Fischer, SK, Cheu, M, Putnam, WS, Murray, E, Scheerens, H, Holweg, CT, Maciuca, R, Gray, S, Doyle, R, McClintock, D, Olsson, J, Matthews, JG; Yen, K	2015
Safety and Immunomodulatory Effects of Three Probiotic Strains Isolated from the Feces of Breast-Fed Infants in Healthy Adults: SETOPROB Study	Plaza-Diaz, , J, Gomez-Llorente, C, Campana-Martin, L, Matencio, E, Ortuno, I, Martinez-Silla, R, Gomez-Gallego, C, Periago, MJ, Ros, G, Chenoll, E, Genoves, S, Casinos, B, Silva, A, Corella, D, Portoles, O, Romero, F, Ramon, D, Perez de la Cruz, A, Gil, A; Fontana, L	2013
Peripheral blood SIRT1 mRNA levels in depression and treatment with electroconvulsive therapy	McGrory, , CL, Ryan, KM, Kolshus, E, Finnegan, M; McLoughlin, DM	2018
Post-transplant sCD30 and neopterin as predictors of chronic allograft nephropathy: impact of different immunosuppressive regimens	Weimer, , R, Süsal, C, Yildiz, S, Staak, A, Pelzl, S, Renner, F, Dietrich, H, Daniel, V, Kamali-Ernst, S, Ernst, W, Padberg, W; Opelz, G	2006
Anti-inflammatory effects of montelukast in mild cystic fibrosis	Schmitt-Grohé, , S, Eickmeier, O, Schubert, R, Bez, C; Zielen, S	2002
Efficacy and safety of ixekizumab at week 52 in biologic-naïve patients with active psoriatic arthritis (SPIRIT-P1)	Lespessailles, , E, Kirkham, B, Nebro, A, Alonso, R, Strehblow, C, Inciarte, J, Porsdal, V, Garcia, M, Kurzawa, M, Sapin, C, Geusens, P, Goupille, P; Tahir, H	2018
A Phase 2/3 Multicenter, Randomized, Open-Label Study to Compare the Efficacy and Safety of Lenalidomide Versus Investigator's Choice in Patients with Relapsed or Refractory Diffuse Large B-Cell Lymphoma	Czuczman, , MS, Trněný, M, Davies, A, Rule, S, Linton, KM, Wagner-Johnston, N, Gascoyne, RD, Slack, GW, Brousset, P, Eberhard, DA, Hernandez-Ilizaliturri, FJ, Salles, G, Witzig, TE, Zinzani, PL, Wright, GW, Staudt, LM, Yang, Y, Williams, PM, Lih, CJ, Russo, J, Thakurta, A, Hagner, P, Fustier, P, Song, D; Lewis, ID	2017
Simvastatin in Secondary Progressive Multiple Sclerosis	NCT03896217	2019
Increased IL-17A expression in temporal artery lesions is a predictor of sustained response to glucocorticoid treatment in patients with giant-cell arteritis	Espigol-Frigole, , G, Corbera-Bellalta, M, Planas-Rigol, E, Lozano, E, Segarra, M, Garcia-Martinez, A, Prieto-Gonzalez, S, Hernandez-Rodriguez, J, Grau, JM, Rahman, MU; Cid, MC	2013
Selective capacity of metreleptin administration to reconstitute CD4+ T-cell number in females with acquired hypoleptinemia	Matarese, , G, La Rocca, C, Moon, HS, Huh, JY, Brinkoetter, MT, Chou, S, Perna, F, Greco, D, Kilim, HP, Gao, C, Arampatzi, K, Wang, Z; Mantzoros, CS	2013
Phase II study of eribulin in combination with gemcitabine for the treatment of patients with locally advanced or metastatic triple negative breast cancer: ERIGE trial on behalf of the Gruppo Oncologico Italiano di Ricerca Clinica (GOIRC)	Musolino, , A, Porzio, R, Rubino, D, Frassoldati, A, Caldara, A, Schirone, A, Gori, S, Piacentini, F, Berardi, R, Brandes, AA, Cavazzini, G, Foglietta, J, Villa, F, Pellegrino, B, Camisa, R, Todeschini, R, Tognetto, M, Naldi, N, Baldari, D; Montemurro, F	2017
Siltuximab for multicentric Castleman's disease: a randomised, double-blind, placebo-controlled trial	van Rhee, , F, Wong, RS, Munshi, N, Rossi, JF, Ke, XY, Fosså, A, Simpson, D, Capra, M, Liu, T, Hsieh, RK, Goh, YT, Zhu, J, Cho, SG, Ren, H, Cavet, J, Bandekar, R, Rothman, M, Puchalski, TA, Reddy, M, van de Velde, H, Vermeulen, J; Casper, C	2014
Balance of autonomic nervous system predicts who benefits from a self-management intervention program for irritable bowel syndrome	Jarrett, , ME, Cain, KC, Barney, PG, Burr, RL, Naliboff, BD, Shulman, R, Zia, J; Heitkemper, MM	2016
Immunity and clinical efficacy of an inactivated enterovirus 71 vaccine in healthy Chinese children: a report of further observations	Liu, , L, Mo, Z, Liang, Z, Zhang, Y, Li, R, Ong, KC, Wong, KT, Yang, E, Che, Y, Dong, C, Feng, M, Pu, J, Liao, Y, Jiang, L, Tan, SH, David, P, Huang, T, Zhou, Z, Wang, X, Xia, J, Guo, L, Wang, L, Xie, Z, Cui, W, Mao, Q, Liang, Y, Zhao, H, Na, R, Cui, P, Shi, H, Wang, J; Li, Q	2015
Ixekizumab, an interleukin-17A specific monoclonal antibody, for the treatment of biologic-naive patients with active psoriatic arthritis: results from the 24-week randomised, double-blind, placebo-controlled and active (adalimumab)-controlled period of the phase III trial SPIRIT-P1	Mease, , PJ, van der Heijde, D, Ritchlin, CT, Okada, M, Cuchacovich, RS, Shuler, CL, Lin, CY, Braun, DK, Lee, CH; Gladman, DD	2017
Ixabepilone administered weekly or every three weeks in HER2-negative metastatic breast cancer patients; a randomized non-comparative phase II trial	Fountzilas, , G, Kotoula, V, Pectasides, D, Kouvatseas, G, Timotheadou, E, Bobos, M, Mavropoulou, X, Papadimitriou, C, Vrettou, E, Raptou, G, Koutras, A, Razis, E, Bafaloukos, D, Samantas, E, Pentheroudakis, G; Skarlos, DV	2013
Pilot study of interferon-alpha-ribavirin-interleukin-2 for treatment of nonresponder patients with severe liver disease infected by hepatitis C virus genotype 1	Alric, , L, Thebault, S, Peron, JM, Balard, P, Metivier, S, Pipy, B, Izopet, J; Vinel, JP	2006
Pharmacokinetic, pharmacodynamic and clinical effects of a humanized IgG1 anti-CD4 monoclonal antibody in the peripheral blood and synovial fluid of rheumatoid arthritis patients	Choy, , EH, Connolly, DJ, Rapson, N, Jeal, S, Brown, JC, Kingsley, GH, Panayi, GS; Johnston, JM	2000
Oral 4-aminosalicylic acid versus 5-aminosalicylic acid slow release tablets. Double blind, controlled pilot study in the maintenance treatment of Crohn's ileocolitis	Schreiber, , S, Howaldt, S; Raedler, A	1994

Secukinumab efficacy and safety in Japanese patients with moderate-to-severe plaque psoriasis: subanalysis from ERASURE, a randomized, placebo-controlled, phase 3 study	Ohtsuki, , M, Morita, A, Abe, M, Takahashi, H, Seko, N, Karpov, A, Shima, T, Papavassilis, C; Nakagawa, H	2014
NEURAPRO-E study protocol: a multicentre randomized controlled trial of omega-3 fatty acids and cognitive-behavioural case management for patients at ultra high risk of schizophrenia and other psychotic disorders	Markulev, , C, McGorry, PD, Nelson, B, Yuen, HP, Schaefer, M, Yung, AR, Thompson, A, Berger, G, Mossaheb, N, Schlogelhofer, M, Smesny, S, de Haan, L, Riecher-Rossler, A, Nordentoft, M, Chen, EYH, Verma, S, Hickie, I; Amminger, GP	2017
Total body irradiation, etoposide, cyclophosphamide, and autologous peripheral blood stem-cell transplantation followed by randomization to therapy with interleukin-2 versus observation for patients with non-Hodgkin lymphoma: results of a phase 3 randomized trial by the Southwest Oncology Group (SWOG 9438)	Thompson, , JA, Fisher, RI, Leblanc, M, Forman, SJ, Press, OW, Unger, JM, Nademanee, AP, Stiff, PJ, Petersdorf, SH; Fefer, A	2008
Double blind clinical trial in a series of 115 patients with seborrheic dermatitis: prevention of relapses using a topical modulator of Toll like receptor 2	Ionescu, , MA, Baroni, A, Brambilla, L, Cannavò, SP, Cristaudo, A, Vedove, CD, Frasca, M, Girolomoni, G, Gnecchi, L, Peris, K, Trifirò, C, Matta, AM; Robert, G	2011
Acute peripheral administration of synthetic human GLP-1 (7-36 amide) decreases circulating IL-6 in obese patients with type 2 diabetes mellitus: a potential role for GLP-1 in modulation of the diabetic pro-inflammatory state?	Daousi, , C, Pinkney, JH, Cleator, J, Wilding, JP; Ranganath, LR	2013
The contribution of skin and joint improvements to the health-related quality of life of patients with active psoriatic arthritis	Kavanaugh, , A, Gottlieb, AB, Morita, A, Merola, J, Birt, J, Lin, C-Y, Shuler, C; Thaci, D	2017
Recombinant human interleukin-1 receptor type I in the treatment of patients with active rheumatoid arthritis	Drevlow, , BE, Lovis, R, Haag, MA, Sinacore, JM, Jacobs, C, Blosche, C, Landay, A, Moreland, LW; Pope, RM	1996
ImmuniCell® in patients with advanced cancers	ISRCTN26113637	2016
Effects Of extra-fine particle HFA-becLomethasone (HFA-QVAR) Versus course particle treatment In smokers and ex-smokers with Asthma	EUCTR2012-005350-39-NL	2013
Impact of daclizumab HYP monotherapy on healthrelated quality of life in relapsing-remitting multiple sclerosis patients	Selmaj, , K, Havrdova, E, Gold, R, Greenberg, S, Phillips, G, Umans, K; Elkins, J	2012
Two-year clinical response to brodalumab, an anti-il-17 receptor antibody, in patients with psoriatic arthritis	Mease, , P, Genovese, MC, Greenwald, MW, Ritchlin, CT, Beaulieu, A, Deodhar, A, Newmark, R, Feng, J, Erondu, N; Nirula, A	2015
SATURN: Effect of secukinumab in the treatment of psoriatic arthritis	ISRCTN16488621	2016
A randomized, double-blind, placebo-controlled, parallel group study to evaluate the effects of a 4-week treatment of 15 mg b.i.d BIBW 2948 BS (inhalation powder, hard capsule for HandiHaler®) on epithelial mucin stores and the safety and efficacy in COPD patients with symptoms associated with chronic bronchitis - Effects of BIBW 1948 on epithelial mucin	EUCTR2006-001975-40-DE	2006
A Phase I/IIa Study of Low Dose Subcutaneous Interleukin-2 (IL-2) for Treatment of Refractory Chronic Graft Versus Host Disease (GVHD)	JPRN-UMIN000022253	2016
A Study Comparing GSK2118436 to Dacarbazine (DTIC) in Previously Untreated Subjects With BRAF Mutation Positive Advanced (Stage III) or Metastatic (Stage IV) Melanoma	ACTRN12611000311954	2011
Effect of Borago officinalis extract on moderate persistent asthma	IRCT201408052695N6	2014
The effect of laser therapy on gingival inflammation	IRCT2017011620523N2	2017
Abatacept to Silence anti-Citrullinated protein Antibody-expressing B cells in Rheumatoid Arthritis	EUCTR2017-002878-38-NL	2017
Efficacy and safety of guselkumab in patients with psoriasis who have an inadequate response to ustekinumab: results of the randomized, double-blind, phase III NAVIGATE trial	Langley, , RG, Tsai, TF, Flavin, S, Song, M, Randazzo, B, Wasfi, Y, Jiang, J, Li, S; Puig, L	2018
Maintenance treatment with mirikizumab, a p19-directed IL-23 antibody: 52-week results in patients with moderately-to-severely active ulcerative colitis	Geert, , G, D'Haens, R, Sandborn, WJ, Ferrante, M, Bhandari, BR, Berliba, E, Hibi, T, Tuttle, J, Canavan, JB, Friedrich, S, Durante, M, Arora, V; Feagan, B	2019
Multicenter, Open-label, Exploratory Phase I Pilot Study to Investigate Safety, Pharmacodynamics and Pharmacokinetics of Immunological Effects and Activity of Combining Multiple Doses of IMAB362 With Immunomodulation (Zoledronic Acid, Interleukin-2) in Patients With Advanced Adenocarcinoma of the Stomach, the Lower Esophagus or the Gastro-esophageal Junction	DRKS00005206	2014
Efficacy of Symptom-driven treatment with inhaled budesonide/formoterol versus fixed dose fluticasone/salmeterol in COPD	EUCTR2014-001563-12-NL	2015
A PHASE II STUDY OF ATEZOLIZUMAB WITH RITUXIMAB, GEMCITABINE AND OXALIPLATIN IN PATIENTS WITH RELAPSED OR REFRACTORY DIFFUSE LARGE B-CELL LYMPHOMA WHO ARE NOT CANDIDATES FOR HIGH-DOSE THERAPY	EUCTR2016-002654-21-GB	2018
Effect of tralokinumab, an interleukin-13 neutralising monoclonal antibody, on eosinophilic airway inflammation in uncontrolled moderate-to-severe asthma (MESOS): a multicentre, double-blind, randomised, placebo-controlled phase 2 trial	Russell, , RJ, Chachi, L, FitzGerald, JM, Backer, V, Olivenstein, R, Titlestad, IL, Ulrik, CS, Harrison, T, Singh, D, Chaudhuri, R, Leaker, B, McGarvey, L, Siddiqui, S, Wang, M, Braddock, M, Nordenmark, LH, Cohen, D, Parikh, H, Colice, G; Brightling, CE	2018
A study to investigate the effects of lumicitabine (JNJ-64041575) in hospitalized adults infected with human metapneumovirus	EUCTR2017-001696-22-PL	2018
Effect of vitamin D and calcium supplementation on serum levels of hormones affecting postpartum depression	IRCT2016111016123N12	2017

Dupilumab with concomitant topical corticosteroid treatment in adults with atopic dermatitis with an inadequate response or intolerance to ciclosporin A or when this treatment is medically inadvisable: a placebo-controlled, randomized phase III clinical trial (LIBERTY AD CAFÉ)	de Bruin-Weller, , M, Thaci, D, Smith, CH, Reich, K, Cork, MJ, Radin, A, Zhang, Q, Akinlade, B, Gadkari, A, Eckert, L, Hultsch, T, Chen, Z, Pirozzi, G, Graham, NMH; Shumel, B	2018
Effect of Propolis on moderate asthma	IRCT201209302695N4	2015
Individual Therapy by Gene Expression for Colon Cancer Patients	EUCTR2012-001307-20-ES	2012
MAINTENENCE TREATMENT WITH MIRIKIZUMAB, A P19-DIRECTED IL-23 ANTIBODY: 52-WEEK RESULTS IN PATIENTS WITH MODERA℡Y-TOSEVERELY ACTIVE ULCERATIVE COLITIS	D'Haens, , GR, Sandborn, WJ, Ferrante, M, Bhandari, BR, Berliba, E, Hibi, T, Tuttle, J, Friedrich, S, Durante, M, Arora, V, Feagan, BG; Canavan, J	2019
Efficacy and safety of dupilumab in adults with moderate-to-severe atopic dermatitis inadequately controlled by topical treatments: a randomised, placebo-controlled, dose-ranging phase 2b trial	Thaçi, , D, Simpson, EL, Beck, LA, Bieber, T, Blauvelt, A, Papp, K, Soong, W, Worm, M, Szepietowski, JC, Sofen, H, Kawashima, M, Wu, R, Weinstein, SP, Graham, NM, Pirozzi, G, Teper, A, Sutherland, ER, Mastey, V, Stahl, N, Yancopoulos, GD; Ardeleanu, M	2016
The effect of Docosahexaenoic acid on factors influencing vascular thickness and function in diabetic patients	IRCT2013071213964N1	2013
A trial of bortezomib with R-CHOP for diffuse large B cell lymphoma (DLBCL)	EUCTR2010-022422-32-GB	2010
A Phase II repeated dose, randomized and blinded trial of CIM331 in patients with eczema in whom products applied to the skin do not provide sufficient relief or produce side effects	EUCTR2013-002470-46-GB	2013
Effect of angiotensin-converting enzyme inhibitors on systemic inflammation and myocardial sympathetic innervation in normotensive patients with type two diabetes mellitus	ISRCTN76608550	2007
Exclusive nutritional therapy and intestinal homeostasis in pediatric inflammatory bowel disease	DRKS00003548	2012
AMG 714 (ANTI-IL-15 MAB) AMELIORATES THE EFFECTS OF GLUTEN CONSUMPTION IN CELIAC DISEASE: a PHASE 2A, RANDOMIZED, DOUBLE-BLIND, PLACEBO-CONTROLLED STUDY EVALUATING AMG 714 IN ADULT PATIENTS WITH CELIAC DISEASE EXPOSED TO A HIGH-DOSE GLUTEN CHALLENGE	Lahdeaho, , M-L, Scheinin, M, Pesu, M, Kivela, L, Lovro, Z, Keisala, J, Leon, F; Maki, M	2018
Comparing peripherally inserted central catheter (PICC) materials to prevent infections and blood clots: a randomized controlled trial	ACTRN12619000022167	2019
BASELINE CHARACTERISTICS AND CORRELATION BETWEEN DYSPHAGIA AND DISEASE ACTIVITY IN PATIENTS WITH EOSINOPHILIC ESOPHAGITIS IN A RANDOMIZED, PLACEBO-CONTROLLED, PHASE 2 DUPILUMAB TRIAL	Collins, , MH, Hirano, I, Dellon, ES, Peterson, KA, Chehade, M, Schoepfer, AM, Safroneeva, E, Rothenberg, ME, Falk, GW, Assouline-Dayan, Y, Chen, Z, Pirozzi, G, Mannent, L, Graham, NM, Ruddy, M, Hamilton, JD; Radin, A	2018
Safety and Pharmacology of NOX-H94 in renal dialysis patients with ESA resistant anaemia	EUCTR2013-003585-14-AT	2015
Overlap/switch to adefovir monotherapy for lamivudine-resistant patients who responded to combination therapy: a pilot controlled study	Aizawa, , M, Tsubota, A, Fujise, K, Sato, K, Baba, M, Takamatsu, M, Namiki, Y, Ohkusa, T; Tajiri, H	2010
The V-QUIN MDR TRIAL: a randomized controlled trial of six months of daily levofloxacin for the prevention of tuberculosis among household contacts of patients with multi-drug resistant tuberculosis	ACTRN12616000215426	2016
Shockwave treatment for heart failure	EUCTR2013-004333-33-GB	2013
The effect of vitamin D3 and calcium supplementation in treatment of postpartum depression	IRCT2016091416123N9	2017
Dupilumab efficacy and safety in adults with uncontrolled persistent asthma despite use of medium-to-high-dose inhaled corticosteroids plus a long-acting β2 agonist: a randomised double-blind placebo-controlled pivotal phase 2b dose-ranging trial	Wenzel, , S, Castro, M, Corren, J, Maspero, J, Wang, L, Zhang, B, Pirozzi, G, Sutherland, ER, Evans, RR, Joish, VN, Eckert, L, Graham, NM, Stahl, N, Yancopoulos, GD, Louis-Tisserand, M; Teper, A	2016
The analysis of Th2 cell subsets in house dust mite allergic rhinitis patients after sublingual immunotherapy	Ihara, , F, Sakurai, D; Okamoto, Y	2019
Randomized trial of treatment with cisplatin and interleukin-2 either alone or in combination with interferon-alpha-2a in patients with metastatic melanoma: a Federation Nationale des Centres de Lutte Contre le Cancer Multicenter, parallel study	Dorval, , T, Négrier, S, Chevreau, C, Avril, MF, Baume, D, Cupissol, D, Oskam, R, de Peuter, R, Vinke, J, Herrera, A; Escudier, B	1999
INTRAVENOUS IMMUNOGLOBULIN HUMAN FOR THE TREATMENT OF ABORTION	EUCTR2014-005419-18-ES	2016
Implementation of Individualized Medicine: education, Prediction and Treatment	ISRCTN90092922	2014
Differential lipogenic effects of cilostazol and pentoxifylline in patients with intermittent claudication: potential role for interleukin-6	Lee, , TM, Su, SF, Hwang, JJ, Tseng, CD, Chen, MF, Lee, YT; Wang, SS	2001
Anti-TNF discontinuation in patients with inflammatory bowel disease	EUCTR2015-001410-10-ES	2016
Proof-of-Concept study to assess the efficacy, safety and tolerability of SAR440340 (anti-IL-33 mAb) in patients with moderate-to-severe chronic obstructive pulmonary disease (COPD)	EUCTR2017-003290-34-PL	2018
Adipose tissue-derived mesenchymal stromal cells as part of therapy for chronic graft-versus-host disease: a phase I/II study	Jurado, , M, De La Mata, C, Ruiz-García, A, López-Fernández, E, Espinosa, O, Remigia, MJ, Moratalla, L, Goterris, R, García-Martín, P, Ruiz-Cabello, F, Garzón, S, Pascual, MJ, Espigado, I; Solano, C	2017
Comparison of 2 and 4 doses hydrocortison substitution with regard to plasma concentration and quality of life. - CHEQ	EUCTR2005-001768-30-SE	2005

An open label multi facilities cooperation randomized control trial to verify renoprotective effects of pioglitazone therapy in diabetic nephropathy	JPRN-UMIN000001126	2008
Effect of Empagliflozin on Endothelial Function in Cardiovascular High Risk Diabetes Mellitus: multi-Center Placebo-Controlled Double-Blind Randomized Trial	JPRN-UMIN000024502	2016
Evaluation of the efficacy of high throughput genome analysis as a therapeutic decision tool for patients with metastatic non small cell lung cancer - SAFIR 02 Lung	EUCTR2013-001653-27-FR	2015
A study to evaluate the effectiveness and safety of the study drug, when patients are given dosage of ILT-101 with active moderate to severe systemic lupus erythematosus(SLE)	EUCTR2016-000488-17-PT	2016
A Phase 2b study for adults hospitalized with Respiratory Syncytial Virus	EUCTR2016-001653-40-NL	2016
A study to investigate the effects of lumicitabine (JNJ-64041575) in hospitalized infants and children infected with Respiratory Syncytial Virus (RSV)	EUCTR2017-001862-56-PT	2017
A Randomized, Placebo-Controlled, Phase 1b/2 Study of Rilotumumab or Ganitumab in Combination With Platinum-Based Chemotherapy as First-Line Treatment for Extensive-Stage Small-Cell Lung Cancer	Glisson, , B, Besse, B, Dols, MC, Dubey, S, Schupp, M, Jain, R, Jiang, Y, Menon, H, Nackaerts, K, Orlov, S, Paz-Ares, L, Ramlau, R, Tang, R, Zhang, Y; Zhu, M	2017
Selumetinib plus dacarbazine versus placebo plus dacarbazine as first-line treatment for BRAF-mutant metastatic melanoma: a phase 2 double-blind randomised study	Robert, , C, Dummer, R, Gutzmer, R, Lorigan, P, Kim, KB, Nyakas, M, Arance, A, Liszkay, G, Schadendorf, D, Cantarini, M, Spencer, S; Middleton, MR	2013
Randomized phase Ib/II trial of rilotumumab or ganitumab with panitumumab versus panitumumab alone in patients with wild-type KRAS metastatic colorectal cancer	Van Cutsem, , E, Eng, C, Nowara, E, Swieboda-Sadlej, A, Tebbutt, NC, Mitchell, E, Davidenko, I, Stephenson, J, Elez, E, Prenen, H, Deng, H, Tang, R, McCaffery, I, Oliner, KS, Chen, L, Gansert, J, Loh, E, Smethurst, D; Tabernero, J	2014
Effect of Anti-diabetic Drugs on Glycemic Variability	NCT02925559	2016
Comparative Study of Strategies for Management of Duchenne Myopathy (DM)	NCT03633565	2018
Transcranial Magnetic Stimulation for the Treatment of Veterans With Alcohol Use Disorders	NCT03191266	2017
IFN-γ analysis in blood and tissue as a potential prognostic and/or predictive biomarker	Kallinteris, , N, Horn, L, Tang, M, Guenne, T, Yin, S, Lai, J, Shan, J; Sanborn, RE	2017
Fecal microbial transplantation in multiple sclerosis: trial design	Wing, , AC; Kremenchutzky, M	2018
Efficacy, safety, and tolerability of lacosamide in patients with gain-of-function Nav1.7 mutation-related small fiber neuropathy: study protocol of a randomized controlled trial-the LENSS study	de Greef, , BT, Merkies, IS, Geerts, M, Faber, CG; Hoeijmakers, JG	2016
Efficacy of Epratuzumab, an Anti-CD22 Monoclonal IgG Antibody, in Systemic Lupus Erythematosus Patients With Associated Sjogren's Syndrome: post Hoc Analyses From the EMBODY Trials	Gottenberg, , J-E, Dorner, T, Bootsma, H, Devauchelle-Pensec, V, Bowman, SJ, Mariette, X, Bartz, H, Oortgiesen, M, Shock, A, Koetse, W, Galateanu, C, Bongardt, S, Wegener, WA, Goldenberg, DM, Meno-Tetang, G, Kosutic, G; Gordon, C	2018
Efficacy and safety of bilastine in Japanese patients with chronic spontaneous urticaria: a multicenter, randomized, double-blind, placebo-controlled, parallel-group phase II/III study	Hide, , M, Yagami, A, Togawa, M, Saito, A; Furue, M	2017
A Slow- Compared with a Fast-Release Form of Oral Arginine Increases Its Utilization for Nitric Oxide Synthesis in Overweight Adults with Cardiometabolic Risk Factors in a Randomized Controlled Study	Deveaux, , A, Fouillet, H, Petzke, KJ, Hermier, D, André, E, Bunouf, P, Lantoine-Adam, F, Benamouzig, R, Mathé, V, Huneau, JF; Mariotti, F	2016
l-Arginine Supplementation Alleviates Postprandial Endothelial Dysfunction When Baseline Fasting Plasma Arginine Concentration Is Low: a Randomized Controlled Trial in Healthy Overweight Adults with Cardiometabolic Risk Factors	Deveaux, , A, Pham, I, West, SG, André, E, Lantoine-Adam, F, Bunouf, P, Sadi, S, Hermier, D, Mathé, V, Fouillet, H, Huneau, JF, Benamouzig, R; Mariotti, F	2016
Evaluation of a Novel Intervention for Infants At Risk for Neurodevelopmental Disorders	NCT03388294	2018
Schizophrenia with history of alcohol use disorder suffers a worse course of psychosis: results from NIMH catie study analyses	Pathak, , S, Jiang, Y, DiPetrillo, L, Todtenkopf, M, Liu, Y, Silverman, B; Correll, C	2016
Impact of amoxicillin on pneumococcal colonization compared with other therapies for acute otitis media	Toltzis, , P, Dul, M, O'Riordan, MA, Toltzis, H; Blumer, JL	2005
A preliminary study on T-786C endothelial nitric oxide synthase gene and renal hemodynamic and blood pressure responses to dietary sodium	Dengel, , DR, Brown, MD, Ferrell, RE, Reynolds, TH; Supiano, MA	2007
Diagnostic and Prognostic Value of Plasma Levels of Cardiac Myosin Binding Protein-C as a Novel Biomarker in Heart Failure	El Amrousy, , D, Hodeib, H, Suliman, G, Hablas, N, Salama, ER; Esam, A	2017
Improving Chronic Disease Management With Pieces	NCT02587936	2015
Novel treatments for acute asthma	Saglani, S	2017
Effects of serelaxin on the outcome of patients with or without substantial peripheral edema: a subgroup analysis from the RELAX-AHF trial	Gimpelewicz, , C, Metra, M, Cleland, JGF, Szecsödy, P, Chang Wun, CC, Boer-Martins, L, Cotter, G, Davison, BA, Felker, GM, Filippatos, G, Greenberg, BH, Pang, P, Ponikowski, P, Severin, T, Voors, AA; Teerlink, JR	2017
Impact of cocoa consumption on inflammation processes—a critical review of randomized controlled trials	Ellinger, , S; Stehle, P	2016
VTD-melphalan is well tolerated and results in very high rates of stringent CR and MRD-negative status in multiple myeloma	Nadiminti, , K, Singh Abbi, KK, Mott, SL, Dozeman, L, Tricot, A, Schultz, A, Behrends, S, Zhan, F; Tricot, G	2017
Do we treat our patients or rather periodontal microbes with adjunctive antibiotics in periodontal therapy? A 16S rDNA microbial community analysis	Hagenfeld, , D, Koch, R, Jünemann, S, Prior, K, Harks, I, Eickholz, P, Hoffmann, T, Kim, TS, Kocher, T, Meyle, J, Kaner, D, Schlagenhauf, U, Ehmke, B; Harmsen, D	2018

St. John's Wort in the Treatment of Raynaud's Phenomenon	NCT00351117	2006
Baseline analysis of PXT3003 phase 2 data reveals two blood early candidate biomarkers for therapeutic response in CMT1A	Laffaire, , J, Mandel, J, Bertrand, V, Scart-Gres, C, Milet, A, Hajj, R, Chumakov, I, Nabirotchkin, S, Guedj, M; Cohen, D	2015
Intranasal oxytocin reduces provoked symptoms in female patients with posttraumatic stress disorder despite exerting sympathomimetic and positive chronotropic effects in a randomized controlled trial	Sack, , M, Spieler, D, Wizelman, L, Epple, G, Stich, J, Zaba, M; Schmidt, U	2017
Can We Stop Antibiotic Therapy When Signs and Symptoms Have Resolved in Diabetic Foot Infection Patients?	Chu, , Y, Wang, C, Zhang, J, Wang, P, Xu, J, Ding, M, Li, X, Hou, X, Feng, S; Li, X	2015
Suppression of T Cell Activation and Collagen Accumulation by an Anti-IFNAR1 mAb, Anifrolumab, in Adult Patients with Systemic Sclerosis	Guo, , X, Higgs, BW, Bay-Jensen, AC, Karsdal, MA, Yao, Y, Roskos, LK; White, WI	2015
Effects of HDV infection and pegylated interferon α treatment on the natural killer cell compartment in chronically infected individuals	Lunemann, , S, Malone, DF, Grabowski, J, Port, K, Béziat, V, Bremer, B, Malmberg, KJ, Manns, MP, Sandberg, JK, Cornberg, M, Ljunggren, HG, Wedemeyer, H; Björkström, NK	2015
Phase 2 baseline versus treatment clinical trial of the HIV drug raltegravir in patients with active relapsing remitting multiple sclerosis: the INSPIRE study results	Marta, , M, MacManus, D, Yousry, T, Miller, D, Altmann, DR, Meier, UC, Christensen, T, Maruszak, H, Holden, D, Bianchi, L, Adiutori, R, Schmierer, K, Turner, B, Giovannoni, G; Gold, J	2016
The effect of basal insulin glargine on the fibrinolytic system and von Willebrand factor in people with dysglycaemia and high risk for cardiovascular events: swedish substudy of the Outcome Reduction with an Initial Glargine Intervention trial	Rautio, , A, Boman, K, Gerstein, HC, Hernestål-Boman, J, Lee, SF, Olofsson, M; Mellbin, LG	2017
The impact of topical calcipotriol and betamethasone on human β-defensin 2 expression and serum level in psoriatic patients	Abdelmaksood, , R; Hashad, D	2013
EMERGE: a Randomized Phase II Study of the Antibody-Drug Conjugate Glembatumumab Vedotin in Advanced Glycoprotein NMB-Expressing Breast Cancer	Yardley, , DA, Weaver, R, Melisko, ME, Saleh, MN, Arena, FP, Forero, A, Cigler, T, Stopeck, A, Citrin, D, Oliff, I, Bechhold, R, Loutfi, R, Garcia, AA, Cruickshank, S, Crowley, E, Green, J, Hawthorne, T, Yellin, MJ, Davis, TA; Vahdat, LT	2015
Lineage classification of childhood acute lymphoblastic leukemia according to the EGIL recommendations: results of the ALL-BFM 2000 trial	Ratei, , R, Schabath, R, Karawajew, L, Zimmermann, M, Möricke, A, Schrappe, M; Ludwig, WD	2013
Outcome after induction chemotherapy for older patients with acute myeloid leukemia is not improved with mitoxantrone and etoposide compared to cytarabine and daunorubicin: a Southwest Oncology Group study	Anderson, , JE, Kopecky, KJ, Willman, CL, Head, D, O'Donnell, MR, Luthardt, FW, Norwood, TH, Chen, IM, Balcerzak, SP, Johnson, DB; Appelbaum, FR	2002
The natural history of the progression of atrophy secondary to stargardt disease (ProgStar) studies: design and Baseline characteristics: progstar Report No. 1		2016
Epicardial adipose tissue volume increase in hemodialysis patients treated with sevelamer or calcium-based phosphate binders: a substudy of the Renagel in new dialysis trial	Ko, , SM, Zhang, C, Chen, Z, D'Marco, L, Bellasi, A, Stillman, AE, Block, G; Raggi, P	2016
The C677T MTHFR genotypes influence the efficacy of B9 and B12 vitamins supplementation to lowering plasma total homocysteine in hemodialysis	Achour, , O, Elmtaoua, S, Zellama, D, Omezzine, A, Moussa, A, Rejeb, J, Boumaiza, I, Bouacida, L, Rejeb, NB, Achour, A; Bouslama, A	2016
Antiangiogenic therapy in recurrent breast cancer with lymphangitic spread to the chest wall: a randomized phase II trial of bevacizumab with sequential or concurrent oral vinorelbine and capecitabine	Curigliano, , G, Bagnardi, V, Bertolini, F, Alcalay, M, Locatelli, MA, Fumagalli, L, Rabascio, C, Calleri, A, Adamoli, L, Criscitiello, C, Viale, G; Goldhirsch, A	2015
Post-discharge electrocardiogram Holter monitoring in recently hospitalised individuals with chronic atrial fibrillation to enhance therapeutic monitoring and identify potentially predictive phenotypes	Ball, , J, Carrington, MJ, Thompson, DR, Horowitz, JD; Stewart, S	2015
Autologous transplantation as consolidation for high risk aggressive T-cell non-hodgkin's lymphoma: a SWOG S9704 intergroup trial subgroup analysis	Stiff, , PJ, Li, H, Cook, JR, Constine, LS, Couban, S, Stewart, DA, Shea, TC, Porcu, P, Winter, JN, Kahl, BS, Smith, SM, Marcellus, D, Barton, K, Mills, GM, LeBlanc, M, Rimsza, L, Forman, SJ, Leonard, JP, Fisher, RI; Friedberg, J	2016
Short telomeres are independent prognostic factors associated WITH poor outcome IN chronic lymphocytic leukemia (CLL) Treated WITH chlorambucil with or without the addition of rituximab or obinutuzumab: results from CLL11 Trial of the Gcllsg	Chelliah Jebaraj, , BM, Scheffold, A, Robrecht, S, Bahlo, J, Goede, V, Tausch, E, Estenfelder, S, Opatrna, V, Ritgen, M, Van Dongen, JJ, Langerak, AW, Fingerle-Rowson, G, Kneba, M, Fischer, K, Hallek, MJ, Dohner, H; Stilgenbauer, S	2017
Tailored adjunctive cilostazol therapy based on CYP2C19 genotyping in patients with acute myocardial infarction — The CALDERA-GENE study	Kaikita, , K, Yoshimura, H, Ishii, M, Kudoh, T, Yamada, Y, Yamamoto, E, Izumiya, Y, Kojima, S, Shimomura, H, Tsunoda, R, Matsui, K, Ogawa, H; Tsujita, K	2018
α-Smooth muscle actin expression and desmoplastic stromal reaction in pancreatic cancer: results from the CONKO-001 study	Sinn, , M, Denkert, C, Striefler, JK, Pelzer, U, Stieler, JM, Bahra, M, Lohneis, P, Dörken, B, Oettle, H, Riess, H; Sinn, BV	2014
A placebo controlled observer blind immunocytochemical and histologic study of epithelium adjacent to anogenital warts in patients treated with systemic interferon alpha in combination with cryotherapy or cryotherapy alone	Handley, , JM, Maw, RD, Horner, T, Lawther, H, Walsh, M; Dinsmore, WW	1992
NCI#9673 phase II study of nivolumab in refractory metastatic squamous cell carcinoma of the anal canal: immunologic correlates of response	Morris, , V, Mahvash, A, Vence, L, Blando, J, Wolff, RA, Ohinata, A, Ohaji, C, Allison, J, Sharma, P; Eng, C	2016
Improved survival outcomes for patients with extra-nodal NK/T lymphoma: data from 140 patients prospectively registered in the international T-cell project	Fox, , CP, Bellei, M, Manni, M, Kim, SJ, Ko, YH, Shustov, AR, Cabrera, ME, Chiattone, CS, Horwitz, SM, Spina, M, Advani, RH, Angrilli, F, De Souza, CA, Dlouhy, I, Fernandez-Alvarez, R, Gabus, R, Hitz, F, Laszlo, D, Montoto, S, Nagler, A, Pavlovsky, A, Vitolo, U, Zoppegno, L, Federico, M; Kim, WS	2017
DA.R-EPOCH Vs. R-CHOP for high-risk diffuse large b-cell lymphoma: the mayo clinic florida experience	Dholaria, , B, Vishnu, P, Roy, V, Tun, HW, Sher, T, Rivera, CE, Ailawadhi, S, Diehl, N, Hodge, DO; Moreno-Vanegas, YA	2017

Minimal residual disease and outcomes in relapsed/refractory follicular lymphoma (FL) in the phase III gadolin trial of obinutuzumab and bendamustine vs bendamustine	Pott, , C, Belada, D, Danesi, N, Fingerle-Rowson, GR, Gribben, J, Harbron, CG, Hoster, E, Kahl, BS, Kehden, B, Mundt, KE, Nicolas-Virelizier, E, Sehn, LH; Cheson, BD	2017
Role of oxidative stress in diabetic kidney disease	Hakim, , FA; Pflueger, A	2010
High percentage of CD34-positive cells in autologous AML peripheral blood stem cell products reflects inadequate in vivo purging and low chemotherapeutic toxicity in a subgroup of patients with poor clinical outcome	Feller, , N, Schuurhuis, GJ, van der Pol, MA, Westra, G, Weijers, GW, van Stijn, A, Huijgens, PC; Ossenkoppele, GJ	2003
Mutations linked to drug resistance, human immunodeficiency virus type 1 biologic phenotype and their association with disease progression in children receiving nucleoside reverse transcriptase inhibitors	Englund, , JA, Raskino, C, Vavro, C, Palumbo, P, Ross, LL, McKinney, R, Nikolic-Djokic, D, Colgrove, RC; Baker, CJ	2004
Effect of resistance exercise on skeletal muscle myopathy in heart transplant recipients	Braith, , RW, Magyari, PM, Pierce, GL, Edwards, DG, Hill, JA, White, LJ; Aranda, JM	2005
Relationship between measures of infliximab exposure and clinical outcome of infliximab intensification at therapeutic failure in crohn's disease	Steenholdt, , C, Edlund, H, Ainsworth, MA, Brynskov, J, Thomsen, O, Huisinga, W; Kloft, C	2015
Randomized, Double Blind, Placebo-Controlled, Phase IIB Trial of Ketorolac Mouth Rinse Evaluating the Effect of Cyclooxygenase Inhibition on Oropharyngeal Leukoplakia: collaborative Study of the NCI, NIDCD and the NIDCR	NCT00001698	1999
Effect of remote ischemic preconditioning on electrophysiological and biomolecular parameters in nonvalvular paroxysmal atrial fibrillation (RIPPAF study): rationale and study design of a randomized, controlled clinical trial	Kosiuk, , J, Milani, R, Ueberham, L, Uhe, T, Stegmann, C, Hindricks, G; Bollmann, A	2016
Baseline specific IgE levels are useful to predict safety of oral immunotherapy in egg-allergic children	Vazquez-Ortiz, , M, Alvaro, M, Piquer, M, Dominguez, O, Machinena, A, Martin-Mateos, MA; Plaza, AM	2014
A double-blind placebo-controlled trial of antioxidant therapy in limited cutaneous systemic sclerosis	Herrick, , AL, Hollis, S, Schofield, D, Rieley, F, Blann, A, Griffin, K, Moore, T, Braganza, JM; Jayson, MI	2000
Fish Oil-Derived Fatty Acids in Pregnancy and Wheeze and Asthma in Offspring	Bisgaard, , H, Stokholm, J, Chawes, BL, Vissing, NH, Bjarnadóttir, E, Schoos, AM, Wolsk, HM, Pedersen, TM, Vinding, RK, Thorsteinsdóttir, S, Følsgaard, NV, Fink, NR, Thorsen, J, Pedersen, AG, Waage, J, Rasmussen, MA, Stark, KD, Olsen, SF; Bønnelykke, K	2016
Effects of short-term treatment with metformin on markers of endothelial function and inflammatory activity in type 2 diabetes mellitus: a randomized, placebo-controlled trial	De Jager, , J, Kooy, A, Lehert, P, Bets, D, Wulffelé, MG, Teerlink, T, Scheffer, PG, Schalkwijk, CG, Donker, AJ; Stehouwer, CD	2005
The effects of vitamin D supplementation on airway functions in mild to moderate persistent asthma	Arshi, , S, Fallahpour, M, Nabavi, M, Bemanian, MH, Javad-Mousavi, SA, Nojomi, M, Esmaeilzadeh, H, Molatefi, R, Rekabi, M, Jalali, F; Akbarpour, N	2014
Efficacy in Walked Distance of Indacaterol vs Tiotropium in Women With COPD Secondary to Biomass (EMERALD)	NCT02473237	2015
Effect of Periodontal Treatment on HbA1c among Patients with Prediabetes	Kocher, , T, Holtfreter, B, Petersmann, A, Eickholz, P, Hoffmann, T, Kaner, D, Kim, TS, Meyle, J, Schlagenhauf, U, Doering, S, Gravemeier, M, Prior, K, Rathmann, W, Harks, I, Ehmke, B; Koch, R	2019
Clinical signs of epithelial surface disruption impact pain and sexual Health in Patients with moderate-to-severe genital psoriasis	Schloebe, A	2019
Deacetylase inhibitors: an advance in myeloma therapy?	Laubach, , JP, San-Miguel, JF, Hungria, V, Hou, J, Moreau, P, Lonial, S, Lee, JH, Einsele, H, Alsina, M; Richardson, PG	2017
Evaluation of preload functional status and cardiac output in sepsis resuscitation	Douglas, , I, Alapat, P, Corl, K, Exline, M, Forni, L, Holder, A, Kaufman, D, Khan, A, Levy, M, Martin, G, Sahatjian, J, Seely, E, Self, W, Shapiro, N, Williams, M, Winterbottom, C; Hansell, D	2018
Evaluation of a corticotropin releasing hormone type 1 receptor antagonist in women with posttraumatic stress disorder: study protocol for a randomized controlled trial	Dunlop, , BW, Rothbaum, BO, Binder, EB, Duncan, E, Harvey, PD, Jovanovic, T, Kelley, ME, Kinkead, B, Kutner, M, Iosifescu, DV, Mathew, SJ, Neylan, TC, Kilts, CD, Nemeroff, CB; Mayberg, HS	2014
Atezolizumab versus docetaxel for patients with previously treated non-small-cell lung cancer (POPLAR): a multicentre, open-label, phase 2 randomised controlled trial	Fehrenbacher, , L, Spira, A, Ballinger, M, Kowanetz, M, Vansteenkiste, J, Mazieres, J, Park, K, Smith, D, Artal-Cortes, A, Lewanski, C, Braiteh, F, Waterkamp, D, He, P, Zou, W, Chen, DS, Yi, J, Sandler, A; Rittmeyer, A	2016
The impact of allogeneic hematopoietic cell transplantation (HCT) and treatment with ruxolitinib on survival in patients with myelofibrosis	Jaekel, , N, Wang, S-Y, Krahl, R, Hubert, K, Schulze, S, Stengel, R, Schneider, M, Roskos, M, Niederwieser, D; Al-Ali, HK	2015
Up-regulation of the immunoregulatory enzyme indoleamine-2,3-dioxygenase (IDO) with consecutive tryptophan depletion predicts death in patients with neuroendocrine neoplasia	Pschowski, , R, Pape, UF, Fusch, G, Jann, H, Maasberg, S, Arsenic, R, Baur, A, Wiedenmann, B, Pavel, M; Schefold, JC	2016
Targeting human γδ T cells with zoledronate and interleukin-2 for immunotherapy of hormone-refractory prostate cancer	Dieli, , F, Vermijlen, D, Fulfaro, F, Caccamo, N, Meraviglia, S, Cicero, G, Roberts, A, Buccheri, S, D'Asaro, M, Gebbia, N, Salerno, A, Eberl, M; Hayday, AC	2007
Determination of the optimal minimum radioiodine dose in patients with Graves' disease: a clinical outcome study	Howarth, , D, Epstein, M, Lan, L, Tan, P; Booker, J	2001
OASIS-HIT: design of a pharmacogenomic dose-finding study	Staessen, , JA, Kuznetsova, T, Acceto, R, Bacchieri, A, Brand, E, Burnier, M, Celis, H, Citterio, L, de Leeuw, PW, Filipovsky, J, Fournier, A, Kawecka-Jaszcz, K, Manunta, P, Nikitin, Y, O'Brien, ET, Redon, J, Thijs, L, Ferrari, P, Valentini, G; Bianchi, G	2005
Fulranumab in patients with interstitial cystitis/bladder pain syndrome: observations from a randomized, double-blind, placebo-controlled study	Wang, , H, Russell, LJ, Kelly, KM, Wang, S; Thipphawong, J	2017

Pirfenidone in patients with unclassifiable progressive fibrosing interstitial lung disease: design of a double-blind, randomised, placebo-controlled phase II trial	Maher, , TM, Corte, TJ, Fischer, A, Kreuter, M, Lederer, DJ, Molina-Molina, M, Axmann, J, Kirchgaessler, K-U; Cottin, V	2018
Highlights from: the 43rd Annual Meeting of the American Society of Clinical Oncology; Chicago, IL; June 2007		2007
Utilization of an "n-of-1" study design to test the effect of ivacaftor in CF patients with residual CFTR function and FEV1 >40% of predicted	Nick, , JA, Rodman, D, St Clair, C, Jones, MC, Li, H; Higgins, M	2014
The relationship of pharmacodynamics (PD) and pharmacokinetics (PK) to clinical outcomes in a phase I study of OX40 agonistic monoclonal antibody (mAb) PF-04518600 (PF-8600)	El-Khoueiry, , AB, Hamid, O, Thompson, JA, Ros, W, Eskens, F, Doi, T, Hu-Lieskovan, S, Chou, J, Liao, K, Ganguly, BJ, Fleener, C, Joh, T; Diab, A	2017
Effects of Botulinum Toxin Injections in Patients With Hereditary Spastic Paraplegia	NCT02604186	2015
Short Term Effects of Ivacaftor in Non-G551D Cystic Fibrosis Patients	NCT01784419	2013
A multicenter, randomized study of decitabine as epigenetic priming with induction chemotherapy in children with AML	Gore, , L, Triche, TJ, Farrar, JE, Wai, D, Legendre, C, Gooden, GC, Liang, WS, Carpten, J, Lee, D, Alvaro, F, Macy, ME, Arndt, C, Barnette, P, Cooper, T, Martin, L, Narendran, A, Pollard, J, Meshinchi, S, Boklan, J, Arceci, RJ; Salhia, B	2017
Dexamethasone-based therapy for childhood acute lymphoblastic leukaemia: results of the prospective Dutch Childhood Oncology Group (DCOG) protocol ALL-9 (1997-2004)	Veerman, , AJ, Kamps, WA, van den Berg, H, van den Berg, E, Bökkerink, JP, Bruin, MC, van den Heuvel-Eibrink, MM, Korbijn, CM, Korthof, ET, van der Pal, K, Stijnen, T, van Weel Sipman, MH, van Weerden, JF, van Wering, ER; van der Does-van den Berg, A	2009
Phase II study of olaratumab with paclitaxel/carboplatin (P/C) or P/C alone in previously untreated advanced NSCLC	Gerber, , DE, Swanson, P, Lopez-Chavez, A, Wong, L, Dowlati, A, Pennell, NA, Cronier, DM, Qin, A, Ilaria, R, Cosaert, J, Shahir, A; Baggstrom, MQ	2017
SPARC expression in resected pancreatic cancer patients treated with gemcitabine: results from the CONKO-001 study	Sinn, , M, Sinn, BV, Striefler, JK, Lindner, JL, Stieler, JM, Lohneis, P, Bischoff, S, Bläker, H, Pelzer, U, Bahra, M, Dietel, M, Dörken, B, Oettle, H, Riess, H; Denkert, C	2014
PD-L1 expression on circulating CD45(-) cells is an independent prognostic factor for overall survival (OS) in patients (Pts) across all stages of treatment-naive lung cancer in a prospective, multicenter study	Graf, , R, Lu, D, Krupa, R, Louw, J, Dugan, L, Jendrisak, A, Orr, S, Bethel, K, Wang, Y, Suraneni, M, Landers, M, Boffa, D, Nieva, J, Bazhenova, L, Salazar, M, Makani, S, Magana, M; Dittamore, R	2016
Effect of pregabalin on radiotherapy-related neuropathic pain in patients with head and neck cancer: a randomized controlled trial	Jiang, , J, Li, Y, Shen, Q, Rong, X, Huang, X, Li, H, Zhou, L, Mai, H-Q, Zheng, D, Chen, M-Y, Xu, Y, Li, J, Hui, X, Simone, CB, Gaertner, J, Argyriou, AA, Chow, E, Chen, P; Tang, Y	2019
Dynamic changes in CD45RA-Foxp3high regulatory T-cells in chronic hepatitis C patients during antiviral therapy	Li, , Z, Ping, Y, Yu, Z, Wang, M, Yue, D, Zhang, Z, Li, J, Zhang, B, Shi, X; Zhang, Y	2016
Resistance training increases muscle mitochondrial biogenesis in patients with chronic kidney disease	Balakrishnan, , VS, Rao, M, Menon, V, Gordon, PL, Pilichowska, M, Castaneda, F; Castaneda-Sceppa, C	2010
Periodontal treatment effects on endothelial function and cardiovascular disease biomarkers in subjects with chronic periodontitis: protocol for a randomized clinical trial	Ramírez, , JH, Arce, RM; Contreras, A	2011
Variants in the APOA5 gene region and the response to combination therapy with statins and fenofibric acid in a randomized clinical trial of individuals with mixed dyslipidemia	Brautbar, , A, Covarrubias, D, Belmont, J, Lara-Garduno, F, Virani, SS, Jones, PH, Leal, SM; Ballantyne, CM	2011
Ascorbic acid for Charcot-Marie-Tooth disease type 1A in children: a randomised, double-blind, placebo-controlled, safety and efficacy trial	Burns, , J, Ouvrier, RA, Yiu, EM, Joseph, PD, Kornberg, AJ, Fahey, MC; Ryan, MM	2009
Effect of Self- and Family Management of Diabetic Foot Ulcers Programs on Health Outcomes	NCT03909802	2019
A placebo-controlled trial of silymarin in patients with nonalcoholic fatty liver disease	Hashemi, , SJ, Hajiani, E; Sardabi, EH	2009
Early peripheral blast cell clearance assessed by flow cytometry in induction is a novel powerful prognostic indicator in acute myeloid leukemia: a northern Italy leukemia group (NILG) study	Gianfaldoni, , G, Mannelli, F, Intermesoli, T, Cutini, I, Giupponi, D, Bencini, S, Bonetti, MI, Masciulli, A, Marmont, F, Ferrero, D, Cavazzina, R, Bosi, A, Rambaldi, A; Bassan, R	2014
In Children With Nonalcoholic Fatty Liver Disease, Cysteamine Bitartrate Delayed Release Improves Liver Enzymes but Does Not Reduce Disease Activity Scores	Wilson, , LA, Neuschwander-Tetri, BA, Xanthakos, SA, Barlow, SE, Vos, MB, Karpen, SJ, Jain, AK, Murray, KF, Van Natta, ML, Doo, E, Abrams, SH, Himes, R, Krisnamurthy, R, Maldonado, L, Mahabir, R, Bernstein, K, Bramlage, K, Cecil, K, DeVore, S, Kohli, R, Lake, K, Podberesky, D, Towbin, A, Xanthakos, S, Behr, G, Lavine, JE, Lefkowitch, JH, Mencin, A, Reynoso, E, Alazraki, A, Cleeton, R, Karpen, S, Cruz Munos, J, Raviele, N, Vos, M, Bozic, M, Cummings, OW, Klipsch, A, Molleston, JP, Munson, S, Sandrasegaran, K, Subbarao, G, Kafka, K, Scheimann, A, Amsden, K, Fishbein, MH, Kirwan, E, Mohammad, S, Rigsby, C, Sharda, L, Whitington, PF, Barlow, S, Derdoy, J, Jain, A, King, D, Osmack, P, Siegner, J, Stewart, S, Torretta, S, Wriston, K, Baker, SS, Zhu, L, Africa, J, Angeles, J, Arroyo, S, Awai, H, Behling, C, Bross, C, Durelle, J, Middleton, M, Newton, K, Paiz, M, Sanford, J, Schwimmer, JB, Sirlin, C, Ugalde-Nicalo, P, Villarreal, MD, Aouizerat, B, Courtier, J, Ferrell, LD, Fleck, S, Gill, R, Langlois, C, Perito, ER, Rosenthal, P, Tsai, P, Cooper, K, Horslen, S, Hsu, E, Murray, K, Otto, R, Yeh, M, Young, M, Brunt, EM, Fowler, K, Kleiner, DE, Brown, S, Doo, EC, Hoofnagle, JH, Robuck, PR, Sherker, A, Torrance, R, Belt, P, Clark, JM, Donithan, M, Hallinan, E, Isaacson, M, May, KP, Miriel, L, Sternberg, A, Tonascia, J, Van Natta, M, Vaughn, I, Wilson, L; Yates, K	2016

Phase 2, randomised placebo-controlled trial to evaluate the efficacy and safety of an anti-GM-CSF antibody (KB003) in patients with inadequately controlled asthma	Molfino, , NA, Kuna, P, Leff, JA, Oh, CK, Singh, D, Chernow, M, Sutton, B; Yarranton, G	2016
Ready-to-use food supplement, with or without arginine and citrulline, with daily chloroquine in Tanzanian children with sickle-cell disease: a double-blind, random order crossover trial	Cox, , SE, Ellins, EA, Marealle, AI, Newton, CR, Soka, D, Sasi, P, Luca Di Tanna, G, Johnson, W, Makani, J, Prentice, AM, Halcox, JP; Kirkham, FJ	2018
Anti-oxidative treatment with vitamin E improves peripheral vascular function in patients with diabetes mellitus and Haptoglobin 2-2 genotype: a double-blinded cross-over study	Alshiek, , JA, Dayan, L, Asleh, R, Blum, S, Levy, AP; Jacob, G	2017
Rituximab improves peripheral B cell abnormalities in human systemic lupus erythematosus	Anolik, , JH, Barnard, J, Cappione, A, Pugh-Bernard, AE, Felgar, RE, Looney, RJ; Sanz, I	2004
Assessment of the Effects of a DPP-4 Inhibitor (Sitagliptin) Januvia on Immune Function in Healthy Individuals	NCT00813228	2008
Long-term clinical effects of CFTR Co-therapy with Lumacaftor/Ivacaftor	De Boeck, C	2015
Risk adjusted management of newly diagnosed high and intermediate flipi follicular lymphoma using (90)Y ibritumomab tiuxetan	Berinstein, , NL, Pennell, NM, Weerasinghe, R, Cheung, MC, Piliotis, E, Imrie, KR, Cussen, M-A, Buckstein, R, Ghorab, Z, Miles, E; Reis, MD	2016
Role of cytomegalovirus in the expansion of CD4+CD28-T cells in patients with ANCA-associated vasculitis: a proof-of-concept, randomised controlled trial	Chanouzas, , D, Sagmeister, M, Dyall, L, Nightingale, P, Ferro, C, Moss, P, Morgan, M; Harper, L	2017
Evaluation of the H1-Coil TMS- Double Blind Placebo Controlled Study in Adolescent Patients Suffering From Major Depression	NCT03541707	2018
Individualized therapy for persistent asthma in young children	Fitzpatrick, , AM, Jackson, DJ, Mauger, DT, Boehmer, SJ, Phipatanakul, W, Sheehan, WJ, Moy, JN, Paul, IM, Bacharier, LB, Cabana, MD, Covar, R, Holguin, F, Lemanske, RF, Martinez, FD, Pongracic, JA, Beigelman, A, Baxi, SN, Benson, M, Blake, K, Chmiel, JF, Daines, CL, Daines, MO, Gaffin, JM, Gentile, DA, Gower, WA, Israel, E, Kumar, HV, Lang, JE, Lazarus, SC, Lima, JJ, Ly, N, Marbin, J, Morgan, W, Myers, RE, Olin, JT, Peters, SP, Raissy, HH, Robison, RG, Ross, K, Sorkness, CA, Thyne, SM; Szefler, SJ	2016
Dose adjusted repoch in untreated de novo diffuse large B cell lymphoma-A single centre singapore experience	Tso, , ACY, Sampath, VS; Ong, KH	2016
Is It Better to Use Ipilimumab Combined With a PD-1 Inhibitor or a PD-1 Inhibitor Alone as Initial Immunotherapy in Patients With Metastatic Melanoma?	Espindola, , LMD, Salhab, RM, Dos Anjos, C, Garicochea, B; Munhoz, RR	2017
Repeated application of gene therapy in patients with cystic fibrosis	EUCTR2011-004761-33-GB	2011
The effect of lacosamide in peripheral neuropathic pain: a randomized, double-blind, placebo-controlled, stratified beased on clinical characteristics	EUCTR2018-003110-40-DK	2018
A Randomized, Open Label, Multicenter, Phase III, 2-Arm Study of Androgen Deprivation with Leuprolide, +/- Docetaxel for Clinically Asymptomatic Prostate Cancer Subjects with a Rising PSA Following Definitive Local Therapy	EUCTR2007-000323-17-SK	2008
Randomized study with a run-in feasibility phase to assess the added value of Clofarabine in combination with standard remission-induction chemotherapy in patients aged 18-65 years with previously untreated acute myeloid leukemia (AML) or myelodysplasia (MDS) (RAEB with IPSS = 1.5)	EUCTR2009-011613-24-NL	2009
Study of the efficacy and safety indicators of two different iron chelators in patients with iron overload (Estudio de los indicadores de eficacia y seguridad de dos quelantes del hierro en pacientes con sobrecarga ferrica)	ISRCTN52984371	2011
Clinical effectiveness and effect on COX-2 expression of the sequential treatment of actinic keratoses (AK) with photodynamic therapy (PDT) and diclofenac 3% gel: a phase IV clinical trial	EUCTR2013-000110-37-ES	2013
To investigate whether treatment with the SGLT-2 inhibitor dapagliflozin improves the perception of hypoglycemia in people with type 1 diabetes with impaired awareness of hypoglycemia	EUCTR2018-001569-17-NL	2018
A Prospective randomised trial of sentinel node biopsy for high risk non-melanoma skin cancer	ACTRN12611000713998	2011
This clinical research study will assess the effectiveness (how well it works), safety, tolerability (how well the body stands the drug) and blood levels of combined doses of BMS-663068, Raltegravir and Tenofovir	EUCTR2011-000437-36-ES	2011
GWMD09126 - GWP42003: GWP42004 antipsychotic(s) weight gain treatment	EUCTR2011-000180-28-GB	2011
Risk-stratified randomized controlled trial in paediatric Crohn's Disease: methotrexate versus azathioprine or adalimumab for maintaining remission in patients at low or at high risk for aggressive disease course, respectively – a treatment strategy	EUCTR2016-000522-18-PL	2018
14F7 in chronic B-cell lymphoproliferative syndrome	RPCEC00000266	2018
mRNA-transfected dendritic cell vaccination in high risk uveal melanoma patients - mRNA-DC vaccination in uveal melanoma	EUCTR2008-001974-33-NL	2008
TWO study: cell therapy trial in renal transplantation	ISRCTN11038572	2018

A multicentre, multinational, double-blind, randomised, parallel group, placebo controlled trial of ethyl-EPA ethyl-Icosapent in patients with Huntington s Disease - ND	EUCTR2005-002088-98-IT	2006
Combining a TLR9 agonist with broadly neutralizing antibodies for reservoir reduction and immunological control of HIV infection: an investigator-initiated randomized, placebo-controlled, phase IIa trial (TITAN)	EUCTR2018-001165-16-DK	2018
Vitamin D action in the treatment of Depression and reduction of Cardiovascular Risk	RBR-6yj8sj	2018
Inhaler technology study	ISRCTN90986892	2017
Effects of JAK inhibition on rheumatoid arthritis-related comorbidities: link between bone and vascular effects (investigator-initiated grant proposal)	EUCTR2015-002523-26-HU	2015
Impact of Tight blood Glucose Control within normal fasting ranges with insulin titration prescribed by the Leuven algorithm in adult critically ill patients	EUCTR2018-000756-17-BE	2018
Saline hypertonic in preschoolers and lung structure as measured by computed tomography	EUCTR2015-004143-39-NL	2015
A Phase IIa randomized, partially blinded trial of telaprevir (VX-950) in treatment-naïve subjects with chronic genotype 4 hepatitis C infection	EUCTR2007-005281-12-FR	2007
ReBeL study: a randomized phase I/II trial of lenalidomide and rituximab with or without bendamustine in patients = 18 years with relapsed follicular lymphoma. A HOVON/GLSG study	NTR3028	2011
Multicenter, randomized, prospective study to establish the clinical efficacy and the mechanisms of tolerance following immunosuppression withdrawal in liver transplantation	EUCTR2017-004983-37-ES	2018
Mediastinal large B-cell lymphoma: clinical and immunohistological findings in 18 patients treated with different third-generation regimens	Falini, , B, Venturi, S, Martélli, M, Santucci, A, Pileri, S, Pescarmona, E, Giovannini, M, Mazza, P, Martelli, MF; Pasqualucci, L	1995
MULTICENTER STUDY WITH RANDOM DISTRIBUTION OF TWO TREATMENTS USED IN PARALLEL TO DEMOSTRATE THE EFFICACY AND SAEFTY OF A SOLUTION OF CLODRONATE 200mg / 4 ml ADMINISTRED INTRAMUSCULARLY ONCE EVERY TWO WEEKS WITH 1% LIDOCAINE VS CLODRONATE 100 MG/3,3ML ADMINISTRED INTRAMUSCOLARY ONCE A WEEK WITH 1% LIDOCAINE IN OF WOMEN WITH POSTMENOPAUSAL OSTEOPOROSIS	EUCTR2010-022060-13-IT	2011
Eplerenone in Metabolic Syndrome: an investigation into the effects of Eplerenone on perivascular adipose tissue and small artery tone in obesity - Eplerenone in Metabolic Syndrome	EUCTR2010-022308-34-GB	2011
Low-dose agalsidase beta treatment in male pediatric patients with Fabry disease: a 5-year randomized controlled trial	Ramaswami, , U, Bichet, DG, Clarke, LA, Dostalova, G, Fainboim, A, Fellgiebel, A, Forcelini, CM, An Haack, K, Hopkin, RJ, Mauer, M, Najafian, B, Scott, CR, Shankar, SP, Thurberg, BL, Tondel, C, Tylki-Szymanska, A, Benichou, B; Wijburg, FA	2019
Clinical efficacy and safety of R0002 cream in the initial and maintenance therapies of lamellar ichthyosis (LI)	ISRCTN62315004	2011
A Phase I/II, Ascending, Multi-Dose Study of BMS-663513, An Agonistic Anti-CD137 Monoclonal Antibody, Administered Every Three Weeks to Patients with Metastatic or Locally Advanced Solid Malignancies. Revised Protocol Number 07, incorporating Amendments 01, 02, 03, 04, 05, 06, 07 and Administrative Letter Dated 10-Mar-2006	EUCTR2007-000416-91-FR	2007
Study of the efficacy and safety of the combination of rituximab and CMC544 with rituximab, gemcitabine and oxaliplatine in diffuse large B-cell lymphoma at first or second relapse	EUCTR2011-003849-18-BE	2012
The randomized controlled trial of comparing the efficacy of pegaspargase+CHOP regimen or Hyper-CVAD regimen in the incipient peripheral T-cell lymphoma	ChiCTR-TRC-14004426	2014
A PHASE 2B RANDOMIZED, ACTIVE-CONTROLLED, STAGED, OPEN-LABEL TRIAL TO INVESTIGATE SAFETY AND EFFICACY OF BMS-955176 IN COMBINATION WITH DOLUTEGRAVIR AND ATAZANAVIR (WITH OR WITHOUT RITONAVIR) IN TREATMENT-EXPERIENCED HIV-1 INFECTED ADULTS	PER-019-15	2015
ACT-1 (younger patients) A randomized phase III study to evaluate the efficacy of chemoimmunotherapy with the monoclonal antibody Campath-1H (Alemtuzumab) given in combination with 2-weekly CHOP versus 2-weekly CHOP alone and consolidated by autologous stem cell transplant, in young patients with previously untreated systemic peripheral T-cell lymphomas - ACT-1 (Alemtuzumab and CHOP in T-cell lymphoma)	EUCTR2006-006130-17-PT	2008
Double-blind crossover trial of trimethoprim-sulfamethoxazole in spinocerebellar ataxia type 3/Machado-Joseph disease	Schulte, , T, Mattern, R, Berger, K, Szymanski, S, Klotz, P, Kraus, PH, Przuntek, H; Schöls, L	2001
MARCH	EUCTR2011-002107-15-IE	2011
A phase II study of Nivolumab for relapsed/refractory adult T-cell leukemia/lymphoma (ATL)	JPRN-UMIN000020601	2016
Long Term Administration of Inhaled Mannitol in Cystic Fibrosis – A Safety and Efficacy Trial in Adult Cystic Fibrosis Subjects	EUCTR2013-005357-79-CZ	2014

A Phase III, Multicenter, Placbo-Controlled, Double-Blind, Randomized Clinical Trial to Evaluate the Efficacy of Bevacizumab in Combination with Tarceva (erlotinib) Compared With Tarceva Alone For Treatment of Advanced Non-Small Cell Lung Cancer (NSCLC) After Failure of Standard First-Line Chemotherapy	EUCTR2006-006626-26-SE	2007
Study of the safety and effectiveness of drug AZD4017 at reducing pressure in the brain, in idiopathic intracranial hypertension	EUCTR2013-003643-31-GB	2013
Osteopathic effects in patientes with heart disease	RBR-3st9f3	2015
A 36 wk three-center double-blind randomized three-way cross-over trial comparing metabolic effects of candesartan, hydrochlorothiazide and placebo. The MEDICA study Mechanisms for the diabetes-preventing effect of candesartan - The MEDICA study	EUCTR2004-005013-11-SE	2004
Topical ionic contra-viral therapy in actinic keratosis	EUCTR2018-000034-36-NL	2018
THE ROLE OF HYPERGLYCEMIA, HYPERINSULINEMIA AND ELEVATED FREE FATTY ACIDS FOR CARDIAC FUNCTION IN PATIENTS WITH TYPE 2 DIABETES	EUCTR2017-002101-35-DK	2017
Selected mesenchymal stromal cells to reduce liver inflammation	ISRCTN73586959	2017
A prospective phase I/II study to evaluate allogeneic mesenchymal stromal cells for the treatment of skin disease in children with recessive dystrophic epidermolysis bullosa	EUCTR2012-001394-87-GB	2012
HIV Reservoir Dynamics After Switching To Dolutegravir in Patients on a PI/r Based Regimen. A Phase IV Open Randomized Trial	EUCTR2014-004331-39-ES	2014
Efficacy of melatonin in rapid eye movement (REM sleep) behaviour disorder with coexisting Parkinsonism	ACTRN12613000648729	2013
A clinical trial looking at the safety and effectiveness of a new drug for inhalation, looking to treat Alpha-1 Antitrypsin Deficient Patients with Emphysema	EUCTR2008-005326-36-DK	2009
A randomised, open-label study to evaluate the efficacy and safety of maraviroc (MVC) as a switch for either nucleoside or nucleotide analogue reverse transcriptase inhibitors (N(t)RTI) or boosted protease inhibitors (PI/r) in HIV-1 infected individuals with stable, well-controlled plasma HIV-RNA while taking their first N(t)RTI + PI/r regimen of combination antiretroviral therapy (cART): MARCH study	ACTRN12611000816954	2011
Optimal pathway for treating neuropathic pain in diabetes mellitus	EUCTR2016-003146-89-GB	2018
Double-Blind, parallel-group comparison, investigators initiated phase II clinical trial of IDEC-C2B8 (Rituximab) in patients with Systemic Sclerosis	JPRN-UMIN000030139	2017
Effects of exercise training on the human body	RBR-3yxds4	2016
Proof of biological activity of SAR100842 in Systemic Sclerosis	EUCTR2012-001369-34-IT	2012
T4 immunotherapy of head and neck cancer	ISRCTN81726461	2015
A Phase II Study of Intravenous CNTO328 in Patients with Recurrent Epithelial Ovarian Cancer - CNTO328 in ovarian cancer	EUCTR2006-005704-13-GB	2007
A Phase I/II clinical study of IMA970A plus CV8102 and Cyclophosphamide for patients with liver cancer	EUCTR2015-003389-10-DE	2017
Comparing treatments for severe chronic hand eczema	EUCTR2014-004741-27-GB	2015
A clinical study to test how safe, tolerable and effective MT-8554 is for patients with Painful Diabetic Peripheral Neuropathy	EUCTR2016-002571-10-HU	2017
Strength Training for patients with Parkinson's Disease identified by a biochemical examination	RBR-36cw3y	2017
A study to compare masitinib with dexamethasone and gemcitabine in the treatment of patients with relapsed or refractory peripheral T-cell lymphoma	EUCTR2010-021091-28-SK	2015
Phase II trial in platinum-refractory ovarian cancer: a randomized multicenter trial with SU11248 to evaluate dosage, tolerability, toxicity and effectiveness of a multitargeted receptor tyrosine kinase inhibitor monotherapy	EUCTR2007-003089-16-DE	2008
Efficacy and safety of canakinumab in patients with Schnitzler syndrome	EUCTR2010-024156-28-DE	2011
Clinical trial with L-asparaginase encapsulated in erythrcoytes in patients affected by metastatic pancreatic cancer after first line treatment	EUCTR2013-004262-34-FR	2014
Comparison of short infusion versus prolonged infusion of ceftolozane-tazobactam among patients with ventilator associated-pneumonia to Pseudomonas aeruginosa in intensive care units	EUCTR2018-000059-42-FR	2018
Belimumab after B Cell depletion therapy as a new treatment for patients with systemic lupus erythematosus (SLE)	ISRCTN47873003	2016
PPI in secondary hemochromatosis	EUCTR2017-003777-34-NL	2018
overall survival of patients with recurrent ovarian, primary peritoneal or fallopian tube cancers of BRCA mutated or BRCAness phenotype treated with trabectedin compared to a standard treatment	EUCTR2015-004472-30-IT	2017
Personalized treatment of functional dyspepsia with nortriptyline	EUCTR2017-003307-21-NL	2017

Proof of concept trial of the role of clopidogrel in preventing allergen induced inflammation in asthma	EUCTR2012-003698-25-GB	2012
Efficacité et Tolérance de la Plitidepsine chez les patients souffrant d'un liposarcome dédifférencié (DLPS) avancé, non opérable ou métastatique, en rechute/réfractaire : une étude exploratoire multicentrique de phase II	EUCTR2009-010980-18-FR	2009
Periodontitis treatment in patients with reumatoid arthritis and high clinical activity in the. Randomized clinical trial	EUCTR2017-003259-40-ES	2017
Capecitabine oral chemotherapy with radium-223 in breast cancer patients with bone metastases (CARBON)	ISRCTN92755158	2016
A phase II double-blind, randomised, placebo-controlled trial of neuroprotection with phenytoin in acute optic neuritis	Raftopoulos, , RE, Hickman, S, Toosy, A, Wheeler-Kingshott, CA, Altmann, D, Schmierer, K, Sharrack, B, Sheridan, R, Giovannoni, G, Miller, DH; Kapoor, R	2012
The effect of mud pack therapy on serum YKL-40 and hsCRP levels in patients with knee osteoarthritis	Güngen, , G, Ardic, F, Fındıkoğlu, G; Rota, S	2012
MR and CT imaging characteristics and ablation zone volumetry of locally advanced pancreatic cancer treated with irreversible electroporation	Vroomen, , LGPH, Scheffer, HJ, Melenhorst, MCAM, de Jong, MC, van den Bergh, JE, van Kuijk, C, van Delft, F, Kazemier, G; Meijerink, MR	2017
The role of circulating HPV-16 DNA detection in HNSCC patients treated by definitive radiation or chemo-radiation	Hajduk, , AB, Rutkowski, T, Mazurek, A; Skladowski, K	2015
Peripheral parameters of oxidative stress in patients with infiltrative Graves' ophthalmopathy treated with corticosteroids	Bednarek, , J, Wysocki, H; Sowiński, J	2004
Anorexia nervosa treatment: a systematic review of randomized controlled trials	Bulik, , CM, Berkman, ND, Brownley, KA, Sedway, JA; Lohr, KN	2007
Evaluation of a commercial web-based weight loss and weight loss maintenance program in overweight and obese adults: a randomized controlled trial	Collins, , CE, Morgan, PJ, Jones, P, Fletcher, K, Martin, J, Aguiar, EJ, Lucas, A, Neve, M, McElduff, P; Callister, R	2010
Switch Rates During Acute Treatment for Bipolar II Depression With Lithium, Sertraline, or the Two Combined: a Randomized Double-Blind Comparison	Altshuler, , LL, Sugar, CA, McElroy, SL, Calimlim, B, Gitlin, M, Keck, PE, Aquino-Elias, A, Martens, BE, Fischer, EG, English, TL, Roach, J; Suppes, T	2017
Relation between augmentation index and adiponectin during one-year metformin treatment for nonalcoholic steatohepatosis: effects beyond glucose lowering?	Shargorodsky, , M, Omelchenko, E, Matas, Z, Boaz, M; Gavish, D	2012
Thalidomide treatment for prurigo nodularis in human immunodeficiency virus-infected subjects: efficacy and risk of neuropathy	Maurer, , T, Poncelet, A; Berger, T	2004
Dapagliflozin maintains glycaemic control while reducing weight and body fat mass over 2 years in patients with type 2 diabetes mellitus inadequately controlled on metformin	Bolinder, , J, Ljunggren, Ö, Johansson, L, Wilding, J, Langkilde, AM, Sjöström, CD, Sugg, J; Parikh, S	2014
Flaxseed Lignan-Enriched Complex (FLC) for the Treatment of Patients With Ulcerative Colitis (UC): a Pilot Assessment	NCT02201758	2014
Pantoprazole rapidly improves health-related quality of life in patients with heartburn: a prospective, randomized, double blind comparative study with nizatidine	Paré, , P, Armstrong, D, Pericak, D; Pyzyk, M	2003
Abstracts from the International Combined Meeting BHS-SIdA		2015
Changes in inflammatory mediators in gingival crevicular fluid following periodontal disease treatment in pregnancy: relationship to adverse pregnancy outcome	Penova-Veselinovic, , B, Keelan, JA, Wang, CA, Newnham, JP; Pennell, CE	2015
A randomized, phase 2 trial of Docetaxel with or without PX-866, an irreversible oral phosphatidylinositol 3-kinase inhibitor, in patients with relapsed or metastatic non-small-cell lung cancer	Levy, , B, Spira, A, Becker, D, Evans, T, Schnadig, I, Camidge, DR, Bauman, JE, Hausman, D, Walker, L, Nemunaitis, J, Rudin, CM, Halmos, B; Bowles, DW	2014
Effect of Long Term α-Lipoic Acid Treatment on Endothelial Function in Patients With Type 2 Diabetes	NCT00490867	2007
Montelukast reduces serum levels of eosinophil-derived neurotoxin in preschool asthma	Kim, , C-K, Callaway, Z, Park, J-S, Nishimori, H, Ogino, T, Nagao, M; Fujisawa, T	2018
Clinical outcome and atherothrombogenic risk profile after prolonged wash-out following long-term treatment with high doses of n-3 PUFAs in patients with an acute myocardial infarction	Grundt, , H, Nilsen, DW, Hetland, Ø; Mansoor, MA	2004
Motivational Interviewing and Cognitive Behavioural Therapy for Generalized Anxiety Disorder	NCT01488799	2011
Probiotic dietary supplementation in patients with stage 3 and 4 chronic kidney disease: a 6-month pilot scale trial in Canada	Ranganathan, , N, Friedman, EA, Tam, P, Rao, V, Ranganathan, P; Dheer, R	2009
Maternal-fetal outcomes of lamivudine treatment administered during late pregnancy to highly viremic mothers with HBeAg+ chronic hepatitis B	Jiang, , HX, Han, GR, Wang, CM; Ji, Y	2012
Efficacy of controlled-release niacin in treatment of metabolic syndrome: correlation to surrogate markers of atherosclerosis, vascular reactivity, and inflammation	Vaccari, , CS, Nagamia, S, Thoenes, M, Oguchi, A, Hammoud, R; Khan, BV	2007
Oxidative stress in hemodialysis patients receiving intravenous iron therapy and the role of N-acetylcysteine in preventing oxidative stress	Swarnalatha, , G, Ram, R, Neela, P, Naidu, MU; Dakshina Murty, KV	2010
Assessment of glomerular filtration rate based on alterations of serum brain-derived neurotrophic factor in type 2 diabetic subjects treated with amlodipine/benazepril or valsartan/hydrochlorothiazide	Lee, , I-T, Sheu, WH-H, Hung, Y-J, Chen, J-F, Wang, C-Y; Lee, W-J	2015
Effects of an antioxidant-enriched multivitamin supplement on inflammation and oxidative stress in CF	Sagel, , SD, Baines, A, Abdulhamid, I, Borowitz, D, Clancy, JP, Daines, C, Dunitz, JM, Graff, GR, Jain, R, Livingston, F, McCoy, KS, Noe, J, Orenstein, DM, Rock, MJ, Slovis, BS, Strausbaugh, S; Khan, U	2016

Usefulness of HFA-BDP for adult patients with bronchial asthma: randomized crossover study with fluticasone	Horiguchi, , T, Hayashi, N, Ohira, D, Torigoe, H, Ito, T, Hirose, M, Sasaki, Y, Shiga, M, Miyazaki, J, Kondo, R; Tachikawa, S	2006
A double-blind, randomized, placebo-controlled trial evaluating safety and efficacy of an ayurvedic botanical formulation in reducing menopausal symptoms in otherwise healthy women	Steels, , E, Steele, M, Harold, M, Adams, L; Coulson, S	2018
Clinical trials for drugs against diabetic neuropathy: can we combine scientific needs with clinical practicalities?	Ziegler, , D; Luft, D	2002
Longitudinal Antimalarial Combinations in Uganda	NCT00123552	2005
PRECREST: a phase II prevention and biomarker trial of creatine in at-risk Huntington disease	Rosas, , HD, Doros, G, Gevorkian, S, Malarick, K, Reuter, M, Coutu, JP, Triggs, TD, Wilkens, PJ, Matson, W, Salat, DH; Hersch, SM	2014
Azithromycin for episodes with asthma-like symptoms in young children aged 1-3 years: a randomised, double-blind, placebo-controlled trial	Stokholm, , J, Chawes, BL, Vissing, NH, Bjarnadottir, E, Pedersen, TM, Vinding, RK, Schoos, A-MM, Wolsk, HM, Thorsteinsdottir, S, Hallas, HW, Arianto, L, Schjorring, S, Krogfelt, KA, Fischer, TK, Pipper, CB, Bonnelykke, K; Bisgaard, H	2016
Acute effects of continous positive airway pressure (CPAP)in hemodynamics and cardiac performance in patients with hypertrophic cardiomyopathy	Nerbass, , FB, Pedrosa, RP, Ferreira Filho, JA, Salemi, VMC, Arteaga-Fernandez, E, Drager, LF; Lorenzi-Filho, G	2013
Effects of triple antioxidant therapy on measures of cardiovascular autonomic neuropathy and on myocardial blood flow in type 1 diabetes: a randomised controlled trial	Pop-Busui, , R, Stevens, MJ, Raffel, DM, White, EA, Mehta, M, Plunkett, CD, Brown, MB; Feldman, EL	2013
Joalis Analerg Bambi - Alimentary supplement in the treatment of allergic diseases (Multicentric double blind, placebo-controlled study)	Bystron, , J, Kopriva, F; Smekalova, A	2002
Intrahepatic G-CSF stimulated autologous bone marrow stem cell transplantation versus standard of care in patients with decompensated alcoholic liver disease: a randomised study	Chalandon, , Y, Terraz, S, Kindler, V, Passweg, J, Rubbia-Brandt, L, Hadengue, A; Spahr, L	2012
The benefits and tolerance of exercise in myasthenia gravis (MGEX): study protocol for a randomised controlled trial	Birnbaum, , S, Hogrel, J-Y, Porcher, R, Portero, P, Clair, B, Eymard, B, Demeret, S, Bassez, G, Gargiulo, M, Berrih-Aknin, S, Jobic, A, Aegerter, P, Thoumie, P, Sharshar, T, Behin, A, Bolgert, F, Younes, ND-A, Domingo, N-Y, Friedman, D, Frenkian, M, Laforet, P, Ledoux, I, Louet, E, Mendelson, J, Misdrahi, S, Bedos, CO, Rohaut, B, Soler, E, Truffault, F, Weiss, N; William, L	2018
Effect of exercise training on postexercise oxygen uptake kinetics in patients with reduced ventricular function	Myers, , J, Gianrossi, R, Schwitter, J, Wagner, D; Dubach, P	2001
Endocan, a novel marker of endothelial dysfunction in patients with essential hypertension: comparative effects of amlodipine and valsartan	Celık, , T, Balta, S, Karaman, M, Ahmet Ay, S, Demırkol, S, Ozturk, C, Dınc, M, Unal, HU, Yılmaz, MI, Kılıc, S, Kurt, G, Tas, A, Iyısoy, A, Quartı-Trevano, F, Fıcı, F; Grassı, G	2015
A randomized, controlled, molecular study of condylomata acuminata clearance during treatment with imiquimod	Tyring, , SK, Arany, I, Stanley, MA, Tomai, MA, Miller, RL, Smith, MH, McDermott, DJ; Slade, HB	1998
The Randomized Olmesartan and Diabetes Microalbuminuria Prevention (ROADMAP) observational follow-up study: benefits of RAS blockade with olmesartan treatment are sustained after study discontinuation	Menne, , J, Ritz, E, Ruilope, LM, Chatzikyrkou, C, Viberti, G; Haller, H	2014
The combined effect of Parathyroid hormone (1-34) and whole-body Vibration exercise in the treatment of OSteoporosis (PaVOS)- study protocol for a randomized controlled trial	Jepsen, , DB, Ryg, J, Jorgensen, NR, Hansen, S; Masud, T	2018
Effects of ginger on serum glucose, advanced glycation end products, and inflammation in peritoneal dialysis patients	Imani, , H, Tabibi, H, Najafi, I, Atabak, S, Hedayati, M; Rahmani, L	2015
A systematic review: thiopurine monitoring in children with inflammatory bowel disease	Konidari, , A, Anagnostopoulos, A, Bonnett, LJ, Pirmohamed, M; El Matary, W	2013
Peripheral CD5+ B cells in antineutrophil cytoplasmic antibody-associated vasculitis	Unizony, , S, Lim, N, Phippard, DJ, Carey, VJ, Miloslavsky, EM, Tchao, NK, Iklé, D, Asare, AL, Merkel, PA, Monach, PA, Seo, P, St Clair, EW, Langford, CA, Spiera, R, Hoffman, GS, Kallenberg, CG, Specks, U; Stone, JH	2015
The Effects of Testosterone Supplementation on Cognitive Functioning in Older Men	Wahjoepramono, , EJ, Asih, PR, Aniwiyanti, V, Taddei, K, Dhaliwal, SS, Fuller, SJ, Foster, J, Carruthers, M, Verdile, G, Sohrabi, HR; Martins, RN	2016
Maternal plasma interleukin-6, interleukin-1β and C-reactive protein as indicators of tocolysis failure and neonatal outcome after preterm delivery	Skrablin, , S, Lovric, H, Banovic, V, Kralik, S, Dijakovic, A; Kalafatic, D	2007
Triple pharmacological blockade of the renin-angiotensin-aldosterone system in nondiabetic CKD: an open-label crossover randomized controlled trial	Tylicki, , L, Rutkowski, P, Renke, M, Larczyński, W, Aleksandrowicz, E, Lysiak-Szydlowska, W; Rutkowski, B	2008
Prevention of myocardial damage during coronary intervention	Pasceri, , V, Patti, G; Di Sciascio, G	2006
Antioxidant-rich dietary intervention for improving asthma control in pregnancies complicated by asthma: study protocol for a randomized controlled trial	Grieger, , JA, Wood, LG; Clifton, VL	2014
Pioneer study: pPARgamma activation results in overall improvement of clinical and metabolic markers associated with insulin resistance independent of long-term glucose control	Pfützner, , A, Hohberg, C, Lübben, G, Pahler, S, Pfützner, AH, Kann, P; Forst, T	2005
Angiotensinogen gene M235T polymorphism and reduction in wall thickness in response to antihypertensive treatment	Bozec, , E, Fassot, C, Tropeano, AI, Boutouyrie, P, Jeunemaitre, X, Lacolley, P, Dabire, H; Laurent, S	2003
Control Crohn Safe Trial	NCT03917303	2019
Attention deficit and hyperactivity disorder scores are elevated and respond to N-acetylcysteine treatment in patients with systemic lupus erythematosus	Garcia, , RJ, Francis, L, Dawood, M, Lai, ZW, Faraone, SV; Perl, A	2013

Development and evaluating multimarker models for guiding treatment decisions	Tajik, , P, Zafarmand, MH, Zwinderman, AH, Mol, BW; Bossuyt, PM	2018
Final analysis of the prospective WSG-AGO EC-Doc versus FEC phase III trial in intermediate-risk (pN1) early breast cancer: efficacy and predictive value of Ki67 expression	Nitz, , U, Gluz, O, Huober, J, Kreipe, HH, Kates, RE, Hartmann, A, Erber, R, Moustafa, Z, Scholz, M, Lisboa, B, Mohrmann, S, Möbus, V, Augustin, D, Hoffmann, G, Weiss, E, Böhmer, S, Kreienberg, R, Du Bois, A, Sattler, D, Thomssen, C, Kiechle, M, Jänicke, F, Wallwiener, D, Harbeck, N; Kuhn, W	2014
Changes in Inflammatory and Bone Turnover Markers After Periodontal Disease Treatment in Patients With Diabetes	Izuora, , KE, Ezeanolue, EE, Neubauer, MF, Gewelber, CL, Allenback, GL, Shan, G; Umpierrez, GE	2016
The clinical benefit of adjunctive dexamethasone in tuberculous meningitis is not associated with measurable attenuation of peripheral or local immune responses	Simmons, , CP, Thwaites, GE, Quyen, NT, Chau, TT, Mai, PP, Dung, NT, Stepniewska, K, White, NJ, Hien, TT; Farrar, J	2005
QTc duration is independently associated with tissue palmitoleic acid levels but is not affected by omega-3 fatty acid treatment in patients with non-alcoholic fatty liver disease and features of the metabolic syndrome	Palmer, , CE, Scorletti, E, Clough, GF, McCormick, KG, Bhatia, L, Targher, G, Curzen, NP, Calder, PC; Byrne, CD	2014
Empagliflozin reduces markers of arterial stiffness, vascular resistance and cardiac workload in empa-reg outcome	Chilton, , RJ, Gullestad, L, Fitchett, D, Inzucchi, SE, Mattheus, M, Woerle, HJ; Johansen, OE	2016
Long-term creatine supplementation is safe in aged patients with Parkinson disease	Bender, , A, Samtleben, W, Elstner, M; Klopstock, T	2008
Iron supplementation in premature infants using the zinc protoporphyrin to heme ratio: short- and long-term outcomes	Miller, SM	2013
PHAGE Study: bacteriophages as Novel Prebiotics	NCT03269617	2017
Short-Term, High-Dose Fish Oil Supplementation Increases the Production of Omega-3 Fatty Acid-Derived Mediators in Patients With Peripheral Artery Disease (the OMEGA-PAD I Trial)	Grenon, , SM, Owens, CD, Nosova, EV, Hughes-Fulford, M, Alley, HF, Chong, K, Perez, S, Yen, PK, Boscardin, J, Hellmann, J, Spite, M; Conte, MS	2015
T2 relaxometry helps prognosticate seizure outcome in patients with solitary cerebral cysticercosis	de Souza, , A, Nalini, A, Saini, J; Thennarasu, K	2017
Low-dose theophylline in childhood asthma: a placebo-controlled, double-blind study	Suessmuth, , S, Freihorst, J; Gappa, M	2003
Clinical features and outcomes of focal segmental glomerulosclerosis pathologic variants in Korean adult patients	Kwon, , YE, Han, SH, Kie, JH, An, SY, Kim, YL, Park, KS, Nam, KH, Leem, AY, Oh, HJ, Park, JT, Chang, TI, Kang, EW, Kang, SW, Choi, KH, Lim, BJ, Jeong, HJ; Yoo, TH	2014
Does vitamin D administered to children with asthma treated with inhaled glucocorticoids affect short-term growth or bone turnover?	Schou, , AJ, Heuck, C; Wolthers, OD	2003
Effects of late acute rejection on graft survival and outcome in pediatric renaltransplant recipients	Baskin, , E, Kantar, A, Gulleroglu, K, Ozmen, E, Ozdemir, H, Kirnap, M, Moray, G; Haberal, M	2015
Effects of nonsurgical periodontal therapy on C-reactive protein and serum lipids in Jordanian adults with advanced periodontitis	Kamil, , W, Al Habashneh, R, Khader, Y, Al Bayati, L; Taani, D	2011
Drospirenone/ethinyl estradiol versus rosiglitazone treatment in overweight adolescents with polycystic ovary syndrome: comparison of metabolic, hormonal, and cardiovascular risk factors	Tfayli, , H, Ulnach, JW, Lee, S, Sutton-Tyrrell, K; Arslanian, S	2011
Pharmacokinetics of miltefosine in children and adults with Cutaneous leishmaniasis	Del Mar Castro, , M, Gomez, MA, Kip, AE, Cossio, A, Ortiz, E, Navas, A, Dorlo, TPC; Saravia, NG	2017
Suvorexant in the Management Comorbid Sleep Disorder and Alcohol Dependence	NCT03897062	2019
Multicenter clinical study about the action of Fuzheng Huayu Capsule against liver fibrosis with chronic hepatitis B	Liu, , P, Hu, YY, Liu, C, Xu, LM, Liu, CH, Sun, KW, Hu, DC, Yin, YK, Zhou, XQ, Wan, MB, Cai, X, Zhang, ZQ, Ye, J, Tang, BZ; He, J	2003
Changes in the immune response after treatment with benznidazole versus no treatment in patients with chronic indeterminate Chagas disease	Vallejo, , A, Monge-Maillo, B, Gutiérrez, C, Norman, FF, López-Vélez, R; Pérez-Molina, JA	2016
Adding HRV biofeedback to psychotherapy increases heart rate variability and improves the treatment of major depressive disorder	Caldwell, , YT; Steffen, PR	2018
Intrathecal Rituximab in Progressive Multiple Sclerosis	NCT02545959	2015
Phosphatidylethanol (PEth) in blood as marker of alcohol relapse (AR) in Subjects with Severe Alcoholic Hepatitis (sAH) in the ELAD Phase 3 Clinical Trial	Mac Nicholas, , R, Subramanian, RM, Thompson, JA, Al-khafaji, A, Reich, DJ, Hassanein, TI, Teperman, LW, Li, Z; Ashley, R	2016
Angiotensin-converting enzyme inhibition improves vascular function in rheumatoid arthritis	Flammer, , AJ, Sudano, I, Hermann, F, Gay, S, Forster, A, Neidhart, M, Künzler, P, Enseleit, F, Périat, D, Hermann, M, Nussberger, J, Luscher, TF, Corti, R, Noll, G; Ruschitzka, F	2008
Serum interferon-gamma is a psoriasis severity and prognostic marker	Abdallah, , MA, Abdel-Hamid, MF, Kotb, AM; Mabrouk, EA	2009
Altered monocyte CD44 expression in peripheral arterial disease is corrected by fish oil supplementation	Madden, , J, Shearman, CP, Dunn, RL, Dastur, ND, Tan, RM, Nash, GB, Rainger, GE, Brunner, A, Calder, PC; Grimble, RF	2009
IgG Fc N-glycosylation in Guillain-Barré syndrome treated with immunoglobulins	Fokkink, , WJ, Selman, MH, Dortland, JR, Durmuş, B, Kuitwaard, K, Huizinga, R, van Rijs, W, Tio-Gillen, AP, van Doorn, PA, Deelder, AM, Wuhrer, M; Jacobs, BC	2014
Impact of Specialist Led Integrated Care in COPD	NCT03482700	2018
Antipruritic Effect of Acupuncture in Patients With Atopic Dermatitis	NCT02844452	2016
Acute rhinosinusitis (ARS). Diagnosis and treatment of adults in general practice	Hansen, JG	2014
Serum selenium levels in patients with head and neck cancer	Yadav, , SP, Gera, A, Singh, I; Chanda, R	2002
Study of the Effects of Dopaminergic Medications on Dopamine Transporter Imaging in Parkinson's Disease	NCT00096720	2004

Rationale and design of a multicenter placebo-controlled double-blind randomized trial to evaluate the effect of empagliflozin on endothelial function: the EMBLEM trial	Tanaka, , A, Shimabukuro, M, Okada, Y, Taguchi, I, Yamaoka-Tojo, M, Tomiyama, H, Teragawa, H, Sugiyama, S, Yoshida, H, Sato, Y, Kawaguchi, A, Ikehara, Y, Machii, N, Maruhashi, T, Shima, KR, Takamura, T, Matsuzawa, Y, Kimura, K, Sakuma, M, Oyama, JI, Inoue, T, Higashi, Y, Ueda, S; Node, K	2017
Rilotumumab in combination with epirubicin, cisplatin, and capecitabine as first-line treatment for gastric or oesophagogastric junction adenocarcinoma: an open-label, dose de-escalation phase 1b study and a double-blind, randomised phase 2 study	Iveson, , T, Donehower, RC, Davidenko, I, Tjulandin, S, Deptala, A, Harrison, M, Nirni, S, Lakshmaiah, K, Thomas, A, Jiang, Y, Zhu, M, Tang, R, Anderson, A, Dubey, S, Oliner, KS; Loh, E	2014
Cytotoxic markers associate with protection against malaria in human volunteers immunized with Plasmodium falciparum sporozoites	Bijker, , EM, Teirlinck, AC, Schats, R, van Gemert, GJ, van de Vegte-Bolmer, M, van Lieshout, L, IntHout, J, Hermsen, CC, Scholzen, A, Visser, LG; Sauerwein, RW	2014
A comparison of fish oil, flaxseed oil and hempseed oil supplementation on selected parameters of cardiovascular health in healthy volunteers	Kaul, , N, Kreml, R, Austria, JA, Richard, MN, Edel, AL, Dibrov, E, Hirono, S, Zettler, ME; Pierce, GN	2008
CBS Treatment in Hand Osteoarthritis and Psoriatic Arthritis	NCT03693833	2018
Randomized, double-blind pilot study of transendocardial injection of autologous aldehyde dehydrogenase-bright stem cells in patients with ischemic heart failure	Perin, , EC, Silva, GV, Zheng, Y, Gahremanpour, A, Canales, J, Patel, D, Fernandes, MR, Keller, LH, Quan, X, Coulter, SA, Moore, WH, Herlihy, JP; Willerson, JT	2012
Initial results of an ongoing study for CYP2C19 genetic profile and association with clopidogrel therapy outcome in different ethnic groups from R. Macedonia	Nestorovska, , AK, Cvetkovska, A, Dimovski, AJ; Suturkova, L	2012
The establishment of the objective diagnostic markers and personalized medical intervention in patients with major depressive disorder: rationale and protocol	Lv, , X, Si, T, Wang, G, Wang, H, Liu, Q, Hu, C, Wang, J, Su, Y, Huang, Y, Jiang, H; Yu, X	2016
Epstein-Barr Virus Suppression in Chronic Obstructive Pulmonary Disease (EViSCO): a Phase 2 Randomised Control Trial	NCT03699904	2018
Platelet-Derived Growth Factor as an Antidepressant Treatment Selection Biomarker: higher Levels Selectively Predict Better Outcomes with Bupropion-SSRI Combination	Jha, , MK, Minhajuddin, A, Gadad, BS; Trivedi, MH	2017
Adjuvant randomized study with Docetaxel and Carboplatin with or without radiotherapy in patients with operable gastric cancer	ACTRN12609000757213	2009
Study of the possibility to increase remnant insulin production in patients with type 1 diabetes using the glucagon-like peptide 1 mimicking drug liraglutide	EUCTR2014-005174-11-SE	2015
Treatment of nerve pain in patients with diabetes with a capsaicin (chilli pepper) patch (skin plaster)	EUCTR2017-004746-17-GB	2018
A MULTI-CENTER, PHASE 2B, RANDOMIZED, DOUBLE-BLIND, PLACEBO-CONTROLLED, PARALLEL-GROUP, DOSE-RANGING STUDY FOLLOWED BY AN EXTENSION PERIOD EVALUATING THE EFFICACY AND SAFETY OF DAPIROLIZUMAB PEGOL IN SUBJECTS WITH MODERATE TO SEVERE ACTIVE SYSTEMIC LUPUS ERYTHEMATOSUS	PER-044-16	2017
STudying Acute exaceRbations and Responses: COPD STARR Study. v1.0	ISRCTN90445176	2015
Nintedanib (BIBF 1120) in Mesothelioma	EUCTR2012-005201-48-SE	2015
Study with the herbal preparation STW5-II in patients with mild to moderate ulcerative colitis (chronic inflammatory bowel disease)	EUCTR2013-000891-13-DE	2014
A clinical trial to test if the drug SMT C1100 is safe and well absorbed compared to placebo when given to children with Duchenne Muscular Dystrophy (DMD), who follow a balanced diet	EUCTR2014-003100-78-GB	2014
A 52-Week Efficacy and Safety Study of Reslizumab Subcutaneous Dosing in Patients with Uncontrolled Asthma and Elevated Blood Eosinophils	EUCTR2015-000865-29-PL	2015
Treatment of a blood-clotting disorder as a consequence of a major injury with fresh plasma or blood-clotting factor concentrats (RETIC)	EUCTR2011-004139-29-AT	2011
The effect of a novel drug (CCX140-B) on albuminuria in patients with type 2 diabetes mellitus	EUCTR2011-003049-16-NL	2011
Effect of Probiotic and Smectite Gel on NAFLD	NCT03614039	2018
Diet as a Potential Treatment for Autosomal Dominant Polycystic Kidney Disease	NCT02225860	2014
CORAIL Trial	EUCTR2014-005251-39-CZ	2015
Effects of antihypertensive drugs on soluble (pro)renin receptor in hypertensive patients: open-label parallel-group controlled study	JPRN-UMIN000006342	2011
Treatment of maternal depression during pregnancy-efects for the mother and the child	EUCTR2013-004444-31-SE	2015
A Double-Blind, Randomized Controlled 12-Week Follow-Up Trial to Evaluate the Efficacy and Safety of Polycan in Combination with Glucosamine for the Treatment of Knee Osteoarthritis	Truong, , TTT, Lim, JM, Cho, H-R, Kim, YS, Dao, DG, Tran, QH; Choi, J-S	2019
A multicenter randomised study comparing the efficacy of pegylated interferon-alfa-2a plus placebo vs. pegylated interfeorn-alfa-2a plus tenofovir for the treatment of chronic delta hepatitis- The Hep-Net International Delta hepatits Interventional Trial II (HIDIT-II) - HIDIT-II	EUCTR2008-005560-13-GR	2009
IL-6 inhibition in patients with depression and low-grade inflammation: the Insight study	ISRCTN16942542	2018

A Phase IV, open-label, prospective, randomised clinical trial to evaluate the usefulness of measuring nitric oxide in exhaled air in the therapeutic management of adult patients with mild asthma	EUCTR2012-000372-42-ES	2012
(M)ultifactorial (I)ntervention in Patients with (P)eripheral (A)rterial (D)isease - A Randomised Controlled Trial	ACTRN12618000250235	2018
The effect of liraglutide in patients with prediabetes and kidney failure	EUCTR2014-001778-32-DK	2014
Clinical trial to compare the effect of liraglutide and glimepiride, both in combination with metformin in patients with type 2 diabetes	EUCTR2012-000311-87-ES	2012
RAltegravir Switch STudy: effects on Endothelial Recovery	EUCTR2011-003298-26-NL	2011
Research study to evaluate the experimental drug ferumoxytol, to see how well it works for children with chronic kidney disease (CKD) and iron deficiency anemia (IDA) or who are at risk of developing IDA, and to see how safe it is compared to another marketed iron product – iron sucrose	EUCTR2017-004026-15-HU	2018
A prospective study of therapeutic outcome in patients with locally advanced Head and Neck Cancers treated with chemo-radiation in combination with Nimorazole	CTRI/2017/09/009921	2017
Tyrosine kinase Inhibitors in DysplAsia of Lung epithelium Study 1 - TIDAL 1 Version 1.0	EUCTR2010-023355-29-GB	2011
Refractory Rheumatoid Arthritis	DRKS00005807	2014
Can-Art Effect and safety of using Canabis derivatives for the treatment of pain in patients with inflammatory Arthritis, such as reumatoid arthritis and ankylosing spondylitis, the latter being a type of arthritis that causes a long term inflammation of the joints of the spine. A randomized, double blinded, placebo controlled trial, i.e. in this drug trial, a control group is given a placebo while another group is given the Cannabis derivative being studied	EUCTR2017-004226-15-DK	2018
Changes in cerebral blood flow velocity after treatment with sumatriptan or placebo and implications for the pathophysiology of migraine	Limmroth, , V, May, A, Auerbach, P, Wosnitza, G, Eppe, T; Diener, HC	1996
An open Phase II study to assess the clinical activity and safety of recMAGE-A3 + AS15 cancer immunotherapeutic in patients with metastatic cutaneous melanoma, and to explore its immunogenic properties, including their relation to tumor infiltration, genomics and proteomics - MAGE3-AS15-MEL-004 (MET)	EUCTR2008-001301-42-FR	2008
Effects of Neupro on cardiovascular observations in patients with restless legs syndrome	EUCTR2011-000053-23-DE	2011
PRIMUS001: a study looking at two different treatments for pancreatic cancer that has spread to other parts of the body	ISRCTN75002153	2017
Can the drug acipimox relieve muscle symptoms in patients with mitochondrial myopathy?	ISRCTN12895613	2019
New treatment regimens for treatment of Post Kala Azar Dermal Leishmaniasis patients in India and Bangladesh region	CTRI/2017/04/008421	2017
A study in newborn children in need for intensive treatment resulting from insufficient oxygen supply to the brain	EUCTR2011-002502-74-Outside-EU/EEA	2014
Renoprotective effects of ursodeoxycholic acid	ISRCTN14333327	2017
Treatment with Iloprost and Integrilin compared to standard care in severe pneumonia patients with severe sepsis	EUCTR2011-002254-31-FI	2011
Treatment of Graves´ ophthalmopathy with diclofenak or simvastatin	EUCTR2015-004334-99-SE	2015
functional MRI study to evaluate improvement of hand performance after combined motor exercise and aminopiridine assumption	EUCTR2012-000287-17-IT	2012
Mycophenolate Treatment for Longstanding Complex Regional Pain Syndrome (MYPS I)	EUCTR2015-000263-14-GB	2015
An exploratory study on the related factors of pharmacokinetics and therapeutic markers of clozapine in schizophrenia patients	ChiCTR1800015631	2018
Efficacy of Methotrexate on Chronic Chondrocalcinosis	EUCTR2007-003479-37-IE	2007
balANZ Study: a multicentre, randomised, controlled trial to determine whether peritoneal dialysis treatment with a low GDP, neutral pH peritoneal dialysis (PD) solution (balance) compared to standard PD solution is associated with superior preservation of residual renal function	ACTRN12606000044527	2006
Comparing the Lutein formulations - Product A and Product B (Lutein 25 mg/capsule) supplementation in healthy, adult humans, after receiving high-fat, high-calorie breakfast	CTRI/2018/01/011167	2018
The effect of long term antibiotic therapy on exacerbation (flare-up) frequency in patients with Stable Chronic Obstructive Pulmonary Disease (COPD)	EUCTR2012-002199-15-GB	2013
Assessment of Therapeutic Response to Benznidazole in patients with Chronic Chagas Disease	EUCTR2011-002022-41-ES	2011
Multicenter, randomized, open-label study to compare immunoglobulin to immunoglobulin plus cyclosporin A combination therapy in patients with severe Kawasaki disease	JPRN-UMIN000017585	2015
The effect of bezafibrate on cholesterol levels after an oral fatload in patients with Familial Dysbetalipoproteinemia	EUCTR2014-000524-26-NL	2015

Gastrointestinal and lung cancer, effects of the fish oil intake on nutritional status, quality of life and imunnometabolic outcomes	RBR-2xcd99	2016
Neoadjuvant plus adjuvant or only adjuvant nab-Paclitaxel plus Gemcitabine for resectable pancreatic cancer - The AIO-NEONAX trial (AIO-PAK-0313) A prospective, randomized, controlled, phase II study of the AIO Pancreatic Cancer Group	EUCTR2013-005559-34-DE	2014
Effects Of Autologus Platelet- Rich Plasma on Pregnancy Rate in Intra Cytoplasmic Sperm Injection (ICSI) cycles in Repeated Implantation Failure Patients	IRCT2017061034422N1	2017
A double-blind, randomised, placebo-controlled interventional study to evaluate the effect of orally-dosed Almega PL on cardio-metabolic parameters and inflammatory markers in men and women with hypercholesterolemia	ACTRN12618000275268	2018
AML-BFM 2012: clinical trial for the treatment of acute myeloid leukemia in children and adolescents	ISRCTN78830591	2013
Iron treatment in patients with known kidney disease	EUCTR2010-020452-64-GB	2010
Rituximab in Graves' disease (RIGD)	ISRCTN20381716	2016
Metabolic Dysfunctions Associated with Pharmacological Treatment of Schizophrenia	EUCTR2018-000178-31-ES	2018
A clinical trial for the effects of testosterone replacement therapy in male patients with hypogonadism on arterial function markers	EUCTR2014-005179-10-GR	2015
Anti-inflammatory effects of roflumilast in chronic obstructive pulmonary disease (ROBERT): a 16-week, randomised, placebo-controlled trial	Rabe, , KF, Watz, H, Baraldo, S, Pedersen, F, Biondini, D, Bagul, N, Hanauer, G, Göhring, UM, Purkayastha, D, Román, J, Alagappan, VKT; Saetta, M	2018
Prediction and prevention of kidney disease in patients with diabetes	EUCTR2012-000452-34-NL	2013
Parental communication deviance: its stability and the effect of family treatment in recent-onset schizophrenia	Nugter, , MA, Dingemans, PM, Linszen, DH, Van der Does, AJ; Gersons, BP	1997
A phase IIa study to characterize the effects of CCL2 inhibition with the Spiegelmer® NOX-E36 in patients with type 2 diabetes mellitus and albuminuria	EUCTR2011-005710-11-PL	2012
Control of periodontal inflammation, systemic inflammatory responses and cognitive decline in Alzheimer?'s disease	ISRCTN34471493	2013
A European trial to evaluate the effect of Allopurinol administered very early after birth on brain injury in children with oxygen deficiency during birth	EUCTR2016-000222-19-BE	2018
Use of Omalizumab will increase the pregnancy rate, proof of concept study, where women with asthma and infertility will be treated three times with weight and IgE balanced dosis at the first day of their period bleeding	EUCTR2018-001137-41-DK	2018
Escherichia coli strain Nissle 1917 - Suspension for treatment of patients with Clostridium difficile associated diarrhoea	EUCTR2017-004531-36-DE	2018
Impact of EECP-treatment (Enhanced External Counterpulsation) on myocardial adaptive arteriogenesis in patients suffering from stable symptomatic coronary heart disease	ISRCTN08877757	2007
A study to assess the effect of rilapladib (SB 659032) on Alzheimer’s disease	EUCTR2010-020993-41-SE	2011
A Randomised Controlled Trial to assess a food supplement (baker’s yeast extract) to support immune function and prevent cold and flu symptoms in a 50 to 70 year old population	EUCTR2011-004910-41-GB	2011
The trial investigates the effect of 500 µg roflumiliast tablets once daily on acute worsening of symptoms (exacerbations) treated with standard therapy in patiens with chronic obstructive pulmonary disease (COPD)	EUCTR2011-002905-31-GB	2011
A European trial to evaluate the effect of Allopurinol administered very early after birth on brain injury in children with oxygen deficiency during birth	EUCTR2016-000222-19-DE	2017
Efficacy and safety of low-dose IL-2 (ld-IL-2) as a Treg enhancer for controlling neuro-inflammation in newly diagnosed Amyotrophic Lateral Sclerosis (ALS) patients	EUCTR2015-005347-14-GB	2016
2-Iminobiotin vas a treatment for neonates born with severe oxygen shortage at birth	EUCTR2015-003063-12-Outside-EU/EEA	2016
Automimmune Diabetes Accelerator Prevention Trial (adAPT)	EUCTR2015-000748-41-GB	2015
Double-blind, masked, randomized, three period crossover study to evaluate the changes in exhaled nitric oxide (eNO) following treatment with fluticasone propionate aerosol delivered by a metered dose inhaler in patients with chronic mild to moderate persistent asthma	EUCTR2012-003395-40-DE	2012
A study to evaluate the effectiveness and safety of Apremilast in subjects with active psoriatic arthritis	EUCTR2013-001590-25-EE	2013
Targeted retreatment with antibiotics following exacerbations of Chronic Obstructive Pulmonary Disease	EUCTR2012-002198-72-GB	2013
Can we reduce asthma attacks in children using exhaled nitric oxide measurements?	ISRCTN67875351	2017
FOCUS4: molecular selection of therapy in colorectal cancer	EUCTR2012-005111-12-GB	2013
The effects of adjuvant Tualang honey therapy on disease activity markers in systemic lupus erythematosus patients	ACTRN12611000074998	2011

Efficacy of Roflumilast and Montelukast in the treatment of severe uncontrolled asthma	EUCTR2012-002064-27-HU	2012
A Dehydroepiandrosterone and Pharmacokinetics in Trauma study (ADaPT)	ISRCTN12961998	2018
A 2 part clinical study that will evaluate the safety and effectiveness of LJN452A to treat patients with fatty liver disease for a period of 12 weeks. The first part will be used for LJN452 dose selection for the second part of the study	EUCTR2015-005215-33-SK	2016
A study to investigate the safety and efficacy of a six month oral treatment with a twice daily dose of BAY 1142524 in comparison to placebo in patients with reduced left-ventricular ejection fraction after acute myocardial infarction	EUCTR2016-002167-33-ES	2016
Individualizing duration of antibiotic therapy in hospitalized patients with pneumonia	EUCTR2011-003176-36-IT	2011
A Prospective, Randomized, Double-Blind, Double-Dummy, Multicenter Study to Assess the Safety and Efficacy of Doripenem Compared With Imipenem-Cilastatin in the Treatment of Subjects with Ventilator-Associated Pneumonia	EUCTR2007-004646-33-DE	2008
Efficacy of metformin in gestational diabetes	EUCTR2015-000361-31-ES	2015
Proteinuria in Glomerulonephritis: myfortic® (GloMY)	ISRCTN11937028	2010
Treatment og chronic non bacteriel osteomyelitis with pamidronate	EUCTR2015-002038-36-DK	2015
Can treatment with drugs developed to treat thin and weakened osteoporotic bones make knee implants stay fixed in the bones and last longer?	EUCTR2012-004046-15-DK	2013
The Impact of Gender and Protein Intake on the Success of Weight Maintenance and Associated Cardiovascular Risk Benefits, Independent of the Mode of Food Provision: the DiOGenes Randomized Trial	Navas-Carretero, , S, Holst, C, Saris, WH, van Baak, MA, Jebb, SA, Kafatos, A, Papadaki, A, Pfeiffer, AF, Handjieva-Darlenska, T, Hlavaty, P, Stender, S, Larsen, TM, Astrup, A; Martinez, JA	2016
Plasma 3-nitrotyrosine and outcome in neonates with severe bronchopulmonary dysplasia after inhaled nitric oxide	Lorch, , SA, Banks, BA, Christie, J, Merrill, JD, Althaus, J, Schmidt, K, Ballard, PL, Ischiropoulos, H; Ballard, RA	2003
Effects of a pulmonary rehabilitation program on physical capacity, peripheral muscle function and inflammatory markers in asthmatic children and adolescents: study protocol for a randomized controlled trial	Reimberg, , MM, Castro, RA, Selman, JP, Meneses, AS, Politti, F, Mallozi, MC, Wandalsen, GF, Solé, D, De Angelis, K, Dal Corso, S; Lanza, FC	2015
Dopamine agonist monotherapy in Parkinson's disease	Clarke, , CE; Guttman, M	2002
Evaluating osteopathic manipulative therapy (OMT) as a treatment modality for the reduction of chronic stress in medical students	Tyner, , TJ, Cavayero II, C, Philips II, A, Johnson III, B; Quinn, T	2015
Minimal residual disease analysis in patients with follicular lymphoma treated with obinutuzumab plus bendamustine versus bendamustine alone in GADOLIN, a phaseII study of relapsed/refractory indolent non-Hodgkin lymphoma	Pott, , C, Belada, D, Danesi, N, Fingerle-Rowson, G, Gribben, J, Harbron, C, Hoster, E, Kahl, B, Mundt, K, Sebban, C, Sehn, LH; Cheson, BD	2016
Longitudinal neurocognitive effects of combined electroconvulsive therapy and pharmacotherapy in geriatric major depressive disorder	Lisanby, , S, McClintock, S, Alexopoulos, G, Bailine, S, Bernhardt, E, Briggs, M, Cullum, CM, Deng, Z-D, Geduldig, E, Greenberg, R, Husain, M, Kaliora, S, Knapp, R, Liebman, L, Latoussakis, V, McCall, WV, Mueller, M, Petrides, G, Prudic, J, Rosenquist, P, Rudorfer, M, Sampson, S, Teklehaimanot, A, Tobias, K, Weiner, R, Young, R; Kellner, C	2017
Pembrolizumab for patients with advanced prostate adenocarcinoma: preliminary results from the KEYNOTE-028 study	Hansen, , A, Massard, C, Ott, PA, Haas, N, Lopez, J, Ejadi, S, Wallmark, J, Keam, B, Delord, J-P, Aggarwal, R, Gould, M, Qiu, P, Saraf, S, Keefe, S; Piha-Paul, SA	2016
Changes in echocardiographic parameters in iron deficiency patients with heart failure and chronic kidney disease treated with intravenous iron	Toblli, , JE, Di Gennaro, F; Rivas, C	2015
The effect of N-acetylcysteine on proteinuria and markers of tubular injury in non-diabetic patients with chronic kidney disease. A placebo-controlled, randomized, open, cross-over study	Renke, , M, Tylicki, L, Rutkowski, P, Larczyński, W, Aleksandrowicz, E, Lysiak-Szydłowska, W; Rutkowski, B	2008
The protective effects of angiotensin II blockade with olmesartan medoxomil on resistance vessel remodeling (The VIOS study): rationale and baseline characteristics	Smith, , RD, Yokoyama, H, Averill, DB, Cooke, L, Brosnihan, KB, Schiffrin, EL; Ferrario, CM	2006
Dual blockade of the renin-angiotensin-aldosterone system with high-dose angiotensin-converting enzyme inhibitor for nephroprotection: an open, controlled, randomized study	Tylicki, , L, Renke, M, Rutkowski, P, Larczyński, W, Aleksandrowicz, E, Lysiak-Szydlowska, W; Rutkowski, B	2008
Randomized placebo-controlled trial assessing the effect of 24-week fenofibrate therapy on circulating markers of abdominal aortic aneurysm: outcomes from the FAME-2 trial	Pinchbeck, , JL, Moxon, JV, Rowbotham, SE, Bourke, M, Lazzaroni, S, Morton, SK, Matthews, EO, Hendy, K, Jones, RE, Bourke, B, Jaeggi, R, Favot, D, Quigley, F, Jenkins, JS, Reid, CM, Velu, R; Golledge, J	2018
Combined continuous ethinyl estradiol/norethindrone acetate does not improve forearm blood flow in postmenopausal women at risk for cardiovascular events: a pilot study	Duvernoy, , CS, Rose, PA, Kim, HM, Kehrer, C; Brook, RD	2007
A post-hoc analysis of a novel endpoint for asthma exacerbations in a SQ HDM SLIT-tablet phase III allergic asthma trial	Eriksson, , G, Nolte, H, Ljorring, C, Riis, B; Virchow, JC	2018
Atorvastatin and simvastatin in patients on hemodialysis: effects on lipoproteins, C-reactive protein and in vivo oxidized LDL	van den Akker, , JM, Bredie, SJ, Diepenveen, SH, van Tits, LJ, Stalenhoef, AF; van Leusen, R	2003
Addition of fexofenadine to inhaled corticosteroid therapy to reduce inflammatory biomarkers in atopic asthma	Fardon, , TC, Lee, DK, Hodge, MR; Lipworth, BJ	2005
Predictors of delayed culture conversion among Ugandan patients	Atwine, , D, Orikiriza, P, Taremwa, I, Ayebare, A, Logoose, S, Mwanga-Amumpaire, J, Jindani, A; Bonnet, M	2017

Changes in inflammatory mediators in gingival crevicular fluid following periodontal disease treatment in pregnant women	Penova-Veselinovic, , B, Keelan, J, Newnham, JP; Pennell, CE	2014
Description and status of the azithromycin and coronary events study (ACES)	Jackson, LA	2000
Oral Health Intervention Program for Children With Congenital Heart Defects	NCT03311438	2017
Psychological interventions to improve adherence to oral hygiene instructions in adults with periodontal diseases	Renz, , A, Ide, M, Newton, T, Robinson, PG; Smith, D	2007
Evaluating the validity of probing attachment loss as a surrogate for tooth mortality in a clinical trial on the elderly	Hujoel, , PP, Leroux, BG, DeRouen, TA, Powell, LV; Kiyak, HA	1997
Effects of Pycnogenol on endothelial function in patients with stable coronary artery disease: a double-blind, randomized, placebo-controlled, cross-over study	Enseleit, , F, Sudano, I, Périat, D, Winnik, S, Wolfrum, M, Flammer, AJ, Fröhlich, GM, Kaiser, P, Hirt, A, Haile, SR, Krasniqi, N, Matter, CM, Uhlenhut, K, Högger, P, Neidhart, M, Lüscher, TF, Ruschitzka, F; Noll, G	2012
Chronic Beta-blockade and Cardiopulmonary Exercise in COPD	NCT02380053	2015
Inhaled heparin in cystic fibrosis	Serisier, , DJ, Shute, JK, Hockey, PM, Higgins, B, Conway, J; Carroll, MP	2006
Levetiracetam vs. Older antiepileptic drugs: a randomized prospective bone health study	Hakami, , T, O'Brien, T, Petty, S, Sakellarides, M, Christie, J, Todaro, M; Wark, JD	2011
Short-term rosuvastatin treatment for the prevention of contrast-induced acute kidney injury in patients receiving moderate or high volumes of contrast media: a sub-analysis of the TRACK-D study	Zhang, , J, Li, Y, Tao, GZ, Chen, YD, Hu, TH, Cao, XB, Jing, QM, Wang, XZ, Ma, YY, Wang, G, Liu, HW, Wang, B, Xu, K, Li, J, Deng, J; Han, YL	2015
Changes of biochemical indexes in patients with acute cerebral infarction after treatment with simvastatin	Yang, C	2006
A 7-week, randomized, double-blind trial of olanzapine/fluoxetine combination versus lamotrigine in the treatment of bipolar I depression	Brown, , EB, McElroy, SL, Keck, PE, Deldar, A, Adams, DH, Tohen, M; Williamson, DJ	2006
Efficacy and safety of avandamet or uptitrated metformin treatment in patients with type 2 diabetes inadequately controlled with metformin alone: a multicenter, randomized, controlled trial	Cai, , XL, Chen, YL, Zhao, JJ, Shan, ZY, Qiu, MC, Li, CJ, Gu, W, Tian, HM, Yang, HZ, Xue, YM, Yang, JK, Hong, TP; Ji, LN	2015
Efficacy and safety of Canakinumab in patients with periodic fever syndromes (colchicine-resistant fmf, hids/mkd and traps): results from a phase 3, pivotal, umbrella trial	De Benedetti, , F, Anton, J, Gattorno, M, Lachmann, H, Kone-Paut, I, Ozen, S, Frenkel, J, Simon, A, Zeft, A, Ben-Chetrit, E, Hoffman, HM, Joubert, Y, Lheritier, K, Speziale, A; Guido, J	2017
Using a randomized controlled trial to test whether modifications to contingency management improve outcomes for heavy drinkers with serious mental illness	Oluwoye, , O, Skalisky, J, Burduli, E, Chaytor, NS, McPherson, S, Murphy, SM, Herron, J, Hirchak, K, Burley, M, Ries, RK, Roll, JM; McDonell, MG	2018
Treatment of generalized pustular psoriasis during pregnancy: a real challenge	Zaouak, , A, Sassi, W, Hammami, H; Fenniche, S	2016
Comparison of changes in early inflammatory markers between sirolimus- and paclitaxel-eluting stent implantation	Li, , JJ, Yan, HB, Xiang, XP, Qin, XW; Zhang, CY	2009
Supplementation with a blend of krill and salmon oil is associated with increased metabolic risk in overweight men	Albert, , BB, Derraik, JG, Brennan, CM, Biggs, JB, Garg, ML, Cameron-Smith, D, Hofman, PL; Cutfield, WS	2015
A comparison of the effect of glitazones on serum sialic acid in patients with type 2 diabetes	ur Rahman, , I, Idrees, M, Salman, M, Khan, RU, Khan, MI, Amin, F; Jan, NU	2012
Safety and efficacy of ferric citrate in patients with nondialysis-dependent chronic kidney disease and iron deficiency anemia: post hoc analysis in patients with orwithout heart failure	McCullough, , PA, Uhlig, K, Neylan, JF; Fishbane, S	2017
Clinical effects of "pioglitazone", an insulin sensitizing drug, on psoriasis vulgaris and its co-morbidities, a double blinded randomized controlled trialx1	Hafez, , VG, Bosseila, M, Abdel Halim, MRE, Shaker, OG, Kamal, M; Kareem, HS	2015
A retrospective review identifying resistant microbial strains, antimicrobial sensitivities and risk stratification of first line antibiotic use in adult cancer patients with neutropenic sepsis	Danga, , A, Hui, YMT, Lazarus, K; Sugai, T	2017
A Randomized-Controlled Trial of Inhaled Hypertonic Saline (7%) to Evaluate the Lung Clearance Index	NCT02276898	2014
Effects of azithromycin on intracellular cytokine responses and mucocutaneous manifestations in Behçet's disease	Mumcu, , G, Inanç, N, Özdemir, FT, Tulunay, A, Ekşioğlu-Demiralp, E, Ergun, T; Direskeneli, H	2013
Efficacy of combined orthodontic-periodontic treatment for patients with periodontitis and its effect on inflammatory cytokines: a comparative study	Zhang, , J, Zhang, AM, Zhang, ZM, Jia, JL, Sui, XX, Yu, LR; Liu, HT	2017
Primary prevention: phytoprevention and chemoprevention of colorectal cancer	Turini, , ME; DuBois, RN	2002
Increased CSF APPs-α levels in patients with Alzheimer disease treated with acitretin	Endres, , K, Fahrenholz, F, Lotz, J, Hiemke, C, Teipel, S, Lieb, K, Tüscher, O; Fellgiebel, A	2014
The endogenous system is not implicated in the pathogenesis of functional constipation; results of a randomised, double blind, study investigating naloxone hydrochloride sustained release capsules (NalcolTM) as a treatment for functional constipation	Bignell, , M, Hart, A; Rhodes, M	2014
Early effects of metformin in women with polycystic ovary syndrome: a prospective randomized, double-blind, placebo-controlled trial	Eisenhardt, , S, Schwarzmann, N, Henschel, V, Germeyer, A, von Wolff, M, Hamann, A; Strowitzki, T	2006
The effect of raloxifene after discontinuation of long-term alendronate treatment of postmenopausal osteoporosis	Michalská, , D, Stepan, JJ, Basson, BR; Pavo, I	2006
The effect of a low molecular mass thrombin inhibitor, inogatran, and heparin on thrombin generation and fibrin turnover in patients with unstable coronary artery disease	Linder, , R, Oldgren, J, Egberg, N, Grip, L, Larson, G, Siegbahn, A; Wallentin, L	1999
A diabetes outcome progression trial (ADOPT): an international multicenter study of the comparative efficacy of rosiglitazone, glyburide, and metformin in recently diagnosed type 2 diabetes	Viberti, , G, Kahn, SE, Greene, DA, Herman, WH, Zinman, B, Holman, RR, Haffner, SM, Levy, D, Lachin, JM, Berry, RA, Heise, MA, Jones, NP; Freed, MI	2002

Clopidogrel does not suppress blood markers of coagulation activation in aspirin-treated patients with non-ST-elevation acute coronary syndromes	Eikelboom, , JW, Weitz, JI, Budaj, A, Zhao, F, Copland, I, Maciejewski, P, Johnston, M; Yusuf, S	2002
Vitamin D and immunomodulation in early rheumatoid arthritis: a randomized double-blind placebo-controlled study	Buondonno, , I, Rovera, G, Sassi, F, Rigoni, MM, Lomater, C, Parisi, S, Pellerito, R, Isaia, GC; D'Amelio, P	2017
Mode of death in heart failure: findings from the ATLAS trial	Poole-Wilson, , PA, Uretsky, BF, Thygesen, K, Cleland, JG, Massie, BM; Rydén, L	2003
Supplementation with dairy calcium and/or flaxseed fibers in conjunction with orlistat augments fecal fat excretion without altering ratings of gastrointestinal comfort	Kristensen, , M, Juul, SR, Sorensen, KV, Lorenzen, JK; Astrup, A	2017
A Phase II study to assess safety and efficacy of olesoxime (TRO19622) in 3-25 year old Spinal Muscular Atrophy (SMA) patients	Dessaud, , E, Andre, C, Scherrer, B, Berna, P, Pruss, R, Cuvier, V, Hauke, W; Bertini, E	2014
Effects of metformin or rosiglitazone on serum concentrations of homocysteine, folate, and vitamin B12 in patients with type 2 diabetes mellitus	Sahin, , M, Tutuncu, NB, Ertugrul, D, Tanaci, N; Guvener, ND	2007
The effects of bright light therapy on depression, sleep and circadian rhythm in patients with Parkinson's disease and a depressive disorder: results of a double-blind randomized controlled trial	Rutten, , S, Vriend, C, Smit, J, Berendse, H, Van Someren, E, Hoogendoorn, A, Twisk, J, Werf, YVD; Heuvel, OVD	2018
A randomised controlled trial of short term growth and collagen turnover in asthmatics treated with inhaled formoterol and budesonide	Heuck, , C, Heickendorff, L; Wolthers, OD	2000
Short-course antibiotic treatment for community-acquired alveolar pneumonia in ambulatory children: a double-blind, randomized, placebo-controlled trial	Greenberg, , D, Givon-Lavi, N, Sadaka, Y, Ben-Shimol, S, Bar-Ziv, J; Dagan, R	2014
Electrocortical changes associated with minocycline treatment in fragile X syndrome	Schneider, , A, Leigh, MJ, Adams, P, Nanakul, R, Chechi, T, Olichney, J, Hagerman, R; Hessl, D	2013
Exploratory biomarkers that predict for clinical outcomes in a Phase II trial with ipilimumab plus finite androgen deprivation therapy for metastatic non-castrate prostate cancer	Subudhi, , SK, Aparicio, A, Gao, J, Zurita-Saavedra, A, Araujo, JC, Logothetis, CJ, Brinda, KR, Allison, JP, Vence, L, Emerson, RO, Yusko, E, Vignali, M, Robins, HS, Sun, J; Sharma, P	2016
Predictive value of bevacizumab-related hypertension and proteinuria in patients with mCRC in the real practice	Moretti, , A, Nisi, C, Carandina, I, Marzola, M, Banno, E, Da Ros, L; Frassoldati, A	2015
Effect of low-dose dual blockade of renin-angiotensin system on urinary TGF-beta in type 2 diabetic patients with advanced kidney disease	Song, , JH, Cha, SH, Lee, HJ, Lee, SW, Park, GH, Lee, SW; Kim, MJ	2006
Arterial stiffening and systemic endothelial activation induced by smoking: the role of COX-1 and COX-2	Vlachopoulos, , C, Aznaouridis, K, Bratsas, A, Ioakeimidis, N, Dima, I, Xaplanteris, P, Stefanadis, C; Tousoulis, D	2015
Long-term effect of interferon alpha alone or after prednisone withdrawal in chronic hepatitis B. Interim report and review of the literature	Gonzalez-Mateos, , F, Garcia-Monzon, C, Garcia-Buey, L, Garcia-Sanchez, A, Pajares, JM; Moreno-Otero, R	1995
Randomized study of nutritional status and treatment toxicities of oral arginine, glutamine, and omega-3 fatty acids during concurrent chemoradiotherapy for head and neck cancer patients	Chitapanarux, , I, Pisprasert, V, Tharavichitkul, E, Jakrabhandu, S, Klunklin, P, Onchan, W, Supawongwattana, B, Traisathit, P, Rattanachaiwong, S; Sattasiri, WM	2016
Alternative multiple breath washout outcomes for clinical trials in cystic fibrosis	Ratjen, , F, Salazar, J, Jensen, R, Amin, R, Gustafsson, P, Stanojevic, S; Subbarao, P	2012
AST-120 (spherical carbon adsorbent) in the treatment of perianal fistulae in mild-to-moderate Crohn's disease: fHAST-1, a phase 3, multicenter, placebo-controlled study	Reinisch, , W, Travis, S, Hanauer, S, Wang, H, Shara, N; Harris, MS	2014
A randomized study of the safety, absorption and efficacy of pimecrolimus cream 1% applied twice or four times daily in patients with atopic dermatitis	Ling, , M, Gottlieb, A, Pariser, D, Caro, I, Stewart, D, Scott, G; Abrams, K	2005
Intravenous colistin sulphomethate in acute respiratory exacerbations in adult patients with cystic fibrosis	Conway, , SP, Pond, MN, Watson, A, Etherington, C, Robey, HL; Goldman, MH	1997
Soluble CD206 plasma levels in rheumatoid arthritis reflect decrease in disease activity	Heftdal, , LD, Stengaard-Pedersen, K, Ørnbjerg, LM, Hetland, ML, Hørslev-Petersen, K, Junker, P, Østergaard, M, Hvid, M, Deleuran, B, Møller, HJ; Greisen, SR	2017
Randomised phase II study of axitinib or bevacizumab combined with paclitaxel/carboplatin as first-line therapy for patients with advanced non-small-cell lung cancer	Twelves, , C, Chmielowska, E, Havel, L, Popat, S, Swieboda-Sadlej, A, Sawrycki, P, Bycott, P, Ingrosso, A, Kim, S, Williams, JA, Chen, C, Olszanski, AJ, de Besi, P; Schiller, JH	2014
Effects of angiotensin II receptor blockade in systemic sclerosis: randomized controlled trial	Abou-Raya, , A, Abou-Raya, S; Helmii, M	2013
High-dose vitamin D3 supplementation in children and young adults with HIV: a randomized, placebo-controlled trial	Stallings, , VA, Schall, JI, Hediger, ML, Zemel, BS, Tuluc, F, Dougherty, KA, Samuel, JL; Rutstein, RM	2015
Personalizing twin delivery management: a secondary analysis of a randomized clinical trial comparing planned caesarean or planned vaginal delivery	Zafarmand, , MH, Goossens, SM, Tajik, P, Bossuyt, PM, Asztalos, EV, Gardener, G, Willan, AR, Roumen, FJ, Mol, BW; Barrett, JY	2018
Simultaneous study of docetaxel based anthracycline free adjuvant treatment evaluation, as well as lifestyle intervention strategies: the SUCCESS-C-trial	Salmen, , J, Ortmann, U, Hagenbeck, C, Hauner, D, Hauner, H, Neugebauer, J, Andergassen, U, Rack, B, Schneeweiss, A, Beckmann, MW; Janni, W	2012
A randomized trial of stress management for the prevention of new brain lesions in MS	Mohr, , DC, Lovera, J, Brown, T, Cohen, B, Neylan, T, Henry, R, Siddique, J, Jin, L, Daikh, D; Pelletier, D	2012
Why capillaroscopy can predict disease severity and prognosis	Cutolo, M	2017
Studying Tailored Exercise Prescriptions in Breast Cancer Patients	NCT02802826	2016
Curative effect of alpha-lipoic acid on peripheral neuropathy in type 2 diabetes: a clinical study	Liu, , F, Zhang, Y, Yang, M, Liu, B, Shen, YD, Jia, WP; Xiang, KS	2007
Six-month results of a double-blind, placebo-controlled trial of etanercept treatment in patients with active ankylosing spondylitis	Brandt, , J, Khariouzov, A, Listing, J, Haibel, H, Sörensen, H, Grassnickel, L, Rudwaleit, M, Sieper, J; Braun, J	2003
Rationale and Design of the PROSPECTIVE Trial: probucol Trial for Secondary Prevention of Atherosclerotic Events in Patients with Prior Coronary Heart Disease	Yamashita, , S, Masuda, D, Ohama, T, Arai, H, Bujo, H, Kagimura, T, Kita, T, Matsuzaki, M, Saito, Y, Fukushima, M; Matsuzawa, Y	2016

Cardiovascular outcomes and achieved blood pressure in patients with and without diabetes at high cardiovascular risk	Bohm, , M, Schumacher, H, Teo, KK, Lonn, EM, Mahfoud, F, Mann, JFE, Mancia, G, Redon, J, Schmieder, RE, Marx, N, Sliwa, K, Weber, MA, Williams, B; Yusuf, S	2019
Aerobic exercise training does not alter vascular structure and function in chronic obstructive pulmonary disease	Gelinas, , JC, Lewis, NC, Harper, MI, Melzer, B, Agar, G, Rolf, JD; Eves, ND	2017
Prevention of reinfection by L-methionine in patients with recurrent urinary tract infection	Fünfstück, , R, Straube, E, Schildbach, O; Tietz, U	1997
Erythropoietin improves anemia exercise tolerance and renal function and reduces B-type natriuretic peptide and hospitalization in patients with heart failure and anemia	Palazzuoli, , A, Silverberg, D, Iovine, F, Capobianco, S, Giannotti, G, Calabrò, A, Campagna, SM; Nuti, R	2006
Study of Nasal Insulin to Fight Forgetfulness - Long-acting Insulin Detemir - 120 Days (SL120)	NCT01595646	2012
CFH and ARMS2 genetic polymorphisms predict response to antioxidants and zinc in patients with age-related macular degeneration	Awh, , CC, Lane, AM, Hawken, S, Zanke, B; Kim, IK	2013
Masitinib in the treatment of active rheumatoid arthritis: results of a multicentre, open-label, dose-ranging, phase 2a study	Tebib, , J, Mariette, X, Bourgeois, P, Flipo, RM, Gaudin, P, Le Loët, X, Gineste, P, Guy, L, Mansfield, CD, Moussy, A, Dubreuil, P, Hermine, O; Sibilia, J	2009
Dovitinib versus sorafenib for third-line targeted treatment of patients with metastatic renal cell carcinoma: an open-label, randomised phase 3 trial	Motzer, , RJ, Porta, C, Vogelzang, NJ, Sternberg, CN, Szczylik, C, Zolnierek, J, Kollmannsberger, C, Rha, SY, Bjarnason, GA, Melichar, B, De Giorgi, U, Grünwald, V, Davis, ID, Lee, JL, Esteban, E, Urbanowitz, G, Cai, C, Squires, M, Marker, M, Shi, MM; Escudier, B	2014
Portfolio of clinical research in adult cardiovascular disease as reflected in clinicalTrials.gov	Alexander, , KP, Kong, DF, Starr, AZ, Kramer, J, Chiswell, K, Tasneem, A; Califf, RM	2013
Clinical experiment of cytokines induced killer cells for treatment of benzene poisoning	Liu, , WW, Chen, JY, Yu, W, Yang, ZQ, Wu, LB, Zhang, C; Yang, DM	2007
A randomized controlled trial with an anti-CCL2 (anti-monocyte chemotactic protein 1) monoclonal antibody in patients with rheumatoid arthritis	Haringman, , JJ, Gerlag, DM, Smeets, TJ, Baeten, D, van den Bosch, F, Bresnihan, B, Breedveld, FC, Dinant, HJ, Legay, F, Gram, H, Loetscher, P, Schmouder, R, Woodworth, T; Tak, PP	2006
Natural antioxidants from tomato extract reduce blood pressure in patients with grade-1 hypertension: a double-blind, placebo-controlled pilot study	Engelhard, , YN, Gazer, B; Paran, E	2006
Improvement of impaired albumin binding capacity in acute-on-chronic liver failure by albumin dialysis	Klammt, , S, Mitzner, SR, Stange, J, Loock, J, Heemann, U, Emmrich, J, Reisinger, EC; Schmidt, R	2008
Serum osteopontin as a predictive marker of responsiveness to methotrexate in juvenile idiopathic arthritis	Masi, , L, Ricci, L, Zulian, F, Del Monte, F, Simonini, G, Capannini, S, De Martino, M, Brandi, ML; Falcini, F	2009
Cholecalciferol plus calcium suppresses abnormal PBMC reactivity in patients with multiple sclerosis	Kimball, , S, Vieth, R, Dosch, HM, Bar-Or, A, Cheung, R, Gagne, D, O'Connor, P, D'Souza, C, Ursell, M; Burton, JM	2011
Takotsubo-like myocardial dysfunction in ischemic stroke: a hospital-based registry and systematic literature review	Jung, , J-M, Kim, J-G, Kim, JB, Cho, K-H, Yu, S, Oh, K, Kim, Y-H, Choi, J-Y; Seo, W-K	2016
A randomized, double-blind, phase II, exploratory trial evaluating the palliative benefit of either continuing pamidronate or switching to zoledronic acid in patients with high-risk bone metastases from breast cancer	Jacobs, , C, Kuchuk, I, Bouganim, N, Smith, S, Mazzarello, S, Vandermeer, L, Dranitsaris, G, Dent, S, Gertler, S, Verma, S, Song, X, Simos, S, Cella, D; Clemons, M	2016
Treatment of light chain amyloidosis	Palladini, , G; Milani, P	2017
Combination of branched-chain amino acid and angiotensin-converting enzyme inhibitor improves liver fibrosis progression in patients with cirrhosis	Yoshiji, , H, Noguchi, R, Ikenaka, Y, Kaji, K, Aihara, Y, Douhara, A, Yamao, J, Toyohara, M, Mitoro, A, Sawai, M, Yoshida, M, Morioka, C, Fujimoto, M, Uemura, M; Fukui, H	2012
Hydrogel Spacer Prospective Multicenter Randomized Controlled Pivotal Trial: dosimetric and Clinical Effects of Perirectal Spacer Application in Men Undergoing Prostate Image Guided Intensity Modulated Radiation Therapy	Mariados, , N, Sylvester, J, Shah, D, Karsh, L, Hudes, R, Beyer, D, Kurtzman, S, Bogart, J, Hsi, RA, Kos, M, Ellis, R, Logsdon, M, Zimberg, S, Forsythe, K, Zhang, H, Soffen, E, Francke, P, Mantz, C, Rossi, P, Deweese, T, Hamstra, DA, Bosch, W, Gay, H; Michalski, J	2015
Ethylglucuronide based continency management for alcohol in seriously mentally ill outpatients	McDonell, , MG, Leickly, E, McPherson, S, Skalisky, J, Nepom, JR, Srebnik, D, Roll, JM; Ries, RK	2016
Potential spironolactone effects on collagen metabolism biomarkers in patients with uncontrolled blood pressure	Ferreira, , JP, Rossignol, P, Pizard, A, MacHu, J-L, Collier, T, Girerd, N, Huby, A-C, Gonzalez, A, Diez, J, Lopez, B, Sattar, N, Cleland, JG, Sever, PS; Zannad, F	2018
Efficacy comparison of Pueraria mirifica (PM) against conjugated equine estrogen (CEE) with/without medroxyprogesterone acetate (MPA) in the treatment of climacteric symptoms in perimenopausal women: phase III study	Chandeying, , V; Sangthawan, M	2007
A review of teriparatide and its clinical efficacy in the treatment of osteoporosis	Dobnig, H	2004
Treating to target patients with primary hyperlipidaemia: comparison of the effects of ATOrvastatin and ROSuvastatin (the ATOROS study)	Milionis, , HJ, Rizos, E, Kostapanos, M, Filippatos, TD, Gazi, IF, Ganotakis, ES, Goudevenos, J, Mikhailidis, DP; Elisaf, MS	2006
Is the therapeutic efficacy of coenzyme Q10 replicated in a geographical subgroup of the Q-SYMBIO study?	Mortensen, , SA, Dolliner, P, Filipiak, KJ, Alehagen, U, Pella, D, Steurer, G, Littarru, GP, Kumar, A; Rosenfeldt, F	2015
Few adverse effects of long-term creatine supplementation in a placebo-controlled trial	Groeneveld, , GJ, Beijer, C, Veldink, JH, Kalmijn, S, Wokke, JH; van den Berg, LH	2005
Long term outcome of bisphosphonate therapy in patients with primary hyperparathyroidism	Segula, , D, Nikolova, S, Marks, E, Ranganath, L; Mishra, V	2014
One-year overall survival (OS) and biomarker correlates from a phase II study of ipilimumab (IPI) with carboplatin and paclitaxel (CP) in patients with unresectable stage III or IV metastatic melanoma (MM)	Jamal, , R, Van Kempen, L, Thebault, P, Belanger, K, Friedmann, JE, Ayoub, J-PM, Cocolakis, E, Kazemi, S, Dionne, J, Lambert, C, Le, HB, Grange, C, Cailhier, J-F, Spatz, A, Lapointe, R; Miller, WH	2015

A randomized, double-blind, placebo-controlled, parallel-group study to assess the efficacy and safety of dual ACE/NEP inhibitor GW660511X in mild-to-moderate hypertensive patients	Johnson, , AG, Pearce, GL; Danoff, TM	2006
Childhood and adolescent large-cell lymphoma (LCL): a review of the Children's Cancer Group experience	Cairo, , MS, Sposto, R, Hoover-Regan, M, Meadows, AT, Anderson, JR, Siegel, SE, Kadin, ME, Kjeldsberg, CR, Wilson, JF, Perkins, SL, Lones, MA, Morris, E; Finlay, JL	2003
The ideal time of systemic metronidazole and amoxicillin administration in the treatment of severe periodontitis: study protocol for a randomized controlled trial	Feres, , M, Retamal-Valdes, B, Mestnik, MJ, de Figueiredo, LC, Faveri, M, Duarte, PM, Fritoli, A, Faustino, E, Souto, MLS, de Franco Rodrigues, M, Giudicissi, M, Nogueira, BCL, Saraiva, L, Romito, GA; Pannuti, CM	2018
Analysis of minimal residual disease in follicular lymphoma patients in gadolin, a phase III study of obinutuzumab plus bendamustine versus bendamustine in relapsed/refractory indolent non-hodgkin lymphoma	Pott, , C, Belada, D, Danesi, N, Fingerle-Rowson, G, Gribben, J, Harbron, C, Hoster, E, S Kahl B, , Mundt, K, Sebban, C, Sehn, LH; Cheson, BD	2015
Assessment of inhaled BDP-dose dependency of exhaled nitric oxide and local and serum eosinophilic markers in steroids-naive nonatopic asthmatics	Dal Negro, , R, Micheletto, C, Tognella, S, Turco, P, Rossetti, A; Cantini, L	2003
Clinical characteristics predict benefits from eptifibatide therapy during coronary stenting: insights from the enhanced suppression of the platelet IIb/IIIa receptor with integrilin therapy (ESPRIT) Trial	Puma, , JA, Banko, LT, Pieper, KS, Sacchi, TJ, O'Shea, JC, Dery, JP; Tcheng, JE	2006
Comparison of additional metformin or NPH insulin to mealtime insulin lispro therapy with mealtime human insulin therapy in secondary OAD failure	Altuntas, , Y, Ozen, B, Ozturk, B, Sengul, A, Ucak, S, Ersoy, O; Karul, S	2003
Effects of renin-angiotensin-aldosterone system inhibitors and beta-blockers on markers of arterial stiffness	Koumaras, , C, Tziomalos, K, Stavrinou, E, Katsiki, N, Athyros, VG, Mikhailidis, DP; Karagiannis, A	2014
Eosinophil-guided corticosteroid therapy in patients admitted to hospital with COPD exacerbation (CORTICO-COP): a multicentre, randomised, controlled, open-label, non-inferiority trial	Sivapalan, , P, Lapperre, TS, Janner, J, Laub, RR, Moberg, M, Bech, CS, Eklof, J, Holm, FS, Armbruster, K, Sivapalan, P, Mosbech, C, Ali, AKM, Seersholm, N, Wilcke, JT, Brondum, E, Sonne, TP, Ronholt, F, Andreassen, HF, Ulrik, CS, Vestbo, J; Jensen, J-US	2019
Effects of Freeze-Dried Grape Powder on High-Density Lipoprotein Function in Adults with Metabolic Syndrome: a Randomized Controlled Pilot Study	Millar, , CL, Duclos, Q, Garcia, C, Norris, GH, Lemos, BS, DiMarco, DM, Fernandez, ML; Blesso, CN	2018
Study of the Efficacy and Safety of Olokizumab in Patients with Moderately to Severely Active Rheumatoid Arthritis	EUCTR2015-005309-35-HU	2017
A Study of the Efficacy and Safety of Atezolizumab Plus Chemotherapy for Patients With Early Relapsing Recurrent Triple-Negative Breast Cancer	RPCEC00000280	2018
Combretastin A-4 phosphate in combination with carboplatin and paclitaxel chemotherapy in patients with advanced cancer	ISRCTN57858356	2010
GOLMePsA	EUCTR2013-004122-28-GB	2015
Development of measuring method to monitor progression of cognitive decline and its improvement by intervention in dementia and mild cognitive impairment (MCI)	JPRN-UMIN000025053	2016
The CURE - CD Trial	NCT03555058	2018
A prospective open randomised controlled trial of women diagnosed with premature ovarian failure (POF) to investigate the effects of active treatment with HRT (hormone replacement therapy) or COCP (combined oral contraceptive pill), and observation of patients who choose to have no treatment, on bone density, markers of cardiovascular disease, markers of bone metabolism, menopausal symptoms, quality of life, depression score, sexual function and ovarian function over 2 years. - Treatment of Premature Ovarian Failure	EUCTR2008-002599-86-GB	2009
Alzheimer Disease Proof of Concept Study with BI 409306 versus Placebo	EUCTR2013-005031-24-BE	2014
AN OPEN-LABEL, RANDOMIZED PHASE 3 TRIAL OF NIVOLUMAB, OR NIVOLUMAB PLUS IPILIMUMAB, VERSUS PLATINUM DOUBLET CHEMOTHERAPY IN SUBJECTS WITH CHEMOTHERAPY-NAÏVE STAGE IV OR RECURRENT NON-SMALL CELL LUNG CANCER (NSCLC)	PER-049-15	2016
Ginger effect on chemotherapy-induced neuropathy	IRCT2013121415780N1	2014
Acute effects of continuous positive air way pressure on pulse pressure in chronic heart failure	Quintão, , M, Chermont, S, Marchese, L, Brandão, L, Bernardez, SP, Mesquita, ET, Novaes Rocha, Nd; Nóbrega, AC	2014
Assessment of the probiotic Symprove as a dietary supplement in patients with symptomatic diverticular disease	ISRCTN71661079	2013
A randomised, double-blind, one-year controlled trial comparing Aclasta (zoledronic acid) 5 mg intravenously (i/v) against placebo infusion in patients with anti-epileptic (AED) drug induced bone loss	ACTRN12611000507987	2011
A study evaluating the effects of RGB-03 and MabThera combined with Methotrexate in patients with Rheumatoid Arthritis	EUCTR2014-003255-54-CZ	2014
Effect of an oral nutritional supplement therapy on muscle mass, bone metabolism, body composition, growth, and disease activity in pediatric Crohn’s Disease: a controlled trial	DRKS00010278	2016
A RANDOMIZED, DOUBLE-BLIND, PHASE 3 STUDY OF THE JAK1/2 INHIBITOR RUXOLITINIB OR PLACEBO IN COMBINATION WITH CAPECITABINE IN SUBJECTS WITH ADVANCED OR METASTATIC ADENOCARCINOMA OF THE	PER-001-15	2015

PANCREAS WHO HAVE FAILED OR ARE INTOLERANT TO FIRST-LINE CHEMOTHERAPY (THE JANUS 2 STUDY)		
Prospective, phase II randomized study to compare busulfan-fludarabine reduced-intensity conditioning (RIC) with thiotepa-fludarabine RIC regimen prior to allogeneic transplantation of hematopoietic cells for the treatment of myelofibrosis	EUCTR2010-022052-23-IT	2011
Multicenter phase II study of modified FOLFOX6 and bevacizumab in chemonaive advanced or recurrent colorectal cancer	JPRN-UMIN000001490	2008
OUTREACH study: urine analysis and antihypertensive treatment	ISRCTN15911100	2017
Double-blind trial of the efficacy of pentoxifylline vs thalidomide for the treatment of type II reaction in leprosy	Sales, , AM, de Matos, HJ, Nery, JA, Duppre, NC, Sampaio, EP; Sarno, EN	2007
A randomized controlled trial of the effect of substantial weight loss on obstructive sleep apnoea as measured by the apnoea/hypopnoea index	ACTRN12605000161628	2005
Sulfasalazine in the treatment of spondylarthropathy. A randomized, multicenter, double-blind, placebo-controlled study	Dougados, , M, vam der Linden, S, Leirisalo-Repo, M, Huitfeldt, B, Juhlin, R, Veys, E, Zeidler, H, Kvien, TK, Olivieri, I; Dijkmans, B	1995
PPALM - Palm oil and Pentoxifylline Against Late Morbidity	EUCTR2012-004211-31-GB	2014
A trial to assess whether the addition of atezolizumab to current standard treatment for patients with relapsed or refractory Diffuse Large B-Cell Lymphoma, who are not able to have high dose therapy, improves survival outcomes	ISRCTN11965217	2017
A study to test how well a new medicine called sirukumab works in patients with Severe Uncontrolled Asthma	EUCTR2016-001244-19-ES	2016
Are inorganic nitrates protective for fatty liver?	ISRCTN36524222	2017
Can atorvastatin improve vascular function in women with a history preeclampsia? A Randomised, double-blinded, placebo-controlled crossover trial of atorvastatin in women with a history of preeclampsia. - Preeclampsia Vascular Studies Project 3 PVS3	EUCTR2008-005759-21-GB	2011
Edoxaban treatment versus anticoagulant treatment (here Vitamin K antagonist) in patients with atrial fibrilation undergoing a catheter ablation	EUCTR2016-003069-25-HU	2017
Genetic determinants of the response to bezafibrate treatment in the lower extremity arterial disease event reduction (LEADER) trial	Jamshidi, , Y, Flavell, DM, Hawe, E, MacCallum, PK, Meade, TW; Humphries, SE	2002
Osteogenesis and osteoclast inhibition in rheumatoid arthritis patients after more than 4 years of treatment with bisphosphonates or bisphosphonates with pitavastatin	ISRCTN45993953	2012
Randomised Controlled Trial Addressing Wound Debridement Frequency and Healing Outcomes in Diabetic Foot Ulcers	ACTRN12618000703202	2018
Ayurvedic treatment for Metabolic Syndrome a disease which includes obesity, elevated blood pressure, abnormal lipids with early diabetes at one place	CTRI/2015/06/005899	2015
OPEN LABEL RANDOMIZED BIOEQUIVALENCE STUDY TO EVALUATE THE PHARMACOKINETIC (PK) AND SAFETY PROFILE OF BEVACIZUMAB BIOSIMILAR (BEVZ92) IN COMBINATION WITH FOLFOX OR FOLFIRI VERSUS BEVACIZUMAB (AVASTIN®) IN COMBINATION WITH FOLFOX OR FOLFIRI AS FIRST-LINE TREATMENT IN PATIENTS WITH METASTATIC COLORECTAL CANCER (MCRC)	PER-076-13	2014
The ALPHA Trial	EUCTR2014-002305-38-GB	2014
A study in newborn children in need for intensive treatment resulting from insufficient oxygen supply to the brain	EUCTR2011-002502-74-LT	2011
A Randomized, Open-Label, Active-Controlled Study of the Safety, Efficacy, and Pharmacokinetics of Ferumoxytol Compared with Oral Iron for the Treatment of Iron Deficiency Anemia in Pediatric Subjects with Nondialysis-dependent Chronic Kidney Disease	EUCTR2010-019388-12-RO	2011
IBDmindfulness-mindfulness for youth with Inflammatory Bowel Disease (IBD) and depression trial	ACTRN12617000876392	2017
A 4-week double-blind placebo-controlled pilot study, evaluating niacin-induced flushing and lipid parameter effects of V0002 CA 1g 3 capsules/day associated with Niaspan® (from 375 mg to 1000 mg) in addition to usual care statin, in patients suffering from dyslipidemia with uncontrolled elevated triglycerides	EUCTR2007-006393-29-FR	2008
A randomized controlled trial for Qingjinhuazhuo formula for shortening course of antibiotic treatment of infectious acute exacerbation of chronic obstructive pulmonary disease (AECOPD)	ChiCTR1800019723	2018
Aspirin as a new treatment for a disease called ARDS	EUCTR2014-002564-32-GB	2015
A phase 2, double-blind, placebo-controlled study of the safety and tolerability of etanercept in patients with Alzheimer's disease - Safety and Tolerability of Etanercept in Alzheimer's Disease	EUCTR2009-013400-31-GB	2010
A CR-UK phase I study of BKM120 in patients with non-small cell lung cancer (NSCLC) receiving thoracic radiotherapy	ISRCTN04813360	2013
A study to assess the efficacy and safety of GSK2586184 in SLE	EUCTR2012-001645-41-SE	2012
Prospective randomized ARMYDA 600x2 STUDY	EUCTR2011-006210-13-IT	2012

A study to investigate the efficacy of sirukumab in addition to the current antidepressant treatment in adults with depression	EUCTR2014-005206-37-PL	2015
A Study of Gantenerumab in Patients with Mild Alzheimer Disease	EUCTR2013-003390-95-BE	2014
Clinical Efficacy and Safety of Baminercept, a Lymphotoxin β Receptor Fusion Protein, in Primary Sjögren's Syndrome: results From a Phase II Randomized, Double-Blind, Placebo-Controlled Trial	St Clair, , EW, Baer, AN, Wei, C, Noaiseh, G, Parke, A, Coca, A, Utset, TO, Genovese, MC, Wallace, DJ, McNamara, J, Boyle, K, Keyes-Elstein, L, Browning, JL, Franchimont, N, Smith, K, Guthridge, JM, Sanz, I; James, JA	2018
The MET REMODEL Study	EUCTR2014-003189-26-GB	2014
CALC trial: aprepitant for the treatment of cough in lung cancer	ISRCTN16200035	2013
REMODEL-AMI	EUCTR2014-005407-24-CZ	2015
Comparative bioavailability/ bioequivalence study of Lutein formulations - Product C and Product D (Lutein 20 mg/capsule) supplementation in healthy, adult humans, after receiving high-fat, high-calorie breakfast	CTRI/2018/08/015403	2018
Randomised controlled trial evaluating strategies to optimize disease activity control in RA patients treated with infliximab in clinical practice. - Re3	EUCTR2005-001889-13-PT	2006
A Randomized, Open-Label, Phase III Study of Taxane Based Chemotherapy With Lapatinib or Trastuzumab as First-Line Therapy for Women With Human Epidermal Growth Factor Receptor 2 (HER2)/Neu Positive Metastatic Breast Cancer	ACTRN12609000143224	2009
Effect of azithromycin treatment on endothelial function in patients with coronary artery disease and evidence of Chlamydia pneumoniae infection	Parchure, , N, Zouridakis, EG; Kaski, JC	2002
Crossover and randomized clinical trial on the effect of Resincolestiramine in the intestinal absorption of new uremic toxins in patients in hemodialysis with chronic renal insufficiency	EUCTR2017-001899-32-ES	2017
PPALM - Palm oil and Pentoxifylline Against Late Morbidity	ISRCTN17415294	2015
Effect of Liraglutide on lipid synthesis and metabolism after mixed meal. A single-center randomized controlled study	EUCTR2013-005075-40-FI	2014
Study with investigational drug PF-06463922 and comparator crizotinib in patients with a specific type of advanced lung cancer	EUCTR2016-003315-35-PL	2017
Comparison of two antiplatelet agents in patients with acute coronary syndrome	EUCTR2012-002404-41-IT	2012
Role of VSL#3 in patients with nonalcoholic fatty liver disease	CTRI/2008/091/000074	2008
Zoledronic acid in the management of Mesothelioma - a feasibility trial	EUCTR2015-004433-26-GB	2016
Multi-centre, European study to compare how well the standard course of the antibiotic vancomycin works compared to a new vancomycin course in babies aged less than 91 days with sepsis occurring after 3 days of life	EUCTR2015-000203-89-EE	2016
A Phase IIb, Multicenter, Randomized, Double-Blind, Placebo-Controlled Dose-Ranging Study to Evaluate the Safety, Tolerability and Efficacy of Two Doses of Subcutaneous IPP-201101 Plus Standard of Care, Versus Placebo Plus Standard of Care in Subjects with Systemic Lupus Erythematosus (SLE)	EUCTR2007-004892-21-BG	2008
Orally inhaled heparin in patients with cystic fibrosis (CF)	ACTRN12610000328077	2010
Phase II study Nivolumab in previously treated non-small-cell lung cancer subjects to evaluate the peripheral blood mononuclear cell immunological biomarker	JPRN-UMIN000028468	2017
Prognostic value of serum cardiac troponin I and T in chronic dialysis patients: a 1-year outcomes analysis	Apple, , FS, Sharkey, SW, Hoeft, P, Skeate, R, Voss, E, Dahlmeier, BA; Preese, LM	1997
Study of a new drug's effect on anemia in subjects with impaired kidney function who are not on dialysis	EUCTR2012-004050-29-DE	2012
Randomized controlled trial of a TiO2 Semiconductor toothbrush on mild-to-moderate periodontitis	Quest, DW	2015
PAF-acetylhydrolase activity in plasma of patients with chronic kidney disease. Effect of long-term therapy with erythropoietin	Papavasiliou, , EC, Gouva, C, Siamopoulos, KC; Tselepis, AD	2006
Comparison of efficacy between combination therapy with iguratimod and sulfasalazine with methotrexate in japanese patients with rheumatoid arthritis: propensity score analysis	Tokunaga, , K, Shiraishi, A, Oshikawa, H, Hagino, N, Nishino, J; Tohma, S	2017
Adding monounsaturated fatty acids to a dietary portfolio of cholesterol-lowering foods in hypercholesterolemia	Jenkins, , DJ, Chiavaroli, L, Wong, JM, Kendall, C, Lewis, GF, Vidgen, E, Connelly, PW, Leiter, LA, Josse, RG; Lamarche, B	2010
Clinical efficacy and immunological effects of weekly administration of interferon-β in patients with type B chronic hepatitis	Katayama, , K, Hayashi, N, Ito, T, Masuzawa, M, Kishida, Y, Yoshihara, H, Akeyama, T, Shimizu, Y, Moriyama, M, Kasahara, A, Fusamoto, H; Kamada, T	1999
Ixekizumab treatment shows a neutral impact on cardiovascular parameters in patients with moderate-to-severe plaque psoriasis: results from UNCOVER-1, UNCOVER-2, and UNCOVER-3	Egeberg, , A, Wu, JJ, Korman, N, Solomon, JA, Goldblum, O, Zhao, F; Mallbris, L	2018
Effect of catheter ablation on the left ventricular mass index and other echocardiograph parameters in atrial fibrillation patients: comparison with antiarrhythmic drug treatment	Kim, , K-H, Na, JO, Nam, G-B, Jin, E-S, Choi, H, Kim, S-H, Choi, K-J; Kim, Y-H	2011

The effect of vitamin D supplementation on serum lipids in postmenopausal women with diabetes: a randomized controlled trial	Munoz-Aguirre, , P, Flores, M, Macias, N, Quezada, AD, Denova-Gutierrez, E; Salmeron, J	2015
Rheohaemapheresis in the treatment of nonvascular age-related macular degeneration	Blaha, , M, Rencova, E, Langrova, H, Studnicka, J, Blaha, V, Rozsival, P, Lanska, M; Sobotka, L	2013
Oral ferrous fumarate or intravenous iron sucrose for patients with inflammatory bowel disease	Erichsen, , K, Ulvik, RJ, Nysaeter, G, Johansen, J, Ostborg, J, Berstad, A, Berge, RK; Hausken, T	2005
Effects of a Combined Nutraceutical on Lipid Pattern, Glucose Metabolism and Inflammatory Parameters in Moderately Hypercholesterolemic Subjects: a Double-blind, Cross-over, Randomized Clinical Trial	Cicero, , AF, Colletti, A, Fogacci, F, Bove, M, Rosticci, M; Borghi, C	2017
Use of cytokines in the treatment of acute myelocytic leukemia: a critical review	Geller, RB	1996
Results from a Phase IIA Parallel Group Study of JNJ-40346527, an Oral CSF-1R Inhibitor, in Patients with Active Rheumatoid Arthritis despite Disease-modifying Antirheumatic Drug Therapy	Genovese, , MC, Hsia, E, Belkowski, SM, Chien, C, Masterson, T, Thurmond, RL, Manthey, CL, Yan, XD, Ge, T, Franks, C; Greenspan, A	2015
Peroxisome proliferator-activated receptor γ agonist effect on rheumatoid arthritis: a randomized controlled trial	Ormseth, , MJ, Oeser, AM, Cunningham, A, Bian, A, Shintani, A, Solus, J, Tanner, S; Stein, CM	2013
Xuezhikang, an extract of cholestin, protects endothelial function through antiinflammatory and lipid-lowering mechanisms in patients with coronary heart disease	Zhao, , SP, Liu, L, Cheng, YC, Shishehbor, MH, Liu, MH, Peng, DQ; Li, YL	2004
Bay leaves improve glucose and lipid profile of people with type 2 diabetes	Khan, , A, Zaman, G; Anderson, RA	2009
Comparison of effectiveness of rosuvastatin versus atorvastatin on the achievement of combined C-reactive protein (<2 mg/L) and low-density lipoprotein cholesterol (< 70 mg/dl) targets in patients with type 2 diabetes mellitus (from the ANDROMEDA study)	Betteridge, , DJ, Gibson, JM; Sager, PT	2007
Eicosapentaenoic acid for the prevention of recurrent atrial fibrillation	Watanabe, , E, Sobue, Y, Sano, K, Okuda, K, Yamamoto, M; Ozaki, Y	2011
Itraconazole comedication increases systemic levels of inhaled fluticasone in lung transplant recipients	Naef, , R, Schmid, C, Hofer, M, Minder, S, Speich, R; Boehler, A	2007
Initial treatment with rosiglitazone/metformin fixed-dose combination therapy compared with monotherapy with either rosiglitazone or metformin in patients with uncontrolled type 2 diabetes	Rosenstock, , J, Rood, J, Cobitz, A, Biswas, N, Chou, H; Garber, A	2006
Effects of anthocyanins on cardiovascular risk factors and inflammation in pre-hypertensive men: a double-blind randomized placebo-controlled crossover study	Hassellund, , SS, Flaa, A, Kjeldsen, SE, Seljeflot, I, Karlsen, A, Erlund, I; Rostrup, M	2013
DHA-enriched high-oleic acid canola oil improves lipid profile and lowers predicted cardiovascular disease risk in the canola oil multicenter randomized controlled trial	Jones, , PJ, Senanayake, VK, Pu, S, Jenkins, DJ, Connelly, PW, Lamarche, B, Couture, P, Charest, A, Baril-Gravel, L, West, SG, Liu, X, Fleming, JA, McCrea, CE; Kris-Etherton, PM	2014
The effects of garlic extract upon endothelial function, vascular inflammation, oxidative stress and insulin resistance in adults with type 2 diabetes at high cardiovascular risk. A pilot double blind randomized placebo controlled trial	Atkin, , M, Laight, D; Cummings, MH	2016
The rate of clinical progression and brain atrophy is greater with increasing severity of Alzheimer's disease: results from the volumetric MRI substudies of two phase III trials with bapineuzumab	Novak, , G, Einstein, S, Tudor, IC, Gregg, K, Collins, P, Wyman, B, Yuen, E, Nielsen, C, Grundman, M, Brashear, RH, Fox, N; Liu, E	2013
A phase ib study of alpelisib (BYL719), a PI3Kalpha-specific inhibitor, with letrozole in ER+/HER2- metastatic breast cancer	Mayer, , IA, Abramson, VG, Formisano, L, Balko, JM, Estrada, MV, Sanders, ME, Juric, D, Solit, D, Berger, MF, Won, HH, Li, Y, Cantley, LC, Winer, E; Arteaga, CL	2017
Baicalin reduces blood lipids and inflammation in patients with coronary artery disease and rheumatoid arthritis: a randomized, double-blind, placebo-controlled trial	Hang, , Y, Qin, X, Ren, T; Cao, J	2018
DEMO-II trial. Aerobic exercise versus stretching exercise in patients with major depression-a randomised clinical trial	Krogh, , J, Videbech, P, Thomsen, C, Gluud, C; Nordentoft, M	2012
Effects of Long-Term Etanercept Treatment on Clinical Outcomes and Objective Signs of Inflammation in Early Nonradiographic Axial Spondyloarthritis: 104-Week Results From a Randomized, Placebo-Controlled Study	Dougados, , M, van der Heijde, D, Sieper, J, Braun, J, Citera, G, Lenaerts, J, van den Bosch, F, Wei, JC, Pedersen, R, Bonin, R, Jones, H, Marshall, L, Logeart, I, Vlahos, B, Bukowski, JF; Maksymowych, WP	2017
Effect of interval exercise training programme on C-reactive protein in the non-pharmacological management of hypertension: a randomized controlled trial	Lamina, , S; Okoye, GC	2012
Beneficial Effects of Pentoxifylline Plus Losartan Dual Therapy in Type 2 Diabetes with Nephropathy	Rabizadeh, , S, Dehghani Firouzabadi, F, Noshad, S, Esteghamati, S, Afarideh, M, Ghajar, A, Ganji, M, Saadat, M, Heidari, B, Najafi, MT, Nakhjavani, M; Esteghamati, A	2018
Drug concentrations, adherence, patient-reported symptoms and health-related quality of life in HIV-infected, virologically controlled patients switching to maraviroc+darunavir/ritonavir or continuing the previous triple therapy: sub-studies from the randomized GUSTA trial	Rossetti, , B, Gagliardini, R, Lisi, L, Masini, M, Lamonica, S, Vignale, F, Sterrantino, G, Orofino, G, Colafigli, M, Latini, A, Tosti, A, Rusconi, S, Trezzi, M, D'Avino, A, Grima, P, Mezzaroma, I, Di Biagio, A, Navarra, P, Di Giambenedetto, S; De Luca, A	2016
Steroid-sparing effects of pentoxifylline in pulmonary sarcoidosis	Park, , MK, Fontana, null, Babaali, H, Gilbert-McClain, LI, Stylianou, M, Joo, J, Moss, J; Manganiello, VC	2009
Controlled Trial of an Internet-Based Risk Reduction Intervention in HIV+ Men Who Have Sex With Men	Milam, , J, Jain, S, Daar, E, Dube, M, Seefreid, E, Ellorin, E, Jimenez-Maggiora, G A, Morris, S R; Haubrich, R H	2014
Oral nutritional supplementation is effective in the maintenance of remission in Crohn's disease	Verma, , S, Kirkwood, B, Brown, S; Giaffer, MH	2000
Chagas Cardiomyopathy Bisoprolol Intervention Study: charity	NCT00323973	2006

Randomized Trial of Bicarbonate or Saline Study for the Prevention of Contrast-Induced Nephropathy in Patients with CKD	Solomon, , R, Gordon, P, Manoukian, SV, Abbott, JD, Kereiakes, DJ, Jeremias, A, Kim, M; Dauerman, HL	2015
Pirfenidone in patients with unclassifiable progressive fibrosing interstitial lung disease: demographic and baseline characteristics	Maher, , TM, Corte, TJ, Fischer, A, Kreuter, M, Lederer, DJ, Molina-Molina, M, Samara, K, Axmann, J, Kirchgaessler, K; Cottin, V	2018
Efficacy of pre-exercise low-level laser therapy on isokinetic muscle performance in individuals with type 2 diabetes mellitus: study protocol for a randomized controlled trial	Gomes, , CA, Leal-Junior, EC, Biasotto-Gonzalez, DA, El-Hage, Y, Politti, F, Gonzalez Tde, O, Dibai-Filho, AV, de Oliveira, AR, Frigero, M, Antonialli, FC, Vanin, AA; de Tarso Camillo de Carvalho, P	2014
Efficacy and safety of mavrilimumab in Japanese subjects with rheumatoid arthritis: findings from a Phase IIa study	Takeuchi, , T, Tanaka, Y, Close, D, Godwood, A, Wu, CY; Saurigny, D	2015
Baseline myeloid-derived suppressor cell and eosinophil cell counts predict clinical efficacy in patients with non-small cell lung cancer (NSCLC) treated with nivolumab in second line	Mancuso, , P, Passaro, A, Labanca, V, Gandini, S, Spitaleri, G, Guerini, E, Barberis, M, Noberasco, C, Del Signore, E, Pochesci, A, Catania, C, De Marinis, F; Bertolini, F	2017
Phase II, multicenter, open-label, randomized study of YM155 plus docetaxel as first-line treatment in patients with HER2-negative metastatic breast cancer	Clemens, , MR, Gladkov, OA, Gartner, E, Vladimirov, V, Crown, J, Steinberg, J, Jie, F; Keating, A	2015
Long-term follow-up of a multicenter, randomized controlled trial of ursodeoxycholic acid in primary sclerosing cholangitis	Hilscher, , M, Tabibian, JH, Enders, FB, Carey, EJ; Lindor, KD	2015
The effect of sea buckthorn supplement on oral health, inflammation, and DNA damage in hemodialysis patients: a double-blinded, randomized crossover study	Rodhe, , Y, Woodhill, T, Thorman, R, Möller, L; Hylander, B	2013
Effects of trimetazidine on myocardial perfusion and left ventricular systolic function in type 2 diabetic patients with ischemic cardiomyopathy	Belardinelli, , R, Cianci, G, Gigli, M, Mazzanti, M; Lacalaprice, F	2008
Plasma oxysterols: biomarkers for diagnosis and treatment in spastic paraplegia type 5	Marelli, , C, Lamari, F, Rainteau, D, Lafourcade, A, Banneau, G, Humbert, L, Monin, M-L, Petit, E, Debs, R, Castelnovo, G, Ollagnon, E, Lavie, J, Pilliod, J, Coupry, I, Babin, PJ, Guissart, C, Benyounes, I, Ullmann, U, Lesca, G, Thauvin-Robinet, C, Labauge, P, Odent, S, Ewenczyk, C, Wolf, C, Stevanin, G, Hajage, D, Durr, A, Goizet, C; Mochel, F	2018
Progressive Resistance Exercise Training in CKD: a Feasibility Study	Watson, , EL, Greening, NJ, Viana, JL, Aulakh, J, Bodicoat, DH, Barratt, J, Feehally, J; Smith, AC	2015
Phase I pilot clinical trial of antenatal maternally administered melatonin to decrease the level of oxidative stress in human pregnancies affected by pre-eclampsia (PAMPR): study protocol	Hobson, , SR, Lim, R, Gardiner, EE, Alers, NO; Wallace, EM	2013
Deflazacort versus methylprednisolone in polymyalgia rheumatica: clinical equivalence and relative antiinflammatory potency of different treatment regimens	Di Munno, , O, Imbimbo, B, Mazzantini, M, Milani, S, Occhipinti, G; Pasero, G	1995
Relation of inflammation and benefit of statins after percutaneous coronary interventions	Chan, , AW, Bhatt, DL, Chew, DP, Reginelli, J, Schneider, JP, Topol, EJ; Ellis, SG	2003
Safety, efficacy, tolerability and pharmacokinetics of azithromycin plus piperaquine as a candidate for intermittent preventive treatment for malaria in pregnancy	Moore, , BR, Benjamin, J, Salman, S, Page-Sharp, M, Laman, M, Batty, KT, Mueller, I, Rogerson, S; Davis, TM	2018
A randomised double-blind placebo-controlled pilot trial of a combined extract of sage, rosemary and melissa, traditional herbal medicines, on the enhancement of memory in normal healthy subjects, including influence of age	Perry, , NSL, Menzies, R, Hodgson, F, Wedgewood, P, Howes, MR, Brooker, HJ, Wesnes, KA; Perry, EK	2018
Inorganic nitrate supplementation enhances functional capacity and lower-limb microvascular reactivity in patients with peripheral artery disease	Bock, , JM, Treichler, DP, Norton, SL, Ueda, K, Hughes, WE; Casey, DP	2018
Full-spectrum light phototherapy for atopic dermatitis	Byun, , HJ, Lee, HI, Kim, B, Kim, MN, Hong, H, Choi, Y, Jo, Y, Cho, KH; Mun, SK	2011
Eicosapentaenoic acid (EPA) efficacy for colorectal aberrant crypt foci (ACF): a double-blind randomized controlled trial	Higurashi, , T, Hosono, K, Endo, H, Takahashi, H, Iida, H, Uchiyama, T, Ezuka, A, Uchiyama, S, Yamada, E, Ohkubo, H, Sakai, E, Maeda, S, Morita, S, Natsumeda, Y, Nagase, H; Nakajima, A	2012
The effect of HLA-DRB1 genes, rheumatoid factor, and treatment on radiographic disease progression in rheumatoid arthritis over 6 years	Rau, , R, Herborn, G, Zueger, S; Fenner, H	2000
Does exercise intensity affect blood pressure and heart rate in obese adolescents? A 6-month multidisciplinary randomized intervention study	Farah, , BQ, Ritti-Dias, RM, Balagopal, PB, Hill, JO; Prado, WL	2014
Changes in basophil activation during grass-pollen sublingual immunotherapy do not correlate with clinical efficacy	Van Overtvelt, , L, Baron-Bodo, V, Horiot, S, Moussu, H, Ricarte, C, Horak, F, Zieglmayer, P, Zieglmayer, R, Montagut, A, Galvain, S, De Beaumont, O, Le Gall, M; Moingeon, P	2011
Placebo response in two long-term randomized psoriasis studies that were negative for rosiglitazone	Ellis, , CN, Barker, JN, Haig, AE, Parker, CA, Daly, S; Jayawardene, DA	2007
Decreased oxidative stress in patients with ulcerative colitis supplemented with fish oil omega-3 fatty acids	Barbosa, , DS, Cecchini, R, El Kadri, MZ, Rodríguez, MA, Burini, RC; Dichi, I	2003
Physical activity coaching of patients with rheumatoid arthritis in everyday practice: a long-term follow-up	Sjöquist, , ES, Brodin, N, Lampa, J, Jensen, I; Opava, CH	2011
Effects of sulfurous, salty, bromic, iodic thermal water nasal irrigations in nonallergic chronic rhinosinusitis: a prospective, randomized, double-blind, clinical, and cytological study	Ottaviano, , G, Marioni, G, Staffieri, C, Giacomelli, L, Marchese-Ragona, R, Bertolin, A; Staffieri, A	2011
Omega-3 polyunsaturated fatty acid in peripheral arterial disease: effect on lipid pattern, disease severity, inflammation profile, and endothelial function	Schiano, , V, Laurenzano, E, Brevetti, G, De Maio, JI, Lanero, S, Scopacasa, F; Chiariello, M	2008
Aggressive hydraTion in patients with ST-Elevation Myocardial infarction undergoing Primary percutaneous coronary intervention to	Liu, , Y, Chen, JY, Huo, Y, Ge, JB, Xian, Y, Duan, CY, Chen, SQ, Jiang, W, Chen, PY; Tan, N	2016

prevenT contrast-induced nephropathy (ATTEMPT): study design and protocol for the randomized, controlled trial, the ATTEMPT, RESCIND 1 (First study for REduction of contraSt-induCed nephropathy followINg carDiac catheterization) trial		
A randomised controlled trial of early insulin therapy in very low birth weight infants, "NIRTURE" (neonatal insulin replacement therapy in Europe)	Beardsall, , K, Vanhaesebrouck, S, Ogilvy-Stuart, AL, Ahluwalia, JS, Vanhole, C, Palmer, C, Midgley, P, Thompson, M, Cornette, L, Weissenbruch, M, Thio, M, de Zegher, F; Dunger, D	2007
Fluvastatin improves arterial stiffness in patients with coronary artery disease and hyperlipidemia: a 5-year follow-up study	Hongo, , M, Tsutsui, H, Mawatari, E, Hidaka, H, Kumazaki, S, Yazaki, Y, Takahashi, M, Kinoshita, O; Ikeda, U	2008
Platelet response to aspirin 50 and 100 mg in patients with coronary heart disease over a five-year period	Berent, , R, Auer, J, Franklin, B, Schmid, P; von Duvillard, SP	2011
Carnitine supplementation in premature neonates: effect on plasma and red blood cell total carnitine concentrations, nutrition parameters and morbidity	Crill, , CM, Storm, MC, Christensen, ML, Hankins, CT, Bruce Jenkins, M; Helms, RA	2006
A double-blinded, randomized controlled trial of zoledronate therapy for HIV-associated osteopenia and osteoporosis	Huang, , J, Meixner, L, Fernandez, S; McCutchan, JA	2009
Effect of immune modulation therapy on cardiac function and T-bet/GATA-3 gene expression in aging male patients with chronic cardiac insufficiency	Han, , LN, Guo, SL, Li, TL, Ding, GL, Zhang, YJ; Ma, JL	2013
Improvements in seasonal allergic disease with Lactobacillus plantarum No. 14	Nagata, , Y, Yoshida, M, Kitazawa, H, Araki, E; Gomyo, T	2010
Clinical safety evaluation of marine oil derived from Calanus finmarchicus	Tande, , KS, Vo, TD; Lynch, BS	2016
First-line pemetrexed plus cisplatin followed by gefitinib maintenance therapy versus gefitinib monotherapy in East Asian patients with locally advanced or metastatic non-squamous non-small cell lung cancer: a randomised, phase 3 trial	Yang, , JC, Kang, JH, Mok, T, Ahn, MJ, Srimuninnimit, V, Lin, CC, Kim, DW, Tsai, CM, Barraclough, H, Altug, S, Orlando, M; Park, K	2014
Effect of allopurinol on the glomerular filtration rate of children with chronic kidney disease	Ghane Sharbaf, , F; Assadi, F	2018
Percutaneous cholecystostomy: a definite treatment for the the acalculous and calculous acute cholecystitis in the extreme morbid patients by the well-designed cholecystostomy management protocol	Cha, , BH, Lee, SH, Kim, YN, Jeon, WJ, Lee, SJ, Kim, JD; Lee, HH	2012
Potassium supplementation and long-term outcomes in chronic peritoneal dialysis patients with end-stage renal disease: a propensity score matching study	Zhang, , Y-F, Wang, Q, Su, Y-Y, Yang, S, Guo, J, Luo, J, Tang, J-M; Li, H-Y	2016
A Randomized Controlled Pilot Study Comparing the Impact of Aprepitant and Fosaprepitant on Chemotherapy Induced Nausea and Vomiting in Patients Treated for Gynecologic Cancer	Micha, , JP, Rettenmaier, MA, Brown, JV, Mendivil, A, Abaid, LN, Lopez, KL; Goldstein, BH	2016
Clinical and immunological consequences of total glucosides of paeony treatment in Sjögren's syndrome: a randomized controlled pilot trial	Zhou, , Y, Jin, L, Kong, F, Zhang, H, Fang, X, Chen, Z, Wang, G, Li, X; Li, X	2016
Rifaximin and Norfloxacin for Prevention of SBP in Adults With Decompensated Cirrhosis	NCT03695705	2018
Safety and efficacy of adalimumab treatment in Japanese patients with psoriasis: results of SALSA study	Asahina, , A, Torii, H, Ohtsuki, M, Tokimoto, T, Hase, H, Tsuchiya, T, Shinmura, Y, Reyes Servin, O; Nakagawa, H	2016
Investigation into the efficiency of ultrasound of different intensities in degenerative tendopathy of the shoulder	Smolenski, , UC, Jeremies, KJ; Schreiber, TU	2001
Ocrelizumab, a humanized anti-CD20 monoclonal antibody, in the treatment of patients with rheumatoid arthritis: a phase I/II randomized, blinded, placebo-controlled, dose-ranging study	Genovese, , MC, Kaine, JL, Lowenstein, MB, Del Giudice, J, Baldassare, A, Schechtman, J, Fudman, E, Kohen, M, Gujrathi, S, Trapp, RG, Sweiss, NJ, Spaniolo, G; Dummer, W	2008
Elevated relapse rate under oral methotrexate versus leflunomide for maintenance of remission in Wegener's granulomatosis	Metzler, , C, Miehle, N, Manger, K, Iking-Konert, C, de Groot, K, Hellmich, B, Gross, WL; Reinhold-Keller, E	2007
GOG-3003: identifying biomarkers predictive of immune and clinical response in a phase 2 RCT of chemo-immunotherapy in recurrent ovarian cancer	Coukos, , G, Brady, MF, Lankes, HA, Aghajanian, C, Gottardo, R, Swisher, EM, Kandalaft, L, Foukas, PG, Rizack, T, Leach, J, Salani, R, Higgins, R, Hanjani, P, Morgan, M, Edwards, R, Bryan, JK, Manjarrez, KL, Mannel, RS, Hershberg, RM; Monk, BJ	2016
Two doses of pamidronate in infants with osteogenesis imperfecta	Senthilnathan, , S, Walker, E; Bishop, NJ	2008
Prognostic Value of the Delivery Dialysis Dose on Twice-Weekly Hemodialysis Patients	Fan, , Q, Yan, Y, Gu, L, He, L, Chen, N, Jiang, G, Yuan, L, Xue, J, Zhang, Y, Ma, J, Jin, H, Yuan, W, Guo, Z, Guo, L, Wang, N, Zhang, W, Ye, Z, Mao, P, Pi, X, Lu, R, Zhu, M, Zhang, W, Ni, Z, Qian, J; Pang, H	2017
Strategies To OPpose Sugars With Non-nutritive Sweeteners Or Water (STOP Sugars NOW) Trial	NCT03543644	2018
Higher incidence of secondary AML and adverse molecular markers, together with lower CR and higher AML related death rates in elderly compared to younger patients: results from 2435 patients included in the two german AML intergroup studies	Niederwieser, , D, Hoffmann, VS, Krug, U, Krahl, R, Sauerland, C, Spiekermann, K, Bohlander, SK, Hegenbart, U, Poenisch, W, Vucinic, V, Cross, M, Jaekel, N, Pfirrmann, M, Schulze, A, Kraemer, A, Maschmeyer, G, Junghanss, C, Scholl, S, Jakob, C, Heinicke, T, Haehling, D, Peter, N, Kreibich, U, Busemann, C, Al-Ali, HK, Elmaagacli, AH, Opitz, B, Wolf, H-H, Hiddemann, W, Berdel, WE; Buechner, T	2015
A randomized controlled trial of taurolidine heparin versus taurolidine urokinase lock to improve catheter function in hemodialysis	Mohammad, , Z, Hamad, A, Hamdy, A, Chandra, P, Abdul-Latif, T, Ibrahim, R, Fouda, T; Al-Ali, F	2016
Metformin reduces arterial stiffness and improves endothelial function in young women with polycystic ovary syndrome: a randomized, placebo-controlled, crossover trial	Agarwal, , N, Rice, SP, Bolusani, H, Luzio, SD, Dunseath, G, Ludgate, M; Rees, DA	2010
Prognostic value of karyotypic analysis in children and adults with high-risk acute lymphoblastic leukemia included in the PETHEMA ALL-93 trial	Ribera, , JM, Ortega, JJ, Oriol, A, Granada, I, Hernández-Rivas, JM, Parody, R, Bethencourt, C, Rivas, C, Bastida, P, del Potro, E, González-Valentín, ME, Moreno, MJ, Besalduch, J, Fernández-	2002

	Calvo, J, Tormo, M, Arias, J, Molinés, A, Sanz, MA, Maldonado, J, Millá, F, Feliu, E; San Miguel, JF	
A Mixture of 3 Bifidobacteria Decreases Abdominal Pain and Improves the Quality of Life in Children with Irritable Bowel Syndrome	Giannetti, , E, Maglione, M, Alessandrella, A, Strisciuglio, C, De Giovanni, D, Campanozzi, A, Miele, E; Staiano, A	2017
A randomized, placebo-controlled trial of certolizumab pegol (CDP870) for treatment of Crohn's disease	Schreiber, , S, Rutgeerts, P, Fedorak, RN, Khaliq-Kareemi, M, Kamm, MA, Boivin, M, Bernstein, CN, Staun, M, Thomsen, OØ; Innes, A	2005
Abnormal Ventilatory Response to Carbon Dioxide: a Potential Biomarker for Seizure Induced Respiratory Depression & Modification by SSRI	NCT02929667	2016
Canakinumab for acute gouty arthritis in patients with limited treatment options: results from two randomised, multicentre, active-controlled, double-blind trials and their initial extensions	Schlesinger, , N, Alten, RE, Bardin, T, Schumacher, HR, Bloch, M, Gimona, A, Krammer, G, Murphy, V, Richard, D; So, AK	2012
Natural honey and cardiovascular risk factors; effects on blood glucose, cholesterol, triacylglycerole, CRP, and body weight compared with sucrose	Yaghoobi, , N, Al-Waili, N, Ghayour-Mobarhan, M, Parizadeh, SM, Abasalti, Z, Yaghoobi, Z, Yaghoobi, F, Esmaeili, H, Kazemi-Bajestani, SM, Aghasizadeh, R, Saloom, KY; Ferns, GA	2008
The ENHANCE trial: analysis and clinical significance	Padial, LR	2010
Ivacaftor in subjects with cystic fibrosis who are homozygous for the F508del-CFTR mutation	Flume, , PA, Liou, TG, Borowitz, DS, Li, H, Yen, K, Ordoñez, CL; Geller, DE	2012
Plasma visfatin concentrations after a lifestyle intervention were directly associated with inflammatory markers	Bo, , S, Ciccone, G, Baldi, I, Gambino, R, Mandrile, C, Durazzo, M, Gentile, L, Cassader, M, Cavallo-Perin, P; Pagano, G	2009
Effect of renin-angiotensin-aldosterone system inhibition, dietary sodium restriction, and/or diuretics on urinary kidney injury molecule 1 excretion in nondiabetic proteinuric kidney disease: a post hoc analysis of a randomized controlled trial	Waanders, , F, Vaidya, VS, van Goor, H, Leuvenink, H, Damman, K, Hamming, I, Bonventre, JV, Vogt, L; Navis, G	2009
Alendronate is more effective than etidronate for increasing bone mass in osteopenic patients with primary biliary cirrhosis	Guañabens, , N, Parés, A, Ros, I, Alvarez, L, Pons, F, Caballería, L, Monegal, A, Martínez de Osaba, MJ, Roca, M, Peris, P; Rodés, J	2003
Long-term follow-up of a randomized trial on 118 patients with polyarteritis nodosa or microscopic polyangiitis without poor-prognosis factors	Samson, , M, Puechal, X, Devilliers, H, Ribi, C, Cohen, P, Bienvenu, B, Ruivard, M, Terrier, B, Pagnoux, C, Mouthon, L, Guillevin, L, Alcalay, M, Arnal, C, Battesti, J-P, Bernardeau, C, Boullanger, N, Sao, AB, Buzon, J-J, Cabane, J, Cacoub, P, Cantagrel, A, Cevallos, R, Charbonneau, J, Costedoat, N, Creange, A, Dadoniene, J, de Faucal, P, Defuentes, G, de Seze, J, Delaunay, C, Dubertret, L, Dubois, A, Duhamel, E, Neau, D, Esteve, E, Fior, R, Foullon, P, Gaida, T, Galperine, T, Garcin, J-M, Gil, H, Gilson, B, Girard, T, Godeau, B, Godmer, P, Gueit, I, Hamidou, M, Labussiere, AS, Landron, C, Lanoux, P, Le Berruyer, P-Y, Lhote, F, Lidove, O, Loustaud-Ratti, V, Maillard, H, Mejjad, O, Miossec, P, Paris, J-F, Penalba, C, Pertuiset, E, Philippe, J-M, Ponge, T, Quere, I, Randrianjohany, A, Rankin, E, Saraux, J-L, Schaeffer, A, Schmidt, C, Schneebeli, S, Seigneuric, C, Smail, A, Sparsa, A, Stirnemann, J, Swiader, L, Taillandier, J-P, Thevenot, C, Tiev, K-P, Toledano, C, Velmans, N, Viallard, J-F, Vidal, E, Wahl, D, Wattiaux, M-J, Witte, A, Ziza, J-M, Zoulim, A; Bergere, A	2014
Nutraceutical Effects on Glucose and Lipid Metabolism in Patients with Impaired Fasting Glucose: a Pilot, Double-Blind, Placebo-Controlled, Randomized Clinical Trial on a Combined Product	Cicero, , AFG, Fogacci, F, Morbini, M, Colletti, A, Bove, M, Veronesi, M, Giovannini, M; Borghi, C	2017
PIRATE project: point-of-care, informatics-based randomised controlled trial for decreasing overuse of antibiotic therapy in Gram-negative bacteraemia	Huttner, , A, Albrich, WC, Bochud, P-Y, Gayet-Ageron, A, Rossel, A, Von Dach, E, Harbarth, S; Kaiser, L	2017
Cobimetinib (C) + paclitaxel (P) as first-line treatment in patients (pts) with advanced triple-negative breast cancer (TNBC): updated results and biomarker data from the phase 2 COLET study	Brufsky, , A, Kim, S-B, Velu, TJ, Saenz, JAG, Tan-Chiu, E, Sohn, J, Dirix, LY, Vanasek, J, Borms, MV, Mingorance, JID, Liu, M-C, Moezi, MM, Kozloff, M, Sparano, JA, Hsu, JJ, Wongchenko, M, Simmons, BP, McNally, VA; Miles, D	2016
Neurostimulation for abdominal pain-related functional gastrointestinal disorders in adolescents: a randomised, double-blind, sham-controlled trial	Kovacic, , K, Hainsworth, K, Sood, M, Chelimsky, G, Unteutsch, R, Nugent, M, Simpson, P; Miranda, A	2017
6-mercaptopurine or methotrexate added to prednisone induces and maintains remission in steroid-dependent inflammatory bowel disease	Maté-Jiménez, , J, Hermida, C, Cantero-Perona, J; Moreno-Otero, R	2000
Effect of butyrylcholinesterase genotype on the response to rivastigmine or donepezil in younger patients with Alzheimer's disease	Blesa, , R, Bullock, R, He, Y, Bergman, H, Gambina, G, Meyer, J, Rapatz, G, Nagel, J; Lane, R	2006
Association of baseline C-reactive protein levels with periprocedural myocardial injury in patients undergoing percutaneous bifurcation intervention: a CACTUS study subanalysis	Niccoli, , G, Sgueglia, GA, Latib, A, Crea, F; Colombo, A	2014
Effects of alpha-lipoic acid supplementation in peripheral arterial disease: a pilot study	Vincent, , HK, Bourguignon, CM, Vincent, KR; Taylor, AG	2007
Pmlasma neurofilament heavy chain levels correlate with disease severity and progression on multiple clinical outcomes in amyotrophic lateral sclerosis (ALS)	Han, , S, Liu, D, Ciotti, S, Ferguson, T, Ray, S; Johns, D	2017
L/N-type calcium channel blocker cilnidipine added to renin-angiotensin inhibition improves ambulatory blood pressure profile and suppresses cardiac hypertrophy in hypertension with chronic kidney disease	Kanaoka, , T, Tamura, K, Wakui, H, Ohsawa, M, Azushima, K, Uneda, K, Kobayashi, R, Fujikawa, T, Tsurumi-Ikeya, Y, Maeda, A, Yanagi, M, Toya, Y; Umemura, S	2013
Time course and dose response of alpha tocopherol on oxidative stress in haemodialysis patients	Reed, , A, Cho, YJ, Coombes, JS; Fassett, RG	2009
C-reactive protein, an early marker of community-acquired sepsis resolution: a multi-center prospective observational study	Povoa, , P, Teixeira-Pinto, AM; Carneiro, AH	2011

Results of a two-year followup study of patients with rheumatoid arthritis who received a combination of abatacept and methotrexate	Kremer, , JM, Genant, HK, Moreland, LW, Russell, AS, Emery, P, Abud-Mendoza, C, Szechiński, J, Li, T, Teng, J, Becker, JC; Westhovens, R	2008
The medical management of metastatic renal cell carcinoma: integrating new guidelines and recommendations	Bellmunt, , J; Guix, M	2009
Therapeutic angiogenesis for patients with limb ischaemia by autologous transplantation of bone-marrow cells: a pilot study and a randomised controlled trial	Tateishi-Yuyama, , E, Matsubara, H, Murohara, T, Ikeda, U, Shintani, S, Masaki, H, Amano, K, Kishimoto, Y, Yoshimoto, K, Akashi, H, Shimada, K, Iwasaka, T; Imaizumi, T	2002
Effects of telmisartan and losartan on cardiovascular protection in Japanese hypertensive patients	Hasegawa, , H, Takano, H, Narumi, H, Ohtsuka, M, Mizuguchi, T, Namiki, T, Kobayashi, Y; Komuro, I	2011
Individualizing Incentives for Alcohol in the Severely Mentally Ill	NCT03481049	2018
De-escalation of tyrosine kinase inhibitor dose in patients with chronic myeloid leukaemia with stable major molecular response (DESTINY): an interim analysis of a non-randomised, phase 2 trial	Clark, , RE, Polydoros, F, Apperley, JF, Milojkovic, D, Pocock, C, Smith, G, Byrne, JL, de Lavallade, H, O'Brien, SG, Coffey, T, Foroni, L; Copland, M	2017
Effect of Vitamin D3 Supplementation on Cardiometabolic Risk	NCT02359214	2015
Ulcerative Colitis Relapse Prevention by Prebiotics	NCT02865707	2016
C-reactive protein as a cardiovascular risk factor and its therapeutic implications in end-stage renal disease patients	Park, , JS; Kim, SB	2003
Randomized comparison of the effects of rosiglitazone vs. placebo on peak integrated cardiovascular performance, cardiac structure, and function	McGuire, , DK, Abdullah, SM, See, R, Snell, PG, McGavock, J, Szczepaniak, LS, Ayers, CR, Drazner, MH, Khera, A; de Lemos, JA	2010
Beneficial Effects of Quercetin in Chronic Obstructive Pulmonary Disease (COPD)	NCT01708278	2012
Comparison of clinical characteristics and treatment outcomes between eosinophilic and neutrophilic exacerbations of COPD requiring hospital admission	Kang, , HS, Jeon, SH, Rhee, CK, Lee, SH; Kim, YH	2016
A study to look at the efficacy and safety of ALN TTRSC in patients with an inherited condition that causes certain protein molecules to deposit in the heart	EUCTR2014-003835-20-ES	2015
Phase 3 Study of Nivolumab or Nivolumab plus Ipilimumab Versus Ipilimumab Alone in Previously Untreated Advanced Melanoma	EUCTR2012-005371-13-ES	2013
The Effects of Tai Chi on Markers of Atherosclerosis, Lower-limb Physical Function, and Cognitive Ability in Adults Aged Over 60: a Randomized Controlled Trial	Zhou, , S, Zhang, Y, Kong, Z, Loprinzi, PD, Hu, Y, Ye, J, Liu, S, Yu, JJ; Zou, L	2019
Assessment of blood concentration of an enzyme named Active Thrombin-Activatable Fibrinolysis Inhibitor (TAFIa) in Patients at acute stage of Stroke	EUCTR2017-002760-41-ES	2017
Alternate day fasting for obesity treatment	ISRCTN13374800	2017
Effect of Vitamin C on Stress in Peritoneal Dialysis Patients	IRCT2016050227675N2	2017
A Phase 3 Study of Guselkumab in Subjects with Active Psoriatic Arthritis	EUCTR2016-001163-37-PL	2017
Tai Chi for stress and cardiovascular function	ACTRN12616001204437	2016
Nexavar in Relapsed Peripheral T-cell Non-Hodgkins Lymphoma - NEXT	EUCTR2008-000306-36-SE	2008
A Multicenter, Randomized, Parallel-Groups, Double-Blind Placebo Controlled Study Comparing The Efficacy, Safety, And Tolerability Of Administration Of Ezetimibe/Simvastatin tablet 10/20 Mg Versus Doubling the Dose of Simvastatin 20 mg Simvastatin 40 mg In Subjects With Primary Hypercholesterolemia And Coronary Heart Disease - ND	EUCTR2005-004848-31-IT	2006
A study to primarily determine how safe and tolerable the study treatment, PTG-200 is in comparison to a matching placebo, when taken by healthy participants as a single dose or multiple dose treatment	ACTRN12617001426370	2017
Liraglutide Kidney: a study investigating the short-term impact of liraglutide on kidney function in diabetic patients	EUCTR2012-003577-26-DK	2012
PHASE III, RANDOMISED, DOUBLE BLIND, STRATIFIED COMPARATIVE, PLACEBO CONTROLLED, PARALLEL GROUP, MULTICENTRE STUDY TO ASSESS THE EFFECT OF DEEP SUBCUTANEOUS INJECTIONS OF LANREOTIDE AUTOGEL 120 MG ADMINISTERED EVERY 28 DAYS ON TUMOUR PROGRESSION FREE SURVIVAL IN PATIENTS WITH NON FUNCTIONING ENTERO-PANCREATIC ENDOCRINE TUMOUR - ND	EUCTR2005-004904-35-IT	2007
Electrical stimulation in cirrhotic in peripheral muscle strength and exercise capacity	RBR-3sst2s	2017
Evaluation of muscle strength, exercise capacity, heart rate and blood inflammatory of patients with chronic obstructive pulmonary disease submited to physical exercises	RBR-7kcr2p	2016
Effect of Quercetin supplementation on women with Polycystic ovary syndrome	IRCT2016082215536N4	2017
The role of mindfulness in promoting wellbeing in patients with Crohnâ€™s disease: an exploratory randomised control trial	ACTRN12618002009291	2018
Shen-Shuai-Ning granule decreased serum concentrations of indoxyl sulphate in uremic patients undergoing peritoneal dialysis	Chen, , X, Gao, S, Ruan, M, Chen, S, Xu, J, Xing, X, Pan, X, Mei, C; Mao, Z	2018

A Phase 3 Study of Guselkumab in Subjects with Active Psoriatic Arthritis	EUCTR2016-001224-63-CZ	2017
Prevention and treatment of intestinal bacterial translocation in patients with dycompensated cirrhosis by Tongfu method and its mechanism	ChiCTR1800018280	2018
The effect of Saffron supplement on active rheumatoid arthritis	IRCT201707139472N14	2017
A Phase 3 Study of Guselkumab in Subjects with Active Psoriatic Arthritis	EUCTR2016-001224-63-PL	2017
A Phase III, randomized, double-blind, placebo-controlled study to assess the efficacy and safety of LEBRIKIZUMAB in patients with uncontrolled asthma who are on inhaled corticosteroids and second controller medication	EUCTR2011-004205-24-ES	2012
Bioavailability study of Fentanyl Citrate	CTRI/2014/10/005091	2014
Efficacy and Safety Study of Guselkumab in the Treatment of Participants With Active Psoriatic Arthritis (PsA)	EUCTR2014-003697-17-RO	2015
Atorvastatin vs Colchicine in Decrease of Troponin I of High Sensitivity in Patients With Rheumatoid Arthritis	NCT04056039	2019
A trial to compare Z102 against Prednisone in patients with rheumatoid arthritis	EUCTR2011-002392-41-BG	2012
A clinical study of Pembrolizumab plus axitinib vs. sunitinib in advanced kidney cancer	EUCTR2016-000588-17-FR	2016
A Phase IIb Multicentric Controlled Study Evaluating the Therapeutic Vaccine TG4010 (MVA-MUC1-IL2) as an Adjunct to Standard Chemotherapy in Advanced Non Small Cell Lung Cancer	EUCTR2005-001015-22-HU	2005
dark chocolate effect on cardiovascular side effect of diabetes	IRCT2015022116123N5	2015
A Clinical Trial to Look at the Effects of the Drug Bempedoic Acid when adding it to Ezetimibe Therapy in Patients with High Cholesterol. Patients to be randomly allocated to either placebo or investigational drug; assignment will be unknown to patient and doctor	EUCTR2016-004084-39-HU	2017
A Dose-Ranging Study of the Effects of SCH 527123 in Subjects With Moderate to Severe COPD	EUCTR2008-003780-38-FR	2009
Use of standard enteral formula versus enteric formula with prebiotic content in nutrition therapy: a randomized controlled study among neuro-critical care patients	Tuncay, , P, Arpaci, F, Doganay, M, Erdem, D, Sahna, A, Ergun, H; Atabey, D	2018
A phase 2, multi-centre, randomised, double blind, placebo controlled study evaluating the efficacy, safety and pharmacokinetics of two doses of a candidate Disease Modifying Anti-Rheumatic Drug (DMARD), (SMP-114 120 mg and 240 mg once daily), administered in combination with ongoing methotrexate treatment in patients with active rheumatoid arthritis. - ASPECTS- Assessment of Safety, Pharmacokinetics and Efficacy in a Combination Treatment with SMP-114	EUCTR2005-003436-21-CZ	2005
Early statin therapy restores endothelial function in children with familial hypercholesterolemia	de Jongh, , S, Lilien, MR, op't Roodt, J, Stroes, ES, Bakker, HD; Kastelein, JJ	2002
Identification of specifically reduced Th2 cell subsets in allergic rhinitis patients after sublingual immunotherapy	Ihara, , F, Sakurai, D, Yonekura, S, Iinuma, T, Yagi, R, Sakurai, T, Ito, T, Matsuura, A, Morimoto, Y, Arai, T, Suzuki, S, Katayama, K, Nakayama, T; Okamoto, Y	2018
Effect of atorvastatin on apolipoprotein B48 metabolism and low-density lipoprotein receptor activity in normolipidemic patients with coronary artery disease	Dane-Stewart, , CA, Watts, GF, Pal, S, Chan, D, Thompson, P, Hung, J; Mamo, JC	2003
Determination of the efficacious and safe dose of ivabradine in paediatric patients with dilated cardiomyopathy and symptomatic chronic heart failure aged from 6 months to less than 18 years	EUCTR2011-001292-39-ES	2012
Fast Track to Health. The effect of a modified alternate day fasting dietary pattern on weight loss and well-being among young people above a healthy weight	ACTRN12617001630303	2017
Clinical randomized double-blind placebo-controlled study of Bifidobacterium Lactobacillus M8 in the treatment of ankylosing spondylitis	ChiCTR1800017111	2018
A Study Comparing SAIT101 to MabThera® or Rituxan® in Patients with Rheumatoid Arthritis (RA)	EUCTR2014-005368-13-CZ	2016
Comparing cultured limbal stem cell transplantation (CLET) with direct limbal lenticule transplantation (SLET) in eyes with total limbal stem cell deficiency due to ocular burns	ISRCTN12217540	2018
Oral treatment of allergy with a non-pathogenic microorganism: a randomised clinical trial	JPRN-UMIN000001070	2008
A study to check how safe, beneficial and tolerable the drug canakinumab is for patients with SJIA	EUCTR2008-005476-27-Outside-EU/EEA	2012
An Open-Label, Multicenter, Randomized, Phase Ib/II Study of E7080 in Combination with Carboplatin + Gemcitabine versus Carboplatin + Gemcitabine Alone as Second Line Therapy in Patients with Platinum-Sensitive Recurrent Ovarian Cancer by CA125	EUCTR2009-016050-41-PL	2010
A Phase III, randomized, double-blind, placebo-controlled study to assess the efficacy and safety of LEBRIKIZUMAB in patients with uncontrolled asthma who are on inhaled corticosteroids and second controller medication	EUCTR2011-004218-41-ES	2012
Itolizumab for Moderate-to-Severe Psoriasis-phase 3	RPCEC00000201	2015

Efficacy and Safety Study in Mild Asthmatic Subjects Undergoing Allergen Challenge	EUCTR2012-001729-28-DE	2012
A Phase 3 Study of Guselkumab in Subjects with Active Psoriatic Arthritis	EUCTR2016-001163-37-HU	2017
Randomized, multicenter cross-over study investigating the effect of bicarbonate-based solutions (Physioneal 35 vs. 40) on protein metabolism in children and adolescents on chronic peritoneal dialysis - Nephropaed-PD-01	EUCTR2005-005434-12-DE	2008
BAX 326 (recombinant Factor IX): a Phase 1/3 Prospective, Controlled, Multicenter Study Evaluating Pharmacokinetics, Efficacy, Safety, and Immunogenicity in Previously Treated Patients with Severe (FIX level < 1%) or Moderately Severe (FIX level = 2%) Hemophilia B - BAX 326 pivotal	EUCTR2009-016720-31-BG	2010
Ajuste del tratamiento con tirofibán en pacientes que presentan resistencia a la aspirina o al clopidogrel: 3T/2R Tailoring Treatment with Tirofiban in patients showing Resistance to aspirin and/or Resistance to clopidogrel: 3T/2R - 3T/2R	EUCTR2007-002427-32-ES	2008
A randomized, blinded study, using multiple increasing doses of RO5310074, studying the effects of RO5310074 in patients with psoriatic arthritis	ACTRN12610000694011	2010
Phase IIa study to evaluate the safety, blood concentration and effect on the body of repeated administrations of PRS-080#022-DP to patients with chronic kidney disease undergoing dialysis and suffering from decreased amount of red blood cells	EUCTR2016-004472-21-CZ	2017
A Randomized, Double-Blind, Multi-center, Phase II Fixed Dose Study of Multiple Doses of Ipilimumab (MDX-010) Monotherapy in Patients with Previously Treated Unresectable Stage III or IV Melanoma Revised Protocol 2, incorporating Amendments 2 and 7 - version 5.0, dated 28-Sep-07 + Protocol Amendment 01-Pharmacogenetics Blood Sample; + Protocol Amendment 03-Pharmacogenomics Tissue Sample; + Protocol Amendment 04-Pharmacogenomic Biomarker Sample. + Amendment 5 Country Specific - Germany - version 1.0, dated 04-Apr-06 + Amendment 6 Country Specific - Germany - version 1.0, dated 11-Aug-06	EUCTR2005-003265-16-DE	2006
A RANDOMIZED, CONTROLLED, DOUBLE-BLIND, PARALLEL-GROUP, PHASE 3 STUDY TO COMPARE THE PHARMACOKINETICS, EFFICACY AND SAFETY BETWEEN CT-P10, RITUXAN AND MABTHERA IN PATIENTS WITH RHEUMATOID ARTHRITIS	PER-022-14	2014
Efficacy and Safety of REGN727/SAR236553 in patients with primary hypercholesterolemia who are intolerant to statins	EUCTR2012-001221-27-GB	2013
No effects of probiotics on atopic dermatitis in infancy: a randomized placebo-controlled trial	Brouwer, , ML, Wolt-Plompen, SA, Dubois, AE, van der Heide, S, Jansen, DF, Hoijer, MA, Kauffman, HF; Duiverman, EJ	2006
Comparison of the effects of combination atorvastatin (40 mg) + ezetimibe (10 mg) versus atorvastatin (40 mg) alone on secretory phospholipase A2 activity in patients with stable coronary artery disease or coronary artery disease equivalent	Azar, , M, Valentin, E, Badaoui, G, Kassab, R, Sarkis, A; Azar, RR	2011
Design of FLAT-SUGAR: randomized trial of prandial insulin versus prandial glp-1 receptor agonist together with basal insulin and metformin for high-risk type 2 diabetes	Probstfield, JL	2015
Effect of metformin treatmenton intrafollicular cytokines, ovarian response to gonadotropin and in vitro fertilization outcome in patients with polycystic ovary syndrome	Kim, , C, Jeung, Y, Moon, J; Kang, B	2016
Final results of a phase 2 trial of extended treatment (TX) with Carfilzomib (CFZ), Lenalidomide (LEN), and Dexamethasone (KRd) plus autologous stem cell transplantation (ASCT) in newly diagnosed multiple myeloma (NDMM)	Zimmerman, , T, Raje, NS, Vij, R, Reece, D, Berdeja, JG, Stephens, LA, McDonnell, K, Rosenbaum, CA, Jasielec, J, Richardson, PG, Gurbuxani, S, Nam, J, Severson, E, Wolfe, BD, Rosebeck, S, Stefka, A, Dytfeld, D, Griffith, K; Jakubowiak, A	2016
Evaluation of low-dose rituximab for the retreatment of patients with active rheumatoid arthritis: a non-inferiority randomised controlled trial	Mariette, , X, Rouanet, S, Sibilia, J, Combe, B, Le Loët, X, Tebib, J, Jourdan, R; Dougados, M	2014
Improvements in the systolic and pulse pressure components of blood pressure, in arterial stiffness and in left ventricular hypertrophy in hypertensive patients treated with the perindopril/indapamide combination: the REASON study	Blacher, , J; Safar, M	2004
Clinical trial: preliminary efficacy and safety study of a new Budesonide-MMX® 9 mg extended-release tablets in patients with active left-sided ulcerative colitis	D'Haens, , GR, Kovács, A, Vergauwe, P, Nagy, F, Molnár, T, Bouhnik, Y, Weiss, W, Brunner, H, Lavergne-Slove, A, Binelli, D, Di Stefano, AF; Marteau, P	2010
Lowered platelet count as a prognostic factor in pregnancy induced hypertension - A prospective study	Kamatar, , SC, Rajesh, BP; Raju, VS	2015
Peritonitis outcomes in patients with HIV and end-stage renal failure on peritoneal dialysis: a prospective cohort study	Ndlovu, , KCZ, Sibanda, W; Assounga, A	2017
Comorbidities, alone and in combination with D-dimer, as risk factors for recurrence after a first episode of unprovoked venous thromboembolism in the extended follow-up of the PROLONG study	Cosmi, , B, Legnani, C, Tosetto, A, Pengo, V, Ghirarduzzi, A, Testa, S, Prisco, D, Poli, D, Tripodi, A; Palareti, G	2010
Efficacy and safety of infliximab in patients with ankylosing spondylitis: results of a randomized, placebo-controlled trial (ASSERT)	van der Heijde, , D, Dijkmans, B, Geusens, P, Sieper, J, DeWoody, K, Williamson, P; Braun, J	2005
Fulvestrant plus palbociclib versus fulvestrant plus placebo for treatment of hormone-receptor-positive, HER2-negative metastatic breast cancer that progressed on previous endocrine therapy (PALOMA-3): final	Cristofanilli, , M, Turner, NC, Bondarenko, I, Ro, J, Im, SA, Masuda, N, Colleoni, M, DeMichele, A, Loi, S, Verma, S, Iwata,	2016

analysis of the multicentre, double-blind, phase 3 randomised controlled trial	H, Harbeck, N, Zhang, K, Theall, KP, Jiang, Y, Bartlett, CH, Koehler, M; Slamon, D	
A comparative study of etanercept plus methotrexate and methotrexate alone in Taiwanese patients with active rheumatoid arthritis: a 12-week, double-blind, randomized, placebo-controlled study	Lan, , JL, Chou, SJ, Chen, DY, Chen, YH, Hsieh, TY; Young, M	2004
Trial Evaluating Chemoprophylaxis Against Travelers' Diarrhea - Prevent TD	NCT02498301	2015
Evidence-Based Review of Clinical Studies on Trauma		2009
Electrocardiographic remodeling in patients paced for heart failure	Vogt, , J, Krahnefeld, O, Lamp, B, Hansky, B, Kirkels, H, Minami, K, Korfer, R, Horstkotte, D, Kloss, M; Auricchio, A	2000
Pentoxifylline in Diabetic Kidney Disease	NCT03625648	2018
Volasertib Versus Chemotherapy in Platinum-Resistant or -Refractory Ovarian Cancer: a Randomized Phase II Groupe des Investigateurs Nationaux pour l'Etude des Cancers de l'Ovaire Study	Pujade-Lauraine, , E, Selle, F, Weber, B, Ray-Coquard, IL, Vergote, I, Sufliarsky, J, Del Campo, JM, Lortholary, A, Lesoin, A, Follana, P, Freyer, G, Pardo, B, Vidal, L, Tholander, B, Gladieff, L, Sassi, M, Garin-Chesa, P, Nazabadioko, S, Marzin, K, Pilz, K; Joly, F	2016
Efficacy and safety of coadministration of ezetimibe and simvastatin in African-American patients with primary hypercholesterolemia	Rodney, , RA, Sugimoto, D, Wagman, B, Zieve, F, Kerzner, B, Strony, J, Yang, B, Suresh, R; Veltri, E	2006
Nivolumab in Treating Patients With Localized Kidney Cancer Undergoing Nephrectomy	NCT03055013	2017
Functional changes in adipose tissue in a randomised controlled trial of physical activity	Sjögren, , P, Sierra-Johnson, J, Kallings, LV, Cederholm, T, Kolak, M, Halldin, M, Brismar, K, de Faire, U, Hellénius, ML; Fisher, RM	2012
Cytokine and clinical response to Saccharomyces boulardii therapy in diarrhea-dominant irritable bowel syndrome: a randomized trial	Abbas, , Z, Yakoob, J, Jafri, W, Ahmad, Z, Azam, Z, Usman, MW, Shamim, S; Islam, M	2014
Usefulness of sparfloxacin against Chlamydia pneumoniae infection in patients with bronchial asthma	Horiguchi, , T, Miyazaki, J, Ohira, D, Watanabe, Y, Hayashi, N, Kobayashi, K, Torigoe, T, Fukumoto, K, Ito, T, Hirose, M, Sasaki, Y, Shiga, M, Kondo, R, Handa, M; Tachikawa, S	2005
Effects of Nopales (Opuntia Spp.) on Lipoprotein Profile and Oxidative Stress in Moderately Hypercholesterolemic Adults	NCT02209493	2014
Inflammation and pro-resolution inflammation after hepatobiliary surgery	Cata, , JP, Velasquez, JF, Ramirez, MF, Vauthey, JN, Gottumukkala, V, Conrad, C, Kim, BJ; Aloia, T	2017
Sulfasalazine in ankylosing spondylitis: a prospective, randomized, double-blind placebo-controlled study and comparison with other controlled studies	Schmidt, , WA, Wierth, S, Milleck, D, Droste, U; Gromnica-Ihle, E	2002
Effects of oral alendronate on BMD in adult patients with osteogenesis imperfecta: a 3-year randomized placebo-controlled trial	Chevrel, , G, Schott, AM, Fontanges, E, Charrin, JE, Lina-Granade, G, Duboeuf, F, Garnero, P, Arlot, M, Raynal, C; Meunier, PJ	2006
Randomized double blind crossover trial of Aloe vera, Cnidoscolus chayamansa and placebo for reducing hyperglycemia in women with early metabolic syndrome	Cardenas-Ibarra, , L, Villarreal-Perez, JZ, Lira-Castillo, JC; Nava-Aleman, A	2017
Probiotic therapy for spondyloarthritis: a randomised controlled trial	Jenks, , K, Stebbings, S, Burton, J, Schultz, M, Herbison, P; Highton, J	2010
Rosiglitazone Evaluated for Cardiac Outcomes and Regulation of Glycaemia in Diabetes (RECORD): study design and protocol	Home, , PD, Pocock, SJ, Beck-Nielsen, H, Gomis, R, Hanefeld, M, Dargie, H, Komajda, M, Gubb, J, Biswas, N; Jones, NP	2005
Development of a highly sensitive and specific method for detection of circulating tumor cells harboring somatic mutations in non-small-cell lung cancer patients	Breitenbuecher, , F, Hoffarth, S, Worm, K, Cortes-Incio, D, Gauler, TC, Köhler, J, Herold, T, Schmid, KW, Freitag, L, Kasper, S; Schuler, M	2014
A placebo-controlled pilot study of intensification of antiretroviral therapy with mycophenolate mofetil	Kaur, , R, Bedimo, R, Kvanli, MB, Turner, D, Shaw, L; Margolis, D	2006
Effect of exercise training on estimated GFR, vascular health, and cardiorespiratory fitness in patients with CKD: a pilot randomized controlled trial	Greenwood, , SA, Koufaki, P, Mercer, TH, MacLaughlin, HL, Rush, R, Lindup, H, O'Connor, E, Jones, C, Hendry, BM, Macdougall, IC; Cairns, HS	2015
Propranolol inhibits stress-related gene expression profiles associated with adverse clinical outcomes in autologous hematopoietic cell transplantation recipients	Knight, , J, Rizzo, JD, Hari, P, Kerswill, S, D'Souza, A, Logan, BR, Hamadani, M, Chhabra, S, Dhakal, B, Shah, N; Cole, S	2018
The effect of a high-egg diet on cardiovascular risk factors in people with type 2 diabetes: the Diabetes and Egg (DIABEGG) study-a 3-mo randomized controlled trial	Fuller, , NR, Caterson, ID, Sainsbury, A, Denyer, G, Fong, M, Gerofi, J, Baqleh, K, Williams, KH, Lau, NS; Markovic, TP	2015
Mortality in dialysis patients may not be associated with ESA dose: a 2-year prospective observational study	McMahon, , LP, Cai, MX, Baweja, S, Holt, SG, Kent, AB, Perkovic, V, Leikis, MJ; Becker, GJ	2012
The use of a Cissus quadrangularis formulation in the management of weight loss and metabolic syndrome	Oben, , J, Kuate, D, Agbor, G, Momo, C; Talla, X	2006
Autologous tumor lysate-pulsed dendritic cell immunotherapy with cytokine-induced killer cells improves survival in gastric and colorectal cancer patients	Gao, , D, Li, C, Xie, X, Zhao, P, Wei, X, Sun, W, Liu, HC, Alexandrou, AT, Jones, J, Zhao, R; Li, JJ	2014
Extensive peritoneal lavage decreases postoperative C-reactive protein concentrations: a RCT	De Cicco, , C, Schonman, R, Ussia, A; Koninckx, PR	2015
High-volume online haemodiafiltration improves erythropoiesis-stimulating agent (ESA) resistance in comparison with low-flux bicarbonate dialysis: results of the REDERT study	Panichi, , V, Scatena, A, Rosati, A, Giusti, R, Ferro, G, Malagnino, E, Capitanini, A, Piluso, A, Conti, P, Bernabini, G, Migliori, M, Caiani, D, Tetta, C, Casani, A, Betti, G; Pizzarelli, F	2015
The effect of neutral peritoneal dialysis solution with low glucose-degradation-product on the fluid status and body composition - A randomized control trial	Szeto, , C-C, Kwan, BCH, Chow, K-M, Cheng, PMS, Kwong, VWK, Choy, ASM, Law, M-C, Leung, C-B; Li, PKT	2015
Simvastatin effects on androgens, inflammatory mediators, and endogenous pituitary gonadotropins among patients with PCOS	Rashidi, , B, Abediasl, J, Tehraninejad, E, Rahmanpour, H; Sills, ES	2011

undergoing IVF: results from a prospective, randomized, placebo-controlled clinical trial		
A Randomized Controlled Trial of Atorvastatin in Patients With Bronchiectasis Infected With Pseudomonas Aeruginosa: a Proof of Concept Study	Bedi, , P, Chalmers, JD, Graham, C, Clarke, A, Donaldson, S, Doherty, C, Govan, JRW, Davidson, DJ, Rossi, AG; Hill, AT	2017
Improved estimation of glomerular filtration rate by serum cystatin C in preventing contrast induced nephropathy by N-acetylcysteine or zinc–preliminary results	Kimmel, , M, Butscheid, M, Brenner, S, Kuhlmann, U, Klotz, U; Alscher, DM	2008
The effects of rosiglitazone, metformin, and estradiol-cyproterone acetate on lean patients with polycystic ovary syndrome	Cetinkalp, , S, Karadeniz, M, Erdogan, M, Ozgen, G, Saygl, F; Ylmaz, C	2009
Golimumab in patients with active rheumatoid arthritis despite methotrexate therapy: results through 2 years of the GO-FORWARD study extension	Keystone, , EC, Genovese, MC, Hall, S, Miranda, PC, Bae, SC, Palmer, W, Wu, Z, Xu, S; Hsia, EC	2013
Circulating histones for predicting prognosis after cardiac surgery: a prospective study	Gao, , H, Zhang, N, Lu, F, Yu, X, Zhu, L, Mo, X; Wang, W	2016
Long-term safety and feasibility of three-vessel multimodality intravascular imaging in patients with ST-elevation myocardial infarction: the IBIS-4 (integrated biomarker and imaging study) substudy	Taniwaki, , M, Radu, MD, Garcia-Garcia, HM, Heg, D, Kelbaek, H, Holmvang, L, Moschovitis, A, Noble, S, Pedrazzini, G, Saunamaki, K, Dijkstra, J, Landmesser, U, Wenaweser, P, Meier, B, Stefanini, GG, Roffi, M, Luscher, TF, Windecker, S; Raber, L	2015
Safety, efficacy, and pharmacokinetics of intravenous rigosertib in Japanese patients with recurrent/relapsed or refractory myelodysplastic syndromes: a multicenter, open-label, phase i study	Ogura, , M, Kobayashi, Y, Kubonishi, S, Hidaka, M, Uchida, T; Takamatsu, Y	2017
Comparison of early enteral nutrition in severe acute pancreatitis with prebiotic fiber supplementation versus standard enteral solution: a prospective randomized double-blind study	Karakan, , T, Ergun, M, Dogan, I, Cindoruk, M; Unal, S	2007
Flaxseed consumption reduces blood pressure in patients with hypertension by altering circulating oxylipins via an α-linolenic acid-induced inhibition of soluble epoxide hydrolase	Caligiuri, , SP, Aukema, HM, Ravandi, A, Guzman, R, Dibrov, E; Pierce, GN	2014
Randomized trial of atorvastatin for reduction of postoperative atrial fibrillation in patients undergoing cardiac surgery: results of the ARMYDA-3 (Atorvastatin for Reduction of MYocardial Dysrhythmia After cardiac surgery) study	Patti, , G, Chello, M, Candura, D, Pasceri, V, D'Ambrosio, A, Covino, E; Di Sciascio, G	2006
Randomized phase II trial of erlotinib or standard chemotherapy in patients with advanced non-small-cell lung cancer and a performance status of 2	Lilenbaum, , R, Axelrod, R, Thomas, S, Dowlati, A, Seigel, L, Albert, D, Witt, K; Botkin, D	2008
High-Dose Versus Low-Dose Pitavastatin in Japanese Patients With Stable Coronary Artery Disease (REAL-CAD): a Randomized Superiority Trial	Taguchi, , I, Iimuro, S, Iwata, H, Takashima, H, Abe, M, Amiya, E, Ogawa, T, Ozaki, Y, Sakuma, I, Nakagawa, Y, Hibi, K, Hiro, T, Fukumoto, Y, Hokimoto, S, Miyauchi, K, Yamazaki, T, Ito, H, Otsuji, Y, Kimura, K, Takahashi, J, Hirayama, A, Yokoi, H, Kitagawa, K, Urabe, T, Okada, Y, Terayama, Y, Toyoda, K, Nagao, T, Matsumoto, M, Ohashi, Y, Kaneko, T, Fujita, R, Ohtsu, H, Ogawa, H, Daida, H, Shimokawa, H, Saito, Y, Kimura, T, Inoue, T, Matsuzaki, M; Nagai, R	2018
Addition of Liraglutide to Insulin in Patients With Type 1 Diabetes: a Randomized Placebo-Controlled Clinical Trial of 12 Weeks	Kuhadiya, , ND, Dhindsa, S, Ghanim, H, Mehta, A, Makdissi, A, Batra, M, Sandhu, S, Hejna, J, Green, K, Bellini, N, Yang, M, Chaudhuri, A; Dandona, P	2016
Associations between C-reactive protein levels and cognition during the first 6 months after acute psychosis	Fathian, , F, Løberg, EM, Gjestad, R, Steen, VM, Kroken, RA, Jørgensen, HA; Johnsen, E	2019
Probiotics Ameliorate Stool Consistency in Patients with Chronic Constipation: a Randomized, Double-Blind, Placebo-Controlled Study	Yoon, , JY, Cha, JM, Oh, JK, Tan, PL, Kim, SH, Kwak, MS, Jeon, JW; Shin, HP	2018
ATTIRE: albumin To prevenT Infection in chronic liveR failurE: study protocol for a single-arm feasibility trial	China, , L, Muirhead, N, Skene, SS, Shabir, Z, De Maeyer, RP, Maini, A, Gilroy, DW; O'Brien, AJ	2016
A new paradigm between mechanical scaling and root planing combined with adjunctive chemotherapy for glycated hemoglobin improvement in diabetics	Al Mubarak, , S, Abou Rass, M, Alsuwyed, A, Al-Zoman, K, Al Sohail, A, Sobki, S, Tariq, M, Alwin Robert, A, Ciancio, S; Dandona, P	2010
Factor VIII-mimetic function of humanized bispecific antibody in hemophilia A	Shima, , M, Hanabusa, H, Taki, M, Matsushita, T, Sato, T, Fukutake, K, Fukazawa, N, Yoneyama, K, Yoshida, H; Nogami, K	2016
The neutrophil/lymphocyte ratio (N/L) is a prognostic marker in patients with diffuse large b cell lymphoma: a prospective study from the lazio lymphoma registry	Annibali, , O, Hohaus, S, Tomarchio, V, Borza, PA, Cantonetti, M, Battistini, R, Montanaro, M, Angelis, FD, Cimino, G, Palombi, F, Liberati, AM, Abruzzese, E, Tesei, C, Tisi, MC, Alma, E, Rizzuto, G, Pupo, L, Zoli, V, Maiolo, E, Ansuinelli, M, Naso, V, Bianchi, MP; Cox, MC	2016
Micronutrient supplemented probiotic yogurt for HIV-infected adults taking HAART in London, Canada	Hemsworth, , JC, Hekmat, S; Reid, G	2012
Interleukin-1beta inhibition and the prevention of recurrent cardiovascular events: rationale and Design of the Canakinumab Anti-inflammatory Thrombosis Outcomes Study (CANTOS)	Ridker, , PM, Thuren, T, Zalewski, A; Libby, P	2011
Dietary Flaxseed Reduces Central Aortic Blood Pressure Without Cardiac Involvement but Through Changes in Plasma Oxylipins	Caligiuri, , SP, Rodriguez-Leyva, D, Aukema, HM, Ravandi, A, Weighell, W, Guzman, R; Pierce, GN	2016
Duration of remission after halving of the etanercept dose in patients with ankylosing spondylitis: a randomized, prospective, long-term, follow-up study	Cantini, , F, Niccoli, L, Cassara, E, Kaloudi, O; Nannini, C	2013
Efficacy of a meal replacement diet plan compared to a food-based diet plan after a period of weight loss and weight maintenance: a randomized controlled trial	Davis, , LM, Coleman, C, Kiel, J, Rampolla, J, Hutchisen, T, Ford, L, Andersen, WS; Hanlon-Mitola, A	2010
Active anti-amyloid immunotherapy with UB-311 vaccine: design, baseline data and study update of a phase IIa, randomized, doubleblind, placebo-controlled, 3-arm parallelgroup, multicenter study	Verma, , A, Yu, HJ, Chen, H-C; Wang, CY	2018

Impact of an 8-Month Trial Using Height-Adjustable Desks on Children's Classroom Sitting Patterns and Markers of Cardio-Metabolic and Musculoskeletal Health	Contardo Ayala, , AM, Salmon, J, Timperio, A, Sudholz, B, Ridgers, ND, Sethi, P; Dunstan, DW	2016
Influence of perindopril, rosuvastatin, or omega-3 polyunsaturated fatty acids on efficacy of antirecurrence therapy with sotalol in patients with persistent atrial fibrillation	Kanorskiĭ, , SG, Bodrikova, VV; Kanorskaia, IuS	2007
Randomized Phase II Trial of Seribantumab in Combination With Paclitaxel in Patients With Advanced Platinum-Resistant or -Refractory Ovarian Cancer	Liu, , JF, Ray-Coquard, I, Selle, F, Poveda, AM, Cibula, D, Hirte, H, Hilpert, F, Raspagliesi, F, Gladieff, L, Harter, P, Siena, S, Del Campo, JM, Tabah-Fisch, I, Pearlberg, J, Moyo, V, Riahi, K, Nering, R, Kubasek, W, Adiwijaya, B, Czibere, A, Naumann, RW, Coleman, RL, Vergote, I, MacBeath, G; Pujade-Lauraine, E	2016
Adalimumab improves sleep and sleep quality in patients with active ankylosing spondylitis	Rudwaleit, , M, Gooch, K, Michel, B, Herold, M, Thorner, A, Wong, R, Kron, M, Chen, N; Kupper, H	2011
C-reactive protein level and the incidence of eligibility for statin therapy: the multi-ethnic study of atherosclerosis	Mann, , DM, Shimbo, D, Cushman, M, Lakoski, S, Greenland, P, Blumenthal, RS, Michos, ED, Lloyd-Jones, DM; Muntner, P	2013
Clinical and Functional Outcomes Associated With Myocardial Injury After Transfemoral and Transapical Transcatheter Aortic Valve Replacement: a Subanalysis From the PARTNER Trial (Placement of Aortic Transcatheter Valves)	Paradis, , JM, Maniar, HS, Lasala, JM, Kodali, S, Williams, M, Lindman, BR, Damiano, RJ, Moon, MR, Makkar, RR, Thourani, VH, Babaliaros, V, Xu, K, Ayele, GM, Svensson, L, Leon, MB; Zajarias, A	2015
BEAC or BEAM for high-dose therapy in patients with non-Hodgkin's lymphoma? A single centre analysis on toxicity and efficacy	Jantunen, , E, Kuittinen, T; Nousiainen, T	2003
Worsening Renal Function in Patients With Acute Heart Failure Undergoing Aggressive Diuresis Is Not Associated With Tubular Injury	Ahmad, , T, Jackson, K, Rao, VS, Tang, WHW, Brisco-Bacik, MA, Chen, HH, Felker, GM, Hernandez, AF, O'Connor, CM, Sabbisetti, VS, Bonventre, JV, Wilson, FP, Coca, SG; Testani, JM	2018
The Effects of a Low-Carbohydrate Diet vs. a Low-Fat Diet on Novel Cardiovascular Risk Factors: a Randomized Controlled Trial	Hu, , T, Yao, L, Reynolds, K, Whelton, PK, Niu, T, Li, S, He, J; Bazzano, LA	2015
Prevalence and incidence of clinically diagnosed knee, hip and hand osteoarthritis in women with polycystic ovary syndrome: a national register-based study	Sanchez-Santos, , MT, Rubin, KH, Glintborg, D, Andersen, MS, Kluzek, S; Abrahamsen, B	2018
The HYPERFlax trial for determining the anti-HYPERtensive effects of dietary flaxseed in newly diagnosed stage 1 hypertensive patients: study protocol for a randomized, double-blinded, controlled clinical trial	Caligiuri, , SP, Penner, B; Pierce, GN	2014
Secukinumab and Sustained Improvement in Signs and Symptoms of Patients With Active Ankylosing Spondylitis Through Two Years: results From a Phase III Study	Marzo-Ortega, , H, Sieper, J, Kivitz, A, Blanco, R, Cohen, M, Martin, R, Readie, A, Richards, HB; Porter, B	2017
Acute coronary syndromes and diabetes: is intensive lipid lowering beneficial? Results of the PROVE IT-TIMI 22 trial	Ahmed, , S, Cannon, CP, Murphy, SA; Braunwald, E	2006
RELAY+, an Exploratory Study of Gefitinib with Ramucirumab in Untreated Patients with EGFR Mutation-Positive Metastatic NSCLC	Nishio, , M, Nishio, K, Suzukawa, K, Omori, Y, Enatsu, S, Visseren-Grul, C; Nakagawa, K	2018
Effectiveness of disease-specific cognitive-behavioural therapy on depression, anxiety, quality of life and the clinical course of disease in adolescents with inflammatory bowel disease: study protocol of a multicentre randomised controlled trial (HAPPY-IBD)	van den Brink, , G, Stapersma, L, Marroun, HE, Henrichs, J, Szigethy, EM, Utens, EM; Escher, JC	2016
Similar Impact of Clopidogrel or Ticagrelor on Carotid Atherosclerotic Plaque Inflammation	Oh, , M, Lee, CW, Lee, HS, Chang, M, Ahn, J-M, Park, D-W, Kang, S-J, Lee, S-W, Kim, Y-H, Moon, DH, Park, S-W; Park, S-J	2016
Omega-3 fatty acids are protective against paclitaxel-induced peripheral neuropathy: a randomized double-blind placebo controlled trial	Ghoreishi, , Z, Esfahani, A, Djazayeri, A, Djalali, M, Golestan, B, Ayromlou, H, Hashemzade, S, Asghari Jafarabadi, M, Montazeri, V, Keshavarz, SA; Darabi, M	2012
Long-term erythromycin therapy is associated with decreased chronic obstructive pulmonary disease exacerbations	Seemungal, , TA, Wilkinson, TM, Hurst, JR, Perera, WR, Sapsford, RJ; Wedzicha, JA	2008
Epicardial fat thickness regression with continuous positive airway pressure therapy in patients with obstructive sleep apnea: assessment by two-dimensional echocardiography	Çetin, , S, Vural, MG, Gündüz, H, Akdemir, R; Fırat, H	2016
Use of rosuvastatin in HIV-associated chronic obstructive pulmonary disease	Morris, , A, Fitzpatrick, M, Bertolet, M, Qin, S, Kingsley, L, Leo, N, Kessinger, C, Michael, H, Mcmahon, D, Weinman, R, Stone, S, Leader, JK, Kleerup, E, Huang, L; Wisniewski, SR	2017
Safety, pharmacokinetics and pharmacodynamics of the oral toll-like receptor 7 agonist GS-9620 in treatment-naive patients with chronic hepatitis C	Lawitz, , E, Gruener, D, Marbury, T, Hill, J, Webster, L, Hassman, D, Nguyen, AH, Pflanz, S, Mogalian, E, Gaggar, A, Massetto, B, Subramanian, GM, McHutchison, JG, Jacobson, IM, Freilich, B; Rodriguez-Torres, M	2015
Study on the Optimal Strategy for Acute-on-chronic Liver Failure With Integrative Treatment	NCT03577938	2018
Korean traditional Chungkookjang improves body composition, lipid profiles and atherogenic indices in overweight/obese subjects: a double-blind, randomized, crossover, placebo-controlled clinical trial	Byun, , MS, Yu, OK, Cha, YS; Park, TS	2016
Antioxidative capacity of a dietary supplement on retinal hemodynamic function in a human lipopolysaccharide (LPS) model	Told, , R, Schmidl, D, Palkovits, S, Boltz, A, Gouya, G, Wolzt, M, Witkowska, KJ, Popa-Cherecheanu, A, Werkmeister, RM, Garhöfer, G; Schmetterer, L	2014
A randomised controlled trial evaluating the impact of targeted vitamin D supplementation on endothelial function in type 2 diabetes mellitus: the DIMENSION trial	Dalan, , R, Liew, H, Assam, PN, Chan, ES, Siddiqui, FJ, Tan, AW, Chew, DE, Boehm, BO; Leow, MK	2016
Impact of a community health workers-led structured program on blood glucose control among latinos with type 2 diabetes: the DIALBEST trial	Pérez-Escamilla, , R, Damio, G, Chhabra, J, Fernandez, ML, Segura-Pérez, S, Vega-López, S, Kollannor-Samuel, G, Calle, M, Shebl, FM; D'Agostino, D	2015
Activity of a triamcinolone acetonide/laurocapram formulation: double-blind comparisons with triamcinolone acetonide, placebo vehicle, and mid-strength (Class IV) to potent (Class II) corticosteroids	Cato, , A, Sutton, L, Kaplan, AS; Manning III, GN	2001

Catumaxomab with and without prednisolone premedication for the treatment of malignant ascites due to epithelial cancer: results of the randomised phase IIIb CASIMAS study	Sehouli, , J, Pietzner, K, Wimberger, P, Vergote, I, Rosenberg, P, Schneeweiss, A, Bokemeyer, C, Salat, C, Scambia, G, Berton-Rigaud, D, Santoro, A, Cervantes, A, Trédan, O, Tournigand, C, Colombo, N, Dudnichenko, AS, Westermann, A, Friccius-Quecke, H; Lordick, F	2014
Effects of a 5-lipoxygenase-activating protein inhibitor on biomarkers associated with risk of myocardial infarction: a randomized trial	Hakonarson, , H, Thorvaldsson, S, Helgadottir, A, Gudbjartsson, D, Zink, F, Andresdottir, M, Manolescu, A, Arnar, DO, Andersen, K, Sigurdsson, A, Thorgeirsson, G, Jonsson, A, Agnarsson, U, Bjornsdottir, H, Gottskalksson, G, Einarsson, A, Gudmundsdottir, H, Adalsteinsdottir, AE, Gudmundsson, K, Kristjansson, K, Hardarson, T, Kristinsson, A, Topol, EJ, Gulcher, J, Kong, A, Gurney, M, Thorgeirsson, G; Stefansson, K	2005
Whey protein lowers blood pressure and improves endothelial function and lipid biomarkers in adults with prehypertension and mild hypertension: results from the chronic Whey2Go randomized controlled trial	Fekete, , ÁA, Giromini, C, Chatzidiakou, Y, Givens, DI; Lovegrove, JA	2016
Relative efficacy of atorvastatin 80 mg and pravastatin 40 mg in achieving the dual goals of low-density lipoprotein cholesterol <70 mg/dl and C-reactive protein <2 mg/l: an analysis of the PROVE-IT TIMI-22 trial	Ridker, , PM, Morrow, DA, Rose, LM, Rifai, N, Cannon, CP; Braunwald, E	2005
Human herpesvirus 8 (KSHV) contamination of peripheral blood and autograft products from multiple myeloma patients	Vescio, , RA, Wu, CH, Zheng, L, Sheen, D, Ma, H, Liu, J, Stewart, AK, Ballester, O, Noga, SJ, Rugo, H, Freytes, C, Stadtmauer, E, Sahebi, F, Tarantolo, S, Stiff, P, Schiller, GJ, White, M, Jacobs, C, DiPersio, J, Anderson, KC; Berenson, JR	2000
Beneficial effect of refined red palm oil on lipid peroxidation and monocyte tissue factor in HCV-related liver disease: a randomized controlled study	Catanzaro, , R, Zerbinati, N, Solimene, U, Marcellino, M, Mohania, D, Italia, A, Ayala, A; Marotta, F	2016
Endocannabinoid blockade for improving glycemic control and lipids in patients with type 2 diabetes mellitus	Hollander, P	2007
Determining the identity and functional specialization of peritoneal mononuclear phagocytes in peritoneal dialysis patients	Liao, , C-T, Fraser, DJ, Topley, N; Taylor, PR	2015
n-3 polyunsaturated fatty acids and adiponectin in patients with end-stage renal disease	Harving, , F, Svensson, M, Flyvbjerg, A, Schmidt, EB, Jørgensen, KA, Eriksen, HH; Christensen, JH	2015
Lack of efficacy of an immunomodulatory macrolide in childhood HIV related bronchiectasis: a randomised, placebo-controlled trial	Masekela, , R, Anderson, R, Gongxeka, H, Steel, HC, Becker, PJ; Green, RJ	2013
Effects of Exercise by Neuromuscular Stimulation in Dialysis Patients	NCT03517553	2018
Subcutaneous Injection of Triamcinolone and Lidocaine to Prevent Postherpetic Neuralgia	Ni, , J, Wang, X, Tang, Y, Yang, L, Zeng, Y; Guo, Y	2017
Short-term high-dose atorvastatin for periprocedural myocardial infarction prevention in patients with renal dysfunction	Toso, , A, Leoncini, M, Maioli, M, Gallopin, M, Tedeschi, D, Amato, M; Bellandi, F	2011
Topical resiquimod 0.01% gel decreases herpes simplex virus type 2 genital shedding: a randomized, controlled trial	Mark, , KE, Corey, L, Meng, TC, Magaret, AS, Huang, ML, Selke, S, Slade, HB, Tyring, SK, Warren, T, Sacks, SL, Leone, P, Bergland, VA; Wald, A	2007
The effects of a whole grain-enriched hypocaloric diet on cardiovascular disease risk factors in men and women with metabolic syndrome	Katcher, , HI, Legro, RS, Kunselman, AR, Gillies, PJ, Demers, LM, Bagshaw, DM; Kris-Etherton, PM	2008
Application of urinary proteomics as possible risk predictor of renal and cardiovascular complications in patients with type 2-diabetes and microalbuminuria	Oellgaard, , J, Gaede, P, Persson, F, Rossing, P, Parving, H-H; Pedersen, O	2018
Triterpenoids and polysaccharide peptides-enriched Ganoderma lucidum: a randomized, double-blind placebo-controlled crossover study of its antioxidation and hepatoprotective efficacy in healthy volunteers	Chiu, , HF, Fu, HY, Lu, YY, Han, YC, Shen, YC, Venkatakrishnan, K, Golovinskaia, O; Wang, CK	2017
Aerobic exercise improves gastrointestinal motility in psychiatric inpatients	Kim, , YS, Song, BK, Oh, JS; Woo, SS	2014
Efficacy and safety of mavrilimumab in subjects with rheumatoid arthritis	Burmester, , GR, Weinblatt, ME, McInnes, IB, Porter, D, Barbarash, O, Vatutin, M, Szombati, I, Esfandiari, E, Sleeman, MA, Kane, CD, Cavet, G, Wang, B, Godwood, A; Magrini, F	2013
The effects of vagus nerve stimulation on tryptophan metabolites in children with intractable epilepsy	Klinkenberg, , S, van den Borne, CJH, Aalbers, MW, Verschuure, P, Kessels, AG, Leenen, L, Rijkers, K, Aldenkamp, AP, Vles, JSH; Majoie, HJM	2014
A long-term, open-label trial of the safety and efficacy of etanercept (Enbrel) in patients with rheumatoid arthritis not treated with other disease-modifying antirheumatic drugs	Klareskog, , L, Gaubitz, M, Rodriguez-Valverde, V, Malaise, M, Dougados, M; Wajdula, J	2006
Effectiveness of dapagliflozin on vascular endothelial function and glycemic control in patients with early-stage type 2 diabetes mellitus: DEFENCE study	Shigiyama, , F, Kumashiro, N, Miyagi, M, Ikehara, K, Kanda, E, Uchino, H; Hirose, T	2017
Twenty years of systemic therapy for breast cancer	Mina, , L; Sledge Jr, GW	2006
Neurodevelopment of Preterm Infants at 24 Months After Neonatal Supplementation of a Prebiotic Mix: a Randomized Trial	van den Berg, , JP, Westerbeek, EA, Bröring-Starre, T, Garssen, J; van Elburg, RM	2016
Genetic Counselling in the Prevention of Mental Health Consequences of Cannabis Use	NCT03601026	2018
A phase III, double-blind, randomized trial of palonosetron compared with ondansetron in preventing chemotherapy-induced nausea and vomiting following highly emetogenic chemotherapy	Aapro, , MS, Grunberg, SM, Manikhas, GM, Olivares, G, Suarez, T, Tjulandin, SA, Bertoli, LF, Yunus, F, Morrica, B, Lordick, F; Macciocchi, A	2006
Short- and long-term efficacy of intra-articular injections with betamethasone as part of a treat-to-target strategy in early rheumatoid arthritis: impact of joint area, repeated injections, MRI findings, anti-CCP, IgM-RF and CRP	Hetland, , ML, Østergaard, M, Ejbjerg, B, Jacobsen, S, Stengaard-Pedersen, K, Junker, P, Lottenburger, T, Hansen, I, Andersen, LS, Tarp, U, Svendsen, A, Pedersen, JK, Skjødt, H, Ellingsen, T, Lindegaard, H, Pødenphant, J; Hørslev-Petersen, K	2012

Rationale and Design of the ARREST Trial Investigating Mesenchymal Stem Cells in the Treatment of Small Abdominal Aortic Aneurysm	Wang, , SK, Green, LA, Gutwein, AR, Drucker, NA, Motaganahalli, RL, Fajardo, A, Babbey, CM; Murphy, MP	2018
Effects of a 14-month low-cost maintenance training program in patients with chronic systolic heart failure: a randomized study	Prescott, , E, Hjardem-Hansen, R, Dela, F, Ørkild, B, Teisner, AS; Nielsen, H	2009
Introduction of a prognostic biomarker to strengthen risk stratification of acutely admitted patients: rationale and design of the TRIAGE III cluster randomized interventional trial	Sandø, , A, Schultz, M, Eugen-Olsen, J, Rasmussen, LS, Køber, L, Kjøller, E, Jensen, BN, Ravn, L, Lange, T; Iversen, K	2016
Soluble CD40 ligand in acute and chronic heart failure	Ueland, , T, Aukrust, P, Yndestad, A, Otterdal, K, Frøland, SS, Dickstein, K, Kjekshus, J, Gullestad, L; Damås, JK	2005
A randomized, placebo-controlled trial of pentoxifylline on erythropoiesis-stimulating agent hyporesponsiveness in anemic patients with CKD: the Handling Erythropoietin Resistance With Oxpentifylline (HERO) trial	Johnson, , DW, Pascoe, EM, Badve, SV, Dalziel, K, Cass, A, Clarke, P, Ferrari, P, McDonald, SP, Morrish, AT, Pedagogos, E, Perkovic, V, Reidlinger, D, Scaria, A, Walker, R, Vergara, LA; Hawley, CM	2015
Effect of folic acid and betaine supplementation on flow-mediated dilation: a randomized, controlled study in healthy volunteers	Olthof, , MR, Bots, ML, Katan, MB; Verhoef, P	2006
Host and microbiological factors related to dental caries development	De Soet, , JJ, van Gemert-Schriks, MC, Laine, ML, van Amerongen, WE, Morré, SA; van Winkelhoff, AJ	2008
Sulphasalazine in ankylosing spondylitis: a prospective, randomized, double-blind, placebo-analysis of other controlled studies	Schmidt, , WA, Wierth, S, Milleck, D, Droste, U; Gromnica-Ihle, E	2002
Infliximab reduces the frequency of interleukin 17-producing cells and the amounts of interleukin 17 in patients with rheumatoid arthritis	Shen, , H, Xia, L, Lu, J; Xiao, W	2010
Effect of a high-fiber diet vs a fiber-supplemented diet on C-reactive protein level	King, , DE, Egan, BM, Woolson, RF, Mainous, AG, Al-Solaiman, Y; Jesri, A	2007
Taurine supplementation has anti-atherogenic and anti-inflammatory effects before and after incremental exercise in heart failure	Ahmadian, , M, Roshan, VD, Aslani, E; Stannard, SR	2017
A Placebo-Controlled Double-Blind Study Demonstrates the Clinical Efficacy of a Novel Herbal Formulation for Relieving Joint Discomfort in Human Subjects with Osteoarthritis of Knee	Karlapudi, , V, Prasad Mungara, AVV, Sengupta, K, Davis, BA; Raychaudhuri, SP	2018
Postprandial effects of long-term niacin/laropiprant use on glucose and lipid metabolism and on cardiovascular risk in patients with polycystic ovary syndrome	Aye, , MM, Kilpatrick, ES, Afolabi, P, Wootton, SA, Rigby, AS, Coady, AM, Sandeman, DD; Atkin, SL	2014
Myocardial oxidative stress, and cell injury comparing three different techniques for coronary artery bypass grafting	van Boven, , WJ, Gerritsen, WB, Driessen, AH, Morshuis, WJ, Waanders, FG, Haas, FJ, van Dongen, EP; Aarts, LP	2008
Etrolizumab induction therapy improved endoscopic score, patient-reported outcomes, and inflammatory biomarkers in patients with moderate to severe UC who had failed TNF antagonist therapy: results from the hickory open-label induction (OLI) trial	Peyrin-Biroulet, , L, Rubin, DT, Feagan, BG, Oh, YS, Arulmani, U, Tyrrell, H, Maciuca, R, Williams, S, Tole, S; Thommes, J	2017
Standardized parenteral alanyl-glutamine dipeptide supplementation is not beneficial in autologous transplant patients: a randomized, double-blind, placebo controlled study	Pytlík, , R, Benes, P, Patorková, M, Chocenská, E, Gregora, E, Procházka, B; Kozák, T	2002
Ivabradine and Bisoprolol on Doppler-derived Coronary Flow Velocity Reserve in Patients with Stable Coronary Artery Disease: beyond the Heart Rate	Tagliamonte, , E, Cirillo, T, Rigo, F, Astarita, C, Coppola, A, Romano, C; Capuano, N	2015
Improved glutathione status in young adult patients with cystic fibrosis supplemented with whey protein	Grey, , V, Mohammed, SR, Smountas, AA, Bahlool, R; Lands, LC	2003
TElmisartan in the management of abDominal aortic aneurYsm (TEDY): the study protocol for a randomized controlled trial	Morris, , DR, Cunningham, MA, Ahimastos, AA, Kingwell, BA, Pappas, E, Bourke, M, Reid, CM, Stijnen, T, Dalman, RL, Aalami, OO, Lindeman, JH, Norman, PE, Walker, PJ, Fitridge, R, Bourke, B, Dear, AE, Pinchbeck, J, Jaeggi, R; Golledge, J	2015
Recurrent Pericarditis	Imazio, , M, Gribaudo, E; Gaita, F	2017
Long-term follow-up of cardiovascular risk factors after exercise training in obese children	Maggio, , AB, Aggoun, Y, Martin, XE, Marchand, LM, Beghetti, M; Farpour-Lambert, NJ	2011
Beneficial effects of a Paleolithic diet on cardiovascular risk factors in type 2 diabetes: a randomized cross-over pilot study	Jönsson, , T, Granfeldt, Y, Ahrén, B, Branell, UC, Pålsson, G, Hansson, A, Söderström, M; Lindeberg, S	2009
Patient-reported outcomes and the association with clinical response in patients with active psoriatic arthritis treated with golimumab: findings through 2 years of a phase III, multicenter, randomized, double-blind, placebo-controlled trial	Kavanaugh, , A, McInnes, IB, Krueger, GG, Gladman, D, Beutler, A, Gathany, T, Mack, M, Tandon, N, Han, C; Mease, P	2013
Effect of cilostazol treatment on adiponectin and soluble CD40 ligand levels in diabetic patients with peripheral arterial occlusion disease	Hsieh, , CJ; Wang, PW	2009
Buccal administration of human colostrum: impact on the oral microbiota of premature infants	Sohn, , K, Kalanetra, KM, Mills, DA; Underwood, MA	2016
MTRX1011A, a humanized anti-CD4 monoclonal antibody, in the treatment of patients with rheumatoid arthritis: a phase I randomized, double-blind, placebo-controlled study incorporating pharmacodynamic biomarker assessments	Scheerens, , H, Su, Z, Irving, B, Townsend, MJ, Zheng, Y, Stefanich, E, Chindalore, V, Bingham, CO; Davis, JC	2011
Randomized phase III trial of pegfilgrastim versus filgrastim after autologus peripheral blood stem cell transplantation	Gerds, , A, Fox-Geiman, M, Dawravoo, K, Rodriguez, T, Toor, A, Smith, S, Kiley, K, Fletcher-Gonzalez, D, Hicks, C; Stiff, P	2010
Febuxostat improves endothelial function in hemodialysis patients with hyperuricemia: a randomized controlled study	Tsuruta, , Y, Kikuchi, K, Tsuruta, Y, Sasaki, Y, Moriyama, T, Itabashi, M, Takei, T, Uchida, K, Akiba, T, Tsuchiya, K; Nitta, K	2015
A study comparing the safety and clinical effects of different formulations of a new vaccine, ACI-24 with placebo in patients with mild Alzheimer's disease	EUCTR2018-000445-39-FI	2018
Low doses of losartan and trandolapril improve arterial stiffness in hemodialysis patients	Ichihara, , A, Hayashi, M, Kaneshiro, Y, Takemitsu, T, Homma, K, Kanno, Y, Yoshizawa, M, Furukawa, T, Takenaka, T; Saruta, T	2005

C-reactive protein and future cardiovascular events in statin-treated patients with angina pectoris: the extended TRUTH study	Nozue, , T, Fukui, K, Yamamoto, S, Kunishima, T, Umezawa, S, Onishi, Y, Tohyama, S, Takeyama, Y, Morino, Y, Yamauchi, T, Hibi, K, Sozu, T, Terashima, M; Michishita, I	2013
A Multi-Center Single Arm Phase II Study of MDX-010 (BMS-734016) Monotherapy in Patients with Previously Treated Unresectable Stage III or IV Melanoma. Revised Protocol 01, incorporating Amendment 02; and Pharmacogenetics Blood Sample Amendment #01, version 1.0 dated 01-Jul-05, Pharmacogenomics Archived Tissue Sample Amendment #03, version 1 dated 29-Mar-06 and Pharmacogenomic Biomarker Sample Amendment #04, version 1 dated 03-Apr-06	EUCTR2005-002051-41-NO	2006
Phase II clinical trial, conducted in different sites, with random assignation of treatment, where neither the patient or the medical doctor know the assigned treatment, drug or placebo, to evaluate the efficacy and safety of Rifaximin delayed release 400 mg tablet in the prevention of post-operative endoscopic Crohn's disease recurrence	EUCTR2017-002258-36-BE	2017
Effect of short-term colchicine treatment on endothelial function in patients with coronary artery disease	Kajikawa, , M, Higashi, Y, Tomiyama, H, Maruhashi, T, Kurisu, S, Kihara, Y, Mutoh, A; Ueda, S-I	2019
Parenteral nutrition-associated hyperglycemia in non-critically iII inpatients increases the risk of in- hospital mortality (multicenter study)	Olveira, , G, Tapia, MJ, Ocon, J, Cabrejas-Gomez, C, Ballesteros-Pomar, MD, Vidal-Casariego, A, Arraiza-Irigoyen, C, Olivares, J, Conde-Garcia, MDC, Garcia-Manzanares, A, Botella-Romero, F, Quilez-Toboso, RP, Cabrerizo, L, Matia, P, Chicharro, L, Burgos, R, Pujante, P, Ferrer, M, Zugasti, A, Prieto, J, Dieguez, M, Carrera, MJ, Vila-Bundo, A, Urgeles, JR, Aragon-Valera, C, Rovira, A, Breton, I, Garcia-Peris, P, Munoz-Garach, A, Marquez, E, Olmo, DD, Pereira, JL; Tous, MC	2013
Randomized, Double-Blind, Placebo-Controlled Phase II Study Comparing the Safety of Ipilimumab Administered With or Without Prophylactic Oral Budesonide (Entocort EC) in Patients with Unresectable Stage III or IV Malignant Melanoma Revised Protocol 03 Incorporating Amendment 02, 03 and 05 (version 1.0, dated 12-Mar-07) + Pharmacogenetics Blood Sample Amendment 01 - Site Specific (version 1.0 dated 01-Aug-05)	EUCTR2005-002678-31-GB	2006
p53-Adjusted Neoadjuvant Chemotherapy for potentially resectable Oesophageal Cancer "pANCHO". - pANCHO	EUCTR2006-006647-31-AT	2007
221AD302 Phase 3 Study of Aducanumab in Early Alzheimer's Disease	EUCTR2015-000967-15-FR	2015
A Study of Lebrikizumab in Patients with Chronic Obstructive Pulmonary Disease	EUCTR2015-001122-42-HU	2015
The effect of vitamin D3 supplementation in treatment of Irritable Bowel Syndrome	IRCT2015042521935N1	2015
Von Willebrand factor, soluble P-selectin, and target organ damage in hypertension: a substudy of the Anglo-Scandinavian Cardiac Outcomes Trial (ASCOT)	Spencer, , CG, Gurney, D, Blann, AD, Beevers, DG; Lip, GY	2002
Receptor for Hyaluronan-Mediated Motility (RHAMM [CD168]) peptide vaccination for patients with haematological malignancies: a phaseI/II pilot study	ISRCTN32763606	2007
DOUBLE BLIND, DOUBLE DUMMY, RANDOMISED, PARALLEL GROUP, MONOCENTRIC CLINICAL TRIAL ON THE EFFECTS OF CHF 1535 MDI OR SERETIDE DPI ON BIOLOGICAL AND FUNCTIONAL MARKERS OF SMALL AIRWAYS IN PATIENTS WITH ASTHMA: a PILOT STUDY - ND	EUCTR2006-000512-25-IT	2007
to Determine Efficacy and Safety of Extended Drotrecogin Alfa (Activated) Therapy in Patients With Persistent Requirement for Vasopressor Support After 96-Hour Infusion With Commercial Drotrecogin Alfa (Activated	EUCTR2004-001319-71-ES	2012
Estudio aleatorizado, abierto y con control activo sobre la seguridad, la eficacia y la farmacocinética de ferumoxitol en comparación con hierro oral para el tratamiento de la anemia ferropénica en sujetos pediátricos con nefropatía crónica dependiente de diálisis. A Randomized, Open-Label, Active-Controlled Study of the Safety, Efficacy, and Pharmacokinetics of Ferumoxytol Compared with Oral Iron for the Treatment of Iron Deficiency Anemia in Pediatric Subjects with Dialysis dependent Chronic Kidney Disease	EUCTR2010-019387-37-ES	2010
Comparison of Efficacy and Safety of Two Biphasic Defibrillators in Cardioversion of Atrial Fibrillation	NCT04032678	2019
Oral tetrahydrouridine and decitabine for non-cytotoxic epigenetic gene regulation in sickle cell disease: a randomized phase 1 study	Molokie, , R, Lavelle, D, Gowhari, M, Pacini, M, Krauz, L, Hassan, J, Ibanez, V, Ruiz, MA, Ng, KP, Woost, P, Radivoyevitch, T, Pacelli, D, Fada, S, Rump, M, Hsieh, M, Tisdale, JF, Jacobberger, J, Phelps, M, Engel, JD, Saraf, S, Hsu, LL, Gordeuk, V, DeSimone, J; Saunthararajah, Y	2017
Study to assess the safety, tolerability, and efficacy of PF-05212384 in combination with chemotherapy in patients with metastatic colorectal cancer	EUCTR2013-002096-18-ES	2014
Tiral of Invasive versus Medical therapy of Early coronary artery disease in Diabetes (TIME-DM) - TIME-DM	EUCTR2006-001430-40-DE	2006
NEBIVOLOL OR METOPROLOL IN ARTERIAL OCCLUSIVE DISEASE A DOUBLE-BLIND, RANDOMISED CLINICAL Deutscher Titel: nebivolol im Vergleich zu Metoprolol bei peripherer arterieller	EUCTR2005-003583-31-DE	2006

Verschlusskrankheit Eine randomisierte, doppel-blinde klinische Studie - NORMA		
Kefir and Metabolic Syndrome	NCT03649828	2018
The BP2 (Blood Pressure Postpartum) study: a 3-arm, randomised trial of follow-up and lifestyle behaviour change strategies in the first 12 months after hypertensive disorders of pregnancy	ACTRN12618002004246	2018
Cognitive Behavioural Therapy for Insomnia on Sleep in Rheumatoid Arthritis	NCT03766100	2018
IBD NURSE INTERVENTION FOR PATIENTS ASSIGNED TO BIOLOGIC THERAPY DECREASES UNCERTAINTY AND IMPROVES PATIENTSâ€™ REPORTED OUTCOMES	Barkan, , R, Goren, I, Avni-Biron, I, Snir, Y, Broitman, Y, Leibovitzh, H, Banai, H, Siterman, M, Hazan, R, Pfeffer-Gik, T, Godny, L, Zonensain, K, Dotan, I; Yanai, HA	2019
Selective COX-2 inhibition improves endothelial function in coronary artery disease	Chenevard, , R, Hürlimann, D, Béchir, M, Enseleit, F, Spieker, L, Hermann, M, Riesen, W, Gay, S, Gay, RE, Neidhart, M, Michel, B, Lüscher, TF, Noll, G; Ruschitzka, F	2003
Study to evaluate the efficacy and safety of maintenance therapy with PankoMab-GEX™ after chemotherapy in patients with recurrent epithelial ovarian carcinoma	EUCTR2013-000931-28-DE	2013
Statin use and leg functioning in patients with and without lower-extremity peripheral arterial disease	McDermott, , MM, Guralnik, JM, Greenland, P, Pearce, WH, Criqui, MH, Liu, K, Taylor, L, Chan, C, Sharma, L, Schneider, JR, Ridker, PM, Green, D; Quann, M	2003
An Open-Label, Multicenter, Phase III Trial of ABI-007 vs. Dacarbazine in Previously Untreated Patients with Metastatic Malignant Melanoma - ND	EUCTR2007-004097-32-IT	2009
221AD301 Phase 3 Study of Aducanumab in Early Alzheimer's Disease	EUCTR2015-000966-72-ES	2015
Endothelin and Ischemic Optic Neuropathy study group	EUCTR2014-000848-14-FR	2015
Multi-centre, randomized, double-blind, placebo-controlled, phase II study of SER150TBS in type II diabetic patients	EUCTR2014-003985-25-DE	2015
Improvement in the outcome of rejection with pentoxifylline in renal transplantation: a randomized controlled trial	Noel, , C, Hazzan, M, Labalette, M, Coppin, MC, Jude, B, Dessaint, JP; Lelievre, G	1998
Cytomegalovirus-specific immune monitoring to guide pre-emptive therapy surveillance protocol for prevention of cytomegalovirus infection after renal transplantation	ACTRN12618000913279	2018
Comparison of Preconditioning Effect of High Dose and Low Dose Atorvastatin Treatment in Patients Underwent CABG	IRCT2017080619470N61	2017
Peptide-based vaccine in combination or not with chemotherapy in melanoma patients: a phase II randomized clinical study. - ND	EUCTR2008-008211-26-IT	2009
Clinical efficacy and cell mobilization activity of pegfilgrastim in patients with gynaecological malignancies in therapy with topotecan. - ND	EUCTR2007-006069-34-IT	2007
Interventions for maintenance of surgically induced remission in Crohn's disease: a network meta-analysis	Iheozor-Ejiofor, , Z, Gordon, M, Clegg, A, Freeman, SC, Gjuladin-Hellon, T, MacDonald, JK; Akobeng, AK	2019
Public health deworming programmes for soil-transmitted helminths in children living in endemic areas	Taylor-Robinson, , DC, Maayan, N, Donegan, S, Chaplin, M; Garner, P	2019
Topical cyclosporine A therapy for dry eye syndrome	de Paiva, , CS, Pflugfelder, SC, Ng, SM; Akpek, EK	2019
Interventions for uterine fibroids: an overview of Cochrane Reviews	Brito, , LG, Stewart, EA, Olivi Chaim, SO, Martins, WP; Farquhar, C	2019
Presurgery conditioning interventions (prehabilitation) in adults undergoing lower limb surgery for peripheral arterial disease	Palmer, , J, Pymer, S, Smith, GE, Harwood, AE, Ingle, L, Huang, C; Chetter, IC	2019
Drugs to reduce bleeding and transfusion in adults undergoing cardiac surgery: a systematic review and network meta-analysis	Beverly, , A, Ong, G, Wilkinson, KL, Doree, C, Welton, NJ; Estcourt, LJ	2019
Birth room transition support for preterm infants: a Cochrane overview	Brown, , JVE, Walsh, V; McGuire, W	2019
Information and promotional strategies by pharmaceutical companies for clinicians	Mintzes, , B, Fabbri, A, Grundy, Q, Spurling, GKP, Lexchin, J, McKenzie, JE; Bero, L	2019
Interventions for replacing missing teeth: maintaining and recovering soft tissue health around dental implants	Grusovin, , MG, Coulthard, P, Worthington, HV, George, P; Esposito, M	2010
Interventions for replacing missing teeth: different times for loading dental implants	Esposito, , M, Grusovin, MG, Maghaireh, H; Worthington, HV	2013
Interventions for replacing missing teeth: management of soft tissues for dental implants	Esposito, , M, Maghaireh, H, Grusovin, MG, Ziounas, I; Worthington, HV	2012
Interventions for replacing missing teeth: attachment systems for implant overdentures in edentulous jaws	Payne, , AGT, Alsabeeha, NHM, Atieh, MA, Esposito, M, Ma, S; Anas El-Wegoud, M	2018
Reinforcement of anchorage during orthodontic brace treatment with implants or other surgical methods	Jambi, , S, Walsh, T, Sandler, J, Benson, PE, Skeggs, RM; O'Brien, KD	2014
Routine scale and polish for periodontal health in adults	Lamont, , T, Worthington, HV, Clarkson, JE; Beirne, PV	2018
Home use of interdental cleaning devices, in addition to toothbrushing, for preventing and controlling periodontal diseases and dental caries	Worthington, , HV, MacDonald, L, Poklepovic Pericic, T, Sambunjak, D, Johnson, TM, Imai, P; Clarkson, JE	2019
Chlorhexidine mouthrinse as an adjunctive treatment for gingival health	James, , P, Worthington, HV, Parnell, C, Harding, M, Lamont, T, Cheung, A, Whelton, H; Riley, P	2017
Supportive periodontal therapy (SPT) for maintaining the dentition in adults treated for periodontitis	Manresa, , C, Sanz-Miralles, EC, Twigg, J; Bravo, M	2018
Hormone therapy for preventing cardiovascular disease in post-menopausal women	Boardman, , HMP, Hartley, L, Eisinga, A, Main, C, Roqué i Figuls, M, Bonfill Cosp, X, Gabriel Sanchez, R; Knight, B	2015

Long-term hormone therapy for perimenopausal and postmenopausal women	Marjoribanks, , J, Farquhar, C, Roberts, H, Lethaby, A; Lee, J	2017
Interventions for treating proximal humeral fractures in adults	Handoll, , HHG; Brorson, S	2015
Surgical options for lumbar spinal stenosis	Machado, , GC, Ferreira, PH, Yoo, RIJ, Harris, IA, Pinheiro, MB, Koes, BW, van Tulder, MW, Rzewuska, M, Maher, CG; Ferreira, ML	2016
Testosterone for peri and postmenopausal women	Somboonporn, , W, Bell, RJ; Davis, SR	2005
Wound drainage after plastic and reconstructive surgery of the breast	Khan, , SM, Smeulders, MJC; Van der Horst, CM	2015
Urethral injection therapy for urinary incontinence in women	Kirchin, , V, Page, T, Keegan, PE, Atiemo, KOM, Cody, JD, McClinton, S; Aluko, P	2017
Antifibrinolytic therapy for preventing oral bleeding in people on anticoagulants undergoing minor oral surgery or dental extractions	Engelen, , ET, Schutgens, REG, Mauser-Bunschoten, EP, van Es, RJJ; van Galen, KPM	2018
Implantable loop recorder versus conventional diagnostic workup for unexplained recurrent syncope	Solbiati, , M, Costantino, G, Casazza, G, Dipaola, F, Galli, A, Furlan, R, Montano, N; Sheldon, R	2016
Anaesthetic techniques for risk of malignant tumour recurrence	Cakmakkaya, , OS, Kolodzie, K, Apfel, CC; Pace, NL	2014
Fresh frozen plasma for cardiovascular surgery	Desborough, , M, Sandu, R, Brunskill, SJ, Doree, C, Trivella, M, Montedori, A, Abraha, I; Stanworth, S	2015
Antimicrobial agents for preventing peritonitis in peritoneal dialysis patients	Campbell, , D, Mudge, DW, Craig, JC, Johnson, DW, Tong, A; Strippoli, GFM	2017
Plasma transfusions prior to insertion of central lines for people with abnormal coagulation	Hall, , DP, Estcourt, LJ, Doree, C, Hopewell, S, Trivella, M; Walsh, TS	2016
Comparison of different platelet transfusion thresholds prior to insertion of central lines in patients with thrombocytopenia	Estcourt, , LJ, Desborough, M, Hopewell, S, Doree, C; Stanworth, SJ	2015
Implantable defibrillators versus medical therapy for cardiac channelopathies	McNamara, , DA, Goldberger, JJ, Berendsen, MA; Huffman, MD	2015
Closure versus medical therapy for preventing recurrent stroke in patients with patent foramen ovale and a history of cryptogenic stroke or transient ischemic attack	Li, , J, Liu, J, Liu, M, Zhang, S, Hao, Z, Zhang, J; Zhang, C	2015
Lightweight versus Heavyweight mesh for open repair of inguinal hernia	Sajid, , MS, Leaver, C, Baig, MK; Sains, P	2013
Accommodative intraocular lens versus standard monofocal intraocular lens implantation in cataract surgery	Ong, , HS, Evans, JR; Allan, BDS	2014
Intraoperative interventions for preventing surgical site infection: an overview of Cochrane Reviews	Liu, , Z, Dumville, JC, Norman, G, Westby, MJ, Blazeby, J, McFarlane, E, Welton, NJ, O'Connor, L, Cawthorne, J, George, RP, Crosbie, EJ, Rithalia, AD; Cheng, HY	2018
Endovascular repair of abdominal aortic aneurysm	Paravastu, , SCV, Jayarajasingam, R, Cottam, R, Palfreyman, SJ, Michaels, JA; Thomas, SM	2014
Open retropubic colposuspension for urinary incontinence in women	Lapitan, , MCM, Cody, JD; Mashayekhi, A	2017
Surgical versus non-surgical interventions for treating humeral shaft fractures in adults	Gosler, , MW, Testroote, M, Morrenhof, JW; Janzing, HMJ	2012
Bladder neck needle suspension for urinary incontinence in women	Glazener, , CMA, Cooper, K; Mashayekhi, A	2017
Anterior vaginal repair for urinary incontinence in women	Glazener, , CMA, Cooper, K; Mashayekhi, A	2017
Interventions for replacing missing teeth: Alveolar ridge preservation techniques for dental implant site development	Atieh M.A.; Alsabeeha N.H.; Payne A.G.; Duncan W.; Faggion C.M.; Esposito M.	2015
Interventions for replacing missing teeth: Different types of dental implants	Esposito M.; Ardebili Y.; Worthington H.V.	2014
Interventions for missing teeth: Removable prostheses for the edentulous mandible	Jahangiri, , L, Choi, M, Moghadam, M; Jawad, S	2015
Periarticular analgesia for postoperative pain following total hip replacement	Hadi, , M, Sprowson, A, Bucknall, T, Underwood, M, Costa, ML; Buchbinder, R	2014
Periarticular analgesia for postoperative pain following total knee replacement	Sprowson, , A, Hadi, M, Underwood, M, Parsons, N, Bucknall, T, Costa, ML; Buchbinder, R	2014
Interventions for replacing missing teeth: Implant placement at different levels in relation to crestal bone	Khouly I.; Veitz-Keenan A.; Loomer P.M.; Esposito M.	2017
Ridge Regeneration in Three Wall Sockets	NCT03548896	2018
Prospective pilot study to investigate the one-Abutment-one-time procedure concerning the periimplant tissue	DRKS00013174	2017
Comparative analysis in implant-supported metal ceramic and hibrid ceramic crown	RBR-9w9m83	2018
Levels of osteoclastogenesis-related factors in the peri-implant crevicular fluid and clinical parameters of immediately loaded implants in patients with osteopenia: a short-term report	Onuma, , T, Aquiar, K, Duarte, PM, Feres, M, Giro, G, Coelho, P, Cassoni, A; Shibli, JA	2015
The effect of keratinized mucosa width on peri-implant outcome under supportive postimplant therapy	Frisch, Eberhard; Ziebolz, Dirk; Vach, Kirstin; Ratka-Krüger, Petra	2015
The influence of controlled occlusal overload on peri-implant tissue. Part 4: a histologic study in monkeys.	Miyata, Takashi; Kobayashi, Yukinao; Araki, Hisao; Ohto, Takaichi; Shin, Kitetsu	2002
Detection of Prostaglandin E 2 and Matrix Metalloproteinases in Implant Crevicular Fluid.	Aboyoussef, Hoda; Carter, Cristina; Jandinski, John J.; Panagakos, Fotinos S.	1998
Peri-implant bone level around implants with platform-switched abutments	Fickl, Stefan; Zuhr, Otto; Stein, Jamal M.; Hürzeler, Markus B.	2010
The causes of early implant bone loss: myth or science?	Oh, Tae-Ju; Yoon, Joongkyo; Misch, Carl E.; Wang, Hom-Lay	2002

Effects of local administration of growth hormone in peri-implant bone: an experimental study with implants in rabbit tibiae.	Tresguerres, Isabel F.; Blanco, Luis; Clemente, Celia; Tresguerres, Jesús AF	2003
Effect of immobilized bone morphogenic protein 2 coating of titanium implants on peri-implant bone formation	Schliephake, Henning; Aref, Arash; Scharnweber, Dieter; Bierbaum, Susanne; Roessler, Sophie; Sewing, Andreas	2005
Oral implant surfaces: Part 1–review focusing on topographic and chemical properties of different surfaces and in vivo responses to them.	Albrektsson, Tomas; Wennerberg, Ann	2004
The impact of different torques for the insertion of immediately loaded implants on the peri-implant levels of angiogenesis- and bone-related markers	Verrastro Neto A.; Andrade R.; Corrêa M.G.; Casarin R.C.V.; Casati M.Z.; Pimentel S.P.; Ribeiro F.V.; Cirano F.R.	2018
Comparison of two different techniques used for the maintenance of peri-implant soft tissue health: a pilot randomized clinical trial	Al Ghazal L.; O'Sullivan J.; Claffey N.; Polyzois I.	2017
Bone Response and Soft Tissue Changes Around Implants With/Without Abutments Supporting Fixed Partial Dentures: Results From a 3-Year, Prospective, Randomized, Controlled Study	Göthberg C.; André U.; Gröndahl K.; Thomsen P.; Slotte C.	2016
Maxillary sinus floor elevation surgery with BioOss® mixed with a bone marrow concentrate or autogenous bone: Test of principle on implant survival and clinical performance	Rickert D.; Vissink A.; Slot W.J.; Sauerbier S.; Meijer H.J.A.; Raghoebar G.M.	2014
Gene Expression of Inflammation and Bone Healing in Peri-Implant Crevicular Fluid after Placement and Loading of Dental Implants. A Kinetic Clinical Pilot Study Using Quantitative Real-Time PCR	Slotte C.; Lennerås M.; Göthberg C.; Suska F.; Zoric N.; Thomsen P.; Nannmark U.	2012
Comparison of pro-inflammatory cytokines and bone metabolism mediators around titanium and zirconia dental implant abutments following a minimum of 6 months of clinical function	Barwacz, Christopher A.; Brogden, Kim A.; Stanford, Clark M.; Dawson, Deborah V.; Recker, Erica N.; Blanchette, Derek	2015
Can degradation products released from dental implants affect peri-implant tissues?	Noronha Oliveira, M.; Schunemann, W. V. H.; Mathew, M. T.; Henriques, B.; Magini, R. S.; Teughels, W.; Souza, J. C. M.	2018
Composition of human peri-implantitis and periodontitis lesions.	Carcuac, O.; Berglundh, T.	2014
Role of toll-like receptor 2 in inflammation and alveolar bone loss in experimental peri-implantitis versus periodontitis.	Yu, X.; Hu, Y.; Freire, M.; Yu, P.; Kawai, T.; Han, X.	2018
Biomarkers and Bacteria Around Implants and Natural Teeth in the Same Individuals.	Gurlek, Onder; Gumus, Pinar; Nile, Christopher J.; Lappin, David F.; Buduneli, Nurcan	2017
Prediction of individual implant bone levels and the existence of implant "phenotypes".	Papantonopoulos, Georgios; Gogos, Christos; Housos, Efthymios; Bountis, Tassos; Loos, Bruno G.	2017
Ligature induced peri-implantitis: tissue destruction and inflammatory progression in a murine model.	Nguyen Vo, Trang N.; Hao, Jia; Chou, Josh; Oshima, Masamitsu; Aoki, Kazuhiro; Kuroda, Shinji; Kaboosaya, Boosana; Kasugai, Shohei	2017
A bacterial-biofilm-induced oral osteolytic infection can be successfully treated by immuno-targeting an extracellular nucleoid-associated protein.	Freire, M. O.; Devaraj, A.; Young, A.; Navarro, J. B.; Downey, J. S.; Chen, C.; Bakaletz, L. O.; Zadeh, H. H.; Goodman, S. D.	2017
Biology of soft tissue wound healing and regeneration--consensus report of Group 1 of the 10th European Workshop on Periodontology.	Hammerle, Christoph H. F.; Giannobile, William V.	2014
The role of lasers in the treatment of peri-implant diseases: A review.	Alshehri, Fahad Ali	2016
Clinical and radiographic peri-implant parameters and proinflammatory cytokine levels among cigarette smokers, smokeless tobacco users, and nontobacco users.	Akram, Zohaib; Vohra, Fahim; Bukhari, Ishfaq A.; Sheikh, Saeed A.; Javed, Fawad	2018
Host-derived biomarkers at teeth and implants in partially edentulous patients. A	Ramseier, Christoph A.; Eick, Sigrun; Bronnimann, Claudia; Buser, Daniel; Bragger, Urs; Salvi, Giovanni E.	2016
Comparison of Peri-Implant Soft Tissue Parameters and Crestal Bone Loss Around Immediately Loaded and Delayed Loaded Implants in Smokers and Non-Smokers: 5-Year Follow-Up Results.	Al Amri, Mohammad D.; Kellesarian, Sergio Varela; Abduljabbar, Tariq S.; Al Rifaiy, Mohammad Q.; Al Baker, Abdulaziz M.; Al-Kheraif, Abdulaziz A.	2017
Comparison between inflammation-related markers in peri-implant crevicular fluid and clinical parameters during osseointegration in edentulous jaws.	Bielemann, Amalia M.; Marcello-Machado, Raissa M.; Leite, Fabio Renato Manzolli; Martinho, Frederico Canato; Chagas-Junior, Otacilio Luiz; Antoninha Del Bel Cury, Altair; Faot, Fernanda	2018
Haplotypes in BMP4 and FGF Genes Increase the Risk of Peri-Implantitis.	Coelho, Renata Barboza; Goncalves, Roberto Junior; Villas-Boas, Ricardo de Mello; Bonato, Leticia Ladeira; Quinelato, Valquiria; Pinheiro, Aristides da Rosa; Machado, Aldir; Nunes, Carlos Henrique Ramirez; Goncalves, Rackel; Vieira, Alexandre Resende; Granjeiro, Jose Mauro; Casado, Priscila Ladeira	2016
The Influence of Smoking on the Peri-Implant Microbiome.	Tsigarida, A. A.; Dabdoub, S. M.; Nagaraja, H. N.; Kumar, P. S.	2015
The pathology of bone tissue during peri-implantitis.	Schminke, B.; Vom Orde, F.; Gruber, R.; Schliephake, H.; Burgers, R.; Miosge, N.	2015
Establishment of Epithelial Attachment on Titanium Surface Coated with Platelet Activating Peptide.	Sugawara, Shiho; Maeno, Masahiko; Lee, Cliff; Nagai, Shigemi; Kim, David M.; Da Silva, John; Nagai, Masazumi; Kondo, Hisatomo	2016
An Exploratory Case-Control Study on the Impact of IL-1 Gene Polymorphisms on Early Implant Failure.	Cosyn, Jan; Christiaens, Veronique; Koningsveld, Vincent; Coucke, Paul J.; De Coster, Peter; De Paepe, Anne; De Bruyn, Hugo	2016
Diagnosing peri-implant disease using the tongue as a 24/7 detector.	Ritzer, J.; Luhmann, T.; Rode, C.; Pein-Hackelbusch, M.; Immohr, I.; Schedler, U.; Thiele, T.; Stubinger, S.; Rechenberg, B. V.; Waser-Althaus, J.; Schlottig, F.; Merli, M.; Dawe, H.; Karpisek, M.; Wyrwa, R.; Schnabelrauch, M.; Meinel, L.	2017
Drugs and diseases: Summary and consensus statements of group 1. The 5(th) EAO Consensus Conference 2018.	Schliephake, Henning; Sicilia, Alberto; Nawas, Bilal Al; Donos, Nikos; Gruber, Reinhard; Jepsen, Soren; Milinkovic, Iva; Mombelli, Andrea; Navarro, Jose Manuel; Quirynen, Marc; Rocchietta, Isabella; Schiodt, Morten; Schou, Soren; Stahli, Alexandra; Stavropoulos, Andreas	2018

Mechanism and Prevention of Titanium Particle-Induced Inflammation and Osteolysis.	Eger, Michal; Hiram-Bab, Sahar; Liron, Tamar; Sterer, Nir; Carmi, Yaron; Kohavi, David; Gabet, Yankel	2018
Clinical peri-implant parameters and inflammatory cytokine profile among smokers of cigarette, e-cigarette, and waterpipe.	AlQahtani, Mohammed Ayedh; Alayad, Abdullah Saeed; Alshihri, Abdulmonem; Correa, Fernanda Oliveira Bello; Akram, Zohaib	2018
Impact of heavy smoking on the clinical, microbiological and immunological parameters of patients with dental implants: a prospective cross-sectional study.	Ata-Ali, Javier; Flichy-Fernandez, Antonio Juan; Alegre-Domingo, Teresa; Ata-Ali, Fadi; Penarrocha-Diago, Miguel	2016
Clinical and Radiographic Peri-Implant Parameters and Whole Salivary Interleukin-1beta and Interleukin-6 Levels among Type-2 Diabetic and Nondiabetic Patients with and without Peri-Implantitis.	Al-Askar, Mansour; Ajlan, Sumaiah; Alomar, Nuha; Al-Daghri, Nasser M.	2018
Bone and soft tissue outcomes, risk factors, and complications of implant-supported prostheses: 5-Years RCT with different abutment types and loading protocols.	Gothberg, Catharina; Grondahl, Kerstin; Omar, Omar; Thomsen, Peter; Slotte, Christer	2018
Effect of history of periodontitis on implant success: meta-analysis and systematic review.	Ramanauskaite, Ausra; Baseviciene, Nomeda; Wang, Hom-Lay; Tozum, Tolga F.	2014
Genetic involvement in dental implant failure: Association with polymorphisms of genes modulating inflammatory responses and bone metabolism.	Chen, Xun; Zhao, Yu	2019
Proinflammatory cytokine levels and peri-implant parameters among cigarette smokers, individuals vaping electronic cigarettes, and non-smokers.	ArRejaie, Aws S.; Al-Aali, Khulud Abdulrahman; Alrabiah, Mohammed; Vohra, Fahim; Mokeem, Sameer A.; Basunbul, Ghadeer; Alrahlah, Ali; Abduljabbar, Tariq	2019
Significant Enrichment and Diversity of the Staphylococcal Arginine Catabolic Mobile Element ACME in Staphylococcus epidermidis Isolates From Subgingival Peri-implantitis Sites and Periodontal Pockets.	O'Connor, Aoife M.; McManus, Brenda A.; Kinnevey, Peter M.; Brennan, Grainne I.; Fleming, Tanya E.; Cashin, Phillipa J.; O'Sullivan, Michael; Polyzois, Ioannis; Coleman, David C.	2018
Effect of lentiviral vector overexpression alpha-calcitonin gene-related peptide on titanium implant osseointegration in alpha-CGRP-deficient mice.	Xiang, Lin; Ma, Li; Wei, Na; Wang, Tianlu; Yao, Qianqian; Yang, Bo; Xiong, Yi; Wu, Yingying; Gong, Ping	2017
Sophocarpine attenuates wear particle-induced implant loosening by inhibiting osteoclastogenesis and bone resorption via suppression of the NF-kappaB signalling pathway in a rat model.	Zhou, Chen-He; Shi, Zhong-Li; Meng, Jia-Hong; Hu, Bin; Zhao, Chen-Chen; Yang, Yu-Te; Yu, Wei; Chen, Ze-Xin; Heng, Boon Chin; Parkman, Virginia-Jeni Akila; Jiang, Shuai; Zhu, Han-Xiao; Wu, Hao-Bo; Shen, Wei-Liang; Yan, Shi-Gui	2018
Identification of microbiota in peri-implantitis pockets by matrix-assisted laser desorption/ionization time-of-flight mass spectrometry.	Yeh, Hwey-Chin; Lu, Jang-Jih; Chang, Shih-Cheng; Ge, Mao-Cheng	2019
Influence of titanium on in vitro fibroblast-Porphyromonas gingivalis interaction in peri-implantitis.	Irshad, Muhammad; Scheres, Nina; Crielaard, Wim; Loos, Bruno G.; Wismeijer, Daniel; Laine, Marja L.	2013
Bone loss biomarkers associated with peri-implantitis. A cross-sectional study.	Rakic, Mia; Lekovic, Vojislav; Nikolic-Jakoba, Natasa; Vojvodic, Danilo; Petkovic-Curcin, Aleksandra; Sanz, Mariano	2013
Clinical and laboratory evaluation of the effects of different treatment modalities on titanium healing caps: a randomized, controlled clinical trial.	Schmidt, Kristina Emily; Auschill, Thorsten Mathias; Heumann, Christian; Frankenberger, Roland; Eick, Sigrun; Sculean, Anton; Arweiler, Nicole Birgit	2018
VEGF, Microvessel Density, and CD44 as Inflammation Markers in Peri-implant Healthy Mucosa, Peri-implant Mucositis, and Peri-implantitis: Impact of Age, Smoking, PPD, and Obesity.	Lucarini, Guendalina; Zizzi, Antonio; Rubini, Corrado; Ciolino, Francesco; Aspriello, Simone Domenico	2019
Surface treatments on titanium implants via nanostructured ceria for antibacterial and anti-inflammatory capabilities.	Li, Xue; Qi, Manlin; Sun, Xiaolin; Weir, Michael D.; Tay, Franklin R.; Oates, Thomas W.; Dong, Biao; Zhou, Yanmin; Wang, Lin; Xu, Hockin H. K.	2019
Influence of titanium ions on cytokine levels of murine splenocytes stimulated with periodontopathic bacterial lipopolysaccharide.	Nishimura, Kota; Kato, Tetsuo; Ito, Taichi; Oda, Takashi; Sekine, Hideshi; Yoshinari, Masao; Yajima, Yasutomo	2014
Soft tissue wound healing at teeth, dental implants and the edentulous ridge when using barrier membranes, growth and differentiation factors and soft tissue substitutes.	Vignoletti, Fabio; Nunez, Javier; Sanz, Mariano	2014
Clinical outcome of a nonsurgical and surgical treatment protocol in different types of peri-implantitis: a case series.	Thierbach, Rene; Eger, Thomas	2013
The role of nicotine, cotinine and caffeine on the electrochemical behavior and bacterial colonization to cp-Ti.	Barao, Valentim A. R.; Ricomini-Filho, Antonio P.; Faverani, Leonardo P.; Del Bel Cury, Altair A.; Sukotjo, Cortino; Monteiro, Douglas R.; Yuan, Judy Chia-Chun; Mathew, Mathew T.; do Amaral, Regiane C.; Mesquita, Marcelo F.; da Silva, Wander J.; Assuncao, Wirley G.	2015
The impact of Aggregatibacter actinomycetemcomitans biofilm-derived effectors following antimicrobial photodynamic therapy on cytokines production in human gingival fibroblasts.	Peeridogaheh, Hadi; Pourhajibagher, Maryam; Barikani, Hamid Reza; Bahador, Abbas	2019
In vivo osteoprotegerin gene therapy preventing bone loss induced by periodontitis.	Tang, H.; Mattheos, N.; Yao, Y.; Jia, Y.; Ma, L.; Gong, P.	2015
Would nitric oxide be an effective marker for earlier stages of peri-implant disease? An analysis in human peri-implant sulcular fluid.	Vieira, Andreia Espindola; Moura, Camilla Christian Gomes; de Souza, Maria Aparecida; Zanetta-Barbosa, Darceny; Dechichi, Paula	2013
Cytokine, chemokine, and growth factor levels in peri-implant sulcus during wound healing and osseointegration after piezosurgical versus conventional implant site preparation: Randomized, controlled, split-mouth trial.	Gurkan, Ali; Tekdal, Gozde Peker; Bostanci, Nagihan; Belibasakis, Georgios N.	2019
Analysis of cathepsin-K activity at tooth and dental implant sites and the potential of this enzyme in reflecting alveolar bone loss.	Yamalik, Nermin; Gunday, Sevim; Uysal, Serdar; Kilinc, Kamer; Karabulut, Erdem; Tozum, Tolga F.	2012
Antimicrobial activity of nanoparticulate metal oxides against peri-implantitis pathogens.	Vargas-Reus, Miguel A.; Memarzadeh, Kaveh; Huang, Jie; Ren, Guogang G.; Allaker, Robert P.	2012
Concentration- and time-dependent response of human gingival fibroblasts to fibroblast growth factor 2 immobilized on titanium dental implants.	Ma, Qianli; Wang, Wei; Chu, Paul K.; Mei, Shenglin; Ji, Kun; Jin, Lei; Zhang, Yumei	2012

Biological factors involved in implant-anchored orthodontics and in prosthetic-implant therapy: a literature review.	Antoszewska, Joanna; Raftowicz-Wojcik, Katarzyna; Kawala, Beata; Matthews-Brzozowska, Teresa	2010
Impact of implant overloading on the peri-implant bone in inflamed and non-inflamed peri-implant mucosa.	Kozlovsky, Avital; Tal, Haim; Laufer, Ben-Zion; Leshem, Roy; Rohrer, Michael D.; Weinreb, Miron; Artzi, Zvi	2007
Collagenase-2 (MMP-8) as a point-of-care biomarker in periodontitis and cardiovascular diseases. Therapeutic response to non-antimicrobial properties of tetracyclines.	Sorsa, Timo; Tervahartiala, Taina; Leppilahti, Jussi; Hernandez, Marcela; Gamonal, Jorge; Tuomainen, Anita M.; Lauhio, Anneli; Pussinen, Pirkko J.; Mantyla, Paivi	2011
Effects of diabetes mellitus on periodontal and peri-implant conditions: update on associations and risks.	Salvi, Giovanni E.; Carollo-Bittel, Barbara; Lang, Niklaus P.	2008
Two-stage implant systems.	Fritz, M. E.	1999
A novel bionic design of dental implant for promoting its long-term success using nerve growth factor (NGF): utilizing nano-springs to construct a stress-cushioning structure inside the implant.	He, Hao; Yao, Yang; Wang, Yanying; Wu, Yingying; Yang, Yang; Gong, Ping	2012
Occult peri-implant oroantral fistulae: posterior maxillary peri-implantitis/sinusitis of zygomatic or dental implant origin. Treatment and prevention with bone morphogenetic protein-2/absorbable collagen sponge sinus grafting.	Jensen, Ole T.; Adams, Mark; Cottam, Jared R.; Ringeman, Jason	2013
Single versus double flap approach in periodontal regenerative treatment.	Schincaglia, Gian Pietro; Hebert, Eric; Farina, Roberto; Simonelli, Anna; Trombelli, Leonardo	2015
Immediate implant placement into fresh extraction sites with chronic periapical pathologic features combined with plasma rich in growth factors: preliminary results of single-cohort study.	Del Fabbro, Massimo; Boggian, Cristiano; Taschieri, Silvio	2009
Bone formation in peri-implant defects grafted with microparticles: a pilot animal experimental study.	Moest, Tobias; Koehler, Franz; Prechtl, Christopher; Schmitt, Christian; Watzek, Georg; Schlegel, Karl Andreas	2014
Early colonization of non-submerged dental implants in patients with a history of advanced aggressive periodontitis.	De Boever, Annemarie L.; De Boever, Jan A.	2006
Evaluation of implant osseointegration with different regeneration techniques in the treatment of bone defects around implants: an experimental study in a rabbit model.	Guerra, Isabel; Morais Branco, Fernando; Vasconcelos, Mario; Afonso, Americo; Figueiral, Helena; Zita, Raquel	2011
Mesenchymal stem cell transplantation to promote bone healing.	Gao, Chan; Seuntjens, Jan; Kaufman, Gabriel N.; Tran-Khanh, Nicolas; Butler, Alison; Li, Ailian; Wang, Huifen; Buschmann, Michael D.; Harvey, Edward J.; Henderson, Janet E.	2012
Evaluation of implants coated with recombinant human bone morphogenetic protein-2 and vacuum-dried using the critical-size supraalveolar peri-implant defect model in dogs.	Decker, John F.; Lee, Jaebum; Cortella, Carlo Alberto; Polimeni, Giuseppe; Rohrer, Michael D.; Wozney, John M.; Hall, Jan; Susin, Cristiano; Wikesjo, Ulf M. E.	2010
Mandibular defect reconstruction using three-dimensional polycaprolactone scaffold in combination with platelet-rich plasma and recombinant human bone morphogenetic protein-2: de novo synthesis of bone in a single case.	Schuckert, Karl-Heinz; Jopp, Stefan; Teoh, Swee-Hin	2009
Alveolar ridge augmentation using implants coated with recombinant human bone morphogenetic protein-7 (rhBMP-7/rhOP-1): histological observations.	Susin, Cristiano; Qahash, Mohammed; Polimeni, Giuseppe; Lu, Ping Hung; Prasad, Hari S.; Rohrer, Michael D.; Hall, Jan; Wikesjo, Ulf M. E.	2010
Utility of intracerebral theta burst electrical stimulation to attenuate interhemispheric inhibition and to promote motor recovery after cortical injury in an animal model.	Barry, Melissa D.; Boddington, Laura J.; Igelstrom, Kajsa M.; Gray, Jason P.; Shemmell, Jon; Tseng, Kuei Y.; Oorschot, Dorothy E.; Reynolds, John N. J.	2014
Levels of osteoclastogenesis-related factors in the peri-implant crevicular fluid and clinical parameters of immediately loaded implants in patients with osteopenia: a short-term report	Onuma, , T, Aquiar, K, Duarte, PM, Feres, M, Giro, G, Coelho, P, Cassoni, A; Shibli, JA	2015
Enhancing osteoblast functions on biofilm-contaminated titanium alloy by concentration-dependent use of methylene blue-mediated antimicrobial photodynamic therapy	Huang T.-C.; Chen C.-J.; Chen C.-C.; Ding S.-J.	2019
Adjunctive application of antimicrobial photodynamic therapy in the prevention of medication-related osteonecrosis of the jaw following dentoalveolar surgery: A case series	Poli P.P.; Souza F.Á.; Ferrario S.; Maiorana C.	2019
Urinothorax causing massive left side pleural effusion in a young lady; case report and literature review	Bahjat A.S.; Sadeeq K.J.; Tahir A.M.S.; Mohammed A.A.	2019
Interventions for peri-implantitis and their effects on further bone loss: A retrospective analysis of a registry-based cohort	Karlsson K.; Derks J.; Håkansson J.; Wennström J.L.; Petzold M.; Berglundh T.	2019
Current status of dental implants survival and peri-implant bone loss in patients with uncontrolled type-2 diabetes mellitus	Chambrone L.; Palma L.F.	2019
Immediate versus delayed loading of mandibular implant-retained overdentures: A 60-month follow-up of a randomized clinical trial	Salman A.; Thacker S.; Rubin S.; Dhingra A.; Ioannidou E.; Schincaglia G.P.	2019
Implant treatment of two failing or missing central incisors in the aesthetic region: a treatment protocol and 1-year prospective study	van Nimwegen W.G.; Raghoebar G.M.; Vissink A.; Meijer H.J.A.	2019
Influence of negative pressure wound therapy on peri-prosthetic tissue vascularization and inflammation around porous titanium percutaneous devices	Pawar D.R.L.; Jeyapalina S.; Hafer K.; Bachus K.N.	2019
Clinical and organizational management of cardiac implantable electronic device replacements: An Italian Survey promoted by AIAC (Italian Association of Arrhythmology and Cardiac Pacing)	Palmisano P.; Ziacchi M.; Belotti G.; Rapacciuolo A.; Santini L.; Stabile G.; Zoni Berisso M.; De Ponti R.; Landolina M.; Ricci R.P.; Boriani G.	2019
Prevalence of peri-implant diseases among an Italian population of patients with metabolic syndrome: a cross-sectional study	Papi P.; Murro B.D.; Pranno N.; Bisogni V.; Saracino V.; Letizia C.; Polimeni A.; Pompa G.	2019
Discomfort/pain due to periodontal and peri-implant probing with/without platform switching	Parvini P.; Saminsky M.; Stanner J.; Klum M.; Nickles K.; Eickholz P.	2019
Comparison of yeasts species in the subgingival oral biofilm of type 2-diabetic and non-diabetic individuals with peri-implantitis	Alsahhaf A.; Al-Aali K.A.; Alshagroud R.S.; Alshiddi I.F.; Alrahlah A.; Abduljabbar T.; Javed F.; Vohra F.	2019

Ultrasound-triggered antibiotic release from PEEK clips to prevent spinal fusion infection: Initial evaluations	Delaney L.J.; MacDonald D.; Leung J.; Fitzgerald K.; Sevit A.M.; Eisenbrey J.R.; Patel N.; Forsberg F.; Kepler C.K.; Fang T.; Kurtz S.M.; Hickok N.J.	2019
Comparison of biofilm removal using glycine air polishing versus sodium bicarbonate air polishing or hand instrumentation on full-arch fixed implant rehabilitations: a split-mouth study	Menini M.; Setti P.; Dellepiane E.; Zunino P.; Pera P.; Pesce P.	2019
Long-Term Survival and Peri-Implant Health of Titanium Implants with Zirconia Abutments: A Systematic Review and Meta-Analysis	Cao Y.; Yu C.; Wu Y.; Li L.; Li C.	2019
Retrospective cohort study of 4,591 dental implants: Analysis of risk indicators for bone loss and prevalence of peri-implant mucositis and peri-implantitis	French D.; Grandin H.M.; Ofec R.	2019
Clinical evaluation of three surgical modalities in the treatment of peri-implantitis: A randomized controlled clinical trial	Toma S.; Brecx M.C.; Lasserre J.F.	2019
Antimicrobial efficacy of copper-doped titanium surfaces for dental implants	Astasov-Frauenhoffer M.; Koegel S.; Waltimo T.; Zimmermann A.; Walker C.; Hauser-Gerspach I.; Jung C.	2019
Tapered Versus Cylindrical Implant: Which Shape Inflicts Less Pain After Dental Implant Surgery? A Clinical Trial	Samieirad S.; Mianbandi V.; Shiezadeh F.; Hosseini-Abrishami M.; Tohidi E.	2019
Biological width establishment around dental implants is influenced by abutment height irrespective of vertical mucosal thickness: A cluster randomized controlled trial	Spinato S.; Stacchi C.; Lombardi T.; Bernardello F.; Messina M.; Zaffe D.	2019
Development of a novel nanotextured titanium implant. An experimental study in rats	Pelegrine A.A.; Moy P.K.; Moshaverinia A.; Escada A.L.A.; Calvo-Guirado J.; Claro A.P.R.A.	2019
Stud vs Bar Attachments for Maxillary Four-Implant-Supported Overdentures: 3- to 9-year Results from a Retrospective Study	Lian M.; Zhao K.; Wang F.; Huang W.; Zhang X.; Wu Y.	2019
Immediate dental implants placed into infected sites present a higher risk of failure than immediate dental implants placed into non-infected sites: Systematic review and meta-analysis	de Oliveira-Neto O.-B.; Lemos C.-A.-A.; Barbosa F.-T.; de Sousa-Rodrigues C.-F.; Camello de Lima F.-J.	2019
Implants in the Posterior Maxilla: Open Sinus Lift Versus Conventional Implant Placement. A Systematic Review	Romero-Millán J J.; Aizcorbe-Vicente J.; Peñarrocha-Diago M.; Galindo-Moreno P.; Canullo L.; Peñarrocha-Oltra D.	2019
Influence of abutment height and implant depth position on interproximal peri-implant bone in sites with thin mucosa: A 1-year randomized clinical trial	Pico A.; Martín-Lancharro P.; Caneiro L.; Nóvoa L.; Batalla P.; Blanco J.	2019
The influence of bone loss on peri-implant bending strain under overdentures	Pham N.Q.; Gonda T.; Takahashi T.; Maeda Y.	2019
Comparison of BALP, CTX-I, and IL-4 levels around miniscrew implants during orthodontic tooth movement between two different amounts of force	Cesur M.G.; Ozturk V.O.; Afacan B.; Sirin F.B.; Alkan A.; Ozer T.	2019
Mechanical Thrombectomy Using Retrievable Stents in Pediatric Acute Ischemic Stroke	Bhatti A.; Huded V.; Vyas D.; Mushtaq M.; Kekatpure M.; Hiremath S.; Sapare A.; Aggarwal R.	2019
Platelets and osteointegration during experimental augmentation of bone defects	Gilev M.V.; Antropova I.P.; Volokitina E.A.; Bazarny V.V.	2019
The effect of supportive care in preventing peri-implant diseases and implant loss: A Systematic review and Meta-analysis	Lin C.-Y.; Chen Z.; Pan W.-L.; Wang H.-L.	2019
Role of Dental Implant Superstructure in Patients with Severe Periodontal Disease	Ito T.; Nishimura K.; Ogasawara R.; Furuya Y.; Yajima Y.	2019
Patients who are obese probably have worse peri-implant outcomes than those who are not, but there is not sufficient evidence that the implant survival is different	Brignardello-Petersen R.	2019
Clinical, radiographic, and restorative peri-implant measurements of narrow and standard diameter implants in obese and nonobese patients: A 3-year retrospective follow-up study	Alshiddi I.F.; Alsahhaf A.; Alshagroud R.S.; Al-Aali K.A.; Vohra F.; Abduljabbar T.	2019
Implant treatment in immunosuppressed renal transplant patients: A prospective case-controlled study	Hernández G.; Paredes V.; López-Pintor R.M.; de Andrés A.; de Vicente J.C.; Sanz M.	2019
Improvement of quality of life with implant-supported mandibular overdentures and the effect of implant type and surgical procedure on bone and soft tissue stability: A three-year prospective split-mouth trial	Doornewaard R.; Glibert M.; Matthys C.; Vervaeke S.; Bronkhorst E.; De Bruyn H.	2019
Healing differences in narrow diameter implants submitted to immediate and conventional loading in mandibular overdentures: A randomized clinical trial	Bielemann A.M.; Marcello-Machado R.M.; Schuster A.J.; Chagas Júnior O.L.; Del Bel Cury A.A.; Faot F.	2019
Regeneration of alveolar ridge defects. Consensus report of group 4 of the 15th European Workshop on Periodontology on Bone Regeneration	Jepsen S.; Schwarz F.; Cordaro L.; Derks J.; Hämmerle C.H.F.; Heitz-Mayfield L.J.; Hernández-Alfaro F.; Meijer H.J.A.; Naenni N.; Ortiz-Vigón A.; Pjetursson B.; Raghoebar G.M.; Renvert S.; Rocchietta I.; Roccuzzo M.; Sanz-Sánchez I.; Simion M.; Tomasi C.; Trombelli L.; Urban I.	2019
Prediction of titanium implant success by analysis of microRNA expression in peri-implant tissue. A 5-year follow-up study	Menini M.; Pesce P.; Baldi D.; Vargas G.C.; Pera P.; Izzotti A.	2019
Discrepancy between patient satisfaction and biologic complication rate in patients rehabilitated with overdentures and not participating in a structured maintenance program after 7 to 12 years of loading	Romandini M.; Cordaro M.; Donno S.; Cordaro L.	2019
Application of periodontal ligament-derived multipotent mesenchymal Stromal cell sheets for periodontal regeneration	Onizuka S.; Iwata T.	2019
Influence of implant neck and abutment characteristics on peri-implant tissue health and stability. Oral reconstruction foundation consensus report	Schwarz F.; Messias A.; Sanz-Sánchez I.; Carrillo de Albornoz A.; Nicolau P.; Taylor T.; Beuer F.; Schär A.; Sader R.; Guerra F.; Sanz M.	2019
What Affects Postoperative Sinusitis and Implant Failure after Dental Implant: A Meta-analysis	Kim J.S.; Choi S.M.; Yoon J.H.; Lee E.J.; Yoon J.; Kwon S.H.; Yeo C.D.; Ryu J.S.; Lee J.H.; You Y.S.; Kim S.G.; Lee M.H.; Han B.H.	2019

The effect of five mechanical instrumentation protocols on implant surface topography and roughness: A scanning electron microscope and confocal laser scanning microscope analysis	Cha J.-K.; Paeng K.; Jung U.-W.; Choi S.-H.; Sanz M.; Sanz-Martín I.	2019
Dental Implants With a Platform-Switched Morse Taper Connection and an Osteo Growth Induction Surface	Ghensi P.; Tonetto G.; Soldini C.; Bettio E.; Mortellaro C.; Soldini C.	2019
Accuracy of low dose imaging protocols of CBCT in assessment of peri-implant bone defects	Aboelmaaty W.; Morgan N.; Abdelfadil E.; Ashmawy M.	2019
Treatment of Resorbed Mandibles with Titanium Plate and Immediate Implant-Supported Prosthesis - Case Series	Fabris V.; Reginato V.F.; Smaniotto C.; Bacchi A.; Consani R.L.X.	2019
Long-term effectiveness of maxillary sinus floor augmentation: A systematic review and meta-analysis	Raghoebar G.M.; Onclin P.; Boven G.C.; Vissink A.; Meijer H.J.A.	2019
The effect of different occlusal contact situations on peri-implant bone stress - A contact finite element analysis of indirect axial loading	Brune A.; Stiesch M.; Eisenburger M.; Greuling A.	2019
Effectiveness and clinical performance of early implant placement for the replacement of single teeth in anterior areas: A systematic review	Graziani F.; Chappuis V.; Molina A.; Lazarin R.; Schmid E.; Chen S.; Salvi G.E.	2019
Challenges of the interface of oral medicine and periodontology: Some lessons for the future?	Porter S.; Johnson N.W.; Fedele S.	2019
Use of Porous Implants for the Prosthetic Rehabilitation of Fibula Free Flap Reconstructed Patients	Brauner E.; Di Carlo S.; Ciolfi A.; Pompa G.; Jamshir S.; De Angelis F.; Della Monaca M.; Valentini V.	2019
Left atrial appendage closure with WATCHMAN in Asian patients: 2year outcomes from the WASP registry	Phillips K.P.; Santoso T.; Sanders P.; Alison J.; Chan J.L.K.; Pak H.-N.; Chandavimol M.; Stein K.M.; Gordon N.; Razali O.B.	2019
REAL-WORLD PRACTICE PATTERNS IN THE ERA OF RECTAL INDOMETHACIN FOR PROPHYLAXIS AGAINST POST-ERCP PANCREATITIS: A LARGE POPULATION-BASED STUDY	Smith Z.L.; Elmunzer B.J.; Cooper G.S.; Chak A.	2019
Biological & technical outcomes of restored implants after maxillary sinus augmentation - results at 1 year loading	Alayan J.; Ivanovski S.	2019
In the Dental Implant Era - Why We Still Bother Saving Teeth?	Clark D.; Levin L.	2019
Neurogenic Inflammation in Periimplant and Periodontal Disease: A Case-Control Split-Mouth Study	Sert S.; Sakallioğlu U.; Lütfioğlu M.; Aydoğdu A.; Acarel E.; Günaydın M.	2019
Short-term performance of implant-supported restorations fitted in general dental practice: A retrospective study	Klotz A.-L.; Ott L.; Krisam J.; Schmitz S.; Seyidaliyeva A.; Rammelsberg P.; Zenthöfer A.	2019
Intra-oral single site comparisons of periodontal and peri-implant microbiota in health and disease	Yu X.-L.; Chan Y.; Zhuang L.; Lai H.-C.; Lang N.P.; Leung W.K.; Watt R.M.	2019
Veneered zirconia abutments cemented on non-original titanium bases: 1-year results of a prospective case series	Asgeirsson A.G.; Sailer I.; Gamper F.; Jung R.E.; Hämmerle C.H.F.; Thoma D.S.	2019
Systemic sclerostin antibody treatment increases osseointegration and biomechanical competence of zoledronic-acid-coated dental implants in a rat osteoporosis model	Korn P.; Kramer I.; Schlottig F.; Tödtman N.; Eckelt U.; Bürki A.; Ferguson S.J.; Kautz A.; Schnabelrauch M.; Range U.; Kneissel M.; Stadlinger B.	2019
Safety of Accelerated Recovery on a Cardiology Ward and Early Discharge Following Minimalist TAVR in the Catheterization Laboratory: The Vancouver Accelerated Recovery Clinical Pathway	Sathananthan J.; Webb J.G.; Polderman J.; Achtem L.; Hensey M.; Murdoch D.; Moss R.; Shook A.; Bruce S.; Blanke P.; Bancroft C.; Andrews H.; Leipsic J.; Wood D.; Lauck S.	2019
Keratinized mucosa width is associated with severity of peri-implant mucositis. A cross-sectional study	Grischke J.; Karch A.; Wenzlaff A.; Foitzik M.M.; Stiesch M.; Eberhard J.	2019
Prevalence and risk indicators of peri-implantitis in a sample of university-based dental patients in Italy: A cross-sectional study	Vignoletti F.; Di Domenico G.L.; Di Martino M.; Montero E.; de Sanctis M.	2019
Analyses of clinical and osteoimmunological parameters on keratinized mucosa around dental implants	Sukuroglu E.; Baltacioglu E.	2019
Epstein-Barr Virus–Positive Mucocutaneous Ulcer Mimicking Peri-Implantitis in a Patient With Systemic Lupus Erythematosus	Takahama A.; de Lima H.G.; Ito F.A.; Ribeiro-Silva A.; León J.E.	2019
Implantoplasty associated with the regenerative treatment of peri-implantitis	Austoni C.; Azzola F.; Cavalli N.; Morandi P.; Francetti L.	2019
Re-osseointegration following reconstructive surgical therapy of experimental peri-implantitis. A pre-clinical in vivo study	Almohandes A.; Carcuac O.; Abrahamsson I.; Lund H.; Berglundh T.	2019
Biologically guided implant therapy: A diagnostic and therapeutic strategy of conservation and preservation based on periodontal staging and grading	Greenwell H.; Wang H.-L.; Kornman K.S.; Tonetti M.S.	2019
Peri implant microflora in health and disease	Ramanathan S.; Prakash P.S.G.; Appukuttan D.; Subramanian S.; Victor D.J.	2019
The W.A.I.O.T. definition of high-grade and low-grade peri-prosthetic joint infection	Romanò C.L.; Khawashki H.A.; Benzakour T.; Bozhkova S.; del Sel H.; Hafez M.; Johari A.; Lob G.; Sharma H.K.; Tsuchiya H.; Drago L.	2019
Peri-implant marginal bone loss rate pre- and post-loading: An exploratory analysis of associated factors	Acharya A.; Leung M.C.T.; Ng K.T.; Fan M.H.M.; Fokas G.; Mattheos N.	2019
Outcome of early dental implant placement versus other dental implant placement protocols: A systematic review and meta-analysis	Bassir S.H.; El Kholy K.; Chen C.-Y.; Lee K.H.; Intini G.	2019
Clinical and radiological evaluation of delayed and early loading of single-tooth implant placement: A 6-month, prospective, randomized, follow-up clinical study	Sekar S.; Suthanthiran T.; Thangavelu A.; Karupannan S.; Prem F.; Rajendran D.	2019
Significance of keratinized mucosa/gingiva on peri-implant and adjacent periodontal conditions in erratic maintenance compliers	Monje A.; Blasi G.	2019
Flapless surgery and peri-implant late-stage inflammation and healing	Schultze-Mosgau S.	2019
Orthognathic surgery deteriorates the osseointegration of dental implants: a propensity-matched multicenter cohort study	Lee H.; Kim J.W.; Lim H.K.; Byun S.H.; Choi Y.J.; Lee U.L.	2019
Immediate implant placement in infected sites: a systematic review	Samara E.; Wates E.; Ria B.	2019

Success and survival rates of dental implants inserted in homologous bone grafts	Rosa M.M.; Leite G.B.; dos Santos L.E.; Vivacqua C.P.P.; Motta S.H.G.	2019
Mandibular Overdentures Supported by Two vs Four Endosteal Implants: A Retrospective Cohort Study	Mahgoli H.; Asadi S.; Hajmahmoudi M.; Moharrami M.; Arshad M.	2019
Breast implant–associated anaplastic large-cell lymphoma	Collins K.; DiGiuseppe J.A.	2019
Assessment of peri implant bone loss in basal implant: a prospective clinical study	Debnath S.C.; Memmi L.; Vishnuraj R.; Bhagawati A.	2019
Pre-procedure anxiety: The origin of pain during implantation of cardiac implantable electronic devices	Senior J.; Liu B.; Jani A.; Apostolakis M.; Goyal D.; Taylor R.; Foster W.; Brewster N.; Wilson D.G.	2019
DEVICE RELATED THROMBOSIS AFTER WATCHMAN IMPLANTATION: IMPACT OF POST-PROCEDURAL ANTICOAGULATION	Apte N.M.; Pierpoline M.; Patel A.; Longmore L.; Dar T.A.; Robinson A.; Chandler J.; Khan A.; Timins A.; Gupta K.; Wilson K.; Jipescu D.; Aftabizadeh S.; Johnson D.; Osorio J.; Earnest M.; Aryana A.; Ellis C.R.; Thambidorai S.; Reddy Y.V.	2019
The Effects of Ultraviolet Light-Emitting Diodes with Different Wavelengths on Periodontopathic Bacteria In Vitro	Aung N.; Aoki A.; Takeuchi Y.; Hiratsuka K.; Katagiri S.; Kong S.; Shujaa Addin A.; Meinzer W.; Sumi Y.; Izumi Y.	2019
Short-term complications and their risk factors after breast surgery utilizing the NSQIP database	Marumoto A.; Woodruff S.; Wong L.; Yee L.	2019
Laparoscopic artificial urinary sphincter implantation in women with stress urinary incontinence: update on 13 years' experience in a single centre	Bracchitta D.; Costa P.; Borojeni S.; Ménard J.; Bryckaert P.-E.; Mandron É.	2019
Mandibular implant-supported overdentures in (frail) elderly: A prospective study with 20-year follow-up	Bakker M.H.; Vissink A.; Meijer H.J.A.; Raghoebar G.M.; Visser A.	2019
Association of Statin Use and Mortality After Transcatheter Aortic Valve Replacement	Peri-Okonny P.A.; Liu Y.; Malaisrie S.C.; Huded C.P.; Kapadia S.; Thourani V.H.; Kodali S.K.; Webb J.; McAndrew T.C.; Leon M.B.; Cohen D.J.; Arnold S.V.	2019
Network meta-analysis of survival rate and complications in implant-supported single crowns with different abutment materials	Hu M.; Chen J.; Pei X.; Han J.; Wang J.	2019
Complications and survival rates of 55 metal-ceramic implant-supported fixed complete-arch prostheses: A cohort study with mean 5-year follow-up	Papaspyridakos P.; Bordin T.B.; Natto Z.S.; El-Rafie K.; Pagni S.E.; Chochlidakis K.; Ercoli C.; Weber H.-P.	2019
Computer-assisted, template-guided immediate implant placement and loading in the mandible: a case report	Spielau T.; Hauschild U.; Katsoulis J.	2019
Peri-Implantitis and Peri-Implant Mucositis Case Definitions in Dental Research: A Systematic Assessment	Natto Z.S.; Almeganni N.; Alnakeeb E.; Bukhari Z.; Jan R.; Iacono V.J.	2019
Clinical and radiographic peri-implant health status around narrow diameter implant-supported single and splinted crowns	Al-Aali K.A.; ArRejaie A.S.; Alrahlah A.; AlFawaz Y.F.; Abduljabbar T.; Vohra F.	2019
Prospective controlled clinical study investigating long-term clinical parameters, patient satisfaction, and microbial contamination of zirconia implants	Lorenz J.; Giulini N.; Hölscher W.; Schwiertz A.; Schwarz F.; Sader R.	2019
Peri-implantitis prevalence, incidence rate, and risk factors: A study of electronic health records at a U.S. dental school	Kordbacheh Changi K.; Finkelstein J.; Papapanou P.N.	2019
Longitudinal study on risk indicators for peri-implantitis using survival-time analysis	Mameno T.; Wada M.; Onodera Y.; Fujita D.; Sato H.; Ikebe K.	2019
Double Full-Arch Fixed Implant-Supported Prostheses: Outcomes and Complications after a Mean Follow-Up of 5 Years	Papaspyridakos P.; Bordin T.B.; Natto Z.S.; Kim Y.-J.; El-Rafie K.; Tsigarida A.; Chochlidakis K.; Weber H.-P.	2019
Experimental peri-implant mucositis around titanium and zirconia implants in comparison to a natural tooth: part 2—clinical and microbiological parameters	Clever K.; Schlegel K.A.; Kniha H.; Conrads G.; Rink L.; Modabber A.; Hölzle F.; Kniha K.	2019
Diagnosis of Periimplant Disease	Hirooka H.; Renvert S.	2019
Comparison of three different types of implant-supported fixed dental prostheses: A long-term retrospective study of clinical outcomes and cost-effectiveness	Ravidà A.; Tattan M.; Askar H.; Barootchi S.; Tavelli L.; Wang H.-L.	2019
Treatment of buccal soft tissue dehiscence around single implant: 5-year results from a prospective study	Roccuzzo M.; Dalmasso P.; Pittoni D.; Roccuzzo A.	2019
Clinical and radiographic peri-implant outcomes of short dental implants placed in posterior jaws of patients with treated generalized aggressive periodontitis: A 3-year follow-up study	Akram Z.; Vohra F.; Sheikh S.A.; Albaijan R.; Bukhari I.A.; Hussain M.	2019
Oral and peri-implant conditions of patients at risk for medication-related osteonecrosis of the jaw (MRONJ)	Renata V.; Sabrina X.; Antonio F.; Antonio C.; Leonardo T.	2019
Soft-Tissue Conditions Around Dental Implants: A Literature Review	Lin G.-H.; Madi I.M.	2019
Survival of adjacent-dental-implants in prediabetic and systemically healthy subjects at 5-years follow-up	Alrabiah M.; Alrahlah A.; Al-Hamdan R.S.; Al-Aali K.A.; Labban N.; Abduljabbar T.	2019
Comparison of clinical peri-implant indices and crestal bone levels around narrow and regular diameter implants placed in diabetic and non-diabetic patients: A 3-year follow-up study	Al-Shibani N.; Al-Aali K.A.; Al-Hamdan R.S.; Alrabiah M.; Basunbul G.; Abduljabbar T.	2019
Systemic Risk Factors for the Development of Periimplant Diseases	Kumar P.S.	2019
Crestal Bone Loss Around Submerged and Non-Submerged Dental Implants in Individuals with Type-2 Diabetes Mellitus: A 7-Year Prospective Clinical Study	Al Zahrani S.; Al Mutairi A.A.	2019
Soft-Tissue Management as Part of the Surgical Treatment of Periimplantitis: A Narrative Review	Sculean A.; Romanos G.; Schwarz F.; Ramanauskaite A.; Leander K.P.; Khoury F.; Koo K.-T.; Cosgarea R.	2019
Squamous cell carcinoma around a dental implant: A case report and literature review	Malthiéry E.; De Boutray M.; Koren C.; Albouy J.-P.; Torres J.-H.; Fauroux M.-A.	2019
Treatment Planning for Periimplant Mucositis and Periimplantitis	Polyzois I.	2019

Short implants (5-8 mm) vs. long implants in augmented bone and their impact on peri-implant bone in maxilla and/or mandible: Systematic review	Amine M.; Guelzim Y.; Benfaida S.; Bennani A.; Andoh A.	2019
Biomechanical analysis of 4 types of short dental implants in a resorbed mandible	Lee H.; Park S.; Noh G.	2019
Marginal bone response of submerged and non-submerged osteoconductive alkali-etched implants in thick and thin biotypes: A 2-year clinical follow-up study	Novák Z.; Strnad J.; Nesvadba R.; Kamprle J.; Strnad Z.	2019
Maintenance of Implant Patients: A Narrative Review	Kelekis-Cholakis A.; Rothney J.	2019
Implant vs abutment level connection in implant supported screw-retained fixed partial dentures with cobalt-chrome framework: 1-year interim results of a randomized clinical study	Toia M.; Stocchero M.; Becktor J.P.; Chrcanovic B.; Wennerberg A.	2019
Implant Surface Decontamination by Surgical Treatment of Periimplantitis: A Literature Review	Koo K.-T.; Khoury F.; Leander Keeve P.; Schwarz F.; Ramanauskaite A.; Sculean A.; Romanos G.	2019
Immediate implant placement following 1-year treatment with oral versus intravenous bisphosphonates: a histomorphometric canine study on peri-implant bone	Khojasteh A.; Dehghan M.M.; Nazeman P.	2019
MicroRNA-29a-3p regulates osteoblast differentiation and peri-implant osseointegration in a rat model of hyperlipidemia by modulating Frizzled 4 expression	Liu F.; Wang Z.-F.; Liu F.-F.; Xu J.-Z.; Liu Q.; Lan J.	2019
The Effect of Left Ventricular Assist Devices (LVAD) on Pulmonary Hypertension: A Single Center Review	Tremblay P.L.; Stewart S.A.; Baskett R.J.	2019
Opportunities for mechanistic studies of stroke in collaboration with the Cardiothoracic Surgical Trials Network	Kirkwood K.A.; Messe S.R.; Taylor D.A.	2019
Comparison of treatment of medium-sized renal stones in terms of cost effectiveness	Soares D.; Berman I.; Paul E.; Shahbaz S.; McCahy P.	2019
Presence of Candida species in the subgingival oral biofilm of patients with peri-implantitis	Alrabiah M.; Alshagroud R.S.; Alsahhaf A.; Almojaly S.A.; Abduljabbar T.; Javed F.	2019
A 5-year longitudinal cohort study on crown to implant ratio effect on marginal bone level in single implants	Ramaglia L.; Di Spirito F.; Sirignano M.; La Rocca M.; Esposito U.; Sbordone L.	2019
Longitudinal assessment of clinical and radiographic periimplant status around narrow and regular diameter implants placed in cigarette-smokers and nonsmokers	Alasqah M.N.; Alfawaz Y.F.; Aldahiyan N.; Vohra F.; Alotaibi B.M.; Abduljabbar T.	2019
Impaired osseointegration of dental implants associated with orthognathic surgery: Possible regional acceleratory phenomenon	Kim J.-W.; Kim H.-Y.; Kim S.-Y.; Kim S.-J.	2019
Soft tissue status and crestal bone loss around conventionally-loaded dental implants placed in cigarette- and waterpipe (narghile) smokers: 8-years' follow-up results	Alahmari F.; Javed F.; Ahmed Z.U.; Romanos G.E.; Al-Kheraif A.A.	2019
Hydroxyl radicals generated by hydrogen peroxide photolysis recondition biofilm-contaminated titanium surfaces for subsequent osteoblastic cell proliferation	Nakamura K.; Shirato M.; Tenkumo T.; Kanno T.; Westerlund A.; Örtengren U.; Sasaki K.; Niwano Y.	2019
Clinical performance of tooth- or implant-supported veneered zirconia single crowns: 42-month results	Cantner F.; Cacaci C.; Mücke T.; Randelzhofer P.; Hajtó J.; Beuer F.	2019
Interproximal contact loss in a retrospective cross-sectional study of 4325 implants: Distribution and incidence and the effect on bone loss and peri-implant soft tissue	French D.; Naito M.; Linke B.	2019
Influence on peri-implant bone loss of different fixed partial dentures retained on 2 implants	Solá-Ruíz M.F.; Martí-Martí B.; Rech-Ortega C.; Fernández-Estevan L.; Agustín-Panadero R.	2019
HOSTILE TRICUSPID VALVE OVERCOME BY SELECTING THE MIDDLE CARDIAC VEIN: AN ALTERNATIVE FOR THE STANDARD RIGHT VENTRICULAR LEAD PLACEMENT OF BIVENTRICULAR ICD	Mando R.; Akoegbe G.; Shah R.; Malas H.; Maskoun W.	2019
Prescribing trends of systemic antibiotics by periodontists in Australia	Ong A.; Kim J.; Loo S.; Quaranta A.; Rincon A J.C.	2019
Oculo-facio-cardio-dental syndrome: A case report about a rare pathological condition	Martinho J.; Ferreira H.; Paulo S.; Paula A.; Marto C.-M.; Carrilho E.; Marques-Ferreira M.	2019
The amount of keratinized mucosa may not influence peri-implant health in compliant patients: A retrospective 5-year analysis	Lim H.-C.; Wiedemeier D.B.; Hämmerle C.H.F.; Thoma D.S.	2019
Screw-retained monolithic zirconia vs. cemented porcelain-fused-to-metal implant crowns: a prospective randomized clinical trial in split-mouth design	Weigl P.; Saarepera K.; Hinrikus K.; Wu Y.; Trimpou G.; Lorenz J.	2019
Ten years follow-up retrospective study on implant survival rates and prevalence of peri-implantitis in implant-supported full-arch rehabilitations	Francetti L.; Cavalli N.; Taschieri S.; Corbella S.	2019
Does Apico-Coronal Implant Position Influence Peri-Implant Marginal Bone Loss? A 36-Month Follow-Up Randomized Clinical Trial	Pellicer-Chover H.; Peñarrocha-Diago M.; Aloy-Prosper A.; Canullo L.; Peñarrocha-Diago M.; Peñarrocha-Oltra D.	2019
Ethical and legal aspects in periodontal disease diagnosis and therapy	Voinescu I.; Ferechide D.; Cristache C.M.; Burlibasa L.; Burlibasa M.	2019
Clinical evaluation of full-arch screw-retained implant-supported fixed prostheses and full-arch telescopic-retained implant-supported fixed prostheses: A 5-12 year follow-up retrospective study	Mori G.; Oda Y.; Sakamoto K.; Ito T.; Yajima Y.	2019
Peri-implant soft tissue status and crestal bone levels around adjacent implants placed in cigarette smokers and never smokers: Five-year follow-up results	ArRejaie A.S.; Al-Aali K.A.; Alrabiah M.; Mokeem S.A.; Vohra F.; Abduljabbar T.	2019
Clinical and patient-reported outcomes of implants placed in autogenous bone grafts and implants placed in native bone: A case-control study with a follow-up of 5-16 years	Thoma D.S.; Maggetti I.; Waller T.; Hämmerle C.H.F.; Jung R.E.	2019

The Role of Implant-Tooth Distance on Marginal Bone Levels and Esthetics	Wang T.; De Kok I.J.; Zhong S.; Vo C.; Mendonça G.; Nares S.; Cooper L.F.	2019
Retrospective analysis of porous tantalum trabecular metal-enhanced titanium dental implants	Edelmann A.R.; Patel D.; Allen R.K.; Gibson C.J.; Best A.M.; Bencharit S.	2019
A randomized, controlled, clinical study on a new titanium oxide abutment surface for improved healing and soft tissue health	Hall J.; Neilands J.; Davies J.R.; Ekestubbe A.; Friberg B.	2019
Bone Regeneration of Canine Peri-implant Defects Using Cell Sheets of Adipose-Derived Mesenchymal Stem Cells and Platelet-Rich Fibrin Membranes	Ding L.; Tang S.; Liang P.; Wang C.; Zhou P.-F.; Zheng L.	2019
Topographic characterization of multispecies biofilms growing on dental implant surfaces: An in vitro model	Bermejo P.; Sánchez M.C.; Llama-Palacios A.; Figuero E.; Herrera D.; Sanz M.	2019
Biomechanical aspects of reinforced implant overdentures: A systematic review	Gibreel M.F.; Khalifa A.; Said M.M.; Mahanna F.; El-Amier N.; Närhi T.O.; Perea-Lowery L.; Vallittu P.K.	2019
Rare diseases with periodontal manifestations	Hanisch M.; Hoffmann T.; Bohner L.; Hanisch L.; Benz K.; Kleinheinz J.; Jackowski J.	2019
Basic structural features of a cardiac telemedicine center for patients with heart failure and implanted devices, cardiac arrhythmias, and increased risk of sudden cardiac death: Recommendations of the working group 33 Telemonitoring of the German Cardiac Society	Helms T.M.; Stockburger M.; Köhler F.; Leonhardt V.; Müller A.; Rybak K.; Sack S.; Schwab J.O.; Störk S.; Zippel-Schultz B.; Perings C.A.	2019
Mid-term Results of a Novel Dedicated Venous Stent for the Treatment of Chronic Thoracic Central Vein Obstruction of Benign Aetiology	Tan G.M.; Chi K.W.K.; Yan B.P.Y.	2019
Impact of morbid obesity (BMI > 40 kg/m2) on complication rate and outcome following minimally invasive transforaminal lumbar interbody fusion (MIS TLIF)	Krüger M.T.; Naseri Y.; Hohenhaus M.; Hubbe U.; Scholz C.; Klingler J.-H.	2019
Bariatric surgery for severe type 2 diabetes-really a lost cause?	McGlone E.R.; Carey I.; Leonard C.; Velickovic V.; Le Roux C.; Batterham R.; Small P.; Khan O.	2019
Paclitaxel-Eluting Stents in the Superficial Femoral Artery at 12-Month Follow-Up Are Associated With Persistent Inflammation	Shaar M.; Lendel V.; Hoyt T.; Marmagkiolis K.; Ates I.; Mego D.; Paixao A.; Park J.; Mcelroy A.; Dijkstra J.; Avci R.; Milner T.; Feldman M.; Cilingiroglu M.	2019
Dental Implant Rehabilitation After Jaw Reconstruction Assisted by Virtual Surgical Planning	Zhu H.; Zhang L.; Cai Z.; Shan X.	2019
Oral hygiene maintenance of locator attachments implant overdentures in edentulous population: A longitudinal study	Liu X.Q.; Chen Q.W.; Feng H.L.; Wang B.; Qu J.; Sun Z.; Heng M.D.; Pan S.X.	2019
Research progress of dental implant in patients with oral lichen planus	Fu L.; A L.; Li C.Y.; Zhou Y.M.	2019
Clinical indices and local levels of inflammatory biomarkers in per-implant health of obese and nonobese individuals	Alasqah M.N.; Al-Shibani N.; Al-Aali K.A.; Qutub O.A.; Abduljabbar T.; Akram Z.	2019
Evaluation of 0.2% delmopinol mouth rinse for prevention of peri-implant mucositis and peri-implantitis: A randomized controlled canine study	Levin L.; Barbu H.; Kurgan S.; Comăneanu R.M.; Referendaru D.; Lorean A.	2019
Prevalence of peri-implant disease and risk indicators in a Japanese population with at least 3 years in function-A multicentre retrospective study	Wada M.; Mameno T.; Onodera Y.; Matsuda H.; Daimon K.; Ikebe K.	2019
Implant health and factors affecting peri-implant marginal bone alteration for implants placed in staged maxillary sinus augmentation: A 5-year prospective study	Krennmair S.; Hunger S.; Forstner T.; Malek M.; Krennmair G.; Stimmelmayr M.	2019
Use of TiBrush for surface decontamination at peri-implantitis sites in dogs: Radiographic and histological outcomes	Viganò P.; Apaza Alccayhuaman K.A.; Sakuma S.; Amari Y.; Bengazi F.; Botticelli D.	2019
Understanding peri-implantitis as a plaque-associated and site-specific entity: On the local predisposing factors	Monje A.; Insua A.; Wang H.-L.	2019
A peri-implant disease risk score for patients with dental implants: Validation and the influence of the interval between maintenance appointments	De Araújo Nobre M.; Salvado F.; Nogueira P.; Rocha E.; Ilg P.; Maló P.	2019
Influence of restorative margins position on one-stage laser-microgrooved implants-supported single screwed crowns: A clinical, biochemical, and microbiological analysis	Guarnieri R.; Di Nardo D.; Gaimari G.; Miccoli G.; Testarelli L.	2019
TREATMENT OF CROHN'S DISEASE AND ULCERATIVE COLITIS WITH PROTON PUMP INHIBITORS: EFFECT ON BONE LOSS AT DENTAL IMPLANTS	Ursomanno B.L.; Cohen R.E.; Levine M.J.; Yerke L.M.	2019
On inflammation-immunological balance theory-A critical apprehension of disease concepts around implants: Mucositis and marginal bone loss may represent normal conditions and not necessarily a state of disease	Albrektsson T.; Jemt T.; Mölne J.; Tengvall P.; Wennerberg A.	2019
Effects of commensal biofilms on a peri-implant mucosa in a three dimensional model	Mikolai C.; Ingendoh-Tsakmakidis A.; Winkel A.; Kommerein N.; Falk C.S.; Rossi A.; Walles H.; Stiesch M.	2019
Clinical and radiographic peri-implant variables around short dental implants in type 2 diabetic, prediabetic, and non-diabetic patients	Mokeem S.; Alfadda S.A.; Al-Shibani N.; Alrabiah M.; Al-Hamdan R.S.; Vohra F.; Abduljabbar T.	2019
In Vitro Evaluation of Cell Compatibility of Dental Cements Used with Titanium Implant Components	Marvin J.C.; Gallegos S.I.; Parsaei S.; Rodrigues D.C.	2019
Research on implants and osseointegration	Guglielmotti M.B.; Olmedo D.G.; Cabrini R.L.	2019
Extra-Sinus Zygomatic Implants to Avoid Chronic Sinusitis and Prosthetic Arch Malposition: 12 Years of Experience	Aleksandrowicz P.; Kusa-Podkańska M.; Grabowska K.; Kotuła L.; Szkatuła-Łupina A.; Wysokińska-Miszczuk J.	2019
Treatment of peri-implant recession with a screw-retained, interim implant restoration: A clinical report	Schoenbaum T.R.; Solnit G.S.; Alawie S.; Sadowsky S.J.	2019
Periodic Assessment of Peri-implant Tissue Changes: Imperative for Implant Success	Rahman S.A.; Muhammad H.; Haque S.; Alam M.K.	2019

Clinical Success of Dental Implants Placed in Posterior Mandible Augmented With Interpositional Block Graft: 3-Year Results From a Prospective Cohort Clinical Study	Marconcini S.; Covani U.; Giammarinaro E.; Velasco-Ortega E.; De Santis D.; Alfonsi F.; Barone A.	2019
Evaluation of Immediate Implants Augmented with Autogenous Bone/Melatonin Composite Graft in the Esthetic Zone: A Randomized Controlled Trial	Hazzaa H.H.A.; El-Kilani N.S.; Elsayed S.A.-E.; Abd El Massieh P.M.	2019
Relationship Between Crestal Bone Levels and Crown-to-Implant Ratio of Ultra-Short Implants With a Microrough Surface: A Prospective Study With 48 Months of Follow-Up	Malchiodi L.; Giacomazzi E.; Cucchi A.; Ricciotti G.; Caricasulo R.; Bertossi D.; Gherlone E.	2019
Short vs. Standard Laser-Microgrooved Implants Supporting Single and Splinted Crowns: A Prospective Study with 3 Years Follow-Up	Guarnieri R.; Di Nardo D.; Gaimari G.; Miccoli G.; Testarelli L.	2019
The Conometric Concept: A Two-Year Follow-Up of Fixed Partial CEREC Restorations Supported By Cone-In-Cone Abutments	Degidi M.; Nardi D.; Sighinolfi G.; Degidi D.; Piattelli A.	2019
Influence of pH on osteoclasts treated with zoledronate and alendronate	Manzano-Moreno F.J.; Ramos-Torrecillas J.; de Luna-Bertos E.; Illescas-Montes R.; Arnett T.R.; Ruiz C.; García-Martínez O.	2019
Innovative biomaterials for bone regrowth	Iaquinta M.R.; Mazzoni E.; Manfrini M.; D'Agostino A.; Trevisiol L.; Nocini R.; Trombelli L.; Barbanti-Brodano G.; Martini F.; Tognon M.	2019
PEEK materials as an alternative to titanium in dental implants: A systematic review	Mishra S.; Chowdhary R.	2019
Effect of Implant Position on Clinical and Radiographic Outcomes of Locator-Retained Mandibular Overdentures: A 1-Year Prospective Study	ELsyad M.A.; Maryod W.H.; Mostafa A.Z.	2019
Maintenance therapy for teeth and implants	Mombelli A.	2019
Changes in pulmonary artery pressure before and after left ventricular assist device implantation in patients utilizing remote haemodynamic monitoring	Kilic A.; Katz J.N.; Joseph S.M.; Brisco-Bacik M.A.; Uriel N.; Lima B.; Agarwal R.; Bharmi R.; Farrar D.J.; Lee S.	2019
NCCT and CTA-based imaging protocol for endovascular treatment selection in late presenting or wake-up strokes	Santos T.; Carvalho A.; Cunha A.A.; Rodrigues M.; Gregório T.; Paredes L.; Costa H.; Roriz J.M.; Pinho J.; Veloso M.; Castro S.; Barros P.; Ribeiro M.	2019
Intraluminal Thrombi in the Cervico-Cephalic Arteries	Singh R.-J.; Chakraborty D.; Dey S.; Ganesh A.; Al Sultan A.S.; Eesa M.; Wong J.H.; Goyal M.; Hill M.D.; Menon B.K.	2019
Novel bioactive and therapeutic dental polymeric materials to inhibit periodontal pathogens and biofilms	Chi M.; Qi M.; Lan A.; Wang P.; Weir M.D.; Melo M.A.; Sun X.; Dong B.; Li C.; Wu J.; Wang L.; Xu H.H.K.	2019
Early postoperative mortality similar between cemented and uncemented hip arthroplasty: a register study based on Finnish national data	Ekman E.; Palomäki A.; Laaksonen I.; Peltola M.; Häkkinen U.; Mäkelä K.	2019
An evaluation of the relationship between peri-implant soft tissue biotype and the severity of peri-implantitis: A cross-sectional study	Isler S.C.; Uraz A.; Kaymaz O.; Cetiner D.	2019
Peri-implant Mucosal Tissues and Inflammation: Clinical implications	Fiorellini J.P.; Luan K.W.; Chang Y.-C.; Minjoon Kim D.; Sarmiento H.L.	2019
Influence of insertion torque on clinical and biological outcomes before and after loading of mandibular implant-retained overdentures in atrophic edentulous mandibles	Faot F.; Bielemann A.M.; Schuster A.J.; Marcello-Machado R.M.; Del Bel Cury A.A.; Nascimento G.G.; Chagas-Junior O.-L.L.	2019
The effects of annual maintenance on peri-implant health in patients rehabilitated with overdentures: A retrospective cohort study	Roman-Torres C.V.G.; Pasquinelli F.; Pimentel A.C.; de Melo M.P.; Rego R.O.; Sendyk W.R.	2019
Midfacial tissue assessment of the effect of amount of keratinized mucosa on immediate temporarization of fresh socket implants: 8-year follow-up	Crespi R.; Capparé P.; Crespi G.; Gastaldi G.; Romanos G.E.; Gherlone E.	2019
Etiology and measurement of peri-implant crestal bone loss (CBL)	Naveau A.; Shinmyouzu K.; Moore C.; Avivi-Arber L.; Jokerst J.; Koka S.	2019
Nonsurgical Management of Peri-implant Bone Loss Induced by Residual Cement: Retrospective Analysis of Six Cases	De Martinis Terra E.; Berardini M.; Trisi P.	2019
Er:YAG and diode lasers in treatment of peri-implantitis–A case report	Nikitas A.	2019
Graftless sinus floor augmentation with an internal-port implant: Long-term experience	Nahlieli O.; Boiangiu A.; Abramson A.; Aba M.; Nahlieli D.D.; Srouji S.	2019
Reuterinos® as adjuvant for peri-implant treatment: A pilot study	Lauritano D.; Carinci F.; Palmieri A.; Cura F.; Caruso S.; Candotto V.	2019
Factors contributing to peri-implantitis - A practitioner's perspective	Hemani K.; Ganapathy D.; Ramanathan V.	2019
Is there an association between overweight/obesity and dental implant complications? A systematic review and meta-analysis	Monteiro J.L.G.C.; Pellizzer E.P.; Araújo Lemos C.A.; de Moraes S.L.D.; do Egito Vasconcelos B.C.	2019
Undesirable course of an oral implant rehabilitation in a patient with a long history of bulimia nervosa: Case report and review of the literature	Turhani D.; Ohlmeier K.H.; Sutter W.; Kielbassa A.M.	2019
Three-year prospective randomized comparative assessment of anterior maxillary single implants with different abutment interfaces	Cooper L.F.; Reside G.; Stanford C.; Barwacz C.; Feine J.; Nader S.A.; Scheyer T.; McGuire M.	2019
Evaluation of implant success in patients with dental aplasia	Attia S.; Schaper E.; Schaaf H.; Pons-Kühnemann J.; Schlenz M.A.; Streckbein P.; Böttger S.; Howaldt H.-P.; Wilbrand J.-F.; Sartori M.	2019
Peri-implant Infection Concomitant with a Flare-up Episode of Chronic Periodontitis: An unusual regeneration following treatment and a 5-year follow-up	Tawil G.; Tawil P.	2019
Periodontal bacterial supernatants modify differentiation, migration and inflammatory cytokine expression in human periodontal ligament stem cells	Ramenzoni L.L.; Russo G.; Moccia M.D.; Attin T.; Schmidlin P.R.	2019
Implant placement for patients with cleft lip and palate: A clinical report and guidelines for treatment	Pucciarelli M.G.R.; Lopes A.C.O.; Lopes J.F.S.; Soares S.	2019

Relationship between primary/mechanical and secondary/biological implant stability	Monje A.; Ravidà A.; Wang H.-L.; Helms J.A.; Brunski J.B.	2019
A double-crown concept for restorations in patients with generalized aggressive periodontitis: Two case reports with 22- and 25-year follow-ups	Mengel R.; Peleska B.	2019
Risk factors associated with short dental implant success: a long-term retrospective evaluation of patients followed up for up to 9 years	Hasanoglu Erbasar G.N.; Hocaoğlu T.P.; Erbasar R.C.	2019
Impact of soft tissue grafts to reduce peri-implant alterations after immediate implant placement and provisionalization in compromised sockets	Frizzera F.; Moreno De Freitas R.; Muñoz-Chávez O.F.; Cabral G.; Shibli J.A.; Marcantonio E.	2019
Clinical and radiographic evaluation of early loaded maxillary anterior implants: Three-year follow-up	Kahramanoglu E.; Aslan Y.U.; Özkan Y.; Özkan Y.	2019
Onlay bone grafts from iliac crest: a retrospective analysis	Sethi A.; Kaus T.; Cawood J.I.; Plaha H.; Boscoe M.; Sochor P.	2019
Esthetic and functional rehabilitation of bilateral congenital absence of maxillary lateral incisors: Minimally invasive surgical and prosthetic approach	Ruales-Carrera E.; Engler M.L.P.D.; Vaz P.; Özcan M.; Volpato C.A.M.	2019
Titanium dental implants with different collar design and surface modifications: A systematic review on survival rates and marginal bone levels	Messias A.; Nicolau P.; Guerra F.	2019
Immediate dental implant placement in calvarial bone grafts to rehabilitate the severely resorbed edentulous maxilla: A prospective pilot study	Putters T.F.; Raghoebar G.M.; Klein-Nulend J.; Vissink A.; Schortinghuis J.	2019
Comparison of Three Different Types of Two-Implant-Supported Magnetic Attachments on the Stress Distribution in Edentulous Mandible	Hu F.; Gong Y.; Bian Z.; Zhang X.; Xu B.; Zhang J.; Shi X.; Yu Y.; Song L.	2019
Long-term results of endosseous implants as retention elements of orbital epitheses, reconstruction techniques, and aftercare following radical tumor resection	Baum S.H.; Klein M.; Mohr C.; Weischer T.	2019
Breast implant-associated anaplastic large cell lymphoma: Clinical and imaging findings at a large US cancer center	Dashevsky B.Z.; Gallagher K.M.; Grabenstetter A.; Cordeiro P.G.; Dogan A.; Morris E.A.; Horwitz S.M.; Sutton E.J.	2019
Safe hydration to prevent contrast-induced acute kidney injury and worsening heart failure in patients with renal insufficiency and heart failure undergoing coronary angiography or percutaneous coronary intervention	Bei W.-J.; Wang K.; Li H.-L.; Guo X.-S.; Guo W.; Abuduaini T.; Chen S.-Q.; Islam S.M.S.; Chen P.-Y.; Chen J.-Y.; Liu Y.; Tan N.	2019
Plate-on-plate technique for treating peri-implant fractures of distal femoral locking plate: a retrospective study of 11 patients	Ma C.-H.; Chiu Y.-C.; Wu C.-H.; Tsai K.-L.; Wen T.-K.; Jou I.-M.; Tu Y.-K.	2019
Resveratrol reverses the negative effect of smoking on peri-implant repair in the tibia of rats	Ribeiro F.V.; Pimentel S.P.; Corrêa M.G.; Bortoli J.P.; Messora M.R.; Casati M.Z.	2019
A novel perspective for predicting gingival biotype via dentopapillary measurements on study models in the Saudi population: Cross-sectional study	Nagate R.R.; Tikare S.; Chaturvedi S.; AlQahtani N.A.; Kader M.A.; Gokhale S.T.	2019
Is the time to revision surgery after peri-prosthetic fracture of the knee associated with increased rates of post-operative complications?	Boddapati V.; Held M.B.; Lee N.J.; Geller J.A.; Cooper H.J.; Shah R.P.	2019
Totally endoscopic ventricular septal defect repair using bilateral femoral arterial cannulation in an 8-year-old girl	Dang H.Q.; Le H.T.	2019
Safety, efficacy, and removability of a fully covered multi-hole metal stent in a swine model of hilar biliary stricture: A feasibility study	Park J.-S.; Jeong S.; Kobayashi M.; Lee D.H.	2019
Feasibility and safety of left atrial appendage closure using the lambre device in patients with nonvalvular atrial fibrillation with or without prior catheter ablation: A single centre experience	Feng X.-F.; Zhang P.-P.; Sun J.; Wang Q.-S.; Li Y.-G.	2019
Saphenous vein graft sacrifice following native vessel PCI is safe and associated with favourable longer-term outcomes	Wilson S.J.; Hanratty C.G.; Spence M.S.; Owens C.G.; Rigger J.; Spratt J.C.; Walsh S.J.	2019
Second generation drug-eluting stents for endovascular treatment of ostial vertebral artery stenosis: A single center experience	Ortega-Gutierrez S.; Lopez G.V.; Edgell R.C.; Mendez A.A.; Dandapat S.; Roa J.A.; Zevallos C.B.; Holcombe A.L.; Hasan D.; Derdeyn C.P.; Rossen J.; Samaniego E.A.	2019
Percutaneous Left Atrial Appendage Closure: Single Centre Experience using Three Different Devices	Iwai S.; Worthley S.; Sanders P.; Young G.	2019
Left atrial appendage closure. Monocentric registry of the first 100 cases: Indications, peri-operative anticoagulation strategies, complications and follow-up	Roux J.; Granier M.; Massin F.; Cade S.; Pasquié J.L.	2019
Estimation of the diagnostic accuracy of clinical parameters for monitoring peri-implantitis progression: An experimental canine study	Monje A.; Insua A.; Rakic M.; Nart J.; Moyano-Cuevas J.L.; Wang H.-L.	2018
Could the biomarker levels in saliva help distinguish between healthy implants and implants with peri-implant disease? A systematic review	Gomes A.M.; Douglas-de-Oliveira D.W.; Oliveira Costa F.	2018
A two-piece dental implant comprising a replaceable thin titanium sleeve may be a new approach to re-osseointegration following peri-implant disease	Liat C.; Roni S.; Adir G.; Gavriel C.	2018
Survival rates of hybrid rough surface implants and their alveolar bone level alterations	Lee C.-T.; Tran D.; Jeng M.-D.; Shen Y.-T.	2018
Periodontitis as a risk factor for peri-implantitis: Systematic review and meta-analysis of observational studies	Ferreira S.D.; Martins C.C.; Amaral S.A.; Vieira T.R.; Albuquerque B.N.; Cota L.O.M.; Esteves Lima R.P.; Costa F.O.	2018
Impact of jaw location on clinical and radiological status of dental implants placed in cigarette-smokers and never-smokers: 5-year follow-up results	Al-Aali K.A.; Alrabiah M.; Al-Hamdan R.S.; Al-Hamoudi N.; Aldahian N.; Abduljabbar T.	2018
Peri-implant inflammation and marginal bone level changes around dental implants in relation to proximity with and bone level of adjacent teeth	Ng K.T.; Fan M.; Leung M.C.; Fokas G.; Mattheos N.	2018

Randomized controlled clinical study of veneered zirconia abutments for single implant crowns: Clinical, histological, and microbiological outcomes	Thoma D.S.; Sailer I.; Mühlemann S.; Gil A.; Jung R.E.; Hämmerle C.H.F.	2018
Long-term follow-up of single crowns supported by short, moderately rough implants-A prospective 10-year cohort study	Rossi F.; Lang N.P.; Ricci E.; Ferraioli L.; Baldi N.; Botticelli D.	2018
Comparing 4-mm dental implants to longer implants placed in augmented bones in the atrophic posterior mandibles: One-year results of a randomized controlled trial	Rokn A.R.; Monzavi A.; Panjnoush M.; Hashemi H.M.; Kharazifard M.J.; Bitaraf T.	2018
Orthognathic surgery Deteriorates the osseointegration of dental implants: A propensity-matched multicentre cohort study	Kim J.-W.; Lee H.; Lim H.-K.; Kim J.-W.; Byun S.-H.; Choi Y.-J.; Lee U.-L.	2018
Single tooth implants in the esthetic zone following a two-stage all flapless approach: A retrospective analysis	Hingsammer L.; Pommer B.; Fürhauser R.; Mailath-Pokorny G.; Haas R.; Busenlechner D.	2018
Antibiofilm peptides against biofilms on titanium and hydroxyapatite surfaces	Wang D.; Haapasalo M.; Gao Y.; Ma J.; Shen Y.	2018
Early Healing of Immediate Implants Connected With Two Types of Healing Abutments: A Prospective Cohort Study	Hu C.; Lin W.; Gong T.; Zuo Y.; Qu Y.; Man Y.	2018
Immediate Postextractive Implants With and Without Bone Graft: 3-year Follow-up Results From a Multicenter Controlled Randomized Trial	Mastrangelo F.; Gastaldi G.; Vinci R.; Troiano G.; Tettamanti L.; Gherlone E.; Lo Muzio L.	2018
Effect of Human Wnt10b Transgene Overexpression on Peri-Implant Osteogenesis in Ovariectomized Rats	Liu H.; Zhang N.; Liu Y.; Liu L.; Yin G.; En L.	2018
Efficacy of an ethyl alcohol gel in symptomatic disc herniation	Marcia S.; Bellini M.; Hirsch J.A.; Chandra R.V.; Piras E.; Marras M.; Sanna A.M.; Saba L.	2018
Rivaroxaban or vitamin-K antagonists following early endovascular thrombus removal and stent placement for acute iliofemoral deep vein thrombosis	Sebastian T.; Hakki L.O.; Spirk D.; Baumann F.A.; Périard D.; Banyai M.; Spescha R.S.; Kucher N.; Engelberger R.P.	2018
Mitral valve repair complicated by left circumflex coronary artery occlusion: An under-recognized but potentially deadly complication	Ahmad T.A.; Nudy M.; Pabst D.; Kozak M.	2018
Transcatheter valve-in-valve versus redo surgical aortic valve replacement for the treatment of degenerated bioprosthetic aortic valve: A systematic review and meta-analysis	Tam D.Y.; Vo T.X.; Wijeysundera H.C.; Dvir D.; Friedrich J.O.; Fremes S.E.	2018
Comparative Evaluation of Different Bone Markers in Peri-implant Crevicular Fluid of Immediate Loaded and Nonloaded Dental Implants	Haque M.I.U.; Sharma P.; Tiwari A.; Subhas S.; Rana M.; Kumar V.	2018
Quercitrin nanocoated implant surfaces reduce osteoclast activity in vitro and in vivo	Córdoba A.; Manzanaro-Moreno N.; Colom C.; Rønold H.J.; Lyngstadaas S.P.; Monjo M.; Ramis J.M.	2018
Prosthetic Complications Affecting Single-Tooth Morse-Taper Connection Implants	Mangano F.; Lucchina A.G.; Brucoli M.; Migliario M.; Mortellaro C.; Mangano C.	2018
Effect of curcumin on bone tissue in the diabetic rat: repair of peri-implant and critical-sized defects	Cirano F.R.; Pimentel S.P.; Casati M.Z.; Corrêa M.G.; Pino D.S.; Messora M.R.; Silva P.H.F.; Ribeiro F.V.	2018
Straight and tilted implants for supporting screw-retained full-arch dental prostheses in atrophic maxillae: A 2-year prospective study	Menéndez-Collar M.; Serrera-Figallo M.-A.; Hita-Iglesias P.; Castillo-Oyagüe R.; Casar-Espinosa J.-C.; Gutiérrez-Corrales A.; Gutiérrez-Perez J.-L.; Torres-Lagares D.	2018
Citation Network Analysis of Dental Implant Literature from 2007 to 2016	Yeung A.W.K.; Leung W.K.	2018
Survival Rate and Bone Resorption in Immediate Loading of Atrophic Maxillary Arches Using Normal and Long Implants: A Pilot Observational Study	Pesce P.; Pera F.; Bruno D.; Menini M.	2018
Zirconia dental implants: A clinical and radiographic evaluation	Rodriguez A.E.; Monzavi M.; Yokoyama C.L.; Nowzari H.	2018
Metal artifact reduction in virtual monoenergetic spectral dual-energy CT of patients with metallic orthopedic implants in the distal radius	Yoo H.J.; Hong S.H.; Chung B.M.; Moon S.J.; Choi J.-Y.; Chae H.D.; Chang M.-Y.	2018
Evaluating the primary stability of standard vs lateralised cementless femoral stems – A finite element study using a diverse patient cohort	Al-Dirini R.M.A.; Martelli S.; Huff D.; Zhang J.; Clement J.G.; Besier T.; Taylor M.	2018
Efficacy of topical tranexamic acid within a blood-saving programme for primary total hip arthroplasty: A pragmatic, open-label randomised study	Pérez-Jimeno N.; Muñoz M.; Mateo J.; Mayoral A.P.; Herrera A.	2018
Comparison of graded balloon atrial septostomy to continued medical therapy or novel interventional techniques in patients with advanced pulmonary arterial hypertension	Khan M.S.; Sreenivasan J.; Memon M.M.; Amin E.; Ochani R.K.; Shaikh A.; Fatima K.; Asmi N.; Krasuski R.	2018
MicroRNA-29a-3p enhances dental implant osseointegration of hyperlipidemic rats via suppressing dishevelled 2 and frizzled 4	Liu F.; Wang Z.; Liu F.; Xu J.; Liu Q.; Yin K.; Lan J.	2018
Group 4 ITI Consensus Report: Risks and biologic complications associated with implant dentistry	Heitz-Mayfield L.J.; Aaboe M.; Araujo M.; Carrión J.B.; Cavalcanti R.; Cionca N.; Cochran D.; Darby I.; Funakoshi E.; Gierthmuehlen P.C.; Hashim D.; Jahangiri L.; Kwon Y.; Lambert F.; Layton D.M.; Lorenzana E.R.; McKenna G.; Mombelli A.; Müller F.; Roccuzzo M.; Salvi G.E.; Schimmel M.; Srinivasan M.; Tomasi C.; Yeo A.	2018
The diagnosis of peri-implantitis: A systematic review on the predictive value of bleeding on probing	Hashim D.; Cionca N.; Combescure C.; Mombelli A.	2018
Diagnostic accuracy of the implant stability quotient in monitoring progressive peri-implant bone loss: An experimental study in dogs	Monje A.; Insua A.; Monje F.; Muñoz F.; Salvi G.E.; Buser D.; Chappuis V.	2018
How do peri-implant biologic parameters correspond with implant survival and peri-implantitis? A critical review	Doornewaard R.; Jacquet W.; Cosyn J.; De Bruyn H.	2018
Comparison of clinical and radiographic peri-implant parameters among obese and non-obese patients: A 5-year study	Alkhudhairy F.; Vohra F.; Al-Kheraif A.A.; Akram Z.	2018
Biological aspects: Summary and consensus statements of group 2. The 5th EAO Consensus Conference 2018	Sanz M.; Klinge B.; Alcoforado G.; Bienz S.P.; Cosyn J.; De Bruyn H.; Derks J.; Figuero E.; Gurzawska K.; Heitz-Mayfield L.; Jung R.E.; Ornekul T.; Sagado A.	2018
Clinical outcomes of peri-implantitis treatment and supportive care: A systematic review	Roccuzzo M.; Layton D.M.; Roccuzzo A.; Heitz-Mayfield L.J.	2018

Long-term clinical outcome of single implants inserted flaplessly or conventionally	Naeini E.N.; Dierens M.; Atashkadeh M.; De Bruyn H.	2018
Clinical and radiographic outcomes of a surgical reconstructive approach in the treatment of peri-implantitis lesions: A 5-year prospective case series	La Monaca G.; Pranno N.; Annibali S.; Cristalli M.P.; Polimeni A.	2018
Peri-implant diseases	Klinge B.; Klinge A.; Bertl K.; Stavropoulos A.	2018
Surgical treatment of peri-implantitis lesions with or without the use of a bone substitute-a randomized clinical trial	Renvert S.; Roos-Jansåker A.-M.; Persson G.R.	2018
Long-term biological complications of dental implants placed either in pristine or in augmented sites: A systematic review and meta-analysis	Salvi G.E.; Monje A.; Tomasi C.	2018
Long-term results of osseointegrated implant-based dental rehabilitation in oncology patients reconstructed with a fibula free flap	Pellegrino G.; Tarsitano A.; Ferri A.; Corinaldesi G.; Bianchi A.; Marchetti C.	2018
The Association Between Buccal Mucosa Thickness and Periimplant Bone Loss and Attachment Loss: A Cross-Sectional Study	Mailoa J.; Arnett M.; Chan H.-L.; George F.M.; Kaigler D.; Wang H.-L.	2018
Evaluation of peri-implant marginal tissues around tissue-level and bone-level implants in patients with a history of chronic periodontitis	Vianna T.T.; Taiete T.; Casarin R.C.V.; Giorgi M.C.C.; Aguiar F.H.B.; Silvério K.G.; Nociti Júnior F.H.; Sallum E.A.; Casati M.Z.	2018
What is the impact of titanium particles and biocorrosion on implant survival and complications? A critical review	Mombelli A.; Hashim D.; Cionca N.	2018
Impact of dental cement on the peri-implant biofilm-microbial comparison of two different cements in an in vivo observational study	Korsch M.; Marten S.-M.; Walther W.; Vital M.; Pieper D.H.; Dötsch A.	2018
Dental implants - are they better than natural teeth?	Pjetursson B.E.; Heimisdottir K.	2018
Biological effect of the abutment material on the stability of peri-implant marginal bone levels: A systematic review and meta-analysis	Sanz-Sánchez I.; Sanz-Martín I.; Carrillo de Albornoz A.; Figuero E.; Sanz M.	2018
Survival of dental implants placed in HIV-positive patients: a systematic review	Lemos C.A.A.; Verri F.R.; Cruz R.S.; Santiago Júnior J.F.; Faverani L.P.; Pellizzer E.P.	2018
A randomized controlled clinical trial assessing initial crestal bone remodeling of implants with a different surface roughness	Glibert M.; Matthys C.; Maat R.-J.; De Bruyn H.; Vervaeke S.	2018
Is there an effect of crown-to-implant ratio on implant treatment outcomes? A systematic review	Meijer H.J.A.; Boven C.; Delli K.; Raghoebar G.M.	2018
Implant rehabilitation of the edentulous jaws: Does tilting of posterior implants at an angle greater than 45° affect bone resorption and implant success?: A retrospective study	Malchiodi L.; Moro T.; Cattina D.P.; Cucchi A.; Ghensi P.; Nocini P.F.	2018
The prosthetic abutment height can affect marginal bone loss around dental implants	Lee B.-A.; Kim B.-H.; Kweon H.H.I.; Kim Y.-T.	2018
Sub-periosteal peri-implant augmented layer technique for horizontal bone augmentation at implant placement	Trombelli L.; Severi M.; Pramstraller M.; Farina R.	2018
Performance and outcome of zirconia dental implants in clinical studies: A meta-analysis	Roehling S.; Schlegel K.A.; Woelfler H.; Gahlert M.	2018
Histological and micro-CT analysis of peri-implant soft and hard tissue healing on implants with different healing abutments configurations	Souza A.B.; Alshihri A.; Kämmerer P.W.; Araújo M.G.; Gallucci G.O.	2018
Five-year cohort prospective study on single implants in the esthetic area restored using one-abutment/one-time prosthetic approach	Canullo L.; Omori Y.; Amari Y.; Iannello G.; Pesce P.	2018
The use of platelet-rich fibrin to enhance the outcomes of implant therapy: A systematic review	Strauss F.J.; Stähli A.; Gruber R.	2018
Retrospective radiographic observational study of 1692 Straumann tissue-level dental implants over 10 years. II. Marginal bone stability	Kang M.-H.; Jung U.-W.; Cho K.-S.; Lee J.-S.	2018
The influence of local delivery of bisphosphonate on osseointegration of dental implants	Khamis A.K.; Elsharkawy S.	2018
Biological and technical complications of tilted implants in comparison with straight implants supporting fixed dental prostheses. A systematic review and meta-analysis	Apaza Alccayhuaman K.A.; Soto-Peñaloza D.; Nakajima Y.; Papageorgiou S.N.; Botticelli D.; Lang N.P.	2018
Efficacy of carotid artery stenting on stroke prevention of octogenarians	Mohammadian R.; Sharifipour E.; Taheraghdam A.; Mansourizadeh R.; Altafi D.; Fattahzadeh G.; Sariaslani P.; Yousefshahi P.; Ebrahimzadeh K.; Vahedian M.; Golzari S.E.J.	2018
Extracting infected cardiac implantable electronic devices, a significant healthcare cost	Brough C.E.P.; Wright D.J.	2018
Eus-guided pancreatic drainage: A steep learning curve	Tyberg A.; Kedia P.; Tarnasky P.; Ali Khan M.; Novikov A.; Shah S.; Gaidhane M.; Ardengh J.C.; Kahaleh M.	2018
Upgrades from a previous device compared to de novo cardiac resynchronization therapy in the European Society of Cardiology CRT Survey II	Linde C.M.; Normand C.; Bogale N.; Auricchio A.; Sterlinski M.; Marinskis G.; Sticherling C.; Bulava A.; Pérez Ó.C.; Maass A.H.; Witte K.K.; Rekvava R.; Abdelali S.; Dickstein K.	2018
Impact of peri-procedural and technical factors and patient characteristics on revascularization and clinical outcome in dawn trial	Tekle W.; Hassan A.; Jadhav A.; Liebeskind D.; Jovin T.; Nogueira R.	2018
Transesophageal echocardiography and fluoroscopy for percutaneous closure of atrial septal defects: A comparative study	Xu W.; Li J.; Ye J.; Yu J.; Yu J.; Zhang Z.	2018
Resective Treatment of Peri-implantitis: Clinical and Radiographic Outcomes After 2 Years	Englezos E.; Cosyn J.; Koole S.; Jacquet W.; De Bruyn H.	2018
Surgical Alternatives for Treating Peri-implantitis	Sarmiento H.L.; Norton M.; Korostoff J.; Ko K.I.; Fiorellini J.P.	2018
Dense cancellous bone as evidenced by a high HU value is predictive of late implant failure: a preliminary study	Miyamoto I.; Takahashi T.; Tanaka T.; Hirayama B.; Tanaka K.; Yamazaki T.; Morimoto Y.; Yoshioka I.	2018
Microstructural Evaluation of Contaminated Implant Surface Treated by Laser, Photodynamic Therapy, and Chlorhexidine 2 percent	Saffarpour A.; Nozari A.; Fekrazad R.; Saffarpour A.; Heibati M.N.; Iranparvar K.	2018

Liquid-infused titanium as biofilm-repellent implant surface	Doll K.; Yang I.; Fadeeva E.; Schaeske J.; Kommerein N.; Szafrański S.P.; Greuling A.; Winkel A.; Chichkov B.N.; Stumpp N.S.; Stiesch M.	2018
Anaerobic bacteria in implants and homologous teeth 2-14 years after implantation	Savić I.; Bošnjak A.; Beader N.; Lovrić Ž.; Salihagić A.; Gašparac I.	2018
Sodium bicarbonate: A review and its uses in dentistry	Madeswaran S.; Jayachandran S.	2018
Confocal fluorescence imaging to evaluate the effect of antimicrobial photodynamic therapy depth on P. gingivalis and T. denticola biofilms	Rogers S.; Honma K.; Mang T.S.	2018
Influence of the abutment height and connection timing in early peri-implant marginal bone changes: A prospective randomized clinical trial	Borges T.; Leitão B.; Pereira M.; Carvalho Á.; Galindo-Moreno P.	2018
Morbidity following transcrestal and lateral sinus floor elevation: A randomized trial	Farina R.; Franceschetti G.; Travaglini D.; Consolo U.; Minenna L.; Schincaglia G.P.; Riccardi O.; Bandieri A.; Maietti E.; Trombelli L.	2018
Wound Healing in Dental Implant Surgery in Patients with or without Antibiotic Prophylaxis	Shinde S.V.; Chansoria S.; Limaye M.; Vijay M.; Bansal S.; Dhakne V.M.	2018
Peri-implant Bone Loss of Tissue-Level and Bone-Level Implants in the Esthetic Zone with Gingival Biotype Analysis	Wallner G.; Rieder D.; Wichmann M.G.; Heckmann S.M.	2018
Titanium implants and silent inflammation in jawbone—a critical interplay of dissolved titanium particles and cytokines TNF-α and RANTES/CCL5 on overall health?	Lechner J.; Noumbissi S.; von Baehr V.	2018
A Histologic and Histomorphometric Retrospective Analysis of the Outcomes of Ridge Preservation Using Anorganic Bovine Bone Minerals and a Nonresorbable Membrane	Min S.; Freire M.; Bakshallian N.; Wu I.; Zadeh H.H.	2018
Clinical and Radiographic Evaluation of Bar, Telescopic, and Locator Attachments for Implant-Stabilized Overdentures in Patients with Mandibular Atrophied Ridges: A Randomized Controlled Clinical Trial	ELsyad M.A.; Denewar B.A.; Elsaih E.A.	2018
Rituximab in idiopathic retroperitoneal fibrosis	Boyeva V.; Alabsi H.; Seidman M.; Paterson R.; Kur J.; Chang S.; Chen L.; Carruthers M.	2018
Pessary versus poise impressa® trial (PPIT) forthe management of stress urinary incontinence, a randomized cross-over trial	Elshatanoufy S.; Hicks B.; Estanol M.V.C.; Richardson D.A.; Atiemo H.; Luck A.M.	2018
Use of takayasu arteritis damage score (TADS) to measure damage in takayasu arteritis	Rajappa S.M.; Venkataraman K.	2018
The outcomes of ureterolysis during surgery for deep infiltrating endometriosis in stented patients	Smith D.; Mikhail M.; Fisher G.; Arumuham V.; Allen S.; Vashisht A.; Saridogan E.; Cutner A.	2018
Patients' perspectives on urethral bulk injection therapy and mid-urethral sling surgery for stress urinary incontinence	Casteleijn F.M.; Zwolsman S.E.; Kowalik C.R.; Roovers J.-P.P.W.R.	2018
Perioperative interventions in pelvic organ prolapse surgery	Haya N.; Feiner B.; Baessler K.; Christmann-Schmid C.; Maher C.	2018
Association of advanced glycation end products with peri-implant inflammation in prediabetes and type 2 diabetes mellitus patients	Alrabiah M.; Al-Aali K.A.; Al-Sowygh Z.H.; Binmahfooz A.M.; Mokeem S.A.; Abduljabbar T.	2018
The association between three attitude-related indexes of oral hygiene and secondary implant failures: A retrospective longitudinal study	Tecco S.; Grusovin M.G.; Sciara S.; Bova F.; Pantaleo G.; Capparé P.	2018
Ten-year follow-up of dental implants used for immediate loading in the edentulous mandible: A prospective clinical study	Windael S.; Vervaeke S.; Wijnen L.; Jacquet W.; De Bruyn H.; Collaert B.	2018
Influence of keratinized mucosa on peri-implant soft tissue health among patients with chronic periodontitis susceptibility	Qian J.-L.; Shu R.; Wang Y.-W.; Xie Y.-F.	2018
Implant survival rates and biologic complications with implant-supported fixed complete dental prostheses: A retrospective study with up to 12-year follow-up	Papaspyridakos P.; Barizan Bordin T.; Kim Y.-J.; DeFuria C.; Pagni S.E.; Chochlidakis K.; Rolim Teixeira E.; Weber H.-P.	2018
Surface property alterations and osteoblast attachment to contaminated titanium surfaces after different surface treatments: An in vitro study	Lee B.-S.; Shih K.-S.; Lai C.-H.; Takeuchi Y.; Chen Y.-W.	2018
Ultrasonography for noninvasive and real-time evaluation of peri-implant tissue dimensions	Chan H.-L.; Sinjab K.; Li J.; Chen Z.; Wang H.-L.; Kripfgans O.D.	2018
In vitro and in vivo studies of anti-bacterial copper-bearing titanium alloy for dental application	Liu R.; Tang Y.; Zeng L.; Zhao Y.; Ma Z.; Sun Z.; Xiang L.; Ren L.; Yang K.	2018
In Vivo Periodontium Formation Around Titanium Implants Using Periodontal Ligament Cell Sheet	Washio K.; Tsutsumi Y.; Tsumanuma Y.; Yano K.; Srithanyarat S.S.; Takagi R.; Ichinose S.; Meinzer W.; Yamato M.; Okano T.; Hanawa T.; Ishikawa I.	2018
Peri-implant soft tissue status and crestal bone levels around adjacent implants placed in patients with and without type-2 diabetes mellitus: 6 years follow-up results	Alasqah M.N.; Alrabiah M.; Al-Aali K.A.; Mokeem S.A.; Binmahfooz A.M.; ArRejaie A.S.; Abduljabbar T.	2018
One-year clinical outcomes of locking taper Equator attachments retaining mandibular overdentures to narrow diameter implants	Marcello-Machado R.M.; Faot F.; Schuster A.J.; Bielemann A.M.; Chagas Júnior O.L.; Del Bel Cury A.A.	2018
Apicoectomy and Scanning Electron Microscopy Analysis of an Implant Infected by Apical (Retrograde) Peri-implantitis: A Case Letter	Manfro R.; Garcia G.F.; Bortoluzzi M.C.; Fabris V.; Bacchi A.; Elias C.N.	2018
Rehabilitation of Postextractive Socket in the Premaxilla: A 12-Year Study on 27 Titanium Plasma Spray Resorbable Calcium Phosphate Coated Single Implants	Perrotti V.; Vozza I.; Tumedei M.; Pompa G.; Iaculli F.; Quaranta A.	2018
Survival of dental implants placed in vascularised fibula free flaps after jaw reconstruction	Attia S.; Wiltfang J.; Pons-Kühnemann J.; Wilbrand J.-F.; Streckbein P.; Kähling C.; Howaldt H.-P.; Schaaf H.	2018
The influence of implant-abutment connection to peri-implant bone loss: A systematic review and meta-analysis	Caricasulo R.; Malchiodi L.; Ghensi P.; Fantozzi G.; Cucchi A.	2018
Peri-implant tissue healing at implants with different designs and placement protocols: An experimental study in dogs	Oskarsson M.; Otsuki M.; Welander M.; Abrahamsson I.	2018
Influence of the Buccal Bone Crest Width on Peri-Implant Hard and Soft Tissues Dimensions: A Histomorphometric Study in Humans	Omori Y.; Iezzi G.; Perrotti V.; Piattelli A.; Ferri M.; Nakajima Y.; Botticelli D.	2018

Clinical evaluation of short implants in atrophic posterior region	Zhang J.; Jiang H.-H.; Shang J.; Xu S.; Liu Z.-H.	2018
The Conometric Concept: Definitive Fixed Lithium Disilicate Restorations Supported by Conical Abutments	Degidi M.; Nardi D.; Sighinolfi G.; Piattelli A.	2018
Adjunctive stent use during endovascular intervention to the femoropopliteal artery with drug coated balloons: Insights from the XLPAD registry	Kokkinidis D.G.; Jeon-Slaughter H.; Khalili H.; Brilakis E.S.; Shammas N.W.; Banerjee S.; Armstrong E.J.	2018
Laparoscopic sleeve gastrectomy in adolescents with or without syndromic obesity: two years follow-up	Iossa A.; De Peppo F.; Caccamo R.; Watkins B.M.; Abbatini F.; Ceriati E.; Silecchia G.	2018
Tricuspid valve repair at the time of mitral valve surgery is safe and reduces the incidence of late tricuspid regurgitation and reoperation: A meta-analysis	Tam D.; Tran A.; Friedrich J.O.; Mazine A.; Tang G.L.; Gaudino M.F.L.; Calafiore A.M.; Fremes S.E.	2018
Assessment of the effect of addition of 24 hours of oral tranexamic acid post-operatively to a single intraoperative intravenous dose of tranexamic acid on calculated blood loss following primary hip and knee arthroplasty (TRAC-24): A study protocol for a randomised controlled trial	Hill J.; Magill P.; Dorman A.; Hogg R.; Eggleton A.; Benson G.; McFarland M.; Murphy L.; Gardner E.; Bryce L.; Martin U.; Adams C.; Bell J.; Campbell C.; Agus A.; Phair G.; Molloy D.; Mockford B.; O'Hagan S.; Beverland D.	2018
Peri-implant Tissue Health and Bone Resorption in Patients with Immediately Loaded, Implant-Supported, Full-Arch Prostheses	Menini M.; Setti P.; Pera P.; Pera F.; Pesce P.	2018
"Burden of the Triumph:" Burden of peri-implantitis in Indian population - A mathematical model	Thavarajah R.; Sudharsan R.; Joseph I.; Elizabeth J.; Umadevi K.R.; Ranganathan K.	2018
Reosseointegration After Regenerative Surgical Therapy Using a Synthetic Bone Substitute for Peri-implantitis: Human Autopsy Study	Kim S.; Hu K.-S.; Jung U.-W.	2018
Classification Systems for Peri-implantitis: A Narrative Review with a Proposal of a New Evidence-Based Etiology Codification	Tallarico M.; Canullo L.; Wang H.-L.; Cochran D.L.; Meloni S.M.	2018
A 13- to 32-Year Retrospective Study of Bone Stability for Machined Dental Implants	Simion M.; Nevins M.; Rasperini G.; Tironi F.	2018
Impact of different placement depths on the crestal bone level of immediate versus delayed placed platform-switched implants	Madani E.; Smeets R.; Freiwald E.; Sanj M.S.; Jung O.; Grubeanu D.; Hanken H.; Henningsen A.	2018
Is Antimicrobial Photodynamic Therapy Effective for Microbial Load Reduction in Peri-implantitis Treatment? A Systematic Review and Meta-Analysis	Fraga R.S.; Antunes L.A.A.; Fontes K.B.F.D.C.; Küchler E.C.; Iorio N.L.P.P.; Antunes L.S.	2018
Dental implants placed in severely atrophic jaws reconstructed with autogenous calvarium, bovine bone mineral, and collagen membranes: A 3- to 19-year retrospective follow-up study	Chiapasco M.; Tommasato G.; Palombo D.; Scarnò D.; Zaniboni M.; Del Fabbro M.	2018
Five-Year Survival of Short Single-Tooth Implants (6 mm): A Randomized Controlled Clinical Trial	Naenni N.; Sahrmann P.; Schmidlin P.R.; Attin T.; Wiedemeier D.B.; Sapata V.; Hämmerle C.H.F.; Jung R.E.	2018
A Prospective Multicenter Study on Radiographic Crestal Bone Changes Around Dental Implants Placed at Crestal or Subcrestal Level: One-Year Findings	Gatti C.; Gatti F.; Silvestri M.; Mintrone F.; Rossi R.; Tridondani G.; Piacentini G.; Borrelli P.	2018
Impact of Implant Number on Mandibular Implant-Supported Profile Prostheses: A Systematic Review	Lima L.B.; de Freitas N.R.; Novais V.R.; Simamoto Júnior P.C.	2018
Intra- and inter-examiner agreement when assessing radiographic implant bone levels: Differences related to brightness, accuracy, participant demographics and implant characteristics	Walton T.R.; Layton D.M.	2018
Are Intraoral Radiographs Accurate in Determining the Peri-implant Marginal Bone Level?	Cassetta M.; Di Giorgio R.; Barbato E.	2018
In vitro antibacterial and cytotoxic activities of carvacrol and terpinen-4-ol against biofilm formation on titanium implant surfaces	Maquera-Huacho P.M.; Tonon C.C.; Correia M.F.; Francisconi R.S.; Bordini E.A.F.; Marcantonio É.; Spolidorio D.M.P.	2018
Omega-3 Polyunsaturated Fatty Acid Supplementation to Prevent Arteriovenous Fistula and Graft Failure: A Systematic Review and Meta-analysis of Randomized Controlled Trials	Viecelli A.K.; Irish A.B.; Polkinghorne K.R.; Hawley C.M.; Johnson D.W.; Mori T.A.; Pascoe E.M.; Strippoli G.F.M.; Lok C.E.; Palmer S.C.	2018
Clinical characteristics of peri-implant mucositis and peri-implantitis	Ramanauskaite A.; Becker K.; Schwarz F.	2018
A 5-year randomized clinical trial comparing minimally with moderately rough implants in patients with severe periodontitis	Raes M.; D'hondt R.; Teughels W.; Coucke W.; Quirynen M.	2018
Peri-implant conditions and levels of advanced glycation end products among patients with different glycemic control	Al-Sowygh Z.H.; Ghani S.M.A.; Sergis K.; Vohra F.; Akram Z.	2018
Influence of the width of keratinized tissue on the development and resolution of experimental peri-implant mucositis lesions in humans	Schwarz F.; Becker J.; Civale S.; Sahin D.; Iglhaut T.; Iglhaut G.	2018
A new classification scheme for periodontal and peri-implant diseases and conditions - Introduction and key changes from the 1999 classification	Caton J.G.; Armitage G.; Berglundh T.; Chapple I.L.C.; Jepsen S.; Kornman K.S.; Mealey B.L.; Papapanou P.N.; Sanz M.; Tonetti M.S.	2018
Peri-implant parameters, tumor necrosis factor-alpha, and interleukin-1 beta levels in vaping individuals	Al-Aali K.A.; Alrabiah M.; ArRejaie A.S.; Abduljabbar T.; Vohra F.; Akram Z.	2018
A retrospective study on the crestal bone loss associated with different implant surfaces in chronic periodontitis patients under maintenance	Gallego L.; Sicilia A.; Sicilia P.; Mallo C.; Cuesta S.; Sanz M.	2018
Is osteoporosis a risk factor for implant survival or failure?	Grisa A.; Veitz-Keenan A.	2018
Staging and grading of periodontitis: Framework and proposal of a new classification and case definition	Tonetti M.S.; Greenwell H.; Kornman K.S.	2018
Staging and grading of periodontitis: Framework and proposal of a new classification and case definition	Tonetti M.S.; Greenwell H.; Kornman K.S.	2018
Peri-implant bone loss clinical and radiographic evaluation around rough neck and microthread implants: a 5-year study	Calvo-Guirado J.L.; López-López P.J.; Pérez-Albacete Martínez C.; Javed F.; Granero-Marín J.M.; Maté Sánchez de Val J.E.; Ramírez Fernández M.P.	2018
Peri-implant squamous cell carcinoma	Ito K.; Takahashi K.; Eda T.; Kondoh T.; Goss A.	2018

Evaluation of peri-implant bone density changes in maxillary premolar region using computed tomography: A retrospective study	Aldaili M.; Ganji K.K.; Alanazi A.F.; Alam M.K.	2018
TiO_2/Ag-TiO_2 Nanohybrid Films are Cytocompatible with Primary Epithelial Cells of Human Origin: An In Vitro Study	Masa R.; Deák Á.; Braunitzer G.; Tóth Z.; Kopniczky J.; Pelsőczi-Kovács I.; Ungvári K.; Dékány I.; Turzó K.	2018
Effect of Various Laser Wavelengths on Temperature Changes During Periimplantitis Treatment: An in vitro Study	Monzavi A.; Fekrazad R.; Chinipardaz Z.; Shahabi S.; Behruzi R.; Chiniforush N.	2018
Surgery without papilla incision: tunneling flap procedures in plastic periodontal and implant surgery	Zuhr O.; Rebele S.F.; Cheung S.L.; Hürzeler M.B.	2018
Comparative evaluation of soft and hard tissue changes following endosseous implant placement using flap and flapless techniques in the posterior edentulous areas of the mandible-a randomized controlled trial	Kumar D.; Sivaram G.; Shivakumar B.; Kumar T.	2018
Fiber-Reinforced Resin Fixed Prostheses on 4 Short Implants in Severely Atrophic Maxillas: 1-Year Results of a Prospective Cohort Study	Wagner F.; Seemann R.; Marincola M.; Ewers R.	2018
Maxillary Sinus Floor Augmentation With Synthetic Bone Substitutes Compared With Other Grafting Materials: A Systematic Review and Meta-analysis	Starch-Jensen T.; Mordenfeld A.; Becktor J.P.; Jensen S.S.	2018
Complications of endovascular treatment for acute ischemic stroke: Prevention and management	Balami J.S.; White P.M.; McMeekin P.J.; Ford G.A.; Buchan A.M.	2018
Fluoroless single-step EUS-guided gallbladder drainage with cautery-enhanced lumen-apposing metal stent: A multicenter study	Mohamed A.M.; Garg R.; Barawi M.; Vyas N.; Sachdev M.; Diehl D.L.; Khara H.S.; Adler D.G.; Rustagi T.	2018
Risk Factors for Surgical Site Infection Following Lower Limb Arthroplasty: A Retrospective Cohort Analysis of 3932 Lower Limb Arthroplasty Procedures in a High Volume Arthroplasty Unit	Almustafa M.A.; Ewen A.M.; Deakin A.H.; Picard F.; Clarke J.V.; Mahmood F.F.	2018
Evaluation on clinical application of Florida probes in probing peri-implant soft tissue	Ding Z.; Wang W.; Jin D.; Yu G.; Xuan D.	2018
The effect of smoking on the outcome of dental implant treatment: A literature review	Baskaran K.; Nirosa T.; Jayashri P.	2018
Expression Levels of Semaphorins 3A, 3B, 4A, and 4D on Human Peri-implantitis	Bastos M.F.; de Franco L.; Garcia Tebar A.C.; Giro G.; Shibli J.A.	2018
Contributing factors for peri-implantitis in endosseous dental implants - A review	Hemani K.; Dhanraj M.; Jain A.R.	2018
Site-level risk predictors of peri-implantitis: A retrospective analysis	Kumar P.S.; Dabdoub S.M.; Hegde R.; Ranganathan N.; Mariotti A.	2018
Clinical outcomes of experimental gingivitis and peri-implant mucositis treatment with professionally administered plaque removal and photodynamic therapy	Zeza B.; Farina R.; Pilloni A.; Mongardini C.	2018
Clinical and Radiographic Performance of Rough Surfaced Implants Placed in the Atrophic Posterior Maxilla With Sinus Membrane Elevation Without Bone Grafting: A Prospective and Preliminary Study	Park S.; Yoon H.-J.	2018
The impact of different torques for the insertion of immediately loaded implants on the peri-implant levels of angiogenesis- and bone-related markers	Verrastro Neto A.; Andrade R.; Corrêa M.G.; Casarin R.C.V.; Casati M.Z.; Pimentel S.P.; Ribeiro F.V.; Cirano F.R.	2018
Marginal bone loss at implants with different surface characteristics - A 20-year follow-up of a randomized controlled clinical trial	Donati M.; Ekestubbe A.; Lindhe J.; Wennström J.L.	2018
Clinical and Radiographic Assessment of Three-Implant-Supported Fixed-Prosthesis Rehabilitation of the Edentulous Mandible: Immediate Versus Delayed Loading	Primo B.T.; Mezzari L.M.; da Fontoura Frasca L.C.; Linderman R.; Rivaldo E.G.	2018
Prevalence of metabolic syndrome in hypertensive patients affected by peri-implant diseases	Bisogni V.; Papi P.; Saracino V.; Di Murro B.; Penna D.; Concistrè A.; Petramala L.; Celi M.; Olmati F.; Iannucci G.; Pompa G.; Letizia C.	2018
Fixed, Fiber-Reinforced Resin Bridges on 5.0-mm Implants in Severely Atrophic Mandibles: Up to 5 Years' Follow-Up of a Prospective Cohort Study	Seemann R.; Wagner F.; Marincola M.; Ewers R.	2018
Adherence to best practice consensus guidelines for implant-based breast reconstruction: Results from the iBRA national practice questionnaire survey	Mylvaganam S.; Conroy E.J.; Williamson P.R.; Barnes N.L.P.; Cutress R.I.; Gardiner M.D.; Jain A.; Skillman J.M.; Thrush S.; Whisker L.J.; Blazeby J.M.; Potter S.; Holcombe C.; Barnes N.L.P.; Blazeby J.M.; Branford O.A.; Conroy E.J.; Cutress R.I.; Gardiner M.D.; Holcombe C.; Jain A.; McEvoy K.; Mills N.; Mylvaganam S.; Potter S.; Skillman J.M.; Teasdale E.M.; Thrush S.; Whisker L.J.; Williamson P.R.; Tang L.; Nguyen D.; Johnson R.; Muralikrishnan V.; Chopra S.; Reid A.; Benyon S.; Murphy C.; Soliman F.; Lefemine V.; Saha S.; Ogedegbe K.; Olyinka O.S.; Dicks J.R.; Manoloudakis N.; Conroy F.; Irwin G.; McIntosh S.; Michalakis I.; Hignett S.; Linforth R.; Rathinaezhil R.; Osman H.; Anesti K.; Griffiths M.; Jacklin R.; Waterworth A.; Foulkes R.; Davies E.; Bisarya K.; Allan A.; Leon-Villapalos J.; Mazari F.A.K.; Azmy I.; George S.; Fahmy F.S.; Hargreaves A.; Seward J.; Hignett S.; Henton J.; Collin T.; Irwin G.; Mallon P.; Turner J.; Sarakbi W.; Athanasiou I.; Rogers C.; Youssef M.; Graja T.; Huf S.; Deol H.; Brindle R.; Gawne S.; Egbeare D.; Dash I.; Galea M.; Laws S.; Tayeh S.; Parvanta L.; Down S.; Westbroek D.; Roberts J.W.; Massey J.; Turton P.; Achuthan R.; Fawzy M.; Dickson M.; Carmichael A.R.; Akingboye A.; James R.; Kirkpatrick K.; Nael E.; Vidya R.; Potter S.; Thorne A.; Rostom M.; Depasquale I.; Cawthorn S.J.; Gangamihardja T.; Joglekar S.; Smith J.; Halka A.; MacMillan D.; Clark S.; Pearce B.; Mansfield L.; King I.; Hazari A.; Smith B.; Volleamere A.J.; Egbeare D.; Ferguson D.; Barnes N.; Holcombe C.; Knight A.; MacNeill F.; Conway A.; Irvine T.;	2018

	Mylavaganam S.; Dunne N.; Kohlhardt S.; Hoo C.; Kirk S.; Hu J.; Ledwidge S.; Tang S.; Banerjee D.; Waheed S.; Voynov V.; Soumian S.; Henderson J.; Harvey J.; Robertson S.; Cutress R.I.; Mylvaganam S.; Waters R.; Carbone A.; Skillman J.; Farooq A.; Tafazal H.; Clarke D.; Cocker D.; Lai L.M.; Winter Beatty J.; Barkeji M.; Vinayagam R.; McEvoy K.; Mullan M.; Osborne C.; Baker E.; Piper J.	
Cost effective scoliosis surgery: Comparison between low cost indian implants and imported implants	Kanna R.M.; Shetty A.; Rajasekaran S.	2018
Clinical outcomes after fractional flow reserve-guided treatment of oncology patients	Donisan T.; Dayah T.; Balanescu D.V.; Karimzad K.; Boone D.L.; Lopez-Mattei J.; Kim P.Y.; Durand J.-B.; Yang E.H.; Herrmann J.; Marmagkiolis K.; Johnson N.P.; Iliescu C.	2018
A case series of trans-baffle lead extraction procedures involving patients with surgically corrected dextro-transposition of the great arteries (D-TGA)	Breuer A.; Barnett A.; Triphan J.; Adsit G.; Roberts M.J.; Leal M.A.	2018
Carotid revascularization and medical management for asymptomatic carotid stenosis: Crest-2 update	Howard G.; Meschia J.; Lal B.; Roubin G.; Brown R.; Barrett K.; Chaturvedi S.; Chimowitz M.; Demaerschalk B.; Howard V.; Huston J.; Lazar R.; Moore W.; Moy C.; Turan T.; Voeks J.; Brott T.	2018
Long-term in vivo evolution of high-purity Mg screw degradation — Local and systemic effects of Mg degradation products	Yu Y.; Lu H.; Sun J.	2018
Cost-benefit analysis of antibiofilm microbiological techniques for peri-prosthetic joint infection diagnosis	Romanò C.L.; Trentinaglia M.T.; De Vecchi E.; Logoluso N.; George D.A.; Morelli I.; Drago L.	2018
Long-term implant performance and patients' satisfaction in oligodontia	Filius M.A.P.; Vissink A.; Cune M.S.; Raghoebar G.M.; Visser A.	2018
Long-term outcome of surgical treatment of peri-implantitis. A 2-11-year retrospective study	Berglundh T.; Wennström J.L.; Lindhe J.	2018
Intraoral versus extraoral cementation of implant-supported single crowns: Clinical, biomarker, and microbiological comparisons	Kıran B.; Toman M.; Buduneli N.; Lappin D.F.; Toksavul S.; Nizam N.	2018
Re-establishment of Biocompatibility of the In Vitro Contaminated Titanium Surface Using Osteoconductive Powders With Air-Abrasive Treatment	Tastepe C.S.; Lin X.; Donnet M.; Doulabi B.Z.; Wismeijer D.; Liu Y.	2018
The Influence of Prosthesis Design on the Outcomes of Tooth Implants Immediately Placed and Loaded by Means of One-Piece Titanium Machined Restoration	Toti P.; Marconcini S.; Enrica G.; Pedretti G.; Barone A.; Covani U.	2018
Intrasinusal locking technique: a novel use of the ring block technique at sinus perforations for simultaneous implant placement	Sindel A.; Özarslan M.M.; Özalp Ö.	2018
Dental implants in patients with osteoporosis: a systematic review with meta-analysis	de Medeiros F.C.F.L.; Kudo G.A.H.; Leme B.G.; Saraiva P.P.; Verri F.R.; Honório H.M.; Pellizzer E.P.; Santiago Junior J.F.	2018
Evaluation of peri-implant bone levels and soft tissue dimensions around zirconia implants—a three-year follow-up study	Kniha K.; Schlegel K.A.; Kniha H.; Modabber A.; Hölzle F.; Kniha K.	2018
Short dental implants in patients with oral lichen planus: a long-term follow-up	Anitua E.; Piñas L.; Escuer-Artero V.; Fernández R.S.; Alkhraisat M.H.	2018
Periodontal prosthesis in contemporary dentistry	Liu C.-L.S.	2018
Indolent Rib Osteomyelitis Following Breast Implant Reconstruction: An Unusual Case and Review of the Literature	Watson L.; Dunn D.; Fraser-Kirk G.	2018
Influence of collars on the primary stability of cementless femoral stems: A finite element study using a diverse patient cohort	Al-Dirini R.M.A.; Huff D.; Zhang J.; Besier T.; Clement J.G.; Taylor M.	2018
Surgical management of supra hepatic vena cava inferior thrombosis post liver transplantation via a transatrial approach. a case report	Gundlach J.-P.	2018
Effect of high-frequency loading and parathyroid hormone administration on peri-implant bone healing and osseointegration	Shibamoto A.; Ogawa T.; Duyck J.; Vandamme K.; Naert I.; Sasaki K.	2018
Periodontal status of tooth adjacent to implant with peri-implantitis	Sung C.-E.; Chiang C.-Y.; Chiu H.-C.; Shieh Y.-S.; Lin F.-G.; Fu E.	2018
Peri-implant test is a proposal of a new procedure to prevent peri-implantitis and forensic claims	Gaudio R.M.; Ottria L.; Lauritano D.; Palmieri A.; Cura F.; Tagliabue A.; Tettamanti L.	2018
Incidence of Peri-implant Diseases on Implants With and Without Laser-Microgrooved Collar: A 5-Year Retrospective Study Carried Out in Private Practice Patients	Guarnieri R.; Grande M.; Zuffetti F.; Testori T.	2018
Photodynamic therapy for the treatment of peri-implant diseases: A network meta-analysis of randomized controlled trials	Sivaramakrishnan G.; Sridharan K.	2018
Is Metal Particle Release Associated with Peri-implant Bone Destruction? An Emerging Concept	Fretwurst T.; Nelson K.; Tarnow D.P.; Wang H.-L.; Giannobile W.V.	2018
The medically compromised patient: Are dental implants a feasible option?	Vissink A.; Spijkervet F.K.L.; Raghoebar G.M.	2018
Mechanical, chemical and biological aspects of titanium and titanium alloys in implant dentistry	Ottria L.; Lauritano D.; Andreasi Bassi M.; Palmieri A.; Candotto V.; Tagliabue A.; Tettamanti L.	2018
The Efficacy of an Anatase-Coated Collar Surface in Inhibiting the Bacterial Colonization of Oral Implants: A Pilot Prospective Study in Humans	Cucchi A.; Molè F.; Rinaldi L.; Marchetti C.; Corinaldesi G.	2018
A Survey Exploring the Experiences & Attitudes of Dental Implant Clinicians in the Management of Peri-implantitis within the United Kingdom	Kane G.; Fang V.; Simon S.; Girdler J.; Adeyinka O.; Alhilou A.	2018
Bone response to functionally loaded, two-piece zirconia implants: A preclinical histometric study	Janner S.F.M.; Gahlert M.; Bosshardt D.D.; Roehling S.; Milz S.; Higginbottom F.; Buser D.; Cochran D.L.	2018
Retrospective Evaluation of Factors Related to the Outcomes of Regenerative Therapy for Implants Affected by Peri-implantitis	Froum S.J.; Rosen P.S.; Wang W.C.; Froum S.H.; Vinayak S.	2018

Prominence of scientific publications towards peri-implant complications in implantology: A bibliometric analysis using the H-classics method	Chiang H.-S.; Huang R.-Y.; Weng P.-W.; Mau L.-P.; Tsai Y.-W.C.; Chung M.-P.; Chung C.-H.; Yeh H.-W.; Shieh Y.-S.; Cheng W.-C.	2018
Microbial findings in patients with implant supported dentures and electromagnetic field stimulation in the therapy of periimplantitis	Žužul I.; Carek A.	2018
Single-Dose Bioavailability for Prophylactic Coverage in Patients Undergoing Dental Implant Surgery	Aravena P.C.; Oyarzún C.P.; Arias M.F.; Monardes H.; Jerez A.; Benso B.	2018
A case of peri-mucositis: The importance of the oral cavity exam in evaluating new behavioral disturbance in a patient with advanced dementia	Blloshmi K.	2018
Osteogenic efficacy of BMP-2 mixed with hydrogel and bone substitute in peri-implant dehiscence defects in dogs: 16 weeks of healing	Cha J.-K.; Jung U.-W.; Thoma D.S.; Hämmerle C.H.F.; Jung R.E.	2018
Clinically Oriented Patient Maintenance Protocol: A Clinical Consensus of Experts	Del Fabbro M.; Nevins M.; Venturoli D.; Weinstein R.L.; Testori T.	2018
The Effect of Moderately Controlled Type 2 Diabetes on Dental Implant Survival and Peri-implant Bone Loss: A Long-Term Retrospective Study	Ormianer Z.; Block J.; Matalon S.; Kohen J.	2018
Decision Tree for Vertical Ridge Augmentation	Plonka A.B.; Urban I.A.; Wang H.-L.	2018
Peri-Implant Mucosal Creeping: Two Case Reports	Parra C.; Capri D.	2018
Immediate Reconstruction of Failed Implants in the Esthetic Zone Using a Flapless Technique and Autogenous Composite Tuberosity Graft	Raghoebar G.M.; Meijer H.J.A.; van Minnen B.; Vissink A.	2018
Crestal Bone Level Around Tissue-Level Implants Restored with Platform Matching and Bone-Level Implants Restored with Platform Switching: A 5-Year Randomized Controlled Trial	Lago L.; da Silva L.; Martinez-Silva I.; Rilo B.	2018
PD-l1 expression in cases of breast implantassociated anaplastic large cell lymphoma reveals a new target for therapy	Solorzano J.L.; Francisco-Cruz A.; Medeiros L.J.; Young K.H.; Clemens M.W.; Wistuba I.; Miranda R.	2018
Learning from clinical phenotypes: Low-dose biophotonics therapies in oral diseases	Rahman S.U.; Mosca R.C.; Govindool Reddy S.; Nunez S.C.; Andreana S.; Mang T.S.; Arany P.R.	2018
Zinc plus octenidine: A new formulation for treating periodontal pathogens. A single blind study	Lauritano D.; Candotto V.; Bignozzi C.A.; Pazzi D.; Carinci F.; Cura F.; Tagliabue A.; Tettamanti L.	2018
Surgical implants and business ethics?	Cosson M.; Fernandez H.	2018
The role of zinc plus octenidine in the regulation of gene expression: An in vitro study	Lauritano D.; Candotto V.; Bignozzi C.A.; Pazzi D.; Carinci F.; Cura F.; Tagliabue A.; Tettamanti L.	2018
The Woven EndoBridge (WEB) for endovascular therapy of intracranial aneurysms: Update of a systematic review with meta-analysis	Tau N.; Sadeh-Gonik U.; Aulagner G.; Turjman F.; Gory B.; Armoiry X.	2018
Safety of uninterrupted dabigatran in cardiovascular interventions in the GLORIA-AF registry program	Lip G.Y.H.; Van Der Wall S.J.; Teutsch C.; Rothman K.J.; Diener H.C.; Dubner S.J.; Ma C.S.; Paquette M.; Lu S.; Riou Franca L.; Zint K.; Halperin J.L.; Huisman M.V.	2018
Variation in the rates of surgical treatment for female stress urinary incontinence in England: An ecological study	Geary R.; Duckett J.; El-Hamamsy D.; Mahmood T.; Monga A.; Toozs-Hobson P.; Wilson A.; Van Der Meulen J.; Tincello D.; Mamza J.; Gurol U.	2018
Limiting complication rates in implant-based breast reconstruction	Locke M.B.; Malins W.L.E.; Le See J.; Kenealy J.	2018
Rationale and design of a global registry to evaluate real-world clinical outcomes in patients with atrial fibrillation and high risk of stroke treated with left atrial appendage occlusion using the AMPLATZER amulet device—Perspective of available/ongoing registries of catheter-based LAA occlusion	Hildick-Smith D.; Diener H.-C.; Schmidt B.; Paul V.; Settergren M.; Teiger E.; Camm J.; Tondo C.; Landmesser U.	2018
Peri-implant conditions and marginal bone loss around cemented and screw-retained single implant crowns in posterior regions: A retrospective cohort study with up to 4 years follow-up	Shi J.-Y.; Ni J.; Zhuang L.-F.; Zhang X.-M.; Fan L.-F.; Lai H.-C.	2018
Restoration contour is a risk indicator for peri-implantitis: A cross-sectional radiographic analysis	Katafuchi M.; Weinstein B.F.; Leroux B.G.; Chen Y.-W.; Daubert D.M.	2018
Experimental research on Arginine-gingipain A gene vaccine from Porphyromonas gingivalis that prevents peri-implantitis in Beagle dogs	Chuanhua L.; Zhifeng W.; Lina Z.; Xin F.; Jing L.	2018
Comparison of peri-implant clinical and radiographic status around short (6 mm in length) dental implants placed in cigarette-smokers and never-smokers: Six-year follow-up results	Abduljabbar T.; Al-Hamoudi N.; Al-Sowygh Z.H.; Alajmi M.; Javed F.; Vohra F.	2018
Comparison of peri-implant clinical and radiographic inflammatory parameters among cigarette and waterpipe (narghile) smokers and never-smokers	ALHarthi S.S.; BinShabaib M.S.; Ahmed H.B.; Mehmood A.; Khan J.; Javed F.	2018
The periodontal pocket	Donos N.	2018
Susceptibility of different mouse strains to peri-implantitis	Hiyari S.; Naghibi A.; Wong R.; Sadreshkevary R.; Yi-Ling L.; Tetradis S.; Camargo P.M.; Pirih F.Q.	2018
Evaluation of gingival crevicular fluid and peri-implant sulcus fluid levels of periostin: A preliminary report	Akman A.C.; Buyukozdemir Askin S.; Guncu G.N.; Nohutcu R.M.	2018
Implants With and Without Laser-Microtextured Collar: A 10-Year Clinical and Radiographic Comparative Evaluation	Guarnieri R.; Ceccarelli R.; Ricci J.L.; Testori T.	2018
Risk factors for post-operative complications after procedures for autologous bone augmentation from different donor sites	Sakkas A.; Schramm A.; Winter K.; Wilde F.	2018
Hard tissue volume stability of guided bone regeneration during the healing stage in the anterior maxilla: A clinical and radiographic study	Jiang X.; Zhang Y.; Di P.; Lin Y.	2018
Mini-implants and narrow diameter implants as mandibular overdenture retainers: A systematic review and meta-analysis of clinical and radiographic outcomes	Marcello-Machado R.M.; Faot F.; Schuster A.J.; Nascimento G.G.; Del Bel Cury A.A.	2018

New approach for vertical bone regeneration using in situ gelling and sustained BMP-2 releasing poly(phosphazene) hydrogel system on peri-implant site with critical defect in a canine model	Seo B.-B.; Chang H.-I.; Choi H.; Koh J.-T.; Yun K.-D.; Lee J.-Y.; Song S.-C.	2018
Definitive Abutments Placed at Implant Insertion and Never Removed: Is It an Effective Approach? A Systematic Review and Meta-Analysis of Randomized Controlled Trials	Tallarico M.; Caneva M.; Meloni S.M.; Xhanari E.; Covani U.; Canullo L.	2018
Peri-conceptional progesterone treatment in women with unexplained recurrent miscarriage: a randomized double-blind placebo-controlled trial	Ismail A.M.; Abbas A.M.; Ali M.K.; Amin A.F.	2018
The European Registry for Patients with Mechanical Circulatory Support (EUROMACS) of the European Association for Cardio-Thoracic Surgery (EACTS): Second report	de By T.M.M.H.; Mohacsi P.; Gahl B.; Zittermann A.; Krabatsch T.; Gustafssonm F.; Leprince P.; Meyns B.; Netuka I.; Caliskan K.; Castedo E.; Musumeci F.; Vincentelli A.; Hetzer R.; Gummert J.	2018
Guidelines of the American Venous Forum on venous and lymphatic disorders	Gloviczki P.	2018
A novel cold atmospheric pressure air plasma jet for peri-implantitis treatment: An in vitro study	Yang Y.; Guo J.; Zhou X.; Liu Z.; Wang C.; Wang K.; Zhang J.; Wang Z.	2018
Effect of implant placement depth on the peri-implant bone defect configurations in ligature-induced peri-implantitis: An experimental study in dogs	Huang B.; Zhang L.; Xu L.; Zhu W.; Witek L.; Tovar N.; Coelho P.G.; Meng H.	2018
International guidelines for groin hernia management	The Herniasurge Group; Simons M.P.; Smietanski M.; Bonjer H.J.; Bittner R.; Miserez M.; Aufenacker T.J.; Chowbey P.K.; Tran H.M.; Sani R.; Berrevoet F.; Bingener J.; Bisgaard T.; Bury K.; Campanelli G.; Chen D.C.; Conze J.; Cuccurullo D.; de Beaux A.C.; Eker H.H.; Fortelny R.H.; Gillion J.F.; van Den Heuvel B.J.; Hope W.W.; Jorgensen L.N.; Klinge U.; Köckerling F.; Kukleta J.F.; Konate I.; Liem A.L.; Lomanto D.; Loos M.J.A.; Lopez-Cano M.; Misra M.C.; Montgomery A.; Morales-Conde S.; Muysoms F.E.; Niebuhr H.; Nordin P.; Pawlak M.; van Ramshorst G.H.; Reinpold W.M.J.; Sanders D.L.; Schouten N.; Smedberg S.; Simmermacher R.K.J.; Tumtavitikul S.; van Veenendaal N.; Weyhe D.; Wijsmuller A.R.; Jeekel J.; Sharma A.; Ramshaw B.	2018
Evaluation of peri-implant tissues around nanopore surface implants with or without platelet rich fibrin: A clinico-radiographic study	Khan Z.A.; Jhingran R.; Bains V.K.; Madan R.; Srivastava R.; Rizvi I.	2018
Comparison of outcome measures and complication rates following three different approaches for primary total hip arthroplasty: A pragmatic randomised controlled trial	Talia A.J.; Coetzee C.; Tirosh O.; Tran P.	2018
Is History of Periodontitis a Risk Factor for Peri-implant Disease? A Pilot Study	Altay M.A.; Tozoğlu S.; Yıldırımyan N.; Özarslan M.M.	2018
The Ability of Quantitative, Specific, and Sensitive Point-of-Care/Chair-Side Oral Fluid Immunotests for aMMP-8 to Detect Periodontal and Peri-Implant Diseases	Alassiri S.; Parnanen P.; Rathnayake N.; Johannsen G.; Heikkinen A.-M.; Lazzara R.; Van Der Schoor P.; Van Der Schoor J.G.; Tervahartiala T.; Gieselmann D.; Sorsa T.	2018
Management of peri-implantitis - A survey	Swetha V.; Ganapathy D.; Jain A.R.	2018
Supportive peri-implant therapy following anti-infective surgical peri-implantitis treatment: 5-year survival and success	Heitz-Mayfield L.J.A.; Salvi G.E.; Mombelli A.; Loup P.-J.; Heitz F.; Kruger E.; Lang N.P.	2018
Surgical treatment of peri-implantitis: Prognostic indicators of short-term results	Koldsland O.C.; Wohlfahrt J.C.; Aass A.M.	2018
Clinical and radiographic assessment of implant-supported rehabilitation of partial and complete edentulism: A 2 to 8 years clinical follow-up	Manicone P.F.; Passarelli P.C.; Bigagnoli S.; Pastorino R.; Manni A.; Pasquantonio G.; D'Addona A.	2018
Biology of teeth and implants: Host factors - pathology, regeneration, and the role of stem cells	Eggert F.-M.; Levin L.	2018
Comparison of Clinical, Radiographic, and Immunologic Inflammatory Parameters around Crestally and Subcrestally Placed Dental Implants: 5-Year Retrospective Results	Al Amri M.D.; Alfadda S.A.; Labban N.Y.; Alasqah M.N.; Alshehri F.A.; Al-Rasheed A.S.	2018
Implant treatment in pharmacologically immunosuppressed liver transplant patients: A prospective-controlled study	Paredes V.; López-Pintor R.M.; Torres J.; de Vicente J.C.; Sanz M.; Hernández G.	2018
Narrow- and regular-diameter implants in the posterior region of the jaws to support single crowns: A 3-year split-mouth randomized clinical trial	de Souza A.B.; Sukekava F.; Tolentino L.; César-Neto J.B.; Garcez-Filho J.; Araújo M.G.	2018
Five-year outcomes of a randomized clinical trial comparing bone-level implants with either submerged or transmucosal healing	Flores-Guillen J.; Álvarez-Novoa C.; Barbieri G.; Martín C.; Sanz M.	2018
Influence of Microtextured Implant Surfaces on Peri-implantitis and Its Treatment: A Preclinical Trial	Rodriguez J.C.; Koticha T.; Eubanks D.L.; Rudek I.; Molz F.J.; Chiavaccini L.; Claude A.; Elder S.; Wang H.-L.	2018
5-year outcomes after coverage of soft tissue dehiscence around single implants: A prospective cohort study	Zucchelli G.; Felice P.; Mazzotti C.; Marzadori M.; Mounssif I.; Monaco C.; Stefanini M.	2018
Long-term outcomes of narrow diameter implants in posterior jaws: A retrospective study with at least 8-year follow-up	Shi J.-Y.; Xu F.-Y.; Zhuang L.-F.; Gu Y.-X.; Qiao S.-C.; Lai H.-C.	2018
Peri-implantitis Management in the Esthetic Zone in a Periodontally Compromised Patient: Five-Year Results Including Cone Beam Computed Tomography	Kourkouta S.	2018
The use of antibiotics: Data analysis of a survey among a sample of Italian dentists	Giargia M.; Scotti E.; Marchetti S.; Mensi M.	2018
Digital versus analog procedures for the prosthetic restoration of single implants: A randomized controlled trial with 1 year of follow-up	Mangano F.; Veronesi G.	2018
Peri-implant clinical and radiographic status and whole salivary cotinine levels among cigarette and waterpipe smokers and never-smokers	BinShabaib M.S.; Mehmood A.; Akram Z.; ALHarthi S.S.	2018

Long-Term Effects of Bone Morphogenetic Protein-2-Loaded Calcium Phosphate on Maxillary Sinus Lift Surgery for Delayed and Simultaneous Dental Implantation	Ding Y.; Wang X.	2018
Bone minimodeling, a special modeling pattern and potential as therapeutic target for osteoporosis	Li X.; Zhang J.; Wang X.	2018
Peri-implant Tissues and Patient Satisfaction After Treatment of Vertically Augmented Atrophic Posterior Mandibles with Intraoral Onlay Block Bone Grafts: A Retrospective 3-Year Case Series Follow-up Study	Aloy-Prósper A.; Peñarrocha-Oltra D.; Peñarrocha-Diago M.; Hernández-Alfaro F.; Peñarrocha-Diago M.	2018
Chronic Periodontitis and RANKL/OPG Ratio in Peri-Implant Mucosae Inflammation	Costa L.C.; Fonseca M.A.D.; Pinheiro A.D.R.; Aguiar T.R.D.S.; Machado A.N.; Quinelato V.; Bonato L.L.; Aguiar D.P.; Vieira T.; Almeida F.L.D.; Lobo J.C.; Jordão M.; Lomardo P.G.; Granjeiro J.M.; Casado P.L.	2018
Nitric oxide and arginase levels in peri-implant tissues after delayed loading	Gokmenoglu C.; Ozmeric N.; Sungur C.; Sahin Bildik R.; Erguder I.; Elgun S.	2018
The effect of smoking on peri-implant marginal bone loss in periodontally compromised patients	Meric P.; Gurlek O.; Kanmaz B.; Buduneli N.	2018
Minocycline hydrochloride ointment is available for the treatment of peri-implantitis	Liu O.; Gao Z.; Li T.; Zhou B.	2018
Peri-implant Crestal Bone Changes Around Zirconia Implants in Periodontally Healthy and Compromised Patients	Kniha K.; Milz S.; Kniha H.; Ayoub N.; Hölzle F.; Modabber A.	2018
Performance and marginal bone level alteration around immediately loaded narrow-diameter implants. A prospective clinical study: Results after 1 year	Worni A.; Hicklin S.P.; Mericske-Stern R.; Enkling N.	2018
Effects of modified abutment characteristics on peri-implant soft tissue health: A systematic review and meta-analysis	Sanz-Martín I.; Sanz-Sánchez I.; Carrillo de Albornoz A.; Figuero E.; Sanz M.	2018
What is the survival rate of dental implants in Turkey? A systematic review	Cabbar F.; Burdurlu M.Ç.; Işiksaçan N.S.; Atalay B.; Çapar G.D.	2018
One-Piece Implants with Smooth Concave Neck to Enhance Soft Tissue Development and Preserve Marginal Bone Levels: A Retrospective Study with 1- to 6-Year Follow-Up	Axiotis J.-P.; Nuzzolo P.; Barausse C.; Gasparro R.; Bucci P.; Pistilli R.; Sammartino G.; Felice P.	2018
Surface characteristics of dental implants: A review	Rupp F.; Liang L.; Geis-Gerstorfer J.; Scheideler L.; Hüttig F.	2018
Peri-implant tissue score (PITS) as a measure of success, applied to 869 dental implants from a retrospective clinical study	Mazur Z.; Korábek L.; Mazur D.	2018
One-piece zirconia oral implants for single-tooth replacement: Three-year results from a long-term prospective cohort study	Kohal R.-J.; Spies B.C.; Bauer A.; Butz F.	2018
Immediate, early (6 weeks) and delayed loading (3 months) of single, partial and full fixed implant supported prostheses: 1-year post-loading data from a multicentre randomised controlled trial	Mitsias M.; Siormpas K.; Pistilli V.; Trullenque-Eriksson A.; Esposito M.	2018
Posterior atrophic jaws rehabilitated with prostheses supported by 6 mm long × 4 mm wide implants or by longer implants in augmented bone. 3-year post-loading results from a randomised controlled trial	Felice P.; Barausse C.; Pistilli V.; Piattelli M.; Ippolito D.R.; Esposito M.	2018
Posterior atrophic jaws rehabilitated with prostheses supported by 5 × 5 mm implants with a nanostructured calcium-incorporated titanium surface or by longer implants in augmented bone. 3-year results from a randomised controlled trial	Gastaldi G.; Felice P.; Pistilli V.; Barausse C.; Ippolito D.R.; Esposito M.	2018
Effect of implantoplasty on fracture resistance and surface roughness of standard diameter dental implants	Costa-Berenguer X.; García-García M.; Sánchez-Torres A.; Sanz-Alonso M.; Figueiredo R.; Valmaseda-Castellón E.	2018
4 mm long vs longer implants in augmented bone in posterior atrophic jaws: 1-year post-loading results from a multicentre randomised controlled trial	Bolle C.; Felice P.; Barausse C.; Pistilli V.; Trullenque-Eriksson A.; Esposito M.	2018
One Abutment at One Time Concept for Platform-Switched Morse Implants: Systematic Review and Meta-Analysis	Santos J.S.; Santos T.S.; Martins-Filho P.R.S.; Krockow N.V.; Weigl P.; Pablo H.	2018
Volumetric Bone Measurement Around Dental Implants Using 3D Image Superimposition: A Methodological and Clinical Pilot Study	Villarinho E.A.; Correia A.; Vigo A.; Ramos N.V.; Pires Vaz M.A.; Arai Shinkai R.S.	2018
Bone Regeneration of Peri-Implant Defects Using a Collagen Membrane as a Carrier for Recombinant Human Bone Morphogenetic Protein-2	Sun Y.-K.; Cha J.-K.; Thoma D.S.; Yoon S.-R.; Lee J.-S.; Choi S.-H.; Jung U.-W.	2018
Prevalence of Dental Implants and Evaluation of Peri-implant Bone Levels in Patients Presenting to a Dental School: A Radiographic Cross-Sectional 2-Year Study	Alkan E.A.; Mau L.P.; Schoolfield J.; Guest G.F.; Cochran D.L.	2018
Tobacco use, nicotine, and oral microorganisms	Hanioka T.; Ojima M.; Ogawa H.	2018
Raloxifene but not alendronate can compensate the impaired osseointegration in osteoporotic rats	Faverani L.P.; Polo T.O.B.; Ramalho-Ferreira G.; Momesso G.A.C.; Hassumi J.S.; Rossi A.C.; Freire A.R.; Prado F.B.; Luvizuto E.R.; Gruber R.; Okamoto R.	2018
A New Prognostication System for Dental Implants	Najafi B.; Kheirieh P.; Torabi A.; Cappetta E.G.; Najafi A.; Singh S.M.	2018
Peri-implant bone resorption during healing abutment placement: The effect of a 0.20% chlorhexidine gel vs. Placebo—a randomized double blind controlled human study	Sinjari B.; D'Addazio G.; De Tullio I.; Traini T.; Caputi S.	2018
A systematic review and meta-analysis of long-term studies (five or more years) assessing maxillary sinus floor augmentation	Starch-Jensen T.; Aludden H.; Hallman M.; Dahlin C.; Christensen A.-E.; Mordenfeld A.	2018
The role of dental implant abutment design on the aesthetic outcome: preliminary 3-month post-loading results from a multicentre split-mouth randomised controlled trial comparing two different abutment designs	Esposito M.; Cardaropoli D.; Gobbato L.; Scutellà F.; Fabianelli A.; Mascellani S.; Delli Ficorelli G.; Mazzocco F.; Sbricoli L.; Trullenque-Eriksson A.	2018
Immediate definitive individualized abutments reduce peri-implant bone loss: a randomized controlled split-mouth study on 16 patients	Erhan Çömlekoğlu M.; Nizam N.; Çömlekoğlu M.D.	2018

Effect of DSR-based internal sinus floor elevation with implantation on atrophic maxilla	Li F.; Wang Y.-H.; Wang D.-S.; Wang W.-X.; Jiang S.; Jin H.-Y.; Gao L.; Teng M.-H.; Liang J.-Y.; Chu Y.-N.; Zhi K.-Q.; Zhao B.-D.	2018
Local bone treatment in osteoporosis	Kurth A.	2018
A rare case of thoracic endometriosis presenting with hemopneumothorax	Yang X.; Steiger D.; Miyakawa L.; Filopei J.	2018
Repercussion of a national intervention on patient outcomes of primary knee arthroplasty to enhance the recovery pathway: 2008-2016 England trends	Garriga C.; Prats-Uribe A.; Price A.; Prieto-Alhambra D.; Carr A.; Arden N.K.; Leal J.; Gooberman-Hill R.; Peat G.; Rangan A.; Fitzpatrick R.; Barker K.; Cooper C.; Judge A.	2018
Fracture neck of femur - A local experience	Ong L.H.; Sabaruddin A.; Rutel A.; Jaffar M.S.A.; Kamaruddin F.	2018
Recurrent stenosis following carotid artery stenting treated with a drug-eluting balloon: a single-center retrospective analysis	Pohlmann C.; Höltje J.; Zeile M.; Bonk F.; Urban P.P.; Brüning R.	2018
Response of soft tissue to different abutment materials with different surface topographies: a review of the literature	Al Rezk F.; Trimpou G.; Lauer H.-C.; Weigl P.; Krockow N.	2018
Cavernous sinus thrombosis in a patient using etonogestrel/ethyinyl estradiol vaginal ring	Selim M.A.; Kassim T.A.; Rakhra A.; Jabbari J.; Valenta C.	2018
Safety and feasibility of angioplasty and stenting in symptomatic internal carotid artery occlusive disease	Panczykowski D.M.; Jadhav A.P.; Jumaa M.A.; Aghaebrahim A.; Ranginani M.; Nguyen F.; Grandhi R.; Ducruet A.F.; Gross B.A.; Jankowitz B.T.; Jovin T.G.	2018
Experimental pilot study of combined thoracoscopic surgical stapling and intrabronchial valve intervention for incomplete lobar fissures in swine	Kheir F.; Gangadharan S.; Alape D.; Chee A.C.; Parikh M.S.; DeVore L.; Agnew A.; Majid A.	2018
Percutaneous patent foramen ovale closure for the secondary prevention of cryptogenic cerebral ischemia: A network meta-analysis	Tsivgoulis G.; Katsanos A.H.; Mavridis D.; Frogoudaki A.; Vrettou A.-R.; Ikonomidis I.; Parissis J.; Deftereos S.; Karapanayiotides T.; Palaiodimou L.; Filippatou A.; Perren F.; Hadjigeorgiou G.; Alexandrov A.W.; Mitsias P.D.; Alexandrov A.V.	2018
Prevalence of Epstein-Barr virus DNA and Porphyromonas gingivalis in Japanese peri-implantitis patients	Kato A.; Imai K.; Sato H.; Ogata Y.	2017
Influence of implantoplasty on stress distribution of exposed implants at different bone insertion levels	Tribst J.P.M.; Dal Piva A.M.O.; Shibli J.A.; Borges A.L.S.; Tango R.N.	2017
Immediately loaded implant-supported full-arches: Peri-implant status after 1-9years in a private practice	Cercadillo-Ibarguren I.; Sánchez-Torres A.; Figueiredo R.; Schwarz F.; Gay-Escoda C.; Valmaseda-Castellón E.	2017
A retro-prospective effectiveness study on 3448 implant operations at one referral clinic: A multifactorial analysis. Part II: Clinical factors associated to peri-implantitis surgery and late implant failures	Jemt T.	2017
Mandibular Osteomyelitis Following Implant Placement	Schlund M.; Raoul G.; Ferri J.; Nicot R.	2017
Can resonance frequency analysis detect narrow marginal bone defects around dental implants? An ex vivo animal pilot study	Yao C.J.; Ma L.; Mattheos N.	2017
Parameters to Define Peri-Implantitis: A Review and a Proposed Multi-Domain Scale	Lin G.-H.; Kapila Y.; Wang H.-L.	2017
Randomized controlled clinical trial comparing one-piece and two-piece dental implants supporting fixed and removable dental prostheses: 4- to 6-year observations	Gamper F.B.; Benic G.I.; Sanz-Martin I.; Asgeirsson A.G.; Hämmerle C.H.F.; Thoma D.S.	2017
The effect of antimicrobial photodynamic therapy (APDT) and light-activated disinfection (LAD) on contaminated titanium and zirconia implants-in vitro study	Azizi B.; Budimir A.; Mehmeti B.; Kelmendi J.; Gabric D.	2017
Short implants (6 mm) vs. vertical bone augmentation and standard-length implants (≥9 mm) in atrophic posterior mandibles: a 5-year retrospective study	Pieri F.; Forlivesi C.; Caselli E.; Corinaldesi G.	2017
Radiographic and clinical outcomes of rooted, platform-switched, microthreaded implants with a sandblasted, large-grid, and acid-etched surface: A 5-year prospective study	Mei D.M.; Zhao B.; Xu H.; Wang Y.	2017
Assessment of the effectiveness of platelet rich fibrin in the treatment of Schneiderian membrane perforation	Öncü E.; Kaymaz E.	2017
Survival rate of titanium-zirconium narrow diameter dental implants versus commercially pure titanium narrow diameter dental implants: A systematic review	Iegami C.M.; Uehara P.N.; Sesma N.; Pannuti C.M.; Tortamano Neto P.; Mukai M.K.	2017
Long-term evaluation of osteotome sinus floor elevation and simultaneous placement of implants without bone grafts: 10-Year radiographic and clinical follow-up	Caban J.; Fermergård R.; Abtahi J.	2017
Maxillary implant-supported overdentures opposed by (partial) natural dentitions: a 5-year prospective case series study	Boven G.C.; Slot J.W.A.; Raghoebar G.M.; Vissink A.; Meijer H.J.A.	2017
Prognostic Classification System for Implant Recession Defects	Decker A.M.; Suárez-López Del Amo F.; Urban I.A.; Miller P.D.; Wang H.-L.	2017
The influence of crown-to-implant ratio on marginal bone levels around splinted short dental implants: A radiological and clincial short term analysis	Hingsammer L.; Watzek G.; Pommer B.	2017
Organic degradation potential of a TiO2/H2O2/UV-vis system for dental applications	Janson O.; Unosson E.; Strømme M.; Engqvist H.; Welch K.	2017
A particularly case of tricuspid valve endocarditis due to jet lesion in a patient with congenital heart disease	Dentamaro I.; Gutierrez Garcia-Moreno L.; Fernandez-Galera R.; Gonzalez-Alujas M.T.; Villalva N.; Granato C.; Italiano G.; Espriu Simon M.; Rodriguez-Palomares J.F.; Evangelista Masip A.	2017
The role of intravascular ultrasound in lower limb revascularization in patients with peripheral arterial disease	Makris G.C.; Chryrysafi P.; Little M.; Patelatel R.; Ratby M.B.; Wigham A.; Anthony S.; Uberoi R.	2017

Self expanding metallic stents placement-A tertiary care experience	Radhakrishnan N.; Rabindranath E.; Sheikh M.K.; Kini S.R.; Kumar K.P.	2017
Hydromorphone Hydrochloride Use During Various Interventional Radiology Procedures—Pain Control and Adverse Events: A Case Series Audit Analysis	Moran T.; Prabhakar A.; Diaz J.H.; Kaye A.D.	2017
Location of unaccessible implant surface areas during debridement in simulated peri-implantitis therapy	Steiger-Ronay V.; Merlini A.; Wiedemeier D.B.; Schmidlin P.R.; Attin T.; Sahrmann P.	2017
Peri-implant maintenance therapy: Association of preventive maintenance therapy compliance and peri-implant disease: A cross-sectional study		2017
Long-term follow up of implant therapy: Occurrence of cases with peri-implant mucositis or peri-implantitis in a 21-26-year follow-up study		2017
Anti-Bacteria and Microecosystem-Regulating Effects of Dental Implant Coated with Dimethylaminododecyl Methacrylate	Li B.; Ge Y.; Wu Y.; Chen J.; Xu H.H.K.; Yang M.; Li M.; Ren B.; Feng M.; Weir M.D.; Peng X.; Cheng L.; Zhou X.	2017
Peri-procedural myocardial infarction: Plaques and patients "at-risk"	Goldstein J.A.	2017
VASO (Vitamin D and Arthroplasty Surgery Outcomes) study - supplementation of vitamin D deficiency to improve outcomes after total hip or knee replacement: Study protocol for a randomised controlled feasibility trial	Morrison R.J.M.; Bunn D.; Gray W.K.; Baker P.N.; White C.; Rangan A.; Rankin K.S.; Reed M.R.	2017
Air-abrasive debridement with glycine powder versus manual debridement and chlorhexidine administration for the maintenance of peri-implant health status: a six-month randomized clinical trial	Lupi S.M.; Granati M.; Butera A.; Collesano V.; Rodriguez Y Baena R.	2017
Patient-Centered Perspectives and Understanding of Peri-Implantitis	Insua A.; Monje A.; Wang H.-L.; Inglehart M.	2017
Classification and Clinical Management of Retrograde Peri-implantitis Associated with Apical Periodontitis: A Proposed Classification System and Case Report	Sarmast N.D.; Wang H.H.; Sajadi A.S.; Angelov N.; Dorn S.O.	2017
Microbiome of peri-implantitis affected and healthy dental sites in patients with a history of chronic periodontitis	Apatzidou D.; Lappin D.F.; Hamilton G.; Papadopoulos C.A.; Konstantinidis A.; Riggio M.P.	2017
Implant-Abutment Connections: A Review of Biologic Consequences and Peri-implantitis Implications	Sasada Y.; Cochran D.L.	2017
Periapical implant lesion: A systematic review	Blaya-Tárraga J.-A.; Cervera-Ballester J.; Peñarrocha-Oltra D.; Peñarrocha-Diago M.	2017
The effect of metal ions released from different dental implant-abutment couples on osteoblast function and secretion of bone resorbing mediators	Alrabeah G.O.; Brett P.; Knowles J.C.; Petridis H.	2017
Compliance with Supportive Periodontal Treatment in Patients with Dental Implants	Hu K.-F.; Lin Y.-C.; Ho K.-Y.; Chou Y.-H.	2017
Intra-surgical vs. radiographic bone level assessments in measuring peri-implant bone loss	Serino G.; Sato H.; Holmes P.; Turri A.	2017
Peri-implant bleeding on probing: a cross-sectional multilevel analysis of associated factors	Merli M.; Bernardelli F.; Giulianelli E.; Toselli I.; Mariotti G.; Nieri M.	2017
Associations of alveolar bone loss and interleukin-1β levels in one- and two-stage surgical procedures: a randomized prospective trial	Talo Yildirim T.; Acun Kaya F.; Yokus B.; Colak M.; Ozdemir Kaya E.; Tekin G.; Saribas E.; Uysal E.; Guncu G.N.	2017
Peri-Implantitis: A multiwavelength approach	Rocca J.P.; Zhang Y.; Merigo E.; Zhen Y.; Fornaini C.; Zhao Z.	2017
Laser minimally invasive bactericidal approch for the 21 century dentistry	Kornblit R.	2017
Soft tissue changes and crestal bone loss around platform-switched implants placed at crestal and subcrestal levels: 36-month results from a prospective split-mouth clinical trial	Al Amri M.D.; Al-Johany S.S.; Al Baker A.M.; Al Rifaiy M.Q.; Abduljabbar T.S.; Al-Kheraif A.A.	2017
Does local delivery of bisphosphonates influence the osseointegration of titanium implants? A systematic review	Guimarães M.B.; Antes T.H.; Dolacio M.B.; Pereira D.D.; Marquezan M.	2017
Early bone formation around immediately loaded implants with nanostructured calcium-incorporated and machined surface: a randomized, controlled histologic and histomorphometric study in the human posterior maxilla	Mangano F.G.; Iezzi G.; Shibli J.A.; Pires J.T.; Luongo G.; Piattelli A.; Mangano C.	2017
Implant maintenance for the prevention of biological complications: Are you ready for the next challenge?	Goh E.X.J.; Lim L.P.	2017
Comparison of peri-implant bone loss between conventional drilling with irrigation versus low-speed drilling without irrigation	Pellicer-Chover H.; Peñarrocha-Oltra D.; Aloy-Prosper A.; Sanchis-Gonzalez J.-C.; Peñarrocha-Diago M.; Peñarrocha-Diago M.	2017
Peri-Implant Bone Loss of External Hexagon and Morse Taper in Patients Wearing Immediately Loaded Overdentures	Melo L.A.; Souza M.B.C.; Barbosa G.A.S.; Carreiro A.D.F.P.	2017
Simultaneous lateral bone augmentation and implant placement using a particulated synthetic bone substitute around chronic peri-implant dehiscence defects in dogs	Jung U.-W.; Cha J.-K.; Vignoletti F.; Nuñez J.; Sanz J.; Sanz M.	2017
Partial humeral replacement for peri-prosthetic fractures of the humerus	Manohara R.; Howie C.R.	2017
Aberrant artery identified during necrosectomy for walled-off pancreatic necrosis	Queliza K.; Fishman D.	2017
Stabilizing peri-stent restenosis using a novel therapeutic carrier	Moody M.R.; Huang S.-L.; Kim H.; Yin X.; Peng T.; Laing S.T.; Klegerman M.E.; Genstler C.; Haworth K.J.; Holland C.K.; McPherson D.D.; Kee P.	2017
Is using bisphosphonate a contraindication for dental treatments?	Heidari M.; Motevali S.; Maboodi A.; Sobouti F.	2017
Comparison of two different techniques used for the maintenance of peri-implant soft tissue health: a pilot randomized clinical trial	Al Ghazal L.; O'Sullivan J.; Claffey N.; Polyzois I.	2017

Association of Preventive Maintenance Therapy Compliance and Peri-Implant Diseases: A Cross-Sectional Study	Monje A.; Wang H.-L.; Nart J.	2017
Failing to meet the goals of periodontal recall programs. What next?	McCracken G.; Asuni A.; Ritchie M.; Vernazza C.; Heasman P.	2017
Comparison of clinical and radiographic parameters around short (6 to 8 mm in length) and long (11 mm in length) dental implants placed in patients with and without type 2 diabetes mellitus: 3-year follow-up results	Al Amri M.D.; Abduljabbar T.S.; Al-Johany S.S.; Al Rifaiy M.Q.; Alfarraj Aldosari A.M.; Al-Kheraif A.A.	2017
Implant rehabilitation in fibula-free flap reconstruction: A retrospective study of cases at 1–18 years following surgery	Sozzi D.; Novelli G.; Silva R.; Connelly S.T.; Tartaglia G.M.	2017
Zirconia abutments for single-tooth implant restorations: a 10- to 11-year follow-up study	Ekfeldt A.; Fürst B.; Carlsson G.E.	2017
Clinical, radiological, and gene expression analyses in smokers and non-smokers, Part 2: RCT on the late healing phase of osseointegration	Sayardoust S.; Omar O.; Norderyd O.; Thomsen P.	2017
Comparison of Dental Implant Performance Following Vertical Alveolar Bone Augmentation With Alveolar Distraction Osteogenesis or Autogenous Onlay Bone Grafts: A Retrospective Cohort Study	Zhao K.; Wang F.; Huang W.; Wang X.; Wu Y.	2017
Influence of different forms and materials (zirconia or titanium) of abutments in peri-implant soft-tissue healing using matrix metalloproteinase-8: A randomized pilot study	Kumar Y.; Jain V.; Chauhan S.S.; Bharate V.; Koli D.; Kumar M.	2017
Immediate dental implant placement following sinus elevation in maxillary bone levels less than 6mm	Young H.; Kwok J.; Patel D.; Patel V.	2017
Current Evidence on the Socket-Shield Technique: A Systematic Review	Gharpure A.S.; Bhatavadekar N.B.	2017
Effects of Four Different Crown Materials on the Peri-Implant Clinical Parameters and Composition of Peri-Implant Crevicular Fluid	Yu S.-J.; Shan W.-L.; Liu Y.-X.; Huang X.-Y.; Zhu G.-X.	2017
Randomized clinical study of the peri-implant healing to hydrophilic and hydrophobic implant surfaces in patients receiving anticoagulants	Marković A.; Đinić A.; Calvo Guirado J.L.; Tahmaseb A.; Šćepanović M.; Janjić B.	2017
Effect of prosthetic restoration on implant survival and success	Rammelsberg P.; Lorenzo-Bermejo J.; Kappel S.	2017
The role of IL-1 gene polymorphisms (IL1A, IL1B, and IL1RN) as a risk factor in unsuccessful implants retaining overdentures	Sampaio Fernandes M.; Vaz P.; Braga A.C.; Sampaio Fernandes J.C.; Figueiral M.H.	2017
Osteoarthritis-associated calcium-containing crystals and biomaterial microparticles both drive m1 macrophage polarization in a syk and map kinase-dependent manner	McCarthy G.M.; Mahon O.; O'Hanlon S.; Kelly D.; Dunne A.	2017
Post dental implant neuro integration around or remote?	Tewari S.; Sharma R.K.; Narula S.C.; Bala K.; Tanwar N.	2017
Long-Term Clinical and Radiographic Observation of Periimplant Tissues After Autogenous Soft Tissue Grafts: A 15-Year Retrospective Study	Park W.-B.; Kang K.L.; Han J.-Y.	2017
In-vitro cytocompatibility of dental resin monomers on osteoblast-like cells	Kraus D.; Wolfgarten M.; Enkling N.; Helfgen E.-H.; Frentzen M.; Probstmeier R.; Winter J.; Stark H.	2017
Immediate Early and Delayed Implants: A 2-Year Prospective Cohort Study of 131 Transmucosal Flapless Implants Placed in Sites With Different Pre-extractive Endodontic Infections	Prati C.; Zamparini F.; Pirani C.; Gatto M.R.; Piattelli A.; Gandolfi M.G.	2017
One-time versus repeated abutment connection for platform-switched implant: A systematic review and meta-analysis	Wang Q.-Q.; Dai R.; Cao C.Y.; Fang H.; Han M.; Li Q.-L.	2017
Increasing popularity of waterpipe tobacco smoking and electronic cigarette use: Implications for oral healthcare	Ramôa C.P.; Eissenberg T.; Sahingur S.E.	2017
Patient self-reported pain and contributing factors during the implant of cardiac implantable electronic devices	Brewster N.; Wilson D.; Foster W.; Taylor R.; Goyal D.	2017
Short-term dabigatran interruption before cardiac rhythm device implantation: Multi-centre experience from the RE-LY trial	Essebag V.; Proietti R.; Birnie D.H.; Wang J.; Douketis J.; Coutu B.; Parkash R.; Lip G.Y.H.; Hohnloser S.H.; Moriarty A.; Oldgren J.; Connolly S.J.; Ezekowitz M.; Healey J.S.	2017
Sevelamer-associated rectosigmoid ulcers in an end-stage renal disease patient presenting as hematochezia	Uy P.P.D.	2017
Preliminary audiologic and peri-operative outcomes of the Sophono™ transcutaneous bone conduction device: A systematic review	Bezdjian A.; Bruijnzeel H.; Daniel S.J.; Grolman W.; Thomeer H.G.X.M.	2017
Outcomes After Primary Percutaneous Coronary Intervention for ST-Elevation Myocardial Infarction Caused by Ectatic Infarct Related Arteries	Bogana Shanmugam V.; J. Psaltis P.; T.L. Wong D.; T. Meredith I.; Malaiapan Y.; Ahmar W.	2017
Intracardiac Echocardiography for Structural Heart and Electrophysiological Interventions	Basman C.; Parmar Y.J.; Kronzon I.	2017
Single-center retrospective review of ultrasound-accelerated versus traditional catheter-directed thrombolysis for acute lower extremity deep venous thrombosis	Lu T.; Loh T.M.; El-Sayed H.F.; Davies M.G.	2017
Decreasing Surgical Site Infections after Ventral Hernia Repair: A Quality-Improvement Initiative	Cherla D.V.; Holihan J.L.; Flores-Gonzalez J.R.; Lew D.F.; Escamilla R.J.; Ko T.C.; Kao L.S.; Liang M.K.	2017
Tb of porta hepatis presenting with obstructive Jaundice: A case report	Alsabbak H.; Zakharia K.; Malik A.; Ashraf H.; Sunstrum J.	2017
Silver-coated modular Megaendoprostheses in salvage revision arthroplasty after periimplant infection with extensive bone loss - A pilot study of 34 patients	Zajonz D.; Birke U.; Ghanem M.; Prietzel T.; Josten C.; Roth A.; Fakler J.K.M.	2017
Association of Cytokine Gene Polymorphism with Peri-implantitis Risk	Petkovic-Curcin A.; Zeljic K.; Cikota-Aleksic B.; Dakovic D.; Tatic Z.; Magic Z.	2017
Retrospective Analysis on Survival Rate, Template-Related Complications, and Prevalence of Peri-implantitis of 694 Anodized Implants Placed Using Computer-Guided Surgery: Results Between 1 and 10 Years of Follow-Up	Tallarico M.; Meloni S.M.	2017

Prognosis of Dental Implants Immediately Placed in Sockets Affected by Peri-implantitis: A Retrospective Pilot Study	Anitua E.; Piñas L.; Begoña L.; Alkhraisat M.H.	2017
Influence of implant location in patients with and without type 2 diabetes mellitus: 2-year follow-up	Abduljabbar T.; Javed F.; Malignaggi V.R.; Vohra F.; Kellesarian S.V.	2017
Implant Patient Compliance Varies by Periodontal Treatment History	Zeza B.; Pilloni A.; Tatakis D.N.; Mariotti A.; Di Tanna G.L.; Mongardini C.	2017
Evaluation of four designs of short implants placed in atrophic areas with reduced bone height: a three-year, retrospective, clinical and radiographic study	Lopez Torres J.A.; Gehrke S.A.; Calvo Guirado J.L.; Aristazábal L.F.R.	2017
Comparison of Proinflammatory Cytokine Levels in Gingival Crevicular Fluid around Dental Implants with Ceramic and Titanium Abutments	Negahdari R.; Rahbar M.; Fakhrzadeh V.; Eslami H.; Akbari T.; Bohluli S.	2017
Impact of implant-abutment connection on osteoimmunological and microbiological parameters in short implants: a randomized controlled clinical trial	Öztürk V.Ö.; Emingil G.; Bostanci N.; Belibasakis G.N.	2017
Rehabilitation of patients with extreme mandibular alveolar ridge atrophy by means of slim and short mini dental implants and mandibular overdenture	Kovacic I.; Kiršic S.P.; Celebic A.	2017
Immediate implant placement into fresh extraction sockets versus delayed implants into healed sockets: A systematic review and meta-analysis	Mello C.C.; Lemos C.A.A.; Verri F.R.; dos Santos D.M.; Goiato M.C.; Pellizzer E.P.	2017
Long-Term Retrospective Evaluation of Success of Narrow-Diameter Implants in Esthetic Areas: A Consecutive Case Series with 3 to 14 Years Follow-up	Froum S.J.; Shi Y.; Fisselier F.; Cho S.-C.	2017
Assessment of the gingival microcirculation at teeth and dental implants; A preclinical in-vivo pilot study	Deák A.; Fülöp Z.S.; Tánczos B.; Sógor V.; Sári K.; Varga I.; Németh N.; Stavropoulos A.; Hegedus C.S.	2017
Early volumetric changes after vertical augmentation of the atrophic posterior mandible with interpositional block graft versus onlay bone graft: A retrospective radiological study	Barone A.; Toti P.; Menchini-Fabris G.-B.; Felice P.; Marchionni S.; Covani U.	2017
Three-year Prospective Evaluation of Immediately Loaded Mandibular Implant Overdentures retained with Locator Attachments	Al-Dharrab A.	2017
Breast implant-associated anaplastic large cell lymphoma: A review and assessment of cutaneous manifestations	Shahriari N.; Ferenczi K.; Heald P.W.	2017
Influence of Abutment Height on Maintenance of Peri-implant Crestal Bone at Bone-Level Implants: A 3-Year Follow-up Study	Nóvoa L.; Batalla P.; Caneiro L.; Pico A.; Liñares A.; Blanco J.	2017
New approach to improve the keratinised peri-implant soft tissues in patients with intraoral osteocutaneous reconstruction using a free flap	Patel N.; Patel D.; Kwok J.	2017
Interproximal Papilla Stability Around CAD/CAM and Stock Abutments in Anterior Regions: A 2-Year Prospective Multicenter Cohort Study	Lops D.; Parpaiola A.; Paniz G.; Sbricoli L.; Magaz V.R.; Venezze A.C.; Bressan E.; Stellini E.	2017
How TAVI registries report clinical outcomes—A systematic review of endpoints based on VARC-2 definitions	Zhang S.; Kolominsky-Rabas P.L.	2017
National cohort study of preoperative Bacteriuria, surgical prophylaxis, and postoperative outcomes	Salazar J.G.; Strymish J.; Branch-Elliman W.; Itani K.; O'Brien W.; Gupta K.	2017
The efficacy and safety of using extension catheters in complex coronary interventions: A single center experience	Huang M.-S.; Wu C.-I.; Chang F.-H.; Chang H.-Y.; Lee P.-T.; Chen J.-Y.; Lee W.-H.; Lin C.-C.; Chan S.-H.; Liu P.-Y.; Lee C.-H.	2017
Effect of morbid obesityon mid-urethral sling effecacy forthe managementofstress urinary incontinence	ElShatanoufy S.; Wang Y.; Matthews A.; Jamil M.L.; Yousif M.; Gutta S.; Gill H.; Luck A.	2017
Logothetopulos: Control of intraoperative hemorrhage with pelvic pressure pack	Gonzalez J.; Davidson S.A.; Muffly T.M.	2017
A multi-centre analysis of antibiotic susceptibility of urinary tract isolates in patients with ureteric stents and associated UTI	McPhee A.; Smyth N.; Hinckley M.; Ho W.; Clark D.; Jones B.; Cottom L.	2017
Survey of consultants in restorative dentistry in the UK regarding ongoing care of patients provided with dental implants	Beddis H.P.; Durey K.A.; Chan M.F.W.Y.	2017
Peri-implant soft-tissue parameters and crestal bone levels among narghile smokers and nonsmokers	Abduljabbar T.; Al-Hamoudi N.; AlQunayan M.; AlAhmari A.; AlMalki A.; Gholamiazizi E.; Vohra F.	2017
Chitosan brush for professional removal of plaque in mild peri-implantitis	Zeza B.; Wohlfahrt C.; Pilloni A.	2017
Effects of uncontrolled periodontitis on marginal bone alterations around implants: A case-control study	Wang X.; Qin L.; Lei C.; Li Y.; Li D.	2017
Gene expression in peri-implant crevicular fluid of smokers and nonsmokers. 1. The early phase of osseointegration	Sayardoust S.; Omar O.; Thomsen P.	2017
Comparison of different maintenance strategies within supportive implant therapy for prevention of peri-implant inflammation during the first year after implant restoration. A randomized, dental hygiene practice-based multicenter study	Ziebolz D.; Klipp S.; Schmalz G.; Schmickler J.; Rinke S.; Kottmann T.; Fresmann S.; Einwag J.	2017
Does osteoporosis influence the marginal peri-implant bone level in female patients? A cross-sectional study in a matched collective	Wagner F.; Schuder K.; Hof M.; Heuberer S.; Seemann R.; Dvorak G.	2017
Mesenchymal stromal cell and osteoblast responses to oxidized titanium surfaces pre-treated with λ = 808 nm GaAlAs diode laser or chlorhexidine: in vitro study	Chellini F.; Giannelli M.; Tani A.; Ballerini L.; Vallone L.; Nosi D.; Zecchi-Orlandini S.; Sassoli C.	2017
Insufficient evidence about benefits of antimicrobial photodynamic therapy as an adjunct to mechanic curettage in smokers with peri-implant mucositis	Brignardello-Petersen R.	2017
Combination of straight and tilted implants for supporting screw-retained dental prostheses in atrophic posterior maxillae: A 2-year prospective study	Casar-Espinosa J.-C.; Castillo-Oyagüe R.; Serrera-Figallo M.Á.; Garrido-Serrano R.; Lynch C.D.; Menéndez-Collar M.; Torres-Lagares D.; Gutiérrez-Pérez J.-L.	2017

Dental implants in the elderly population: a systematic review and meta-analysis	Srinivasan M.; Meyer S.; Mombelli A.; Müller F.	2017
Eight-year clinical results of SLA surface implant for dentition defect	Ren M.-M.; Liu L.-P.; Liu Z.-H.	2017
Position paper for the evaluation and management of oral status in patients with valvular disease: Groupe de Travail Valvulopathies de la Société Française de Cardiologie, Société Française de Chirurgie Orale, Société Française de Parodontologie et d'Implantologie Orale, Société Française d'Endodontie et Société de Pathologie Infectieuse de Langue Française	Millot S.; Lesclous P.; Colombier M.-L.; Radoi L.; Messeca C.; Ballanger M.; Charrier J.-L.; Tramba P.; Simon S.; Berrebi A.; Doguet F.; Lansac E.; Tribouilloy C.; Habib G.; Duval X.; Iung B.	2017
Immediate and delayed loading of two-piece reduced-diameter implants with locator-analog attachments in edentulous mandibles: One-year results from a randomized clinical trial examining clinical outcome and patient expectation	Giannakopoulos N.N.; Ariaans K.; Eberhard L.; Klotz A.-L.; Oh K.; Kappel S.	2017
Risk factors for single crowns supported by short (6-mm) implants in the posterior region: A prospective clinical and radiographic study	Villarinho E.A.; Triches D.F.; Alonso F.R.; Mezzomo L.A.M.; Teixeira E.R.; Shinkai R.S.A.	2017
Dimensional alterations following vertical ridge augmentation using collagen membrane and three types of bone grafting materials: A retrospective observational study	Park Y.-H.; Choi S.-H.; Cho K.-S.; Lee J.-S.	2017
Outcomes in a resource-constrained economy: Results from the multi-centre South African SHARE-TAVI registry	Weich H.; Scherman J.; Schaafsma E.; Ntsekhe M.	2017
Contribution of post-stent irregular protrusion to in-stent neoatheroscrelosis after second-generation drug-eluting stent implantation. - Optical coherence tomography study-	Sanuki Y.; Shinjo S.; Shimizu A.; Takami H.; Anai R.; Muraoka Y.; Tsuda Y.; Araki M.; Otsuji Y.	2017
Oral infection with Porphyromonas gingivalis induces peri-implantitis in a murine model: Evaluation of bone loss and the local inflammatory response	Tzach-Nahman R.; Mizraji G.; Shapira L.; Nussbaum G.; Wilensky A.	2017
Basis of bone metabolism around dental implants during osseointegration and peri-implant bone loss	Insua A.; Monje A.; Wang H.-L.; Miron R.J.	2017
Non-surgical treatment of peri-implant mucositis and peri-implantitis at two-piece zirconium implants: A clinical follow-up observation after up to 3 years	John G.; Becker J.; Schmucker A.; Schwarz F.	2017
Clinical Performance of Dental Implants with a Moderately Rough (TiUnite) Surface: A Meta-Analysis of Prospective Clinical Studies	Karl M.; Albrektsson T.	2017
Comparison of Clinical, Radiographic, and Immunologic Inflammatory Parameters Around Dental Implants with Cement-Retained and Screw-Retained Restorations: A 5-Year Prospective Cohort Study in Men	Al Amri M.D.; Al-Rasheed A.S.; Al-Kheraif A.A.; Alfadda S.A.	2017
Peri-implant and Paracrestal Inflammatory Biomarkers at Failing Versus Surviving Implant Sites in a Beagle Dog Study	Montero J.; Aragón F.; Blanco L.A.; Guadilla Y.; García-Cenador B.; López-Valverde A.	2017
Effects of Two Diode Lasers With and Without Photosensitization on Contaminated Implant Surfaces: An Ex Vivo Study	Valente N.A.; Mang T.; Hatton M.; Mikulski L.; Andreana S.	2017
Retrospective Evaluation of the Influence of the Collar Surface Topography on Peri-implant Bone Preservation	Mendonça J.A.; Senna P.M.; Francischone C.E.; Francischone Junior C.E.; de Souza Picorelli Assis N.M.; Sotto-Maior B.S.	2017
Microbial colonization at the implant-abutment interface and its possible influence on periimplantitis: A systematic review and meta-analysis	Tallarico M.; Canullo L.; Caneva M.; Özcan M.	2017
Influence of Laser-Microtextured Surface Collar on Marginal Bone Loss and Peri-Implant Soft Tissue Response: A Systematic Review and Meta-Analysis	Chen Z.; Zhang Y.; Li J.; Wang H.-L.; Yu H.	2017
Inflammatory odontogenic cyst on an osseointegrated implant: A peri-implant cyst? New entity proposed	Kochaji N.	2017
Discomfort/pain due to periodontal and peri-implant probing: Implant type and age	Stanner J.; Klum M.; Parvini P.; Zuhr O.; Nickles K.; Eickholz P.	2017
Premature exposure of dental implant cover screws. A retrospective evaluation of risk factors and influence on marginal peri-implant bone level changes	Hertel M.; Roh Y.-C.; Neumann K.; Strietzel F.P.	2017
Bone and Soft Tissue Response in Bone-Level Implants Restored with Platform Switching: A 5-Year Clinical Prospective Study	Lago L.; da Silva L.; Gude F.; Rilo B.	2017
Comparison of periodontal and peri-implant inflammatory parameters among patients with prediabetes, type 2 diabetes mellitus and non-diabetic controls	Abduljabbar T.; Al-Sahaly F.; Al-Kathami M.; Afzal S.; Vohra F.	2017
In vitro effects of dental cements on hard and soft tissues associated with dental implants	Rodriguez L.C.; Saba J.N.; Chung K.-H.; Wadhwani C.; Rodrigues D.C.	2017
Single-staged vs. two-staged implant placement in vertically deficient alveolar ridges using bone ring technique - Part 2: implant osseointegration	Nakahara K.; Haga-Tsujimura M.; Sawada K.; Kobayashi E.; Schaller B.; Saulacic N.	2017
Marginal healing using Polyetheretherketone as healing abutments: an experimental study in dogs	Rea M.; Ricci S.; Ghensi P.; Lang N.P.; Botticelli D.; Soldini C.	2017
Short implants placed with or without grafting into atrophic sinuses: the 5-year results of a prospective randomized controlled study	Nedir R.; Nurdin N.; Abi Najm S.; El Hage M.; Bischof M.	2017
Bone augmentation at peri-implant dehiscence defects comparing a synthetic polyethylene glycol hydrogel matrix vs. standard guided bone regeneration techniques	Thoma D.S.; Jung U.-W.; Park J.-Y.; Bienz S.P.; Hüsler J.; Jung R.E.	2017
One-year results of maxillary overdentures supported by 2 titanium-zirconium implants - implant survival rates and radiographic outcomes	Zembic A.; Tahmaseb A.; Jung R.E.; Wismeijer D.	2017
Use of platelet-rich fibrin in regenerative dentistry: a systematic review	Miron R.J.; Zucchelli G.; Pikos M.A.; Salama M.; Lee S.; Guillemette V.; Fujioka-Kobayashi M.; Bishara M.; Zhang Y.; Wang H.-L.; Chandad F.; Nacopoulos C.; Simonpieri A.; Aalam	2017

	A.A.; Felice P.; Sammartino G.; Ghanaati S.; Hernandez M.A.; Choukroun J.	
Peri-Prosthetic Knee Infection Management: Spacers Loaded with Two or Three Antibiotic Agents	Ortola D.J.; Fenga D.; Marcellino S.; Rosi M.; Centofanti F.; Rosa M.A.	2017
Anaesthesia and peri-interventional morbidity of rigid bronchoscopy: A retrospective analysis	Tan J.; Kirthinanda D.; Yu D.E.; Poopalalingam R.; Kothandan H.	2017
Practice variations in addressing acute tandem carotid occlusions in emergent large vessel occlusion strokes	Coffman S.; Tro S.; Alhajer A.; Fraser J.	2017
Local infiltration analgesia or femoral nerve block for postoperative pain management in patients undergoing total hip arthroplasty. A randomized, double-blind study	Kuchálik J.; Magnuson A.; Lundin A.; Gupta A.	2017
Transcatheter closure of the patent ductus arteriosus in premature infants is an effective alternative to video assisted thoracoscopic surgical closure	Breinholt J.; Wang E.; Srinivasan C.; Douglas W.	2017
Myeloperoxidase Mediates Postischemic Arrhythmogenic Ventricular Remodeling	Mollenhauer M.; Friedrichs K.; Lange M.; Gesenberg J.; Remane L.; Kerkenpaß C.; Krause J.; Schneider J.; Ravekes T.; Maass M.; Halbach M.; Peinkofer G.; Saric T.; Mehrkens D.; Adam M.; Deuschl F.G.; Lau D.; Geertz B.; Manchanda K.; Eschenhagen T.; Kubala L.; Rudolph T.K.; Wu Y.; Tang W.H.W.; Hazen S.L.; Baldus S.; Klinke A.; Rudolph V.	2017
AUM voice prosthesis: A $1 novel modified tracheo-esophageal voice prosthesis for total laryngectomy patients (TEP)	Rao V.U.S.; Chatterjee S.	2017
Quality assessment of systematic reviews of the significance of keratinized mucosa on implant health	Moraschini V.; Luz D.; Velloso G.; Barboza E.D.P.	2017
Augmentation of keratinized gingiva around dental implants	Kissa J.; El Kholti W.; Laalou Y.; El Farouki M.	2017
Bacterial colonization of the implant-Abutment interface of conical connection with an internal octagon: An in vitro study using real-Time PCR	Baj A.; Beltramini G.A.; Bolzoni A.; Cura F.; Palmieri A.; Scarano A.; Ottria L.; Gianni A.B.	2017
Osteoblast growth, after cleaning of biofilm-covered titanium discs with air-polishing and cold plasma	Matthes R.; Duske K.; Kebede T.G.; Pink C.; Schlüter R.; von Woedtke T.; Weltmann K.-D.; Kocher T.; Jablonowski L.	2017
Cement-associated peri-implant mucositis. A 1-year follow-up after excess cement removal on the peri-implant tissue of dental implants	Korsch M.; Walther W.; Bartols A.	2017
Immediate implant and rehabilitation based on All-on-4 concept in patients with generalized aggressive periodontitis: A medium-term prospective study	Li S.; Di P.; Zhang Y.; Lin Y.	2017
Dental Implant Survival and Complication Rate over 2 Years for Individuals with Poorly Controlled Type 2 Diabetes Mellitus	Eskow C.C.; Oates T.W.	2017
Immediate loading of short implants in posterior maxillae: Case series	Anitua E.	2017
Chronic periodontitis and implant dentistry	Smith M.M.; Knight E.T.; Al-Harthi L.; Leichter J.W.	2017
How to treat two adjacent missing teeth with dental implants. A systematic review on single implant-supported two-unit cantilever FDP's and results of a 5-year prospective comparative study in the aesthetic zone	Van Nimwegen W.G.; Raghoebar G.M.; Tymstra N.; Vissink A.; Meijer H.J.A.	2017
Graft-Free Maxillary Sinus Floor Elevation: A Systematic Review and Meta-Analysis	Duan D.-H.; Fu J.-H.; Qi W.; Du Y.; Pan J.; Wang H.-L.	2017
A Comparative Analysis on Two Types of Oral Implants, Bone-Level and Tissue-Level, with Different Cantilever Lengths of Fixed Prosthesis	Mosavar A.; Nili M.; Hashemi S.R.; Kadkhodaei M.	2017
Long-term clinical and prosthetic outcomes of soft liner and clip attachments for bar/implant overdentures: a randomised controlled clinical trial	ELsyad M.A.; Shaheen N.H.; Ashmawy T.M.	2017
Bidirectional flux of fluids & microbiota at implant-Abutment connection of FMD storm implant system: An in vitro study using RT-PCR	Baj A.; Romano M.; Segna E.; Palmieri A.; Cura F.; Scarano A.; Ottria L.; Gianni A.B.	2017
In silico identification of a therapeutic target for photo-activated disinfection with indocyanine green: Modeling and virtual screening analysis of Arg-gingipain from Porphyromonas gingivalis	Pourhajibagher M.; Bahador A.	2017
Wound haematoma following defibrillator implantation: Incidence and predictors in the Shockless Implant Evaluation (SIMPLE) trial	Masiero S.; Connolly S.J.; Birnie D.; Neuzner J.; Hohnloser S.H.; Vinolas X.; Kautzner J.; O'Hara G.; Vanerven L.; Gadler F.; Wang J.; Mabo P.; Glikson M.; Kutyifa V.; Wright D.J.; Essebag V.; Healey J.S.	2017
The use of bacteriophages to biocontrol oral biofilms	Szafrański S.P.; Winkel A.; Stiesch M.	2017
Survival/Success of Dental Implants with Acid-Etched Surfaces: A Retrospective Evaluation After 8 to 10 Years	Nicoli L.G.; Oliveira G.J.P.L.; Lopes B.M.V.; Marcantonio C.; Zandim-Barcelos D.L.; Marcantonio E.	2017
Survival Rates and Bone and Soft Tissue Level Changes Around One-Piece Dental Implants Placed with a Flapless or Flap Protocol: 8.5-Year Results	Froum S.J.; Khouly I.	2017
Peri-implant Soft Tissue Stability After Single Implant Restorations Using Either Guided Bone Regeneration or a Connective Tissue Graft: A Randomized Clinical Trial	D'Elia C.; Baldini N.; Cagidiaco E.F.; Nofri G.; Goracci C.; de Sanctis M.	2017
Various Tip Applications and Temperature Changes of Er,Cr:YSGG-Laser Irradiated Implants In Vitro	Romanos G.E.; Montanaro N.J.; Sacks D.; Miller R.J.; Javed F.; Calvo-Guirado J.L.; Delgado-Ruiz R.A.	2017
Radiopacity of Luting Cements as a Potential Factor in Peri-implantitis: An In Vitro Comparative Study	Cal E.; Guneri P.; Unal S.; Türk A.G.; Ulusoy M.; Boyacioglu H.	2017
Oral Rehabilitation of a Patient With Ectodermal Dysplasia Treated With Fresh-Frozen Bone Allografts and Computer-Guided Implant Placement: A Clinical Case Report	Maiorana C.; Poli P.P.; Poggio C.; Barbieri P.; Beretta M.	2017

Carbon fibre versus metal framework in full-arch immediate loading rehabilitations of the maxilla - a cohort clinical study	Pera F.; Pesce P.; Solimano F.; Tealdo T.; Pera P.; Menini M.	2017
Interprosthetic and Peri-Implant Fractures: Principles of Operative Fixation and Future Directions	Liporace F.A.; Yoon R.S.; Collinge C.A.	2017
Erbium, Chromium:Yttrium-Scandium-Gallium-Garnet Laser Effectively Ablates Single-Species Biofilms on Titanium Disks Without Detectable Surface Damage	Strever J.M.; Lee J.; Ealick W.; Peacock M.; Shelby D.; Susin C.; Mettenberg D.; El-Awady A.; Rueggeberg F.; Cutler C.W.	2017
Treatment Outcomes for Mandibular Mini-Implant-Retained Overdentures: A Systematic Review	Park J.-H.; Lee J.-Y.; Shin S.-W.	2017
Expansion of the peri-implant attached gingiva with a three-dimensional collagen matrix in head and neck cancer patients-results from a prospective clinical and histological study	Lorenz J.; Blume M.; Barbeck M.; Teiler A.; Kirkpatrick C.J.; Sader R.A.; Ghanaati S.	2017
Comparison of Marginal Bone Level Changes of Immediately Loaded Implants, Delayed Loaded Nonsubmerged Implants, and Delayed Loaded Submerged Implants: A Randomized Clinical Trial	Giacomel M.C.; Camati P.; Souza J.; Deliberador T.	2017
Retrospective Cohort Study Examining Reduced Intensity and Duration of Anticoagulant and Antiplatelet Therapy Following Left Atrial Appendage Occlusion with the WATCHMAN Device	Tung M.K.; Ramkumar S.; Cameron J.D.; Pang B.; Nerlekar N.; Kotschet E.; Alison J.	2017
Salvage of infected breast implants with continuous periprosthetic antibiotic irrigation: A retrospective audit	Kelsall J.; Gutteridge E.; Whisker L.	2017
Use of a lyophilized bovine pericardium graft to repair tunical defect in patients with Peyronie's disease: Experience in a clinical setting	Otero J.R.; Gómez B.G.; Polo J.M.; Mateo C.P.; Barreras S.G.; Cruz E.G.; De La Riva De La Viña J.; Antolín A.R.	2017
Introduction to the centers for disease control and prevention and healthcare infection control practices advisory committee guideline for prevention of surgical site infection: Prosthetic joint Arthroplasty section	Segreti J.; Parvizi J.; Berbari E.; Ricks P.; Berríos-Torres S.I.	2017
Stent retriever thrombectomywith the mindframe capture LP in peripheral anterior cerebral circulation occlusions	Dobrocky T.; Kurmann R.; Piechowiak E.; Zibold F.; Jung S.; Fischer U.; Arnold M.; Gralla J.; Mordasini P.	2017
Interventions for replacing missing teeth: Implant placement at different levels in relation to crestal bone	Khouly I.; Veitz-Keenan A.; Loomer P.M.; Esposito M.	2017
Long-Term Effect of Surface Roughness and Patients' Factors on Crestal Bone Loss at Dental Implants. A Systematic Review and Meta-Analysis	Doornewaard R.; Christiaens V.; De Bruyn H.; Jacobsson M.; Cosyn J.; Vervaeke S.; Jacquet W.	2017
Dental implants: Assessment of pain, discomfort, and altered sensations	Vera Resendiz C.; LaPrade J.; Bair E.; Hicks M.; Albury T.; Salo P.; Khan A.; Cooper L.; Maixner W.	2017
Altered expectations on dental implant therapy; views of patients referred for treatment of peri-implantitis	Abrahamsson K.H.; Wennström J.L.; Berglundh T.; Abrahamsson I.	2017
Influence of maintenance care in periodontally susceptible and non-susceptible subjects following implant therapy	Tan W.C.; Ong M.M.; Lang N.P.	2017
Immediate versus delayed loading of implant for replacement of missing mandibular first molar: A randomized prospective six years clinical study	Chidagam P.R.L.V.; Gande V.C.; Yadlapalli S.; Venkata R.Y.; Kondaka S.; Chedalawada S.	2017
Implant-supported removable partial dentures in the mandible: A 3-16 year retrospective study	Jensen C.; Meijer H.J.A.; Raghoebar G.M.; Kerdijk W.; Cune M.S.	2017
The effect of one-time abutment placement on interproximal bone levels and peri-implant soft tissues: a prospective randomized clinical trial	Molina A.; Sanz-Sánchez I.; Martín C.; Blanco J.; Sanz M.	2017
Esthetic Assessment of Implants Placed into Fresh Extraction Sockets for Single-Tooth Replacements Using a Flapless Approach	Kolerman R.; Mijiritsky E.; Barnea E.; Dabaja A.; Nissan J.; Tal H.	2017
Comparative Clinical Study of Conventional Dental Implants and Mini Dental Implants for Mandibular Overdentures: A Randomized Clinical Trial	Aunmeungtong W.; Kumchai T.; Strietzel F.P.; Reichart P.A.; Khongkhunthian P.	2017
A Retrospective Survival Study of Trabecular Tantalum Implants Immediately Placed in Posterior Extraction Sockets Using a Flapless Technique	El Chaar E.; Castaño A.	2017
The NobelGuide® All-on-4® Treatment Concept for Rehabilitation of Edentulous Jaws: A Retrospective Report on the 7-Years Clinical and 5-Years Radiographic Outcomes	Lopes A.; Maló P.; de Araújo Nobre M.; Sánchez-Fernández E.; Gravito I.	2017
Effect of a Connective Tissue Graft in Combination With a Single Flap Approach in the Regenerative Treatment of Intraosseous Defects	Trombelli L.; Simonelli A.; Minenna L.; Rasperini G.; Farina R.	2017
Mesenchymal stem cells in the aseptic loosening of total joint replacements	Pajarinen J.; Lin T.-H.; Nabeshima A.; Jämsen E.; Lu L.; Nathan K.; Yao Z.; Goodman S.B.	2017
Phage therapy: A new horizon in the antibacterial treatment of oral pathogens	Shlezinger M.; Khalifa L.; Houri-Haddad Y.; Coppenhagen-Glazer S.; Resch G.; Que Y.-A.; Beyth S.; Dorfman E.; Hazan R.; Beyth N.	2017
Biofilm-mediated antibiotic-resistant oral bacterial infections: Mechanism and combat strategies	Kanwar I.; Sah A.K.; Suresh P.K.	2017
Incidence of early right heart failure after left ventricular assist device implantation in patients managed without inhaled nitric oxide	Michaud C.; Marco G.; Schaap A.; Dickinson M.; Wilton P.	2017
Outcome of knee revisions for osteoarthritis and inflammatory arthritis with postero-stabilized arthroplasties: a mean ten-year follow-up with 90 knee revisions	Hernigou P.; Dubory A.; Potage D.; Roubineau F.; Flouzat-Lachaniette C.H.	2017
Prevalence and risk factors for peri-implant diseases in Japanese adult dental patients	Ogata Y.; Nakayama Y.; Tatsumi J.; Kubota T.; Sato S.; Nishida T.; Takeuchi Y.; Onitsuka T.; Sakagami R.; Nozaki T.; Murakami S.; Matsubara N.; Tanaka M.; Yoshino T.; Ota J.; Nakagawa T.; Ishihara Y.; Ito T.; Saito A.; Yamaki K.; Matsuzaki E.; Hidaka T.; Sasaki D.; Yaegashi T.; Yasuda T.; Shibutani T.; Noguchi K.; Araki H.; Ikumi N.; Aoyama Y.; Kogai H.; Nemoto K.; Deguchi S.; Takiguchi T.; Yamamoto M.; Inokuchi K.; Ito T.; Kado T.; Furuichi Y.; Kanazashi M.; Gomi K.; Takagi Y.; Kubokawa K.;	2017

	Yoshinari N.; Hasegawa Y.; Hirose T.; Sase T.; Arita H.; Kodama T.; Shin K.; Izumi Y.; Yoshie H.	
Implant maintenance treatment and peri-implant health	Howe M.-S.	2017
Prevalence of peri-implantitis in patients not participating in well-designed supportive periodontal treatments: a cross-sectional study	Rokn A.; Aslroosta H.; Akbari S.; Najafi H.; Zayeri F.; Hashemi K.	2017
Histologic Evaluation of Sinus Grafting Materials After Peri-implantitis-Induced Failure: A Case Series	Scarano A.; Cholakis A.K.; Piattelli A.	2017
Short Dental Implants Retaining Two-Implant Mandibular Overdentures in Very Old, Dependent Patients: Radiologic and Clinical Observation Up to 5 Years	Maniewicz S.; Buser R.; Duvernay E.; Vazquez L.; Loup A.; Perneger T.V.; Schimmel M.; Müller F.	2017
Implant outcomes poorer in patients with history of periodontal disease	Veitz-Keenan A.; Keenan J.R.	2017
Comparison of clinical and radiographic status around immediately loaded versus conventional loaded implants placed in patients with type 2 diabetes: 12- and 24-month follow-up results	Al Amri M.D.; Alfarraj Aldosari A.M.; Al-Johany S.S.; Al Baker A.M.; Al Rifaiy M.Q.; Al-Kheraif A.A.	2017
Hybrid Implants in Healthy and Periodontally Compromised Patients: A Preliminary Clinical and Radiographic Study	Spinato S.; Bernardello F.; Sassatelli P.; Zaffe D.	2017
Titanium surface dependent regulation of bone remodeling in osseointegration implicates semaphorins	Lotz E.M.; Berger M.B.; Hyzy S.L.; Schwartz Z.; Boyan B.D.	2017
Outcome assessment of All-on-four and All-on-six implant restoration in edentulous patients in Indian population	Kumar K.A.	2017
A randomized controlled clinical trial comparing small buccal dehiscence defects around dental implants treated with guided bone regeneration or left for spontaneous healing	Jung R.E.; Herzog M.; Wolleb K.; Ramel C.F.; Thoma D.S.; Hämmerle C.H.	2017
Feasibility of implant treatment provided by public health insurance in our hospital	Yanai C.; Hiraga Y.; Koresawa K.; Kuraji M.; Ishii M.; Oshima M.; Yamada M.; Ogura S.	2017
Retrospective evaluation of bone graft and dental implants survival after combined one step double-team endoscopic sinus surgery and sinus floor elevation procedure	Pesis M.; Koren I.; Safadi A.; Reiser V.; Benjamin S.; Shuster A.; Kleinman S.	2017
Immediate postextraction dental implantation with fixation of implant to buccal and palatal bone wall of tooth socket using miniplates (case report of 10 years of success)	Farajov E.; Arkhmammadov A.; Ibrahimov R.	2017
Compatible CAD-CAM titanium abutments for posterior single-implant tooth replacement: A retrospective case series	Hsu K.-W.; Shen Y.-F.; Wei P.-C.	2017
Definition, etiology, prevention and treatment of periimplantitis	Bošnjak A.	2017
Outcomes of implant therapy in patients with a history of aggressive periodontitis. A systematic review and meta-analysis	Theodoridis C.; Grigoriadis A.; Menexes G.; Vouros I.	2017
Minimally invasive technique for preservation of stabile periimplant soft tissues after immediate implant placement - A clinical study	Smojver I.; Illeš D.; Sušic M.; Gabric D.	2017
Developing a preclinical model of hematogenous implant infection	Mills R.; Cheng T.; Mikulec K.; Peacock L.; Little D.; Schindeler A.	2017
Vertical Ridge Augmentation in the Atrophic Mandible: A Systematic Review and Meta-Analysis	Elnayef B.; Monje A.; Gargallo-Albiol J.; Galindo-Moreno P.; Wang H.-L.; Hernández-Alfaro F.	2017
Results of morphological studies on the effectiveness of dental implantation using low-intensity pulsed ultrasound	Khomich I.; Rubnikovich S.; Khomich S.; Khomich A.	2017
Burden of noninfectious inflammatory eye diseases: A systematic literature review	Rice J.; White A.; Scarpati L.; Philbin M.; Wan G.; Nelson W.	2017
Liposomal bupivacaine in total hip arthroplasty: Do the results justify the cost?	Beachler J.A.; Kopolovich D.M.; Tubb C.C.; Sayeed S.A.	2017
A rare case of persistently recurrent hyperkalemia in end stage renal disease (ESRD) following bio prosthetic aortic valve replacement - Could the new potassium-binding polymer, patiromer, have been useful here?	Onuigbo M.; Agbasi N.	2017
Barriers to Early Discharge after Elective Percutaneous Coronary Intervention (BED PCI): A Single-Center Study	Graziano F.D.; Banga S.; Busman D.K.; Muthusamy P.; Wohns D.H.	2017
Reliability of periodontal diagnostic tools for monitoring peri-implant health and disease	Coli P.; Christiaens V.; Sennerby L.; Bruyn H.	2017
Implant surface roughness and patient factors on long-term peri-implant bone loss	De Bruyn H.; Christiaens V.; Doornewaard R.; Jacobsson M.; Cosyn J.; Jacquet W.; Vervaeke S.	2017
Comparison of clinical and radiographic status around dental implants placed in patients with and without prediabetes: 1-year follow-up outcomes	Al Amri M.D.; Abduljabbar T.S.; Al-Kheraif A.A.; Romanos G.E.; Javed F.	2017
Risk indicators for Peri-implantitis. A cross-sectional study with 916 implants	Dalago H.R.; Schuldt Filho G.; Rodrigues M.A.; Renvert S.; Bianchini M.A.	2017
Occlusal trauma and mucositis or peri-implantitis?	Passanezi E.; Sant'Ana A.C.; Damante C.A.	2017
Osseointegration of titanium, titanium alloy and zirconia dental implants: current knowledge and open questions	Bosshardt D.D.; Chappuis V.; Buser D.	2017
Stock Versus CAD/CAM Customized Zirconia Implant Abutments - Clinical and Patient-Based Outcomes in a Randomized Controlled Clinical Trial	Schepke U.; Meijer H.J.; Kerdijk W.; Raghoebar G.M.; Cune M.	2017
Enterococcus faecalis and Dental Implants	Flanagan D.	2017
Bone Remodeling Around Implants with External Hexagon and Morse-Taper Connections: A Randomized, Controlled, Split-Mouth, Clinical Trial	Pessoa R.S.; Sousa R.M.; Pereira L.M.; Neves F.D.; Bezerra F.J.; Jaecques S.V.; Sloten J.V.; Quirynen M.; Teughels W.; Spin-Neto R.	2017

Use of cone beam computed tomography in implant dentistry: current concepts, indications and limitations for clinical practice and research	Bornstein M.M.; Horner K.; Jacobs R.	2017
Titanium ions form particles that activate and execute interleukin-1β release from lipopolysaccharide-primed macrophages	Pettersson M.; Kelk P.; Belibasakis G.N.; Bylund D.; Molin Thorén M.; Johansson A.	2017
Coverage of mucosal recessions at dental implants	Sculean A.; Chappuis V.; Cosgarea R.	2017
Modern implant dentistry based on osseointegration: 50 years of progress, current trends and open questions	Buser D.; Sennerby L.; De Bruyn H.	2017
In vitro cleaning potential of three implant debridement methods. Simulation of the non-surgical approach	Ronay V.; Merlini A.; Attin T.; Schmidlin P.R.; Sahrmann P.	2017
Guided bone regeneration and abutment connection augment the buccal soft tissue contour: 3-year results of a prospective comparative clinical study	Benic G.I.; Ge Y.; Gallucci G.O.; Jung R.E.; Schneider D.; Hämmerle C.H.	2017
Effects of Porphyromonas gingivalis on Titanium Surface by Different Clinical Treatment	Batsukh N.; Feng S.W.; Lee W.F.; Leu S.-J.; Tsai P.-Y.; Ho K.-N.; Lin C.T.; Su C.-H.; Chang W.-J.	2017
Subgingival airpolishing: A simple and cost effective medical insurance	Karmakar S.; Kamath D.G.	2017
Long-term Evaluation of Peri-implant Bone Level after Reconstruction of Severely Atrophic Edentulous Maxilla via Vertical and Horizontal Guided Bone Regeneration in Combination with Sinus Augmentation: A Case Series with 1 to 15 Years of Loading	Urban I.A.; Monje A.; Lozada J.L.; Wang H.-L.	2017
Patient-reported outcomes of implant placement performed concomitantly with transcrestal sinus floor elevation or entirely in native bone	Franceschetti G.; Rizzi A.; Minenna L.; Pramstraller M.; Trombelli L.; Farina R.	2017
Outcomes of 6.5-mm Hydrophilic Implants and Long Implants Placed with Lateral Sinus Floor Elevation in the Atrophic Posterior Maxilla: A Prospective, Randomized Controlled Clinical Comparison	Yu H.; Wang X.; Qiu L.	2017
Comparison of clinical and radiographic status of platform-switched implants placed in patients with and without type 2 diabetes mellitus: a 24-month follow-up longitudinal study	Al Amri M.D.; Abduljabbar T.S.	2017
Stability and Crestal Bone Behavior Following Simultaneous Placement of Multiple Dental Implants (Two or More) with the Bone Splitting Technique: A Clinical and Radiographic Evaluation	Gehrke S.A.; Maté Sánchez de Val J.E.; Ramírez Fernández M.P.; Shibli J.A.; Rossetti P.H.; Calvo Guirado J.L.	2017
Implant-retained prostheses: ball vs. conus attachments - A randomized controlled clinical trial	Cepa S.; Koller B.; Spies B.C.; Stampf S.; Kohal R.-J.	2017
Lateral Ridge Augmentation Using Autogenous Block Grafts and Guided Bone Regeneration: A 10-Year Prospective Case Series Study	Chappuis V.; Cavusoglu Y.; Buser D.; von Arx T.	2017
Ridge Dimensions of the Edentulous Mandible in Posterior Sextants: An Observational Study on Cone Beam Computed Tomography Radiographs	Bressan E.; Ferrarese N.; Pramstraller M.; Lops D.; Farina R.; Tomasi C.	2017
Periprocedural stroke and myocardial infarction as risks for long-term mortality in CREST	Jones M.R.; Roubin G.S.; Clark W.M.; Mackey A.; Blackshear J.; Hill M.D.; Cohen D.J.; Hughes S.E.; Voeks J.H.; Meschia J.F.; Brott T.G.	2017
Interim report of the weave trial: First 102 consecutive on label patients	Alexander M.J.; Chaloupka J.C.; Zauner A.; Baxter B.; Callison R.; Yu W.	2017
Biodegradable Magnesium Screws Accelerate Fibrous Tissue Mineralization at the Tendon-Bone Insertion in Anterior Cruciate Ligament Reconstruction Model of Rabbit	Wang J.; Xu J.; Fu W.; Cheng W.; Chan K.; Yung P.S.-H.; Qin L.	2017
Long-term prospective cohort study on dental implants: clinical and microbiological parameters	Tenenbaum H.; Bogen O.; Séverac F.; Elkaim R.; Davideau J.-L.; Huck O.	2017
Evaluation of Metalloproteinase-8 Levels in Crevicular Fluid of Patients with Healthy Implants or Periodontitis	Aleksandrowicz P.; Zelechowska P.; Agier J.; Starska K.; Kędzierski K.; Wysokińska-Miszczuk J.; Brzezińska-Błaszczyk E.	2017
Periimplant disease complications	Sachdeva S.; Saluja H.; Mani A.; Tandon P.	2017
Attributable fractions, modifiable risk factors and risk stratification using a risk score for peri-implant pathology	de Araújo Nobre M.; Mano Azul A.; Rocha E.; Maló P.; Salvado F.	2017
A randomized clinical trial about presence of pathogenic micro-flora and risk of peri-implantitis: Comparison of two different types of implant-abutment connections	Mencio F.; De Angelis F.; Papi P.; Rosella D.; Pompa G.; Di Carlo S.	2017
In Vitro Laser Treatment Platform Construction with Dental Implant Thread Surface on Bacterial Adhesion for Peri-Implantitis	Kuo H.-N.; Mei H.-I.; Liu T.-K.; Liu T.-Y.; Lo L.-J.; Lin C.-L.	2017
Treatment of peri-implantitis: clinical outcome of chloramine as an adjunctive to non-surgical therapy, a randomized clinical trial	Roos-Jansåker A.-M.; Almhöjd U.S.; Jansson H.	2017
Clinicopathologic evaluation of malignancy adjacent to dental implants	Kaplan I.; Zeevi I.; Tal H.; Rosenfeld E.; Chaushu G.	2017
Fixed full arches supported by tapered implants with knife-edge thread design and nanostructured, calcium-incorporated surface: A short-term prospective clinical study	Bechara S.; Lukosiunas A.; Dolcini G.A.; Kubilius R.	2017
The use of short dental implants in rigid fixed bridge for multiple missing teeth: A 2-year follow-up visit	Zhang N.; Xiong Z.-J.; Wang X.-X.; Li H.-M.	2017
Intraoral radiography lacks accuracy for the assessment of peri-implant bone level - a controlled clinical study	Christiaens V.; Jacobs R.; Dierens M.; Vervaeke S.; De Bruyn H.; Koole S.; Cosyn J.	2017
An immediate peri-implantitis induction model to study regenerative peri-implantitis treatments	Park S.-Y.; Kim K.-H.; Rhee S.-H.; Lee J.-C.; Shin S.-Y.; Lee Y.-M.; Seol Y.-J.	2017
Bleeding on probing around dental implants: a retrospective study of associated factors	Farina R.; Filippi M.; Brazzioli J.; Tomasi C.; Trombelli L.	2017
Satisfaction and Clinical Outcomes Among Patients with Immediately Loaded Mandibular Overdentures Supported by One or Two Dental Implants: Results of a 5-Year Prospective Randomized Clinical Trial	Kronstrom M.; Davis B.; Loney R.; Gerrow J.; Hollender L.	2017

Long-Term Results of Peri-implant Conditions in Periodontally Compromised Patients Following Lateral Bone Augmentation	Keeve P.L.; Khoury F.	2017
Comparison of enzyme beta glucuronidase and alkaline phosphatase levels in peri implant sulcular fluid around healthy and diseased implants - A clinical pilot study	Natarajan P.M.; Chandran C.R.; Prabhu P.	2017
Detection of various width and depth of peri-implant crestal bone defects with different imaging modalities	Morgan N.; Aboelmaaty W.; Gadalla L.; Fadil E.A.	2017
Exploring the impact of oral health on quality of life of eating disorders patients	Vecchiattini R.; Manzato E.; Mobilio N.; Scardovelli I.; Pisani F.A.; Trombelli L.	2017
Impact of different implant surfaces topographies on peri-implant tissues: An update of current available data on dental implants retrieved from human jaws	Shibli J.A.; Pires J.T.; Piattelli A.; Iezzi G.; Mangano C.; Mangano F.; de Souza S.L.S.; Gehrke S.A.; Wang H.-L.; Dohan Ehrenfest D.M.	2017
Local complications of dental implant treatment	Matej A.; Željko R.; Boras V.; Gabrić D.; Terlevic D.; Pelivan I.; Ana A.-R.	2017
Complex prophylaxis and prognosis of inflammatory complications after dental implantation	Mustafaev M.Sh.; Kharaeva Z.F.; Vissarionov V.A.; Mustafaeva F.M.; Zhigunov A.K.; Atmurzaev M.M.	2017
Evaluation of Ultrashort and Longer Implants with Microrough Surfaces: Results of a 24- to 36-Month Prospective Study	Malchiodi L.; Caricasulo R.; Cucchi A.; Vinci R.; Agliardi E.; Gherlone E.	2017
Immediate, immediate-delayed (6 weeks) and delayed (4 months) post-extractive single implants: 1-year post-loading data from a randomised controlled trial	Esposito M.; Zucchelli G.; Cannizzaro G.; Checchi L.; Barausse C.; Trullenque-Eriksson A.; Felice P.	2017
Soft tissue augmentation around osseointegrated and uncovered dental implants: a systematic review	Bassetti R.G.; Stähli A.; Bassetti M.A.; Sculean A.	2017
Clinical study of flapless immediate implant placement and immediate restoration of single tooth in the oral aesthetic region	Zhang B.; Zhou Y.; Cai Q.; Li B.; Yin C.; Zhao Y.; Meng W.	2017
Subcrestal placement of dental implants with an internal conical connection of 0.5 mm versus 1.5 mm: Outcome of a multicentre randomised controlled trial 1 year after loading	Gualini F.; Salina S.; Rigotti F.; Mazzarini C.; Longhin D.; Grigoletto M.; Trullenque-Eriksson A.; Sbricoli L.; Esposito M.	2017
Corrosion behavior of dental implants immersed into human saliva: Preliminary results of an in vitro study	Barbieri M.; Mencio F.; Papi P.; Rosella D.; Di Carlo S.; Valente T.; Pompa G.	2017
Sinus Disventilation and Atrophy of the Upper Maxilla: A Combined Surgical Approach Is Possible?	Cipriani O.; Vellone V.; Arangio P.; Della Rocca F.	2017
Immediate Loading of Single Implants: A 2-Year Prospective Multicenter Study	Mangano C.; Raes F.; Lenzi C.; Eccellente T.; Ortolani M.; Luongo G.; Mangano F.	2017
Development of Implant Stability Quotient values of implants placed with simultaneous sinus floor elevation - results of a prospective study with 109 implants	Kuchler U.; Chappuis V.; Bornstein M.M.; Siewczyk M.; Gruber R.; Maestre L.; Buser D.	2017
The use of hyaluronic acid as an adjuvant in the management of mucositis	Lopez M.A.; Manzulli N.; D'Angelo A.; Candotto V.; Casale M.; Lauritano D.	2017
Tapered, Double-Lead Threads Single Implants Placed in Fresh Extraction Sockets and Healed Sites of the Posterior Jaws: A Multicenter Randomized Controlled Trial with 1 to 3 Years of Follow-Up	Cucchi A.; Vignudelli E.; Franco S.; Levrini L.; Castellani D.; Pagliani L.; Rea M.; Modena C.; Sandri G.; Longhi C.	2017
Four-year post-loading results of full-arch rehabilitation with immediate placement and immediate loading implants: A retrospective controlled study	Simonpieri A.; Gasparro R.; Pantaleo G.; Mignogna J.; Riccitiello F.; Sammartino G.	2017
An open, prospective, non-randomized, controlled, multicentre study to evaluate the clinical outcome of implant treatment in women over 60 years of age with osteoporosis/osteopenia: 1-year results	Temmerman A.; Rasmusson L.; Kübler A.; Thor A.; Quirynen M.	2017
Safety and efficacy of a biomimetic monolayer of permanently bound multiphosphonic acid molecules on dental implants: 3 years post-loading results from a pilot quadruple-blinded randomised controlled trial	Esposito M.; Dojcinovic I.; Buchini S.; Péchy P.; Aronsson B.-O.	2017
Synthesis, coating and in vitro study of antimicrobial copolymers for dental implants	Stelljes S.; Waßmann M.; Winkel A.; Stiesch M.; Menzel H.	2017
Short term sodium alendronate administration improves the peri-implant bone quality in osteoporotic animals	Oliveira D.; Hassumi J.S.; Gomes-Ferreira P.H.; Polo T.O.; Ferreira G.R.; Faverani L.P.; Okamoto R.	2017
A clinical retrospective study of distal extension removable partial denture with implant surveyed bridge or stud type attachment	Bae E.-B.; Kim S.-J.; Choi J.-W.; Jeon Y.-C.; Jeong C.-M.; Yun M.-J.; Lee S.-H.; Huh J.-B.	2017
Do repeated changes of abutments have any influence on the stability of peri-implant tissues? One-year post-loading results from a multicentre randomised controlled trial	Esposito M.; Bressan E.; Grusovin M.G.; D'Avenia F.; Neumann K.; Sbricoli L.; Luongo G.	2017
Prognostic value of a simplified method for periodontal risk assessment during supportive periodontal therapy	Trombelli L.; Minenna L.; Toselli L.; Zaetta A.; Checchi L.; Checchi V.; Nieri M.; Farina R.	2017
Residual root preparation for socket-shield procedures: a facial window approach	Roe P.; Kan J.Y.K.; Rungcharassaeng K.	2017
Dental Implant Surrounding Marginal Bone Level Evaluation: Platform Switching versus Platform Matching - One-Year Retrospective Study	Salamanca E.; Lin J.C.-Y.; Tsai C.-Y.; Hsu Y.-S.; Huang H.-M.; Teng N.-C.; Wang P.D.; Feng S.-W.; Chen M.-S.; Chang W.-J.	2017
Implant success does not differ significantly according to implant type	Coe J.M.	2017
Associated lesions of peri-implant mucosa in immediate versus delayed loading of dental implants	Iliescu A.A.; Zurac S.A.; Nicolae V.; Iliescu M.G.; Perlea P.	2017
Early Bone Formation around Immediately Loaded Transitional Implants Inserted in the Human Posterior Maxilla: The Effects of Fixture Design and Surface	Mangano C.; Shibli J.A.; Pires J.T.; Luongo G.; Piattelli A.; Iezzi G.	2017
Immediate loading of two (fixed-on-2) vs four (fixed-on-4) implants placed with a flapless technique supporting mandibular cross-arch fixed prostheses: 3-year results from a pilot randomised controlled trial	Cannizzaro G.; Felice P.; Gherlone E.; Barausse C.; Ferri V.; Leone M.; Trullenque-Eriksson A.; Esposito M.	2017
Prospective clinical study of prosthetic treatment outcome of implantretained-removable-partial-denture during 5 year-follow-ups	Bahrami M.; Alsharbaty M.H.M.	2017

Reconstruction of horizontovertical alveolar defects. Presentation of a novel split-thickness flap design for guided bone regeneration: A case report with 5-year follow-up	Windisch P.; Martin A.; Shahbazi A.; Molnar B.	2017
Dependence of spatial resolution of cone beam computed tomography on pixel size of flat panel detector	Honda K.; Suzuki T.; Hayashi Y.; Hirai S.; Arai Y.	2017
Post-surgical clinical monitoring of soft tissue wound healing in periodontal and implant surgery	Pippi R.	2017
Technique for placement of peri-implant antibiotics using antibiotic putty	Firoozabadi R.; Miranda S.; Tornetta P.	2017
A comparison of two implants with conical vs internal hex connections: 1-year post-loading results from a multicentre, randomised controlled trial	Cannata M.; Grandi T.; Samarani R.; Svezia L.; Grandi G.	2017
The Mechanical Properties and Biometrical Effect of 3D Preformed Titanium Membrane for Guided Bone Regeneration on Alveolar Bone Defect	Lee S.-H.; Moon J.-H.; Jeong C.-M.; Bae E.-B.; Park C.-E.; Jeon G.-R.; Lee J.-J.; Jeon Y.-C.; Huh J.-B.	2017
Influence of Age on Factors associated with Peri-implant Bone Loss after Prosthetic Rehabilitation over Osseointegrated Implants	Pedro R.E.; De Carli J.P.; Linden M.S.; Lima I.F.; Paranhos L.R.; Costa M.D.; Bós ÂJ.	2017
A clinically relevant in vivo model for the assessment of scaffold efficacy in abdominal wall reconstruction	Chan J.C.Y.; Burugapalli K.; Huang Y.-S.; Kelly J.L.; Pandit A.	2017
Radiographic and Clinical Results of Freehand S2 Alar-Iliac Screw Placement for Spinopelvic Fixation	Park Y.-S.; Hyun S.-J.; Park J.-H.; Kim K.-J.; Jahng T.-A.; Kim H.-J.	2017
Use of non-warfarin oral anticoagulants instead of warfarin during left atrial appendage closure with the Watchman device	Enomoto Y.; Gadiyaram V.K.; Gianni C.; Horton R.P.; Trivedi C.; Mohanty S.; Di Biase L.; Al-Ahmad A.; Burkhardt J.D.; Narula A.; Janczyk G.; Price M.J.; Afzal M.R.; Atoui M.; Earnest M.; Swarup V.; Doshi S.K.; van der Zee S.; Fisher R.; Lakkireddy D.R.; Gibson D.N.; Natale A.; Reddy V.Y.	2017
Novel Oral Anticoagulants in the Peri-Endoscopic Period	Genualdi M.; Lebovics E.; Frishman W.H.	2017
Percutaneous closure of left atrial appendage with a watchman device: A single center study	Bonnet G.; Salaun E.; Deffarges S.; Mouret J.; Cuisset T.; Bonnet J.	2017
ONe swallow does not a summer make but many swallows do: Accumulating clinical evidence for nearly-eliminated peri-procedural and 30-day complications with meshcovered stents transforms the carotid revascularisation field	Musiałek P.; Hopkins L.N.; Siddiqui A.H.	2017
Longterm follow up in patients with mid-urethral slings with BMI greater than 40	Elshatanoufy S.; Griffin M.; Wang Y.; Jamil M.; Matthews A.; Yousif M.; Richardson D.; Atiemo H.O.; Luck A.	2017
Comparison of open and robotic nephron sparing surgery: a single centre experience	Jelley C.R.; Kurukulaarachchi K.A.S.H.; Forster L.; Bardgett H.; Singh R.; Addla S.K.	2017
The impact of prophylactic pancreatic stenting on post-ERCP pancreatitis: A nationwide, register-based study	Olsson G.; Lübbe J.; Arnelo U.; Jonas E.; Törnqvist B.; Lundell L.; Enochsson L.	2017
Left main coronary artery ostial stenosis and pseudoaneurysm diagnosed five years post bentall's procedure and aortic valve replacement, due to new onset cardiac ischaemia in the setting of bleeding	Whitehead N.; Mylabathula S.; Thambar S.	2017
Post-operative medical and surgical complications after primary total joint arthroplasty in solid organ transplant recipients: a case series	Palmisano A.C.; Kuhn A.W.; Urquhart A.G.; Pour A.E.	2017
Dental implantation and soft tissue augmentation after ridge preservation in a molar site: a case report	Zhao L.P.; Zhan Y.L.; Hu W.J.; Wang H.J.; Wei Y.P.; Zhen M.; Xu T.; Liu Y.S.	2016
Should implants be considered for patients with periodontal disease?	King E.; Patel R.; Patel A.; Addy L.	2016
Clinical features of peri-implant medication-related osteonecrosis of the jaw: Is there an association to peri-implantitis?	Troeltzsch M.; Cagna D.; Stähler P.; Probst F.; Kaeppler G.; Troeltzsch M.; Ehrenfeld M.; Otto S.	2016
Peri-Implant Disease--A Significant Complication of Dental Implant Supported Restorative Treatment	Hamada Y.; Shin D.; John V.	2016
Prognostic indicators for surgical peri-implantitis treatment	de Waal Y.C.; Raghoebar G.M.; Meijer H.J.; Winkel E.G.; van Winkelhoff A.J.	2016
Evaluation of bone loss in antibacterial coated dental implants: An experimental study in dogs	Godoy-Gallardo M.; Manzanares-Céspedes M.C.; Sevilla P.; Nart J.; Manzanares N.; Manero J.M.; Gil F.J.; Boyd S.K.; Rodríguez D.	2016
A Novel Treatment Decision Tree and Literature Review of Retrograde Peri-Implantitis	Sarmast N.D.; Wang H.H.; Soldatos N.K.; Angelov N.; Dorn S.; Yukna R.; Iacono V.J.	2016
Effect of the Keratinized Mucosa Width on the Health Status of Periimplant and Contralateral Periodontal Tissues: A Cross-sectional Study	Ueno D.; Nagano T.; Watanabe T.; Shirakawa S.; Yashima A.; Gomi K.	2016
Long-Term Outcomes of Early Loading of Straumann Implant-Supported Fixed Segmented Bridgeworks in Edentulous Maxillae: A 10-Year Prospective Study	Zhang X.-X.; Shi J.-Y.; Gu Y.-X.; Lai H.-C.	2016
Comparison of peri-implant clinical and radiographic inflammatory parameters and whole salivary destructive inflammatory cytokine profile among obese and non-obese men	Abduljabbar T.; Al-Sahaly F.; Kellesarian S.V.; Kellesarian T.V.; Al-Anazi M.; Al-Khathami M.; Javed F.; Vohra F.	2016
Success of dental implants in vascularised fibular osteoseptocutaneous flaps used as onlay grafts after marginal mandibulectomy	Chang Y.-M.; Pan Y.-H.; Shen Y.-F.; Chen J.-K.; ALDeek N.F.; Wei F.-C.	2016
Immediate implant placement and provisionalization in the aesthetic zone using a flapless or a flap-involving approach: a randomized controlled trial	Stoupel J.; Lee C.-T.; Glick J.; Sanz-Miralles E.; Chiuzan C.; Papapanou P.N.	2016
Peri-implant bone loss around platform-switched Morse taper connection implants: a prospective 60-month follow-up study	Cassetta M.; Driver A.; Brandetti G.; Calasso S.	2016
Interest in dental implantology and preferences for implant therapy: a survey of Victorian dentists	Cheung M.C.; Kao P.; Lee N.; Sivathasan D.; Vong C.W.; Zhu J.; Polster A.; Darby I.	2016

Antimicrobial photodynamic therapy of S. mutans biofilms attached to relevant dental materials	Zoccolillo M.L.; Rogers S.C.; Mang T.S.	2016
Light emitting diode mediated photobiomodulation therapy improves orthodontic tooth movement and miniscrew stability: A randomized controlled clinical trial	Ekizer A.; Türker G.; Uysal T.; Güray E.; Taşdemir Z.	2016
Application of 3-Dimensional Computed Tomographic Image Guidance to WATCHMAN Implantation and Impact on Early Operator Learning Curve: Single-Center Experience	Wang D.D.; Eng M.; Kupsky D.; Myers E.; Forbes M.; Rahman M.; Zaidan M.; Parikh S.; Wyman J.; Pantelic M.; Song T.; Nadig J.; Karabon P.; Greenbaum A.; O'Neill W.	2016
Effect of conscious sedation vs general anesthesia on early neurological improvement among patients with ischemic stroke undergoing endovascular thrombectomy: A randomized clinical trial	Schönenberger S.; Uhlmann L.; Hacke W.; Schieber S.; Mundiyanapurath S.; Purrucker J.C.; Nagel S.; Klose C.; Pfaff J.; Bendszus M.; Ringleb P.A.; Kieser M.; Möhlenbruch M.A.; Bösel J.	2016
Feasibility and safety of Reveal LINQ insertion in a sterile procedure room versus electrophysiology laboratory	Wong G.R.; Lau D.H.; Middeldorp M.E.; Harrington J.A.; Stolcman S.; Wilson L.; Twomey D.J.; Kumar S.; Munawar D.A.; Khokhar K.B.; Mahajan R.; Sanders P.	2016
Pro-inflammatory cytokines at zirconia implants and teeth. A cross-sectional assessment	Cionca N.; Hashim D.; Cancela J.; Giannopoulou C.; Mombelli A.	2016
Effect of oral hygiene maintenance on HbA1c levels and peri-implant parameters around immediately-loaded dental implants placed in type-2 diabetic patients: 2 years follow-up	Al Amri M.D.; Kellesarian S.V.; Al-Kheraif A.A.; Malmstrom H.; Javed F.; Romanos G.E.	2016
Assessment of Myeloperoxidase and Nitric Levels around Dental Implants and Natural Teeth as a Marker of Inflammation: A Comparative Study	Kulkarni G.H.; Jadhav P.; Kulkarni K.; Shinde S.V.; Patil Y.B.; Kumar M.	2016
The effects of diode laser on Staphylococcus aureus biofilm and Escherichia coli lipopolysaccharide adherent to titanium oxide surface of dental implants. An in vitro study	Giannelli M.; Landini G.; Materassi F.; Chellini F.; Antonelli A.; Tani A.; Zecchi-Orlandini S.; Rossolini G.M.; Bani D.	2016
Implants in free fibula flap supporting dental rehabilitation – Implant and peri-implant related outcomes of a randomized clinical trial	Kumar V.V.; Ebenezer S.; Kämmerer P.W.; Jacob P.C.; Kuriakose M.A.; Hedne N.; Wagner W.; Al-Nawas B.	2016
Internal- vs External-Connection Single Implants: A Retrospective Study in an Italian Population Treated by Certified Prosthodontists	Vigolo P.; Gracis S.; Carboncini F.; Mutinelli S.	2016
Long-term outcomes after vestibuloplasty with a porcine collagen matrix (Mucograft®) versus the free gingival graft: a comparative prospective clinical trial	Schmitt C.M.; Moest T.; Lutz R.; Wehrhan F.; Neukam F.W.; Schlegel K.A.	2016
A Two-Year Evaluation of a Sloped Marginal Contour Implant System Placed in Healed Sites	Lee P.K.; Siu A.S.	2016
Assessment of Marginal Peri-implant Bone-Level Short-Length Implants Compared with Standard Implants Supporting Single Crowns in a Controlled Clinical Trial: 12-Month Follow-up	Mendoza-Azpur G.; Lau M.; Valdivia E.; Rojas J.; Muñoz H.; Nevins M.	2016
Esthetic assessment of immediately restored implants combined with GBR and free connective tissue graft	Kolerman R.; Nissan J.; Mijiritsky E.; Hamoudi N.; Mangano C.; Tal H.	2016
Clinical, microbiologic and radiologic assessment of soft and hard tissues surrounding zygomatic implants: a retrospective study	Lombardo G.; D'Agostino A.; Trevisiol L.; Romanelli M.G.; Mascellaro A.; Gomez-Lira M.; Pardo A.; Favero V.; Nocini P.F.	2016
Peri-implant bone response to retrieved human zirconia oral implants after a 4-year loading period: A histologic and histomorphometric evaluation of 22 cases	Kohal R.-J.; Schwindling F.S.; Bächle M.; Spies B.C.	2016
Different configuration of socket shield technique in peri-implant bone preservation: An experimental study in dog mandible	Guirado J.L.C.; Troiano M.; López-López P.J.; Ramírez-Fernandez M.P.; de Val J.E.M.S.; Marin J.M.G.; Gehrke S.A.	2016
A Comparison of Tapered and Nontapered Implants in the Minipig	Cochran D.; Stavropoulos A.; Obrecht M.; Pippenger B.; Dard M.	2016
Soft and Hard Tissue Changes Following Immediate Placement or Immediate Restoration of Single-Tooth Implants in the Esthetic Zone: A Systematic Review and Meta-Analysis	Yan Q.; Xiao L.-Q.; Su M.-Y.; Mei Y.; Shi B.	2016
Micromovement Evaluation of Original and Compatible Abutments at the Implant-abutment Interface	Berberi A.; Maroun D.; Kanj W.; Amine E.Z.; Philippe A.	2016
Saving an Integrating Implant Involved with Endodontic Implant Pathology Using Surgical Treatment	Chung S.H.; Park Y.-S.; Bae K.-S.; Baek S.-H.; Kum K.-Y.; Lee W.; Shon W.-J.	2016
Retrospective Cohort Study of 4,591 Straumann Implants Placed in 2,060 Patients in Private Practice with up to 10-Year Follow-up: The Relationship Between Crestal Bone Level and Soft Tissue Condition	French D.; Cochran D.L.; Ofec R.	2016
Orbital and Periorbital Emphysema Following Maxillary Sinus Floor Elevation: A Case Report and Literature Review	Farina R.; Zaetta A.; Minenna L.; Trombelli L.	2016
Current perioperative use of orodental antiseptics in implant surgery in Bulgaria	Shuleva S.; Zkik A.; Chalem Y.	2016
Long term outcomes of same-day discharged patient after percutaneous coronary intervention in stand-alone centers	Blachut A.; Pruski M.; Trendel W.; Janas A.; Kiesz R.S.	2016
Left atrial appendage characteristics evaluated by computed tomography following closure with Watchman device	Kupsky D.; Wang D.D.; Eng M.; Song T.; Pantelic M.; Nadig J.; Greenbaum A.; O'Neill W.	2016
Fate of irregular protrusion after second-generation drug-eluting stent implantation: Serial optical coherence tomography study	Sonoda S.; Sanuki Y.; Muraoka Y.; Takami H.; Shimizu A.; Kitagawa M.; Tsuda Y.; Araki M.; Otsuji Y.	2016
Percutaneous nephrolithotripsy in elderly patients: Assessing perioperative morbidity and one-year renal function	Zaidun S.M.; Nagappan P.; Malek R.	2016
Internal carotid web (atypical fibromuscular dysplasia): High stroke recurrence and amenability to stenting	Hausssen D.; Bouslama M.; Grossberg J.A.; Belagaje S.R.; Allan J.; Pradilla G.; Frankel M.R.; Nogueira R.G.	2016
Impact of a chronic smoking habit on the osteo-immunoinflammatory mediators in the peri-implant fluid of clinically healthy dental implants	Negri B.M.; Pimentel S.P.; Casati M.Z.; Cirano F.R.; Casarin R.C.; Ribeiro F.V.	2016
Changes in Peri-Implant Bone Level and Effect of Potential Influential Factors on Dental Implants in Irradiated and Nonirradiated Patients	Ernst N.; Sachse C.; Raguse J.D.; Stromberger C.; Nelson K.; Nahles S.	2016

Following Multimodal Therapy Due to Head and Neck Cancer: A Retrospective Study		
Management of Peri-implant Diseases	Patel A.	2016
Current status of the influence of osteoporosis on periodontology and implant dentistry	Chambrone L.	2016
Evaluation of peri-implant crevicular fluid prostaglandin E2 levels in augmented extraction sockets by different biomaterials	Alkan E.A.; Tüter G.; Parlar A.; Yücel A.; Kurtiş B.	2016
The Effect of Platform Switching on Periimplant Crevicular Fluid Content During Early Wound Healing	Chien H.-H.; Meng H.-W.; Gross A.C.; Eubank T.D.; Yildiz V.O.; Leblebicioglu B.	2016
Apical/Retrograde Periimplantitis/Implant Periapical Lesion: Etiology, Risk Factors, and Treatment Options: A Systematic Review	Ramanauskaite A.; Juodzbalys G.; Tözüm T.F.	2016
Effect of vertical misfit and clip material on stress distribution of overdentures under masticatory loading	dos Santos M.B.F.; Zen B.M.; Bacchi A.	2016
Controlled release drug delivery systems to improve post-operative pharmacotherapy	Bhusal P.; Harrison J.; Sharma M.; Jones D.S.; Hill A.G.; Svirskis D.	2016
Safety and efficacy of endoscopic ultrasound guided drainage of walled-off pancreatic necrosis using lumen apposing metal stents (hot axios)	Cooper C.J.; Dawwas M.; Mardini H.	2016
Cardiovascular Outcomes Following Rotational Atherectomy: A UK Multicentre Experience	Eftychiou C.; Barmby D.S.; Wilson S.J.; Ubaid S.; Markwick A.J.; Makri L.; Blaxill J.M.; Spratt J.C.; Gunning M.; Greenwood J.P.	2016
Radioactive stent for esophagus carcinoma	Herskovic A.; Rivard M.J.; Hoopes P.J.; Rothstein R.; Moodie K.L.; Hussain Z.; Swanson L.; Strawbridge R.R.; Saunders J.K.; Corcoran K.; Townsend A.; Hingston J.; Clerc C.	2016
Rosuvastatin for Reduction of Myocardial Damage during Coronary Angioplasty - the Remedy Trial	Briguori C.; Madonna R.; Zimarino M.; Calabrò P.; Quintavalle C.; Salomone M.; Condorelli G.; De Caterina R.	2016
Does pre-operative sampling predict intra-operative cultures and antibiotic sensitivities in knee replacements revised for infection?: a study using the NJR dataset	Holleyman R.J.; Deehan D.J.; Charlett A.; Gould K.; Baker P.N.	2016
Acute pancreatitis after removal of retained pancreatic duct stent placed for post-ERCP pancreatitis prophylaxis: A rare complication	Farrell R.; Shirodkar M.; Ho I.K.	2016
Resolution of pancreatopleural fistula with pancreatic duct stent	Sunkara T.; Campo L.; Etienne D.; Upadhyay S.; Babich J.P.	2016
Antibiofilm activity of sandblasted and laser-modified titanium against microorganisms isolated from peri-implantitis lesions	Drago L.; Bortolin M.; De Vecchi E.; Agrappi S.; Weinstein R.L.; Mattina R.; Francetti L.	2016
Effects of Surface Conditions of Titanium Dental Implants on Bacterial Adhesion	Chen C.-J.; Ding S.-J.; Chen C.-C.	2016
Maintaining Osseointegration: Understanding, preventing, and treating Risk Factors for Peri-implant Diseases	Danesh-Sani S.A.	2016
Temperature evaluation of dental implant surface irradiated with high-power diode laser	Rios F.G.; Viana E.R.; Ribeiro G.M.; González J.C.; Abelenda A.; Peruzzo D.C.	2016
A Pilot Study on Mandibular Overdentures Retained By Mini Dental Implants: Marginal Bone Level Changes and Patient-Based Ratings of Clinical Outcome	Zygogiannis K.; Wismeijer D.; Parsa A.	2016
Letter to the Editor: Re: Decontamination of Anodized Implant Surface With Different Modalities for Peri-Implantitis Treatment: Lasers and Mechanical Debridement With Citric Acid	Giannelli M.; Bani D.	2016
Transmucosal Implant Placement with Submarginal Connective Tissue Graft in Area of Shallow Buccal Bone Dehiscence: A Three-Year Follow-Up Case Series	Stefanini M.; Felice P.; Mazzotti C.; Marzadori M.; Gherlone E.F.; Zucchelli G.	2016
A Double-Blind Randomized Trial Comparing Implants with Laser-Microtextured and Machined Collar Surfaces: Microbiologic and Clinical Results	Guarnieri R.; Rappelli G.; Piemontese M.; Procaccini M.; Quaranta A.	2016
Immediate Loading of Implants in the Edentulous Maxilla with a Fixed Provisional Restoration without Bone Augmentation: A Report on 5-Year Outcomes Data Obtained from a Prospective Clinical Trial	Toljanic J.A.; Ekstrand K.; Baer R.A.; Thor A.	2016
Split-Mouth Comparison of Splinted and Nonsplinted Prostheses on Short Implants: 3-Year Results	Clelland N.; Chaudhry J.; Rashid R.G.; McGlumphy E.	2016
Soft tissue augmentation procedures at second-stage surgery: a systematic review	Bassetti R.G.; Stähli A.; Bassetti M.A.; Sculean A.	2016
High risk population for MRONJ onset: Oral condition of oncologic and hematologic patients undergoing individual preventive program	Vecchiatini R.; Petrucci V.; Mobilio N.; Guarnelli M.E.; Frassoldati A.; Cuneo A.; Trombelli L.	2016
Comparison of Marginal Bone Changes with Internal Conus and External Hexagon Design Implant Systems: A Prospective, Randomized Study	Cooper L.F.; Tarnow D.; Froum S.; Moriarty J.; De Kok I.J.	2016
Immediate placement of dental implants in infected dental extraction sockets	Patel N.; Young H.; Anwar S.; Kwok J.	2016
Effects of glycemic control in patients with type II diabetes mellitus on dental implant survival	Lee J.S.; Tolomeo P.G.; Miller E.J.	2016
Selective serotonin reuptake inhibitors and dental implant osseointegration	Tolomeo P.G.; Lee J.S.; Miller E.J.	2016
Computer-aided flapless implant surgery and immediate loading. A technical note	Baruffaldi A.; Poli P.P.; Baruffaldi A.; Giberti L.; Pigozzo M.; Maiorana C.	2016
Early loading of delayed versus immediately placed implants in the anterior mandible: A pilot comparative clinical study	Koirala D.P.; Singh S.V.; Chand P.; Siddharth R.; Jurel S.K.; Aggarwal H.; Tripathi S.; Ranabhatt R.; Mehrotra D.	2016
Marginal and apical bone stability after staged sinus floor augmentation using bone condensing implants with variable-thread design: a two-dimensional analysis	Younes F.; Eghbali A.; De Troyer S.; De Bruyckere T.; Cleymaet R.; Cosyn J.	2016

Netrin-1 is highly expressed and required in inflammatory infiltrates in wear particle-induced osteolysis	Mediero A.; Ramkhelawon B.; Wilder T.; Purdue P.E.; Goldring S.R.; Dewan M.Z.; Loomis C.; Moore K.J.; Cronstein B.N.	2016
Unstable proximal femur fractures treated with proximal femoral locking plates: A retrospective, multicenter study of 111 cases	Collinge C.A.; Hymes R.; Archdeacon M.; Streubel P.; Obremskey W.; Weber T.; Watson J.T.; Lowenberg D.	2016
Prolonged antimicrobial durability of a sodium mercaptoethane sulfonate + minocycline + rifampin combination in an in situ melting, bioabsorbable wrap for preventing tissue expander infections following breast reconstruction surgeries	Rosenblatt J.; Reitzel R.; Viola G.M.; Selber J.; Raad I.	2016
PECS block and parasternal block in a patient with recent ischemic stroke	Fusco P.; Paladini G.; Petrucci E.; Scimia P.; Vacca F.; Tonelli E.; Pozone T.; Marinangeli F.	2016
Balloon tibioplasty for reduction of depressed tibial plateau fractures: Preliminary radiographic and clinical results	Ollivier M.; Turati M.; Munier M.; Lunebourg A.; Argenson J.-N.; Parratte S.	2016
Longterm follow up in patients with mid-urethral slings with BMI greater than 40	ElShatanoufy S.; Griffin M.A.; Wang Y.; Richardson D.; Atiemo H.; Jamil M.; Yousif M.; Matthews A.; Luck A.	2016
Does bariatric surgery prior to total hip or knee arthroplasty reduce post-operative complications and improve clinical outcomes for obese patients?: Systematic review and meta-analysis	Smith T.O.; Aboelmagd T.; Hing C.B.; MacGregor A.	2016
Risk of peri-implantitis in patients with diabetes mellitus: A meta-analysis	Tseng K.-C.; Zheng X.-Y.; Qu X.-H.; Lu E.-Y.	2016
An innovative therapy for peri-implantitis based on radio frequency electric current: numerical simulation results and clinical evidence	Cosoli G.; Scalise L.; Tricarico G.; Tomasini E.P.; Cerri G.	2016
Discomfort/pain due to pocket probing at teeth and endosseous implants: a cross-sectional study	Ringeling J.; Parvini P.; Weinbach C.; Nentwig G.-H.; Nickles K.; Eickholz P.	2016
Complications with allogeneic, cancellous bone blocks in vertical alveolar ridge augmentation: prospective clinical case study and review of the literature	Draenert F.G.; Kämmerer P.W.; Berthold M.; Neff A.	2016
Periimplant bone regeneration in hydroxyapatite block grafts with mesenchymal stem cells and bone morphogenetic protein-2	Park J.-H.; Jung Y.-E.; Kim M.-J.; Hwang S.J.	2016
Early loading of splinted implants supporting a two-unit fixed partial denture in the posterior maxilla: 13-month results from a randomized controlled clinical trial of two different implant systems	Ryu H.-S.; Namgung C.; Heo Y.-K.; Lee J.-H.; Lim Y.-J.	2016
The SSTARS (STeroids and Stents Against Re-Stenosis) Trial: Different stent alloys and the use of peri-procedural oral corticosteroids to prevent in-segment restenosis after percutaneous coronary intervention	Adam Z.; Turley A.; Mason J.M.; Kasim A.S.; Newby D.; Mills N.; Padfield G.; Thompson L.; Morley R.; Hall J.A.; Wright R.A.; Muir D.F.; Sutton A.G.C.; Swanson N.; Carter J.; Bilous R.; Jones S.; De Belder M.A.	2016
Leadless Cardiac Devices—Pacemakers and Implantable Cardioverter-Defibrillators	Rutzen-Lopez H.; Silva J.; Helm R.H.	2016
Osteomyelitis-Induced Sequester of Bovine-Derived Bone Mineral/Host Bone Composite 8 Years Post-Sinus Graft: A Case Report	Norton M.R.	2016
Management considerations for patients with uterine fibroids and concurrent venous thromboembolism	Moulder J.K.; Siedhoff M.T.; Till S.R.; Moll S.	2016
Low-cost, hand-made, mid-urethral sling for the treatment of female stress urinary incontinence: A single-center's 10 year experience	Alvarez J.; Urzua M.J.; Rondini C.; Kaplan F.; Troncoso F.R.	2016
Somatosensory evoked potentials in carotid artery stenting: Effectiveness in ascertaining cerebral ischemic events	Adhikari R.B.; Takeda M.; Kolakshyapati M.; Sakamoto S.; Morishige M.; Kiura Y.; Okazaki T.; Shinagawa K.; Ichinose N.; Yamaguchi S.; Kurisu K.	2016
Trends in mesh utilization, surgical complications, and resident participation in transvaginal procedures for pelvic organ prolapse repair: An audit of the national surgical quality improvement program	Matthews C.A.; Badlani G.; Mirzazadeh M.	2016
Effectiveness of hypochlorous acid to reduce the biofilms on titanium alloy surfaces in vitro	Chen C.-J.; Chen C.-C.; Ding S.-J.	2016
Zirconia Dental Implants: Investigation of Clinical Parameters, Patient Satisfaction, and Microbial Contamination	Holländer J.; Lorenz J.; Stübinger S.; Hölscher W.; Heidemann D.; Ghanaati S.; Sader R.	2016
Bacterial Biofilm Morphology on a Failing Implant with an Oxidized Surface: A Scanning Electron Microscope Study	Simion M.; Kim D.M.; Pieroni S.; Nevins M.; Cassinelli C.	2016
Antimicrobial Agents Used in the Treatment of Peri-Implantitis Alter the Physicochemistry and Cytocompatibility of Titanium Surfaces	Kotsakis G.A.; Lan C.; Barbosa J.; Lill K.; Chen R.; Rudney J.; Aparicio C.	2016
Emerging Issues Associated With Peri-implant Disease	Rosen P.S.; Froum S.J.	2016
Initial Crestal Bone Loss After Implant Placement with Flapped or Flapless Surgery-A Prospective Cohort Study	Maier F.-M.	2016
Do we need keratinized mucosa for a healthy peri-implant soft tissue?	Esfahanizadeh N.; Daneshparvar N.; Motallebi S.; Akhondi N.; Askarpour F.; Davaie S.	2016
Influence of Connection Types and Implant Number on the Biomechanical Behavior of Mandibular Full-Arch Rehabilitation	Sousa R.M.; Simamoto-Junior P.C.; Fernandes-Neto A.J.; Sloten J.V.; Jaecques S.V.; Pessoa R.S.	2016
Implant-Related Gingival Recession: Pilot Case Series Presents Novel Technique and Scoring Template	El Askary Ae.lS.; Ghallab N.A.; Tan S.-C.; Rosen P.S.; Shawkat A.	2016
Bilamina cortical tenting grafting technique for three-dimensional reconstruction of severely atrophic alveolar ridges in anterior maxillae: A 6-year prospective study	Yu H.; Chen L.; Zhu Y.; Qiu L.	2016
Partial Rehabilitation with Distally Tilted and Straight Implants in the Posterior Maxilla with Immediate Loading Protocol: A Retrospective Cohort Study with 5-Year Follow-up	Queridinha B.M.; Almeida R.F.; Felino A.; de Araújo Nobre M.; Maló P.	2016
Peri-implant bony overgrowth as a cause of revision surgery in auditory osseointegrated implantation	Tompkins J.J.; Petersen D.K.; Sharbel D.D.; McKinnon B.J.; MacDonald C.B.	2016
Complications and functional outcome after fixation of distal tibia fractures with locking plate - A multicentre study	Viberg B.; Kleven S.; Hamborg-Petersen E.; Skov O.	2016

Chronic Total Occlusion – Percutaneous Coronary Intervention (CTO-PCI) Experience in a Single, Multi-operator Australian Centre: Need for dedicated CTO-PCI programs	BoganaShanmugam V.; Psaltis P.J.; Wong D.T.; Seneviratne S.; Cameron J.; Meredith I.T.; Malaiapan Y.	2016
Health, Maintenance, and Recovery of Soft Tissues around Implants	Wang Y.; Zhang Y.; Miron R.J.	2016
10-year prospective cohort follow-up of immediately restored XiVE implants	Degidi M.; Nardi D.; Piattelli A.	2016
Clinical association of Spirochaetes and Synergistetes with peri-implantitis	Belibasakis G.N.; Mir-Mari J.; Sahrmann P.; Sanz-Martin I.; Schmidlin P.R.; Jung R.E.	2016
Retrospective analysis of the prevalence of peri-implant diseases in non-smoking patients rehabilitated with a fixed full-arch restoration, supported by two mesial axial and two distal tilted implants	Cavalli N.; Austoni C.; Corbella S.; Taschieri S.; Barbaro B.; Azzola F.; Francetti L.	2016
The influence of peri-implant keratinized mucosa on brushing discomfort and peri-implant tissue health	Souza A.B.; Tormena M.; Matarazzo F.; Araújo M.G.	2016
Success of 6-mm Implants with Single-Tooth Restorations: A 3-year Randomized Controlled Clinical Trial	Sahrmann P.; Naenni N.; Jung R.E.; Held U.; Truninger T.; Hämmerle C.H.; Attin T.; Schmidlin P.R.	2016
Post-treatment supportive care for the natural dentition and dental implants	Armitage G.C.; Xenoudi P.	2016
Long-term Survival of Straumann Dental Implants with TPS Surfaces: A Retrospective Study with a Follow-up of 12 to 23 Years	Becker S.T.; Beck-Broichsitter B.E.; Rossmann C.M.; Behrens E.; Jochens A.; Wiltfang J.	2016
Residual Roots as an Anatomical Guide for Implant Placement: Case Series With Two-Year Follow-up	Mahesh L.; Kurtzman G.M.; Schwartz D.; Shukla S.	2016
Successful Surgical Protocols in the Treatment of Peri-Implantitis: A Narrative Review of the Literature	Froum S.J.; Dagba A.S.; Shi Y.; Perez-Asenjo A.; Rosen P.S.; Wang W.C.	2016
The Effect of Insertion Torque on the Clinical Outcome of Single Implants: A Randomized Clinical Trial	Barone A.; Alfonsi F.; Derchi G.; Tonelli P.; Toti P.; Marchionni S.; Covani U.	2016
Compromised Hard- and Soft-Tissue Architecture: Immediate Implant Placement and Provisionalization in the Esthetic Zone	Gober D.D.; Weitz M.L.; Korn R.J.	2016
Radiographic evaluation of conical tapered platform-switched implants in the posterior mandible: 1-year results of a two-center prospective study	Moergel M.; Rocha S.; Messias A.; Nicolau P.; Guerra F.; Wagner W.	2016
Analysis of Effectiveness of Different Rotational Instruments in Implantoplasty: An In Vitro Study	de Souza Júnior J.M.; Oliveira de Souza J.G.; Pereira Neto A.L.; Iaculli F.; Piattelli A.; Bianchini M.A.	2016
Osteotome Sinus Floor Elevation without Grafting: A 10-Year Prospective Study	Nedir R.; Nurdin N.; Vazquez L.; Abi Najm S.; Bischof M.	2016
Effects of angulated and non-angulated mini-implants abutment supporting mandibular overdenture on peri-implant bone height	Arafa K.A.O.	2016
Maxillary Four Implant-retained Overdentures via Locator® Attachment: Intermediate-term Results from a Retrospective Study	Wang F.; Monje A.; Huang W.; Zhang Z.; Wang G.; Wu Y.	2016
“Partner prosthesis Panic”: Management of female sexual dysfunction in partners of men undergoing penile prosthesis implantation	Hartzell R.; King S.A.; Goldstein I.	2016
The modified stepwise ablation guided by low-dose ibutilide in chronic atrial fibrillation trial (The MAGIC-AF Study)	Singh S.M.; D'Avila A.; Kim Y.-H.; Aryana A.; Mangrum J.M.; Michaud G.F.; Dukkipati S.R.; Barrett C.D.; Heist E.K.; Parides M.K.; Thorpe K.E.; Reddy V.Y.	2016
Pancreatic duct guidewire placement for biliary cannulation for the prevention of post-endoscopic retrograde cholangiopancreatography (ERCP) pancreatitis	Tse F.; Yuan Y.; Bukhari M.; Leontiadis G.I.; Moayyedi P.; Barkun A.	2016
Dental floss as a possible risk for the development of peri-implant disease: an observational study of 10 cases	van Velzen F.J.; Lang N.P.; Schulten E.A.; Ten Bruggenkate C.M.	2016
Collagen-Coated Bovine Bone in Peri-implantitis Defects: A Pilot Study on a Novel Approach	Rotenberg S.A.; Steiner R.; Tatakis D.N.	2016
Long-term outcomes of oral rehabilitation with dental implants in HIV-positive patients: A retrospective case series	Gay-Escoda C.; Pérez-Álvarez D.; Camps-Font O.; Figueiredo R.	2016
Removal of dental implants: review of five different techniques	Stajčić Z.; Stojčev Stajčić L.J.; Kalanović M.; Đinić A.; Divekar N.; Rodić M.	2016
Bactericidal Effect of Erbium-Doped Yttrium Aluminum Garnet Laser and Photodynamic Therapy on Aggregatibacter Actinomycetemcomitans Biofilm on Implant Surface	Saffarpour A.; Fekrazad R.; Heibati M.N.; Bahador A.; Saffarpour A.; Rokn A.R.; Iranparvar A.; KharaziFard M.J.	2016
Management of Limited Vertical Bone Height in the Posterior Mandible: Short Dental Implants Versus Nerve Lateralization With Standard Length Implants	Dursun E.; Keceli H.G.; Uysal S.; Güngör H.; Muhtarogullari M.; Tözüm T.F.	2016
Peri-implant Diseases: What do We know and What do We need to know?	Danesh-Sani S.A.	2016
Transfer of Bacteria into the Internal Cavity of Dental Implants After Application of Disinfectant or Sealant Agents In Vitro	Podhorsky A.; Biscoping S.; Rehmann P.; Streckbein P.; Domann E.; Wöstmann B.	2016
Insufficient evidence to support the use of laser therapy for peri-implantitis	Hur Y.; Ogata Y.	2016
Research progress in peri-implant soft tissue engineering augmentation method	Pei T.; Yu H.; Wen C.; Guo T.; Zhou Y.; Peng H.	2016
Histopathological Features of Secondary Squamous Cell Carcinoma Around a Dental Implant in the Mandible after Chemoradiotherapy: A Case Report with a Clinicopathological Review	Nariai Y.; Kanno T.; Sekine J.	2016
Turned Implants in Vertical Augmented Bone: A Retrospective Study with 13 to 21 Years Follow-Up	Simion M.; Ferrantino L.; Idotta E.; Zarone F.	2016
The Pathogenesis of Implant-Related Reactive Lesions: A Clinical, Histologic and Polarized Light Microscopy Study	Halperin-Sternfeld M.; Sabo E.; Akrish S.	2016
Psychological Interventions for Poor Oral Health: A Systematic Review	Werner H.; Hakeberg M.; Dahlström L.; Eriksson M.; Sjögren P.; Strandell A.; Svanberg T.; Svensson L.; Wide Boman U.	2016

Evaluation of Temperature and Roughness Alteration of Diode Laser Irradiation of Zirconia and Titanium for Peri-Implantitis Treatment	Kushima S.S.; Nagasawa M.; Shibli J.A.; Brugnera A.; Rodrigues J.A.; Cassoni A.	2016
Botulinum Toxin Type A as Preoperative Treatment for Immediately Loaded Dental Implants Placed in Fresh Extraction Sockets for Full-Arch Restoration of Patients With Bruxism	Mijiritsky E.; Mortellaro C.; Rudberg O.; Fahn M.; Basegmez C.; Levin L.	2016
Titanium Mesh Technique in Rehabilitation of Totally Edentulous Atrophic Maxillae: A Retrospective Case Series	Pellegrino G.; Lizio G.; Corinaldesi G.; Marchetti C.	2016
A Long-Term Prospective Evaluation of Marginal Bone Level Change Around Different Implant Systems	Lee S.-Y.; Koak J.-Y.; Kim S.-K.; Rhyu I.-C.; Ku Y.; Heo S.-J.; Han C.-H.	2016
The effect of piezoelectric surgery implant osteotomy on radiological and molecular parameters of peri-implant crestal bone loss: a randomized, controlled, split-mouth trial	Peker Tekdal G.; Bostanci N.; Belibasakis G.N.; Gürkan A.	2016
Medication-Related Osteonecrosis of the Jaw Around Dental Implants: Implant Surgery-Triggered or Implant Presence-Triggered Osteonecrosis?	Giovannacci I.; Meleti M.; Manfredi M.; Mortellaro C.; Greco Lucchina A.; Bonanini M.; Vescovi P.	2016
Predictability of short implants (< 10 mm) as a treatment option for the rehabilitation of atrophic maxillae. A systematic review	Sierra-Sánchez J.-L.; García-Sala-Bonmatí F.; Martínez-González A.; García-Dalmau C.; Mañes-Ferrer J.-F.; Brotons-Oliver A.	2016
Ridge augmentation in an organ transplant patient	Dalla Torre D.; Burtscher D.	2016
Effectivity of air-abrasive powder based on glycine and tricalcium phosphate in removal of initial biofilm on titanium and zirconium oxide surfaces in an ex vivo model	John G.; Becker J.; Schwarz F.	2016
Contraceptive hormone therapy induced spontaneous coronary artery dissection	Shekar C.; Perucki W.; Wankhade C.; Krishna N.	2016
The Effect of Crown-to-Implant Ratio on the Clinical Performance of Extra-Short Locking-Taper Implants	Mangano F.; Frezzato I.; Frezzato A.; Veronesi G.; Mortellaro C.; Mangano C.	2016
Parameters Associated with Marginal Bone Loss around Implant after Prosthetic Loading	Koller C.D.; Pereira-Cenci T.; Boscato N.	2016
Catheter ablation for management of ventricular tachycardia in patients on left ventricular assist device support	Shabari F.R.; Raman A.S.; Davagustto G.; Sharma S.; Fteddy S.; Mukerji S.S.; Hematpour K.; Venkataraman R.; Gregoric I.D.; Nathan S.S.; Ftajapreyar I.N.; Loyalka P.; Kar B.; Hariharan R.	2016
EUS-guided drainage of peri-pancreatic fluid collections without fluoroscopic guidance	Mohamadnejad M.; Sohrabpour A.A.; Yazdi N.; Zamani F.; Malekzadeh R.	2016
Esophago-jejunal anastomosis defect: Beyond a simple leak!	Fernandez M.; Lemmers A.; Vandermeeren A.; Toussaint E.; Eisendrath P.; Blero D.; Loly J.-P.; Le Moine O.; Deviere J.; Arvanitakis M.	2016
Acute stroke endovascular intervention with emergent carotid artery stenting 22.5 hours after symptom onset - Emphasizing the importance of brain perfusion CT for case selection	Hsieh M.-Y.; Wang W.-J.; Tee B.L.	2016
Multi-center post-market registry for the evaluation of robotic assisted PCI	Madder R.; Campbell P.; Mahmud E.; Wohns D.; Stys T.; Caputo R.; Leimbach M.; Parikh M.; Kasi V.; Weisz G.	2016
Effect of interleukin 6 –174G>C gene polymorphism on opioid requirements after total hip replacement	Białecka M.; Jurewicz A.; Machoy-Mokrzyńska A.; Kurzawski M.; Leźnicka K.; Dziedziejko V.; Safranow K.; Droździk M.; Bohatyrewicz A.	2016
Single centre experience of endovascular intervention in extra and intracranial vertebro-basilar stenosis at a tertiary care centre	Kaur G.; Sharma S.; Latorre J.	2016
Assessment and evaluation of quality of life (OHRQOL) of patients with dental implants using the oral health impact profile (OHIP-14) - A clinical study	Alzarea B.K.	2016
Impact of Maintenance Therapy for the Prevention of Peri-implant Diseases: A Systematic Review and Meta-analysis	Monje A.; Aranda L.; Diaz K.T.; Alarcón M.A.; Bagramian R.A.; Wang H.L.; Catena A.	2016
Systemic antibiotics and the risk of superinfection in peri-implantitis	Verdugo F.; Laksmana T.; Uribarri A.	2016
Peri-implantitis - onset and pattern of progression	Derks J.; Schaller D.; Håkansson J.; Wennström J.L.; Tomasi C.; Berglundh T.	2016
Recommendations on the clinical application of air polishing for the management of peri-implant mucositis and peri-implantitis	Schwarz F.; Becker K.; Bastendorf K.-D.; Cardaropoli D.; Chatfield C.; Dunn I.; Fletcher P.; Einwag J.; Louropoulou A.; Mombelli A.; Ower P.; Pavlovic P.; Sahrmann P.; Salvi G.E.; Schmage P.; Takeuchi Y.; Van Der Weijden F.; Renvert S.	2016
Reosseointegration Following Regenerative Therapy of Tissue-Engineered Bone in a Canine Model of Experimental Peri-Implantitis	Xu L.; Sun X.; Bai J.; Jiang L.; Wang S.; Zhao J.; Xia L.; Zhang X.; Wen J.; Li G.; Jiang X.	2016
Long-term retrospective follow-up of turned and moderately rough implants in the edentulous jaw	Vandeweghe S.; Ferreira D.; Vermeersch L.; Mariën M.; De Bruyn H.	2016
A combined peri-implant plastic surgery approach for implants in the esthetic zone using a novel technique: A series of 10 cases	Baltacıoğlu E.; Korkmaz Y.T.; Korkmaz F.M.; Bağış N.	2016
Clinical Decisions: Determining When to Save or Remove an Ailing Implant	Tarnow D.P.; Chu S.J.; Fletcher P.D.	2016
Keratinized mucosa around implants in partially edentulous posterior mandible: 10-year results of a prospective comparative study	Roccuzzo M.; Grasso G.; Dalmasso P.	2016
Retrospective study on bone-level and soft-Tissue-level cylindrical implants	Lopez M.A.; Bassi M.A.; Confalone L.; Gaudio R.M.; Lombardo L.; Lauritano D.	2016
Alumina reinforced zirconia implants: 1-year results from a prospective cohort investigation	Spies B.C.; Sperlich M.; Fleiner J.; Stampf S.; Kohal R.-J.	2016
Modified connective tissue punch technique to increase the vestibular/buccal keratinized tissue on flapless implant surgery: A case series	Bassi M.A.; Andrisani C.; Lopez M.A.; Gaudio R.M.; Lombardo L.; Lauritano D.	2016

Bone Response and Soft Tissue Changes Around Implants With/Without Abutments Supporting Fixed Partial Dentures: Results From a 3-Year, Prospective, Randomized, Controlled Study	Göthberg C.; André U.; Gröndahl K.; Thomsen P.; Slotte C.	2016
Histological and histomorphometric analyses of narrow implants, crestal and subcrestally placed in severe alveolar atrophy: a study in foxhound dogs	Calvo-Guirado J.L.; Delgado Ruiz R.A.; Ramírez-Fernández M.P.; Abboud M.; Janjic B.; Maté Sánchez de Val J.E.	2016
Locators versus magnetic attachment effect on peri-implant tissue health of immediate loaded two implants retaining a mandibular overdenture: a 1-year randomised trial	Elsyad M.A.; Mahanna F.F.; Elshahat M.A.; Elshoukouki A.H.	2016
Recommendations for implant-retained nasal prostheses after ablative tumor surgery: Minimal surgical aftercare, high implant survival, and satisfied patients	Korfage A.; Raghoebar G.M.; Noorda W.D.; Plaat B.E.; Vissink A.; Visser A.	2016
Implant Abutment Cleaning by Plasma of Argon: 5-Year Follow-Up of a Randomized Controlled Trial	Canullo L.; Tallarico M.; Peñarrocha-Oltra D.; Monje A.; Wang H.-L.; Peñarrocha-Diago M.	2016
Periimplantitis: A problem possible to resolve	Kiernicka M.; Wysokińska-Miszczuk J.	2016
Surgical Treatment of Implants Affected by Periimplantitis After 15 Years of Loading: A Case Report	Nícoli L.G.; Pigossi S.C.; Marcantonio C.; Leal Zandim-Barcelos D.; Marcantonio E.	2016
Long-term safety of SPG stimulation for the treatment of cluster headache (10338)	Goodman A.; Hillerup S.; Assaf A.T.; Rostgaard J.J.R.; Puche M.; Barloese M.; Jurgens T.; Caparso A.V.; May A.; Jensen R.H.	2016
Three-Year Clinical and Radiographic Implant Follow-up in Sinus-Lifted Maxilla With Lateral Window Technique	Erdem N.F.; Çiftçi A.; Acar A.H.	2016
Atrial fibrillation during or after TAVI: Incidence, implications and therapeutical considerations	Vavuranakis M.; Kolokathis A.-M.; Vrachatis D.A.; Kalogeras K.; Magkoutis N.A.; Fradi S.; Ghostine S.; Karamanou M.; Tousoulis D.	2016
Biological mesh implants for abdominal hernia repair: US food and drug administration approval process and systematic review of its efficacy	Huerta S.; Varshney A.; Patel P.M.; Mayo H.G.; Livingston E.H.	2016
Cochlear implantation in early deafened, late implanted adults: Do they benefit?	Kumar R.S.; Mawman D.; Sankaran D.; Melling C.; O'Driscoll M.; Freeman S.M.; Lloyd S.K.	2016
Invasive hemodynamic testing in ambulatory left ventricular assist device patients	Badoe N.; Abdullah K.; Phillips S.; Nabut J.; Rongione A.J.; Desai S.S.; Shah P.	2016
Infection rates of spinal cord stimulator implants among high risk populations: A multi-center study (10301)	Hoelzer B.; Bendel M.A.; Deer T.R.; Qu W.; Moeschler S.; Falowski S.; Wassef IV C.; Kim C.; Bowman R.; Choi D.; Choi D.Y.; Choe D.; Kim E.; Lerman I.; Mora R.; Wang Z.; Hayek S.M.; Hanes M.; Ade T.; Neuman S.A.	2016
In vitro activity of taurolidine gel on bacteria associated with periodontitis	Eick S.; Gloor N.; Püls C.; Zumbrunn J.; Sculean A.	2016
Slipped capital femoral epiphysis and associated hypothyroidism. A review of the literature with two classic case examples	Moyer J.; Jacks L.; Hunter J.D.; Chan G.	2016
Outcomes of total hip arthroplasty, as a salvage procedure, following failed internal fixation of intracapsular fractures of the femoral neck: A systematic review and meta-analysis	Mahmoud S.S.S.; Pearse E.O.; Hing C.B.; Smith T.O.	2016
Neuromuscular exercise prior to joint arthroplasty in patients with osteoarthritis of the hip or knee	Villadsen A.	2016
Role of preoperative biliary stents, bile contamination and antibiotic prophylaxis in surgical site infections after pancreaticoduodenectomy	Gavazzi F.; Ridolfi C.; Capretti G.; Angiolini M.R.; Morelli P.; Casari E.; Montorsi M.; Zerbi A.	2016
1-year outcomes with the Absorb bioresorbable scaffold in patients with coronary artery disease: A patient-level, pooled meta-analysis	Stone G.W.; Gao R.; Kimura T.; Kereiakes D.J.; Ellis S.G.; Onuma Y.; Cheong W.-F.; Jones-Mcmeans J.; Su X.; Zhang Z.; Serruys P.W.	2016
Modification of Titanium Substrates with Chimeric Peptides Comprising Antimicrobial and Titanium-Binding Motifs Connected by Linkers To Inhibit Biofilm Formation	Liu Z.; Ma S.; Duan S.; Xuliang D.; Sun Y.; Zhang X.; Xu X.; Guan B.; Wang C.; Hu M.; Qi X.; Zhang X.; Gao P.	2016
Surgical therapy of single peri-implantitis intrabony defects, by means of deproteinized bovine bone mineral with 10% collagen	Roccuzzo M.; Gaudioso L.; Lungo M.; Dalmasso P.	2016
Into the Paradigm of Local Factors as Contributors for Peri-implant Disease: Short Communication	Monje A.; Galindo-Moreno P.; Tözüm T.F.; Suárez-López del Amo F.; Wang H.-L.	2016
Clinical and microbiological findings in patients with peri-implantitis: a cross-sectional study	Canullo L.; Peñarrocha-Oltra D.; Covani U.; Botticelli D.; Serino G.; Penarrocha M.	2016
The association between shallow vestibular depth and peri-implant parameters: a retrospective 6 years longitudinal study	Halperin-Sternfeld M.; Zigdon-Giladi H.; Machtei E.E.	2016
The implant surface characteristics and peri-implantitis. An evidence-based update	Peixoto C.D.; Almas K.	2016
Survival, Function, and Complications of Oral Implants Placed in Bone Flaps in Jaw Rehabilitation: A Systematic Review	Zhang L.; Ding Q.; Liu C.; Sun Y.; Xie Q.; Zhou Y.	2016
Peri-implant Outcomes with Laser vs Nanosurface Treatment of Early Loaded Implant-Retaining Mandibular Overdentures	Hegazy S.; Elmekawy N.; Emera R.M.	2016
Posterior jaws rehabilitated with partial prostheses supported by 4.0 x 4.0 mm or by longer implants: One-year post-loading results from a multicenter randomised controlled trial	Felice P.; Checchi L.; Barausse C.; Pistilli R.; Sammartino G.; Masi I.; Ippolito D.R.; Esposito M.	2016
Immediate occluding definitive partial fixed prosthesis versus non-occluding provisional restorations - 4-month post-loading results from a pragmatic multicenter randomised controlled trial	Heinemann F.; Grufferty B.; Papavasiliou G.; Dominiak M.; García J.J.; Trullenque-Eriksson A.; Esposito M.	2016
Effects of Topical Antibiotic Prophylaxis on Wound Healing After Flapless Implant Surgery: A Pilot Study	Xu L.; Wang Y.; Nguyen V.T.; Chen J.	2016
Bone augmentation at implant dehiscences and fenestrations. A systematic review of randomised controlled trials	Merli M.; Merli I.; Raffaelli E.; Pagliaro U.; Nastri L.; Nieri M.	2016
Regenerative Medicine for Periodontal and Peri-implant Diseases	Larsson L.; Decker A.M.; Nibali L.; Pilipchuk S.P.; Berglundh T.; Giannobile W.V.	2016

Relationship Between Long-Term Marginal Bone Loss and Bone Quality, Implant Width, and Surface	Ibañez C.; Catena A.; Galindo-Moreno P.; Noguerol B.; Magán-Fernández A.; Mesa F.	2016
Past, present and future of local and systemic adjuvant therapies in non-surgical treatment of peri-implantitis and periodontitis: Indications and limitations	Mensi M.; Scotti E.; Francetti L.; Giargia M.; Latronico M.; Camurati A.; Guarnelli M.E.; Rotundo R.	2016
Selective responses of human gingival fibroblasts and bacteria on carbon fiber reinforced polyetheretherketone with multilevel nanostructured TiO2	Wang X.; Lu T.; Wen J.; Xu L.; Zeng D.; Wu Q.; Cao L.; Lin S.; Liu X.; Jiang X.	2016
Errors in planning implanto-prosthodontic rehabilitation - Case report	Barić J.; Sušić M.; Gabrić D.	2016
Clinical and Retrospective Evaluation of 4.1- or 4.3-mm-Diameter Implants Placed Immediately in the Molar Region: A Preliminary Study	Jung D.-W.; Yoon H.-J.	2016
Antimicrobial photodynamic therapy (aPDT) induction of biofilm matrix architectural and bioadhesive modifications	Mang T.; Rogers S.; Keinan D.; Honma K.; Baier R.	2016
A minimally invasive technique for lateral maxillary sinus floor elevation: a Bayesian network study	Merli M.; Moscatelli M.; Mariotti G.; Pagliaro U.; Bernardelli F.; Nieri M.	2016
A retrospective study of esophagectomy outcomes: An institutional review	Horst V.; Patel H.; Touliatos J.G.	2016
Multi-center analysis of operative safety of surgery in patients with amyotrophic lateral sclerosis: The diaphragm pacing experience	Onders R.; Carlin A.; Dunkin B.; Jossart G.; Marohn M.; Menegaux F.; Morton J.; Smith C.D.	2016
Use of endovascular stents for management of pediatric May-Thurner syndrome: A single-center experience	Goldman R.; Arendt V.; Kothary N.; Kuo W.; Sze D.; Hofmann L.; Lungren M.	2016
Safety, efficacy, and short-term follow-up of the use of Pipeline™ Embolization Device in small (<2.5mm) cerebral vessels for aneurysm treatment: single institution experience	Puri A.S.; Massari F.; Asai T.; Marosfoi M.; Kan P.; Hou S.Y.; Howk M.; Perras M.; Brooks C.; Clarencon F.; Gounis M.J.; Wakhloo A.K.	2016
Endovascular treatment in cerebral artery tandem lesions	Han J.; Li X.; He Q.; Zhao H.; Ye S.; Dong G.; Luan J.; Wang C.	2016
Transcatheter mitral valve implantation for inoperable severely calcified native mitral valve disease: A systematic review	Puri R.; Abdul-Jawad Altisent O.; Del Trigo M.; Campelo-Parada F.; Regueiro A.; Barbosa Ribeiro H.; Delarochellière R.; Paradis J.-M.; Dumont E.; Rodés-Cabau J.	2016
Peri-implant evaluation of immediately loaded implants placed in esthetic zone in patients with diabetes mellitus type 2: a two-year study	Aguilar-Salvatierra A.; Calvo-Guirado J.L.; González-Jaranay M.; Moreu G.; Delgado-Ruiz R.A.; Gómez-Moreno G.	2016
Histopathological Verification of Osteoimmunological Mediators in Peri-Implantitis and Correlation to Bone Loss and Implant Functional Period	Konermann A.; Götz W.; Le M.; Dirk C.; Lossdörfer S.; Heinemann F.	2016
Crestal bone resorption in augmented bone using mineralized freeze-dried bone allograft or pristine bone during submerged implant healing: a prospective study in humans	Huang H.; Ogata Y.; Hanley J.; Finkelman M.; Hur Y.	2016
A New Approach for Treating Peri-Implantitis: Reversibility of Osseointegration	Anitua E.	2016
Effect of Induced Periimplantitis on Dental Implants With and Without Ultrathin Hydroxyapatite Coating	Madi M.; Zakaria O.; Ichinose S.; Kasugai S.	2016
Ultrasonic Assessment of Mucosal Thickness around Implants: Validity, Reproducibility, and Stability of Connective Tissue Grafts at the Buccal Aspect	Eghbali A.; De Bruyn H.; Cosyn J.; Kerckaert I.; Van Hoof T.	2016
A 9-Year Prospective Case Series Using Multivariate Analyses to Identify Predictors of Early and Late Peri-Implant Bone Loss	Vervaeke S.; Collaert B.; Cosyn J.; De Bruyn H.	2016
Short Implants Placed with or without Grafting in Atrophic Sinuses: The 3-Year Results of a Prospective Randomized Controlled Study	Nedir R.; Nurdin N.; Khoury P.; Bischof M.	2016
Biofilm removal from implants supported restoration using different instruments: a 6-month comparative multicenter clinical study	Blasi A.; Iorio-Siciliano V.; Pacenza C.; Pomingi F.; Matarasso S.; Rasperini G.	2016
Use of Lasers to Treat Failing Dental Implants	Cranska J.P.	2016
Effect of Ultraviolet Irradiation of the Implant Surface on Progression of Periimplantitis--A Pilot Study in Dogs	Ishii K.; Matsuo M.; Hoshi N.; Takahashi S.-S.; Kawamata R.; Kimoto K.	2016
Influence of placement depth and abutment connection pattern on bone remodeling around 1-stage implants: a prospective randomized controlled clinical trial	Palaska I.; Tsaousoglou P.; Vouros I.; Konstantinidis A.; Menexes G.	2016
Dynamic locking plate vs. simple cannulated screws for nondisplaced intracapsular hip fracture: A comparative study	Warschawski Y.; Sharfman Z.T.; Berger O.; Steinberg E.L.; Amar E.; Snir N.	2016
Does exchange arthroplasty of an infected shoulder prosthesis provide better eradication rate and better functional outcome, compared to a permanent spacer or resection arthroplasty? a systematic review Orthopedics and biomechanics	George D.A.; Volpin A.; Scarponi S.; Haddad F.S.; Romanò C.L.	2016
Endovascular treatment of traumatic internal carotid artery pseudoaneurysm	Spanos K.; Karathanos C.; Stamoulis K.; Giannoukas A.D.	2016
Multicenter prospective trial of intracranial stent placement in patients with symptomatic high-grade intracranial stenosis	Gao P.; Jiao L.; Zhao Z.; Cai Y.; Li T.; Shi H.; Wu W.; He W.; Yin L.; Huang S.; Zhu F.; Wang D.; Qureshi A.I.; Ji X.; Ling F.	2016
Peri-procedural heparin use in stent-retriever thrombectomy	Haussen D.C.; Nogueira R.G.; Liebeskind D.S.; Smith W.; Lutsep H.; Jovin T.; Xiang B.	2016
Prognosis of implant longevity in terms of annual bone loss: a methodological finite element study	Demenko V.; Linetskiy I.; Linetska L.; Nesvit V.; Shevchenko A.; Yefremov O.; Weisskircher H.-W.	2016
Differentiating success from survival in modern implantology--key considerations for case selection, predicting complications and obtaining consent	Beaumont J.; McManus G.; Darcey J.	2016
Prevalence of Peri-implantitis in Medically Compromised Patients and Smokers: A Systematic Review	Turri A.; Rossetti P.H.; Canullo L.; Grusovin M.G.; Dahlin C.	2016

Association between tumor necrosis factor-alpha G-308A polymorphism and dental peri-implant disease risk A meta-analysis	Mo Y.-Y.; Zeng X.-T.; Weng H.; Cen Y.; Zhao Q.; Wen X.	2016
Reconstruction of Peri-implant Osseous Defects: A Multicenter Randomized Trial	Jepsen K.; Jepsen S.; Laine M.L.; Anssari Moin D.; Pilloni A.; Zeza B.; Sanz M.; Ortiz-Vigon A.; Roos-Jansåker A.M.; Renvert S.	2016
Effectiveness of Implant Therapy Analyzed in a Swedish Population: Prevalence of Peri-implantitis	Derks J.; Schaller D.; Håkansson J.; Wennström J.L.; Tomasi C.; Berglundh T.	2016
Prevalence of peri-implant disease on platform switching implants: a cross-sectional pilot study	Duque A.D.; Aristizabal A.G.; Londoño S.; Castro L.; Alvarez L.G.	2016
Natural resistance associated macrophage protein 1 gene polymorphism is associated with chronic periodontitis not peri-implantitis in an Iranian population: A cross sectional study	Kadkhodazadeh M.; Ebadian A.R.; Amid R.; Zarnegarnia P.; Mollaverdi F.; Aghamohammadi N.	2016
Factors influencing severity of peri-implantitis	Saaby M.; Karring E.; Schou S.; Isidor F.	2016
Accuracy of periapical radiography in assessing bone level in implants affected by peri-implantitis: a cross-sectional study	García-García M.; Mir-Mari J.; Benic G.I.; Figueiredo R.; Valmaseda-Castellón E.	2016
Peri-implantitis and diabetes mellitus	Liu J.; Zhu G.-H.; Zhang E.; Sun Y.-C.	2016
The influence of "Conical plus octagonal" internal connection on implant survival and success rate: A retrospective study of 66 fixtures	Lopez M.A.; Andreasi Bassi M.; Confalone L.; Gaudio R.M.; Lombardo L.; Lauritano D.	2016
Dental implant management and maintenance: How to improve long-term implant success?	Clark D.; Levin L.	2016
Are Dental Implants a Panacea or Should We Better Strive to Save Teeth?	Giannobile W.V.; Lang N.P.	2016
Peri-implant Hard and Soft Tissue Stability in Implants Placed Simultaneously Versus Delayed with Intraoral Block Bone Grafts in Horizontal Defects: A Retrospective Case Series Study	Aloy-Prósper A.; Peñarrocha-Oltra D.; Peñarrocha-Diago M.; Camacho-Alonso F.; Peñarrocha-Diago M.	2016
Implant-prosthetic rehabilitation after radiation treatment in head and neck cancer patients: A case-series report of outcome	Cotic J.; Jamsek J.; Kuhar M.; Hren N.I.; Kansky A.; Özcan M.; Jevnikar P.	2016
Six-Year Radiographic, Clinical, and Soft Tissue Outcomes of Immediately Loaded, Straight-Walled, Platform-Switched, Titanium-Alloy Implants with Nanosurface Topography	Glibert M.; De Bruyn H.; Östman P.-O.	2016
The use of lasers in the treatment of inflammatory tissue periodontal disease	Poorian B.; Dehghani N.; Chavoshinejad M.	2016
Evaluation of the clinical and aesthetic outcomes of Straumann(®) Standard Plus implants supported single crowns placed in non-augmented healed sites in the anterior maxilla: a 5-8 years retrospective study	Zhao X.; Qiao S.-C.; Shi J.-Y.; Uemura N.; Arai K.; Lai H.-C.	2016
Immediate versus conventional loaded single implants in the posterior mandible: A meta-analysis of randomized controlled trials	Moraschini V.; Porto Barboza E.	2016
A 10-year report from a multicentre randomised controlled trial: Immediate non-occlusal versus early loading of dental implants in partially edentulous patients	Zuffetti F.; Esposito M.; Galli F.; Capelli M.; Grandi G.; Testori T.	2016
Dental implants with internal versus external connections: 5-year post-loading results from a pragmatic multicenter randomised controlled trial	Esposito M.; Maghaireh H.; Pistilli R.; Grusovin M.G.; Lee S.T.; Trullenque-Eriksson A.; Gualini F.	2016
Factors affecting peri-implant bone resorption in four Implant supported mandibular full-arch restorations: a 3-year prospective study	Krennmair S.; Weinländer M.; Forstner T.; Krennmair G.; Stimmelmayr M.	2016
Clinical retrospective study of self-reported penicillin allergy on dental implant failures and infections	French D.; Noroozi M.; Shariati B.; Larjava H.	2016
Effects of different dental implant systems on the peri-implant bone absorption	Zhu Y.; Wan P.-B.; Zhao W.; Wang X.-L.; Liu J.; Wei K.-K.; Liu J.-X.	2016
Regulation of osteoclasts by osteoblast lineage cells is titanium surface dependent	Lotz E.; Berger M.; Hyzy S.; Schwartz Z.; Boyan B.D.	2016
Prevention of bacterial leakage at implant-abutment connection level: An in vitro study of the efficacy of three different implant systems	Carinci F.; Lauritano D.; Cura F.; Lopez M.A.; Andreasi Bassi M.; Confalone L.; Pezzetti F.	2016
Evaluation of Implant Collar Surfaces for Marginal Bone Loss: A Systematic Review and Meta-Analysis	Koodaryan R.; Hafezeqoran A.	2016
Immediate loading of two (fixed-on-2) versus three (fixed-on-3) implants placed flapless supporting cross-arch fixed prostheses: One-year results from a randomised controlled trial	Cannizzaro G.; Loi I.; Viola P.; Ferri V.; Leone M.; Trullenque-Eriksson A.; Esposito M.	2016
Alveolar Ridge Reconstruction with Titanium Meshes and Simultaneous Implant Placement: A Retrospective, Multicenter Clinical Study	Zita Gomes R.; Paraud Freixas A.; Han C.-H.; Bechara S.; Tawil I.	2016
The effects of healing abutments of different size and anatomic shape placed immediately in extraction sockets on peri-implant hard and soft tissues. A pilot study in foxhound dogs	López-López P.J.; Mareque-Bueno J.; Boquete-Castro A.; Aguilar-Salvatierra Raya A.; Martínez-González J.M.; Calvo-Guirado J.L.	2016
Immediate, immediate-delayed (6 weeks) and delayed (4 months) post-extractive single implants: 4-month post-loading data from a randomised controlled trial	Felice P.; Zucchelli G.; Cannizzaro G.; Barausse C.; Diazzi M.; Trullenque-Eriksson A.; Esposito M.	2016
Complete Edentulous Rehabilitation Using an Immediate Function Protocol and an Implant Design Featuring a Straight Body, Anodically Oxidized Surface, and Narrow Tip with Engaging Threads Extending to the Apex of the Implant: A 5-year Retrospective Clinical Study	Maló P.; de Araújo Nobre M.; Lopes A.; Ferro A.; Gravito I.	2016
A new micromotor allows an intraoperative assessment of bone density and primary implant stability in prosthetic rehabilitation: A case report	Greco G.B.; Kraus D.; Arosio P.; Di Stefano D.A.	2016
Provisional Restorations Used in Immediate Implant Placement Provide a Platform to Promote Peri-implant Soft Tissue Healing: A Pilot Study	Saito H.; Chu S.J.; Reynolds M.A.; Tarnow D.P.	2016
Single post-extractive ultra-wide 7 mm-diameter implants versus implants placed in molar healed sites after socket preservation for molar	Tallarico M.; Xhanari E.; Pisano M.; De Riu G.; Tullio A.; Meloni S.M.	2016

replacement: 6-month post-loading results from a randomised controlled trial		
Te impact on bone tissues of immediate implant-supported mandibular overdentures with cusped and cuspless teeth	Arafa K.A.	2016
Guided bone regeneration in distal mandibular atrophy by means of a preformed titanium foil: A case series	Andreasi Bassi M.; Andrisani C.; Lopez M.A.; Gaudio R.M.; Lombardo L.; Lauritano D.	2016
Marginal Bone Remodeling around healing Abutment vs Final Abutment Placement at Second Stage Implant Surgery: A 12-month Randomized Clinical Trial	Nader N.; Aboulhosn M.; Berberi A.; Manal C.; Younes R.	2016
Four mm-long versus longer implants in augmented bone in atrophic posterior jaws: 4-month post-loading results from a multicentre randomised controlled trial	Esposito M.; Zucchelli G.; Barausse C.; Pistilli R.; Trullenque-Eriksson A.; Felice P.	2016
Performance of zirconia abutments for implant-supported single-tooth crowns in esthetic areas: a retrospective study up to 12-year follow-up	Passos S.P.; Linke B.; Larjava H.; French D.	2016
Effect of roughened micro-threaded implant neck and platform switching on marginal bone loss: a multicenter retrospective study with 6-year follow-up	Di Stefano D.A.; Giacometti E.; Greco G.B.; Gastaldi G.; Gherlone E.	2016
Augmentation of the alveolar ridge compared with shorter implants in atrophic jaws: A meta-analysis based on randomised controlled trials	Yang J.; Cheng Z.; Shi B.	2016
Impact of radiotherapy on implant-based prosthetic rehabilitation in patients with head and neck cancer: A prospective observational study on implant survival and quality of life—Preliminary results	Ettl T.; Weindler J.; Gosau M.; Müller S.; Hautmann M.; Zeman F.; Koller M.; Papavasileiou D.; Bürgers R.; Driemel O.; Schneider I.; Klingelhöffer C.; Meier J.; Wahlmann U.; Reichert T.E.	2016
A 36-month follow-up prospective cohort study on peri-implant bone loss of Morse Taper connection implants with platform switching	Cassetta M.; Di Mambro A.; Giansanti M.; Brandetti G.; Calasso S.	2016
Timing of single implant placement and long-term observation of marginal bone levels	Schropp L.; Wenzel A.	2016
Patient selection and peri-interventional management	Vedantham S.	2016
Safety of anticoagulation with uninterrupted warfarin vs. interrupted dabigatran in patients requiring an implantable cardiac device	Madan S.; Muthusamy P.; Mowers K.L.; Elmouchi D.A.; Finta B.; Gauri A.J.; Woelfel A.K.; Fritz T.D.; Davis A.T.; Chalfoun N.T.	2016
The obese patient a problem of larger consequence	Perry K.I.; MacDonald S.J.	2016
The use of CALLOS®, a calcium phosphate cement, in open reduction and internal fixation (ORIF) for Tibial plateau fractures: a comparison with non supplemented ORIF	Perez-Viloria M.; De Angelis P.; Walley K.; Rodriguez E.; Appleton P.	2016
The obese patient: A problem of larger consequence	Perry K.I.; MacDonald S.J.	2016
Guided growth for tibia vara (Blount's disease)	Heflin J.A.; Ford S.; Stevens P.	2016
Pyeloplasty may not be needed after laparoscopic pyelolithotomy for giant staghorn in a horseshoe kidney	Chen K.; Lu J.; Tiong H.	2016
Adjudicating paravalvular leaks of transcatheter aortic valves: A critical appraisal	Abdelghani M.; Soliman O.I.I.; Schultz C.; Vahanian A.; Serruys P.W.	2016
Stability of simultaneously placed dental implants with autologous bone grafts harvested from the iliac crest or intraoral jaw bone	Kang Y.-H.; Kim H.-M.; Byun J.-H.; Kim U.-K.; Sung I.-Y.; Cho Y.-C.; Park B.-W.	2015
Prevalence of Peri-implant Diseases: Analyses of Associated Factors	Ferreira C.F.; Buttendorf A.R.; de Souza J.G.; Dalago H.; Guenther S.F.; Bianchini M.A.	2015
Peri-implant complications for posterior endosteal implants	Esquivel-Upshaw J.; Mehler A.; Clark A.; Neal D.; Gonzaga L.; Anusavice K.	2015
Dramatic course of osteomyelitis in a patient treated with immediately placed dental implants suffering from uncontrolled diabetes: A case report	Doll C.; Hartwig S.; Nack C.; Nahles S.; Nelson K.; Raguse J.-D.	2015
Sub-crestal positioning of implants results in higher bony crest resorption: an experimental study in dogs	Cesaretti G.; Lang N.P.; Salata L.A.; Schweikert M.T.; Gutierrez Hernandez M.E.; Botticelli D.	2015
A History of Periodontitis Suggests a Higher Risk for Implant Loss	Chrcanovic B.R.	2015
Immediate loading of post-extractive versus delayed placed single implants in the anterior maxilla: outcome of a pragmatic multicenter randomised controlled trial 1-year after loading	Esposito M.; Barausse C.; Pistilli R.; Jacotti M.; Grandi G.; Tuco L.; Felice P.	2015
Immediate non-occlusal loading of immediate post-extractive versus delayed placement of single implants in preserved sockets of the anterior maxilla: 1-year post-loading outcome of a randomised controlled trial	Felice P.; Pistilli R.; Barausse C.; Trullenque-Eriksson A.; Esposito M.	2015
Malpositioned implants in the anterior maxilla: a novel restorative approach to reestablish peri-implant tissue health and acceptable esthetics. Part II: Case report and discussion	Moráguez O.D.; Vailati F.; Belser U.C.	2015
Less Invasive Surgical Procedures Using Narrow-Diameter Implants: A Prospective Study in 20 Consecutive Patients	Lambert F.E.; Lecloux G.; Grenade C.; Bouhy A.; Lamy M.; Rompen E.H.	2015
Comparative Study of Immediate Functional Loading and Immediate Non-Functional Loading of Monocortical Implants	Singh J.P.; Gupta A.K.; Dhiman R.K.; Roy Chowdhury S.K.	2015
Immediately loaded machined versus rough surface dental implants in edentulous jaws: One-year postloading results of a pilot randomised controlled trial	Esposito M.; Felice P.; Barausse C.; Pistilli R.; Grandi G.; Simion M.	2015
The efficacy of BMP-2 preloaded on bone substitute or hydrogel for bone regeneration at peri-implant defects in dogs	Jung U.-W.; Lee I.-K.; Park J.-Y.; Thoma D.S.; Hämmerle C.H.; Jung R.E.	2015
Marginal masticatory mucosa dimensional changes in immediate post-extractive implants: a 2 year prospective cohort study	Ramaglia L.; Sbordone C.; Saviano R.; Martuscelli R.; Sbordone L.	2015
Short implants as an alternative to crestal sinus lift: A 1-year multicentre randomised controlled trial	Felice P.; Pistilli R.; Barausse C.; Bruno V.; Trullenque-Eriksson A.; Esposito M.	2015

Soft tissues stability of cad-cam and stock abutments in anterior regions: 2-year prospective multicentric cohort study	Lops D.; Bressan E.; Parpaiola A.; Sbricoli L.; Cecchinato D.; Romeo E.	2015
Food Impaction and Periodontal/Peri-Implant Tissue Conditions in Relation to the Embrasure Dimensions Between Implant-Supported Fixed Dental Prostheses and Adjacent Teeth: A Cross-Sectional Study	Jeong J.-S.; Chang M.	2015
Effects of Abutment Removal and Reconnection on Inflammatory Cytokine Production Around Dental Implants	Kuppusamy M.; Watanabe H.; Kasugai S.; Kuroda S.	2015
Effects of posterior ridge resorption and attachment wear on periimplant strain in mandibular two-implant-supported overdentures	Jo D.-W.; Dong J.-K.	2015
Adherence of oral streptococci to nanostructured titanium surfaces	Narendrakumar K.; Kulkarni M.; Addison O.; Mazare A.; Junkar I.; Schmuki P.; Sammons R.; Iglič A.	2015
High revision rate but good healing capacity of atypical femoral fractures. A comparison with common shaft fractures	Schilcher J.	2015
Do all patients need intense anticoagulation and antiplatelet regimens followingWATCHMAN device implantation? A case series of five patients	Ramkumar S.; Tung M.; Cameron J.; Pang B.; Nerlekar N.; Kotschet E.; Alison J.	2015
Peri-procedural myocardial injury and long-term mortality in percutaneous coronary intervention for stable coronary artery disease	Goh C.; Yudi M.; Lu K.; Al-Fiadh A.; Mok M.; Clark D.; Farouque O.	2015
Utility of intravascular ultrasound (IVUS) and its immediate impact on decision making on patients undergoing coronary intervention at a tertiary referral centre	Mirzaee S.; Sganzerla E.; Weerasooriya S.; Yadav R.	2015
Comparison of percutaneous device closure versus surgical closure of peri-membranous ventricular septal defects: A systematic review and meta-analysis	Saurav A.; Kaushik M.; Mahesh Alla V.; White M.D.; Satpathy R.; Lanspa T.; Mooss A.N.; Delcore M.G.	2015
Associations of known complications of transcatheter implantation of balloon-expandable edwards-sapien XT aortic valves with peri-procedural mortality-an insight from the 2014-2015 reports from the fda maude database	Ranjan P.; Chatterjee S.; Gupta D.; Sardar P.; Giri J.; Bhinder J.; Mukherjee D.; Correa A.; Kumbhani D.J.	2015
Trend and predictors of prolonged length of hospital stay following percutaneous coronary intervention for st elevation myocardial infarction in the United States: A 10 year perspective	Panchal H.B.; Veeranki S.P.; Bhatheja S.; Abusara A.; Paul T.	2015
Prevalence of peri-implant inflammatory disease in patients with a history of periodontal disease who receive supportive periodontal therapy	Aguirre-Zorzano L.A.; Estefanía-Fresco R.; Telletxea O.; Bravo M.	2015
Salvaging Implants With an Nd:YAG Laser: A Novel Approach to a Growing Problem	Suzuki J.B.	2015
Transglutaminase 2 May Be Associated with Peri-implant Gingival Overgrowth: Preliminary Assessments	Ceruti P.; Asioli S.; Mussano F.; Righi A.; Baldi I.; Schierano G.; Cassoni P.; Carossa S.	2015
Soft tissue and crestal bone changes around implants with platform-switched abutments placed nonsubmerged at subcrestal position: a 2-year clinical and radiographic evaluation	Aimetti M.; Ferrarotti F.; Mariani G.M.; Ghelardoni C.; Romano F.	2015
Titanium-zirconium narrow-diameter versus titanium regular-diameter implants for anterior and premolar single crowns: 3-year results of a randomized controlled clinical study	Ioannidis A.; Gallucci G.O.; Jung R.E.; Borzangy S.; Hämmerle C.H.; Benic G.I.	2015
Effect of Framework in an Implant-Supported Full-Arch Fixed Prosthesis: 3D Finite Element Analysis	Menini M.; Pesce P.; Bevilacqua M.; Pera F.; Tealdo T.; Barberis F.; Pera P.	2015
Reducing Fracture Risk Adjacent to a Plate with an Angulated Locked End Screw	Peck J.B.; Charpentier P.M.; Flanagan B.P.; Srivastava A.K.; Atkinson P.J.	2015
Comparison of Azithromycin and Amoxicillin Before Dental Implant Placement: An Exploratory Study of Bioavailability and Resolution of Postoperative Inflammation	Escalante M.G.; Eubank T.D.; Leblebicioglu B.; Walters J.D.	2015
Use of buccal fat pad to repair post-extraction peri-implant bone defects in the posterior maxilla. A preliminary prospective study	Peñarrocha-Diago M.; Alonso-González R.; Aloy-Prósper A.; Peñarrocha-Oltra D.; Camacho F.; Peñarrocha-Diago M.	2015
Dynamic locking plate vs simple cannulated screws for nondisplaced intracapsular hip fracture: A comparative study	Warschawski Y.; Snir N.; Steinberg E.	2015
Prophylactic antibiotics in elective hip and knee arthroplasty: An analysis of organisms reported to cause infections and national survey of clinical practice	Hickson C.J.; Metcalfe D.; Elgohari S.; Oswald T.; Masters J.P.; Rymaszewska M.; Reed M.R.; Sprowson A.P.	2015
Evaluation in a dog model of three antimicrobial glassy coatings: Prevention of bone loss around implants and microbial assessments	López-Píriz R.; Solá-Linares E.; Rodriguez-Portugal M.; Malpica B.; Díaz-Güemes I.; Enciso S.; Esteban-Tejeda L.; Cabal B.; Granizo J.J.; Moya J.S.; Torrecillas R.	2015
Deep brain stimulation complicated by bilateral large cystic cavitation around the leads in a patient with Parkinson's disease	Jagid J.; Madhavan K.; Desai N.; Bregy A.; Desai M.; Ruiz A.; Quencer R.; Landy H.J.	2015
5-year outcome of consecutive left main coronary artery percutaneous coronary interventions	Piroth Z.; Ferenci T.; Fontos G.; Szonyi T.; Nemeth J.; Szoke S.; Chaurasia A.K.; Andreka P.	2015
Endoscopic Transpapillary Gallbladder Stent Placement Is Safe and Effective in High-Risk Patients Without Cirrhosis	McCarthy S.T.; Tujios S.; Fontana R.J.; Rahnama-Moghadam S.; Elmunzer B.J.; Kwon R.S.; Wamsteker E.J.; Anderson M.A.; Scheiman J.M.; Elta G.H.; Piraka C.R.	2015
Efficacy of air polishing for the non-surgical treatment of peri-implant diseases: a systematic review	Schwarz F.; Becker K.; Renvert S.	2015
10-year survival rate and the incidence of peri-implant disease of 374 titanium dental implants with a SLA surface: a prospective cohort study in 177 fully and partially edentulous patients	van Velzen F.J.; Ofec R.; Schulten E.A.; Ten Bruggenkate C.M.	2015
Twelve-Year Retrospective Follow-Up of Machined Implants in the Posterior Maxilla: Radiographic and Peri-Implant Outcome	Simion M.; Gionso L.; Grossi G.B.; Briguglio F.; Fontana F.	2015
Experimental Peri-Implantitis around Different Types of Implants - A Clinical and Radiographic Study in Dogs	Fickl S.; Kebschull M.; Calvo-Guirado J.L.; Hürzeler M.; Zuhr O.	2015

Peri-Implantitis Associated with Type of Cement: A Retrospective Analysis of Different Types of Cement and Their Clinical Correlation to the Peri-Implant Tissue	Korsch M.; Walther W.	2015
Implant in vascularized fibula graft reconstructed for segment mandibular defect	Wu Y.; Yao J.; Shi W.; Yang X.; Ding X.; Song X.	2015
A comparative study for the long-term stability of simultaneously placed dental implants with autologous bone graft harvested from iliac crest and maxillomandibular bone	Kang Y.; Byun J.; Kim H.; Park B.	2015
Peri-Implant Response and Microflora in Organ Transplant Patients 1 Year after Prosthetic Loading: A Prospective Controlled Study	Montebugnoli L.; Venturi M.; Cervellati F.; Servidio D.; Vocale C.; Pagan F.; Landini M.P.; Magnani G.; Sambri V.	2015
Long-Term Retrospective Clinical and Radiographic Follow-up of 205 Brånemark System Mk III TiUnite Implants Submitted to Either Immediate or Delayed Loading	Imburgia M.; Del Fabbro M.	2015
Clinical and Radiographic Evaluation of Marginal Bone Changes around Platform-Switching Implants Placed in Crestal or Subcrestal Positions: A Randomized Controlled Clinical Trial	Kütan E.; Bolukbasi N.; Yildirim-Ondur E.; Ozdemir T.	2015
Effect of Platform Shift/Switch and Concave Abutments on Crestal Bone Levels and Mucosal Profile following Flap and Flapless Implant Surgery	Gamborena I.; Lee J.; Fiorini T.; Wenzel B.A.; Schüpbach P.; Wikesjö U.M.; Susin C.	2015
Increasing the Width of Keratinized Mucosa in Maxillary Implant Areas Using a Split Palatal Bridge Flap: Surgical Technique and 1-Year Follow-Up	Frisch E.; Ratka-Krüger P.; Ziebolz D.	2015
A Comparison of Zirconia and Titanium Abutments for Microleakage	Abdelhamed M.I.; Galley J.D.; Bailey M.T.; Johnston W.M.; Holloway J.; McGlumphy E.; Leblebicioglu B.	2015
Early Periimplant Tissue Healing on 1-Piece Implants With a Concave Transmucosal Design: A Histomorphometric Study in Dogs	Bolle C.; Gustin M.-P.; Fau D.; Exbrayat P.; Boivin G.; Grosgogeat B.	2015
Retrospective analysis of 26 complete-arch implant-supported monolithic zirconia prostheses with feldspathic porcelain veneering limited to the facial surface	Venezia P.; Torsello F.; Cavalcanti R.; D'Amato S.	2015
Four-Millimeter-Long Posterior-Mandible Implants: 5-Year Outcomes of a Prospective Multicenter Study	Slotte C.; Grønningsaeter A.; Halmøy A.-M.; Öhrnell L.-O.; Mordenfeld A.; Isaksson S.; Johansson L.	2015
Determination of implant length in maxillary anterior region	Ebenezer V.; Balakrishnan R.; Muthumani T.; Prakash D.	2015
Restoration of Oral Function for Adult Edentulous Patients with Ectodermal Dysplasia: A Prospective Preliminary Clinical Study	Wu Y.; Wang X.D.; Wang F.; Huang W.; Zhang Z.; Zhang Z.; Kaigler D.; Zou D.	2015
A Clinical Study Evaluating Changes in the Microbial Flora Around Dental Implants During Various Stages of Implant Restoration	Herekar M.; Sethi M.; Prithviraj D.R.; Bhat K.; Fernandes A.; Patil V.	2015
Can Periimplantitis Be Treated?	Fu J.-H.; Wang H.-L.	2015
Single-Tooth Rehabilitations Supported by Dental Implants Used in an Immediate-Provisionalization Protocol: Report on Long-Term Outcome with Retrospective Follow-Up	Maló P.; de Araújo Nobre M.; Lopes A.; Ferro A.; Gravito I.	2015
IL1RN gene polymorphism in a Portuguese population with implant-supported overdentures - An observational study	Sampaio-Fernandes M.; Vaz P.C.; Braga A.C.; Figueiral M.H.	2015
The NobelGuide® All-on-4® Treatment Concept for Rehabilitation of Edentulous Jaws: A Prospective Report on Medium- and Long-Term Outcomes	Lopes A.; Maló P.; de Araújo Nobre M.; Sanchez-Fernández E.	2015
Usefullness of laser in oral and maxillofacial surgery	Lal K.; Parthiban J.; Sargunar B.; Prakash C.A.; Anandh B.	2015
Role of horizontal gap on the success of immediate implant	Ghanem W.A.	2015
Blood transfusion and anaemia in patients undergoing posterior lumbar interbody fusion	Lalabekyan B.; Moss C.; Luoma A.	2015
Management challenges of congenital & early onset childhood hearing loss in a sub-Saharan African country	Adedeji T.O.; Tobih J.E.; Sogebi O.A.; Daniel A.D.	2015
Intrathoracic impedance monitoring for heart failure	Khosla A.; Shanahan C.; Scatena R.	2015
Anterior lumbar interbody fusion with integrated fixation and adjunctive posterior stabilization: A comparative biomechanical analysis	Yeager M.S.; Dupre D.A.; Cook D.J.; Oh M.Y.; Altman D.T.; Cheng B.C.	2015
Outcomes of percutaneous transhepatic cholangiography for the relief of malignant jaundice in England between 2001 and 2014	Rees J.; Trudgill N.; Evison F.; Mytton J.; Patel P.	2015
Metastatic breast cancer in medication-related osteonecrosis around mandibular implants	Favia G.; Tempesta A.; Limongelli L.; Crincoli V.; Piattelli A.; Maiorano E.	2015
Transcatheter aortic valve implantation - update and evidence	Van Linden A.; Doss M.; Arsalan M.; Kim W.K.; Walther T.	2015
Peri-implant evaluation in type 2 diabetes mellitus patients: a 3-year study	Gómez-Moreno G.; Aguilar-Salvatierra A.; Rubio Roldán J.; Guardia J.; Gargallo J.; Calvo-Guirado J.L.	2015
Preventing and Treating Peri-Implantitis: A Cost-Effectiveness Analysis	Schwendicke F.; Tu Y.-K.; Stolpe M.	2015
The patient undergoing implant therapy. Summary and consensus statements. The 4th EAO Consensus Conference 2015	Klinge B.; Flemming T.; Cosyn J.; De Bruyn H.; Eisner B.M.; Hultin M.; Isidor F.; Lang N.P.; Lund B.; Meyle J.; Mombelli A.; Navarro J.M.; Pjetursson B.; Renvert S.; Schliephake H.	2015
Implants failures related to endodontic treatment. An observational retrospective study	López-Martínez F.; Gómez Moreno G.; Olivares-Ponce P.; Eduardo Jaramillo D.; Eduardo Maté Sánchez de Val J.; Calvo-Guirado J.L.	2015
Technical and biological complications of single-molar implant restorations	Rinke S.; Roediger M.; Eickholz P.; Lange K.; Ziebolz D.	2015
Prevalence and Possible Risk Factors of Peri-implantitis: A Concept Review	Marcantonio C.; Nicoli L.G.; Marcantonio Junior E.; Zandim-Barcelos D.L.	2015

Risk indicators for peri-implantitis. A narrative review	Renvert S.; Quirynen M.	2015
Characterization of Cement Particles Found in Peri-implantitis-Affected Human Biopsy Specimens	Burbano M.; Wilson T.G.; Valderrama P.; Blansett J.; Wadhwani C.P.; Choudhary P.K.; Rodriguez L.C.; Rodrigues D.C.	2015
Peri-implantitis Treatment with a Regenerative Approach: Clinical Outcomes on Reentry	Parma-Benfenati S.; Roncati M.; Galletti P.; Tinti C.	2015
Peri-implant mucosal recession: Clinical significance and therapeutic opportunities	Fickl S.	2015
Change in the Gingival Margin Profile After the Single Flap Approach in Periodontal Intraosseous Defects	Farina R.; Simonelli A.; Minenna L.; Rasperini G.; Schincaglia G.P.; Tomasi C.; Trombelli L.	2015
The effect of zirconia or titanium as abutment material on soft peri-implant tissues: a systematic review and meta-analysis	Linkevicius T.; Vaitelis J.	2015
Unsplinted implants and teeth supporting maxillary removable partial dentures retained by telescopic crowns: a retrospective study with >6 years of follow-up	Frisch E.; Ratka-Krüger P.; Wenz H.-J.	2015
Posterior jaws rehabilitated with partial prostheses supported by 4.0 x 4.0 mm or by longer implants: Four-month post-loading data from a randomised controlled trial	Esposito M.; Barausse C.; Pistilli R.; Checchi V.; Diazzi M.; Gatto M.R.; Felice P.	2015
Short implants versus bone augmentation for placing longer implants in atrophic maxillae: One-year post-loading results of a pilot randomised controlled trial	Esposito M.; Barausse C.; Pistilli R.; Sammartino G.; Grandi G.; Felice P.	2015
Evaluation of Clinical Parameters and Levels of Proinflammatory Cytokines in the Crevicular Fluid Around Dental Implants in Patients with Type 2 Diabetes Mellitus	Dŏgan ŞB.; Kurtiş M.B.; Tüter G.; Serdar M.; Watanabe K.; Karakış S.	2015
Safety and effectiveness of early loaded maxillary titanium implants with a novel nanostructured calcium-incorporated surface (Xpeed): 3-year results from a pilot multicenter randomised controlled trial	Felice P.; Grusovin M.G.; Barausse C.; Grandi G.; Esposito M.	2015
Implant site preparation using a single bur versus multiple drilling steps: 4-month post-loading results of a multicenter randomised controlled trial	Guazzi P.; Grandi T.; Grandi G.	2015
Marginal bone and soft tissue behavior following platform switching abutment connection/disconnection--a dog model study	Alves C.C.; Muñoz F.; Cantalapiedra A.; Ramos I.; Neves M.; Blanco J.	2015
Comparing membranes and bone substitutes in a one-stage procedure for horizontal bone augmentation. A double-blind randomised controlled trial	Merli M.; Moscatelli M.; Mariotti G.; Pagliaro U.; Raffaelli E.; Nieri M.	2015
The Concept of Platform Switching to Preserve Peri-implant Bone Level: Assessment of Methodologic Quality of Systematic Reviews	Monje A.; Pommer B.	2015
Crestal bone loss and periimplant inflammatory parameters around zirconia implants: A systematic review	Vohra F.; Al-Kheraif A.A.; Ab Ghani S.M.; Abu Hassan M.I.; Alnassar T.; Javed F.	2015
Factors affecting peri-implant bone loss: a post-five-year retrospective study	Vázquez Álvarez R.; Pérez Sayáns M.; Gayoso Diz P.; García García A.	2015
Bone and Calcium Metabolisms Associated with Dental and Oral-Maxillofacial Diseases. Destructive bone resorption by osteoclasts and oral disease	Nakamura M.; Koide M.; Nakamura H.; Udagawa N.	2015
Associated lumbar scoliosis does not affect outcomes in patients undergoing focal minimally invasive transforaminal lumbar interbody fusion (MISTLIF) for neurogenic symptoms: A minimum 2-year follow-up study	Bassi A.; Tay K.S.; Yeo W.; Yue W.M.	2015
Ligature-induced peri-implantitis in mice	Pirih F.Q.; Hiyari S.; Barroso A.D.; Jorge A.C.; Perussolo J.; Atti E.; Tetradis S.; Camargo P.M.	2015
Maintenance therapy in patients following the surgical treatment of peri-implantitis: a 5-year follow-up study	Serino G.; Turri A.; Lang N.P.	2015
Assessment of periodontal and opportunistic flora in patients with peri-implantitis	Albertini M.; López-Cerero L.; O'Sullivan M.G.; Chereguini C.F.; Ballesta S.; Ríos V.; Herrero-Climent M.; Bullón P.	2015
A Prognosis System for Periimplant Diseases	Decker A.M.; Sheridan R.; Lin G.-H.; Sutthiboonyapan P.; Carroll W.; Wang H.-L.	2015
Changes of Marginal Bone Level in Patients with "Progressive Bone Loss" at Brånemark System® Implants: A Radiographic Follow-Up Study over an Average of 9 Years	Jemt T.; Sundén Pikner S.; Gröndahl K.	2015
Long-Term (9-12 Years) Outcomes of Titanium Implants With an Oxidized Surface: A Retrospective Investigation on 209 Implants	Mozzati M.; Gallesio G.; Del Fabbro M.	2015
Medium- and Long-Term Complications in Full-Arch Rehabilitations Supported by Upright and Tilted Implants	Francetti L.; Corbella S.; Taschieri S.; Cavalli N.; Del Fabbro M.	2015
Immediate loading short implants inserted on low bone quantity for the rehabilitation of the edentulous maxilla using an All-on-4 design	Maló P.; de Araújo Nobre M.A.; Lopes A.V.; Rodrigues R.	2015
Association of Keratinized Mucosa and Periimplant Soft Tissue Stability Around Implants With Platform Switching	Romanos G.; Grizas E.; Nentwig G.-H.	2015
Evaluation of Hi-Tec implant restoration in mandibular first molar region- A prospective clinical study	Sreeram R.R.S.; Krishna Prasad L.; Chakravarthi P.S.; Devi N.N.; Kattimani V.S.; Sreeram S.K.	2015
Lasers in Implant Dentistry, Part 2	van As G.A.	2015
The Effect of Parathyroid Hormone on Osseointegration in Insulin-Treated Diabetic Rats	Rybaczek T.; Tangl S.; Dobsak T.; Gruber R.; Kuchler U.	2015
Impact of optical coherence tomography findings during percutaneous coronary intervention on 9-month follow-up outcomes	Sugiyama T.; Kimura S.; Yamakami Y.; Kojima K.; Sagawa Y.; Ohtani H.; Hishikari K.; Hikita H.; Takahashi A.; Isobe M.	2015
Microbiologic and Clinical Findings of Implants in Healthy Condition and with Peri-Implantitis	Canullo L.; Peñarrocha-Oltra D.; Covani U.; Rossetti P.H.	2015
Significance of the width of keratinized mucosa on peri-implant health	Chiu Y.-W.; Lee S.-Y.; Lin Y.-C.; Lai Y.-L.	2015

Maintenance and Monitoring of Dental Implants in General Dental Practice	Tarawali K.	2015
What Are the Implications of Peri-Implantitis?	Nevins M.	2015
Buccal bone deficiency in fresh extraction sockets: a prospective single cohort study	Barone A.; Ricci M.; Romanos G.E.; Tonelli P.; Alfonsi F.; Covani U.	2015
The Effect of Interimplant Distance on Peri-implant Bone and Soft Tissue Dimensional Changes: A Nonrandomized, Prospective, 2-Year Follow-up Study	Koutouzis T.; Neiva R.; Lipton D.; Lundgren T.	2015
Clinical Outcome of Double Crown-Retained Mandibular Removable Dentures Supported by a Combination of Residual Teeth and Strategic Implants	Rinke S.; Ziebolz D.; Ratka-Krüger P.; Frisch E.	2015
Influence of collar design on peri-implant tissue healing around immediate implants: A pilot study in Foxhound dogs	Calvo-Guirado J.L.; López-López P.J.; Maté Sánchez de Val J.E.; Mareque-Bueno J.; Delgado-Ruiz R.A.; Romanos G.E.	2015
An Up-to-15-Year Comparison of the Survival and Complication Burden of Three-Unit Tooth-Supported Fixed Dental Prostheses and Implant-Supported Single Crowns	Walton T.R.	2015
Biological width formation to immediate implants placed at different level in relation to the crestal bone: an experimental study in dogs	Negri B.; López Marí M.; Maté Sánchez de Val J.E.; Iezzi G.; Bravo González L.A.; Calvo Guirado J.L.	2015
Marginal bone loss evaluation around immediate non-occlusal microthreaded implants placed in fresh extraction sockets in the maxilla: a 3-year study	Calvo-Guirado J.L.; Gómez-Moreno G.; Aguilar-Salvatierra A.; Guardia J.; Delgado-Ruiz R.A.; Romanos G.E.	2015
The influence of the connection, length and diameter of an implant on bone biomechanics	Borie E.; Orsi I.A.; de Araujo C.P.	2015
Narrow- versus mini-implants at crestal and subcrestal bone levels. Experimental study in beagle dogs at three months	Calvo-Guirado J.L.; Pérez-Albacete C.; Aguilar-Salvatierra A.; de Val Maté-Sánchez J.E.; Delgado-Ruiz R.A.; Abboud M.; Velasco E.; Gómez-Moreno G.; Romanos G.E.	2015
Implant-Supported Telescopic Crown-Retained Overdentures for Oral Rehabilitation of Patients with Severe Bony Defects: A 5-Year Retrospective Study	Zou D.; Wang F.; Wu Y.; Huang W.; Zhang C.; Zhang Z.	2015
Particle characterisation and cytokine expression in failed small-diameter metal-on-metal total hip arthroplasties	Singh G.; Nuechtern J.V.; Meyer H.; Fiedler G.M.; Awiszus F.; Junk-Jantsch S.; Bruegel M.; Pflueger G.; Lohmann C.H.	2015
An audit of the roll out of the health service executive's national bone anchored hearing aid programme in the south infirmary Victoria university hospital	O'Leary J.; O'Sullivan P.	2015
An exploratory study to investigate if patients are able to aid the early diagnosis of peri-implant complications	Wright S.; Archer C.	2015
Animal models for peri-implant mucositis and peri-implantitis	Schwarz F.; Sculean A.; Engebretson S.P.; Becker J.; Sager M.	2015
Clinical approaches to treat peri-implant mucositis and peri-implantitis	Renvert S.; Polyzois I.N.	2015
Soft and Hard Tissue Response to Zirconia versus Titanium One-Piece Implants Placed in Alveolar and Palatal Sites: A Randomized Control Trial	Siddiqi A.; Kieser J.A.; De Silva R.K.; Thomson W.M.; Duncan W.J.	2015
Soft tissue grafting to improve the attached mucosa at dental implants: A review of the literature and proposal of a decision tree	Bassetti M.; Kaufmann R.; Salvi G.E.; Sculean A.; Bassetti R.	2015
Risk factors of peri-implant pathology	de Araújo Nobre M.; Mano Azul A.; Rocha E.; Maló P.	2015
Modified implant surface with slower and less initial biofilm formation	John G.; Becker J.; Schwarz F.	2015
Dimensions of the healthy gingiva and peri-implant mucosa	Parpaiola A.; Cecchinato D.; Toia M.; Bressan E.; Speroni S.; Lindhe J.	2015
Survival of implants in immediate extraction sockets of anterior teeth: Early clinical results	Sabir M.; Alam M.N.	2015
Immediate, early (3 weeks) and conventional loading (4 months) of single implants: Preliminary data at 1 year after loading from a pragmatic multicenter randomised controlled trial	Grandi T.; Guazzi P.; Samarani R.; Tohme H.; Khoury S.; Sbricoli L.; Grandi G.; Esposito M.	2015
Do repeated changes of abutments have any influence on the stability of peri-implant tissues? Four-month post-loading preliminary results from a multicentre randomised controlled trial	Luongo G.; Bressan E.; Grusovin M.G.; d'Avenia F.; Neumann K.; Sbricoli L.; Esposito M.	2015
Periodontal and peri-implant wound healing following laser therapy	Aoki A.; Mizutani K.; Schwarz F.; Sculean A.; Yukna R.A.; Takasaki A.A.; Romanos G.E.; Taniguchi Y.; Sasaki K.M.; Zeredo J.L.; Koshy G.; Coluzzi D.J.; White J.M.; Abiko Y.; Ishikawa I.; Izumi Y.	2015
Laser Therapy is Safe but not Superior to Conventional Treatment of Peri-implantitis	Romanos G.E.; Javed F.	2015
Plaque score change as an indicator for periimplant health in periodontal patients with immediately restored dental implants	Gabay E.; Levin L.; Zuabi O.; Horwitz J.	2015
Raloxifene enhances peri-implant bone healing in osteoporotic rats	Ramalho-Ferreira G.; Faverani L.P.; Prado F.B.; Garcia I.R.; Okamoto R.	2015
Peri-implant bone response following immediate implants placed in the esthetic zone and with immediate provisionalization--a case series study	Martin C.; Thomé G.; Melo A.C.; Fontão F.N.	2015
Chlorhexidine anaphylaxis: The hidden trigger	Pettipher A.; Duggleby P.	2015
Distal femoral fractures: The need to review the standard of care	Smith J.R.A.; Halliday R.; Aquilina A.L.; Morrison R.J.M.; Yip G.C.K.; McArthur J.; Hull P.; Gray A.; Kelly M.B.	2015
Perioperative behavioral therapy & pelvic muscle strengthening does not improve quality of life after apical prolapse surgery: A randomized controlled trial	Weidner A.C.; Barber M.D.; Markland A.D.; Rahn D.D.; Hsu Y.; Mueller E.R.; Jakuswaldman S.; Dyer K.Y.; Warren L.K.; Gantz M.; Meikle S.	2015

Interventions for replacing missing teeth: Alveolar ridge preservation techniques for dental implant site development	Atieh M.A.; Alsabeeha N.H.; Payne A.G.; Duncan W.; Faggion C.M.; Esposito M.	2015
Cost-effectiveness of non-surgical peri-implantitis treatments	Listl S.; Frühauf N.; Dannewitz B.; Weis C.; Tu Y.-K.; Chang H.-J.; Faggion C.M.	2015
Unintentional root fragment retention in proximity to dental implants: a series of six human case reports	Langer L.; Langer B.; Salem D.	2015
Minimally invasive extraction and flapless immediate implant placement with no bone substitute: A 1year retrospective analysis	Li H.; Kong X.; Chen J.; Chen X.; Zhang Z.; Cao W.; Ge Y.; Fang S.	2015
Hard and soft tissue surgical complications in dental implantology	Aziz S.R.	2015
A multicenter randomized comparative trial of implants with different abutment interfaces to replace anterior maxillary single teeth	Cooper L.F.; Reside G.; Stanford C.; Barwacz C.; Feine J.; Abi Nader S.; Scheyer E.T.; McGuire M.	2015
Treatment of Peri-implant Bone Defects with Platelet-Rich Fibrin	Hamzacebi B.; Oduncuoglu B.; Alaaddinoglu E.E.	2015
Implant angulation: 2-year retrospective analysis on the influence of dental implant angle insertion on marginal bone resorption in maxillary and mandibular osseous onlay grafts	Ramaglia L.; Toti P.; Sbordone C.; Guidetti F.; Martuscelli R.; Sbordone L.	2015
Fate of the buccal bone at implants placed early, delayed, or late after tooth extraction analyzed by cone beam CT: 10-year results from a randomized, controlled, clinical study	Schropp L.; Wenzel A.; Spin-Neto R.; Stavropoulos A.	2015
FGDP(UK) Divisional Update: Contemporary Periodontal and Peri-implant Management	Lane N.	2015
Key nurse-sensitive outcomes in post-transcatheter aortic valve implantation (TAVI) procedure recovery	Johnston R.	2015
Prospective trial of intracranial stent placement in patients with symptomatic high-grade intracranial stenosis (70-99%)	Gao P.; Jiao L.; Zhao Z.; Cai Y.; Li T.; Shi H.; Wu W.; He W.; Yin L.; Huang S.; Wang D.	2015
Filter particle analysis correlates intracerebral microembolization risk during angioplasty and stenting of vulnerable carotid plaque	Li J.; Mani V.; Giannarelli C.; Gordon E.; Krishnan P.; Tadros R.; Fayad Z.; Faries P.; Wiley J.	2015
Novel applications for lumen apposing metal stents: How far can WE go?	Sharaiha R.Z.; Tyberg A.; Desai A.P.; Benias P.C.; Kumbhari V.; Dimaio C.J.; Sejpal D.V.; Sethi A.; Ho S.; Tieu A.H.; Gaidhane M.; Khashab M.; Carr-Locke D.L.; Kahaleh M.	2015
Hip abductor strength association with functional performance of patients after unilateral total knee arthroplasty: A longitudinal study	Chan A.C.M.; Yung B.P.K.; Tsui A.S.K.; Kwok A.C.F.; Tong J.M.C.	2015
Clinical, microbiological, and immunological aspects of healthy versus peri-implantitis tissue in full arch reconstruction patients: a prospective cross-sectional study	Ata-Ali J.; Flichy-Fernández A.J.; Alegre-Domingo T.; Ata-Ali F.; Palacio J.; Peñarrocha-Diago M.	2015
Primary prevention of peri-implantitis: managing peri-implant mucositis	Jepsen S.; Berglundh T.; Genco R.; Aass A.M.; Demirel K.; Derks J.; Figuero E.; Giovannoli J.L.; Goldstein M.; Lambert F.; Ortiz-Vigon A.; Polyzois I.; Salvi G.E.; Schwarz F.; Serino G.; Tomasi C.; Zitzmann N.U.	2015
Treatment of peri-implantitis and the failing implant	Robertson K.; Shahbazian T.; MacLeod S.	2015
Marginal bone loss as success criterion in implant dentistry: beyond 2 mm	Galindo-Moreno P.; León-Cano A.; Ortega-Oller I.; Monje A.; O'Valle F.; Catena A.	2015
Primary and secondary prevention of periodontal and peri-implant diseases: Introduction to, and objectives of the 11th European Workshop on Periodontology consensus conference	Tonetti M.S.; Chapple I.L.; Jepsen S.; Sanz M.	2015
Risk indicators for peri-implant mucositis: a systematic literature review	Renvert S.; Polyzois I.	2015
Effect of different prosthetic abutments on peri-implant soft tissue. A randomized controlled clinical trial	Ferrari M.; Cagidiaco M.C.; Garcia-Godoy F.; Goracci C.; Cairo F.	2015
Dental implants are a viable alternative for compensating oligodontia in adolescents	Heuberer S.; Dvorak G.; Mayer C.; Watzek G.; Zechner W.	2015
Effect of Platelet Rich Fibrin (PRF) on peri-implant soft tissue and crestal bone in one-stage implant placement: A randomized controlled trial	Boora P.; Rathee M.; Bhoria M.	2015
Photodynamic therapy and periodontal treatment	Gümü P.; Buduneli N.	2015
Effect of professional mechanical plaque removal on secondary prevention of periodontitis and the complications of gingival and periodontal preventive measures: consensus report of group 4 of the 11th European Workshop on Periodontology on effective prevention of periodontal and peri-implant diseases	Sanz M.; Bäumer A.; Buduneli N.; Dommisch H.; Farina R.; Kononen E.; Linden G.; Meyle J.; Preshaw P.M.; Quirynen M.; Roldan S.; Sanchez N.; Sculean A.; Slot D.E.; Trombelli L.; West N.; Winkel E.	2015
Principles in prevention of periodontal diseases: Consensus report of group 1 of the 11th European Workshop on Periodontology on effective prevention of periodontal and peri-implant diseases	Tonetti M.S.; Eickholz P.; Loos B.G.; Papapanou P.; van der Velden U.; Armitage G.; Bouchard P.; Deinzer R.; Dietrich T.; Hughes F.; Kocher T.; Lang N.P.; Lopez R.; Needleman I.; Newton T.; Nibali L.; Pretzl B.; Ramseier C.; Sanz-Sanchez I.; Schlagenhauf U.; Suvan J.E.	2015
A 5-year retrospective study on Replace Select Tapered dental implants	Pettersson P.; Sennerby L.	2015
All-ceramic restoration of zirconia two-piece implants--a randomized controlled clinical trial	Payer M.; Heschl A.; Koller M.; Arnetzl G.; Lorenzoni M.; Jakse N.	2015
The use of acellular dermal matrix membrane for vertical soft tissue augmentation during submerged implant placement: a case series	Puisys A.; Vindasiute E.; Linkevciene L.; Linkevicius T.	2015
Full Mouth Implant-supported Rehabilitation of a Patient with Ectodermal Dysplasia: Clinical Report and Literature Review	Kutkut A.; Abu-Eid R.; Sharab L.; Abadi B.; Van Sickels J.	2015
One-Year Prospective Study of 7-mm-Long Implants in the Mandible: Installation Technique and Crown/Implant Ratio of 1.5 or Less	Kim Y.-K.; Yun P.-Y.; Yi Y.-J.; Bae J.-H.; Kim S.-B.; Ahn G.-J.	2015

Dehydrated human amniotic membrane allograft nerve wrap around the prostatic neurovascular bundle accelerates early return to continence and potency following radical robot assisted radical prostatectomy : A propensity score matched analysis	Kumar A.; Samavedi S.; Bates A.; Coelho R.; Rocco B.; Marquinez J.; Camacho I.; Jenson C.; Palmer K.; Patel V.	2015
Effect of professional mechanical plaque removal performed on a long-term, routine basis in the secondary prevention of periodontitis: a systematic review	Trombelli L.; Franceschetti G.; Farina R.	2015
Periimplant bacteria associated with different transmucosal designs or smoking habits	Vacharaksa A.; Kuptanon M.; Sripitiroj R.; Serichetaphongse P.	2015
Cellular microparticles as predictive markers for adverse events in patients with implanted ventricular assist devices	Walenga J.; Jeske W.; Schwartz J.; Escalante V.; Menapace B.; Coglianese E.; Kumar E.; Heroux A.; Bakhos M.	2015
Introduction of the LINX® reflux management system in a community heartburn center	Woody M.; Willems K.; Vassaur H.; Wheeless C.J.; Buckley III F.P.	2015
Comparison of procedural complications and in-hospital clinical outcomes between patients with successful and failed percutaneous intervention of coronary chronic total occlusions: A Meta-Analysis of Observational Studies	Khan M.F.; Brilakis E.S.; Wendel C.S.; Thai H.	2015
Bariatric surgery in the elderly is associated with similar surgical morbidity and significant long term health benefits	Kaplan U.; Penner S.; Farrokhyar F.; Gmora S.; Hong D.; Anvari M.	2015
Implant materials generate different peri-implant inflammatory factors: Poly-ether-ether-ketone promotes fibrosis and microtextured titanium promotes osteogenic factors	Olivares-Navarrete R.; Hyzy S.L.; Slosar P.J.; Schneider J.M.; Schwartz Z.; Boyan B.D.	2015
Different contribution of BRINP3 gene in chronic periodontitis and peri-implantitis: a cross-sectional study	Casado P.L.; Aguiar D.P.; Costa L.C.; Fonseca M.A.; Vieira T.C.; Alvim-Pereira C.C.; Alvim-Pereira F.; Deeley K.; Granjeiro J.M.; Trevilatto P.C.; Vieira A.R.	2015
Prevalence and predictive factors for peri-implant disease and implant failure: a cross-sectional analysis	Daubert D.M.; Weinstein B.F.; Bordin S.; Leroux B.G.; Flemming T.F.	2015
Peri-implant diseases: an overview	Warreth A.; Boggs S.; Ibieyou N.; El-Helali R.; Hwang S.	2015
Multiscale analyses of the bone-implant interface	Cha J.Y.; Pereira M.D.; Smith A.A.; Houschyar K.S.; Yin X.; Mouraret S.; Brunski J.B.; Helms J.A.	2015
Immediate loading with a novel implant featured by variable-threaded geometry, internal conical connection and platform shifting: three-year results from a prospective cohort study	Pozzi A.; Tallarico M.; Moy P.K.	2015
The outcome of oral implants placed in bone with limited bucco-oral dimensions: a 3-year follow-up study	Temmerman A.; Keestra J.A.; Coucke W.; Teughels W.; Quirynen M.	2015
Periodontal considerations in older individuals	Darby I.	2015
Influence of the crown-implant connection on the preservation of peri-implant bone: a retrospective multifactorial analysis	Galindo-Moreno P.; Fernández-Jiménez A.; O'Valle F.; Monje A.; Silvestre F.J.; Juodzbalys G.; Sánchez-Fernández E.; Catena A.	2015
Immediate loading of fixed cross-arch prostheses supported by flapless-placed supershort or long implants: 1-year results from a randomised controlled trial	Cannizzaro G.; Felice P.; Buti J.; Leone M.; Ferri V.; Esposito M.	2015
Clinical evaluation of two dental implant macrostructures on peri-implant bone loss: a comparative, retrospective study	Dagorne C.; Malet J.; Bizouard G.; Mora F.; Rangé H.; Bouchard P.	2015
Impact of platform switching on marginal peri-implant bone-level changes. A systematic review and meta-analysis	Strietzel F.P.; Neumann K.; Hertel M.	2015
Crestal bone remodeling around implants placed using a short drilling protocol	Bratu E.; Mihali S.; Shapira L.; Bratu D.C.; Wang H.-L.	2015
Masticatory rehabilitation following upper and lower jaw reconstruction using vascularised free fibula flap and enossal implants-19 years of experience with a comprehensive concept	Hakim S.G.; Kimmerle H.; Trenkle T.; Sieg P.; Jacobsen H.-C.	2015
Effect of Porphyromonas gingivalis lipopolysaccharide on bone marrow mesenchymal stem cell osteogenesis on a titanium nanosurface	Xing H.; Taguchi Y.; Komasa S.; Yamawaki I.; Sekino T.; Umeda M.; Okazaki J.	2015
Histomorphometric assessment of the influence of low-level laser therapy on peri-implant tissue healing in the rabbit mandible	Massotti F.P.; Gomes F.V.; Mayer L.; de Oliveira M.G.; Baraldi C.E.; Ponzoni D.; Puricelli E.	2015
Self-reported pain perception of patients after mucosal graft harvesting in the palatal area	Burkhardt R.; Hämmerle C.H.; Lang N.P.	2015
Comparison of stapling techniques and management of the mesoappendix in laparoscopic appendectomy	Paul Wright G.; Mitchell E.J.; McClure A.M.; Onesti J.K.; Moyo S.C.; Brown A.R.; Peshkepija A.; Scott G.L.; Chung M.H.	2015
Osteoprotegerin expression in the peri-implant sulcus fluid after mechanical stimulations at different angles	Fang M.; Wang Z.-Y.; Yu J.; Li M.; Jin D.	2015
A cross-sectional assessment of biomarker levels around implants versus natural teeth in periodontal maintenance patients	Recker E.N.; Avila-Ortiz G.; Fischer C.L.; Pagan-Rivera K.; Brogden K.A.; Dawson D.V.; Elangovan S.	2015
The effect of the local use of chlorhexidine in surgical treatment of experimental peri-implantitis in dogs	Carcuac O.; Abrahamsson I.; Charalampakis G.; Berglundh T.	2015
Long-term evaluation of periodontal parameters and implant outcomes in periodontally compromised patients: a systematic review	Zangrando M.S.; Damante C.A.; Sant'Ana A.C.; Rubo de Rezende M.L.; Greghi S.L.; Chambrone L.	2015
Clinical evaluation of 262 osseointegrated implants placed in sites grafted with calcium phosphosilicate putty: a retrospective study	Babbush C.A.; Kanawati A.	2015
Peri-implant conditions around sintered porous-surfaced (SPS) implants. A 36-month prospective cohort study	Malchiodi L.; Ghensi P.; Cucchi A.; Pieroni S.; Bertossi D.	2015
Water jet with adjunct chlorhexidine gel for nonsurgical treatment of peri-implantitis	Levin L.; Frankenthal S.; Joseph L.; Rozitsky D.; Levi G.; Machtei E.E.	2015
Periodontopathogen and Epstein-Barr virus-associated periapical periodontitis may be the source of retrograde infectious peri-implantitis	Verdugo F.; Castillo A.; Simonian K.; Castillo F.; Farez-Vidal E.; D'Addona A.	2015
Monitoring bone morphogenetic protein-2 and -7, soluble receptor activator of nuclear factor-κB ligand and osteoprotegerin levels in the	Dolanmaz D.; Saglam M.; Inan O.; Dundar N.; Alniacık G.; Gursoy Trak B.; Kocak E.; Hakki S.S.	2015

peri-implant sulcular fluid during the osseointegration of hydrophilic-modified sandblasted acid-etched and sandblasted acid-etched surface dental implants		
LASER sintered one-piece early-loaded dental implants for mandibular premolars replacement	El-Gammal M.; Ghoneem N.; Tawfik H.; Madina M.A.; Maria O.M.	2015
Immediate loading of mandibular overdentures supported by one-piece, direct metal laser sintering mini-implants: a short-term prospective clinical study	Mangano F.G.; Caprioglio A.; Levrini L.; Farronato D.; Zecca P.A.; Mangano C.	2015
Adherent endotoxin on dental implant surfaces: a reappraisal	Morra M.; Cassinelli C.; Bollati D.; Cascardo G.; Bellanda M.	2015
Short implants in maxillary and mandibular rehabilitations: interim results (6 to 42 months) of a prospective study	Taschieri S.; Corbella S.; Molinari R.; Saita M.; Del Fabbro M.	2015
Dimensional changes in soft tissues around dental implants following free gingival grafting: an experimental study in dogs	Bengazi F.; Lang N.P.; Caroprese M.; Urbizo Velez J.; Favero V.; Botticelli D.	2015
Implant survival after preparation of the implant site using a single bur: a case series	Bettach R.; Taschieri S.; Boukhris G.; Del Fabbro M.	2015
A review of oral surgery-related papers published in the British Journal of Oral and Maxillofacial Surgery during 2011 and 2012	Tahim A.S.; Goodson A.M.C.; Payne K.F.B.; Brennan P.A.	2015
Clinical and radiographic evaluation of narrow- vs. regular-diameter dental implants: a 3-year follow-up. A retrospective study	Zweers J.; van Doornik A.; Hogendorf E.A.; Quirynen M.; Van der Weijden G.A.	2015
Comparative analysis of dental implant treatment outcomes following mandibular reconstruction with double-barrel fibula bone grafting or vertical distraction osteogenesis fibula: a retrospective study	Wang F.; Huang W.; Zhang C.; Sun J.; Kaigler D.; Wu Y.	2015
Marginal bone response of implants with platform switching and non-platform switching abutments in posterior healed sites: a 1-year prospective study	Wang Y.-C.; Kan J.Y.; Rungcharassaeng K.; Roe P.; Lozada J.L.	2015
Radiographic comparison of periimplant bone resorption and assessment of survival rates of 2 implant systems: a 10-year prospective multicenter study	Perrotti V.; Ravera L.; Ricci L.; Doi K.; Piattelli A.; Shibli J.; Iezzi G.	2015
Long-term results after subcrestal or crestal placement of delayed loaded implants	Romanos G.E.; Aydin E.; Gaertner K.; Nentwig G.-H.	2015
Titanium particles and mechanical instability of implants induce osteoclast differentiation through indistinguishable inflammatory pathways	Amirhosseini M.; Andersson G.; Aspenberg P.; Fahlgren A.	2015
Ridge expansion alone or in combination with guided bone regeneration to facilitate implant placement in narrow alveolar ridges: a retrospective study	Tang Y.-L.; Yuan J.; Song Y.-L.; Ma W.; Chao X.; Li D.-H.	2015
Dramatic osteonecrosis of the jaw associated with oral bisphosphonates, periodontitis, and dental implant removal	Fernández Ayora A.; Herion F.; Rompen E.; Reginster J.Y.; Magremanne M.; Lambert F.	2015
Evaluation of human periimplant soft tissues around nonsubmerged machined standard and platform-switched abutments	Collins J.R.; Berg R.W.; Rodríguez M.; Rodríguez I.; Coelho P.G.; Tovar N.	2015
Double crown-retained maxillary overdentures: 5-year follow-up	Frisch E.; Ziebolz D.; Ratka-Krüger P.; Rinke S.	2015
Minimally invasive treatment of soft tissue deficiency around an implant-supported restoration in the esthetic zone: modified VISTA technique case report	Lee C.-T.; Hamalian T.; Schulze-Späte U.	2015
Effects of peri-implant plastic surgery and hyperbaric oxygen therapy on mandibular avascular necrosis after implant surgery	Baltacıoğlu E.; Bağış B.; Arslan A.; Kehribar M.A.; Yuva P.; Aydın G.; Yılmaz M.; Şenel F.Ç	2015
Assessment of functional improvement with implant-supported prosthetic rehabilitation after mandibular reconstruction with a microvascular free fibula flap: A study of 25 patients	Bodard A.-G.; Salino S.; Desoutter A.; Deneuve S.	2015
Mixed donor chimerism after allogeneic transplant in patients with wiskott-aldrich syndrome	Ngwube A.; Forbes L.; Krance R.A.; Brenner M.; Heslop H.E.; Leung K.; Hanson I.C.; Sasa G.; Rosenblatt H.; Leen A.M.; Shearer W.; Naik S.; Ahmed N.M.; Gottschalk S.; Allen C.; Martinez C.	2015
Financial analysis of revision knee surgery based on NHS tariffs and hospital costs Does it pay to provide a revision service?	Kallala R.F.; Ibrahim M.S.; Sarmah S.; Haddad F.S.; Vanhegan I.S.	2015
Staged versus multi-vessel revascularization in primary percutaneous coronary intervention for acute ST-segment elevation myocardial infarction based on peri-procedural success rate: A pilot study	Saad M.; Rashed A.; El-Haddad M.A.; El-Kilany W.; Wadee B.; Nassar A.	2015
Abnormal uterine bleeding as the first manifestation of hemostasis system abnormalities	Saidova R.A.; Makatsariya A.D.	2015
Peri-implant diseases: Diagnosis and treatment	Frau Sancho S.; Espadas Dominguez M.; De Clemente-Rodríguez De Rivera E.; Teixidor Olmo I.; Arranz Obispo C.; López López J.	2015
Peri-implant infections of oral biofilm etiology	Belibasakis G.N.; Charalampakis G.; Bostanci N.; Stadlinger B.	2015
Nonsurgical periodontal management of iatrogenic peri-implantitis: A clinical report	Roncati M.; Lauritano D.; Tagliabue A.; Tettamanti L.	2015
The effect of keratinized mucosa width on peri-implant outcome under supportive postimplant therapy	Frisch E.; Ziebolz D.; Vach K.; Ratka-Krüger P.	2015
Group D. Initiator paper. Implants--peri-implant (hard and soft tissue) interactions in health and disease: the impact of explosion of implant manufacturers	Ivanovski S.	2015
Role of reactive oxygen species and advanced glycation end products in the malfunctioning of dental implants	Guo M.; Liu L.; Zhang J.; Liu M.	2015
Implant-based rehabilitation in oncology patients can be performed with high long-term success	Hessling S.A.; Wehrhan F.; Schmitt C.M.; Weber M.; Schlittenbauer T.; Scheer M.	2015

Predictors of excess cement and tissue response to fixed implant-supported dentures after cementation	Korsch M.; Robra B.-P.; Walther W.	2015
Practice-based clinical evaluation of zirconia abutments for anterior single-tooth restorations	Rinke S.; Lattke A.; Eickholz P.; Kramer K.; Ziebolz D.	2015
Clinical study of 19 cases resulting in dental implant removal	Suzuki H.; Takahashi D.; Matsuo K.; Kimoto A.; Matsumoto K.; Hasegawa T.; Akashi M.; Komori T.	2015
Erythritol-Enriched Air-Polishing Powder for the Surgical Treatment of Peri-Implantitis	Taschieri S.; Weinstein R.; Del Fabbro M.; Corbella S.	2015
Foreign bodies associated with peri-implantitis human biopsies	Wilson T.G.; Valderrama P.; Burbano M.; Blansett J.; Levine R.; Kessler H.; Rodrigues D.C.	2015
Palatal positioned implants in severely atrophic maxillae versus conventional implants to support fixed full-arch prostheses: Controlled retrospective study with 5 years of follow-up	Candel-Marti E.; Peñarrocha-Oltra D.; Bagán L.; Peñarrocha-Diago M.; Peñarrocha-Diago M.	2015
Ligature-induced peri-implant infection in crestal and subcrestal implants: A clinical and radiographic study in dogs	Huang B.; Piao M.; Zhang L.; Wang X.; Xu L.; Zhu W.; Meng H.	2015
Low-level laser therapy improves peri-implant bone formation: Resonance frequency, electron microscopy, and stereology findings in a rabbit model	Gomes F.V.; Mayer L.; Massotti F.P.; Baraldi C.E.; Ponzoni D.; Webber J.B.B.; De Oliveira M.G.	2015
Rehabilitation of irradiated patients with chemically modified and conventional SLA implants: five-year follow-up	Nack C.; Raguse J.-D.; Stricker A.; Nelson K.; Nahles S.	2015
Dental implant status of patients receiving long-term nursing care in Japan	Kimura T.; Wada M.; Suganami T.; Miwa S.; Hagiwara Y.; Maeda Y.	2015
A multifactorial analysis to identify predictors of implant failure and peri-implant bone loss	Vervaeke S.; Collaert B.; Cosyn J.; Deschepper E.; De Bruyn H.	2015
Application of high resolution synchrotron micro-CT radiation in dental implant osseointegration	Neldam C.A.; Lauridsen T.; Rack A.; Lefolii T.T.; Jørgensen N.R.; Feidenhans'L R.; Pinholt E.M.	2015
Macrophage migration inhibitory factor in lipopolysaccharide-induced peri-implant inflammation of bone marrow mesenchymal stem cells	Xia D.-J.; Pei H.	2015
Clinical evaluation of immediate loading of titanium orthodontic implants	Chopra S.S.; Chakranarayan S.L.C.A.	2015
Cone beam computed tomography evaluation of regenerated buccal bone 5 years after simultaneous implant placement and guided bone regeneration procedures--a randomized, controlled clinical trial	Jung R.E.; Benic G.I.; Scherrer D.; Hämmerle C.H.	2015
Long-term evaluation of ANKYLOS® dental implants, part i: 20-year life table analysis of a longitudinal study of more than 12,500 implants	Krebs M.; Schmenger K.; Neumann K.; Weigl P.; Moser W.; Nentwig G.-H.	2015
Immediately loaded mini dental implants as overdenture retainers: 1-Year cohort study of implant stability and peri-implant marginal bone level	Šćepanović M.; Todorović A.; Marković A.; Patrnogić V.; Miličić B.; Moufti A.M.; Mišić T.	2015
Failures in dental implants: A literature review	Ramesh Reddy J.; Muthu Kumar B.; Ahila S.C.; Suganya S.	2015
Long-term retrospective evaluation of the peri-implant bone level in onlay grafted patients with iliac bone from the anterior superior iliac crest	Fretwurst T.; Nack C.; Al-Ghrairi M.; Raguse J.D.; Stricker A.; Schmelzeisen R.; Nelson K.; Nahles S.	2015
The influence of the periodontal biotype on peri-implant tissues around immediate implants with and without xenografts. Clinical and micro-computerized tomographic study in small Beagle dogs	Maia L.P.; Reino D.M.; Muglia V.A.; de Souza S.L.; Palioto D.B.; Novaes A.B.	2015
Etiology and clinical characteristics of symptomatic unilateral maxillary sinusitis: A review of 174 cases	Troeltzsch M.; Pache C.; Troeltzsch M.; Kaeppler G.; Ehrenfeld M.; Otto S.; Probst F.	2015
Long-term results of mandibular reconstruction of continuity defects with fibula free flap and implant-borne dental rehabilitation	Fang W.; Liu Y.-P.; Ma Q.; Liu B.-L.; Zhao Y.	2015
Osseointegrated implants placed at supracrestal level may harbour higher counts of A. gerencseriae and S. constellatus - A randomized, controlled pilot study	de Moraes Rego M.R.; Torres M.F.; Santiago L.C.; Lira-Junior R.; Lourenço E.J.; de Moraes Telles D.; Figueredo C.M.	2015
Fresh frozen versus autogenous iliac bone for the rehabilitation of the extremely atrophic maxilla with onlay grafts and endosseous implants: preliminary results of a prospective comparative study	Chiapasco M.; Di Martino G.; Anello T.; Zaniboni M.; Romeo E.	2015
High Insertion Torque and Peri-Implant Bone Loss: Is There a Relationship?	Bidgoli M.; Soheilifar S.; Faradmal J.; Soheilifar S.	2015
Tissue-engineered oral mucosa grafts for intraoral lining reconstruction of the maxilla and mandible with a fibula flap	Sieira Gil R.; Pagés C.M.; Díez E.G.; Llames S.; Fuertes A.F.; Vilagran J.L.	2015
Complications with computer-aided designed/computer-assisted manufactured titanium and soldered gold bars for mandibular implant-overdentures: short-term observations	Katsoulis J.; Wälchli J.; Kobel S.; Gholami H.; Mericske-Stern R.	2015
Cement-associated signs of inflammation: retrospective analysis of the effect of excess cement on peri-implant tissue	Korsch M.; Robra B.-P.; Walther W.	2015
Plasma deposited silicon oxide films for controlled permeation of copper as antimicrobial agent	Lehmann A.; Rupf S.; Schubert A.; Zylla I.-M.; Seifert H.J.; Schindler A.; Arnold T.	2015
A prospective, randomized, controlled study using OsseoSpeed™ implants placed in maxillary fresh extraction socket: soft tissues response	Cecchinato D.; Lops D.; Salvi G.E.; Sanz M.	2015
Influence of prosthetic parameters on peri-implant bone resorption in the first year of loading: a multi-factorial analysis	Hof M.; Pommer B.; Zukic N.; Vasak C.; Lorenzoni M.; Zechner W.	2015
Influence of immediate loading on healing of implants installed with different insertion torques--an experimental study in dogs	Rea M.; Botticelli D.; Ricci S.; Soldini C.; González G.G.; Lang N.P.	2015
Application of concentrated growth factor in maxillary sinus lift: Variation in peri-implant bone levels	Huang N.; Wu F.; Li P.; Liu Z.-P.; Lin J.; Tang Y.-C.; Wang Y.-Q.	2015
Hard and soft tissue changes around implants installed in regular-sized and reduced alveolar bony ridges. An experimental study in dogs	Baffone G.; Lang N.P.; Pantani F.; Favero G.; Ferri M.; Botticelli D.	2015

Influence of oral bisphosphonates in patients with dental implants	Jalil-Abumalham D.; Hurtado Celotti D.; Buesa Bárez J.M.; Rubio Alonso L.; Martínez-González J.M.	2015
Combined local and systemic antibiotic treatment is effective against experimental Staphylococcus aureus peri-implant biofilm infection	Van Der Horst A.S.; Medda S.; Ledbetter E.; Liu A.; Weinhold P.; Del Gaizo D.J.; Dahners L.	2015
Patients With Transmandibular Implants Are at Risk for Severe Complications When Becoming Frail	Hoeksema A.R.; Raghoebar G.M.; Vissink A.; Visser A.	2015
Survival rate of osseointegrated implants in atrophic maxillae grafted with calvarial bone: A retrospective study	Quiles J.C.; Souza F.A.; Bassi A.P.F.; Garcia I.R.; França M.T.; Carvalho P.S.P.	2015
Reverse oblique and transverse intertrochanteric femoral fractures treated with the long cephalomedullary nail	Irgit K.; Richard R.D.; Beebe M.J.; Bowen T.R.; Kubiak E.; Horwitz D.S.	2015
Assessment of the efficacy of a novel antibiotic coating in decreasing periprosthetic infection using an established in vivo mouse model	Loftin A.H.; Zhu S.; Stavrakis A.I.; Manegold J.E.; Richman S.A.; Segura T.; Bernthal N.M.	2015
Histomorphological analysis of peri-implant bone tissue of an implant in use for 10 years: A case report	Fuentes Fernández R.; Valdivia I.; Borie E.; Beltran V.; Bucchi C.	2015
Long-term peri-implant bone level changes of non-vascularized fibula bone grafted edentulous patients	Duttenhoefer F.; Nack C.; Doll C.; Raguse J.-D.; Hell B.; Stricker A.; Nelson K.; Nahles S.	2015
Peri-prosthetic infection - An algorithmic approach to diagnosis and management	Ailabouni R.; Jennings A.; Hooper G.	2015
Peri-implant bone loss of dental implants with platform-switching design after 5 years of loading: a cross-sectional study	Joda T.; Michelaki I.; Heydecke G.	2015
The CD200 tolerance-signaling molecule and its receptor, CD200R1, are expressed in human placental villus trophoblast and in peri-implant decidua by 5 weeks' gestation	Clark D.A.; Arredondo J.L.; Dhesy-Thind S.	2015
Sclerostin antibody treatment improves implant fixation in a model of severe osteoporosis	Virdi A.S.; Irish J.; Sena K.; Liu M.; Ke H.Z.; McNulty M.A.; Sumner D.R.	2015
Fate of modular cardiac tissue constructs in a syngeneic rat model	Leung B.M.; Miyagi Y.; Li R.-K.; Sefton M.V.	2015
Prospective randomised evaluation of a collagen/thrombin and autologous platelet haemostatic agent during cementless total hip arthroplasty	Joyce D.M.; Klika A.K.; Mutnal A.; Krebs V.; Molloy R.; Knothe U.; Barsoum W.K.	2015
Clinical outcomes of total hip arthroplasty for fractured neck of femur in patients over 75 years	Travis E.C.; Tan R.S.; Funaki P.; McChesney S.J.; Patel S.C.; Brogan K.	2015
(viii) Peri-prosthetic fractures of the hip: A new approach	Das R.; Haddad F.S.	2015
Successful Use of Pre and Post-operative ECMO for Pulmonary Endarterectomy, Mitral Valve Replacement and Myomectomy in a Patient with Chronic Thromboembolic Pulmonary Hypertension and Hypertrophic Cardiomyopathy	Williams L.; Kermeen F.; Mullany D.; Thomson B.	2015
Impact of radiation history, gender and age on bone quality in sites for orthodontic skeletal anchorage device placement	Konermann A.; Appel T.; Wenghoefer M.; Sirokay S.; Dirk C.; Jäger A.; Götz W.	2015
Incorporation of raloxifene-impregnated allograft around orthopedic titanium implants impairs early fixation but improves new bone formation: A 4-week study in 12 dogs	Hermansen L.L.; Sørensen M.; Barckman J.; Bechtold J.E.; Søballe K.; Baas J.	2015
Subjective and objective outcomes 5 years after mid-urethral sling	St Louis S.; Gurshumov E.; Salamon C.; Mushonga N.C.; Pagnillo J.; Culligan P.	2015
Risk of pocket hematoma in patients on chronic anticoagulation with warfarin undergoing electrophysiological device implantation: A comparison of different peri-operative management strategies	Proietti R.; Porto I.; Levi M.; Leo A.; Russo V.; Kalfon E.; Biondi-Zoccai G.; Roux J.-F.; Birnie D.H.; Essebag V.	2015
Potential Savings in Prescription Drug Costs for Hypertension, Hyperlipidemia, and Diabetes Mellitus by Equivalent Drug Substitution in Austria: A Nationwide Cohort Study	Heinze G.; Hronsky M.; Reichardt B.; Baumgärtel C.; Müllner M.; Bucsics A.; Winkelmayer W.C.	2014
The XB130/Tks5 adaptor protein interaction regulates Src-mediated signal transduction	Moodley S.; Liu M.	2014
Balloon angioplasty, with and without stenting, versus medical therapy for hypertensive patients with renal artery stenosis	Jenks S.; Yeoh S.E.; Conway B.R.	2014
Incidence of peri-implant mucositis and peri-implantitis in edentulous patients with an implant-retained mandibular overdenture during a 10-year follow-up period	Meijer H.J.; Raghoebar G.M.; de Waal Y.C.; Vissink A.	2014
Early, delayed, or late single implant placement: 10-year results from a randomized controlled clinical trial	Schropp L.; Wenzel A.; Stavropoulos A.	2014
Research advances in peri-implantitis relevant factors and therapy	Shen Y.; Zou D.; Wu Y.	2014
Immediately loaded platform-switched implants in the anterior mandible with fixed prostheses: a randomized, split-mouth, masked prospective trial	Romanos G.E.; Malmstrom H.; Feng C.; Ercoli C.; Caton J.	2014
Three-year results from a randomised controlled trial comparing prostheses supported by 5-mm long implants or by longer implants in augmented bone in posterior atrophic edentulous jaws	Esposito M.; Pistilli R.; Barausse C.; Felice P.	2014
Short implants versus longer implants in vertically augmented posterior mandibles: a randomised controlled trial with 5-year after loading follow-up	Felice P.; Cannizzaro G.; Barausse C.; Pistilli R.; Esposito M.	2014
Synthesis of new antibacterial composite coating for titanium based on highly ordered nanoporous silica and silver nanoparticles	Massa M.A.; Covarrubias C.; Bittner M.; Fuentevilla I.A.; Capetillo P.; Von Marttens A.; Carvajal J.C.	2014
Implant treatment planning: endodontic considerations	Simonian K.; Frydman A.; Verdugo F.; Roges R.; Kar K.	2014
A comparison of two dental implant systems in partially edentulous patients: 1-year post-loading results from a pragmatic multicentre randomised controlled trial	Felice P.; Barausse C.; Blasone R.; Favaretto G.; Stacchi C.; Calvo M.; Marin C.; Buti J.; Esposito M.	2014

Effects of immediate and delayed loading on peri-implant trabecular structures: a cone beam CT evaluation	Huang Y.; Van Dessel J.; Liang X.; Depypere M.; Zhong W.; Ma G.; Lambrichts I.; Maes F.; Jacobs R.	2014
Crestal approach for maxillary sinus augmentation in patients with ≤ 4 mm of residual alveolar bone	Gonzalez S.; Tuan M.-C.; Ahn K.M.; Nowzari H.	2014
The influence of platform switching on clinical, laboratory, and image-based measures: a prospective clinical study	Dursun E.; Tulunoglu I.; Ozbek S.M.; Uysal S.; Akalın F.A.; Kilinc K.; Karabulut E.; Tözüm T.F.	2014
An in vitro study of hard tissue formation around implants with culture of HMS0014 cells in cellmatrix type I-A collagen gel 3D scaffold	Iwai Y.; Morishita A.; Nakatsuka M.; Ueno K.; Kumabe S.	2014
Melatonin as a candidate therapeutic drug for protecting bone cells from chlorhexidine-induced damage	Proksch S.; Strobel S.L.; Vach K.; Abouassi T.; Tomakidi P.; Ratka-Krüger P.; Hellwig E.	2014
Piezoelectric vs. conventional drilling in implant site preparation: pilot controlled randomized clinical trial with crossover design	Canullo L.; Peñarrocha D.; Peñarrocha M.; Rocio A.-G.; Penarrocha-Diago M.	2014
Novel Broad-Spectrum Antimicrobial Photoinactivation of In Situ Oral Biofilms by Visible Light plus Water-Filtered Infrared A	Karygianni L.; Ruf S.; Follo M.; Hellwig E.; Bucher M.; Anderson A.C.; Vach K.; Al-Ahmad A.	2014
Revision Surgery Occurs Frequently After Percutaneous Fixation of Stable Femoral Neck Fractures in Elderly Patients	Kain M.S.; Marcantonio A.J.; Iorio R.	2014
Higher levels of plasminogen-activator inhibitor 1 are associated with progression of coronary artery calcification in middle-aged women: The study of women's health across the nation (SWAN) heart study	Wang N.C.; Matthews K.A.; Barinas-Mitchell E.J.; Chang C.-C.H.; El Khoudary S.R.	2014
Incidence and predictors of peri-procedure complications with transvenous lead extractions in the real world: Data from NCDR ICD registry	Sood N.; Martin D.; Clancy J.; Curtis J.; Parzynski C.S.; Lampert R.	2014
Heart fat, but not peri-aortic fat depots were significantly associated with presence and severity of coronary calcification in women at midlife: The study of women's health across the nation (SWAN) ectopic cardiovascular fat ancillary study	El Khoudary S.R.; Shields K.; Budoff M.; Barinas-Mitchell E.; Janssen I.; Everson-Rose S.; Matthews K.A.	2014
Biofunctionalization of microgroove titanium surfaces with an antimicrobial peptide to enhance their bactericidal activity and cytocompatibility	Zhou L.; Lai Y.; Huang W.; Huang S.; Xu Z.; Chen J.; Wu D.	2015
Estimation of bone loss biomarkers as a diagnostic tool for peri-implantitis	Rakic M.; Struillou X.; Petkovic-Curcin A.; Matic S.; Canullo L.; Sanz M.; Vojvodic D.	2014
Prevalence of peri-implantitis in patients with implant-supported fixed prostheses	Schuldt Filho G.; Dalago H.R.; Oliveira de Souza J.G.; Stanley K.; Jovanovic S.; Bianchini M.A.	2014
Surgical treatment of peri-implantitis using a bone substitute with or without a resorbable membrane: a 5-year follow-up	Roos-Jansåker A.-M.; Persson G.R.; Lindahl C.; Renvert S.	2014
Follow-up study of implants with turned or oxidized surfaces placed after sinus augmentation	Jungner M.; Legrell P.E.; Lundgren S.	2014
Biologic width adjacent to loaded implants with machined and rough collars in the dog	Cochran D.L.; Obrecht M.; Weber K.; Dard M.; Bosshardt D.; Higginbottom F.L.; Wilson T.G.; Jones A.A.	2014
kgp, rgpA, and rgpB DNA vaccines induce antibody responses in experimental peri-implantitis	Guo M.; Wang Z.; Fan X.; Bian Y.; Wang T.; Zhu L.; Lan J.	2014
The impact of subcrestal placement on short locking-taper implants placed in posterior maxilla and mandible: A retrospective evaluation on hard and soft tissues stability after 2 years of loading	Lombardo G.; Corrocher G.; Pighi J.; Faccioni F.; Rovera A.; Marincola M.; Nocini P.F.	2014
Antibiotic prophylaxis patterns of Finnish dentists performing dental implant surgery	Pyysalo M.; Helminen M.; Antalainen A.-K.; Sándor G.K.; Wolff J.	2014
Clinical and radiographic evaluation of early loaded maxillary anterior single-tooth bone-level implants	Vanlıoğlu B.A.; Kahramanoğlu E.; Ozkan Y.; Kulak-Özkan Y.	2014
Impact of implant-abutment connection, positioning of the machined collar/microgap, and platform switching on crestal bone level changes. Camlog Foundation Consensus Report	Schwarz F.; Alcoforado G.; Nelson K.; Schaer A.; Taylor T.; Beuer F.; Strietzel F.P.	2014
Long-term stability of soft tissues following alveolar ridge preservation: 10-year results of a prospective study around nonsubmerged implants	Roccuzzo M.; Gaudioso L.; Bunino M.; Dalmasso P.	2014
Influence of two different machined-collar heights on crestal bone loss	Herrero-Climent M.; Romero Ruiz M.M.; Díaz-Castro C.M.; Bullón P.; Ríos-Santos J.V.	2014
A retrospective radiographic study on the effect of natural tooth-implant proximity and an introduction to the concept of a bone-loading platform switch	Urdaneta R.A.; Seemann R.; Dragan I.-F.; Lubelski W.; Leary J.; Chuang S.-K.	2014
Role of photofunctionalization in mitigating impaired osseointegration associated with type 2 diabetes in rats	Sugita Y.; Honda Y.; Kato I.; Kubo K.; Maeda H.; Ogawa T.	2014
Effect of cantilevers for implant-supported prostheses on marginal bone loss and prosthetic complications: systematic review and meta-analysis	Torrecillas-Martínez L.; Monje A.; Lin G.-H.; Suarez F.; Ortega-Oller I.; Galindo-Moreno P.; Wang H.-L.	2014
Short-term retrospective case series of implant-assisted removable partial dentures with locator abutments	Ortiz-Puigpelat O.; Gargallo-Albiol J.; Hernández-Alfaro F.; Cabratosa-Termes J.	2014
An observational study on epidemiology and risk factors of peri-prosthetic joint infection	Toro G.; Rizzo V.; Lepore L.; Gison M.; Viafora G.; Ascione T.; Toro A.	2014
Implementation of international chart review studies: An assessment of ethics and regulatory considerations	Jean-Mary J.; Stein D.; Yeomans K.; Payne K.A.	2014
Management of peri-implant mucositis and peri-implantitis	Figuero E.; Graziani F.; Sanz I.; Herrera D.; Sanz M.	2014
Generalized aggressive periodontitis as a risk factor for dental implant failure: a systematic review and meta-analysis	Monje A.; Alcoforado G.; Padial-Molina M.; Suarez F.; Lin G.-H.; Wang H.-L.	2014
Treatment outcome following use of the erbium, chromium:yttrium, scandium, gallium, garnet laser in the non-surgical management of peri-implantitis: a case series	Al-Falaki R.; Cronshaw M.; Hughes F.J.	2014
Repair of peri-implant bone loss after occlusal adjustment: a case report	Merin R.L.	2014

Supportive post-implant therapy: patient compliance rates and impacting factors: 3-year follow-up	Frisch E.; Ziebolz D.; Vach K.; Ratka-Krüger P.	2014
The effect of loading on peri-implant bone: a critical review of the literature	Duyck J.; Vandamme K.	2014
Long-term results of a three arms prospective cohort study on implants in periodontally compromised patients: 10-year data around sandblasted and acid-etched (SLA) surface	Roccuzzo M.; Bonino L.; Dalmasso P.; Aglietta M.	2014
Peri-implantitis. Part 3: current modes of management	Alani A.; Bishop K.	2014
Effects of antibiofilm compounds on the cellular and bacterial colonization of polimeric surfaces: A step towards biofunctionalization of implantable devices	Neto A.R.L.P.; Piaia L.; Paes C.Q.; Aragones A.; De Souza Magini R.; Porto L.M.; Pereira U.A.; De Almeida Barbosa L.C.; De Lima Pimenta A.	2014
Influence of surgical and prosthetic techniques on marginal bone loss around titanium implants. Part I: immediate loading in fresh extraction sockets	Berberi A.N.; Tehini G.E.; Noujeim Z.F.; Khairallah A.A.; Abousehlib M.N.; Salameh Z.A.	2014
Immediate implants at fresh extraction sockets: from myth to reality	Vignoletti F.; Sanz M.	2014
Etiology and treatment of periapical lesions around dental implants	Temmerman A.; Lefever D.; Teughels W.; Balshi T.J.; Balshi S.F.; Quirynen M.	2014
A prospective multicenter study of immediate function of 1-piece implants: a 3-year follow-up report	Sato J.; Watanabe G.; Ando M.; Shimoo Y.; Shizukuda K.; Kimura T.; Kobayashi M.	2014
Periimplant bone-level reduction in relation to hydroxyapatite-coated dental implants that act as mandibular overdenture retainers: results at 6 to 10 years	Leventi E.; Malden N.J.; Lopes V.R.	2014
Implant therapy: 40 years of experience	Quirynen M.; Herrera D.; Teughels W.; Sanz M.	2014
A systematic review on marginal bone loss around short dental implants (<10 mm) for implant-supported fixed prostheses	Monje A.; Suarez F.; Galindo-Moreno P.; García-Nogales A.; Fu J.-H.; Wang H.-L.	2014
Generation of an rhBMP-2-loaded beta-tricalcium phosphate/hydrogel composite and evaluation of its efficacy on peri-implant bone formation	Lee J.H.; Ryu M.Y.; Baek H.-R.; Seo J.-H.; Lee K.-M.; Lee J.-H.	2014
The platform switching concept revisited. An experimental study in dogs	Bressan E.; Lang N.P.; Corazza B.; Ricci S.; Almagro Urrutia Z.; Botticelli D.	2014
Impact of insertion torque and implant neck design on peri-implant bone level: a randomized split-mouth trial	Hof M.; Pommer B.; Strbac G.D.; Vasak C.; Agis H.; Zechner W.	2014
Implant over implant: an alternative method for solving malpositioned osseointegrated implants at the sinus floor	Man Y.; Wang T.; Mo A.; Qu Y.	2014
Rare case of leptomeningeal carcinomatosis without evidence of brain metastasis presenting as first sign of recurrent NSCLC	Lowe D.; Sinha S.; Kahlon A.	2014
A unique conjoined orbital and maxillary removable “Bottle opener” prosthesis for hemi-maxillectomy defect with orbital exenteration	Kusanale A.; Silk H.; Aldridge T.; Ilankovan V.	2014
Reducing joint failure in total tempormandibular joint replacement: A survey of UK surgeons and preliminary data for national guidelines	Richardson S.; Saeed N.	2014
The value of myocardial perfusion imaging in predicting cardiac events and hospital length of stay in abdominal aortic aneurysm repair	Ratcliffe Law T.; Fludder V.; Dizdarevic S.; Singh N.; Yusuf S.; Thorburn P.	2014
Haemodynamic performance and early safety after rapid deployment aortic valve replacement: Real-world outcomes of 512 patients	Laufer G.; Kocher A.; Cassese M.; Alamanni F.; Holzinger C.; Young C.P.; Duhay F.G.; Glauber M.	2014
Can intra-vesical mitomycin-c hyperthermia challenge radical cystectomy as standard of care in the management of high-risk non-muscle invasive bladder cancer?	Nair R.; Pai A.; Ayres B.; Bailey M.; Perry M.; Issa R.	2014
Procedural alterations in oxidative, and nitrosative stress parametres in patientsa undergoing percutaneous coronary interventions	Gelişgen R.; Erman H.; Ikitimur B.; Çakmak H.A.; Sözer V.; Korkmaz G.; Karadaı B.; Şimşek G.; Öngen Z.; Uzun H.	2014
Analytic morphomics identifies predictors of new-onset diabetes after transplantation	Vaughn-Sandler V.; Cron D.C.; Terjimanian M.; Gala Z.; Wang S.C.; Su G.L.; Volk M.	2014
Bleeding outcomes after non-emergency percutaneous coronary intervention in the elderly	Boganashanmugam V.; Psaltis P.; Rashid H.; Meredith I.; Malaiapan Y.	2014
Obsessive-compulsive symptoms in a population-based birth cohort, and their association with level of functioning	Werbeloff N.; Dohrenwend B.P.; Levav I.; Weiser M.	2014
Costs of peri-procedural complications among patients treated with a self-expanding transcatheter aortic valve prosthesis: Results from the corevalve us pivotal extreme risk study	Baron S.J.; Arnold S.V.; Reynolds M.R.; Lei Y.; Magnuson E.; Byrne T.J.; Bajwa T.; O'Hair D.; Adams D.; Popma J.; Cohen D.	2014
Evaluation of protein markers of oral cavity biological fluids in immediate dental implantation	Bazikyan E.; Vavilova T.; Savich O.; Labis V.	2014
Inter-rater agreement in the diagnosis of mucositis and peri-implantitis	Merli M.; Bernardelli F.; Giulianelli E.; Toselli I.; Moscatelli M.; Pagliaro U.; Nieri M.	2014
Does the number of implants have any relation with peri-implant disease?	Passoni B.B.; Dalago H.R.; Schuldt Filho G.; Oliveira de Souza J.G.; Benfatti C.A.; Magini Rd.e S.; Bianchini M.A.	2014
Long-term follow-up of simultaneous guided bone regeneration using native and cross-linked collagen membranes over 6 years	Schwarz F.; Hegewald A.; Sahm N.; Becker J.	2014
Dental implants: A good treatment option in patients with Sjögren's syndrome	Korfage A.; Raghoebar G.M.; Arends S.; Meiners P.M.; Visser A.; Kroese F.G.M.; Bootsma H.; Vissink A.	2014
Influence of crown/implant ratio on marginal bone loss: a systematic review	Garaicoa-Pazmiño C.; Suárez-López del Amo F.; Monje A.; Catena A.; Ortega-Oller I.; Galindo-Moreno P.; Wang H.-L.	2014
Tissue stability of implants placed in fresh extraction sockets: a 5-year prospective single-cohort study	Covani U.; Canullo L.; Toti P.; Alfonsi F.; Barone A.	2014
Prevention and treatment of periodontal diseases in primary care	Matthews D.C.	2014
Platform switching minimises crestal bone loss around dental implants: truth or myth?	Romanos G.E.; Javed F.	2014

Manage patients in peri-implant health	Hernandez M.	2014
Single crowns in the resorbed posterior maxilla supported by either 6-mm implants or by 11-mm implants combined with sinus floor elevation surgery: a 1-year randomised controlled trial	Guljé F.L.; Raghoebar G.M.; Vissink A.; Meijer H.J.	2014
Immediate loading of implants placed in patients with untreated periodontal disease: a 5-year prospective cohort study	Maló P.; Nobre Md.e A.; Lopes A.; Ferro A.; Gravito I.	2014
Management of the ailing implant: An innovative technique for the treatment of labial gingival recession around dental implants	Le B.T.; Borzabadi-Farahani A.; Nielsen B.	2014
Peri-implantitis. Part 1: Scope of the problem	Alani A.; Kelleher M.; Bishop K.	2014
Clinical outcomes of dental implant therapy in alveolar cleft patients: a systematic review	Wang F.; Wu Y.; Zou D.; Wang G.; Kaigler D.	2014
Strain distribution of immediately loaded implants with fixed prosthesis using the "all-on-four" protocol in the edentulous mandible: Nonlinear three-dimensional finite element analysis	Horita S.; Sugiura T.; Yamamoto K.; Kawakami M.; Murakami K.; Kirita T.	2014
Cone beam computed tomographic evaluation of implants with platform-switched Morse taper connection with the implant-abutment interface at different levels in relation to the alveolar crest	Koutouzis T.; Neiva R.; Nair M.; Nonhoff J.; Lundgren T.	2014
A simplified pocket reduction procedure around a stud attachment without suturing	Lee J.-H.; Sohn D.-S.; Cho J.-H.	2014
Influence of presence or absence of keratinized mucosa on the alveolar bony crest level as it relates to different buccal marginal bone thicknesses. An experimental study in dogs	Bengazi F.; Botticelli D.; Favero V.; Perini A.; Urbizo Velez J.; Lang N.P.	2014
Patient-based and clinical outcomes of implant telescopic attachment-retained mandibular overdentures: a 1-year longitudinal prospective study	Yunus N.; Saub R.; Taiyeb Ali T.B.; Salleh N.M.; Baig M.R.	2014
The effects of off-axial loading on periimplant marginal bone loss in a single implant	Lee D.-W.; Park K.-H.; Moon I.-S.	2014
Computer-guided versus free-hand placement of immediately loaded dental implants: 1-year post-loading results of a multicentre randomised controlled trial	Pozzi A.; Tallarico M.; Marchetti M.; Scarfò B.; Esposito M.	2014
Mandibular osteomyelitis following explantation of a peri-implanter infected blade implant	Ofluoğlu D.; Ergun S.; Olgaç V.; Tanyeri H.	2014
HIFU	Napoli A.	2014
Patency vs. clinical success of revascularisation	Spiliopoulos S.	2014
Dental care providers' decision making regarding maintenance of compromised teeth and implant therapy indication: an analysis of gender and enrollment in teaching positions	Junges R.; Zitzmann N.U.; Walter C.; Rösing C.K.	2014
IR relevant sedation and analgesia techniques	Vari A.	2014
Post-interventional patient management	Cleveland T.J.	2014
High FSH level increase endometrial atrophy through cross-talk with TGF beta signal transduction pathway in post-menopause women	Li J.; Zhang D.; Xu G.; Chen L.; Qian Y.; Liu Y.; Zhou C.; Huang H.	2014
A meta-analysis of non-compliance or planned discontinuation of aspirin and the risk of mortality and non-fatal myocardial infarction in patients with established or at risk of coronary artery disease	Luni F.K.; Riaz H.; Riaz I.B.; Riaz T.; Husnain M.; Khan S.; Cooper C.	2014
Indications for carotid artery stenting-patient triage	Cleveland T.J.	2014
High-frequency loading positively impacts titanium implant osseointegration in impaired bone	Chatterjee M.; Hatori K.; Duyck J.; Sasaki K.; Naert I.; Vandamme K.	2014
First steps in using machine learning on fMRI data to predict intrusive memories of traumatic film footage	Clark I.A.; Niehaus K.E.; Duff E.P.; Di Simplicio M.C.; Clifford G.D.; Smith S.M.; Mackay C.E.; Woolrich M.W.; Holmes E.A.	2014
Collagen-functionalised titanium surfaces for biological sealing of dental implants: Effect of immobilisation process on fibroblasts response	Marín-Pareja N.; Salvagni E.; Guillem-Marti J.; Aparicio C.; Ginebra M.-P.	2014
Comparison of peri-implant crevicular fluid levels of adrenomedullin and human beta defensins 1 and 2 from mandibular implants with different implant stability quotient levels in nonsmoker patients	Ertugrul A.S.; Tekin Y.; Alpaslan N.Z.; Bozoglan A.; Sahin H.; Dikilitas A.	2014
Survival and complication rates of fixed restorations supported by locking-taper implants: a prospective study with 1 to 10 years of follow-up	Mangano F.; Macchi A.; Caprioglio A.; Sammons R.L.; Piattelli A.; Mangano C.	2014
Periodontal stress-Cellular and molecular aspects	Gotz W.	2014
Influence of Implant transmucosal design on early peri-implant tissue responses in beagle dogs	Huh J.-B.; Rheu G.-B.; Kim Y.-S.; Jeong C.-M.; Lee J.-Y.; Shin S.-W.	2014
The extremely resorbed mandible, 10-year results of a randomized controlled trial on 3 treatment strategies	Stellingsma K.; Raghoebar G.M.; Visser A.; Vissink A.; Meijer H.J.	2014
Clinical investigation on axial versus tilted implants for immediate fixed rehabilitation of edentulous arches: preliminary results of a single cohort study	Agnini A.; Agnini A.M.; Romeo D.; Chiesi M.; Pariente L.; Stappert C.F.	2014
Implant treatment choice after extraction of a vertically fractured tooth. A proposal for a clinical classification of bony defects based on a systematic review of literature	Corbella S.; Taschieri S.; Samaranayake L.; Tsesis I.; Nemcovsky C.; Del Fabbro M.	2014
Failed dental implants: diagnosis, removal and survival of reimplantations	Greenstein G.; Cavallaro J.	2014

Impact of immediate loading on early soft tissue healing at two-piece implants placed in fresh extraction sockets: an experimental study in the beagle dog	Mareque S.; Liñares A.; Pérez J.; Muñoz F.; Ramos I.; Blanco J.	2014
Long-term evaluation of immediately loaded implants in the edentulous mandible using fixed bridges and platform shifting	Romanos G.E.; Gaertner K.; Nentwig G.H.	2014
Influence of platform switching on periimplant bone loss: a systematic review and meta-analysis	Herekar M.; Sethi M.; Mulani S.; Fernandes A.; Kulkarni H.	2014
Comparison of functional outcomes with purely laparoscopic sacrocolpopexy and robot-assisted sacrocolpopexy in obese women	Thubert T.; Deffieux X.; Villefranque V.; Panel P.; Lefranc J.; Vaessen C.; Chartier-Kastler E.; Rouprêt M.	2014
Interventions for replacing missing teeth: Different types of dental implants	Esposito M.; Ardebili Y.; Worthington H.V.	2014
Neutrophil-gelatinase associated lipocalin (N-GAL) to assess perioperative acute kidney injury in minimal access donor nephrectomy: A pilot study	Aitken E.; Vesey A.; Glen J.; Steven M.; Clancy M.	2014
Predictors of functional status at discharge after medical intensive care unit admission among patients with lung transplantation	Banga A.; Budev M.; Sasidhar M.	2014
Clinical and radiographic outcomes of a combined resective and regenerative approach in the treatment of peri-implantitis: a prospective case series	Matarasso S.; Iorio Siciliano V.; Aglietta M.; Andreuccetti G.; Salvi G.E.	2014
Mucosal inflammation and incidence of crestal bone loss among implant patients: a 10-year study	Cecchinato D.; Parpaiola A.; Lindhe J.	2014
Prosthetic resolution of peri-implant mucositis: promoting soft-tissue health through proper restorative design	Azar D.E.	2014
Cement-associated peri-implantitis: a retrospective clinical observational study of fixed implant-supported restorations using a methacrylate cement	Korsch M.; Obst U.; Walther W.	2014
Use of Er:YAG laser to decontaminate infected dental implant surface in preparation for reestablishment of bone-to-implant contact	Nevins M.; Nevins M.L.; Yamamoto A.; Yoshino T.; Ono Y.; Wang C.-W.; Kim D.M.	2014
Clinical, microbiological, and salivary biomarker profiles of dental implant patients with type 2 diabetes	Tatarakis N.; Kinney J.S.; Inglehart M.; Braun T.M.; Shelburne C.; Lang N.P.; Giannobile W.V.; Oh T.-J.	2014
Immediate placement of dental implants in the esthetic zone: a systematic review and pooled analysis	Slagter K.W.; den Hartog L.; Bakker N.A.; Vissink A.; Meijer H.J.; Raghoebar G.M.	2014
Prognosis of implants and abutment teeth under combined tooth-implant-supported and solely implant-supported double-crown-retained removable dental prostheses	Rammelsberg P.; Bernhart G.; Lorenzo Bermejo J.; Schmitter M.; Schwarz S.	2014
Atraumatic extraction, implant placement and immediate provisionalization	Tavarez R.R.; Calixto A.M.; Maia Filho E.M.; Bandeca M.C.; Firoozmand L.M.; Gomes M.G.; Malheiros A.S.	2014
Influence of implant framework and mandibular flexure on the strain distribution on a Kennedy class II mandible restored with a long-span implant fixed restoration: a pilot study	Law C.; Bennani V.; Lyons K.; Swain M.	2014
An innovative multidisciplinary approach to urogynecology clinical care	Schulz J.A.; Ding J.; Flood C.G.; Tyrrell J.; Janes-Kelley S.	2014
Using botulinum a toxin for hyperhidrosis as an adjunct treatment in wound care	Punj V.; Ota D.; Flavin K.	2014
Mid-and long term efficacyand safety of retropubic TVT and TVT-O: Analysis of the literature	Tommaselli G.A.; Fabozzi A.; Formisano C.; Di Carlo C.; Nappi C.	2014
Incidence and predictors of permanent pacemaker implantation after valve surgery-A single centre experience	Ayub B.; Nawaz Y.; Saif H.; Guthier J.; Weiss M.; Phillips T.; Wu J.; Singer R.; Freudenberger R.; Malacoff R.	2014
Rotating titanium brush for plaque removal from rough titanium surfaces--an in vitro study	John G.; Becker J.; Schwarz F.	2014
Co-development of pyogenic granuloma and capillary hemangioma on the alveolar ridge associated with a dental implant: A case report	Kang Y.-H.; Byun J.-H.; Choi M.-J.; Lee J.-S.; Jang J.-H.; Kim Y.-I.; Park B.-W.	2014
Management of peri-implant disease: a current appraisal	Mishler O.P.; Shiau H.J.	2014
A 10-year retrospective analysis of clinical results of implant-retained/ supported prostheses in full edentulous patients	Hu X.; Luo J.; Li J.; Qiu L.; Di P.; Lin Y.	2014
Adverse events related to dental implants	Pigatto P.D.; Ronchi A.; Brambilla L.; Ferrucci S.M.; Spadari F.; Passoni M.; Bombeccari G.P.; Guzzi G.	2014
Crestal bone changes at teeth and implants in periodontally healthy and periodontally compromised patients. A 10-year comparative case-series study	Rasperini G.; Siciliano V.I.; Cafiero C.; Salvi G.E.; Blasi A.; Aglietta M.	2014
Fractal analysis of ibuprofen effect on experimental dog peri-implantitis	Pârvu A.E.; Tălu S.; Taulescu M.A.; Bota A.; Cătoi F.; Crăciun C.; Alb C.; Pârvu O.; Alb S.F.	2014
Scale	Volchansky A.	2014
Insufficient irrigation induces peri-implant bone resorption: an in vivo histologic analysis in sheep	Trisi P.; Berardini M.; Falco A.; Podaliri Vulpiani M.; Perfetti G.	2014
Associations of clinical characteristics and interval between maintenance visits with peri-implant pathology	de Araújo Nobre M.A.; Maló P.S.; Oliveira S.H.	2014
Surgical treatment of buccal soft tissue recessions around single implants: 1-year results from a prospective pilot study	Roccuzzo M.; Gaudioso L.; Bunino M.; Dalmasso P.	2014
Effect of metformin on periimplant wound healing in a rat model of type 2 diabetes	Inouye K.A.; Bisch F.C.; Elsalanty M.E.; Zakhary I.; Khashaba R.M.; Borke J.L.	2014
Preparation and anti-bacterial tests of silver-modified titanium surface	Liao J.; Fei W.; Guo J.; Li P.	2014
Clinical performance of a highly porous beta-TCP as the grafting material for maxillary sinus augmentation	Bettach R.; Guillaume B.; Taschieri S.; Del Fabbro M.	2014

Comparative analysis of the faults, complications, follow up in patients with wide spectrum for rheumatic diseases attached to total hip replacement	Pavlov V.P.; Makarov S.A.	2014
Immunisation and general anaesthesia-audit of practice in a specialist paediatric hospital	Lonsdale H.; Sivaprakasam J.	2014
NHSN definitions: Worksheets that work	Pruden J.; Tomlin K.; Hessels A.; Young-Ruckriegel C.; Marek M.; De Chirico V.; Desai B.; Mayo J.	2014
Determining Evidence-Based Practice for Site Care: Consensus and Guidelines for CVADs	Broadhurst D.; Moureau N.	2014
Long-term analysis of osseointegrated implants in non-smoker patients with a previous history of periodontitis	Meyle J.; Gersok G.; Boedeker R.-H.; Gonzales J.R.	2014
The case for routine maintenance of dental implants	Wilson T.G.; Valderrama P.; Rodrigues D.B.	2014
OHRQoL, masticatory performance and crestal bone loss with single-implant, magnet-retained mandibular overdentures with conventional and shortened dental arch	Grover M.; Vaidyanathan A.K.; Veeravalli P.T.	2014
Computer-assisted flapless implant surgery in edentulous elderly patients: a 2-year follow up	di Torresanto V.M.; Milinkovic I.; Torsello F.; Cordaro L.	2014
A comparison between 4 and 6 implants in the maxillary posterior region to support an overdenture; 1-year results from a randomized controlled trial	Slot W.; Raghoebar G.M.; Vissink A.; Meijer H.J.	2014
Intraoral use of extraoral implants for oral rehabilitation of a pediatric patient after resection of ewing sarcoma of the mandible and reconstruction with iliac osteocutaneous free flap	Aksu A.E.; Dursun E.; Calis M.; Ersu B.; Safak T.; Tözüm T.F.	2014
Controversies on hormonal contraception in adolescence and bone metabolism	Valdoleiros S.; Gonc¸alves A.; Antunes I.; Martins I.; Palma F.	2014
Crossing of infra-inguinal peripheral arterial chronic total occlusion with viance™ blunt microdissection catheter	Sarode K.; Raffoul J.; Thomas R.; Mohammad A.; Hadidi O.; Baig M.S.; Mody P.; Gigliotti O.S.; Klein A.; Ishti Ali M.; Abu-Fadel M.; Shammas N.W.; Prasad A.; Brilakis E.S.; Banerjee S.	2014
A review of the resuscitation training and equipment in a UK hospital without a Resuscitation Officer	Scott K.; Staber M.	2014
Barriers to early discharge after elective percutaneous coronary intervention: A single-center study	Graziano F.; Wohns D.; Robinett D.; Busman D.; Muthusamy P.; Banga S.	2014
Factors related to peri-implantitis - a retrospective study	Renvert S.; Aghazadeh A.; Hallström H.; Persson G.R.	2014
The influence of initial soft tissue thickness on peri-implant bone remodeling	Vervaeke S.; Dierens M.; Besseler J.; De Bruyn H.	2014
Is keratinized mucosa indispensable to maintain peri-implant health? A systematic review of the literature	Brito C.; Tenenbaum H.C.; Wong B.K.C.; Schmitt C.; Nogueira-Filho G.	2014
Long-term results for maxillary rehabilitation with dental implants after tumor resection	Huang W.; Wu Y.; Zou D.; Zhang Z.; Zhang C.; Sun J.; Xu B.; Zhang Z.	2014
Mini-invasive osteotome sinus floor elevation in partially edentulous atrophic maxilla using reduced length dental implants: interim results of a prospective study	Taschieri S.; Corbella S.; Del Fabbro M.	2014
Efficacy of two antibiotic regimens in the reduction of early dental implant failure: A pilot study	El-Kholey K.E.	2014
Soft tissue wound healing around teeth and dental implants	Sculean A.; Gruber R.; Bosshardt D.D.	2014
Flap approaches in plastic periodontal and implant surgery: critical elements in design and execution	de Sanctis M.; Clementini M.	2014
Predicting peri-operative right heart failure, post-implant survival and timing of mechanical ventricular assistance	Harb M.; Lui R.; Robson D.; Jabbour A.; Kotlyar E.; Keogh A.; Granger E.; Spratt P.; Macdonald P.S.; Jansz P.; Hayward C.S.; Dhital K.	2014
Renal impairment post vad implantation in patients with normal pre-operative renal function	Lui R.; Harb M.; Anthony C.; Robson D.; Sevastos J.; Granger E.; Hayward C.S.; Jabbour A.; Spratt P.M.; Keogh A.M.; Kotlyar E.; Macdonald P.; Janz P.; Dhital K.	2014
Pre-emptive ID specialist consultation reduces MCS drive line infections	Hadi A.; Hellman Y.; Gradus-Pizlo I.; Malik A.; Caccamo M.; Wozniak T.; Hashmi Z.; Wang I.; Pickrell J.	2014
United Kingdom national database study of intravitreal dexamethasone implant (Ozurdex®) for retinal vein occlusion related macular oedema. Patients' pretreatment characteristics and treatment procedure	Sallam A.A.I.; Zarranz-Ventura J.; Mohamed Q.; Johnston R.	2014
Contemporary comparison between retropubic mid urethral sling (MUS) and autologous pubovaginal sling (PVS) for stress urinary incontinence (SUI) after the fda advisory warning	Mock S.; Angelle J.; Reynolds W.S.; Osborn D.J.; Dmochowski R.R.; Gomelsky A.	2014
Anchoring heuristics in diagnosis	Ukaigwe A.; Espana-Schmidt C.	2014
Partial versus radical nephrectomy for T1 renal tumours: An analysis from the British Association of Urological Surgeons Nephrectomy Audit	Hadjipavlou M.; Khan F.; Fowler S.; Keeley F.X.; Sriprasad S.	2014
Peri-implant conditions and their relationship with periodontal conditions in Chinese patients: a cross-sectional study	Wang R.; Zhao W.; Tang Z.H.; Jin L.J.; Cao C.F.	2014
Evaluation of a variable-thread tapered implant in extraction sites with immediate temporization: a 3-year multicenter clinical study	Kolinski M.L.; Cherry J.E.; McAllister B.S.; Parrish K.D.; Pumphrey D.W.; Schroering R.L.	2014
Minimally invasive transcrestal sinus floor elevation with deproteinized bovine bone or β-tricalcium phosphate: a multicenter, double-blind, randomized, controlled clinical trial	Trombelli L.; Franceschetti G.; Stacchi C.; Minenna L.; Riccardi O.; Di Raimondo R.; Rizzi A.; Farina R.	2014
Peri-implant outcome of immediately loaded implants with a full-arch implant fixed denture: a 5-year prospective case series	Martens F.; Vandeweghe S.; Browaeys H.; De Bruyn H.	2014

Is a grooved collar implant design superior to a machined design regarding bone level alteration? An observational pilot study	Bassetti R.; Kaufmann R.; Ebinger A.; Mericske-Stern R.; Enkling N.	2014
Systemic risk factors for peri-implant bone loss: A systematic review and meta-analysis	Clementini M.; Rossetti P.H.O.; Penarrocha D.; Micarelli C.; Bonachela W.C.; Canullo L.	2014
Implant-prosthetic patient: Hygienic maintenance and occlusal control	Montaruli G.; Paparusso M.	2014
Crestal bone reactions to immediate implants placed at different levels in relation to crestal bone. A pilot study in Foxhound dogs	Calvo-Guirado J.L.; Boquete-Castro A.; Negri B.; Delgado Ruiz R.; Gómez-Moreno G.; Iezzi G.	2014
Reconstruction of the edentulous mandible with fresh frozen bone grafts and implants: a 4-year report of a prospective clinical study	Pimentel A.C.; Napimoga M.H.; Manzi M.R.; Sendyk W.R.	2014
The current status of bridging anticoagulation	Patel J.P.; Arya R.	2014
Management of portal and mesenteric vein stenosis after pancreatic coduodenectomy with endovascular stent placement	Ferral H.; Alonzo M.; Talamonti M.	2014
Establishing chronic total occlusion programme in a tertiary australian centre -description of early experience	Shanmugam V.B.; Wong D.; Psaltis P.; Seneviratne S.; Meredith I.; Malaiapan Y.	2014
Monitoring of adverse reactions to DePuy ASR devices in patients with metal-on-metal hip arthroplasty	Rodriguezde La Flor Garcia M.; Ramos Aparicio R.; Puente MartíNez C.P.; HernáNdez Vaquero D.	2014
High Prevalence of Undiagnosed Cryptococcus at HIV Diagnosis Across the CD4 Spectrum in Durban	Drain P. K.; Kleene J. M.; Losina E.; Coleman S. M.; Giddy J.; Ross D.; Katz J.; Walensky R. P.; Freedberg K. A.; Bassett I. V.	2014
Functional and morphological changes following whipple's pancreaticoduodenectomy for periampullary carcinoma	Saluja S.S.; Godhi S.; Mishra P.K.; Patil N.; Ramaswamy D.	2014
Peri-interventional Subarachnoid Hemorrhage During Mechanical Thrombectomy with stent retrievers in Acute Stroke: A Retrospective Case-Control Study	Yilmaz U.; Walter S.; Körner H.; Papanagiotou P.; Roth C.; Simgen A.; Behnke S.; Ragoschke-Schumm A.; Fassbender K.; Reith W.	2015
Peri-implantitis induced by a retained retraction cord	Salem D.; Alshihri A.; Levin L.	2014
Evaluation of clinical outcomes and bone loss around titanium implants with oxidized surface: six-year follow-up results from a prospective case series study	Francetti L.; Azzola F.; Corbella S.; Taschieri S.; Del Fabbro M.	2014
Dental implants in patients with oral lichen planus: a cross-sectional study	López-Jornet P.; Camacho-Alonso F.; Sánchez-Siles M.	2014
Implant periapical lesion: A case-report and medicolegal considerations	Zangari F.	2014
Impact of platform switching on peri-implant bone remodeling around short implants in the posterior region, 1-year results from a split-mouth clinical trial	Telleman G.; Raghoebar G.M.; Vissink A.; Meijer H.J.	2014
Nonsurgical management of inflammatory periimplant disease caused by food impaction: a clinical report	Bidra A.S.	2014
A randomized controlled clinical multicenter trial comparing the clinical and histological performance of a new, modified polylactide-co-glycolide acid membrane to an expanded polytetrafluorethylene membrane in guided bone regeneration procedures	Schneider D.; Weber F.E.; Grunder U.; Andreoni C.; Burkhardt R.; Jung R.E.	2014
Interventions for edentate elders--what is the evidence?	Müller F.	2014
Immediate vs. early loading of SLA implants in the posterior mandible: 5-year results of randomized controlled clinical trial	Kokovic V.; Jung R.; Feloutzis A.; Todorovic V.S.; Jurisic M.; Hämmerle C.H.	2014
Maxillary sinus floor elevation surgery with BioOss® mixed with a bone marrow concentrate or autogenous bone: Test of principle on implant survival and clinical performance	Rickert D.; Vissink A.; Slot W.J.; Sauerbier S.; Meijer H.J.A.; Raghoebar G.M.	2014
The impact of cantilevers on biological and technical success outcomes of implant-supported fixed partial dentures. A retrospective cohort study	Kim P.; Ivanovski S.; Latcham N.; Mattheos N.	2014
Influence of crown-implant ratio on implant success rates and crestal bone levels: a 36-month follow-up prospective study	Malchiodi L.; Cucchi A.; Ghensi P.; Consonni D.; Nocini P.F.	2014
The effect of opposing structures, natural teeth vs. implants on crestal bone levels surrounding single-tooth implants	Urdaneta R.A.; Leary J.; Panetta K.M.; Chuang S.-K.	2014
Maintenance of marginal bone support and soft tissue esthetics at immediately provisionalized OsseoSpeed implants placed into extraction sites: 2-year results	Noelken R.; Neffe B.A.; Kunkel M.; Wagner W.	2014
Implants in reconstructed bone: a comparative study on the outcome of Straumann® tissue level and bone level implants placed in vertically deficient alveolar ridges treated by means of autogenous onlay bone grafts	Chiapasco M.; Casentini P.; Zaniboni M.	2014
Microcalorimetric determination of the effects of amoxicillin, metronidazole, and their combination on in vitro biofilm	Astasov-Frauenhoffer M.; Braissant O.; Hauser-Gerspach I.; Weiger R.; Walter C.; Zitzmann N.U.; Waltimo T.	2014
Stent length and GP2B/3A Inhibitor use but not eGFR predict peri-procedural myocardial infarction in elective percutaneous coronary intervention	Goh C.; Al-Fiadh A.; Clark D.; Farouque O.	2014
Contemporary comparison between retropubic mid urethral sling (MUS) and autologous pubovaginal sling (PVS) for stress urinary incontinence (SUI) after the FDA advisory warning	Mock S.; Angelle J.; Reynolds W.S.; Osborn D.J.; Dmochowski R.R.; Gomelsky A.	2014
Single-incision mini-slings versus standard midurethral slings in surgical management of female stress urinary incontinence: An updated systematic review and meta-analysis of effectiveness and complications	Mostafa A.; Lim C.P.; Hopper L.; Madhuvrata P.; Abdel-Fattah M.	2014
Peri-implantitis: A review	Saini R.; Giri P.A.; Saini S.; Saini S.R.	2014
A retrospective 3- to 5-year study of the reconstruction of oral function using implant-supported prostheses in patients with hypohidrotic ectodermal dysplasia.	Zou D.; Wu Y.; Wang X.D.; Huang W.; Zhang Z.; Zhang Z.	2014

The clinical outcome of dental implants placed through skin flaps	Li B.-H.; Byun S.-H.; Kim S.-M.; Lee J.-H.	2014
The effects of anti-infective preventive measures on the occurrence of biologic implant complications and implant loss: a systematic review.	Salvi G.E.; Zitzmann N.U.	2014
Biological implant complications and their management.	Hsu Y.T.; Mason S.A.; Wang H.L.	2014
Osseointegration and re-osseointegration	Meenakshi Priyanka V.; Ramamoorthy J.	2014
Overdentures on primary mandibular implants in patients with oral cancer: A follow-up study over 14 years	Korfage A.; Raghoebar G.M.; Slater J.J.R.H.; Roodenburg J.L.N.; Witjes M.J.H.; Vissink A.; Reintsema H.	2014
Single-blind randomized clinical trial to evaluate clinical and radiological outcomes after one year of immediate versus delayed implant placement supporting full-arch prostheses	Pellicer-Chover H.; Peñarrocha-Oltra D.; Bagán L.; Fichy-Fernandez A.-J.; Canullo L.; Peñarrocha-Diago M.	2014
Interleukin-17 in peri-implantitis: Bone protector or destroyer?	Liu B.; Song N.; Bu J.; Feng W.	2014
Peri-implantitis diagnosis and treatment by New Zealand periodontists and oral maxillofacial surgeons.	Russell A.A.; Tawse-Smith A.; Broadbent J.M.; Leichter J.W.	2014
Subcutaneous emphysema following non-surgical peri-implantitis therapy using an air abrasive device: a case report	Bassetti M.; Bassetti R.; Sculean A.; Salvi G.E.	2014
Microbial analysis of biofilms on cement surfaces: An investigation in cement-associated peri-implantitis	Korsch M.; Walther W.; Marten S.-M.; Obst U.	2014
Immediate functional loading of single implants: a 1-year interim report of a 5-year prospective multicentre study.	Luongo G.; Lenzi C.; Raes F.; Eccellente T.; Ortolani M.; Mangano C.	2014
Radiographic bone level changes of implant-supported restorations in edentulous and partially dentate patients: 5-year results	Gholami H.; Mericske-Stern R.; Kessler-Liechti G.; Katsoulis J.	2014
Immediate versus early loading of mini-implants supporting mandibular overdentures: a preliminary 3-year clinical outcome report	Maryod W.H.; Ali S.M.; Shawky A.F.	2014
Effects of Er,Cr:YSGG laser irradiation on the surface characteristics of titanium discs: An in vitro study	Ercan E.; Arin T.; Kara L.; Çandirli C.; Uysal C.	2014
Biomechanics and peri-implantitis: The effect of a subcrestal wing-thread to decrease alveolar crestal bone strain. theory, finite element analysis, and clinical application.	Laster Z.; Weissberg I.; Kablan F.	2014
A two-stage surgical approach to the treatment of severe peri-implant defect: a 30-month clinical follow-up report.	Kim J.E.; Kim H.Y.; Huh J.B.; Lee J.Y.; Shin S.W.	2014
Antibiotic resistance in human peri-implantitis microbiota	Rams T.E.; Degener J.E.; van Winkelhoff A.J.	2014
Dental implants: Monitoring and hygiene are essential	Buxeraud J.	2014
Evaluation of the influence of keratinized mucosa width on peri-implant soft and hard tissue health	Zhang Y.-X.; Shu R.; Xie Y.-F.	2014
The association of clinical and microbiologic parameters with histologic observations in relatively healthy peri-implant conditions- a preliminary short-term in vivo study	van Brakel R.; Meijer G.J.; de Putter C.; Verhoeven J.W.; Jansen J.; Cune M.S.	2014
Three-year post-loading results of a randomised, controlled, split-mouth trial comparing implants with different prosthetic interfaces and design in partially posterior edentulous mandibles.	Pozzi A.; Tallarico M.; Moy P.K.	2014
Saliva versus peri-implant inflammation: quantification of IL-1β in partially and totally edentulous patients.	Rocha F.S.; Jesus R.N.; Rocha F.M.; Moura C.C.; Zanetta-Barbosa D.	2014
Clinical and radiological evaluation of a template-guided (NobelGuide™) treatment concept	Vasak C.; Kohal R.J.; Lettner S.; Rohner D.; Zechner W.	2014
Nanocrystalline hydroxyapatite-based material already contributes to implant stability after 3 months: a clinical and radiologic 3-year follow-up investigation.	Ghanaati S.; Lorenz J.; Obreja K.; Choukroun J.; Landes C.; Sader R.A.	2014
Clinical study of flap design to increase the keratinized gingiva around implants: 4-year follow-up.	Bruschi G.B.; Crespi R.; Capparé P.; Gherlone E.	2014
Immediate loading of two flapless placed mandibular implants supporting cross-arch fixed prostheses: a 3-year follow-up prospective single cohort study.	Cannizzaro G.; Felice P.; Boveri M.; Lazzarini M.; Ferri V.; Leone M.; Esposito M.	2014
Complications in implant dentistry.	Pi-Anfruns J.	2014
Immediately loaded distally cantilevered fixed mandibular prostheses supported by four implants placed in both in fresh extraction and healed sites: 2-year results from a prospective study.	Krennmair S.; Seemann R.; Weinländer M.; Krennmair G.; Piehslinger E.	2014
Esthetic outcomes following immediate and early implant placement in the anterior maxilla--a systematic review.	Chen S.T.; Buser D.	2014
Loading protocols for single-implant crowns: a systematic review and meta-analysis.	Benic G.I.; Mir-Mari J.; Hämmerle C.H.	2014
One abutment-one time versus a provisional abutment in immediately loaded post-extractive single implants: a 1-year follow-up of a multicentre randomised controlled trial.	Grandi T.; Guazzi P.; Samarani R.; Maghaireh H.; Grandi G.	2014
Effects of acidic sodium fluoride-treated, commercially pure titanium on periodontal pathogens and rat bone marrow cells	Shimogishi M.; Tsutsumi Y.; Kuroda S.; Munakata M.; Hanawa T.; Kasugai S.	2014
The impact of a laser-microtextured collar on crestal bone level and clinical parameters under various placement and loading protocols.	Guarnieri R.; Serra M.; Bava L.; Grande M.; Farronato D.; Iorio-Siciliano V.	2014
In which direction will future clinical research go?	Esposito M.	2014
A study of interleukin 1β levels in peri-miniscrew crevicular fluid (PMCF)	Monga N.; Chaurasia S.; Kharbanda O.P.; Duggal R.; Rajeswari M.R.	2014

1-stage versus 2-stage lateral sinus lift procedures: 1-year post-loading results of a multicentre randomised controlled trial.	Felice P.; Pistilli R.; Piattelli M.; Soardi E.; Barausse C.; Esposito M.	2014
A short-term assessment of angulated abutments for the implant denture restorations in posterior teeth	Feng W.; Geng J.-Y.; Sun Y.-Y.; Geng H.-X.	2014
Influence of occlusal loading on peri-implant clinical parameters. A pilot study	Pellicer-Chover H.; Viña-Almunia J.; Romero-Millán J.; Peñarrocha-Oltra D.; García-Mira B.; Peñarrocha-Diago M.	2014
Immediate loading with fixed full-arch prostheses in the maxilla: Review of the literature	Peñarrocha-Oltra D.; Covani U.; Peñarrocha-Diago M.; Peñarrocha-Diago M.	2014
Clinical and radiologic experience with moderately rough oxidized titanium implants: up to 10 years of retrospective follow-up.	Pozzi A.; Mura P.	2014
Soft tissue augmentation procedures for mucogingival defects in esthetic sites.	Levine R.A.; Huynh-Ba G.; Cochran D.L.	2014
Comparison of two different abutment designs on marginal bone loss and soft tissue development.	Patil R.C.; den Hartog L.; van Heereveld C.; Jagdale A.; Dilbaghi A.; Cune M.S.	2014
[Enlargement of keratinized peri-implant mucosa at the time of second stage surgery (re-entry)]	Kaufmann R.; Bassetti R.; Mericske-Stern R.; Enkling N.	2014
Flapless dental implant surgery: a retrospective study of 1,241 consecutive implants.	Doan N.V.; Du Z.; Reher P.; Xiao Y.	2014
Implant-retained overdentures for young children with severe oligodontia: A series of four cases	Filius M.A.P.; Vissink A.; Raghoebar G.M.; Visser A.	2014
Efficacy and safety of a diode laser in second-stage implant surgery: A comparative study	El-Kholey K.E.	2014
Effects of currently available surgical and restorative interventions on reducing midfacial mucosal recession of immediately placed single-tooth implants: A systematic review	Lin G.-H.; Chan H.-L.; Wang H.-L.	2014
Immediate versus delayed loading of dental implants in edentulous patients' maxillae: a 6-year prospective study.	Tealdo T.; Menini M.; Bevilacqua M.; Pera F.; Pesce P.; Signori A.; Pera P.	2014
Animal models for craniofacial reconstruction by Stem/stromal cells	Liu N.; Lyu X.; Fan H.; Shi J.; Hu J.; Luo E.	2014
Immediate, non-submerged, root-analogue direct laser metal sintering (DLMS) implants: A 1-year prospective study on 15 patients	Mangano F.G.; De Franco M.; Caprioglio A.; MacChi A.; Piattelli A.; Mangano C.	2014
Correlation between interimplant distance and crestal bone loss in internal connection implants with platform switching.	Jo D.W.; Yi Y.J.; Kwon M.J.; Kim Y.K.	2014
Prosthodontic management of maxillectomy patients with dental implants in residual zygomatic bone: a preliminary report	El-Sayed W.M.; Gad M.A.; Medra A.M.	2014
The fate of marginal bone around axial vs. tilted implants: a systematic review.	Del Fabbro M.; Ceresoli V.	2014
Conversion of an existing metal ceramic crown to an interim restoration and nonfunctional loading of a single implant in the maxillary esthetic zone: A clinical report	Michalakis K.; Kalpidis C.D.R.; Hirayama H.	2014
Accuracy of peri-implant bone evaluation using cone beam CT, digital intra-oral radiographs and histology.	Ritter L.; Elger M.C.; Rothamel D.; Fienitz T.; Zinser M.; Schwarz F.; Zöller J.E.	2014
Consensus statements and clinical recommendations for prevention and management of biologic and technical implant complications.	Heitz-Mayfield L.J.; Needleman I.; Salvi G.E.; Pjetursson B.E.	2014
Anaplastic large-cell lymphoma associated with breast implants: A unique entity within the spectrum of peri-implant effusions	Chai S.M.; Kavangh S.; Ooi S.S.; Sterrett G.F.; Cull G.; Plunkett M.; Spagnolo D.; Amanuel B.; Joske D.; Leslie C.; Barham T.; Frost F.	2014
Combined surgical therapy of peri-implantitis	Santoro G.; Masci A.; Scacchetti M.; Borgonovo A.	2014
Chondroitin sulfate and sulfated hyaluronan-containing collagen coatings of titanium implants influence peri-implant bone formation in a minipig model	Korn P.; Schulz M.C.; Hintze V.; Range U.; Mai R.; Eckelt U.; Schnabelrauch M.; Möller S.; Becher J.; Scharnweber D.; Stadlinger B.	2014
Implant abutment connection: Biomechanical perspectives	Shetty M.; Krishna Prasad D.; Shetty N.H.G.; Jaiman R.	2014
Implant treatment in atrophic posterior mandibles: vertical regeneration with block bone grafts versus implants with 5.5-mm intrabony length.	Peñarrocha-Oltra D.; Aloy-Prósper A.; Cervera-Ballester J.; Peñarrocha-Diago M.; Canullo L.; Peñarrocha-Diago M.	2014
Accuracy of CBCT images in the assessment of buccal marginal alveolar peri-implant defects: effect of field of view.	Kamburoğlu K.; Murat S.; Kiliç C.; Yüksel S.; Avsever H.; Farman A.; Scarfe W.C.	2014
The use of a polycaprolactone-tricalcium phosphate scaffold for bone regeneration of tooth socket facial wall defects and simultaneous immediate dental implant placement in Macaca fascicularis	Goh B.T.; Chanchareonsook N.; Tideman H.; Teoh S.H.; Chow J.K.F.; Jansen J.A.	2014
Narrow-diameter implants: Are they a predictable treatment option? A literature review	Sierra-Sánchez J.-L.; Martínez-González A.; García-Sala Bonmatí F.; Mañes-Ferrer J.-F.; Brotons-Oliver A.	2014
[A tooth or an implant--literature based decision making].	Bar On H.; Sharon E.; Lipovezky-Adler M.; Haramaty O.; Smidt A.	2014
Graftless sinus augmentation technique with contextual placement of implants: A case report	Chipaila N.; Marini R.; Sfasciotti G.L.; Cielo A.; Bonanome L.; Monaco A.	2014
Distal cantilever in full-arch prostheses and immediate loading: a retrospective clinical study.	Romanos G.E.; Gupta B.; Gaertner K.; Nentwig G.H.	2014
Effects of connective tissue grafting on the facial gingival level following single immediate implant placement and provisionalization in the esthetic zone: a 1-year randomized controlled prospective study.	Yoshino S.; Kan J.Y.; Rungcharassaeng K.; Roe P.; Lozada J.L.	2014
Clinical outcome of narrow-diameter (3.3-mm) locking-taper implants: a prospective study with 1 to 10 years of follow-up.	Mangano F.; Shibli J.A.; Sammons R.L.; Veronesi G.; Piattelli A.; Mangano C.	2014
Synergistic effects of bisphosphonate and calcium phosphate nanoparticles on peri-implant bone responses in osteoporotic rats	Alghamdi H.S.; Bosco R.; Both S.K.; Iafisco M.; Leeuwenburgh S.C.G.; Jansen J.A.; Van den Beucken J.J.J.P.	2014
Complication rate in 200 consecutive sinus lift procedures: Guidelines for prevention and treatment	Moreno Vazquez J.C.; Gonzalez De Rivera A.S.; Gil H.S.; Mifsut R.S.	2014

Characterization of macrophage polarizing cytokines in the aseptic loosening of total hip replacements	Jämsen E.; Kouri V.-P.; Olkkonen J.; Cör A.; Goodman S.B.; Konttinen Y.T.; Pajarinen J.	2014
A systematic review of implant-supported overdentures in the edentulous maxilla, compared to the mandible: how many implants?	Raghoebar G.M.; Meijer H.J.; Slot W.; Slater J.J.; Vissink A.	2014
Buccal bone resorption around posterior implants after surgery: a 1-year prospective study.	Takuma T.; Oishi K.; Manabe T.; Yoneda S.; Nagata T.	2014
Initial effects of a treatment by fixed partial dentures supported by mini dental implants from a patient's point of view	Peršić S.; Palac A.; Vojvodić D.; Čelebić A.	2014
Autologous bone marrow aspirate concentrate repairs peri-implant bone defect	Yang Y.; Zhong W.-J.; Liu G.; Ma G.-W.	2014
Cosci versus Summers technique for crestal sinus lift: 3-year results from a randomised controlled trial.	Esposito M.; Cannizzaro G.; Barausse C.; Cosci F.; Soardi E.; Felice P.	2014
Applications of lasers in dental implantology	Mu Y.; Li Q.; Zhao J.-Z.	2014
Reconstruction of the extremely atrophied mandible with iliac crest onlay grafts followed by two endosteal implants: A retrospective study with long-term follow-up	Boven G.C.; Meijer H.J.A.; Vissink A.; Raghoebar G.M.	2014
Tension band wiring for proximal femoral varus osteotomy fixation in children	Maranho D.A.; Pagnano R.G.; Volpon J.B.	2014
The outcome of surgical treatment for periacetabular metastases	Shahid M.; Saunders T.; Jeys L.; Grimer R.	2014
Maxillary sinus floor elevation using the osteotome technique in the presence of antral pseudocysts: a retrospective study with an average follow-up of 27 months.	Feng Y.; Tang Y.; Liu Y.; Chen F.; Li D.	2014
Systematic review and meta-analysis of the additional benefit of local prophylactic antibiotic therapy for infection rates in open tibia fractures treated with intramedullary nailing	Craig J.; Fuchs T.; Jenks M.; Fleetwood K.; Franz D.; Iff J.; Raschke M.	2014
Inhibition of biofilm formation and antibacterial properties of a silver nano-coating on human dentine	Besinis A.; De Peralta T.; Handy R.D.	2014
The performance of cross-linked acellular arterial scaffolds as vascular grafts; pre-clinical testing in direct and isolation loop circulatory models	Pennel T.; Fercana G.; Bezuidenhout D.; Simionescu A.; Chuang T.-H.; Zilla P.; Simionescu D.	2014
Nerve damage assessment following implant placement in human cadaver jaws: an ex vivo comparative study.	Murat S.; Kamburoğlu K.; Kiliç C.; Ozen T.; Gurbuz A.	2014
Mid-term survival following primary hinged total knee replacement is good irrespective of the indication for surgery	Baker P.; Critchley R.; Gray A.; Jameson S.; Gregg P.; Port A.; Deehan D.	2014
Peri-strut low-intensity areas in optical coherence tomography correlate with peri-strut inflammation and neointimal proliferation: An in-vivo correlation study in the familial hypercholesterolemic coronary swine model of in-stent restenosis	Tellez A.; Afari M.E.; Buszman P.P.; Seifert P.; Cheng Y.; Milewski K.; McGregor J.C.; Garza J.A.; Roberts M.B.; Yi G.H.; Kaluza G.L.; Granada J.F.	2014
Perioperative adverse events after minimally invasive abdominal sacrocolpopexy	Unger C.A.; Paraiso M.F.R.; Jelovsek J.E.; Barber M.D.; Ridgeway B.	2014
Clinical use of optical coherence tomography to identify angiographic silent stent thrombosis	Steigen S.E.; Holm N.R.; Butt N.; Maeng M.; Otsuka F.; Virmani R.; Ladich E.; Steigen T.K.	2014
Effect of total-body prehabilitation on postoperative outcomes: A systematic review and meta-analysis	Santa Mina D.; Clarke H.; Ritvo P.; Leung Y.W.; Matthew A.G.; Katz J.; Trachtenberg J.; Alibhai S.M.H.	2014
Predicative modelling for regional intensive care stroke workload: A review of annual neurointensive care workload	Thompson R.; Laws P.	2014
Ultrastructural analysis of nanoparticles and ions released in periprosthetic membranes	Sabbatini M.; Gatti G.; Renò F.; Bosetti M.; Marchese L.; Cannas M.	2014
Relationship between peri-operative outcomes and hospital surgical volume of total hip arthroplasty in Japan	Kaneko T.; Hirakawa K.; Fushimi K.	2014
Influence of ureter stenting before ureterorenoscopic treatment of ureteral calculi	Mueller J.; Riechert-Mühe N.; Schrader A.J.; Leitenberger A.; Steinestel J.; Kuczyk M.A.; Steffens S.; Hofmann R.; Sotelino J.	2014
Ischemia and functional status of the left arm and quality of life after left subclavian artery coverage during stent grafting of thoracic aortic diseases	Klocker J.; Koell A.; Erlmeier M.; Goebel G.; Jaschke W.; Fraedrich G.	2014
Percutaneous transcatheter left atrial appendage closure devices: Role in the long-term management of atrial fibrillation	Gan C.H.; Bhat A.; Davis L.; Denniss A.R.	2014
Peri-procedural management of anti-platelets and anticoagulation in patients undergoing MitraClip procedure	Alsidawi S.; Effat M.	2014
Predictors of hospital length of stay after implantation of a left ventricular assist device: An analysis of the INTERMACS registry	Cotts W.G.; McGee Jr. E.C.; Myers S.L.; Naftel D.C.; Young J.B.; Kirklin J.K.; Grady K.L.	2014
Role of cardiovascular intervention as a bridge to liver transplantation	Raval Z.; Harinstein M.E.; Flaherty J.D.	2014
Five-level sub-axial cervical vertebrectomy and reconstruction: Technical report	Reig A.; Parker S.L.; McGirt M.J.	2014
Randomized controlled trial comparing two procedures for anterior vaginal wall prolapse	Minassian V.A.; Parekh M.; Poplawsky D.; Gorman J.; Litzy L.	2014
Current concepts in the management of periprosthetic fungal joint infection using antifungal bone cement	Goff T.A.J.; Rambani R.; Ng A.B.	2014
Comparative effectiveness of ruptured cerebral aneurysm therapies: Propensity score analysis of clipping versus coiling	McDonald J.S.; McDonald R.J.; Fan J.; Kallmes D.F.; Lanzino G.; Cloft H.J.	2014
Stent outcomes for infrapopliteal arterial occlusive disease	Mwipatayi B.P.; Thomas S.; Angel D.; Wong J.; Vijayan V.	2013
Ten-year survival and success rate of implant-prosthodontic treatment	Ihan Hren N.; Kiteska B.; Kopač I.	2013
Microbiological aspects of human mandibular subperiosteal dental implants.	Rams T.E.; Balkin B.E.; Roberts T.W.; Molzan A.K.	2013

Periodontitis. Oral Complication of Diabetes.	Preshaw P.M.; Bissett S.M.	2013
Minimum intervention dentistry: Periodontics and implant dentistry	Darby I.B.; Ngo L.	2013
Release of bone markers in immediately loaded and nonloaded dental implants: A randomized clinical trial	Prati A.J.; Casati M.Z.; Ribeiro F.V.; Cirano F.R.; Pastore G.P.; Pimentel S.P.; Casarin R.C.V.	2013
Periimplant changes in different transplanted soft tissues around loaded endosseous implants in patients after oral tumor surgery	Kovács A.F.; Wallowy P.; Stefenelli U.; Landau S.	2013
Long-term stability of early implant placement with contour augmentation	Buser D.; Chappuis V.; Kuchler U.; Bornstein M.M.; Wittneben J.G.; Buser R.; Cavusoglu Y.; Belser U.C.	2013
Assessment of buccal marginal alveolar peri-implant and periodontal defects using a cone beam CT system with and without the application of metal artefact reduction mode.	Kamburoglu K.; Kolsuz E.; Murat S.; Eren H.; Yüksel S.; Paksoy C.S.	2013
[Risk assessment in patients undergoing osseous antiresorptive therapy in dentistry. An update].	Borm J.M.; Moser S.; Locher M.; Damerau G.; Stadlinger B.; Grätz K.W.; Jacobsen C.	2013
Influence of platform switching on bone-level alterations: A three-year randomized clinical trial	Enkling N.; Jöhren P.; Katsoulis J.; Bayer S.; Jervøe-Storm P.-M.; Mericske-Stern R.; Jepsen S.	2013
A retrospective study of implant-abutment connections on crestal bone level	Lin M.-I.; Shen Y.-W.; Huang H.-L.; Hsu J.-T.; Fuh L.-J.	2013
Photodynamic therapy in dentistry: A literature review	Gursoy H.; Ozcakir-Tomruk C.; Tanalp J.; Yilmaz S.	2013
Long-term outcomes of dental implants with a titanium plasma-sprayed surface: a 20-year prospective case series study in partially edentulous patients	Chappuis V.; Buser R.; Brägger U.; Bornstein M.M.; Salvi G.E.; Buser D.	2013
A case of a late and atypical knee prosthetic infection by no-biofilm producer Pasteurella multocida strain identified by pyrosequencing	Romanò C.L.; De Vecchi E.; Vassena C.; Manzi G.; Drago L.	2013
Orthopaedic patients undergoing total hip or knee replacement: Their dental hygiene status and perceptions of risk of peri-operative infection associated with future dental treatment	Baskaran D.; Ujam A.; Ranjith R.	2013
Is there a gender difference in the outcome of cardiac interventions?	Subramanian G.; Chidambaram S.; Sangareddi V.	2013
Clinical presentation and management strategies of tubercular pseudoaneyrsm arising from large arteries: A tertiary care centre experience	Singh R.; Mohan B.; Wander P.; Tandon R.; Chhabra S.T.; Aslam N.	2013
Post-operative complications following primary ACL reconstruction using allogenic and autogenic soft tissue grafts: Increased relative morbidity risk is associated with increased graft diameter	Crawford D.C.; Hallvik S.E.; Petering R.C.; Quilici S.M.; Black L.O.; Lavigne S.A.; Lapidus J.; Marshall L.M.	2013
Effect of operator volume on mortality and complications of carotid artery stenting: United states perspective of last 5 years	Chothani A.; Patel N.J.; Mehta K.; Shah N.; Savani G.T.; Arora S.; Grover P.; Tokas A.; Singh V.; Deshmukh A.; Rathod A.; Tuliani T.; Sudhakar R.; Badheka A.O.; Mohamad T.; Elder M.; Kondur A.; Schreiber T.L.	2013
Three-year clinical data of the biosolve-I study with the paclitaxel-eluting bioabsorbable magnesium scaffold (DREAMS) and multi-modality imaging analysis	Haude M.; Erbel R.; Erne P.; Verheye S.; Vermeersch P.; Degen H.; Böse D.; Waksman R.; Weissman N.; Prati F.; Koolen J.	2013
A technique elucidating the retrieval of an adhered cover screw in a dental implant - A case report	Bhuvaneswarri J.; Chandrasekaran S.C.	2013
Influence of implant neck design and implant-abutment connection type on peri-implant health. Radiological study	Peñarrocha-Diago M.A.; Flichy-Fernández A.J.; Alonso-González R.; Peñarrocha-Oltra D.; Balaguer-Martínez J.; Peñarrocha-Diago M.	2013
Thermodynamic effects of laser irradiation of implants placed in bone: An in vitro study	Leja C.; Geminiani A.; Caton J.; Romanos G.E.	2013
Ailing and failing oral implants: initial therapy and surgical management	Sakka S.; Idrees M.; Alissa R.; Kujan O.	2013
Peri-implant diseases and collaborative treatment.	Froum S.J.	2013
Behaviour of soft tissues healing around single bone-level-implants placed immediately after tooth extraction A 1 year prospective cohort study	Lops D.; Romeo E.; Chiapasco M.; Procopio R.M.; Oteri G.	2013
The importance of oral health in (frail) elderly people - A review	Van Der Putten G.-J.; De Visschere L.; Van Der Maarel-Wierink C.; Vanobbergen J.; Schols J.	2013
Silk fibroin and 4-hexylresorcinol incorporation membrane for guided bone regeneration	Lee S.-W.; Kim S.-G.; Song J.-Y.; Kweon H.; Jo Y.-Y.; Lee K.-G.; Kang S.-W.; Yang B.-E.	2013
In patients with peri-implantitis, access flap surgery may be more effective than mechanical debridement in terms of clinical attachment gain although both treatments lead to improved clinical parameters (UT CAT #2432).	Bautista L.; Huynh-Ba G.	2013
No-flow fraction and peri-shock pause during BLS given by healthcare professionals before and after a BLS course and after additional video instructions	Eichelkraut A.; Poenicke C.; Karg S.; Rudolf C.; Papkalla N.; Richter T.; Koch T.; Koerndle H.; Narciss S.; Muller M.	2013
Outcomes and total costs of outpatient vs. inpatient peri-procedural anticoagulation management of mechanical prosthetic heart valve patients	Attaya H.; Shah N.D.; Wysokinski W.E.; Van Houten H.K.; Heit J.A.; Mcbane II R.D.	2013
Identification of high-risk plaques associated with peri-procedural myocardial injury following elective percutaneous coronary intervention: Assessment by high-sensitivity troponin-T measurements and optical coherence tomography	Yamamoto M.; Seino Y.; Otsuka T.; Kurihara O.; Negishi K.; Murakami D.; Takano M.; Mizuno K.	2013
Patient specific mandible reconstruction-virtual preoperative planning for ideal oral rehabilitation	Essig H.; Schumann P.; Lindhorst D.; Rücker M.; Gellrich N.C.	2013

Surgical reconstruction of peri-implant bone defects with prehydrated and collagenated porcine bone and collagen barriers: case presentations	Slotte C.; Lindfors N.; Nannmark U.	2013
Effects of smoking on periimplant health status and IL-1β, TNF-α, and PGE2 levels in periimplant crevicular fluid: a cross-sectional study on well-maintained implant recall patients	Tatli U.; Damlar I.; Erdoğan O.; Esen E.	2013
PCL-TCP scaffold for bone regeneration of tooth socket facial wall defects and simultaneous immediate implant placement in Macaca fascicularis	Goh B.T.; Chanchareonsook N.; Tideman H.; Swee Hin T.; Jansen J.	2013
Implant surface detoxification: a comprehensive review	Suarez F.; Monje A.; Galindo-Moreno P.; Wang H.-L.	2013
Management of peri-implant soft tissues for rehabilitation of patients with resected mandibles reconstructed using free fibula flap	Subramanian A.V.; C J.P.; Kuriakose M.A.; S B.A.; Kumar V.V.	2013
Surgical treatment of pathologic fractures of the proximal femur due to metastases	Rossi B.; Piccioli A.; Di Giacomo G.; Scudieri G.; Maccauro G.	2013
An in vitro study of alginate oligomer therapies on oral biofilms	Roberts J.L.; Khan S.; Emanuel C.; Powell L.C.; Pritchard M.F.; Onsøyen E.; Myrvold R.; Thomas D.W.; Hill K.E.	2013
Soft and hard tissues healing at immediate transmucosal implants placed into molar extraction sites with collagen membrane uncovered: a 12-month prospective study	Cafiero C.; Marenzi G.; Blasi A.; Siciliano V.I.; Nicolò M.; Sammartino G.	2013
The composite serratus anterior/rib free flap: A viable alternative to the fibula in head and neck reconstruction	Ramirez C.; Petrisor D.; Pittman A.; Wax M.; Fernandes R.	2013
Transfer of hip arthroplasty patients leads to increased cost and resource utilization in the receiving hospital	Kamath A.F.; Austin D.C.; Derman P.B.; Israelite C.L.	2013
Management of pancreatic collections with a novel endoscopically placed fully covered self expanding metal stent: The australian experience	Chandran S.; Efthymiou M.; Kaffes A.; Kwan V.; Chen J.W.; Murray M.; Williams D.; Welch C.; Chong A.; Gupta S.; Tam W.; Devereaux B.; Nguyen N.; Vaughan R.	2013
Successful treatment of duodenal perforation with over-the-scope clip after failed surgical repair	Fleming J.; Greenspan N.; Feller E.; Shah S.	2013
Influence of PRF in the healing of bone and gingival tissues. Clinical and histological evaluations	Marrelli M.; Tatullo M.	2013
Effect of zinc ions on improving implant fixation in osteoporotic bone	Li X.; Li Y.; Peng S.; Ye B.; Lin W.; Hu J.	2013
Re-stability of dental implants following treatment of peri-implantitis	Javed F.; Hussain H.A.; Romanos G.E.	2013
Managing an extreme peri-implantitis	Materni A.	2013
Supportive periodontal therapy and periodontal biotype as prognostic factors in implants placed in patients with a history of periodontitis	Aguirre-Zorzano L.-A.; Vallejo-Aisa F.-J.; Estefanía-Fresco R.	2013
Immediate provisionalisation of single post-extractive implants versus implants placed in healed sites in the anterior maxilla: 1-year results from a multicentre controlled cohort study	Grandi T.; Guazzi P.; Samarani R.; Grandi G.	2013
Use of platform-switched implants with internal conical anti-rotational connection in single-tooth implant replacement	Di Iorioa E.; Berardini M.	2013
Safety and efficacy of a biomimetic monolayer of permanently bound multi-phosphonic acid molecules on dental implants: 1 year post-loading results from a pilot quadruple-blinded randomised controlled trial	Esposito M.; Dojcinovic I.; Germon L.; Lévy N.; Curno R.; Buchini S.; Péchy P.; Aronsson B.-O.	2013
Immediate loading of 'All-on-4' maxillary prostheses using trans-sinus tilted implants without sinus bone grafting: a retrospective study reporting the 3-year outcome	Maló P.; Nobre Md.; Lopes A.	2013
Hard tissue response to argon plasma cleaning/sterilisation of customised titanium abutments versus 5-second steam cleaning: results of a 2-year post-loading follow-up from an explanatory randomised controlled trial in periodontally healthy patients	Canullo L.; Penarrocha D.; Micarelli C.; Massidda O.; Bazzoli M.	2013
Skin-reducing mastectomy and one-stage implant reconstruction with a myodermal flap: A safe and effective technique in risk-reducing and therapeutic mastectomy	Irwin G.W.; Black A.; Refsum S.E.; McIntosh S.A.	2013
Stenting vs. Endarterectomy vs. Medical therapy trials update	Taylor P.R.	2013
Platelet reactivity measurements before and after percutaneous coronary intervention among patients undergoing early invasive management for acute coronary syndrome	Jing M.X.; Carvalho L.; Mejin M.; Fong A.; Troughton R.; Chin C.T.; Teo Z.L.; Byan; Richards A.M.; Tang H.C.; Chan M.	2013
Utility of week one radiographs post total hip arthroplasty	Stevens J.; van Tonder F.; Schlicht S.; Love B.; Goldwasser M.; Choong P.	2013
Results of the surgical correction of urinary stress incontinence according to the type of transobturator tape utilized	Padilla-Fernández B.; García-Cenador M.B.; Gómez-García A.; Mirón-Canelo J.A.; Gil-Vicente Á.; Silva-Abuín J.M.; Lorenzo-Gómez M.F.	2013
Clinical application of artificial lumbar disc replacement: Present and future	Bai W.-Y.; Gu H.-S.; Liao Z.-H.; Liu W.-Q.	2013
Ozone applications in dentistry: An overview	Ahmed J.; Binnal A.; Rajan B.; Denny C.; Shenoy N.	2013
Network meta-analysis for evaluating interventions in implant dentistry: the case of peri-implantitis treatment	Faggion C.M.; Chambrone L.; Listl S.; Tu Y.-K.	2013
Radiographic analysis of a transalveolar sinus-lift technique: A multipractice retrospective study with a mean follow-up of 5 years	Soardi E.; Cosci F.; Checchi V.; Pellegrino G.; Bozzoli P.; Felice P.	2013
Long-term results after treatment of periodontitis in patients with Papillon-Lefèvre syndrome: Success and failure	Nickles K.; Schacher B.; Ratka-Krüger P.; Krebs M.; Eickholz P.	2013
The bleeding site: A multi-level analysis of associated factors	Farina R.; Tomasi C.; Trombelli L.	2013

Impact of excessive occlusal load on successfully-osseointegrated dental implants: a literature review.	Chang M.; Chronopoulos V.; Mattheos N.	2013
Overdentures in the edentulous mandible supported by implants and retained by a Dolder bar: a 5-year prospective study	Heschl A.; Payer M.; Clar V.; Stopper M.; Wegscheider W.; Lorenzoni M.	2013
Reduction of precocious peri-implant resorption cone	De Santis D.; Zanotti G.; Morandini S.; Bordanzi A.; Gerosa R.; Rodella L.F.; Rossetto A.; Chiarini L.; Nocini P.F.; Bertossi D.	2013
B-type natriuretic peptide guided therapy reduces length of hospital stay post left ventricular assist device implantation	Hellman Y.; Malik A.S.; Wang I.-W.; Wozniak T.C.; Pickrell J.; Hashmi Z.A.; Gradus-Pizlo I.; Hadi A.	2013
Percutaneous coronary intervention improves ejection fraction in ischemic cardiomyopathy	Chonde M.; Morrissey R.; Azarbal B.; Herr J.; Hamilton M.	2013
Dabigatran-demonstrates the need for comprehensive approaches to optimise the use of new drugs	Malmstrom R.E.; Godman B.; Diogene E.; Bennie M.; Furst J.; Gutiérrez-Ibarluzea I.; McCullagh L.; Vlahovic-Palcevski V.; Gustafsson L.L.	2013
Inpatient procedures in elderly women: An analysis over time	Oliphant S.S.; Ghetti C.; McGough III R.L.; Wang L.; Bunker C.H.; Lowder J.L.	2013
Multi-contrast late enhancement CMR determined gray zone and papillary muscle involvement predict appropriate ICD therapy in patients with ischemic heart disease	Yang Y.; Connelly K.A.; Zeidan-Shwiri T.; Lu Y.; Paul G.; Roifman I.; Zia M.I.; Graham J.J.; Dick A.J.; Crystal E.; Wright G.A.	2013
Enhanced recovery in Sri Lanka	Fernando P.M.	2013
Deep circumflex iliac artery flap with osseointegrated implants for reconstruction of mandibular benign lesions: Clinical experience of 33 cases	Qu X.; Zhang C.; Yang W.; Wang M.	2013
Long-term follow-up of turned single implants placed in periodontally healthy patients after 16 to 22 years: Microbiologic outcome	Dierens M.; Vandeweghe S.; Kisch J.; Persson G.R.; Cosyn J.; De Bruyn H.	2013
Altered sensation caused by peri-implantitis: A case report	Kim J.-E.; Shim J.-S.; Huh J.-B.; Rim J.-S.; Lee J.-Y.; Shin S.-W.	2013
Tilted trans-sinus implants for the treatment of maxillary atrophy: Case series of 35 consecutive patients	Testori T.; Mandelli F.; Mantovani M.; Taschieri S.; Weinstein R.L.; Del Fabbro M.	2013
Vestibuloplasty: Porcine collagen matrix versus free gingival graft: A clinical and histologic study	Schmitt C.M.; Tudor C.; Kiener K.; Wehrhan F.; Schmitt J.; Eitner S.; Agaimy A.; Schlegel K.A.	2013
Accuracy of peri-implant bone thickness and validity of assessing bone augmentation material using cone beam computed tomography	Wang D.; Künzel A.; Golubovic V.; Mihatovic I.; John G.; Chen Z.; Becker J.; Schwarz F.	2013
A two-center retrospective analysis of long-term clinical and radiologic data of TiUnite and turned implants placed in the same mouth.	Polizzi G.; Gualini F.; Friberg B.	2013
Teeth--too early to eulogize!	Levin L.; Kessler-Baruch O.	2013
Dental implants placed on bone subjected to vertical alveolar distraction show the same performance as those placed on primitive bone	Pérez-Sayáns M.; León-Camacho M.L.A.; Somoza-Martín J.-M.; Fernández-González B.; Blanes-Vázquez-Gundín S.; Gándara-Rey J.-M.; García-García A.	2013
Evaluation of a compression resistant matrix for recombinant human bone morphogenetic protein-2	Lu S.X.; Fiorini T.; Lee J.; Prasad H.S.; Buxton A.N.; Bisch F.C.; Dixon D.R.; Susin C.; Wikesjö U.M.E.	2013
A lower border augmentation technique to allow implant placement after a bilateral mandibular fracture as a complication of vertical distraction osteogenesis: A case report	Perdijk F.B.T.; Meijer G.J.; Soehardi A.; Koole R.	2013
Transcatheter AVR-Does this technology have merit and in whom? An anesthesiologist's perspective	Shanewise J.	2013
Peri-implant galactocele following vacuum-assisted core biopsy of the breast: A cautionary tale	Taylor D.; Kulawansa S.T.; McCallum D.D.; Saunders C.	2013
Radiographic evaluation of modern oral implants with emphasis on crestal bone level and relevance to peri-implant health	De Bruyn H.; Vandeweghe S.; Ruyffelaert C.; Cosyn J.; Sennerby L.	2013
Immediate placement and provisionalization of single-tooth implants involving a definitive individual abutment: A clinical and radiographic retrospective study	Hartlev J.; Kohberg P.; Ahlmann S.; Gotfredsen E.; Andersen N.T.; Isidor F.; Schou S.	2013
Manual versus sonic-powered toothbrushing for plaque reduction in patients with dental implants: an explanatory randomised controlled trial.	Swierkot K.; Brusius M.; Leismann D.; Nonnenmacher C.; Nüsing R.; Lubbe D.; Schade-Brittinger C.; Mengel R.	2013
Peri-implant disease: current understanding and future direction.	Murray C.M.; Knight E.T.; Russell A.A.; Tawse-Smith A.; Leichter J.W.	2013
A comparison of teeth and implants during maintenance therapy in terms of the number of disease-free years and costs - An in vivo internal control study	Fardal Ø.; Grytten J.	2013
Evaluation of implant success: A review of past and present concepts	Karthik K.; Sivakumar; Sivaraj; Thangaswamy V.	2013
Techniques to control or avoid cement around implant-retained restorations	Present S.; Levine R.A.	2013
The effect of varying implant position in immediately loaded implant-supported mandibular overdentures.	Shaarawy M.A.; Aboelross E.M.	2013
Immediate loading of 2(all-on-2) versus 4 (all-on-4) implants placed with a flapless technique supporting mandibular cross-arch fixed prostheses: 1-year results from a pilot randomised controlled trial.	Cannizzaro G.; Felice P.; Soardi E.; Ferri V.; Leone M.; Lazzarini M.; Audino S.; Esposito M.	2013
1-stage versus 2-stage lateral maxillary sinus lift procedures: 4-month post-loading results of a multicenter randomised controlled trial.	Felice P.; Pistilli R.; Piattelli M.; Soardi E.; Pellegrino G.; Corvino V.; Esposito M.	2013
The patient with severe heart disease requiring OLV for non cardiac surgery	Rovira I.	2014
The comparison of acellular dermal matrix allografts with free gingival grafts in the augmentation of peri-implant attached mucosa: a randomised controlled trial.	Basegmez C.; Karabuda Z.C.; Demirel K.; Yalcin S.	2013
Application of ozone in the treatment of periodontal disease	Srikanth A.; Sathish M.; Harsha A.V.S.	2013

A review of factors influencing the incidence and severity of plaque-induced gingivitis	Trombelli L.; Farina R.	2013
The role of pericranium grafts in the reduction of postoperative dehiscences and bone resorption after reconstruction of severely deficient edentulous ridges with autogenous onlay bone grafts	Chiapasco M.; Autelitano L.; Rabbiosi D.; Zaniboni M.	2013
Photodynamic therapy: The imminent milieu for treating oral lesions	Mohanty N.; Jalaluddin M.; Kotina S.; Routray S.; Ingale Y.	2013
Preoperative optimization of anemia improves outcome?	Gombotz H.	2014
Transcatheter AVR – Does this technology have merit and in whom? An anesthesiologist's perspective	Shanewise J.	2014
Native mitral valve endocarditis caused by staphylococcus capitis	Del Rosario R.; Aldeen M.B.; Ahmed M.	2013
Aneurysm rupture after endovascular flow diversion: The possible role of persistent flows through the transition zone associated with device deformation	Darsaut T.E.; Rayner-Hartley E.; Makoyeva A.; Salazkin I.; Berthelet F.; Raymond J.	2013
Increased Crown-To-Implant Ratio May Not Be a Risk Factor for Dental Implant Failure under Appropriate Plaque Control	Okada S.; Koretake K.; Miyamoto Y.; Oue H.; Akagawa Y.	2013
One-piece zirconia oral implants: One-year results from a prospective case series. 2. Three-unit fixed dental prosthesis (FDP) reconstruction	Kohal R.-J.; Patzelt S.B.M.; Butz F.; Sahlin H.	2013
Single unit attachments improve peri-implant soft tissue conditions in mandibular overdentures supported by four implants	Cordaro L.; di Torresanto V.M.; Petricevic N.; Jornet P.R.; Torsello F.	2013
Recurrent oral squamous cell carcinoma after resection and mandibular implant rehabilitation: A preliminary report of eight patients	Beck-Mannagetta J.; Eirisch G.; Etzelsdorfer M.; Hutarew G.	2013
A 10-year retrospective clinical evaluation of immediately loaded tapered maxillary implants.	Harel N.; Piek D.; Livne S.; Palti A.; Ormianer Z.	2013
The characteristics and new treatment paradigm of dental implant-related chronic rhinosinusitis	Chen Y.-W.; Huang C.-C.; Chang P.-H.; Chen C.-W.; Wu C.-C.; Fu C.-H.; Lee T.-J.	2013
Immediate loading with LASER-treated surface implant: two-year resonance frequency analysis follow-up	Duvina M.; Barbato L.; Buti J.; Delle Rose G.; Brancato L.; Casella G.; Longoni S.; Sartori M.; Amunni F.; Tonelli P.	2013
A palatal roll envelope technique for peri-implant mucosa reconstruction: A prospective case series study	Man Y.; Wang Y.; Qu Y.; Wang P.; Gong P.	2013
Reconstruciton of bilateral maxillectomy defect	Joseph S.T.; Thankappan K.; Rahul B.; Shetty S.; Chandarasekhara Rao L.M.; Kuriakose M.A.; Iyer S.	2013
Zygoma Implant Reconstruction of Acquired Maxillary Bony Defects	Vega L.G.; Gielincki W.; Fernandes R.P.	2013
Impact of local and systemic factors on additional peri-implant bone loss	de Souza J.G.; Neto A.R.; Filho G.S.; Dalago H.R.; de Souza Júnior J.M.; Bianchini M.A.	2013
A novel standard of quality evaluation in bradyarrhythmia pacing	Brough C.E.P.; McGee C.; Rao A.; Wright D.J.	2013
Painful prosthesis: Approaching the patient with persistent pain following total hip and knee arthroplasty	Piscitelli P.; Iolascon G.; Innocenti M.; Civinini R.; Rubinacci A.; Muratore M.; D'Arienzo M.; Tranquilli Leali P.; Carossino A.M.; Brandi M.L.	2013
1st Global Conference on Contraception, Reproductive and Sexual Health 2013		2013
Safety of slow pathway ablation in patients with atrioventricular nodal reentry tachycardia and prolonged pr intervals	Turner J.W.; Doris J.M.; Rostomian A.; Gupta N.; Ji S.J.; Kangavari S.; Lee M.J.; Nageh M.F.; Wu J.; Brar S.S.	2013
3 years results of endovascular therapy with a new generation nitinol stent for femoro-popliteal artery lesions. Insights from a single-centre outcome analysis of a MISAGO2 subgroup	Boos I.; Kralj I.; Müller-Bühl U.	2013
Hybrid coronary revascularization: A case series	Duarte Rodrigues J.D.R.; Mariana Vasconcelos M.V.; Rui Rodrigues R.R.; Sergio Machado Leite S.M.L.; Mario Amorim M.A.; Manuel Campelo M.C.; Jose Pedro Nunes J.P.N.; Joao Carlos Silva J.C.S.; Paulo Pinho P.P.; Maria Julia Maciel M.J.M.	2013
Comparison of different designs of implant-retained overdentures and fixed full-arch implant-supported prosthesis on stress distribution in edentulous mandible - A computed tomography-based three-dimensional finite element analysis	Barão V.A.R.; Delben J.A.; Lima J.; Cabral T.; Assunção W.G.	2013
Receptor activator of nuclear factor kappa B (RANK) as a determinant of peri-implantitis	Rakic M.; Nikolic-Jakoba N.; Struillou X.; Petkovic-Curcin A.; Stamatović N.; Matić S.; Jankovic S.; Aleksic Z.; Vasilić D.; Lekovic V.; Vojvodic D.	2013
Dental phobia is no contraindication for oral implant therapy	Enkling N.; Hardt K.; Katsoulis J.; Ramseier C.A.; Colombo A.; Jöhren P.; Mericske-Stern R.	2013
Oral lichen planus and dental implants - A retrospective study	Czerninski R.; Eliezer M.; Wilensky A.; Soskolne A.	2013
The role of cements in dental lant success, Part I.	Wadhwani C.P.; Schwedhelm E.R.	2013
The care and maintenance of dental implants: a team approach.	Joyce M.	2013
Peri-implant bone density in senile osteoporosis-changes from implant placement to osseointegration	Beppu K.; Kido H.; Watazu A.; Teraoka K.; Matsuura M.	2013
An assessment of different crown-to-implant ratio for implant teeth in the posterior region over 5 years	Zhao B.; Feng W.; Wang D.; Jang L.; Deng C.; Shang D.; Zhang C.	2013
Implant failure associated with actinomycosis in a medically compromised patient.	Sun C.X.; Henkin J.M.; Ririe C.; Javadi E.	2013
Impact of immediate loading on early bone healing at two-piece implants placed in fresh extraction sockets: An experimental study in the beagle dog	Blanco J.; Mareque S.; Liñares A.; Pérez J.; Muñoz F.; Ramos I.	2013

Clinical outcome and bone healing of implants placed with high insertion torque: 12-month results from a multicenter controlled cohort study	Grandi T.; Guazzi P.; Samarani R.; Grandi G.	2013
Infection control and prevention practices for mechanical circulatory support: An international survey	Kusne S.; Danziger-Isakov L.; Mooney M.; Grossi P.; Husain S.; Pagani F.; Silveira F.P.; Hannan M.M.; Huprikar S.; Jaroszewski D.E.; Chang Y.-H.H.; Staley L.; Arabia F.	2013
A budget impact model for paclitaxel-eluting stent in femoropopliteal disease in France	De Cock E.; Sapoval M.; Julia P.; De Lissovoy G.; Lopes S.	2013
Allogeneic transfusion reduction with the use of tranexamic acid in total hip replacement	Serra N.; Barbero M.; Maric M.; Peral A.; Rey P.	2013
Simulation of peri-implant bone healing due to immediate loading in dental implant treatments	Chou H.-Y.; Müftü S.	2013
Differences in peri-implant conditions between fully and partially edentulous subjects: A systematic review	De Waal Y.C.M.; Van Winkelhoff A.J.; Meijer H.J.A.; Raghoebar G.M.; Winkel E.G.	2013
The use of porous titanium granules for treatment of peri-implantitis lesions: preliminary clinical and radiographic results in humans	Mijiritsky E.; Yatzkaier G.; Mazor Z.; Lorean A.; Levin L.	2013
Efficacy of triclosan-based toothpastes in the prevention and treatment of plaque-induced periodontal and peri-implant diseases	Trombelli L.; Farina R.	2013
Comparison of soft tissue healing around implants in beagle dogs: Flap surgery versus flapless surgery	Lei Q.; Chen J.; Jiang J.; Fu X.; Lin H.; Cai Z.	2013
Aetiology, microbiology and therapy of periapical lesions around oral implants: A retrospective analysis	Lefever D.; Van Assche N.; Temmerman A.; Teughels W.; Quirynen M.	2013
Early implant loading in the atrophic posterior maxilla: 1-stage lateral versus crestal sinus lift and 8 mm hydroxyapatite-coated implants. A 5-year randomised controlled trial.	Cannizzaro G.; Felice P.; Minciarelli A.F.; Leone M.; Viola P.; Esposito M.	2013
Influence of buccal bony crest width on marginal dimensions of peri-implant hard and soft tissues after implant installation. An experimental study in dogs	Baffone G.M.; Botticelli D.; Pereira F.P.; Favero G.; Schweikert M.; Lang N.P.	2013
Definitive abutment-driven stage-two surgery as a means to reduce peri-implant soft tissue changes: introduction of a new concept.	Pelekanos S.; Ntounis A.; Jovanovic S.A.; Euwe E.	2013
Immediate provisionalization of mini-implants with friction-engaging abutments in the mandibular anterior region: a 1-year retrospective study.	Choi K.S.; Yoon H.C.; Cho Y.S.	2013
Alterations in soft tissue levels and aesthetics over a 16-22year period following single implant treatment in periodontally-healthy patients: A retrospective case series	Dierens M.; De Bruecker E.; Vandeweghe S.; Kisch J.; De Bruyn H.; Cosyn J.	2013
Influence of presence or absence of teeth adjacent to implants installed immediately into extraction sockets on peri-implant hard tissue levels: An experimental study in the dog	Favero G.; Botticelli D.; Rea M.; Pantani F.; León I.G.; Lang N.P.	2013
Antimicrobial photodynamic therapy using visible light plus water-filtered infrared-A (wIRA)	Al-Ahmad A.; Tennert C.; Karygianni L.; Wrbas K.T.; Hellwig E.; Altenburger M.J.	2013
Maxillary overdentures supported by four or six implants in the anterior region; 1-year results from a randomized controlled trial	Slot W.; Raghoebar G.M.; Vissink A.; Meijer H.J.A.	2013
Comparative study of Chinese hamster ovary cell versus escherichia coli-derived bone morphogenetic protein-2 using the critical-size supraalveolar peri-implant defect model	Lee J.; Lee E.N.; Yoon J.; Chung S.-M.; Prasad H.; Susin C.; Wikesjö U.M.E.	2013
Effect of misfit of cement-retained implant single crowns on crestal bone changes.	Chen C.J.; Papaspyridakos P.; Guze K.; Singh M.; Weber H.P.; Gallucci G.O.	2013
The prognosis of partial implant-supported fixed dental prostheses with cantilevers. A 5-year retrospective cohort study.	Maló P.; de Araujo Nobre M.; Lopes A.	2013
Effect of platelet-rich plasma and platelet-rich fibrin on peri-implant bone defects in dogs	Jeong K.-I.; Kim S.-G.; Oh J.-S.; Lee S.-Y.; Cho Y.-S.; Yang S.-S.; Park S.-C.; You J.-S.; Lim S.-C.; Jeong M.-A.; Kim J.-S.; Lee S.-Y.	2013
Mechanical left ventricular support using a 50 cc 8 Fr fibre-optic intra-aortic balloon technology: A case report	Mulholland J.; Yarham G.; Clements A.; Morris C.; Loja D.	2013
Sacrocolpopexy (robotic assisted and laparoscopic): Is outcome better with fellowship trained urogynecologist?	Bagaria M.; Luck A.M.; Abed H.; Holmquist L.; Atiemo H.; Richardson D.; Rivers K.	2013
Experiences of young women living with developmental dysplasia of the hip: Insight into their experiences of surgery and recovery	Gambling T.S.; Long A.F.	2013
Associated factors with repeat coronary angioplasty during the drug eluting stent era: A high volume center investigation	Liu C.; Yan H.-B.; Zhao H.-J.; Song L.; Zheng B.; Chi Y.-P.; Wang S.-P.; Wang J.; Peng H.-Y.; Wu Z.	2013
A comparative analysis of periimplant bone levels of immediate and conventionally loaded implants	Guruprasada M.; Thapliyal G.K.; Pawar V.R.	2013
Reimplantation of Dental Implants following Ligature-Induced Peri-Implantitis: A Pilot Study in Dogs	Levin L.; Zigdon H.; Coelho P.G.; Suzuki M.; Machtei E.E.	2013
Implant decontamination during surgical peri-implantitis treatment: A randomized, double-blind, placebo-controlled trial	De Waal Y.C.M.; Raghoebar G.M.; Huddleston Slater J.J.R.; Meijer H.J.A.; Winkel E.G.; Van Winkelhoff A.J.	2013
Three-years clinical outcome of immediate provisionalization of single Osseospeed™ implants in extraction sockets and healed ridges	De Bruyn H.; Raes F.; Cooper L.F.; Reside G.; Garriga J.S.; Tarrida L.G.; Wiltfang J.; Kern M.	2013
A single-tooth, two-piece zirconia implant located in the anterior maxilla: A clinical report	Aydin C.; Yilmaz H.; Bankoğlu M.	2013
Long-Term Clinical, Microbiological, and Radiographic Outcomes of Brånemark™ Implants Installed in Augmented Maxillary Bone for Fixed Full-Arch Rehabilitation	De Bruyn H.; Bouvry P.; Collaert B.; De Clercq C.; Persson G.R.; Cosyn J.	2013
Acute Inflammatory Response to Laser-Induced Micro- and Nano-Sized Titanium Surface Features	Palmquist A.; Johansson A.; Suska F.; Brånemark R.; Thomsen P.	2013

Radiological evaluation of single implants in maxillary anterior sites with special emphasis on their relation to adjacent teeth - a 3-year follow-up study	Vilhjálmsson V.H.; Klock K.S.; Størksen K.; Bårdsen A.	2013
Clinical outcomes in the presence and absence of keratinized mucosa in mandibular guided implant surgeries: a pilot study with a proposal for the modification of the technique	Maló P.; Rigolizzo M.; Nobre Md.; Lopes A.; Agliardi E.	2013
Case of severe bone atrophy of the posterior maxilla rehabilitated with blocks of equine origin bone: Histological results	Pistilli R.; Signorini L.; Pisacane A.; Lizio G.; Felice P.	2013
Innate immune receptor expression in peri-implant tissues of patients with different susceptibility to periodontal diseases	Koutouzis T.; Catania D.; Neiva K.; Wallet S.M.	2013
Long-term outcomes of short dental implants supporting single crowns in posterior region: A clinical retrospective study of 5-10 years	Lai H.-C.; Si M.-S.; Zhuang L.-F.; Shen H.; Liu Y.-L.; Wismeijer D.	2013
A 5-year retrospective study on postsurgical periimplant infection during initial bone healing period: Clinical characteristics, management, and prognosis	Ma W.; Duan Y.; Sun B.; Liu Y.; Xie C.; Li D.	2013
Less invasive polyaxial locking plate fixation in periprosthetic and peri-implant fractures of the femur - A prospective study of 41 patients	Ruchholtz S.; El-Zayat B.; Kreslo D.; Bücking B.; Lewan U.; Krüger A.; Zettl R.	2013
High bone-implant contact achieved by photofunctionalization to reduce periimplant stress: A three-dimensional finite element analysis	Ohyama T.; Uchida T.; Shibuya N.; Nakabayashi S.; Ishigami T.; Ogawa T.	2013
Metastatic colon cancer, version 3.2013: Featured updates to the NCCN guidelines	Benson III A.B.; Bekaii-Saab T.; Chan E.; Chen Y.-J.; Choti M.A.; Cooper H.S.; Engstrom P.F.; Enzinger P.C.; Fakih M.G.; Fenton M.J.; Fuchs C.S.; Grem J.L.; Hunt S.; Kamel A.; Leong L.A.; Lin E.; May K.S.; Mulcahy M.F.; Murphy K.; Rohren E.; Ryan D.P.; Saltz L.; Sharma S.; Shibata D.; Skibber J.M.; Jr W.S.; Sofocleous C.T.; Venook A.P.; Willett C.G.; Gregory K.M.; Freedman-Cass D.A.	2013
RIRS versus mPCNL for single renal stone of 2-3 cm: Clinical outcome and cost-effective analysis in Chinese medical setting	Pan J.; Chen Q.; Xue W.; Chen Y.; Xia L.; Chen H.; Huang Y.	2013
Prevalence and risk factors for peri-implant disease in Belgian adults	Marrone A.; Lasserre J.; Bercy P.; Brecx M.C.	2013
The peri-implant health in patients attending an annual recall program: A clinical and microbiological study in 74 patients from the Tübingen Implant Registry	Lachmann S.; Stehberger A.; Axmann D.; Weber H.	2013
Probing at implants with peri-implantitis and its relation to clinical peri-implant bone loss	Serino G.; Turri A.; Lang N.P.	2013
Long-term results of implant-supported over-dentures retained by double crowns: A practice-based retrospective study after minimally 10 years follow-up	Frisch E.; Ziebolz D.; Rinke S.	2013
The effect of keratinized mucosa width on peri-implant health: a systematic review	Gobbato L.; Avila-Ortiz G.; Sohrabi K.; Wang C.-W.; Karimbux N.	2013
Long-term effect of overdenture bar design on peri-implant tissues	Lehmann K.M.; Kämmerer P.W.; Karbach J.; Scheller H.; Al-Nawas B.; Wagner W.	2013
The frequency of peri-implant diseases: A systematic review and meta-Analysis	Atieh M.A.; Alsabeeha N.H.M.; Faggion Jr. C.M.; Duncan W.J.	2013
The significance of keratinized mucosa on implant health: A systematic review	Lin G.-H.; Chan H.-L.; Wang H.-L.	2013
Peri-implantitis progression around thin sputtered hydroxyapatite-coated implants: clinical and radiographic evaluation in dogs	Madi M.; Zakaria O.; Noritake K.; Fuji M.; Kasugai S.	2013
Treatment outcome in patients with peri-implantitis in a periodontal clinic: A retrospective study	Lagervall M.; Jansson L.E.	2013
Exfoliative cytology and titanium dental implants: A pilot study	Olmedo D.G.; Nalli G.; Verdú S.; Paparella M.L.; Cabrini R.L.	2013
Inflammatory implant periapical lesion prior to osseointegration: a case series study	Penarrocha-Diago M.; Maestre-Ferrín L.; Penarrocha-Oltra D.; Canullo L.; Piattelli A.; Penarrocha-Diago M.	2013
Clinical evaluation of marginal bone loss and stability in two types of submerged dental implants	Gultekin B.A.; Gultekin P.; Leblebicioglu B.; Basegmez C.; Yalcin S.	2013
Implant-supported single tooth restoration in the aesthetic zone: Transmucosal and submerged healing provide similar outcome when simultaneous bone augmentation is needed	Cordaro L.; Torsello F.; Chen S.; Ganeles J.; Brägger U.; Hämmerle C.	2013
Clinical performance and peri-implant parameters of 132 implants supporting locator-retained overdentures: a case series of 33 patients	Troeltzsch M.; Troeltzsch V.; Brodine A.H.; Frankenberger R.; Messlinger K.; Troeltzsch M.	2013
Treatment with implant-supported fixed dental prostheses in patients with congenital and acquired neurologic disabilities: a prospective study	Ekfeldt A.; Zellmer M.; Carlsson G.E.	2013
Treatment of peri-implantitis around TiUnite-surface implants using Er:YAG laser microexplosions.	Yamamoto A.; Tanabe T.	2013
Significance of keratinized mucosa around dental implants: A prospective comparative study	Boynueğri D.; Nemli S.K.; Kasko Y.A.	2013
Long-term evaluation of Astra Tech and Brånemark implants in patients treated with full-arch bridges. Results after 12-15 years	Ravald N.; Dahlgren S.; Teiwik A.; Gröndahl K.	2013
Impact of implant surface topography: A clinical study with a mean functional loading time of 85 months	Arnhart C.; Dvorak G.; Trefil C.; Huber C.; Watzek G.; Zechner W.	2013
A cross-sectional study on the prevalence of marginal bone loss among implant patients	Cecchinato D.; Parpaiola A.; Lindhe J.	2013
Inpatient evaluation of periodontal, esthetic and inflammatory parameters around dental implants and natural teeth	Güncü G.N.; Büyüktepe G.; Aşkin S.B.; Kilinç K.; Tümer C.; Tözüm T.F.	2013
Combination Therapy Including Serratiopeptidase Improves Outcomes of Mechanical-Antibiotic Treatment of Periimplantitis	Sannino G.; Gigola P.; Puttini M.; Pera F.; Passariello C.	2013

Performance of the Straumann Bone Level Implant system for anterior single-tooth replacements in augmented and nonaugmented sites: A prospective cohort study with 60 consecutive patients	Santing H.J.; Raghoebar G.M.; Vissink A.; Den Hartog L.; Meijer H.J.A.	2013
Palatal positioning of implants in severely atrophic edentulous maxillae: five-year cross-sectional retrospective follow-up study	Peñarrocha-Oltra D.; Candel-Martí E.; Peñarrocha-Diago M.; Martínez-González J.M.; Aragoneses J.M.; Peñarrocha-Diago M.	2013
The effect of glycine-powder airflow and hand instrumentation on peri-implant soft tissues: a split-mouth pilot study.	Mussano F.; Rovasio S.; Schierano G.; Baldi I.; Carossa S.	2013
Accuracy of enhancement filters in measuring in vitro peri-implant bone level	de Azevedo Vaz S.L.; Neves F.S.; Figueirêdo E.P.; Haiter-Neto F.; Campos P.S.F.	2013
A prospective, controlled clinical trial evaluating the clinical radiological and aesthetic outcome after 5 years of immediately placed implants in sockets exhibiting periapical pathology	Jung R.E.; Zaugg B.; Philipp A.O.H.; Truninger T.C.; Siegenthaler D.W.; Hämmerle C.H.F.	2013
Comparison of the effect of two interdental cleaning devices around implants on the reduction of bleeding: a 30-day randomized clinical trial.	Magnuson B.; Harsono M.; Stark P.C.; Lyle D.; Kugel G.; Perry R.	2013
Immediate implant placement without bone grafting: A retrospective study of 110 cases with 5 years of follow-up	Atalay B.; Öncü B.; Emes Y.; Bultan O.; Aybar B.; Yalçn S.	2013
The team approach to managing dental implant complications: strategies for treating peri-implantitis.	Rosen P.S.	2013
[Prophylaxis and treatment of periimpalntitis].	Mandzhavidze N.; Vadachkoriia N.; Gumberidze N.	2013
Induction of periimplantitis in dental implants	Becker S.T.; Föge M.; Beck-Broichsitter B.E.; Gavrilova O.; Bolte H.; Rosenstiel P.; Wiltfang J.	2013
Long-term stability of contour augmentation with early implant placement following single tooth extraction in the esthetic zone: A prospective, cross-sectional study in 41 patients with a 5-to 9-year follow-up	Buser D.; Chappuis V.; Bornstein M.M.; Wittneben J.-G.; Frei M.; Belser U.C.	2013
Patient satisfaction and aesthetic outcome of implant-supported single-tooth replacements performed by dental students: a retrospective evaluation 8 to 12 years after treatment.	Bonde M.J.; Stokholm R.; Schou S.; Isidor F.	2013
Longevity of implant crowns and 2-unit cantilevered resin-bonded bridges	Lam W.Y.H.; Botelho M.G.; Mcgrath C.P.J.	2013
[Dental implants: a solution for lost dental elements].	Raghoebar G.M.; Meijer H.J.; Cune M.S.; Vissink A.	2013
Posterior atrophic jaws rehabilitated with prostheses supported by 6 mm long 4 mm wide implants or by longer implants in augmented bone. One-year post-loading results from a pilot randomised controlled trial.	Pistilli R.; Felice P.; Cannizzaro G.; Piatelli M.; Corvino V.; Barausse C.; Buti J.; Soardi E.; Esposito M.	2013
Decontamination of dental implant surfaces by means of photodynamic therapy	Marotti J.; Tortamano P.; Cai S.; Ribeiro M.S.; Franco J.E.M.; De Campos T.T.	2013
Short implants with a nanometer-sized CaP surface provided with either a platform-switched or platform-matched abutment connection in the posterior region: A randomized clinical trial	Telleman G.; Meijer H.J.A.; Vissink A.; Raghoebar G.M.	2013
Posterior atrophic jaws rehabilitated with prostheses supported by 5 x 5 mm implants with a novel nanostructured calcium-incorporated titanium surface or by longer implants in augmented bone. One-year results from a randomised controlled trial.	Pistilli R.; Felice P.; Piattelli M.; Gessaroli M.; Soardi E.; Barausse C.; Buti J.; Corvino V.	2013
Piezoelectric alveolar ridge-splitting technique with simultaneous implant placement: a cohort study with 2-year radiographic results	Bassetti R.; Bassetti M.; Mericske-Stern R.; Enkling N.	2013
The All-on-Four implant therapy protocol in the management of edentulous Chinese patients	Di P.; Lin Y.; Li J.-H.; Luo J.; Qiu L.; Chen B.; Cui H.	2013
Behavior of endosseous one-piece yttrium stabilized zirconia dental implants placed in posterior areas	Borgonovo A.E.; Vavassori V.; Censi R.; Calvo J.L.; Re D.	2013
Immediate provisionalization of mini-implants with friction-engaging abutments in the mandibular anterior region: a 1-year retrospective study	Choi K.; Yoon H.; Cho Y.	2013
A 3-year multicenter study of marginal bone level and soft tissue health of a 1-piece implant	Baer R.A.; Shanaman R.; Clark P.K.; Medley M.	2013
Clinical and radiographic outcome of implants placed using stereolithographic guided surgery: a prospective monocenter study	D'haese J.; Vervaeke S.; Verbanck N.; De Bruyn H.	2013
Clinical, radiographic, microbiological, and immunological outcomes of flapped vs. flapless dental implants: A prospective randomized controlled clinical trial	Tsoukaki M.; Kalpidis C.D.R.; Sakellari D.; Tsalikis L.; Mikrogiorgis G.; Konstantinidis A.	2013
Long-term results after immediate loading of platform-switched implants in smokers versus nonsmokers with full-arch restorations	Romanos G.E.; Gaertner K.; Aydin E.; Nentwig G.-H.	2013
The effect of healing abutment reconnection and disconnection on soft and hard peri-implant tissues: a short-term randomized controlled clinical trial	Koutouzis T.; Koutouzis G.; Gadalla H.; Neiva R.	2013
Placement of implants with platform-switched Morse taper connections with the implant-abutment interface at different levels in relation to the alveolar crest: a short-term (1-year) randomized prospective controlled clinical trial	Koutouzis T.; Neiva R.; Nonhoff J.; Lundgren T.	2013
Peri-implant and periodontal tissues: a review of differences and similarities.	Dhir S.; Mahesh L.; Kurtzman G.M.; Vandana K.L.	2013
Preoperative gingival conditioning as a guide for implant installation and to achieve proper dentogingival contours	de Jesus Tavarez R.R.; Malheiros A.S.; Benatti B.B.; Tonetto M.R.; Bandéca M.C.	2013
The effect of platform switching on stress in peri-implant bone in a condition of marginal bone resorption: a three-dimensional finite element analysis	Xia H.; Wang M.; Ma L.; Zhou Y.; Li Z.; Wang Y.	2013
How to manage a patient with peri-implantitis	Hsu A.; Kim J.-W.M.	2013

Iliac crest fresh-frozen allografts and autografts in maxillary and mandibular reconstruction: A histologic and histomorphometric evaluation	Chiapasco M.; Giammattei M.; Carmagnola D.; Autelitano L.; Rabbiosi D.; Dellavia C.	2013
Containment and contouring (CoCoon) technique: A biologically adequate approach to less invasive autogenous preimplant augmentation of bone	Gellrich N.-C.; Bormann K.-H.; Tehranchian S.; Kokemüller H.; Suarez-Cunqueiro M.M.	2013
The bony lid approach in pre-implant and implant surgery: a prospective study.	Khoury F.	2013
Clinical outcome and peri-implant findings of four-implant-supported distal cantilevered fixed mandibular prostheses: five-year results	Krennmair G.; Seemann R.; Weinländer M.; Krennmair S.; Piehslinger E.	2013
The effect of implant surface modifications on survival and bone loss of immediately loaded implants in the edentulous mandible	Vervaeke S.; Collaert B.; De Bruyn H.	2013
Influence of transmucosal designs of dental implant on tissue regeneration in beagle dogs	Kim W.-H.; Heo Y.-K.; Jeong C.-M.; Cho D.-W.; Ryu J.-J.; Huh J.-B.	2013
Immediate loading of implants in the maxilla: survival and bone loss after at least 2 years in function.	Vervaeke S.; Collaert B.; De Bruyn H.	2013
Dental management of the female patient	Otomo-Corgel J.	2013
Aesthetic analysis of an implant-supported denture at the cleft area	Filho J.F.F.; De Almeida A.L.P.F.	2013
Closure of 1.5-cm alveolar oral antral fistula with intra-alveolar sinus membrane elevation and bone morphogenetic protein-2/collagen graft followed by dental implant restoration: case report	Cottam J.R.; Jensen O.T.; Beatty L.; Ringeman J.	2013
A randomized controlled clinical trial of two types of tapered implants on immediate loading in the posterior maxilla and mandible	Kim Y.-K.; Lee J.-H.; Lee J.-Y.; Yi Y.-J.	2013
Retrospective results of implants for partially edentulous posterior jaws according to time points of early loading	Kim J.-H.; Yang J.-Y.; Kim Y.-K.; Heo Y.-K.; Yeo I.-S.	2013
Early-loaded one-stage implants retaining mandibular overdentures by two different mechanisms: 5-year results	Akça K.; Çavuşoğlu Y.; Sağirkaya E.; Çehreli M.C.	2013
A prospective, open-ended, single-cohort clinical trial on early loaded Titanium-zirconia alloy implants in partially edentulous patients: up-to-24-month results	Akca K.; Cavusoglu Y.; Uysal S.; Cehreli M.C.	2013
Immediate versus conventional loading for the maxilla with implants placed into fresh and healed extraction sites to support a full-arch fixed prosthesis: nonrandomized controlled clinical study	Peñarrocha-Oltra D.; Covani U.; Aparicio A.; Ata-Ali J.; Peñarrocha-Diago M.; Peñarrocha-Diago M.	2013
Miniscrew design and bone response: defining a correlation	Bhalla K.; Kalha A.S.	2013
A 5- to 8-year retrospective study comparing the clinical results of implant-supported telescopic crown versus bar overdentures in patients with edentulous maxillae	Zou D.; Wu Y.; Huang W.; Zhang Z.; Zhang Z.	2013
Complete regeneration of peri-implantitisinduced bony defects using guided bone regeneration is unpredictable	Elangovan S.	2013
A 3-year prospective clinical study of telescopic crown, bar, and locator attachments for removable four implant-supported maxillary overdentures	Zou D.; Wu Y.; Huang W.; Wang F.; Wang S.; Zhang Z.; Zhang Z.	2013
Alterations in bone quality after socket preservation with grafting materials: a systematic review	Chan H.-L.; Lin G.-H.; Fu J.-H.; Wang H.-L.	2013
Prosthetic soft tissue management following two periimplant graft failures: A clinical report	Issarayangkul C.; Schoenbaum T.R.; McLaren E.A.	2013
Biomechanical effect of one session of low-level laser on the bone-titanium implant interface	Boldrini C.; De Almeida J.M.; Fernandes L.A.; Ribeiro F.S.; Garcia V.G.; Theodoro L.H.; Pontes A.E.F.	2013
Comparison of plaque accumulation and soft-tissue blood flow with the use of full-arch implant-supported fixed prostheses with mucosal surfaces of different materials: A randomized clinical study	Kanao M.; Nakamoto T.; Kajiwara N.; Kondo Y.; Masaki C.; Hosokawa R.	2013
Comparative clinical study of three-unit fixed partial prostheses supported by two or three implants	Yi Y.-J.; Lee J.-Y.; Kim Y.-K.	2013
PTH [1-34] enhances bone response around titanium implants in a rabbit model of osteoporosis	Almagro M.I.; Roman-Blas J.A.; Bellido M.; Castañeda S.; Cortez R.; Herrero-Beaumont G.	2013
Correction of buccal dehiscence at the time of implant placement without barrier membranes: a retrospective cone beam computed tomographic study	Cortes A.R.; Cortes D.N.; Arita E.S.	2013
Three-month cumulative incidence of thromboembolism and bleeding after periprocedural anticoagulation management of arterial vascular bypass patients	Attaya H.; Wysokinski W.E.; Bower T.; Litin S.; Daniels P.R.; Slusser J.; Heit J.A.; Mcbane R.D.	2013
What are the risks of prophylactic pinning to prevent contralateral slipped capital femoral epiphysis? Hip	Sankar W.N.; Novais E.N.; Lee C.; Al-Omari A.A.; Choi P.D.; Shore B.J.	2013
Risk factors for infection following total joint arthroplasty in rheumatoid arthritis	Somayaji R.; Barnabe C.; Martin L.	2013
Influence of prophylactic antibiotics on tissue integration versus bacterial colonization on poly(methyl methacrylate)	Subbiahdoss G.; Aleyt T.; Kuijer R.; Busscher H.J.; van der Mei H.C.	2012
Influence of surgical experience in the learning curve of a new approach in hip replacement: Anterior mini-invasive vs. standard lateral	Pogliacomi F.; Paraskevopoulos A.; Costantino C.; Marenghi P.; Ceccarelli F.	2012
The incidence of peri-implantitis for two different implant systems over a period of thirteen years	Renvert S.; Lindahl C.; Rutger Persson G.	2012
Peri-implantitis	Algraffee H.; Borumandi F.; Cascarini L.	2012
Crestal bone loss and oral implants	Albrektsson T.; Buser D.; Sennerby L.	2012

Reasons for marginal bone loss around oral implants	Qian J.; Wennerberg A.; Albrektsson T.	2012
Dental implantation: An opportunity to stop smoking	Kadkhodazadeh M.; Amid R.; Mollaverdi F.; Amirbandeh O.	2012
Prospective 10-year cohort study based on a randomized controlled trial (RCT) on implant-supported full-arch maxillary prostheses. Part 1: sandblasted and acid-etched implants and mucosal tissue	Fischer K.; Stenberg T.	2012
Accuracy of cone-beam computed tomography to assess the configuration and extent of ligature-induced peri-implantitis defects. A pilot study	Golubovic V.; Mihatovic I.; Becker J.; Schwarz F.	2012
Peri-implantitis prevalence and treatment in implant-oriented private practices: a cross-sectional postal and Internet survey.	Schmidlin P.R.; Sahrmann P.; Ramel C.; Imfeld T.; Müller J.; Roos M.; Jung R.E.	2012
Squamous cell carcinoma in association with dental implants: an assessment of previously hypothesized carcinogenic mechanisms and a case report.	Bhatavadekar N.B.	2012
10-year survival and success rates of 511 titanium implants with a sandblasted and acid-etched surface: a retrospective study in 303 partially edentulous patients	Buser D.; Janner S.F.; Wittneben J.-G.; Brägger U.; Ramseier C.A.; Salvi G.E.	2012
Immediate implant placement in molar regions: A 12-month prospective, randomized follow-up study	Urban T.; Kostopoulos L.; Wenzel A.	2012
Evaluation of soft tissues around single tooth implants in the anterior maxilla restored with cemented and screw-retained crowns.	Cutrim E.S.; Peruzzo D.C.; Benatti B.	2012
Ten years later. Results from a prospective single-centre clinical study on 121 oxidized (TiUnite™) Brånemark implants in 46 patients	Östman P.-O.; Hellman M.; Sennerby L.	2012
Cemented versus screw-retained implant-supported single-tooth crowns: a 10-year randomised controlled trial.	Vigolo P.; Mutinelli S.; Givani A.; Stellini E.	2012
A double-blind randomized controlled trial (RCT) of Titanium-13Zirconium versus Titanium Grade IV small-diameter bone level implants in edentulous mandibles--results from a 1-year observation period	Al-Nawas B.; Brägger U.; Meijer H.J.; Naert I.; Persson R.; Perucchi A.; Quirynen M.; Raghoebar G.M.; Reichert T.E.; Romeo E.; Santing H.J.; Schimmel M.; Storelli S.; ten Bruggenkate C.; Vandekerckhove B.; Wagner W.; Wismeijer D.; Müller F.	2012
Microbial colonization in orthodontic mini-implants	de Freitas A.O.A.; Alviano C.S.; Alviano D.S.; Siqueira Jr. J.F.; Nojima L.I.; Nojima M.C.G.	2012
10-year follow-up of immediately loaded implants with TiUnite porous anodized surface	Degidi M.; Nardi D.; Piattelli A.	2012
The experience of pain and patient satisfaction with den tal implant-prosthetic treatment	Szymańska J.; Koszuta A.	2012
Early peri-implant endosseous healing of two implant surfaces placed in surgically created circumferential defects. A histomorphometric and fluorescence study in dogs	de Barros R.R.M.; Novaes A.B.; Queiroz A.; de Almeida A.L.G.	2012
Immediate loading of single implants inserted flapless with medium or high insertion torque: a 6-month follow-up of a split-mouth randomised controlled trial.	Cannizzaro G.; Leone M.; Ferri V.; Viola P.; Federico G.; Esposito M.	2012
Ultrasound imagery for dental implant diagnosis and treatment planning in a porcine model	Choi M.; Culjat M.O.; Singh R.S.; White S.N.	2012
[Use of erbium laser for treatment of dental implant complications].	Kulakov A.A.; Khamraev T.K.; Kasparov A.S.; Amirov A.R.	2012
The experience of pain and patient satisfaction with dental implant-prosthetic treatment	Szymańska J.; Koszuta A.	2012
Osseointegrated dental implants in patients with intellectual disability: a pilot study.	Feijoo J.F.; Limeres J.; Diniz M.; Del Llano A.; Seoane J.; Diz P.	2012
Outcome of the HORIZONS-AMI trial: Bivalirudin enhances long-term survival in patients with ST-elevation myocardial infarction undergoing angioplasty	Shah A.; Feldman D.N.	2012
Incidence and multivariable correlates of long-term mortality in patients treated with surgical or percutaneous revascularization in the Synergy between Percutaneous Coronary Intervention with Taxus and Cardiac Surgery (SYNTAX) trial	Farooq V.; Serruys P.W.; Bourantas C.; Vranckx P.; Diletti R.; Garcia Garcia H.M.; Holmes D.R.; Kappetein A.-P.; MacK M.; Feldman T.; Morice M.C.; Colombo A.; Morel M.-A.; De Vries T.; Van Es G.A.; Steyerberg E.W.; Dawkins K.D.; Mohr F.W.; James S.; Ståhle E.	2012
Transvenous implantation of the melody valve for bioprosthetic mitral and tricuspid valve dysfunction: A case series	Cullen M.W.; Cabalka A.K.; Alli O.O.; Pislaru S.V.; Sorajja P.; Nkomo V.T.; Cetta F.; Hagler D.J.; Rihal C.S.	2012
Six and twelve-month clinical and imaging results of the multicenter first-in-man biosolve-i study with the paclitaxel-eluting bioabsorbable magnesium scaffold (DREAMS)	Haude M.; Erbel R.; Erne P.; Verheye S.; Vermeersch P.; Degen H.; Waksman R.; Weissman N.; Prati F.; Koolen J.	2012
The management of retrograde peri-implantitis: A case report	Mohamed J.B.; Alam M.N.; Singh G.; Chandrasekaran S.C.	2012
Multiple teeth replacement with endosseous one-piece yttrium-stabilized zirconia dental implants	Borgonovo A.-E.; Fabbri A.; Vavassori V.; Censi R.; Maiorana C.	2012
Two-year follow-up of early- and conventionally-placed two-stage implants supporting fixed prostheses.	Bekcioglu B.; Sagirkaya E.; Karasoy D.; Cehreli M.	2012
Peri-implant marginal bone level: A systematic review and meta-analysis of studies comparing platform switching versus conventionally restored implants	Annibali S.; Bignozzi I.; Cristalli M.P.; Graziani F.; La Monaca G.; Polimeni A.	2012
Radiographic evaluation of marginal bone maintenance around tissue level implant and bone level implant: A randomised controlled trial. A 1-year follow-up	Fernández-Formoso N.; Rilo B.; Mora M.J.; Martínez-Silva I.; Díaz-Afonso A.M.	2012
Factors associated with early and late failure of dental implants.	Sakka S.; Baroudi K.; Nassani M.Z.	2012

Bone density and miniscrew stability in orthodontic patients.	Samrit V.; Kharbanda O.P.; Duggal R.; Seith A.; Malhotra V.	2012
Patient and implant survival following 4323 total hip replacements for acute femoral neck fracture: A retrospective cohort study using national joint registry data	Jameson S.S.; Kyle J.; Baker P.N.; Mason J.; Deehan D.J.; McMurtry I.A.; Reed M.R.	2012
Comparison of cardiac surgery with left atrial surgical ablation vs. cardiac surgery without atrial ablation in patients with coronary and/or valvular heart disease plus atrial fibrillation: Final results of the PRAGUE-12 randomized multicentre study	Budera P.; Straka Z.; Osmančík P.; Vaněk T.; Jelínek Š.; Hlavička J.; Fojt R.; Červinka P.; Hulman M.; Šmíd M.; Mal M.; Widimsk P.	2012
Prediction of arrhythmic events in ischemic and dilated cardiomyopathy patients referred for implantable cardiac defibrillator evaluation of multiple scar quantification measures for late gadolinium enhancement magnetic resonance imaging	Gao P.; Yee R.; Gula L.; Krahn A.D.; Skanes A.; Leong-Sit P.; Klein G.J.; Stirrat J.; Fine N.; Pallaveshi L.; Wisenberg G.; Thompson T.R.; Prato F.; Drangova M.; White J.A.	2012
Is there a need for keratinized mucosa around implants to maintain health and tissue stability?	Wennström J.L.; Derks J.	2012
A proposed classification for peri-implantitis.	Froum S.J.; Rosen P.S.	2012
Mucositis, peri-implantitis, implant success, and survival of implants in patients with treated generalized aggressive periodontitis: 3- to 16-year results of a prospective long-term cohort study	Swierkot K.; Lottholz P.; Flores-De-Jacoby L.; Mengel R.	2012
Tenascin-C and Matrix Metalloproteinase-9 Levels in Crevicular Fluid of Teeth and Implants	Özçakir-Tomruk C.; Chiquet M.; Mericske-Stern R.	2012
Gene Expression of Inflammation and Bone Healing in Peri-Implant Crevicular Fluid after Placement and Loading of Dental Implants. A Kinetic Clinical Pilot Study Using Quantitative Real-Time PCR	Slotte C.; Lennerås M.; Göthberg C.; Suska F.; Zoric N.; Thomsen P.; Nannmark U.	2012
Influence of etiologic factors in peri-implantitis: literature review and case report.	Cortes A.R.; Ferraz P.; Tosta M.	2012
Occlusal overload and bone/implant loss	Naert I.; Duyck J.; Vandamme K.	2012
Peri-implant tissue destruction. The Third EAO Consensus Conference 2012	Klinge B.; Meyle J.; Claffey N.; Flemmig T.; Flemming I.; Mombelli A.; Naert I.; Renvert S.; Rocchietta I.; Schou S.; Schwarz F.; Teughels W.; Valentini P.; Wennerberg A.	2012
Biological complications and peri-implant clinical and radiographic changes at immediately placed dental implants. A prospective 5-year cohort study	Rodrigo D.; Martin C.; Sanz M.	2012
Bone Level Changes Around Axial and Tilted Implants in Full-Arch Fixed Immediate Restorations. Interim Results of a Prospective Study	Francetti L.; Romeo D.; Corbella S.; Taschieri S.; Del Fabbro M.	2012
Relationship between smoking and bleeding on probing.	de Souza J.G.; Bianchini M.A.; Ferreira C.F.	2012
Soft tissue response to zirconia and titanium implant abutments: An in vivo within-subject comparison	Van Brakel R.; Meijer G.J.; Verhoeven J.W.; Jansen J.; De Putter C.; Cune M.S.	2012
Titanium-zirconium alloy narrow-diameter implants (Straumann Roxolid®) for the rehabilitation of horizontally deficient edentulous ridges: Prospective study on 18 consecutive patients	Chiapasco M.; Casentini P.; Zaniboni M.; Corsi E.; Anello T.	2012
Immediate rehabilitation of the edentulous mandible with screw type implants: Results after up to 10 years of clinical function	Heschl A.; Payer M.; Platzer S.; Wegscheider W.; Pertl C.; Lorenzoni M.	2012
Are marginal bone levels and implant stability/mobility affected by single-stage platform switched dental implants? A comparative clinical study	Dursun E.; Tulunoglu I.; Canpinar P.; Uysal S.; Akalin F.A.; Tözüm T.F.	2012
A systematic review of the survival and complication rates of implant-supported fixed dental prostheses (FDPs) after a mean observation period of at least 5 years	Pjetursson B.E.; Thoma D.; Jung R.; Zwahlen M.; Zembic A.	2012
What is the optimal number of implants for removable reconstructions? A systematic review on implant-supported overdentures	Roccuzzo M.; Bonino F.; Gaudioso L.; Zwahlen M.; Meijer H.J.A.	2012
Metal concentrations in patients with failed metal-on-metal hip prostheses determine the inflammatory phenotype in peri-implant tissue	Paukkeri E.-L.; Korhonen R.; Eskelinen A.; Pesu M.; Vasama K.; Moilanen T.; Moilanen E.	2012
Optimizing anterior esthetics of a single-tooth implant through socket augmentation and immediate provisionalization: A case report with 7-year follow-up	Fu P.-S.; Wu Y.-M.; Wang J.-C.; Huang T.-K.; Chen W.-C.; Huang J.-W.; Hung C.-C.	2012
The effect of implant size 5 × 8 mm on crestal bone levels around single-tooth implants	Urdaneta R.A.; Leary J.; Lubelski W.; Emanuel K.M.; Chuang S.-K.	2012
Early implant bone loss: Preventable or inevitable?	Tatarakis N.; Bashutski J.; Wang H.-L.; Oh T.-J.	2012
A single center experience of stenting in symptomatic intracranial atherosclerosis	Castaño C.; García-Bermejo P.; García M.R.	2012
Obstructive jaundice as presenting symptom of small cell lung cancer	Niazi M.; De Las Casas L.; Assaly R.; Youssef W.	2012
Modern approach to the identification and elimination of periodontal infection	Atanasovska-Stojanovska Dr. A.	2012
Therapy of peri-implant diseases. Where is the evidence?	Romanos G.E.; Weitz D.	2012
Correlation between clinical parameters characterising peri-implant and periodontal health: A practice-based research in Spain in a series of patients with implants installed 4-5 years ago	Lopez-Piriz R.; Morales A.; Giménez M.-J.; Bowen A.; Carroquino R.; Aguilar L.; Corral I.; del Val C.; González I.; Ilzarbe L.-M.; Maestre J.-R.; Padullés E.; Torres-Lear F.; Granizo J.-J.; San-Román F.; Hernández S.; Prieto J.	2012
Short (5 and 7 mm long) porous implants in the posterior atrophic maxilla: a 5-year report of a prospective single-cohort study.	Perelli M.; Abundo R.; Corrente G.; Saccone C.	2012
Decontamination of titanium implant surface and re-osseointegration to treat peri-implantitis: a literature review.	Subramani K.; Wismeijer D.	2012

Influence of placement depth on bone remodeling around tapered internal connection implant: A clinical and radiographic study in dogs	Huang B.; Meng H.; Piao M.; Xu L.; Zhang L.; Zhu W.	2012
Evaluation of 3- to 8-year treatment outcomes and success rates with 6 implant brands in partially edentulous patients.	Moeintaghavi A.; Radvar M.; Arab H.R.; Boostani H.R.; Ghiami E.	2012
Assessment of success for short dental implants (8mm) restored with single unit, non-splinted restorations	Le B.T.; Follmar T.; Borzabadi-Farahani A.	2012
Immediate loading of single post-extractive implants in the anterior maxilla: 12-month results from a multicenter clinical study.	Grandi T.; Garuti G.; Samarani R.; Guazzi P.; Forabosco A.	2012
Adverse effects of BMP2 on bone formation and osseointegration	Hyzy S.; Olivares-Navarrete R.; Boyan B.; Schwartz Z.	2012
Evaluation of peri-implant bone resorption around Straumann Bone Level© implants placed in areas reconstructed with autogenous vertical onlay bone grafts	Chiapasco M.; Casentini P.; Zaniboni M.; Corsi E.	2012
The effect of internal versus external abutment connection modes on crestal bone changes around dental implants: A radiographic analysis	Koo K.-T.; Lee E.-J.; Kim J.-Y.; Seol Y.-J.; Han J.S.; Kim T.-I.; Lee Y.M.; Ku Y.; Wikesjö U.M.E.; Rhyu I.-C.	2012
Reconstruction of the severely atrophic maxilla or acquired maxillary defects using zygomatic implants: Outcome Study of 41 implants	Vega L.G.; Brewer K.	2012
Safety and effectiveness of maxillary early loaded titanium implants with a novel nanostructured calcium-incorporated surface (Xpeed): 1-year results from a pilot multicenter randomised controlled trial.	Esposito M.; Grusovin M.G.; Pellegrino G.; Soardi E.; Felice P.	2012
Experimental custom-made zirconia abutments for narrow implants in esthetically demanding regions: a 5-year follow-up.	Vanlioglu B.A.; Özkan Y.; Evren B.; Özkan Y.K.	2012
A within-implant comparison to evaluate the concept of platform switching: a randomised controlled trial.	Vandeweghe S.; De Bruyn H.	2012
Long-term marginal bone loss in 217 machined-surface implants placed in 68 patients with 5 to 9 years of follow-up: a retrospective study.	Mir-Mari J.; Mir-Orfila P.; Valmaseda-Castellón E.; Gay-Escoda C.	2012
Influence of hyperbaric oxygen therapy on peri-implant bone healing in rats with alloxan-induced diabetes	Oliveira P.A.D.; Oliveira A.M.S.D.; Pablos A.B.; Costa F.O.; Silva G.A.B.; Santos J.N.D.; Cury P.R.	2012
Immediate rehabilitation of completely edentulous arches with a four-implant prosthesis concept in difficult conditions: an open cohort study with a mean follow-up of 2 years.	Malo P.; Nobre M.A.; Lopes A.	2012
Effect of the ridge preservation graft on posterior implant placement	Schreiber G.M.; Maxfield M.; Lam D.	2012
Short dental implants: Indications and outcomes	Boffano P.; Smurra P.; Gallesio C.	2012
Reliability of computer designed surgical guides in six implant rehabilitations with two years follow-up.	Giordano M.; Ausiello P.; Martorelli M.; Sorrentino R.	2012
Medium-term clinical outcomes following trocar-guided mesh repair of vaginal prolapse using partially absorbable mesh	Lucente V.; Hinoul P.; Gauld J.; Jones P.C.; Khandwala S.; Van Drie D.M.; Cosson M.; Milani A.L.	2012
Percutaneous ASD Closure in a Large Australian Series: Short- and Long-Term Outcomes	Walters D.L.; Boga T.; Burstow D.; Scalia G.; Hourigan L.A.; Aroney C.N.	2012
Endovascular repair of an inflammatory abdominal aortic aneurysm causing bilateral ureteric obstruction	McKenna A.J.; O'Donnell M.E.; Collins A.; Harkin D.W.	2012
Carotid stenting should be offered to symptomatic patients instead of endarterectomy: Con	Taylor P.R.; Clough R.E.; Mani K.	2012
Prevalence of pre-operative anemia in elective orthopedic surgery patients	Yazer M.H.; Barr T.M.; Chen A.F.; Waters J.H.; Silverman J.F.; Triulzi D.J.	2012
Repair of a chronic high sleeve gastrectomy leak by revision to gastric bypass	Kim K.; Krzyzanowski S.; Buffington C.K.	2012
Acute traumatic rupture of the thoracic aorta. A contemporary, expectant management	Fox W.C.; Komorowski D.J.; Morano G.S.; Sydnor M.K.; Tisnado J.; Tisnado J.; Brinster D.R.	2012
Prospective cohort study of robotic sacrocolpopexy using lightweight polypropylene y-mesh	Culligan P.; Gurshumov E.L.; Komar J.; Priestley J.; Lewis C.M.; Salamon C.	2012
Peri-implant marginal bone loss: an academic controversy or a clinical challenge?	Klinge B.	2012
Mechanical, chemical and laser treatments of the implant surface in the presence of marginal bone loss around implants.	Meyle J.	2012
The impact of the type and configuration of abutments and their (repeated) removal on the attachment level and marginal bone.	Rompen E.	2012
Identifying occlusal overload and how to deal with it to avoid marginal bone loss around implants.	Fu J.H.; Hsu Y.T.; Wang H.L.	2012
Surgical treatments of peri-implantitis.	Mombelli A.; Moëne R.; Décaillet F.	2012
Working Group on the Treatment Options for the Maintenance of Marginal Bone Around Endosseous Oral Implants, Stockholm, Sweden, 8 and 9 September 2011. Consensus statements.	Esposito M.; Klinge B.; Meyle J.; Mombelli A.; Rompen E.; van Steenberghe D.; Van Dyke T.; Wang H.L.; van Winkelhoff A.J.	2012
Clinical success of implant-supported and tooth-implant-supported double crown-retained dentures	Bernhart G.; Koob A.; Schmitter M.; Gabbert O.; Stober T.; Rammelsberg P.	2012
An introduction to the implant crown with an esthetic adhesive margin (ICEAM)	Wadhwani C.P.K.; Piñeyro A.; Akimoto K.	2012
Immediate loading of dental implants placed in severely resorbed edentulous maxillae reconstructed with le fort i osteotomy and interpositional bone grafting	Pieri F.; Lizio G.; Bianchi A.; Corinaldesi G.; Marchetti C.	2012
Early outcome of an implant system with a resorbable adhesive calcium-phosphate coating-a prospective clinical study in partially dentate patients	Palarie V.; Bicer C.; Lehmann K.M.; Zahalka M.; Draenert F.G.; Kämmerer P.W.	2012
Long-term outcomes of three types of implant-supported mandibular overdentures in smokers	Stoker G.; Van Waas R.; Wismeijer D.	2012

Splinting osseointegrated implants and natural teeth in partially edentulous patients: a systematic review of the literature.	Mamalis A.; Markopoulou K.; Kaloumenos K.; Analitis A.	2012
Treatment planning and sequence for implant therapy in a young adult with generalized aggressive periodontitis.	Bidra A.S.; Shaqman M.	2012
Clinical and radiographic outcome of dental implants supporting fixed prostheses: The relevance of cortical bone formation	Saulacic N.; Abboud M.; Pohl Y.; Wahl G.	2012
Retrospective clinical evaluation of tapered implants: 10-year follow-up of delayed and immediate placement of maxillary implants	Ormianer Z.; Piek D.; Livne S.; Lavi D.; Zafrir G.; Palti A.; Harel N.	2012
Protocol for immediate implant replacement of infected teeth	Jofre J.; Valenzuela D.; Quintana P.; Asenjo-Lobos C.	2012
Peri-implant bone changes following tooth extraction, immediate placement and loading of implants in the edentulous maxilla	Barbier L.; Abeloos J.; de Clercq C.; Jacobs R.	2012
Treatment planning of implants when 3 mandibular posterior teeth are missing: A 3-dimensional finite element analysis	Chen X.-Y.; Zhang C.-Y.; Nie E.-M.; Zhang M.-C.	2012
Multicentre prospective randomised study of single-incision midurethral sling (SIMS- Ajust©) versus tension-free vaginal tapeobturator (TVT-OTM) in management of female stress urinary incontinence (SUI): A minimum of one year follow-up	Mostafa A.; Agur W.; Abdel-All M.; Guerrero K.; Lim C.; Allam M.; Yousef M.; N'dow J.; Abdel-Fattah M.	2012
Impact of abutment material and dis-/re-connection on soft and hard tissue changes at implants with platform-switching	Becker K.; Mihatovic I.; Golubovic V.; Schwarz F.	2012
Hemodialysis Vascular Access Dysfunction: Molecular Mechanisms and Treatment	Mima A.	2012
Peri-implantitis susceptibility as it relates to periodontal therapy and supportive care	Pjetursson B.E.; Helbling C.; Weber H.-P.; Matuliene G.; Salvi G.E.; Brägger U.; Schmidlin K.; Zwahlen M.; Lang N.P.	2012
A single-centre randomized controlled clinical trial on the adjunct treatment of intra-bony defects with autogenous bone or a xenograft: Results after 12 months	Aghazadeh A.; Rutger Persson G.; Renvert S.	2012
The effect of various topical peri-implantitis antiseptics on Staphylococcus epidermidis, Candida albicans, and Streptococcus sanguinis	Bürgers R.; Witecy C.; Hahnel S.; Gosau M.	2012
Accuracy of cone beam computed tomography in assessing peri-implant bone defect regeneration: A histologically controlled study in dogs	Fienitz T.; Schwarz F.; Ritter L.; Dreiseidler T.; Becker J.; Rothamel D.	2012
A pilot study to evaluate the success and survival rate of titanium-zirconium implants in partially edentulous patients: Results after 24 months of follow-up	Barter S.; Stone P.; Brägger U.	2012
Oral rehabilitation of a patient with bruxism and cluster implant failures in the edentulous maxilla: A clinical report	Lin W.-S.; Ercoli C.; Lowenguth R.; Yerke L.M.; Morton D.	2012
Impact of platform switching on inter-proximal bone levels around short implants in the posterior region; 1-year results from a randomized clinical trial	Telleman G.; Raghoebar G.M.; Vissink A.; Meijer H.J.A.	2012
Osseointegration: The slow delivery of BMP-2 enhances osteoinductivity	Hunziker E.B.; Enggist L.; Küffer A.; Buser D.; Liu Y.	2012
The dual-zone therapeutic concept of managing immediate implant placement and provisional restoration in anterior extraction sockets.	Chu S.J.; Salama M.A.; Salama H.; Garber D.A.; Saito H.; Sarnachiaro G.O.; Tarnow D.P.	2012
One-year re-entry results of guided bone regeneration around immediately placed implants with immediate or conventional loading: a case series.	Shibly O.; Kutkut A.; Albandar J.M.	2012
Detection of crestal radiolucencies around dental implants: An in vitro experimental study	Sirin Y.; Horasan S.; Yaman D.; Basegmez C.; Tanyel C.; Aral A.; Guven K.	2012
Extraoral implants in the rehabilitation of craniofacial defects: Implant and prosthesis survival rates and peri-implant soft tissue evaluation	Curi M.M.; Oliveira M.F.; Molina G.; Cardoso C.L.; De Groot Oliveira L.; Branemark P.-I.; De Cássia Braga Ribeiro K.	2012
Functional and clinical outcome of tha using 4th generation large diameter ceramic couples. Alternative big balls	Raman R.; Johnson G.; Gopal S.; Shaw C.	2012
Interventions for replacing missing teeth: treatment of peri-implantitis.	Esposito M.; Grusovin M.G.; Worthington H.V.	2012
Post-implant neuropathy of the trigeminal nerve. A case series.	Renton T.; Dawood A.; Shah A.; Searson L.; Yilmaz Z.	2012
Long-term dental implant success and survival - A clinical study after an observation period up to 6 years	Charyeva O.; Altynbekov K.; Zhartybaev R.; Sabdanaliev A.	2012
Evaluation of periodontal and periimplant tissues in patients with dental implants	Legović R.; Aurer A.	2012
Hierarchical decisions on teeth vs. implants in the periodontitis-susceptible patient: The modern dilemma	Donos N.; Laurell L.; Mardas N.	2012
Transmission of Periodontopathic Bacteria from Natural Teeth to Implants	Aoki M.; Takanashi K.; Matsukubo T.; Yajima Y.; Okuda K.; Sato T.; Ishihara K.	2012
Systemic antibiotics and debridement of peri-implant mucositis. A randomized clinical trial	Hallström H.; Persson G.R.; Lindgren S.; Olofsson M.; Renvert S.	2012
[Clinical, microbiological and immunological findings in peri-implantitis patients with bar-retained lower removable partial dentures, compared to a healthy control group (12-month-follow-up)].	Mesmer C.; Forster A.; Antal M.; Nagy K.	2012
Treatment of peri-implantitis with deproteinised bovine bone and tetracycline: a case report.	Park J.B.	2012
Implant treatment in patients with oral lichen planus: A prospective-controlled study	Hernández G.; Lopez-Pintor R.M.; Arriba L.; Torres J.; de Vicente J.C.	2012
Metagenomic analysis of the peri-implant and periodontal microflora in patients with clinical signs of gingivitis or mucositis	Heuer W.; Kettenring A.; Stumpp S.N.; Eberhard J.; Gellermann E.; Winkel A.; Stiesch M.	2012
Single Implant Treatment in Healing versus Healed Sites of the Anterior Maxilla: A Clinical and Radiographic Evaluation	Eghbali A.; De Bruyn H.; De Rouck T.; Cleymaet R.; Wyn I.; Cosyn J.	2012

Survival and success rates of immediately and early loaded implants: 12-month results from a multicentric randomized clinical study.	Grandi T.; Garuti G.; Guazzi P.; Tarabini L.; Forabosco A.	2012
Comparison of variable-thread tapered implant designs to a standard tapered implant design after immediate loading. A 3-year multicentre randomised controlled trial.	Arnhart C.; Kielbassa A.M.; Martinez-de Fuentes R.; Goldstein M.; Jackowski J.; Lorenzoni M.; Maiorana C.; Mericske-Stern R.; Pozzi A.; Rompen E.; Sanz M.; Strub J.R.	2012
One-piece zirconia oral implants: One-year results from a prospective cohort study. 1. Single tooth replacement	Kohal R.-J.; Knauf M.; Larsson B.; Sahlin H.; Butz F.	2012
Clinical and radiographic evaluation of immediately loaded one-piece implants placed into fresh extraction sockets.	Soardi C.M.; Bianchi A.E.; Zandanel E.; Spinato S.	2012
Current knowledge and perspectives for the use of Platelet-Rich Plasma (PRP) and Platelet-Rich Fibrin (PRF) in oral and maxillofacial surgery part 2: Bone graft, implant and reconstructive surgery	Simonpieri A.; Del Corso M.; Vervelle A.; Jimbo R.; Inchingolo F.; Sammartino G.; Dohan Ehrenfest D.M.	2012
The comparison of two techniques to increase the amount of peri-implant attached mucosa: free gingival grafts versus vestibuloplasty. One-year results from a randomised controlled trial.	Basegmez C.; Ersanli S.; Demirel K.; Bölükbasi N.; Yalcin S.	2012
The effect of smoking on survival and bone loss of implants with a fluoride-modified surface: A 2-year retrospective analysis of 1106 implants placed in daily practice	Vervaeke S.; Collaert B.; Vandeweghe S.; Cosyn J.; Deschepper E.; De Bruyn H.	2012
Pouch roll technique for implant soft tissue augmentation: a variation of the modified roll technique.	Park S.H.; Wang H.L.	2012
A comparative Study between early occlusal loading at 1 and 6 weeks in implant-retained mandibular overdentures	Gadallah A.A.; Youssef H.G.; Shawky Y.M.	2012
Histological analysis of soft and hard tissues in a periimplantitis lesion: A human case report	Jung S.-R.; Bashutski J.D.; Jandali R.; Prasad H.; Rohrer M.; Wang H.-L.	2012
Immediate versus early loading of 6.5 mm-long flapless-placed single implants: a 4-year after loading report of a split-mouth randomised controlled trial.	Cannizzaro G.; Felice P.; Leone M.; Ferri V.; Viola P.; Esposito M.	2012
Identification of interleukin 2, 6, and 8 levels around miniscrews during orthodontic tooth movement	Hamamci N.; Acun Kaya F.; Uysal E.; Yokuş B.	2012
Retrievable Metal Ceramic Implant-Supported Fixed Prostheses with Milled Titanium Frameworks and All-Ceramic Crowns: Retrospective Clinical Study with up to 10 Years of Follow-Up	Maló P.; de Araújo Nobre M.; Borges J.; Almeida R.	2012
The Effect of Platelet-Rich Plasma on Healing of Palatal Donor Site following Connective Tissue Harvesting: A Pilot Study in Dogs	Shayesteh Y.S.; Eshghyar N.; Moslemi N.; Dehghan M.M.; Motahhary P.; Ghobadi Z.; Golestan B.	2012
Cervical prosthesis and cervical spine mobility. Case study analysis with a 3 years minimum follow-up	Pugliese R.; Lombardi F.; Butti G.; Bellantoni G.; Bongetta D.; Fratto A.; Adinolfi D.; Arienta C.	2012
Cardiac complications in post PCI patients undergoing non cardiac surgery	Ragoonanan T.; Syed S.; Wijeysundera D.; Harsha P.; Beattie S.; Wasowicz M.	2012
The sex specificity of hip-joint muscles offers an explanation for better results in men after total hip arthroplasty	Preininger B.; Schmorl K.; Von Roth P.; Winkler T.; Matziolis G.; Perka C.; Tohtz S.	2012
Interventions for replacing missing teeth: management of soft tissues for dental implants.	Esposito M.; Maghaireh H.; Grusovin M.G.; Ziounas I.; Worthington H.V.	2012
ATTACHE: A phase III, multicenter, randomized comparison of chemotherapy given prior to and post surgical resection versus chemotherapy given post surgical resection for hepatic metastases from colorectal cancer	Fawcett J.; Sjoquist K.M.; Padbury R.; Christophi C.; Tebbutt N.C.; Charles H.C.P.; Gebski V.; Wong N.; Aiken C.; Goldstein D.	2012
Serial troponin-I measurement after elective percutaneous coronary intervention: Is it worth?	Sousa A.; Lebreiro A.; Sousa C.; Rangel I.; Lopes R.; Amorim S.; Martins E.; Campelo M.; Silva J.; Maciel M.J.	2012
Prevention of wear particle-induced osteolysis by a novel V-ATPase inhibitor saliphenylhalamide through inhibition of osteoclast bone resorption	Qin A.; Cheng T.S.; Lin Z.; Cao L.; Chim S.M.; Pavlos N.J.; Xu J.; Zheng M.H.; Dai K.R.	2012
Managing Peri-Implant Bone Loss: Current Understanding	Aljateeli M.; Fu J.-H.; Wang H.-L.	2012
Seven-Year Follow-Up Results of TiUnite Implants Supporting Mandibular Overdentures: Early versus Delayed Loading	Turkyilmaz I.; Tozum T.F.; Fuhrmann D.M.; Tumer C.	2012
Bactericidal effect of CO2 laser and Tigran brush on dental implant surfaces: A comparative in vitro study	Sadighi M.; Pourabbas R.; Mobaiyen H.; Sadighi M.; Rikhtegaran S.; Fekrazad R.; Parviz S.	2012
A 1-Year Prospective Study on Co-Axis® Implants Immediately Loaded with a Full Ceramic Crown	Vandeweghe S.; Cosyn J.; Thevissen E.; Van den Berghe L.; De Bruyn H.	2012
Platform switching and matrix metalloproteinase-8 levels in peri-implant sulcular fluid	Canullo L.; Iannello G.; Netuschil L.; Jepsen S.	2012
Retrospective Comparison of Semipermanent and Permanent Cementation of Implant-Supported Single Crowns and FDPs with Regard to the Incidence of Survival and Complications	Schwarz S.; Schröder C.; Corcodel N.; Hassel A.J.; Rammelsberg P.	2012
The Effect of Maximum Bite Force, Implant Number, and Attachment Type on Marginal Bone Loss around Implants Supporting Mandibular Overdentures: A Retrospective Study	Geckili O.; Mumcu E.; Bilhan H.	2012
Four-Millimeter Implants Supporting Fixed Partial Dental Prostheses in the Severely Resorbed Posterior Mandible: Two-Year Results	Slotte C.; Grønningsaeter A.; Halmøy A.-M.; Öhrnell L.-O.; Stroh G.; Isaksson S.; Johansson L.-A.; Mordenfeld A.; Eklund J.; Embring J.	2012
Immediate Fixed Implant Rehabilitation of the Atrophic Edentulous Maxilla after Bilateral Sinus Floor Augmentation: A 12-Month Pilot Study	Pieri F.; Aldini N.N.; Fini M.; Marchetti C.; Corinaldesi G.	2012
A Comparison of Fit of CNC-Milled Titanium and Zirconia Frameworks to Implants	Abduo J.; Lyons K.; Waddell N.; Bennani V.; Swain M.	2012
Tissue structure modification in late stage osteoarthritis is feasible	Lafeber F.; Mastbergen S.	2012

Intracoronal mandibular Kennedy Class I implant-tooth supported removable partial overdenture: a 2-year multicenter prospective study.	El Mekawy N.H.; El-Negoly S.A.; Grawish M.A.; El-Hawary Y.M.	2012
Acute safety results from ACP European multicenter post-market observational Study	Park J.W.; Sievert H.; Schillinger W.; Lickfett L.; Lopez-Minguez J.R.; Heyder O.; Valdes Chavarri M.; Hennen B.; Ormerod O.; Walsh K.; Neuzil P.; Schräder R.; Garcia E.; Hildick-Smith D.; Lezaun R.	2012
Comparative cost-effectiveness analysis of coflex interlaminar stabilization versus posterolateral fusion for lumbar stenosis and lowgrade spondylolisthesis	Auerbach J.; Zigler J.; Davis R.; Pettine K.; Yeung A.	2012
Steroids for the prevention of restenosis in bare metal stents - A systematic review and meta-analysis	Chatterjee S.; Sardar P.; Mukherjee D.; Garratt K.	2012
Development of an index to assess diseaserelated damage in takayasu arteritis and use in outcome of vascular interventions	Sivakumar R.; Misra R.; Danda D.; Mahendranath K.M.; Bacon P.A.	2012
Assessing real-world European patient profiles in the Helping Evaluate Reduction in Obesity (HERO) study	Dixon J.B.; Nancy Dreyer N.; Finkelstein E.A.; Su Z.; Globe D.; Okerson T.	2012
Twelve-month clinical and angiographic results of the multicenter first-in-man BIOSOLVE-I study with the paclitaxel-eluting bioabsorbable magnesium scaffold	Haude M.; Erbel R.; Verheye S.; Waksman R.; Degen H.; Dirk B.; Vermeersch P.; Erne P.; Koolen J.	2012
Efficacy and safety of extra-cranial carotid stent placement-a nation-wide case-series report	Lin J.W.; Chang C.-H.; Lai M.-S.	2012
Evaluation of FactORs ImpacTing CLinical Outcome and Cost EffectiveneSS of the S-ICD: Design and rationale of the EFFORTLESS S-ICD registry	Pedersen S.S.; Lambiase P.; Boersma L.V.A.; Murgatroyd F.; Johansen J.B.; Reeve H.; Stuart A.G.; Adragao P.; Theuns D.A.M.J.	2012
Endovascular treatment of middle cerebral artery aneurysm: Single center case series	Pandya D.; Lazzaro M.; Fitzsimmons B.-F.; Lynch J.; Zaidat O.	2012
Effect of different localizations of microgap on clinical parameters and inflammatory cytokines in peri-implant crevicular fluid: A prospective comparative study	Boynueğri A.D.; Yalim M.; Nemli S.K.; Ergüder B.I.; Gökalp P.	2012
Ten-year results of a three arms prospective cohort study on implants in periodontally compromised patients. Part 2: Clinical results	Roccuzzo M.; Bonino F.; Aglietta M.; Dalmasso P.	2012
Soft Tissue Conditions and Marginal Bone Changes around Immediately Loaded Implants Inserted in Edentate Jaws Following Computer Guided Treatment Planning and Flapless Surgery: A ≥1-Year Clinical Follow-Up Study	Komiyama A.; Hultin M.; Näsström K.; Benchimol D.; Klinge B.	2012
Peri-implant mucositis and peri-implantitis: commentary.	Feller L.; Khammissa R.A.; Meyerov R.; Lemmer J.	2012
Implant complications: risk evaluation, diagnosis, management and outcomes	Peake G.G.	2012
Peri-implant soft tissue level secondary to a connective tissue graft in conjunction with immediate implant placement: a 2-year follow-up report of 11 consecutive cases.	Lee Y.M.; Kim D.Y.; Kim J.Y.; Kim S.H.; Koo K.T.; Kim T.I.; Seol Y.J.	2012
Long-Term Results of Endosteal Implants Following Radical Oral Cancer Surgery with and without Adjuvant Radiation Therapy	Linsen S.S.; Martini M.; Stark H.	2012
Topical Application of Melatonin and Growth Hormone Accelerates Bone Healing around Dental Implants in Dogs	Muñoz F.; López-Peña M.; Miño N.; Gómez-Moreno G.; Guardia J.; Cutando A.	2012
Comparison between graft and no-graft in an immediate placed and immediate nonfunctional loaded implant	Spinato S.; Agnini A.; Chiesi M.; Agnini A.M.; Wang H.L.	2012
Restoration of a peri-implant defect by platelet-rich fibrin	Lee J.-W.; Kim S.-G.; Kim J.-Y.; Lee Y.-C.; Choi J.-Y.; Dragos R.; Rotaru H.	2012
Influence of implants with different sizes and configurations installed immediately into extraction sockets on peri-implant hard and soft tissues: An experimental study in dogs	Caneva M.; Botticelli D.; Rossi F.; Cardoso L.C.; Pantani F.; Lang N.P.	2012
Presence of aspartate aminotransferase in peri-implant crevicular fluid with and without mucositis.	Sánchez-Pérez A.; Moya-Villaescusa M.J.; Caffesse R.G.	2012
Marginal bone loss adjacent to conventional and immediate loaded two implants supporting a ball-retained mandibular overdenture: A 3-year randomized clinical trial	Elsyad M.A.; Al-Mahdy Y.F.; Fouad M.M.	2012
Immediate loading of mandibular dental implants in partially edentulous patients: a prospective randomized comparative study.	Margossian P.; Mariani P.; Stephan G.; Margerit J.; Jorgensen C.	2012
Removable four implant-supported mandibular overdentures rigidly retained with telescopic crowns or milled bars: A 3-year prospective study	Krennmair G.; Sütö D.; Seemann R.; Piehslinger E.	2012
Crestal bone changes on platform-switched implants and adjacent teeth when the tooth-implant distance is less than 1.5 mm.	Vela X.; Méndez V.; Rodríguez X.; Segalá M.; Tarnow D.P.	2012
Use of Covered Self Expandable Metal Stents (CSEMS) for endoscopic management of non-stricture related benign biliary diseases	Akbar A.; Baron T.H.; Irani S.; Topazian M.; Petersen B.T.; Gostout C.J.; Levy M.J.; Gan S.I.; Gluck M.; Ross A.S.; Kozarek R.A.	2012
Guideline quandary in VTE prophylaxis management in elective joint replacement	Prager J.	2012
Comparison of Ho: YAG laser and coblation for interface tissue removal in minimally invasive hip refixation procedures	Kraaij G.; Malan D.F.; van der Heide H.J.L.; Dankelman J.; Nelissen R.G.H.H.; Valstar E.R.	2012
Collagen and 8-isoprostane are key factors of enhanced platelet aggregability observed in patients who underwent percutaneous coronary intervention	Soontrapa S.; Nantsupawat T.; Simoni G.; Simoni J.; Roongsritong C.; Meyerrose G.; Kumar A.; Tech T.	2012
Morphofunctional aspects of dental implants	Meyer G.; Fanghänel J.; Proff P.	2012
Residual periodontal pockets are a risk indicator for peri-implantitis in patients treated for periodontitis	Cho-Yan Lee J.; Mattheos N.; Nixon K.C.; Ivanovski S.	2012

Peri-implant mucositis and peri-implantitis: bacterial infection.	Khammissa R.A.; Feller L.; Meyerov R.; Lemmer J.	2012
Porous titanium granules in the surgical treatment of peri-implant osseous defects: a randomized clinical trial.	Wohlfahrt J.C.; Lyngstadaas S.P.; Rønold H.J.; Saxegaard E.; Ellingsen J.E.; Karlsson S.; Aass A.M.	2012
Three-year prospective multicenter study evaluating marginal bone levels and soft tissue health around a one-piece implant system.	Finne K.; Rompen E.; Toljanic J.	2012
A new submerged split-thickness skin graft technique to rebuild peri-implant keratinized soft tissue in composite flap reconstructed mandible or maxilla	Fang W.; Ma W.; Ma W.-G.; Li D.-H.; Liu B.-L.	2012
Clinical efficacy of a xenogeneic collagen matrix in augmenting keratinized mucosa around implants: A randomized controlled prospective clinical trial	Lorenzo R.; García V.; Orsini M.; Martin C.; Sanz M.	2012
Implant crest module: A review of biomechanical considerations	Aparna I.N.; Dhanasekar B.; Lingeshwar D.; Gupta L.	2012
Ten-year retrospective follow-up study of the TiOblast™ dental implant	Al-Nawas B.; Kämmerer P.W.; Morbach T.; Ladwein C.; Wegener J.; Wagner W.	2012
Nasal floor augmentation for the reconstruction of the atrophic maxilla: A case series	El-Ghareeb M.; Pi-Anfruns J.; Khosousi M.; Aghaloo T.; Moy P.	2012
A 10-year prospective study of single tooth implants placed in the anterior maxilla	Gotfredsen K.	2012
A randomized, controlled clinical evaluation of a synthetic gel membrane for guided bone regeneration around dental implants: clinical and radiologic 1- and 3-year results.	Ramel C.F.; Wismeijer D.A.; Hämmerle C.H.; Jung R.E.	2012
Immediate positioning of definitive abutments versus repeated abutment replacements in immediately loaded implants: effects on bone healing at the 1-year follow-up of a multicentre randomised controlled trial.	Grandi T.; Guazzi P.; Samarani R.; Garuti G.	2012
Soft tissue remodeling technique as a non-invasive alternative to second implant surgery.	Vela X.; Méndez V.; Rodríguez X.; Segalà M.; Gil J.A.	2012
Immediate loading of two unsplinted implants retaining the existing complete mandibular denture in elderly edentulous patients: 1-year results from a multicentre prospective cohort study.	Grandi T.; Guazzi P.; Samarani R.; Garuti G.; Grandi G.	2012
Changes in the fractal dimension on pre- and post-implant panoramic radiographs	Sansare K.; Singh D.; Karjodkar F.	2012
Effect of mismatching abutments on implants with wider platforms - an experimental study in dogs	Baffone G.M.; Botticelli D.; Canullo L.; Scala A.; Beolchini M.; Lang N.P.	2012
Acute traumatic rupture of the thoracic aorta: Contemporary expectant management	Fox W.C.; Komorowski D.J.; Morano G.S.; Sydnor M.K.; Tisnado J.; Brinster D.	2012
Clinical research on peri-implant diseases: Consensus report of Working Group 4	Sanz M.; Chapple I.L.	2012
Impact of the outcome of guided bone regeneration in dehiscence-type defects on the long-term stability of peri-implant health: Clinical observations at 4 years	Schwarz F.; Sahm N.; Becker J.	2012
Peri-implant disease in subjects with and without preventive maintenance: A 5-year follow-up	Costa F.O.; Takenaka-Martinez S.; Cota L.O.M.; Ferreira S.D.; Silva G.L.M.; Costa J.E.	2012
Long-term follow-up of turned single implants placed in periodontally healthy patients after 16-22 years: Radiographic and peri-implant outcome	Dierens M.; Vandeweghe S.; Kisch J.; Nilner K.; de Bruyn H.	2012
Anti-infective surgical therapy of peri-implantitis. A 12-month prospective clinical study	Heitz-Mayfield L.J.A.; Salvi G.E.; Mombelli A.; Faddy M.; Lang N.P.	2012
Quality assessment of reporting of animal studies on pathogenesis and treatment of peri-implant mucositis and peri-implantitis. A systematic review using the ARRIVE guidelines	Schwarz F.; Iglhaut G.; Becker J.	2012
Clinical research in implant dentistry: Study design, reporting and outcome measurements: Consensus report of Working Group 2 of the VIII European Workshop on Periodontology	Tonetti M.; Palmer R.	2012
Successful management of peri-implantitis with a regenerative approach: a consecutive series of 51 treated implants with 3- to 7.5-year follow-up.	Froum S.J.; Froum S.H.; Rosen P.S.	2012
Spontaneous progression of experimental peri-implantitis at implants with different surface characteristics: An experimental study in dogs	Albouy J.-P.; Abrahamsson I.; Berglundh T.	2012
Reversibility of experimental peri-implant mucositis compared with experimental gingivitis in humans	Salvi G.E.; Aglietta M.; Eick S.; Sculean A.; Lang N.P.; Ramseier C.A.	2012
Immediate implant placement in molar regions: Risk factors for early failure	Urban T.; Kostopoulos L.; Wenzel A.	2012
The critical-size supraalveolar peri-implant defect model: Characteristics and use	Wikesjö U.M.; Susin C.	2012
Local delivery of antimicrobial peptides using self-organized TiO2 nanotube arrays for peri-implant infections	Ma M.; Kazemzadeh-Narbat M.; Hui Y.; Lu S.; Ding C.; Chen D.D.; Hancock R.E.; Wang R.	2012
Dental implant failure associated with bacterial infection and long-term bisphosphonate usage: A case report	Yuan K.; Chen K.-C.; Chan Y.-J.; Tsai C.-C.; Chen H.-H.; Shih C.-C.	2012
Submerged and transmucosal healing yield the same clinical outcomes with two-piece implants in the anterior maxilla and mandible: Interim 1-year results of a randomized, controlled clinical trial	Hämmerle C.H.F.; Jung R.E.; Sanz M.; Chen S.; Martin W.C.; Jackowski J.; Ivanoff C.-J.; Cordaro L.; Ganeles J.; Weingart D.; Wiltfang J.; Gahlert M.	2012
Influence of prognostic risk indicators on osseointegrated dental implant failure: a matched case-control analysis.	Alissa R.; Oliver R.J.	2012
Peri-implant bone reactions to immediate implants placed at different levels in relation to crestal bone. Part II: A pilot study in dogs	Negri B.; Calvo-Guirado J.L.; Ramírez-Fernández M.P.; Maté Sánchez-de Val J.; Guardia J.; Muñoz-Guzón F.	2012
A critical comparison of pre-clinical animal models for analyzing peri-implant hard tissue	Stübinger S.; Dard M.	2012

Buccal bone plate in the immediately placed and restored maxillary single implant: A 7-year retrospective study using computed tomography	Degidi M.; Nardi D.; Daprile G.; Piattelli A.	2012
Peri-implant bone reactions to immediate implants placed at different levels in relation to crestal bone. Part I: A pilot study in dogs	Negri B.; Calvo-Guirado J.L.; Pardo-Zamora G.; Ramírez-Fernández M.P.; Delgado-Ruíz R.A.; Muñoz-Guzón F.	2012
Bone response between platform switched implants in mini pigs	Elian N.	2012
Sustained release of adiponectin improves osteogenesis around hydroxyapatite implants by suppressing osteoclast activity in ovariectomized rabbits	Luo E.; Hu J.; Bao C.; Li Y.; Tu Q.; Murray D.; Chen J.	2012
Clinical and imaging outcomes for target aneurysm retreatment for remnant intracranial aneursym	Pandya D.J.; Issa M.A.; Lazzaro M.A.; Sweis R.; Taqi M.; Abraham M.; Gheith A.; Lynch J.R.; Zaidat O.O.	2012
Retrospective and descriptive study for aneurysms-anesthesia aspekt	Majstorovic B.M.; Kastratovic D.A.; Vukasinovic D.I.; Nestorovic D.B.	2012
Specialists' management decisions and attitudes towards mucositis and peri-implantitis	Mattheos N.; Collier S.; Walmsley A.D.	2012
Sem evaluation of 10 infected implants retrieved from man	Brunelli G.; Carinci F.; Zollino I.; Candotto V.; Scarano A.; Lauritano D.	2012
Guidance for the maintenance care of dental implants: clinical review	Todescan S.; Lavigne S.; Kelekis-Cholakis A.	2012
The role of keratinized mucosa in peri-implant health	Esper L.A.; Ferreira Jr. S.B.; Kaizer R.D.O.F.; De Almeida A.L.P.F.	2012
Patient satisfaction after prosthetic rehabilitation of bone-grafted alveolar clefts with nonsubmerged ITI Straumann dental implants loaded at three months	Landes C.A.; Buñdgen L.; Laudemann K.; Ghanaati S.; Sader R.	2012
Surface analysis of failed oral titanium implants after irradiated with ErCR:YSGG 2780 laser	Scarano A.; Sinjari B.; Diiorio D.; Murmura G.; Carinci F.; Lauritano D.	2012
Immediately restored one-piece single-tooth implants with reduced diameter: One-year results of a multi-center study	Zembić A.; Johannesen L.H.; Schou S.; Malo P.; Reichert T.; Farella M.; Hämmerle C.H.F.	2012
Peri-implant bone remodeling around an extraction socket: predictions of bone maintenance by finite element method	Chou H.-Y.; Romanos G.; Müftü A.; Müftü S.	2012
Single-flap approach for surgical debridement of deep intraosseous defects: A randomized controlled trial	Trombelli L.; Simonelli A.; Schincaglia G.P.; Cucchi A.; Farina R.	2012
Prevalence of complications after the oral rehabilitation with implant-supported hybrid prostheses	Real-Osuna J.; Almendros-Marqués N.; Gay-Escoda C.	2012
Clinical outcomes of single dental implants with external connections: results after 2 to 13 years	Camargos Gd.e V.; do Prado C.J.; das Neves F.D.; Sartori I.A.	2012
Immediate provisionalization of 3.0-mm-diameter implants replacing single missing maxillary and mandibular incisors: 1-year prospective study.	Oyama K.; Kan J.Y.; Rungcharassaeng K.; Lozada J.	2012
Immediate loading of mandibular overdentures supported by unsplinted direct laser metal-forming implants: Results from a 1-year prospective study	Mangano C.; Mangano F.G.; Shibli J.A.; Ricci M.; Perrotti V.; D'Avila S.; Piattellii A.	2012
Implant-supported first molar restorations: correlation of finite element analysis with clinical outcomes.	Ormianer Z.; Palti A.; Demiralp B.; Heller G.; Lewinstein I.; Khayat P.G.	2012
Prospective clinical evaluation of 201 direct laser metal forming implants: Results from a 1-year multicenter study	Mangano C.; Mangano F.; Shibli J.A.; Luongo G.; De Franco M.; Briguglio F.; Figliuzzi M.; Eccellente T.; Rapani C.; Piombino M.; MacChi A.	2012
Establishment of the epithelial attachment and connective tissue adaptation to implants installed under the concept of "platform switching": A histologic study in minipigs	Farronato D.; Santoro G.; Canullo L.; Botticelli D.; Maiorana C.; Lang N.P.	2012
Peri-implant hard tissue response to glow-discharged abutments: Prospective study. Preliminary radiological results	Canullo L.; Götz W.	2012
Soft-tissue healing and impression technique in implant dentistry: A case report	Varvara G.; Caputi S.; D'Arcangelo C.; Murmura G.	2012
Short dental implants: A systematic review	Annibali S.; Cristalli M.P.; Dell'Aquila D.; Bignozzi I.; La Monaca G.; Pilloni A.	2012
Clinical and radiologic evaluation of 70 immediately loaded single implants in the maxillary esthetic zone: preliminary results after 1 year of functional loading.	Di Alberti L.; Donnini F.; Di Alberti C.; Camerino M.; Sgaramella N.; Lo Muzio L.	2012
Injectable magnesium-enriched hydroxyapatite putty in peri-implant defects: a histomorphometric analysis in pigs.	Crespi R.; Capparè P.; Addis A.; Gherlone E.	2012
Effect of implant surface properties on peri-implant bone healing: implant stability and microcomputed tomographic analysis.	Al-Hamdan S.H.; Al-Hamdan K.; Junker R.; Jansen J.A.	2012
Trans-sinus dental implants, bone morphogenetic protein 2, and immediate function for all-on-4 treatment of severe maxillary atrophy	Jensen O.T.; Cottam J.; Ringeman J.; Adams M.	2012
Stability and marginal bone loss with three types of early loaded implants during the first year after loading.	Liaje A.; Ozkan Y.K.; Ozkan Y.; Vanlioğlu B.	2012
The microvascular corticocancellous femur flap for reconstruction of the anterior maxilla in adult cleft lip, palate, and alveolus patients	Gaggl A.; Bürger H.; Virnik S.; Schachner P.; Chiari F.	2012
FGF18 augments osseointegration of intra-medullary implants in osteopenic FGFR3-/- mice	Carli A.; Gao C.; Khayyat-Kholghi M.; Li A.; Wang H.; Ladel C.; Harvey E.J.; Henderson J.E.	2012
Effect of the platform-switching technique on preservation of peri-implant marginal bone: a systematic review.	Al-Nsour M.M.; Chan H.L.; Wang H.L.	2012
Does obesity influence clinical outcome at nine years following total knee replacement?	Collins R.A.; Walmsley P.J.; Amin A.K.; Brenkel I.J.; Clayton R.A.E.	2012
Current guidelines for endoscopy in patients receiving antithrombotic medication	Khor C.J.L.; Hartono J.L.	2012

A multicentre prospective randomised study of single-incision mini-sling (Ajust®) versus tension-free vaginal tape-obturator (TVT-O™) in the management of female stress urinary incontinence: Pain profile and short-term outcomes	Mostafa A.; Agur W.; Abdel-All M.; Guerrero K.; Lim C.; Allam M.; Yousef M.; N'Dow J.; Abdel-Fattah M.	2012
Correlation between different genotypes of human cytomegalovirus and Epstein-Barr virus and peri-implant tissue status	Jankovic S.; Aleksic Z.; Dimitrijevic B.; Lekovic V.; Milinkovic I.; Kenney B.	2011
Short (5 and 7 mm long) porous implant in the posterior atrophic mandible: a 5-year report of a prospective study.	Perelli M.; Abundo R.; Corrente G.; Saccone C.	2011
The peri-implant sulcus compared with internal implant and suprastructure components: A microbiological analysis	Cosyn J.; van Aelst L.; Collaert B.; Persson G.R.; de Bruyn H.	2011
Dental implant outcome after primary implantation into double-barreled fibula osteoseptocutaneous free flap-reconstructed mandible.	Chang Y.M.; Wallace C.G.; Tsai C.Y.; Shen Y.F.; Hsu Y.M.; Wei F.C.	2011
Effects of flapless implant surgery on soft tissue profiles: A prospective clinical study	Lee D.-H.; Choi B.-H.; Jeong S.-M.; Xuan F.; Kim H.-R.	2011
Ethical issues in replacing a periodontally involved tooth with dental implants: Thoughts, beliefs, and evidence	Levin L.	2011
Clinician- and patient-reported long-term evaluation of screw- and cement-retained implant restorations: A 5-year prospective study	Sherif S.; Susarla S.M.; Hwang J.-W.; Weber H.-P.; Wright R.F.	2011
Guided bone regeneration with and without a bone substitute at single post-extractive implants: 1-year post-loading results from a pragmatic multicentre randomised controlled trial.	De Angelis N.; Felice P.; Pellegrino G.; Camurati A.; Gambino P.; Esposito M.	2011
Patient-related risk factors for implant therapy. A critique of pertinent literature	Liddelow G.; Klineberg I.	2011
A 3-year post-loading report of a randomised controlled trial on the rehabilitation of posterior atrophic mandibles: short implants or longer implants in vertically augmented bone?	Esposito M.; Cannizarro G.; Soardi E.; Pellegrino G.; Pistilli R.; Felice P.	2011
Summary of current consensus on the effect of smoking on implant therapy.	Snider T.N.; Cottrell D.; Batal H.	2011
Undersized implant site preparation to enhance primary implant stability in poor bone density: A prospective clinical study	Alghamdi H.; Anand P.S.; Anil S.	2011
Osseointegration of Cobalt-chrome alloy implants	Mavrogenis A.F.; Papagelopoulos P.J.; Babis G.C.	2011
Maxillary sinus floor augmentation using bovine bone grafts with simultaneous implant placement: A 5-year prospective follow-up study	Özkan Y.; Akoğlu B.; Kulak-Özkan Y.	2011
Peri-prosthetic joint infection - A diagnostic and treatment hospital analysis	Cismąiu R.S.; Vasile L.	2011
Carotid endarterectomy is more cost-effective than carotid artery stenting	Sternbergh W.C.; Crenshaw G.D.; Smith T.A.; Bazan H.A.	2011
Pre-operative transfusion reduces serious adverse events in patients with Sickle Cell Disease (SCD): Results from the Transfusion Alternatives Preoperatively in Sickle Cell Disease (TAPS) randomised controlled multicentre clinical trial	Howard J.; Malfroy M.; Charlotte L.; Choo L.; Rees D.; Walker I.; Johnson T.; Tillyer L.; Fijnvandraat K.; Kirby-Allen M.; Hodge R.; Purohit S.; Davies S.C.; Williamson L.M.	2011
Clinical outcomes of peri-procedural myocardial infarction after intracoronary stent implantation for bifurcation lesion: Results from coronary bifurcation stenting registry (COBIS)	Park K.-H.; Ahn Y.; Jeong H.C.; Kim H.-S.; Seong I.-W.; Yang J.-Y.; Rha S.W.; Jang Y.; Yoon J.H.; Tahk S.-J.; Seung K.B.; Park S.-J.; Gwon H.-C.; Jeong M.H.	2011
Outcome of surgical treatment of peri-implantitis: Results from a 2-year prospective clinical study in humans	Serino G.; Turri A.	2011
Mandibular bone harvesting for alveolar reconstruction and implant placement: Subjective and objective cross-sectional evaluation of donor and recipient site up to 4 years	Cordaro L.; Torsello F.; Tindara Miuccio M.; Mirisola di Torresanto V.; Eliopoulos D.	2011
Maxillary sinus augmentation with bovine bone and simultaneous dental implant placement in conditions of severe alveolar atrophy: a retrospective analysis of a consecutively treated case series.	Sivolella S.; Bressan E.; Gnocco E.; Berengo M.; Favero G.A.	2011
Retrospective clinical evaluation of an internal tube-in-tube dental implant after 4 years, with special emphasis on peri-implant bone resorption.	Al-Nawas B.; Kämmerer P.W.; Morbach T.; Ophoven F.; Wagner W.	2011
Immediate loading of unsplinted implants in the anterior mandible for overdentures: 3-year results.	Roe P.; Kan J.Y.; Rungcharassaeng K.; Lozada J.L.	2011
To splint or not to splint oral implants in the implant-supported overdenture therapy? A systematic literature review	Stoumpis C.; Kohal R.-J.	2011
Microbiologic evaluation of compromised periodontal sites before and after immediate intrasocket implant placement.	Tripodakis A.P.; Nakou M.	2011
The clinical and radiographic outcome of immediately loaded mini implants supporting a mandibular overdenture. A 3-year prospective study	Elsyad M.A.; Gebreel A.A.; Fouad M.M.; Elshoukouki A.H.	2011
Digital radiographic evaluation of the level of alveolar bone crest in external hexagon implants submitted to 2 types of implant abutments under immediate loading	Ponzoni D.; Gadotti R.J.; Sartori I.A.D.M.; Liotto Jr. E.M.	2011
Surgical experience influences 2-stage implant osseointegration	Zoghbi S.A.; De Lima L.A.P.A.; Saraiva L.; Romito G.A.	2011
Drug-eluting stents and noncardiac surgery: VA provider perceptions and management practices	Maddox T.M.; Graham L.A.; Hawn M.T.	2011
Acute primary total knee arthroplasty for peri-articular knee fractures in patients over 65 years of age	Malviya A.; Reed M.R.; Partington P.F.	2011
Drug-eluting stents and noncardiac surgery: VA provider perioperative practice based on guideline agreement and experience with stent thrombosis	Maddox T.M.; Graham L.A.; Hawn M.T.	2011

Comparison of enoxaparin and unfractionated heparin in endovascular interventions for the treatment of peripheral arterial occlusive disease: A randomized controlled trial	Duschek N.; Vafaie M.; Skrinjar E.; Hirsch K.; Waldhör T.; Hübl W.; Bergmayr W.; Knoebl P.; Assadian A.	2011
Removing biofilms from microstructured titanium Ex Vivo: A novel approach using atmospheric plasma technology	Rupf S.; Idlibi A.N.; Marrawi F.A.; Hannig M.; Schubert A.; von Mueller L.; Spitzer W.; Holtmann H.; Lehmann A.; Rueppell A.; Schindler A.	2011
Peri-implantitis and late implant failures in postmenopausal women: A cross-sectional study	Dvorak G.; Arnhart C.; Heuberer S.; Huber C.D.; Watzek G.; Gruber R.	2011
The clinical significance of keratinized gingiva around dental implants.	Greenstein G.; Cavallaro J.	2011
Understanding peri-implantitis: a strategic review.	Singh P.	2011
Flapless implant surgery and its effect on periimplant soft tissue	Höckl K.; Stoll P.; Stoll V.; Bähr W.; Bach G.	2011
Expression of HMGB1 and HMGN2 in gingival tissues, GCF and PICF of periodontitis patients and peri-implantitis	Luo L.; Xie P.; Gong P.; Tang X.-H.; Ding Y.; Deng L.-X.	2011
Morse taper connection implants supporting "planned" maxillary and mandibular bar-retained overdentures: A 5-year prospective multicenter study	Mangano C.; Mangano F.; Shibli J.A.; Ricci M.; Sammons R.L.; Figliuzzi M.	2011
Clinical and radiographic evaluation of three-implant-retained mandibular overdentures: a 3-year retrospective study.	Geckili O.; Bilhan H.; Mumcu E.	2011
Tilted implants treatment without maxillary sinus grafting in severely resorbed posterior maxilla	Wu Y.; Huang W.; Zhang Z.; Wang X.; Wang F.; Wang S.	2011
Single-tooth titanium implants produced with direct laser metal-forming technology. Results from a 1-year prospective multicenter study	Mangano F.; Mangano C.; Eccellentec T.; De Franco M.; Briguglio F.; Figliuzzi M.; Rapani M.; Luongo G.	2011
Endoscope guided internal sinus floor elevation and simultaneous dental implant placement	Schleier P.	2011
Effect of platform switching on peri-implant bone levels: A randomized clinical trial	Enkling N.; Jöhren P.; Klimberg V.; Bayer S.; Mericske-Stern R.; Jepsen S.	2011
Marginal bone loss around implants supporting fixed restorations.	Mumcu E.; Bilhan H.; Cekici A.	2011
Bruxism: Overview of current knowledge and suggestions for dental implants planning	Manfredini D.; Bucci M.B.; Sabattini V.B.; Lobbezoo F.	2011
Effect of the osteotome technique in cases of deficient edentulous ridge	Rammelsberg P.; Gabbert O.; Schmitter M.; Koob A.; Pritsch M.; Faggion Jr. C.M.	2011
Immediate single tooth replacement with subepithelial connective tissue graft using platform switching implants: a case series.	Chung S.; Rungcharassaeng K.; Kan J.Y.; Roe P.; Lozada J.L.	2011
Outcome of vascular interventions in takayasu arteritis using the takayasu arteritis damage score	Rajappa S.M.	2011
Initial clinical experience with vascular reconstruction device (enterprise) for the treatment of intracranial cerebral aneurysms in Japan	Takayama K.; Myouchin K.; Wada T.; Miyasaka T.; Akashi T.; Sakamoto M.; Taoka T.; Nakagawa H.; Kichikawa K.; Kurokawa S.	2011
Analysis of cathepsin-K levels in biologic fluids from healthy or diseased natural teeth and dental implants.	Yamalik N.; Günday S.; Kilinc K.; Karabulut E.; Berker E.; Tözüm T.F.	2011
A follow-up study of peri-implantitis cases after treatment	Charalampakis G.; Rabe P.; Leonhardt A.; Dahlén G.	2011
The success of endosseous implants in human immunodeficiency virus-positive patients receiving antiretroviral therapy: A pilot study	Oliveira M.A.; Gallottini M.; Pallos D.; Maluf P.S.Z.; Jablonka F.; Ortega K.L.	2011
Outcome of implant therapy involving localised lateral alveolar ridge and/or sinus floor augmentation: a clinical and radiographic retrospective 1-year study.	Hansen E.J.; Schou S.; Harder F.; Hjorting-Hansen E.	2011
A 5-year follow-up of 80 implants in 44 patients placed immediately after the lateral trap-door window procedure to accomplish maxillary sinus elevation without bone grafting.	Lin I.C.; Gonzalez A.M.; Chang H.J.; Kao S.Y.; Chen T.W.	2011
Influence of a machined collar on crestal bone changes around titanium implants: A histometric study in the canine mandible	Hermann J.S.; Jones A.A.; Bakaeen L.G.; Buser D.; Schoolfield J.D.; Cochran D.L.	2011
Immediate placement and provisionalization of single-tooth implants involving a final individual abutment. A 3-year clinical and radiographic retrospective study	Hartlev J.; Kohberg P.; Gotfredsen E.; Isidor F.; Schou S.	2011
[Complications in patients with oral implants. Recommendations for routine preventive inspections].	Meijer H.J.; Raghoebar G.M.; Goené R.J.; van der Weijden G.A.	2011
One year survival of 6-mm short dental implants related to implant site	Neldam C.A.; Gerds T.; Pinholt E.	2011
Significance of dental implants for health care	Schwarz F.; Terheyden H.	2011
The effect of smoking on early bone remodeling on surface modified Southern Implants®	Vandeweghe S.; De Bruyn H.	2011
Implant-prosthodontic rehabilitation of anterior partial edentulism: a clinical review.	Krennmair G.; Seemann R.; Weinländer M.; Wegscheider W.; Piehslinger E.	2011
Influence of microgap location and configuration on radiographic bone loss around submerged implants: an experimental study in dogs.	Weng D.; Nagata M.J.; Bosco A.F.; de Melo L.G.	2011
Clinical decision making: natural tooth salvage or implant?	Garg A.; Guez G.	2011
Computer-aided implant surgery in free flaps reconstructed patients, one year follow-up results of a prospective randomized clinical study	Meloni S.M.; De Riu G.; Pisano M.; Massarelli O.; Tullio A.	2011
A paralleling technique modification to determine the bone crest level around dental implants	Fernández-Formoso N.; Rilo B.; Mora M.J.; MartÍnez-Silva I.; Santana U.	2011
Increased sofat mrna expression during osteoclast development, evidence that sofat is not onlyassociated with T cells	Dharmapatni A.; Fitzpatrick D.; Cantley M.D.; Haynes D.R.	2011

Numerical simulation of bone remodelling around dental implants.	Ojeda J.; Martínez-Reina J.; García-Aznar J.M.; Domínguez J.; Doblaré M.	2011
Influence of microgap location and configuration on radiographic bone loss in nonsubmerged implants: an experimental study in dogs.	Weng D.; Nagata M.J.; Leite C.M.; de Melo L.G.; Bosco A.F.	2011
Diabetes mellitus and dental implants	Sahm N.; Schwarz F.; Becker J.	2011
Optical flow analysis of peri-stenting digital subtraction angiography in high grade carotid stenosis: Initial experience	Lin C.; Wu T.; Huang T.; Guo W.; Jhu W.; Li S.	2011
Color-coded x-rayangiography of intracranial vascular disorders: Peri-therapeutic quantitative analysis and correlation with MR perfusion and intra-operative doppler	Guo W.; Lin C.J.; Hung A.S.Z.; Chang F.C.; Luo C.B.; Lin J.F.; Chu W.F.	2011
The European Cardiac Resynchronization Therapy Survey: Comparison of outcomes between de novo cardiac resynchronization therapy implantations and upgrades	Bogale N.; Witte K.; Priori S.; Cleland J.; Auricchio A.; Gadler F.; Gitt A.; Limbourg T.; Linde C.; Dickstein K.; Fruhwald F.; Strohmer B.; Goethals M.; Vijgen J.; Trochu J.N.; Gras D.; Kindermann M.; Stellbrink C.; McDonnald K.; Keane D.; Ben Gal T.; Glikson M.; Metra M.; Gasparini M.; Maass A.; Jordaens L.; Alings M.; Larsen A.I.; Færestrand S.; Delgado J.; Mont L.; Persson H.; Brunner-La Rocca H.P.; Osswald S.; Squire I.; Morgan J.	2011
A trocar-free procedure for vaginal prolapse repair using mesh and a vaginal support device-an observational registry	Khandwala S.; Slack M.; Hinoul P.; Urquhart C.; Al-Salihi S.	2011
Multidisciplinary management of congenitally missing teeth with osseointegrated dental implants: a long-term report.	Hu X.L.; Li J.H.; Luo J.; Qiu L.X.; Lin Y.	2011
Surgical therapy of peri-implantitis lesions by means of a bovine-derived xenograft: Comparative results of a prospective study on two different implant surfaces	Roccuzzo M.; Bonino F.; Bonino L.; Dalmasso P.	2011
Clinical evaluation of an implant maintenance protocol for the prevention of peri-implant diseases in patients treated with immediately loaded full-arch rehabilitations.	Corbella S.; Del Fabbro M.; Taschieri S.; De Siena F.; Francetti L.	2011
Prevalence of periimplant disease in partially edentulous patients: A practice-based cross-sectional study	Rinke S.; Ohl S.; Ziebolz D.; Lange K.; Eickholz P.	2011
Immediate single-tooth implants in the anterior maxilla: 3-year results of a case series on hard and soft tissue response and aesthetics	Cosyn J.; Eghbali A.; De Bruyn H.; Collys K.; Cleymaet R.; De Rouck T.	2011
Sensitivity of resonance frequency analysis method to assess implant stability.	Turkyilmaz I.; Company A.M.	2011
Decision tree for the management of periimplant diseases	Okayasu K.; Wang H.-L.	2011
Stability, marginal bone loss and survival of standard and modified sand-blasted, acid-etched implants in bilateral edentulous spaces: A prospective 15-month evaluation	Karabuda Z.C.; Abdel-Haq J.; Arisan V.	2011
Early peri-implant tissue reactions on different titanium surface topographies	Yamano S.; Al-Sowygh Z.H.; Gallucci G.O.; Wada K.; Weber H.-P.; Sukotjo C.	2011
Healing of peri-implant tissues after flapless and flapped implant installation	Bayounis A.M.A.; Alzoman H.A.; Jansen J.A.; Babay N.	2011
Recombinant human bone morphogenetic protein-2 for peri-implant bone regeneration: A case report	Rotenberg S.A.; Tatakis D.N.	2011
A review of histomorphometric analysis techniques for assessing implant-soft tissue interface	Chai W.L.; Moharamzadeh K.; Brook I.M.; Van Noort R.	2011
Spiral implants bearing full-arch rehabilitation: analysis of clinical outcome.	Danza M.; Grecchi F.; Zollino I.; Casadio C.; Carinci F.	2011
Diabetes mellitus negatively affects peri-implant bone formation in the diabetic domestic pig	Von Wilmowsky C.; Stockmann P.; Harsch I.; Amann K.; Metzler P.; Lutz R.; Moest T.; Neukam F.W.; Schlegel K.A.	2011
A randomised prospective single-blinded study comparing “inside-out” versus “outside-in” transobturator tapes in the management of female stress urinary incontinence (E-TOT study); 3 years follow-up	Abdel-Fattah M.; Familusi A.; Ramsay I.; N'Dow J.	2011
A tricuspid valve endocarditis with a large vegetation encroaching on the papillary muscle and right ventricular cavity in patient with a ventricular septal defect	Alafify A.A.; Khuwaitir T.A.; Sharary M.A.; Sajid N.U.	2011
Retrograde Peri-implantitis: A case report introducing an approach to its management	Chan H.-L.; Wang H.-L.; Bashutski J.D.; Edwards P.C.; Fu J.-H.; Oh T.-J.	2011
Osmotic pressure of periimplant sulcular and gingival crevicular fluids: A split-mouth, randomized study of its measurement and clinical significance	Sakallioğlu U.; Lütfioğlu M.; Sakallioğlu E.E.; Sert S.; Ceylan G.	2011
Intramarrow bone morphogenetic protein 4 gene delivery enhances early implant stability in femurs of ovariectomized rabbits	Lai Y.-L.; Kuo N.-C.; Hsiao W.-K.; Yew T.-L.; Lee S.-Y.; Chen H.-L.	2011
Early loading of sandblasted and acid-etched titanium implants in the edentulous mandible: a prospective 5-year study.	Lethaus B.; Kälber J.; Petrin G.; Brandstätter A.; Weingart D.	2011
Clinical and histomorphometric observations around dual acid-etched and calcium phosphate nanometer deposited-surface implants.	Artzi Z.; Nemcovsky C.E.; Tal H.; Weinberg E.; Weinreb M.; Prasad H.; Rohrer M.D.; Kozlovsky A.	2011
Dental restoration of reconstructed mandible after wide resections for oral cancer: Beyond the fibular height deficiency by prosthodontics	Gessaroli M.; Manfredi M.; Lombardini F.; Bassi M.; Campobassi A.	2011
Part III: crystalline fluorapatite-coated hydroxyapatite; potential use as a bacteriostatic agent for both pre-implant cases and retreatment of infected implant sites: a report of 4 cases.	Nordquist W.D.; Krutchkoff D.J.	2011
[Peri-implantitis: tools and techniques for disinfecting the implant surface].	Bories C.; Struillou X.; Badran Z.; Soueidan A.	2011
The evaluation of bacterial flora in progress of peri-implant disease	Sato J.; Gomi K.; Makino T.; Kawasaki F.; Yashima A.; Ozawa T.; Maeda N.; Arai T.	2011

Early bacterial colonization and soft tissue health around zirconia and titanium abutments: An in vivo study in man	Van Brakel R.; Cune M.S.; Van Winkelhoff A.J.; De Putter C.; Verhoeven J.W.; Van der Reijden W.	2011
Identification of periodontal pathogens in healthy periimplant sites	Casado P.L.; Otazu I.B.; Balduino A.; De Mello W.; Barboza E.P.; Duarte M.E.L.	2011
Long-term stability of surgical bone regenerative procedures of peri-implantitis lesions in a prospective case-control study over 3 years	Roos-Jansåker A.-M.; Lindahl C.; Persson G.R.; Renvert S.	2011
Peri-implant management of patients with aggressive periodontitis.	Chee W.	2011
Oral rehabilitation with dental implants after cancer treatment	Barrowman R.A.; Wilson P.R.; Wiesenfeld D.	2011
[Replacement of extracted tooth by means of implant-supported direct abutment. A case report].	Fazekas R.; Fazekas A.	2011
Isotropic micropatterned silica coatings on zirconia induce guided cell growth for dental implants.	Pelaez-Vargas A.; Gallego-Perez D.; Magallanes-Perdomo M.; Fernandes M.H.; Hansford D.J.; De Aza A.H.; Pena P.; Monteiro F.J.	2011
The immediate placement of dental implants into extraction sites with periapical lesions: A retrospective chart review	Bell C.L.; Diehl D.; Bell B.M.; Bell R.E.	2011
The evaluation of the success of immediately placed single implants: A retrospective clinical study	Bilhan H.; Mumcu E.; Geçkili O.; Atalay B.	2011
Care and Aftercare Related to Implant-Retained Dental Crowns in the Maxillary Aesthetic Region: A 5-Year Prospective Randomized Clinical Trial	Visser A.; Raghoebar G.M.; Meijer H.J.; Meijndert L.; Vissink A.	2011
Volumetric analysis of in vivo bone-to-implant contact ratio using intrinsic thresholding	Lee W.; Kim D.; Yi W.; Cho Y.; Kim H.; Lee S.; Lee S.; Choi S.; Heo M.	2011
Radiographic evaluation of healing tissues of early-failing implants after therapy: a case report.	Sakka S.; Alissa R.	2011
Evaluation of Stress Distribution in Overdenture-Retaining Bar with Different Levels of Vertical Misfit	Spazzin A.O.; Abreu R.T.; Noritomi P.Y.; Consani R.L.X.; Mesquita M.F.	2011
Effects of growth hormone on initial bone formation around dental implants: A dog study	Calvo-Guirado J.L.; Mate-Sanchez J.; Delgado-Ruiz R.; Ramirez-Fernández M.P.; Cutando-Soriano A.; Peña M.	2011
Aprospectivemulticentre studyofadjustable single-incision mini-sling (Ajust®) in management of stress urinary incontinence in women: 1-Year follow-up study	Abdel-Fattah M.; Agur W.; Abdel-All M.; Guerrero K.; Allam M.; MacKintosh A.; Mostafa A.; Yousef M.	2011
A multicentre randomised trial comparing single-incision mini-sling (Ajust©) and tension-free vaginal tape-obturator (TVT-O) in management of female stress urinary incontinence	Mostafa A.; Agur W.; Abdel-All M.; Yousef M.; Allam M.; Guerrero K.; Lim C.; Abdel-Fattah M.	2011
Towards a new IHE-domain “surgery”	Burgert O.; Liebmann P.; Treichel T.; Lemke H.U.	2011
Pancreatic resection in the elderly patient-does age count?	Connell L.; Mulvihill E.; Keeffe M.O.; Suilleabhain C.O.; Reilly S.O.; Power D.	2011
Effect of replacement of mandibular defects with a modular endoprosthesis on bone mineral density in a monkey model	Wong R.C.W.; Lee S.; Tideman H.; Merkx M.A.W.; Jansen J.; Liao K.	2011
Evaluation of transobturator tapes in management of female stress urinary incontinence with versus without concomitant prolapse repair at 1 year follow-up	Abde-Fattah M.; Ayansina D.; Ramsay I.; Pringle S.; Hardwick C.; Bjornsson S.; Mostafa A.; Ali H.	2011
Implant-supported overdenture in an elderly patient with Huntington's disease.	Deniz E.; Kokat A.M.; Noyan A.	2011
A robotic cadaveric flatfoot analysis of stance phase	Jackson L.T.; Aubin P.M.; Cowley M.S.; Sangeorzan B.J.; Ledoux W.R.	2011
Radiographic bone fill of peri-implantitis defects following nonsurgical therapy: report of three cases.	Nibali L.; Donos N.	2011
Emerging antibacterial biomaterial strategies for the prevention of peri-implant inflammatory diseases.	Bumgardner J.D.; Adatrow P.; Haggard W.O.; Norowski P.A.	2011
The effects of laser microtexturing of the dental implant collar on crestal bone levels and peri-implant health.	Botos S.; Yousef H.; Zweig B.; Flinton R.; Weiner S.	2011
Development of an animal model for Aggregatibacter actinomycetemcomitans biofilm-mediated oral osteolytic infection: A preliminary study	Freire M.O.; Sedghizadeh P.P.; Schaudinn C.; Gorur A.; Downey J.S.; Choi J.-H.; Chen W.; Kook J.-K.; Chen C.; Goodman S.D.; Zadeh H.H.	2011
Adherent endotoxins on bone implant surfaces: A reappraisal	Morra M.; Cassinelli C.; Bollati D.; Cascardo G.	2011
Single implants in the esthetic zone: analysis of recent peri-implant soft tissue alterations and patient satisfaction. A photographic study.	Luo Z.; Zeng R.; Luo Z.; Chen Z.	2011
Marginal bone loss in relation to the implant neck surface: An update	Aloy-Prósper A.; Maestre-Ferrín L.; Peñarrocha-Oltra D.; Peñarrocha-Diago M.	2011
Comparison of ball and telescopic crown attachments in implant-retained mandibular overdentures: a 5-year prospective study.	Krennmair G.; Seemann R.; Weinländer M.; Piehslinger E.	2011
Comparison of two early loading protocols in full arch reconstructions in the edentulous maxilla using the Cresco prosthetic system: A three-arm parallel group randomized-controlled trial	Jokstad A.; Ellner S.; Gussgard A.	2011
Maintenance of implant-supported maxillary prostheses: a 2-year controlled clinical trial.	Katsoulis J.; Brunner A.; Mericske-Stern R.	2011
Implant-supported fixed prostheses in the edentulous maxilla: 8-year prospective results	Mertens C.; Steveling H.G.	2011
Equicrestal and subcrestal dental implants: A histologic and histomorphometric evaluation of nine retrieved human implants	Degidi M.; Perrotti V.; Shibli J.A.; Novaes A.B.; Piattelli A.; Iezzi G.	2011
Peri-implant bone tissue assessment by comparing the outcome of intra-oral radiograph and cone beam computed tomography analyses to the histological standard	Dos Santos Corpas L.; Jacobs R.; Quirynen M.; Huang Y.; Naert I.; Duyck J.	2011

Treatment concept of the edentulous mandible with prefabricated telescopic abutments and immediate functional loading.	Romanos G.E.; May S.; May D.	2011
Intermittent parathyroid hormone fails to stimulate osseointegration in diabetic rats	Kuchler U.; Spilka T.; Baron K.; Tangl S.; Watzek G.; Gruber R.	2011
The influence of individual bone patterns on peri-implant bone loss: preliminary report from a 3-year randomized clinical and histologic trial in patients treated with implants restored with matching-diameter abutments or the platform-switching concept.	Canullo L.; Iannello G.; Götz W.	2011
Radiographic evaluation of marginal bone level around internal-hex implants with switched platform: a clinical case report series.	de Almeida F.D.; Carvalho A.C.; Fontes M.; Pedrosa A.; Costa R.; Noleto J.W.; Mourão C.F.	2011
Five-year treatment outcomes with four types of implants in the posterior maxilla and mandible in partially edentulous patients: a retrospective study.	Ozkan Y.; Akoğlu B.; Kulak-Ozkan Y.	2011
Serial troponin-I measurement after elective percutaneous coronary intervention: Is it worth?	Sousa A.; Lebreiro A.; Sousa C.; Rangel I.; Lopes R.; Amorim S.; Martins E.; Campelo M.; Silva J.C.; Maciel M.J.	2011
Practice pattern of retrievable inferior vena CAVA filters in a tertiary care institution	Chakraborti D.; Bandyopadhyay T.	2011
Immediate loading of dental implants in the edentulous maxilla.	Eccellente T.; Piombino M.; Piattelli A.; D'Alimonte E.; Perrotti V.; Iezzi G.	2011
Teeth and implant surroundings: clinical health indices and microbiologic parameters.	Vered Y.; Zini A.; Mann J.; Kolog H.; Steinberg D.; Zambon J.J.; Haraszthy V.I.; DeVizio W.; Sreenivasan P.	2011
Assessment of pain associated with insertion torque of dental implants. A prospective, randomized-controlled study	Scarano A.; Piattelli A.; Assenza B.; Sollazzo V.; Lucchese A.; Carinci F.	2011
Influence of various implant platform configurations on peri-implant tissue dimensions: An experimental study in dog	Baffone G.M.; Botticelli D.; Pantani F.; Cardoso L.C.; Schweikert M.T.; Lang N.P.	2011
Open or submerged healing of implants with platform switching: A randomized, controlled clinical trial	Enkling N.; Jöhren P.; Klimberg T.; Mericske-Stern R.; Jervøe-Storm P.-M.; Bayer S.; Gülden N.; Jepsen S.	2011
Extraoral prostheses using extraoral implants	Pekkan G.; Tuna S.H.; Oghan F.	2011
Optimization of wound healing through coating of meshes for hernia reparation with the anti-ageing hormone DHEA	Röth A.A.; Fiebeler A.; Lehert H.; Neumann U.P.; Klinge U.	2011
Transcatheter aortic valve implant - Addressing the unique needs of this elderly population	Teehan S.	2011
Peri-implantitis: A risk factor in implant failure	Gupta H.K.; Garg A.; Bedi N.K.	2011
The association between selected risk indicators and severity of peri-implantitis using mixed model analyses	Koldsland O.C.; Scheie A.A.; Aass A.M.	2011
Prevalence of human cytomegalovirus and Epstein-Barr virus in subgingival plaque at peri-implantitis, mucositis and healthy sites. A pilot study	Jankovic S.; Aleksic Z.; Dimitrijevic B.; Lekovic V.; Camargo P.; Kenney B.	2011
Er:YAG laser in the clinical management of severe peri-implantitis: a case report.	Badran Z.; Bories C.; Struillou X.; Saffarzadeh A.; Verner C.; Soueidan A.	2011
Long-term results of mandibular implants supporting an overdenture: implant survival, failures, and crestal bone level changes.	Ueda T.; Kremer U.; Katsoulis J.; Mericske-Stern R.	2011
Platelet-derived growth factor applications in periodontal and peri-implant bone regeneration	Kaigler D.; Avila G.; Wisner-Lynch L.; Nevins M.L.; Nevins M.; Rasperini G.; Lynch S.E.; Giannobile W.V.	2011
Dimensional changes of peri-implant soft tissue over 2 years with single-implant crowns in the anterior maxilla	Gallucci G.O.; Grütter L.; Chuang S.-K.; Belser U.C.	2011
Marginal Bone Level Changes at Dental Implants after 5 Years in Function: A Meta-Analysis	Laurell L.; Lundgren D.	2011
Peri-implant tissue response following connective tissue and bone grafting in conjunction with immediate single-tooth replacement in the esthetic zone: a case series.	Tsuda H.; Rungcharassaeng K.; Kan J.Y.; Roe P.; Lozada J.L.; Zimmerman G.	2011
Stability of contour augmentation and esthetic outcomes of implant-supported single crowns in the esthetic zone: 3-year results of a prospective study with early implant placement postextraction.	Buser D.; Wittneben J.; Bornstein M.M.; Grütter L.; Chappuis V.; Belser U.C.	2011
Short implants in posterior jaws. A prospective 1-year study.	Maló P.; Nobre M.; Lopes A.	2011
Rehabilitation of postrior atrophic edentulous jaws: prostheses supported by 5 mm short implants or by longer implants in augmented bone? One-year results from a pilot randomised clinical trial.	Esposito M.; Pellegrino G.; Pistilli R.; Felice P.	2011
Influence of implant geometry on primary insertion stability and simulated peri-implant bone loss: an in vitro study using resonance frequency analysis and damping capacity assessment.	Lachmann S.; Laval J.Y.; Axmann D.; Weber H.	2011
Clinical and radiological outcomes of 1- versus 2-stage implant placement: 1-year results of a randomised clinical trial.	Tallarico M.; Vaccarella A.; Marzi G.C.	2011
Three-dimensional finite element analysis of fixed complete-arch prostheses supported by 4 immediate-loaded implants in the completely edentulous maxilla using clinical computerized tomography data.	Kimura K.; Fukase Y.; Makino M.; Masaki C.; Nakamoto T.; Hosokawa R.	2011
Do mucositis lesions around implants differ from gingivitis lesions around teeth?	Lang N.P.; Bosshardt D.D.; Lulic M.	2011
Bone regeneration using particulate grafts: An update	Aloy-Prósper A.; Maestre-Ferrin L.; Peñarrocha-Oltra D.; Peñarrocha-Diago M.	2011
Computer planning and bone density evaluation of jaws reconstructed with bone grafts from living donors	Grecchi F.; Zollino I.; Gallo F.; Rubino G.; Motroni A.; Carinci F.	2011
Peri-operative management of ophthalmic patients taking antithrombotic therapy	Lip G.Y.H.; Durrani O.M.; Roldan V.; Lip P.L.; Marin F.; Reuser T.Q.	2011

Influence of the peri-implantitis to the microscrew-bone interface	Hu Y.; Zheng L.; Tang T.; Zhao Z.; Song J.; Deng F.	2011
Dental implant treatment for two adjacent missing teeth in the maxillary aesthetic zone: A comparative pilot study and test of principle	Tymstra N.; Raghoebar G.M.; Vissink A.; Meijer H.J.A.	2011
Retrospective evaluation of crestal bone changes around implants with reduced abutment diameter placed mnon-submerged and at subcrestal npositions: The effect of bone grafting at implant placement	Koutouzis T.; Fetner M.; Fetner A.; Lundgren T.	2011
Assessment of quality of life after implant-retained prosthetically reconstructed maxillae and mandibles postcancer treatments	Dholam K.P.; Bachher G.K.; Yadav P.S.; Quazi G.A.; Pusalkar H.A.	2011
Eleven dental implants placed in a liver transplantation patient: A case report and 5-year clinical evaluation	Gu L.; Wang Q.; Yu Y.-C.	2011
Histological evaluation of the effect of using growth hormone around immediate dental implants in fresh extraction sockets: An experimental study	Hossam Eldein A.M.; Elghamrawy S.H.; Osman S.M.; Elhak A.R.	2011
A prospective, controlled clinical trial evaluating the clinical and radiological outcome after 3 years of immediately placed implants in sockets exhibiting periapical pathology	Truninger T.C.; Philipp A.O.H.; Siegenthaler D.W.; Roos M.; Hämmerle C.H.F.; Jung R.E.	2011
Implant surface characteristics influence the outcome of treatment of peri-implantitis: An experimental study in dogs	Albouy J.-P.; Abrahamsson I.; Persson L.G.; Berglundh T.	2011
Peri-implant soft tissue management through use of cultured gingival graft: A case report	Mohammadi M.; Mofid R.; Shokrgozar M.A.	2011
A 6-month study of the effects of 0.3% triclosan/copolymer dentifrice on dental implants	Sreenivasan P.K.; Vered Y.; Zini A.; Mann J.; Kolog H.; Steinberg D.; Zambon J.J.; Haraszthy V.I.; Da Silva M.P.; De Vizio W.	2011
Prevalence of bleeding on probing: a cohort study in a specialist periodontal clinic.	Farina R.; Scapoli C.; Carrieri A.; Guarnelli M.E.; Trombelli L.	2011
A novel surgical procedure for coronally repositioning of the buccal implant mucosa using acellular dermal matrix: A case report	Mareque-Bueno S.	2011
A 1-year prospective clinical study of soft tissue conditions and marginal bone changes around dental implants after flapless implant surgery	Jeong S.-M.; Choi B.-H.; Kim J.; Xuan F.; Lee D.-H.; Mo D.-Y.; Lee C.-U.	2011
The effect of different implant neck configurations on soft and hard tissue healing: A randomized-controlled clinical trial	Tan W.C.; Lang N.P.; Schmidlin K.; Zwahlen M.; Pjetursson B.E.	2011
A 10-year retrospective analysis of marginal bone-level changes around implants in periodontally healthy and periodontally compromised tobacco smokers	Aglietta M.; Siciliano V.I.; Rasperini G.; Cafiero C.; Lang N.P.; Salvi G.E.	2011
Facial gingival tissue stability following immediate placement and provisionalization of maxillary anterior single implants: a 2- to 8-year follow-up.	Kan J.Y.; Rungcharassaeng K.; Lozada J.L.; Zimmerman G.	2011
Five-year treatment outcomes with three brands of implants supporting mandibular overdentures.	Akoglu B.; Ucankale M.; Ozkan Y.; Kulak-Ozkan Y.	2011
Three-dimensional modeling and finite element analysis in treatment planning for orthodontic tooth movement	Ammar H.H.; Ngan P.; Crout R.J.; Mucino V.H.; Mukdadi O.M.	2011
Volume gain and stability of peri-implant tissue following bone and soft tissue augmentation: 1-year results from a prospective cohort study	Schneider D.; Grunder U.; Ender A.; Hämmerle C.H.F.; Jung R.E.	2011
Influence of implant-abutment interface design on bone and soft tissue levels around immediately placed and restored single-tooth implants: a randomized controlled clinical trial.	Pieri F.; Aldini N.N.; Marchetti C.; Corinaldesi G.	2011
Esthetic outcomes with porcelain-fused-to-ceramic and all-ceramic single-implant crowns: A randomized clinical trial	Gallucci G.O.; Grütter L.; Nedir R.; Bischof M.; Belser U.C.	2011
Complication and failure rates of fixed dental prostheses in patients treated for periodontal disease	Brägger U.; Hirt-Steiner S.; Schnell N.; Schmidlin K.; Salvi G.E.; Pjetursson B.; Matuliene G.; Zwahlen M.; Lang N.P.	2011
Implant-prosthetic rehabilitation of the edentulous maxilla and mandible with immediately loaded implants: preliminary data from a retrospective study, considering time of implantation.	Strietzel F.P.; Karmon B.; Lorean A.; Fischer P.P.	2011
Soft tissues around long-term platform switching implant restorations: A histological human evaluation. Preliminary results	Canullo L.; Pellegrini G.; Allievi C.; Trombelli L.; Annibali S.; Dellavia C.	2011
Long-term outcome of dental implants placed in revascularized fibula free flaps used for the reconstruction of maxillo-mandibular defects due to extreme atrophy	Chiapasco M.; Romeo E.; Coggiola A.; Brusati R.	2011
Deproteinized bovine bone in periodontal and implant surgery.	Baldini N.; De Sanctis M.; Ferrari M.	2011
Prognosis in implant dentistry: a system for classifying the degree of peri-implant mucosal inflammation.	Nogueira-Filho G.; Iacopino A.M.; Tenenbaum H.C.	2011
In vivo performance of titanium implants functionalized with eicosapentaenoic acid and UV irradiation	Petzold C.; Rubert M.; Lyngstadaas S.P.; Ellingsen J.E.; Monjo M.	2011
Micro CT and human histological analysis of a peri-implant osseous defect grafted with porous titanium granules: a case report.	Wohlfahrt J.C.; Aass A.M.; Ronold H.J.; Lyngstadaas S.P.	2011
Sinus lift with guided bone regeneration or anorganic bovine bone: 1-year post-loading results of a pilot randomised clinical trial.	Esposito M.; Piattelli M.; Pistilli R.; Pellegrino G.; Felice P.	2010
Influence of metals on cytokines production in connection with successful implantation therapy in dentistry	Podzimek S.; Tomka M.; Nemeth T.; Himmlova L.; Matucha P.; Prochazkova J.	2010
Clinical effect of implant healing between submerged and non-submerged dental implants: a randomized controlled study	Zhao P.; Shi J.	2010
Vertical augmentation with interpositional blocks of anorganic bovine bone vs. 7-mm-long implants in posterior mandibles: 1-year results of a randomized clinical trial	Felice P.; Pellegrino G.; Checchi L.; Pistilli R.; Esposito M.	2010
Crestal bone resorption around platform-switched dental implants with fine threaded neck after immediate and delayed loading.	Heinemann F.; Hasan I.; Schwahn C.; Biffar R.; Mundt T.	2010

Immediate positioning of a definitive abutment versus repeated abutment replacements in post-extractive implants: 3-year follow-up of a randomised multicentre clinical trial.	Canullo L.; Bignozzi I.; Cocchetto R.; Cristalli M.P.; Iannello G.	2010
A split-mouth comparative study up to 16 years of two screw-shaped titanium implant systems	Jacobs R.; Pittayapat P.; Van Steenberghe D.; De Mars G.; Gijbels F.; Van Der Donck A.; Li L.; Liang X.; Van Assche N.; Quirynen M.; Naert I.	2010
Comparison of crestal bone loss and implant stability among the implants placed with conventional procedure and using osteotome technique: a clinical study.	Padmanabhan T.V.; Gupta R.K.	2010
Removal of an oral squamous cell carcinoma including parts of osseointegrated implants in the marginal mandibulectomy. A case report	Meijer G.J.; Dieleman F.J.; Bergé S.J.; Merkx M.A.W.	2010
Evaluation of peri-implant marginal bone loss using modified abutment connections at various crestal level placements.	Veis A.; Parissis N.; Tsirlis A.; Papadeli C.; Marinis G.; Zogakis A.	2010
Implant primary stability determined by resonance frequency analysis in surgically created defects: A pilot cadaver study	Chan H.-L.; El-Kholy K.; Fu J.-H.; Galindo-Moreno P.; Wang H.-L.	2010
Zirconia removable telescopic dentures retained on teeth or implants for maxilla rehabilitation. Three-year observation of three cases.	Zafiropoulos G.G.; Rebbe J.; Thielen U.; Deli G.; Beaumont C.; Hoffmann O.	2010
Ridge augmentation of the atrophic posterior mandible with inlay and onlay iliac bone grafting : A prospective controlled clinical trial for the comparison of two techniques	Lizio G.; Felice P.; Pellegrino G.; Pistilli R.; Marchetti C.	2010
Replacement of vertically root-fractured endodontically treated teeth with immediate implants in conjunction with a synthetic bone cement	Taschieri S.; Rosano G.; Weinstein T.; Del Fabbro M.	2010
Early complications associated with total hip arthroplasty due to femoral neck fracture	Gallo J.; Čechová I.; Zapletalová J.	2010
Clinical follow-up of unilateral, fixed dental prosthesis on maxillary implants.	Wahlström M.; Sagulin G.B.; Jansson L.E.	2010
A 4-year evaluation of the peri-implant parameters of immediately loaded implants placed in fresh extraction sockets	Crespi R.; Capparè P.; Gherlone E.	2010
Peri-implant disease: the basics.	Garg A.	2010
Osteotome sinus floor elevation technique without grafting: A 5-year prospective study	Nedir R.; Nurdin N.; Vazquez L.; Szmukler-Moncler S.; Bischof M.; Bernard J.-P.	2010
Clinical evaluation of marginal bone level change of multiple adjacent implants restored with splinted and nonsplinted restorations: a 5-year prospective study.	Vigolo P.; Zaccaria M.	2010
Clinical and microbiological effects of mechanical instrumentation and local antimicrobials during periodontal supportive therapy in aggressive periodontitis patients: Smoker versus non-smoker patients	Guarnelli M.E.; Farina R.; Cucchi A.; Trombelli L.	2010
The clinical and radiographic outcome of implants placed in the posterior maxilla with a guided flapless approach and immediately restored with a provisional rehabilitation: a randomized clinical trial.	Van de Velde T.; Sennerby L.; De Bruyn H.	2010
Implant treatment in grafted and native bone in patients affected by ectodermal dysplasia	Grecchi F.; Pagliani L.; Mancini G.E.; Zollino I.; Carinci F.	2010
Combined effects of implant insertion depth and alveolar bone quality on periimplant bone strain induced by a wide-diameter, short implant and a narrow-diameter, long implant	Chou H.-Y.; Müftü S.; Bozkaya D.	2010
Discomfort experienced after immediate implant placement associated with three different regenerative techniques.	Urban T.; Wenzel A.	2010
Evidence-based treatment for a smoker with periimplantitis	Wang P.; Xie H.-X.; Qu Y.-L.; Man Y.	2010
Flapless surgery and immediately loaded implants: a retrospective comparison between implantation with and without computer-assisted planned surgical stent.	Danza M.; Carinci F.	2010
Effects of occlusal overload on peri-implant tissue health: A systematic review of animal-model studies	Chambrone L.; Chambrone L.A.; Lima L.A.	2010
[Consensus on peri-implant infections].	van Winkelhoff A.J.	2010
Dental implants in patients with rheumatoid arthritis: Clinical outcome and peri-implant findings	Krennmair G.; Seemann R.; Piehslinger E.	2010
Peri-implant diseases.	Heasman P.	2010
Short-term clinical retrospective study of implants in geriatric patients older than 70 years	Lee H.-J.; Kim Y.-K.; Park J.-Y.; Kim S.-G.; Kim M.-J.; Yun P.-Y.	2010
Platform switching for marginal bone preservation around dental implants: A systematic review and meta-analysis	Atieh M.A.; Ibrahim H.M.; Atieh A.H.	2010
Immediate loading of two implants supporting a magnet attachment-retained overdenture: One-year clinical study	Pae A.; Kim J.-W.; Kwon K.-R.	2010
Astra Tech, Brånemark, and ITI implants in the rehabilitation of partial edentulism: Two-year results	Bilhan H.; Kutay O.; Arat S.; Çekici A.; Cehreli M.C.	2010
Implant rehabilitation in grafted and native bone in patients affected by ectodermal dysplasia: Evaluation of 78 implants inserted in 8 patients	Grecchi F.; Zingari F.; Bianco R.; Zollino I.; Casadio C.; Carinci F.	2010
Three-dimensional bone and soft tissue requirements for optimizing esthetic results in compromised cases with multiple implants.	Ishikawa T.; Salama M.; Funato A.; Kitajima H.; Moroi H.; Salama H.; Garber D.	2010
Complex rehabilitation with homologous bone grafts and latest-generation zirconia implants	Borgonovo A.E.; Boninsegna R.; Dolci M.; Ghirlanda G.; Censi R.	2010
Bone resorption and complications in alveolar distraction osteogenesis	Ettl T.; Gerlach T.; Schüsselbauer T.; Gosau M.; Reichert T.E.; Driemel O.	2010

A technique for managing and accurate registration of periimplant soft tissues	Ntounis A.; Petropoulou A.	2010
[Implants have big disadvantages. Yes].	van Winkelhoff A.J.	2010
A single dose of zoledronic acid reverses the deleterious effects of glucocorticoids on titanium implant osseointegration	Carvas J.S.B.; Pereira R.M.R.; Caparbo V.F.; Fuller P.; Silveira C.A.; Lima L.A.P.; Bonfa E.; Mello S.B.V.	2010
Incidence, severity and predictors of congestive heart failure following primary PCI: The HORIZONS-AMI trial	Kelly D.J.; Gershlick T.; Witzenbichler B.; Guagliumi G.; Mehran R.; Stone G.W.	2010
Evaluation of everolimus-eluting and paclitaxel-eluting stents in patients with jailed side branches: The SPIRIT IV trial	Lansky A.J.; Yaqub M.; Sood P.; Applegate R.J.; Wang J.C.; Rizvi A.; Skelding K.; Smith R.S.; Cao S.; Hermiller J.B.; Sudhir K.; Stone G.W.	2010
A 5-year report from a multicentre randomised clinical trial: immediate non-occlusal versus early loading of dental implants in partially edentulous patients.	Capelli M.; Esposito M.; Zuffetti F.; Galli F.; Del Fabbro M.; Testroi T.	2010
A 12-year retrospective analytic study of the implant survival rate in 177 consecutive maxillary sinus augmentation procedures.	Cho-Lee G.Y.; Naval-Gias L.; Castrejon-Castrejon S.; Capote-Moreno A.L.; Gonzalez-Garcia R.; Sastre-Perez J.; Munoz-Guerra M.F.	2010
Implant placement in patients with oral bisphosphonate therapy: A case series	Shabestari G.O.; Shayesteh Y.S.; Khojasteh A.; Alikhasi M.; Moslemi N.; Aminian A.; Masaeli R.; Eslami B.; Treister N.S.	2010
Using the best available evidence to support clinical decisions in implant dentistry.	Faggion Jr. C.M.; Schmitter M.	2010
A 10-year retrospective analysis of radiographic bone-level changes of implants supporting single-unit crowns in periodontally compromised vs. periodontally healthy patients	Matarasso S.; Rasperini G.; Iorio Siciliano V.; Salvi G.E.; Lang N.P.; Aglietta M.	2010
Risk of implant failure and marginal bone loss in subjects with a history of periodontitis: A systematic review and meta-analysis	Safii S.H.; Palmer R.M.; Wilson R.F.	2010
Timing of implant placement after tooth extraction: immediate, immediate-delayed or delayed implants? A Cochrane systematic review.	Esposito M.; Grusovin M.G.; Polyzos I.P.; Felice P.; Worthington H.V.	2010
Immediate loading of unsplinted implants in the anterior mandible for overdentures: a case series.	Roe P.; Kan J.Y.; Rungcharassaeng K.; Lozada J.L.; Kleinman A.S.; Goodacre C.J.; Chen J.W.	2010
Mechanical anchorage and peri-implant bone formation of surface-modified zirconia in minipigs	Schliephake H.; Hefti T.; Schlottig F.; Gédet P.; Staedt H.	2010
Benefits of dental implants installed during ablative tumour surgery in oral cancer patients: A prospective 5-year clinical trial	Korfage A.; Schoen P.J.; Raghoebar G.M.; Roodenburg J.L.N.; Vissink A.; Reintsema H.	2010
A no-contact vibration device for measuring implant stability	Hayashi M.; Kobayashi C.; Ogata H.; Yamaoka M.; Ogiso B.	2010
Iliac crest bone grafting with sinus lift for restoration of severe posterior atrophy	Castagnola M.; Sacco R.; De Micheli L.	2010
Implant treatment software planning and guided flapless surgery with immediate provisional prosthesis delivery in the fully edentulous maxilla. A retrospective analysis of 15 consecutively treated patients.	Meloni S.M.; De Riu G.; Pisano M.; Cattina G.; Tullio A.	2010
Complications in 775 XLIF surgeries	Rodgers W.B.; Gerber E.J.	2010
Update on antiplatelets	Tepe G.	2010
Current guidelines for carotid stenosis management	Van Den Berg J.C.	2010
Transcatheter aortic valve replacement: State of the art and perspectives	Cribier A.	2010
A prospective multicenter study evaluating Elevate™ apical and posterior (AandP) for treatment of posterior and/or apical vaginal wall prolapse: One-Year follow-up	Lukban J.; VanDrie D.; Beyer R.; Erickson T.; Moore R.; Nguyen J.; Patel M.	2010
A review of clinical trials and registries in descending thoracic aortic aneurysms	Knepper J.; Upchurch G.R.	2010
Transfusion practice in orthopedic surgery	Waters J.H.; Wisniewski M.; Dyga R.M.; Yazer M.	2010
Management of failed laparoscopic pyeloplasty	Tan H.; Wolf J.S.	2010
Safety of ureteroscopy with biopsy or holmium laser lithotripsy in coagulopathic patients	Elkoushy M.A.; Violette P.; Andonian S.	2010
Interventions for replacing missing teeth: maintaining and recovering soft tissue health around dental implants.	Grusovin M.G.; Coulthard P.; Worthington H.V.; George P.; Esposito M.	2010
Peri-implantitis in a specialist clinic of periodontology clinical features and risk indicators	Carcuac O.; Jansson L.	2010
Restoration of peri-implant defects in immediate implant installations by Choukroun platelet-rich fibrin and silk fibroin powder combination graft	Jang E.-S.; Park J.-W.; Kweon H.; Lee K.-G.; Kang S.-W.; Baek D.-H.; Choi J.-Y.; Kim S.-G.	2010
Low pulse energy Nd:YAG laser irradiation exerts a biostimulative effect on different cells of the oral microenvironment: "An in vitro study"	Chellini F.; Sassoli C.; Nosi D.; Deledda C.; Tonelli P.; Zecchi-Orlandini S.; Formigli L.; Giannelli M.	2010
Soft tissue considerations in implant site development	Geurs N.C.; Vassilopoulos P.J.; Reddy M.S.	2010
Transmucosal healing around peri-implant defects: Crestal and subcrestal implant placement in dogs	Tran B.L.T.; Chen S.T.; Caiafa A.; Davies H.M.S.; Darby I.B.	2010
Relevant anatomic and biomechanical studies for implant possibilities on the atrophic maxilla: Critical appraisal and literature review	Rossetti P.H.O.; Bonachela W.C.; Rossetti L.M.N.	2010
Porcine bone used in sinus augmentation procedures: A 5-year retrospective clinical evaluation	Scarano A.; Piattelli A.; Assenza B.; Quaranta A.; Perrotti V.; Piattelli M.; Iezzi G.	2010
Prefabricated zirconium dioxide implant abutments for single-tooth replacement in the posterior region: Evaluation of peri-implant tissues and superstructures after 12 months of function	Nothdurft F.; Pospiech P.	2010

Considerations of implant abutment and crown contour: critical contour and subcritical contour.	Su H.; Gonzalez-Martin O.; Weisgold A.; Lee E.	2010
Influence of material of overdenture-retaining bar with vertical misfit on three-dimensional stress distribution	Abreu R.T.; Spazzin A.O.; Noritomi P.Y.; Consani R.L.X.; Mesquita M.F.	2010
A prospective multicenter study evaluating Elevate™ apical and posterior for treatment of posterior and/or apical vaginal wall prolapse: Twelve-month follow-up	Lukban J.; Roovers J.; Moore R.D.; Patel M.; Courtieu C.; Mayne C.; Flaherty J.; Liedl B.; Lange R.; Beyer R.	2010
A prospective multicenter study evaluating elevate™ apical and posterior for treatment of posterior and/or apical vaginal wall prolapse: Twelve-month follow-up	Lukban J.; Roovers J.; Moore R.D.; Patel M.; Courtieu C.; Mayne C.; Flaherty J.; Liedl B.; Lange R.; Beyer R.	2010
Ten concepts for ethical practice and psychiatric care of transplant recipients and living organ donors	Surman O.S.	2010
Long-term implant survival and success: a 10-16-year follow-up of non-submerged dental implants	Simonis P.; Dufour T.; Tenenbaum H.	2010
Nonsurgical treatment of retrograde peri-implantitis: a case report.	Waasdrop J.; Reynolds M.	2010
Vertical ridge augmentation with autogenous bone grafts 3 years after loading: resorbable barriers versus titanium-reinforced barriers. A randomized controlled clinical trial.	Merli M.; Lombardini F.; Esposito M.	2010
Immediate loading of two freestanding implants retaining a mandibular overdenture: 1-year pilot prospective study.	Liao K.Y.; Kan J.Y.; Rungcharassaeng K.; Lozada J.L.; Herford A.S.; Goodacre C.J.	2010
Bone alterations at implant-supported FDPs in relation to inter-unit distances: A 5-year radiographic study	Chang M.; Wennström J.L.	2010
Maximum use of the anterior maxillary buttress in severe maxillary atrophy with tilted, palatally positioned implants: a preliminary study.	Peñarrocha M.; Carrillo C.; Boronat A.; Peñarrocha M.	2010
Zirconia as a dental implant abutment material: a systematic review.	Nakamura K.; Kanno T.; Milleding P.; Ortengren U.	2010
Oral Bisphosphonate-Associated Osteonecrosis of the Jaw After Implant Surgery: A Case Report and Literature Review	Bedogni A.; Bettini G.; Totola A.; Saia G.; Nocini P.F.	2010
Influence of graft quality and marginal bone loss on implants placed in maxillary grafted sinus: a finite element study.	Inglam S.; Suebnukarn S.; Tharanon W.; Apatananon T.; Sitthiseripratip K.	2010
Retrospective study of implant-retained orbital prostheses: Implant survival and patient satisfaction	Karakoca Nemli S.; Aydin C.; Yilmaz H.; Sarisoy S.; Kasko Y.	2010
Low-level Er: YAG laser irradiation enhances osteoblast proliferation through activation of MAPK/ERK	Aleksic V.; Aoki A.; Iwasaki K.; Takasaki A.A.; Wang C.-Y.; Abiko Y.; Ishikawa I.; Izumi Y.	2010
Total shoulder replacement for osteoarthritis in patients 80 years of age and older	Foruria A.M.; Sperling J.W.; Ankem H.K.; Oh L.S.; Cofield R.H.	2010
Outcomes after implantation of the taxus paclitaxel-eluting stent in saphenous vein graft lesions: Results from the ARRIVE (TAXUS Peri-Approval Registry: A Multicenter Safety Surveillance) program	Brilakis E.S.; Lasala J.M.; Cox D.A.; Berger P.B.; Bowman T.S.; Starzyk R.M.; Dawkins K.D.	2010
Impact of successful and failed revascularization of chronic total occlusion on left ventricular function and infarct size	Paul G.A.; Zia M.; Connelly K.A.; Fefer P.; Strauss B.H.; Dick A.J.; Wright G.A.	2010
Clinical application of titanium mesh with fixed dental implant increases initial-stage stability	Wang L.-P.; Zhang S.-B.; Fan C.-B.; Fang Y.	2010
Pectin-coated titanium implants are well-tolerated in vivo	Kokkonen H.; Niiranen H.; Schols H.A.; Morra M.; Stenbäck F.; Tuukkanen J.	2010
Assessment of endpoints in studies on peri-implantitis treatment--a systematic review.	Faggion Jr. C.M.; Listl S.; Tu Y.K.	2010
Marginal bone level changes and prosthetic maintenance of mandibular overdentures supported by 2 implants: A 5-Year randomized clinical trial	Cehreli M.C.; Uysal S.; Akca K.	2010
Indirect sinus floor elevation for osseointegrated prostheses. A 10-year prospective study.	Agamy E.M.; Niedermeier W.	2010
Immediate occlusal versus non-occlusal loading of single zirconia implants. A multicentre pragmatic randomised clinical trial.	Cannizarro G.; Torchio C.; Felice P.; Leone M.; Esposito M.	2010
Factors affecting soft tissue level around anterior maxillary single-tooth implants	Nisapakultorn K.; Suphanantachat S.; Silkosessak O.; Rattanamongkolgul S.	2010
Evaluation of implants coated with rhBMP-2 using two different coating strategies: a critical-size supraalveolar peri-implant defect study in dogs	Lee J.; Decker J.F.; Polimeni G.; Cortella C.A.; Rohrer M.D.; Wozney J.M.; Hall J.; Susin C.; Wikesjö U.M.	2010
Treatment outcome and patient satisfaction with two adjacent implant-supported restorations in the esthetic zone.	Tymstra N.; Meijer H.J.; Stellingsma K.; Raghoebar G.M.; Vissink A.	2010
Influence of frequent clinical probing during the healing phase on healthy peri-implant soft tissue formed at different titanium implant surfaces: A histomorphometrical study in dogs	Schwarz F.; Mihatovic I.; Ferrari D.; Wieland M.; Becker J.	2010
Factors and techniques influencing peri-implant papillae	Chow Y.C.; Wang H.-L.	2010
A new treatment concept for immediate loading of implants inserted in the edentulous mandible.	Eccellente T.; Piombino M.; Piattelli A.; Perrotti V.; Iezzi G.	2010
A mandibular implant-supported fixed complete dental prosthesis in a patient with sjogren syndrome: Case report	Spinato S.; Soardi C.M.; Zane A.M.	2010
Periosteum-derived cells as an alternative to bone marrow cells for bone tissue engineering around dental implants. A histomorphometric study in beagle dogs	Ribeiro F.V.; Suaid F.F.; Ruiz K.G.S.; Salmon C.R.; Paparotto T.; Nociti Jr. F.H.; Sallum E.A.; Casati M.Z.	2010
Free gingival graft immobilization: a pilot study on a newly designed stent.	Hassani A.; Sadrimanesh R.; Vahdati S.A.; Sadr-eshkevari P.	2010
Biological width following immediate implant placement in the dog: Flap vs. flapless surgery	Blanco J.; Alves C.C.; Nuñez V.; Aracil L.; Muñoz F.; Ramos I.	2010
Evaluation of post-market performance of total hip arthroplasty devices using the kaiser permanente total joint replacement registry	Paxton E.; Ake C.; Namba R.	2010

Surgical management of secondary tricuspid valve regurgitation: Annulus, commissure, or leaflet procedure?	Navia J.L.; Nowicki E.R.; Blackstone E.H.; Brozzi N.A.; Nento D.E.; Atik F.A.; Rajeswaran J.; Gillinov A.M.; Svensson L.G.; Lytle B.W.	2010
A biofilm approach to detect bacteria on removed spinal implants	Sampedro M.F.; Huddleston P.M.; Piper K.E.; Karau M.J.; Dekutoski M.B.; Yaszemski M.J.; Currier B.L.; Mandrekar J.N.; Osmon D.R.; McDowell A.; Patrick S.; Steckelberg J.M.; Patel R.	2010
Impact of defect configuration on the clinical outcome following surgical regenerative therapy of peri-implantitis	Schwarz F.; Sahm N.; Schwarz K.; Becker J.	2010
Severity and pattern of peri-implantitis-associated bone loss	Fransson C.; Tomasi C.; Pikner S.S.; Gröndahl K.; Wennström J.L.; Leyland A.H.; Berglundh T.	2010
Influence of microgap location and configuration on peri-implant bone morphology in nonsubmerged implants: an experimental study in dogs.	Wang D.; Nagata M.J.; Bell M.; de Melo L.G.; Bosco A.F.	2010
Peri-implant soft tissue and bone crest alterations at fixed dental prostheses: a 3-year prospective study.	Chang M.; Wennström J.L.	2010
Comparison of two full-mouth approaches in the treatment of peri-implant mucositis: a pilot study.	Thöne-Mühling M.; Swierkot K.; Nonnenmacher C.; Mutters R.; Flores-de-Jacoby L.; Mengel R.	2010
Advantages and esthetic results of erbium, chromium:yttrium-scandium-gallium-garnet laser application in second-stage implant surgery in patients with insufficient gingival attachment: A report of three cases	Arnabat-Domínguez J.; Bragado-Novel M.; España-Tost A.J.; Berini-Aytés L.; Gay-Escoda C.	2010
Parathormone, but not alendronate, improves osseointegration of dental titanium implants into the tibia in a rabbit model of ovariectomy plus glucorticoid-induced osteoporosis	Largo R.; Almagro M.I.; Bellido M.; Román-Blas J.A.; Castañeda S.; Herrero-Beaumont G.; Cortez R.	2010
Ten-year results of a three-arm prospective cohort study on implants in periodontally compromised patients. Part 1: implant loss and radiographic bone loss.	Roccuzzo M.; De Angelis N.; Bonino L.; Aglietta M.	2010
Use of 4 immediately loaded zygomatic fixtures for retreatment of atrophic edentulous maxilla after complications of maxillary reconstruction	Kuabara M.R.; Ferreira E.J.; Gulinelli J.L.; Panzarini S.R.	2010
Peri-implant bone level around implants with platform-switched abutments.	Fickl S.; Zuhr O.; Stein J.M.; Hürzeler M.B.	2010
In vitro antibacterial efficacy of tetracycline hydrochloride adsorbed onto Bio-Oss® bone graft	Dashti A.; Ready D.; Salih V.; Knowles J.C.; Barralet J.E.; Wilson M.; Donos N.; Nazhat S.N.	2010
Treatment outcome of extraoral implants in the craniofacial region	Karayazgan-Saracoglu B.; Zulfikar H.; Atay A.; Gunay Y.	2010
Removable implant-prosthodontic rehabilitation of the edentulous mandible: five-year results of different prosthetic anchorage concepts.	Weinländer M.; Piehslinger E.; Krennmair G.	2010
Estradiol and G1 reduce infarct size and improve immunosuppression after experimental stroke	Zhang B.; Subramanian S.; Dziennis S.; Jia J.; Uchida M.; Akiyoshi K.; Migliati E.; Lewis A.D.; Vandenbark A.A.; Offner H.; Hurn P.D.	2010
Risk and Fate of Cerebral Embolism After Transfemoral Aortic Valve Implantation. A Prospective Pilot Study With Diffusion-Weighted Magnetic Resonance Imaging	Ghanem A.; Müller A.; Nähle C.P.; Kocurek J.; Werner N.; Hammerstingl C.; Schild H.H.; Schwab J.O.; Mellert F.; Fimmers R.; Nickenig G.; Thomas D.	2010
Fluid exudates from inflamed bone-anchored hearing aids demonstrate elevated levels of cytokines and biomarkers of tissue and bone metabolism.	Grant M.M.; Monksfield P.; Proops D.; Brine M.; Addison O.; Sammons R.L.; Matthews J.B.; Reid A.; Chapple I.L.	2010
A prospective, multicenter, randomized-controlled 5-year study of hybrid and fully etched implants for the incidence of peri-implantitis	Zetterqvist L.; Feldman S.; Rotter B.; Vincenzi G.; Wennström J.L.; Chierico A.; Stach R.M.; Kenealy J.N.	2010
Effects of soft tissue punch size on the healing of peri-implant tissue in flapless implant surgery	Lee D.-H.; Choi B.-H.; Jeong S.-M.; Xuan F.; Kim H.-R.; Mo D.-Y.	2010
Effectiveness of 980-mm diode and 1064-nm extra-long-pulse neodymium-doped yttrium aluminum garnet lasers in implant disinfection.	Gonçalves F.; Zanetti A.L.; Zanetti R.V.; Martelli F.S.; Avila-Campos M.J.; Tomazinho L.F.; Granjeiro J.M.	2010
Influence of interimplant distances and placement depth on peri-implant bone remodeling of adjacent and immediately loaded Morse cone connection implants: a histomorphometric study in dogs	Barros R.R.M.; Novaes A.B.; Muglia V.A.; Iezzi G.; Piattelli A.	2010
Immediate loading of full-arch fixed prostheses supported by axial and tilted implants for the treatment of edentulous atrophic mandibles.	Agliardi E.; Clericò M.; Ciancio P.; Massironi D.	2010
Do patients with rheumatoid arthritis exhibit differential cellular responses to implant wear debris and do the implant failure mechanisms differ from non-RA patients?	Vasudevan A.; DiCarlo E.F.; Wright T.; Chen D.; Goldring S.R.; Mandl L.A.	2010
Prophylaxis for venous thrombo-embolism in neurocritical care: A critical appraisal	Raslan A.M.; Fields J.D.; Bhardwaj A.	2010
Cell salvage and re-infusion: Experiences with a new device	Froessler B.; Bauze A.	2010
Incidence, range and clinical impact of hemoglobin decrease after transradial coronary stenting and maximal antiplatelet therapy	Bertrand O.F.; Rodes-Cabau J.; Larose E.; Rinfret S.; Dery J.-P.; Gleeton O.; Nguyen C.M.; Proulx G.; Roy L.; Costerousse O.; Poirier P.; Larochelliere R.D.	2010
Peri-implant parameters in head and neck reconstruction: Influence of extraoral skin or intraoral mucosa	Kwon Y.-D.; Karbach J.; Wagner W.; Al-Nawas B.	2010
Which device is more accurate to determine the stability mobility of dental implants? A human cadaver study	Tözüm T.F.; Bal B.T.; Turkyilmaz I.; Gülay G.; Tulunoglu I.	2010
Outcome of implant-supported single-tooth replacements performed by dental students. A 10-year clinical and radiographic retrospective study.	Bonde M.J.; Stokholm R.; Isidor F.; Schou S.	2010
Oral health maintenance dental implants.	Kracher C.M.; Smith W.S.	2010
Prospective clinical evaluation of 307 single-tooth morse taper-connection implants: a multicenter study.	Mangano C.; Mangano F.; Piattelli A.; Iezzi G.; Mangano A.; La Colla L.	2010
Osseointegration--the influence of implant surface.	Ivanovski S.	2010

Connective tissue grafts for thickening peri-implant tissues at implant placement. One-year results from an explanatory split-mouth randomised controlled clinical trial.	Wiesner G.; Esposito M.; Worthington H.; Schlee M.	2010
Clinical outcome of root-shaped dental implants of various diameters: 5-year results.	Krennmair G.; Seemann R.; Schmidinger S.; Ewers R.; Piehslinger E.	2010
Postsurgical care for rehabilitation with implant-retained extraoral prostheses	Goiato M.C.; Takamiya A.S.; Alves L.M.N.; Dos Santos D.M.	2010
Early implant loading after vertical ridge augmentation (VRA) using e-PTFE titanium-reinforced membrane and nano-structured hydroxyapatite: 2-year prospective study.	Canullo L.; Sisti A.	2010
The microbiota associated with implants restored with platform switching: A preliminary report	Canullo L.; Quaranta A.; Teles R.P.	2010
Tooth-implant connection: A bibliographic review	Hita-Carrillo C.; Hernández-Aliaga M.; Calvo-Guirado J.-L.	2010
Evaluation of 316 narrow diameter implants followed for 5-10 years: A clinical and radiographic retrospective study	Arsan V.; Bölükbaş N.; Ersanli S.; Özdemir T.	2010
Early loading of implants in vertically augmented bone with non-resorbable membranes and deproteinised anorganic bovine bone. An uncontrolled prospective cohort study.	Todisco M.	2010
Early loading of implants with fixed dental prostheses in edentulous mandibles: 4.5-year clinical results from a prospective study	Schwarz S.; Gabbert O.; Hassel A.J.; Schmitter M.; Séché C.; Rammelsberg P.	2010
Sinus bone graft using new alloplastic bone graft material (Osteon)-II: clinical evaluation	Bae J.-H.; Kim Y.-K.; Kim S.-G.; Yun P.-Y.; Kim J.-S.	2010
Osseointegration of orthodontic micro-screws after immediate and early loading	Zhang L.; Zhao Z.; Li Y.; Wu J.; Zheng L.; Tang T.	2010
The immunology of fibrosis: innate and adaptive responses	Wick G.; Backovic A.; Rabensteiner E.; Plank N.; Schwentner C.; Sgonc R.	2010
Prevalence of peri-implantitis related to severity of the disease with different degrees of bone loss	Koldsland O.C.; Scheie A.A.; Aass A.M.	2010
Comparison of the effects of treatment of peri-implant infection in animal and human studies: Systematic review and meta-analysis	Faggion Jr. C.M.; Chambrone L.; Gondim V.; Schmitter M.; Tu Y.-K.	2010
Rationale for esthetic tissue preservation of a fresh extraction socket by an implant treatment concept simulating a tooth replantation	Trimpou G.; Weigl P.; Krebs M.; Parvini P.; Nentwig G.-H.	2010
Dental implants placed into alveolar clefts reconstructed with tongue flaps and bone grafts	Sándor G.K.B.; Carmichael R.P.; Brkovic B.M.B.	2010
Assessing peri-implant tissue infection prevention in a percutaneous model	Perry E.L.; Beck J.P.; Williams D.L.; Bloebaum R.D.	2010
Implant dentistry in disabled patients rehabilitation	Fabbri G.; Cristini A.; Pozzani E.; Guerrini F.	2010
Psychopathology in patients with icds over time: Results of a prospective study	Kapa S.; Rotondi-Trevisan D.; Mariano Z.; Aves T.; Irvine J.; Dorian P.; Hayes D.L.	2010
Perioperative chemotherapy with bevacizumab and liver resection for colorectal cancer liver metastasis	Chaudhury P.; Hassanain M.; Bouganim N.; Salman A.; Kavan P.; Metrakos P.	2010
Design of the carotid revascularization endarterectomy vs. stenting trial (CREST)	Sheffet A.J.; Roubin G.; Howard G.; Howard V.; Moore W.; Meschia J.F.; Hobson II R.W.; Brott T.G.	2010
Extracorporeal shock wave therapy: A potential adjuvant treatment for peri-implantitis	Li X.; Chen M.; Li L.; Qing H.; Zhu Z.	2010
Implant prosthodontic rehabilitation of patients with rheumatic disorders: a case series report.	Weinlander M.; Krennmair G.; Piehslinger E.	2010
Quantitative assessment of orthodontic mini-implant displacement: the effect of initial force application.	Holst A.I.; Karl M.; Karolczak M.; Goellner M.; Holst S.	2010
Marginal bone loss and prosthetic maintenance of bar-retained implant-supported overdentures: a prospective study.	Akca K.; Cehreli M.C.; Uysal S.	2010
Numerical simulation of the effect of time-to-loading on peri-implant bone	Akça K.; Eser A.; Canay S.	2010
Dental implants placed simultaneously with bone grafts in horizontal defects: a clinical retrospective study with 37 patients.	Boronat A.; Carrillo C.; Penarrocha M.; Pennarocha M.	2010
Endosseous implant anchorage is critically dependent on mechanostructural determinants of peri-implant bone trabeculae	Gabet Y.; Kohavi D.; Voide R.; Mueller T.L.; Müller R.; Bab I.	2010
Technical problems and complications in the removal of the less invasive stabilization system	Suzuki T.; Smith W.R.; Stahel P.F.; Morgan S.J.; Baron A.J.; Hak D.J.	2010
The correlation between crown-implant ratios and marginal bone resorption: a preliminary clinical study.	Gomez-Polo M.; Bartens F.; Sala L.; Tamini F.; Celemin A.; Del Rio J.	2010
Evaluation of transobturator tapes (E-TOT) study: randomised prospective single-blinded study comparing inside-out vs. outside-in transobturator tapes in management of urodynamic stress incontinence: Short term outcomes	Abdel-fattah M.; Ramsay I.; Pringle S.; Hardwick C.; Ali H.	2010
Stress and periodontal health	Strooker H.; de Geus E.; van der Reijden W.A.; Laine M.L.; van Winkelhoff A.J.	2010
Clinical and radiographic study of implant treatment outcome in periodontally susceptible and non-susceptible patients: A prospective long-term study	De Boever A.L.; Quirynen M.; Coucke W.; Theuniers G.; De Boever J.A.	2009
The effect of a triclosan dentifrice on mucositis in subjects with dental implants: A six-month clinical study	Ramberg P.; Lindhe J.; Botticelli D.; Botticelli. A.	2009
History of treated periodontitis and smoking as risks for implant therapy.	Heitz-Mayfield L.J.; Huynh-Ba G.	2009
Vertical ridge augmentation of the atrophic posterior mandible with interpositional bloc grafts: Bone from the iliac crest vs. bovine anorganic	Felice P.; Marchetti C.; Iezzi G.; Piattelli A.; Worthington H.; Pellegrino G.; Esposito M.	2009

bone. Clinical and histological results up to one year after loading from a randomized-controlled clinical trial		
Zirconium dioxide implant abutments for posterior single-tooth replacement: First results	Nothdurft F.P.; Pospiech P.R.	2009
Endodontic treatment of teeth induces retrograde peri-implantitis	Zhou W.; Han C.; Li D.; Li Y.; Song Y.; Zhao Y.	2009
A case of rapidly progressive peri-implantitis around a short sintered porous-surfaced implant.	Malchiodi L.; Cucchi A.; Ghensi P.; Bondì V.	2009
Oral health and oral implant status in edentulous patients with implant-supported dental prostheses who are receiving long-term nursing care.	Isaksson R.; Becktor J.P.; Brown A.; Laurizohn C.; Isaksson S.	2009
Clinical outcome of submerged vs. non-submerged implants placed in fresh extraction sockets	Cordaro L.; Torsello F.; Roccuzzo M.	2009
Clinical and esthetic outcomes of implants placed in postextraction sites.	Chen S.T.; Buser D.	2009
Implant loading protocols for partially edentulous maxillary posterior sites.	Roccuzzo M.; Aglietta M.; Cordaro L.	2009
Implant loading protocols for the partially edentulous posterior mandible.	Cordaro L.; Torsello F.; Roccuzzo M.	2009
Comparative analysis of peri-implant marginal bone loss based on microthread location: A 1-year prospective study after loading	Song D.-W.; Lee D.-W.; Kim C.-K.; Park K.-H.; Moon I.-S.	2009
Immediate implant placement after removal of a failed implant: a clinical and histological case report.	Covani U.; Marconcini S.; Crespi R.; Barone A.	2009
Early loading of nonsubmerged titanium implants with a chemically modified sand-blasted and acid-etched surface: 6-month results of a prospective case series study in the posterior mandible focusing on peri-implant crestal bone changes and implant stability quotient (ISQ) values	Bornstein M.M.; Hart C.N.; Halbritter S.A.; Morton D.; Buser D.	2009
Comparative analysis of immediate functional loading and immediate nonfunctional loading to traditional healing periods: A 5-year follow-up of 550 dental implants	Degidi M.; Iezzi G.; Perrotti V.; Piattelli A.	2009
CaPO4 blasted implants inserted into iliac crest homologue frozen grafts.	Franco M.; Rigo L.; Viscione A.; De Santis B.; Tropina E.; Brunelli G.; Guidi R.; Avantaggiato A.; Carinci F.	2009
Alveolar bone regeneration by transplantation of periodontal ligament stem cells and bone marrow stem cells in a canine peri-implant defect model: A pilot study	Kim S.-H.; Kim K.-H.; Seo B.-M.; Koo K.-T.; Kim T.-I.; Seol Y.-J.; Ku Y.; Rhyu I.-C.; Chung C.-P.; Lee Y.-M.	2009
Bone-anchored titanium implants for auricular rehabilitation: Case report and review of literature	Gumieiro E.H.; Dib L.L.; Jahn R.S.; Dos Santos Jr. J.F.; Nannmark U.; Granström G.; Abrahão M.	2009
A 3-year clinical and radiographic study of implants placed simultaneously with maxillary sinus floor augmentations using a new nanocrystalline hydroxyapatite.	Heinemann F.; Mundt T.; Biffar R.; Gedrange T.; Goetz W.	2009
Soft tissue stability with immediate implants and concave abutments.	Redemagni M.; Cremonesi S.; Garlini G.; Maiorana C.	2009
Influence of the implant cervical topography on the crestal bone resorption and immediate implant survival.	Heinemann F.; Bourauel C.; Hasan I.; Gedrange T.	2009
Edentulous site enhancement: A regenerative approach to the management of edentulous areas. Part 2: Peri-implant tissues	Calesini G.; Micarelli C.; Coppe S.; Scipioni A.	2009
Methods to avoid dental implant failure and disadvantages	Schmage P.	2009
Bacterial adhesion on smooth and rough titanium surfaces after treatment with different instruments	Duarte P.M.; Reis A.F.; Freitas P.M.D.; Ota-Tsuzuki C.	2009
Management of Vascular and Nonvascular Complications After Renal Transplantation	Hedegard W.; Saad W.E.A.; Davies M.G.	2009
Guidelines for patient selection and performance of carotid artery stenting	Bladin C.; Chambers B.; New G.; Denton M.; Lawrence-Brown M.; Levi C.; David Muller D.; Davis S.; Donnan G.; Crimmins D.; Frayne J.; Phatouros C.; Scroop R.; Harrington T.	2009
Economic analysis of dabigatran etexilate in the primary prevention of venous thromboembolism after a total hip and knee arthroplasty	Monreal M.; González-Rojas N.; Vieta A.; Wolowacz S.E.	2009
Quantitation of changes in VWF and FVIII following elective orthopedic surgery in normal individuals	Kahlon A.; Grabell J.; Tuttle A.; Engen D.; Hopman W.; Lillicrap D.; James P.	2009
Effect of surface topography of screw-shaped titanium implants in humans on clinical and radiographic parameters: A 12-year prospective study	Vroom M.G.; Sipos P.; De Lange G.L.; Gründemann L.J.M.M.; Timmerman M.F.; Loos B.G.; Van Der Velden U.	2009
Microsurgical reconstruction of the jaw with fibular grafts and implants.	Raoul G.; Ruhin B.; Briki S.; Lauwers L.; Haurou Patou G.; Capet J.P.; Maes J.M.; Ferri J.	2009
Implant-supported fixed cantilever prosthesis in partially edentulous jaws: A cohort prospective study	Romeo E.; Tomasi C.; Finini I.; Casentini P.; Lops D.	2009
The use of extraoral implants for distal-extension removable dentures: a clinical evaluation up to 8 years.	Minoretti R.; Triaca A.; Saulacic N.	2009
Laser wavelengths and oral implantology	Romanos G.E.; Gutknecht N.; Dieter S.; Schwarz F.; Crespi R.; Sculean A.	2009
One-step oral rehabilitation by means of implants' insertion, Le Fort I, grafts, and immediate loading.	Grecchi F.; Zollino I.; Parafioriti A.; Mineo G.; Pricolo A.; Carinci F.	2009
Facial gingival tissue stability after connective tissue graft with single immediate tooth replacement in the esthetic zone: consecutive case report.	Kan J.Y.; Rungcharassaeng K.; Morimoto T.; Lozada J.	2009
The potential of the horizontal ramus of the mandible as a donor site for block and particular grafts in pre-implant surgery	Soehardi A.; Meijer G.J.; Strooband V.F.M.H.; de Koning M.; Stoelinga P.J.W.	2009
Maternal complications and pregnancy outcome in women with mechanical prosthetic heart valves treated with enoxaparin	McLintock C.; McCowan L.M.E.; North R.A.	2009

The Carotid Revascularization Endarterectomy vs. Stenting Trial completes randomization: Lessons learned and anticipated results	Lal B.K.; Brott T.G.	2009
Five-year evaluation of the influence of keratinized mucosa on peri-implant soft-tissue health and stability around implants supporting full-arch mandibular fixed prostheses	Schrott A.R.; Jimenez M.; Hwang J.-W.; Fiorellini J.; Weber H.-P.	2009
Influence of abutment design on clinical status of peri-implant tissues	Taiyeb-Ali T.B.; Toh C.G.; Siar C.H.; Seiz D.; Ong S.T.	2009
Assessment of replication of research evidence from animals to humans in studies on peri-implantitis therapy	Faggion Jr. C.M.; Schmitter M.; Tu Y.-K.	2009
Dimensional ridge alterations following immediate implant placement in molar extraction sites: A six-month prospective cohort study with surgical re-entry	Matarasso S.; Salvi G.E.; Iorio Siciliano V.; Cafiero C.; Blasi A.; Lang N.P.	2009
Application of antimicrobial photodynamic therapy in periodontal and peri-implant diseases	Takasaki A.A.; Aoki A.; Mizutani K.; Schwarz F.; Sculean A.; Wang C.-Y.; Koshy G.; Romanos G.; Ishikawa I.; Izumi Y.	2009
The impact of laser application on periodontal and peri-implant wound healing	Schwarz F.; Aoki A.; Sculean A.; Becker J.	2009
Economic analysis of dabigatran etexilate for the primary prevention of venous thromboembolism following total hip or knee replacement in Spain	Gonzalez-Rojas N.; Vieta A.; Monreal M.; Wolowacz S.E.	2009
A critical and ironic instant in science and attitude of health personnel	De Bruyn H.	2009
Implant periapical lesion a case series report	Rosendahl K.; Dahlberg G.; Kisch J.; Nilner K.	2009
Bone ingrowth on the surface of endosseous implants. Part 1: Mathematical model	Moreo P.; García-Aznar J.M.; Doblaré M.	2009
Re-osseointegration on previously contaminated surfaces: A systematic review	Renvert S.; Polyzois I.; Maguire R.	2009
Apical peri-implantitis: Possible predisposing factors, case reports, and surgical treatment suggestions	Dahlin C.; Nikfarid H.; Alsén B.; Kashani H.	2009
Surgical regenerative treatment of peri-implantitis lesions using a nanocrystalline hydroxyapatite or a natural bone mineral in combination with a collagen membrane: A four-year clinical follow-up report	Schwarz F.; Sahm N.; Bieling K.; Becker J.	2009
Plaque accumulation on exposed titanium surfaces and peri-implant tissue behavior. A preliminary 1-year clinical study.	Baldi D.; Menini M.; Pera F.; Ravera G.; Pera P.	2009
Implant surfaces and design (Working Group 4)	Lang N.P.; Jepsen S.	2009
Periodontal and periimplant maintenance: a critical factor in long-term treatment success.	Shumaker N.D.; Metcalf B.T.; Toscano N.T.; Holtzclaw D.J.	2009
Biomimetic ceramics in implant dentistry	Padial-Molina M.; Galindo-Moreno P.; Avila-Ortiz G.	2009
Clinical outcomes of GBR procedures to correct peri-implant dehiscences and fenestrations: A systematic review	Chiapasco M.; Zaniboni M.	2009
To what extent does the crown-implant ratio affect the survival and complications of implant-supported reconstructions? A systematic review	Blanes R.J.	2009
Survival and complication rates of implant-supported fixed partial dentures with cantilevers: A systematic review	Zurdo J.; Romão C.; Wennström J.L.	2009
Biomechanical study of mandible bone supporting a four-implant retained bridge. Finite element analysis of the influence of bone anisotropy and foodstuff position	Bonnet A.S.; Postaire M.; Lipinski P.	2009
Problems with dental implants that were placed on vertically distracted fibular free flaps after resection: A report of six cases	Lizio G.; Corinaldesi G.; Pieri F.; Marchetti C.	2009
Mandibular overdentures supported by two brånemark, IMZ or ITI implants: A ten-year prospective randomized study	Meijer H.J.A.; Raghoebar G.M.; Batenburg R.H.K.; Vissink A.	2009
Factors that influence the position of the peri-implant soft tissues: A review	Sorní-Bröker M.; Peñarrocha-Diago M.; Peñarrocha-Diago M.	2009
Redefining the success rate of bulking agents: Role of composite endpoints	Renganathan A.; Errichiello L.; Robinson D.; Cardozo L.D.	2009
Evaluation of peri-implant tissue in nonsubmerged dental implants: a multicenter retrospective study	Kim Y.-K.; Kim S.-G.; Oh H.-K.; Choi Y.-G.; Cho Y.-S.; Oh Y.-H.; Son J.-S.; Ong J.L.	2009
Determinants of heterotopic ossification after total hip replacement surgery	Fransen M.; Neal B.; Cameron I.D.; Crawford R.; Tregonning G.; Winstanley J.; Norton R.	2009
Maxillary sinus floor elevation using the (transalveolar) osteotome technique with or without grafting material. Part I: Implant survival and patients' perception	Pjetursson B.E.; Rast C.; Brägger U.; Schmidlin K.; Zwahlen M.; Lang N.P.	2009
Transalveolar maxillary sinus floor elevation using osteotomes with or without grafting material. Part II: Radiographic tissue remodeling	Pjetursson B.E.; Ignjatovic D.; Matuliene G.; Brägger U.; Schmidlin K.; Lang N.P.	2009
Professional and patient-based evaluation of oral rehabilitation in patients with tooth agenesis	Dueled E.; Gotfredsen K.; Damsgaard M.T.; Hede B.	2009
Comparison between implants inserted with and without computer planning and custom model coordination	Danza M.; Zollino I.; Carinci F.	2009
Peri-procedural anticoagulation management of mechanical prosthetic heart valve patients	Daniels P.R.; McBane R.D.; Litin S.C.; Ward S.A.; Hodge D.O.; Dowling N.F.; Heit J.A.	2009
Bacterial colonization of oral implants from nondental sources	Emrani J.; Chee W.; Slots J.	2009
The use of CO2 laser in the treatment of peri-implantitis	Romanos G.; Ko H.-H.; Froum S.; Tarnow D.	2009
Five-year results of fixed implant-supported rehabilitations with distal cantilevers for the edentulous mandible	Gallucci G.O.; Doughtie C.B.; Hwang J.W.; Fiorellini J.P.; Weber H.-P.	2009

A review of dental implants and infection	Pye A.D.; Lockhart D.E.A.; Dawson M.P.; Murray C.A.; Smith A.J.	2009
Bone-integration and bone-biomimicry of dental implants: Role of peri-implant infections	Sánchez-Espinel J.A.; Sánchez-Porto A.	2009
Clinical outcome of overdenture treatment on two nonsubmerged and nonsplinted astra tech microthread™ Implants	De Bruyn H.; Besseler J.; Raes F.; Vaneker M.	2009
Clinical evaluation of two-implant-supported single molar restoration	Bi W.; Yu Y.; Wang Q.; Gong Y.; Sun J.	2009
The biologic width around titanium implants: histometric analysis of the implantogingival junction around immediately and early loaded implants.	Bakaeen L.; Quinlan P.; Schoolfield J.; Lang N.P.; Cochran D.L.	2009
Influence of early cover screw exposure on crestal bone loss around implants: intraindividual comparison of bone level at exposed and non-exposed implants	Kim T.-H.; Lee D.-W.; Kim C.-K.; Park K.-H.; Moon I.-S.	2009
Bone quality and the immediate loading of implants-critical aspects based on literature, research, and clinical experience	Romanos G.E.	2009
Miniscrews in orthodontics	Peuch-Lestrade G.-R.; Le Marie M.; Guillaumot G.; Decker A.	2009
Clinical and radiographic comparison of implants in regenerated or native bone: 5-year results	Benić G.I.; Jung R.E.; Siegenthaler D.W.; Hämmerle C.H.F.	2009
Er,Cr:YSGG laser assisted surgical treatment of peri-implantitis with one year re-entry and 18 months follow-up: A case report	Azzeh M.	2009
Short-term bone level observations associated with platform switching in immediately placed and restored single maxillary implants: a preliminary report.	Canullo L.; Goglia G.; Iurlaro G.; Iannello G.	2009
A systematic review of the survival and complication rates of implant supported fixed dental prostheses with cantilever extensions after an observation period of at least 5 years	Aglietta M.; Siciliano V.I.; Zwahlen M.; Brägger U.; Pjetursson B.E.; Lang N.P.; Salvi G.E.	2009
Development of novel implant abutments using the shape memory alloy nitinol: preliminary results.	Pautke C.; Kolk A.; Brokate M.; Wehrstedt J.C.; Kneissl F.; Miethke T.; Steinhauser E.; Horch H.H.; Deppe H.	2009
Comparison of five parameters as risk factors for peri-mucositis.	Karbach J.; Callaway A.; Kwon Y.D.; d'Hoedt B.; Al-Nawas B.	2009
Soft tissues healing at immediate transmucosal implants placed into molar extraction sites with buccal self-contained dehiscences. A 12-month controlled clinical trial	Siciliano V.I.; Salvi G.E.; Matarasso S.; Cafiero C.; Blasi A.; Lang N.P.	2009
Dental implants in persons with severe epilepsy and multiple disabilities: a long-term retrospective study.	Cune M.S.; Strooker H.; van der Reijden W.A.; de Putter C.; Laine M.L.; Verhoeven J.W.	2009
Gingivomorphometry - Esthetic evaluation of the crown-mucogingival complex: A new method for collection and measurement of standardized and reproducible data in oral photography	Weinländer M.; Lekovic V.; Spadijer-Gostovic S.; Milicic B.; Krennmair G.; Plenk H.	2009
Evaluation of Er;Cr:YSGG laser effects on debridement of dental implants. (SEM evaluation)	Fekrazd R.; Gholami G.A.; Lotfi Gh.; Kalhori K.	2009
Laser application in oral surgery and in periodontics	Zeinoun T.	2009
A novel design for a randomized trial to test if icd implantation without any defibrillation testing is as effective as standard treatment	Healey J.S.; Hohnloser S.H.; Glikson M.; Neuzner J.; Kautzner J.; Gadler F.; Mabo P.; Vinolas X.; Van Erven L.; Appl U.; Gilkerson J.; Connolly S.J.	2009
IVUS-guided management of late stent malaposition with peri-stent restenosis with coronary artery aneurysm following drug-eluting stent implantation (paxlitaxel-eluting stent)	Lanjewar C.P.; Sharma A.; Sheth T.	2009
Primary stenting for recurrent stenosis following carotid endarterectomy	Bussière M.; Pelz D.; Lownie S.	2009
Secondary stroke prevention strategies for the oldest patients: Possibilities and challenges	Bushnell C.D.; Colón-Emeric C.S.	2009
Effects of decontamination and implant surface characteristics on re-osseointegration following treatment of peri-implantitis	Parlar A.; Bosshardt D.D.; Etiner D.; Schafroth D.; Ünsal B.; Hayta C.; Lang N.P.	2009
Successful outcome of splinted implants supporting a 'planned' maxillary overdenture: A retrospective evaluation and comparison with fixed full dental prostheses	Sanna A.; Nuytens P.; Naert I.; Quirynen M.	2009
Infectious complications with dental implants	van Winkelhoff A.J.; van der Avoort P.G.; Wismeijer D.	2009
Use of sintered porous-surfaced dental implants to restore single teeth in the maxilla: a 7- to 9-year follow-up.	MacDonald K.; Pharoah M.; Todescan R.; Deporter D.	2009
Effects of implant geometry, surface properties, and TGF-β1 on peri-implant bone response: An experimental study in goats	Schouten C.; Meijer G.J.; Van Den Beucken J.J.J.P.; Spauwen P.H.M.; Jansen J.A.	2009
Clinical and microbiological determinants of ailing dental implants	Tabanella G.; Nowzari H.; Slots J.	2009
Clinical assessment of peri-implant tissues in patients with varying severity of chronic periodontitis	Aloufi F.; Bissada N.; Ficara A.; Faddoul F.; Al-Zahrani M.S.	2009
Clinical peri-implant sounding accuracy in the presence of chronic inflammation of peri-implant tissues. Clinical observation study.	Romeo E.; Lops D.; Storelli S.; Ghisolfi M.	2009
Role of fibroblast populations in peri-implantitis.	Bordin S.; Flemmig T.F.; Verardi S.	2009
Evaluation of peri-implant tissue response according to the presence of keratinized mucosa	Kim B.-S.; Kim Y.-K.; Yun P.-Y.; Yi Y.-J.; Lee H.-J.; Kim S.-G.; Son J.-S.	2009
Subcrestal placement of two-part implants	Welander M.; Abrahamsson I.; Berglundh T.	2009
Influence of defect depth on resonance frequency analysis and insertion torque values for implants placed in fresh extraction sockets: A human cadaver study	Turkyilmaz I.; Sennerby L.; Yilmaz B.; Bilecenoglu B.; Ozbek E.N.	2009

Interforaminal implant therapy using a split skin graft--treatment outcome after 13 years.	Karl M.; Fenner M.; Amann K.; Heckmann J.G.; Wichmann M.G.; Heckmann S.M.	2009
Bivalirudin and Clopidogrel With and Without Eptifibatide for Elective Stenting: Effects on Platelet Function, Thrombelastographic Indexes, and Their Relation to Periprocedural Infarction. Results of the CLEAR PLATELETS-2 (Clopidogrel With Eptifibatide to Arrest the Reactivity of Platelets) Study	Gurbel P.A.; Bliden K.P.; Saucedo J.F.; Suarez T.A.; DiChiara J.; Antonino M.J.; Mahla E.; Singla A.; Herzog W.R.; Bassi A.K.; Hennebry T.A.; Gesheff T.B.; Tantry U.S.	2009
Eradication of multidrug-resistant Acinetobacter baumannii in a female patient with total hip arthroplasty, with debridement and retention: A case report	Beieler A.M.; Belknap R.W.; Dayton M.R.; Price C.S.; Morgan S.J.	2009
Biomaterial and antibiotic strategies for peri-implantitis	Norowski Jr. P.A.; Bumgardner J.D.	2009
Peri-implantitis in partially edentulous patients: Association with inadequate plaque control	Serino G.; Ström C.	2009
Changes in inflammatory mediators in peri-implant fluid after implant insertion	Venza M.; Visalli M.; Lo Giudice G.; Cicciù M.; Passi P.; Teti D.	2009
A 12-month evaluation of nitrite oxide metabolism around immediate and conventionally loaded dental implants	Güncü G.N.; Tözüm T.F.; Güncü M.B.; Yamalik N.; Tümer C.	2009
Theoretical knowledge in implant dentistry for undergraduate students.	Hicklin S.P.; Albrektsson T.; Hämmerle C.H.	2009
The rationale for the introduction of implant dentistry into the dental curriculum.	Lang N.P.; De Bruyn H.	2009
Long-term effects of magnetron-sputtered calcium phosphate coating on osseointegration of dental implants in non-human primates	Alexander F.; Christian U.; Stefan T.; Christoph V.; Reinhard G.; Georg W.	2009
Presentation and outcomes after percutaneous intervention for acute myocardial infarction in the NLHBI Dynamic Registry: Saphenous vein grafts versus native coronary arteries	Iqbal S.N.; Selzer F.; Naidu S.S.; Doucet S.; Faxon D.; Jacobs A.; Slater J.	2009
Coronary revascularization approaches combining total endoscopic bypass grafting and percutaneous interventions using different timing protocols	Friedrich G.J.; Bonaros N.; Schachner T.; Laufer G.; Pachinger O.; Bonatti J.O.	2009
Bleeding on probing and pocket probing depth in relation to probing pressure and mucosal health around oral implants	Gerber J.A.; Tan W.C.; Balmer T.E.; Salvi G.E.; Lang N.P.	2009
Morphogenesis of the peri-implant mucosa: a comparison between flap and flapless procedures in the canine mandible	You T.-M.; Choi B.-H.; Li J.; Xuan F.; Jeong S.-M.; Jang S.-O.	2009
360° peri-implant, keratinised, soft-tissue grafting with stereolithographic-aided dressing plate	Kwasnicki A.; Butterworth C.	2009
Inflammatory Implant Periapical Lesion: Etiology, Diagnosis, and Treatment-Presentation of 7 Cases	Peñarrocha-Diago M.; Boronat-Lopez A.; García-Mira B.	2009
Outcome evaluation of early placed maxillary anterior single-tooth implants using objective esthetic criteria: A cross-sectional, retrospective study in 45 patients with a 2- to 4-year follow-up using pink and white esthetic scores	Belser U.C.; Grütter L.; Vailati F.; Bornstein M.M.; Weber H.-P.; Buser D.	2009
Early implant placement with simultaneous guided bone regeneration following single-tooth extraction in the esthetic zone: 12-Month results of a prospective study with 20 consecutive patients	Buser D.; Halbritter S.; Hart C.; Bornstein M.M.; Grütter L.; Chappuis V.; Belser U.C.	2009
Zygoma implants for midfacial prosthetic rehabilitation using telescopes: 9-year follow-up.	Landes C.A.; Paffrath C.; Koehler C.; Thai V.D.; Stübinger S.; Sader R.; Lauer H.C.; Piwowarczyk A.	2009
An analysis of the effect of a vent hole on excess cement expressed at the crown-abutment margin for cement-retained implant crowns	Patel D.; Invest J.C.F.; Tredwin C.J.; Setchell D.J.; Moles D.R.	2009
Success rate of second-generation palatal implants	Jung B.A.; Kunkel M.; Göllner P.; Liechti T.; Wehrbein H.	2009
Side-branch occlusion with XIENCE V everolimus-eluting and TAXUS EXPRESS2 paclitaxel-eluting stents: 2-year results from the SPIRIT III randomized trial	Lansky A.; Applegate R.; Hermiller J.B.; Cristea E.; Dressler O.; Sudhir K.; Doostzadeh J.; Su X.; Koo K.; Stone G.	2009
A new short uncemented, proximally fixed anatomic femoral implant with a prominent lateral flare: Design rationals and study design of an international clinical trial	Renkawitz T.; Santori F.S.; Grifka J.; Valverde C.; Morlock M.M.; Learmonth I.D.	2008
Possible impact of inflammatory status on C-telopeptide pyridinoline cross-links of Type i collagen and osteocalcin levels around oral implants with peri-implantitis: A controlled clinical trial	Tümer C.; Aksoy Y.; Güncü G.N.; Nohutcu R.M.; Kilinc K.; Tözüm T.F.	2008
Single-tooth Morse taper connection implants after 1 year of functional loading: a multicentre study on 302 patients.	Mangano C.; Mangano F.; Piatelli A.; Lezzi G.; Mangano A.; La Colla L.; Mangano A.	2008
Osteoradionecrosis and dental implants	Ben Slama L.; Hasni W.; De Labrouhe C.; Bado F.; Bertrand J.-C.	2008
Implant treatment of patients with edentulous jaws: A 20-year follow-up	Åstrand P.; Ahlqvist J.; Gunne J.; Nilson H.	2008
Effect of compromised cortical bone on implant load distribution	Akça K.; Fanuscu M.I.; Caputo A.A.	2008
Peri-implantitis	Vervaeke S.; De Bruyn H.	2008
Long-term marginal bone loss around single dental implants affected by current and past smoking habits	Levin L.; Hertzberg R.; Har-Nes S.; Schwartz-Arad D.	2008
Flap vs. flapless surgical techniques at immediate implant function in predominantly soft bone for rehabilitation of partial edentulism: a prospective cohort study with follow-up of 1 year.	Maló P.; Nobre M.	2008
Reproducibility of the pink esthetic score - Rating soft tissue esthetics around single-implant restorations with regard to dental observer specialization	Gehrke P.; Lobert M.; Dhom G.	2008

Immediate versus early loading of 7-mm-long flapless-placed single implants: a split-mouth randomised controlled clinical trial.	Cannizzaro G.; Leone M.; Torchio C.; Viola P.; Esposito M.	2008
Halitosis: a review of associated factors and therapeutic approach.	Cortelli J.R.; Barbosa M.D.; Westphal M.A.	2008
Pulsed electromagnetic fields as adjuvant therapy in bone healing and peri-implant bone formation: an experimental study in rats.	Grana D.R.; Marcos H.J.; Kokubu G.A.	2008
Can gender differences be evaluated in a rhesus macaque (macaca mulatto) model of focal cerebral ischemia?	Murphy S.J.; Kirsch J.R.; Zhang W.; Grafe M.R.; West G.A.; Del Zoppo G.J.; Traystman R.J.; Hurn P.D.	2008
The internal medicine specialist and the preoperative risk evaluation and management	Fontana M.G.; Capitelli M.; Cioni G.	2008
Sandblasted/acid-etched vs smooth-surface implants: implant clinical reaction to experimentally induced peri-implantitis in Beagle dogs.	Martines R.T.; Sendyk W.R.; Gromatzky A.; Cury P.R.	2008
Soft tissue preservation and crestal bone loss around single-tooth implants.	Sunitha R.V.; Ramakrishnan T.; Kumar S.; Emmadi P.	2008
Conventional and early loading of two-implant supported mandibular overdentures. A systematic review.	Rutkunas V.; Mizutani H.; Puriene A.	2008
Dental implant-related infections	López-Cerero L.	2008
Osteogenesis distraction and platelet-rich plasma: Combined use in restoration of severe atrophic mandible. Long-term results	Robiony M.; Zorzan E.; Polini F.; Sembronio S.; Toro C.; Politi M.	2008
The effect of thick mucosa on peri-implant tissues: An experimental study in dogs	Jeong S.-M.; Choi B.-H.; Li J.; Xuan F.	2008
Injectable calcium phosphate cement as a bone-graft material around peri-implant dehiscence defects: a dog study.	Arisan V.; Ozdemir T.; Anil A.; Jansen J.A.; Ozer K.	2008
Alveolar ridge augmentation with titanium mesh and a combination of autogenous bone and anorganic bovine bone: A 2-year prospective study	Pieri F.; Corinaldesi G.; Fini M.; Aldini N.N.; Giardino R.; Marchetti C.	2008
Retrospective clinical evaluation of tapered screw-vent implants: results after up to eight years of clinical function.	Ormianer Z.; Palti A.	2008
Composition of supra- and subgingival biofilm of subjects with healthy and diseased implants	Shibli J.A.; Melo L.; Ferrari D.S.; Figueiredo L.C.; Faveri M.; Feres M.	2008
Er,Cr:YSGG laser-assisted surgical treatment of peri-implantitis with 1-year reentry and 18-month follow-up	Azzeh M.M.	2008
Early wound healing following one-stage dental implant placement with and without antibiotic prophylaxis: A pilot study	Khoury S.B.; Thomas L.; Walters J.D.; Sheridan J.F.; Leblebicioglu B.	2008
Clinical analysis of the soft tissue integration of non-submerged (ITI) and submerged (3i) implants: A prospective-controlled cohort study	Garcia R.V.; Kraehenmann M.A.; Bezerra F.J.B.; Mendes C.M.C.; Rapp G.E.	2008
Bone level changes at implants supporting crowns or fixed partial dentures with or without cantilevers	Hälg G.A.; Schmid J.; Hämmerle C.H.F.	2008
Relationships between implant stability, image-based measures and nitric oxide levels	Güncü G.N.; Tözüm T.F.; Güncü M.B.; Yamalik N.	2008
A clinical observation of therapeutic alliance of orthodontics and implant prosthodontics for dentition defect with malocclusion	Zhou G.; Liu D.; Liang X.; Wang J.; Tang L.; Zhang N.	2008
Performance of dental implants after staged sinus floor elevation procedures: 5-year results of a prospective study in partially edentulous patients	Bornstein M.M.; Chappuis V.; Von Arx T.; Buser D.	2008
Long-term results of mandibular reconstruction with autogenous bone grafts and oral implants after tumor resection	Chiapasco M.; Colletti G.; Romeo E.; Zaniboni M.; Brusati R.	2008
A modified approach for vestibuloplasty in severely resorbed mandible using an implant-retained postoperative stent: a case report	Melo L.G.N.; Almeida A.L.P.F.; Lopes J.F.S.; Rezende M.L.R.; Neto J.S.M.; Ciporkin F.; Nagata M.J.H.	2008
Locally delivered antimicrobials: clinical evidence and relevance.	Paquette D.W.; Ryan M.E.; Wilder R.S.	2008
Peri-implant diseases: Diagnosis and risk indicators	Heitz-Mayfield L.J.A.	2008
Peri-implant diseases: Consensus Report of the Sixth European Workshop on Periodontology	Lindhe J.; Meyle J.	2008
Microbiology and cytokine levels around healthy dental implants and teeth	Nowzari H.; Botero J.E.; DeGiacomo M.; Villacres M.C.; Rich S.K.	2008
A systematic review of the success of sinus floor elevation and survival of implants inserted in combination with sinus floor elevation: Part I: Lateral approach	Pjetursson B.E.; Tan W.C.; Zwahlen M.; Lang N.P.	2008
The safety and acceptability of an electric toothbrush on peri-implant mucosa in patients with oral implants in aesthetic areas: a prospective cohort study.	Rasperini G.; Pellegrini G.; Cortella A.; Rocchietta I.; Consonni D.; Simion M.	2008
Surgical treatment of peri-implantitis	Claffey N.; Clarke E.; Polyzois I.; Renvert S.	2008
Early implant placement with simultaneous guided bone regeneration following single-tooth extraction in the esthetic zone: A croslsectional, retrospective study in 45 subjects with a 2- to 4-year follow-up	Buser D.; Bornstein M.M.; Weber H.P.; Grutter L.; Schmid B.; Belser U.C.	2008
Soft tissue management at implant sites	Cairo F.; Pagliaro U.; Nieri M.	2008
A systematic review of the success of sinus floor elevation and survival of implants inserted in combination with sinus floor elevation Part II: Transalveolar technique	Tan W.C.; Lang N.P.; Zwahlen M.; Pjetursson B.E.	2008
Light-microscopic evaluation of the dimensions of peri-implant mucosa around immediately loaded and submerged titanium implants in monkeys	Quaranta A.; Piattelli A.; Scarano A.; Quaranta M.; Pompa G.; Iezzi G.	2008
Carotid Stenting versus Carotid Endarterectomy: Evidence Basis and Cost Implications	Janssen M.P.; de Borst G.J.; Mali W.P.Th.M.; Kappelle L.J.; Moll F.L.; Ackerstaff R.G.A.; Rothwell P.M.; Brown M.M.; van Sambeek M.R.; Buskens E.	2008

The FG Syndromes (Online Mendelian Inheritance in Man 305450): Perspective in 2008	Opitz J.M.; Smith J.F.; Santoro L.	2008
Radiographic follow-up analysis of Brånemark dental implants.	Pikner S.S.	2008
Primary oral squamous cell carcinoma arising around dental osseointegrated implants mimicking peri-implantitis	Valle A.E.D.; Llamosas R.M.-C.; Vicente J.L.; Etxebarria A.U.; Urizar J.M.A.	2008
Evaluation of peri-implant bone loss around platform-switched implants	Cappiello M.; Luongo R.; Di Iorio D.; Bugea C.; Cocchetto R.; Celletti R.	2008
Interventions for replacing missing teeth: Maintaining and recovering soft tissue health around dental implants	Grusovin M.G.; Coulthard P.; Jourabchian E.; Worthington H.V.; Esposito M.A.B.	2008
Effect of ovariectomy and alendronate on implant osseointegration in rat maxillary bone.	Viera-Negrón Y.E.; Ruan W.H.; Winger J.N.; Hou X.; Sharawy M.M.; Borke J.L.	2008
Conventional and advanced implant treatment in the type II diabetic patient: Surgical protocol and long-term clinical results	Tawil G.; Younan R.; Azar P.; Sleilati G.	2008
Five-year prospective evaluation of mandibular overdentures retained by two microthreaded, TiOBlast nonsplinted implants and retentive ball anchors	Cooper L.F.; Moriarty J.D.; Guckes A.D.; Klee L.B.; Smith R.G.; Almgren C.; Felton D.A.	2008
Evaluation of the presence or absence of papilla between tooth and implant	Kawai E.S.; Almeida A.L.P.F.	2008
Early loading of mandibular full-arch bridges screw retained after 1 week to four to five Monotype® implants: 3-Year results from a prospective multicentre study	Arvidson K.; Esselin O.; Felle-Persson E.; Jonsson G.; Smedberg J.-I.; Soderstrom U.	2008
The influence of abutment angulation on micromotion level for immediately loaded dental implants: A 3-D finite element analysis	Kao H.-C.; Gung Y.-W.; Chung T.-F.; Hsu M.-L.	2008
Immediate functional loading of TiOblast dental implants in full-arch edentulous mandibles: A 3-year prospective study	De Bruyn H.; Van De Velde T.; Collaert B.	2008
Ridge alterations following immediate implant placement in the dog: Flap versus flapless surgery	Blanco J.; Nuñez V.; Aracil L.; Muñoz F.; Ramos I.	2008
Interventions for replacing missing teeth: Treatment of perimplantitis	Esposito M.; Grusovin M.G.; Kakisis I.; Coulthard P.; Worthington H.V.	2008
Altered systemic serologic parameters in patients with silicone mammary implants	Wolfram D.; Oberreiter B.; Mayerl C.; Soelder E.; Ulmer H.; Piza-Katzer H.; Wick G.; Backovic A.	2008
Diagnosis and management of peri-implant diseases	Heitz-Mayfield L.J.A.	2008
The efficacy of interventions to treat peri-implantitis: a Cochrane systematic review of randomised controlled clinical trials.	Esposito M.; Grusovin M.G.; Coulthard P.; Worthington H.V.	2008
Biological basis for soft tissue management in implant dentistry	Yeung S.C.H.	2008
Evaluation of soft-tissue alteration around implant-supported single-tooth restoration in the anterior maxilla: The pink esthetic score	Lai H.-C.; Zhang Z.-Y.; Wang F.; Zhuang L.-F.; Liu X.; Pu Y.-P.	2008
Immediate non-occlusal vs. early loading of dental implants in partially edentulous patients: A multicentre randomized clinical trial. Peri-implant bone and soft-tissue levels	Galli F.; Capelli M.; Zuffetti F.; Testori T.; Esposito M.	2008
Hard tissue implant interface	Albrektsson T.	2008
The use of a linear oscillating device in periodontal treatment: A review	Guentsch A.; Preshaw P.M.	2008
Immediate versus early loading of flapless-placed implants supporting maxillary full-arch prostheses: a randomised controlled clinical trial.	Cannizzaro G.; Torchio C.; Leone M.; Esposito M.	2008
Implant placement in fresh extraction sockets and simultaneous osteotome sinus floor elevation: A case series	Barone A.; Cornelini R.; Ciaglia R.; Covani U.	2008
Regenerative therapy of deep peri-implant lnfrabony defects after CO 2 laser implant surface decontamination	Romanos G.E.; Nentwig G.H.	2008
Lung transplantation and cystic fibrosis	Spencer H.; Aurora P.	2008
Rehabilitation of atrophic maxilla: A review of 101 zygomatic implants	Urgell J.P.; Gutiérrez V.R.; Escoda C.G.	2008
Histologic analysis of human peri-implant bone in type 1 osteoporosis.	Shibli J.A.; Aguiar K.C.; Melo L.; Ferrari D.S.; D'Avila S.; Iezzi G.; Piattelli A.	2008
An alternative method to treat a case with severe maxillary atrophy by the use of angled implants instead of complicated augmentation procedures: a case report.	Bilhan H.	2008
Systematic review of implant outcomes in treated periodontitis subjects	Ong C.T.T.; Ivanovski S.; Needleman I.G.; Retzepi M.; Moles D.R.; Tonetti M.S.; Donos N.	2008
Influence of amine fluoride/stannous fluoride mouthwashes with and without chlorhexidine on secretion of proinflammatory molecules by peri-implant crevicular fluid cells.	Di Carlo F.; Quaranta A.; Di Alberti L.; Ronconi L.F.; Quaranta M.; Piattelli A.	2008
A 5-year prospective multicenter study on 1-stage smooth-surface brånemark system implants with early loading in edentulous mandibles	Friberg B.; Raghoebar G.M.; Grunert I.; Hobkirk J.A.; Tepper G.	2008
Immediate loading in the fully edentulous maxilla without bone grafting: the V-II-V technique.	Agliardi E.L.; Francetti L.; Romeo D.; Taschieri S.; Del Fabbro M.	2008
Influence of abutment material on stability of peri-implant tissues: A systematic review	Linkevicius T.; Apse P.	2008
Immediate loading using cross-arch fixed restorations in heavy smokers: Nine consecutive case reports for edentulous arches	Romanos G.E.; Nentwig G.-H.	2008
Simultaneous flapless implant placement and peri-implant defect correction: An experimental pilot study in dogs	Jeong S.-M.; Choi B.-H.; Li J.; Xuan F.	2008
The treatment of peri-mucositis and peri-implantitis 1.	Otto M.; Gluckman H.	2008

Soft tissue dehiscence coverage around endosseous implants: A prospective cohort study	Burkhardt R.; Joss A.; Lang N.P.	2008
Implant-retained auricular prostheses: An assessment of implant success and prosthetic complications	Aydin C.; Karakoca S.; Yilmaz H.; Yilmaz C.	2008
Costosternal chondrodynia simulating recurrent breast cancer unveiled by FDG PET	Mathew A.S.; El-Haddad G.; Lilien D.L.; Takalkar A.M.	2008
Biomechanical strength of the Peri-Loc® proximal tibial plate: A comparison of all-locked versus hybrid locked/nonlocked screw configurations	Estes C.; Rhee P.; Shrader M.W.; Csavina K.; Jacofsky M.C.; Jacofsky D.J.	2008
Paresthesia of the mental nerve stem from periapical infection of mandibular canine tooth: a case report	Ozkan B.T.; Celik S.; Durmus E.	2008
Quantitative computed tomography bone mineral density measurements in irradiated and non-irradiated minipig alveolar bone: An experimental study	Verdonck H.W.D.; Meijer G.J.; Nieman F.H.; Stoll C.; Riediger D.; De Baat C.	2008
Intra-arterial thrombolysis of acute iatrogenic intracranial arterial occlusion attributable to neuroendovascular procedures or coronary angiography.	Arnold M.; Fischer U.; Schroth G.; Nedeltchev K.; Isenegger J.; Remonda L.; Windecker S.; Brekenfeld C.; Mattle H.P.	2008
In-patient comparison of immediate and conventional loaded implants in mandibular molar sites within 12 months	Güncü M.B.; Aslan Y.; Tümer C.; Güncü G.N.; Uysal S.	2008
The dimensions of keratinized mucosa around implants affect clinical and immunological parameters	Zigdon H.; MacHtei E.E.	2008
Bone level alterations at implants placed in the posterior segments of the dentition: Outcome of submerged/non-submerged healing. A 5-year multicenter, randomized, controlled clinical trial	Cecchinato D.; Bengazi F.; Blasi G.; Botticelli D.; Cardarelli I.; Gualini F.	2008
Treatment of extracranial carotid artery pseudoaneurysms with stent grafts: Case series	Gupta R.; Thomas A.J.; Masih A.; Horowitz M.B.	2008
Human histologic evaluation of a six-year-old threaded implant retrieved from a subject with osteoporosis.	de Melo L.; Piattelli A.; Lezzi G.; d'Avila S.; Zenóbio E.G.; Shibli J.A.	2008
Outcome of dental implants in partially edentulous patients with and without a history of periodontitis: a 5-year interim analysis of a cohort study.	Gatti C.; Gatti F.; Chiapasco M.; Esposito M.	2008
Maintaining and recovering soft tissue health around dental implants: a Cochrane systematic review of randomised controlled clinical trials.	Grusovin M.G.; Coulthard P.; Worthington H.V.; Esposito M.	2008
Width of keratinized gingiva and the health status of the supporting tissues around dental implants	Bouri Jr. A.; Bissada N.; Al-Zahrani M.S.; Faddoul F.; Nouneh I.	2008
One-year bacterial colonization patterns of Staphylococcus aureus and other bacteria at implants and adjacent teeth	Salvi G.E.; Fürst M.M.; Lang N.P.; Persson G.R.	2008
A 10-year prospective clinical and radiographic study of one-stage dental implants	Kim D.M.; Badovinac R.L.; Lorenz R.L.; Fiorellini J.P.; Weber H.P.	2008
Success rates of microimplants in edentulous patients with residual ridge resorption	Morneburg T.R.; Pröschel P.A.	2008
Implant-supported maxillary overdentures retained with milled bars: Maxillary anterior versus maxillary posterior concept - A retrospective study	Krennmair G.; Krainhöfner M.; Piehslinger E.	2008
A systematic review of the 5-year survival and complication rates of implant-supported single crowns	Jung R.E.; Pjetursson B.E.; Glauser R.; Zembic A.; Zwahlen M.; Lang N.P.	2008
Early loading of sandblasted and acid-etched implants: A randomized-controlled double-blind split-mouth study. Five-year results	Roccuzzo M.; Aglietta M.; Bunino M.; Bonino L.	2008
Re-osseointegration on rough implant surfaces previously coated with bacterial biofilm: An experimental study in the dog	Alhag M.; Renvert S.; Polyzois I.; Claffey N.	2008
Peri-implant tissue and radiographic bone levels in the immediately restored single-tooth implant: A retrospective analysis	Degidi M.; Nardi D.; Piattelli A.	2008
Analysis of the possibility of the relationship between various implant-related measures: An 18-month follow-up study	Tözüm T.F.; Turkyilmaz I.; Yamalik N.; Karabulut E.; Türkyilmaz A.S.; Eratalay K.	2008
Bone healing dynamics at buccal peri-implant sites	Qahash M.; Susin C.; Polimeni G.; Hall J.; Wikesjö U.M.E.	2008
Roles of bone scintigraphy and resonance frequency analysis in evaluating osseointegration of endosseous implant	Zhou Y.; Jiang T.; Qian M.; Zhang X.; Wang J.; Shi B.; Xia H.; Cheng X.; Wang Y.	2008
Similar cost for elective open and endovascular AAA repair in a population-based setting	Mani K.; Björck M.; Lundkvist J.; Wanhainen A.	2008
Implant treatment in periodontitis-susceptible patients: A systematic review	Schou S.	2008
Two-year clinical results following treatment of peri-implantitis lesions using a nanocrystalline hydroxyapatite or a natural bone mineral in combination with a collagen membrane	Schwarz F.; Sculean A.; Bieling K.; Ferrari D.; Rothamel D.; Becker J.	2008
The impact of prosthetic design on the stability, marginal bone loss, peri-implant sulcus fluid volume, and nitric oxide metabolism of conventionally loaded endosseous dental implants: A 12-month clinical study	Tözüm T.F.; Qüncü Q.N.; Yamalik N.; Turkyilmaz I.; Baris Güncü M.	2008
Immediate maxillary lateral incisor implants with nonocclusal loading provisional crowns	Penarrocha M.; Lamas J.; Penarrocha M.; Garcia B.	2008
Prosthodontic rehabilitation of oral function in head-neck cancer patients with dental implants placed simultaneously during ablative tumour surgery: an assessment of treatment outcomes and quality of life	Schoen P.J.; Raghoebar G.M.; Bouma J.; Reintsema H.; Burlage F.R.; Roodenburg J.L.N.; Vissink A.	2008
Evaluation of human bone around a dental implant retrieved from a subject with osteoporosis	Shibli J.A.; Grande P.A.; D'Avila S.; Iezzi G.; Piattelli A.	2008
Correlation between early perforation of cover screws and marginal bone loss: A retrospective study	Van Assche N.; Collaert B.; Coucke W.; Quirynen M.	2008

Asepsis during periodontal surgery involving oral implants and the usefulness of peri-operative antibiotics: A prospective, randomized, controlled clinical trial	Abu-Ta'a M.; Quirynen M.; Teughels W.; Van Steenberghe D.	2008
Peri-implant bone loss caused by occlusal overload: Repair of the peri-implant defect following correction of the traumatic occlusion. A case report	Tawil G.	2008
Sonography of periimplant buccal bone defects in periodontitis patients: A pilot study	Bertram S.; Emshoff R.	2008
Complications and short-term results of two different transobturator techniques for surgical treatment of women with urinary incontinence: A randomized study	But I.; Faganelj M.	2008
Mycobacterium fortuitum infection following total knee arthroplasty: A case report and literature review	Cheung I.; Wilson A.	2008
Reliability of findings around healthy implants in association with oral hygiene measures: A clinical, microbiological, and immunological follow-up in edentulous patients	Lachmann S.; Kimmerle-Müller E.; Axmann D.; Gomez-Roman G.; Weber H.; Haas R.	2007
A comprehensive and critical review of dental implant prognosis in periodontally compromised partially edentulous patients: Review	Karoussis I.K.; Kotsovilis S.; Fourmousis I.	2007
Clinical and radiographic evaluation of periodontal and peri-implant conditions in patients with implant-supported prosthesis.	Abreu M.H.; Bianchini M.A.; Magini R.S.; Rösing C.K.	2007
Resolution of an active peri-implantitis in a chronic steroid user by bone augmentation with PepGen P-15 and a barrier membrane.	Lu S.Y.; Huang C.C.	2007
Failing factors associated with osseointegrated dental implant loss	Montes C.C.; Pereira F.A.; Thomé G.; Alves E.D.M.; Acedo R.V.; De Souza J.R.; Melo A.C.M.; Trevilatto P.C.	2007
A 10-year prospective study of ITI dental implants placed in the posterior region. I: Clinical and radiographic results	Blanes R.J.; Bernard J.P.; Blanes Z.M.; Belser U.C.	2007
Osseointegrated implants in subjects treated for generalized aggressive periodontitis: 10-Year results of a prospective, long-term cohort study	Mengel R.; Behle M.; Flores-de-Jacoby L.	2007
Managing Complications after Midurethral Sling for Stress Urinary Incontinence	Costantini E.; Lazzeri M.; Porena M.	2007
Experimental evaluation of tooth movement in the beagle dog with the mini-screw implant for orthodontic anchorage	Owens S.E.; Buschang P.H.; Cope J.B.; Franco P.F.; Rossouw P.E.	2007
Implant Placement Immediately After the Lateral Approach of the Trap Door Window Procedure to Create a Maxillary Sinus Lift Without Bone Grafting: A 2-Year Retrospective Evaluation of 47 Implants in 33 Patients	Chen T.-W.; Chang H.-S.; Leung K.-W.; Lai Y.-L.; Kao S.-Y.	2007
Preservation of peri-implant soft and hard tissues using platform switching of implants placed in immediate extraction sockets: A proof-of-concept study with 12- to 36-month follow-up	Canullo L.; Rasperini G.	2007
Dental implants as strategic supplementary abutments for implant-tooth-supported telescopic crown-retained maxillary dentures: A retrospective follow-up study for up to 9 years	Krennmair G.; Krainhöfner M.; Waldenberger O.; Piehslinger E.	2007
Implant-supported mandibular overdentures retained with a milled bar: A retrospective study	Krennmair G.; Krainhöfner M.; Piehslinger E.	2007
Interleukin 1beta levels around microscrew implants during orthodontic tooth movement.	Sari E.; Uçar C.	2007
Laser applications in oral surgery and implant dentistry	Deppe H.; Horch H.-H.	2007
Efficacy and economics of postoperative blood salvage in patients undergoing elective total hip replacement	Mirza S.B.; Campion J.; Dixon J.H.; Panesar S.S.	2007
A review of the aetiology and management of fractured dental implants and a case report	Virdee P.; Bishop K.	2007
Long time follow up of implant therapy and treatment of peri-implantitis.	Roos-Jansåker A.M.	2007
Effects of smoking on the outcome of implant treatment: A literature review	Baig M.R.; Rajan M.	2007
Early soft tissue healing around one-stage dental implants: Clinical and microbiologic parameters	DeAngelo S.J.; Kumar P.S.; Beck F.M.; Tatakis D.N.; Leblebicioglu B.	2007
Implant supported prostheses with Bone System implant system: a retrospective study with follow-up period up to 13-years about 1021 fixtures.	Carbone M.; Goss E.; Borione M.; Bava L.; Broccoletti R.; Carrozzo M.; Gandolfo S.	2007
A 5-year follow-up study on one-stage implants inserted concomitantly with localized alveolar ridge augmentation	Juodzbalys G.; Raustia A.M.; Kubilius R.	2007
Bone level changes at axial- and non-axial-positioned implants supporting fixed partial dentures. A 5-year retrospective longitudinal study	Koutouzis T.; Wennström J.L.	2007
Deleterious effects of dihydrotestosterone on cerebral ischemic injury	Cheng J.; Alkayed N.J.; Hurn P.D.	2007
Schwann cell graft: A method to promote sensory responses of osseointegrated implants	Yuan Q.; Gong P.; Tan Z.	2007
Er:YAG laser therapy for peri-implant infection: A histological study	Takasaki A.A.; Aoki A.; Mizutani K.; Kikuchi S.; Oda S.; Ishikawa I.	2007
Impact of supportive periodontal therapy and implant surface roughness on implant outcome in patients with a history of periodontitis	Quirynen M.; Abarca M.; Van Assche N.; Nevins M.; Van Steenberghe D.	2007
Immediate loading of two implants supporting a ball attachment-retained mandibular overdenture: A prospective clinical study	Marzola R.; Scotti R.; Fazi G.; Schincaglia G.P.	2007
Single-tooth implant reconstructions: esthetic factors influencing the decision between titanium and zirconia abutments in anterior regions.	Sailer I.; Zembic A.; Jung R.E.; Hämmerle C.H.; Mattiola A.	2007

Dental implant design and oral and systemic health.	Callan D.P.	2007
Analysis of the potential association of implant stability, laboratory, and image-based measures used to assess osteotomy sites: Early versus delayed loading	Tözüm T.F.; Turkyilmaz I.; Yamalik N.; Karabulut E.; Eratalay K.	2007
Effect of cortical bone thickness and implant placement torque on stability of orthodontic mini-implants	Motoyoshi M.; Yoshida T.; Ono A.; Shimizu N.	2007
Two-piece implants with turned versus microtextured collars	Cosyn J.; Sabzevar M.M.; De Wilden P.; De Rouck T.	2007
Biomaterial strategies to reduce implant-associated infections	Qiu Y.; Zhang N.; An Y.H.; Wen X.	2007
Clinical and radiological results after augmentation procedures - A prospective study	Schlegel K.A.; Karascholi T.; Fenner M.; Nkenke E.	2007
Anti-platelet and anti-thrombotic approaches in patients undergoing percutaneous coronary intervention	Garg R.; Uretsky B.F.; Lev E.I.	2007
Infection at titanium implants with or without a clinical diagnosis of inflammation	Renvert S.; Roos-Jansåker A.-M.; Lindahl C.; Renvert H.; Rutger Persson G.	2007
Submerged healing following surgical treatment of peri-implantitis: a case series	Roos-Jansåker A.-M.; Renvert H.; Lindahl C.; Renvert S.	2007
Infections in implantology: From prophylaxis to treatment	Antolín A.B.; García M.T.P.; Nasimi A.	2007
Survival and success rates of 3i implants in partially edentulous patients: Results of a prospective study with up to 84-months' follow-up	Knauf M.; Gerds T.; Muche R.; Strub J.R.	2007
A 1- To 12-year clinical evaluation of 106 endosseous implants supporting fixed and removable prostheses	Aykent F.; Inan O.; Ozyesil A.G.; Alptekin N.O.	2007
Autogenous onlay bone grafts vs. alveolar distraction osteogenesis for the correction of vertically deficient edentulous ridges: A 2-4-year prospective study on humans	Chiapasco M.; Zaniboni M.; Rimondini L.	2007
Influence of low-level laser treatment on bone regeneration and osseointegration of dental implants following sinus augmentation: An experimental study on sheep	Jakse N.; Payer M.; Tangl S.; Berghold A.; Kirmeier R.; Lorenzoni M.	2007
Surgical treatment of peri-implantitis using a bone substitute with or without a resorbable membrane: A prospective cohort study	Roos-Jansåker A.-M.; Renvert H.; Lindahl C.; Renvert S.	2007
Bone regeneration around implants in the canine mandible with cultured fibroblasts in polyglactin mesh	Sparks M.S.; Kerns D.G.; Wilson T.G.; Hallmon W.W.; Spears R.; Haghighat N.	2007
Effects of a cell adhesion molecule coating on the blasted surface of titanium implants on bone healing in the rabbit femur	Park J.-W.; Lee S.-G.; Choi B.-J.; Suh J.-Y.	2007
Influence of immediate implant loading on peri-implant soft tissue morphology in the edentulous maxilla	Gallucci G.O.; Mavropoulos A.; Bernard J.-P.; Belser U.C.	2007
Biomolecular surface coating to enhance orthopaedic tissue healing and integration	Reyes C.D.; Petrie T.A.; Burns K.L.; Schwartz Z.; García A.J.	2007
Adjunctive local antibiotic therapy in the treatment of peri-implantitis II: Clinical and radiographic outcomes	Salvi G.E.; Persson G.R.; Heitz-Mayfield L.J.A.; Frei M.; Lang N.P.	2007
Peri-implant bone regeneration with calcium sulfate: A light and transmission electron microscopy case report	Scarano A.; Orsini G.; Pecora G.; Iezzi G.; Perrotti V.; Piattelli A.	2007
Factors influencing the preservation of the periimplant marginal bone	Hermann F.; Lerner H.; Palti A.	2007
The effect on bone regeneration of a liposomal vector to deliver BMP-2 gene to bone grafts in peri-implant bone defects	Park J.; Lutz R.; Felszeghy E.; Wiltfang J.; Nkenke E.; Neukam F.W.; Schlegel K.A.	2007
Analysis of early biofilm formation on oral implants in man	Heuer W.; Elter C.; Demling A.; Neumann A.; Suerbaum S.; Hannig M.; Heidenblut T.; Bach F.W.; Stiesch-Scholz M.	2007
Vertical crestal bone changes around implants placed into fresh extraction sockets	Covani U.; Cornelini R.; Barone A.	2007
Biomolecular modification of implant surfaces	Morra M.	2007
Maxillary sinus lift for single implant-supported restorations: A clinical study	Krennmair G.; Krainhöfner M.; Schmid-Schwap M.; Piehslinger E.	2007
Two human hydroxyapatite-coated dental implants retrieved after a 14-year loading period: A histologic and histomorphometric case report	Iezzi G.; Scarano A.; Petrone G.; Piattelli A.	2007
Long-Term Results of Jaw Reconstruction With Microsurgical Fibula Grafts and Dental Implants	Gbara A.; Darwich K.; Li L.; Schmelzle R.; Blake F.	2007
Platelet-enriched fibrin glue and platelet-rich plasma in the repair of bone defects adjacent to titanium dental implants	You T.-M.; Choi B.-H.; Zhu S.-J.; Jung J.-H.; Lee S.-H.; Huh J.-Y.; Lee H.-J.; Li J.	2007
Removable partial denture on osseointegrated implants and natural teeth	Chang L.-C.; Wang J.-C.; Tasi C.-C.	2007
Surgical laser use in implantology and endodontics	Parker S.	2007
Comparison of naturally occurring and ligature-induced peri-implantitis bone defects in humans and dogs	Schwarz F.; Herten M.; Sager M.; Bieling K.; Sculean A.; Becker J.	2007
Therapy of peri-implantitis with resective surgery. A 3-year clinical trial on rough screw-shaped oral implants. Part II: Radiographic outcome	Romeo E.; Lops D.; Chiapasco M.; Ghisolfi M.; Vogel G.	2007
Soft and hard tissue assessment of immediate implant placement: A case series	Juodzbalys G.; Wang H.-L.	2007
Risks and complications of orthodontic miniscrews	Kravitz N.D.; Kusnoto B.	2007
Scanning electron microscopic analysis of diode laser-treated titanium implant surfaces	Castro G.L.; Gallas M.; Núñez I.R.; Borrajo J.L.L.; Álvarez J.C.; Varela L.G.	2007
Clinical analysis of immediate implant and implant-supported complete denture for 12 patients with severe periodontitis	Geng W.; Su Y.C.; Zhang X.J.; Wu L.C.; Huang Q.; Jiang X.Y.	2007

Rehabilitation of oral function in head and neck cancer patients after radiotherapy with implant-retained dentures: Effects of hyperbaric oxygen therapy	Schoen P.J.; Raghoebar G.M.; Bouma J.; Reintsema H.; Vissink A.; Sterk W.; Roodenburg J.L.N.	2007
The effect of platelet-rich plasma on bone healing around implants placed in bone defects treated with Bio-Oss: a pilot study in the dog tibia	You T.-M.; Choi B.-H.; Li J.; Jung J.-H.; Lee H.-J.; Lee S.-H.; Jeong S.-M.	2007
Impact of tobacco use on the periodontium--an update. Part 2: Clinical and radiographic changes in the periodontium and effects on periodontal and implant therapy	Saxer U.P.; Walter C.; Bornstein M.M.; Klingler K.; Ramseier C.A.	2007
Inhibition of Porphyromonas gingivalis adherence on titanium surface grafted by poly(sodium styrene sulfonate)	Zhou J.; Pavon-Djavid G.; Anagnostou F.; Migonney V.	2007
Analysis of stress and strain around orthodontically loaded implants: An animal study	Cattaneo P.M.; Dalstra M.; Melsen B.	2007
Canal atresia: "Surgery or implantable hearing devices? The expert's question is revisited"	Evans A.K.; Kazahaya K.	2007
A patient-centered perspective on surgery avoidance for hip or knee arthritis: Lessons for the future	Ballantyne P.J.; Gignac M.A.M.; Hawker G.A.	2007
Characterization of the antioxidant profile of human saliva in peri-implant health and disease	Liskmann S.; Vihalemm T.; Salum O.; Zilmer K.; Fischer K.; Zilmer M.	2007
Le Fort I osteotomy with interpositional bone grafts and delayed oral implants for the rehabilitation of extremely atrophied maxillae: A 1-9-year clinical follow-up study on humans	Chiapasco M.; Brusati R.; Ronchi P.	2007
Peri-implant bone loss around posterior mandible dental implants placed after distraction osteogenesis: Preliminary findings	Polo W.C.K.; de Araujo N.S.; Lima Y.B.O.; Joly J.C.; Sendyk W.R.; Cury P.R.	2007
Three-year treatment outcomes with three brands of implants placed in the posterior maxilla and mandible of partially edentulous patients	Ozkan Y.; Ozcan M.; Akoglu B.; Ucankale M.; Kulak-Ozkan Y.	2007
Diagnostic accuracy of in vitro panoramic radiographs depending on the exposure	Kaeppler G.; Dietz K.; Reinert S.	2007
A comparison of implant-supported, bar- Or ball-retained mandibular overdentures: A retrospective clinical, microbiologic, and immunologic study of 10 edentulous patients attending a recall visit	Lachmann S.; Kimmerle-Müller E.; Gehring K.; Axmann D.; Gomez-Roman G.; Watzek G.; Weber H.	2007
Factors affecting late implant bone loss: A retrospective analysis	Chung D.M.; Oh T.-J.; Lee J.; Misch C.E.; Wang H.-L.	2007
Immediate functional loading of implants placed with flapless surgery in the edentulous maxilla: 1-Year follow-up of a single cohort study	Cannizzaro G.; Leone M.; Esposito M.	2007
Treatment of experimental peri-implantitis using autogenous bone grafts and platelet-enriched fibrin glue in dogs	You T.-M.; Choi B.-H.; Zhu S.-J.; Jung J.-H.; Lee S.-H.; Huh J.-Y.; Lee H.-J.; Li J.	2007
Conventional versus CO2 laser-assisted treatment of peri-implant defects with the concomitant use of pure-phase beta-tricalcium phosphate: a 5-year clinical report.	Deppe H.; Horch H.H.; Neff A.	2007
The effect of delayed versus early loading on nitric oxide metabolism around dental implants: An 18-month comparative follow-up study	Tözüm T.F.; Türkyilmaz I.; Yamalik N.; Tümer C.; Kilinç A.; Kilinç K.; Karabulut E.; Eratalay K.	2007
Retrieval of blade implants with piezosurgery: two clinical cases.	Sivolella S.; Berengo M.; Fiorot M.; Mazzuchin M.	2007
Prevalence and risk variables for peri-implant disease in Brazilian subjects	Ferreira S.D.; Silva G.L.M.; Cortelli J.R.; Costa J.E.; Costa F.O.	2006
Re-osseointegration of peri-implantitis bone defects: an experimental study in dogs	Zou D.; Zhu H.; Qu X.	2006
Neodymium:yttrium aluminum garnet laser irradiation with low pulse energy: A potential tool for the treatment of peri-implant disease	Giannini R.; Vassalli M.; Chellini F.; Polidori L.; Dei R.; Giannelli M.	2006
Radiological and clinical follow-up of machined- and anodized-surface implants after mean functional loading for 33 months	Watzak G.; Zechner W.; Busenlechner D.; Arnhart C.; Gruber R.; Watzek G.	2006
Nonsurgical treatment of moderate and advanced periimplantitis lesions: A controlled clinical study	Schwarz F.; Bieling K.; Bonsmann M.; Latz T.; Becker J.	2006
Retrospective evaluation of mandibular incisor replacement with narrow neck implants	Cordaro L.; Torsello F.; Mirisola Di Torresanto V.; Rossini C.	2006
A 2-year clinical report of patients treated with two loading protocols for mandibular overdentures: Early versus conventional loading	Turkyilmaz I.; Tözüm T.F.; Tumer C.; Ozbek E.N.	2006
Numerical simulation of in vivo intraosseous torsional failure of a hollow-screw oral implant.	Cehreli M.; Akkocaoglu M.; Akca K.	2006
Probe penetration in periodontal and peri-implant tissues: An experimental study in the beagle dog	Abrahamsson I.; Soldini C.	2006
Effects of sinus membrane perforations on the success of dental implants placed in the augmented sinus	Karabuda C.; Arisan V.; Özyuvaci H.	2006
Bacterial plaque colonization around dental implant surfaces	Covani U.; Marconcini S.; Crespi R.; Barone A.	2006
Long-term clinical evaluation of tapered multi-threaded implants: results and influences of potential risk factors.	Ormianer Z.; Palti A.	2006
Five-year outcome of 111 immediate nonfunctional single restorations.	Degidi M.; Piattelli A.; Gehrke P.; Felice P.; Carinci F.	2006
Increasing the width of keratinized mucosa around endosseous implant using acellular dermal matrix allograft	Park J.-B.	2006
Immediately loaded titanium implants with a porous anodized surface with at least 36 months of follow-up	Degidi M.; Perrotti V.; Piattelli A.	2006
A new era in periodontal and periimplant regeneration: use of growth-factor enhanced matrices incorporating rhPDGF.	Lynch S.E.; Wisner-Lynch L.; Nevins M.; Nevins M.L.	2006
Role of physiotherapy in peri-operative management in total knee and hip surgery	Lenssen A.F.; de Bie R.A.	2006

Syncrystallization: A technique for temporization of immediately loaded implants with metal-reinforced acrylic resin restorations	Degidi M.; Gehrke P.; Spanel A.; Piattelli A.	2006
The effect of smoking on osseointegrated dental implants. Part II: Peri-implant bone loss	DeLuca S.; Zarb G.	2006
Calprotectin and cross-linked N-terminal telopeptides in peri-implant and gingival crevicular fluid	Friedmann A.; Friedrichs M.; Kaner D.; Kleber B.-M.; Bernimoulin J.-P.	2006
Outcome of implant therapy in patients with previous tooth loss due to periodontitis	Schou S.; Holmstrup P.; Worthington H.V.; Esposito M.	2006
A systematic review of marginal soft tissue at implants subjected to immediate loading or immediate restoration	Glauser R.; Zembic A.; Hämmerle C.H.F.	2006
Comparison of hard tissue density changes around implants assessed in digitized conventional radiographs and subtraction images	Bittar-Cortez J.A.; Passeri L.A.; Bóscolo F.N.; Haiter-Neto F.	2006
Clinical applications of mini-implants as orthodontic anchorage and the peri-implant tissue reaction upon loading.	Cho H.J.	2006
Premature withdrawal and alternative therapies to dual oral antiplatelet therapy	Collet J.-P.; Montalescot G.	2006
Matrix metalloproteinases: Contribution to pathogenesis, diagnosis and treatment of periodontal inflammation	Sorsa T.; Tjäderhane L.; Konttinen Y.T.; Lauhio A.; Salo T.; Lee H.-M.; Golub L.M.; Brown D.L.; Mäntylä P.	2006
Clinical report with up to 4 years of follow-up on a cervically modified stepped screw-type implant	Weibrich G.; Streckbein P.; Krummenauer F.; Wagner W.	2006
Retrospective evaluation of temporary cemented, tooth and implant supported fixed partial dentures	Heinemann F.; Mundt T.; Biffar R.	2006
Peri-implant soft-tissue health surrounding cement- and screw-retained implant restorations: A multicenter, 3-year prospective study	Weber H.P.; Kim D.M.; Ng M.W.; Hwang J.W.; Fiorellini J.P.	2006
IL-1RN gene polymorphism is associated with peri-implantitis	Laine M.L.; Leonhardt Å.; Roos-Jansåker A.-M.; Peña A.S.; Van Winkelhoff A.J.; Winkel E.G.; Renvert S.	2006
Influence of different treatment approaches on non-submerged and submerged healing of ligature induced peri-implantitis lesions: An experimental study in dogs	Schwarz F.; Jepsen S.; Herten M.; Sager M.; Rothamel D.; Becker J.	2006
Comparison of early telescope loading of non-submerged ITI implants in irradiated and non-irradiated oral cancer patients	Landes C.A.; Kovács A.F.	2006
Stress and inflammation as a detrimental combination for peri-implant bone loss	Heckmann S.M.; Linke J.J.; Graef F.; Foitzik Ch.; Wichmann M.G.; Weber H.-P.	2006
Update in dental implant periapical surgery	Peñarrocha Diago M.; Boronat López A.; Lamas Pelayo J.	2006
Treatment of periapical dental implant pathology with guided bone regeneration	Tözüm T.F.; Erdal C.; Saygun I.	2006
Correlations between clinical parameters and interleukin-6 and interleukin-10 levels in saliva from totally edentulous patients with peri-implant disease	Liskmann S.; Vihalemm T.; Salum O.; Zilmer K.; Fischer K.; Zilmer M.	2006
Healing of intrabony peri-implantitis defects following application of a nanocrystalline hydroxyapatite (Ostim™) or a bovine-derived xenograft (Bio-Oss™) in combination with a collagen membrane (Bio-Gide™). A case series	Schwarz F.; Bieling K.; Latz T.; Nuesry E.; Becker J.	2006
A new device in immediately loaded implant treatment in the edentulous mandible	De Vasconcellos D.K.; Bottino M.A.; Saad P.A.; Faloppa F.	2006
Application of implant-supported telescopic overdenture in edentulous cases	Hu X.L.; Cui H.Y.; Wang T.; Li J.H.; Qiu L.X.; Lin Y.	2006
Fixed partial prostheses supported by 2 or 3 implants: A retrospective study up to 18 years	Eliasson A.; Eriksson T.; Johansson A.; Wennerberg A.	2006
Effect of mechanical and antiseptic therapy on peri-implant mucositis: An experimental study in monkeys	Trejo P.M.; Bonaventura G.; Weng D.; Caffesse R.G.; Bragger U.; Lang N.P.	2006
Lethal photosensitization and guided bone regeneration in treatment of peri-implantitis: An experimental study in dogs	Shibli J.A.; Martins M.C.; Ribeiro F.S.; Garcia V.G.; Nociti Jr. F.H.; Marcantonio Jr. E.	2006
Dental implants in radiated areas: A series of 33 patients	Bodard A.G.; Gourmet R.; Lucas R.; Bonnet E.; Breton P.	2006
Bone microvascular pattern around loaded dental implants in a canine model	Traini T.; Assenza B.; Roman F.S.; Thams U.; Caputi S.; Piattelli A.	2006
Feasibility and influence of the microgap of two implants placed in a non-submerged procedure: A five- year follow-up clinical trial	Heijdenrijk K.; Raghoebar G.M.; Meijer H.J.A.; Stegenga B.; van der Reijden W.A.	2006
Fat necrosis of the breast-A review	Tan P.H.; Lai L.M.; Carrington E.V.; Opaluwa A.S.; Ravikumar K.H.; Chetty N.; Kaplan V.; Kelley C.J.; Babu E.D.	2006
Anterior teeth esthetics with the SPI implant system	Kessler-Liechti G.; Mericske-Stern R.	2006
Clinical study of periocline on peri-implantitis treatment	Zhou L.; Lin Y.; Qiu L.X.; Chen B.; Hu X.L.; Wang X.	2006
Non-surgical treatment of peri-implant pathology.	De Araújo Nobre M.; Capelas C.; Alves A.; Almeida T.; Carvalho R.; Antunes E.; Oliveira D.; Cardador A.; Maló P.	2006
Implant Surfaces	Puleo D.A.; Thomas M.V.	2006
Expression of interleukin 1-beta, transforming growth factor beta-1, and vascular endothelial growth factor in soft tissue over the implant before uncovering	Schultze-Mosgau S.; Wehrhan F.; Wichmann M.; Schlegel K.A.; Holst S.; Thorwarth M.	2006
Clinical evaluation of 3 overdenture concepts with tooth roots and implants: 2-Year results	Hug S.; Mantokoudis D.; Mericske-Stern R.	2006
Bone height measurements around a dental implant after a 6-month space flight: A case report	Haigneré C.; Jonas P.; Khayat P.; Girot G.	2006

Comparison of abciximab with "high-dose" tirofiban in patients undergoing percutaneous coronary intervention	Gunasekara A.P.; Walters D.L.; Aroney C.N.	2006
Safety of percutaneous coronary interventions	Schöbel W.A.	2006
Nine- to fourteen-year follow-up of implant treatment. Part II: Presence of peri-implant lesions	Roos-Jansåker A.-M.; Lindahl C.; Renvert H.; Renvert S.	2006
Nine- to fourteen-year follow-up of implant treatment. Part III: Factors associated with peri-implant lesions	Roos-Jansåker A.-M.; Renvert H.; Lindahl C.; Renvert S.	2006
Cathepsin K levels in the crevicular fluid of dental implants: A pilot study	Strbac G.D.; Monov G.; Cei S.; Kandler B.; Watzek G.; Gruber R.	2006
Soft tissue around three different implant types after 1.5 years of functional loading without oral hygiene: A preliminary study in baboons	Watzak G.; Zechner W.; Tangl S.; Vasak C.; Donath K.; Watzek G.	2006
Tissue alterations at implant-supported single-tooth replacements: A 1-year prospective clinical study	Cardaropoli G.; Lekholm U.; Wennström J.L.	2006
Clinical and radiographic evaluation of small-diameter (3.3-mm) implants followed for 1-7 years: A longitudinal study	Romeo E.; Lops D.; Amorfini L.; Chiapasco M.; Ghisolfi M.; Vogel G.	2006
Clinical outcome of implants placed immediately after implant removal	Covani U.; Barone A.; Cornelini R.; Crespi R.	2006
Evaluation of stress induced in peri-implant bone tissue by misfit in multi-implant prosthesis.	Natali A.N.; Pavan P.G.; Ruggero A.L.	2006
Gene therapeutics for periodontal regenerative medicine	Ramseier C.A.; Abramson Z.R.; Jin Q.; Giannobile W.V.	2006
Preliminary clinical study of microimplants in the restoration of edentulous jaws	Chen B.; Lin Y.; di P.; Li J.H.; Qiu L.X.	2006
The consequences of neural degeneration regarding optimal cochlear implant position in scala tympani: A model approach	Briaire J.J.; Frijns J.H.M.	2006
Estradiol alters only GAD67 mRNA levels in ischemic rat brain with no consequent effects on GABA	Joh H.-D.; Searles R.V.; Selmanoff M.; Alkayed N.J.; Koehler R.C.; Hurn P.D.; Murphy S.J.	2006
The influence of hormonal replacement therapy on alveolar ridge bone in perimenopuasal women	Bakalczuk M.; Bakalczuk S.; Rózyło T.K.; Kleinrok J.	2006
Analysis of aspartate aminotransferase and alkaline phosphatase in crevicular fluid from implants with and without peri-implantitis.	Paknejad M.; Emtiaz S.; Khoobyari M.M.; Gharb M.T.; Yazdi M.T.	2006
Histomorphometric and fluorescence microscopic evaluation of interfacial bone healing around 3 different dental implants before and after radiation therapy	Weinlaender M.; Beumer III J.; Kenney E.B.; Lekovic V.; Holmes R.; Moy P.K.; Plenk Jr. H.	2006
Peri-implant papilla reconstruction: Realities and limitations	Pradeep A.R.; Karthikeyan B.V.	2006
Profilometric and standard error of the mean analysis of rough implant surfaces treated with different instrumentations.	Ramaglia L.; di Lauro A.E.; Morgese F.; Squillace A.	2006
Clinical and radiological results of patients treated with two loading protocols for mandibular overdentures on Brånemark implants	Turkyilmaz I.	2006
Influence of prosthetic parameters on the survival and complication rates of short implants	Tawil G.; Aboujaoude N.; Younan R.	2006
Implant-supported mandibular overdentures retained with ball or telescopic crown attachments: A 3-year prospective study	Krennmair G.; Weinländer M.; Krainhöfner M.; Piehslinger E.	2006
A clinical retrospective study of 10 years implant results	Lin Y.; Li J.H.; Qiu L.X.; Di P.; Hu X.L.; Wang X.	2006
Rehabilitation by means of osseointegrated implants in oral cancer patients with about four to six years follow-up	Sekine J.; Sano K.; Ikeda H.; Inokuchi T.	2006
The implantable gastric stimulator for obesity: An update of the European experience in the LOSS (Laparoscopic Obesity Stimulation Survey) study	Miller K.; Hoeller E.; Aigner F.	2006
Bacterial colonisation of interior implant threads with and without sealing	Proff P.; Steinmetz I.; Bayerlein T.; Dietze S.; Fanghänel J.; Gedrange T.	2006
Long-term evaluation of hollow screw and hollow cylinder dental implants: Clinical and radiographic results after 10 years	Telleman G.; Meijer H.J.A.; Raghoebar G.M.	2006
Peri-implant bone organization under immediate loading state. Circularly polarized light analyses: A minipig study	Neugebauer J.; Traini T.; Thams U.; Piattelli A.; Zöller J.E.	2006
Implant-borne prosthetic rehabilitation of bone-grafted cleft versus traumatic anterior maxillary defects	Landes C.A.	2006
Prosthetic treatment of maxillary lateral incisor agenesis with osseointegrated implants: A 24-39-month prospective clinical study	Zarone F.; Sorrentino R.; Vaccaro F.; Russo S.	2006
Bone graft substitutes in periodontal and peri-implant bone regeneration	Bayerlein T.; Mundt T.; Mack F.; Bienengräber V.; Proff P.; Gedrange T.	2006
Investigation of peri-implant status and risk variables for implant failure in body of maxilla after oral tumor surgery	Rao N.; Cao Y.; Wei W.	2006
Outcomes of fixed prostheses supported by immediately loaded endosseous implants	Tortamano P.; Orii T.C.; Yamanochi J.; Nakamae A.E.M.; Guarnieri T.D.C.	2006
Diabetic Nephropathy and heart surgery - Prevention of perioperative acute renal failure	Lonnemann G.	2006
Cerebral protection devices for use during carotid artery angioplasty with stenting: A health technology assessment	Menon D.; Stafinski T.	2006
Unexpected return of sensation following 4.5 years of paresthesia: case report.	Elian N.; Mitsias M.; Eskow R.; Jalbout Z.N.; Cho S.C.; Froum S.; Tarnow D.P.	2005
Detection and measurements of soluble intercellular adhesion molecules at implants and teeth: A comparative study	Oezcakir C.; Fekry-Khedr M.; Mericske-Stern R.	2005

Early loading of non-submerged titanium implants with a sandblasted and acid-etched surface. 5-year results of a prospective study in partially edentulous patients.	Bornstein M.M.; Schmid B.; Belser U.C.; Lussi A.; Buser D.	2005
Bone response to submerged, unloaded implants inserted in poor bone sites: a histological and histomorphometrical study of 8 titanium implants retrieved from man.	Iezzi G.; Degidi M.; Scarano A.; Perrotti V.; Piattelli A.	2005
Implant rebilitation of severely traumatised anterior alveolar ridge	Kao S.-Y.; Fong H.-J.; Lui M.-T.; Chou S.-J.; Yeung T.-C.; Hung K.-F.; Wu C.-H.; Yu E.-H.; Wu H.-T.; Chang C.-S.	2005
Endpoints and survival analysis for successful osseointegration of dental implants	Gerds T.A.; Vogeler M.	2005
Implant stability during initiation and resolution of experimental periimplantitis: An experimental study in the dog	Sennerby L.; Persson L.G.; Berglundh T.; Wennerberg A.; Lindhe J.	2005
Common complications after cardiac surgery in the adult: Anecdotes, biases... and some evidence	Foot C.L.; Fraser J.F.; Mullany D.V.	2005
A new implant design for crestal bone preservation: initial observations and case report.	Baumgarten H.; Cocchetto R.; Testori T.; Meltzer A.; Porter S.	2005
Principles and mechanisms of peri-implant soft tissue healing	Schultze-Mosgau S.; Blatz M.B.; Wehrhan F.; Schlegel K.A.; Thorwart M.; Holst S.	2005
Bilaminar subepithelial connective tissue grafts for immediate implant placement and provisionalization in the esthetic zone.	Kan J.Y.; Rungcharassaeng K.; Lozada J.L.	2005
Current concepts in periodontal bioengineering.	Taba Jr. M.; Jin Q.; Sugai J.V.; Giannobile W.V.	2005
Guided bone regeneration around non-submerged implants in narrow alveolar ridges: a prospective long-term clinical study.	De Boever A.L.; De Boever J.A.	2005
Predisposing conditions for retrograde peri-implantitis, and treatment suggestions.	Quirynen M.; Vogels R.; Alsaadi G.; Naert I.; Jacobs R.; van Steenberghe D.	2005
Peri-implant osteogenesis in health and osteoporosis	Marco F.; Milena F.; Gianluca G.; Vittoria O.	2005
A comparison between enamel matrix derivative and a bioabsorbable membrane to enhance healing around transmucosal immediate post-extraction implants	Cangini F.; Cornelini R.	2005
Immediate restoration of implants placed into fresh extraction sockets for single-tooth replacement: A prospective clinical study	Cornelini R.; Cangini F.; Covani U.; Wilson Jr. T.G.	2005
Influence of alcohol and tobacco habits on peri-implant marginal bone loss: a prospective study.	Galindo-Moreno P.; Fauri M.; Avila-Ortiz G.; Fernández-Barbero J.E.; Cabrera-León A.; Sánchez-Fernández E.	2005
A method using vestibulo-sulcoplasty combining a split-thickness skin graft and a palatal keratinized mucosa graft for peri-implant tissue secondary to oral cancer surgery.	Kao S.Y.; Lui M.T.; Fong J.; Wu D.C.; Wu C.H.; Tu H.F.; Hung K.F.; Yeung T.C.	2005
Performance of short implants in partial restorations: 3-Year follow-up of Osseotite® implants	Goené R.; Bianchesi C.; Hüerzeler M.; Del Lupo R.; Testori T.; Davarpanah M.; Jalbout Z.	2005
Implant-prosthodontic treatment for special care patients: A case series study	Öczakir C.; Balmer S.; Mericske-Stern R.	2005
Combined sinus lift procedure and lateral augmentation. A treatment concept for the surgical and prosthodontic rehabilitation of the extremely atrophic maxilla	Weingart D.; Bublitz R.; Petrin G.; Kälber J.; Ingimarsson S.	2005
Sinus floor elevation: a revised osteotome technique and its biological concept.	Li T.F.	2005
Progression of experimental chronic peri-implantitis in dogs: Clinical and radiographic evaluation	Martins M.C.; Shibli J.A.; Abi-Rached R.S.G.; Marcantonio Jr. E.	2005
Comparative study between the effects of photodynamic therapy and conventional therapy on microbial reduction in ligature-induced peri-implantitis in dogs	Hayek R.R.A.; Araújo N.S.; Gioso M.A.; Ferreira J.; Baptista-Sobrinho C.A.; Yamada Jr. A.M.; Ribeiro M.S.	2005
Implants in regenerated bone in patients treated for generalized aggressive periodontitis: A prospective longitudinal study	Mengel R.; Flores-De-Jacoby L.	2005
Peri-implant bone response to orthodontic loading: Part 1. a histomorphometric study of the effects of implant surface design	Oyonarte R.; Pilliar R.M.; Deporter D.; Woodside D.G.	2005
Peri-implant bone response to orthodontic loading: Part 2. Implant surface geometry and its effect on regional bone remodeling	Oyonarte R.; Pilliar R.M.; Deporter D.; Woodside D.G.	2005
Comparative evaluation of implant designs: influence of diameter, length, and taper on strains in the alveolar crest. A three-dimensional finite-element analysis.	Petrie C.S.; Williams J.L.	2005
Non-invasive method to measure the length of soft tissue from the top of the papilla to the crestal bone	Lee D.-W.; Kim C.-K.; Park K.-H.; Cho K.-S.; Moon I.-S.	2005
Peri-implant bone reaction to immediate, early, and delayed orthodontic loading in humans	Trisi P.; Rebaudi A.	2005
Future perspectives in the treatment of femoro-popliteal arterial occlusions	Gisbertz S.S.; Hissink R.J.; Devries J.-P.P.M.; Moll F.L.	2005
Peri-implantitis	Klinge B.; Hultin M.; Berglundh T.	2005
Analysis of thte possible impact of inflammation severity and early and delayed loading on nitric oxide metabolism around dental implants.	Tözüm T.F.; Türkyilmaz I.; Yamalik N.; Tümer C.; Kilinç A.; Kilinç K.; Karabulut E.; Eratalay K.	2005
Cutting balloons versus conventional long balloons for PCI of long coronary lesions	Han B.; Aboud M.; Nahir M.; Noem F.; Hasin Y.	2005
Clinical consequences of IL-1 genotype on early implant failures in patients under periodontal maintenance	Jansson H.; Hamberg K.; De Bruyn H.; Bratthall G.	2005
Detection of implant crevicular fluid prostaglandin E2 levels for the assessment of peri-implant health: A pilot study	Yalçin S.; Başeğmez C.; Mijiritsky E.; Yalçin F.; Işik G.; Onan U.	2005

Treatment of peri-implantitis by the Vector system.	Karring E.S.; Stavropoulos A.; Ellegaard B.; Karring T.	2005
Implant-supported single-tooth restorations: A 5-year prospective study	Wennström J.L.; Ekestubbe A.; Gröndahl K.; Karlsson S.; Lindhe J.	2005
Long-term results and survival rate of implants treated with guided bone regeneration: a 5-year case series prospective study.	Blanco J.; Alonso A.; Sanz M.	2005
The impact of nicotine on bone healing and osseointegration.	Balatsouka D.; Gotfredsen K.; Lindh C.H.; Berglundh T.	2005
Peri-operative management of patients on Warfarin: A survey of current practice	Shetty A.; Manimaran N.; Faber R.G.; Galland R.B.	2005
Peri-implant care with the CO2 laser: In vitro and in vivo results	Deppe H.; Horch H.-H.; Greim H.; Brill T.; Wagenpfeil S.; Donath K.	2005
Anti-infective therapy with an Er:YAG laser: Influence on peri-implant healing	Sculean A.; Schwarz F.; Becker J.	2005
Clinical and radiographic performance of delayed-immediate single-tooth implant placement associated with peri-implant bone defects. A 2-year prospective, controlled, randomized follow-up report	Schropp L.; Kostopoulos L.; Wenzel A.; Isidor F.	2005
Crestal bone changes around titanium implants. Part I: A retrospective radiographic evaluation in humans comparing two non-submerged implant designs with different machined collar lengths	Hänggi M.P.; Hänggi D.C.; Schoolfield J.D.; Meyer J.; Cochran D.L.; Hermann J.S.	2005
A 5-year prospective study on small diameter screw-shaped oral implants	Comfort M.B.; Chu F.C.S.; Chai J.; Wat P.Y.P.; Chow T.W.	2005
Initial subgingival colonization of 'pristine' pockets.	Quirynen M.; Vogels R.; Pauwels M.; Haffajee A.D.; Socransky S.S.; Uzel N.G.; van Steenberghe D.	2005
Modulating the effects of diabetes on osseointegration with aminoguanidine and doxycycline	Kopman J.A.; Kim D.M.; Rahman S.S.; Arandia J.A.; Karimbux N.Y.; Fiorellini J.P.	2005
Does dedicated pre-abortion contraception counselling help to improve post-abortion contraception uptake?	Yassin A.S.; Cordwell D.	2005
Post-treatment residual tissue in idiopathic retroperitoneal fibrosis: Active residual disease or silent "scar"? A study using 18F-fluorodeoxyglucose positron emission tomography	Vaglio A.; Greco P.; Versari A.; Filice A.; Cobelli R.; Manenti L.; Salvarani C.; Buzio C.	2005
Endovascular stent-graft treatment of aortic dissection: Determinants of post-interventional outcome	Eggebrecht H.; Herold U.; Kuhnt O.; Schmermund A.; Bartel T.; Martini S.; Lind A.; Naber C.K.; Kienbaum P.; Kühl H.; Peters J.; Jakob H.; Erbel R.; Baumgart D.	2005
Therapy of peri-implantitis with resective surgery. A 3-year clinical trial on rough screw-shaped oral implants. Part I: clinical outcome.	Romeo E.; Ghisolfi M.; Murgolo N.; Chiapasco M.; Lops D.; Vogel G.	2005
Clinical evaluation of an Er:YAG laser for nonsurgical treatment of peri-implantitis: a pilot study.	Schwarz F.; Sculean A.; Rothamel D.; Schwenzer K.; Georg T.; Becker J.	2005
Mandibular overdentures supported by two or four endosseous implants. A 5-year prospective study.	Visser A.; Raghoebar G.M.; Meijer H.J.; Batenburg R.H.; Vissink A.	2005
Using photodynamic therapy to treat peri-implantitis. Interview.	Neugebauer J.	2005
Analysis of the presence of pathogens which predict the risk of disease at peri-implant sites through polymerase chain reaction (PCR).	de Leitão J.A.; De Lorenzo J.L.; Avila-Campos M.J.; Sendyk W.R.	2005
Halitosis: Classification, causes, and diagnostic as well as treatment approach - A review	Mahadeva Rao U.S.; Utharkar S.M.; Sundaram C.S.	2005
DNA probe identification of bacteria colonizing internal surfaces of the implant-abutment interface: A preliminary study	Callan D.P.; Cobb C.M.; Williams K.B.	2005
Endovascular aortic aneurysm repair by a multidisciplinary team: Lessons learned and six-year clinical update	Du Toit D.F.; Saaiman J.A.; Carpenter J.P.; Geldenhuys K.M.	2005
Diagnostic parameters for monitoring peri-implant conditions	Salvi G.E.; Lang N.P.	2004
Antimicrobial treatment of peri-implant diseases	Heitz-Mayfield L.J.A.; Lang N.P.	2004
Surgical treatment of peri-implantitis	Schou S.; Berglundh T.; Lang N.P.	2004
Treatment of periimplantitis with laser or ultrasound. A review of the literature	Schwarz F.; Bieling K.; Sculean A.; Herten M.; Becker J.	2004
A systematic review of the survival and complication rates of fixed partial dentures (FPDs) after an observation period of at least 5 years: I. Implant-supported FPDs	Pjetursson B.E.; Tan K.; Lang N.P.; Brägger U.; Egger M.; Zwahlen M.	2004
Immediate or early placement of implants following tooth extraction: Review of biologic basis, clinical procedures, and outcomes	Chen S.T.; Wilson Jr. T.G.; Hämmerle C.H.F.	2004
A systematic review of the survival and complication rates of fixed partial dentures (FPDs) after an observation period of at least 5 years: II. Combined tooth-implant-supported FPDs	Lang N.P.; Pjetursson B.E.; Tan K.; Brägger U.; Egger M.; Zwahlen M.	2004
Private practice results of dental implants. Part I: Survival and evaluation of risk factors - Part II: Surgical and prosthetic complications	Kourtis S.G.; Sotiriadou S.; Voliotis S.; Challas A.	2004
Periimplantitis.	Sánchez-Gárces M.A.; Gay-Escoda C.	2004
Long-term implant survival in the grafted maxilla: Results of a 12-year retrospective study	Yerit K.C.; Posch M.; Hainich S.; Turhani D.; Klug C.; Wanschitz F.; Wagner A.; Watzinger F.; Ewers R.	2004
Interventions for replacing missing teeth: maintaining health around dental implants.	Esposito M.; Worthington H.V.; Thomsen P.; Coulthard P.	2004
A three-year follow-up report of a comparative study of ITI Dental Implants® and Brånemark System® implants in the treatment of the partially edentulous maxilla	Åstrand P.; Engquist B.; Anzén B.; Bergendal T.; Hallman M.; Karlsson U.; Kvint S.; Lysell L.; Rundcranz T.	2004
Bucco-lingual crestal bone changes after immediate and delayed implant placement	Covani U.; Bortolaia C.; Barone A.; Sbordone L.	2004

Carbon dioxide laser and hydrogen peroxide conditioning in the treatment of periimplantitis: An experimental study in the dog	Persson L.G.; Mouhyi J.; Berglundh T.; Sennerby L.; Lindhe J.	2004
Perioperative anticoagulation for patients with mechanical heart valves: A survey of current practice	Garcia D.A.; Ageno W.; Libby E.N.; Bibb J.; Douketis J.; Crowther M.A.	2004
Implant plastic surgery: a review and rationale.	Kazor C.E.; Al-Shammari K.; Sarment D.P.; Misch C.E.; Wang H.L.	2004
Reversible and irreversible peri-implant lesions: report and etiopathogenic analysis of 7 cases.	Wiskott H.W.; Dubrez B.; Scherrer S.S.; Belser U.C.	2004
Experimental peri-implant tissue breakdown around different dental implant surfaces: Clinical and radiographic evaluation in dogs	Martins M.C.; Abi-Rached R.S.G.; Shibli J.A.; Araujo M.W.B.; Marcantonio Jr. E.	2004
Immediate loading of dental implants placed in revascularized fibula free flaps: A clinical report on 2 consecutive patients	Chiapasco M.; Gatti C.	2004
A comparison of characteristics of implant failure and survival in periodontally compromised and periodontally healthy patients: A clinical report	Rosenberg E.S.; Cho S.-C.; Elian N.; Jalbout Z.N.; Froum S.; Evian C.I.	2004
Immediate loading of 2 interforaminal dental implants supporting an overdenture: Clinical and radiographic results after 24 months	Stricker A.; Gutwald R.; Schmelzeisen R.; Gellrich N.G.	2004
Effects of post-surgical cleansing protocols on early plaque control in periodontal and/or periimplant wound healing	Heitz F.; Heitz-Mayfield L.J.A.; Lang N.P.	2004
[Clinical application of miniscrew implant for maximum anchorage cases].	Xun C.L.; Zeng X.L.; Wang X.	2004
Treatment of midfacial defects using prostheses supported by ITI dental implants	Scolozzi P.; Jaques B.	2004
Controversies in cervical discectomy and fusion: Practice patterns among Canadian surgeons	Pickett G.E.; Van Soelen J.; Duggal N.	2004
Correlation of peri-implant health and myeloperoxidase levels: a cross-sectional clinical study.	Liskmann S.; Zilmer M.; Vihalemm T.; Salum O.; Fischer K.	2004
Sustained release of doxycycline for the treatment of peri-implantitis: Randomised controlled trial	Büchter A.; Meyer U.; Kruse-Lösler B.; Joos U.; Kleinheinz J.	2004
Submental cutaneous sinus tract as a result of progressive peri-implantitis: A case report	Nkenke E.; Amann K.; Reich S.; Neukam F.W.; Frankenberger R.	2004
Mandibular two-implant telescopic overdentures.	Heckmann S.M.; Schrott A.; Graef F.; Wichmann M.G.; Weber H.P.	2004
Treatment of incipient peri-implant infections using topical minocycline microspheres versus topical chlorhexidine gel as an adjunct to mechanical debridement.	Renvert S.; Lessem J.; Lindahl C.; Svensson M.	2004
Management and prevention of retrograde peri-implant infection from retained root tips: Two case reports	Park S.-H.; Sorensen W.P.; Wang H.-L.	2004
Reconstruction of maxillary and mandibular defects using prefabricated microvascular fibular grafts and osseointegrated dental implants -- a prospective study.	Jaquiéry C.; Rohner D.; Kunz C.; Bucher P.; Peters F.; Schenk R.K.; Hammer B.	2004
Effects of combined estrogen and progesterone on brain infarction in reproductively senescent female rats	Toung T.J.; Chen T.-Y.; Littleton-Kearney M.T.; Hurn P.D.; Murphy S.J.	2004
Oral rehabilitation with implant-supported fixed partial dentures in periodontitis-susceptible subjects: A 5-year prospective study	Wennström J.L.; Ekestubbe A.; Gröndahl K.; Karlsson S.; Lindhe J.	2004
Clinical analysis of wide-diameter frialit-2 implants	Krennmair G.; Waldenberger O.	2004
A 10-year randomized clinical trial on the influence of splinted and unsplinted oral implants retaining mandibular overdentures: Peri-implant outcome	Naert I.; Alsaadi G.; Van Steenberghe D.; Quirynen M.	2004
Loaded custom-made zirconia and titanium implants show similar osseointegration: An animal experiment	Kohal R.J.; Weng D.; Bächle M.; Strub J.R.	2004
A clinical evaluation of the zygoma fixture: One year of follow-up at 16 clinics	Hirsch J.-M.; Hrnell L.-O.; Henry P.J.; Andreasson L.; Brnemark P.-I.; Chiapasco M.; Gynther G.; Finne K.; Higuchi K.W.; Isaksson S.; Kahnberg K.-E.; Malevez C.; Neukam F.W.; Sevetz Jr. E.; Urgell J.P.; Widmark G.; Bolind P.	2004
Effect of the difference of bone turnover on peri-titanium implant osteogenesis in ovariectomized rats	Okamura A.; Ayukawa Y.; Iyama S.; Koyano K.	2004
Moderate hypothermia for 359 operations to clip cerebral aneurysms	Kimme P.; Fridrikssen S.; Engdahl O.; Hillman J.; Vegfors M.; Sjöberg F.	2004
The use of short, wide implants in posterior areas with reduced bone height: A retrospective investigation	Griffin T.J.; Cheung W.S.	2004
Potential applications of Erbium:YAG laser in periodontics	Ishikawa I.; Aoki A.; Takasaki A.A.	2004
Crestal bone resorption 5 years after implant loading: Clinical and radiologic results with a 2-stage implant system	Ricci G.; Aimetti M.; Stablum W.; Guasti A.	2004
Critical soft tissue parameters of the zygomatic implant	Al-Nawas B.; Wegener J.; Bender C.; Wagner W.	2004
Soft tissue healing around implants placed immediately after tooth extraction without incision: A clinical report	Covani U.; Barone A.; Cornelini R.; Crespi R.	2004
Mandibular overdentures supported by two Brånemark, IMZ or ITI implants: A 5-year prospective study	Meijer H.J.A.; Batenburg R.H.K.; Raghoebar G.M.; Vissink A.	2004
Does excessive occlusal load affect osseointegration? An experimental study in the dog.	Heitz-Mayfield L.J.; Schmid B.; Weigel C.; Gerber S.; Bosshardt D.D.; Jönsson J.; Lang N.P.; Jönsson J.	2004
Implant therapy following liver transplantation: Clinical and microbiological results after 10 years	Heckmann S.H.; Heckmann J.G.; Linke J.J.; Hohenberger W.; Mombelli A.	2004
Single-tooth replacement by immediate implant and connective tissue graft: a 1-9-year clinical evaluation.	Bianchi A.E.; Sanfilippo F.	2004

Prophylactic root resection and periapical grafting for anterior implant aethetics: an integration of tissue-reconstruction and tissue-preservation concepts.	Kan J.Y.; Rungcharassaeng K.	2004
The Agility total ankle arthroplasty: Seven to sixteen-year follow-up	Knecht S.I.; Estin M.; Callaghan J.J.; Zimmerman M.B.; Alliman K.J.; Alvine F.G.; Saltzman C.L.	2004
Intraoperative hip fractures: Ruining your day	Cameron H.U.	2004
Expression of soluble intercellular adhesion molecule-1 in peri-implant sulcular fluid	Xie Z.-G.; Huang P.; Liang X.; Chen M.; Yang X.-Y.	2004
Peri-implant diseases. A systematic review of the literature.	Romeo E.; Ghisolfi M.; Carmagnola D.	2004
A technique for standardized evaluation of soft and hard peri-implant tissues in partially edentulous patients	Meijndert L.; Meijer H.J.A.; Raghoebar G.M.; Vissink A.	2004
Experimental zirconia abutments for implant-supported single-tooth restorations in esthetically demanding regions: 4-Year results of a prospective clinical study	Glauser R.; Sailer I.; Wohlwend A.; Studer S.; Schibli M.; Schärer P.	2004
Early loading of ITI implants supporting a maxillary full-arch prosthesis: 1-Year data of a prospective, randomized study	Fischer K.; Stenberg T.	2004
A prospective evaluation of Frialoc implants with ball-abutments in the edentulous mandible: 1-year results.	Cune M.S.; Verhoeven J.W.; Meijer G.J.	2004
Submerged or non-submerged healing of endosseous implants to be used in the rehabilitation of partially dentate patients.	Cecchinato D.; Olsson C.; Lindhe J.	2004
Evaluation of Matrix Metalloproteinase-9 and Tissue Inhibitor of Metalloproteinase-1 Levels in Peri-implant Sulcular Fluid	Lu S.-H.; Liang X.; Xie Z.-G.; Zhang J.-Y.; Xu L.; Sun H.-Q.	2004
Immediate loading of hydroxyapatite-coated implants in the maxillary premolar area: Three-year results of a pilot study	Proussaefs P.; Lozada J.	2004
Cemented versus screw-retained implant-supported single-tooth crowns: A 4-year prospective clinical study	Vigolo P.; Givani A.; Majzoub Z.; Cordioli G.	2004
Comparative value of attachment measurements in implant dentistry	Deppe H.; Wagenpfeil S.; Donath K.	2004
Effect of platelet-rich plasma on the peri-implant bone response: an experimental study.	Fontana S.; Olmedo D.G.; Linares J.A.; Guglielmotti M.B.; Crosa M.E.	2004
Long-term survival and success of oral implants in the treatment of full and partial arches: A 7-year prospective study with the ITI dental implant system	Romeo E.; Lops D.; Margutti E.; Ghisolfi M.; Chiapasco M.; Vogel G.	2004
The vanguard endovascular stent-graft: Mid-term results from a single centre	Holtham S.J.; Rose J.D.G.; Jackson R.W.; Lees T.A.; Wyatt M.G.	2004
Effect of implant design on survival and success rates of titanium oral implants: a 10-year prospective cohort study of the ITI Dental Implant System.	Karoussis I.K.; Brägger U.; Salvi G.E.; Bürgin W.; Lang N.P.	2004
Association between periodontal and peri-implant conditions: a 10-year prospective study.	Karoussis I.K.; Müller S.; Salvi G.E.; Heitz-Mayfield L.J.; Brägger U.; Lang N.P.	2004
Histomorphometrical and clinical comparison of submerged and nonsubmerged implants subjected to experimental peri-implantitis in dogs.	Zechner W.; Kneissel M.; Kim S.; Ulm C.; Watzek G.; Plenk Jr. H.	2004
Factors regulating osteoclast formation in human tissues adjacent to peri-implant bone loss: Expression of receptor activator NFκB, RANK ligand and osteoprotegerin	Crotti T.N.; Smith M.D.; Findlay D.M.; Zreiqat H.; Ahern M.J.; Weedon H.; Hatzinikolous G.; Capone M.; Holding C.; Haynes D.R.	2004
The role of keratinized tissue and attached gingiva in maintaining periodontal/peri-implant health.	Marquez I.C.	2004
Clinical Evaluation of Small-Diameter ITI Implants: A Prospective Study	Zinsli B.; Sägesser T.; Mericske E.; Mericske-Stern R.	2004
A Prospective Study of the Risk Factors Associated with Failure of Mini-implants Used for Orthodontic Anchorage	Cheng S.-J.; Tseng I.-Y.; Lee J.-J.; Kok S.-H.	2004
[The clinical evaluation on aesthetic effectiveness of implant supported dentures in partially edentulous patients].	Tang Q.; Liang X.; Huang L.; Zhang Q.H.	2004
Prospective, Randomized Evaluation of Thrombectomy Prior to Percutaneous Intervention in Diseased Saphenous Vein Grafts and Thrombus-Containing Coronary Arteries	Stone G.W.; Cox D.A.; Babb J.; Nukta D.; Bilodeau L.; Cannon L.; Stuckey T.D.; Hermiller J.; Cohen E.A.; Low R.; Bailey S.R.; Lansky A.J.; Kuntz R.E.	2003
Levels and molecular forms of MMP-7 (matrilysin-1) and MMP-8 (collagenase-2) in diseased human peri-implant sulcular fluid	Kivelä-Rajamäki M.; Maisi P.; Srinivas R.; Tervahartiala T.; Teronen O.; Husa V.; Salo T.; Sorsa T.	2003
Simultaneous or staged installation with guided bone augmentation of transmucosal titanium implants: A 3-year prospective cohort study	Christensen D.K.; Karoussis I.K.; Joss A.; Hämmerle C.H.F.; Lang N.P.	2003
Dental implants: Maintenance, care and treatment of peri-implant infection	Chen S.; Darby I.	2003
Rotational Panoramic Versus Intraoral Rectangular Radiographs for Evaluation of Peri-implant Bone Loss in the Anterior Atrophic Mandible	Zechner W.; Watzak G.; Gahleitner A.; Busenlechner D.; Tepper G.; Watzek G.	2003
Four-year Follow-up of Larger-Diameter Implants Placed in Fresh Extraction Sockets Using a Resorbable Membrane or a Resorbable Alloplastic Material	Prosper L.; Gherlone E.F.; Redaelli S.; Quaranta M.	2003
Interventions for replacing missing teeth: different types of dental implants.	Esposito M.; Worthington H.V.; Thomsen P.; Coulthard P.	2003
Early loading of nonsubmerged titanium implants with a sandblasted and acid-etched (SLA) surface: 3-Year results of a prospective study in partially edentulous patients	Bornstein M.M.; Lussi A.; Schmid B.; Belser U.C.; Buser D.	2003
Early loading of endosseous implants in the augmented maxilla: A 1-year prospective study	Raghoebar G.M.; Schoen P.; Meijer H.J.A.; Stellingsma K.; Vissink A.	2003

Gelatinase B is associated with peri-implant bone loss	Ma J.; Kitti U.; Hanemaaijer R.; Teronen O.P.; Sorsa T.A.; Natah S.; Tensing E.-K.; Konttinen Y.T.	2003
Early Functional Loading of Unsplinted Roughened Surface Implants with Mandibular Overdentures 2 Weeks after Surgery	Payne A.G.T.; Tawse-Smith A.; Thompson W.M.; Kumara R.	2003
Histologic analysis of clinically retrieved immediately loaded titanium implants: A report of 11 cases	Degidi M.; Scarano A.; Petrone G.; Piattelli A.	2003
Tissue reactions to abutment shift: An experimental study in dogs	Abrahamsson I.; Berglundh T.; Sekino S.; Lindhe J.	2003
Oral microbial biofilms and plaque-related diseases: microbial communities and their role in the shift from oral health to disease.	Sbordone L.; Bortolaia C.	2003
Dental Implants in Reconstructed Jaws: Implant Longevity and Peri-Implant Tissue Outcomes	Cheung L.K.; Leung A.C.F.	2003
Implant Treatment in the Edentulous Mandible: A Prospective Study on Brånemark System Implants over More than 20 Years	Ekelund J.-A.; Lindquist L.W.; Carlsson G.E.; Jemt T.	2003
Abciximab Reduces Monocyte Tissue Factor in Carotid Angioplasty and Stenting	Kopp C.W.; Steiner S.; Nasel C.; Seidinger D.; Mlekusch I.; Lang W.; Bartok A.; Ahmadi R.; Minar E.	2003
Five-year clinical, microbiological, and radiological outcome following treatment of peri-implantitis in man	Leonhardt Å.; Dahlén G.; Renvert S.	2003
Extra-oral endosseous craniofacial implants: Current status and future developments	Abu-Serriah M.M.; McGowan D.A.; Moos K.F.; Bagg J.	2003
Dental implants in patients with type 2 diabetes mellitus: a clinical study.	Peled M.; Ardekian L.; Tagger-Green N.; Gutmacher Z.; Machtei E.E.	2003
Peri-implant mucosal level in patients treated with implant-supported fixed prostheses: A 1-year follow-up study	Ekfeldt A.; Eriksson A.; Johansson L.-Å.	2003
3-year prospective multicenter study on one-stage implant surgery and early loading in the edentulous mandible.	Raghoebar G.M.; Friberg B.; Grunert I.; Hobkirk J.A.; Tepper G.; Wendelhag I.	2003
Mandibulotomy and implant insertion	Schoen P.J.; Raghoebar G.M.; Vissink A.; Roodenburg J.L.N.	2003
A clinical study on unfavorable cases of dental implant	Noriko T.; Kento T.; Tsuneji O.; Motohiro M.; Kozue M.; Makoto S.; Shohei K.	2003
Implant-retained mandibular overdentures with immediate loading: a 3- to 8-year prospective study on 328 implants.	Chiapasco M.; Gatti C.	2003
A prospective 5-year multicenter study of the cresco implantology concept	Helldén L.; Ericson G.; Elliot A.; Fornell J.; Holmgren K.; Nilner K.; Olsson C.O.	2003
The current status of laser applications in dentistry	Walsh L.J.	2003
Manipulation of the peri-implant tissue for better maintenance: a periodontal perspective.	Maksoud M.A.	2003
Influence of an Er:YAG laser on the surface structure of titanium implants	Schwarz F.; Rothamel D.; Becker J.	2003
A multidisciplinary approach to enhance implant esthetics: case report.	el Salam el Askary A.	2003
The effects of tobacco use on oral health	Sham A.S.K.; Cheung L.K.; Jin L.J.; Corbet E.F.	2003
Autogenous bone graft and ePTFE membrane in the treatment of peri-implantitis. I. Clinical and radiographic observations in cynomolgus monkeys.	Schou S.; Holmstrup P.; Jørgensen T.; Stoltze K.; Hjørting-Hansen E.; Wenzel A.	2003
Clinical and radiographic evaluation of soft and hard tissue changes around implants: A pilot study	Joly J.C.; Martorelli de Lima A.F.; Carvalho da Silva R.	2003
Implant surface preparation in the surgical treatment of experimental peri-implantitis with autogenous bone graft and ePTFE membrane in cynomolgus monkeys.	Schou S.; Holmstrup P.; Jørgensen T.; Skovgaard L.T.; Stoltze K.; Hjørting-Hansen E.; Wenzel A.	2003
Understanding peri-implant endosseous healing.	Davies J.E.	2003
Clinical complications with implants and implant prostheses.	Goodacre C.J.; Bernal G.; Rungcharassaeng K.; Kan J.Y.	2003
Differential diagnosis and treatment strategies for peri-implant diseases	Kozlovsky A.; Mozes O.; Nemcovsky C.E.; Artzi Z.	2003
Bone-implant contact on machined and dual acid-etched surfaces after 2 months of healing in the human maxilla	Trisi P.; Lazzara R.; Rebaudi A.; Rao W.; Testori T.; Porter S.S.	2003
Mandibular fracture caused by peri-implant bone loss: Report of a case	Meijer H.J.A.; Raghoebar G.M.; Visser A.	2003
Long-term implant prognosis in patients with and without a history of chronic periodontitis: a 10-year prospective cohort study of the ITI Dental Implant System.	Karoussis I.K.; Salvi G.E.; Heitz-Mayfield L.J.; Brägger U.; Hämmerle C.H.; Lang N.P.	2003
Treatment of peri-implant infections: a literature review.	Roos-Jansåker A.M.; Renvert S.; Egelberg J.	2003
Implant-supported fixed cantilever prostheses in partially edentulous arches. A seven-year prospective study.	Romeo E.; Lops D.; Margutti E.; Ghisolfi M.; Chiapasco M.; Vogel G.	2003
Clinical and radiologic evaluation of 2-stage IMZ implants placed in a single-stage procedure: 2-year results of a prospective comparative study.	Heydenrijk K.; Raghoebar G.M.; Meijer H.J.; Stegenga B.	2003
Laminin-5 gamma2-chain and collagenase-2 (MMP-8) in human peri-implant sulcular fluid.	Kivelä-Rajamäki M.J.; Teronen O.P.; Maisi P.; Husa V.; Tervahartiala T.I.; Pirilä E.M.; Salo T.A.; Mellanen L.; Sorsa T.A.	2003
Fixed bridgework rehabilitation of an edentulous mandible using different implant systems for superior results--review of the literature and a case report	Kolerman R.; Venezia E.	2003
Imaging of periimplant bone levels of implants with buccal bone defects.	Schliephake H.; Wichmann M.; Donnerstag F.; Vogt S.	2003

Trapezial arthroplasty with silicone rubber implantation for advanced osteoarthritis of the trapeziometacarpal joint of the thumb	MacDermid J.C.; Roth J.H.; Raj Rampersaud Y.; Bain G.I.	2003
Effect of intracrevicular restoration margins on peri-implant health: clinical, biochemical, and microbiologic findings around esthetic implants up to 9 years.	Giannopoulou C.; Bernard J.P.; Buser D.; Carrel A.; Belser U.C.	2003
Treatment of ligature-induced peri-implantitis by lethal photosensitization and guided bone regeneration: A preliminary histologic study in dogs	Shibli J.A.; Martins M.C.; Nociti Jr. F.H.; Garcia V.G.; Marcantonio Jr. E.	2003
Lethal photosensitization in microbiological treatment of ligature-induced peri-implantitis: a preliminary study in dogs.	Shibli J.A.; Martins M.C.; Theodoro L.H.; Lotufo R.F.; Garcia V.G.; Marcantonio E.J.	2003
Persistent acute inflammation at the implant-abutment interface.	Broggini N.; McManus L.M.; Hermann J.S.; Medina R.U.; Oates T.W.; Schenk R.K.; Buser D.; Mellonig J.T.; Cochran D.L.	2003
Management of peri-implantitis	Triplett R.G.; Andrews J.A.; Hallmon W.W.	2003
Bucco-lingual bone remodeling around implants placed into immediate extraction sockets: A case series	Covani U.; Cornelini R.; Barone A.	2003
Treatment of advanced periodontal destruction with immediately loaded implants and simultaneous bone augmentation: A case report	Romanos G.E.	2003
Temperature changes induced by 809-nm GaAlAs laser at the implant-bone interface during simulated surface decontamination.	Kreisler M.; Al Haj H.; D'Hoedt B.	2003
Soft tissue reaction to microgrooved poly-L-lactic acid implants loaded with transforming growth factor-β3	Parker J.A.T.C.; Walboomers X.F.; Von Den Hoff J.W.; Maltha J.C.; Jansen J.A.	2003
The use of a locking custom contoured blade plate for peri-articular nonunions	Harvey E.J.; Henley M.B.; Swiontkowski M.F.; Agel J.; Benirschke S.K.	2003
Immunohistochemical characteristics of inflammatory lesions at implants.	Gualini F.; Berglundh T.	2003
Immediate placement and provisionalization of maxillary anterior single implants: 1-year prospective study.	Kan J.Y.; Rungcharassaeng K.; Lozada J.	2003
In vitro effects on rough implant surfaces of different instrumentations used in the surgical therapy of peri-implantitis	Espedito Di Lauro A.; Morgese F.; Squillace A.; Ramaglia L.	2003
Two-implant mandibular overdentures: simple to fabricate and easy to wear.	Klemetti E.; Chehade A.; Takanashi Y.; Feine J.S.	2003
Influence of platelet-rich plasma on osseous healing of dental implants: a histologic and histomorphometric study in minipigs.	Zechner W.; Tangl S.; Tepper G.; Fürst G.; Bernhart T.; Haas R.; Mailath G.; Watzek G.	2003
Subantroscopic laterobasal sinus floor augmentation (SALSA): an up-to-5-year clinical study.	Engelke W.; Schwarzwäller W.; Behnsen A.; Jacobs H.G.	2003
Evaluation of distraction implants for prosthetic treatment after vertical alveolar ridge distraction: a clinical investigation.	Feichtinger M.; Gaggl A.; Schultes G.; Kärcher H.	2003
Collagen as an implantable material in medicine and dentistry.	Patino M.G.; Neiders M.E.; Andreana S.; Noble B.; Cohen R.E.	2002
Apical (retrograde) peri-implantitis: a case report of an active lesion.	Flanagan D.	2002
Osteocalcin, deoxypyridinoline and interleukin-1beta in peri-implant crevicular fluid of patients with peri-implantitis.	Murata M.; Tatsumi J.; Kato Y.; Suda S.; Nunokawa Y.; Kobayashi Y.; Takeda H.; Araki H.; Shin K.; Okuda K.; Miyata T.; Yoshie H.	2002
Healing after standardized clinical probing of the perlimplant soft tissue seal: a histomorphometric study in dogs.	Etter T.H.; Håkanson I.; Lang N.P.; Trejo P.M.; Caffesse R.G.	2002
A systematic review of the incidence of biological and technical complications in implant dentistry reported in prospective longitudinal studies of at least 5 years.	Berglundh T.; Persson L.; Klinge B.	2002
Three-dimensional finite element analysis of implant stability in the atrophic posterior maxilla: a mathematical study of the sinus floor augmentation.	Tepper G.; Haas R.; Zechner W.; Krach W.; Watzek G.	2002
A systematic review of the survival of implants in bone sites augmented with barrier membranes (guided bone regeneration) in partially edentulous patients.	Hämmerle C.H.; Jung R.E.; Feloutzis A.	2002
Immediate loading of Brånemark implants: a 24-month follow-up of a comparative prospective pilot study between mandibular overdentures supported by Conical transmucosal and standard MK II implants.	Gatti C.; Chiapasco M.	2002
Nonsubmerged and submerged implants in the treatment of the partially edentulous maxilla.	Astrand P.; Engquist B.; Anzén B.; Bergendal T.; Hallman M.; Karlsson U.; Kvint S.; Lysell L.; Rundcrantz T.	2002
Local administration of growth hormone enhances periimplant bone reaction in an osteoporotic rabbit model.	Tresguerres I.F.; Clemente C.; Donado M.; Gómez-Pellico L.; Blanco L.; Alobera M.A.; Tresguerres J.A.	2002
Conventional and early loading of unsplinted ITI implants supporting mandibular overdentures.	Payne A.G.; Tawse-Smith A.; Duncan W.D.; Kumara R.	2002
Diagnoses, clinical classification, and proposed treatment of spontaneous early exposure of submerged implants.	Barboza E.P.; Caúla A.L.	2002
Artificial disc: Preliminary results of a prospective study in the United States	Hochschuler S.H.; Ohnmeiss D.D.; Guyer R.D.; Blumenthal S.L.	2002
A 5-year longitudinal study of the clinical effectiveness of ITI solid-screw implants in the treatment of mandibular edentulism.	Behneke A.; Behneke N.; d'Hoedt B.	2002
Microbiota around root-form endosseous implants: a review of the literature.	Heydenrijk K.; Meijer H.J.; van der Reijden W.A.; Raghoebar G.M.; Vissink A.; Stegenga B.	2002
Temperature changes at the implant-bone interface during simulated surface decontamination with an Er:YAG laser.	Kreisler M.; Al Haj H.; d'Hoedt B.	2002
Clinical response to 2 different therapeutic regimens to treat peri-implant mucositis	Porras R.; Anderson G.B.; Caffesse R.; Narendran S.; Trejo P.M.	2002

Interleukin-1beta, tumor necrosis factor-alpha levels and neutrophil elastase activity in peri-implant crevicular fluid.	Ataoglu H.; Alptekin N.O.; Haliloglu S.; Gursel M.; Ataoglu T.; Serpek B.; Durmus E.	2002
Influence of the size of the microgap on crestal bone levels in non-submerged dental implants: A radiographic study in the canine mandible	King G.N.; Hermann J.S.; Schoofield J.D.; Busen D.; Cochran D.L.	2002
Bone growth factors in maxillofacial skeletal reconstruction	Schliephake H.	2002
Outcome of implant therapy in relation to experienced loss of periodontal bone support: a retrospective 5- year study.	Hardt C.R.; Gröndahl K.; Lekholm U.; Wennström J.L.	2002
Peri-implant alveolar bone loss with respect to bone quality after use of the osteotome technique: results of a retrospective study.	Strietzel F.P.; Nowak M.; Küchler I.; Friedmann A.	2002
Implant-retained mandibular overdentures with ITI implants.	Romeo E.; Chiapasco M.; Lazza A.; Casentini P.; Ghisolfi M.; Iorio M.; Vogel G.	2002
Invasive infection due to Penicillium species other than P. marneffei	Lyratzopoulos G.; Ellis M.; Nerringer R.; Denning D.W.	2002
Hip fracture surgery: Is the pre-operative American Society of Anesthesiologists (ASA) score a predictor of functional outcome?	Michel J.-P.; Klopfenstein C.; Hoffmeyer P.; Stern R.; Grab B.	2002
Dental implant treatment in diabetic patients.	Farzad P.; Andersson L.; Nyberg J.	2002
Rehabilitation of a bulimic patient using endosteal implants.	Ambard A.; Mueninghoff L.	2002
Titanium deposition after peri-implant care with the carbon dioxide laser.	Deppe H.; Greim H.; Brill T.; Wagenpfeil S.	2002
A follow-up study of maxillary implants supporting an overdenture: clinical and radiographic results.	Mericske-Stern R.; Oetterli M.; Kiener P.; Mericske E.	2002
Regeneration of an osseous peri-implantitis lesion.	El Chaar E.S.; Jalbout Z.N.	2002
The single-tooth implant: A viable alternative for single-tooth replacement	Mayer T.M.; Hawley C.E.; Gunsolley J.C.; Feldman S.	2002
Two-stage IMZ implants and ITI implants inserted in a single-stage procedure. A prospective comparative study.	Heydenrijk K.; Raghoebar G.M.; Meijer H.J.; van der Reijden W.A.; van Winkelhoff A.J.; Stegenga B.	2002
Non contrast computerized tomography and dynamic renal scintigraphy in the evaluation of patients with renal colic: Are both necessary?	German I.; Lantsberg S.; Crystal P.; Assali M.; Rachinsky I.; Kaneti J.; Neulander E.Z.	2002
Interventions for replacing missing teeth: maintaining and re-establishing healthy tissues around dental implants.	Esposito M.; Worthington H.V.; Coulthard P.; Jokstad A.	2002
Preliminary results of a phase I/II study of HDR brachytherapy alone for T1/T2 breast cancer	Wazer D.E.; Berle L.; Graham R.; Chung M.; Rothschild J.; Graves T.; Cady B.; Ulin K.; Ruthazer R.; DiPetrillo T.A.	2002
Crestal bone loss around submerged and exposed unloaded dental implants: a radiographic and microbiological descriptive study.	Barboza E.P.; Caúla A.L.; Carvalho W.R.	2002
Expression of endothelial adhesion molecules in the alveolar ridge mucosa, gingiva and periimplant mucosa.	Zitzmann N.U.; Berglundh T.; Marinello C.P.; Lindhe J.	2002
Microbiology and antimicrobial therapy of peri-implantitis.	Mombelli A.	2002
The influence of controlled occlusal overload on peri-implant tissue. part 4: a histologic study in monkeys.	Miyata T.; Kobayashi Y.; Araki H.; Ohto T.; Shin K.	2002
The accuracy and reliability of radiographic methods for the assessment of marginal bone level around oral implants	De Smet E.; Jacobs R.; Gijbels F.; Naert I.	2002
Biofilms of the oral cavity. Formation, development and involvement in the onset of diseases related to bacterial plaque increase	Bortolaia C.; Sbordone L.	2002
The causes of early implant bone loss: Myth or science?	Oh T.-J.; Yoon J.; Misch C.E.; Wang H.-L.	2002
Early loading of unsplinted implants supporting mandibular overdentures using a one-stage operative procedure with two different implant systems: a 2-year report.	Tawse-Smith A.; Payne A.G.; Kumara R.; Thomson W.M.	2002
A review on peri-implant crevicular fluid assays potential in monitoring and predicting peri-implant tissue responses.	Kaklamanos E.G.; Tsalikis L.	2002
Long-term follow-up of osseointegrated titanium implants using clinical, radiographic and microbiological parameters.	Leonhardt A.; Gröndahl K.; Bergström C.; Lekholm U.	2002
Clinical utility of a genetic susceptibility test for severe chronic periodontitis: a critical evaluation.	Greenstein G.; Hart T.C.	2002
Treatment of the infected implant site using platelet-rich plasma.	Petrungaro P.S.	2002
The use of reduced healing times on ITI implants with a sandblasted and acid-etched (SLA) surface: early results from clinical trials on ITI SLA implants.	Cochran D.L.; Buser D.; ten Bruggenkate C.M.; Weingart D.; Taylor T.M.; Bernard J.P.; Peters F.; Simpson J.P.	2002
Long-term clinical effectiveness of oral implants in the treatment of partial edentulism. Seven-year life table analysis of a prospective study with ITI dental implants system used for single-tooth restorations.	Romeo E.; Chiapasco M.; Ghisolfi M.; Vogel G.	2002
Peri-implant bone regeneration using recombinant human bone morphogenetic protein-2 in a canine model: a dose-response study.	Tatakis D.N.; Koh A.; Jin L.; Wozney J.M.; Rohrer M.D.; Wikesjö U.M.	2002
Immediate implant placement and temporization in extraction and healing sites.	Saadoun A.P.	2002
Long-term stability of osseointegrated implants in augmented bone: a 5-year prospective study in partially edentulous patients.	Buser D.; Ingimarsson S.; Dula K.; Lussi A.; Hirt H.P.; Belser U.C.	2002
Stability of dental implants in microvascular osseous transplants	Schultes G.; Gaggl A.; Kärcher H.	2002
Flapless implant surgery: a 10-year clinical retrospective analysis.	Campelo L.D.; Camara J.R.	2002
Patient selection for endosseous dental implants: oral and systemic considerations.	Sugerman P.B.; Barber M.T.	2002

Peri-implant tissue response of immediately loaded, threaded, HA-coated implants: 1-year results.	Rungcharassaeng K.; Lozada J.L.; Kan J.Y.; Kim J.S.; Campagni W.V.; Munoz C.A.	2002
A long-term follow-up of 76 Bränemark single-tooth implants.	Haas R.; Polak C.; Fürhauser R.; Mailath-Pokorny G.; Dörtbudak O.; Watzek G.	2002
Progressive bone adaptation of titanium implants during and after orthodontic load in humans.	Trisi P.; Rebaudi A.	2002
Single-tooth replacement with the Frialit-2 system: a retrospective clinical analysis of 146 implants.	Krennmair G.; Schmidinger S.; Waldenberger O.	2002
Surgical therapy of peri-implant disease: A 3-year follow-up study of cases treated with 3 different techniques of bone regeneration	Khoury F.; Buchmann R.	2001
Re-osseointegration after treatment of peri-implantitis at different implant surfaces. An experimental study in the dog.	Persson L.G.; Berglundh T.; Lindhe J.; Sennerby L.	2001
One-stage operative procedure using two different implant systems: a prospective study on implant overdentures in the edentulous mandible.	Tawse-Smith A.; Perio C.; Payne A.G.; Kumara R.; Thomson W.M.	2001
Orthodontic aspects of the use of oral implants in adolescents: a 10-year follow-up study.	Thilander B.; Odman J.; Lekholm U.	2001
Implant-supported denture in a patient with Huntington's disease: interdisciplinary aspects.	Jackowski J.; Andrich J.; Käppeler H.; Zöllner A.; Jöhren P.; Müller T.	2001
Implant-supported fixed prostheses for the rehabilitation of periodontally compromised dentitions: a 3-year prospective clinical study.	Yi S.W.; Ericsson I.; Kim C.K.; Carlsson G.E.; Nilner K.	2001
Influence of the size of the microgap on crestal bone changes around titanium implants. A histometric evaluation of unloaded non-submerged implants in the canine mandible	Hermann J.S.; Schoolfied J.D.; Schenk R.K.; Buser D.; Cochran D.L.	2001
A multi-center study comparing dual acid-etched and machined-surfaced implants in various bone qualities	Khang W.; Feldman S.; Hawley C.E.; Gunsolley J.	2001
Abnormal echocardiography during weaning from cardiopulmonary bypass	Tolley P.M.; Martin R.R.	2001
Predictable peri-implant gingival aesthetics: surgical and prosthodontic rationales.	Kois J.C.; Kan J.Y.	2001
A longitudinal study on mandibular implants supporting an overdenture: the influence of retention mechanism and anatomic-prosthetic variables on periimplant parameters.	Oetterli M.; Kiener P.; Mericske-Stern R.	2001
Use of upper-lid blepharoplasty full-thickness skin for peri-implant keratinized tissue grafting: A report of 2 cases	Deeb G.R.; Le B.T.; Ueeck B.A.; Dierks E.J.	2001
Connective tissue orientation around dental implants in a canine model.	Comut A.A.; Weber H.P.; Shortkroff S.; Cui F.Z.; Spector M.	2001
Evaluation following functional loading of titanium fixtures placed in ridges augmented by deproteinized bone mineral. A human case study.	Mayfield L.J.; Skoglund A.; Hising P.; Lang N.P.; Attström R.	2001
Brånemark System and ITI Dental Implant System for treatment of mandibular edentulism. A comparative randomized study: 3-year follow-up.	Moberg L.E.; Köndell P.A.; Sagulin G.B.; Bolin A.; Heimdahl A.; Gynther G.W.	2001
Effect of total body irradiation on peri-implant tissue reaction: an experimental study.	Renou S.J.; Guglielmotti M.B.; de la Torre A.; Cabrini R.L.	2001
Absorbable versus nonabsorbable membranes and bone grafts in the treatment of ligature-induced peri-implantitis defects in dogs: a histometric investigation.	Nociti Jr. F.H.; Machado M.A.; Stefani C.M.; Sallum E.A.	2001
A prospective study of treatment of severely resorbed maxillae with narrow nonsubmerged implants: results after 1 year of loading.	Hallman M.	2001
The effect of dental implant spacing on peri-implant bone using the rabbit (Oryctolagus cuniculus) tibia model.	Hatley C.L.; Cameron S.M.; Cuenin M.F.; Parker M.H.; Thompson S.H.; Harvey S.B.	2001
Alumina ceramic implant abutments used for single-tooth replacement: a prospective 1- to 3-year multicenter study.	Andersson B.; Taylor A.; Lang B.R.; Scheller H.; Schärer P.; Sorensen J.A.; Tarnow D.	2001
Stability of dental implants in microvascular scapula and iliac crest transplants	Gaggl A.; Schultes G.; Kärcher H.	2001
Per-implant care of ailing implants with the carbon dioxide laser.	Deppe H.; Horch H.H.; Henke J.; Donath K.	2001
Transmission electron microscopy of intracellular particles of polyethylene from joint replacement prostheses: Size distribution and cellular response	Benz E.B.; Federman M.; Godleski J.J.; Bierbaum B.E.; Thornhill T.S.; Spector M.	2001
Incidence and primary prevention of complications related to the placement of dental implants	Torelli S.; Bercy P.	2001
Clinical and microbiological evaluation of ligature-induced peri-implantitis and periodontitis in dogs.	Nociti Jr. F.H.; Cesco De Toledo R.; Machado M.A.; Stefani C.M.; Line S.R.; Gonçalves R.B.	2001
The influence of plaque and/or excessive loading on marginal soft and hard tissue reactions around Brånemark implants: a review of literature and experience.	De Smet E.; van Steenberghe D.; Quirynen M.; Naert I.	2001
Osseointegrated implants in patients treated for generalized chronic periodontitis and generalized aggressive periodontitis: 3- and 5-year results of a prospective long-term study.	Mengel R.; Schröder T.; Flores-de-Jacoby L.	2001
Clinical evaluation and prosthetic complications of single tooth replacements by non-submerged implants.	Mericske-Stern R.; Grütter L.; Rösch R.; Mericske E.	2001
Retrospective analysis of the influence of abutment structure design on the success of implant unit. A 3-year controlled follow-up study.	Bambini F.; Lo Muzio L.; Procaccini M.	2001
Nonsubmerged implants in the treatment of the edentulous lower jaw: a 5-year prospective longitudinal study of ITI hollow screws.	Hellem S.; Karlsson U.; Almfeldt I.; Brunell G.; Hamp S.E.; Astrand P.	2001
One-year prospective evaluation of the early loading of unsplinted conical Brånemark fixtures with mandibular overdentures immediately following surgery.	Payne A.G.; Tawse-Smith A.; Kumara R.; Thomson W.M.	2001

Treatment of peri-implant defects with the vertical ridge augmentation procedure: a patient report.	Tinti C.; Parma-Benfenati S.	2001
Crestal bone changes around titanium implants: a methodologic study comparing linear radiographic with histometric measurements.	Hermann J.S.; Schoolfield J.D.; Nummikoski P.V.; Buser D.; Schenk R.K.; Cochran D.L.	2001
Five-year prospective follow-up report of the Astra tech standard dental implant in clinical treatment.	Weibrich G.; Buch R.S.; Wegener J.; Wagner W.	2001
Influence of patient age on the success rate of dental implants supporting an overdenture in an edentulous mandible: a 3-year prospective study.	Meijer H.J.; Batenburg R.H.; Raghoebar G.M.	2001
Implant-retained mandibular overdentures with Brånemark System MKII implants: a prospective comparative study between delayed and immediate loading.	Chiapasco M.; Abati S.; Romeo E.; Vogel G.	2001
Spontaneous early exposure of submerged endosseous implants resulting in crestal bone loss: a clinical evaluation between stage I and stage II surgery.	Tal H.; Artzi Z.; Moses O.; Nemcovsky C.E.; Kozlovsky A.	2001
Immediate postextraction implant placement with root-analog stepped implants: surgical procedure and statistical outcome after 6 years.	Gomez-Roman G.; Kruppenbacher M.; Weber H.; Schulte W.	2001
Tooth-implant supported fixed prostheses: a retrospective multicenter study.	Lindh T.; Dahlgren S.; Gunnarsson K.; Josefsson T.; Nilson H.; Wilhelmsson P.; Gunne J.	2001
Retrospective assessment of clinical and microbiological factors affecting periimplant tissue conditions.	Rutar A.; Lang N.P.; Buser D.; Bürgin W.; Mombelli A.	2001
General and oral aspects of osteoporosis: a review.	von Wowern N.	2001
Long-term evaluation of non-submerged hollow cylinder implants. Clinical and radiographic results.	Merickse-Stern R.; Aerni D.; Geering A.H.; Buser D.	2001
Glycosaminoglycans in peri-implant sulcus fluid from implants placed in sinus-inlay bone grafts.	Johansson B.; Smedberg J.I.; Langley M.; Embery G.	2001
Vascular endothelial growth factor and microvessel density around healthy and failing dental implants.	Cornelini R.; Artese L.; Rubini C.; Fioroni M.; Ferrero G.; Santinelli A.; Piattelli A.	2001
Development and treatment of retrograde peri-implantitis involving a site with a history of failed endodontic and apicoectomy procedures: a series of reports.	Ayangco L.; Sheridan P.J.	2001
Long-term results of implants treated with guided bone regeneration: a 5-year prospective study.	Zitzmann N.U.; Schärer P.; Marinello C.P.	2001
Bone and soft tissue integration to titanium implants with different surface topography: an experimental study in the dog.	Abrahamsson I.; Zitzmann N.U.; Berglundh T.; Wennerberg A.; Lindhe J.	2001
A clinical and histological evaluation of titanium mini-implants as anchors for orthodontic intrusion in the beagle dog.	Ohmae M.; Saito S.; Morohashi T.; Seki K.; Qu H.; Kanomi R.; Yamasaki K.I.; Okano T.; Yamada S.; Shibasaki Y.	2001
Augmentation of alveolar bone and dental implant osseointegration: clinical implications of studies with rhBMP-2.	Wikesjö U.M.; Sorensen R.G.; Wozney J.M.	2001
Long-term performance of Osseotite implants: a 6-year clinical follow-up.	Sullivan D.Y.; Sherwood R.L.; Porter S.S.	2001
Reproducibility of peri-implant probing using a force-controlled probe.	Eickholz P.; Grotkamp F.L.; Steveling H.; Mühling J.; Staehle H.J.	2001
Bisphosphonate inhibits alveolar bone resorption in experimentally-induced peri-implantitis in dogs.	Shibutani T.; Inuduka A.; Horiki I.; Luan Q.; Iwayama Y.	2001
Inter-abutment and peri-abutment mucosal enlargement with mandibular implant overdentures.	Payne A.G.; Solomons Y.F.; Tawse-Smith A.; Lownie J.F.	2001
CrossLaps and beta-glucuronidase in peri-implant and gingival crevicular fluid.	Schubert U.; Kleber B.M.; Strietzel F.P.; Dörfling P.	2001
The implant periapical lesion: four case reports and review of the literature.	Jalbout Z.N.; Tarnow D.P.	2001
Osseintegration following treatment of peri-implantitis and replacement of implant components. An experimental study in the dog.	Persson L.G.; Ericsson I.; Berglundh T.; Lindhe J.	2001
Maxillary overdentures retained by splinted and unsplinted implants: a retrospective study.	Närhi T.O.; Hevinga M.; Voorsmit R.A.; Kalk W.	2001
Effectiveness of maxillary overdentures supported by implants: maintenance and prosthetic complications.	Kiener P.; Oetterli M.; Mericske E.; Mericske-Stern R.	2001
Biological and technical complications and failures with fixed partial dentures (FPD) on implants and teeth after four to five years of function.	Brägger U.; Aeschlimann S.; Bürgin W.; Hämmerle C.H.; Lang N.P.	2001
Single stage surgery combining transmucosal implant placement with guided bone regeneration and bioresorbable materials.	Hämmerle C.H.; Lang N.P.	2001
The influences of molar intrusion on the inferior alveolar neurovascular bundle and root using the skeletal anchorage system in dogs.	Daimaruya T.; Nagasaka H.; Umemori M.; Sugawara J.; Mitani H.	2001
Rehabilitation of patients with severely resorbed maxillae by means of implants with or without bone grafts: a 3- to 5-year follow-up clinical report.	Widmark G.; Andersson B.; Carlsson G.E.; Lindvall A.M.; Ivanoff C.J.	2001
Clinical and radiographic evaluation of 108 dental implants after 1-year loading	Tang Z.; Sha Y.; Lin Y.	2001
Vertical distraction osteogenesis of edentulous ridges for improvement of oral implant positioning: a clinical report of preliminary results.	Chiapasco M.; Romeo E.; Vogel G.	2001
Periodontal-type measurements associated with hydroxyapatite-coated and non-HA-coated implants: uncovering to 36 months.	Morris H.F.; Ochi S.; Spray J.R.; Olson J.W.	2000
A clinical, radiographic, and microbiologic comparison of Astra Tech and Brånemark single tooth implants.	Puchades-Roman L.; Palmer R.M.; Palmer P.J.; Howe L.C.; Ide M.; Wilson R.F.	2000
Nonsubmerged implants in the treatment of the edentulous upper jaw: a prospective clinical and radiographic study of ITI implants--results after 1 year.	Astrand P.; Anzén B.; Karlsson U.; Sahlholm S.; Svärdström P.; Hellem S.	2000

Diagnostic characteristics of clinical and microbiological tests for monitoring periodontal and peri-implant mucosal tissue conditions during supportive periodontal therapy (SPT).	Luterbacher S.; Mayfield L.; Brägger U.; Lang N.P.	2000
The influence of smoking on 3-year clinical success of osseointegrated dental implants.	Lambert P.M.; Morris H.F.; Ochi S.	2000
Biological complications with dental implants: their prevention, diagnosis and treatment.	Lang N.P.; Wilson T.G.; Corbet E.F.	2000
Surgical procedures in partially edentulous patients with ITI implants.	Buser D.; von Arx T.	2000
Marginal bone level around implants assessed in digital and film radiographs: in vivo study in the dog.	Borg E.; Gröndahl K.; Persson L.G.; Gröndahl H.G.	2000
Factors associated with radiographic vertical bone loss around implants placed in a clinical study.	Manz M.C.	2000
Histopathologic observations on late oral implant failures.	Esposito M.; Thomsen P.; Ericson L.E.; Sennerby L.; Lekholm U.	2000
Re-establishment of the atomic composition and the oxide structure of contaminated titanium surfaces by means of carbon dioxide laser and hydrogen peroxide: an in vitro study.	Mouhyi J.; Sennerby L.; Wennerberg A.; Louette P.; Dourov N.; van Reck J.	2000
Clinical outcomes of three Parkinson's disease patients treated with mandibular implant overdentures.	Heckmann S.M.; Heckmann J.G.; Weber H.P.	2000
Implant-supported overdenture therapy: a 3- to 8-year prospective study.	Inan O.; Aykent F.; Alptekin N.	2000
Conventional versus laser-assisted therapy of periimplantitis: a five-year comparative study.	Bach G.; Neckel C.; Mall C.; Krekeler G.	2000
Experimental study of Ti-75(AlMoZrTi alloy) dental implants in dog mandible	Tao C.Z.; Zhou J.; Cheng B.S.; Liu Y.; Hao L.	2000
Influence of the prosthetic restoration modality on bone loss around dental implants placed in vascularized iliac bone grafts for mandibular reconstruction	Kovács A.F.	2000
Combined periodontal treatment with dental implants	van der Avoort P.G.; Hosman H.J.; van Waas M.A.	2000
Apical-coronal implant position: recent surgical proposals. Technical note.	Davarpanah M.; Martinez H.; Tecucianu J.F.	2000
Does surgical prophylaxis with teicoplanin constitute a therapeutic advance?	Mini E.; Nobili S.; Periti P.	2000
Tissue inhibitor of metalloproteinases-1, matrix metalloproteinases-1 and -8, and collagenase activity levels in peri-implant crevicular fluid after implantation.	Nomura T.; Ishii A.; Shimizu H.; Taguchi N.; Yoshie H.; Kusakari H.; Hara K.	2000
The longitudinal clinical effectiveness of ITI solid-screw implants in partially edentulous patients: a 5-year follow-up report.	Behneke A.; Behneke N.; d'Hoedt B.	2000
Treatment of ligature-induced peri-implantitis defects by regenerative procedures. Part II: A histometric study in dogs.	Machado M.A.; Stefani C.M.; Sallum E.A.; Sallum A.W.; Tramontina V.A.; Nogueira-Filho G.R.; Nociti Júnior F.H.	2000
Peri-implant mucosal inflammation and bone loss: clinical and radiographic evaluation of 108 dental implants after 1-year loading.	Tang Z.; Sha Y.; Lin Y.; Zhang G.; Wang X.; Cao C.	2000
Long-term evaluation of osseointegrated implants in regenerated and nonregenerated bone.	Corrente G.; Abundo R.; Cardaropoli D.; Cardaropoli G.; Martuscelli G.	2000
Patient perception of local anesthesia for prostate brachytherapy	Smathers S.; Wallner K.; Simpson C.; Roof J.	2000
Correlation of peri-implant health and aspartate aminotransferase levels: a cross-sectional clinical study.	Fiorellini J.P.; Nevins M.L.; Sekler J.; Chung A.; Oringer R.J.	2000
Aspartate aminotransferase activity in crevicular fluid from dental implants.	Paolantonio M.; Di Placido G.; Tumini V.; Di Stilio M.; Contento A.; Spoto G.	2000
Experimentally induced peri-implantitis: a review of different treatment methods described in the literature.	Baron M.; Haas R.; Dörtbudak O.; Watzek G.	2000
Actinobacillus actinomycetemcomitans-associated peri-implantitis in an edentulous patient. A case report.	van Winkelhoff A.J.; Wolf J.W.	2000
A one-year study on enzyme level in gingival crevicular fluid around osseointegrated dental implants	Li X.; Sha Y.; Cao C.	2000
Soft tissue management in oral implantology: a review of surgical techniques for shaping an esthetic and functional peri-implant soft tissue structure.	Khoury F.; Happe A.	2000
Long-term marginal periimplant bone loss in edentulous patients.	Carlsson G.E.; Lindquist L.W.; Jemt T.	2000
Retrospective analysis of Frialit-2 implants in the augmented sinus.	Lorenzoni M.; Pertl C.; Wegscheider W.; Keil C.; Penkner K.; Polansky R.; Bratschko R.O.	2000
Lethal photosensitization, autogenous bone, and e-PTFE membrane for the treatment of peri-implantitis: preliminary results.	Haas R.; Baron M.; Dörtbudak O.; Watzek G.	2000
The influence of controlled occlusal overload on peri-implant tissue. Part 3: A histologic study in monkeys.	Miyata T.; Kobayashi Y.; Araki H.; Ohto T.; Shin K.	2000
Use of short endosseous implants and an overdenture in the extremely resorbed mandible: A five-year retrospective study	Stellingsina C.; Meijer H.J.A.; Raghoebar G.M.	2000
A 5-year prospective clinical and radiographic study of non-submerged dental implants.	Weber H.P.; Crohin C.C.; Fiorellini J.P.	2000
Peri-implant infections.	Clarizio L.F.	2000
Implant-supported mandibular overdentures retained with ball or bar attachments: a randomized prospective 5-year study.	Gotfredsen K.; Holm B.	2000
The fate of osseointegrated implants in patients following oral cancer surgery and mandibular reconstruction	Kovács A.F.	2000

Implants in partially edentulous patients with history of periodontal disease	Barone A.; Porzio M.; Ramaglia L.; Sbordone L.	2000
Treatment of peri-implantitis defects with autogenous bone grafts: six-month to 3-year results of a prospective study in 17 patients.	Behneke A.; Behneke N.; d'Hoedt B.	2000
The efficacy of a counter-rotational powered toothbrush in the maintenance of endosseous dental implants.	Truhlar R.S.; Morris H.F.; Ochi S.	2000
Treatment of ligature-induced peri-implantitis defects by regenerative procedures: a clinical study in dogs.	Machado M.A.; Stefani C.M.; Sallum E.A.; Sallum A.W.; Tramontina V.A.; Nociti Júnior F.H.	1999
A ten-year clinical follow-up study of prosthetic rehabilitation of the edentulous lower jaw with endosteal dental implants	Vajdovich I.; Fazekas A.	1999
Osseointegrated implant failures.	O'Mahony A.; Spencer P.	1999
Longitudinal study of dental implants in a periodontally compromised population.	Sbordone L.; Barone A.; Ciaglia R.N.; Ramaglia L.; Iacono V.J.	1999
Microbial findings at failing implants.	Leonhardt A.; Renvert S.; Dahlén G.	1999
The barrier between the keratinized mucosa and the dental implant. An experimental study in the dog.	Moon I.S.; Berglundh T.; Abrahamsson I.; Linder E.; Lindhe J.	1999
Qualitative analysis of peripheral peri-implant bone and influence of alendronate sodium on early bone regeneration	Meraw S.J.; Reeve C.M.	1999
Clinical and radiographic evaluation of early loaded free-standing dental implants with various coatings in beagle dogs.	Corso M.; Sirota C.; Fiorellini J.; Rasool F.; Szmukler-Moncler S.; Weber H.P.	1999
Bone tissue reaction around implants placed in a compromised jaw.	Carmagnola D.; Araújo M.; Berglundh T.; Albrektsson T.; Lindhe J.	1999
Ramus hinges for excessive movements of the condyles: a new dimension in mandibular tripodal subperiosteal implants.	Linkow L.I.; Ghalili R.	1999
Prevention of bacterial leakage into and from prefabricated screw-retained crowns on implants in vitro.	Besimo C.E.; Guindy J.S.; Lewetag D.; Meyer J.	1999
Local anesthesia for prostate brachytherapy	Wallner K.; Simpson C.; Roof J.; Arthurs S.; Korssjoen T.; Sutlief S.	1999
In vitro response of human fibroblasts to commercially pure titanium	Mostardi R.A.; Meerbaum S.O.; Kovacik M.W.; Gradisar Jr. I.A.	1999
Standardization of radiographs for mandibular implant-supported overdentures: review and innovation.	Payne A.G.; Solomons Y.F.; Lownie J.F.	1999
Treatment of a patient with severe osteoporosis and chronic polyarthritis with fixed implant-supported prosthesis: a case report.	Eder A.; Watzek G.	1999
Ceramic implant abutments used for short-span fixed partial dentures: a prospective 2-year multicenter study.	Andersson B.; Schärer P.; Simion M.; Bergström C.	1999
Peri-implant tissue health in reconstructed atrophic maxillae--report of 88 patients and 470 implants.	Kaptein M.L.; De Lange G.L.; Blijdorp P.A.	1999
Influence of inflammatory reactions vs. occlusal loading on peri-implant marginal bone level.	van Steenberghe D.; Naert I.; Jacobs R.; Quirynen M.	1999
Peri-implant tissue response to pathological insults.	Jovanovic S.A.	1999
Resolution of peri-implantitis following treatment. An experimental study in the dog.	Persson L.G.; Araújo M.G.; Berglundh T.; Gröndahl K.; Lindhe J.	1999
Longitudinal peri-implant clinical responses in anterior mandibles of female patients: a preliminary report.	Ow R.K.; Zarb G.A.; Schmitt A.	1999
In vitro models of biological responses to implant microbiological models.	Mombelli A.	1999
Longitudinal evaluation of aspartate aminotransferase in the crevicular fluid of implants with bone loss and signs of progressive disease.	Rühling A.; Jepsen S.; Kocher T.; Plagmann H.C.	1999
Oral rehabilitation of tumor patients with endosseous implants. Implant success with special reference to peri-implant tissue	Betz T.; Purps S.; Pistner H.; Bill J.; Reuther J.	1999
Prognosis and prognostic factors of endosseous implants in the irradiated jaw	Grötz K.A.; Wahlmann U.W.; Krummenauer F.; Wegener J.; al-Nawas B.; Kuffner H.D.; Wagner W.	1999
Osseointegration of rough acid-etched titanium implants: 5-year follow-up of 100 minimatic implants.	De Leonardis D.; Garg A.K.; Pecora G.E.	1999
Synthetic bone grafts in peri-implant bone dehiscences: histological results in humans.	Passi P.; Girardello G.; Piattelli A.; Scarano A.	1999
Comparative studies of Brånemark implants in the irradiated and not irradiated mandible	Esser E.; Neukirchen S.; Wagner W.	1999
Horse shoe Le Fort I osteotomy. Surgical technique for reconstruction of the extremely atrophied maxilla	Gössweiner S.; Watzinger F.; Ewers R.	1999
Clinical outcome of Screw Vent implants. A 7-year prospective follow-up study.	De Bruyn H.; Collaert B.; Lindén U.; Johansson C.; Albrektsson T.	1999
Five- to six-year results of a prospective clinical trial using the ENDOPORE dental implant and a mandibular overdenture.	Deporter D.; Watson P.; Pharoah M.; Levy D.; Todescan R.	1999
Attempts to obtain re-osseointegration following experimental peri-implantitis in dogs.	Wetzel A.C.; Vlassis J.; Caffesse R.G.; Hämmerle C.H.; Lang N.P.	1999
Peri-implant conditions in periodontally compromised patients following maxillary sinus augmentation. A long-term post-therapy trial.	Buchmann R.; Khoury F.; Faust C.; Lange D.E.	1999
A radiographic evaluation of bone healing around submerged and non-submerged dental implants in beagle dogs.	Fiorellini J.P.; Buser D.; Paquette D.W.; Williams R.C.; Haghighi D.; Weber H.P.	1999
Microbiota of successful osseointegrated dental implants.	Lee K.H.; Maiden M.F.; Tanner A.C.; Weber H.P.	1999
Recombinant human bone morphogenetic protein-2 stimulation of bone formation around endosseous dental implants.	Cochran D.L.; Schenk R.; Buser D.; Wozney J.M.; Jones A.A.	1999

Preventive periimplant protocol.	Eskow R.N.; Smith V.S.	1999
Use of alendronate in peri-implant defect regeneration	Meraw S.J.; Reeve C.M.; Wollan P.C.	1999
Scientific basis of a matrix metalloproteinase-8 specific chair-side test for monitoring periodontal and peri-implant health and disease	Sorsa T.; Mäntylä P.; Rönkä H.; Kallio P.; Kallis G.-B.; Lundqvist C.; Kinane D.F.; Salo T.; Golub L.M.; Teronen O.; Tikanoja S.	1999
C-telopeptide pyridinoline cross-links (ICTP) and periodontal pathogens associated with endosseous oral implants.	Oringer R.J.; Palys M.D.; Iranmanesh A.; Fiorellini J.P.; Haffajee A.D.; Socransky S.S.; Giannobile W.V.	1998
Bactericidal efficacy of carbon dioxide laser against bacteria-contaminated titanium implant and subsequent cellular adhesion to irradiated area	Kato T.; Kusakari H.; Hoshino E.	1998
Mandibular overdentures supported by two Brånemark, IMZ or ITI implants. A prospective comparative preliminary study: one-year results.	Batenburg R.H.; Meijer H.J.; Raghoebar G.M.; Van Oort R.P.; Boering G.	1998
Treatment of patients with extreme maxillary atrophy using sinus floor augmentation and implants: preliminary results.	Watzek G.; Weber R.; Bernhart T.; Ulm C.; Haas R.	1998
Clinical and microbiologic effects of chemical versus mechanical cleansing in professional supportive implant therapy.	Strooker H.; Rohn S.; Van Winkelhoff A.J.	1998
Biologic width and its relation to periodontal biotypes.	Sanavi F.; Weisgold A.S.; Rose L.F.	1998
Implants: success and failure--a literature review.	Koutsonikos A.	1998
Microbial colonization of the peri-implant environment and its relevance to long-term success of osseointegrated implants.	Ellen R.P.	1998
The mucosal attachment at different abutments. An experimental study in dogs.	Abrahamsson I.; Berglundh T.; Glantz P.O.; Lindhe J.	1998
Failure of oral implants: aetiology, symptoms and influencing factors.	Duyck J.; Naert I.	1998
The implant-mucosal interface and its role in the long-term success of endosseous oral implants: a review of the literature.	Koka S.	1998
Do healing abutments influence the outcome of implant treatment? A three-year multicenter study.	Watson R.; Marinello C.; Kjellman O.; Rundcrantz T.; Fåhraeus J.; Lithner B.	1998
Peri-implantitis.	Forna N.; Burlui V.; Luca I.C.; Indrei A.	1998
Successful bone formation at immediate transmucosal implants: a clinical report.	Hämmerle C.H.; Brägger U.; Schmid B.; Lang N.P.	1998
The effect of different transplanted soft tissues on bone resorption around loaded endosseous implants in patients after oral tumor surgery.	Kovács A.	1998
Rehabilitation of patients with severely resorbed maxillae by means of implants with or without bone grafts. A 1-year follow-up study.	Widmark G.; Andersson B.; Andrup B.; Carlsson G.E.; Ivanoff C.J.; Lindvall A.M.	1998
Biological factors contributing to failures of osseointegrated oral implants. (II). Etiopathogenesis.	Esposito M.; Hirsch J.M.; Lekholm U.; Thomsen P.	1998
A 5-year randomized clinical trial on the influence of splinted and unsplinted oral implants in the mandibular overdenture therapy. Part I: Peri-implant outcome.	Naert I.; Gizani S.; Vuylsteke M.; van Steenberghe D.	1998
The diagnosis and treatment of peri-implantitis.	Mombelli A.; Lang N.P.	1998
The EsthetiCone abutment: three-year results of a prospective multicenter investigation.	Kastenbaum F.; Lewis S.; Naert I.; Palmquist C.	1998
Cemented single crowns on osseointegrated implants after 5 years: results from a prospective study on CeraOne.	Andersson B.; Odman P.; Lindvall A.M.; Brånemark P.I.	1998
Nonhormonal alternatives to treating menopausal symptoms	Cass I.; Runowicz C.D.	1998
A retrospective multicenter evaluation of single tooth implant experience at three centers in the United Kingdom.	McMillan A.S.; Allen P.F.; Bin Ismail I.	1998
Localized ridge augmentation using a micro titanium mesh: a report on 27 implants followed from 1 to 3 years after functional loading.	von Arx T.; Wallkamm B.; Hardt N.	1998
Soft tissue response to plaque formation at different implant systems. A comparative study in the dog.	Abrahamsson I.; Berglundh T.; Lindhe J.	1998
Implant placement and simultaneous peri-implant bone grafting using a micro titanium mesh for graft stabilization.	von Arx T.; Kurt B.	1998
Changes in peri-implant tissues subjected to orthodontic forces and ligature breakdown in monkeys.	Hürzeler M.B.; Quiñones C.R.; Kohal R.J.; Rohde M.; Strub J.R.; Teuscher U.; Caffesse R.G.	1998
Use of the Endopore dental implant to restore single teeth in the maxilla: protocol and early results.	Deporter D.A.; Todescan R.; Watson P.A.; Pharoah M.; Levy D.; Nardini K.	1998
Overdentures stabilised by two IMZ implants in the lower jaw--a 5-8 year retrospective study.	Heydenrijk K.; Batenburg R.H.; Raghoebar G.M.; Meijer H.J.; van Oort R.P.; Stegenga B.	1998
Osseointegrated implants and orbital defects: U.C.L.A. experience.	Nishimura R.D.; Roumanas E.; Moy P.K.; Sugai T.; Freymiller E.G.	1998
Microbiota associated with experimental peri-implantitis and periodontitis in adult Macaca mulatta monkeys.	Eke P.I.; Braswell L.D.; Fritz M.E.	1998
Biological factors contributing to failures of osseointegrated oral implants. (I). Success criteria and epidemiology.	Esposito M.; Hirsch J.M.; Lekholm U.; Thomsen P.	1998
The key to success: maintaining the long-term health of implants.	Silverstein L.; Garg A.; Callan D.; Shatz P.	1998
Histological evaluation of Bio-Oss in a 2-stage sinus floor elevation and implantation procedure. A human case report.	Valentini P.; Abensur D.; Densari D.; Graziani J.N.; Hämmerle C.	1998
Evaluation of bone healing in patients with bone grafts and endosseous implants using single photon emission tomography (SPECT).	Schliephake H.; Berding G.	1998
The mandibular staple bone plate: A long-term retrospective evaluation	Meijer H.J.A.; Van Oort R.P.; Raghoebar G.M.; Schoen P.J.	1998

Evaluation of three different dental implants in ligature-induced peri-implantitis in the beagle dog. Part II. Histology and microbiology.	Tillmanns H.W.; Hermann J.S.; Tiffee J.C.; Burgess A.V.; Meffert R.M.	1998
Endosseous implant management of tumor patients with the bone lock system. A 5-year study	Kovács A.	1998
Bone regeneration for reintegration in peri-implant destruction.	Artzi Z.; Tal H.; Chweidan H.	1998
Radiographic assessment of peri-implant vertical bone loss: DICRG interim report no. 9	Manz M.C.; Morris H.F.	1997
Diagnosis and management of peri-implant disease.	Kao R.T.; Curtis D.A.; Murray P.A.	1997
Treatment of retrograde peri-implantitis: clinical report.	Bretz W.A.; Matuck A.N.; de Oliveira G.; Moretti A.J.; Bretz W.A.	1997
Etiology, diagnosis, and treatment considerations in peri-implantitis.	Mombelli A.	1997
Prosthodontic and periodontal considerations for implant-supported dental restorations.	Cooper L.; Moriarty J.	1997
Comparison of periodontal and peri-implant probing by depth-force pattern analysis.	Mombelli A.; Mühle T.; Brägger U.; Lang N.P.; Bürgin W.B.	1997
Reproducibility of automated periodontal probing around teeth and osseointegrated oral implants.	Christensen M.M.; Joss A.; Lang N.P.	1997
Nonsteroidal anti-inflammatory drugs as adjuncts in the management of periodontal diseases and peri-implantitis.	Salvi G.E.; Williams R.C.; Offenbacher S.	1997
Treatment of severe peri-implant bone loss using autogenous bone and a resorbable membrane. Case report and literature review.	von Arx T.; Kurt B.; Hardt N.	1997
Therapy of peri-implant inflammation with the CO2 swift laser system. An in vitro and in vivo study	Deppe H.; Horch H.H.; Hiermer T.; Lebelt G.; Milatovic D.; Henke J.; Erhardt W.	1997
Regeneration of peri-implant infrabony defects using PerioGlas: a pilot study in rabbits.	Johnson M.W.; Sullivan S.M.; Rohrer M.; Collier M.	1997
Associations between clinical parameters assessed around implants and teeth.	Brägger U.; Bürgin W.B.; Hämmerle C.H.; Lang N.P.	1997
Preliminary results of a multicenter study evaluating a chemically enhanced surface for machined commercially pure titanium implants.	Sullivan D.Y.; Sherwood R.L.; Mai T.N.	1997
Association between marginal bone loss around osseointegrated mandibular implants and smoking habits: a 10-year follow-up study.	Lindquist L.W.; Carlsson G.E.; Jemt T.	1997
The combined use of endosteal implants and iliac crest onlay grafts in the severely atrophic mandible: a longitudinal study.	Verhoeven J.W.; Cune M.S.; Terlou M.; Zoon M.A.; de Putter C.	1997
Effects of guided bone regeneration around commercially pure titanium and hydroxyapatite-coated dental implants. II. Histologic analysis.	Stentz W.C.; Mealey B.L.; Gunsolley J.C.; Waldrop T.C.	1997
Controlled local delivery of tetracycline HCl in the treatment of periimplant mucosal hyperplasia and mucositis. A controlled case series.	Schenk G.; Flemmig T.F.; Betz T.; Reuther J.; Klaiber B.	1997
Crevicular fluid analysis around two failing dental implants: a clinical report.	Curtis D.A.; Kao R.; Plesh O.; Finzen F.; Franz L.	1997
Human neutrophil collagenase MMP-8 in peri-implant sulcus fluid and its inhibition by clodronate.	Teronen O.; Konttinen Y.T.; Lindqvist C.; Salo T.; Ingman T.; Lauhio A.; Ding Y.; Santavirta S.; Sorsa T.	1997
Evaluation of three different dental implants in ligature-induced peri-implantitis in the beagle dog. Part I. Clinical evaluation.	Tillmanns H.W.; Hermann J.S.; Cagna D.R.; Burgess A.V.; Meffert R.M.	1997
Bone formation and reosseointegration in peri-implantitis defects following surgical implantation of rhBMP-2.	Hanisch O.; Tatakis D.N.; Boskovic M.M.; Rohrer M.D.; Wikesjö U.M.	1997
Early detection of threatened implant loss in tumor patients	Weischer T.; Mohr C.	1997
Periodontal parameters of osseointegrated dental implants. A 4-year controlled follow-up study.	Nishimura K.; Itoh T.; Takaki K.; Hosokawa R.; Naito T.; Yokota M.	1997
Hard-tissue augmentation for the placement of anterior dental implants.	Weber H.P.; Fiorellini J.P.; Buser D.A.	1997
The mucosal barrier following abutment dis/reconnection. An experimental study in dogs.	Abrahamsson I.; Berglundh T.; Lindhe J.	1997
Mandibular overdentures anchored to single implants: a five-year prospective study.	Cordioli G.; Majzoub Z.; Castagna S.	1997
Long-term efficacy of hydroxyapatite-coated cylindrical implants.	Hahn J.; Vassos D.M.	1997
Long-term evaluation of non-submerged ITI implants. Part 1: 8-year life table analysis of a prospective multi-center study with 2359 implants.	Buser D.; Mericske-Stern R.; Bernard J.P.; Behneke A.; Behneke N.; Hirt H.P.; Belser U.C.; Lang N.P.	1997
Vertical ridge augmentation using polylactic membranes in conjunction with immediate implants in periodontally compromised extraction sites: an experimental study in dogs.	Schliephake H.; Kracht D.	1997
The Frialit-2 implant system: five-year clinical experience in single-tooth and immediately postextraction applications.	Gomez-Roman G.; Schulte W.; d'Hoedt B.; Axman-Krcmar D.	1997
Implant-supported telescopic restorations in maxillofacial prosthetics.	Weischer T.; Schettler D.; Mohr C.	1997
Complications and maintenance requirements of implant-supported prostheses provided in a UK dental hospital.	Allen P.F.; McMillan A.S.; Smith D.G.	1997
A comparative prospective clinical study of two single-tooth implants: a preliminary report of 102 implants.	Kemppainen P.; Eskola S.; Ylipaavalniemi P.	1997
Effects of subgingival chlorhexidine irrigation on peri-implant maintenance.	Felo A.; Shibly O.; Ciancio S.G.; Lauciello F.R.; Ho A.	1997
Periodontal bacteria colonizing oral mucous membranes in edentulous patients wearing dental implants.	Danser M.M.; van Winkelhoff A.J.; van der Velden U.	1997
A controlled clinical trial of implant-retained mandibular overdentures: clinical aspects.	Boerrigter E.M.; van Oort R.P.; Raghoebar G.M.; Stegenga B.; Schoen P.J.; Boering G.	1997

Effects of guided bone regeneration around commercially pure titanium and hydroxyapatite-coated dental implants. I. Radiographic analysis.	Stentz W.C.; Mealey B.L.; Nummikoski P.V.; Gunsolley J.C.; Waldrop T.C.	1997
Long-term evaluation of single crystal sapphire implants as abutments in fixed prosthodontics.	Fartash B.; Arvidson K.	1997
Responses of peri-implant tissues to undisturbed plaque formation in dogs: clinical, radiographic, and microbiological findings.	Saito A.; Hosaka Y.; Sekiguchi K.; Kigure T.; Isobe S.; Shibukawa Y.; Sumii H.; Ito T.; Nakagawa T.; Yamada S.	1997
Composition of plaque-associated lesions in the gingiva and the peri-implant mucosa in partially edentulous subjects.	Liljenberg B.; Gualini F.; Berglundh T.; Tonetti M.; Lindhe J.	1997
Healing at implants with and without primary bone contact. An experimental study in dogs.	Scipioni A.; Bruschi G.B.; Giargia M.; Berglundh T.; Lindhe J.	1997
Experimental peri-implant tissue breakdown around hydroxyapatite-coated implants.	Hanisch O.; Cortella C.A.; Boskovic M.M.; James R.A.; Slots J.; Wikesjö U.M.	1997
Three-year evaluation of Brånemark implants connected to angulated abutments.	Balshi T.J.; Ekfeldt A.; Stenberg T.; Vrielinck L.	1997
Osseointegration of rough acid-etched implants: one-year follow-up of placement of 100 minimatic implants.	De Leonardis D.; Garg A.K.; Pecora G.E.; Andreana S.	1997
A nationwide evaluative study on implant-retained overdentures.	Cune M.S.; de Putter C.; Hoogstraten J.	1997
Implant-supported single-tooth replacements: risk of implant and prosthesis failure.	Walther W.; Klemke J.; Wörle M.; Heners M.	1996
Advanced imaging methods	Matteson S.R.; Deahl S.T.; Alder M.E.; Nummikoski P.V.	1996
Recession of the soft tissue margin at oral implants. A 2-year longitudinal prospective study.	Bengazi F.; Wennström J.L.; Lekholm U.	1996
Guided bone regeneration in the treatment of periimplantitis.	Persson L.G.; Ericsson I.; Berglundh T.; Lindhe J.	1996
Nasal defects and osseointegrated implants: UCLA experience.	Nishimura R.D.; Roumanas E.; Moy P.K.; Sugai T.	1996
The effect of antimicrobial therapy on periimplantitis lesions. An experimental study in the dog.	Ericsson I.; Persson L.G.; Berglundh T.; Edlund T.; Lindhe J.	1996
Horseshoe Le Fort I osteotomy in combination with endosteal implants--a median-term follow-up study.	Watzinger F.; Ewers R.; Millesi W.; Kirsch A.; Glaser C.; Ackermann K.L.	1996
Peri-arteritis causing ureteric obstruction	Sharma S.D.; Burgess N.A.; Doble A.; Doyle P.T.	1996
Periodontitis vs. peri-implantitis: The same disease? The same treatment?	Meffert R.M.	1996
Rehabilitation of mandibular edentulism by single crystal sapphire implants and overdentures: 3-12 year results in 86 patients. A dual center international study.	Fartash B.; Tangerud T.; Silness J.; Arvidson K.	1996
Immediate transmucosal implants using the principle of guided tissue regeneration (II). A cross-sectional study comparing the clinical outcome 1 year after immediate to standard implant placement.	Brägger U.; Hämmerle C.H.; Lang N.P.	1996
Correlations between radiographic, clinical and mobility parameters after loading of oral implants with fixed partial dentures. A 2-year longitudinal study.	Brägger U.; Hugel-Pisoni C.; Bürgin W.; Buser D.; Lang N.P.	1996
The influence of abutment surface roughness on plaque accumulation and peri-implant mucositis.	Bollen C.M.; Papaioanno W.; Van Eldere J.; Schepers E.; Quirynen M.; van Steenberghe D.	1996
Efficacy of threaded hydroxyapatite-coated implants in the anterior mandible supporting overdentures.	Buchs A.U.; Hahn J.; Vassos D.M.	1996
Osseointegrated implants in patients treated for generalized severe adult periodontitis. An interim report.	Mengel R.; Stelzel M.; Hasse C.; Flores-de-Jacoby L.	1996
Reconstruction of the severely resorbed maxilla with dental implants in the augmented maxillary sinus: a 5-year clinical investigation.	Hürzeler M.B.; Kirsch A.; Ackermann K.L.; Quiñones C.R.	1996
Periodontal tissue engineering by growth factors	Giannobile W.V.	1996
Progressive peri-implantitis. Incidence and prediction of peri-implant attachment loss.	Jepsen S.; Rühling A.; Jepsen K.; Ohlenbusch B.; Albers H.K.	1996
Efficacy of threaded hydroxyapatite-coated implants placed in the posterior mandible in support of fixed prostheses.	Buchs A.U.; Hahn J.; Vassos D.M.	1996
Loss of osseointegration caused by occlusal load of oral implants. A clinical and radiographic study in monkeys.	Isidor F.	1996
The Orthosystem--a new implant system for orthodontic anchorage in the palate.	Wehrbein H.; Glatzmaier J.; Mundwiller U.; Diedrich P.	1996
Crevicular fluid enzymes from endosseous dental implants and natural teeth.	Boutros S.M.; Michalowicz B.S.; Smith Q.T.; Aeppli D.M.	1996
A 5-year prospective multicenter follow-up report on overdentures supported by osseointegrated implants.	Jemt T.; Chai J.; Harnett J.; Heath M.R.; Hutton J.E.; Johns R.B.; McKenna S.; McNamara D.C.; van Steenberghe D.; Taylor R.; Watson R.M.; Herrmann I.	1996
An analysis of the diagnostic criteria in assessing peri-implant tissues. A critical analysis of the literature	Weinstein R.L.; Francetti L.; Sironi R.	1996
Peri-implantitis.	Bower R.C.	1996
Similarities between the development and treatment of plaque-induced peri-implantitis and periodontitis.	Soehren S.E.	1996
Comparison of healed tissues adjacent to submerged and non-submerged unloaded titanium dental implants. A histometric study in beagle dogs.	Weber H.P.; Buser D.; Donath K.; Fiorellini J.P.; Doppalapudi V.; Paquette D.W.; Williams R.C.	1996
Immediate placement of implants into extraction sockets: implant survival.	Rosenquist B.; Grenthe B.	1996

The effect of an antiseptic mouthrinse on implant maintenance: Plaque and peri-implant gingival tissues	Ciancio S.G.; Lauciello F.; Shibly O.; Vitello M.; Mather M.	1995
Efficacy of threaded hydroxyapatite-coated implants placed in the anterior mandible.	Buchs A.U.; Hahn J.; Vassos D.M.	1995
Nifedipine-induced gingival enlargement around dental implants: a clinical report.	Silverstein L.H.; Koch J.P.; Lefkove M.D.; Garnick J.J.; Singh B.; Steflik D.E.	1995
Plaque-induced peri-implantitis in the presence or absence of keratinized mucosa. An experimental study in monkeys.	Warrer K.; Buser D.; Lang N.P.; Karring T.	1995
Influence of the suprastructure on the peri-implant tissues in beagle dogs.	Hürzeler M.B.; Quiñones C.R.; Schüpbach P.; Vlassis J.M.; Strub J.R.; Caffesse R.G.	1995
Auricular prostheses and osseointegrated implants: UCLA experience.	Nishimura R.D.; Roumanas E.; Sugai T.; Moy P.K.	1995
Successful bone fill in late peri-implant defects using guided tissue regeneration. A short communication.	Hämmerle C.H.; Fourmousis I.; Winkler J.R.; Weigel C.; Brägger U.; Lang N.P.	1995
Implant-retained overdentures. Part 1. Clinical findings from an evaluation study	Cune M.S.; de Putter C.; Hoogstraten J.	1995
Periimplant tissue considerations for optimal implant results.	Saadoun A.P.	1995
Evidence-based periodontal treatment. II. Predictable regeneration treatment.	Newman M.G.; McGuire M.K.	1995
Brånemark single tooth implants: a preliminary report of 76 implants.	Haas R.; Mensdorff-Pouilly N.; Mailath G.; Watzek G.	1995
Antimicrobial susceptibility of periodontopathic bacteria associated with failing implants	Sbordone L.; Barone A.; Ramaglia L.; Ciaglia R.N.; Iacono V.J.	1995
Histologic probe penetration in healthy and inflamed peri-implant tissues.	Lang N.P.; Wetzel A.C.; Stich H.; Caffesse R.G.	1994
Experimentally induced peri-implant mucositis. A clinical study in humans.	Pontoriero R.; Tonelli M.P.; Carnevale G.; Mombelli A.; Nyman S.R.; Lang N.P.	1994
The rationale for soft-tissue grafting and vestibuloplasty in association with endosseous implants: a literature review.	Hoelscher D.C.; Simons A.M.	1994
Contaminated implant surfaces: An in vitro comparison of implant surface coating and treatment modalities for decontamination	Dennison D.K.; Huerzeler M.B.; Quinones C.; Caffese R.G.	1994
Inhibition of peri-implant bone loss with the nonsteroidal anti-inflammatory drug flurbiprofen in beagle dogs. A preliminary study.	Weber H.P.; Fiorellini J.P.; Paquette D.W.; Howell T.H.; Williams R.C.	1994
Evaluation of the effects of diagnostic radiation on titanium dental implant osseointegration in the micropig.	Basquill P.J.; Steflik D.E.; Brennan W.A.; Horner J.; Van Dyke T.E.	1994
A progressive treatment protocol for maintaining tissue associated with implants.	Love W.B.; Backman G.W.	1994
Diagnosis and treatment of peri-implant disease.	Jovanovic S.A.	1994
Assessing bacterial risk factors for periodontitis and peri-implantitis: using evidence to enhance outcomes.	Newman M.G.; Marinho V.C.	1994
Treatment outcome with implant-retained overdentures: Part I--Clinical findings and predictability of clinical treatment outcome.	Cune M.S.; de Putter C.; Hoogstraten J.	1994
Technetium 99m-MDP scintigraphy of patients undergoing implant prosthetic procedures: a follow-up study.	Meidan Z.; Weisman S.; Baron J.; Binderman I.	1994
The influence of the masticatory mucosa on the peri-implant soft tissue condition.	Wennström J.L.; Bengazi F.; Lekholm U.	1994
Peri-implant mucosal aspects of ITI implants supporting overdentures. A five-year longitudinal study.	Mericske-Stern R.; Steinlin Schaffner T.; Marti P.; Geering A.H.	1994
Complications and maintenance requirements for fixed prostheses and overdentures in the edentulous mandible: a 5-year report.	Hemmings K.W.; Schmitt A.; Zarb G.A.	1994
Characteristics associated with the loss and peri-implant tissue health of endosseous dental implants.	Weyant R.J.	1994
Forces on implants supporting overdentures: a preliminary study of morphologic and cephalometric considerations.	Mericske-Stern R.	1993
A proposed radiographic index for assessment of the current status of osseointegration.	Nasr H.F.; Meffert R.M.	1993
The management of peri-implant breakdown around functioning osseointegrated dental implants.	Jovanovic S.A.	1993
Glycosaminoglycans in peri-implant sulcus fluid from implants supporting fixed or removable prostheses.	Smedberg J.I.; Beck C.B.; Embery G.	1993
Clinical applications compared in implantology	Itro A.; D'Amato S.	1993
Reproducibility and detection threshold of peri-implant diagnostics.	van Steenberghe D.; Quirynen M.	1993
Application of digital radiography to implantology.	Jeffcoat M.K.	1993
Oral and dental infections with anaerobic bacteria: Clinical features, predominant pathogens, and treatment	Tanner A.; Stillman N.	1993
The regenerative potential of plaque-induced peri-implant bone defects treated by a submerged membrane technique: an experimental study.	Jovanovic S.A.; Kenney E.B.; Carranza Jr. F.A.; Donath K.	1993
Effects of drill cooling and bone structure on IMZ implant fixation.	Haider R.; Watzek G.; Plenk H.	1993
The use of free gingival grafts in the treatment of peri-implant soft tissue complications: clinical report.	Simons A.M.; Darany D.G.; Giordano J.R.	1993
Antimicrobial treatment of peri-implant infections.	Mombelli A.; Lang N.P.	1992

Plaque-induced marginal tissue reactions of osseointegrated oral implants: a review of the literature.	Schou S.; Holmstrup P.; Hjørting-Hansen E.; Lang N.P.	1992
Biological requirements for material integration.	O'Neal R.B.; Sauk J.J.; Somerman M.J.	1992
The biology and morphology of the implant-tissue interface.	Weber H.P.; Fiorellini J.P.	1992
Soft tissue management during implant maintenance.	Rapley J.W.; Mills M.P.; Wylam J.	1992
Use of an antibiotic-bonded graft for in situ reconstruction after prosthetic graft infections	Colburn M.D.; Moore W.S.; Chvapil M.; Gelabert H.A.; Quinones-Baldrich W.J.; McIntyre Jr. K.E.; Reilly L.M.	1992
Soft tissue reactions to non-submerged unloaded titanium implants in beagle dogs.	Buser D.; Weber H.P.; Donath K.; Fiorellini J.P.; Paquette D.W.; Williams R.C.	1992
The occurrence of periodontitis-related microorganisms in relation to titanium implants.	Ong E.S.; Newman H.N.; Wilson M.; Bulman J.S.	1992
Microbial etiology of periodontal diseases. Where are we? Where are we going?	Tanner A.	1992
A comparative study of the clinical efficacy of Screw Vent implants versus Brånemark fixtures, installed in a periodontal clinic.	De Bruyn H.; Collaert B.; Lindén U.; Flygare L.	1992
Prospective review of integral implants.	Block M.S.; Kent J.N.	1992
Control of peri-implant inflammation.	Hillenburg K.L.; Kosinski T.F.; Mentag P.J.	1991
Levels of glycosaminoglycans in peri-implant sulcus fluid as a means of monitoring bone response to endosseous dental implants.	Beck C.B.; Embery G.; Langley M.S.; Waddington R.J.	1991
Microbiologic characterization of ligature-induced peri-implantitis in the microswine model.	Hickey J.S.; O'Neal R.B.; Scheidt M.J.; Strong S.L.; Turgeon D.; Van Dyke T.E.	1991
Implant maintenance: debridement and peri-implant home care.	Bauman G.R.; Mills M.; Rapley J.W.; Hallmon W.W.	1991
Clinical and microbiological findings on newly inserted hydroxyapatite-coated and pure titanium human dental implants.	Rams T.E.; Roberts T.W.; Feik D.; Molzan A.K.; Slots J.	1991
Periimplant alterations under cyclosporin A drug treatment	Günay H.; Evers B.	1991
Maintenance and osseointegrated implants	Detienville R.; Besnainou B.; Mirot F.	1991
Protease activities in peri-implant sulcus fluid from patients with permucosal osseointegrated dental implants. Correlation with clinical parameters.	Eley B.M.; Cox S.W.; Watson R.M.	1991
What causes peri-implantitis?	Meffert R.M.	1991
Peri-implant osseous regeneration	Celletti R.; Davarpanah M.; Pecora G.	1991
Tissue integration of non-submerged implants. 1-year results of a prospective study with 100 ITI hollow-cylinder and hollow-screw implants.	Buser D.; Weber H.P.; Lang N.P.	1990
Maintaining periodontal treatment.	Wilson Jr. T.G.	1990
Periodontal requirements in osseointegrated and biointegrated implantology	Saadoun A.P.	1990
Dental professional's role in monitoring and maintenance of tissue-integrated prostheses.	Orton G.S.; Steele D.L.; Wolinsky L.E.	1989
Soft-tissue modifications of surgical techniques for placement and uncovering of osseointegrated implants.	Moy P.K.; Weinlaender M.; Kenney E.B.	1989
Conditions of the gingiva around endosteal implants with attached and unattached mucosa	Lill W.; Forster H.; Eckhardt C.; Matejka M.; Watzek G.	1989
Microbiota and crevicular fluid collagenase activity in the osseointegrated dental implant sulcus: a comparison of sites in edentulous and partially edentulous patients.	Apse P.; Ellen R.P.; Overall C.M.; Zarb G.A.	1989
Periodontal considerations of implants and implant associated microbiota.	Newman M.G.; Flemmig T.F.	1988
The ceramic coated cementless total hip arthroplasty: Basic concepts and surgical technique	Bousquet G.; Gazielly D.F.; Debiesse J.L.	1985
Total prosthetic replacement of aortic valve in calves	Larson R.E.; McGoon D.C.	1964
Analysis of the association of IL1B (C+3954T) and IL1RN (intron 2) polymorphisms with dental implant loss in a Brazilian population	Montes, Claudia Cristina; Alvim-Pereira, Fabiano; de Castilhos, Bruno Borges; Lopes Sakurai, Marcos Luciano; Olandoski, Marcia; Trevilatto, Paula Cristina	2009
Biomarkers Associated With Periimplant Diseases	Li, Jeff Yuanjun; Wang, Hom-Lay	2014
Fluid Exudates From Inflamed Bone-Anchored Hearing Aids Demonstrate Elevated Levels of Cytokines and Biomarkers of Tissue and Bone Metabolism	Grant, Melissa M.; Monksfield, Peter; Proops, David; Brine, Maxine; Addison, Owen; Sammons, Rachel L.; Matthews, John B.; Reid, Andrew; Chapple, Iain L. C.	2010
Metal Nanoparticles Released from Dental Implant Surfaces: Potential Contribution to Chronic Inflammation and Peri-Implant Bone Loss	Bressan, Eriberto; Ferroni, Letizia; Gardin, Chiara; Bellin, Gloria; Sbricoli, Luca; Sivolella, Stefano; Brunello, Giulia; Schwartz-Arad, Devorah; Mijiritsky, Eitan; Penarrocha, Miguel; Penarrocha, David; Taccioli, Cristian; Tatullo, Marco; Piattelli, Adriano; Zavan, Barbara	2019
The effect of orally administered probiotic Lactobacillus reuteri-containing tablets in peri-implant mucositis: a double-blind randomized controlled trial.	Flichy-Fernandez, A. J.; Ata-Ali, J.; Alegre-Domingo, T.; Candel-Marti, E.; Ata-Ali, F.; Palacio, J. R.; Penarrocha-Diago, M.	2015
The pathology of bone tissue during peri-implantitis.	Schminke, B.; Vom Orde, F.; Gruber, R.; Schliephake, H.; Burgers, R.; Miosge, N.	2015

Diagnosing peri-implant disease using the tongue as a 24/7 detector.	Ritzer, J.; Luhmann, T.; Rode, C.; Pein-Hackelbusch, M.; Immohr, I.; Schedler, U.; Thiele, T.; Stubinger, S.; Rechenberg, B. V.; Waser-Althaus, J.; Schlottig, F.; Merli, M.; Dawe, H.; Karpisek, M.; Wyrwa, R.; Schnabelrauch, M.; Meinel, L.	2017
Calprotectin and cross-linked N-telopeptides of type I collagen levels in crevicular fluid from implant sites with peri-implant diseases: a pilot study.	Sakamoto, Eijiro; Kido, Rie; Tomotake, Yoritoki; Naitou, Yoshihito; Ishida, Yuichi; Kido, Jun-Ichi	2018
Genetic involvement in dental implant failure: Association with polymorphisms of genes modulating inflammatory responses and bone metabolism.	Chen, Xun; Zhao, Yu	2019
In vivo osteoprotegerin gene therapy preventing bone loss induced by periodontitis.	Tang, H.; Mattheos, N.; Yao, Y.; Jia, Y.; Ma, L.; Gong, P.	2015
Relationship between interleukin-1 genotype and peri-implantitis: a literature review.	Andreiotelli, Marina; Koutayas, Spiridon-Oumvertos; Madianos, Phoebus N.; Strub, Jorg-Rudolf	2008
Biological factors involved in implant-anchored orthodontics and in prosthetic-implant therapy: a literature review.	Antoszewska, Joanna; Raftowicz-Wojcik, Katarzyna; Kawala, Beata; Matthews-Brzozowska, Teresa	2010
Collagenase-2 (MMP-8) as a point-of-care biomarker in periodontitis and cardiovascular diseases. Therapeutic response to non-antimicrobial properties of tetracyclines.	Sorsa, Timo; Tervahartiala, Taina; Leppilahti, Jussi; Hernandez, Marcela; Gamonal, Jorge; Tuomainen, Anita M.; Lauhio, Anneli; Pussinen, Pirkko J.; Mantyla, Paivi	2011
Detection and measurement of inflammatory cytokines in implant crevicular fluid: a pilot study.	Panagakos, F. S.; Aboyoussef, H.; Dondero, R.; Jandinski, J. J.	1996
A critical assessment of interleukin-1 (IL-1) genotyping when used in a genetic susceptibility test for severe chronic periodontitis.	Greenstein, Gary; Hart, Thomas C.	2002
Analysis of aspartate aminotransferase and alkaline phosphatase in crevicular fluid from implants with and without peri-implantitis.	Paknejad, Mojgan; Emtiaz, Shahram; Khoobyari, Mohammad Mohsen; Gharb, Mehrnoush Teimouri; Yazdi, Mojtaba Tabatabaei	2006
Role of fibroblast populations in peri-implantitis.	Bordin, Sandra; Flemmig, Thomas F.; Verardi, Simone	2009
Human neutrophil collagenase MMP-8 in peri-implant sulcus fluid and its inhibition by clodronate.	Teronen, O.; Konttinen, Y. T.; Lindqvist, C.; Salo, T.; Ingman, T.; Lauhio, A.; Ding, Y.; Santavirta, S.; Sorsa, T.	1997
Detection of Prostaglandin E 2 and Matrix Metalloproteinases in Implant Crevicular Fluid.	Aboyoussef, Hoda; Carter, Cristina; Jandinski, John J.; Panagakos, Fotinos S.	1998
Inhibition of the degradation of connective tissue matrix protein components in mammals	Teronen, Olli Pekka; Sorsa, Timo Arto; Salo, Tuula Anneli	1997
[truncated: 288,892 more chars]
